# DNA methylation Landscape of body size variation in sheep

**DOI:** 10.1038/srep13950

**Published:** 2015-10-16

**Authors:** Jiaxue Cao, Caihong Wei, Dongming Liu, Huihua Wang, Mingming Wu, Zhiyuan Xie, Terence D. Capellini, Li Zhang, Fuping Zhao, Li Li, Tao Zhong, Linjie Wang, Jian Lu, Ruizao Liu, Shifang Zhang, Yongfei Du, Hongping Zhang, Lixin Du

**Affiliations:** 1 National Center for Molecular Genetics and Breeding of Animal, Institute of Animal Sciences, Chinese Academy of Agricultural Sciences, Beijing 100193, China; 2 Farm Animal Genetic Resources Exploration and Innovation Key Laboratory of Sichuan Province, Sichuan Agricultural University, Ya’an 625014, China; 3 Department of Human Nutrition, Food, and Exercise, Virginia Polytechnic Institute, Blacksburg, Virginia 24061, United States; 4 BGI-Shenzhen, Shenzhen, Guangdong 518083, China; 5 Department of Human Evolutionary Biology, Harvard University, Cambridge 02138, United States; 6 National Center of Preservation & Utilization of Genetic Resources of Animal, Beijing 100194, China

## Abstract

Sub-populations of Chinese Mongolian sheep exhibit significant variance in body mass. In the present study, we sequenced the whole genome DNA methylation in these breeds to detect whether DNA methylation plays a role in determining the body mass of sheep by Methylated DNA immunoprecipitation – sequencing method. A high quality methylation map of Chinese Mongolian sheep was obtained in this study. We identified 399 different methylated regions located in 93 human orthologs, which were previously reported as body size related genes in human genome-wide association studies. We tested three regions in *LTBP1*, and DNA methylation of two CpG sites showed significant correlation with its RNA expression. Additionally, a particular set of differentially methylated windows enriched in the “development process” (GO: 0032502) was identified as potential candidates for association with body mass variation. Next, we validated small part of these windows in 5 genes; DNA methylation of *SMAD1*, *TSC1* and *AKT1* showed significant difference across breeds, and six CpG were significantly correlated with RNA expression. Interestingly, two CpG sites showed significant correlation with TSC1 protein expression. This study provides a thorough understanding of body size variation in sheep from an epigenetic perspective.

Body size such as height, weight or body mass, is a complex trait determined by environment factors and genetic factors^1^. It has been extensively reported and studied, as it was important to farming, and also an important parameter of human growth and health. Body size is usually considered to have been influenced by hundreds of genes. In the past years, a number of genome-wide association studies (GWAS) have investigated the genetic basis of body size variation in cattle^2^ and dogs^3^. And until recently, more than 20 GWASs have identified over 400 candidate genes been associated with human height. However, the molecular basis for size variation besides DNA change remains largely unknown in sheep.

Epigenetics is the study of heritable changes in gene expression that do not involve changes to DNA sequence^4^. The most common forms of epigenetic regulation of gene expression are DNA methylation and histone acetylation or methylation. DNA methylation is involved in many biological processes such as genomic stability, cell differentiation, genomic imprinting, X chromosome inactivation, transposon silencing, and various chronic diseases^5^,^6^,^7^,^8^. In general, promoter hypermethylation leads to gene repression^9^, whereas gene body methylation is associated with increased transcriptional activity^10^ and splicing regulation^11^. Numerous reports have confirmed that DNA methylation has a close relationship with phenotype of creature under different environment^12^, and emerging evidence suggests that DNA methylation relates to transgenerational inheritance^13^,^14^,^15^. DNA methylation reprograms in each generation, and it is possible for it to participate in phenotype divergence and transmit through multiple generations.

Recent studies suggested that DNA methylation might play an important role in phenotype variation^16^,^17^,^18^. Feinberg *et al.* found that four different methylated regions (DMR) have significant correlation with body mass index (BMI)^19^. Relton *et al.* suggested that DNA methylation has a close relationship with altered gene expression as well as children body size^20^. Dick *et al.* identified five probes strongly associated with BMI in European origin individuals^21^. Besides one SNP we obtained close to MBD5 (Methyl-CpG-binding domain protein 5), which was a candidate gene affecting post-weaning gain in sheep^22^. This gene has one domain to bind methylated DNA, which suggested DNA methylation may be involved with post-weaning development. Hence, we suppose that DNA methylation may associate with sheep development, and therefore may contribute to body size.

Chinese Mongolian sheep produces high quality meat with the ability to adapt and survive in a variety of climatic and grazing conditions. As a result, this sheep is popularly raised various regions in China including Inner Mongolia (Ujumqin sheep, UQ), Ningxia (Tan sheep, Tan), Shandong (Small-tailed-Han sheep, StH) and Jiangsu provinces (Hu sheep, Hu) (Fig. 1a). In this study, we measured modified body mass index (MBMI, kg/m^2^) in female UQ (n = 30), Tan (n = 77), StH (n = 90) and Hu (n = 52) at the age of 375 ± 21 days old. UQ exhibited the significant largest body size with 84.78 ± 7.94 (P < 1.0*10^−7^), while StH and Hu displayed significant smaller body sizes of 72.78 ± 9.46 and 73.19 ± 9.97 respectively (P < 1.0*10^−7^). No significant differences were observed between StH and Hu (P = 0.79). Tan sheep exhibited the smallest body size with 61.54 ± 6.42 (P < 1.0*10^−7^, Fig. 1b). We randomly selected two female sheep each from UQ, Tan, StH and Hu, along with two male sheep from UQ in order to detect DNA variation by Methylated DNA immunoprecipitation – sequencing (MeDIP – seq). These studies investigate whether the extent of genome-wide DNA methylation is related to differences in body mass of these four sheep breeds.

## Results

### Data collection

In total, 76.3G of raw sequence data was obtained from these 10 samples. After filtering out low quality data, 69.3G data were mapped to *Ovis aries Oar*_v3.1^23^ and used to perform a correlation analysis. As we can see, all biological replicates showing high correlation within breeds (Pearson correlation coefficient are 99.4%, 98.0%, 94.3%, 99.8%, 99.3% in UQ females, UQ males, Tan females, StH females and Hu females, respectively. All P values are less than 2.2e-16. Additional Figure 1). Next, 40.0G of uniquely mapped data was divided into 300 bp bins on each chromosome, with regions exhibiting less than 10 reads across all individuals were filtered out to avoid false enrichment. As the limitation of MeDIP-seq, it could not estimate approximate methylation level from 0–100%, then we calculated DNA methylation enrichment score of retained bins for further statistical analysis. Given the high correlation of breed replicates, we next averaged each bins of two sample data and used them reconstruct the genome wide landscape of genetic element distribution.

### Validating MeDIP-seq Data by MassARRAY

In order to validate MeDIP-seq data, two regions with lower methylation and two regions with relatively higher methylation were chosen randomly and examined using Sequenom’s MassARRAY Epi-TYPER protocol. The results from the MassARRY analysis were in consistent with the MeDIP-seq data (Additional Figure 2).

### The DNA methylation landscape of the *Ovis aries*

**Figure 2:**
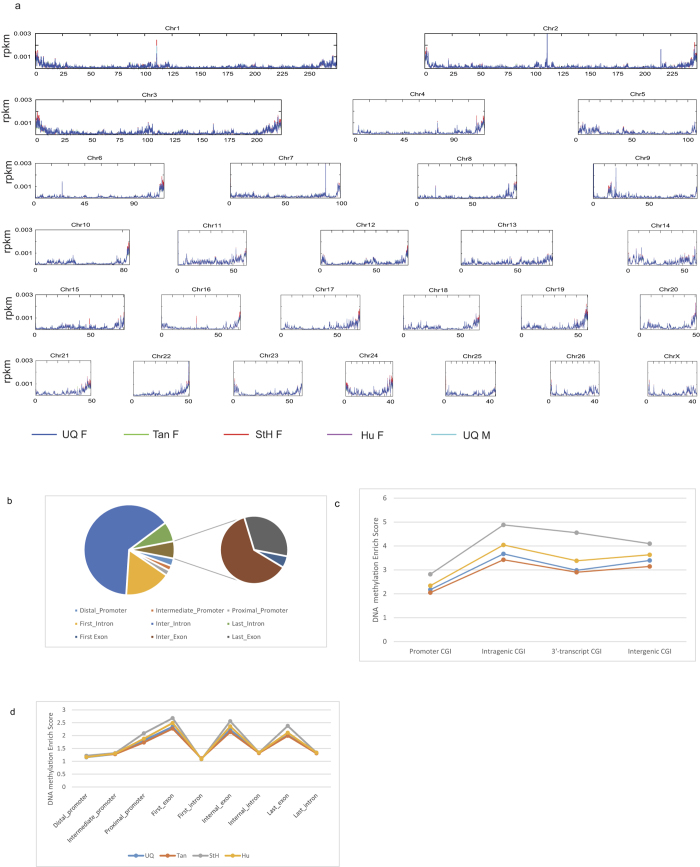
Chromosomal DNA methylation and its distribution patterns among sheep breeds. (**a**) Landscape of DNA methylation across sheep genome. X ray is the chromosome, and Y ray is the 100 K value of RPKM. Each colour line represent one breed as shown in picture. Dark blue is UQ female, light bule is UQ male, green is Tan female, red is StH female, and purple is Hu female. (**b**) The distribution of DNA methylation across genomic elements. (**c**) DNA methylation levels across genomic elements. Promoters were subdivided into three types - Distal promoters (−2200 to −1000), Intermediate promoters (−1000 to −200) and Proximal promoters (−200 to +500). (**d**) CGI DNA methylation pattern in different regions and breeds. Promoter CGI (−1000 to +300), Intragenic CGI (300 bp downstream of TSS and 300 bp upstream of TES), 3′-transcript CGI (300 bp upstream and 1000 bp downstream of TES), and Intergenic CGI (1000 bp downstream of TES to another’s gene 1000 bp upstream of TSS).

100 K windows of RPKM were calculated to obtain an overview of DNA methylation across the sheep genome (Fig. 2a). To get better understanding of DNA methylation level at genomic elements, we next divided the landscape into promoters^24^, exons and introns. Collectively, 2,275,109 bins of 300 bp were obtained, with 876,625 (37.2%) bins located in intergenic regions and 445,692 (19.6%) bins mapped to annotated genes. Among the regions located in genes, approximately 82.3% of windows were found in introns, and 11.7% and 6.0% windows in exons and promoters, respectively (Fig. 2b). Although most introns are methylated, the overall DNA methylation levels in exons are higher than those in introns. As to promoter regions, proximal regions have the highest DNA methylation enrichment scores, intermediate promoters have medium scores, and distal regions of promoters have lowest scores (Fig. 2d). CpG islands (CGI), a 200 bp CpG-enriched region, in which CpG_O/E_ is higher than 0.6. Across all breeds 45,507 windows located in CGI, 887 (1.9%) windows in promoter regions, 23,275 (51.1%) windows in intragenic region, 2,237 (5.0%) windows in 3′-transcript regions, and 19,108 (42.0%) windows in intergenic regions. The CGI enrichment score reached at peak value in intragenic regions, then went down in 3′-transcript regions, and slightly rose again in intergenic regions (Fig. 2c). Among the four breeds, StH had much higher DNA methylation levels, Hu came next, with Tan having the lowest levels of DNA methylation. Across all breeds, exons displayed much higher DNA methylation levels than those of introns, a finding that agree with previous studies in other mammals^11^,^25^,^26^. Methylation levels of promoters in CGI was much lower than other genomic elements, agree with data from previous studies indicate that promoter CGI are rarely methylated^27^. We found that different breeds showed different DNA methylation levels in proximal promoters and exons, suggesting different regulatory role of methylation in those breeds. This findings indicate that these breeds have different genome-wide DNA methylation patterns.

### Hierarchical clustering tree of the four breeds

**Figure 3:**
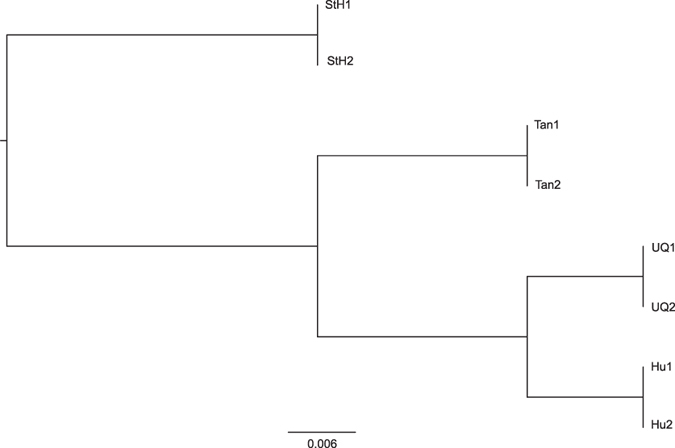
Hierarchical clustering tree of intergenic region among four breeds. 10,061 windows of intergenic regions were used to construct the hierarchical clustering tree by MeV_4.7.3 software.

We next compared each breed bin to obtain DMRs by multiple t-test (P < 0.05), and defined bins that were significant in one comparison (UQ vs. Tan, UQ vs. StH, UQ vs. Hu, Tan vs. StH, Tan vs. Hu, StH vs. Hu) as DMRs. And total 74,348 DMRs were detected, among them, 13,382 DMRs (18.0%) were in annotated genes and 29,359 DMRs (39.5%) were in intergenic region. We first constructed a hierarchical clustering tree of intergenic regions based on a DNA methylation coverage greater than 10 reads in all eight females. StH did not cluster with all other breeds and displayed significant genetic distance based on these datasets (Fig. 3). To confirm the genetic distance, we calculated Fst values in 167 individuals by sequencing mitochondrial D-loop region. Fst value in all breeds were lower than 0.05 (Table 1, P > 0.05), indicating that these breeds have moderate to slight genetic diversity. Interestingly, StH showed higher Fst values compared with other breeds, consistent with our DNA methylation Hierarchical clustering tree, and suggesting that methylation levels are associated with the evolutionary history of these breeds.

### Different methylated genes that were orthologs to human known body size-related genes

GWASs for height, body weight and BMI in humans provide one of the best resources for investigating the genetic variation in body size. We identified 399 DMRs (fold change large than 2 or lower than 0.5 at least in one comparison among six groups) located in 93 genes that were orthologs to human reported body size related genes (Additional Table 1). Among 10 genes identified by 3 or more GWASs (showed in bold), we randomly selected *LTBP1* to see how is the DNA methylation and its RNA expression pattern in skeletal muscle. As skeletal muscle is the most abundant tissue in the sheep body. Pure meat percentage of UQ is 46.0 ± 1.3%, Tan is 36.5%, StH is 43.6 ± 2.2% and Hu is 42.7%^28^, which is in line with their respective BMI phenotypes.

**Figure 4:**
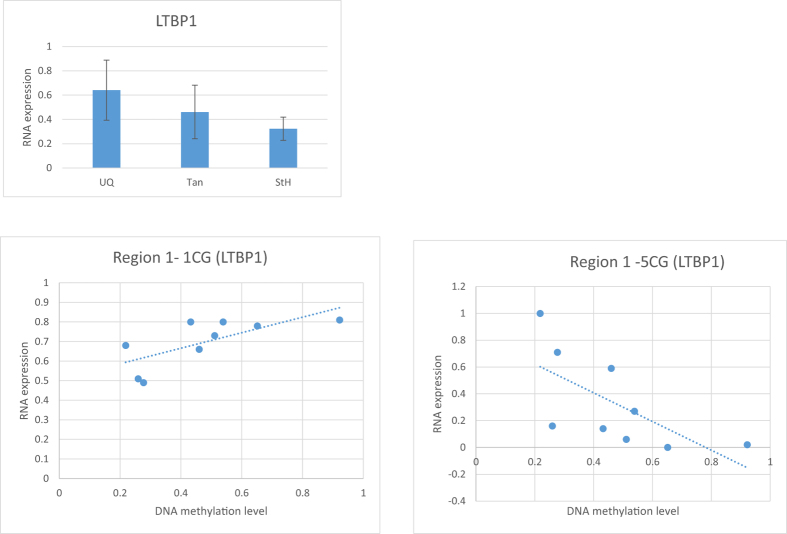
LTBP1 RNA expression and its correlation with DNA methylation level. There is no significant difference observed of the LTBP1 RNA expression level. Region 1 is on Chromosome 3 from 90,573,923 to 90,574,210. 1 CpG and 5 CpG site in region 1 showed significant correlation between DNA methylation level and its RNA expression level (P < 0.05).

Three potential regions were selected from *LTBP1*, no significant difference observed in DNA methylation pattern (Additional Figure 3). Next, we tested its RNA expression levels in three breeds. UQ showed the highest RNA expression, but didn’t reach to significant level. 1CpG in region 1 is significantly positive correlated with RNA expression, while 5CpG in region 1 is significantly negative correlated with RNA expression (Fig. 4).

### Putative DMRs associated with body mass

To search for methylated genes that are putatively associated with body mass variation among breeds, we first identified 8,374 windows of DMRs specific to UQ, 14,866 windows specific to Tan, 10,528 windows specific to StH, and 7,982 windows specific to Hu (Additional Table 2 and Additional Figure 4). Within each there were varying number of differently methylated genes (DMG); specifically, there were 1,054 DMG were observed in UQ, 1,676 DMG in Tan, 1,346 DMG in StH and 980 genes in Hu (Additional Table 3). Next, we used DAVID Functional Annotation Tool and examined if these DMG were significantly enriched to putative gene ontology term^29^. “Developmental process” (GO: 0032502) was found significantly enriched in UQ-specific, Hu-specific, and StH-specific DMG, and “skeletal system development” (GO: 0001501) was identified significantly enriched in Hu (Additional Table 4).

As body size is dimorphic between females and males^30^, We measured body mass in UQ females and males and found as expected that male sheep are bigger in body mass than female sheep (Additional Figure 5a). 86,972 significant DMRs were detected between UQ females and males. Among them, 15,997 DMRs were identified to gene regions (Additional Table 5), and females showed higher methylation levels as compared with males (Additional Figure 5b). Functional enrichments on 1,989 DMG (P < 0.01) by GO analysis (Additional Table 4, Benjamin - adjusted p < 0.05), again yielded “Developmental process” (GO: 0032502) as enriched between female and male methylated genes.

Specific DMG of fatter sheep in UQ, StH and Hu were all enriched in “Developmental process” (GO: 0032502), while there was no Development related GO term observed in the leaner sheep Tan. We next explored genes within the “Development process” category and considered them as candidate genes related to variation in body mass (Additional Table 6), these genes include *BMPR1B, SMAD1, SMURF1, AKT1* and *TSC1*. With STH, Region 4 and Region 10 in *BMPR1B* and *AKT1* respectively were significantly higher compared to other breeds, whereas Region 5 in *BMPR1B* of StH was significantly lower compared with others (P < 0.05). Within the largest sheep UQ, Region 6 and Region 8 in *SMAD1* and *SMURF1* respectively showed highest DNA methylation levels, whereas in the leaner, Tan, displayed highest DNA methylation level among all breeds.

### Candidate DMRs associate with gene expression

**Figure 5:**
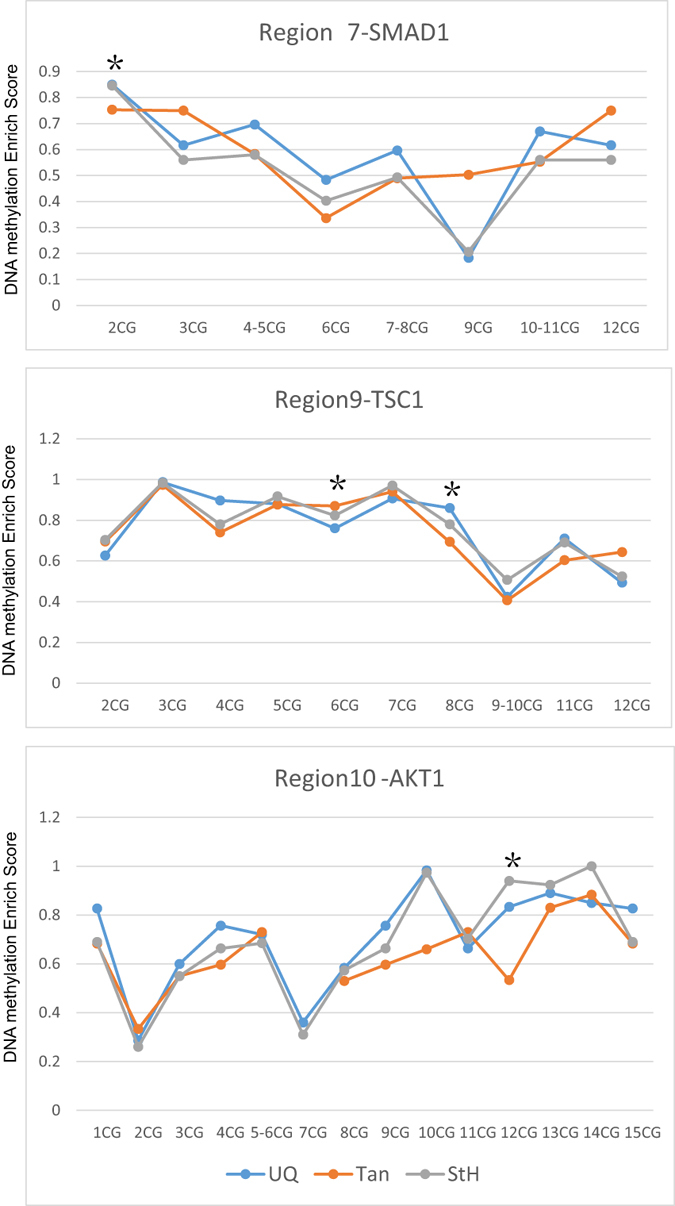
Significant DNA methylation levels of CG sites among breeds. X ray is the CpG locus, and Y ray is the DNA methylation level from 0 (no methylation) to 1(100% methylation). Region 7 is located in *SMAD1* region which is on Chromosome 17 from 12,487,811 to 12,488,083. Region 9 is located in *TSC1* region which is on Chromosome 3 from 3925441 to 3925953. Region 10 is located in *AKT1* region which is on Chromosome 18 from 67,877,536 to 67,878,025.

To scan DNA methylation levels at CpG Site in 7 regions we used MassARRAY method. With the exception of Region 5, which showed similar DNA methylation levels in three breeds, all other regions displayed different methylation tendencies. Region 4 of *BMPR1B* and Region 9 of *TSC1* in fattest body mass UQ sheep showed slightly different methylation profiles compared with other breeds, while Tan sheep displayed mild differences in Region 6 and region 7 of *SMAD1*, Region 8 of *SMURF1* and Region 10 of *AKT1*. Four CpG sites showed significant DNA methylation levels in three breeds. Large UQ and StH showed significant higher methylation levels compared with lean Tan in CpG site 2 (2CG) located in Region 7 of *SMAD1* and 12CG in Region 10 of *AKT1* compared with Tan. UQ sheep also displayed lower methylation levels in 6CG of Region 9, but showed higher methylation levels in 8CG of Region 9 located in *TSC1* (t test, all p < 0.05, Fig. 5 and Additional Figure 6).

**Figure 6:**
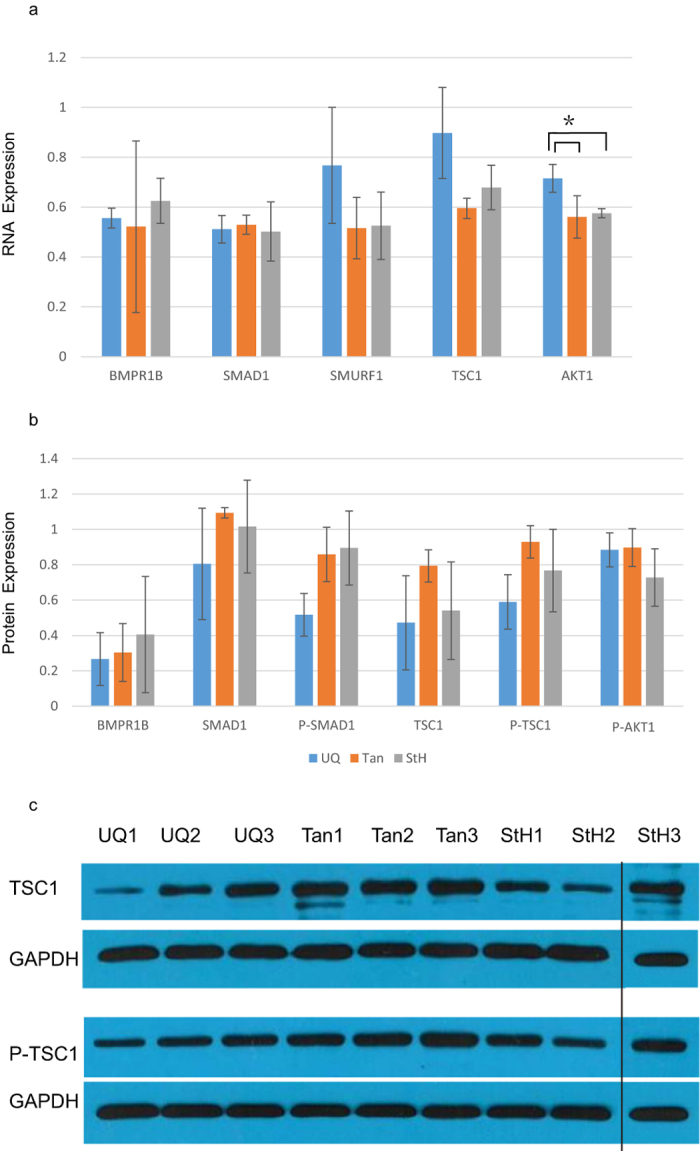
Candidate genes expression leads related to body mass variation. (**a**) RNA expression levels of *BMPR1B*, *SMAD1*, *SUMARF1*, *TSC1*, and *AKT1*. Interestingly, *AKT1* showed significant level in UQ compared with StH and Tan. (**b**) Protein expression levels of BMPR1B, SMAD1, TSC1 and AKT1. There is no significant protein expression level observed among breeds. (**c**) TSC1 and TSC1 (Ser505) protein expression levels among UQ, Tan, and StH.

We used *ACTB* and *RPL19* as reference genes to normalize gene expression. *SMURF1* and *TSC1* in longissimus muscle showed higher expression levels in UQ. *AKT1* expression in UQ was significant higher than Tan and StH (t test, p = 0.029). StH displayed higher *BMPR1B* expression levels compared with other breeds, whereas *SMAD1* in three breeds showed a similar expression level (Fig. 6a).

**Figure 7:**
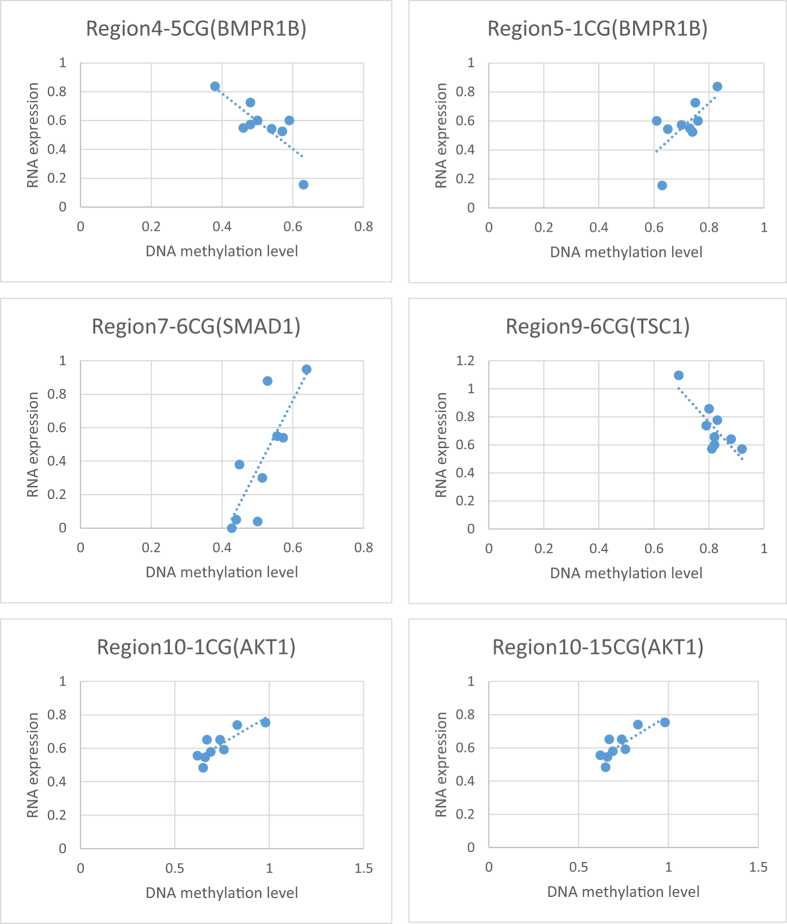
The correlation of DNA methylation and RNA expression. Y ray represents the RNA expression level, and x ray represents the DNA methylation level from 0 to 1. There is a significant correlation observed between DNA methylation level in candidate gene regions and their corresponding RNA expression levels.

We further determined the relationship between gene expression and DNA methylation. We found that six CpG sites in five regions were significantly correlated with their RNA expression levels (p < 0.05, Fig. 7). 5CpG of Region 4 was negatively correlated with *BMPR1B* expression, while 1CpG of Region 5 was positively correlated with *BMPR1B*. 6CpG of Region 9 were negatively correlated with *TSC1*, whereas 1CpG and 15CpG of Region 10 and 6CpG of Region 7 were positively correlated with *AKT1* and *SMAD1* expression. No significant correlation observed in *SMURF1* regions.

As we found that there were significant correlations between GpG sites methylation and RNA expression for *BMPR1B*, *SMAD1*, *TSC1* and *AKT1*, we further examined whether this correlation is reflected at the protein level. Using western blot analysis, we found no significant differences protein expression among breeds, and no significant correlations between the methylation of CpG sites and the level of protein expression in BMPR1B, SMAD1, SMAD1 (Ser465) and AKT1 (Thr308). Furthermore, their RNA expression levels had no significant correlation with protein levels. However, *TSC1* showed significant inverse correlation between protein expression and RNA level (P < 0.05). Besides, 11CpG in Region 9 had significant negative correlations with TSC1 protein levels, and 6CG in Region 6 was positively correlated with TSC1 and TSC1 (Ser505) level. Strikingly, TSC1 protein levels in lean Tan sheep were approximately 168% and 146% greater than in larger UQ and StH sheep, respectively, whereas TSC1(Ser505) was 158% and 121% greater than found in UQ and StH, respectively (Fig. 6b,c).

## Discussion

In this study we investigated whether body size variation in sheep is related to DNA methylation patterns. Mongolian sheep were used for this study as this breed has reduced genetic variation^28^. Indeed, the Fst values of the mitochondria D-Loop region confirmed that the four breeds examined here shared the same genetic origin and have reduced genetic variation. Interestingly, StH showed higher Fst values and further hierarchical clustering of DNA methylation compared with other breeds. This suggests that DNA methylation is involved in breed evolution. Using similar techniques, Irene *et al.* also found that methylation patterns in human, chimpanzee, gorilla, and orangutan track their phylogenetic relationships^31^,^32^, suggesting that DNA methylation participates in phenotype variation among species. In addition, a high quality sheep genomic landscape of DNA was obtained in this study, and all breeds showed similar pattern except StH showed higher DNA methylation density at particular regions including several sub-telomeric regions. A small region at *AKT1* tested in this study, which located at 18 chromosome and close to the telomere, showed that StH had higher DNA methylation level compared to UQ and Tan (Additional Figure 7). Higher frequency methylation in telomere regions was previously reported as playing important role in controlling of telomere length and recombination^33^.

Remarkably, in this study 399 DMRs were identified in sheep that located in 93 human orthologs associated with weight, height and BMI, which indicating that DNA methylation plays a role in those gene expression. Indeed, as we tested in LTBP1 region, significant correlation was observed between DNA methylation and RNA expression. These regions provide us with a new way of understanding the body size- related GWASs.

Putative regions that we assumed they plays important role in body size, enriched in “development process” (GO: 0032502). We detected DNA methylation levels in 7 regions of 5 genes and its gene expression levels. Importantly, genes involved in the bone morphogenetic protein (BMP) signaling pathway including *BMPR1B, SMAD1*and *SMURF1*, are involved in many cellular processes including cell growth, cell differentiation and apoptosis. Activation of type-I BMP receptors phosphorylates SMAD proteins, which then form heteromeric complexes with SMAD4 ultimately regulate target gene transcription. Interestingly, in mice lacking Bone morphogenetic protein receptor type-1B (*BMPR1B*) the main receptor within the BMP pathway or having a truncated receptor, display lower body weight compared with wild-type mice^34^. *SMAD1* has been shown interact with osteoblast and chondrocyte marker gene - *HOXC8*^35^. Specific E3 ubiquitin protein ligase 1 (*SMURF1*), an ubiquitin ligases, plays negative roles in BMP signaling by targeting the TGF-β type I receptor via downstream SMAD7^36^. *AKT1* encodes a serine-threonine protein kinase, which plays important role in transmitting growth promoting signals. Furthermore, it is a central player in phosphorylating and activating downstream substrates. Interestingly, mice deficient in *AKT1* have a 20% reduction in body weight compared with WT control^37^. In addition, pathways downstream of PI3K and AKT in Drosophila were indicated a role for these gens in the control of cell size^38^,^39^. Tuberous sclerosis 1 (*TSC1*) which forms a heterodimeric complex with *TSC2* (TSC1/TSC2), is an upstream negative regulator of the *mTOR* (two functional complexes, mTORC1 and mTORC2), which plays a key role in a wide array of cellular processes, including cell growth^40^. Mouse embryonic fibroblast cells lacking *TSC1* fail to alter mTORC1 activity in mouse embryonic fibroblast cells^41^.

We previously found that Myostatin mutant sheep (Texel) have significantly higher AKT1 protein levels in the longissimus muscle compared to UQ sheep, specifically at embryonic stage^42^. *AKT1* may play a role in DNA methylation via stabilizing DNA methyltransferase (DNMTs)y and maintaining DNA methylation and chromatin structure^43^,^44^. In breast cancer, aberrant *AKT1* activation has been reported to be associated with promoter hypermethylation^45^. Most recently, *AKT1* was shown to regulate the HoxC gene expression by epigenetic modifications^46^. Furthermore, a inverse correlation between DNA methylation status and HOX expression was observed in *AKT1*-null murine embryo fibroblasts indicating *AKT1* has a role in *HoxC* expression^47^. Phosphorylated AKT1 can activate a set of substrates, including proteins that inhibit apoptosis, induce protein synthesis, gene transcription and cell proliferation^48^,^49^. We found that larger UQ sheep displayed significant higher *AKT1* RNA expression levels, methylation levels at some CpG sites positively correlated with *AKT1* expression level. However, AKT1 (Thr308) content in UQ sheep displayed the same levels as lean body size Tan sheep indicating *AKT1* methylation has a close relationship with transcriptional control of RNA expression. Further research is needed to investigate the relationship between *AKT1* the regulation of body mass.

TSC, an autosomal dominant tumor suppressor gene, was observed in the development of benign tumors in multiple organ systems indicating that it influences cell and tissue growth. Studies on the Drosophila orthologs of TSC1 and TSC2 reveal that they regulate many cellular processes, including cell size, cellular proliferation and cell cycle^50^,^51^,^52^. Indeed, overexpression of both dTSC2 and dTSC1 resulted in decreased cell size and number, whereas cells mutant for dTSC1 were greatly increased in size^51^. While *TSC1*-knockout mouse (TSC1^−/−^) induced embryonic lethal, embryos were smaller compared to the controls (not individuals)^53^. This is in line with evidence revealing that *TSC1* null murine embryo fibroblasts exhibit slower growth rates compared with TSC1^+/+^ and TSC1^+/−^ mice^54^. Furthermore, TSC1-deficient in astrocytes showed larger soma size^55^, These data agree with the roles of TSC in body size control; larger UQ and StH exhibit lower levels of TSC1 and P-TSC1 compared with lean Tan sheep. Indeed, 6CG site methylation significantly correlated with both *TSC1* RNA expression and protein expression. This indicates that most of DMRs listed in this study may be directly related to body mass variation in sheep breeds.

Due to the limitations such as small sample size, CpG site methylation level and gene expression did not reach significant levels, as evidence by our studies on *SMURF1*. On the other hand, significant DNA methylation levels were observed in *SMAD1*, although *SMAD1* RNA expression level was similar across the three breeds examined. There is significant correlation between DNA methylation of *BMPR1B* region and RNA expression, however there is no significant DNA methylation variation detected among breeds.

We firstly constructed a DNA methylation maps for different breeds of Mongolian sheep and in that context identified DMRs located in human orthologs which associated with body size and established a list of DMRs potentially associated with body size variation. We further examined seven regions in five genes to study the relationship between DNA methylation and MBMI, and found that two genes (AKT1 and TSC1) showed strong correlation between DNA methylation and expression. Further study is needed to determine the extent to which the methylation of these genes is correlated with the body size or fat mass of different sheep breeds.

## Methods

### Ethics Statement

All blood and muscle samples were carried out in accordance with Guidelines for the Care and Use of Experimental Animals established by the Ministry of Agriculture of China. Blood and muscle sampling was approved by the Biological Studies Animal Care and Use Committee, Peoples Republic of China. The feeding was in line with the Instructive Notions with Respect to Caring for Laboratory Animals that was published in 2006 by the Science and Technology Department of China (Approval No. S20072911).

### Sample Collection

Jugular venous blood of ten sheep was used to investigate whole genome DNA methylation. Blood samples were collected and stored at −80 °C until genomic DNA isolation. Nine longissimus muscle were rapidly dissected from UQ, Tan and StH sheep (each were three replicates), and immediately frozen in liquid nitrogen and stored at −80 °C until DNA and RNA extraction.

### DNA and RNA Extraction

Blood genomic DNA and muscle genomic DNA were isolated according to the manufacturer’s protocol of TIANamp Blood DNA Kit (DP318) and TIANamp Genomic DNA Kit (DP304-02) (TIANGEN, Beijing) respectively, Total RNA was extracted from longissimus muscle by Trizol method (Invitrogen). DNA and RNA integrity and quality were evaluated by agarose gel electrophoresis and Nano-Drop spectrophotometer.

### Mitochondria Control Region Sequencing and Analysis

The mitochondrial D-loop region (about 1200 bp) was amplified with previously published primers: Forward 5′-CTCACCATCAACCCCCAAAGC-3′; Reverse 5′- TCATCTAGGCATTTTCAGTG-3′. PCRs volume (25-uL) contained 50 ng DNA, 10 uM Primers, 1U taq polymerase, 2.5 ul 10* Taq Buffer (ET101, TIANGEN, Beijing) and 2.5 mM dNTP. PCR cycling conditions were as follows: 95 °C for 5 min, followed by 35 cycles of 94 °C for 30 s, 55 °C for 30 s, 72 °C for 60 s, final extension at 72 °C for 8 min. PCR products were purified and then sent to Invitrogen to be sequenced. 167 novel sequences were obtained and edited by DNASTAR software. As repeat sequences of D-Loop region have different evolution patterns, we manually deleted repeat sequences. Haplotypes of all sequences were analyzed by DNASP 5.0, and Fst values were calculated by Arlequin3.5.1.2 software.

### Preparation for MeDIP-Seq

Prior to the MeDIP assay, genomic DNA was sonicated to produce DNA fragments ranging from 100 to 500 bp. Next, Illumina sequencing primer adaptors were ligated to the DNA. Subsequently, adaptor-ligated DNA was used for MeDIP enrichment based on the manufacturer’s recommendation of Magnetic Methylated DNA Immunoprecipitation kit (Diagenod, Liège, Belgium), and qualifying DNA was used for PCR amplification. After agarose gel electrophoresis was performed, the bands (220 ~ 320 bp) were next gel purified with QIAquick Gel Extraction Kit (Qiagen, Valencia, CA, USA). Quant-iTTM dsDNA HS Assay Kit (Invitrogen, Carlsbad, CA, USA) was used to quantify purified DNA libraries. To check the efficiency of enrichment q-PCR was performed. qPCR concentration for UQ female are 78.28 and 91.92, for UQ male are 45.14 and 50.94, for Tan female are 89.88 and 86.07, for StH are 81.67 and 80.23, for Hu are 47.9 and 80.66, respectively. Finally, 50-bp reads were generated on the Illumina Hiseq 2000 (Illumina, San Diego, CA, USA) by the Beijing Genomics Institute (BGI, Shenzhen, Guangdong, China).

### Bioinformatic Analysis

Clean data (i.e. not exceeding 2 bp mismatches) was next mapped to the genome of *ovis aries*^23^ by SOAP aligner v 2.21 (http://soap.genomics.org.cn/). 300 bp windows were constructed to calculate local DNA methylation enrich scores, totally approximately 8 million bins enrichment score was next calculated by following formula:

Enrichment Score = reads number of 300 bp*10^9^/(300*total unique mapped reads).

All statistical analyses in this study was analysed by R software. DAVID Functional Annotation Tool (http://david.abcc.ncifcrf.gov/) was used for subsequent gene ontology and pathway analyse.

### MassArray

The DNA isolated from longissimus muscle was treated with bisulphite using EZ DNA methylation-Gold Kit (ZYMO Research). Quantitative methylation levels of 14 DMRs was performed on the Sequenom MassARRAY platform (Bio Miao Biological Technology, Beijing, China) as reported previously^56^. EpiDesigner software (Sequenom) was used to design PCR primers (Additional Table 7). The DNA methylation level of each CpG site or multiple CpG sites was analysed with EpiTyper software v1.0 (Sequenom).

### Quantitative PCR

RNase-free DNase I (TaKaRa) was used to avoid genomic DNA contamination. cDNA was synthesised using PrimeScript RT Master Mix kit (TaKaRa). To obtain accurate mRNA expression levels, each sample was performed in triplicate with appropriate negative controls. q-PCR was next performed to measure mRNA levels using the Real-Time PCR detection system (ABI). Primers of 9 target genes and 5 housekeeping genes are listed in Additional Table 8. After analysing GAPDH, B2M, ACTB, RPL19 and HPRT housekeeping genes using geNorm software, and ACTB and RPL19 were more stable than others, then they were used as reference genes to normalize gene expression. Relative expression levels were calculated by E^−ΔΔCT^ method as described previously^57^.

### Western blot

Protein concentrations were detected by the BCA Kit according to the manufacturer’s protocol (Thermo Scientific, Brookfield, WI, USA). 20 μg of protein per sample was run on 8% or 12% sodium dodecyl sulfate polyacrylamide electrophoresis gels (SDS-PAGE) gels. Separated proteins were then transferred onto nitrocellulose membranes, which were incubated overnight with one of the following primary antibodies: BMPR1B(1:1000, 40–9400, Invitrogen), TSC1(1:1000, bs-3837R, Bioss), TSC1/Ser505 (1:1000, bs-5600R, Bioss), AKT1/Thr308 (1:1000, 2965, CST), SMURF1(1:1000, bs-9391R, Bioss), SMAD1(1:1000, 6944, CST), SMAD1/Ser465(1:500, sc-101800, Santa). Anti- GAPDH antibodies (1:4000, 042, TDY) were used as a control. The membranes were next washed (5 × 3 min) with TBST and then incubated with secondary antibodies [goat anti-rabbit IgG (H + L), HRP (Jackson), 1:20000] for 40 minutes at room temperature. The membranes were then washed above, and HRP activity was visualized using the Fusion X7 imaging system with the Bio1D software (Vuilbert-Lourmat, Marne-la-Vallée, France).

## Additional Information

**Accession codes:** The high-throughput sequencing data has been deposited in NCBI’s Gene Expression Omnibus under GEO Series accession numbers GSE62345 GEO Submission.

**How to cite this article**: Cao, J. *et al.* DNA methylation Landscape of body size variation in sheep. *Sci. Rep.* **5**, 13950; doi: 10.1038/srep13950 (2015).

## Supplementary Material

Supplementary Information

## Figures and Tables

**Figure 1:**
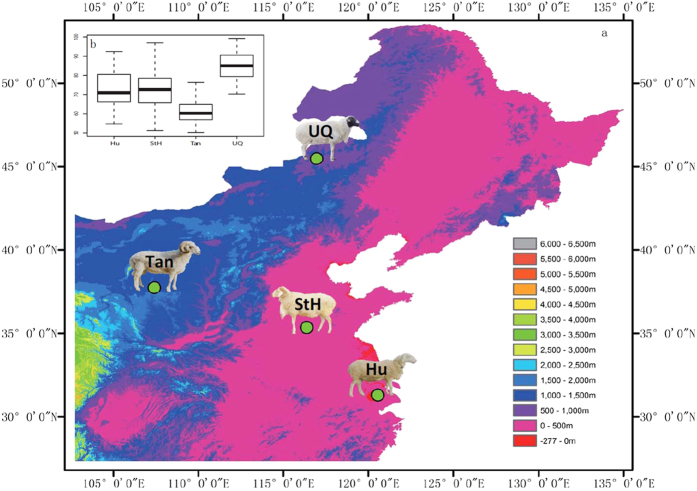
Geographical location and MBMI of the sample. (**a**) Geographical location of breeds. UQ - Dong Ujumqin Qi, Inner Mongolia Autonomous Region, China (N45° 30′, E116° 57′). Tan - Yanchi, Ningxia Hui Autonomous Region, China (N37° 46′, E107° 24′). StH - Jiaxiang, Shandong Province, China (N35° 23′, E116° 21′). Hu - Suzhou, Jiangsu Province, China (N31° 18′, E120° 34′). This map is generated by ArcGIS 10.1 software, and different colour represent different latitude as shown in map. StH and Hu live in lower latitude compared to UQ and Tan. (**b**) The comparison of MBMI among UQ, Tan, StH, and Hu. MBMI = body weight/body length^2^. Body length is measured from shoulder to ischium.

**Table 1:** Pairwise FSTs for four sheep breeds.

	Hu	Tan	UQ	StH
Hu		0.33333 ± 0.0560	0.13514 ± 0.0412	0.07207 ± 0.0264
Tan	0.00258		0.70270 ± 0.0522	0.07207 ± 0.0227
UQ	0.02145	−0.01169		0.06306 ± 0.0194
StH	0.03375	0.03150	0.03323	

Lower triangular matrix represents Fst value among breeds, upper triangular matrix are P values. We found 97 haplotypes among 167 individuals (UQ = 46, Tan = 37, StH = 42 and Hu = 42), as calculated by Arlequin3.5.1.2 software.
