# Supplementary material for: DNA methylation Landscape of body size variation in sheep
Source: Sci Rep. 2015 Oct 16;5:13950. doi: 10.1038/srep13950 (PMC4607979; doi:10.1038/srep13950)
Supplement: Supplementary Information [file srep13950-s1.pdf]

DNA methylation Landscape of body size variation in sheep

Jiaxue Cao, Caihong Wei, Dongming Liu, Huihua Wang, Mingming Wu, Zhiyuan Xie, Terence D. Capellini, Li Zhang, Fuping Zhao, Li Li, Tao Zhong, Linjie Wang, Jian Lu, Ruizao Liu, Shifang Zhang, Yongfei Du, Hongping Zhang, Lixin Du

Additional Table 1. 399 DMRs were orthologs to human know body-size related genes

Chr	Start	End	UQ	Tan	StH	Hu	Human ortholog genes	Trait	Reference
chr5	14846701	14847000	0.15806	0.327761	0.64634	0.365254	ADAMTS10	Height	1, 2
chr18	5511301	5511600	1.00818	0.842814	0.42359	0.851628	**ADAMTS17**	Height	3,2,1
chr18	5528401	5528700	0.45333	0.256607	0.296422	0.62847	**ADAMTS17**	Height	3,2,1
chr18	5542201	5542500	0.69207	0.490722	0.896296	0.380039	**ADAMTS17**	Height	3,2,1
chr18	5565301	5565600	0.5598	0.775338	0.526202	0.273704	**ADAMTS17**	Height	3,2,1
chr18	5569501	5569800	2.74742	2.760719	1.369001	2.058969	**ADAMTS17**	Height	3,2,1
chr18	5569801	5570100	2.26335	2.165317	0.965586	1.597867	**ADAMTS17**	Height	3,2,1
chr18	5637001	5637300	0.1888	0.488883	0.135932	0.106335	**ADAMTS17**	Height	3,2,1
chr18	5654701	5655000	4.46521	1.493244	4.068385	4.336798	**ADAMTS17**	Height	3,2,1
chr18	5663401	5663700	0.7453	0.516892	0.699837	0.217914	**ADAMTS17**	Height	3,2,1
chr18	5727601	5727900	0.69207	0.722998	0.258719	0.801083	**ADAMTS17**	Height	3,2,1
chr18	5748601	5748900	0.79524	0.749168	0.296422	0.653743	**ADAMTS17**	Height	3,2,1
chr18	5749201	5749500	1.45656	0.786796	3.312404	1.958823	**ADAMTS17**	Height	3,2,1
chr18	5780701	5781000	0.26618	0.116138	0.205222	0.527379	**ADAMTS17**	Height	3,2,1
chr24	40469701	40470000	0.9275	0.747329	1.734713	1.206395	AMZ1	Height	3
chr24	40472401	40472700	0.16301	0.09	1.243566	0.0863	AMZ1	Height	3
chr24	40479001	40479300	0.40009	0.676599	2.677475	0.558841	AMZ1	Height	3
chr11	6672901	6673200	0.18385	0.162961	0.370094	0.633714	ANKFN1	Height	4
chr11	6682801	6683100	0.18715	0.539384	0.156108	0.471589	ANKFN1	Height	4
chr11	6708301	6708600	1.46316	0.844653	0.860326	0.694747	ANKFN1	Height	4
chr11	6708601	6708900	1.00983	0.730354	0.613019	0.466345	ANKFN1	Height	4

chr11	6748501	6748800	0.1855	0.352092	0.185047	0.62847	ANKFN1	Height	4
chr3	38981101	38981400	0.48077	0.350253	0.267483	0.223158	ANTXR1	Height	5
chr3	39038401	39038700	0.23709	0.653683	0.296422	0.339981	ANTXR1	Height	5
chr3	39111901	39112200	1.62782	0.530189	1.1059	0.968451	ANTXR1	Height	5
chr3	39112501	39112800	0.1855	0.114299	0.448148	0.223158	ANTXR1	Height	5
chr21	17776501	17776800	0.3743	0.795991	0.234162	0.370498	AQP11	Weight	6
chr15	50460901	50461200	0.1855	0.492561	0.205222	0.441072	ARAP1	BMI	7
chr15	50469001	50469300	0.34521	0.721159	0.205222	0.36001	ARAP1	BMI	7
chr15	50474701	50475000	0.371	0.492561	0.242925	0.471589	ARAP1	BMI	7
chr2	248415001	248415300	1.09216	0.515053	0.641958	0.790595	ATP13A2	Height	8
chr2	248424301	248424600	0.58559	0.783118	1.435643	0.339981	ATP13A2	Height	8
chr24	25949101	25949400	0.45333	0.324083	0.884884	0.770566	ATP2A1	Weight	9
chr4	15192601	15192900	0.37265	0.516892	1.049755	0.887389	C1GALT1	BMI	10
chr5	69261301	69261600	0.26618	0.541223	0.238543	0.136852	C1QTNF2	Weight	6
chr23	33608401	33608700	0.34521	0.305269	0.258719	0.714776	**CABLES1**	Height	3,2,1,8
chr23	33611101	33611400	2.09869	1.245407	0.748952	1.064299	**CABLES1**	Height	3,2,1,8
chr23	33636301	33636600	1.01148	2.305362	0.855945	1.236911	**CABLES1**	Height	3,2,1,8
chr23	33648001	33648300	0.34521	0.421407	0.180665	0.557895	**CABLES1**	Height	3,2,1,8
chr3	186150301	186150600	0.3743	0.913968	0.399033	0.435829	CCDC91	Height	3,1
chr1	228527701	228528000	0.50492	0.370906	0.29204	0.608441	CCNL1	Weight	11, 12
chr14	8581501	8581800	0.66462	0.443899	0.271865	0.638958	CDH13	Height	8
chr14	8649001	8649300	0.10812	0.470069	0.29204	0.217914	CDH13	Height	8
chr14	8661001	8661300	0.3677	0.93646	0.300804	0.603197	CDH13	Height	8
chr14	8769601	8769900	0.29032	0.60686	0.570935	1.191609	CDH13	Height	8
chr14	8780701	8781000	0.26453	0.891476	0.472705	0.669474	CDH13	Height	8
chr14	8784601	8784900	0.0516	0.258446	0.247307	0.572681	CDH13	Height	8
chr14	8801701	8802000	2.91538	1.184863	5.530319	4.025442	CDH13	Height	8
chr14	8824801	8825100	0.10812	0.35577	0.461293	0.745293	CDH13	Height	8
chr14	8825701	8826000	0.1888	0.513214	0.42359	0.471589	CDH13	Height	8
chr14	8831701	8832000	0.15806	0.46823	0.185047	0.167369	CDH13	Height	8
chr14	8890501	8890800	3.55855	2.161639	1.747858	1.405226	CDH13	Height	8

chr14	8910001	8910300	0.29197	0.162961	0.213986	0.491618	CDH13	Height	8
chr14	9022501	9022800	0.13062	0.0936	0.497263	1.140118	CDH13	Height	8
chr14	9034501	9034800	0.26453	0.46823	0.267483	0.0305	CDH13	Height	8
chr14	9040501	9040800	0.23709	0.605021	0.164871	0.248431	CDH13	Height	8
chr14	9083101	9083400	0.89841	0.350253	0.193811	0.203129	CDH13	Height	8
chr14	9114301	9114600	0.63388	0.438382	0.855945	0.061	CDH13	Height	8
chr14	9121501	9121800	1.5098	0.58069	1.016434	0.365254	CDH13	Height	8
chr14	9121801	9122100	2.0663	0.408534	1.134839	0.223158	CDH13	Height	8
chr14	9136201	9136500	0.1855	0.443899	0.16049	0.217914	CDH13	Height	8
chr14	9185701	9186000	0.77275	0.352092	0.258719	0.466345	CDH13	Height	8
chr14	9230701	9231000	0.47582	0.840975	0.555141	0.365254	CDH13	Height	8
chr14	9309901	9310200	0.76945	1.361545	0.575316	0.350468	CDH13	Height	8
chr14	9310201	9310500	0.61139	1.23989	0.501644	0.350468	CDH13	Height	8
chr14	9316501	9316800	0.72611	2.600021	0.748952	0.689503	CDH13	Height	8
chr14	9316801	9317100	0.24039	0.865306	0.374476	0.455858	CDH13	Height	8
chr14	9321301	9321600	0.26453	0.870823	0.209604	0.101092	CDH13	Height	8
chr14	9334501	9334800	0.40009	0.870823	0.798066	1.206395	CDH13	Height	8
chr14	9359401	9359700	0.13392	0.466391	0.209604	0.36001	CDH13	Height	8
chr14	9370501	9370800	0.26618	0.0675	0.539347	0.0863	CDH13	Height	8
chr14	9416701	9417000	0.0807	0.443899	0.140314	0.644201	CDH13	Height	8
chr14	9423601	9423900	1.5164	3.00997	2.937928	4.363531	CDH13	Height	8
chr14	9433201	9433500	0.15971	0.612377	0.135932	0.638958	CDH13	Height	8
chr14	9433501	9433800	0.0807	0.678014	0.102611	0.689503	CDH13	Height	8
chr14	9435001	9435300	0.1855	0.372745	0.399033	0.466345	CDH13	Height	8
chr14	9482401	9482700	1.75843	0.906612	1.076961	1.841054	CDH13	Height	8
chr14	9502801	9503100	0.93245	0.256607	0.543729	0.638958	CDH13	Height	8
chr14	9520501	9520800	0.63388	0.0712	0.0579	0.0505	CDH13	Height	8
chr14	9536701	9537000	0.10977	0.726676	0.13155	0.223158	CDH13	Height	8
chr14	9609601	9609900	0.37265	0.419568	0.127169	0.278948	CDH13	Height	8
chr20	35767801	35768100	0.66132	0.352092	0.764745	0.0758	CDKAL1	Weight	11
chr20	35770501	35770800	0.24039	0.539384	0.345537	0.298977	CDKAL1	Weight	11

chr20	35773801	35774100	0.9275	0.305269	0.662134	0.597954	CDKAL1	Weight	11
chr20	35808901	35809200	0.10812	0.187292	0.419209	0.167369	CDKAL1	Weight	11
chr20	35913001	35913300	0.3743	0.679853	0.563905	0.993724	CDKAL1	Weight	11
chr20	35913901	35914200	1.35174	0.228598	0.173635	0.136852	CDKAL1	Weight	11
chr20	35914201	35914500	1.27931	0.116138	0.144696	0.106335	CDKAL1	Weight	11
chr20	35968801	35969100	0.26618	0.561876	0.238543	0.380039	CDKAL1	Weight	11
chr20	36025201	36025500	0.3743	1.170575	0.65337	1.277915	CDKAL1	Weight	11
chr20	36035101	36035400	0.24039	0.211623	0.477087	0.354766	CDKAL1	Weight	11
chr20	36186301	36186600	0.31777	0.938299	0.36133	0.446316	CDKAL1	Weight	11
chr20	36192901	36193200	0.55155	1.666814	1.531224	0.77581	CDKAL1	Weight	11
chr20	36193201	36193500	0.65967	1.142566	1.308474	0.329493	CDKAL1	Weight	11
chr20	36217801	36218100	1.68105	1.155439	0.559523	1.80005	CDKAL1	Weight	11
chr20	36244501	36244800	0.21294	0.374584	0.510408	0.192641	CDKAL1	Weight	11
chr20	36279301	36279600	0.16136	0.614216	0.0535	0.162125	CDKAL1	Weight	11
chr20	36332401	36332700	0.32107	0.492561	0.209604	0.248431	CDKAL1	Weight	11
chr5	27884401	27884700	0.5565	0.209784	0.345537	0.699991	CEP120	Height	1
chr5	27898201	27898500	0.26948	0.676175	0.555141	0.162125	CEP120	Height	1
chr2	143585101	143585400	0.36935	1.056276	0.36133	0.461102	COBLL1	BMI	7
chr2	143602801	143603100	0.26288	0.499917	1.257626	0.142096	COBLL1	BMI	7
chr2	143661301	143661600	0.48077	0.237793	0.0781	0.111579	COBLL1	BMI	7
chr2	9417901	9418200	3.21829	2.001932	2.074953	0.978939	COL27A1	Height	2
chr2	9427801	9428100	0.9275	0.895154	0.891914	0.228402	COL27A1	Height	2
chr2	9444901	9445200	1.83416	1.114557	1.707507	2.287371	COL27A1	Height	2
chr2	9447901	9448200	0.74035	0.393398	1.221657	0.826355	COL27A1	Height	2
chr2	9490201	9490500	0.60809	0.350253	0.0868	0.111579	COL27A1	Height	2
chr2	9495901	9496200	1.25352	0.726676	0.613019	0.801083	COL27A1	Height	2
chr8	20329501	20329800	0.92585	1.40469	0.550759	0.679016	DCBLD1	Height	3
chr8	20331901	20332200	0.45168	0.376423	0.530584	0.217914	DCBLD1	Height	3
chr8	20355301	20355600	0.70032	0.678014	0.36133	2.614972	DCBLD1	Height	3
chr8	20355601	20355900	0.16301	0.211623	0.234162	1.72234	DCBLD1	Height	3
chr8	20379601	20379900	0.21459	0.449416	0.276246	0.923149	DCBLD1	Height	3

chr5	18930601	18930900	0.15971	0.801508	0.325361	0.197885	**DOT1L**	Height	3,1,4
chr17	60758401	60758700	0.35016	0.929104	0.218368	0.192641	DTX1	BMI	10
chr3	68118901	68119200	0.26618	0.977766	0.572668	0.304221	**EFEMP1**	Height	3,1, 13,8, 14, 15,2, 16
chr3	68157001	68157300	0.15971	0.471908	0.13155	0.136852	**EFEMP1**	Height	3,1,13,8,14,15,2,16
chr13	64877101	64877400	0.32107	0.162961	0.448148	0.167369	EPB41L1	Height	3
chr1	252172501	252172800	1.59708	2.106045	5.699572	1.831513	EPHB1	BMI	10
chr1	252177301	252177600	0.53236	0.372745	0.267483	0.217914	EPHB1	BMI	10
chr1	252189601	252189900	0.23874	0.279099	0.316597	0.633714	EPHB1	BMI	10
chr1	252264901	252265200	0.43084	1.140727	1.056785	1.420011	EPHB1	BMI	10
chr1	252360901	252361200	0.39844	1.498336	0.213986	0.461102	EPHB1	BMI	10
chr1	252361201	252361500	0.16136	1.151761	0.0491	0.237944	EPHB1	BMI	10
chr1	252405001	252405300	0.26618	0.655522	0.432354	0.613685	EPHB1	BMI	10
chr1	252426301	252426600	1.08556	2.637225	1.740828	1.942146	EPHB1	BMI	10
chr1	252426601	252426900	0.40174	0.63303	0.666516	0.243187	EPHB1	BMI	10
chr1	252465301	252465600	0.53071	0.299752	0.530584	0.851628	EPHB1	BMI	10
chr1	252465601	252465900	1.93238	0.706023	1.484758	1.698959	EPHB1	BMI	10
chr8	75513601	75513900	0.87427	0.985122	0.798066	0.421043	ESR1	Height	3,1
chr8	75599101	75599400	0.0807	0.516892	0.45253	0.496862	ESR1	Height	3,1
chr8	75622801	75623100	1.76008	2.084544	0.793684	1.176824	ESR1	Height	3,1
chr8	75649201	75649500	0.15806	0.541223	0.111375	0.273704	ESR1	Height	3,1
chr9	59009401	59009700	0.0823	0.1648	0.0737	0.593656	EXT1	Height	14
chr9	59099401	59099700	0.31777	0.189131	0.506026	0.243187	EXT1	Height	14
chr9	59112301	59112600	0.47912	0.608699	0.29204	1.13582	EXT1	Height	14
chr9	59125801	59126100	1.43572	1.033784	0.604256	0.815868	EXT1	Height	14
chr9	59140201	59140500	0.47582	0.985122	0.534965	0.633714	EXT1	Height	14
chr9	59159701	59160000	0.29197	0.230437	0.189429	0.68426	EXT1	Height	14
chr9	59179501	59179800	0.23709	0.58069	0.637577	0.806326	EXT1	Height	14
chr9	59179801	59180100	1.13385	2.335634	2.989691	2.47047	EXT1	Height	14
chr9	59212201	59212500	0.53236	0.470069	0.58408	0.192641	EXT1	Height	14
chr9	59213701	59214000	0.74365	0.307108	0.341155	0.471589	EXT1	Height	14
chr9	59214001	59214300	0.63718	0.348414	0.267483	0.466345	EXT1	Height	14

chr9	59247301	59247600	0.63883	0.513214	0.896296	0.192641	EXT1	Height	14
chr9	59279701	59280000	0.5565	0.515053	0.267483	0.253675	EXT1	Height	14
chr21	39658501	39658800	0.69207	0.421407	0.267483	0.298977	FADS1	BMI	7
chr7	47862901	47863200	0.24039	0.0936	0.501644	0.0253	FAM81A	BMI	10
chr7	47882701	47883000	0.26453	0.964469	0.526202	0.273704	FAM81A	BMI	10
chr16	3715801	3716100	0.82268	0.372745	0.555141	1.115791	FBXW11	Height	3,1
chr16	3720301	3720600	0.15806	0.44206	0.213986	0.278948	FBXW11	Height	3,1
chr16	3736801	3737100	0.77275	0.60686	0.74457	0.319006	FBXW11	Height	3,1
chr16	3771601	3771900	0.7711	0.305269	0.3543	0.613685	FBXW11	Height	3,1
chr16	3785101	3785400	0.5631	1.050759	0.481469	0.608441	FBXW11	Height	3,1
chr16	3804001	3804300	0	0.11246	0.164871	0.496862	FBXW11	Height	3,1
chr21	31383901	31384200	2.15853	3.058632	1.526842	2.408491	FLI1	Height	1
chr21	31395601	31395900	0.16136	0.488883	0.111375	0.243187	FLI1	Height	1
chr21	31410901	31411200	1.06307	1.567651	0.777891	0.689503	FLI1	Height	1
chr2	82819801	82820100	0.66297	0.419568	0.0579	0.583168	FREM1	Height	14
chr2	82852201	82852500	0.45003	0.185453	0.258719	0.664231	FREM1	Height	14
chr2	82875901	82876200	0.60974	0.352092	0.374476	0.304221	FREM1	Height	14
chr2	82975201	82975500	0.66297	0.376423	0.798066	0.714776	FREM1	Height	14
chr4	5208601	5208900	1.66951	0.754685	0.71563	0.84733	GRB10	BMI	7
chr4	5220001	5220300	0.26783	2.490391	2.330205	3.848532	GRB10	BMI	7
chr4	5230501	5230800	0.58724	1.02275	0.933998	1.252643	GRB10	BMI	7
chr4	5233201	5233500	0.742	0.560037	0.760363	1.58738	GRB10	BMI	7
chr4	5233501	5233800	0.71786	0.44206	0.472705	1.414767	GRB10	BMI	7
chr4	5239201	5239500	4.91029	3.330375	2.13548	3.077966	GRB10	BMI	7
chr4	5239501	5239800	2.26665	1.129269	0.526202	0.62847	GRB10	BMI	7
chr4	5241901	5242200	1.19863	2.505951	2.151273	2.645489	GRB10	BMI	7
chr4	5242801	5243100	1.1355	2.271836	1.130457	0.654689	GRB10	BMI	7
chr4	5248501	5248800	2.30999	2.305362	1.123427	1.7085	GRB10	BMI	7
chr4	5253301	5253600	0.50657	1.031945	0.880502	0.72002	GRB10	BMI	7
chr4	5253601	5253900	0.42919	2.206199	0.976083	0.883091	GRB10	BMI	7
chr4	5280301	5280600	1.61957	1.045242	0.365712	1.095761	GRB10	BMI	7

chr4	5287801	5288100	2.53448	1.294069	1.796973	2.671708	GRB10	BMI	7
chr2	143889301	143889600	0.10482	0.488883	0.370094	0.253675	GRB14	BMI	7
chr8	32260801	32261100	0.47417	0.822161	0.45253	0.192641	HACE1	Height	2
chr8	32261101	32261400	0.47582	0.582529	0.345537	0.167369	HACE1	Height	2
chr8	32263501	32263800	0.31447	0.608699	0.29204	0.0811	HACE1	Height	2
chr19	58289701	58290000	1.6887	1.036047	4.129826	0.927447	HDAC11	Height	1
chr19	58299601	58299900	2.48124	2.108875	2.265296	1.04427	HDAC11	Height	1
chr19	58307701	58308000	0.13392	0.162961	0.419209	0.385283	HDAC11	Height	1
chr19	58313701	58314000	1.35999	0.842814	1.665423	1.99699	HDAC11	Height	1
chr20	8440201	8440500	0.69207	0.790474	0.242925	0.699991	**HMGA1**	Height	3,5,1,13,8,14,15, 17,2
chr2	242539501	242539800	1.5164	1.043403	0.599874	1.790509	HTR1D	Height	1
chr2	228079801	228080100	0.15806	0.702345	0.189429	0.116823	IRS1	BMI	7
chr2	228090601	228090900	0.87757	1.328019	0.604256	0.714776	IRS1	BMI	7
chr4	68229001	68229300	0.26618	0.702345	0.58408	0.370498	**JAZF1**	Height	3,1,17, 18
chr4	68272501	68272800	0.58559	0.724837	0.407797	0.862116	**JAZF1**	Height	3,1,17,18
chr4	68292601	68292900	0.0823	0.487044	0.0246	0.365254	**JAZF1**	Height	3,1,17,18
chr11	34874701	34875000	0.5598	0.612377	0.106993	0.516891	KCNJ12	Height	1
chr11	34875301	34875600	2.20516	0.578851	2.229327	2.16625	KCNJ12	Height	1
chr11	34878301	34878600	2.18927	0.455357	2.249503	2.128598	KCNJ12	Height	1
chr11	34880101	34880400	0.40174	0.867145	0.341155	0.613685	KCNJ12	Height	1
chr11	34888201	34888500	1.38743	0.318566	0.568286	0.882145	KCNJ12	Height	1
chr21	48212401	48212700	0.15971	0.60686	0.307834	0.162125	KCNQ1	Height	1
chr21	48256201	48256500	2.18927	2.474688	5.852213	2.272585	KCNQ1	Height	1
chr21	48261601	48261900	0	0.862052	3.067744	0.339981	KCNQ1	Height	1
chr21	48279301	48279600	0.0532	0.627513	0.185047	0.349523	KCNQ1	Height	1
chr21	48296401	48296700	0.7744	2.593937	8.585832	0.974641	KCNQ1	Height	1
chr20	38445901	38446200	0.39844	0.631191	0.320979	0.243187	KIF13A	Height	2
chr20	38456401	38456700	0.24039	0.44206	0.213986	0.304221	KIF13A	Height	2
chr20	38484901	38485200	0.26453	0.490722	0.263101	0.608441	KIF13A	Height	2
chr20	38580601	38580900	1.03562	1.056276	0.519172	0.421043	KIF13A	Height	2
chr20	38586301	38586600	0.10647	0.678014	0.185047	0.430585	KIF13A	Height	2

chr20	38646901	38647200	0.71456	1.869243	0.954174	1.054757	KIF13A	Height	2
chr15	43869901	43870200	0.40009	1.290391	0.811212	1.288403	LMO1	BMI	7
chr15	43871701	43872000	1.62452	0.752846	0.753333	0.932691	LMO1	BMI	7
chr2	12390001	12390300	1.49225	0.617894	0.390269	0.851628	LPAR1	Height	1
chr2	12460501	12460800	0.371	0.419568	0.45253	0.162125	LPAR1	Height	1
chr3	90494401	90494700	1.72274	0.915807	0.80683	1.485342	**LTBP1**	Height	3,1,8,14
chr3	90496201	90496500	0.74365	0.752846	0.604256	0.278948	**LTBP1**	Height	3,1,8,14
chr3	90510301	90510600	0.21624	0.492561	0.477087	0.0811	**LTBP1**	Height	3,1,8,14
chr3	90573901	90574200	0.5598	0.185453	0.185047	0.0758	**LTBP1**	Height	3,1,8,14
chr3	90584101	90584400	0.39844	0.352092	0.534965	0.111579	**LTBP1**	Height	3,1,8,14
chr3	90621301	90621600	1.24857	1.23989	1.197099	0.577924	**LTBP1**	Height	3,1,8,14
chr3	90759601	90759900	0.10482	0.423246	0.185047	0.142096	**LTBP1**	Height	3,1,8,14
chr5	37457401	37457700	1.11135	1.344994	0.692807	2.66741	MGAT1	Weight	19
chr5	37461601	37461900	0.1855	0.706023	0.164871	0.147339	MGAT1	Weight	19
chr3	215810101	215810400	0.24039	0.842814	0.271865	0.349523	MKL1	Height	20
chr1	98955901	98956200	0.29362	0.350253	0.189429	0.557895	MTMR11	Height	3,2
chr1	98957101	98957400	0.79854	1.050759	0.399033	0.62847	MTMR11	Height	3,2
chr5	14808601	14808900	1.64701	0.964469	0.834035	0.81157	MYO1F	Height	2
chr5	14811901	14812200	0.21129	0.470069	0.156108	0.111579	MYO1F	Height	2
chr1	47063101	47063400	0.52906	0.794152	0.365712	0.795839	NEGR1	Weight	9
chr1	47106601	47106900	0.29197	0.258446	0.0246	0.577924	NEGR1	Weight	9
chr1	47271001	47271300	0.18385	0.374584	0.506026	0.248431	NEGR1	Weight	9
chr1	47453401	47453700	0.21294	0.515053	0.258719	0.136852	NEGR1	Weight	9
chr1	47470501	47470800	0.32107	0.279099	0.254337	0.72002	NEGR1	Weight	9
chr1	47494801	47495100	0.26618	0.537545	0.316597	0.385283	NEGR1	Weight	9
chr1	47538001	47538300	0.34686	0.443899	0.399033	0.106335	NEGR1	Weight	9
chr1	47649301	47649600	0.15971	0.419568	0.0535	0.162125	NEGR1	Weight	9
chr24	13710301	13710600	0.45168	0.13863	0.258719	0.522135	NTAN1	Height	8
chr20	38678701	38679000	1.00488	1.12743	0.860326	2.033696	NUP153	Height	2
chr20	38699701	38700000	0.69372	0.492561	0.271865	0.714776	NUP153	Height	2
chr20	38736901	38737200	0.34851	1.475844	0.42359	0.781053	NUP153	Height	2

chr1	114545701	114546000	0.10647	0.513214	0.267483	0.156881	PBX1	Height	21
chr1	114587701	114588000	0.40009	0.395237	0.971701	0.39577	PBX1	Height	21
chr1	114623701	114624000	2.07455	3.00219	1.422497	1.820079	PBX1	Height	21
chr1	114636601	114636900	1.40993	0.307108	1.896936	0.608441	PBX1	Height	21
chr1	114648301	114648600	0.9533	0.464552	0.793684	1.774777	PBX1	Height	21
chr1	114684601	114684900	0.1855	0.466391	0.209604	0.842087	PBX1	Height	21
chr1	114711301	114711600	0.74695	1.07509	0.477087	0.689503	PBX1	Height	21
chr1	114733201	114733500	0.26453	0.678014	0.218368	0.577924	PBX1	Height	21
chr1	114741301	114741600	0.40009	0.867145	0.238543	0.653743	PBX1	Height	21
chr1	114771301	114771600	0.74365	0.299752	0.662134	0.86736	PBX1	Height	21
chr1	114786301	114786600	0.26288	0.301591	0.341155	0.745293	PBX1	Height	21
chr1	114796201	114796500	0.3743	0.979605	0.675279	0.248431	PBX1	Height	21
chr22	4459501	4459800	0.50327	0.187292	0.506026	0.223158	PCDH15	Weight	21
chr22	4471801	4472100	0.0532	0.447577	0	0.217914	PCDH15	Weight	21
chr22	4551601	4551900	0.39679	0.419568	0.831387	0.937935	PCDH15	Weight	21
chr22	4582201	4582500	0.52906	0.509536	0.0535	0.557895	PCDH15	Weight	21
chr22	4663501	4663800	0.1855	0.492561	0.111375	0.334737	PCDH15	Weight	21
chr22	4689901	4690200	0.26453	0.0712	0.539347	0.223158	PCDH15	Weight	21
chr22	4690201	4690500	0.15806	0.142308	0.847181	0.284191	PCDH15	Weight	21
chr22	4912201	4912500	0.34686	0.46823	0.0781	0.116823	PCDH15	Weight	21
chr22	5086801	5087100	0.71291	1.749427	2.144243	0.693801	PCDH15	Weight	21
chr22	5087101	5087400	0.47417	1.286713	1.484758	0.420098	PCDH15	Weight	21
chr22	5125801	5126100	0.18715	0.0918	0.702485	0.197885	PCDH15	Weight	21
chr22	5242201	5242500	0.58394	0.258446	0.296422	0.243187	PCDH15	Weight	21
chr22	5262301	5262600	0.23874	0.515053	0.156108	0.167369	PCDH15	Weight	21
chr2	131142601	131142900	0.13227	0.258446	0.506026	0.131608	PDE11A	Height	1
chr2	131168701	131169000	0.45003	0.726676	0.189429	0.89693	PDE11A	Height	1
chr2	131170201	131170500	0.47747	0.235954	0.13155	0.172612	PDE11A	Height	1
chr2	131222701	131223000	0.93245	1.779275	0.798066	1.079084	PDE11A	Height	1
chr2	131275501	131275800	0.47417	1.144405	0.637577	0.192641	PDE11A	Height	1
chr2	131290801	131291100	0.63553	0.848331	1.448788	0.603197	PDE11A	Height	1

chr2	131295601	131295900	1.14375	0.485205	1.16816	1.739017	PDE11A	Height	1
chr2	131297401	131297700	0.15971	0.563715	0.111375	0.365254	PDE11A	Height	1
chr2	131310001	131310300	0.24039	0.535706	0.0781	0.061	PDE11A	Height	1
chr2	131311501	131311800	0.15971	0.370906	0.106993	0	PDE11A	Height	1
chr2	131315701	131316000	3.24904	3.584719	1.111196	0.354766	PDE11A	Height	1
chr2	131316001	131316300	1.96313	1.694823	0.635843	0.4158	PDE11A	Height	1
chr2	131354701	131355000	0.16136	0.722998	0.0982	0.182154	PDE11A	Height	1
chr24	13721701	13722000	0.9566	2.275514	0.918205	1.515859	PDXDC1	Height	8
chr24	13737901	13738200	1.484	0.282777	0.325361	0.770566	PDXDC1	Height	8
chr4	9729301	9729600	0.42424	0.679853	0.329743	0.142096	PEX1	Height	2
chr4	9748801	9749100	0.32272	0.88412	0.407797	0.273704	PEX1	Height	2
chr20	24150301	24150600	0.3743	0.560037	0.448148	0.197885	PKHD1	Weight	22
chr20	24231301	24231600	0.23874	0.724837	0.349918	0.304221	PKHD1	Weight	22
chr20	24231601	24231900	1.04057	2.107036	1.243566	0.765322	PKHD1	Weight	22
chr20	24241801	24242100	0.0532	0.464552	0.127169	0.162125	PKHD1	Weight	22
chr20	24271801	24272100	0.61304	0.610538	0.847181	0.339981	PKHD1	Weight	22
chr20	24285901	24286200	0.74695	0.470069	0.773509	0.36001	PKHD1	Weight	22
chr22	41187601	41187900	0.79689	0.471908	0.403415	0.243187	PLEKHA1	Height	8
chr3	80014201	80014500	1.88905	1.717315	0.793684	1.034728	PPM1B	Height	3
chr12	67708201	67708500	0.34521	0.674336	0.711249	0.664231	PROX1	BMI	7
chr12	67708801	67709100	1.27766	1.619991	2.645888	1.470557	PROX1	BMI	7
chr2	30671701	30672000	0.32107	0.4944	0.0824	0.0558	PTCH1	Height	16
chr2	27387601	27387900	0.23874	0.419568	0.156108	0.248431	PTPDC1	Height	1
chr17	60801901	60802200	0.15971	0.539384	0.42359	0.298977	RASAL1	BMI	10
chr17	60805501	60805800	0.53401	0.488883	1.052403	0.426287	RASAL1	BMI	10
chr19	47802001	47802300	0.63718	0.539384	0.588462	0.243187	RFT1	Height	3
chr19	47808301	47808600	0.31942	0.445738	0.185047	0.167369	RFT1	Height	3
chr19	47808601	47808900	0.0807	0.395237	0.242925	0.192641	RFT1	Height	3
chr11	17620801	17621100	0.37265	0.515053	0.16049	0.167369	RNF135	Height	2
chr20	47034901	47035200	0.29692	0.670658	1.226039	0.4158	RREB1	BMI	7,10
chr1	227085301	227085600	0.1888	0.258446	0.209604	0.496862	RSRC1	Height	3

chr1	227256301	227256600	0.26453	0.447577	0.238543	0.740049		RSRC1	Height	3
chr1	227269801	227270100	0.29197	0.801508	0.370094	0.304221		RSRC1	Height	3
chr1	227287801	227288100	0.23874	0.400754	0.345537	0.0811		RSRC1	Height	3
chr1	227331601	227331900	0.0807	0.515053	0.0491	0.192641		RSRC1	Height	3
chr1	227335201	227335500	0.15806	0.650005	0.0824	0.339981		RSRC1	Height	3
chr1	227371201	227371500	0.47912	0.490722	0.189429	0.329493		RSRC1	Height	3
chr1	227375401	227375700	0.42754	1.002097	0.52182	1.353734		RSRC1	Height	3
chr1	227497201	227497500	0.34686	0.419568	0.263101	0.192641		RSRC1	Height	3
chr7	33901501	33901800	0.50657	1.168736	0.650722	0.572681		RTF1	Height	1
chr1	15325201	15325500	0.66462	0.235954	0.320979	0.948422		**SCMH1**	Height	3,1,16
chr1	15335101	15335400	0.56145	0.726676	0.209604	0.390527		**SCMH1**	Height	3,1,16
chr1	15335401	15335700	1.67116	2.738227	1.337413	2.564427		**SCMH1**	Height	3,1,16
chr1	15386101	15386400	1.3051	0.816644	0.657752	0.400068		**SCMH1**	Height	3,1,16
chr1	15422701	15423000	0.31777	0.419568	0.686691	0.471589		**SCMH1**	Height	3,1,16
chr15	52925701	52926000	0.24039	0.730778	2.874753	0.319952		SERPINH1	Height	23,1
chr15	52927501	52927800	1.1421	1.518989	0.563905	0.821112		SERPINH1	Height	23,1
chr11	57117901	57118200	0.50657	0.325922	0.13155	0.248431		SLC39A11	BMI	10
chr11	57156301	57156600	0.45168	1.312883	0.670898	0.4158		SLC39A11	BMI	10
chr11	57184201	57184500	0.24039	0.46823	0.263101	0.577924		SLC39A11	BMI	10
chr11	57190501	57190800	1.40663	1.432699	0.626165	0.740049		SLC39A11	BMI	10
chr11	57194101	57194400	0.21294	0.443899	0.0982	0.111579		SLC39A11	BMI	10
chr11	57236401	57236700	3.35386	3.015911	0.640225	0.943178		SLC39A11	BMI	10
chr11	57244201	57244500	0.15806	0.634869	0.22978	0.223158		SLC39A11	BMI	10
chr11	57288601	57288900	1.57623	3.421758	1.334765	1.04427		SLC39A11	BMI	10
chr11	57292801	57293100	2.27924	0.919909	0.728776	3.200032		SLC39A11	BMI	10
chr11	57293101	57293400	3.27483	3.468581	1.932905	4.005413		SLC39A11	BMI	10
chr11	57328201	57328500	3.13597	4.054788	1.873293	3.113726		SLC39A11	BMI	10
chr11	57356101	57356400	1.25517	2.756617	0.71563	0.76962		SLC39A11	BMI	10
chr11	57381301	57381600	0.23874	0.79783	0.316597	0.253675		SLC39A11	BMI	10
chr11	57383101	57383400	0.29362	0.339219	1.791677	0.0253		SLC39A11	BMI	10
chr3	193804501	193804800	0.87757	0.726676	0.242925	0.725264		SLCO1C1	Height	1,2

chr3	193841701	193842000	0.71786	0.307108	0.370094	0.243187	SLCO1C1	Height	1,2
chr3	193871401	193871700	0.10647	0.443899	0.263101	0.329493	SLCO1C1	Height	1,2
chr3	193876801	193877100	0.21129	0.608699	0.608637	0.131608	SLCO1C1	Height	1,2
chr1	94554901	94555200	0.45168	0.376423	0.189429	0.653743	**SPAG17**	Height	3,1,16
chr1	94638601	94638900	0.26618	0.353931	0.185047	0.557895	**SPAG17**	Height	3,1,16
chr1	94667701	94668000	1.19863	0.423246	0.753333	0.482077	**SPAG17**	Height	3,1,16
chr1	94773301	94773600	0.95825	1.138888	0.399033	0.435829	**SPAG17**	Height	3,1,16
chr2	23104201	23104500	2.31329	2.464645	0.918205	1.946444	SPIN1	Height	1,2
chr9	46203601	46203900	0.45003	0.586207	0.238543	0.217914	SULF1	Height, BMI	5,7
chr9	46253701	46254000	0.21459	0.516892	0.296422	0.136852	SULF1	Height, BMI	5,7
chr9	46256701	46257000	0.29362	0.348414	0.0824	0.491618	SULF1	Height, BMI	5,7
chr9	46269901	46270200	1.69695	1.003936	2.048662	1.409524	SULF1	Height, BMI	5,7
chr9	46271401	46271700	0.31942	0.470069	0.320979	0.192641	SULF1	Height, BMI	5,7
chr14	9955801	9956100	0.50492	0.792313	1.040992	0.62847	TAF1C	BMI	10
chr15	39382501	39382800	0.61139	0.539384	0.16049	0.253675	TEAD1	Height	1
chr15	39420001	39420300	0.34686	0.71932	0.234162	0.228402	TEAD1	Height	1
chr15	39445801	39446100	0.82433	0.698667	0.365712	0.856872	TEAD1	Height	1
chr15	39532501	39532800	0.47747	0.921324	0.0535	1.04427	TEAD1	Height	1
chr15	39532801	39533100	0.31942	0.492561	0.106993	0.496862	TEAD1	Height	1
chr17	48035101	48035400	0.13227	0.1648	0.419209	0.136852	TMEM132C	Height	24
chr17	48057001	48057300	0.54061	0.589885	0.349918	1.627438	TMEM132C	Height	24
chr17	48057301	48057600	0.10977	0.262124	0.106993	1.27697	TMEM132C	Height	24
chr17	48072901	48073200	0.21294	0.397076	0.169253	0.603197	TMEM132C	Height	24
chr17	48097201	48097500	0.45168	0.679853	0.29204	0.491618	TMEM132C	Height	24
chr17	48162901	48163200	1.80507	1.944075	1.369001	0.546462	TMEM132C	Height	24
chr17	48179701	48180000	0.34686	0.185453	0.296422	0.577924	TMEM132C	Height	24
chr17	48204301	48204600	0.61469	0.889637	1.152366	0.466345	TMEM132C	Height	24

chr17	48205801	48206100	0.371	0.258446	0.287658	0.851628	TMEM132C	Height	24
chr17	48251701	48252000	0.21129	0.605021	0.238543	0.248431	TMEM132C	Height	24
chr17	48258001	48258300	0.69372	0.584368	0.427972	0.278948	TMEM132C	Height	24
chr4	112812001	112812300	0.26288	0.19097	0.793684	0.4158	TMEM176A	Height	3,1
chr10	34677301	34677600	0.84517	1.59566	0.329743	0.39577	TNFRSF19	BMI	10
chr2	218925601	218925900	0.69207	0.721159	0.896296	0.258918	TNS1	Height	5,1
chr2	218946001	218946300	1.113	0.539384	0.769127	0.658987	TNS1	Height	5,1
chr2	219011701	219012000	0.55815	0.352092	0.238543	0.410556	TNS1	Height	5,1
chr2	219070501	219070800	0.21294	0.6592	0.13155	0.101092	TNS1	Height	5,1
chr2	219120001	219120300	0.55815	0.511375	0.267483	0.4158	TNS1	Height	5,1
chr2	219123301	219123600	0.45168	0.30343	0.916471	0.633714	TNS1	Height	5,1
chr18	55648501	55648800	0.18715	0.301591	0.58408	0.106335	**TRIP11**	Height	1,2,4
chr5	43717501	43717800	0.3776	0.262124	0.135932	0.948422	TXNDC15	Height	2
chr5	43717801	43718100	1.93568	1.59566	0.657752	1.567351	TXNDC15	Height	2
chr5	43724401	43724700	0.23709	0.445738	0.102611	0.334737	TXNDC15	Height	2
chr20	8976601	8976900	0.45333	0.372745	0.526202	0.912662	UHRF1BP1	BMI	7
chr14	10355701	10356000	1.08721	0.507697	0.682309	1.333705	USP10	Height	2
chr14	10374001	10374300	0.26618	0.423246	0.135932	0.136852	USP10	Height	2
chr14	10406401	10406700	0.90336	0.964469	0.633195	0.4158	USP10	Height	2
chr7	45081301	45081600	0.0274	0.584368	0.209604	0.248431	VPS13C	BMI	7
chr7	45097201	45097500	0.13227	0.445738	0.102611	0.131608	VPS13C	BMI	7
chr7	45119701	45120000	0.45003	0.280938	0.16049	0.167369	VPS13C	BMI	7
chr7	45133501	45133800	0.87427	2.180029	0.985761	1.049514	VPS13C	BMI	7
chr7	45193201	45193500	1.1454	1.059954	0.510408	0.502106	VPS13C	BMI	7
chr7	45198301	45198600	0.36935	0.676175	0.29204	0.223158	VPS13C	BMI	7
chr7	45219301	45219600	0.61139	1.346833	0.341155	0.192641	VPS13C	BMI	7
chr7	45219601	45219900	0.66297	1.442318	0.534965	0.435829	VPS13C	BMI	7
chr7	45232501	45232800	0.3677	0.837297	0.365712	0.862116	VPS13C	BMI	7
chr14	10041901	10042200	0.21129	0.350253	0.209604	0.613685	WFDC1	BMI	10
chr14	10061401	10061700	0.50492	0.513214	0.370094	0.212671	WFDC1	BMI	10
chr25	34710901	34711200	0.21459	0.541223	0.135932	0.223158	ZMIZ1	Height	3

chr25	34743601	34743900	0.23544	0.825839	0.238543	0.248431	ZMIZ1	Height	3
chr25	34759201	34759500	0.10812	0.938299	0.463941	0.608441	ZMIZ1	Height	3
chr25	34783201	34783500	0.42589	0.492561	0.29204	0.81157	ZMIZ1	Height	3
chr25	34790401	34790700	0.53071	0.986961	0.345537	0.522135	ZMIZ1	Height	3
chr25	34800601	34800900	0.29362	0.702345	0.472705	0.223158	ZMIZ1	Height	3
chr25	34813201	34813500	1.1196	0.865306	0.670898	0.319952	ZMIZ1	Height	3
chr13	62632201	62632500	0.34191	0.561876	0.13155	0.131608	ZNF341	Height	1
chr13	62633701	62634000	0.10647	0.370906	0.0781	0.223158	ZNF341	Height	1
chr13	62637901	62638200	1.03892	1.384037	0.608637	1.04427	ZNF341	Height	1
chr13	76953001	76953300	1.40828	1.125591	0.443766	0.882145	ZNFX1	Height	1

Additional Table 2. 41,750 windows showing breed-specific differential methylation.

Breeds	Chromsome	Start	End	*UQ* score	*Tan* score	*StH* score	*Hu* score
UQ	1	176701	177000	0.210	0.865	0.847	0.588
UQ	1	367201	367500	3.270	2.899	2.107	2.951
UQ	1	923101	923400	0.239	0.792	1.023	0.821
UQ	1	970501	970800	1.996	3.066	2.419	2.779
UQ	1	1238101	1238400	0.613	0.302	0.296	0.193
UQ	1	1339201	1339500	1.565	0.983	0.983	1.029
UQ	1	1582801	1583100	1.966	0.959	1.288	1.145
UQ	1	1694101	1694400	0.666	0.350	0.448	0.472
UQ	1	1782001	1782300	5.957	2.817	3.511	3.000
UQ	1	1904401	1904700	1.884	3.017	3.529	2.368
UQ	1	1930801	1931100	1.355	2.242	1.052	1.962
UQ	1	2223601	2223900	0.587	0.114	0.292	0.187
UQ	1	2736001	2736300	4.235	2.942	2.912	2.789
UQ	1	2787601	2787900	2.452	1.141	1.556	1.546
UQ	1	2820901	2821200	0.292	0.701	0.593	0.918
UQ	1	3168301	3168600	0.532	0.281	0.346	0.223
UQ	1	3408901	3409200	4.306	3.113	3.283	3.437
UQ	1	3509701	3510000	0.108	0.491	0.395	0.365
UQ	1	3610801	3611100	2.844	3.298	2.271	2.286
UQ	1	3971101	3971400	1.011	0.513	0.633	0.360
UQ	1	4101601	4101900	2.550	3.486	1.761	3.580
UQ	1	4378801	4379100	0.666	0.281	0.453	0.497
UQ	1	4439401	4439700	0.773	1.077	1.465	1.197
UQ	1	4505701	4506000	0.824	0.421	0.617	0.441
UQ	1	5117101	5117400	3.560	2.710	2.834	2.692
UQ	1	5277001	5277300	0.319	1.221	1.262	0.882
UQ	1	5278201	5278500	2.896	1.597	1.414	1.373
UQ	1	5287201	5287500	0.558	0.328	0.263	0.274
UQ	1	5305801	5306100	1.779	1.521	1.407	2.074
UQ	1	5346001	5346300	0.582	0.236	0.107	0.213
UQ	1	5352601	5352900	3.010	2.131	2.287	2.165
UQ	1	5445001	5445300	2.367	1.365	0.678	1.424
UQ	1	5445301	5445600	2.688	1.335	0.809	1.587
UQ	1	5455501	5455800	0.106	0.328	0.346	0.497
UQ	1	5583001	5583300	0.987	0.536	0.181	0.583
UQ	1	5644201	5644500	6.532	3.576	3.484	4.056
UQ	1	5801101	5801400	1.244	2.143	2.218	2.556
UQ	1	5801401	5801700	1.773	2.961	2.715	2.850
UQ	1	5805601	5805900	3.491	1.796	1.616	1.424
UQ	1	5805901	5806200	1.947	0.549	0.147	0.359
UQ	1	6355501	6355800	1.463	0.890	0.760	0.720
UQ	1	6465001	6465300	0.589	0.163	0.185	0.223
UQ	1	6567601	6567900	0.103	0.817	0.823	0.700
UQ	1	6745201	6745500	0.586	0.144	0.185	0.248
UQ	1	7169101	7169400	0.319	0.583	0.535	0.051
UQ	1	7285501	7285800	0.345	1.058	1.287	1.258
UQ	1	7728901	7729200	1.729	2.010	1.115	2.099
UQ	1	7847101	7847400	1.037	0.581	0.317	0.446
UQ	1	7946101	7946400	3.325	3.649	2.916	3.640
UQ	1	8209201	8209500	2.473	1.006	1.741	1.582
UQ	1	8247001	8247300	0.426	0.794	0.609	0.715
UQ	1	8352901	8353200	1.918	1.094	0.818	1.415
UQ	1	8476501	8476800	0.213	0.493	0.526	0.441
UQ	1	8650201	8650500	4.410	2.549	3.348	2.921
UQ	1	9408901	9409200	0.531	0.165	0.132	0.162
UQ	1	9531901	9532200	1.179	0.182	0.127	0.137
UQ	1	9625801	9626100	0.134	0.656	0.350	0.421
UQ	1	9791101	9791400	0.211	0.513	0.856	0.507
UQ	1	9840601	9840900	0.883	1.470	0.395	0.380
UQ	1	10012501	10012800	1.008	0.657	0.218	0.218
UQ	1	10319701	10320000	0.448	2.066	1.440	1.044
UQ	1	10428601	10428900	1.752	1.240	0.852	1.242
UQ	1	10845001	10845300	1.565	1.028	0.999	1.024

UQ	1	10997401	10997700	3.692	2.871	2.530	2.870
UQ	1	11073601	11073900	0.716	0.234	0.312	0.279
UQ	1	11311201	11311500	1.436	2.454	0.859	0.867
UQ	1	11571901	11572200	1.015	0.352	0.140	0.299
UQ	1	12249901	12250200	0.663	0.466	0.477	0.248
UQ	1	12295501	12295800	1.144	1.262	1.407	1.496
UQ	1	12471001	12471300	0.608	0.258	0.049	0.025
UQ	1	12538501	12538800	0.268	0.588	0.930	0.776
UQ	1	12605701	12606000	0.637	0.444	0.267	0.497
UQ	1	12763801	12764100	0.292	0.564	1.119	0.720
UQ	1	12964801	12965100	0.557	0.118	0.185	0.228
UQ	1	13853701	13854000	0.611	0.328	0.399	0.553
UQ	1	14234401	14234700	1.199	0.614	0.691	0.614
UQ	1	14934301	14934600	1.566	0.980	0.925	0.983
UQ	1	15031501	15031800	0.505	0.260	0.239	0.142
UQ	1	15107401	15107700	1.539	1.053	0.560	1.131
UQ	1	15224401	15224700	1.168	0.609	0.671	0.634
UQ	1	15256201	15256500	1.618	2.203	1.048	0.988
UQ	1	15325201	15325500	0.665	0.236	0.321	0.948
UQ	1	15386101	15386400	1.305	0.817	0.658	0.400
UQ	1	15869401	15869700	0.371	0.611	0.584	0.690
UQ	1	16025401	16025700	0.824	0.631	0.317	0.690
UQ	1	17140201	17140500	0.931	0.465	0.531	0.507
UQ	1	17779501	17779800	0.532	0.702	0.189	0.167
UQ	1	17895601	17895900	1.942	2.409	1.407	1.694
UQ	1	17937601	17937900	2.947	1.425	2.086	1.922
UQ	1	18234601	18234900	0.395	1.468	1.090	1.055
UQ	1	18409201	18409500	0.586	0.120	0.350	0.274
UQ	1	18788101	18788400	0.821	0.328	0.370	0.259
UQ	1	18939301	18939600	0.560	0.071	0.160	0.147
UQ	1	20816401	20816700	0.213	0.581	0.560	0.553
UQ	1	21120901	21121200	0.797	0.401	0.136	0.304
UQ	1	21917401	21917700	1.381	0.773	0.724	0.740
UQ	1	21991201	21991500	1.305	0.751	0.794	0.751
UQ	1	22071301	22071600	2.174	1.109	1.068	0.725
UQ	1	22079101	22079400	3.984	1.884	1.995	2.220
UQ	1	22147801	22148100	0.510	1.472	1.609	1.136
UQ	1	22151101	22151400	0.242	1.032	1.144	1.319
UQ	1	22166401	22166700	1.195	0.356	0.517	0.319
UQ	1	22200601	22200900	0.265	0.493	0.560	0.472
UQ	1	22293301	22293600	0.503	0.212	0.111	0.137
UQ	1	22317001	22317300	0.908	0.305	0.272	0.157
UQ	1	22805401	22805700	0.718	0.423	0.214	0.309
UQ	1	22981201	22981500	1.200	0.725	0.526	0.578
UQ	1	23186401	23186700	1.355	0.912	0.535	0.563
UQ	1	23186701	23187000	0.584	0.279	0.374	0.360
UQ	1	24204901	24205200	1.278	0.914	0.885	0.740
UQ	1	24557701	24558000	1.357	0.981	1.003	0.893
UQ	1	25531501	25531800	0.426	0.725	0.695	0.725
UQ	1	25543501	25543800	0.213	0.328	0.560	0.391
UQ	1	27187201	27187500	0.186	1.311	0.502	0.776
UQ	1	27322201	27322500	3.110	2.021	1.978	1.957
UQ	1	27746401	27746700	0.745	1.144	1.275	0.533
UQ	1	27803401	27803700	0.534	0.985	0.954	1.111
UQ	1	27918001	27918300	0.716	0.328	0.296	0.182
UQ	1	28006201	28006500	0.720	0.260	0.263	0.411
UQ	1	28089301	28089600	0.878	1.661	1.032	1.080
UQ	1	28138201	28138500	1.013	0.420	0.383	0.335
UQ	1	28417201	28417500	2.179	1.731	1.757	2.454
UQ	1	28793401	28793700	1.037	0.654	0.691	0.654
UQ	1	28981201	28981500	0.507	0.260	0.210	0.193
UQ	1	29336401	29336700	0.637	0.376	0.239	0.304
UQ	1	29358301	29358600	0.106	0.376	0.473	0.416
UQ	1	29514901	29515200	0.586	0.376	0.214	0.193
UQ	1	30187201	30187500	1.594	1.026	1.023	0.695
UQ	1	30648001	30648300	6.107	5.040	4.590	5.105
UQ	1	30778201	30778500	1.063	2.086	1.621	1.917

UQ	1	31196101	31196400	0.610	0.350	0.321	0.142
UQ	1	31273801	31274100	1.140	0.288	0.160	0.497
UQ	1	31380901	31381200	0.503	0.047	0.321	0.051
UQ	1	31381201	31381500	0.610	0.234	0.346	0.218
UQ	1	31421401	31421700	0.531	0.165	0.296	0.299
UQ	1	31632001	31632300	0.692	0.395	0.350	0.340
UQ	1	32747401	32747700	0.582	1.172	1.168	0.913
UQ	1	32803501	32803800	0.477	0.189	0.103	0.162
UQ	1	33039601	33039900	1.620	2.289	2.122	1.987
UQ	1	33147901	33148200	0.850	1.053	0.239	0.492
UQ	1	33846601	33846900	0.479	0.865	0.185	0.999
UQ	1	34139701	34140000	0.239	0.444	0.082	0.472
UQ	1	34751701	34752000	0.507	0.912	0.374	0.360
UQ	1	34827901	34828200	0.828	0.257	0.107	0.288
UQ	1	36351301	36351600	0.213	0.444	0.346	0.391
UQ	1	36671101	36671400	0.503	0.279	0.214	0.304
UQ	1	36836701	36837000	0.558	0.212	0.160	0.218
UQ	1	38380501	38380800	0.532	0.213	0.078	0.173
UQ	1	38553001	38553300	2.576	3.175	2.102	2.956
UQ	1	39365401	39365700	0.531	0.305	0.288	0.329
UQ	1	39591901	39592200	0.718	0.305	0.477	0.826
UQ	1	39696901	39697200	1.621	1.309	1.222	1.166
UQ	1	40002901	40003200	1.249	0.680	0.600	0.583
UQ	1	40058401	40058700	0.795	0.350	0.337	0.340
UQ	1	40118401	40118700	0.850	1.004	0.584	0.639
UQ	1	40522501	40522800	0.639	1.543	1.382	1.096
UQ	1	40601101	40601400	2.341	1.526	1.596	1.755
UQ	1	40601401	40601700	2.604	1.736	1.708	1.785
UQ	1	41292301	41292600	0.586	0.446	0.239	0.329
UQ	1	41835601	41835900	0.160	0.493	0.370	0.411
UQ	1	42015001	42015300	0.799	0.513	0.638	0.583
UQ	1	42147601	42147900	0.581	1.217	1.106	1.197
UQ	1	42292201	42292500	0.532	0.863	0.214	0.385
UQ	1	42372601	42372900	0.505	0.142	0.082	0.117
UQ	1	42421501	42421800	0.079	0.586	0.317	0.329
UQ	1	42755401	42755700	0.745	0.466	0.481	0.436
UQ	1	42769501	42769800	1.091	0.798	0.481	0.547
UQ	1	42999901	43000200	0.853	0.421	0.395	0.350
UQ	1	43371301	43371600	0.532	0.303	0.181	0.309
UQ	1	43837201	43837500	0.611	0.189	0.058	0.101
UQ	1	43863601	43863900	1.545	0.727	0.477	0.603
UQ	1	46326601	46326900	0.534	0.255	0.156	0.137
UQ	1	46545301	46545600	0.189	0.607	0.687	0.441
UQ	1	46557901	46558200	0.532	0.421	0.156	0.081
UQ	1	47085301	47085600	1.037	0.611	0.720	0.776
UQ	1	48185101	48185400	1.600	0.708	0.856	0.963
UQ	1	48191701	48192000	0.053	0.609	0.337	0.497
UQ	1	48632701	48633000	1.116	0.865	0.724	0.690
UQ	1	49485001	49485300	0.106	0.420	0.321	0.248
UQ	1	50315401	50315700	0.505	0.210	0.185	0.106
UQ	1	51145801	51146100	0.611	0.348	0.267	0.193
UQ	1	52172401	52172700	0.582	0.045	0.111	0.187
UQ	1	52632901	52633200	0.958	0.468	0.604	0.472
UQ	1	52911001	52911300	1.063	0.590	0.453	0.391
UQ	1	53278201	53278500	0.479	0.792	0.720	0.279
UQ	1	53494501	53494800	0.584	0.281	0.395	0.335
UQ	1	54440401	54440700	0.132	0.423	0.481	0.497
UQ	1	54668701	54669000	0.532	0.232	0.049	0.203
UQ	1	54695701	54696000	0.266	0.657	0.720	0.715
UQ	1	54707401	54707700	1.565	0.818	1.003	0.704
UQ	1	54900901	54901200	0.745	0.493	0.584	0.472
UQ	1	54901801	54902100	1.302	1.687	2.044	1.714
UQ	1	56043601	56043900	0.713	1.399	1.201	1.764
UQ	1	56895301	56895600	0.505	0.352	0.267	0.086
UQ	1	58131601	58131900	0.450	0.140	0.136	0.248
UQ	1	58259701	58260000	0.426	0.607	0.185	0.553
UQ	1	58637401	58637700	0.534	0.210	0.234	0.279

UQ	1	58734301	58734600	0.587	0.184	0.239	0.274
UQ	1	59477101	59477400	0.266	0.702	0.535	0.502
UQ	1	59949001	59949300	0.584	0.165	0.317	0.324
UQ	1	60198601	60198900	0.079	0.494	0.497	0.416
UQ	1	60306001	60306300	0.665	0.212	0.210	0.436
UQ	1	61109701	61110000	0.529	0.073	0.152	0.081
UQ	1	62388601	62388900	0.186	0.468	0.317	0.360
UQ	1	62457901	62458200	0.186	0.729	0.695	0.583
UQ	1	62901601	62901900	4.464	5.619	6.588	5.436
UQ	1	63072601	63072900	0.742	0.493	0.370	0.441
UQ	1	64104001	64104300	0.771	0.213	0.444	0.223
UQ	1	65690401	65690700	0.773	1.238	1.300	1.303
UQ	1	66521101	66521400	1.912	3.529	2.677	2.743
UQ	1	66690001	66690300	2.028	0.978	1.164	1.242
UQ	1	67228801	67229100	2.317	1.890	1.937	1.278
UQ	1	68243401	68243700	0.640	0.279	0.263	0.137
UQ	1	68296801	68297100	0.481	0.232	0.239	0.162
UQ	1	68362501	68362800	0.239	0.631	0.535	0.517
UQ	1	68673301	68673600	0.481	0.116	0.103	0.086
UQ	1	69617401	69617700	0.477	1.053	0.711	0.826
UQ	1	69703501	69703800	0.373	0.564	0.584	0.634
UQ	1	70017301	70017600	0.932	1.901	2.242	2.144
UQ	1	70045501	70045800	0.661	0.328	0.959	0.963
UQ	1	70334701	70335000	0.797	0.468	0.370	0.634
UQ	1	71360401	71360700	0.531	0.116	0.189	0.223
UQ	1	71707201	71707500	3.326	2.731	2.760	2.632
UQ	1	72159901	72160200	0.666	0.023	0.194	0.106
UQ	1	72215701	72216000	6.612	1.770	4.129	4.790
UQ	1	72567901	72568200	0.345	0.189	0.185	0.634
UQ	1	72806701	72807000	0.716	0.137	0.169	0.061
UQ	1	72847201	72847500	0.665	0.420	0.234	0.421
UQ	1	74133301	74133600	0.800	0.324	0.374	0.132
UQ	1	74926801	74927100	1.763	0.234	0.354	0.663
UQ	1	75009901	75010200	0.637	0.260	0.325	0.304
UQ	1	75021001	75021300	1.140	0.749	0.662	0.259
UQ	1	75038401	75038700	0.477	0.258	0.160	0.086
UQ	1	75767401	75767700	0.505	0.234	0.214	0.243
UQ	1	76566301	76566600	0.905	0.702	0.740	1.247
UQ	1	77013301	77013600	0.450	0.118	0.267	0.081
UQ	1	77349301	77349600	0.794	0.213	0.140	0.254
UQ	1	77521801	77522100	0.105	0.584	0.403	0.365
UQ	1	77558101	77558400	0.161	0.515	0.481	0.360
UQ	1	77831701	77832000	2.579	1.397	1.835	1.739
UQ	1	77854201	77854500	0.213	0.538	0.481	0.522
UQ	1	79206901	79207200	0.529	1.124	1.242	0.948
UQ	1	79874401	79874700	0.690	0.399	0.506	0.416
UQ	1	80643601	80643900	1.492	0.607	0.901	0.893
UQ	1	80643901	80644200	1.118	0.470	0.617	0.608
UQ	1	80671501	80671800	0.745	0.865	0.292	0.603
UQ	1	82394401	82394700	0.747	0.515	0.477	0.106
UQ	1	82987801	82988100	0.294	0.513	0.564	0.634
UQ	1	83154001	83154300	0.000	0.375	0.444	0.248
UQ	1	83827501	83827800	0.531	0.258	0.165	0.248
UQ	1	84501901	84502200	0.907	0.210	0.189	0.309
UQ	1	85727401	85727700	2.946	1.498	1.797	1.896
UQ	1	86079301	86079600	1.171	0.910	0.774	0.963
UQ	1	86079601	86079900	1.326	0.980	0.642	0.974
UQ	1	86229901	86230200	0.560	0.213	0.234	0.137
UQ	1	86319601	86319900	1.408	1.165	0.774	1.019
UQ	1	86883901	86884200	1.091	0.519	0.399	0.411
UQ	1	88043101	88043400	0.453	0.912	0.711	0.913
UQ	1	88503601	88503900	1.570	1.101	0.687	1.293
UQ	1	88763701	88764000	0.347	0.678	0.736	0.684
UQ	1	90100501	90100800	0.081	0.562	0.239	0.329
UQ	1	90582301	90582600	1.116	0.648	0.642	0.547
UQ	1	91883101	91883400	0.611	0.493	0.156	0.304
UQ	1	92112601	92112900	2.918	2.244	2.205	2.241

UQ	1	92156401	92156700	2.020	2.386	1.431	1.638
UQ	1	92580901	92581200	2.050	1.094	1.465	1.314
UQ	1	92865301	92865600	0.960	0.631	0.691	0.553
UQ	1	93300901	93301200	0.715	0.375	0.317	0.365
UQ	1	93776101	93776400	1.492	0.867	1.032	1.024
UQ	1	93834001	93834300	0.532	0.255	0.214	0.299
UQ	1	93937201	93937500	0.692	0.213	0.210	0.207
UQ	1	94132501	94132800	0.265	0.725	0.510	0.497
UQ	1	94413301	94413600	1.228	0.566	0.510	0.619
UQ	1	96272401	96272700	1.649	1.051	1.168	1.348
UQ	1	96376201	96376500	0.534	0.208	0.136	0.112
UQ	1	97270801	97271100	0.371	0.560	0.185	0.771
UQ	1	97791901	97792200	0.477	0.328	0.132	0.304
UQ	1	98280601	98280900	1.931	0.328	0.185	0.173
UQ	1	98282101	98282400	1.056	0.120	0.074	0.031
UQ	1	98640601	98640900	0.610	0.376	0.321	0.304
UQ	1	98842801	98843100	0.586	0.305	0.374	0.329
UQ	1	99012601	99012900	0.742	1.169	1.489	1.136
UQ	1	99629101	99629400	0.505	0.303	0.185	0.193
UQ	1	99707401	99707700	0.160	0.444	0.457	0.547
UQ	1	100170001	100170300	0.639	0.328	0.292	0.502
UQ	1	102287701	102288000	2.075	3.010	1.415	1.613
UQ	1	102373801	102374100	3.493	1.496	2.258	1.659
UQ	1	102578401	102578700	0.878	0.515	0.609	0.634
UQ	1	103161901	103162200	1.699	1.122	1.230	1.303
UQ	1	103170601	103170900	8.161	3.834	5.769	6.394
UQ	1	103183501	103183800	0.479	1.032	0.745	0.715
UQ	1	103694101	103694400	0.347	0.680	0.560	0.862
UQ	1	103695301	103695600	0.744	0.564	0.424	0.167
UQ	1	103961401	103961700	1.436	0.676	0.885	0.846
UQ	1	104110201	104110500	0.589	0.232	0.107	0.193
UQ	1	104294401	104294700	0.481	0.281	0.078	0.198
UQ	1	104320501	104320800	0.450	0.142	0.132	0.112
UQ	1	104848801	104849100	2.446	1.474	1.407	1.755
UQ	1	105237901	105238200	0.586	0.444	0.448	0.441
UQ	1	105511801	105512100	0.134	0.305	0.477	0.304
UQ	1	105614401	105614700	2.878	2.156	1.966	1.764
UQ	1	105698101	105698400	0.931	0.468	0.350	0.416
UQ	1	105720301	105720600	1.142	1.403	0.934	0.466
UQ	1	105818101	105818400	0.558	0.281	0.210	0.198
UQ	1	106365901	106366200	0.560	0.330	0.185	0.223
UQ	1	106367401	106367700	0.611	0.210	0.201	0.218
UQ	1	106755001	106755300	0.666	0.350	0.267	0.391
UQ	1	106755301	106755600	1.116	0.749	0.633	0.679
UQ	1	109367701	109368000	0.640	0.167	0.136	0.309
UQ	1	109472701	109473000	0.531	0.350	0.239	0.218
UQ	1	109507501	109507800	0.534	0.281	0.156	0.279
UQ	1	109700401	109700700	4.264	2.823	2.662	2.861
UQ	1	110076301	110076600	0.053	0.493	0.214	0.279
UQ	1	110361601	110361900	0.213	0.914	0.983	1.267
UQ	1	110361901	110362200	0.319	1.283	1.284	1.450
UQ	1	110612401	110612700	0.690	0.187	0.337	0.329
UQ	1	110814901	110815200	1.113	0.701	0.515	0.117
UQ	1	111053401	111053700	0.507	0.258	0.160	0.081
UQ	1	111117301	111117600	0.611	0.118	0.230	0.248
UQ	1	111483601	111483900	0.426	0.212	0.609	0.137
UQ	1	111537601	111537900	0.773	0.560	0.350	0.441
UQ	1	111859201	111859500	2.847	3.574	1.514	2.301
UQ	1	111885001	111885300	0.558	0.236	0.234	0.274
UQ	1	111913201	111913500	1.113	0.702	0.658	0.608
UQ	1	111964201	111964500	0.319	0.397	0.531	0.553
UQ	1	111974401	111974700	0.187	0.465	0.428	0.441
UQ	1	113322301	113322600	0.319	0.139	0.613	0.051
UQ	1	114115501	114115800	0.373	0.629	0.778	0.664
UQ	1	114636601	114636900	1.410	0.307	1.897	0.608
UQ	1	115023301	115023600	0.797	0.258	0.481	0.324
UQ	1	115075801	115076100	0.689	0.285	0.127	0.162

UQ	1	115307101	115307400	1.834	1.268	1.115	1.206
UQ	1	115441201	115441500	1.197	0.916	0.905	0.634
UQ	1	116118301	116118600	0.507	0.163	0.321	0.274
UQ	1	116289901	116290200	0.694	0.491	0.453	0.857
UQ	1	116974501	116974800	0.611	0.140	0.247	0.279
UQ	1	117703501	117703800	0.982	0.425	0.267	0.588
UQ	1	117817501	117817800	0.371	0.187	0.535	0.081
UQ	1	117990901	117991200	1.652	1.146	0.974	1.242
UQ	1	118429801	118430100	0.640	0.116	0.317	0.198
UQ	1	118566001	118566300	0.532	0.328	0.346	0.385
UQ	1	118616401	118616700	0.558	0.302	0.374	0.162
UQ	1	118892701	118893000	2.124	1.335	1.670	1.658
UQ	1	118894801	118895100	5.680	2.886	4.541	3.498
UQ	1	119049601	119049900	1.413	0.612	0.769	0.812
UQ	1	119121601	119121900	0.345	0.865	0.580	0.583
UQ	1	119147401	119147700	0.824	0.491	0.419	0.391
UQ	1	119383801	119384100	0.769	0.442	0.419	0.137
UQ	1	119807701	119808000	0.239	0.678	0.638	0.507
UQ	1	119947201	119947500	3.478	2.620	2.562	4.066
UQ	1	120330301	120330600	1.091	0.867	0.584	0.806
UQ	1	120578101	120578400	1.515	0.699	0.531	0.861
UQ	1	120605701	120606000	0.586	0.139	0.165	0.309
UQ	1	120706801	120707100	2.016	1.056	1.489	1.577
UQ	1	120743701	120744000	0.610	0.232	0.341	0.299
UQ	1	120875401	120875700	0.398	0.725	0.189	0.162
UQ	1	120976201	120976500	0.558	0.258	0.165	0.051
UQ	1	121503601	121503900	1.699	1.073	1.106	1.091
UQ	1	121784701	121785000	2.397	1.939	1.333	1.719
UQ	1	122359501	122359800	0.132	0.420	0.267	0.385
UQ	1	122562301	122562600	0.240	1.107	0.798	1.019
UQ	1	122584801	122585100	1.249	0.794	0.515	0.466
UQ	1	122587801	122588100	3.562	2.894	2.118	2.718
UQ	1	122812501	122812800	0.211	0.470	0.531	0.416
UQ	1	122918701	122919000	0.639	0.281	0.296	0.284
UQ	1	124451701	124452000	0.558	0.281	0.292	0.243
UQ	1	124452001	124452300	0.507	0.258	0.292	0.187
UQ	1	125313301	125313600	0.637	0.420	0.267	0.187
UQ	1	125341201	125341500	3.060	1.682	2.361	2.469
UQ	1	125539201	125539500	0.794	0.395	0.214	0.304
UQ	1	125683801	125684100	0.769	0.376	0.272	0.162
UQ	1	125740801	125741100	0.768	0.305	0.292	0.304
UQ	1	125765701	125766000	4.128	1.956	2.178	1.023
UQ	1	127095901	127096200	0.582	0.232	0.317	0.193
UQ	1	127600801	127601100	0.132	0.775	0.448	0.522
UQ	1	128193301	128193600	0.505	0.307	0.185	0.081
UQ	1	128359501	128359800	0.611	0.421	0.292	0.360
UQ	1	128869201	128869500	1.010	0.657	0.341	0.639
UQ	1	130520701	130521000	1.197	0.513	0.638	0.695
UQ	1	130797001	130797300	0.481	0.163	0.181	0.137
UQ	1	132654301	132654600	0.477	0.234	0.049	0.051
UQ	1	133184101	133184400	0.477	0.234	0.272	0.000
UQ	1	133913101	133913400	0.984	0.333	0.267	0.279
UQ	1	134148301	134148600	0.160	0.423	0.374	0.391
UQ	1	135277501	135277800	0.689	0.397	0.263	0.274
UQ	1	135594601	135594900	0.636	0.118	0.156	0.193
UQ	1	136163701	136164000	0.532	0.981	0.667	0.385
UQ	1	136626001	136626300	0.527	1.028	0.983	0.223
UQ	1	136776901	136777200	2.218	0.180	0.087	0.427
UQ	1	136777201	136777500	1.173	0.182	0.111	0.330
UQ	1	136887601	136887900	0.666	0.328	0.317	0.335
UQ	1	137005801	137006100	0.426	0.699	0.111	0.187
UQ	1	137513401	137513700	1.645	1.165	0.646	0.781
UQ	1	137897401	137897700	3.855	2.367	3.105	2.656
UQ	1	138370501	138370800	1.431	0.331	0.538	0.106
UQ	1	138370801	138371100	1.460	0.380	0.702	0.182
UQ	1	138677101	138677400	1.086	0.345	0.218	0.426
UQ	1	139961701	139962000	0.505	0.185	0.267	0.112

UQ	1	140302801	140303100	0.557	0.142	0.111	0.228
UQ	1	140352601	140352900	0.481	0.144	0.136	0.031
UQ	1	140984101	140984400	0.081	0.515	0.403	0.335
UQ	1	141821401	141821700	0.852	0.562	0.424	0.472
UQ	1	142554601	142554900	0.721	1.375	1.991	1.425
UQ	1	143097001	143097300	0.690	0.165	0.419	0.248
UQ	1	143144401	143144700	0.663	0.257	0.230	0.101
UQ	1	143477101	143477400	1.170	1.335	0.930	0.608
UQ	1	144033901	144034200	0.584	0.376	0.374	0.167
UQ	1	144947401	144947700	1.113	0.657	0.560	0.608
UQ	1	146868901	146869200	0.053	0.423	0.317	0.274
UQ	1	146972101	146972400	1.036	0.749	0.691	0.700
UQ	1	147266101	147266400	0.265	0.702	0.560	0.492
UQ	1	148376101	148376400	1.229	3.197	3.018	3.356
UQ	1	149469601	149469900	0.292	0.725	0.453	0.547
UQ	1	150319501	150319800	0.932	0.678	0.613	0.588
UQ	1	151498801	151499100	0.531	0.163	0.058	0.182
UQ	1	152709601	152709900	0.161	0.699	0.814	0.857
UQ	1	153468001	153468300	0.481	0.163	0.132	0.051
UQ	1	153516001	153516300	0.398	0.884	0.831	0.887
UQ	1	154018201	154018500	3.026	2.403	1.719	1.770
UQ	1	154568401	154568700	3.672	1.538	2.793	1.861
UQ	1	157354801	157355100	0.455	0.163	0.189	0.081
UQ	1	160576501	160576800	0.908	1.967	1.879	2.251
UQ	1	161670901	161671200	1.221	0.496	0.546	0.558
UQ	1	161691001	161691300	0.450	0.210	0.292	0.112
UQ	1	162368101	162368400	0.821	0.468	0.473	0.335
UQ	1	162472501	162472800	0.716	0.373	0.103	0.446
UQ	1	162710101	162710400	1.412	0.695	0.695	0.801
UQ	1	164720401	164720700	0.640	0.330	0.292	0.193
UQ	1	164973601	164973900	0.158	0.375	0.555	0.329
UQ	1	166745701	166746000	0.690	0.283	0.399	0.421
UQ	1	167099401	167099700	0.610	0.279	0.296	0.000
UQ	1	167872501	167872800	0.187	0.493	0.399	0.360
UQ	1	169071001	169071300	1.118	0.584	0.444	0.421
UQ	1	169386901	169387200	0.424	0.727	0.667	0.968
UQ	1	169403101	169403400	4.034	1.641	2.287	2.596
UQ	1	170751001	170751300	1.065	0.303	0.341	0.288
UQ	1	170928601	170928900	0.532	0.373	0.395	0.832
UQ	1	170934901	170935200	0.265	0.657	0.506	0.553
UQ	1	172017901	172018200	1.489	0.468	0.794	0.481
UQ	1	172018201	172018500	2.078	0.522	0.682	0.420
UQ	1	172281301	172281600	0.132	0.468	0.473	0.446
UQ	1	173163601	173163900	0.345	0.513	1.032	0.608
UQ	1	173167801	173168100	0.452	0.562	0.584	0.553
UQ	1	173471701	173472000	0.661	0.165	0.176	0.198
UQ	1	173668201	173668500	0.132	0.448	0.506	0.477
UQ	1	174748201	174748500	0.663	0.236	0.103	0.243
UQ	1	174759901	174760200	1.245	0.611	0.675	0.492
UQ	1	175193101	175193400	0.347	0.515	0.555	0.918
UQ	1	175269301	175269600	0.213	0.468	0.473	0.502
UQ	1	175350301	175350600	0.636	0.238	0.292	0.223
UQ	1	176244601	176244900	0.584	0.210	0.078	0.329
UQ	1	176436901	176437200	0.716	0.184	0.214	0.380
UQ	1	177189001	177189300	1.889	1.189	1.616	1.334
UQ	1	177202501	177202800	0.347	0.654	0.506	0.639
UQ	1	177660601	177660900	0.319	0.165	0.588	0.106
UQ	1	178057201	178057500	0.747	0.281	0.082	0.289
UQ	1	178439101	178439400	0.326	1.753	1.835	1.892
UQ	1	179020801	179021100	0.773	0.165	0.189	0.187
UQ	1	179315701	179316000	0.613	0.307	0.399	0.112
UQ	1	179526901	179527200	0.132	0.446	0.399	0.461
UQ	1	179830201	179830500	1.360	0.461	0.593	0.558
UQ	1	179844301	179844600	0.213	0.539	0.502	0.411
UQ	1	181158301	181158600	0.239	0.584	0.477	0.137
UQ	1	181541401	181541700	0.423	0.096	0.107	0.112
UQ	1	181982101	181982400	0.187	0.446	0.481	0.391

UQ	1	181997101	181997400	0.477	0.118	0.210	0.223
UQ	1	182079001	182079300	1.915	1.000	1.280	0.963
UQ	1	182453101	182453400	0.957	0.678	0.346	0.335
UQ	1	183473701	183474000	1.279	0.680	0.551	0.664
UQ	1	183586501	183586800	0.769	0.609	0.292	0.391
UQ	1	183717901	183718200	0.558	0.257	0.210	0.248
UQ	1	183874801	183875100	0.266	0.444	0.502	0.167
UQ	1	184761901	184762200	1.168	0.798	0.395	0.781
UQ	1	184955401	184955700	0.587	0.303	0.317	0.081
UQ	1	185019901	185020200	0.929	0.404	0.218	0.391
UQ	1	185030701	185031000	0.318	0.515	0.502	0.527
UQ	1	185969701	185970000	0.397	0.747	0.185	0.776
UQ	1	186103201	186103500	2.920	1.791	1.239	1.561
UQ	1	186109201	186109500	0.239	0.539	0.531	0.644
UQ	1	186822301	186822600	1.011	0.631	0.531	0.441
UQ	1	186856501	186856800	1.116	0.657	0.769	0.740
UQ	1	187154401	187154700	0.186	0.588	0.617	0.522
UQ	1	187171501	187171800	0.239	0.421	0.662	0.502
UQ	1	187239901	187240200	0.794	0.255	0.132	0.248
UQ	1	187389301	187389600	1.828	0.639	0.945	0.918
UQ	1	187389601	187389900	2.465	0.777	1.287	1.334
UQ	1	187805101	187805400	0.984	0.749	0.740	0.553
UQ	1	188018101	188018400	1.274	0.609	0.740	0.623
UQ	1	188151001	188151300	0.531	0.234	0.185	0.218
UQ	1	188697301	188697600	1.324	1.822	2.406	1.912
UQ	1	188811901	188812200	0.079	0.444	0.321	0.355
UQ	1	189053101	189053400	0.586	0.305	0.317	0.243
UQ	1	189107701	189108000	0.186	0.420	0.526	0.497
UQ	1	189157801	189158100	1.224	0.427	0.691	0.228
UQ	1	189172501	189172800	0.160	0.442	0.346	0.355
UQ	1	190188001	190188300	0.773	0.348	0.428	0.492
UQ	1	190743001	190743300	5.317	3.896	4.167	4.806
UQ	1	191719501	191719800	0.213	0.723	0.749	0.598
UQ	1	192477001	192477300	0.108	0.558	0.419	0.578
UQ	1	192798601	192798900	1.063	1.262	0.905	0.852
UQ	1	193793701	193794000	0.558	0.257	0.263	0.391
UQ	1	194345101	194345400	0.986	0.348	0.613	0.634
UQ	1	194396701	194397000	2.371	1.315	1.371	0.664
UQ	1	194440801	194441100	0.240	0.517	0.531	0.472
UQ	1	195059101	195059400	1.089	0.676	0.453	0.887
UQ	1	195263401	195263700	0.477	0.096	0.058	0.101
UQ	1	195482401	195482700	0.850	0.401	0.370	0.391
UQ	1	195911701	195912000	0.105	0.279	0.341	0.527
UQ	1	197357401	197357700	0.160	0.281	0.477	0.279
UQ	1	197665801	197666100	0.318	0.515	0.136	0.142
UQ	1	199113601	199113900	0.213	0.421	0.428	0.608
UQ	1	199116001	199116300	0.586	0.277	0.370	0.167
UQ	1	200709601	200709900	0.213	0.583	0.872	0.466
UQ	1	201010201	201010500	0.984	0.425	0.584	0.573
UQ	1	201058201	201058500	1.833	2.596	2.078	2.601
UQ	1	201426001	201426300	0.931	0.629	0.424	0.492
UQ	1	201994801	201995100	0.745	0.191	0.321	0.173
UQ	1	205133101	205133400	0.984	0.309	0.272	0.258
UQ	1	206272801	206273100	0.345	0.751	0.107	0.112
UQ	1	206655601	206655900	0.665	0.096	0.107	0.162
UQ	1	207542701	207543000	0.505	0.328	0.239	0.167
UQ	1	207820801	207821100	0.532	0.140	0.132	0.218
UQ	1	208268701	208269000	0.400	0.303	0.506	0.304
UQ	1	208557001	208557300	0.160	0.607	0.481	0.461
UQ	1	209506501	209506800	0.694	0.212	0.107	0.335
UQ	1	209690401	209690700	1.437	1.144	0.700	0.913
UQ	1	209947201	209947500	0.507	0.096	0.156	0.081
UQ	1	210042601	210042900	0.613	0.212	0.288	0.218
UQ	1	210621001	210621300	0.984	0.704	0.609	0.608
UQ	1	213459901	213460200	0.715	1.199	0.292	0.218
UQ	1	213698401	213698700	0.771	0.326	0.531	0.411
UQ	1	213935101	213935400	0.477	0.167	0.136	0.137

UQ	1	214650901	214651200	0.665	0.240	0.111	0.218
UQ	1	215287801	215288100	1.013	0.539	0.506	0.279
UQ	1	215489401	215489700	0.611	0.165	0.399	0.826
UQ	1	215662201	215662500	0.531	0.397	0.267	0.304
UQ	1	215980201	215980500	2.432	0.854	0.584	0.877
UQ	1	216001501	216001800	0.639	0.305	0.107	0.223
UQ	1	216318001	216318300	0.797	0.470	0.444	0.472
UQ	1	216880501	216880800	1.118	0.401	0.395	0.400
UQ	1	217551601	217551900	0.481	0.163	0.165	0.142
UQ	1	218864101	218864400	2.149	1.289	1.349	1.303
UQ	1	221392201	221392500	0.692	0.118	0.403	0.198
UQ	1	222224701	222225000	0.586	0.189	0.160	0.299
UQ	1	222225001	222225300	0.774	0.234	0.218	0.416
UQ	1	222860401	222860700	0.452	0.584	0.214	0.659
UQ	1	222901801	222902100	2.842	1.650	1.839	1.977
UQ	1	224636701	224637000	0.557	0.161	0.243	0.142
UQ	1	225030301	225030600	0.319	0.562	0.453	0.441
UQ	1	225474901	225475200	0.558	0.444	0.370	0.335
UQ	1	225972301	225972600	0.586	0.142	0.292	0.365
UQ	1	226512901	226513200	1.707	2.882	2.802	2.758
UQ	1	227088301	227088600	2.870	2.311	2.127	2.083
UQ	1	229152901	229153200	0.692	0.185	0.481	0.502
UQ	1	229188601	229188900	0.452	0.841	0.239	0.360
UQ	1	230797201	230797500	0.557	0.140	0.156	0.061
UQ	1	231006901	231007200	1.091	0.260	0.403	0.578
UQ	1	231007201	231007500	0.878	0.069	0.301	0.294
UQ	1	231354001	231354300	0.481	0.118	0.239	0.193
UQ	1	232221601	232221900	2.473	3.150	2.998	3.235
UQ	1	232798501	232798800	0.481	0.140	0.239	0.248
UQ	1	233042701	233043000	7.311	5.915	6.023	6.378
UQ	1	233238901	233239200	2.397	1.895	1.444	1.516
UQ	1	235212901	235213200	0.690	0.140	0.127	0.218
UQ	1	235213201	235213500	0.876	0.159	0.132	0.405
UQ	1	235659601	235659900	0.398	0.699	0.682	1.055
UQ	1	235765201	235765500	0.319	0.421	0.185	0.497
UQ	1	235923001	235923300	0.131	0.375	0.370	0.466
UQ	1	237290401	237290700	0.668	1.821	1.086	1.318
UQ	1	237915001	237915300	0.637	0.468	0.481	0.304
UQ	1	238424401	238424700	0.453	0.890	0.789	0.751
UQ	1	239013601	239013900	0.426	0.560	0.160	0.329
UQ	1	239569201	239569500	0.926	0.331	0.317	0.122
UQ	1	239862901	239863200	0.532	0.279	0.082	0.254
UQ	1	240563101	240563400	0.476	0.023	0.189	0.112
UQ	1	240672901	240673200	0.663	0.232	0.176	0.193
UQ	1	240774901	240775200	0.532	0.185	0.136	0.360
UQ	1	241007401	241007700	0.718	0.260	0.181	0.178
UQ	1	241424401	241424700	0.294	0.747	0.054	0.167
UQ	1	241466401	241466700	0.984	0.444	0.568	0.553
UQ	1	242328901	242329200	0.587	0.234	0.350	0.086
UQ	1	242610901	242611200	0.584	0.283	0.029	0.198
UQ	1	242974501	242974800	2.739	3.158	2.468	3.047
UQ	1	243177301	243177600	2.531	1.886	0.921	1.770
UQ	1	243594301	243594600	1.710	2.600	0.439	0.765
UQ	1	244090501	244090800	0.347	0.657	0.662	0.684
UQ	1	244270801	244271100	0.213	0.303	0.506	0.497
UQ	1	244863601	244863900	0.261	1.491	1.052	0.893
UQ	1	245148001	245148300	0.955	0.581	0.370	0.583
UQ	1	245361901	245362200	3.489	1.776	2.829	2.621
UQ	1	245879701	245880000	0.828	0.262	0.181	0.187
UQ	1	245947201	245947500	0.616	0.210	0.082	0.223
UQ	1	246376501	246376800	0.558	0.232	0.165	0.167
UQ	1	246385801	246386100	2.011	0.701	0.325	0.370
UQ	1	246418801	246419100	1.699	0.583	0.733	0.878
UQ	1	246450001	246450300	1.657	0.817	0.283	0.263
UQ	1	247376701	247377000	0.132	0.279	0.555	0.248
UQ	1	247510801	247511100	0.853	0.423	0.374	0.329
UQ	1	248373901	248374200	0.879	0.354	0.386	0.218

UQ	1	248835001	248835300	1.140	0.442	0.526	0.588
UQ	1	250108801	250109100	0.586	0.208	0.239	0.198
UQ	1	250270801	250271100	1.092	0.586	0.653	0.228
UQ	1	251711401	251711700	0.613	0.283	0.181	0.360
UQ	1	251817301	251817600	0.557	0.234	0.181	0.193
UQ	1	251971801	251972100	0.503	0.234	0.189	0.137
UQ	1	252177301	252177600	0.532	0.373	0.267	0.218
UQ	1	252264901	252265200	0.431	1.141	1.057	1.420
UQ	1	252426301	252426600	1.086	2.637	1.741	1.942
UQ	1	253041001	253041300	0.319	0.444	0.239	0.472
UQ	1	253249501	253249800	0.000	0.446	0.379	0.223
UQ	1	253374301	253374600	1.140	0.536	0.617	0.391
UQ	1	253395001	253395300	0.558	0.140	0.152	0.112
UQ	1	253572901	253573200	1.382	0.770	0.379	0.796
UQ	1	254096101	254096400	0.771	0.890	0.424	0.466
UQ	1	254433601	254433900	0.774	0.348	0.424	0.304
UQ	1	255562201	255562500	1.063	0.706	0.695	0.634
UQ	1	256386601	256386900	0.082	0.513	0.374	0.304
UQ	1	256449901	256450200	1.116	0.725	0.765	0.563
UQ	1	256450201	256450500	1.036	0.468	0.461	0.452
UQ	1	256645501	256645800	0.718	0.493	0.477	0.299
UQ	1	256711801	256712100	0.982	0.678	0.604	0.497
UQ	1	257725801	257726100	0.561	0.163	0.054	0.193
UQ	1	257788501	257788800	0.929	0.465	0.551	0.329
UQ	1	258512101	258512400	0.532	0.163	0.132	0.025
UQ	1	259590001	259590300	1.544	1.755	1.275	2.013
UQ	1	259956601	259956900	2.686	1.734	2.060	1.942
UQ	1	260022601	260022900	0.745	0.397	0.205	0.213
UQ	1	260023801	260024100	5.023	2.765	2.717	2.659
UQ	1	260029501	260029800	0.531	0.260	0.239	0.223
UQ	1	260050801	260051100	0.026	0.348	0.239	0.466
UQ	1	260160601	260160900	0.532	0.702	0.424	0.416
UQ	1	260181301	260181600	2.609	1.262	0.682	1.502
UQ	1	260596201	260596500	1.916	1.379	1.251	1.541
UQ	1	260603401	260603700	1.887	0.940	1.567	1.552
UQ	1	260668801	260669100	0.955	0.541	0.457	0.411
UQ	1	260782501	260782800	0.932	0.631	0.613	0.527
UQ	1	260984101	260984400	1.755	1.334	2.107	1.389
UQ	1	261310201	261310500	1.091	1.285	0.695	0.751
UQ	1	261373801	261374100	0.560	0.865	0.312	0.943
UQ	1	261405001	261405300	4.364	2.390	4.011	3.012
UQ	1	261437401	261437700	0.532	0.161	0.267	0.137
UQ	1	261468301	261468600	0.694	0.300	0.292	0.274
UQ	1	261579001	261579300	1.357	0.813	1.012	0.608
UQ	1	261619801	261620100	1.807	0.777	0.635	0.578
UQ	1	261645301	261645600	0.584	0.208	0.399	0.385
UQ	1	261873301	261873600	1.194	1.704	1.592	1.739
UQ	1	261885001	261885300	1.436	0.914	0.700	1.080
UQ	1	261889201	261889500	0.665	0.281	0.263	0.365
UQ	1	261912901	261913200	0.558	0.208	0.210	0.279
UQ	1	261937501	261937800	0.502	1.188	1.032	1.552
UQ	1	261949501	261949800	2.657	1.185	0.785	0.822
UQ	1	261949801	261950100	1.674	0.663	0.346	0.472
UQ	1	261973801	261974100	0.747	0.114	0.481	0.335
UQ	1	262024501	262024800	1.170	0.397	0.513	0.365
UQ	1	262033801	262034100	1.007	0.564	0.584	1.465
UQ	1	262067401	262067700	1.249	0.869	0.658	0.720
UQ	1	262160401	262160700	2.762	1.633	1.741	2.089
UQ	1	262229401	262229700	2.975	2.315	3.538	3.884
UQ	1	262412401	262412700	1.307	0.865	0.881	0.801
UQ	1	262598701	262599000	0.745	0.260	0.205	0.106
UQ	1	262710301	262710600	2.746	1.470	1.543	1.537
UQ	1	262908001	262908300	0.373	0.491	0.267	0.279
UQ	1	262960201	262960500	0.931	0.534	0.457	0.466
UQ	1	263098201	263098500	0.295	0.957	0.617	0.720
UQ	1	263118601	263118900	3.860	2.384	2.562	2.926
UQ	1	263157901	263158200	1.032	0.491	0.555	0.527

UQ	1	263196901	263197200	0.505	0.142	0.239	0.193
UQ	1	263200501	263200800	1.391	2.986	3.215	2.611
UQ	1	263739601	263739900	0.613	0.279	0.263	0.309
UQ	1	263739901	263740200	0.848	0.326	0.267	0.329
UQ	1	263814601	263814900	4.388	3.694	3.846	3.291
UQ	1	263895001	263895300	1.279	0.815	0.909	1.627
UQ	1	264132901	264133200	1.333	2.105	1.786	1.780
UQ	1	264280501	264280800	4.183	2.907	3.107	1.851
UQ	1	264878101	264878400	1.432	0.706	0.823	0.948
UQ	1	264906301	264906600	0.132	0.328	0.613	0.391
UQ	1	264909601	264909900	0.081	0.421	0.749	0.452
UQ	1	264931801	264932100	0.828	1.309	1.193	1.267
UQ	1	265668001	265668300	1.542	0.708	0.535	1.044
UQ	1	266610301	266610600	0.876	0.631	0.107	0.492
UQ	1	266778901	266779200	0.000	0.305	0.321	0.436
UQ	1	266972101	266972400	0.800	0.378	0.374	0.421
UQ	1	267012601	267012900	0.694	1.169	0.341	1.212
UQ	1	267065401	267065700	2.229	1.526	1.543	1.197
UQ	1	267377101	267377400	0.052	0.328	0.560	0.167
UQ	1	267591001	267591300	1.303	0.865	0.412	0.573
UQ	1	267692401	267692700	3.675	2.742	2.686	3.006
UQ	1	268090201	268090500	2.421	1.525	1.638	1.618
UQ	1	268185901	268186200	0.452	0.629	0.638	0.304
UQ	1	268422301	268422600	0.507	0.232	0.292	0.243
UQ	1	268713601	268713900	0.663	0.303	0.107	0.329
UQ	1	268887001	268887300	1.379	0.888	0.531	0.720
UQ	1	268956901	268957200	0.720	0.352	0.239	0.416
UQ	1	268962901	268963200	0.853	0.356	0.283	0.248
UQ	1	269383801	269384100	0.373	0.210	0.000	0.466
UQ	1	269725201	269725500	0.345	0.699	0.823	0.558
UQ	1	269795101	269795400	4.181	2.854	2.490	2.074
UQ	1	270208501	270208800	0.886	0.165	0.103	0.142
UQ	1	270212701	270213000	0.666	0.092	0.181	0.051
UQ	1	270293401	270293700	0.584	0.212	0.350	0.304
UQ	1	270348001	270348300	0.373	0.444	0.531	0.664
UQ	1	270804901	270805200	1.644	1.006	0.896	1.044
UQ	1	270981901	270982200	2.155	1.330	1.317	1.471
UQ	1	271343701	271344000	1.779	2.671	2.365	2.484
UQ	1	271619401	271619700	3.299	2.341	2.031	2.013
UQ	1	271679701	271680000	0.773	0.114	0.082	0.385
UQ	1	271680001	271680300	0.715	0.094	0.058	0.329
UQ	1	271836901	271837200	0.292	0.045	0.136	0.527
UQ	1	272063701	272064000	0.611	0.983	0.374	0.391
UQ	1	272118901	272119200	1.676	3.664	2.300	3.615
UQ	1	272219701	272220000	0.745	0.397	0.374	0.274
UQ	1	272553901	272554200	4.306	3.673	3.703	3.006
UQ	1	272700001	272700300	0.826	0.305	0.301	0.466
UQ	1	272703301	272703600	1.302	1.025	0.555	1.024
UQ	1	273079201	273079500	4.794	2.030	3.054	2.165
UQ	1	273257101	273257400	1.434	2.193	1.801	2.074
UQ	1	273476101	273476400	1.279	2.409	1.975	2.357
UQ	1	273996301	273996600	0.747	0.277	0.239	0.223
UQ	1	274110601	274110900	10.670	7.556	7.661	7.389
UQ	1	274119301	274119600	0.292	0.539	0.453	0.695
UQ	1	274668001	274668300	0.477	0.120	0.185	0.187
UQ	1	275045101	275045400	2.071	1.401	1.522	1.683
UQ	1	275204101	275204400	1.884	0.807	0.700	0.887
UQ	1	275328601	275328900	3.194	2.410	2.361	2.231
UQ	10	33601	33900	0.187	0.494	0.555	0.527
UQ	10	1263901	1264200	0.771	0.562	0.638	0.578
UQ	10	1768801	1769100	0.634	0.210	0.181	0.106
UQ	10	2150101	2150400	0.611	0.281	0.794	0.167
UQ	10	2200801	2201100	4.276	2.306	2.160	3.053
UQ	10	2677201	2677500	0.769	0.283	0.140	0.248
UQ	10	2715001	2715300	0.718	0.511	0.267	0.355
UQ	10	3006901	3007200	0.503	0.068	0.214	0.051
UQ	10	5499301	5499600	3.067	1.137	1.658	1.715

UQ	10	6161101	6161400	0.345	0.817	0.881	0.988
UQ	10	8200201	8200500	0.610	0.373	0.160	0.254
UQ	10	8300101	8300400	0.239	0.676	0.506	0.517
UQ	10	9059701	9060000	0.932	0.168	0.227	0.177
UQ	10	9546601	9546900	0.666	0.423	0.214	0.304
UQ	10	9584401	9584700	0.106	0.538	0.473	0.466
UQ	10	10526401	10526700	0.560	0.328	0.239	0.279
UQ	10	10899901	10900200	0.450	0.185	0.189	0.167
UQ	10	10951501	10951800	1.671	1.009	0.798	1.105
UQ	10	10995001	10995300	0.637	0.376	0.103	0.248
UQ	10	11038201	11038500	4.096	3.178	3.098	3.377
UQ	10	11168701	11169000	3.254	1.568	1.444	1.293
UQ	10	11352001	11352300	2.552	0.772	1.187	0.903
UQ	10	11533201	11533500	2.047	2.547	1.556	2.738
UQ	10	11613601	11613900	0.613	0.094	0.214	0.182
UQ	10	11736601	11736900	1.300	2.130	0.930	0.933
UQ	10	11937301	11937600	0.692	0.000	0.140	0.248
UQ	10	11989501	11989800	2.973	4.746	3.999	3.793
UQ	10	12297601	12297900	0.426	0.633	0.239	0.243
UQ	10	12581701	12582000	0.962	1.358	1.538	1.465
UQ	10	13411201	13411500	0.505	0.094	0.054	0.101
UQ	10	13866901	13867200	1.197	0.519	0.374	0.263
UQ	10	13882801	13883100	1.094	0.659	0.531	0.385
UQ	10	14115001	14115300	0.345	0.609	0.165	0.608
UQ	10	14180401	14180700	3.455	3.015	2.702	2.155
UQ	10	14246701	14247000	2.100	1.749	1.217	1.658
UQ	10	14766301	14766600	1.061	0.347	0.272	0.400
UQ	10	15051301	15051600	5.243	3.804	3.879	4.178
UQ	10	15110401	15110700	0.636	0.236	0.243	0.329
UQ	10	15227701	15228000	0.718	1.051	0.448	0.329
UQ	10	15530101	15530400	1.063	0.628	0.575	0.411
UQ	10	15623101	15623400	0.852	0.375	0.609	0.385
UQ	10	15783301	15783600	0.957	1.873	0.609	0.628
UQ	10	16379401	16379700	3.354	2.448	2.267	2.494
UQ	10	17122801	17123100	0.507	0.302	0.185	0.248
UQ	10	18338101	18338400	0.292	0.727	0.103	0.578
UQ	10	19224901	19225200	1.221	1.562	1.942	1.775
UQ	10	19403401	19403700	4.004	2.466	1.743	1.994
UQ	10	20241901	20242200	0.690	0.328	0.560	0.223
UQ	10	20428201	20428500	0.931	0.399	0.410	0.254
UQ	10	20428501	20428800	0.505	0.163	0.263	0.254
UQ	10	20814601	20814900	0.799	0.238	0.189	0.238
UQ	10	20818201	20818500	3.426	2.759	2.978	3.037
UQ	10	21521701	21522000	0.853	0.444	0.453	0.578
UQ	10	21656101	21656400	2.500	1.512	1.032	1.302
UQ	10	21693901	21694200	0.558	0.303	0.247	0.304
UQ	10	21708001	21708300	0.984	1.216	0.633	1.105
UQ	10	21803101	21803400	2.298	0.277	0.116	0.117
UQ	10	21803401	21803700	0.874	0.165	0.025	0.117
UQ	10	22063801	22064100	0.507	0.096	0.263	0.243
UQ	10	22173301	22173600	0.902	0.232	0.078	0.076
UQ	10	22191601	22191900	0.503	1.472	0.892	1.166
UQ	10	22304101	22304400	1.626	0.961	1.041	1.192
UQ	10	22590301	22590600	0.613	0.399	0.321	0.304
UQ	10	22613701	22614000	2.971	2.030	1.866	0.994
UQ	10	22616401	22616700	1.142	2.573	1.739	1.633
UQ	10	22693201	22693500	0.423	1.523	1.197	1.101
UQ	10	23303101	23303400	1.066	0.682	0.457	0.497
UQ	10	24339901	24340200	0.265	0.375	0.560	0.497
UQ	10	25059001	25059300	0.027	0.236	0.267	0.614
UQ	10	25595401	25595700	1.913	1.639	1.387	1.653
UQ	10	25971301	25971600	0.452	1.079	0.745	0.948
UQ	10	26084101	26084400	0.292	0.493	0.136	0.137
UQ	10	27079201	27079500	1.779	1.452	1.275	1.404
UQ	10	28382401	28382700	0.589	0.071	0.136	0.076
UQ	10	29753101	29753400	0.876	0.676	0.662	0.690
UQ	10	29836201	29836500	3.909	3.116	3.250	3.017

UQ	10	30028501	30028800	1.910	1.330	1.378	1.181
UQ	10	30261601	30261900	0.984	0.468	0.555	0.700
UQ	10	30320701	30321000	1.011	0.749	0.827	0.751
UQ	10	30345901	30346200	2.371	0.895	0.883	1.222
UQ	10	30346201	30346500	1.173	0.401	0.638	0.614
UQ	10	30655201	30655500	2.392	1.804	1.362	1.790
UQ	10	30771601	30771900	0.557	0.232	0.136	0.167
UQ	10	30909001	30909300	0.053	0.448	0.341	0.695
UQ	10	31130101	31130400	1.674	0.910	0.814	0.893
UQ	10	31130401	31130700	3.078	1.706	2.089	2.089
UQ	10	31523101	31523400	1.170	0.935	0.695	0.664
UQ	10	31535401	31535700	0.824	0.212	0.564	0.502
UQ	10	31581901	31582200	0.106	0.466	0.239	0.385
UQ	10	31622401	31622700	0.132	0.350	0.267	0.466
UQ	10	32340001	32340300	0.531	1.217	1.317	1.156
UQ	10	32664601	32664900	4.381	3.010	2.887	2.596
UQ	10	32664901	32665200	4.891	3.789	3.005	3.519
UQ	10	32672401	32672700	0.615	0.253	0.160	0.248
UQ	10	33065101	33065400	0.799	0.277	0.292	0.472
UQ	10	33198001	33198300	2.028	1.152	1.032	1.136
UQ	10	33198901	33199200	1.704	1.068	1.052	0.913
UQ	10	33666301	33666600	1.358	0.963	0.720	0.933
UQ	10	34012501	34012800	1.760	0.955	0.916	0.639
UQ	10	34025401	34025700	0.374	0.680	0.823	0.882
UQ	10	34434901	34435200	2.994	1.345	1.558	1.243
UQ	10	34435201	34435500	2.763	1.015	1.735	1.328
UQ	10	34468501	34468800	2.373	0.824	0.778	1.258
UQ	10	34472101	34472400	2.693	0.571	0.818	1.076
UQ	10	34503301	34503600	0.689	0.352	0.234	0.274
UQ	10	34635301	34635600	0.771	1.212	1.411	1.172
UQ	10	34689001	34689300	2.308	0.635	1.340	0.898
UQ	10	34704901	34705200	1.118	0.678	0.473	0.573
UQ	10	34707601	34707900	0.848	0.397	0.497	0.416
UQ	10	34800001	34800300	0.932	0.588	0.424	0.391
UQ	10	35051401	35051700	3.694	2.517	2.640	2.479
UQ	10	35285401	35285700	2.526	1.957	2.015	1.901
UQ	10	35447101	35447400	1.355	0.729	1.012	1.055
UQ	10	35838901	35839200	1.942	1.148	1.329	1.597
UQ	10	35851501	35851800	0.450	1.174	0.925	0.933
UQ	10	36575701	36576000	0.560	0.234	0.292	0.385
UQ	10	36588001	36588300	0.795	1.216	1.119	1.131
UQ	10	36698401	36698700	1.913	1.289	1.316	1.293
UQ	10	37104901	37105200	0.589	0.116	0.185	0.223
UQ	10	37381501	37381800	2.045	1.092	1.358	2.687
UQ	10	37504501	37504800	0.665	0.818	1.090	0.492
UQ	10	37756501	37756800	0.532	0.257	0.370	0.162
UQ	10	38163001	38163300	0.663	0.421	0.346	0.304
UQ	10	38460301	38460600	0.958	0.494	0.468	0.213
UQ	10	39089401	39089700	1.013	0.534	0.205	0.157
UQ	10	39312901	39313200	1.039	0.701	0.457	0.776
UQ	10	39374101	39374400	1.434	0.935	0.473	0.456
UQ	10	41189101	41189400	0.689	0.354	0.296	0.223
UQ	10	41369701	41370000	0.134	0.444	0.510	0.452
UQ	10	41485801	41486100	0.450	0.140	0.185	0.106
UQ	10	42111001	42111300	0.423	0.140	0.000	0.081
UQ	10	42683401	42683700	0.505	0.584	0.399	0.360
UQ	10	42902701	42903000	0.586	0.466	0.321	0.751
UQ	10	42988201	42988500	0.477	0.163	0.185	0.081
UQ	10	43413901	43414200	0.131	0.751	0.531	0.608
UQ	10	43555801	43556100	0.560	0.185	0.185	0.254
UQ	10	43606501	43606800	0.611	0.330	0.346	0.061
UQ	10	44302801	44303100	1.010	0.674	0.711	0.608
UQ	10	44838901	44839200	1.089	0.772	0.765	0.771
UQ	10	45451801	45452100	1.547	0.796	0.756	0.765
UQ	10	46258801	46259100	0.134	0.489	0.633	0.522
UQ	10	46528201	46528500	1.037	1.216	0.667	0.695
UQ	10	47067901	47068200	0.318	0.725	0.658	0.639

UQ	10	47654701	47655000	1.087	1.776	1.592	1.739
UQ	10	49446301	49446600	0.824	0.397	0.609	0.385
UQ	10	49840201	49840500	1.036	0.935	0.823	0.664
UQ	10	50119501	50119800	0.610	1.217	0.399	0.826
UQ	10	50773801	50774100	2.047	2.457	1.213	1.389
UQ	10	50956201	50956500	3.191	1.788	1.668	1.704
UQ	10	51025501	51025800	1.542	1.127	1.008	0.963
UQ	10	51053401	51053700	0.982	0.772	0.687	1.212
UQ	10	51149401	51149700	1.305	0.592	0.575	0.507
UQ	10	51668701	51669000	0.505	0.189	0.165	0.162
UQ	10	52150801	52151100	0.639	0.326	0.259	0.355
UQ	10	53393101	53393400	0.079	0.352	0.486	0.416
UQ	10	53762701	53763000	0.026	0.352	0.535	0.167
UQ	10	54193501	54193800	0.532	0.305	0.165	0.279
UQ	10	54574501	54574800	1.060	2.787	1.908	2.307
UQ	10	55077601	55077900	0.613	0.234	0.243	0.254
UQ	10	57271801	57272100	0.481	0.139	0.078	0.081
UQ	10	57365701	57366000	0.052	0.491	0.210	0.193
UQ	10	57639301	57639600	0.744	0.418	0.317	0.527
UQ	10	58893301	58893600	0.558	0.328	0.263	0.193
UQ	10	59252101	59252400	0.507	0.210	0.370	0.137
UQ	10	61386301	61386600	0.240	0.652	0.424	0.416
UQ	10	61798801	61799100	0.558	0.234	0.123	0.193
UQ	10	62212501	62212800	0.108	0.631	0.292	0.248
UQ	10	62415301	62415600	0.476	0.049	0.107	0.112
UQ	10	63330601	63330900	1.784	0.983	0.934	1.226
UQ	10	67237501	67237800	0.955	0.465	0.510	0.405
UQ	10	67254601	67254900	1.489	2.263	0.749	2.023
UQ	10	67540801	67541100	0.505	0.232	0.234	0.106
UQ	10	68100601	68100900	0.558	0.255	0.267	0.335
UQ	10	69155701	69156000	0.586	0.373	0.296	0.248
UQ	10	69312901	69313200	0.876	0.279	0.477	0.507
UQ	10	69462901	69463200	0.240	0.517	0.983	0.710
UQ	10	69566701	69567000	0.769	0.255	0.049	0.218
UQ	10	69894601	69894900	0.584	0.116	0.054	0.076
UQ	10	69894901	69895200	0.477	0.112	0.029	0.051
UQ	10	71604901	71605200	0.213	0.584	0.082	0.329
UQ	10	71988901	71989200	0.240	0.423	0.049	0.000
UQ	10	73110001	73110300	0.532	0.326	0.321	0.142
UQ	10	73814401	73814700	0.053	0.423	0.399	0.628
UQ	10	74095201	74095500	0.558	0.234	0.259	0.304
UQ	10	74156101	74156400	0.081	0.187	0.531	0.304
UQ	10	74177701	74178000	0.531	0.116	0.103	0.198
UQ	10	74219101	74219400	1.010	0.303	0.568	0.553
UQ	10	74219401	74219700	0.823	0.185	0.403	0.446
UQ	10	74262901	74263200	4.683	3.607	3.397	2.763
UQ	10	74905801	74906100	1.195	0.702	0.593	0.284
UQ	10	75224701	75225000	4.647	3.116	3.067	3.002
UQ	10	75781501	75781800	3.109	2.287	2.002	2.261
UQ	10	75942601	75942900	0.745	0.303	0.205	0.157
UQ	10	75981301	75981600	3.407	2.182	1.917	2.469
UQ	10	76455901	76456200	0.692	0.513	0.399	0.776
UQ	10	76527001	76527300	0.881	0.258	0.481	0.502
UQ	10	76743001	76743300	0.476	1.054	0.856	0.994
UQ	10	76897801	76898100	0.290	0.609	0.613	0.553
UQ	10	77154901	77155200	0.106	0.442	0.239	0.248
UQ	10	77962501	77962800	2.926	2.030	0.713	1.703
UQ	10	78173701	78174000	0.824	0.403	0.428	0.279
UQ	10	78427201	78427500	3.588	2.849	2.949	2.981
UQ	10	78706201	78706500	1.224	0.729	0.711	0.705
UQ	10	79070101	79070400	0.769	0.468	0.346	0.608
UQ	10	79339801	79340100	0.505	0.163	0.078	0.182
UQ	10	80037001	80037300	0.799	0.562	0.481	0.608
UQ	10	80314501	80314800	1.595	0.187	0.243	0.582
UQ	10	81001501	81001800	0.376	0.656	0.983	1.217
UQ	10	81205501	81205800	1.573	2.156	2.015	1.019
UQ	10	81249301	81249600	1.041	0.257	0.214	0.299

UQ	10	81400201	81400500	0.771	0.373	0.341	0.355
UQ	10	81622801	81623100	0.294	0.562	0.399	0.583
UQ	10	81632401	81632700	1.807	1.103	1.086	1.227
UQ	10	81854401	81854700	1.491	0.865	0.987	0.634
UQ	10	81854701	81855000	2.665	1.244	1.223	0.968
UQ	10	82083301	82083600	0.477	0.139	0.098	0.051
UQ	10	82593901	82594200	0.637	0.421	0.156	0.416
UQ	10	82716901	82717200	0.132	0.446	0.424	0.279
UQ	10	82717201	82717500	1.010	0.749	1.329	0.776
UQ	10	83190601	83190900	0.534	0.142	0.156	0.274
UQ	10	83224501	83224800	0.558	0.305	0.350	0.335
UQ	10	83226601	83226900	0.453	1.309	0.584	0.771
UQ	10	83592301	83592600	1.547	0.592	0.247	0.345
UQ	10	83616901	83617200	2.822	1.835	1.313	1.663
UQ	10	83800801	83801100	2.394	1.760	1.723	1.688
UQ	10	83826001	83826300	3.736	2.208	1.944	2.196
UQ	10	83881501	83881800	1.808	0.845	1.102	1.181
UQ	10	84050401	84050700	2.452	1.569	0.979	1.663
UQ	10	84081601	84081900	6.196	4.456	3.727	3.244
UQ	10	84133801	84134100	0.823	1.006	0.560	0.578
UQ	10	84222301	84222600	6.946	4.954	4.882	5.618
UQ	10	84395701	84396000	3.352	1.850	1.953	2.251
UQ	10	84399001	84399300	2.720	1.480	1.486	1.055
UQ	10	84629401	84629700	0.558	0.399	0.370	0.385
UQ	10	84667801	84668100	3.907	2.766	2.216	2.779
UQ	10	84728701	84729000	0.477	0.260	0.132	0.056
UQ	10	85128301	85128600	1.755	1.496	1.246	0.826
UQ	10	85142701	85143000	4.259	3.251	3.119	2.733
UQ	10	85448101	85448400	0.823	0.395	0.531	0.466
UQ	10	85466701	85467000	2.739	1.909	1.580	1.800
UQ	10	85492501	85492800	8.469	4.562	5.957	5.542
UQ	10	85983901	85984200	2.045	1.377	1.108	1.151
UQ	11	199801	200100	2.396	2.875	1.514	1.734
UQ	11	1446601	1446900	0.558	0.236	0.259	0.167
UQ	11	2187301	2187600	0.690	0.277	0.267	0.213
UQ	11	2187601	2187900	0.957	0.257	0.716	0.274
UQ	11	2872801	2873100	1.587	0.403	0.604	0.193
UQ	11	4238101	4238400	0.186	0.352	0.317	0.578
UQ	11	4466401	4466700	0.532	0.097	0.132	0.243
UQ	11	4519801	4520100	0.297	0.985	0.983	0.720
UQ	11	4921801	4922100	0.055	0.566	0.629	0.527
UQ	11	4958101	4958400	1.762	0.972	0.818	2.591
UQ	11	5087101	5087400	1.145	0.440	0.560	0.608
UQ	11	5230201	5230500	3.526	6.881	6.055	5.558
UQ	11	5650501	5650800	0.769	0.423	0.374	0.436
UQ	11	5682001	5682300	0.263	0.680	0.760	0.857
UQ	11	5682301	5682600	0.158	0.539	0.486	0.547
UQ	11	6292201	6292500	0.611	0.305	0.453	0.274
UQ	11	6708301	6708600	1.463	0.845	0.860	0.695
UQ	11	6840001	6840300	0.211	0.564	0.506	0.497
UQ	11	6952201	6952500	1.010	0.583	0.374	0.684
UQ	11	7026001	7026300	1.544	1.049	0.695	0.933
UQ	11	7026601	7026900	0.745	0.142	0.263	0.608
UQ	11	7702501	7702800	0.639	3.152	1.988	1.573
UQ	11	8146501	8146800	0.814	2.319	2.670	2.510
UQ	11	8196901	8197200	0.692	0.423	0.267	0.411
UQ	11	8216101	8216400	1.149	0.493	0.263	0.533
UQ	11	8216401	8216700	2.439	0.852	0.448	0.704
UQ	11	8291701	8292000	1.513	1.216	1.168	0.938
UQ	11	8488801	8489100	0.477	0.167	0.185	0.081
UQ	11	8580601	8580900	1.752	0.463	0.678	1.055
UQ	11	8825401	8825700	1.037	0.348	0.502	0.598
UQ	11	9502201	9502500	3.694	2.426	1.968	2.033
UQ	11	9546601	9546900	0.134	0.513	0.346	0.274
UQ	11	9861001	9861300	2.815	0.929	1.077	2.170
UQ	11	10008901	10009200	0.400	0.725	0.189	0.583
UQ	11	10198201	10198500	0.611	0.423	0.292	0.223

UQ	11	10220401	10220700	0.134	0.536	0.321	0.279
UQ	11	11433601	11433900	0.481	0.163	0.132	0.117
UQ	11	11761501	11761800	0.795	0.515	0.267	0.335
UQ	11	12374401	12374700	2.609	3.180	3.357	3.575
UQ	11	12742801	12743100	0.718	0.470	0.185	0.329
UQ	11	13007701	13008000	0.847	1.525	1.921	1.491
UQ	11	13017301	13017600	1.413	1.099	0.740	1.080
UQ	11	13078801	13079100	0.636	0.236	0.296	0.198
UQ	11	13086901	13087200	4.391	3.137	3.208	3.163
UQ	11	13101301	13101600	1.245	0.704	0.613	0.725
UQ	11	13278601	13278900	0.773	0.444	0.194	0.365
UQ	11	13404301	13404600	0.929	0.609	0.535	0.695
UQ	11	13906801	13907100	0.636	0.234	0.341	0.162
UQ	11	14068501	14068800	1.595	0.624	0.522	0.603
UQ	11	14512201	14512500	3.275	4.085	2.633	2.763
UQ	11	14647501	14647800	0.744	0.212	0.374	0.553
UQ	11	14940301	14940600	0.584	0.232	0.239	0.335
UQ	11	14940601	14940900	0.828	0.371	0.374	0.391
UQ	11	15075301	15075600	0.161	0.468	0.428	0.360
UQ	11	15787201	15787500	1.194	0.676	0.658	0.634
UQ	11	16287901	16288200	0.721	0.283	0.230	0.162
UQ	11	16317901	16318200	3.581	2.448	2.361	2.292
UQ	11	16496401	16496700	0.903	0.326	0.486	0.446
UQ	11	16648501	16648800	1.115	0.912	0.667	0.776
UQ	11	16687801	16688100	0.821	0.257	0.370	0.375
UQ	11	16721701	16722000	0.105	0.493	0.453	0.248
UQ	11	16818001	16818300	4.696	1.993	2.659	2.883
UQ	11	16876801	16877100	6.086	5.464	6.750	6.932
UQ	11	16889101	16889400	0.477	0.189	0.181	0.086
UQ	11	17314501	17314800	1.757	1.283	1.456	2.023
UQ	11	17535601	17535900	0.160	0.350	0.317	0.527
UQ	11	17620801	17621100	0.373	0.515	0.160	0.167
UQ	11	17730601	17730900	2.181	1.098	1.560	1.435
UQ	11	18084001	18084300	0.240	1.515	0.818	0.974
UQ	11	18121801	18122100	0.611	1.079	1.032	1.348
UQ	11	18161701	18162000	0.962	0.517	0.189	0.466
UQ	11	18230101	18230400	0.718	0.373	0.361	0.218
UQ	11	18557101	18557400	0.240	0.607	0.107	0.502
UQ	11	18840001	18840300	1.091	1.309	1.596	0.659
UQ	11	19129501	19129800	1.599	1.332	0.827	1.105
UQ	11	19169401	19169700	1.594	0.918	1.032	1.060
UQ	11	19532701	19533000	3.692	4.817	4.512	4.791
UQ	11	20025901	20026200	1.326	1.959	0.620	0.461
UQ	11	21161701	21162000	0.374	0.538	0.136	0.167
UQ	11	21332701	21333000	0.477	0.189	0.127	0.117
UQ	11	21444301	21444600	1.463	1.008	1.008	1.085
UQ	11	21465001	21465300	0.692	0.354	0.317	0.441
UQ	11	21874801	21875100	0.503	0.212	0.160	0.142
UQ	11	21969601	21969900	0.534	0.047	0.292	0.304
UQ	11	22026301	22026600	3.536	2.058	2.121	1.796
UQ	11	22026901	22027200	1.863	1.101	0.986	1.080
UQ	11	22056901	22057200	1.170	0.749	1.333	0.776
UQ	11	22079101	22079400	0.928	0.513	0.432	0.243
UQ	11	22218301	22218600	0.907	0.633	0.374	0.329
UQ	11	22310401	22310700	0.532	0.884	1.465	1.044
UQ	11	22375801	22376100	1.274	0.959	0.604	0.852
UQ	11	22618801	22619100	0.531	0.725	0.239	0.248
UQ	11	23326201	23326500	4.048	3.161	2.497	2.697
UQ	11	23736901	23737200	0.452	0.747	0.189	0.162
UQ	11	23883301	23883600	1.516	0.742	1.008	0.837
UQ	11	23919601	23919900	1.036	0.612	0.613	0.547
UQ	11	23986501	23986800	5.286	3.323	3.453	4.005
UQ	11	23986801	23987100	4.360	2.955	2.708	2.717
UQ	11	24185701	24186000	0.450	0.165	0.214	0.056
UQ	11	24248101	24248400	0.984	0.543	0.350	0.441
UQ	11	24285601	24285900	0.477	0.210	0.263	0.279
UQ	11	24535801	24536100	0.373	0.562	0.181	0.137

UQ	11	24959401	24959700	1.274	0.729	0.774	0.679
UQ	11	25077901	25078200	2.236	1.538	1.536	0.588
UQ	11	25139401	25139700	0.527	0.116	0.111	0.137
UQ	11	25235701	25236000	0.531	0.212	0.160	0.284
UQ	11	25329301	25329600	1.199	0.935	0.798	0.832
UQ	11	25418401	25418700	1.994	1.403	1.246	1.172
UQ	11	25784401	25784700	4.204	3.289	3.380	2.769
UQ	11	26044801	26045100	1.142	0.678	0.432	0.583
UQ	11	26190901	26191200	1.407	0.912	0.555	1.100
UQ	11	26540701	26541000	0.878	0.629	0.531	0.603
UQ	11	26596501	26596800	1.166	0.463	0.526	0.619
UQ	11	26739901	26740200	0.505	0.187	0.132	0.167
UQ	11	27226201	27226500	1.250	0.652	0.798	0.553
UQ	11	27513301	27513600	1.247	1.517	2.098	1.683
UQ	11	27648001	27648300	1.484	0.985	0.847	0.913
UQ	11	27808201	27808500	0.507	0.232	0.185	0.112
UQ	11	28642801	28643100	0.878	0.279	0.087	0.213
UQ	11	28885201	28885500	3.289	1.815	2.013	2.018
UQ	11	28885501	28885800	2.149	0.929	1.253	1.156
UQ	11	29184601	29184900	0.239	0.140	0.477	0.167
UQ	11	29340601	29340900	0.215	0.654	0.374	0.416
UQ	11	29804101	29804400	1.674	0.472	0.234	0.821
UQ	11	29910301	29910600	1.676	1.008	1.045	0.958
UQ	11	30560101	30560400	0.450	0.187	0.103	0.193
UQ	11	30665401	30665700	0.611	0.491	0.477	0.472
UQ	11	30811801	30812100	0.558	0.863	0.896	0.938
UQ	11	30947701	30948000	0.532	0.232	0.239	0.360
UQ	11	31160701	31161000	0.160	0.444	0.502	0.421
UQ	11	31396201	31396500	0.213	0.466	0.424	0.112
UQ	11	31488601	31488900	1.170	0.704	0.716	0.907
UQ	11	32083201	32083500	1.036	0.727	0.584	0.614
UQ	11	32161501	32161800	0.477	0.208	0.160	0.218
UQ	11	32775001	32775300	5.754	3.931	3.583	4.040
UQ	11	32893501	32893800	1.118	1.216	0.983	1.354
UQ	11	32952601	32952900	5.135	4.469	4.102	4.452
UQ	11	33082201	33082500	0.108	0.468	0.321	0.614
UQ	11	33137401	33137700	1.813	0.961	0.943	0.796
UQ	11	33660601	33660900	1.460	0.536	1.115	1.004
UQ	11	33728401	33728700	0.134	0.373	0.453	0.436
UQ	11	33938701	33939000	1.571	0.817	1.106	0.684
UQ	11	33996901	33997200	0.160	0.395	0.395	0.472
UQ	11	34627201	34627500	3.005	2.197	1.469	2.286
UQ	11	34849501	34849800	0.931	1.195	0.604	0.502
UQ	11	34957201	34957500	0.850	0.354	0.165	0.243
UQ	11	35223901	35224200	0.213	0.654	0.049	0.081
UQ	11	35580901	35581200	0.132	0.395	0.292	0.329
UQ	11	35703601	35703900	1.649	1.107	1.012	0.852
UQ	11	35954101	35954400	2.528	1.793	1.456	0.506
UQ	11	36050101	36050400	3.163	2.397	1.431	2.196
UQ	11	36244501	36244800	0.613	0.303	0.185	0.441
UQ	11	36329101	36329400	0.184	0.629	0.555	0.441
UQ	11	36604501	36604800	1.170	0.749	0.662	0.938
UQ	11	36607201	36607500	0.666	0.466	0.823	0.882
UQ	11	36767401	36767700	2.495	3.295	3.460	3.291
UQ	11	36775501	36775800	1.599	1.354	1.222	0.938
UQ	11	37415101	37415400	6.249	5.774	6.840	4.664
UQ	11	37686301	37686600	0.292	0.558	0.740	0.558
UQ	11	37776601	37776900	1.039	0.749	0.827	0.355
UQ	11	38241301	38241600	0.692	0.584	0.399	0.466
UQ	11	38504701	38505000	1.831	1.289	0.954	1.181
UQ	11	38755801	38756100	0.720	0.328	0.267	0.294
UQ	11	39248701	39249000	1.699	0.373	3.105	1.293
UQ	11	39637801	39638100	1.942	1.214	1.558	2.535
UQ	11	39663901	39664200	3.428	2.780	2.399	2.499
UQ	11	40135801	40136100	0.240	0.466	0.477	0.385
UQ	11	40306201	40306500	0.531	0.258	0.214	0.137
UQ	11	40313701	40314000	0.639	1.171	1.032	1.253

UQ	11	40341601	40341900	4.350	2.830	2.053	2.524
UQ	11	40347001	40347300	0.615	0.210	0.239	0.279
UQ	11	40500301	40500600	0.213	0.420	0.720	0.547
UQ	11	40854601	40854900	0.505	0.161	0.185	0.203
UQ	11	40906501	40906800	1.249	0.510	0.896	0.634
UQ	11	41119501	41119800	1.647	1.101	1.008	0.968
UQ	11	41128501	41128800	0.450	0.163	0.111	0.051
UQ	11	41667601	41667900	1.063	0.727	0.560	0.421
UQ	11	41739301	41739600	4.052	2.167	1.804	1.815
UQ	11	42020701	42021000	0.503	0.843	0.292	0.715
UQ	11	42025801	42026100	0.106	0.448	0.374	0.441
UQ	11	42042301	42042600	0.529	0.236	0.239	0.112
UQ	11	42312001	42312300	2.866	2.315	1.830	2.367
UQ	11	42313501	42313800	1.434	1.144	1.757	1.724
UQ	11	42680701	42681000	0.876	0.258	0.259	0.294
UQ	11	43134301	43134600	0.479	0.609	0.156	0.248
UQ	11	43342501	43342800	0.613	0.302	0.165	0.228
UQ	11	43928401	43928700	0.481	0.212	0.210	0.248
UQ	11	43982701	43983000	2.020	1.753	1.139	0.806
UQ	11	44179801	44180100	0.613	0.303	0.160	0.340
UQ	11	44265301	44265600	0.932	0.680	0.720	1.161
UQ	11	44351401	44351700	1.758	2.079	1.489	1.471
UQ	11	45017401	45017700	0.240	0.631	0.531	0.547
UQ	11	45040801	45041100	0.160	0.678	0.317	0.421
UQ	11	45381301	45381600	0.557	0.258	0.218	0.248
UQ	11	45439501	45439800	3.371	2.577	1.957	2.119
UQ	11	46007101	46007400	0.558	0.161	0.082	0.126
UQ	11	46603501	46603800	0.481	0.213	0.078	0.137
UQ	11	46916401	46916700	1.092	0.701	0.160	0.852
UQ	11	46940701	46941000	1.674	0.946	0.448	0.968
UQ	11	47088301	47088600	1.541	1.172	1.144	1.125
UQ	11	47089501	47089800	5.285	3.270	2.750	2.692
UQ	11	47194501	47194800	5.018	6.329	6.487	6.464
UQ	11	47254801	47255100	1.753	1.330	1.148	1.024
UQ	11	47282101	47282400	1.403	0.699	0.555	0.720
UQ	11	47749801	47750100	1.249	2.055	2.044	2.134
UQ	11	48198601	48198900	2.076	2.849	2.613	2.707
UQ	11	48786301	48786600	0.239	0.468	0.078	0.360
UQ	11	48822001	48822300	0.105	0.491	0.346	0.299
UQ	11	49020001	49020300	0.773	1.446	1.358	1.501
UQ	11	49023901	49024200	0.666	1.191	0.954	1.131
UQ	11	49030501	49030800	1.305	0.695	0.555	0.452
UQ	11	49210201	49210500	0.532	0.305	0.267	0.081
UQ	11	49535401	49535700	1.595	1.472	1.061	1.019
UQ	11	49774501	49774800	2.949	2.407	3.418	2.378
UQ	11	50006701	50007000	3.484	2.444	2.744	2.677
UQ	11	50037301	50037600	1.568	1.143	0.901	1.845
UQ	11	50149801	50150100	0.665	0.444	0.374	0.446
UQ	11	50190601	50190900	1.121	2.109	1.991	1.987
UQ	11	50223301	50223600	3.088	2.386	3.756	3.878
UQ	11	50440801	50441100	0.558	0.094	0.312	0.142
UQ	11	50488801	50489100	1.218	0.049	0.332	0.263
UQ	11	50668201	50668500	0.689	0.397	0.296	0.299
UQ	11	50808601	50808900	1.621	1.034	1.012	0.933
UQ	11	50954401	50954700	1.946	0.629	0.782	1.323
UQ	11	51142801	51143100	2.450	1.452	0.346	1.363
UQ	11	51249901	51250200	0.824	0.465	0.453	0.335
UQ	11	51352201	51352500	0.345	0.515	0.189	0.137
UQ	11	51758101	51758400	1.837	1.304	1.266	0.837
UQ	11	52051201	52051500	1.539	1.032	1.061	0.893
UQ	11	52500901	52501200	0.720	0.468	0.395	0.527
UQ	11	52504501	52504800	1.245	0.236	0.310	0.446
UQ	11	52597201	52597500	0.902	0.564	0.403	0.522
UQ	11	52674601	52674900	4.139	2.927	3.036	3.012
UQ	11	52679101	52679400	1.065	0.609	0.379	0.538
UQ	11	52689001	52689300	0.907	0.416	0.000	0.375
UQ	11	52803601	52803900	1.518	0.238	0.716	0.391

UQ	11	52803901	52804200	1.329	0.215	0.675	0.405
UQ	11	53557801	53558100	3.509	3.038	2.978	2.971
UQ	11	53821501	53821800	0.902	0.401	0.288	0.492
UQ	11	53821801	53822100	1.007	0.331	0.156	0.284
UQ	11	53914201	53914500	0.452	0.234	0.658	0.025
UQ	11	53951701	53952000	0.213	1.146	1.112	1.451
UQ	11	54057601	54057900	0.560	0.326	0.210	0.385
UQ	11	54263101	54263400	0.292	0.466	0.078	0.081
UQ	11	54302401	54302700	0.132	0.330	0.424	0.466
UQ	11	54404701	54405000	0.531	0.234	0.082	0.132
UQ	11	54521101	54521400	1.116	0.678	0.374	0.648
UQ	11	54711001	54711300	0.799	0.562	0.580	0.391
UQ	11	54729001	54729300	0.613	1.590	1.066	0.918
UQ	11	54772501	54772800	3.935	2.553	2.306	2.504
UQ	11	55349401	55349700	0.715	0.283	0.267	0.268
UQ	11	55377601	55377900	5.185	3.081	3.412	3.671
UQ	11	55414501	55414800	0.134	0.352	0.526	0.583
UQ	11	55593301	55593600	0.874	0.399	1.382	0.502
UQ	11	55612801	55613100	5.937	4.637	4.026	4.821
UQ	11	55617601	55617900	1.092	3.358	2.698	3.093
UQ	11	55636501	55636800	1.363	0.701	0.584	0.563
UQ	11	55844401	55844700	0.395	1.519	0.901	0.756
UQ	11	56069101	56069400	2.605	0.773	1.661	1.790
UQ	11	56071801	56072100	1.728	1.144	0.999	1.044
UQ	11	56106001	56106300	2.738	2.036	1.687	1.987
UQ	11	56149201	56149500	0.639	0.300	0.267	0.284
UQ	11	56187601	56187900	1.382	0.432	0.308	0.522
UQ	11	56292601	56292900	1.140	0.727	0.444	0.583
UQ	11	56323501	56323800	1.561	0.588	0.522	0.274
UQ	11	56521201	56521500	0.132	0.493	0.350	0.335
UQ	11	56585101	56585400	3.725	2.100	2.640	2.844
UQ	11	56649301	56649600	6.696	6.215	5.549	8.027
UQ	11	56649601	56649900	2.421	1.866	3.114	2.763
UQ	11	56694001	56694300	1.437	1.049	0.934	0.791
UQ	11	56924401	56924700	2.667	1.519	1.023	1.709
UQ	11	56937601	56937900	2.605	1.845	1.797	1.602
UQ	11	57117901	57118200	0.507	0.326	0.132	0.248
UQ	11	57420901	57421200	1.463	0.541	0.638	0.948
UQ	11	57468901	57469200	0.931	0.513	0.522	0.279
UQ	11	57933601	57933900	0.532	0.279	0.214	0.228
UQ	11	57943201	57943500	0.344	0.727	0.613	0.583
UQ	11	58004401	58004700	1.142	0.935	0.687	0.801
UQ	11	58127401	58127700	1.197	0.684	0.526	0.360
UQ	11	58559401	58559700	5.122	3.175	3.580	3.553
UQ	11	58709401	58709700	8.271	5.984	6.490	7.007
UQ	11	58843201	58843500	0.560	0.303	0.136	0.355
UQ	11	59010301	59010600	0.160	0.538	0.453	0.411
UQ	11	59010601	59010900	0.558	0.187	0.181	0.182
UQ	11	59282401	59282700	0.160	0.305	0.506	0.248
UQ	11	59472901	59473200	1.420	5.233	2.744	3.098
UQ	11	60091201	60091500	2.104	1.167	1.650	1.881
UQ	11	60129001	60129300	1.625	1.118	0.925	1.151
UQ	11	60152401	60152700	1.702	1.776	1.460	2.018
UQ	11	60245401	60245700	11.031	9.481	8.374	9.563
UQ	11	60339601	60339900	0.266	0.560	0.609	0.573
UQ	11	60539701	60540000	5.627	4.091	3.241	3.829
UQ	11	60549001	60549300	1.887	1.094	0.593	0.797
UQ	11	60588301	60588600	4.093	1.650	0.682	2.089
UQ	11	60627601	60627900	2.738	1.826	3.032	2.470
UQ	11	60637501	60637800	2.287	1.188	0.317	0.583
UQ	11	60842701	60843000	0.689	0.279	0.272	0.360
UQ	11	61063801	61064100	9.971	6.436	7.317	6.468
UQ	11	61171501	61171800	7.974	5.021	5.879	5.799
UQ	11	61341001	61341300	0.929	0.541	0.350	0.553
UQ	11	61525201	61525500	0.613	0.330	0.210	0.228
UQ	11	61573201	61573500	3.454	2.502	2.484	2.961
UQ	11	61926301	61926600	0.479	0.772	0.136	0.284

UQ	11	62214901	62215200	0.398	0.867	0.720	0.700
UQ	12	184801	185100	0.852	0.421	0.609	0.522
UQ	12	354301	354600	2.370	1.472	1.665	1.293
UQ	12	354601	354900	2.178	1.405	1.572	1.034
UQ	12	413401	413700	0.932	0.584	0.399	0.527
UQ	12	535501	535800	0.321	1.054	0.687	0.765
UQ	12	665701	666000	2.925	2.575	2.053	2.378
UQ	12	754501	754800	2.322	3.154	1.164	1.526
UQ	12	811801	812100	0.081	0.468	0.370	0.522
UQ	12	852301	852600	0.105	0.744	0.555	0.608
UQ	12	1078801	1079100	0.531	0.159	0.214	0.106
UQ	12	1172701	1173000	4.951	0.941	1.148	1.218
UQ	12	1239301	1239600	0.982	0.466	0.312	0.345
UQ	12	1265701	1266000	1.571	2.034	2.214	2.520
UQ	12	1408201	1408500	1.412	2.175	2.435	2.332
UQ	12	2067901	2068200	1.410	1.054	0.580	1.039
UQ	12	2186401	2186700	2.159	1.015	0.945	0.218
UQ	12	2631301	2631600	0.611	0.491	0.263	0.360
UQ	12	2654101	2654400	3.167	2.178	2.325	2.367
UQ	12	2654701	2655000	0.450	1.126	0.720	0.715
UQ	12	2660401	2660700	2.394	1.354	1.329	1.622
UQ	12	2812501	2812800	1.384	2.111	2.263	2.033
UQ	12	2940901	2941200	1.116	0.727	1.275	0.907
UQ	12	3033601	3033900	0.455	0.985	0.963	0.882
UQ	12	3035701	3036000	0.053	0.493	0.515	0.547
UQ	12	3036001	3036300	0.131	0.702	0.510	0.690
UQ	12	3087601	3087900	1.941	1.098	1.509	1.739
UQ	12	3528001	3528300	0.532	0.140	0.370	0.360
UQ	12	3606601	3606900	3.330	1.832	1.213	1.811
UQ	12	4384801	4385100	0.692	0.234	0.403	0.213
UQ	12	4564201	4564500	0.507	0.212	0.107	0.193
UQ	12	4646101	4646400	2.981	1.611	1.699	2.621
UQ	12	4646401	4646700	3.083	1.676	1.850	2.443
UQ	12	4672201	4672500	0.637	0.210	0.205	0.335
UQ	12	4869901	4870200	0.477	0.139	0.243	0.198
UQ	12	5272801	5273100	0.955	0.397	0.448	0.563
UQ	12	5484001	5484300	0.211	0.562	0.374	0.391
UQ	12	5614201	5614500	0.263	1.099	0.963	0.644
UQ	12	6157201	6157500	0.503	0.047	0.078	0.112
UQ	12	6701101	6701400	0.292	0.116	0.424	0.634
UQ	12	7958401	7958700	0.532	0.213	0.214	0.279
UQ	12	9455401	9455700	0.558	0.118	0.152	0.187
UQ	12	9646201	9646500	0.266	0.905	0.876	0.928
UQ	12	10504201	10504500	0.853	0.302	0.444	0.142
UQ	12	12324901	12325200	2.389	1.427	1.900	1.440
UQ	12	12997801	12998100	0.503	0.257	0.156	0.243
UQ	12	13223401	13223700	0.450	0.116	0.103	0.025
UQ	12	14034301	14034600	0.560	0.981	0.370	0.852
UQ	12	14123701	14124000	1.541	1.028	1.304	1.044
UQ	12	17236801	17237100	0.505	0.281	0.210	0.167
UQ	12	17355601	17355900	0.744	0.375	0.296	0.360
UQ	12	18153901	18154200	1.061	1.262	0.852	1.435
UQ	12	18666301	18666600	0.797	1.098	0.468	1.192
UQ	12	19111801	19112100	0.081	0.513	0.292	0.218
UQ	12	19142101	19142400	0.850	0.583	0.453	0.416
UQ	12	19197601	19197900	0.532	0.348	0.049	0.279
UQ	12	19536301	19536600	0.821	1.706	1.415	1.242
UQ	12	20356801	20357100	1.836	1.054	1.048	0.756
UQ	12	20808001	20808300	0.079	0.494	0.609	0.329
UQ	12	20988901	20989200	0.160	0.328	0.535	0.355
UQ	12	21290401	21290700	1.116	0.702	0.604	0.664
UQ	12	22038301	22038600	0.560	1.032	0.823	0.938
UQ	12	22460101	22460400	1.676	1.139	1.110	0.994
UQ	12	23205901	23206200	1.087	0.723	0.695	0.745
UQ	12	24107101	24107400	1.384	0.373	0.760	0.796
UQ	12	24311401	24311700	0.529	0.094	0.132	0.198
UQ	12	24740701	24741000	1.166	0.770	0.745	0.664

UQ	12	24780601	24780900	0.321	1.558	1.692	1.212
UQ	12	24780901	24781200	0.716	2.145	2.550	2.606
UQ	12	24803701	24804000	6.653	4.624	4.109	4.513
UQ	12	25028101	25028400	0.690	0.258	0.218	0.279
UQ	12	25037701	25038000	1.568	0.824	0.488	0.907
UQ	12	25283701	25284000	0.319	0.794	0.720	0.446
UQ	12	25325701	25326000	0.158	0.350	0.506	0.360
UQ	12	25731901	25732200	0.158	0.581	0.555	0.558
UQ	12	25923601	25923900	0.189	0.444	0.687	0.664
UQ	12	26055301	26055600	0.610	0.328	0.317	0.117
UQ	12	26055601	26055900	0.503	0.328	0.346	0.167
UQ	12	27027301	27027600	1.786	0.654	1.057	1.253
UQ	12	27066001	27066300	0.690	0.331	0.292	0.340
UQ	12	27087601	27087900	0.878	0.539	1.226	1.278
UQ	12	27280201	27280500	0.477	0.165	0.214	0.254
UQ	12	27393001	27393300	0.665	0.914	0.403	0.472
UQ	12	27743101	27743400	0.589	0.210	0.132	0.173
UQ	12	27811201	27811500	1.807	1.422	1.460	1.496
UQ	12	27841201	27841500	0.558	0.348	0.296	0.304
UQ	12	27844801	27845100	0.689	0.257	0.263	0.416
UQ	12	28076101	28076400	1.460	0.792	0.752	0.740
UQ	12	28416001	28416300	0.718	0.375	0.535	0.522
UQ	12	28770901	28771200	2.155	1.686	1.465	1.886
UQ	12	29372101	29372400	0.718	0.165	0.346	0.279
UQ	12	29401501	29401800	0.106	0.444	0.370	0.324
UQ	12	29507101	29507400	1.489	1.126	0.575	0.933
UQ	12	29856001	29856300	0.823	0.421	0.296	0.431
UQ	12	29928901	29929200	0.532	0.399	0.263	0.329
UQ	12	30276001	30276300	1.913	3.274	2.180	1.111
UQ	12	30565501	30565800	1.250	0.401	0.544	0.639
UQ	12	30640801	30641100	1.463	0.749	0.945	1.080
UQ	12	30689101	30689400	2.887	1.749	1.139	1.937
UQ	12	32080801	32081100	0.213	0.656	0.473	0.492
UQ	12	32511001	32511300	0.477	0.139	0.111	0.112
UQ	12	34351801	34352100	0.426	0.513	0.691	0.553
UQ	12	34909501	34909800	0.081	0.305	0.506	0.193
UQ	12	35295601	35295900	1.228	0.399	0.671	0.628
UQ	12	35654701	35655000	1.803	4.038	3.707	2.819
UQ	12	36543301	36543600	1.120	0.208	0.275	0.173
UQ	12	37101001	37101300	0.477	0.302	0.263	0.664
UQ	12	38083501	38083800	2.578	3.060	1.665	1.871
UQ	12	38307301	38307600	0.958	0.437	0.288	0.142
UQ	12	38342401	38342700	0.799	0.236	0.185	0.350
UQ	12	38342701	38343000	1.757	0.142	0.473	0.552
UQ	12	39329701	39330000	0.507	0.257	0.082	0.274
UQ	12	39474301	39474600	0.292	0.654	0.510	0.943
UQ	12	39718201	39718500	3.643	2.733	2.900	2.976
UQ	12	39970501	39970800	3.131	1.877	1.788	1.998
UQ	12	39981301	39981600	0.532	0.890	0.827	0.720
UQ	12	40077601	40077900	0.453	0.772	0.720	0.933
UQ	12	40647001	40647300	2.263	3.512	3.605	4.182
UQ	12	40721401	40721700	0.132	0.444	0.267	0.248
UQ	12	41009701	41010000	1.432	2.551	1.942	2.236
UQ	12	41077201	41077500	1.537	0.824	0.617	0.882
UQ	12	41079001	41079300	1.199	0.583	0.671	0.675
UQ	12	41802301	41802600	3.028	2.195	2.002	2.048
UQ	12	41826601	41826900	0.560	0.255	0.399	0.745
UQ	12	41861701	41862000	1.597	0.708	0.477	0.805
UQ	12	42054001	42054300	0.132	0.448	0.395	0.472
UQ	12	42436801	42437100	1.144	0.446	0.794	0.263
UQ	12	42496801	42497100	1.247	0.543	0.622	0.644
UQ	12	42820501	42820800	1.145	0.867	0.633	0.913
UQ	12	42934501	42934800	0.931	0.303	0.481	0.614
UQ	12	43313101	43313400	0.665	0.796	0.506	0.279
UQ	12	43486501	43486800	2.208	3.094	1.489	2.860
UQ	12	43491301	43491600	0.294	0.607	0.477	0.441
UQ	12	43823701	43824000	0.986	0.654	0.716	0.771

UQ	12	44046601	44046900	0.824	0.631	0.473	0.639
UQ	12	44478001	44478300	3.818	2.165	1.930	2.129
UQ	12	44955301	44955600	5.080	5.422	3.961	4.214
UQ	12	45318601	45318900	0.637	0.094	0.218	0.203
UQ	12	46244101	46244400	4.118	2.716	2.637	2.762
UQ	12	46758301	46758600	0.026	0.371	0.424	0.472
UQ	12	46875901	46876200	0.481	0.189	0.239	0.223
UQ	12	46914001	46914300	1.594	1.127	1.115	0.634
UQ	12	47117401	47117700	0.532	0.210	0.395	0.329
UQ	12	47124001	47124300	0.824	0.983	1.324	1.217
UQ	12	47224801	47225100	0.958	0.303	0.798	0.796
UQ	12	47403301	47403600	2.602	2.128	1.893	2.028
UQ	12	47492101	47492400	1.595	0.964	1.086	0.923
UQ	12	47511301	47511600	0.931	0.420	0.334	0.304
UQ	12	47804401	47804700	0.532	0.747	0.691	0.806
UQ	12	47847001	47847300	0.610	0.397	0.395	0.329
UQ	12	47856301	47856600	2.099	1.755	1.222	1.348
UQ	12	47856901	47857200	1.518	0.981	0.831	1.039
UQ	12	47953501	47953800	1.091	0.446	0.502	0.563
UQ	12	48059101	48059400	0.716	0.491	0.502	0.391
UQ	12	48176401	48176700	0.824	0.725	0.370	0.578
UQ	12	48217201	48217500	2.294	1.212	1.320	0.953
UQ	12	48217501	48217800	1.142	0.702	0.856	0.684
UQ	12	48223501	48223800	1.305	0.538	0.769	0.056
UQ	12	48266401	48266700	0.477	0.165	0.214	0.187
UQ	12	48283201	48283500	0.106	0.652	1.177	0.542
UQ	12	48289801	48290100	1.329	0.603	0.638	0.675
UQ	12	48397201	48397500	0.560	0.421	0.424	0.193
UQ	12	48451501	48451800	2.950	1.717	2.267	0.846
UQ	12	48672601	48672900	0.666	1.905	1.427	1.440
UQ	12	49465801	49466100	0.876	0.466	0.588	0.588
UQ	12	49467901	49468200	2.204	0.685	0.930	1.592
UQ	12	49559701	49560000	0.532	0.281	0.074	0.193
UQ	12	49564801	49565100	3.318	2.407	1.565	2.266
UQ	12	49651201	49651500	0.690	0.215	0.205	0.132
UQ	12	49708501	49708800	0.715	0.281	0.321	0.294
UQ	12	50272801	50273100	1.113	0.676	0.716	0.608
UQ	12	50431801	50432100	0.423	0.116	0.132	0.137
UQ	12	50484001	50484300	0.345	0.702	0.453	0.776
UQ	12	51127501	51127800	0.507	0.305	0.156	0.162
UQ	12	51535501	51535800	0.426	0.094	0.189	0.710
UQ	12	51780001	51780300	0.505	0.142	0.272	0.248
UQ	12	51841501	51841800	1.915	1.122	1.567	2.165
UQ	12	51933901	51934200	0.079	0.232	0.321	0.608
UQ	12	52041301	52041600	0.557	0.210	0.263	0.198
UQ	12	52804801	52805100	0.347	1.221	1.197	0.877
UQ	12	53040301	53040600	0.507	0.144	0.185	0.193
UQ	12	53117101	53117400	1.303	1.824	1.411	1.658
UQ	12	53513701	53514000	0.584	0.168	0.087	0.081
UQ	12	54570601	54570900	7.679	6.291	8.589	9.025
UQ	12	54814501	54814800	1.671	0.753	0.938	0.497
UQ	12	54814801	54815100	1.410	0.871	0.852	0.492
UQ	12	56495701	56496000	0.534	0.185	0.185	0.187
UQ	12	56637601	56637900	0.637	0.397	0.876	0.324
UQ	12	56648101	56648400	0.905	0.513	0.687	0.279
UQ	12	57270301	57270600	0.850	0.609	0.531	0.578
UQ	12	57365701	57366000	0.026	0.257	0.502	0.137
UQ	12	57527101	57527400	1.034	0.375	0.481	0.335
UQ	12	57991201	57991500	3.268	3.551	2.706	2.794
UQ	12	58141501	58141800	0.663	0.212	0.317	0.411
UQ	12	58414201	58414500	0.723	1.219	1.703	1.460
UQ	12	58844401	58844700	1.121	0.466	0.379	0.614
UQ	12	59413801	59414100	0.503	0.747	0.292	0.173
UQ	12	59446801	59447100	1.120	0.845	0.477	0.664
UQ	12	59595001	59595300	0.692	0.376	0.296	0.167
UQ	12	59765701	59766000	0.586	0.747	0.448	0.416
UQ	12	60105001	60105300	2.205	2.875	1.563	1.593

UQ	12	61031701	61032000	1.494	0.399	0.649	0.527
UQ	12	61152601	61152900	0.957	0.539	0.506	0.502
UQ	12	61297801	61298100	1.039	0.427	0.379	0.344
UQ	12	61763401	61763700	0.455	0.142	0.160	0.086
UQ	12	61929301	61929600	0.663	0.376	0.350	0.274
UQ	12	62342701	62343000	0.531	0.281	0.317	0.416
UQ	12	62354101	62354400	0.745	0.122	0.127	0.324
UQ	12	62633701	62634000	0.503	0.236	0.185	0.137
UQ	12	62778301	62778600	2.772	1.425	1.094	1.637
UQ	12	62783101	62783400	1.247	0.000	0.049	0.000
UQ	12	62783401	62783700	0.778	0.000	0.000	0.000
UQ	12	63164101	63164400	0.213	0.611	0.448	0.695
UQ	12	63304501	63304800	0.690	1.124	0.954	0.882
UQ	12	64012501	64012800	0.850	0.678	0.424	0.659
UQ	12	64127701	64128000	0.931	0.609	0.522	0.472
UQ	12	64186501	64186800	0.237	0.657	1.086	1.146
UQ	12	66530101	66530400	1.086	0.631	0.350	0.628
UQ	12	66772501	66772800	0.532	0.167	0.054	0.223
UQ	12	66772801	66773100	0.663	0.234	0.147	0.162
UQ	12	66969301	66969600	0.503	0.047	0.107	0.218
UQ	12	67176001	67176300	1.137	0.541	0.243	0.497
UQ	12	67317601	67317900	0.371	0.656	0.633	0.639
UQ	12	67329901	67330200	2.768	2.133	2.171	1.937
UQ	12	67330201	67330500	1.037	0.305	0.160	0.243
UQ	12	67496101	67496400	0.797	0.354	0.424	0.396
UQ	12	67708201	67708500	0.345	0.674	0.711	0.664
UQ	12	67778701	67779000	0.558	0.279	0.234	0.299
UQ	12	68183701	68184000	0.560	0.798	0.160	0.309
UQ	12	68330101	68330400	0.398	0.257	0.560	0.162
UQ	12	68732401	68732700	0.477	0.232	0.185	0.198
UQ	12	68780401	68780700	3.162	2.556	1.966	2.063
UQ	12	68817301	68817600	0.720	0.539	0.321	0.360
UQ	12	69121801	69122100	0.316	0.682	0.687	0.690
UQ	12	69299701	69300000	6.487	5.484	4.097	5.440
UQ	12	69896401	69896700	1.786	0.805	0.687	0.781
UQ	12	70051201	70051500	1.894	0.682	0.691	0.827
UQ	12	70051501	70051800	2.497	1.590	1.514	1.440
UQ	12	70078501	70078800	1.016	3.694	2.788	3.264
UQ	12	70219201	70219500	0.587	0.118	0.214	0.126
UQ	12	70351501	70351800	0.929	0.449	0.531	0.391
UQ	12	70438201	70438500	2.181	1.489	1.473	1.415
UQ	12	70945201	70945500	0.532	0.326	0.317	0.335
UQ	12	71119801	71120100	1.513	0.236	1.037	0.877
UQ	12	71625001	71625300	2.155	0.300	0.896	1.532
UQ	12	71630101	71630400	0.929	1.124	0.555	0.497
UQ	12	71688301	71688600	1.457	0.485	0.682	0.542
UQ	12	71738101	71738400	1.462	1.871	1.790	1.993
UQ	12	71776801	71777100	0.106	0.491	0.395	0.421
UQ	12	71804101	71804400	3.430	2.124	2.111	2.260
UQ	12	71925601	71925900	2.042	1.131	0.724	0.907
UQ	12	72042901	72043200	0.931	0.416	0.370	0.461
UQ	12	72051901	72052200	0.665	0.210	0.136	0.142
UQ	12	72563401	72563700	0.266	0.444	0.424	0.472
UQ	12	73039801	73040100	0.215	0.444	0.580	0.634
UQ	12	73096201	73096500	3.965	3.478	2.604	3.042
UQ	12	73175101	73175400	0.349	1.289	0.765	0.913
UQ	12	73197601	73197900	3.457	2.714	2.670	2.911
UQ	12	73311301	73311600	1.460	0.701	0.571	0.700
UQ	12	73678201	73678500	2.367	1.257	1.181	1.145
UQ	12	73798801	73799100	0.479	1.169	0.156	0.720
UQ	12	75120901	75121200	1.702	2.577	2.530	2.191
UQ	12	75144301	75144600	5.863	4.392	3.551	3.615
UQ	12	75234601	75234900	6.699	4.915	5.089	4.675
UQ	12	75306601	75306900	0.160	0.515	0.399	0.223
UQ	12	75640801	75641100	2.389	1.654	1.427	1.586
UQ	12	75676801	75677100	1.199	0.423	0.638	0.416
UQ	12	75793201	75793500	0.531	0.328	0.111	0.112

UQ	12	75832501	75832800	0.268	0.466	0.531	0.441
UQ	12	76394101	76394400	0.531	0.303	0.321	0.243
UQ	12	76505401	76505700	0.240	0.515	0.555	0.547
UQ	12	76750501	76750800	4.770	3.705	2.069	2.727
UQ	12	76935301	76935600	3.325	2.523	2.456	2.839
UQ	12	76975201	76975500	1.218	2.276	3.097	3.458
UQ	12	76999501	76999800	2.257	1.472	1.518	1.410
UQ	12	77215201	77215500	0.855	0.420	0.325	0.411
UQ	12	77301901	77302200	1.465	0.699	0.778	1.161
UQ	12	77331901	77332200	1.300	0.609	0.765	0.821
UQ	12	77340001	77340300	0.081	0.444	0.453	0.243
UQ	12	77413801	77414100	2.404	0.319	0.595	0.203
UQ	12	77462401	77462700	0.531	0.747	0.399	0.715
UQ	12	77566201	77566500	0.371	0.867	0.807	0.776
UQ	12	77689501	77689800	0.026	0.446	0.160	0.223
UQ	12	77800801	77801100	2.657	1.217	1.663	1.526
UQ	12	78013501	78013800	0.670	0.045	0.132	0.243
UQ	12	78019201	78019500	0.718	0.466	0.424	0.502
UQ	12	78120301	78120600	2.179	3.206	3.253	3.265
UQ	12	78144001	78144300	1.783	1.233	1.230	1.075
UQ	12	78226801	78227100	8.980	4.251	4.719	5.589
UQ	12	78361801	78362100	3.325	2.573	2.365	2.382
UQ	12	78369301	78369600	2.978	2.482	2.604	2.626
UQ	12	78820801	78821100	0.505	0.212	0.288	0.274
UQ	13	433201	433500	1.063	0.701	0.588	0.497
UQ	13	721201	721500	2.650	1.111	0.923	0.831
UQ	13	721501	721800	2.734	1.087	0.985	0.968
UQ	13	1025701	1026000	0.532	0.283	0.346	0.248
UQ	13	1583101	1583400	2.516	1.120	1.342	1.339
UQ	13	1975501	1975800	0.079	0.425	0.453	0.497
UQ	13	2968501	2968800	2.044	1.592	1.563	1.192
UQ	13	3194701	3195000	1.170	0.560	0.548	0.547
UQ	13	3377101	3377400	2.260	2.547	1.810	2.130
UQ	13	3468901	3469200	0.799	0.562	0.584	0.466
UQ	13	3484201	3484500	0.507	1.908	1.797	2.079
UQ	13	4303801	4304100	0.584	0.466	0.263	0.081
UQ	13	5329201	5329500	0.718	1.588	1.090	0.801
UQ	13	5338801	5339100	0.081	0.682	1.099	0.968
UQ	13	5339101	5339400	0.136	0.682	1.099	1.050
UQ	13	5406601	5406900	0.957	0.517	0.502	0.547
UQ	13	5990701	5991000	0.690	1.289	0.267	0.446
UQ	13	6188701	6189000	1.142	0.631	0.544	0.421
UQ	13	6323101	6323400	1.482	0.657	0.653	0.801
UQ	13	6552001	6552300	0.505	0.397	0.132	0.416
UQ	13	6569401	6569700	0.266	0.725	0.802	0.573
UQ	13	7327501	7327800	1.516	0.940	2.028	1.070
UQ	13	7391401	7391700	0.507	0.350	0.132	0.360
UQ	13	7411201	7411500	0.639	0.326	0.132	0.198
UQ	13	7819801	7820100	0.108	0.631	0.321	0.309
UQ	13	7982401	7982700	1.491	0.912	1.045	0.963
UQ	13	8057701	8058000	1.056	3.906	3.961	3.002
UQ	13	8154001	8154300	1.010	0.584	1.217	0.411
UQ	13	8804101	8804400	0.481	0.024	0.210	0.198
UQ	13	8967901	8968200	0.447	1.056	1.592	1.404
UQ	13	9026101	9026400	0.560	0.234	0.312	0.137
UQ	13	9121201	9121500	0.531	0.350	0.185	0.385
UQ	13	9240001	9240300	0.586	0.701	0.424	0.329
UQ	13	9243001	9243300	0.666	0.258	0.428	0.106
UQ	13	9416701	9417000	0.450	0.163	0.753	0.112
UQ	13	9539701	9540000	0.137	0.817	0.588	1.111
UQ	13	9561601	9561900	1.190	0.348	0.526	0.497
UQ	13	10074001	10074300	1.412	0.820	0.905	0.452
UQ	13	10210501	10210800	0.158	0.328	0.370	0.553
UQ	13	10509001	10509300	4.364	3.362	3.168	2.601
UQ	13	10528201	10528500	1.041	0.656	0.584	0.659
UQ	13	10575001	10575300	5.176	3.568	3.614	3.838
UQ	13	10616701	10617000	1.941	1.453	1.538	1.602

UQ	13	10625701	10626000	6.532	5.095	4.845	4.163
UQ	13	10778401	10778700	0.345	0.605	0.185	0.634
UQ	13	11168101	11168400	1.728	0.938	0.814	0.781
UQ	13	11195401	11195700	0.187	0.491	0.502	0.329
UQ	13	11385601	11385900	4.554	3.100	2.299	2.784
UQ	13	11594701	11595000	4.177	3.053	3.139	3.397
UQ	13	13898401	13898700	3.033	2.405	1.897	2.545
UQ	13	14576101	14576400	0.637	0.350	0.395	0.385
UQ	13	14750401	14750700	0.507	0.350	0.346	0.360
UQ	13	14828101	14828400	0.531	0.189	0.160	0.203
UQ	13	14828401	14828700	0.932	0.356	0.370	0.452
UQ	13	14865301	14865600	2.341	1.240	1.522	1.425
UQ	13	15333001	15333300	1.407	0.305	0.642	0.294
UQ	13	15855901	15856200	1.357	1.073	1.061	0.887
UQ	13	15864001	15864300	0.586	1.358	0.867	0.943
UQ	13	16355401	16355700	0.187	0.588	0.526	0.603
UQ	13	16488601	16488900	0.984	1.450	1.197	1.445
UQ	13	17420101	17420400	0.984	0.704	0.564	0.639
UQ	13	17476201	17476500	0.505	0.208	0.165	0.248
UQ	13	17480401	17480700	2.983	4.025	3.504	4.264
UQ	13	17597101	17597400	0.773	1.053	1.168	0.446
UQ	13	17701501	17701800	0.852	0.515	0.506	0.547
UQ	13	18141001	18141300	0.716	0.423	0.954	0.385
UQ	13	18192301	18192600	0.929	0.702	0.667	0.690
UQ	13	18237001	18237300	0.477	0.187	0.156	0.147
UQ	13	18292801	18293100	0.805	1.519	1.529	1.552
UQ	13	18495901	18496200	0.558	0.139	0.317	0.243
UQ	13	18838801	18839100	5.235	4.038	4.245	4.457
UQ	13	18867001	18867300	0.266	0.749	0.539	0.527
UQ	13	21067501	21067800	0.797	0.330	0.325	0.335
UQ	13	21486301	21486600	0.611	0.277	0.127	0.173
UQ	13	21863701	21864000	0.450	0.094	0.098	0.056
UQ	13	22824001	22824300	0.134	0.348	0.321	0.608
UQ	13	23184301	23184600	0.265	0.491	0.531	0.416
UQ	13	23721301	23721600	0.905	0.463	0.424	0.477
UQ	13	23758501	23758800	0.852	0.654	0.424	0.421
UQ	13	24142501	24142800	0.797	0.446	0.399	0.431
UQ	13	24398401	24398700	1.539	2.362	1.830	1.987
UQ	13	25227601	25227900	1.520	1.054	0.526	0.979
UQ	13	25369201	25369500	2.159	0.991	0.901	1.343
UQ	13	25389601	25389900	0.929	0.286	0.341	0.289
UQ	13	26098201	26098500	0.450	0.140	0.214	0.137
UQ	13	26656201	26656500	0.610	0.255	0.288	0.329
UQ	13	27042601	27042900	1.458	1.053	0.876	0.943
UQ	13	27065101	27065400	0.508	0.165	0.239	0.167
UQ	13	27171601	27171900	0.720	0.936	0.901	0.994
UQ	13	27172801	27173100	3.480	2.107	2.296	2.063
UQ	13	27230401	27230700	2.155	1.502	1.284	1.328
UQ	13	27818101	27818400	0.982	0.470	0.535	0.639
UQ	13	28054501	28054800	0.507	0.234	0.132	0.243
UQ	13	29007001	29007300	0.106	0.489	0.486	0.492
UQ	13	29336101	29336400	0.848	0.491	0.288	0.436
UQ	13	29525101	29525400	1.436	0.661	0.588	0.943
UQ	13	30017101	30017400	0.103	0.539	0.584	0.806
UQ	13	30244801	30245100	0.586	0.073	0.156	0.279
UQ	13	30258901	30259200	0.555	0.142	0.132	0.187
UQ	13	30924001	30924300	0.532	0.236	0.000	0.137
UQ	13	31232701	31233000	1.010	1.708	1.373	1.572
UQ	13	31270801	31271100	0.266	0.539	0.506	0.578
UQ	13	31653901	31654200	0.477	0.140	0.136	0.254
UQ	13	31921801	31922100	0.663	1.148	1.148	0.299
UQ	13	31959901	31960200	0.265	0.629	0.930	0.558
UQ	13	32118601	32118900	0.265	0.491	0.025	0.416
UQ	13	32550901	32551200	1.432	2.178	2.214	2.139
UQ	13	32907001	32907300	0.745	0.936	0.506	0.634
UQ	13	33033901	33034200	0.850	0.279	0.453	0.324
UQ	13	33234601	33234900	1.573	0.684	0.506	0.486

UQ	13	33510901	33511200	0.692	0.470	0.210	0.279
UQ	13	33567301	33567600	6.622	3.776	3.627	5.031
UQ	13	33903901	33904200	0.053	0.212	0.477	0.365
UQ	13	33999601	33999900	0.769	0.468	0.428	1.060
UQ	13	34034401	34034700	0.534	0.303	0.263	0.274
UQ	13	34133401	34133700	2.739	2.504	1.908	2.515
UQ	13	34380301	34380600	1.784	2.341	1.358	1.496
UQ	13	34452001	34452300	0.106	0.257	0.613	0.309
UQ	13	34457101	34457400	0.213	0.489	0.453	0.502
UQ	13	34457401	34457700	0.184	0.468	0.481	0.446
UQ	13	34981201	34981500	0.106	0.536	0.453	0.355
UQ	13	37070701	37071000	0.774	0.258	0.312	0.198
UQ	13	37102501	37102800	1.307	0.331	0.337	0.376
UQ	13	37102801	37103100	0.908	0.238	0.152	0.162
UQ	13	37373401	37373700	0.132	1.006	0.403	0.583
UQ	13	37504501	37504800	1.462	2.221	1.879	2.130
UQ	13	37708201	37708500	0.687	0.212	0.132	0.187
UQ	13	37796701	37797000	0.455	0.092	0.078	0.051
UQ	13	37926901	37927200	1.041	0.448	0.448	0.370
UQ	13	37985101	37985400	1.357	0.294	0.325	0.288
UQ	13	38356801	38357100	0.640	0.395	0.267	0.223
UQ	13	38411401	38411700	2.767	2.180	2.015	2.246
UQ	13	38494501	38494800	0.668	0.258	0.267	0.248
UQ	13	38498101	38498400	1.015	0.654	0.346	0.193
UQ	13	38714401	38714700	0.720	0.420	0.259	0.299
UQ	13	38894101	38894400	1.036	0.745	0.774	1.323
UQ	13	39031501	39031800	2.554	2.221	1.843	1.937
UQ	13	39396301	39396600	0.026	0.421	0.156	0.801
UQ	13	39512401	39512700	0.718	0.328	0.560	0.335
UQ	13	39692101	39692400	0.292	0.446	0.132	0.081
UQ	13	39738601	39738900	1.108	0.184	0.267	0.101
UQ	13	39744301	39744600	1.382	0.401	0.189	0.198
UQ	13	39745501	39745800	0.879	0.285	0.395	0.309
UQ	13	39753601	39753900	0.561	0.094	0.082	0.051
UQ	13	39895801	39896100	1.036	1.238	0.448	0.614
UQ	13	39934801	39935100	1.199	0.656	0.691	0.988
UQ	13	39988801	39989100	0.715	0.257	0.374	0.248
UQ	13	40568701	40569000	1.597	0.817	0.724	0.862
UQ	13	40853701	40854000	0.611	1.051	0.769	0.329
UQ	13	40856401	40856700	0.745	0.446	0.560	0.162
UQ	13	40898101	40898400	0.452	1.030	0.317	0.664
UQ	13	41160301	41160600	1.549	0.356	0.510	0.796
UQ	13	41511901	41512200	1.089	1.332	0.802	0.806
UQ	13	41589901	41590200	0.426	0.978	0.843	1.029
UQ	13	41619601	41619900	1.199	0.820	0.802	0.553
UQ	13	41710801	41711100	3.668	2.718	2.312	2.896
UQ	13	41933101	41933400	1.889	1.313	1.052	1.262
UQ	13	41986801	41987100	0.637	0.326	0.366	0.360
UQ	13	42714001	42714300	0.718	0.376	0.321	0.527
UQ	13	42889201	42889500	0.505	0.305	0.239	0.279
UQ	13	43120801	43121100	1.970	3.034	2.929	2.884
UQ	13	43121101	43121400	2.702	4.514	4.800	4.698
UQ	13	43483501	43483800	0.213	0.605	0.930	0.644
UQ	13	43580101	43580400	0.586	0.144	0.181	0.248
UQ	13	43797601	43797900	7.648	5.621	5.890	6.414
UQ	13	43877401	43877700	0.503	0.000	0.111	0.137
UQ	13	44245201	44245500	0.800	0.348	0.214	0.553
UQ	13	44478001	44478300	3.909	2.573	2.896	3.737
UQ	13	44568001	44568300	0.482	0.047	0.029	0.112
UQ	13	45470701	45471000	2.149	1.545	1.674	1.627
UQ	13	45534301	45534600	1.570	0.699	1.003	1.141
UQ	13	46643401	46643700	0.347	0.657	0.613	0.076
UQ	13	47182801	47183100	0.160	0.420	0.054	0.081
UQ	13	49293001	49293300	0.582	0.238	0.239	0.223
UQ	13	50043301	50043600	0.505	0.184	0.165	0.223
UQ	13	50292901	50293200	2.770	3.993	3.411	3.910
UQ	13	51180301	51180600	1.254	0.981	0.481	0.882

UQ	13	51210001	51210300	0.505	0.120	0.185	0.051
UQ	13	51327901	51328200	0.477	0.678	0.695	0.690
UQ	13	51438901	51439200	0.211	0.373	0.638	0.553
UQ	13	51854101	51854400	1.115	0.472	0.580	0.583
UQ	13	52112401	52112700	0.319	0.538	0.078	0.198
UQ	13	52155001	52155300	0.824	0.935	0.720	0.522
UQ	13	52517401	52517700	0.745	0.376	0.165	0.304
UQ	13	52636501	52636800	1.563	0.373	0.847	0.756
UQ	13	53159101	53159400	1.091	0.747	0.765	0.771
UQ	13	53211601	53211900	1.384	1.073	1.144	1.044
UQ	13	53820601	53820900	0.931	0.727	0.453	0.715
UQ	13	53859001	53859300	0.294	0.983	0.909	0.588
UQ	13	54165301	54165600	0.452	0.281	0.234	0.664
UQ	13	54585001	54585300	1.066	0.442	0.724	0.365
UQ	13	54683401	54683700	0.477	1.004	0.798	0.776
UQ	13	54747601	54747900	0.106	0.446	0.267	0.274
UQ	13	54776701	54777000	0.265	0.562	0.769	0.533
UQ	13	55089901	55090200	0.505	0.375	0.267	0.198
UQ	13	55130401	55130700	0.639	0.096	0.317	0.335
UQ	13	55825801	55826100	0.740	0.281	0.160	0.248
UQ	13	56033701	56034000	0.960	0.515	0.535	0.628
UQ	13	56077501	56077800	1.168	1.427	1.728	1.359
UQ	13	56389501	56389800	0.477	0.281	0.054	0.198
UQ	13	56638201	56638500	0.611	0.352	0.350	0.385
UQ	13	56857501	56857800	6.059	4.791	4.780	5.369
UQ	13	57589501	57589800	1.116	0.680	0.638	0.628
UQ	13	57611101	57611400	1.862	2.379	2.394	2.459
UQ	13	57731401	57731700	2.604	2.148	2.254	1.901
UQ	13	57862501	57862800	1.118	0.633	0.464	0.578
UQ	13	58114801	58115100	1.037	0.421	0.321	0.882
UQ	13	58228501	58228800	0.134	1.000	2.069	1.257
UQ	13	58228801	58229100	0.053	1.214	2.633	1.886
UQ	13	58266001	58266300	0.852	0.562	1.037	0.527
UQ	13	58551901	58552200	0.720	0.375	0.531	0.497
UQ	13	58815001	58815300	3.089	2.549	2.550	2.652
UQ	13	58929001	58929300	1.066	1.566	0.740	1.404
UQ	13	59425201	59425500	0.689	0.159	0.189	0.304
UQ	13	59441101	59441400	4.544	1.826	5.663	3.655
UQ	13	59542501	59542800	4.256	3.218	3.513	3.519
UQ	13	59576101	59576400	1.197	1.701	1.761	0.735
UQ	13	59963701	59964000	1.595	1.332	1.300	1.795
UQ	13	60091801	60092100	0.557	0.210	0.239	0.137
UQ	13	61045801	61046100	2.662	1.798	1.670	0.385
UQ	13	61098901	61099200	1.886	1.079	0.889	0.872
UQ	13	61283101	61283400	0.637	0.350	0.140	0.279
UQ	13	61553401	61553700	2.076	1.631	1.485	1.298
UQ	13	61734601	61734900	1.068	1.493	1.806	1.496
UQ	13	62239201	62239500	0.611	1.379	0.881	0.893
UQ	13	62527501	62527800	0.637	0.466	0.267	0.446
UQ	13	63458401	63458700	0.187	0.489	0.424	0.492
UQ	13	63845401	63845700	0.898	0.330	0.263	0.243
UQ	13	63868201	63868500	0.481	0.118	0.103	0.076
UQ	13	64454101	64454400	1.223	0.818	0.881	0.725
UQ	13	64520401	64520700	2.533	1.777	1.081	1.673
UQ	13	64737901	64738200	0.663	0.328	0.263	0.213
UQ	13	64753801	64754100	3.890	1.137	2.777	1.471
UQ	13	65005201	65005500	0.531	0.255	0.292	0.304
UQ	13	65330701	65331000	0.477	0.236	0.127	0.056
UQ	13	65593201	65593500	0.106	0.423	0.448	0.441
UQ	13	65793901	65794200	0.477	0.234	0.292	0.106
UQ	13	65855101	65855400	0.081	0.348	0.317	0.497
UQ	13	65887801	65888100	0.532	0.397	0.239	0.112
UQ	13	65891701	65892000	1.697	0.629	0.658	0.791
UQ	13	65989201	65989500	1.568	0.918	0.881	0.675
UQ	13	65989501	65989800	1.437	0.753	0.909	0.796
UQ	13	66098701	66099000	0.826	0.605	0.292	0.355
UQ	13	66672601	66672900	5.451	3.772	4.035	3.842

UQ	13	66909001	66909300	0.771	0.560	0.292	0.533
UQ	13	67212301	67212600	1.436	0.888	0.921	0.704
UQ	13	67318801	67319100	0.266	0.515	0.370	0.583
UQ	13	67665901	67666200	1.492	1.026	1.008	0.771
UQ	13	67882501	67882800	0.527	0.118	0.054	0.112
UQ	13	67885201	67885500	0.531	0.326	0.160	0.218
UQ	13	67958401	67958700	3.955	3.154	2.929	2.783
UQ	13	68007601	68007900	0.584	0.210	0.769	0.243
UQ	13	68664601	68664900	0.481	0.845	0.210	0.218
UQ	13	68726401	68726700	0.345	0.609	0.604	0.603
UQ	13	68829901	68830200	1.118	1.311	1.275	0.887
UQ	13	68866201	68866500	3.830	2.517	2.472	2.353
UQ	13	68943601	68943900	0.879	0.470	0.346	0.452
UQ	13	68988901	68989200	0.692	0.212	0.165	0.112
UQ	13	69048901	69049200	1.010	0.588	0.564	0.238
UQ	13	69182101	69182400	1.329	1.590	1.567	1.825
UQ	13	69304801	69305100	2.174	1.633	1.551	0.943
UQ	13	69388201	69388500	1.358	0.727	0.633	0.248
UQ	13	69388501	69388800	1.573	0.805	0.638	0.324
UQ	13	69400501	69400800	1.912	0.744	0.733	0.958
UQ	13	69400801	69401100	1.592	0.626	0.809	0.751
UQ	13	69747001	69747300	1.036	1.878	1.875	1.719
UQ	13	69858001	69858300	1.373	0.348	0.386	0.193
UQ	13	69858301	69858600	0.582	0.213	0.181	0.081
UQ	13	70428901	70429200	1.281	0.678	0.847	0.639
UQ	13	70492501	70492800	1.089	0.521	0.395	0.243
UQ	13	70787401	70787700	2.358	1.249	1.337	1.227
UQ	13	71046601	71046900	0.160	0.373	0.535	0.304
UQ	13	71076601	71076900	0.720	0.305	0.288	0.365
UQ	13	71440201	71440500	0.210	0.609	0.526	0.522
UQ	13	71485501	71485800	2.755	1.554	1.522	1.328
UQ	13	71846701	71847000	1.520	2.221	2.082	2.423
UQ	13	72036601	72036900	0.450	0.303	0.132	0.304
UQ	13	72296101	72296400	4.317	2.916	3.306	3.316
UQ	13	72601501	72601800	1.757	1.217	1.028	1.258
UQ	13	72627901	72628200	0.455	0.165	0.029	0.086
UQ	13	72637501	72637800	0.718	0.631	0.609	0.527
UQ	13	72653101	72653400	0.586	0.257	0.259	0.193
UQ	13	72672901	72673200	0.426	1.172	0.662	0.664
UQ	13	72775501	72775800	0.668	0.352	0.214	0.329
UQ	13	72837301	72837600	0.931	1.075	0.827	1.273
UQ	13	73010401	73010700	3.981	3.062	2.793	2.773
UQ	13	73561801	73562100	0.615	0.234	0.247	0.198
UQ	13	73744201	73744500	0.665	0.257	0.243	0.335
UQ	13	74336101	74336400	2.100	1.264	1.213	1.627
UQ	13	74446201	74446500	2.550	1.455	1.028	1.400
UQ	13	74548501	74548800	0.773	0.140	0.111	0.264
UQ	13	74793001	74793300	1.839	1.126	0.954	1.217
UQ	13	74821201	74821500	0.560	0.140	0.136	0.137
UQ	13	75212701	75213000	0.532	0.185	0.374	0.385
UQ	13	75589801	75590100	2.184	1.103	0.896	1.172
UQ	13	75751801	75752100	7.616	2.079	2.608	5.449
UQ	13	75865201	75865500	1.195	1.474	0.831	0.522
UQ	13	75985201	75985500	0.874	0.281	0.399	0.076
UQ	13	75999301	75999600	0.584	0.185	0.428	0.391
UQ	13	76118101	76118400	1.857	1.236	0.624	1.060
UQ	13	76162801	76163100	1.834	1.358	1.324	1.177
UQ	13	76358701	76359000	3.457	1.967	1.768	2.002
UQ	13	76460701	76461000	5.824	3.816	1.197	1.934
UQ	13	76480501	76480800	1.968	0.818	0.805	0.821
UQ	13	76556401	76556700	0.481	0.257	0.132	0.142
UQ	13	76654201	76654500	1.116	0.841	0.613	0.806
UQ	13	76716901	76717200	0.586	0.206	0.292	0.304
UQ	13	76733401	76733700	1.755	1.077	0.724	1.009
UQ	13	77202001	77202300	2.025	1.103	2.513	1.460
UQ	13	77203501	77203800	0.665	0.444	0.181	0.254
UQ	13	77600101	77600400	1.518	2.105	1.971	1.739

UQ	13	77634601	77634900	1.274	1.075	0.798	0.659
UQ	13	77856001	77856300	6.096	3.643	3.793	2.915
UQ	13	77871601	77871900	0.450	0.232	0.214	0.218
UQ	13	78540901	78541200	1.360	0.886	0.691	0.796
UQ	13	78662401	78662700	1.010	1.568	0.905	1.688
UQ	13	78777601	78777900	2.110	1.172	0.941	1.055
UQ	13	78872401	78872700	0.852	0.279	0.424	0.745
UQ	13	79005601	79005900	1.250	0.957	0.658	0.938
UQ	13	79214401	79214700	1.597	0.729	0.905	0.806
UQ	13	79471801	79472100	1.039	0.583	0.477	0.654
UQ	13	79500301	79500600	0.852	1.659	1.152	1.131
UQ	13	79695601	79695900	0.211	0.560	0.580	0.639
UQ	13	79852201	79852500	0.584	0.373	0.214	0.416
UQ	13	79870501	79870800	0.349	0.749	0.843	0.887
UQ	13	80153101	80153400	2.070	1.334	1.025	1.435
UQ	13	80172601	80172900	0.690	0.328	0.555	0.360
UQ	13	80225701	80226000	1.355	1.101	0.872	1.050
UQ	13	80352601	80352900	0.105	0.491	0.239	0.304
UQ	13	80569501	80569800	2.504	2.060	1.670	1.851
UQ	13	80883001	80883300	2.129	1.403	1.032	1.699
UQ	13	81164101	81164400	3.346	2.684	2.608	2.733
UQ	13	81439501	81439800	9.069	7.205	6.730	6.693
UQ	13	81497401	81497700	1.121	0.442	0.390	0.603
UQ	13	81637501	81637800	6.150	2.510	3.545	3.075
UQ	13	81637801	81638100	4.851	1.860	2.394	2.175
UQ	13	82018201	82018500	5.104	2.716	2.699	3.116
UQ	13	82200001	82200300	4.652	1.562	2.742	3.140
UQ	13	82614601	82614900	0.503	1.053	0.823	0.918
UQ	13	82638601	82638900	0.718	0.238	0.263	0.391
UQ	13	82848301	82848600	2.292	1.433	1.284	1.177
UQ	13	82928401	82928700	0.690	0.399	0.390	0.329
UQ	13	83010601	83010900	0.453	0.985	0.794	1.019
UQ	13	83020201	83020500	0.611	1.053	0.798	0.882
UQ	14	311101	311400	0.958	0.539	0.502	0.553
UQ	14	311401	311700	1.145	0.139	0.250	0.400
UQ	14	534001	534300	1.487	0.227	0.403	0.785
UQ	14	597601	597900	1.515	2.032	2.782	0.882
UQ	14	1056001	1056300	1.410	0.843	1.012	0.563
UQ	14	1131301	1131600	4.472	3.251	3.337	3.524
UQ	14	1439101	1439400	0.847	0.206	0.176	0.198
UQ	14	1439401	1439700	0.586	0.210	0.132	0.137
UQ	14	1584301	1584600	0.587	1.242	1.081	1.131
UQ	14	3204001	3204300	0.873	0.232	0.296	0.268
UQ	14	3320101	3320400	0.215	0.444	0.025	0.360
UQ	14	3981601	3981900	2.183	1.144	1.355	0.887
UQ	14	4201801	4202100	1.778	0.378	0.901	0.861
UQ	14	4204201	4204500	0.584	0.236	0.247	0.218
UQ	14	4570201	4570500	0.186	0.538	0.292	0.335
UQ	14	4577701	4578000	1.857	0.581	0.410	0.714
UQ	14	4619701	4620000	0.687	0.208	0.181	0.162
UQ	14	4734901	4735200	1.036	1.240	1.280	0.771
UQ	14	4780801	4781100	3.747	0.804	1.311	2.252
UQ	14	4832701	4833000	0.532	0.326	0.214	0.254
UQ	14	4962001	4962300	0.853	0.609	0.181	0.385
UQ	14	5082301	5082600	3.107	3.898	4.911	4.736
UQ	14	5137201	5137500	0.850	0.560	0.560	0.497
UQ	14	5207701	5208000	1.594	1.126	1.106	1.100
UQ	14	5253001	5253300	1.092	0.820	0.740	0.502
UQ	14	5376901	5377200	3.330	4.392	2.633	3.849
UQ	14	5531101	5531400	2.604	1.723	1.608	1.805
UQ	14	5639101	5639400	0.987	0.562	0.613	0.578
UQ	14	5880301	5880600	1.005	0.167	0.223	0.315
UQ	14	6067201	6067500	1.274	1.472	0.695	0.913
UQ	14	6109201	6109500	0.532	0.236	0.205	0.086
UQ	14	6110101	6110400	0.932	0.725	0.588	0.441
UQ	14	6121201	6121500	0.774	0.330	0.132	0.365
UQ	14	6471601	6471900	3.596	2.146	1.982	1.557

UQ	14	6503701	6504000	0.610	0.210	0.370	0.223
UQ	14	6801601	6801900	0.958	0.680	0.769	0.751
UQ	14	7041301	7041600	0.532	0.210	0.321	0.299
UQ	14	7386601	7386900	8.216	7.659	7.058	9.339
UQ	14	7494901	7495200	2.068	1.356	1.094	1.146
UQ	14	7613401	7613700	0.878	0.678	0.473	0.634
UQ	14	7871101	7871400	1.968	0.895	0.918	0.751
UQ	14	7871401	7871700	2.071	1.247	1.132	1.029
UQ	14	7997701	7998000	0.773	0.352	0.444	0.299
UQ	14	8016901	8017200	0.666	0.444	0.218	0.167
UQ	14	8380201	8380500	0.292	0.493	0.584	0.086
UQ	14	8434801	8435100	0.692	0.418	0.428	0.299
UQ	14	8825701	8826000	0.189	0.513	0.424	0.472
UQ	14	8890501	8890800	3.559	2.162	1.748	1.405
UQ	14	9082501	9082800	1.625	1.000	1.037	1.040
UQ	14	9185701	9186000	0.773	0.352	0.259	0.466
UQ	14	9435001	9435300	0.186	0.373	0.399	0.466
UQ	14	9477301	9477600	1.623	1.214	1.295	1.181
UQ	14	9520501	9520800	0.634	0.071	0.058	0.051
UQ	14	9526501	9526800	4.649	3.765	3.808	3.924
UQ	14	9535201	9535500	4.115	3.458	3.353	2.976
UQ	14	9988801	9989100	0.610	0.258	0.267	0.218
UQ	14	10141201	10141500	0.534	0.236	0.185	0.162
UQ	14	10198201	10198500	0.584	0.399	0.239	0.335
UQ	14	10329301	10329600	0.503	4.388	2.335	4.447
UQ	14	10383001	10383300	7.096	6.086	4.517	5.516
UQ	14	10425601	10425900	0.773	0.515	0.267	0.335
UQ	14	10434601	10434900	0.557	0.213	0.132	0.254
UQ	14	10567201	10567500	1.961	1.118	0.789	0.776
UQ	14	10932001	10932300	0.747	0.283	0.403	0.248
UQ	14	10933801	10934100	3.249	1.684	2.321	2.130
UQ	14	10977001	10977300	1.089	0.727	0.740	0.776
UQ	14	11205001	11205300	0.268	1.026	0.872	1.724
UQ	14	11251501	11251800	0.586	0.305	0.428	0.274
UQ	14	11318401	11318700	3.220	4.814	4.775	5.216
UQ	14	11623501	11623800	0.560	0.047	0.218	0.106
UQ	14	11637601	11637900	1.683	0.611	0.872	0.907
UQ	14	11643901	11644200	0.666	0.255	0.272	0.360
UQ	14	12012001	12012300	0.903	0.354	0.642	0.527
UQ	14	12106801	12107100	5.003	2.618	3.904	4.179
UQ	14	12156001	12156300	1.011	0.277	0.283	0.553
UQ	14	12283201	12283500	0.558	0.305	0.691	0.446
UQ	14	12676801	12677100	1.807	0.612	2.303	0.391
UQ	14	13180501	13180800	1.173	0.843	0.765	0.690
UQ	14	13379701	13380000	0.826	1.238	0.395	0.436
UQ	14	13657801	13658100	0.986	0.279	0.526	0.669
UQ	14	13859401	13859700	2.555	1.916	2.033	1.465
UQ	14	13937101	13937400	0.529	0.118	0.165	0.137
UQ	14	14324101	14324400	0.479	0.654	0.181	0.274
UQ	14	14339401	14339700	0.213	1.028	0.885	0.527
UQ	14	14369701	14370000	0.481	0.116	0.214	0.162
UQ	14	14382601	14382900	3.739	0.142	0.917	0.794
UQ	14	14382901	14383200	1.699	0.213	0.330	0.557
UQ	14	14627401	14627700	0.532	0.142	0.239	0.203
UQ	14	15293701	15294000	0.423	0.069	0.049	0.051
UQ	14	16066801	16067100	4.028	2.133	1.451	1.981
UQ	14	16200001	16200300	0.692	0.238	0.098	0.142
UQ	14	16220401	16220700	0.718	0.472	1.061	0.441
UQ	14	16237801	16238100	0.211	0.493	0.054	0.000
UQ	14	16666201	16666500	0.319	0.185	0.160	0.497
UQ	14	16792201	16792500	0.187	0.515	0.317	0.385
UQ	14	16792501	16792800	0.799	1.689	1.799	1.602
UQ	14	16911301	16911600	0.187	0.470	0.453	0.497
UQ	14	16924501	16924800	0.239	0.442	0.745	0.416
UQ	14	16932601	16932900	2.208	0.729	1.180	1.516
UQ	14	16932901	16933200	3.746	1.454	1.813	2.058
UQ	14	16934401	16934700	1.412	0.708	0.934	0.527

UQ	14	17156401	17156700	0.400	0.562	0.132	0.578
UQ	14	17422501	17422800	0.477	0.749	0.843	0.771
UQ	14	17433901	17434200	0.876	0.564	0.502	0.558
UQ	14	17605801	17606100	0.531	0.230	0.263	0.218
UQ	14	17786401	17786700	0.426	0.560	0.263	0.279
UQ	14	17912101	17912400	0.532	0.281	0.210	0.162
UQ	14	17977501	17977800	0.584	0.142	0.321	0.360
UQ	14	18186001	18186300	2.802	1.356	1.650	1.861
UQ	14	18304501	18304800	0.795	0.307	0.234	0.350
UQ	14	18401701	18402000	1.568	0.957	1.041	1.065
UQ	14	18470101	18470400	0.850	0.631	0.210	0.664
UQ	14	18547801	18548100	0.534	0.236	0.181	0.162
UQ	14	18742201	18742500	0.481	0.210	0.218	0.193
UQ	14	18816301	18816600	2.899	0.905	1.141	0.573
UQ	14	19386301	19386600	0.640	0.281	0.312	0.248
UQ	14	19386601	19386900	0.558	0.257	0.341	0.193
UQ	14	19919101	19919400	0.507	0.258	0.711	0.304
UQ	14	20611801	20612100	1.834	1.427	1.081	1.004
UQ	14	21381301	21381600	1.539	0.721	1.144	1.181
UQ	14	21388501	21388800	0.560	1.526	1.148	1.192
UQ	14	21759001	21759300	1.676	2.244	0.909	2.119
UQ	14	21938101	21938400	3.004	2.010	1.696	1.085
UQ	14	21938401	21938700	1.010	0.562	0.687	0.294
UQ	14	21970501	21970800	0.587	0.279	0.165	0.086
UQ	14	23209501	23209800	1.007	0.378	0.383	0.279
UQ	14	23821501	23821800	0.716	0.305	0.296	0.416
UQ	14	24306601	24306900	0.665	1.850	1.516	1.820
UQ	14	24321301	24321600	2.504	2.013	1.826	1.962
UQ	14	24330601	24330900	0.505	0.187	0.156	0.309
UQ	14	24473401	24473700	6.356	4.919	4.702	5.080
UQ	14	24496501	24496800	0.452	0.283	0.185	0.634
UQ	14	24611101	24611400	0.027	0.330	0.317	0.776
UQ	14	25147201	25147500	4.654	3.709	3.393	3.570
UQ	14	25255201	25255500	1.387	0.794	0.711	0.867
UQ	14	25257601	25257900	3.931	2.248	2.354	2.169
UQ	14	25935301	25935600	0.878	0.378	0.288	0.578
UQ	14	25945201	25945500	0.771	0.369	0.346	0.380
UQ	14	26740501	26740800	0.613	0.307	0.267	0.162
UQ	14	28221001	28221300	0.266	0.633	0.428	0.527
UQ	14	28538401	28538700	1.276	0.863	0.667	0.832
UQ	14	28538701	28539000	1.590	0.955	0.909	0.837
UQ	14	29522401	29522700	0.481	0.279	0.214	0.112
UQ	14	30631801	30632100	0.718	0.515	0.399	0.279
UQ	14	31550701	31551000	0.505	0.092	0.116	0.081
UQ	14	32096701	32097000	0.560	0.230	0.103	0.025
UQ	14	32145001	32145300	0.826	0.534	0.531	0.243
UQ	14	32288401	32288700	1.996	1.569	1.572	0.816
UQ	14	32384701	32385000	0.426	0.185	0.555	0.279
UQ	14	32579701	32580000	1.965	1.026	1.093	0.927
UQ	14	32924701	32925000	0.448	1.028	1.123	0.913
UQ	14	33069001	33069300	0.294	0.517	0.667	0.553
UQ	14	33102001	33102300	1.008	0.397	0.564	0.578
UQ	14	33159301	33159600	0.507	0.187	0.136	0.106
UQ	14	33181501	33181800	0.690	0.981	0.823	0.497
UQ	14	33333601	33333900	0.663	0.120	0.111	0.051
UQ	14	33333901	33334200	0.587	0.258	0.029	0.051
UQ	14	33441301	33441600	0.639	0.096	0.263	0.411
UQ	14	33707401	33707700	0.718	0.354	0.127	0.223
UQ	14	33769501	33769800	0.744	0.539	0.481	0.441
UQ	14	33941701	33942000	1.302	0.983	0.613	0.988
UQ	14	34165201	34165500	1.408	0.839	0.901	0.751
UQ	14	34194601	34194900	1.382	0.936	0.613	1.009
UQ	14	34194901	34195200	0.984	0.773	0.502	0.771
UQ	14	34537501	34537800	4.711	4.299	3.081	4.000
UQ	14	34762501	34762800	2.450	3.036	1.670	1.840
UQ	14	35688301	35688600	0.852	0.425	0.346	0.431
UQ	14	36024601	36024900	0.265	0.468	0.580	0.472

UQ	14	36213601	36213900	0.903	0.629	0.502	0.527
UQ	14	36242101	36242400	0.560	0.142	0.214	0.051
UQ	14	37229101	37229400	1.437	0.908	1.061	0.710
UQ	14	37259101	37259400	2.073	1.103	1.188	0.801
UQ	14	37259401	37259700	0.773	0.399	0.424	0.228
UQ	14	37364701	37365000	0.853	0.539	0.473	0.299
UQ	14	37437601	37437900	1.654	1.011	0.749	0.979
UQ	14	37482601	37482900	3.381	2.506	1.632	2.211
UQ	14	37537201	37537500	2.926	2.549	1.897	1.744
UQ	14	37662901	37663200	1.405	0.931	0.794	0.614
UQ	14	37663201	37663500	1.276	0.555	0.604	0.497
UQ	14	38436301	38436600	0.768	0.421	0.341	0.355
UQ	14	38452501	38452800	0.398	0.584	0.239	0.304
UQ	14	38947501	38947800	0.740	0.238	0.243	0.187
UQ	14	39410701	39411000	0.505	0.328	0.267	0.355
UQ	14	39438901	39439200	0.848	1.998	1.942	2.022
UQ	14	39932701	39933000	4.801	2.722	3.286	3.053
UQ	14	40026301	40026600	2.071	1.541	1.436	1.424
UQ	14	40064401	40064700	0.345	0.468	0.502	0.553
UQ	14	40078501	40078800	3.083	1.530	1.846	2.322
UQ	14	40271701	40272000	1.199	0.910	0.818	0.913
UQ	14	40432801	40433100	0.931	1.729	1.061	0.720
UQ	14	40440001	40440300	0.347	0.727	0.736	0.837
UQ	14	40457101	40457400	0.878	0.818	0.584	0.801
UQ	14	40731601	40731900	0.984	0.770	0.749	0.527
UQ	14	40956301	40956600	0.639	0.845	0.321	0.223
UQ	14	41078701	41079000	0.213	0.420	0.617	0.441
UQ	14	41571001	41571300	0.661	0.092	0.205	0.112
UQ	14	41978701	41979000	0.531	0.281	0.346	0.248
UQ	14	42073501	42073800	1.089	1.569	0.700	0.385
UQ	14	42166801	42167100	1.750	2.242	1.099	1.105
UQ	14	42297901	42298200	0.239	0.446	0.526	0.527
UQ	14	42554701	42555000	4.099	2.017	1.115	1.764
UQ	14	43235101	43235400	2.279	3.637	4.222	3.787
UQ	14	43427401	43427700	1.171	0.682	0.477	0.497
UQ	14	43527901	43528200	0.850	0.446	0.481	0.375
UQ	14	43960801	43961100	0.181	2.122	1.213	1.464
UQ	14	44056801	44057100	0.450	0.210	0.111	0.193
UQ	14	44149501	44149800	0.582	0.935	0.247	0.243
UQ	14	44230501	44230800	0.481	0.258	0.185	0.031
UQ	14	44427301	44427600	1.621	0.536	1.966	1.906
UQ	14	44719501	44719800	0.850	0.676	0.424	0.335
UQ	14	44883601	44883900	1.674	0.682	0.526	1.014
UQ	14	44936101	44936400	0.294	1.315	0.814	0.943
UQ	14	45302401	45302700	0.510	0.094	0.132	0.056
UQ	14	45561301	45561600	4.814	3.057	4.242	5.735
UQ	14	45639001	45639300	0.637	0.418	0.160	0.081
UQ	14	45810601	45810900	6.735	4.328	4.229	4.091
UQ	14	46306501	46306800	0.400	0.657	0.107	0.162
UQ	14	46501501	46501800	0.240	0.678	0.526	0.801
UQ	14	46672501	46672800	0.400	0.794	0.239	0.243
UQ	14	46918801	46919100	0.613	0.305	0.288	0.223
UQ	14	46941901	46942200	0.532	0.281	0.374	0.664
UQ	14	47147701	47148000	3.517	2.618	1.988	2.084
UQ	14	47411101	47411400	2.500	1.723	1.872	1.805
UQ	14	47451301	47451600	3.965	2.877	4.702	4.593
UQ	14	47508601	47508900	1.412	0.633	0.959	0.695
UQ	14	47563801	47564100	2.418	1.543	1.543	1.714
UQ	14	47636101	47636400	4.956	3.564	2.423	3.859
UQ	14	47636401	47636700	5.602	4.302	2.477	3.986
UQ	14	47645701	47646000	2.863	1.852	0.921	1.252
UQ	14	47795701	47796000	0.453	0.328	0.638	0.218
UQ	14	48837301	48837600	1.008	0.628	0.292	0.664
UQ	14	48954901	48955200	0.982	0.399	1.420	0.538
UQ	14	49013101	49013400	2.500	2.008	3.003	2.165
UQ	14	49063501	49063800	0.400	0.773	0.778	0.725
UQ	14	49294501	49294800	0.929	0.538	0.370	0.573

UQ	14	49521901	49522200	4.965	4.113	3.052	4.198
UQ	14	49705201	49705500	3.699	2.365	2.423	2.301
UQ	14	49960201	49960500	5.339	4.359	4.064	4.330
UQ	14	50109901	50110200	0.799	0.609	0.341	0.411
UQ	14	50248201	50248500	3.673	2.628	2.677	2.058
UQ	14	50279101	50279400	1.750	0.678	0.354	0.877
UQ	14	50458501	50458800	4.152	3.338	3.447	3.392
UQ	14	50784901	50785200	4.517	3.055	3.328	3.180
UQ	14	50854801	50855100	1.142	0.680	0.390	0.365
UQ	14	51057901	51058200	1.515	1.216	1.358	1.298
UQ	14	51075301	51075600	2.050	1.362	1.480	1.435
UQ	14	51419701	51420000	1.813	0.376	0.633	0.943
UQ	14	51777601	51777900	6.912	5.715	4.622	5.521
UQ	14	51803401	51803700	1.752	1.987	1.514	1.496
UQ	14	52072501	52072800	0.213	0.803	0.823	0.857
UQ	14	52207501	52207800	2.262	1.195	1.122	0.832
UQ	14	52434001	52434300	3.781	2.572	2.258	3.372
UQ	14	53102401	53102700	0.400	0.749	0.189	0.112
UQ	14	53142001	53142300	0.345	0.839	0.609	0.547
UQ	14	53271901	53272200	1.199	0.586	0.642	0.765
UQ	14	53491501	53491800	0.507	0.210	0.136	0.061
UQ	14	53568301	53568600	0.505	0.049	0.103	0.167
UQ	14	53759101	53759400	1.384	0.538	0.408	0.812
UQ	14	53796301	53796600	0.160	0.446	0.424	0.329
UQ	14	54031801	54032100	0.747	0.283	0.243	0.112
UQ	14	54064501	54064800	0.958	0.607	0.317	0.315
UQ	14	54188701	54189000	3.062	1.667	1.596	1.714
UQ	14	54599401	54599700	1.091	0.701	0.560	0.781
UQ	14	54733201	54733500	2.878	1.935	1.723	2.028
UQ	14	55046701	55047000	0.505	0.936	1.572	1.014
UQ	14	55071301	55071600	2.552	2.105	1.558	2.216
UQ	14	55462501	55462800	2.899	2.069	2.049	2.079
UQ	14	55463701	55464000	0.423	0.118	0.132	0.086
UQ	14	55525801	55526100	2.079	1.354	0.941	0.898
UQ	14	55661701	55662000	0.160	0.420	0.025	0.025
UQ	14	55700401	55700700	4.060	2.551	2.695	2.759
UQ	14	55776301	55776600	4.280	3.645	3.469	3.366
UQ	14	56016601	56016900	2.100	1.493	1.543	2.484
UQ	14	56027401	56027700	3.562	2.652	2.443	2.713
UQ	14	56042701	56043000	3.880	2.562	3.197	2.900
UQ	14	56130301	56130600	0.955	0.260	0.223	0.396
UQ	14	56298901	56299200	0.584	0.444	0.847	0.887
UQ	14	56618701	56619000	8.542	8.065	7.700	9.157
UQ	14	57425701	57426000	3.083	2.736	2.365	2.576
UQ	14	58284601	58284900	0.852	0.373	0.247	0.193
UQ	14	58493401	58493700	0.615	1.568	1.458	1.506
UQ	14	58493701	58494000	0.240	1.216	1.001	1.278
UQ	14	58745101	58745400	1.991	1.032	0.568	0.954
UQ	14	58745401	58745700	2.018	1.616	1.041	1.566
UQ	14	58806901	58807200	0.718	0.978	0.453	0.968
UQ	14	58864801	58865100	0.215	0.539	0.448	0.522
UQ	14	59019301	59019600	0.718	0.305	0.259	0.304
UQ	14	59065501	59065800	0.186	0.444	0.584	0.304
UQ	14	59379001	59379300	0.160	0.609	0.502	0.335
UQ	14	59492101	59492400	0.586	0.933	0.346	0.887
UQ	14	60199201	60199500	15.757	4.866	2.009	2.837
UQ	14	60550501	60550800	4.914	4.181	3.982	5.390
UQ	14	60566101	60566400	2.450	1.899	1.378	1.881
UQ	14	60611101	60611400	1.628	1.124	1.008	0.751
UQ	14	60752101	60752400	0.905	0.356	0.477	0.218
UQ	14	60842101	60842400	1.165	2.365	2.040	1.917
UQ	14	61308601	61308900	5.136	4.064	4.521	3.681
UQ	14	61361701	61362000	1.887	2.336	1.522	1.521
UQ	14	62315101	62315400	2.234	1.197	1.059	1.065
UQ	14	62345101	62345400	6.088	7.542	7.813	7.712
UQ	14	62630101	62630400	0.665	0.818	0.881	0.943
UQ	15	41701	42000	2.016	1.176	0.963	1.227

UQ	15	60301	60600	1.307	0.725	0.753	0.542
UQ	15	147901	148200	3.833	2.774	2.332	2.717
UQ	15	481801	482100	0.531	0.212	0.218	0.279
UQ	15	673201	673500	0.429	0.024	0.054	0.000
UQ	15	925501	925800	0.584	0.234	0.243	0.279
UQ	15	1362301	1362600	0.027	0.189	0.560	0.385
UQ	15	1386901	1387200	0.821	0.258	0.205	0.218
UQ	15	2204701	2205000	1.224	0.818	0.847	0.426
UQ	15	2253001	2253300	1.513	0.867	0.954	1.125
UQ	15	2328901	2329200	0.824	0.420	0.337	0.218
UQ	15	2968501	2968800	0.266	0.114	0.107	0.690
UQ	15	3594301	3594600	1.860	1.199	1.197	1.192
UQ	15	3594601	3594900	0.984	0.605	0.662	0.690
UQ	15	4215901	4216200	3.160	6.437	5.317	5.317
UQ	15	5214901	5215200	1.679	1.236	1.173	1.055
UQ	15	5507401	5507700	0.132	0.420	0.321	0.248
UQ	15	7053901	7054200	5.990	4.952	3.970	5.095
UQ	15	7544101	7544400	2.717	1.686	1.398	1.466
UQ	15	7669201	7669500	0.613	0.302	0.321	0.218
UQ	15	7742401	7742700	0.852	0.491	0.448	0.319
UQ	15	8095201	8095500	0.850	0.189	0.510	0.588
UQ	15	8562901	8563200	0.237	1.358	0.831	0.664
UQ	15	8981101	8981400	0.292	0.373	0.477	0.664
UQ	15	9122401	9122700	0.608	0.281	0.107	0.248
UQ	15	9193501	9193800	3.381	1.424	2.414	1.673
UQ	15	9444601	9444900	0.848	0.425	0.424	0.162
UQ	15	10197001	10197300	0.536	0.116	0.160	0.081
UQ	15	11095801	11096100	0.426	0.676	0.000	0.086
UQ	15	11742901	11743200	0.663	0.120	0.234	0.385
UQ	15	11838001	11838300	0.108	0.800	0.687	1.095
UQ	15	12063301	12063600	0.505	0.232	0.132	0.031
UQ	15	12223201	12223500	0.720	0.491	0.424	0.583
UQ	15	13618201	13618500	0.639	0.446	0.395	0.198
UQ	15	13748401	13748700	0.266	0.562	0.374	0.522
UQ	15	13823101	13823400	0.934	0.210	0.214	0.000
UQ	15	15071401	15071700	0.797	0.142	0.346	0.198
UQ	15	15119401	15119700	1.374	0.635	0.428	0.639
UQ	15	15246601	15246900	0.558	0.279	0.136	0.243
UQ	15	16941001	16941300	0.694	0.210	0.127	0.446
UQ	15	16976401	16976700	0.132	0.399	0.724	0.391
UQ	15	17007601	17007900	1.034	0.515	0.667	0.441
UQ	15	17472901	17473200	1.269	0.541	0.497	0.416
UQ	15	17614801	17615100	0.689	0.285	0.239	0.248
UQ	15	17615101	17615400	0.555	0.096	0.214	0.031
UQ	15	18776401	18776700	0.531	0.137	0.140	0.193
UQ	15	19329001	19329300	0.106	0.187	0.263	0.497
UQ	15	19563901	19564200	0.505	0.258	0.189	0.187
UQ	15	20049001	20049300	0.910	0.260	0.272	0.157
UQ	15	20227501	20227800	1.224	0.399	0.740	0.396
UQ	15	20255701	20256000	1.916	1.259	1.382	1.526
UQ	15	20739001	20739300	0.586	0.817	0.428	0.391
UQ	15	21302401	21302700	1.223	0.472	0.729	0.466
UQ	15	21495601	21495900	1.654	1.049	1.037	1.125
UQ	15	21566101	21566400	1.276	0.813	0.847	0.751
UQ	15	21615001	21615300	0.879	0.491	0.526	0.507
UQ	15	21695401	21695700	3.773	3.222	4.659	2.961
UQ	15	22389301	22389600	1.378	0.403	0.432	0.441
UQ	15	22389601	22389900	1.061	0.378	0.444	0.486
UQ	15	22389901	22390200	1.087	0.375	0.497	0.370
UQ	15	22431301	22431600	0.740	0.330	0.132	0.309
UQ	15	22524301	22524600	2.392	1.755	1.592	1.444
UQ	15	22636501	22636800	1.436	1.009	0.856	1.055
UQ	15	22707301	22707600	1.145	0.730	1.572	0.603
UQ	15	23091001	23091300	0.371	0.609	0.769	0.553
UQ	15	23158801	23159100	0.507	0.140	0.263	0.218
UQ	15	23264701	23265000	1.065	1.450	1.567	1.247
UQ	15	23289901	23290200	0.587	0.352	0.132	0.218

UQ	15	23418601	23418900	0.823	1.772	1.699	1.654
UQ	15	23523001	23523300	1.623	1.006	1.025	0.968
UQ	15	23835601	23835900	0.479	0.562	0.107	0.248
UQ	15	24190501	24190800	1.008	0.723	0.243	0.608
UQ	15	24372901	24373200	3.044	0.790	1.371	1.120
UQ	15	24924901	24925200	1.120	0.631	0.588	0.669
UQ	15	25015801	25016100	0.265	0.704	0.609	0.806
UQ	15	26084101	26084400	0.266	0.682	0.716	0.608
UQ	15	26223301	26223600	0.558	0.421	0.132	0.193
UQ	15	26706901	26707200	1.061	0.463	0.609	0.365
UQ	15	26787001	26787300	1.010	0.354	0.437	0.452
UQ	15	27833401	27833700	0.534	0.258	0.218	0.248
UQ	15	27956101	27956400	0.558	0.139	0.160	0.213
UQ	15	28519501	28519800	1.149	0.628	0.613	0.608
UQ	15	28519801	28520100	1.829	0.588	0.805	0.648
UQ	15	28939501	28939800	0.213	0.611	0.716	0.715
UQ	15	29216701	29217000	0.531	0.257	0.160	0.248
UQ	15	29235301	29235600	0.398	1.217	0.695	1.318
UQ	15	29585401	29585700	0.850	0.586	0.506	0.608
UQ	15	29847001	29847300	0.189	0.745	0.555	0.603
UQ	15	29865901	29866200	1.363	0.729	0.477	0.578
UQ	15	31103101	31103400	0.240	0.493	0.054	0.527
UQ	15	31522801	31523100	0.534	0.163	0.292	0.304
UQ	15	31574101	31574400	0.371	0.657	0.823	0.583
UQ	15	31725601	31725900	0.639	0.914	1.086	0.918
UQ	15	31759801	31760100	1.963	1.053	0.809	1.273
UQ	15	32055601	32055900	0.081	0.420	0.243	0.279
UQ	15	32177701	32178000	0.826	0.189	0.214	0.329
UQ	15	32178001	32178300	0.795	0.283	0.317	0.243
UQ	15	32529001	32529300	2.231	0.995	0.970	1.273
UQ	15	32784001	32784300	1.061	0.701	0.667	0.826
UQ	15	33303901	33304200	0.586	0.725	0.745	0.416
UQ	15	34261801	34262100	0.957	1.380	1.501	2.429
UQ	15	34536901	34537200	2.694	1.877	1.810	1.876
UQ	15	34661401	34661700	0.690	0.818	0.823	0.385
UQ	15	34677301	34677600	0.613	0.399	0.267	0.279
UQ	15	35187601	35187900	0.824	0.393	0.078	0.167
UQ	15	36333601	36333900	0.132	0.704	0.667	0.441
UQ	15	36959101	36959400	1.091	2.360	1.509	1.810
UQ	15	37403401	37403700	0.850	0.959	0.745	1.131
UQ	15	37799101	37799400	0.531	0.352	0.317	0.167
UQ	15	38423401	38423700	4.388	3.345	3.103	3.077
UQ	15	38760601	38760900	1.328	0.794	0.798	0.852
UQ	15	39347701	39348000	1.195	0.706	0.774	0.857
UQ	15	39486001	39486300	2.358	1.358	1.307	1.318
UQ	15	39532801	39533100	0.319	0.493	0.107	0.497
UQ	15	40137601	40137900	0.799	1.330	1.476	1.592
UQ	15	40888501	40888800	0.481	0.865	0.774	0.968
UQ	15	40902001	40902300	2.184	1.757	1.275	1.105
UQ	15	41081101	41081400	0.852	0.584	0.613	0.628
UQ	15	41189101	41189400	0.158	0.466	0.539	0.466
UQ	15	41190601	41190900	2.318	1.163	1.583	1.430
UQ	15	41738401	41738700	0.692	1.354	1.340	1.253
UQ	15	41853301	41853600	2.531	0.676	1.793	1.374
UQ	15	41967601	41967900	0.823	0.491	0.555	0.472
UQ	15	42344701	42345000	0.321	0.749	0.560	0.603
UQ	15	42475501	42475800	0.982	0.491	0.749	0.725
UQ	15	42666001	42666300	1.412	0.817	0.760	0.654
UQ	15	42754801	42755100	0.615	0.208	0.078	0.218
UQ	15	43226701	43227000	1.674	1.221	0.765	1.222
UQ	15	43563601	43563900	0.184	0.611	0.580	0.603
UQ	15	43871701	43872000	1.625	0.753	0.753	0.933
UQ	15	43894201	43894500	0.876	0.142	0.292	0.614
UQ	15	44037001	44037300	0.665	0.232	0.283	0.254
UQ	15	44560501	44560800	3.534	2.669	2.640	2.490
UQ	15	44560801	44561100	3.326	2.736	2.584	2.525
UQ	15	44571901	44572200	0.373	0.185	0.181	0.553

UQ	15	44576101	44576400	0.136	0.560	0.526	0.522
UQ	15	45923101	45923400	4.764	3.014	3.032	3.868
UQ	15	46059901	46060200	1.013	0.539	0.341	0.639
UQ	15	47292001	47292300	3.510	1.601	1.117	1.719
UQ	15	47678701	47679000	0.824	1.236	1.465	1.166
UQ	15	47946901	47947200	1.382	1.032	0.909	0.862
UQ	15	49483501	49483800	0.053	0.399	0.341	0.497
UQ	15	50145301	50145600	3.849	4.488	2.934	4.426
UQ	15	50214001	50214300	0.932	1.427	0.662	0.725
UQ	15	50214601	50214900	0.666	0.139	0.419	0.329
UQ	15	50494501	50494800	2.105	1.408	1.300	1.521
UQ	15	50937301	50937600	0.321	0.747	0.560	0.639
UQ	15	50974801	50975100	0.345	0.466	0.214	0.142
UQ	15	51489901	51490200	0.426	0.747	0.243	0.304
UQ	15	51724801	51725100	0.319	0.727	0.671	0.634
UQ	15	52248901	52249200	0.694	0.326	0.210	0.365
UQ	15	52640701	52641000	0.561	0.281	0.160	0.304
UQ	15	52715101	52715400	0.373	0.629	0.769	0.862
UQ	15	52790401	52790700	0.534	0.281	0.292	0.781
UQ	15	52927501	52927800	1.142	1.519	0.564	0.821
UQ	15	53643001	53643300	0.079	0.423	0.350	0.385
UQ	15	53776801	53777100	2.892	1.515	2.225	2.252
UQ	15	53870101	53870400	0.637	0.302	0.189	0.340
UQ	15	54851101	54851400	0.055	0.303	0.531	0.299
UQ	15	55681201	55681500	0.666	0.215	1.308	1.050
UQ	15	57967801	57968100	0.455	0.118	0.156	0.167
UQ	15	58661701	58662000	0.371	0.633	0.716	0.639
UQ	15	59373001	59373300	2.530	1.583	1.872	1.683
UQ	15	59391301	59391600	0.373	0.839	0.671	0.826
UQ	15	59933101	59933400	1.142	2.594	3.471	2.257
UQ	15	59933401	59933700	0.344	1.414	2.209	1.151
UQ	15	59938201	59938500	3.112	1.525	1.103	1.475
UQ	15	60016801	60017100	0.769	0.348	0.267	0.385
UQ	15	60538501	60538800	0.555	0.187	0.185	0.117
UQ	15	60977701	60978000	0.455	0.142	0.025	0.031
UQ	15	61095001	61095300	2.099	0.646	0.415	0.400
UQ	15	61295401	61295700	2.762	1.832	1.567	1.694
UQ	15	61335601	61335900	2.057	1.188	0.535	1.197
UQ	15	61335901	61336200	3.588	1.467	0.272	1.551
UQ	15	61594501	61594800	0.476	0.118	0.165	0.142
UQ	15	62221201	62221500	0.874	0.352	0.432	0.228
UQ	15	62227501	62227800	0.131	0.493	0.506	0.360
UQ	15	62296501	62296800	1.228	0.279	0.194	0.248
UQ	15	62739301	62739600	0.893	4.502	2.491	2.946
UQ	15	63136201	63136500	0.053	0.395	0.185	0.223
UQ	15	63563101	63563400	1.113	0.427	0.366	0.426
UQ	15	63569701	63570000	0.477	0.163	0.025	0.162
UQ	15	63605701	63606000	0.557	0.092	0.054	0.147
UQ	15	63630601	63630900	0.563	0.000	0.000	0.000
UQ	15	63773701	63774000	0.505	0.208	0.165	0.187
UQ	15	63833401	63833700	1.091	0.564	0.424	0.487
UQ	15	64178701	64179000	0.584	0.399	0.341	0.223
UQ	15	64179001	64179300	0.637	0.305	0.267	0.223
UQ	15	64408801	64409100	0.666	0.281	0.189	0.268
UQ	15	64655701	64656000	1.491	1.098	0.831	1.217
UQ	15	64723201	64723500	1.487	1.985	1.016	1.861
UQ	15	64834801	64835100	0.616	0.023	0.078	0.000
UQ	15	64907101	64907400	0.558	0.395	0.399	0.360
UQ	15	65072701	65073000	0.903	1.075	0.798	0.472
UQ	15	65451601	65451900	0.716	0.285	0.243	0.279
UQ	15	65520601	65520900	0.718	0.888	0.403	0.416
UQ	15	65584501	65584800	0.957	0.631	0.432	0.573
UQ	15	66266401	66266700	0.481	0.116	0.267	0.274
UQ	15	67205101	67205400	0.690	0.818	0.453	0.466
UQ	15	67234201	67234500	0.720	0.262	0.292	0.218
UQ	15	67726201	67726500	1.771	0.045	0.809	0.056
UQ	15	67726501	67726800	1.879	0.139	0.836	0.117

UQ	15	67800601	67800900	0.477	0.281	0.263	0.279
UQ	15	67938301	67938600	0.505	0.210	0.210	0.243
UQ	15	68961001	68961300	0.507	0.212	0.374	0.137
UQ	15	69228001	69228300	0.663	0.094	0.283	0.299
UQ	15	69502501	69502800	1.674	2.427	2.205	2.515
UQ	15	71169001	71169300	0.558	0.654	0.453	0.472
UQ	15	71398201	71398500	0.852	0.356	0.390	0.304
UQ	15	71575501	71575800	0.132	0.584	0.428	0.385
UQ	15	72123001	72123300	0.661	0.328	0.239	0.254
UQ	15	72221101	72221400	2.342	3.199	3.119	3.518
UQ	15	72441901	72442200	0.666	0.352	0.370	0.254
UQ	15	72451501	72451800	0.610	0.213	0.230	0.112
UQ	15	72451801	72452100	1.065	0.423	0.403	0.482
UQ	15	72699601	72699900	0.742	0.116	0.136	0.086
UQ	15	72709801	72710100	0.505	0.983	1.023	1.100
UQ	15	72861301	72861600	5.983	4.243	4.759	4.730
UQ	15	72864901	72865200	0.479	0.258	0.214	0.634
UQ	15	72884701	72885000	2.897	1.828	1.538	1.596
UQ	15	72892501	72892800	4.136	1.880	2.891	2.677
UQ	15	72899101	72899400	1.595	0.781	0.276	0.446
UQ	15	72902401	72902700	2.310	0.938	1.252	0.731
UQ	15	72923401	72923700	0.507	0.350	0.239	0.776
UQ	15	73027501	73027800	0.106	0.678	0.243	0.329
UQ	15	73072501	73072800	1.379	0.845	0.843	0.771
UQ	15	73189501	73189800	0.852	0.656	0.502	1.389
UQ	15	73311601	73311900	0.584	0.468	0.876	0.391
UQ	15	73339201	73339500	0.503	0.142	0.239	0.254
UQ	15	73702801	73703100	0.132	0.375	0.477	0.360
UQ	15	73790401	73790700	0.455	0.047	0.049	0.051
UQ	15	74146801	74147100	1.305	0.817	0.818	0.893
UQ	15	74202601	74202900	0.106	0.330	0.477	0.248
UQ	15	74286301	74286600	0.240	0.888	0.774	0.684
UQ	15	74636401	74636700	1.571	0.489	0.916	1.039
UQ	15	75242101	75242400	1.113	1.498	0.794	0.771
UQ	15	75474001	75474300	1.247	0.702	0.847	0.583
UQ	15	75489001	75489300	1.625	1.985	1.099	1.125
UQ	15	75527101	75527400	1.728	0.877	0.881	0.938
UQ	15	75956401	75956700	2.787	3.225	2.356	3.235
UQ	15	76014301	76014600	0.160	0.633	0.453	0.664
UQ	15	76986001	76986300	0.534	1.008	0.769	1.055
UQ	15	77371201	77371500	1.649	0.517	0.774	1.125
UQ	15	77371501	77371800	2.100	0.685	0.934	1.369
UQ	15	77449201	77449500	0.477	0.210	0.720	0.218
UQ	15	77597101	77597400	0.477	0.210	0.132	0.299
UQ	15	77600101	77600400	0.295	0.654	0.588	0.578
UQ	15	77658301	77658600	4.441	3.289	3.126	3.393
UQ	15	77778601	77778900	4.702	3.375	2.949	3.417
UQ	15	77966701	77967000	1.542	1.120	0.818	0.842
UQ	15	78170101	78170400	0.503	0.165	0.205	0.137
UQ	15	78261901	78262200	7.906	4.602	4.023	4.007
UQ	15	78394201	78394500	6.566	1.553	4.589	4.500
UQ	15	78432001	78432300	2.602	3.435	3.772	3.412
UQ	15	78614101	78614400	5.162	3.903	3.127	3.762
UQ	15	78790801	78791100	1.255	0.286	0.325	0.396
UQ	15	79252801	79253100	0.744	0.352	0.346	0.162
UQ	15	79283701	79284000	3.383	2.635	1.674	2.758
UQ	15	79392001	79392300	0.455	0.118	0.049	0.051
UQ	15	79590301	79590600	0.265	0.539	0.477	0.502
UQ	15	79616701	79617000	0.756	3.639	3.511	3.160
UQ	15	79617001	79617300	1.239	4.310	4.202	3.779
UQ	15	79938601	79938900	3.112	1.481	1.607	1.592
UQ	15	80043601	80043900	0.450	1.120	0.896	1.075
UQ	15	80089501	80089800	0.318	1.054	1.032	0.583
UQ	15	80238301	80238600	3.217	1.622	1.908	1.922
UQ	15	80332801	80333100	1.197	0.498	0.486	0.391
UQ	15	80715001	80715300	1.408	0.963	0.959	1.105
UQ	15	80757601	80757900	0.929	0.465	0.564	0.497

UQ	15	80778001	80778300	1.729	2.291	1.888	1.856
UQ	15	80812501	80812800	1.011	1.098	1.436	0.801
UQ	15	80871601	80871900	0.268	0.421	0.584	0.583
UQ	16	177601	177900	1.328	0.539	0.827	2.109
UQ	16	525901	526200	1.223	0.581	0.370	0.320
UQ	16	528001	528300	1.171	1.495	1.358	0.745
UQ	16	599101	599400	0.639	0.796	0.847	0.801
UQ	16	609301	609600	0.584	0.281	0.321	0.360
UQ	16	628201	628500	1.331	0.425	0.448	0.567
UQ	16	628501	628800	2.392	0.831	1.291	1.024
UQ	16	639901	640200	2.549	3.315	3.594	3.422
UQ	16	1151401	1151700	1.281	0.657	0.555	0.634
UQ	16	1178401	1178700	0.318	0.517	0.584	0.553
UQ	16	1492201	1492500	2.873	1.817	2.312	1.982
UQ	16	1718701	1719000	1.594	0.865	0.740	0.817
UQ	16	1832701	1833000	1.255	0.472	0.283	0.254
UQ	16	2154301	2154600	0.718	0.399	0.317	0.405
UQ	16	2265301	2265600	0.345	0.234	0.107	0.466
UQ	16	2292601	2292900	3.005	2.381	2.555	2.079
UQ	16	2504401	2504700	0.881	0.347	0.379	0.279
UQ	16	2531401	2531700	1.568	1.120	1.041	1.029
UQ	16	2765701	2766000	2.971	1.641	1.632	1.912
UQ	16	2801101	2801400	1.616	1.032	1.066	1.080
UQ	16	3715801	3716100	0.823	0.373	0.555	1.116
UQ	16	3969301	3969600	2.310	1.796	1.728	1.836
UQ	16	4191601	4191900	1.279	0.818	0.568	0.659
UQ	16	4237201	4237500	0.531	0.352	0.132	0.329
UQ	16	4267201	4267500	3.057	3.557	3.710	4.061
UQ	16	4355101	4355400	1.278	0.286	0.519	0.639
UQ	16	4542601	4542900	1.516	0.978	0.736	0.720
UQ	16	4672501	4672800	2.362	1.802	1.534	1.856
UQ	16	4718401	4718700	1.941	1.197	1.201	1.328
UQ	16	4858801	4859100	0.665	0.189	0.374	0.355
UQ	16	5346901	5347200	0.605	1.805	1.670	2.170
UQ	16	5409001	5409300	1.010	0.725	0.580	0.360
UQ	16	5429401	5429700	0.345	0.421	0.584	0.223
UQ	16	5778301	5778600	1.913	1.236	1.362	1.298
UQ	16	5889301	5889600	0.079	0.586	0.325	0.583
UQ	16	6087001	6087300	0.160	0.513	0.239	0.416
UQ	16	6225901	6226200	1.010	0.794	0.638	0.664
UQ	16	6333001	6333300	0.690	0.331	0.239	0.335
UQ	16	6460201	6460500	0.239	0.583	0.428	0.578
UQ	16	6975901	6976200	1.412	1.120	0.691	0.948
UQ	16	7380001	7380300	0.797	0.376	0.609	0.583
UQ	16	7754101	7754400	1.091	0.818	0.798	0.801
UQ	16	8174401	8174700	0.373	0.187	0.502	0.142
UQ	16	8398501	8398800	1.092	0.914	0.745	0.360
UQ	16	9317401	9317700	0.215	0.654	0.584	0.679
UQ	16	9501601	9501900	0.294	0.420	0.185	0.527
UQ	16	9689101	9689400	0.720	0.888	0.317	0.411
UQ	16	9794701	9795000	1.171	0.699	0.765	0.862
UQ	16	9906001	9906300	2.326	0.753	0.580	0.547
UQ	16	9916501	9916800	0.531	0.888	0.272	1.044
UQ	16	10269601	10269900	0.026	0.283	0.682	0.608
UQ	16	10290301	10290600	0.718	0.421	0.292	0.380
UQ	16	10633801	10634100	1.120	0.566	0.642	0.690
UQ	16	11838001	11838300	0.507	0.163	0.087	0.157
UQ	16	12025501	12025800	1.278	0.770	0.535	0.487
UQ	16	12187801	12188100	0.690	0.208	0.288	0.167
UQ	16	12203401	12203700	1.913	1.635	2.185	1.430
UQ	16	12743401	12743700	2.416	1.798	1.226	1.536
UQ	16	13326001	13326300	0.558	0.073	0.243	0.187
UQ	16	14524201	14524500	1.649	1.075	0.687	1.039
UQ	16	14821801	14822100	0.453	0.609	0.239	0.304
UQ	16	14862601	14862900	0.505	0.352	0.156	0.248
UQ	16	15414601	15414900	0.771	1.075	1.271	1.166
UQ	16	16440601	16440900	0.611	0.307	0.346	0.254

UQ	16	16666801	16667100	0.581	0.120	0.111	0.025
UQ	16	16806601	16806900	2.047	2.504	2.662	2.931
UQ	16	16808101	16808400	0.907	2.650	2.037	1.678
UQ	16	16995301	16995600	1.466	0.980	0.544	0.527
UQ	16	18133201	18133500	0.450	0.096	0.025	0.086
UQ	16	18736201	18736500	0.532	0.350	0.239	0.309
UQ	16	18751201	18751500	2.633	4.344	4.809	4.011
UQ	16	18753301	18753600	0.666	0.232	0.132	0.056
UQ	16	18821701	18822000	0.637	0.281	0.325	0.365
UQ	16	18890101	18890400	0.536	0.024	0.127	0.142
UQ	16	19032001	19032300	1.513	1.214	0.959	0.745
UQ	16	19344901	19345200	0.534	1.127	1.086	1.039
UQ	16	19346701	19347000	0.558	0.120	0.074	0.187
UQ	16	19706101	19706400	0.586	1.103	1.271	1.091
UQ	16	19968001	19968300	1.755	1.188	1.106	1.262
UQ	16	20403601	20403900	0.694	0.399	0.325	0.385
UQ	16	20565001	20565300	0.802	0.423	0.374	0.416
UQ	16	21347401	21347700	0.186	0.420	0.292	0.522
UQ	16	21391801	21392100	0.397	0.701	0.662	0.948
UQ	16	21612001	21612300	0.187	0.633	0.399	0.355
UQ	16	22229401	22229700	0.769	0.187	0.194	0.309
UQ	16	22805101	22805400	0.532	0.191	0.214	0.198
UQ	16	24155701	24156000	0.371	0.513	0.185	0.137
UQ	16	24268801	24269100	2.528	3.137	2.000	1.947
UQ	16	24854701	24855000	1.170	0.729	1.843	0.751
UQ	16	24957601	24957900	0.532	0.255	0.288	0.304
UQ	16	25597201	25597500	0.929	0.536	0.502	0.396
UQ	16	27118501	27118800	1.244	0.494	0.604	0.614
UQ	16	27118801	27119100	1.037	0.470	0.390	0.558
UQ	16	27225301	27225600	0.476	0.234	0.132	0.193
UQ	16	27829201	27829500	1.618	0.611	0.658	0.963
UQ	16	29136901	29137200	4.038	4.992	2.238	3.209
UQ	16	29913001	29913300	0.455	1.079	1.144	1.050
UQ	16	30113101	30113400	2.389	1.871	2.858	1.744
UQ	16	30251701	30252000	0.106	0.352	0.555	0.690
UQ	16	30633001	30633300	0.292	0.515	0.136	0.051
UQ	16	31327501	31327800	0.557	0.747	0.399	0.391
UQ	16	31398001	31398300	0.345	0.491	0.189	0.223
UQ	16	31566901	31567200	0.663	0.444	0.350	0.106
UQ	16	31844401	31844700	0.292	0.163	0.185	0.578
UQ	16	32773801	32774100	3.936	4.345	3.204	5.116
UQ	16	32803501	32803800	0.720	0.489	0.481	0.411
UQ	16	33338101	33338400	0.799	0.517	0.424	0.329
UQ	16	34605601	34605900	1.279	0.983	0.823	0.583
UQ	16	34812001	34812300	0.532	0.773	0.876	0.690
UQ	16	34994701	34995000	0.186	0.399	0.613	0.502
UQ	16	35072101	35072400	0.531	0.187	0.194	0.132
UQ	16	35915101	35915400	1.254	0.727	0.856	0.857
UQ	16	36135901	36136200	1.087	0.657	0.633	0.360
UQ	16	36184801	36185100	0.081	0.583	0.992	0.461
UQ	16	36241201	36241500	0.505	0.352	0.346	0.385
UQ	16	36476401	36476700	0.319	0.444	0.189	0.162
UQ	16	36703501	36703800	0.294	0.631	0.448	0.106
UQ	16	38232301	38232600	0.158	1.077	1.366	1.430
UQ	16	38277901	38278200	0.586	0.328	0.234	0.324
UQ	16	39173701	39174000	0.053	0.702	0.267	0.472
UQ	16	39654301	39654600	1.168	0.684	0.234	0.477
UQ	16	39964201	39964500	0.503	0.163	0.156	0.193
UQ	16	40818901	40819200	2.207	1.545	1.173	1.541
UQ	16	40893601	40893900	0.507	0.303	0.214	0.248
UQ	16	40932301	40932600	0.690	1.403	0.872	1.105
UQ	16	41219101	41219400	0.481	0.137	0.054	0.000
UQ	16	41406601	41406900	5.319	4.227	3.931	4.138
UQ	16	41489701	41490000	0.902	1.568	2.036	1.293
UQ	16	42932701	42933000	0.586	0.120	0.346	0.304
UQ	16	43959601	43959900	0.637	0.139	0.136	0.446
UQ	16	44510401	44510700	1.441	0.865	0.740	0.867

UQ	16	45357601	45357900	0.476	0.163	0.189	0.162
UQ	16	46680601	46680900	1.434	0.611	1.037	0.649
UQ	16	46800001	46800300	2.262	1.760	0.798	1.212
UQ	16	46842001	46842300	1.778	2.480	2.456	2.966
UQ	16	47352601	47352900	0.292	0.491	0.535	0.527
UQ	16	47976901	47977200	0.210	0.674	0.658	0.608
UQ	16	48095701	48096000	0.640	0.350	0.292	0.299
UQ	16	48156601	48156900	0.423	0.096	0.029	0.031
UQ	16	48889201	48889500	0.958	0.420	0.399	0.513
UQ	16	49682701	49683000	0.295	0.562	0.054	0.086
UQ	16	49965001	49965300	0.347	0.560	0.691	0.893
UQ	16	50160601	50160900	0.582	0.187	0.156	0.051
UQ	16	50751901	50752200	0.799	0.069	0.428	0.081
UQ	16	50771401	50771700	0.764	2.746	2.069	2.870
UQ	16	50812501	50812800	0.797	0.024	0.477	0.000
UQ	16	52287601	52287900	0.507	0.185	0.165	0.173
UQ	16	53955001	53955300	0.852	0.262	0.481	0.279
UQ	16	54725101	54725400	0.799	0.373	0.272	0.553
UQ	16	55037701	55038000	0.640	0.279	0.259	0.248
UQ	16	55360501	55360800	0.531	0.232	0.288	0.198
UQ	16	55593601	55593900	0.905	0.605	0.609	0.614
UQ	16	55866901	55867200	0.371	0.702	0.239	0.527
UQ	16	55896001	55896300	0.053	0.371	0.457	0.365
UQ	16	56025601	56025900	0.558	0.258	0.267	0.056
UQ	16	56119801	56120100	0.503	0.116	0.160	0.248
UQ	16	57390301	57390600	0.720	0.094	0.288	0.162
UQ	16	57456001	57456300	1.329	1.802	1.665	1.739
UQ	16	57793501	57793800	0.000	0.446	0.181	0.167
UQ	16	57795901	57796200	1.010	0.633	0.448	0.710
UQ	16	58200301	58200600	1.700	1.543	1.329	1.247
UQ	16	58378201	58378500	2.665	1.748	1.752	1.410
UQ	16	58503901	58504200	0.742	1.217	0.419	1.156
UQ	16	58534801	58535100	0.081	0.281	0.419	0.472
UQ	16	58759201	58759500	1.542	0.936	0.967	0.608
UQ	16	58768201	58768500	0.160	0.633	0.481	0.578
UQ	16	58930801	58931100	0.266	0.676	0.613	0.553
UQ	16	59293501	59293800	0.477	0.137	0.185	0.056
UQ	16	59652301	59652600	0.532	0.303	0.239	0.299
UQ	16	59935501	59935800	1.886	1.017	0.847	0.512
UQ	16	59957701	59958000	0.505	0.444	0.267	0.167
UQ	16	59969101	59969400	1.563	0.468	0.843	0.735
UQ	16	60653101	60653400	0.160	0.420	0.560	0.274
UQ	16	61146901	61147200	0.771	0.096	0.305	0.056
UQ	16	61147201	61147500	1.226	0.279	0.388	0.147
UQ	16	61416601	61416900	2.394	2.034	2.131	2.160
UQ	16	62001901	62002200	0.957	0.470	0.403	0.223
UQ	16	62191501	62191800	1.381	0.908	0.448	0.765
UQ	16	62295601	62295900	0.747	0.187	0.156	0.335
UQ	16	62343001	62343300	0.163	1.152	2.060	1.079
UQ	16	62450701	62451000	1.329	1.847	0.885	0.902
UQ	16	62650501	62650800	0.505	0.680	0.321	0.335
UQ	16	62695201	62695500	0.426	0.961	0.160	0.279
UQ	16	62780701	62781000	3.567	6.052	5.586	6.012
UQ	16	62859901	62860200	0.615	1.736	1.369	1.785
UQ	16	62892301	62892600	0.373	0.963	0.905	0.913
UQ	16	62939401	62939700	0.555	0.140	0.181	0.081
UQ	16	63087901	63088200	0.958	0.468	0.346	0.279
UQ	16	63755101	63755400	1.407	1.757	0.934	0.968
UQ	16	63914401	63914700	0.265	0.538	0.531	0.385
UQ	16	64014901	64015200	1.010	0.725	0.531	0.882
UQ	16	64059001	64059300	0.610	0.167	0.317	0.198
UQ	16	64310401	64310700	4.020	2.742	2.570	2.692
UQ	16	64733401	64733700	1.942	0.429	0.509	0.147
UQ	16	64733701	64734000	5.401	1.285	1.207	0.406
UQ	16	64868101	64868400	0.503	0.094	0.132	0.248
UQ	16	65195401	65195700	0.108	0.747	0.506	0.502
UQ	16	65206801	65207100	1.708	0.963	0.794	0.781

UQ	16	65242501	65242800	1.913	1.034	0.930	0.817
UQ	16	65331001	65331300	0.771	1.004	0.588	1.293
UQ	16	65342101	65342400	1.328	1.684	0.905	1.597
UQ	16	65355601	65355900	0.477	1.036	0.983	1.075
UQ	16	65725201	65725500	0.584	0.723	0.399	0.248
UQ	16	66315301	66315600	0.105	0.702	0.629	0.644
UQ	16	66413701	66414000	2.099	1.429	0.959	1.257
UQ	16	66558001	66558300	1.999	1.004	1.543	1.060
UQ	16	66576601	66576900	0.876	0.678	0.662	0.466
UQ	16	66729001	66729300	1.679	3.169	3.081	2.835
UQ	16	67080301	67080600	0.984	0.420	0.617	0.517
UQ	16	67104301	67104600	2.047	1.217	1.074	1.298
UQ	16	67113301	67113600	0.718	0.539	0.560	0.329
UQ	16	67756801	67757100	2.342	1.116	1.041	0.907
UQ	16	67779901	67780200	3.713	2.077	2.086	0.699
UQ	16	67780201	67780500	2.918	1.843	1.819	0.553
UQ	16	67856701	67857000	0.769	0.493	0.160	0.472
UQ	16	68043601	68043900	0.532	0.350	0.000	0.360
UQ	16	68067601	68067900	2.233	1.525	1.719	1.668
UQ	16	68183101	68183400	0.663	0.258	0.346	0.213
UQ	16	68339101	68339400	0.503	0.281	0.156	0.274
UQ	16	68790901	68791200	1.542	1.081	1.045	0.887
UQ	16	69280201	69280500	1.432	0.869	0.959	0.857
UQ	16	69283201	69283500	4.231	1.706	1.957	1.450
UQ	16	69284101	69284400	0.611	0.466	0.234	0.304
UQ	16	69287401	69287700	6.866	7.942	7.960	9.100
UQ	16	69373501	69373800	3.567	3.223	2.579	4.041
UQ	16	69576301	69576600	0.505	1.075	0.798	0.821
UQ	16	69676201	69676500	0.690	0.470	0.189	0.441
UQ	16	69774901	69775200	1.144	0.772	0.588	0.547
UQ	16	69786001	69786300	2.826	5.242	1.632	1.627
UQ	16	69794101	69794400	6.687	10.131	4.391	3.719
UQ	16	69796201	69796500	8.141	12.792	3.419	4.691
UQ	16	69798301	69798600	2.717	4.066	1.103	0.968
UQ	16	69798601	69798900	1.807	2.536	0.724	1.014
UQ	16	70279801	70280100	3.283	0.884	1.173	0.751
UQ	16	70291501	70291800	2.918	1.481	1.449	1.723
UQ	16	70297201	70297500	0.534	0.303	0.292	0.309
UQ	16	70331401	70331700	1.355	0.382	0.152	0.234
UQ	16	70640401	70640700	0.826	0.539	0.526	0.466
UQ	16	70698301	70698600	6.411	4.881	5.565	4.097
UQ	16	70908901	70909200	0.532	0.210	0.132	0.081
UQ	16	70952101	70952400	4.023	5.398	5.379	5.527
UQ	16	71066101	71066400	5.982	4.126	3.587	4.217
UQ	16	71096701	71097000	0.347	0.513	0.502	0.608
UQ	16	71112301	71112600	2.476	1.732	1.576	1.881
UQ	16	71226601	71226900	2.786	0.496	1.253	0.573
UQ	16	71418601	71418900	0.479	0.538	0.239	0.304
UQ	16	71621701	71622000	3.770	2.001	2.011	2.338
UQ	17	239401	239700	0.477	0.097	0.127	0.106
UQ	17	1589101	1589400	0.799	0.234	0.424	0.436
UQ	17	1974001	1974300	2.328	0.831	0.860	0.893
UQ	17	2189401	2189700	0.215	0.069	0.399	0.583
UQ	17	2564701	2565000	1.036	0.586	0.584	0.583
UQ	17	3474601	3474900	0.532	0.397	0.453	0.279
UQ	17	3480901	3481200	0.398	0.513	0.292	0.218
UQ	17	3539101	3539400	0.984	0.238	0.301	0.457
UQ	17	3736501	3736800	0.239	0.421	0.535	0.522
UQ	17	3836101	3836400	0.661	0.232	0.267	0.304
UQ	17	3980401	3980700	0.903	0.444	0.720	0.725
UQ	17	4053601	4053900	1.649	0.702	0.847	0.801
UQ	17	4416601	4416900	1.010	0.657	0.526	0.695
UQ	17	4426801	4427100	0.531	0.232	0.234	0.198
UQ	17	4586701	4587000	0.689	0.397	0.296	0.304
UQ	17	4644301	4644600	0.508	0.140	0.160	0.248
UQ	17	5224201	5224500	0.774	0.470	0.346	0.416
UQ	17	5722201	5722500	2.367	1.562	1.665	1.091

UQ	17	5943301	5943600	0.852	1.262	1.518	1.566
UQ	17	5967001	5967300	4.625	6.715	5.745	5.908
UQ	17	6051001	6051300	0.586	0.140	0.267	0.284
UQ	17	6068101	6068400	0.557	0.165	0.210	0.061
UQ	17	6272401	6272700	2.815	2.030	2.122	2.464
UQ	17	6327001	6327300	0.584	0.348	0.107	0.391
UQ	17	6363001	6363300	0.105	0.397	0.497	0.527
UQ	17	6675301	6675600	0.184	0.659	0.667	0.751
UQ	17	6912301	6912600	2.370	0.257	0.648	0.833
UQ	17	7007701	7008000	0.052	0.303	0.399	0.547
UQ	17	7377001	7377300	0.637	0.375	0.374	0.329
UQ	17	7408801	7409100	5.404	3.332	3.105	3.804
UQ	17	8089201	8089500	5.528	3.673	4.011	4.613
UQ	17	8721901	8722200	1.410	1.075	0.522	0.933
UQ	17	9119401	9119700	0.373	0.513	0.580	1.050
UQ	17	9615001	9615300	1.625	0.826	0.361	0.765
UQ	17	9685801	9686100	1.066	0.489	0.560	0.628
UQ	17	9719401	9719700	1.628	2.598	2.871	2.313
UQ	17	9785701	9786000	0.694	1.053	1.115	1.506
UQ	17	9909301	9909600	3.210	1.154	1.451	1.977
UQ	17	9940801	9941100	0.613	0.350	0.156	0.391
UQ	17	10110301	10110600	0.955	0.517	0.638	0.659
UQ	17	10113001	10113300	2.111	5.413	4.837	4.569
UQ	17	10164601	10164900	0.318	0.468	0.078	0.502
UQ	17	10296301	10296600	0.582	1.054	0.934	1.019
UQ	17	10404301	10404600	0.160	0.418	0.346	0.639
UQ	17	10485301	10485600	0.557	0.185	0.243	0.223
UQ	17	10853401	10853700	0.213	0.564	0.502	0.502
UQ	17	11502301	11502600	0.665	0.210	0.078	0.106
UQ	17	11503801	11504100	0.457	0.023	0.025	0.056
UQ	17	11627401	11627700	0.900	0.468	0.350	0.299
UQ	17	11721901	11722200	1.439	1.821	0.691	0.695
UQ	17	12069901	12070200	0.455	0.071	0.132	0.086
UQ	17	12149701	12150000	0.239	0.375	0.613	0.329
UQ	17	12362401	12362700	0.186	0.609	0.473	0.391
UQ	17	12485101	12485400	0.106	0.420	0.374	0.391
UQ	17	12487801	12488100	1.621	1.169	0.921	1.136
UQ	17	12863101	12863400	1.065	0.676	0.662	0.664
UQ	17	12870301	12870600	0.984	0.562	0.424	0.852
UQ	17	13098901	13099200	0.452	0.562	0.691	0.329
UQ	17	13103401	13103700	0.292	0.633	0.510	0.527
UQ	17	13558801	13559100	0.957	0.395	0.403	0.614
UQ	17	13778401	13778700	0.692	0.470	0.185	0.335
UQ	17	14164801	14165100	0.586	0.395	0.317	0.198
UQ	17	14489101	14489400	0.584	0.260	0.263	0.268
UQ	17	15102001	15102300	2.181	0.049	0.074	1.298
UQ	17	15102301	15102600	2.231	0.168	0.132	1.439
UQ	17	15597001	15597300	1.515	1.167	0.662	0.852
UQ	17	16438501	16438800	0.265	1.081	0.642	0.634
UQ	17	16568101	16568400	0.187	0.541	0.580	0.558
UQ	17	16611601	16611900	0.853	0.631	0.341	0.639
UQ	17	16851001	16851300	0.716	1.122	0.959	0.918
UQ	17	17371801	17372100	0.081	0.468	0.296	0.365
UQ	17	17965501	17965800	2.342	1.553	1.641	1.440
UQ	17	18481801	18482100	0.400	0.586	0.633	0.223
UQ	17	18618601	18618900	1.008	0.631	0.749	0.441
UQ	17	19871701	19872000	0.823	0.631	0.399	0.522
UQ	17	20535001	20535300	0.639	0.305	0.210	0.309
UQ	17	22414501	22414800	0.398	0.865	0.765	0.968
UQ	17	24308101	24308400	0.266	0.607	0.370	0.472
UQ	17	24497701	24498000	0.158	0.446	0.346	0.391
UQ	17	25351801	25352100	0.400	0.607	0.218	0.056
UQ	17	25881601	25881900	1.092	0.519	0.544	0.411
UQ	17	25949701	25950000	0.586	0.421	0.156	0.274
UQ	17	26011501	26011800	0.531	0.260	0.078	0.086
UQ	17	26644201	26644500	0.505	0.191	0.165	0.137
UQ	17	27432901	27433200	1.833	0.564	0.702	1.055

UQ	17	29794201	29794500	1.674	0.345	0.392	0.623
UQ	17	30083701	30084000	0.160	0.466	0.292	0.335
UQ	17	30189001	30189300	0.239	0.538	0.555	0.477
UQ	17	36576901	36577200	0.477	0.140	0.103	0.137
UQ	17	37656901	37657200	1.224	0.798	0.609	0.533
UQ	17	38132401	38132700	1.221	0.541	0.818	0.725
UQ	17	38433001	38433300	0.685	0.142	0.136	0.162
UQ	17	38840701	38841000	0.876	0.444	0.383	0.416
UQ	17	39498901	39499200	5.270	3.008	4.456	3.386
UQ	17	39717001	39717300	0.611	0.163	0.296	0.112
UQ	17	39913801	39914100	0.161	0.656	0.597	0.720
UQ	17	40352101	40352400	0.053	0.446	0.267	0.416
UQ	17	40617001	40617300	0.929	0.657	0.560	0.659
UQ	17	41419501	41419800	0.721	0.348	0.210	0.329
UQ	17	41659501	41659800	0.428	1.309	1.226	0.659
UQ	17	42089701	42090000	1.008	0.519	0.584	0.365
UQ	17	42369901	42370200	0.718	0.260	0.292	0.299
UQ	17	42439201	42439500	0.613	0.167	0.087	0.187
UQ	17	43140001	43140300	0.531	0.305	0.156	0.299
UQ	17	43214701	43215000	0.187	0.444	0.403	0.614
UQ	17	43273801	43274100	0.878	0.702	0.531	0.690
UQ	17	43428001	43428300	0.316	0.862	0.959	0.832
UQ	17	44120701	44121000	1.242	2.223	3.576	3.047
UQ	17	44306701	44307000	0.982	0.536	0.502	0.588
UQ	17	44319001	44319300	3.349	2.948	2.949	2.652
UQ	17	44352901	44353200	2.689	1.414	1.039	1.040
UQ	17	44502301	44502600	0.853	0.420	0.374	0.416
UQ	17	44764501	44764800	1.494	0.846	0.818	0.796
UQ	17	44880001	44880300	1.329	0.770	0.979	0.725
UQ	17	44907001	44907300	0.215	0.466	0.531	0.527
UQ	17	45512701	45513000	0.637	0.283	0.243	0.117
UQ	17	46013401	46013700	0.052	0.350	0.551	0.472
UQ	17	46108201	46108500	0.053	0.515	0.292	0.228
UQ	17	46727401	46727700	0.508	0.208	0.185	0.112
UQ	17	46887601	46887900	0.423	0.069	0.127	0.000
UQ	17	47034601	47034900	1.889	0.985	1.480	1.359
UQ	17	47601301	47601600	0.557	0.302	0.317	0.248
UQ	17	48117001	48117300	1.408	0.727	1.144	0.887
UQ	17	48618001	48618300	0.106	0.586	0.379	0.472
UQ	17	49370101	49370400	1.329	0.843	0.234	0.943
UQ	17	49646101	49646400	2.097	0.901	0.873	0.574
UQ	17	49688101	49688400	1.039	1.641	1.329	1.633
UQ	17	49765201	49765500	0.290	0.701	0.078	0.056
UQ	17	49789801	49790100	0.823	0.448	0.370	0.553
UQ	17	49790701	49791000	0.505	0.185	0.074	0.051
UQ	17	50319301	50319600	1.729	1.216	1.099	0.857
UQ	17	50415001	50415300	0.452	0.981	1.028	0.877
UQ	17	50964601	50964900	2.499	1.285	1.739	1.505
UQ	17	51169201	51169500	4.549	5.188	5.047	5.491
UQ	17	51605701	51606000	0.771	0.444	0.243	0.522
UQ	17	51716101	51716400	3.060	2.296	2.517	2.373
UQ	17	52167301	52167600	0.557	1.216	0.794	0.837
UQ	17	52404601	52404900	1.036	0.305	0.325	0.288
UQ	17	52413001	52413300	0.531	0.258	0.185	0.228
UQ	17	52465801	52466100	1.360	0.910	1.037	0.690
UQ	17	52899001	52899300	0.505	0.326	0.263	0.360
UQ	17	53043601	53043900	0.160	0.328	0.263	0.527
UQ	17	53130601	53130900	0.294	0.607	0.132	0.472
UQ	17	53780101	53780400	0.213	0.678	0.424	0.502
UQ	17	54473701	54474000	0.532	0.255	0.160	0.218
UQ	17	54676801	54677100	0.213	0.678	0.555	0.614
UQ	17	54901201	54901500	0.476	0.165	0.136	0.193
UQ	17	55233601	55233900	4.527	3.611	3.212	2.454
UQ	17	55327501	55327800	0.665	0.161	0.263	0.391
UQ	17	55527901	55528200	0.531	0.210	0.321	0.187
UQ	17	55718101	55718400	2.229	1.405	1.398	1.592
UQ	17	55867501	55867800	0.721	0.163	0.317	0.213

UQ	17	56073301	56073600	0.503	0.257	0.210	0.304
UQ	17	56092801	56093100	0.105	0.605	0.374	0.411
UQ	17	56133601	56133900	0.477	0.234	0.136	0.193
UQ	17	56185201	56185500	0.477	0.189	0.185	0.142
UQ	17	56185501	56185800	0.505	0.232	0.210	0.106
UQ	17	56244901	56245200	4.217	0.701	2.060	0.996
UQ	17	56245201	56245500	1.013	0.309	0.337	0.345
UQ	17	56895301	56895600	3.560	2.414	2.028	2.413
UQ	17	57304501	57304800	2.367	1.450	1.875	1.947
UQ	17	57612901	57613200	0.769	0.470	0.296	0.299
UQ	17	57693601	57693900	0.853	0.470	0.189	0.385
UQ	17	58559401	58559700	2.533	1.496	1.268	1.213
UQ	17	58571101	58571400	1.194	1.959	2.336	1.784
UQ	17	58866001	58866300	0.481	0.257	0.263	0.218
UQ	17	58866601	58866900	0.668	0.257	0.321	0.284
UQ	17	58993501	58993800	3.004	2.662	1.855	2.130
UQ	17	59171401	59171700	4.331	3.602	1.973	3.611
UQ	17	59243701	59244000	1.278	3.677	3.094	3.289
UQ	17	59244001	59244300	1.135	3.272	2.724	2.802
UQ	17	59387101	59387400	0.455	0.118	0.132	0.056
UQ	17	59453401	59453700	0.532	0.305	0.341	0.218
UQ	17	59639401	59639700	3.797	2.691	2.455	2.890
UQ	17	59676901	59677200	1.202	0.163	0.111	0.117
UQ	17	59935801	59936100	0.240	1.171	0.560	0.679
UQ	17	60124201	60124500	1.221	0.862	0.564	0.664
UQ	17	60391201	60391500	1.862	2.218	1.518	2.216
UQ	17	60513301	60513600	0.584	0.328	0.346	0.385
UQ	17	60543301	60543600	0.218	1.081	1.099	0.902
UQ	17	60621601	60621900	0.666	0.350	0.317	0.335
UQ	17	60652201	60652500	0.637	0.328	0.399	0.441
UQ	17	60683101	60683400	0.186	0.421	0.531	0.304
UQ	17	60755701	60756000	1.039	1.586	1.741	1.668
UQ	17	62862901	62863200	0.640	1.669	0.054	1.424
UQ	17	63013501	63013800	0.744	0.399	0.379	0.360
UQ	17	63102901	63103200	2.107	1.081	0.881	1.227
UQ	17	63574501	63574800	1.192	0.331	0.082	0.364
UQ	17	63783001	63783300	0.584	0.330	0.350	0.304
UQ	17	64131601	64131900	1.302	1.054	0.560	0.573
UQ	17	64321201	64321500	2.885	1.528	0.852	1.506
UQ	17	64343401	64343700	1.254	0.680	0.477	0.568
UQ	17	64558801	64559100	0.561	0.120	0.049	0.162
UQ	17	64673401	64673700	0.690	0.378	0.346	0.223
UQ	17	64747801	64748100	3.773	2.506	3.019	2.728
UQ	17	64795201	64795500	3.675	2.553	2.722	2.494
UQ	17	64855801	64856100	0.666	1.073	0.234	1.044
UQ	17	64980001	64980300	2.278	1.307	0.996	1.242
UQ	17	65103601	65103900	1.699	1.053	1.066	0.837
UQ	17	65117401	65117700	0.505	0.305	0.317	0.218
UQ	17	65236801	65237100	6.072	3.463	4.447	4.299
UQ	17	65251201	65251500	4.167	2.358	2.575	1.688
UQ	17	65383501	65383800	5.858	3.149	2.710	3.392
UQ	17	65551801	65552100	1.673	1.257	0.638	1.131
UQ	17	65672701	65673000	2.554	2.038	1.716	1.014
UQ	17	65739001	65739300	0.450	1.009	0.827	0.857
UQ	17	65923501	65923800	2.454	4.182	3.418	3.758
UQ	17	66047401	66047700	2.284	1.253	1.246	0.927
UQ	17	66103801	66104100	0.558	0.375	0.263	0.223
UQ	17	66171901	66172200	6.077	3.884	4.000	3.615
UQ	17	66243601	66243900	0.371	0.539	0.531	0.162
UQ	17	66377101	66377400	2.944	1.457	1.462	1.364
UQ	17	66436801	66437100	1.063	0.628	0.152	0.431
UQ	17	66611701	66612000	3.167	4.392	4.407	4.020
UQ	17	66709801	66710100	0.557	0.328	0.317	0.279
UQ	17	66747301	66747600	2.758	1.047	2.151	1.602
UQ	17	66768901	66769200	3.464	1.961	2.004	2.536
UQ	17	66797401	66797700	0.876	1.854	1.592	1.359
UQ	17	66826201	66826500	1.116	0.656	0.740	0.567

UQ	17	66945001	66945300	2.020	1.191	1.358	1.410
UQ	17	66964801	66965100	1.116	1.335	0.720	0.913
UQ	17	67425601	67425900	0.558	0.161	0.111	0.248
UQ	17	67833301	67833600	0.558	0.702	0.395	0.745
UQ	17	67871701	67872000	0.079	0.468	0.234	0.634
UQ	17	68225701	68226000	0.398	0.678	0.662	0.715
UQ	17	68236201	68236500	0.799	0.935	0.662	0.608
UQ	17	68316601	68316900	2.020	2.780	2.889	2.900
UQ	17	68321401	68321700	4.233	3.161	3.123	2.906
UQ	17	68379901	68380200	1.491	1.002	0.724	0.801
UQ	17	68419201	68419500	0.613	2.152	0.876	0.745
UQ	17	68794501	68794800	4.862	3.639	3.768	2.854
UQ	17	68803801	68804100	1.882	0.253	1.324	1.247
UQ	17	68994601	68994900	2.310	1.448	1.242	1.800
UQ	17	69025501	69025800	3.317	1.678	1.784	2.023
UQ	17	69025801	69026100	1.858	1.330	1.193	1.521
UQ	17	69111601	69111900	0.926	0.184	0.301	0.299
UQ	17	69111901	69112200	0.582	0.116	0.189	0.167
UQ	17	69335701	69336000	0.616	0.187	0.082	0.173
UQ	17	69474001	69474300	1.034	0.517	0.481	0.507
UQ	17	69640201	69640500	1.889	1.266	1.567	2.124
UQ	17	69908401	69908700	1.887	0.886	1.259	1.364
UQ	17	70024801	70025100	1.541	1.847	0.662	1.181
UQ	17	70027501	70027800	1.594	0.633	0.613	0.927
UQ	17	70839301	70839600	2.124	2.927	3.266	2.891
UQ	17	71177101	71177400	0.908	0.305	0.457	0.243
UQ	17	71600101	71600400	0.876	0.184	0.325	0.578
UQ	17	72207601	72207900	1.915	1.197	1.217	1.430
UQ	17	72255301	72255600	0.452	0.163	0.560	0.142
UQ	18	1752901	1753200	0.316	0.560	0.029	0.025
UQ	18	1790101	1790400	3.938	2.999	3.270	2.637
UQ	18	1868701	1869000	0.507	0.725	0.239	0.355
UQ	18	1920001	1920300	2.396	1.070	1.316	1.511
UQ	18	3065701	3066000	0.477	0.212	0.239	0.218
UQ	18	3966601	3966900	1.170	0.373	0.609	0.771
UQ	18	4697401	4697700	1.200	0.583	0.745	0.720
UQ	18	5121901	5122200	5.472	4.628	3.277	3.736
UQ	18	5718601	5718900	3.052	1.942	2.143	2.236
UQ	18	5745901	5746200	2.176	1.150	1.340	1.293
UQ	18	5829901	5830200	0.347	0.749	0.736	0.745
UQ	18	5853901	5854200	0.186	0.397	0.740	0.335
UQ	18	6044701	6045000	0.265	0.940	0.667	0.837
UQ	18	6309901	6310200	0.957	0.378	0.473	0.421
UQ	18	6731701	6732000	0.826	0.444	0.473	0.461
UQ	18	6887401	6887700	0.744	0.283	0.252	0.279
UQ	18	6893401	6893700	1.645	0.725	0.814	1.050
UQ	18	6934801	6935100	1.250	0.751	0.831	0.644
UQ	18	6972901	6973200	0.905	0.399	0.531	0.659
UQ	18	7015201	7015500	0.668	0.375	1.003	0.329
UQ	18	7433701	7434000	1.386	1.968	0.827	2.003
UQ	18	7851301	7851600	0.666	0.350	0.346	0.522
UQ	18	7860001	7860300	5.586	3.890	3.908	4.933
UQ	18	8001301	8001600	0.505	0.092	0.078	0.051
UQ	18	8051101	8051400	0.321	0.468	0.107	0.137
UQ	18	8079901	8080200	0.477	0.305	0.156	0.000
UQ	18	9153001	9153300	1.463	0.918	0.736	0.720
UQ	18	9281101	9281400	0.477	0.260	0.025	0.112
UQ	18	9281701	9282000	0.266	0.446	0.477	0.106
UQ	18	9315601	9315900	1.063	0.633	0.740	0.771
UQ	18	9341101	9341400	0.611	0.489	0.160	0.223
UQ	18	9875401	9875700	1.294	0.120	0.152	0.279
UQ	18	9875701	9876000	1.426	0.000	0.025	0.112
UQ	18	10109101	10109400	2.712	1.731	1.913	2.043
UQ	18	10453501	10453800	1.644	0.862	1.041	0.690
UQ	18	10834201	10834500	0.345	0.536	0.740	0.634
UQ	18	11078701	11079000	0.452	0.609	0.263	0.218
UQ	18	11171101	11171400	1.063	2.669	1.839	1.775

UQ	18	11547601	11547900	1.460	0.935	1.094	0.918
UQ	18	11564401	11564700	0.850	0.513	0.535	0.608
UQ	18	11619901	11620200	0.450	0.258	0.239	0.223
UQ	18	11883301	11883600	2.955	2.384	2.356	1.947
UQ	18	12015901	12016200	0.477	0.116	0.296	0.279
UQ	18	12047101	12047400	0.982	0.699	0.671	1.242
UQ	18	12075001	12075300	0.689	0.957	2.361	1.323
UQ	18	12759901	12760200	0.287	0.862	0.901	1.384
UQ	18	12811201	12811500	1.199	0.633	0.374	0.279
UQ	18	12814501	12814800	4.223	3.484	3.188	2.195
UQ	18	12951301	12951600	2.981	2.497	2.530	1.886
UQ	18	13119301	13119600	0.881	2.032	1.320	1.668
UQ	18	13247401	13247700	3.591	4.224	4.191	4.122
UQ	18	13438801	13439100	1.008	0.628	0.720	0.248
UQ	18	13451701	13452000	1.539	1.008	0.827	0.963
UQ	18	13479301	13479600	0.213	0.376	0.584	0.720
UQ	18	13519801	13520100	4.438	5.430	3.739	5.141
UQ	18	13790701	13791000	2.502	1.611	1.264	1.100
UQ	18	13817401	13817700	1.037	0.543	0.419	0.198
UQ	18	13817701	13818000	0.982	0.356	0.303	0.248
UQ	18	14158201	14158500	0.871	0.260	0.243	0.198
UQ	18	14248801	14249100	0.503	0.257	0.267	0.243
UQ	18	14297701	14298000	1.226	0.725	0.575	0.776
UQ	18	14338201	14338500	1.758	1.056	0.794	1.019
UQ	18	15348001	15348300	2.179	1.802	1.743	1.242
UQ	18	15351901	15352200	0.665	0.375	0.321	0.385
UQ	18	15615301	15615600	6.117	3.536	4.839	4.654
UQ	18	15750601	15750900	1.113	0.539	0.539	0.538
UQ	18	16688701	16689000	0.105	0.541	0.506	0.533
UQ	18	16807801	16808100	0.692	0.513	0.448	0.472
UQ	18	16984501	16984800	0.481	0.210	0.234	0.167
UQ	18	18096601	18096900	1.470	0.448	0.490	0.543
UQ	18	18097201	18097500	1.650	0.800	1.025	0.603
UQ	18	18472501	18472800	0.670	0.189	0.054	0.117
UQ	18	18483901	18484200	0.747	1.923	1.324	1.161
UQ	18	18494701	18495000	0.611	0.491	0.341	0.472
UQ	18	18495001	18495300	1.674	0.888	1.398	1.161
UQ	18	19324201	19324500	0.266	0.513	0.399	0.167
UQ	18	19536001	19536300	1.568	0.957	0.852	1.323
UQ	18	19729201	19729500	0.613	0.187	0.127	0.117
UQ	18	19729501	19729800	0.561	0.208	0.127	0.000
UQ	18	19860001	19860300	0.026	0.539	0.263	0.198
UQ	18	19926001	19926300	2.265	1.614	1.440	1.638
UQ	18	20066701	20067000	1.594	0.794	1.128	1.125
UQ	18	20183701	20184000	1.278	0.910	1.061	0.806
UQ	18	20711401	20711700	0.955	0.517	0.477	0.634
UQ	18	20812201	20812500	0.477	0.045	0.132	0.142
UQ	18	20856601	20856900	1.008	1.710	1.427	1.354
UQ	18	20863501	20863800	0.639	0.213	0.185	0.304
UQ	18	20943001	20943300	0.318	0.444	0.132	0.086
UQ	18	21091201	21091500	0.771	0.493	1.119	0.497
UQ	18	21378901	21379200	0.932	1.167	1.596	1.516
UQ	18	21755701	21756000	3.297	2.667	4.196	4.311
UQ	18	21950101	21950400	0.503	0.303	0.156	0.031
UQ	18	21983101	21983400	1.278	0.684	0.399	0.614
UQ	18	22176601	22176900	1.916	1.592	1.308	1.024
UQ	18	22194001	22194300	3.773	3.459	4.492	5.004
UQ	18	23065501	23065800	1.037	0.725	0.716	0.751
UQ	18	23591101	23591400	1.091	1.566	1.514	1.840
UQ	18	24282301	24282600	0.905	0.560	0.531	0.684
UQ	18	24963001	24963300	1.724	1.094	1.181	2.236
UQ	18	25122001	25122300	0.477	0.375	0.214	0.193
UQ	18	25155901	25156200	1.855	0.914	0.941	0.446
UQ	18	26293201	26293500	1.015	0.449	0.395	0.416
UQ	18	27131101	27131400	0.905	0.425	0.584	0.527
UQ	18	27206101	27206400	0.771	0.444	0.165	0.411
UQ	18	27471601	27471900	1.353	1.077	0.876	0.999

UQ	18	27605101	27605400	0.534	0.167	0.132	0.157
UQ	18	27765901	27766200	0.452	0.586	0.321	0.248
UQ	18	27843601	27843900	0.507	0.212	0.049	0.254
UQ	18	27926101	27926400	1.142	0.635	0.580	0.542
UQ	18	28067401	28067700	1.381	0.543	0.642	0.907
UQ	18	28215601	28215900	0.532	0.350	0.185	0.441
UQ	18	28815601	28815900	0.374	0.772	0.638	0.695
UQ	18	29616301	29616600	1.516	0.841	0.876	1.111
UQ	18	29619001	29619300	0.661	0.258	0.267	0.051
UQ	18	29620201	29620500	1.329	1.472	0.876	1.024
UQ	18	30015601	30015900	0.534	0.140	0.214	0.198
UQ	18	30495601	30495900	0.189	0.560	0.535	0.472
UQ	18	30794101	30794400	0.186	0.305	0.560	0.578
UQ	18	30885601	30885900	0.398	0.232	0.613	0.274
UQ	18	31489501	31489800	2.312	0.667	0.918	0.582
UQ	18	31584601	31584900	1.173	0.702	0.477	0.695
UQ	18	31680901	31681200	1.299	0.590	0.419	0.695
UQ	18	32414101	32414400	0.373	0.656	0.156	0.218
UQ	18	32967301	32967600	0.505	0.305	0.346	0.248
UQ	18	33036601	33036900	0.663	1.120	1.168	1.156
UQ	18	33481201	33481500	0.532	0.257	0.243	0.162
UQ	18	34067101	34067400	0.884	2.815	2.450	3.315
UQ	18	34451101	34451400	0.455	0.094	0.160	0.081
UQ	18	34451401	34451700	0.450	0.167	0.185	0.167
UQ	18	35485201	35485500	0.560	0.212	0.374	0.162
UQ	18	37889401	37889700	0.586	0.139	0.321	0.385
UQ	18	38134801	38135100	1.808	1.242	0.749	1.091
UQ	18	38419801	38420100	0.160	0.420	0.399	0.472
UQ	18	38526601	38526900	0.132	0.442	0.321	0.695
UQ	18	39182101	39182400	0.823	0.448	0.502	0.162
UQ	18	41170801	41171100	0.665	0.185	1.645	1.152
UQ	18	41870701	41871000	0.957	0.444	0.629	0.416
UQ	18	42407101	42407400	0.823	0.420	0.288	0.634
UQ	18	42840001	42840300	1.200	0.699	0.667	0.690
UQ	18	42840301	42840600	0.507	0.258	0.185	0.218
UQ	18	43189801	43190100	0.292	0.843	0.506	0.472
UQ	18	43250101	43250400	1.121	1.959	2.044	1.811
UQ	18	44500201	44500500	0.558	0.916	0.823	1.186
UQ	18	44535901	44536200	0.586	0.328	0.156	0.355
UQ	18	45474601	45474900	0.213	0.491	0.453	0.329
UQ	18	47677201	47677500	0.292	0.420	0.078	0.193
UQ	18	47884501	47884800	0.773	0.350	0.415	0.162
UQ	18	50462701	50463000	1.199	0.910	0.740	0.628
UQ	18	52365001	52365300	0.531	0.047	0.029	0.076
UQ	18	54007801	54008100	0.824	0.465	0.395	0.436
UQ	18	54100501	54100800	2.446	3.107	2.102	2.657
UQ	18	54379501	54379800	1.091	0.678	0.337	0.542
UQ	18	54712501	54712800	4.344	3.087	2.871	5.815
UQ	18	54900001	54900300	0.269	0.629	0.749	0.639
UQ	18	55125001	55125300	0.982	1.968	1.901	1.958
UQ	18	55222201	55222500	1.487	1.006	0.457	0.486
UQ	18	56459401	56459700	0.689	0.300	0.185	0.279
UQ	18	56476801	56477100	2.021	1.849	2.151	1.770
UQ	18	56566201	56566500	1.120	2.313	2.047	2.561
UQ	18	56799301	56799600	2.742	1.944	1.966	2.150
UQ	18	57264001	57264300	0.450	0.257	0.214	0.193
UQ	18	57633901	57634200	0.744	1.545	1.081	1.521
UQ	18	57640501	57640800	0.477	0.281	0.370	0.248
UQ	18	57833401	57833700	1.381	0.963	0.745	1.080
UQ	18	57849601	57849900	1.970	1.008	1.074	0.973
UQ	18	58062601	58062900	0.931	0.418	0.638	0.639
UQ	18	58233001	58233300	0.610	0.161	0.189	0.182
UQ	18	58271401	58271700	1.991	1.270	0.925	1.354
UQ	18	58287301	58287600	0.187	0.444	0.292	0.279
UQ	18	58321801	58322100	1.386	0.511	0.359	0.588
UQ	18	58573801	58574100	1.257	0.446	0.395	0.187
UQ	18	58758301	58758600	5.211	4.328	4.249	3.898

UQ	18	59223301	59223600	0.371	0.583	0.905	0.527
UQ	18	59385601	59385900	0.532	0.140	0.321	0.228
UQ	18	59397901	59398200	3.084	2.176	2.566	2.185
UQ	18	59424601	59424900	2.638	0.639	0.745	0.964
UQ	18	59491201	59491500	2.263	0.862	1.044	1.035
UQ	18	59496301	59496600	0.986	0.539	0.160	0.132
UQ	18	59525401	59525700	1.382	1.049	0.667	0.893
UQ	18	59525701	59526000	1.226	0.487	0.439	0.583
UQ	18	59680201	59680500	1.592	1.053	0.950	0.720
UQ	18	59809501	59809800	3.715	1.609	1.407	2.479
UQ	18	60467101	60467400	0.079	0.446	0.317	0.193
UQ	18	60503101	60503400	0.426	0.257	0.267	0.522
UQ	18	61024801	61025100	4.104	5.988	5.036	6.627
UQ	18	61110601	61110900	0.931	0.442	0.379	0.695
UQ	18	61393201	61393500	0.345	0.818	0.555	0.664
UQ	18	61566301	61566600	0.398	0.772	0.584	0.695
UQ	18	61837501	61837800	0.821	0.330	0.399	0.380
UQ	18	62285701	62286000	0.560	0.071	0.214	0.259
UQ	18	62865601	62865900	1.065	0.491	0.546	0.452
UQ	18	62905801	62906100	2.346	1.564	0.987	1.348
UQ	18	62937601	62937900	2.507	1.362	1.282	1.206
UQ	18	62958301	62958600	1.197	0.796	1.353	0.907
UQ	18	62997301	62997600	0.558	0.140	0.160	0.355
UQ	18	63104101	63104400	0.321	0.680	0.580	0.720
UQ	18	63330001	63330300	1.704	0.745	0.872	1.206
UQ	18	63688801	63689100	1.944	0.841	0.872	0.978
UQ	18	63791401	63791700	0.800	0.068	0.136	0.239
UQ	18	63915001	63915300	0.318	0.609	0.580	0.603
UQ	18	63969601	63969900	1.197	0.770	0.544	0.355
UQ	18	64129801	64130100	3.212	2.047	1.835	2.014
UQ	18	64319101	64319400	2.471	1.877	1.587	1.831
UQ	18	64379701	64380000	1.008	0.586	0.424	0.751
UQ	18	64447801	64448100	1.626	1.143	1.090	0.846
UQ	18	64593601	64593900	0.589	0.234	0.239	0.248
UQ	18	64644901	64645200	2.688	3.133	2.189	2.277
UQ	18	64729201	64729500	0.663	0.234	0.234	0.147
UQ	18	64768501	64768800	1.039	1.405	0.617	0.614
UQ	18	64854901	64855200	2.612	1.512	1.723	1.723
UQ	18	65130001	65130300	0.690	0.513	0.477	0.446
UQ	18	65669701	65670000	0.879	0.234	0.181	0.360
UQ	18	65694601	65694900	2.418	1.358	1.415	1.319
UQ	18	65706901	65707200	8.079	5.862	5.900	6.348
UQ	18	65739601	65739900	1.254	0.635	0.823	0.826
UQ	18	66013501	66013800	0.639	1.077	1.242	0.907
UQ	18	66815101	66815400	1.170	2.264	1.716	1.967
UQ	18	66873601	66873900	0.795	0.092	0.419	0.198
UQ	18	66911401	66911700	1.733	1.026	1.110	0.887
UQ	18	66976801	66977100	0.823	1.077	0.531	0.664
UQ	18	67233001	67233300	0.079	0.466	0.341	0.391
UQ	18	67372501	67372800	2.544	1.487	0.847	1.287
UQ	18	67511401	67511700	0.637	0.094	0.370	0.279
UQ	18	67511701	67512000	0.826	0.168	0.477	0.248
UQ	18	67761901	67762200	3.193	2.086	4.068	2.850
UQ	18	68116801	68117100	0.926	0.300	0.272	0.350
UQ	18	68118301	68118600	1.382	0.701	0.691	0.817
UQ	18	68596501	68596800	0.692	0.234	0.205	0.411
UQ	19	11401	11700	18.472	13.157	11.984	12.179
UQ	19	834901	835200	1.037	1.453	0.798	0.690
UQ	19	1150201	1150500	1.408	1.049	1.723	0.963
UQ	19	1569901	1570200	0.665	0.376	0.506	0.218
UQ	19	1596001	1596300	2.748	0.605	0.863	0.710
UQ	19	1596301	1596600	0.879	0.562	0.432	0.411
UQ	19	1601401	1601700	5.269	1.575	0.421	1.158
UQ	19	1601701	1602000	1.358	0.397	0.087	0.501
UQ	19	1629901	1630200	1.700	0.581	0.510	0.487
UQ	19	1630201	1630500	7.417	4.415	4.538	4.406
UQ	19	1936201	1936500	2.293	0.521	0.457	0.452

UQ	19	1936501	1936800	1.996	0.536	0.760	0.964
UQ	19	2019601	2019900	0.186	0.723	0.716	0.674
UQ	19	3587401	3587700	0.582	0.305	0.189	0.304
UQ	19	3777001	3777300	0.690	0.421	0.399	0.385
UQ	19	3924001	3924300	0.531	0.187	0.185	0.248
UQ	19	4070401	4070700	1.194	0.794	0.847	0.781
UQ	19	5074201	5074500	1.144	0.772	0.486	0.720
UQ	19	5076901	5077200	1.702	1.028	1.045	0.882
UQ	19	5424601	5424900	0.637	0.375	0.140	0.360
UQ	19	5475601	5475900	0.505	0.140	0.243	0.187
UQ	19	5540401	5540700	0.771	0.442	0.403	0.477
UQ	19	5546401	5546700	0.213	0.468	0.078	0.355
UQ	19	5656201	5656500	0.769	0.309	0.218	0.248
UQ	19	5689801	5690100	0.106	0.583	0.292	0.279
UQ	19	5884501	5884800	1.991	1.101	1.086	1.658
UQ	19	6117601	6117900	5.551	3.154	3.953	4.630
UQ	19	6393601	6393900	0.079	0.328	0.395	0.664
UQ	19	6419401	6419700	0.586	0.144	0.288	0.112
UQ	19	6730801	6731100	1.728	0.886	1.152	1.151
UQ	19	6762901	6763200	2.862	1.496	1.389	1.064
UQ	19	6879601	6879900	0.398	0.165	0.560	0.137
UQ	19	7741201	7741500	0.265	0.515	0.560	0.771
UQ	19	8032201	8032500	1.118	0.910	0.905	0.913
UQ	19	8522401	8522700	1.299	0.380	0.432	0.538
UQ	19	8766901	8767200	0.637	0.908	0.901	0.329
UQ	19	9547801	9548100	0.692	0.513	0.428	0.497
UQ	19	9566101	9566400	1.942	1.543	1.616	1.683
UQ	19	10128001	10128300	1.654	0.989	1.003	0.614
UQ	19	10289101	10289400	0.773	0.305	0.428	0.583
UQ	19	10422601	10422900	0.692	0.423	0.263	0.421
UQ	19	10473001	10473300	0.531	0.350	0.103	0.061
UQ	19	10890001	10890300	0.694	0.277	0.247	0.218
UQ	19	11552701	11553000	2.902	3.810	3.571	3.960
UQ	19	11794201	11794500	3.984	2.965	2.827	3.382
UQ	19	12047101	12047400	6.033	4.864	8.037	7.184
UQ	19	12117301	12117600	0.558	0.232	0.160	0.112
UQ	19	12433801	12434100	0.560	0.303	0.263	0.355
UQ	19	12657301	12657600	6.617	5.205	4.610	4.396
UQ	19	13012501	13012800	0.586	0.281	0.453	0.664
UQ	19	13370101	13370400	1.676	1.217	1.144	1.039
UQ	19	13646401	13646700	0.878	0.678	0.584	0.776
UQ	19	14062801	14063100	1.224	1.586	1.596	1.465
UQ	19	14325001	14325300	1.195	0.562	0.745	0.735
UQ	19	14337301	14337600	1.326	3.755	2.796	3.874
UQ	19	14337601	14337900	0.345	1.873	0.798	2.083
UQ	19	14427001	14427300	7.491	5.248	5.332	6.423
UQ	19	14485801	14486100	6.459	4.046	4.428	4.330
UQ	19	14921401	14921700	3.078	1.944	2.047	1.886
UQ	19	14967301	14967600	0.240	0.633	0.580	0.745
UQ	19	15062701	15063000	1.115	0.583	0.457	0.603
UQ	19	15081601	15081900	6.926	4.759	4.970	3.042
UQ	19	15105601	15105900	0.239	1.244	0.682	1.131
UQ	19	15256501	15256800	2.260	0.770	1.465	1.439
UQ	19	15656701	15657000	1.037	0.560	0.852	0.715
UQ	19	16503601	16503900	2.849	1.961	1.864	1.916
UQ	19	16586101	16586400	0.347	0.751	0.662	0.690
UQ	19	17283001	17283300	2.183	1.796	1.757	1.739
UQ	19	17424901	17425200	1.115	0.867	0.667	0.497
UQ	19	17740201	17740500	1.305	0.837	0.638	0.446
UQ	19	17756401	17756700	1.568	1.148	0.720	1.014
UQ	19	17790001	17790300	0.905	1.195	0.745	1.105
UQ	19	17871001	17871300	0.531	0.305	0.185	0.355
UQ	19	18463201	18463500	0.507	0.935	1.135	0.776
UQ	19	18619801	18620100	0.265	0.584	0.560	0.553
UQ	19	18897901	18898200	0.747	0.375	0.082	0.472
UQ	19	19377901	19378200	2.720	1.202	0.970	1.009
UQ	19	19378201	19378500	3.309	0.951	0.591	0.812

UQ	19	19427701	19428000	0.610	0.257	0.272	0.228
UQ	19	19917301	19917600	0.507	0.283	0.267	0.193
UQ	19	21251101	21251400	1.036	0.560	0.468	0.690
UQ	19	21263701	21264000	0.797	0.470	0.424	0.446
UQ	19	21638101	21638400	1.037	0.725	0.428	0.690
UQ	19	21671401	21671700	0.318	0.729	0.827	0.695
UQ	19	21726601	21726900	0.081	0.513	0.502	0.274
UQ	19	23286301	23286600	0.610	0.373	0.350	0.360
UQ	19	24441301	24441600	0.450	0.023	0.136	0.061
UQ	19	24799801	24800100	2.633	2.055	1.810	2.200
UQ	19	25837801	25838100	2.844	1.306	1.748	1.221
UQ	19	26775001	26775300	0.902	0.588	0.560	0.472
UQ	19	26925601	26925900	1.654	0.650	0.441	0.405
UQ	19	27392701	27393000	0.560	0.981	0.370	1.166
UQ	19	27393001	27393300	0.637	0.935	0.341	0.852
UQ	19	27934201	27934500	1.226	0.375	0.517	0.132
UQ	19	27968401	27968700	0.586	0.236	0.267	0.248
UQ	19	28266301	28266600	3.689	2.707	2.566	2.753
UQ	19	28314001	28314300	1.357	1.541	1.016	0.360
UQ	19	28381201	28381500	1.144	0.770	0.629	0.781
UQ	19	28402801	28403100	0.397	0.607	0.214	0.553
UQ	19	28578901	28579200	0.586	0.442	0.317	0.801
UQ	19	29055601	29055900	1.249	0.890	0.651	1.825
UQ	19	29619301	29619600	1.170	1.380	1.061	0.994
UQ	19	29657401	29657700	2.210	1.594	1.157	1.303
UQ	19	29952301	29952600	2.236	1.127	1.444	1.308
UQ	19	30132601	30132900	0.423	0.142	0.107	0.137
UQ	19	30608101	30608400	2.980	2.047	1.692	1.932
UQ	19	31071901	31072200	0.611	0.421	0.453	0.162
UQ	19	31442101	31442400	0.845	0.094	0.103	0.218
UQ	19	31562701	31563000	0.769	0.393	0.350	0.248
UQ	19	32326801	32327100	0.505	0.167	0.132	0.187
UQ	19	32598901	32599200	0.532	0.328	0.288	0.329
UQ	19	32781001	32781300	8.286	6.211	6.231	6.663
UQ	19	33206701	33207000	0.266	0.466	0.054	0.162
UQ	19	33268501	33268800	0.395	0.792	0.054	0.771
UQ	19	33537301	33537600	0.424	0.983	0.979	0.927
UQ	19	33990001	33990300	0.292	0.584	0.481	0.106
UQ	19	34124701	34125000	0.665	1.096	1.324	1.485
UQ	19	34426501	34426800	1.436	0.302	0.321	0.304
UQ	19	35056501	35056800	1.516	1.708	1.119	1.359
UQ	19	35064601	35064900	0.878	0.562	0.745	0.614
UQ	19	35382001	35382300	0.981	1.285	0.633	0.553
UQ	19	35669701	35670000	0.878	1.120	1.456	1.247
UQ	19	35977201	35977500	0.558	0.236	0.234	0.187
UQ	19	37480801	37481100	4.620	2.307	3.192	2.982
UQ	19	38428501	38428800	0.852	0.442	0.383	0.365
UQ	19	38833201	38833500	0.426	0.584	0.317	0.248
UQ	19	39127501	39127800	0.558	0.397	0.453	0.360
UQ	19	39353701	39354000	1.458	0.824	0.740	0.846
UQ	19	39421501	39421800	1.563	2.592	2.283	2.306
UQ	19	39814801	39815100	0.477	0.163	0.321	0.218
UQ	19	40142701	40143000	1.594	0.706	0.852	0.517
UQ	19	40268701	40269000	0.636	1.047	1.119	1.131
UQ	19	40361401	40361700	0.477	0.633	0.160	0.274
UQ	19	40812001	40812300	0.560	0.023	0.288	0.248
UQ	19	41423701	41424000	0.400	0.586	0.613	0.578
UQ	19	41458501	41458800	1.760	1.118	2.682	3.189
UQ	19	41953201	41953500	0.823	0.281	0.292	0.360
UQ	19	42231301	42231600	0.000	0.772	0.658	0.619
UQ	19	42333001	42333300	0.744	0.539	0.428	0.441
UQ	19	42573001	42573300	0.685	0.071	0.000	0.092
UQ	19	42700801	42701100	0.984	0.395	0.424	0.603
UQ	19	43050301	43050600	1.329	0.862	0.745	0.608
UQ	19	43831501	43831800	2.061	0.734	0.444	0.396
UQ	19	43831801	43832100	2.408	0.936	0.716	0.482
UQ	19	43892701	43893000	0.503	0.184	0.127	0.167

UQ	19	44045701	44046000	0.824	0.328	0.448	0.614
UQ	19	44126101	44126400	0.184	0.680	0.424	0.391
UQ	19	44212801	44213100	0.477	0.120	0.189	0.167
UQ	19	44757301	44757600	0.481	0.234	0.189	0.167
UQ	19	44797201	44797500	1.360	0.955	0.720	0.477
UQ	19	44855701	44856000	0.744	0.350	0.526	0.522
UQ	19	45040501	45040800	1.036	0.421	0.457	0.477
UQ	19	45040801	45041100	1.516	0.729	0.802	0.679
UQ	19	45273601	45273900	0.532	1.264	1.736	1.140
UQ	19	45321301	45321600	1.836	0.657	0.223	1.091
UQ	19	45551101	45551400	0.613	0.307	0.292	0.279
UQ	19	45594301	45594600	0.345	0.491	0.584	0.198
UQ	19	45841801	45842100	1.805	0.215	1.110	0.614
UQ	19	46110001	46110300	0.586	0.238	0.185	0.279
UQ	19	46661101	46661400	0.642	0.234	0.185	0.187
UQ	19	46661401	46661700	2.004	0.620	0.312	0.283
UQ	19	46693801	46694100	0.905	1.384	1.246	1.328
UQ	19	47073901	47074200	4.202	4.885	3.961	3.752
UQ	19	47187601	47187900	1.489	1.004	0.930	1.100
UQ	19	47193001	47193300	0.744	0.397	0.395	0.421
UQ	19	47617801	47618100	1.034	0.350	0.203	0.365
UQ	19	47715901	47716200	0.687	0.024	0.025	0.182
UQ	19	47808301	47808600	0.319	0.446	0.185	0.167
UQ	19	48127801	48128100	2.183	1.403	1.641	1.354
UQ	19	48604201	48604500	0.558	0.167	0.185	0.268
UQ	19	48814501	48814800	3.613	2.736	2.838	2.159
UQ	19	49233001	49233300	0.160	0.446	0.346	0.304
UQ	19	49244101	49244400	2.480	0.976	1.271	1.577
UQ	19	49244401	49244700	9.923	4.483	6.113	6.412
UQ	19	49348801	49349100	4.785	3.815	4.187	5.440
UQ	19	49388101	49388400	2.021	1.448	1.008	0.918
UQ	19	50040901	50041200	0.769	0.302	0.374	0.391
UQ	19	50383801	50384100	2.100	1.313	1.567	1.658
UQ	19	50781601	50781900	0.826	0.489	0.374	0.492
UQ	19	51115201	51115500	0.532	0.281	0.374	0.198
UQ	19	51188101	51188400	4.048	1.768	2.430	2.622
UQ	19	51255001	51255300	2.152	1.637	1.962	1.577
UQ	19	51260701	51261000	1.034	1.922	1.641	1.526
UQ	19	51303301	51303600	0.848	0.212	0.205	0.137
UQ	19	51303601	51303900	0.476	0.049	0.049	0.000
UQ	19	51312601	51312900	3.246	2.776	2.135	2.758
UQ	19	51542701	51543000	0.477	0.989	1.061	1.029
UQ	19	52035601	52035900	0.106	0.515	0.317	0.441
UQ	19	52188001	52188300	2.470	1.776	1.946	2.155
UQ	19	52236001	52236300	0.718	0.399	0.317	0.421
UQ	19	52312201	52312500	1.733	1.011	0.802	1.125
UQ	19	52532701	52533000	0.079	0.446	0.292	0.254
UQ	19	53146501	53146800	1.091	0.772	0.510	0.472
UQ	19	53278201	53278500	0.928	1.564	2.341	1.328
UQ	19	53327101	53327400	0.636	0.303	0.341	0.329
UQ	19	53496601	53496900	0.000	0.444	0.107	0.162
UQ	19	53617501	53617800	2.100	1.706	1.494	0.974
UQ	19	53913601	53913900	1.462	2.382	2.387	2.495
UQ	19	54006001	54006300	0.611	0.867	0.317	0.309
UQ	19	54049801	54050100	2.234	3.115	2.916	3.083
UQ	19	54202801	54203100	2.207	1.867	1.547	1.613
UQ	19	54268801	54269100	3.113	2.034	2.129	1.877
UQ	19	54297601	54297900	0.290	1.523	0.707	0.720
UQ	19	54298501	54298800	1.511	0.663	0.551	0.461
UQ	19	54309601	54309900	0.266	0.586	0.448	0.634
UQ	19	54420601	54420900	0.878	0.515	0.745	0.329
UQ	19	54530101	54530400	1.037	0.163	0.341	0.370
UQ	19	54740701	54741000	3.964	3.364	4.648	2.961
UQ	19	54988801	54989100	4.736	3.602	3.904	5.475
UQ	19	55176901	55177200	0.742	0.397	0.296	0.112
UQ	19	55242001	55242300	3.507	2.251	1.313	2.124
UQ	19	55662001	55662300	0.081	0.472	0.609	0.862

UQ	19	55662301	55662600	0.110	1.126	1.142	1.764
UQ	19	55711201	55711500	1.224	0.397	0.412	0.344
UQ	19	56224501	56224800	0.848	0.311	0.127	0.254
UQ	19	56314801	56315100	2.468	1.382	1.810	1.754
UQ	19	56631301	56631600	0.586	0.258	0.181	0.157
UQ	19	57050401	57050700	1.908	1.216	1.436	0.751
UQ	19	57137401	57137700	0.132	0.515	0.555	0.385
UQ	19	57270901	57271200	0.852	0.449	0.403	0.304
UQ	19	57570601	57570900	0.586	0.466	0.453	0.335
UQ	19	57657301	57657600	0.745	0.539	0.424	0.466
UQ	19	57848101	57848400	1.328	0.747	0.729	0.812
UQ	19	58135201	58135500	2.420	2.010	2.225	2.850
UQ	19	58143001	58143300	4.243	2.740	2.338	2.702
UQ	19	58143301	58143600	3.623	2.508	1.955	2.474
UQ	19	58146901	58147200	1.891	1.352	1.456	1.460
UQ	19	58191001	58191300	1.334	0.532	0.252	0.452
UQ	19	58533301	58533600	1.808	3.484	3.233	3.616
UQ	19	58640401	58640700	2.654	1.465	1.679	1.471
UQ	19	58831201	58831500	3.407	2.186	1.826	1.927
UQ	19	58948801	58949100	0.534	0.818	0.847	1.364
UQ	19	59294401	59294700	1.042	2.946	4.200	4.431
UQ	19	59314501	59314800	2.260	2.736	1.601	2.652
UQ	19	59433901	59434200	6.806	5.801	5.772	8.149
UQ	19	59503801	59504100	1.568	0.846	0.736	1.136
UQ	19	59694001	59694300	4.340	6.366	5.511	5.126
UQ	19	59879101	59879400	1.800	0.888	0.671	1.009
UQ	19	59961901	59962200	2.099	1.495	1.387	1.593
UQ	19	60009001	60009300	1.489	1.963	1.917	1.886
UQ	19	60114301	60114600	2.818	1.738	1.913	0.978
UQ	19	60222001	60222300	1.925	0.116	0.590	0.173
UQ	2	9601	9900	0.690	0.536	0.876	0.472
UQ	2	68101	68400	0.532	0.841	0.638	0.715
UQ	2	101401	101700	0.640	0.305	0.243	0.223
UQ	2	146401	146700	2.584	1.326	2.049	1.871
UQ	2	222901	223200	2.526	1.292	1.362	2.094
UQ	2	393301	393600	2.578	1.513	2.089	1.633
UQ	2	519001	519300	0.268	0.562	0.662	0.634
UQ	2	531901	532200	2.313	1.826	1.665	1.784
UQ	2	564901	565200	9.711	11.161	8.946	12.970
UQ	2	716701	717000	2.323	1.009	0.695	1.030
UQ	2	870901	871200	0.666	0.350	0.181	0.335
UQ	2	1011001	1011300	4.552	2.764	2.287	3.015
UQ	2	1093201	1093500	2.392	1.944	1.942	1.784
UQ	2	1179001	1179300	3.244	2.098	2.798	3.681
UQ	2	1210801	1211100	0.634	1.588	1.551	1.546
UQ	2	1435801	1436100	2.070	1.139	1.108	1.181
UQ	2	1555801	1556100	0.957	0.560	0.473	0.486
UQ	2	1589701	1590000	9.615	6.797	6.038	6.841
UQ	2	1738801	1739100	7.187	5.621	6.224	6.049
UQ	2	1916101	1916400	3.557	2.311	2.513	2.783
UQ	2	1962001	1962300	2.339	1.468	1.616	1.881
UQ	2	2019601	2019900	3.725	2.825	2.698	3.230
UQ	2	2137801	2138100	0.531	0.305	0.317	0.360
UQ	2	2667601	2667900	1.144	0.869	0.473	0.441
UQ	2	2694901	2695200	0.450	0.142	0.054	0.117
UQ	2	2719201	2719500	1.458	0.369	0.303	0.588
UQ	2	3109801	3110100	2.538	1.200	1.324	0.816
UQ	2	3132901	3133200	1.867	0.845	1.052	0.735
UQ	2	3146701	3147000	5.877	3.205	3.168	3.812
UQ	2	3148801	3149100	1.363	2.466	2.880	2.505
UQ	2	3155401	3155700	0.558	0.163	0.189	0.147
UQ	2	3163201	3163500	3.792	1.433	1.551	1.318
UQ	2	3233101	3233400	0.534	0.073	0.165	0.223
UQ	2	3376501	3376800	1.941	1.478	1.415	1.546
UQ	2	3384001	3384300	0.879	0.562	0.267	0.416
UQ	2	3403801	3404100	1.224	0.869	0.662	0.938
UQ	2	3458701	3459000	1.515	1.122	1.280	0.974

UQ	2	3469501	3469800	0.640	0.161	0.210	0.279
UQ	2	3482401	3482700	0.534	0.234	0.288	0.162
UQ	2	3642601	3642900	1.486	0.723	0.633	0.893
UQ	2	3692701	3693000	0.982	1.332	0.740	1.267
UQ	2	4200901	4201200	1.966	2.407	2.122	2.292
UQ	2	5003401	5003700	0.586	0.796	0.214	0.243
UQ	2	5053501	5053800	1.065	0.725	0.539	0.350
UQ	2	5334001	5334300	0.131	0.584	0.609	0.502
UQ	2	5602801	5603100	0.505	0.140	0.185	0.274
UQ	2	5709601	5709900	1.594	1.212	1.888	1.303
UQ	2	6094501	6094800	0.721	0.092	0.054	0.101
UQ	2	6094801	6095100	1.046	0.047	0.189	0.263
UQ	2	6099001	6099300	0.052	0.964	0.876	0.861
UQ	2	6235501	6235800	1.011	0.517	0.424	0.385
UQ	2	6241501	6241800	6.009	9.442	8.961	9.095
UQ	2	6348901	6349200	0.507	0.142	0.185	0.051
UQ	2	6350101	6350400	3.515	1.695	1.984	2.068
UQ	2	6382501	6382800	2.810	9.787	12.171	9.224
UQ	2	6382801	6383100	0.371	2.300	2.757	2.283
UQ	2	6394801	6395100	0.292	0.444	0.448	0.472
UQ	2	6579001	6579300	1.252	2.176	1.815	1.926
UQ	2	6678901	6679200	0.106	0.303	0.263	0.497
UQ	2	6779101	6779400	1.137	0.354	0.399	0.502
UQ	2	6884101	6884400	0.663	0.161	0.239	0.162
UQ	2	6884401	6884700	1.254	0.489	0.526	0.573
UQ	2	7036501	7036800	4.554	2.808	3.435	3.726
UQ	2	7354201	7354500	1.889	0.961	0.902	0.720
UQ	2	7446301	7446600	0.558	0.049	0.243	0.279
UQ	2	7539601	7539900	0.639	1.172	1.164	1.253
UQ	2	7709101	7709400	0.608	0.189	0.025	0.117
UQ	2	8081701	8082000	5.078	3.991	4.189	3.752
UQ	2	8105401	8105700	0.721	0.210	0.189	0.304
UQ	2	8268001	8268300	0.532	0.283	0.263	0.254
UQ	2	8319001	8319300	0.345	0.515	0.560	0.720
UQ	2	8751601	8751900	1.571	1.081	0.794	0.867
UQ	2	8832301	8832600	0.905	0.586	0.370	0.350
UQ	2	9120601	9120900	1.279	1.824	1.538	1.800
UQ	2	9260401	9260700	6.222	5.518	4.846	5.764
UQ	2	9352801	9353100	0.640	0.305	0.243	0.213
UQ	2	9495901	9496200	1.254	0.727	0.613	0.801
UQ	2	9747601	9747900	1.920	1.290	0.914	1.303
UQ	2	10356901	10357200	0.453	0.772	0.691	0.695
UQ	2	11004601	11004900	0.292	0.560	0.107	0.360
UQ	2	11298001	11298300	1.963	0.983	1.045	1.161
UQ	2	11383501	11383800	1.625	1.234	1.037	0.765
UQ	2	11461801	11462100	0.477	0.633	0.185	0.664
UQ	2	12052201	12052500	1.336	0.429	0.140	0.198
UQ	2	12082501	12082800	1.176	0.466	0.272	0.492
UQ	2	12863401	12863700	0.398	0.560	0.239	0.223
UQ	2	12963001	12963300	4.017	3.272	2.793	2.429
UQ	2	13090501	13090800	1.010	0.704	0.642	0.720
UQ	2	13126801	13127100	0.397	0.633	0.160	0.193
UQ	2	13156201	13156500	0.584	1.054	0.905	0.958
UQ	2	13387501	13387800	0.610	0.073	0.160	0.167
UQ	2	13436701	13437000	0.240	0.444	0.424	0.583
UQ	2	13710901	13711200	0.027	0.751	0.609	0.304
UQ	2	14151301	14151600	0.532	0.770	0.341	0.695
UQ	2	14374201	14374500	1.087	0.281	0.383	0.335
UQ	2	14439001	14439300	0.932	0.631	0.535	0.396
UQ	2	14594101	14594400	0.345	0.560	0.140	0.137
UQ	2	15255601	15255900	0.957	0.163	0.185	0.223
UQ	2	15343801	15344100	0.292	0.701	0.555	0.588
UQ	2	15678301	15678600	0.534	0.328	0.267	0.329
UQ	2	15724801	15725100	3.138	2.521	2.744	2.621
UQ	2	16096801	16097100	0.876	1.075	0.560	1.166
UQ	2	16458601	16458900	0.532	0.303	0.288	0.142
UQ	2	16929001	16929300	2.042	1.223	1.255	0.887

UQ	2	17227201	17227500	0.292	0.493	0.584	0.137
UQ	2	17418901	17419200	1.462	1.777	1.168	1.298
UQ	2	17746501	17746800	0.160	0.446	0.444	0.446
UQ	2	18081601	18081900	0.689	0.324	0.263	0.304
UQ	2	18513001	18513300	0.665	0.395	0.366	0.193
UQ	2	19064101	19064400	1.271	0.749	0.682	0.664
UQ	2	20024101	20024400	2.794	1.399	2.127	2.074
UQ	2	20591401	20591700	0.106	0.446	0.317	0.385
UQ	2	20600701	20601000	0.452	0.326	0.638	0.751
UQ	2	21502801	21503100	1.460	1.777	1.723	2.185
UQ	2	21508201	21508500	0.211	0.702	0.580	0.654
UQ	2	21522901	21523200	2.128	1.594	1.407	1.532
UQ	2	22833901	22834200	0.637	0.184	0.267	0.360
UQ	2	22911601	22911900	0.373	0.068	0.609	0.634
UQ	2	23013301	23013600	0.289	0.770	0.860	0.776
UQ	2	23081401	23081700	0.532	0.328	0.272	0.248
UQ	2	24285901	24286200	2.437	1.157	1.164	1.298
UQ	2	24299101	24299400	1.386	0.891	0.682	0.852
UQ	2	25215301	25215600	0.932	0.678	0.564	0.553
UQ	2	25238101	25238400	0.768	0.281	0.337	0.274
UQ	2	25506301	25506600	0.800	0.444	0.239	0.365
UQ	2	26182201	26182500	1.037	1.478	1.333	1.937
UQ	2	26328601	26328900	0.769	0.395	0.218	0.497
UQ	2	26600701	26601000	1.252	0.886	0.564	0.821
UQ	2	26796601	26796900	0.481	0.236	0.185	0.162
UQ	2	26847901	26848200	0.345	0.633	0.823	0.223
UQ	2	27067201	27067500	2.184	1.526	1.616	1.607
UQ	2	27492601	27492900	0.774	0.258	0.366	0.223
UQ	2	27593401	27593700	0.186	0.607	0.374	0.385
UQ	2	27916801	27917100	0.319	0.444	0.239	0.360
UQ	2	27931801	27932100	0.132	0.187	0.239	0.608
UQ	2	27961501	27961800	1.037	0.723	0.395	0.659
UQ	2	28032901	28033200	0.663	0.983	0.230	0.274
UQ	2	28230601	28230900	0.529	0.071	0.214	0.142
UQ	2	28516801	28517100	1.061	0.448	0.350	0.380
UQ	2	28563901	28564200	0.608	0.090	0.049	0.187
UQ	2	29948401	29948700	0.582	0.305	0.160	0.329
UQ	2	31066801	31067100	1.463	0.961	1.070	0.893
UQ	2	31586701	31587000	0.213	0.468	0.346	0.355
UQ	2	32444701	32445000	0.186	0.418	0.613	0.695
UQ	2	32563201	32563500	0.424	0.723	0.852	0.664
UQ	2	32654701	32655000	0.557	0.234	0.259	0.106
UQ	2	33387301	33387600	0.476	0.047	0.078	0.106
UQ	2	33678601	33678900	0.666	0.206	0.058	0.000
UQ	2	34475401	34475700	2.339	1.845	1.476	1.253
UQ	2	36690001	36690300	0.558	0.912	0.745	0.360
UQ	2	36838201	36838500	2.207	1.540	1.480	1.582
UQ	2	36850201	36850500	2.126	1.686	1.184	1.566
UQ	2	36964201	36964500	1.254	2.395	2.374	2.515
UQ	2	37432201	37432500	0.529	0.163	0.181	0.193
UQ	2	37545301	37545600	1.284	0.611	0.506	0.558
UQ	2	38687101	38687400	0.931	0.560	1.349	1.222
UQ	2	39121501	39121800	0.106	0.350	0.526	0.421
UQ	2	39257101	39257400	0.345	0.560	0.662	0.497
UQ	2	39727801	39728100	0.637	0.348	0.292	0.335
UQ	2	40824601	40824900	2.918	1.011	1.978	0.882
UQ	2	41013001	41013300	0.665	0.303	0.370	0.391
UQ	2	41013301	41013600	0.721	0.330	0.263	0.299
UQ	2	41103901	41104200	0.507	0.185	0.210	0.360
UQ	2	41174401	41174700	0.823	0.466	0.390	0.497
UQ	2	42172201	42172500	0.507	0.208	0.074	0.112
UQ	2	43299601	43299900	0.481	0.142	0.098	0.142
UQ	2	43356301	43356600	1.249	0.701	1.008	1.044
UQ	2	43584001	43584300	1.007	0.564	0.502	0.466
UQ	2	44142601	44142900	1.269	0.305	0.370	0.481
UQ	2	44277601	44277900	0.294	0.699	0.609	0.527
UQ	2	44964901	44965200	0.561	0.187	0.218	0.218

UQ	2	45292501	45292800	1.563	0.918	0.428	0.477
UQ	2	45504301	45504600	0.881	0.468	0.288	0.497
UQ	2	45504601	45504900	0.903	0.517	0.399	0.628
UQ	2	45706201	45706500	0.452	0.772	0.638	0.497
UQ	2	45783901	45784200	0.505	0.069	0.239	0.173
UQ	2	45834601	45834900	1.436	0.356	0.749	0.792
UQ	2	45834901	45835200	1.331	0.257	0.633	0.705
UQ	2	46043401	46043700	1.676	2.476	2.352	2.500
UQ	2	46301101	46301400	0.984	1.283	0.432	0.527
UQ	2	46820101	46820400	0.319	0.421	0.555	0.553
UQ	2	47172601	47172900	0.053	0.468	0.292	0.279
UQ	2	47678701	47679000	0.453	0.910	0.633	0.832
UQ	2	47698501	47698800	1.120	1.450	0.617	1.709
UQ	2	48620101	48620400	2.678	1.588	1.843	1.425
UQ	2	48733201	48733500	0.479	0.631	0.263	0.608
UQ	2	48740101	48740400	0.211	0.397	0.506	0.360
UQ	2	48843301	48843600	0.477	0.212	0.058	0.187
UQ	2	49075501	49075800	1.252	0.603	0.658	0.659
UQ	2	49107601	49107900	2.423	1.914	1.806	1.876
UQ	2	49150801	49151100	4.414	3.454	2.893	3.412
UQ	2	49467601	49467900	0.373	0.586	0.638	0.248
UQ	2	49917001	49917300	0.319	0.185	0.531	0.441
UQ	2	50730601	50730900	0.532	0.212	0.317	0.086
UQ	2	50825101	50825400	0.663	0.208	0.374	0.193
UQ	2	51807901	51808200	0.718	0.631	0.374	0.274
UQ	2	51937201	51937500	0.850	0.230	0.453	0.294
UQ	2	53004301	53004600	0.611	1.384	0.950	0.938
UQ	2	54268501	54268800	0.450	0.096	0.214	0.137
UQ	2	55431601	55431900	0.426	0.749	1.028	0.994
UQ	2	56694901	56695200	4.522	2.624	2.747	3.519
UQ	2	56708401	56708700	1.542	1.071	1.037	1.024
UQ	2	57202201	57202500	0.744	0.165	0.254	0.198
UQ	2	57952201	57952500	0.768	0.049	0.123	0.167
UQ	2	58068601	58068900	6.057	7.192	4.641	5.192
UQ	2	58299301	58299600	1.623	0.680	0.560	0.695
UQ	2	58381201	58381500	0.799	1.028	0.609	0.385
UQ	2	58412101	58412400	0.507	0.165	0.049	0.279
UQ	2	59476801	59477100	0.744	0.539	0.263	0.142
UQ	2	59845801	59846100	0.450	0.210	0.136	0.162
UQ	2	59913601	59913900	1.089	0.702	0.584	0.644
UQ	2	59913901	59914200	1.199	0.560	0.638	0.715
UQ	2	60065101	60065400	0.555	0.165	0.029	0.223
UQ	2	60207301	60207600	0.663	0.302	0.181	0.309
UQ	2	60631201	60631500	0.106	0.420	0.424	0.340
UQ	2	61027201	61027500	0.586	0.116	0.234	0.243
UQ	2	61551301	61551600	1.036	0.534	0.604	0.502
UQ	2	61712101	61712400	0.953	0.236	0.379	0.228
UQ	2	62569501	62569800	1.728	1.289	1.378	1.374
UQ	2	63241201	63241500	0.586	0.397	0.267	0.411
UQ	2	63626401	63626700	0.823	1.424	0.481	1.156
UQ	2	63921601	63921900	0.852	0.515	0.691	0.634
UQ	2	64858501	64858800	0.161	0.326	0.346	0.497
UQ	2	65750701	65751000	0.639	0.820	0.403	0.416
UQ	2	66498301	66498600	0.718	0.326	0.317	0.274
UQ	2	67852201	67852500	2.016	2.783	2.628	2.677
UQ	2	67930201	67930500	1.481	0.294	0.000	0.558
UQ	2	67948501	67948800	3.717	2.686	2.037	2.697
UQ	2	67952701	67953000	0.907	1.429	1.719	1.861
UQ	2	68445901	68446200	0.560	0.727	0.185	0.360
UQ	2	68636101	68636400	0.081	0.607	0.346	0.405
UQ	2	68748001	68748300	2.902	1.680	1.686	1.623
UQ	2	68766301	68766600	1.086	0.187	0.510	0.411
UQ	2	68890201	68890500	0.000	0.420	0.189	0.167
UQ	2	69580501	69580800	1.171	0.399	0.546	0.405
UQ	2	70068301	70068600	0.481	0.140	0.136	0.193
UQ	2	70085101	70085400	5.027	3.789	3.696	3.919
UQ	2	70348801	70349100	1.466	0.935	0.736	0.887

UQ	2	70631401	70631700	0.692	0.303	0.374	0.173
UQ	2	70633201	70633500	0.957	0.629	0.613	0.583
UQ	2	70643401	70643700	0.457	0.024	0.054	0.056
UQ	2	70703101	70703400	0.532	0.165	0.185	0.360
UQ	2	70834801	70835100	3.964	2.845	3.097	2.601
UQ	2	71448901	71449200	0.184	0.468	0.424	0.603
UQ	2	71509501	71509800	0.589	0.187	0.078	0.167
UQ	2	74184901	74185200	0.637	0.303	0.214	0.162
UQ	2	75540601	75540900	0.106	0.493	0.243	0.248
UQ	2	76176001	76176300	0.450	0.023	0.025	0.132
UQ	2	78552001	78552300	0.692	0.163	0.506	0.583
UQ	2	79921801	79922100	0.797	0.560	0.526	0.355
UQ	2	80047801	80048100	0.850	0.558	0.667	0.304
UQ	2	81045001	81045300	0.527	0.094	0.136	0.081
UQ	2	81079201	81079500	1.140	0.584	0.769	0.765
UQ	2	81213001	81213300	0.981	0.564	0.613	0.203
UQ	2	81401401	81401700	1.008	0.397	0.544	0.583
UQ	2	81727501	81727800	0.292	0.980	0.584	0.695
UQ	2	82589101	82589400	0.452	0.234	0.667	0.715
UQ	2	82875901	82876200	0.610	0.352	0.374	0.304
UQ	2	83702401	83702700	0.503	0.234	0.243	0.162
UQ	2	84095401	84095700	0.905	0.448	0.531	0.639
UQ	2	84176701	84177000	0.690	0.423	0.448	0.416
UQ	2	84554401	84554700	4.070	3.092	2.706	2.859
UQ	2	84670201	84670500	0.663	0.331	0.263	0.335
UQ	2	85223701	85224000	0.503	0.210	0.210	0.162
UQ	2	85704601	85704900	1.704	2.381	2.506	2.074
UQ	2	85716901	85717200	1.329	0.796	0.774	0.654
UQ	2	86460301	86460600	7.503	6.091	10.313	6.039
UQ	2	86736301	86736600	1.384	0.674	0.823	0.882
UQ	2	86837401	86837700	2.925	2.592	2.365	2.180
UQ	2	87016201	87016500	0.636	0.139	0.132	0.299
UQ	2	87110701	87111000	0.429	1.049	1.008	0.979
UQ	2	87396301	87396600	0.265	0.069	0.428	0.578
UQ	2	87965101	87965400	0.371	0.818	0.691	0.522
UQ	2	88085701	88086000	1.302	0.657	0.560	0.785
UQ	2	88116001	88116300	1.355	0.772	0.807	0.765
UQ	2	88293901	88294200	0.190	1.006	0.609	0.634
UQ	2	88368601	88368900	0.160	0.444	0.029	0.360
UQ	2	88375201	88375500	0.266	0.444	0.428	0.472
UQ	2	88838101	88838400	0.694	0.468	0.321	0.416
UQ	2	88870801	88871100	0.452	0.629	0.317	0.304
UQ	2	88878901	88879200	0.905	0.558	0.185	0.416
UQ	2	89192701	89193000	0.769	0.489	0.292	0.466
UQ	2	89254201	89254500	0.663	1.124	0.930	1.029
UQ	2	91228201	91228500	2.634	2.004	1.830	1.755
UQ	2	91336201	91336500	0.532	0.283	0.054	0.360
UQ	2	91672801	91673100	1.705	1.028	0.802	0.791
UQ	2	92686201	92686500	0.505	0.234	0.374	0.360
UQ	2	92900701	92901000	1.355	0.837	0.651	0.806
UQ	2	93106801	93107100	0.477	0.047	0.160	0.081
UQ	2	93170701	93171000	0.928	0.277	0.127	0.106
UQ	2	93637201	93637500	0.611	0.350	0.317	0.228
UQ	2	94216801	94217100	0.745	0.236	0.370	0.315
UQ	2	94217101	94217400	0.850	0.330	0.477	0.370
UQ	2	94353001	94353300	0.586	0.935	1.003	0.968
UQ	2	94499101	94499400	0.850	1.122	0.239	0.431
UQ	2	95122801	95123100	1.142	0.678	0.687	0.684
UQ	2	95171701	95172000	0.268	0.468	0.453	0.522
UQ	2	95379601	95379900	0.799	0.493	0.346	0.436
UQ	2	96024001	96024300	0.503	0.258	0.234	0.167
UQ	2	96773701	96774000	0.718	0.420	0.395	0.446
UQ	2	97949401	97949700	1.410	0.886	0.823	1.125
UQ	2	99694801	99695100	1.173	0.560	0.379	0.664
UQ	2	100420801	100421100	1.436	0.916	1.037	1.111
UQ	2	100613101	100613400	0.055	0.491	0.609	0.299
UQ	2	100702801	100703100	0.718	0.420	0.341	0.304

UQ	2	100838401	100838700	1.171	1.641	1.547	1.688
UQ	2	101123401	101123700	0.477	0.796	0.745	0.329
UQ	2	101297101	101297400	3.865	4.652	3.130	3.179
UQ	2	101444101	101444400	1.410	1.053	1.099	1.080
UQ	2	101516401	101516700	0.850	0.371	0.379	0.274
UQ	2	101604901	101605200	4.022	2.980	2.508	2.769
UQ	2	101861701	101862000	0.692	0.375	0.390	0.329
UQ	2	102056401	102056700	1.252	0.534	0.473	0.254
UQ	2	102086101	102086400	0.503	0.210	0.189	0.243
UQ	2	102123601	102123900	0.744	0.137	0.087	0.223
UQ	2	102502801	102503100	0.821	0.303	0.453	0.472
UQ	2	102740701	102741000	0.505	0.302	0.263	0.248
UQ	2	102997201	102997500	1.652	0.616	0.448	0.405
UQ	2	102997501	102997800	0.769	0.234	0.296	0.152
UQ	2	103087801	103088100	0.532	0.116	0.296	0.218
UQ	2	103471801	103472100	1.699	0.701	0.720	0.740
UQ	2	103534501	103534800	0.929	0.564	0.662	0.527
UQ	2	103658701	103659000	1.381	0.843	0.671	0.994
UQ	2	103782601	103782900	0.319	0.560	0.983	0.533
UQ	2	103963501	103963800	5.636	6.597	4.853	4.493
UQ	2	104329501	104329800	0.477	0.140	0.243	0.132
UQ	2	104341201	104341500	0.158	0.491	0.617	0.411
UQ	2	104367901	104368200	0.345	0.727	0.633	0.745
UQ	2	104371801	104372100	0.398	0.090	0.000	0.720
UQ	2	104386501	104386800	2.617	4.186	3.895	3.640
UQ	2	104505301	104505600	0.905	0.564	0.444	0.411
UQ	2	105686401	105686700	0.424	0.704	0.160	0.193
UQ	2	105958201	105958500	0.294	0.607	0.025	0.527
UQ	2	106158601	106158900	0.874	0.472	0.346	0.329
UQ	2	106474801	106475100	0.026	0.607	0.156	0.329
UQ	2	106590901	106591200	1.705	2.551	1.358	1.293
UQ	2	106591201	106591500	1.037	1.474	0.555	0.781
UQ	2	106794901	106795200	0.505	0.258	0.218	0.223
UQ	2	106802701	106803000	0.211	0.350	0.481	0.553
UQ	2	107068201	107068500	0.079	0.493	0.321	0.324
UQ	2	107136301	107136600	0.507	0.867	0.317	0.720
UQ	2	107628901	107629200	0.239	0.656	0.078	0.137
UQ	2	109020001	109020300	0.636	0.257	0.346	0.385
UQ	2	109080001	109080300	0.505	0.328	0.317	0.329
UQ	2	109333801	109334100	0.079	0.232	0.185	0.608
UQ	2	109876801	109877100	0.663	0.139	0.296	0.279
UQ	2	110015401	110015700	1.039	0.682	0.551	0.416
UQ	2	110016301	110016600	1.944	1.002	1.222	1.424
UQ	2	110089501	110089800	0.027	0.371	0.321	0.335
UQ	2	110154901	110155200	0.953	0.260	0.103	0.340
UQ	2	110179201	110179500	1.011	0.397	0.272	0.304
UQ	2	110200501	110200800	1.252	0.631	0.872	0.791
UQ	2	110870101	110870400	2.174	1.379	1.128	0.897
UQ	2	110874001	110874300	1.273	0.592	0.296	0.360
UQ	2	110901901	110902200	3.189	2.069	2.127	1.978
UQ	2	110928601	110928900	2.320	1.053	1.736	1.683
UQ	2	111425401	111425700	2.609	1.967	2.127	2.115
UQ	2	112644901	112645200	2.425	0.837	1.460	1.684
UQ	2	112899601	112899900	0.900	0.375	0.481	0.335
UQ	2	113066701	113067000	0.584	0.116	0.049	0.000
UQ	2	113210701	113211000	2.712	1.678	2.002	0.983
UQ	2	113567401	113567700	0.797	0.656	1.032	0.416
UQ	2	114766801	114767100	0.823	0.421	0.374	0.193
UQ	2	115361401	115361700	0.534	0.258	0.210	0.132
UQ	2	115736401	115736700	0.534	0.092	0.111	0.106
UQ	2	115935601	115935900	0.905	0.446	0.453	0.507
UQ	2	115987201	115987500	0.558	0.307	0.321	0.248
UQ	2	116818801	116819100	1.218	0.448	0.555	0.527
UQ	2	116961601	116961900	1.465	1.028	1.061	0.983
UQ	2	116961901	116962200	1.492	0.753	0.979	0.826
UQ	2	117212701	117213000	1.460	0.359	0.490	0.213
UQ	2	117291901	117292200	1.142	0.414	0.515	0.578

UQ	2	117455101	117455400	3.000	1.212	0.987	0.329
UQ	2	117463801	117464100	4.230	1.916	1.904	1.186
UQ	2	117768901	117769200	1.516	1.212	0.588	1.217
UQ	2	118212901	118213200	0.505	0.983	1.387	0.821
UQ	2	118343101	118343400	0.773	0.375	0.354	0.355
UQ	2	118749301	118749600	0.426	0.326	0.185	0.578
UQ	2	118920601	118920900	0.398	1.193	0.934	0.832
UQ	2	119057101	119057400	0.532	0.279	0.127	0.274
UQ	2	119103001	119103300	0.821	0.161	0.223	0.112
UQ	2	119103301	119103600	0.740	0.092	0.087	0.137
UQ	2	119759401	119759700	0.266	0.727	0.448	0.583
UQ	2	120966301	120966600	0.637	0.375	0.312	0.137
UQ	2	121216201	121216500	1.757	1.219	1.217	1.085
UQ	2	121945801	121946100	0.345	0.468	0.506	0.639
UQ	2	122408101	122408400	0.477	0.234	0.049	0.132
UQ	2	122778601	122778900	1.140	0.773	0.531	0.832
UQ	2	124494901	124495200	1.170	0.701	0.695	0.486
UQ	2	124495201	124495500	1.249	0.584	0.720	0.481
UQ	2	124895401	124895700	0.689	0.395	0.239	0.360
UQ	2	125053801	125054100	0.558	0.144	0.111	0.173
UQ	2	125255401	125255700	1.113	0.373	0.740	0.690
UQ	2	126687001	126687300	0.295	0.867	0.831	1.004
UQ	2	126991201	126991500	1.274	0.959	1.012	1.024
UQ	2	127030201	127030500	0.374	0.818	0.580	0.608
UQ	2	129658801	129659100	0.744	0.442	0.296	0.522
UQ	2	129792901	129793200	1.013	0.680	0.564	0.659
UQ	2	129840601	129840900	0.240	0.468	0.078	0.411
UQ	2	129892801	129893100	0.771	0.656	0.613	0.994
UQ	2	130110001	130110300	1.357	0.543	0.667	0.370
UQ	2	130298101	130298400	0.321	0.560	0.082	0.076
UQ	2	130542901	130543200	2.265	3.583	3.110	3.732
UQ	2	130770001	130770300	1.781	1.261	1.259	0.994
UQ	2	131439001	131439300	1.171	1.053	0.905	1.024
UQ	2	131933401	131933700	0.532	0.373	0.136	0.416
UQ	2	132119701	132120000	0.211	1.317	0.818	0.659
UQ	2	132297001	132297300	1.116	0.257	0.156	0.218
UQ	2	132751801	132752100	0.718	0.560	0.292	0.446
UQ	2	132901801	132902100	0.503	0.841	0.296	0.887
UQ	2	133126201	133126500	0.507	0.187	0.292	0.243
UQ	2	133406101	133406400	0.663	0.890	0.160	0.309
UQ	2	133565701	133566000	0.426	0.981	0.831	0.923
UQ	2	133766401	133766700	0.105	0.560	0.403	0.365
UQ	2	134515501	134515800	0.265	1.351	1.188	1.282
UQ	2	134515801	134516100	0.211	1.897	1.777	1.739
UQ	2	134589301	134589600	0.081	0.605	0.560	0.411
UQ	2	135212701	135213000	2.741	1.126	1.097	1.760
UQ	2	135763201	135763500	0.773	0.399	0.346	0.254
UQ	2	136030201	136030500	0.586	0.258	0.185	0.380
UQ	2	136836601	136836900	1.185	3.002	3.121	2.889
UQ	2	137017501	137017800	0.505	0.185	0.272	0.248
UQ	2	137023201	137023500	0.744	0.260	0.267	0.193
UQ	2	137105101	137105400	0.000	0.116	0.502	0.218
UQ	2	137423401	137423700	0.505	0.234	0.374	0.218
UQ	2	137538001	137538300	0.292	0.444	0.107	0.056
UQ	2	137891101	137891400	0.027	0.302	0.477	0.304
UQ	2	138030001	138030300	0.347	0.513	0.107	0.218
UQ	2	138293101	138293400	0.534	0.116	0.341	0.329
UQ	2	139430401	139430700	0.479	0.187	0.667	0.254
UQ	2	139607401	139607700	0.690	0.330	0.296	0.309
UQ	2	139694401	139694700	1.171	0.444	0.087	0.618
UQ	2	139694701	139695000	1.008	0.421	0.058	0.603
UQ	2	139697401	139697700	0.745	0.165	0.029	0.431
UQ	2	140060401	140060700	0.052	0.562	0.292	0.279
UQ	2	140266501	140266800	0.611	1.098	0.974	0.988
UQ	2	141171001	141171300	0.477	0.140	0.214	0.299
UQ	2	141570001	141570300	0.213	0.444	0.370	0.304
UQ	2	141688801	141689100	0.563	0.024	0.078	0.081

UQ	2	142689901	142690200	2.925	3.621	3.682	4.183
UQ	2	142893901	142894200	1.997	1.337	1.233	1.262
UQ	2	143113501	143113800	1.620	0.839	0.818	0.968
UQ	2	143809501	143809800	0.479	0.820	0.695	1.080
UQ	2	147098101	147098400	0.665	0.328	0.350	0.193
UQ	2	147819601	147819900	0.534	0.281	0.243	0.198
UQ	2	148274401	148274700	0.747	0.466	0.210	0.299
UQ	2	148823101	148823400	0.423	0.000	0.049	0.025
UQ	2	149049001	149049300	0.160	0.376	0.350	0.634
UQ	2	149163301	149163600	0.453	1.053	0.691	0.603
UQ	2	149457901	149458200	0.132	0.420	0.292	0.248
UQ	2	149537701	149538000	0.187	0.631	0.510	0.385
UQ	2	150020101	150020400	0.213	0.607	0.428	0.329
UQ	2	151191001	151191300	0.505	0.142	0.165	0.193
UQ	2	151640701	151641000	1.113	0.468	0.691	0.547
UQ	2	152145901	152146200	3.933	3.624	4.496	5.258
UQ	2	153334501	153334800	0.265	0.584	0.453	0.360
UQ	2	153831001	153831300	0.692	0.236	0.370	0.223
UQ	2	155691301	155691600	0.052	0.493	0.214	0.385
UQ	2	155971801	155972100	0.848	0.421	0.432	0.492
UQ	2	156480901	156481200	1.118	0.865	0.535	0.664
UQ	2	157121101	157121400	1.224	0.723	0.555	0.796
UQ	2	157443601	157443900	0.611	0.069	0.136	0.182
UQ	2	157697401	157697700	1.170	0.365	0.453	0.279
UQ	2	157949101	157949400	1.224	0.320	0.366	0.568
UQ	2	158819101	158819400	1.541	0.751	0.613	2.292
UQ	2	159015601	159015900	0.453	0.629	0.210	0.218
UQ	2	159443101	159443400	0.637	0.397	0.448	0.248
UQ	2	159751501	159751800	1.434	0.863	0.867	0.756
UQ	2	161975701	161976000	0.532	0.139	0.218	0.248
UQ	2	163693801	163694100	1.063	0.772	0.687	0.801
UQ	2	164034001	164034300	3.967	1.283	1.859	2.524
UQ	2	164494201	164494500	0.081	0.749	0.288	0.639
UQ	2	164736901	164737200	1.034	1.551	1.674	2.540
UQ	2	165411301	165411600	0.636	0.142	0.317	0.335
UQ	2	168427201	168427500	0.531	0.232	0.156	0.106
UQ	2	169216801	169217100	0.771	0.328	0.564	0.385
UQ	2	169279501	169279800	0.319	0.116	0.555	0.137
UQ	2	169536001	169536300	2.204	3.428	4.731	3.185
UQ	2	169948501	169948800	0.558	0.421	0.296	0.416
UQ	2	170105701	170106000	0.802	0.330	0.395	0.329
UQ	2	170243101	170243400	1.036	0.654	0.667	0.634
UQ	2	170651401	170651700	0.639	0.255	0.325	0.360
UQ	2	171083701	171084000	0.639	0.167	0.214	0.416
UQ	2	172737901	172738200	0.505	0.140	0.176	0.137
UQ	2	172769101	172769400	0.560	0.232	0.194	0.274
UQ	2	175086901	175087200	0.055	0.466	0.317	0.335
UQ	2	175318501	175318800	2.928	2.246	2.127	1.606
UQ	2	175754401	175754700	0.771	0.395	0.243	0.497
UQ	2	176527201	176527500	1.408	0.985	0.604	1.019
UQ	2	177824401	177824700	0.053	0.371	0.346	0.329
UQ	2	178542901	178543200	1.812	1.068	0.852	1.141
UQ	2	178735501	178735800	1.323	0.378	0.600	0.304
UQ	2	180427201	180427500	0.505	0.185	0.103	0.299
UQ	2	180775501	180775800	0.137	0.729	1.144	1.014
UQ	2	180775801	180776100	0.163	1.339	1.622	1.546
UQ	2	182409301	182409600	1.597	0.848	0.379	0.675
UQ	2	182880901	182881200	0.531	0.232	0.263	0.335
UQ	2	183111601	183111900	0.477	0.212	0.140	0.081
UQ	2	183398701	183399000	0.718	0.185	0.218	0.162
UQ	2	183437701	183438000	0.639	0.399	0.317	0.299
UQ	2	183829201	183829500	3.914	1.827	1.982	1.572
UQ	2	183829501	183829800	5.720	2.309	2.945	2.803
UQ	2	183864001	183864300	0.292	0.609	0.502	0.472
UQ	2	184602601	184602900	0.881	0.352	0.408	0.309
UQ	2	184771201	184771500	1.278	1.053	0.662	1.075
UQ	2	185038201	185038500	0.905	0.513	0.522	0.547

UQ	2	185073601	185073900	3.796	2.384	2.949	2.997
UQ	2	185249401	185249700	0.989	0.513	0.477	0.329
UQ	2	185388901	185389200	1.091	0.749	0.753	0.466
UQ	2	185446201	185446500	0.958	1.380	0.593	0.492
UQ	2	185490301	185490600	1.783	0.940	1.057	0.857
UQ	2	185509801	185510100	1.089	0.865	0.560	0.659
UQ	2	185718601	185718900	1.120	0.701	0.736	0.664
UQ	2	185835301	185835600	0.455	1.171	0.901	0.796
UQ	2	185870401	185870700	0.345	0.910	0.736	0.695
UQ	2	187194601	187194900	0.639	0.491	0.292	0.411
UQ	2	187194901	187195200	0.716	0.491	0.296	0.360
UQ	2	187707901	187708200	0.824	0.324	0.403	0.472
UQ	2	187868101	187868400	0.186	0.562	0.370	0.355
UQ	2	189529501	189529800	0.878	0.208	0.453	0.263
UQ	2	189781501	189781800	0.878	0.605	0.531	0.421
UQ	2	190142401	190142700	0.982	0.770	0.531	0.751
UQ	2	190684201	190684500	0.398	0.562	0.827	0.578
UQ	2	190711801	190712100	1.355	0.517	0.410	0.644
UQ	2	191215201	191215500	6.383	4.109	4.952	4.767
UQ	2	192974101	192974400	0.026	0.468	0.234	0.223
UQ	2	193416901	193417200	0.507	0.796	0.638	0.690
UQ	2	194867701	194868000	0.586	1.244	1.444	1.267
UQ	2	195516001	195516300	0.505	0.375	0.267	0.274
UQ	2	196483801	196484100	1.116	0.257	1.329	0.968
UQ	2	196555501	196555800	1.521	0.749	0.691	0.882
UQ	2	196562401	196562700	3.055	1.266	2.378	2.276
UQ	2	197178301	197178600	1.142	0.680	0.642	0.547
UQ	2	198829501	198829800	0.132	0.609	0.395	0.466
UQ	2	199069201	199069500	0.507	0.165	0.243	0.223
UQ	2	199754101	199754400	1.226	0.798	0.691	0.522
UQ	2	200360101	200360400	0.639	0.326	0.267	0.299
UQ	2	200781001	200781300	0.477	0.258	0.239	0.279
UQ	2	200873401	200873700	0.661	1.328	1.826	1.197
UQ	2	200959501	200959800	1.274	1.053	1.806	0.628
UQ	2	201100201	201100500	0.558	0.420	0.399	0.137
UQ	2	201540001	201540300	1.431	0.515	0.354	0.380
UQ	2	201801601	201801900	0.771	0.326	0.361	0.355
UQ	2	201855901	201856200	0.557	0.142	0.189	0.137
UQ	2	202004701	202005000	0.555	0.167	0.029	0.106
UQ	2	202397701	202398000	3.780	2.499	2.905	2.530
UQ	2	202899601	202899900	1.671	3.028	2.644	2.652
UQ	2	203271601	203271900	0.932	0.515	0.473	0.588
UQ	2	203581201	203581500	0.134	0.536	0.317	0.279
UQ	2	203763601	203763900	0.266	0.538	0.531	0.329
UQ	2	203823001	203823300	0.637	0.305	0.283	0.254
UQ	2	204587701	204588000	1.165	1.759	1.942	2.124
UQ	2	204704701	204705000	1.621	1.144	1.402	1.831
UQ	2	205392901	205393200	0.771	0.468	0.510	0.472
UQ	2	207849301	207849600	1.166	1.541	1.806	1.744
UQ	2	207942601	207942900	0.213	0.375	0.424	0.466
UQ	2	208780201	208780500	0.715	0.167	0.205	0.167
UQ	2	209832901	209833200	0.718	0.631	1.061	0.527
UQ	2	209892301	209892600	1.410	0.466	0.506	0.675
UQ	2	210152701	210153000	0.878	0.489	0.531	0.634
UQ	2	210477001	210477300	0.240	0.444	0.132	0.112
UQ	2	210713701	210714000	0.160	0.579	0.560	0.522
UQ	2	212289001	212289300	0.558	0.446	0.239	0.416
UQ	2	213440701	213441000	0.532	0.120	0.292	0.360
UQ	2	213467701	213468000	0.560	0.234	0.267	0.203
UQ	2	213479401	213479700	0.158	0.493	0.428	0.416
UQ	2	214753201	214753500	0.642	0.139	0.160	0.132
UQ	2	215579701	215580000	0.582	0.208	0.263	0.198
UQ	2	216266401	216266700	1.249	0.910	0.370	0.538
UQ	2	216543301	216543600	2.549	1.897	1.830	2.043
UQ	2	217691101	217691400	2.025	1.545	0.963	1.004
UQ	2	217881601	217881900	1.091	0.678	0.930	0.664
UQ	2	218286001	218286300	5.111	4.106	5.783	5.791

UQ	2	218577001	218577300	0.560	0.796	0.959	0.720
UQ	2	219011701	219012000	0.558	0.352	0.239	0.411
UQ	2	219200701	219201000	0.426	0.609	0.584	0.578
UQ	2	219547801	219548100	0.345	0.961	0.502	0.690
UQ	2	219806701	219807000	0.692	0.444	0.312	0.472
UQ	2	220357201	220357500	1.007	0.376	0.638	0.553
UQ	2	221337301	221337600	0.081	0.210	0.477	0.441
UQ	2	221621701	221622000	0.450	0.023	0.136	0.112
UQ	2	222226801	222227100	0.557	0.279	0.346	0.223
UQ	2	222316501	222316800	0.557	0.232	0.103	0.056
UQ	2	223256701	223257000	0.265	0.611	0.711	0.684
UQ	2	223644301	223644600	1.037	0.654	0.613	0.583
UQ	2	224169601	224169900	0.400	0.560	0.818	1.410
UQ	2	224216401	224216700	0.158	0.466	0.477	0.355
UQ	2	224729101	224729400	0.507	0.140	0.210	0.092
UQ	2	224761501	224761800	1.065	0.491	0.510	0.695
UQ	2	225282601	225282900	0.450	0.167	0.107	0.081
UQ	2	225336301	225336600	1.837	1.335	1.045	0.994
UQ	2	225343801	225344100	0.611	0.328	0.205	0.304
UQ	2	226082101	226082400	0.902	0.584	0.555	0.578
UQ	2	226139401	226139700	1.621	1.777	1.431	1.136
UQ	2	226318201	226318500	0.608	0.281	0.185	0.958
UQ	2	226560601	226560900	0.319	0.515	0.695	0.695
UQ	2	226695601	226695900	3.767	2.191	1.864	1.791
UQ	2	226866601	226866900	0.079	0.444	0.263	0.391
UQ	2	227343001	227343300	0.852	0.493	0.415	0.441
UQ	2	227635201	227635500	0.584	0.772	0.987	0.832
UQ	2	227712301	227712600	0.903	0.560	0.428	0.477
UQ	2	227736601	227736900	0.613	0.212	0.296	0.112
UQ	2	228163201	228163500	0.584	0.702	0.424	0.167
UQ	2	228752401	228752700	0.266	0.493	0.531	0.533
UQ	2	229224001	229224300	0.132	0.468	0.535	0.598
UQ	2	229529401	229529700	0.079	0.279	0.399	0.527
UQ	2	229977601	229977900	0.507	0.165	0.210	0.167
UQ	2	230439601	230439900	0.294	0.560	0.638	0.441
UQ	2	230707201	230707500	1.086	0.586	0.526	0.360
UQ	2	231248101	231248400	3.615	2.176	2.426	1.450
UQ	2	231331501	231331800	0.477	0.137	0.140	0.137
UQ	2	231595201	231595500	0.240	0.729	0.584	0.628
UQ	2	231763801	231764100	2.363	1.650	1.115	1.217
UQ	2	231990601	231990900	0.000	0.258	0.535	0.193
UQ	2	232363801	232364100	1.889	0.910	1.168	0.822
UQ	2	232367401	232367700	7.119	3.529	3.640	3.924
UQ	2	232411201	232411500	0.640	0.260	0.214	0.203
UQ	2	232903201	232903500	0.187	0.536	0.502	0.436
UQ	2	233029501	233029800	2.796	2.148	1.230	1.512
UQ	2	233192401	233192700	0.186	0.421	0.370	0.492
UQ	2	233346001	233346300	0.505	0.255	0.181	0.193
UQ	2	234185101	234185400	0.586	0.165	0.107	0.187
UQ	2	234338401	234338700	1.120	0.751	0.584	0.740
UQ	2	234524101	234524400	0.508	0.841	0.925	0.887
UQ	2	234709201	234709500	3.162	1.974	2.053	1.830
UQ	2	234709501	234709800	3.105	1.888	1.852	1.678
UQ	2	234775801	234776100	0.636	0.981	0.346	0.329
UQ	2	234792601	234792900	0.131	0.609	0.481	0.385
UQ	2	235680601	235680900	2.471	4.765	3.234	3.935
UQ	2	235723201	235723500	2.020	1.261	1.431	1.075
UQ	2	235768501	235768800	0.160	0.234	0.424	0.497
UQ	2	235925101	235925400	1.626	0.983	1.061	1.064
UQ	2	236145001	236145300	1.011	0.586	0.629	0.522
UQ	2	236172301	236172600	0.237	0.794	0.453	0.664
UQ	2	236248201	236248500	0.692	0.444	0.584	0.553
UQ	2	236297101	236297400	0.721	0.168	0.259	0.086
UQ	2	236450401	236450700	3.433	2.590	3.007	2.429
UQ	2	236599201	236599500	0.799	0.331	0.330	0.329
UQ	2	236821501	236821800	0.160	0.820	0.502	0.558
UQ	2	237988501	237988800	0.613	0.326	0.346	0.309

UQ	2	239446801	239447100	1.092	0.326	0.802	0.776
UQ	2	240226801	240227100	1.462	0.983	0.992	0.877
UQ	2	240508501	240508800	1.144	0.727	0.675	0.715
UQ	2	241356901	241357200	0.903	1.053	0.560	1.298
UQ	2	241476901	241477200	1.276	1.776	1.008	1.136
UQ	2	241479901	241480200	1.279	0.654	0.439	0.537
UQ	2	241658101	241658400	0.716	0.421	0.428	0.522
UQ	2	241662901	241663200	0.745	0.376	0.181	0.279
UQ	2	241700701	241701000	3.656	1.244	1.388	1.162
UQ	2	241707601	241707900	2.076	0.803	1.077	0.837
UQ	2	241709701	241710000	3.746	1.843	1.806	1.845
UQ	2	241710001	241710300	4.446	2.804	2.842	2.795
UQ	2	241814701	241815000	0.934	0.283	0.403	0.207
UQ	2	242063701	242064000	0.958	0.680	1.193	0.527
UQ	2	242154901	242155200	0.848	0.255	0.259	0.421
UQ	2	242171701	242172000	0.479	0.680	0.292	0.167
UQ	2	242268601	242268900	1.113	0.633	0.613	0.299
UQ	2	242889001	242889300	0.764	1.523	1.645	1.755
UQ	2	242907901	242908200	0.850	0.418	0.370	0.527
UQ	2	242954101	242954400	1.439	0.652	0.865	0.695
UQ	2	243085801	243086100	0.265	0.706	1.086	1.111
UQ	2	243114601	243114900	0.824	0.586	0.638	0.502
UQ	2	243171601	243171900	2.791	2.246	2.238	2.231
UQ	2	243219901	243220200	1.862	3.529	3.184	2.804
UQ	2	243221101	243221400	2.204	1.611	1.387	1.262
UQ	2	243228601	243228900	0.821	1.687	1.353	1.415
UQ	2	243243601	243243900	1.142	0.324	0.205	0.279
UQ	2	243383401	243383700	1.382	0.283	0.234	0.613
UQ	2	243488101	243488400	0.689	0.468	0.239	0.466
UQ	2	243497101	243497400	4.056	1.517	1.282	1.246
UQ	2	243530101	243530400	0.931	0.747	0.687	0.309
UQ	2	243555601	243555900	0.400	0.773	0.798	0.857
UQ	2	243597001	243597300	1.010	0.747	0.716	0.477
UQ	2	244021801	244022100	1.139	0.210	0.337	0.406
UQ	2	244258801	244259100	1.944	1.245	1.152	1.111
UQ	2	244307401	244307700	4.994	2.869	3.690	3.691
UQ	2	244392601	244392900	0.398	0.678	0.691	0.329
UQ	2	244959301	244959600	1.807	1.500	1.173	2.150
UQ	2	244991401	244991700	0.213	0.468	0.321	0.385
UQ	2	245011801	245012100	0.905	0.281	0.609	0.477
UQ	2	245103901	245104200	2.258	1.257	1.052	0.922
UQ	2	245464501	245464800	0.937	1.642	1.728	1.653
UQ	2	245467801	245468100	1.515	1.099	0.564	1.090
UQ	2	245538601	245538900	2.415	1.444	1.538	1.582
UQ	2	245557201	245557500	1.407	2.074	2.164	2.160
UQ	2	245659201	245659500	0.584	0.210	0.283	0.193
UQ	2	245728201	245728500	0.986	0.727	0.613	0.720
UQ	2	245730301	245730600	0.876	0.277	0.156	0.187
UQ	2	245743501	245743800	1.436	0.841	1.090	0.893
UQ	2	245760901	245761200	3.910	1.953	1.714	1.318
UQ	2	245944501	245944800	0.450	0.071	0.103	0.106
UQ	2	246292501	246292800	2.286	1.290	1.587	1.542
UQ	2	246403801	246404100	3.036	4.306	4.139	4.254
UQ	2	246475201	246475500	0.958	0.558	0.082	0.370
UQ	2	246615901	246616200	0.639	0.305	0.214	0.385
UQ	2	246634201	246634500	1.223	0.753	0.745	0.765
UQ	2	246707701	246708000	3.296	4.154	4.423	2.616
UQ	2	246727201	246727500	1.487	0.981	2.164	1.876
UQ	2	246843001	246843300	3.455	4.158	2.731	3.032
UQ	2	246933601	246933900	3.538	2.201	2.735	2.839
UQ	2	247095901	247096200	0.982	0.652	0.346	0.614
UQ	2	247107301	247107600	5.191	4.143	3.419	3.001
UQ	2	247236001	247236300	3.751	2.856	2.757	2.581
UQ	2	247286101	247286400	3.475	1.699	1.217	1.875
UQ	2	247330201	247330500	1.857	0.762	0.930	1.091
UQ	2	247432201	247432500	0.558	0.330	0.296	0.274
UQ	2	247486501	247486800	1.855	0.938	0.774	1.065

UQ	2	247687201	247687500	3.163	2.398	2.410	2.464
UQ	2	247971601	247971900	9.179	6.484	7.638	7.767
UQ	2	248070901	248071200	1.889	0.629	1.456	1.369
UQ	2	248142901	248143200	3.110	2.547	2.225	2.317
UQ	2	248198701	248199000	3.357	2.334	2.131	1.770
UQ	2	248244901	248245200	0.876	0.586	0.481	0.466
UQ	2	248357701	248358000	0.558	0.326	0.243	0.193
UQ	2	248686501	248686800	0.929	0.657	0.379	0.304
UQ	2	248781001	248781300	4.072	3.240	2.764	2.089
UQ	2	248968801	248969100	0.960	0.470	0.366	0.461
UQ	20	96001	96300	0.532	0.232	0.132	0.248
UQ	20	717601	717900	0.189	0.491	0.457	0.497
UQ	20	1086901	1087200	0.397	0.210	0.638	0.218
UQ	20	1752901	1753200	0.611	0.189	0.288	0.248
UQ	20	2133301	2133600	0.481	0.185	0.185	0.193
UQ	20	2187301	2187600	0.610	0.071	0.272	0.187
UQ	20	2238301	2238600	0.026	0.283	0.584	0.198
UQ	20	2467201	2467500	0.557	0.114	0.263	0.826
UQ	20	2708101	2708400	1.279	0.656	0.925	0.852
UQ	20	2918101	2918400	0.771	0.470	0.243	0.284
UQ	20	3438601	3438900	0.398	0.676	0.555	0.826
UQ	20	3675901	3676200	0.532	0.281	0.185	0.360
UQ	20	3705901	3706200	2.449	1.663	1.872	1.759
UQ	20	4073701	4074000	0.156	0.489	0.638	0.558
UQ	20	4692901	4693200	3.199	2.062	2.336	2.115
UQ	20	5581201	5581500	1.274	1.614	1.037	1.572
UQ	20	6475501	6475800	7.500	5.945	3.947	5.146
UQ	20	6570301	6570600	0.852	0.656	0.555	0.335
UQ	20	6671401	6671700	1.091	0.817	0.687	0.837
UQ	20	6772201	6772500	1.542	0.912	0.551	0.974
UQ	20	6773101	6773400	2.693	0.942	1.344	1.348
UQ	20	7524301	7524600	0.611	0.397	0.424	0.664
UQ	20	7872601	7872900	0.266	0.678	0.876	1.136
UQ	20	7935901	7936200	0.158	0.468	0.370	0.329
UQ	20	8072101	8072400	1.492	0.628	0.827	0.785
UQ	20	8213701	8214000	2.925	2.365	2.200	2.251
UQ	20	8314501	8314800	0.532	0.375	0.395	0.360
UQ	20	8817901	8818200	0.558	0.234	0.205	0.193
UQ	20	9071701	9072000	0.529	0.024	0.000	0.031
UQ	20	9116701	9117000	2.476	1.442	1.514	1.213
UQ	20	9136201	9136500	0.507	0.796	0.218	0.081
UQ	20	9170701	9171000	0.665	0.139	0.160	0.208
UQ	20	9263701	9264000	0.426	0.609	0.296	0.248
UQ	20	9354301	9354600	0.823	1.193	0.481	1.192
UQ	20	9666301	9666600	0.477	0.116	0.078	0.274
UQ	20	9909301	9909600	0.557	0.206	0.160	0.106
UQ	20	10121101	10121400	0.239	0.444	0.054	0.137
UQ	20	10309801	10310100	0.929	0.371	0.419	0.370
UQ	20	10367101	10367400	0.266	0.444	0.214	0.056
UQ	20	10482001	10482300	1.778	1.287	1.329	0.893
UQ	20	10511701	10512000	3.289	1.262	1.184	2.024
UQ	20	10775401	10775700	1.039	0.729	0.716	0.446
UQ	20	10956601	10956900	0.931	0.611	0.473	0.547
UQ	20	11013001	11013300	1.223	0.912	0.901	0.553
UQ	20	11761801	11762100	0.532	0.678	0.185	0.279
UQ	20	12090901	12091200	3.568	2.386	2.664	2.444
UQ	20	12137701	12138000	2.975	1.562	1.895	1.938
UQ	20	12976201	12976500	3.993	2.285	2.934	2.961
UQ	20	13140901	13141200	0.186	0.442	0.477	0.502
UQ	20	13791901	13792200	0.077	0.536	0.444	0.441
UQ	20	13818601	13818900	1.170	0.725	0.580	0.648
UQ	20	14255401	14255700	0.747	1.219	0.292	0.416
UQ	20	14259001	14259300	1.145	0.730	1.596	1.780
UQ	20	14355601	14355900	1.328	0.399	0.390	0.396
UQ	20	14355901	14356200	1.418	0.326	0.439	0.385
UQ	20	14390101	14390400	4.999	3.973	4.133	3.538
UQ	20	14429701	14430000	1.808	2.594	1.986	2.515

UQ	20	14529301	14529600	0.850	1.425	1.378	1.267
UQ	20	14619301	14619600	2.258	1.777	1.514	1.653
UQ	20	14709901	14710200	2.133	1.047	1.148	1.166
UQ	20	14795701	14796000	0.263	1.099	1.032	1.034
UQ	20	14910001	14910300	0.503	0.118	0.181	0.198
UQ	20	15375001	15375300	0.744	0.326	0.399	0.533
UQ	20	15573001	15573300	1.439	0.775	0.782	0.756
UQ	20	16118701	16119000	2.341	1.703	1.850	1.764
UQ	20	16205401	16205700	1.140	0.446	0.584	0.731
UQ	20	16464901	16465200	2.897	2.032	2.599	2.581
UQ	20	16672501	16672800	2.236	1.686	1.650	1.440
UQ	20	17061001	17061300	1.728	1.313	1.387	1.359
UQ	20	17077501	17077800	0.452	0.328	0.555	0.329
UQ	20	17395201	17395500	0.477	0.232	0.078	0.167
UQ	20	17402101	17402400	0.477	0.118	0.074	0.061
UQ	20	17469001	17469300	0.692	0.444	0.317	0.446
UQ	20	17609401	17609700	1.011	1.242	0.584	1.541
UQ	20	18099001	18099300	0.505	0.330	0.185	0.112
UQ	20	18915001	18915300	1.613	0.457	0.477	0.416
UQ	20	18937201	18937500	1.702	1.144	1.119	1.111
UQ	20	19171201	19171500	1.937	1.105	0.847	0.811
UQ	20	19329901	19330200	0.586	0.399	0.769	0.360
UQ	20	20224501	20224800	0.665	0.350	0.341	0.391
UQ	20	20382301	20382600	1.200	1.498	0.428	0.634
UQ	20	21155701	21156000	1.140	0.609	0.700	0.497
UQ	20	21156001	21156300	1.784	0.989	1.193	0.715
UQ	20	21854701	21855000	0.560	0.324	0.107	0.112
UQ	20	21855001	21855300	0.960	0.468	0.259	0.173
UQ	20	21874501	21874800	1.353	0.890	0.695	0.472
UQ	20	21874801	21875100	1.195	0.451	0.296	0.268
UQ	20	22663501	22663800	0.611	0.071	0.214	0.137
UQ	20	23502901	23503200	2.102	1.611	1.563	1.116
UQ	20	24021001	24021300	0.318	0.586	0.633	0.608
UQ	20	24431401	24431700	0.823	0.305	0.346	0.603
UQ	20	24511501	24511800	2.789	3.201	1.752	1.764
UQ	20	24687901	24688200	0.610	0.841	0.847	0.335
UQ	20	25013401	25013700	0.345	0.772	0.638	0.533
UQ	20	25367701	25368000	0.907	0.135	0.127	0.202
UQ	20	26033701	26034000	0.799	0.371	0.111	0.405
UQ	20	26546701	26547000	2.291	1.798	1.275	1.034
UQ	20	26595901	26596200	1.417	3.042	2.910	3.214
UQ	20	26596201	26596500	0.507	0.839	1.090	0.695
UQ	20	26606401	26606700	3.478	2.755	2.378	2.682
UQ	20	26740501	26740800	0.774	0.281	0.243	0.203
UQ	20	27189601	27189900	0.534	0.212	0.189	0.254
UQ	20	27600001	27600300	1.305	0.802	0.453	0.664
UQ	20	28801201	28801500	1.753	1.045	0.535	0.365
UQ	20	29491501	29491800	0.455	0.024	0.054	0.056
UQ	20	30449101	30449400	1.437	0.989	0.205	0.745
UQ	20	30837601	30837900	2.099	1.547	1.115	1.410
UQ	20	31151101	31151400	1.781	1.171	1.358	1.552
UQ	20	31353301	31353600	0.534	1.564	1.373	0.968
UQ	20	31433401	31433700	3.237	1.682	1.817	1.784
UQ	20	31768201	31768500	0.984	0.843	0.560	0.857
UQ	20	31777201	31777500	1.039	0.747	0.551	0.329
UQ	20	31787701	31788000	1.197	0.891	0.486	0.304
UQ	20	31839301	31839600	0.079	0.279	0.560	0.279
UQ	20	32042401	32042700	0.878	0.513	0.453	0.497
UQ	20	32246101	32246400	0.902	2.469	2.242	1.962
UQ	20	32246401	32246700	0.823	1.704	1.772	1.654
UQ	20	32943601	32943900	0.639	0.399	0.267	0.198
UQ	20	34428301	34428600	0.558	1.307	1.217	1.004
UQ	20	34560601	34560900	0.637	0.375	0.243	0.243
UQ	20	34877101	34877400	0.823	1.517	0.506	1.141
UQ	20	35913901	35914200	1.352	0.229	0.174	0.137
UQ	20	35914201	35914500	1.279	0.116	0.145	0.106
UQ	20	36477901	36478200	0.187	0.699	0.481	0.411

UQ	20	36496501	36496800	0.240	0.444	0.082	0.391
UQ	20	36573301	36573600	1.037	0.657	0.745	0.385
UQ	20	37052401	37052700	0.586	0.328	0.263	0.472
UQ	20	37207201	37207500	0.932	0.536	0.560	0.502
UQ	20	37289101	37289400	0.266	0.539	0.160	0.137
UQ	20	37302301	37302600	0.828	0.375	0.453	0.477
UQ	20	37808701	37809000	1.541	1.262	1.271	0.988
UQ	20	37809001	37809300	2.705	1.697	1.587	1.445
UQ	20	38846401	38846700	2.052	1.586	1.255	1.044
UQ	20	39805501	39805800	2.658	1.251	1.547	1.622
UQ	20	39805801	39806100	4.309	1.909	2.292	2.925
UQ	20	39929101	39929400	0.718	0.354	0.140	0.385
UQ	20	40301101	40301400	0.132	0.609	0.296	0.335
UQ	20	40533901	40534200	0.373	0.633	0.156	0.198
UQ	20	41130601	41130900	4.628	1.069	0.682	0.980
UQ	20	41130901	41131200	1.870	0.552	0.428	0.446
UQ	20	41199301	41199600	0.532	0.702	0.691	0.776
UQ	20	41423401	41423700	0.240	0.798	0.963	0.761
UQ	20	41520001	41520300	3.004	1.238	2.028	2.099
UQ	20	41570401	41570700	0.160	0.538	0.477	0.553
UQ	20	41742001	41742300	0.507	0.725	0.210	0.335
UQ	20	41742901	41743200	1.278	0.611	0.484	0.466
UQ	20	41851801	41852100	0.105	0.583	0.695	0.553
UQ	20	41915401	41915700	0.611	0.796	0.909	1.242
UQ	20	41959501	41959800	0.106	0.468	0.568	0.527
UQ	20	42373801	42374100	0.587	0.161	0.165	0.228
UQ	20	42374101	42374400	0.795	0.182	0.296	0.198
UQ	20	42655201	42655500	0.905	0.511	0.502	0.578
UQ	20	43377301	43377600	11.126	7.490	8.994	9.070
UQ	20	43524301	43524600	1.681	0.373	0.979	0.842
UQ	20	43560601	43560900	2.708	1.935	1.728	2.099
UQ	20	43663501	43663800	0.213	2.128	1.565	1.632
UQ	20	44208301	44208600	1.731	1.216	1.061	1.125
UQ	20	44208601	44208900	2.406	1.266	1.050	1.100
UQ	20	44235601	44235900	2.817	1.963	2.187	2.079
UQ	20	44271901	44272200	1.061	0.425	0.374	0.466
UQ	20	44512201	44512500	0.081	0.513	0.399	0.411
UQ	20	44702101	44702400	5.336	3.903	3.354	3.778
UQ	20	44842201	44842500	0.584	0.446	0.292	0.329
UQ	20	45563401	45563700	0.613	0.350	0.267	0.335
UQ	20	45645601	45645900	0.292	0.995	2.546	1.633
UQ	20	45966001	45966300	0.507	0.165	0.239	0.304
UQ	20	46302601	46302900	0.581	1.326	1.362	1.420
UQ	20	46320601	46320900	3.220	1.592	2.595	2.095
UQ	20	46473001	46473300	1.408	1.032	0.798	0.963
UQ	20	46523701	46524000	2.016	0.558	0.836	1.521
UQ	20	46753501	46753800	0.926	0.253	0.243	0.183
UQ	20	46854601	46854900	2.294	1.424	1.251	1.557
UQ	20	47156401	47156700	2.710	1.689	1.732	1.804
UQ	20	47314201	47314500	1.674	1.197	0.519	1.080
UQ	20	47336101	47336400	0.740	0.279	0.239	0.056
UQ	20	47360401	47360700	0.666	0.120	0.267	0.162
UQ	20	47396401	47396700	0.744	0.116	0.346	0.178
UQ	20	47520301	47520600	0.960	0.277	0.461	0.497
UQ	20	47742601	47742900	0.532	0.258	0.292	0.441
UQ	20	47805001	47805300	0.557	0.236	0.156	0.193
UQ	20	47839201	47839500	4.888	3.824	3.192	3.615
UQ	20	47884501	47884800	2.554	1.351	1.336	1.197
UQ	20	48018901	48019200	0.558	1.054	0.983	1.004
UQ	20	48181501	48181800	1.415	2.040	0.785	2.155
UQ	20	48551701	48552000	0.692	0.420	0.317	0.385
UQ	20	48606601	48606900	0.958	0.633	1.242	0.639
UQ	20	48772201	48772500	0.531	1.077	0.769	0.720
UQ	20	48858001	48858300	2.257	2.948	3.279	2.972
UQ	20	49170301	49170600	3.481	4.445	4.989	4.133
UQ	20	49664101	49664400	3.433	2.412	2.443	2.941
UQ	20	49803601	49803900	1.700	1.446	1.061	0.913

UQ	20	50043901	50044200	7.845	8.698	6.143	9.040
UQ	20	50126701	50127000	0.718	0.140	0.214	0.254
UQ	20	50454601	50454900	0.984	1.768	1.739	2.048
UQ	20	50612101	50612400	1.279	0.721	0.881	0.715
UQ	21	374101	374400	0.186	0.303	0.321	0.497
UQ	21	484501	484800	5.477	5.870	5.265	5.805
UQ	21	670501	670800	0.626	2.888	2.753	3.173
UQ	21	1460401	1460700	1.116	0.609	1.427	0.628
UQ	21	1739101	1739400	1.060	0.275	0.210	0.218
UQ	21	2079901	2080200	0.744	0.371	0.350	0.385
UQ	21	2220901	2221200	0.505	0.163	0.160	0.243
UQ	21	4779601	4779900	0.639	0.399	0.214	0.248
UQ	21	5119801	5120100	0.187	0.420	0.317	0.497
UQ	21	5367301	5367600	0.560	0.279	0.185	0.416
UQ	21	6164101	6164400	0.079	0.420	0.341	0.497
UQ	21	6470401	6470700	0.481	0.114	0.107	0.142
UQ	21	6879601	6879900	2.231	1.317	1.440	1.328
UQ	21	7035901	7036200	0.883	0.328	0.370	0.446
UQ	21	7227301	7227600	0.905	0.513	0.588	0.248
UQ	21	7448401	7448700	1.223	0.543	0.642	0.456
UQ	21	7546201	7546500	0.957	1.450	1.119	1.465
UQ	21	7574401	7574700	3.278	1.776	2.222	2.100
UQ	21	7651501	7651800	5.256	8.236	8.126	7.472
UQ	21	7651801	7652100	2.813	4.460	4.044	4.066
UQ	21	7904401	7904700	0.290	1.541	1.070	1.465
UQ	21	8479801	8480100	3.031	2.319	2.238	2.677
UQ	21	8929501	8929800	0.055	0.513	0.341	0.309
UQ	21	9061501	9061800	2.770	3.418	2.115	2.119
UQ	21	9157201	9157500	1.170	0.841	0.827	0.862
UQ	21	9202501	9202800	0.292	0.751	0.531	0.690
UQ	21	9700801	9701100	0.292	0.187	0.346	0.497
UQ	21	9954001	9954300	0.215	0.536	0.502	0.522
UQ	21	10072801	10073100	1.623	0.955	1.094	1.222
UQ	21	10111501	10111800	0.905	0.725	0.424	0.776
UQ	21	10209601	10209900	0.239	2.029	0.865	0.954
UQ	21	10663201	10663500	0.639	0.234	0.395	0.223
UQ	21	11243401	11243700	0.534	0.303	0.296	0.279
UQ	21	11736601	11736900	2.023	2.572	2.702	2.819
UQ	21	11754601	11754900	0.613	0.255	0.370	0.279
UQ	21	12245701	12246000	0.611	0.468	0.424	0.527
UQ	21	12904501	12904800	0.213	0.586	0.457	0.608
UQ	21	12964501	12964800	0.826	0.609	0.691	1.186
UQ	21	13864501	13864800	0.587	0.255	0.234	0.187
UQ	21	13971301	13971600	0.610	2.081	1.518	1.232
UQ	21	14290801	14291100	0.160	0.515	0.239	0.335
UQ	21	14390401	14390700	0.852	1.800	0.609	0.745
UQ	21	14570701	14571000	0.186	0.517	0.584	0.776
UQ	21	15002101	15002400	0.240	0.558	0.642	0.497
UQ	21	15526201	15526500	0.561	0.094	0.087	0.147
UQ	21	15535501	15535800	1.300	0.605	0.740	0.882
UQ	21	15841501	15841800	0.371	0.515	0.979	0.720
UQ	21	15865201	15865500	0.824	0.258	0.254	0.304
UQ	21	15945001	15945300	1.358	0.706	0.531	0.568
UQ	21	16069801	16070100	0.584	0.232	0.259	0.254
UQ	21	16096201	16096500	0.316	0.957	1.094	0.947
UQ	21	16149601	16149900	1.036	0.701	0.629	0.639
UQ	21	16290601	16290900	0.156	1.169	0.950	0.594
UQ	21	16347901	16348200	1.521	2.291	1.086	0.994
UQ	21	16396201	16396500	2.076	1.238	0.876	1.481
UQ	21	16520401	16520700	0.507	0.185	0.210	0.056
UQ	21	16758601	16758900	1.719	2.791	3.119	3.478
UQ	21	16786201	16786500	1.120	0.474	0.453	0.335
UQ	21	16820401	16820700	7.596	5.164	5.274	5.710
UQ	21	17435701	17436000	1.568	0.983	2.134	2.277
UQ	21	17589901	17590200	0.586	0.234	0.127	0.284
UQ	21	17827801	17828100	0.132	0.515	0.239	0.248
UQ	21	18502201	18502500	0.903	0.347	0.424	0.254

UQ	21	18573901	18574200	0.984	0.069	0.613	0.299
UQ	21	18653101	18653400	0.608	0.303	0.263	0.223
UQ	21	18932701	18933000	0.106	0.258	0.609	0.360
UQ	21	20693101	20693400	0.477	0.232	0.127	0.142
UQ	21	20717701	20718000	0.450	0.096	0.127	0.056
UQ	21	21404701	21405000	0.771	0.609	0.243	0.304
UQ	21	21489001	21489300	0.823	0.562	0.560	0.583
UQ	21	21756001	21756300	0.611	0.350	0.263	0.335
UQ	21	21917101	21917400	0.560	0.303	0.185	0.664
UQ	21	21946201	21946500	0.505	0.257	0.267	0.284
UQ	21	22379701	22380000	0.423	0.140	0.103	0.112
UQ	21	22680301	22680600	0.982	1.195	0.506	0.416
UQ	21	22808701	22809000	0.557	0.328	0.239	0.329
UQ	21	23511301	23511600	1.300	0.517	0.597	0.446
UQ	21	23694901	23695200	1.223	0.843	0.707	0.862
UQ	21	23726701	23727000	0.505	0.584	0.267	0.360
UQ	21	23841001	23841300	0.848	0.375	0.535	0.497
UQ	21	24030601	24030900	1.219	0.611	0.658	0.436
UQ	21	24036301	24036600	0.531	0.096	0.272	0.056
UQ	21	24072001	24072300	2.047	0.766	0.947	0.329
UQ	21	24123301	24123600	0.373	0.584	0.214	0.137
UQ	21	24182101	24182400	0.903	0.633	0.531	0.669
UQ	21	24217501	24217800	3.341	1.220	1.716	1.800
UQ	21	24306001	24306300	3.641	2.676	2.073	2.007
UQ	21	24310201	24310500	0.718	0.307	0.239	0.350
UQ	21	25004401	25004700	0.955	1.613	1.304	0.157
UQ	21	25289101	25289400	0.715	0.260	0.243	0.259
UQ	21	25502101	25502400	0.689	0.236	0.210	0.218
UQ	21	26234101	26234400	0.636	0.071	0.259	0.254
UQ	21	27243901	27244200	0.636	0.283	0.259	0.279
UQ	21	27352801	27353100	2.420	1.448	1.674	1.688
UQ	21	27572701	27573000	0.503	0.230	0.214	0.223
UQ	21	27783901	27784200	0.186	0.468	0.555	0.360
UQ	21	27829201	27829500	0.584	0.234	0.312	0.193
UQ	21	27992401	27992700	1.011	1.240	0.749	0.725
UQ	21	28071601	28071900	0.932	0.629	0.428	0.446
UQ	21	28297801	28298100	1.491	0.983	0.510	1.009
UQ	21	28520101	28520400	0.477	0.303	0.263	0.218
UQ	21	28569001	28569300	0.077	0.491	0.267	0.441
UQ	21	28777201	28777500	3.670	2.523	3.020	2.997
UQ	21	28859101	28859400	2.594	1.188	0.856	1.460
UQ	21	28926001	28926300	1.032	0.258	0.366	0.289
UQ	21	28943701	28944000	2.397	3.083	3.168	3.224
UQ	21	28965601	28965900	1.891	3.744	2.570	3.458
UQ	21	28975501	28975800	0.505	1.073	0.802	1.075
UQ	21	28980901	28981200	0.000	0.330	0.667	0.319
UQ	21	29016601	29016900	0.186	0.654	0.502	0.502
UQ	21	29022001	29022300	1.274	0.567	0.403	0.497
UQ	21	29183701	29184000	0.850	0.725	0.584	0.690
UQ	21	29268001	29268300	3.294	2.452	2.521	2.702
UQ	21	29538901	29539200	0.716	0.352	0.292	0.213
UQ	21	30316801	30317100	0.692	0.185	0.140	0.193
UQ	21	30363001	30363300	0.771	0.283	0.276	0.391
UQ	21	31231201	31231500	1.032	0.418	0.448	0.507
UQ	21	31410901	31411200	1.063	1.568	0.778	0.690
UQ	21	31504801	31505100	0.902	0.399	0.370	0.162
UQ	21	31915801	31916100	0.745	0.491	0.156	0.522
UQ	21	32000701	32001000	0.905	0.425	0.317	0.299
UQ	21	32271301	32271600	0.292	0.584	0.560	0.416
UQ	21	32310901	32311200	1.892	1.221	1.021	1.206
UQ	21	32315701	32316000	0.794	0.352	0.160	0.360
UQ	21	32476201	32476500	1.921	1.163	1.061	0.791
UQ	21	32775001	32775300	1.065	0.629	0.682	0.274
UQ	21	32970301	32970600	1.589	0.466	0.604	0.882
UQ	21	33043501	33043800	2.473	1.772	1.687	1.511
UQ	21	33043801	33044100	4.993	3.388	3.406	3.240
UQ	21	33080401	33080700	1.491	2.761	3.208	2.008

UQ	21	33090901	33091200	1.358	1.890	0.987	0.877
UQ	21	33096901	33097200	1.240	0.331	0.234	0.259
UQ	21	33232801	33233100	4.072	2.881	3.105	3.463
UQ	21	33381301	33381600	0.687	0.279	0.136	0.193
UQ	21	33785701	33786000	4.678	5.424	3.221	3.322
UQ	21	33925801	33926100	0.424	0.702	0.872	0.832
UQ	21	34089601	34089900	1.728	0.863	1.206	1.374
UQ	21	34233301	34233600	1.197	1.051	0.769	0.771
UQ	21	34270801	34271100	0.931	0.328	0.214	0.264
UQ	21	34344001	34344300	1.281	0.768	0.720	0.553
UQ	21	34489501	34489800	2.363	1.776	1.358	1.419
UQ	21	34634401	34634700	0.929	1.171	0.740	1.192
UQ	21	34701001	34701300	0.265	0.562	0.054	0.634
UQ	21	34767301	34767600	0.610	0.281	0.189	0.324
UQ	21	34811101	34811400	0.800	0.352	0.473	0.335
UQ	21	34852501	34852800	0.879	0.375	0.361	0.391
UQ	21	35163001	35163300	0.558	0.303	0.317	0.446
UQ	21	35250601	35250900	0.878	0.423	0.403	0.426
UQ	21	35777701	35778000	0.477	0.071	0.160	0.167
UQ	21	35845201	35845500	1.678	0.588	0.765	0.923
UQ	21	36013501	36013800	0.318	0.165	0.531	0.466
UQ	21	36218401	36218700	1.389	0.307	0.604	0.452
UQ	21	36218701	36219000	4.358	0.787	1.514	1.642
UQ	21	36271801	36272100	0.560	0.139	0.234	0.329
UQ	21	36609601	36609900	1.884	1.090	0.847	1.100
UQ	21	37182901	37183200	0.615	0.212	0.239	0.274
UQ	21	38058301	38058600	1.247	0.818	0.716	0.791
UQ	21	38373901	38374200	0.398	0.910	0.078	0.497
UQ	21	39021601	39021900	2.418	2.075	2.818	1.430
UQ	21	39101101	39101400	0.560	0.114	0.103	0.076
UQ	21	39459301	39459600	0.876	0.448	0.288	0.315
UQ	21	39463501	39463800	0.874	0.472	0.185	0.391
UQ	21	39468601	39468900	0.848	0.562	0.584	0.329
UQ	21	39508201	39508500	0.902	0.421	0.415	0.461
UQ	21	39609901	39610200	0.905	0.491	0.691	0.801
UQ	21	39626401	39626700	2.609	1.287	1.839	1.577
UQ	21	39658501	39658800	0.692	0.421	0.267	0.299
UQ	21	39907801	39908100	0.186	0.701	0.872	0.715
UQ	21	40310101	40310400	3.194	2.705	2.040	1.688
UQ	21	40369801	40370100	1.434	0.796	0.794	1.192
UQ	21	40492801	40493100	0.507	0.163	0.181	0.162
UQ	21	40532701	40533000	0.586	0.279	0.185	0.299
UQ	21	41147701	41148000	0.240	0.749	0.477	0.477
UQ	21	41281801	41282100	0.052	0.234	0.448	0.218
UQ	21	41524801	41525100	2.636	3.808	1.921	2.175
UQ	21	41584801	41585100	1.386	0.659	0.798	0.537
UQ	21	41626801	41627100	3.273	2.624	1.908	2.454
UQ	21	41655301	41655600	1.357	1.073	0.872	0.832
UQ	21	42347101	42347400	1.592	0.888	0.945	0.943
UQ	21	42536401	42536700	0.345	0.633	0.078	0.081
UQ	21	42580201	42580500	1.973	0.997	3.429	3.280
UQ	21	42592501	42592800	0.637	0.397	0.350	0.279
UQ	21	42739201	42739500	1.629	0.766	0.847	0.877
UQ	21	42945901	42946200	3.025	1.496	2.448	2.520
UQ	21	43069501	43069800	0.160	0.395	0.346	0.335
UQ	21	43156801	43157100	1.334	0.588	0.468	0.675
UQ	21	43232101	43232400	2.389	1.633	1.547	1.764
UQ	21	43483501	43483800	2.579	1.686	0.802	1.177
UQ	21	43560901	43561200	0.637	1.873	1.324	1.009
UQ	21	43569001	43569300	0.666	0.208	0.301	0.223
UQ	21	43659001	43659300	1.197	1.450	1.830	1.435
UQ	21	43895401	43895700	1.625	1.244	1.188	0.857
UQ	21	43903501	43903800	5.169	3.248	4.204	4.264
UQ	21	44048401	44048700	1.200	0.815	0.794	0.801
UQ	21	44289901	44290200	0.558	0.142	0.218	0.218
UQ	21	44332201	44332500	0.398	0.444	0.453	0.497
UQ	21	44583601	44583900	0.797	0.513	0.613	1.267

UQ	21	45058801	45059100	0.213	0.468	0.132	0.335
UQ	21	45470701	45471000	1.147	0.000	1.964	0.177
UQ	21	45582901	45583200	5.899	4.570	4.222	3.803
UQ	21	45619201	45619500	1.836	1.101	1.534	1.541
UQ	21	46518601	46518900	0.721	0.401	0.317	0.081
UQ	21	46638301	46638600	1.382	1.075	0.934	0.756
UQ	21	46707001	46707300	2.570	1.206	0.613	0.497
UQ	21	46935601	46935900	4.788	3.444	2.780	5.760
UQ	21	46990201	46990500	0.744	0.465	0.296	0.254
UQ	21	47038801	47039100	0.876	1.124	1.329	0.588
UQ	21	47106301	47106600	0.586	0.446	0.428	0.248
UQ	21	47157601	47157900	2.817	1.676	1.850	2.180
UQ	21	47190301	47190600	3.807	2.124	2.486	2.393
UQ	21	47325001	47325300	0.852	0.448	0.531	0.385
UQ	21	47422801	47423100	3.912	2.324	2.358	2.009
UQ	21	47436301	47436600	1.807	1.335	1.123	1.460
UQ	21	47442001	47442300	0.369	1.427	0.720	0.715
UQ	21	47488501	47488800	2.954	0.790	0.938	1.835
UQ	21	47497201	47497500	1.013	0.560	0.354	0.583
UQ	21	47548201	47548500	1.620	0.980	0.753	1.044
UQ	21	47561101	47561400	0.477	0.281	0.160	0.167
UQ	21	48003001	48003300	4.678	3.770	3.830	3.878
UQ	21	48476701	48477000	1.215	2.156	2.485	2.231
UQ	21	48477001	48477300	0.371	1.077	1.008	0.999
UQ	21	48701701	48702000	2.078	0.794	1.534	1.399
UQ	21	48844201	48844500	0.718	0.330	0.419	0.380
UQ	21	49348501	49348800	0.718	1.452	1.099	2.048
UQ	21	49822801	49823100	1.439	1.075	1.781	1.688
UQ	21	49879501	49879800	0.984	0.628	0.671	0.608
UQ	22	85801	86100	0.213	0.468	0.617	0.578
UQ	22	645301	645600	0.881	0.376	0.473	0.421
UQ	22	1650001	1650300	0.611	0.302	1.086	0.218
UQ	22	3212701	3213000	1.223	0.794	0.856	0.765
UQ	22	3586201	3586500	0.534	0.210	0.292	0.781
UQ	22	5242201	5242500	0.584	0.258	0.296	0.243
UQ	22	5384401	5384700	0.663	0.354	0.214	0.243
UQ	22	5662201	5662500	2.104	1.208	1.106	1.151
UQ	22	5699701	5700000	1.458	0.611	0.560	0.872
UQ	22	5829301	5829600	0.744	0.397	0.296	0.608
UQ	22	6066601	6066900	1.676	1.075	1.003	2.393
UQ	22	6129601	6129900	5.980	5.087	4.838	4.158
UQ	22	6929701	6930000	0.077	0.305	0.531	0.299
UQ	22	7237501	7237800	1.384	0.605	0.992	0.466
UQ	22	7404301	7404600	1.065	0.560	0.535	0.436
UQ	22	8052001	8052300	2.370	1.770	0.896	1.714
UQ	22	8528401	8528700	0.665	0.376	0.448	0.472
UQ	22	8536501	8536800	0.505	0.725	0.642	0.279
UQ	22	8979901	8980200	0.369	0.910	0.638	0.608
UQ	22	9269101	9269400	0.586	0.352	0.296	0.391
UQ	22	9811801	9812100	0.319	0.513	0.502	0.497
UQ	22	9830101	9830400	0.536	0.047	0.160	0.218
UQ	22	9830401	9830700	0.640	0.069	0.263	0.248
UQ	22	9862801	9863100	0.373	0.586	0.132	0.081
UQ	22	9963001	9963300	0.452	0.560	0.321	0.167
UQ	22	10196701	10197000	0.931	0.120	0.526	0.203
UQ	22	10825501	10825800	0.505	0.303	0.288	0.248
UQ	22	10899901	10900200	4.276	2.682	2.735	3.007
UQ	22	10937401	10937700	3.344	1.830	1.590	1.693
UQ	22	11076601	11076900	0.240	1.495	0.856	1.080
UQ	22	12049501	12049800	0.586	0.257	0.165	0.193
UQ	22	12469201	12469500	0.531	0.187	0.189	0.284
UQ	22	12481801	12482100	2.391	2.871	4.158	3.260
UQ	22	12849301	12849600	3.764	2.364	1.846	2.165
UQ	22	12950701	12951000	4.075	2.648	2.171	2.561
UQ	22	12951001	12951300	5.015	3.611	3.085	3.200
UQ	22	12965401	12965700	0.749	0.258	0.218	0.254
UQ	22	13834801	13835100	0.529	1.217	1.008	0.983

UQ	22	13959901	13960200	0.823	0.442	0.481	0.472
UQ	22	14160901	14161200	1.678	2.111	0.695	1.055
UQ	22	15016201	15016500	0.450	0.212	0.185	0.142
UQ	22	15065701	15066000	0.321	0.749	0.798	0.583
UQ	22	15103201	15103500	2.318	0.875	0.959	0.881
UQ	22	15179101	15179400	0.531	0.328	0.263	0.243
UQ	22	16236001	16236300	0.853	1.169	1.300	1.050
UQ	22	16236301	16236600	1.144	1.427	1.826	1.445
UQ	22	16623901	16624200	3.247	4.094	4.122	3.859
UQ	22	17182801	17183100	0.211	0.446	0.448	0.497
UQ	22	17477101	17477400	0.186	0.444	0.078	0.274
UQ	22	17650201	17650500	2.549	1.470	1.480	1.201
UQ	22	17658901	17659200	1.826	0.768	0.769	0.669
UQ	22	17659801	17660100	0.908	0.281	0.267	0.375
UQ	22	17836201	17836500	0.529	0.185	0.160	0.056
UQ	22	17953201	17953500	3.002	2.064	2.158	1.221
UQ	22	18095701	18096000	2.263	1.468	1.514	1.647
UQ	22	18357001	18357300	0.690	0.584	0.317	0.329
UQ	22	18582001	18582300	0.639	0.163	0.390	0.385
UQ	22	20618401	20618700	0.586	1.101	0.876	0.963
UQ	22	20710201	20710500	0.639	0.236	0.234	0.142
UQ	22	20771401	20771700	0.347	0.773	0.662	0.583
UQ	22	20872801	20873100	2.818	1.817	2.073	2.089
UQ	22	20873101	20873400	3.273	2.298	2.403	2.515
UQ	22	21922501	21922800	1.334	0.865	1.835	2.079
UQ	22	21930901	21931200	1.089	0.794	1.222	0.938
UQ	22	22199701	22200000	0.953	0.279	0.453	0.289
UQ	22	22203901	22204200	1.223	1.309	1.037	1.161
UQ	22	22394101	22394400	3.139	2.594	2.102	1.825
UQ	22	22668001	22668300	0.081	0.326	0.477	0.279
UQ	22	23942701	23943000	0.744	0.421	0.477	0.441
UQ	22	24268501	24268800	0.716	0.399	0.321	0.355
UQ	22	26078101	26078400	0.531	0.232	0.346	0.254
UQ	22	27247801	27248100	0.347	0.165	0.506	0.137
UQ	22	27502501	27502800	2.870	2.064	2.370	1.633
UQ	22	28144501	28144800	0.507	0.352	0.292	0.193
UQ	22	29384401	29384700	0.294	0.701	0.535	0.472
UQ	22	29617201	29617500	0.345	0.515	0.535	0.198
UQ	22	30273901	30274200	1.783	0.914	0.738	0.603
UQ	22	30276901	30277200	3.647	0.725	0.765	0.958
UQ	22	30692401	30692700	0.586	0.796	0.321	0.690
UQ	22	31612201	31612500	1.278	0.820	0.707	0.639
UQ	22	31619701	31620000	1.223	0.727	1.037	0.776
UQ	22	31907401	31907700	0.800	1.425	1.246	1.562
UQ	22	32027701	32028000	0.476	0.116	0.160	0.051
UQ	22	32153101	32153400	0.611	0.513	0.292	0.360
UQ	22	32516401	32516700	1.145	0.702	0.330	0.477
UQ	22	33966301	33966600	0.184	0.843	0.448	0.715
UQ	22	34401301	34401600	0.910	0.236	0.247	0.294
UQ	22	34439101	34439400	2.133	1.588	1.489	1.328
UQ	22	34530901	34531200	1.673	0.976	0.486	1.019
UQ	22	34536001	34536300	0.666	0.165	0.160	0.106
UQ	22	35569501	35569800	1.065	0.607	0.954	0.608
UQ	22	36837001	36837300	0.055	0.423	0.633	0.380
UQ	22	36984601	36984900	0.347	0.257	0.531	0.664
UQ	22	37666801	37667100	1.245	0.607	0.410	0.391
UQ	22	38143801	38144100	0.450	0.234	0.054	0.198
UQ	22	38275501	38275800	2.689	1.562	2.156	2.099
UQ	22	38522701	38523000	0.505	0.212	0.165	0.254
UQ	22	38573401	38573700	3.697	3.253	4.389	2.997
UQ	22	38629201	38629500	1.199	1.517	1.538	1.572
UQ	22	38749801	38750100	1.274	0.584	0.765	0.751
UQ	22	38957401	38957700	7.611	6.402	6.204	6.282
UQ	22	38986501	38986800	0.476	0.120	0.103	0.086
UQ	22	39843001	39843300	0.665	0.818	0.584	0.472
UQ	22	39882001	39882300	2.607	3.615	3.669	3.129
UQ	22	39954301	39954600	1.037	0.397	0.477	0.538

UQ	22	40339201	40339500	0.745	0.539	0.399	0.198
UQ	22	40417801	40418100	0.505	0.210	0.272	0.137
UQ	22	40804801	40805100	0.108	0.444	0.263	0.385
UQ	22	40975801	40976100	0.349	1.006	0.740	0.771
UQ	22	41329801	41330100	0.507	0.094	0.156	0.086
UQ	22	41379901	41380200	3.615	2.965	2.435	2.936
UQ	22	41559601	41559900	1.249	0.867	1.037	1.471
UQ	22	41776201	41776500	1.382	2.169	1.888	0.847
UQ	22	42222301	42222600	0.319	0.493	0.633	0.553
UQ	22	42514801	42515100	0.477	0.236	0.189	0.162
UQ	22	42528601	42528900	2.317	1.189	1.567	1.343
UQ	22	42599101	42599400	5.541	4.143	3.269	3.939
UQ	22	42608701	42609000	1.334	1.751	0.885	2.079
UQ	22	42615301	42615600	0.242	0.607	0.588	0.558
UQ	22	42960901	42961200	0.557	0.258	0.214	0.335
UQ	22	43116901	43117200	1.573	1.002	0.914	1.019
UQ	22	43127701	43128000	1.092	0.773	0.609	0.573
UQ	22	43133701	43134000	0.718	1.143	0.901	0.527
UQ	22	43137301	43137600	6.030	4.139	4.584	4.705
UQ	22	43177201	43177500	1.542	1.056	0.885	0.700
UQ	22	43177801	43178100	2.045	1.513	1.505	1.491
UQ	22	43333501	43333800	5.717	3.974	4.795	4.061
UQ	22	43530301	43530600	1.037	0.538	0.497	0.360
UQ	22	43647601	43647900	0.908	0.257	0.481	0.086
UQ	22	43903201	43903500	0.053	0.378	0.399	0.614
UQ	22	44165701	44166000	5.094	3.330	3.007	3.351
UQ	22	44248201	44248500	1.089	0.588	0.308	0.578
UQ	22	44301001	44301300	5.662	5.025	4.820	4.482
UQ	22	44351401	44351700	3.830	2.337	3.045	2.865
UQ	22	44557501	44557800	0.561	0.236	0.160	0.248
UQ	22	44781601	44781900	3.796	2.446	2.261	2.261
UQ	22	44881501	44881800	4.578	5.448	6.692	5.313
UQ	22	44938501	44938800	0.610	0.303	0.234	0.279
UQ	22	44984701	44985000	0.424	0.678	0.691	0.659
UQ	22	45084901	45085200	1.278	0.701	0.370	0.608
UQ	22	45093601	45093900	0.720	0.369	0.317	0.335
UQ	22	45098101	45098400	3.134	2.038	2.036	2.687
UQ	22	45325801	45326100	0.507	0.747	1.008	0.877
UQ	22	45442201	45442500	0.505	0.118	0.116	0.167
UQ	22	45461401	45461700	7.790	4.776	5.497	6.049
UQ	22	45592801	45593100	0.850	0.534	0.214	0.492
UQ	22	45613501	45613800	0.690	1.006	0.399	0.882
UQ	22	45663301	45663600	4.147	2.023	2.366	1.714
UQ	22	45824101	45824400	2.871	1.602	2.209	1.491
UQ	22	46068901	46069200	0.824	0.517	0.510	0.360
UQ	22	46208401	46208700	2.258	2.901	2.633	2.931
UQ	22	46336201	46336500	0.079	0.493	0.317	0.391
UQ	22	46524901	46525200	0.661	1.405	1.451	1.541
UQ	22	46563901	46564200	0.769	0.538	0.510	0.497
UQ	22	46660201	46660500	2.207	1.781	1.576	1.293
UQ	22	46666801	46667100	2.396	2.055	1.674	2.130
UQ	22	46820401	46820700	0.345	0.444	0.210	0.248
UQ	22	46823401	46823700	1.223	0.564	0.363	0.588
UQ	22	46825801	46826100	2.023	1.613	1.230	1.678
UQ	22	46839901	46840200	0.610	0.184	0.267	0.162
UQ	22	46862401	46862700	2.370	2.922	1.797	2.875
UQ	22	46890001	46890300	0.720	0.208	0.156	0.193
UQ	22	47023501	47023800	0.477	0.069	0.078	0.187
UQ	22	47036401	47036700	0.744	0.468	0.370	0.340
UQ	22	47123701	47124000	0.797	0.238	0.169	0.238
UQ	22	47127301	47127600	0.292	0.890	0.653	0.690
UQ	22	47303401	47303700	6.107	4.207	4.563	4.609
UQ	22	47867101	47867400	0.560	0.352	0.774	0.309
UQ	22	48000001	48000300	4.360	2.967	3.181	2.956
UQ	22	48105001	48105300	1.781	2.354	0.510	0.786
UQ	22	48192901	48193200	3.246	2.124	2.633	2.419
UQ	22	48305101	48305400	0.398	0.985	0.794	0.740

UQ	22	48430501	48430800	1.310	0.609	0.279	0.472
UQ	22	48477601	48477900	1.357	0.865	0.992	0.634
UQ	22	48520501	48520800	0.344	0.586	0.111	0.664
UQ	22	48526801	48527100	0.960	1.379	0.575	0.533
UQ	22	48533401	48533700	0.903	0.539	0.428	0.634
UQ	22	48678601	48678900	1.063	0.300	0.546	0.299
UQ	22	48696901	48697200	8.745	6.771	6.546	6.694
UQ	22	48697201	48697500	4.611	3.478	2.742	3.503
UQ	22	48992701	48993000	1.382	0.380	0.542	0.288
UQ	22	49086301	49086600	1.941	1.261	1.032	1.663
UQ	22	49264501	49264800	0.292	0.446	0.160	0.441
UQ	22	49272901	49273200	5.478	4.744	4.758	3.980
UQ	22	49453801	49454100	1.250	1.877	0.671	0.578
UQ	22	49670101	49670400	3.909	1.362	2.227	2.495
UQ	22	49691701	49692000	2.901	3.394	2.004	2.135
UQ	22	50007601	50007900	0.692	1.262	0.983	1.095
UQ	22	50071201	50071500	5.082	1.985	2.037	1.881
UQ	22	50232901	50233200	0.720	0.330	0.267	0.259
UQ	22	50253301	50253600	0.582	0.191	0.054	0.086
UQ	22	50635801	50636100	2.423	1.395	1.835	1.886
UQ	22	50804101	50804400	2.317	1.749	1.736	2.981
UQ	23	96601	96900	0.477	0.116	0.214	0.248
UQ	23	487201	487500	0.318	1.034	1.090	0.690
UQ	23	487501	487800	0.371	0.985	1.032	0.796
UQ	23	495901	496200	0.557	0.212	0.049	0.198
UQ	23	828001	828300	6.191	5.194	4.755	5.303
UQ	23	1047301	1047600	2.212	3.735	2.831	2.864
UQ	23	1243501	1243800	2.128	1.725	1.271	1.566
UQ	23	1280401	1280700	3.162	2.236	4.118	4.264
UQ	23	1471801	1472100	0.263	5.676	4.418	4.159
UQ	23	1601101	1601400	3.876	1.566	2.328	1.526
UQ	23	1642201	1642500	2.234	1.148	0.519	0.968
UQ	23	1703101	1703400	1.412	0.912	0.745	0.761
UQ	23	1719301	1719600	0.450	0.163	0.107	0.137
UQ	23	2060101	2060400	1.279	0.629	0.495	0.461
UQ	23	2060401	2060700	3.400	2.077	1.601	1.864
UQ	23	2082001	2082300	2.128	0.423	0.519	0.947
UQ	23	2091601	2091900	0.558	0.373	0.263	0.248
UQ	23	2286901	2287200	5.023	5.977	5.780	6.115
UQ	23	2389801	2390100	0.450	0.045	0.160	0.187
UQ	23	2464201	2464500	0.615	0.258	0.210	0.061
UQ	23	2562901	2563200	0.984	0.350	0.448	0.598
UQ	23	2606701	2607000	2.337	1.586	1.897	1.323
UQ	23	2726701	2727000	1.834	2.296	1.465	1.430
UQ	23	2745901	2746200	0.800	0.352	0.288	0.193
UQ	23	2953801	2954100	6.614	5.079	5.337	5.476
UQ	23	3061501	3061800	0.476	1.009	0.160	0.142
UQ	23	3384901	3385200	0.555	0.116	0.025	0.081
UQ	23	3385201	3385500	3.258	1.491	1.197	0.791
UQ	23	3387901	3388200	1.678	2.845	2.773	2.818
UQ	23	3388201	3388500	1.470	3.139	2.673	3.523
UQ	23	3389401	3389700	0.584	0.257	0.185	0.223
UQ	23	3390301	3390600	0.744	0.466	0.444	0.416
UQ	23	3451801	3452100	0.532	0.279	0.132	0.274
UQ	23	3618901	3619200	3.944	2.244	2.363	2.165
UQ	23	3726001	3726300	6.169	5.102	5.315	5.111
UQ	23	3952501	3952800	0.695	0.258	0.263	0.193
UQ	23	4207801	4208100	0.134	0.444	0.428	0.329
UQ	23	4635601	4635900	0.690	0.538	0.399	0.254
UQ	23	5175301	5175600	0.108	0.631	0.448	0.507
UQ	23	5190301	5190600	1.299	0.376	0.526	0.639
UQ	23	5190601	5190900	2.073	1.009	0.970	0.786
UQ	23	5742901	5743200	0.240	0.444	0.107	0.112
UQ	23	6636601	6636900	0.720	0.305	0.107	0.329
UQ	23	6941701	6942000	1.065	1.496	0.667	0.852
UQ	23	6943501	6943800	0.345	0.163	0.214	0.553
UQ	23	7833301	7833600	0.055	0.682	0.317	0.335

UQ	23	8308801	8309100	1.110	0.071	0.025	0.031
UQ	23	8309101	8309400	0.423	0.049	0.058	0.025
UQ	23	8456701	8457000	0.611	0.139	0.885	0.025
UQ	23	8661001	8661300	0.769	0.277	0.127	0.173
UQ	23	8661301	8661600	0.903	0.541	0.317	0.396
UQ	23	8682901	8683200	0.908	0.258	0.152	0.193
UQ	23	9684301	9684600	0.479	0.794	0.774	0.634
UQ	23	11598001	11598300	2.362	0.882	1.246	0.898
UQ	23	12833701	12834000	0.477	0.118	0.210	0.167
UQ	23	14382301	14382600	0.318	0.890	0.642	0.573
UQ	23	14498401	14498700	0.558	0.167	0.267	0.132
UQ	23	15908101	15908400	0.584	0.210	0.852	0.832
UQ	23	16082101	16082400	0.982	0.538	0.555	0.695
UQ	23	16849801	16850100	0.560	0.096	0.160	0.112
UQ	23	17279101	17279400	0.160	0.375	0.531	0.411
UQ	23	17497801	17498100	0.477	0.189	0.054	0.137
UQ	23	17959501	17959800	0.531	0.257	0.350	0.248
UQ	23	18187201	18187500	1.300	0.023	0.049	0.000
UQ	23	18187501	18187800	1.448	0.045	0.049	0.061
UQ	23	18195601	18195900	1.276	0.024	0.132	0.152
UQ	23	18195901	18196200	0.695	0.069	0.029	0.182
UQ	23	18562501	18562800	3.356	2.264	2.559	1.348
UQ	23	20523001	20523300	0.850	0.678	0.457	0.690
UQ	23	21447901	21448200	1.276	0.749	0.671	0.816
UQ	23	21449401	21449700	0.613	0.165	0.399	0.329
UQ	23	21610201	21610500	0.345	0.605	0.584	0.162
UQ	23	21866101	21866400	0.373	0.513	0.078	0.162
UQ	23	21973801	21974100	0.292	1.568	0.843	0.791
UQ	23	21979801	21980100	0.505	0.189	0.234	0.243
UQ	23	21984601	21984900	0.345	0.444	0.214	0.112
UQ	23	21996001	21996300	0.663	0.069	0.317	0.497
UQ	23	22212901	22213200	6.665	5.666	4.282	5.709
UQ	23	22259101	22259400	2.705	1.557	1.469	1.668
UQ	23	22261501	22261800	0.876	0.302	0.633	0.553
UQ	23	22387501	22387800	1.195	0.656	0.395	0.507
UQ	23	22775701	22776000	0.874	1.450	1.320	1.653
UQ	23	23220301	23220600	0.529	0.213	0.214	0.193
UQ	23	24011701	24012000	1.831	1.377	1.353	1.186
UQ	23	24626101	24626400	0.773	0.258	0.288	0.360
UQ	23	24908701	24909000	0.450	0.139	0.165	0.137
UQ	23	25275901	25276200	0.160	0.517	0.481	0.385
UQ	23	26717701	26718000	1.963	0.989	0.448	0.953
UQ	23	29244001	29244300	0.639	0.328	0.292	0.299
UQ	23	29544301	29544600	0.534	0.303	0.156	0.274
UQ	23	30552001	30552300	1.278	0.657	0.843	0.725
UQ	23	30603601	30603900	0.477	0.212	0.132	0.106
UQ	23	30946801	30947100	0.715	1.849	1.527	2.196
UQ	23	31505701	31506000	1.544	0.850	0.502	0.817
UQ	23	31961101	31961400	0.531	0.890	0.424	0.304
UQ	23	32061301	32061600	2.713	1.290	1.077	1.020
UQ	23	32061601	32061900	0.637	0.120	0.058	0.117
UQ	23	32482201	32482500	0.398	0.539	0.267	0.776
UQ	23	33070501	33070800	0.986	0.543	0.584	0.441
UQ	23	33611101	33611400	2.099	1.245	0.749	1.064
UQ	23	34644001	34644300	0.586	0.210	0.247	0.132
UQ	23	34731001	34731300	1.063	0.676	0.453	0.675
UQ	23	34740601	34740900	1.331	2.292	2.107	1.897
UQ	23	34897501	34897800	10.206	9.093	8.489	8.651
UQ	23	35487901	35488200	0.318	0.491	0.695	0.112
UQ	23	35895001	35895300	0.505	0.747	0.243	0.309
UQ	23	36195301	36195600	0.532	0.212	0.214	0.304
UQ	23	36907201	36907500	1.274	0.773	1.037	1.055
UQ	23	36945601	36945900	0.908	0.538	0.535	0.522
UQ	23	37499701	37500000	2.954	3.628	3.377	3.453
UQ	23	37530901	37531200	2.973	1.888	1.558	2.023
UQ	23	37894801	37895100	0.557	0.281	0.185	0.193
UQ	23	38176201	38176500	0.613	0.230	0.218	0.142

UQ	23	39036301	39036600	0.826	0.305	0.395	0.522
UQ	23	39356701	39357000	0.799	0.204	0.230	0.076
UQ	23	39357001	39357300	0.611	0.114	0.147	0.025
UQ	23	39406501	39406800	1.039	0.403	0.453	0.405
UQ	23	39510901	39511200	1.326	0.725	0.983	0.664
UQ	23	39789301	39789600	0.666	0.258	0.403	0.187
UQ	23	39917101	39917400	0.476	0.069	0.160	0.198
UQ	23	40125901	40126200	0.266	0.564	0.662	0.466
UQ	23	40328401	40328700	0.826	0.446	0.481	0.472
UQ	23	40441801	40442100	1.410	0.511	0.827	1.050
UQ	23	40442101	40442400	2.191	1.240	1.177	1.303
UQ	23	41013601	41013900	0.345	0.680	0.156	0.162
UQ	23	41209201	41209500	1.037	0.583	0.593	0.243
UQ	23	41453701	41454000	4.493	2.579	2.200	2.602
UQ	23	41585701	41586000	6.098	4.401	4.534	5.040
UQ	23	42437401	42437700	0.744	0.466	0.341	0.472
UQ	23	42489301	42489600	1.252	0.330	0.740	0.730
UQ	23	43040401	43040700	0.665	0.466	0.296	0.446
UQ	23	43065001	43065300	0.450	1.573	1.344	1.871
UQ	23	43275301	43275600	2.396	2.920	3.801	4.913
UQ	23	43482601	43482900	1.542	0.667	0.662	0.659
UQ	23	43655701	43656000	2.447	2.030	1.349	2.023
UQ	23	44470801	44471100	2.095	0.468	2.468	1.714
UQ	23	44895301	44895600	0.134	0.444	0.370	0.309
UQ	23	44901601	44901900	0.907	0.303	0.214	0.375
UQ	23	45288301	45288600	0.213	0.583	0.082	0.360
UQ	23	45398701	45399000	1.673	0.938	1.251	0.791
UQ	23	45643201	45643500	1.755	1.021	0.954	0.927
UQ	23	45775201	45775500	0.613	0.116	0.156	0.248
UQ	23	45776101	45776400	0.640	0.324	0.317	0.329
UQ	23	45885001	45885300	0.690	0.843	0.317	0.527
UQ	23	45954901	45955200	0.507	0.116	0.185	0.335
UQ	23	46251001	46251300	1.515	0.618	0.332	0.669
UQ	23	46331701	46332000	2.049	0.820	1.035	0.918
UQ	23	46558501	46558800	1.516	1.798	1.300	1.141
UQ	23	46656901	46657200	0.905	0.193	0.194	0.243
UQ	23	46797601	46797900	1.570	1.127	1.164	1.136
UQ	23	46802401	46802700	0.319	0.656	0.424	0.690
UQ	23	47200201	47200500	1.594	0.895	0.856	0.648
UQ	23	47413201	47413500	0.852	0.425	0.432	0.279
UQ	23	47433001	47433300	0.584	0.236	0.214	0.284
UQ	23	47874901	47875200	0.613	0.446	0.370	0.223
UQ	23	47943001	47943300	0.132	0.862	0.740	0.659
UQ	23	47958901	47959200	0.534	0.891	1.032	1.100
UQ	23	48497401	48497700	1.007	1.684	0.403	0.517
UQ	23	48500401	48500700	1.034	0.303	0.662	0.350
UQ	23	48602401	48602700	1.221	0.938	0.930	0.664
UQ	23	48625801	48626100	6.946	5.420	5.053	5.217
UQ	23	48681301	48681600	0.694	0.234	0.370	0.228
UQ	23	48815401	48815700	0.823	0.468	0.531	0.614
UQ	23	48853201	48853500	0.292	0.583	0.667	0.573
UQ	23	48882601	48882900	7.038	5.853	4.866	6.227
UQ	23	49440601	49440900	1.192	0.555	0.551	0.218
UQ	23	49696801	49697100	1.884	1.122	0.925	1.070
UQ	23	49870201	49870500	1.063	0.607	0.633	0.801
UQ	23	49968001	49968300	0.747	1.075	0.531	0.411
UQ	23	50912401	50912700	0.318	0.491	0.185	0.441
UQ	23	50920201	50920500	0.400	0.657	0.798	0.943
UQ	23	50946601	50946900	1.037	1.571	1.411	1.856
UQ	23	51121501	51121800	1.489	0.980	0.568	0.907
UQ	23	51129901	51130200	0.984	2.289	3.188	2.591
UQ	23	51138901	51139200	1.116	0.654	0.794	1.298
UQ	23	51282001	51282300	2.902	2.165	1.197	1.171
UQ	23	51697201	51697500	0.587	0.097	0.185	0.106
UQ	23	51734401	51734700	0.824	0.517	0.424	0.547
UQ	23	52029601	52029900	0.613	0.328	0.107	0.360
UQ	23	52213201	52213500	0.606	1.261	1.715	1.161

UQ	23	52236601	52236900	0.213	0.493	0.638	0.051
UQ	23	52688701	52689000	0.426	0.163	0.239	0.745
UQ	23	52873201	52873500	0.582	0.257	0.239	0.187
UQ	23	52905301	52905600	0.824	0.465	0.263	0.340
UQ	23	53070301	53070600	0.481	1.847	1.139	1.172
UQ	23	53070601	53070900	0.240	0.959	0.448	0.639
UQ	23	53114401	53114700	0.344	0.515	0.823	0.608
UQ	23	53128801	53129100	1.353	1.054	0.847	1.024
UQ	23	53668501	53668800	0.481	0.257	0.156	0.086
UQ	23	53829601	53829900	1.195	1.616	2.078	1.465
UQ	23	54114301	54114600	3.086	2.382	2.526	2.176
UQ	23	54273301	54273600	1.463	0.959	0.823	1.080
UQ	23	54509401	54509700	0.716	1.309	1.608	1.267
UQ	23	54615601	54615900	0.266	0.891	0.724	0.796
UQ	23	54873901	54874200	0.986	0.311	0.252	0.228
UQ	23	54874801	54875100	1.249	0.631	0.930	0.806
UQ	23	54875101	54875400	3.023	1.757	2.151	1.536
UQ	23	55144501	55144800	2.315	2.010	1.349	1.172
UQ	23	55144801	55145100	2.683	1.895	1.255	1.314
UQ	23	55360201	55360500	2.978	1.822	1.295	1.759
UQ	23	55360501	55360800	3.909	2.199	1.427	2.028
UQ	23	55410301	55410600	0.479	0.538	0.185	0.664
UQ	23	55868401	55868700	2.371	2.755	1.830	1.987
UQ	23	55931101	55931400	3.155	2.150	1.852	1.952
UQ	23	55971301	55971600	1.810	1.115	0.950	1.009
UQ	23	56771701	56772000	0.053	0.446	0.379	0.416
UQ	23	57221701	57222000	1.805	1.025	1.025	0.497
UQ	23	57249601	57249900	0.720	0.305	0.185	0.254
UQ	23	57294901	57295200	2.260	3.165	3.382	3.245
UQ	23	57468601	57468900	0.718	0.257	0.267	0.396
UQ	23	57539101	57539400	0.953	0.421	0.531	0.446
UQ	23	57558901	57559200	0.131	0.515	0.399	0.472
UQ	23	57811501	57811800	1.736	1.028	0.921	1.024
UQ	23	57914101	57914400	2.578	2.057	2.205	1.714
UQ	23	57916201	57916500	3.562	3.956	2.419	5.126
UQ	23	57916801	57917100	3.904	1.760	2.477	2.571
UQ	23	58006201	58006500	1.357	1.704	0.789	1.050
UQ	23	58088701	58089000	0.824	0.420	0.370	0.416
UQ	23	58156501	58156800	0.186	0.444	0.374	0.355
UQ	23	58673701	58674000	2.707	1.748	2.049	1.917
UQ	23	58757401	58757700	1.091	0.674	0.486	0.720
UQ	23	58758601	58758900	5.288	6.668	6.713	6.677
UQ	23	58907401	58907700	0.235	0.631	0.642	0.628
UQ	23	59022601	59022900	3.991	1.388	0.622	1.044
UQ	23	59022901	59023200	1.492	0.479	0.350	0.340
UQ	23	59051701	59052000	0.584	0.120	0.127	0.193
UQ	23	59225101	59225400	0.400	0.794	0.136	0.198
UQ	23	59963701	59964000	1.518	2.420	2.682	2.666
UQ	23	60022801	60023100	0.560	0.071	0.267	0.268
UQ	23	60244501	60244800	3.965	3.064	2.468	3.170
UQ	23	60510601	60510900	0.611	0.444	0.321	0.223
UQ	23	60569101	60569400	1.520	0.251	0.622	0.679
UQ	23	60677401	60677700	0.747	0.260	0.181	0.360
UQ	23	60705901	60706200	1.733	1.356	2.156	1.374
UQ	23	60981001	60981300	0.531	0.796	0.214	0.329
UQ	23	61361101	61361400	2.554	3.036	3.364	3.215
UQ	23	61465501	61465800	1.515	0.350	0.544	0.522
UQ	23	61591201	61591500	7.464	5.263	5.985	5.669
UQ	23	61629901	61630200	0.848	0.515	0.473	0.527
UQ	23	61680301	61680600	0.745	0.369	0.267	0.132
UQ	23	61741501	61741800	0.373	0.584	0.263	0.578
UQ	23	61802101	61802400	10.068	9.185	9.247	11.276
UQ	23	61812301	61812600	5.856	5.085	3.460	4.543
UQ	23	61818001	61818300	1.836	1.429	1.300	1.212
UQ	24	50701	51000	0.265	0.513	0.481	0.578
UQ	24	64201	64500	1.353	1.006	2.073	1.724
UQ	24	171001	171300	1.037	0.586	0.658	0.715

UQ	24	324901	325200	4.591	1.476	1.921	2.077
UQ	24	618901	619200	1.487	0.747	1.168	1.303
UQ	24	650401	650700	0.586	0.232	0.230	0.254
UQ	24	991201	991500	3.526	2.450	2.287	2.393
UQ	24	1039501	1039800	2.086	0.227	3.665	3.337
UQ	24	1254301	1254600	0.134	0.654	0.617	0.715
UQ	24	1472401	1472700	0.720	0.302	0.214	0.299
UQ	24	1783201	1783500	2.670	1.395	1.094	1.383
UQ	24	1809001	1809300	0.823	1.261	0.531	0.639
UQ	24	2212501	2212800	1.970	1.377	1.456	1.597
UQ	24	2237701	2238000	0.694	0.957	0.403	0.385
UQ	24	2570401	2570700	0.134	0.442	0.321	0.578
UQ	24	2664301	2664600	1.008	1.618	1.801	1.642
UQ	24	2903701	2904000	2.234	0.364	0.488	0.284
UQ	24	2955301	2955600	0.510	0.023	0.054	0.056
UQ	24	3140401	3140700	1.329	0.772	0.731	0.623
UQ	24	3171601	3171900	0.852	1.193	0.745	1.131
UQ	24	3437101	3437400	0.105	0.444	0.399	0.299
UQ	24	3614101	3614400	0.611	0.773	0.930	0.857
UQ	24	3970201	3970500	0.371	0.701	0.691	0.725
UQ	24	4045501	4045800	0.769	0.399	0.263	0.193
UQ	24	4057801	4058100	0.953	0.420	0.444	0.477
UQ	24	4226101	4226400	0.265	0.446	0.560	0.441
UQ	24	4739401	4739700	0.531	0.049	0.132	0.274
UQ	24	4800001	4800300	1.623	0.775	0.749	0.725
UQ	24	4958101	4958400	1.120	0.676	0.662	0.659
UQ	24	5173201	5173500	0.850	0.586	0.103	0.279
UQ	24	5174101	5174400	1.970	1.240	1.373	1.374
UQ	24	5232601	5232900	1.515	1.332	1.835	2.236
UQ	24	5494501	5494800	2.412	1.017	0.633	0.907
UQ	24	5610601	5610900	0.663	0.328	0.210	0.421
UQ	24	5961001	5961300	1.115	1.822	0.881	1.359
UQ	24	6177601	6177900	1.815	0.738	0.379	0.655
UQ	24	6387001	6387300	0.349	0.935	0.716	0.669
UQ	24	6733501	6733800	0.878	0.281	2.180	0.319
UQ	24	8317201	8317500	0.186	0.562	0.321	0.411
UQ	24	8402701	8403000	0.821	0.236	0.403	0.304
UQ	24	9136801	9137100	2.075	1.686	0.642	1.618
UQ	24	9321901	9322200	0.744	0.217	0.103	0.167
UQ	24	9594301	9594600	0.611	1.124	0.350	0.994
UQ	24	9691801	9692100	1.623	2.219	0.823	0.832
UQ	24	9749101	9749400	1.276	0.324	0.765	0.720
UQ	24	9804901	9805200	0.745	0.395	0.234	0.198
UQ	24	9864001	9864300	1.110	1.937	0.468	0.426
UQ	24	9941401	9941700	3.220	2.364	2.410	2.099
UQ	24	9988801	9989100	1.118	1.708	0.876	1.638
UQ	24	10357501	10357800	0.611	0.234	0.292	0.391
UQ	24	10466401	10466700	0.902	2.090	1.650	1.688
UQ	24	10469101	10469400	0.558	0.281	0.317	0.441
UQ	24	10635001	10635300	0.265	0.843	0.531	0.472
UQ	24	10677601	10677900	0.402	0.678	0.740	0.690
UQ	24	10998001	10998300	3.572	1.673	2.271	2.484
UQ	24	11035501	11035800	2.523	1.911	1.884	1.906
UQ	24	11076601	11076900	0.345	0.564	0.185	0.167
UQ	24	11113501	11113800	2.044	2.688	1.621	2.500
UQ	24	11191201	11191500	0.636	0.305	0.185	0.385
UQ	24	11499301	11499600	1.171	0.753	0.827	0.776
UQ	24	11659801	11660100	1.405	0.890	0.827	0.994
UQ	24	11882401	11882700	0.586	0.257	0.247	0.142
UQ	24	12813001	12813300	0.079	0.867	0.682	0.517
UQ	24	13367701	13368000	0.531	0.165	0.346	0.086
UQ	24	13449901	13450200	0.531	0.213	0.165	0.223
UQ	24	13737901	13738200	1.484	0.283	0.325	0.771
UQ	24	14033701	14034000	2.749	1.557	1.422	1.374
UQ	24	14110501	14110800	4.683	2.841	3.389	3.554
UQ	24	15098401	15098700	1.229	0.232	0.123	0.132
UQ	24	15098701	15099000	2.854	0.286	0.239	0.263

UQ	24	15225301	15225600	1.623	2.776	2.962	2.368
UQ	24	15326401	15326700	0.474	0.000	0.082	0.000
UQ	24	15751801	15752100	0.481	0.212	0.078	0.198
UQ	24	16308901	16309200	1.808	0.886	0.328	0.917
UQ	24	16415101	16415400	3.830	4.652	4.530	4.842
UQ	24	16458901	16459200	0.268	0.957	0.448	0.497
UQ	24	16889401	16889700	0.160	0.442	0.424	0.553
UQ	24	16972501	16972800	2.207	0.116	0.709	0.701
UQ	24	16972801	16973100	2.710	0.373	1.252	0.858
UQ	24	17015101	17015400	2.257	1.011	1.382	0.741
UQ	24	17160301	17160600	1.063	0.676	0.390	0.664
UQ	24	17478301	17478600	0.764	2.519	2.941	2.623
UQ	24	17614201	17614500	0.536	0.163	0.000	0.162
UQ	24	17897401	17897700	0.240	0.140	0.477	0.137
UQ	24	17946001	17946300	1.063	0.483	0.292	0.248
UQ	24	18033601	18033900	0.507	0.213	0.160	0.193
UQ	24	19959601	19959900	0.529	0.163	0.185	0.254
UQ	24	20056201	20056500	3.639	2.926	2.666	3.123
UQ	24	20263201	20263500	0.850	0.515	0.399	0.461
UQ	24	20508901	20509200	0.903	0.238	0.321	0.461
UQ	24	20903101	20903400	0.613	0.328	0.370	0.329
UQ	24	21188701	21189000	2.470	2.826	1.991	1.942
UQ	24	21626401	21626700	0.847	0.444	0.239	0.411
UQ	24	21888001	21888300	0.957	0.562	0.716	0.740
UQ	24	21999001	21999300	0.720	0.350	0.185	0.477
UQ	24	22530601	22530900	0.339	1.517	1.772	1.319
UQ	24	22615501	22615800	1.649	0.846	0.881	1.303
UQ	24	22818001	22818300	0.347	0.513	0.876	0.639
UQ	24	23805001	23805300	0.852	0.354	0.272	0.345
UQ	24	23892901	23893200	0.534	0.185	0.243	0.218
UQ	24	23986201	23986500	0.186	0.605	0.749	0.502
UQ	24	24042001	24042300	0.744	0.489	0.341	0.309
UQ	24	24542401	24542700	0.584	0.350	0.049	0.324
UQ	24	24741601	24741900	3.694	2.899	2.242	2.600
UQ	24	24741901	24742200	2.396	1.777	1.629	1.338
UQ	24	24815101	24815400	0.215	0.421	0.695	0.416
UQ	24	24866401	24866700	0.905	0.629	0.477	0.228
UQ	24	24998701	24999000	1.749	0.952	0.945	0.862
UQ	24	25218601	25218900	1.570	1.004	0.992	1.105
UQ	24	25294501	25294800	1.147	0.674	0.399	0.628
UQ	24	25483201	25483500	1.808	0.919	0.580	0.964
UQ	24	25551901	25552200	9.478	6.427	7.310	6.953
UQ	24	26091001	26091300	1.724	0.841	1.032	0.947
UQ	24	26091301	26091600	1.941	1.431	1.119	1.197
UQ	24	26283001	26283300	4.910	3.261	2.613	3.093
UQ	24	26447701	26448000	0.665	0.281	0.341	0.157
UQ	24	26558401	26558700	1.355	1.051	0.856	0.725
UQ	24	26784301	26784600	0.450	0.092	0.000	0.101
UQ	24	26863801	26864100	1.231	2.763	2.588	2.281
UQ	24	27102001	27102300	4.767	3.553	3.850	5.695
UQ	24	27209101	27209400	1.724	1.238	1.028	1.217
UQ	24	27222901	27223200	3.904	2.182	2.229	2.667
UQ	24	27462601	27462900	0.318	0.444	0.214	0.112
UQ	24	27555901	27556200	1.011	0.605	0.682	0.690
UQ	24	27996601	27996900	1.833	1.071	0.707	0.878
UQ	24	28047301	28047600	0.398	1.056	0.905	0.740
UQ	24	28307701	28308000	1.382	0.538	0.263	0.462
UQ	24	28308001	28308300	0.982	0.184	0.254	0.254
UQ	24	28361101	28361400	0.134	0.305	0.399	0.583
UQ	24	28378501	28378800	0.744	0.536	0.346	0.355
UQ	24	28408801	28409100	2.025	1.708	1.387	1.633
UQ	24	28513501	28513800	1.358	0.773	0.588	1.075
UQ	24	28857901	28858200	0.477	0.184	0.156	0.106
UQ	24	29156401	29156700	1.065	0.744	0.152	0.690
UQ	24	29398501	29398800	1.039	1.264	1.514	1.267
UQ	24	29788201	29788500	0.771	1.362	1.148	1.856
UQ	24	29819701	29820000	0.586	0.208	0.189	0.132

UQ	24	29942401	29942700	3.168	4.441	4.576	4.365
UQ	24	29972701	29973000	0.826	0.257	0.312	0.198
UQ	24	30045301	30045600	0.799	0.230	0.341	0.335
UQ	24	30677401	30677700	1.250	0.817	0.802	0.893
UQ	24	31008301	31008600	0.053	0.515	0.210	0.223
UQ	24	31740601	31740900	0.481	0.212	0.054	0.198
UQ	24	31931101	31931400	0.373	0.258	0.560	0.223
UQ	24	31947901	31948200	0.507	0.213	0.210	0.081
UQ	24	32082301	32082600	0.642	0.213	0.107	0.112
UQ	24	32462401	32462700	2.128	1.285	1.280	1.414
UQ	24	32526001	32526300	0.665	0.212	0.194	0.304
UQ	24	32697601	32697900	0.400	0.843	0.292	0.248
UQ	24	32829301	32829600	0.531	0.165	0.214	0.254
UQ	24	32924401	32924700	3.139	2.714	2.114	2.353
UQ	24	33122701	33123000	1.171	0.938	0.794	0.690
UQ	24	33213001	33213300	0.986	0.633	0.408	0.522
UQ	24	33249601	33249900	4.391	2.950	3.504	3.940
UQ	24	33662701	33663000	0.505	0.656	0.292	0.279
UQ	24	33667801	33668100	0.507	0.210	0.132	0.137
UQ	24	34068301	34068600	0.584	0.210	0.156	0.284
UQ	24	34513201	34513500	0.266	0.420	0.185	0.193
UQ	24	34536901	34537200	3.531	2.354	2.287	2.667
UQ	24	34851001	34851300	0.239	0.538	0.448	0.411
UQ	24	35079001	35079300	0.800	0.285	0.424	0.421
UQ	24	35263201	35263500	1.408	0.702	1.139	0.852
UQ	24	35651401	35651700	1.226	0.607	0.667	0.684
UQ	24	35734501	35734800	1.249	0.800	0.905	0.639
UQ	24	35922301	35922600	0.824	0.423	0.350	0.497
UQ	24	36531601	36531900	0.505	0.886	0.774	0.857
UQ	24	37014601	37014900	1.171	0.747	0.707	1.465
UQ	24	37087501	37087800	0.611	0.446	0.453	0.360
UQ	24	37205401	37205700	3.120	1.914	1.672	1.613
UQ	24	37205701	37206000	2.614	1.504	0.930	1.424
UQ	24	37305301	37305600	0.694	0.234	0.321	0.228
UQ	24	37360801	37361100	0.529	0.068	0.185	0.137
UQ	24	37366201	37366500	2.959	1.474	1.864	1.581
UQ	24	37544101	37544400	1.199	0.890	0.609	0.776
UQ	24	37608601	37608900	5.695	3.770	3.959	4.229
UQ	24	37736101	37736400	2.901	1.530	5.070	1.662
UQ	24	37884901	37885200	4.712	3.383	3.126	3.194
UQ	24	37925701	37926000	5.636	5.374	4.282	4.969
UQ	24	38154601	38154900	5.408	3.980	3.792	3.934
UQ	24	38154901	38155200	2.030	1.090	0.749	0.968
UQ	24	38465401	38465700	0.373	0.749	0.555	0.695
UQ	24	39259801	39260100	5.296	4.210	3.681	3.894
UQ	24	39396301	39396600	2.822	2.148	2.082	2.114
UQ	24	39426001	39426300	0.957	1.442	1.777	1.886
UQ	24	39597301	39597600	0.902	0.631	0.642	0.522
UQ	24	39711601	39711900	8.748	9.814	5.988	13.719
UQ	24	39832801	39833100	1.726	0.697	0.745	0.973
UQ	24	39874801	39875100	2.078	2.832	1.645	2.434
UQ	24	39991801	39992100	0.055	0.399	0.263	0.527
UQ	24	40121401	40121700	0.771	0.515	0.526	0.411
UQ	24	40160401	40160700	3.878	3.025	3.110	2.804
UQ	24	40203001	40203300	4.733	4.205	3.723	4.132
UQ	24	40319701	40320000	1.728	1.233	1.197	1.242
UQ	24	40365301	40365600	2.817	2.409	2.517	2.079
UQ	24	40762201	40762500	1.597	1.191	1.835	1.247
UQ	24	40991401	40991700	2.396	3.999	3.506	3.190
UQ	24	41015401	41015700	1.358	1.028	0.914	0.553
UQ	24	41170501	41170800	3.007	1.882	1.813	1.202
UQ	24	41539501	41539800	0.847	1.614	1.353	1.485
UQ	24	41673001	41673300	0.853	0.444	0.555	0.380
UQ	24	41673301	41673600	1.728	1.000	1.099	1.014
UQ	24	41843401	41843700	0.237	0.468	0.584	0.502
UQ	25	261001	261300	1.007	0.255	0.357	0.243
UQ	25	438601	438900	1.865	1.053	0.979	0.760

UQ	25	730801	731100	0.637	0.375	0.127	0.117
UQ	25	895501	895800	0.771	0.376	0.584	0.502
UQ	25	925201	925500	0.744	0.983	0.987	0.938
UQ	25	1465801	1466100	0.477	0.305	0.025	0.248
UQ	25	1528201	1528500	1.278	0.440	0.176	0.370
UQ	25	1571101	1571400	0.979	0.345	0.074	0.193
UQ	25	1620601	1620900	0.639	0.236	0.292	0.243
UQ	25	1780201	1780500	0.720	0.376	0.346	0.254
UQ	25	1789501	1789800	0.826	0.324	0.239	0.426
UQ	25	2789401	2789700	0.134	0.536	0.584	0.436
UQ	25	3276601	3276900	1.379	1.051	0.716	0.882
UQ	25	3380101	3380400	1.010	1.193	0.711	0.801
UQ	25	3444901	3445200	0.666	0.446	0.341	0.106
UQ	25	3653701	3654000	0.721	0.373	0.366	0.385
UQ	25	3883201	3883500	0.429	0.094	0.049	0.086
UQ	25	3884101	3884400	0.902	0.466	0.526	0.634
UQ	25	4088401	4088700	0.776	0.257	0.214	0.304
UQ	25	4236001	4236300	0.771	0.584	0.399	0.385
UQ	25	4608301	4608600	1.118	0.890	0.798	0.882
UQ	25	4627501	4627800	2.703	1.401	1.855	1.602
UQ	25	4636501	4636800	0.345	0.468	0.535	0.882
UQ	25	4662601	4662900	1.168	0.397	0.802	0.801
UQ	25	5069101	5069400	0.692	0.210	0.234	0.335
UQ	25	5327101	5327400	0.929	0.350	0.740	1.131
UQ	25	5355601	5355900	3.678	1.204	2.216	0.638
UQ	25	5495401	5495700	0.826	0.560	0.267	0.441
UQ	25	5523001	5523300	0.639	0.513	0.881	0.441
UQ	25	5802601	5802900	1.565	1.098	1.066	0.933
UQ	25	5807101	5807400	0.689	0.212	0.399	0.355
UQ	25	5911501	5911800	1.439	0.348	0.406	0.547
UQ	25	5917801	5918100	0.155	0.955	0.633	0.913
UQ	25	6034501	6034800	0.929	1.287	0.575	0.355
UQ	25	6843901	6844200	1.303	0.236	0.584	0.882
UQ	25	7145101	7145400	0.507	0.865	1.032	0.832
UQ	25	7589401	7589700	0.423	0.163	0.160	0.081
UQ	25	7800001	7800300	0.134	0.446	0.374	0.466
UQ	25	7815901	7816200	0.505	0.258	0.210	0.162
UQ	25	7948501	7948800	0.745	0.444	0.194	0.360
UQ	25	8048101	8048400	1.437	0.378	0.474	0.339
UQ	25	8628901	8629200	0.879	0.423	0.395	0.385
UQ	25	8831101	8831400	1.812	0.942	1.045	1.166
UQ	25	9072001	9072300	1.303	1.004	0.983	1.111
UQ	25	9188701	9189000	2.949	3.722	4.394	2.306
UQ	25	9211801	9212100	0.742	0.281	0.317	0.223
UQ	25	9402901	9403200	7.632	6.168	6.017	8.315
UQ	25	9441901	9442200	0.398	1.731	1.255	1.075
UQ	25	9772201	9772500	0.878	0.489	0.531	0.309
UQ	25	9966001	9966300	0.558	0.723	0.267	0.355
UQ	25	9990901	9991200	1.144	1.793	2.069	1.653
UQ	25	9995701	9996000	0.694	0.187	0.223	0.203
UQ	25	12486001	12486300	0.477	0.210	0.243	0.167
UQ	25	12518101	12518400	1.223	0.772	0.506	0.639
UQ	25	12882301	12882600	0.639	0.232	0.283	0.187
UQ	25	13038601	13038900	1.034	0.420	0.428	0.619
UQ	25	13369501	13369800	1.358	0.848	0.662	0.826
UQ	25	13441201	13441500	1.808	1.352	1.094	1.516
UQ	25	14511601	14511900	1.571	0.732	0.834	0.628
UQ	25	14613001	14613300	1.329	0.862	0.716	0.943
UQ	25	15169201	15169500	0.531	0.116	0.049	0.182
UQ	25	15229501	15229800	0.769	0.069	0.218	0.264
UQ	25	15979501	15979800	0.876	1.358	1.801	1.633
UQ	25	16089001	16089300	0.529	0.097	0.160	0.086
UQ	25	16091701	16092000	1.165	0.258	0.160	0.406
UQ	25	18061801	18062100	0.557	0.210	0.181	0.213
UQ	25	18386701	18387000	1.749	0.609	0.736	0.786
UQ	25	18387001	18387300	1.355	0.451	0.617	0.411
UQ	25	19788001	19788300	1.460	0.421	0.934	0.700

UQ	25	19997701	19998000	0.557	0.255	0.181	0.218
UQ	25	20364301	20364600	0.211	0.562	0.510	0.492
UQ	25	22747501	22747800	0.026	0.283	0.555	0.446
UQ	25	22768201	22768500	0.718	0.513	0.823	0.441
UQ	25	23597701	23598000	0.450	0.097	0.054	0.031
UQ	25	23598001	23598300	1.005	0.241	0.078	0.122
UQ	25	24226501	24226800	0.799	0.584	0.633	0.446
UQ	25	25570501	25570800	2.746	3.321	1.522	2.241
UQ	25	25609801	25610100	0.666	0.303	0.156	0.268
UQ	25	25974301	25974600	0.747	0.302	0.239	0.527
UQ	25	26316301	26316600	0.534	0.794	0.720	0.999
UQ	25	26602201	26602500	5.136	2.864	4.204	4.371
UQ	25	26624401	26624700	0.532	0.118	0.881	0.142
UQ	25	27289801	27290100	0.505	0.212	0.058	0.821
UQ	25	27435901	27436200	0.508	0.118	0.000	0.187
UQ	25	27527401	27527700	1.303	0.697	0.682	0.426
UQ	25	27546601	27546900	0.213	0.629	0.424	0.938
UQ	25	28141201	28141500	0.531	0.165	0.346	0.112
UQ	25	28346101	28346400	0.639	0.262	0.292	0.279
UQ	25	29952301	29952600	0.186	0.798	0.453	0.502
UQ	25	30353401	30353700	0.932	0.631	0.497	0.578
UQ	25	30437701	30438000	2.021	1.144	1.197	1.399
UQ	25	30476701	30477000	0.294	0.491	0.560	0.639
UQ	25	30852601	30852900	0.053	0.562	0.156	0.193
UQ	25	31365301	31365600	0.771	0.448	0.272	0.142
UQ	25	31408201	31408500	0.505	0.258	0.189	0.137
UQ	25	31428901	31429200	0.507	0.234	0.239	0.218
UQ	25	31791601	31791900	0.052	0.489	0.341	0.355
UQ	25	31828801	31829100	0.692	0.118	0.341	0.279
UQ	25	32329801	32330100	0.692	0.193	0.214	0.248
UQ	25	32355001	32355300	0.534	0.230	0.210	0.112
UQ	25	32768101	32768400	0.077	0.395	0.419	0.385
UQ	25	32772001	32772300	1.755	0.586	0.814	1.374
UQ	25	33210301	33210600	2.236	2.012	1.942	1.881
UQ	25	33711301	33711600	0.295	0.678	0.613	0.751
UQ	25	33765001	33765300	1.789	3.248	0.992	2.515
UQ	25	33860401	33860700	0.745	0.184	0.325	0.309
UQ	25	34241701	34242000	1.116	0.721	0.691	0.634
UQ	25	34327201	34327500	5.904	3.435	5.137	4.679
UQ	25	34400101	34400400	0.532	0.210	0.054	0.259
UQ	25	34433101	34433400	1.515	1.635	1.144	0.968
UQ	25	34489801	34490100	2.073	1.519	1.645	1.485
UQ	25	34496401	34496700	1.996	1.023	1.180	0.907
UQ	25	34505401	34505700	0.450	0.090	0.160	0.112
UQ	25	34618801	34619100	0.768	0.258	0.276	0.304
UQ	25	34626901	34627200	5.403	4.272	4.002	3.920
UQ	25	34646701	34647000	1.408	0.751	0.729	0.679
UQ	25	34724701	34725000	1.199	2.030	1.719	1.755
UQ	25	34781101	34781400	4.443	2.793	3.583	3.625
UQ	25	35222401	35222700	1.247	0.817	2.020	0.948
UQ	25	35230801	35231100	0.505	0.165	0.288	0.254
UQ	25	35255101	35255400	0.292	0.656	0.132	0.193
UQ	25	35360701	35361000	0.557	0.281	0.136	0.198
UQ	25	35451301	35451600	2.341	0.333	0.940	0.223
UQ	25	35585101	35585400	0.718	0.959	0.876	0.578
UQ	25	35984401	35984700	1.802	2.733	2.742	3.341
UQ	25	36671701	36672000	2.286	1.521	1.942	1.562
UQ	25	36672001	36672300	1.941	1.311	1.592	1.222
UQ	25	37011001	37011300	2.441	1.541	1.362	1.338
UQ	25	37297801	37298100	0.665	0.139	0.267	0.385
UQ	25	37353901	37354200	1.063	0.729	0.613	0.497
UQ	25	38248501	38248800	2.231	3.137	3.094	3.899
UQ	25	38323201	38323500	1.060	0.538	0.419	0.355
UQ	25	38606701	38607000	0.850	0.352	0.370	0.304
UQ	25	38800801	38801100	5.288	3.828	3.496	4.634
UQ	25	38833201	38833500	0.560	0.163	0.103	0.147
UQ	25	39163501	39163800	0.611	0.096	0.000	0.000

UQ	25	40218001	40218300	1.783	1.174	1.148	1.156
UQ	25	40580401	40580700	1.891	1.380	0.809	1.217
UQ	25	40589701	40590000	1.247	0.657	0.613	0.705
UQ	25	40593301	40593600	1.668	0.659	0.802	0.785
UQ	25	40614001	40614300	1.097	0.331	0.218	0.279
UQ	25	40704901	40705200	1.705	1.101	0.838	0.918
UQ	25	40965601	40965900	0.665	0.069	0.346	0.391
UQ	25	41351101	41351400	0.720	0.423	0.448	0.472
UQ	25	42036601	42036900	2.021	1.594	0.745	1.039
UQ	25	42253801	42254100	0.610	0.305	0.156	0.223
UQ	25	42397501	42397800	1.013	0.680	0.506	0.583
UQ	25	42444901	42445200	0.237	1.120	0.477	0.826
UQ	25	42702901	42703200	0.345	0.491	0.160	0.279
UQ	25	42798901	42799200	1.118	1.332	1.440	1.800
UQ	25	42831301	42831600	0.211	0.652	0.682	1.111
UQ	25	42915301	42915600	2.208	1.472	1.342	1.663
UQ	25	42942301	42942600	0.823	0.275	0.374	0.051
UQ	25	42965101	42965400	1.300	0.820	0.321	0.431
UQ	25	43068001	43068300	0.532	0.350	0.239	0.796
UQ	25	43124101	43124400	2.851	1.613	1.777	1.282
UQ	25	43135801	43136100	3.057	1.882	1.543	2.140
UQ	25	43197301	43197600	1.726	0.931	1.052	1.039
UQ	25	43274401	43274700	1.415	0.657	0.959	0.472
UQ	25	43359901	43360200	2.312	1.783	1.538	1.374
UQ	25	43861801	43862100	0.240	0.609	0.662	0.436
UQ	25	44087401	44087700	0.369	0.961	0.765	1.111
UQ	25	44551501	44551800	0.984	0.817	0.687	0.639
UQ	25	44745001	44745300	3.746	5.932	5.250	5.202
UQ	25	44806801	44807100	0.187	0.352	0.642	0.472
UQ	25	44853001	44853300	1.520	0.440	0.729	0.324
UQ	25	44902801	44903100	0.799	0.142	0.386	0.167
UQ	25	44910901	44911200	1.805	1.217	0.995	1.075
UQ	25	44920501	44920800	0.584	0.350	0.288	0.335
UQ	25	45014101	45014400	1.674	1.452	0.934	0.796
UQ	25	45022501	45022800	1.802	1.006	1.193	1.242
UQ	25	45166201	45166500	3.854	3.227	2.468	2.617
UQ	25	45282001	45282300	0.665	0.303	0.239	0.355
UQ	26	212401	212700	1.010	0.375	0.720	0.700
UQ	26	226201	226500	0.481	0.049	0.082	0.031
UQ	26	327901	328200	0.587	0.281	0.111	0.198
UQ	26	406501	406800	0.852	0.489	0.580	0.441
UQ	26	534601	534900	2.208	1.094	1.505	1.399
UQ	26	715201	715500	0.345	0.609	0.506	0.578
UQ	26	729601	729900	0.319	0.444	0.103	0.112
UQ	26	936301	936600	2.123	1.092	1.030	1.212
UQ	26	990601	990900	1.858	0.532	0.876	0.827
UQ	26	1053601	1053900	0.876	0.168	0.107	0.263
UQ	26	1060501	1060800	0.690	0.260	0.218	0.304
UQ	26	1091701	1092000	2.257	1.420	1.266	1.293
UQ	26	1150201	1150500	0.984	1.144	0.633	0.720
UQ	26	1256401	1256700	0.215	0.470	0.613	0.466
UQ	26	1637401	1637700	0.908	1.354	1.489	1.404
UQ	26	1713601	1713900	4.518	2.013	1.458	2.496
UQ	26	1713901	1714200	1.571	0.908	0.390	0.537
UQ	26	1822801	1823100	1.731	0.912	1.003	1.323
UQ	26	1928701	1929000	4.449	2.240	2.586	2.966
UQ	26	1957201	1957500	0.481	0.118	0.107	0.198
UQ	26	2379601	2379900	0.398	0.607	0.136	0.000
UQ	26	2526901	2527200	0.423	0.792	0.774	0.913
UQ	26	4837801	4838100	0.613	0.350	0.243	0.416
UQ	26	5100001	5100300	1.970	1.272	0.740	0.857
UQ	26	5173801	5174100	0.505	0.092	0.152	0.081
UQ	26	5488201	5488500	1.655	0.637	0.811	0.461
UQ	26	5591101	5591400	1.092	0.491	0.633	0.628
UQ	26	5665201	5665500	1.889	3.025	3.036	3.006
UQ	26	5727901	5728200	2.287	1.362	1.563	1.566
UQ	26	6136801	6137100	1.166	0.721	0.457	0.720

UQ	26	6291601	6291900	1.037	0.680	0.609	0.360
UQ	26	7065301	7065600	0.531	0.260	0.103	0.167
UQ	26	7538701	7539000	0.718	0.210	0.424	0.391
UQ	26	7686601	7686900	0.769	0.446	0.502	0.497
UQ	26	7979701	7980000	0.211	0.583	0.428	0.634
UQ	26	8131201	8131500	1.115	0.633	0.633	0.771
UQ	26	9036001	9036300	0.186	0.444	0.346	0.720
UQ	26	11487901	11488200	0.561	0.213	0.160	0.162
UQ	26	11758201	11758500	2.157	3.010	2.575	3.478
UQ	26	11968201	11968500	0.876	0.331	0.272	0.263
UQ	26	12029101	12029400	1.089	0.494	0.671	0.497
UQ	26	12149101	12149400	0.797	0.493	0.667	0.497
UQ	26	12540001	12540300	0.106	0.586	0.292	0.335
UQ	26	12948001	12948300	0.960	2.015	1.514	1.663
UQ	26	13224901	13225200	2.952	2.292	2.028	2.327
UQ	26	13596001	13596300	1.884	1.259	0.678	0.999
UQ	26	13637401	13637700	3.033	2.592	2.521	2.160
UQ	26	13780201	13780500	2.604	3.133	3.549	3.590
UQ	26	13808401	13808700	0.694	0.348	0.296	0.248
UQ	26	13957801	13958100	1.757	2.079	1.387	1.348
UQ	26	15329701	15330000	0.507	0.118	0.049	0.137
UQ	26	15468001	15468300	0.955	1.661	1.407	1.501
UQ	26	15736801	15737100	0.957	0.635	0.584	0.695
UQ	26	16119601	16119900	0.800	0.185	0.263	0.416
UQ	26	16373101	16373400	0.615	0.350	0.160	0.137
UQ	26	17724001	17724300	0.450	0.727	0.132	0.147
UQ	26	17816701	17817000	1.276	0.820	0.444	0.882
UQ	26	17910001	17910300	1.539	3.141	2.546	3.194
UQ	26	17943001	17943300	0.239	1.195	0.867	0.832
UQ	26	18084601	18084900	0.347	0.631	0.531	0.218
UQ	26	18916201	18916500	0.610	0.238	0.205	0.137
UQ	26	20090701	20091000	0.587	0.184	0.111	0.218
UQ	26	20112601	20112900	0.055	0.470	0.667	0.299
UQ	26	20223301	20223600	0.561	0.069	0.098	0.025
UQ	26	20919601	20919900	0.716	0.420	0.457	0.441
UQ	26	21443101	21443400	1.597	0.980	1.148	1.024
UQ	26	22170301	22170600	0.634	1.217	1.447	1.328
UQ	26	23437801	23438100	0.690	0.395	0.292	0.329
UQ	26	24325801	24326100	2.554	3.409	3.340	4.263
UQ	26	24400201	24400500	1.305	2.051	2.905	1.891
UQ	26	24789301	24789600	1.170	0.633	0.765	0.771
UQ	26	24989701	24990000	0.161	0.515	0.374	0.446
UQ	26	25262101	25262400	0.587	0.210	0.210	0.167
UQ	26	25581301	25581600	1.032	0.000	0.000	0.000
UQ	26	25581601	25581900	5.149	0.000	0.000	0.000
UQ	26	25581901	25582200	2.956	0.000	0.000	0.000
UQ	26	25582501	25582800	0.423	0.000	0.000	0.000
UQ	26	25582801	25583100	0.687	0.000	0.000	0.000
UQ	26	27485401	27485700	0.531	0.350	0.049	0.198
UQ	26	27585001	27585300	1.011	0.534	0.662	0.466
UQ	26	27716401	27716700	1.545	1.030	1.094	1.029
UQ	26	28156801	28157100	0.618	0.069	0.025	0.025
UQ	26	28591801	28592100	0.213	0.466	0.477	0.360
UQ	26	28719901	28720200	1.276	2.392	0.671	0.756
UQ	26	29346601	29346900	0.213	0.493	0.374	0.583
UQ	26	29425801	29426100	2.631	1.708	1.822	1.916
UQ	26	29699701	29700000	2.550	1.493	1.489	1.100
UQ	26	29700001	29700300	1.757	0.914	1.226	0.948
UQ	26	29979001	29979300	0.131	0.515	0.588	0.461
UQ	26	30122401	30122700	0.560	1.193	1.193	1.307
UQ	26	30559801	30560100	0.531	0.049	0.160	0.106
UQ	26	30640801	30641100	4.691	2.901	2.782	3.311
UQ	26	30988201	30988500	0.721	0.234	0.317	0.218
UQ	26	31049401	31049700	0.426	0.560	0.156	0.248
UQ	26	31328401	31328700	0.452	0.629	0.185	0.279
UQ	26	31428601	31428900	0.718	0.373	0.350	0.385
UQ	26	31474801	31475100	5.591	3.716	4.347	4.213

UQ	26	31511701	31512000	1.091	0.676	0.613	0.405
UQ	26	31644901	31645200	0.294	0.468	0.531	0.553
UQ	26	31758901	31759200	0.929	0.376	0.160	0.274
UQ	26	31759201	31759500	1.882	0.588	0.370	0.593
UQ	26	31896001	31896300	1.252	0.401	0.629	0.756
UQ	26	31897201	31897500	0.905	0.539	0.461	0.578
UQ	26	32210401	32210700	5.025	3.107	1.304	3.970
UQ	26	32269501	32269800	2.370	1.892	1.723	1.724
UQ	26	32329501	32329800	0.613	0.257	0.424	0.309
UQ	26	32394301	32394600	0.878	0.448	0.510	0.502
UQ	26	32570401	32570700	1.434	1.706	0.613	1.151
UQ	26	32676001	32676300	0.797	0.444	0.283	0.466
UQ	26	32736601	32736900	8.619	4.726	6.882	5.152
UQ	26	32736901	32737200	1.762	0.745	0.979	0.710
UQ	26	33068101	33068400	0.476	3.002	2.588	1.669
UQ	26	33294601	33294900	0.319	0.629	0.453	0.472
UQ	26	33898801	33899100	0.561	0.187	0.107	0.198
UQ	26	34206001	34206300	0.319	0.442	0.025	0.193
UQ	26	34434901	34435200	0.852	0.116	0.000	0.167
UQ	26	34435201	34435500	0.979	0.184	0.029	0.106
UQ	26	34454701	34455000	1.171	0.749	0.526	0.791
UQ	26	34706701	34707000	0.560	0.120	0.000	0.117
UQ	26	34713301	34713600	1.036	0.257	0.374	0.649
UQ	26	34989601	34989900	0.957	0.510	0.379	0.340
UQ	26	35043901	35044200	2.468	1.802	1.850	1.790
UQ	26	35231701	35232000	2.129	1.703	2.604	1.795
UQ	26	35263201	35263500	0.692	0.281	0.214	0.421
UQ	26	35294101	35294400	0.852	0.562	0.662	0.502
UQ	26	35642101	35642400	1.781	2.264	2.834	2.596
UQ	26	35886601	35886900	0.186	0.609	0.341	0.416
UQ	26	35932201	35932500	2.287	2.034	1.937	1.993
UQ	26	36053701	36054000	0.692	0.303	0.321	0.294
UQ	26	36221401	36221700	2.157	1.281	1.835	2.576
UQ	26	36462601	36462900	0.053	0.656	0.243	0.254
UQ	26	36798301	36798600	3.193	2.339	2.276	2.424
UQ	26	36833101	36833400	0.452	0.071	0.876	0.162
UQ	26	36858901	36859200	2.930	1.571	1.890	1.633
UQ	26	37289101	37289400	2.076	1.523	0.967	2.439
UQ	26	37289401	37289700	1.862	1.354	1.041	1.714
UQ	26	37314301	37314600	1.089	0.543	0.408	0.446
UQ	26	37414201	37414500	0.613	0.303	0.136	0.142
UQ	26	37537201	37537500	1.120	0.489	0.127	0.284
UQ	26	37767001	37767300	0.586	0.397	0.239	0.385
UQ	26	37829401	37829700	2.712	0.991	0.990	1.099
UQ	26	37840501	37840800	0.426	0.656	0.658	0.968
UQ	26	37902001	37902300	3.613	1.757	0.976	1.855
UQ	26	38076301	38076600	0.503	0.140	0.321	0.279
UQ	26	38121001	38121300	0.532	0.189	0.181	0.106
UQ	26	38262601	38262900	1.465	0.796	0.872	1.217
UQ	26	38383801	38384100	0.555	0.023	0.132	0.193
UQ	26	38434201	38434500	3.362	1.407	0.720	1.322
UQ	26	38434501	38434800	1.650	0.751	0.359	0.532
UQ	26	38548801	38549100	0.666	1.266	0.905	0.913
UQ	26	38587801	38588100	1.545	2.429	2.748	2.544
UQ	26	38857501	38857800	0.481	0.024	0.082	0.117
UQ	26	38887501	38887800	0.928	0.631	0.263	0.497
UQ	26	38934601	38934900	7.340	3.778	6.335	5.110
UQ	26	38935201	38935500	4.894	1.639	3.834	2.947
UQ	26	39675001	39675300	2.543	0.837	0.749	0.772
UQ	26	40014301	40014600	1.197	0.678	0.189	0.852
UQ	26	40223101	40223400	1.036	0.633	0.424	0.614
UQ	26	40270201	40270500	5.157	3.941	2.093	6.399
UQ	26	40303201	40303500	0.878	0.438	0.481	0.411
UQ	26	40329901	40330200	0.398	0.818	0.963	0.669
UQ	26	40500301	40500600	1.357	0.867	0.823	0.873
UQ	26	40661701	40662000	0.584	1.566	0.909	1.206
UQ	26	40662301	40662600	1.144	0.704	0.580	0.268

UQ	26	40689301	40689600	3.989	3.386	3.043	3.123
UQ	26	40732201	40732500	0.477	0.798	0.798	0.887
UQ	26	40746301	40746600	2.544	1.154	1.164	1.009
UQ	26	41129701	41130000	0.160	0.466	0.374	0.279
UQ	26	41286001	41286300	1.810	1.167	1.079	1.161
UQ	26	41974501	41974800	0.211	0.629	0.399	0.472
UQ	26	41987401	41987700	1.566	1.261	0.823	1.131
UQ	26	42273901	42274200	1.492	1.006	1.028	0.902
UQ	26	42393901	42394200	2.255	1.315	1.551	0.304
UQ	26	42551101	42551400	0.505	0.234	0.288	0.254
UQ	26	42578401	42578700	0.586	0.420	0.399	0.416
UQ	26	42922501	42922800	1.063	0.725	0.613	0.461
UQ	26	42945001	42945300	0.613	0.118	0.395	0.355
UQ	26	43273501	43273800	1.089	0.440	0.390	0.482
UQ	26	43346701	43347000	0.266	0.491	0.428	0.106
UQ	26	43759201	43759500	0.186	0.328	0.477	0.411
UQ	26	43778101	43778400	0.799	1.146	1.110	1.105
UQ	26	43778401	43778700	0.824	1.098	1.217	1.197
UQ	26	43927201	43927500	0.452	0.865	0.662	0.553
UQ	3	6001	6300	0.476	0.959	0.925	1.136
UQ	3	109501	109800	0.611	0.303	0.502	0.335
UQ	3	111601	111900	0.640	0.303	0.370	0.441
UQ	3	223201	223500	4.498	3.184	3.426	3.787
UQ	3	1004701	1005000	6.521	5.006	5.244	3.746
UQ	3	1322401	1322700	4.400	2.781	2.550	2.707
UQ	3	1322701	1323000	6.174	3.673	3.301	4.257
UQ	3	1520401	1520700	2.562	1.639	1.173	1.537
UQ	3	1570801	1571100	2.635	0.461	0.428	0.162
UQ	3	1770001	1770300	1.249	0.936	1.032	0.751
UQ	3	1785901	1786200	2.925	2.092	1.777	2.043
UQ	3	2315701	2316000	2.315	1.335	1.460	1.136
UQ	3	2504101	2504400	1.120	0.678	0.588	0.472
UQ	3	2517601	2517900	0.903	0.586	0.510	0.553
UQ	3	2815501	2815800	0.639	0.281	0.350	0.355
UQ	3	2907901	2908200	2.234	1.665	1.023	1.379
UQ	3	3101701	3102000	3.079	1.700	1.391	1.856
UQ	3	3145201	3145500	0.584	0.232	0.136	0.284
UQ	3	3176401	3176700	4.341	2.098	1.599	1.615
UQ	3	3176701	3177000	2.157	1.403	0.609	0.582
UQ	3	3205801	3206100	0.505	0.348	0.189	0.112
UQ	3	3210001	3210300	3.670	2.736	2.592	2.561
UQ	3	3541801	3542100	0.800	0.442	0.403	0.411
UQ	3	3603301	3603600	2.865	1.195	1.837	1.430
UQ	3	3767101	3767400	0.534	0.142	0.132	0.162
UQ	3	3957901	3958200	0.771	0.609	0.263	0.583
UQ	3	4184101	4184400	0.929	0.701	0.642	0.690
UQ	3	4225501	4225800	0.958	0.487	0.346	0.553
UQ	3	4225801	4226100	3.378	1.918	2.163	2.778
UQ	3	4642801	4643100	0.319	0.794	0.132	0.583
UQ	3	4699201	4699500	3.663	2.553	1.875	2.530
UQ	3	4829401	4829700	4.018	6.338	7.984	7.617
UQ	3	4985101	4985400	3.514	2.199	2.278	2.035
UQ	3	5108101	5108400	0.955	0.562	0.716	0.715
UQ	3	5352301	5352600	0.398	0.513	0.107	0.248
UQ	3	5766001	5766300	0.426	0.562	0.214	0.583
UQ	3	5774401	5774700	0.350	1.099	0.896	1.014
UQ	3	5786101	5786400	1.491	2.154	0.898	0.826
UQ	3	5916901	5917200	0.134	0.539	0.588	0.385
UQ	3	5918101	5918400	1.408	1.006	1.099	0.695
UQ	3	6105601	6105900	2.075	1.360	1.558	1.348
UQ	3	6241501	6241800	0.718	0.348	0.325	0.472
UQ	3	6352801	6353100	1.195	0.566	0.506	0.913
UQ	3	6629701	6630000	8.226	6.612	6.364	6.825
UQ	3	6649501	6649800	4.506	2.673	6.238	8.273
UQ	3	6957601	6957900	1.568	1.939	2.406	2.241
UQ	3	7095001	7095300	0.692	1.049	1.333	1.156
UQ	3	7168801	7169100	1.951	1.034	0.925	0.898

UQ	3	7271401	7271700	0.720	0.303	0.296	0.198
UQ	3	7459201	7459500	2.988	1.704	0.909	1.740
UQ	3	7473301	7473600	0.640	1.487	1.422	1.759
UQ	3	7539301	7539600	0.640	0.399	0.239	0.335
UQ	3	7618501	7618800	0.400	0.584	0.292	0.304
UQ	3	7853701	7854000	2.181	1.124	0.950	0.964
UQ	3	7935601	7935900	0.349	0.657	0.716	0.826
UQ	3	8075101	8075400	0.105	0.656	0.588	0.421
UQ	3	8164801	8165100	6.578	3.948	3.532	3.252
UQ	3	8475601	8475900	0.213	0.491	0.078	0.329
UQ	3	8606401	8606700	2.870	1.987	1.152	1.648
UQ	3	8682901	8683200	0.053	0.376	0.341	0.497
UQ	3	8717401	8717700	0.771	0.513	0.243	0.497
UQ	3	8801401	8801700	0.666	0.328	0.453	0.497
UQ	3	9219901	9220200	2.773	4.072	3.817	4.071
UQ	3	9581101	9581400	0.744	0.375	0.317	0.289
UQ	3	9613201	9613500	3.846	2.448	2.325	2.265
UQ	3	9654001	9654300	0.584	0.841	0.370	0.106
UQ	3	9659101	9659400	3.352	1.710	2.332	4.116
UQ	3	9756901	9757200	0.800	0.584	0.239	0.309
UQ	3	9777901	9778200	0.240	0.444	0.424	0.472
UQ	3	9990301	9990600	1.412	0.959	1.081	0.994
UQ	3	10040701	10041000	2.449	1.822	1.670	1.694
UQ	3	10041001	10041300	2.923	2.204	1.650	1.764
UQ	3	10260001	10260300	0.265	0.000	0.510	0.031
UQ	3	10357201	10357500	2.236	1.566	1.333	1.572
UQ	3	10437001	10437300	0.560	0.212	0.424	0.304
UQ	3	11345701	11346000	1.087	0.633	0.642	0.695
UQ	3	11443201	11443500	3.117	2.426	2.307	2.454
UQ	3	11933401	11933700	1.781	1.259	1.295	1.440
UQ	3	12419401	12419700	1.542	2.438	2.542	3.524
UQ	3	13029301	13029600	1.650	0.910	0.695	1.100
UQ	3	13398301	13398600	7.772	5.769	5.680	6.282
UQ	3	13416601	13416900	0.687	0.255	0.185	0.167
UQ	3	13753801	13754100	3.081	2.201	3.665	3.630
UQ	3	13926301	13926600	0.824	0.538	0.473	0.396
UQ	3	14111701	14112000	1.753	1.002	1.115	1.334
UQ	3	14270101	14270400	0.584	0.232	0.185	0.365
UQ	3	14535301	14535600	0.611	1.026	0.428	0.385
UQ	3	14788501	14788800	0.929	0.609	0.428	0.715
UQ	3	14944201	14944500	0.213	0.513	0.560	0.634
UQ	3	15073501	15073800	0.852	0.564	0.156	0.502
UQ	3	15590401	15590700	0.558	0.281	0.160	0.142
UQ	3	15750301	15750600	0.560	0.281	0.292	0.173
UQ	3	15891301	15891600	5.185	4.590	3.817	4.016
UQ	3	16047001	16047300	0.611	0.491	0.346	0.446
UQ	3	16686901	16687200	0.745	1.004	0.432	1.100
UQ	3	16713001	16713300	4.101	3.411	4.813	2.586
UQ	3	16749601	16749900	0.902	0.536	0.325	0.304
UQ	3	16838101	16838400	0.532	0.189	0.214	0.147
UQ	3	16887601	16887900	0.505	0.348	0.132	0.056
UQ	3	17348701	17349000	0.481	0.163	0.082	0.248
UQ	3	17744701	17745000	0.745	0.448	0.321	0.466
UQ	3	18206101	18206400	0.534	0.281	0.136	0.218
UQ	3	18322501	18322800	5.985	7.728	6.581	6.835
UQ	3	18487801	18488100	0.531	0.236	0.111	0.304
UQ	3	18497401	18497700	2.044	1.195	0.782	0.963
UQ	3	18497701	18498000	4.786	3.579	2.260	3.016
UQ	3	18512401	18512700	0.823	0.515	0.448	0.533
UQ	3	18546601	18546900	0.108	0.511	0.399	0.411
UQ	3	18550201	18550500	1.039	2.081	2.274	2.464
UQ	3	18731101	18731400	1.147	0.659	0.457	0.695
UQ	3	18793501	18793800	2.382	3.512	3.447	3.579
UQ	3	19210501	19210800	2.555	2.006	1.690	1.627
UQ	3	19299301	19299600	2.065	1.002	0.814	1.131
UQ	3	19332601	19332900	1.058	2.158	2.180	2.074
UQ	3	19506601	19506900	0.932	0.348	0.506	0.441

UQ	3	19513501	19513800	0.639	0.466	0.395	0.112
UQ	3	19568401	19568700	1.463	0.719	0.959	1.075
UQ	3	19785601	19785900	0.160	0.607	0.346	0.309
UQ	3	19930201	19930500	1.520	2.240	2.479	3.413
UQ	3	19979701	19980000	1.329	2.173	1.674	1.435
UQ	3	20093701	20094000	0.744	0.983	0.580	1.024
UQ	3	21256501	21256800	0.637	0.303	0.243	0.228
UQ	3	21307501	21307800	0.771	0.373	0.399	0.299
UQ	3	21469501	21469800	0.347	0.116	0.560	0.132
UQ	3	22056001	22056300	0.161	0.421	0.370	0.522
UQ	3	22244701	22245000	0.611	0.277	0.317	0.826
UQ	3	22298101	22298400	0.557	0.116	0.288	0.025
UQ	3	23032801	23033100	0.239	0.817	0.711	0.472
UQ	3	23143801	23144100	0.610	0.187	0.181	0.137
UQ	3	23144101	23144400	0.557	0.260	0.214	0.142
UQ	3	23394301	23394600	1.358	0.633	0.205	0.132
UQ	3	23427601	23427900	0.776	0.255	0.082	0.167
UQ	3	23439601	23439900	1.082	2.568	2.572	2.292
UQ	3	23498701	23499000	0.852	0.513	0.296	0.507
UQ	3	24004501	24004800	0.507	0.302	0.267	0.274
UQ	3	24055201	24055500	1.142	0.612	0.745	0.608
UQ	3	24233101	24233400	0.855	1.562	1.551	2.612
UQ	3	24995401	24995700	1.439	0.935	0.901	1.029
UQ	3	25399801	25400100	0.613	0.328	0.078	0.360
UQ	3	25816201	25816500	0.718	0.232	0.247	0.213
UQ	3	26175901	26176200	1.808	1.613	1.966	1.166
UQ	3	26502001	26502300	0.505	0.654	0.239	0.695
UQ	3	27447601	27447900	0.560	0.701	0.374	0.416
UQ	3	27904801	27905100	0.477	0.213	0.054	0.031
UQ	3	28207501	28207800	0.716	0.212	0.127	0.152
UQ	3	28251001	28251300	1.089	0.378	0.564	0.573
UQ	3	28330801	28331100	0.213	1.467	1.094	1.313
UQ	3	28442401	28442700	0.263	0.607	0.667	0.553
UQ	3	28603201	28603500	0.560	0.212	0.074	0.137
UQ	3	28777201	28777500	0.795	0.352	0.292	0.304
UQ	3	29296201	29296500	1.387	0.817	0.827	0.817
UQ	3	29333401	29333700	0.720	0.279	0.288	0.335
UQ	3	30184501	30184800	0.558	0.283	0.424	0.167
UQ	3	30273601	30273900	0.611	0.281	0.317	0.329
UQ	3	30602101	30602400	2.284	1.772	1.839	1.633
UQ	3	31051201	31051500	0.292	0.560	0.078	0.547
UQ	3	31180501	31180800	1.700	2.662	2.501	2.413
UQ	3	31235101	31235400	3.218	0.912	0.653	0.802
UQ	3	31235401	31235700	3.178	1.143	0.809	1.125
UQ	3	31237501	31237800	1.708	0.217	0.279	0.112
UQ	3	31305901	31306200	1.089	0.772	0.502	0.284
UQ	3	32028301	32028600	0.132	0.420	0.346	0.391
UQ	3	32057401	32057700	0.160	0.536	0.370	0.608
UQ	3	32163901	32164200	0.637	0.354	0.058	0.365
UQ	3	32466301	32466600	2.925	1.985	1.460	2.551
UQ	3	32500501	32500800	0.773	0.305	0.312	0.319
UQ	3	32580601	32580900	1.807	1.219	0.564	0.999
UQ	3	32600401	32600700	0.586	0.326	0.296	0.243
UQ	3	33278701	33279000	0.874	0.444	0.453	0.522
UQ	3	33672601	33672900	1.518	1.214	1.119	1.247
UQ	3	33681901	33682200	0.740	0.159	0.127	0.132
UQ	3	34284601	34284900	0.666	0.328	0.354	0.223
UQ	3	34348501	34348800	0.452	0.657	0.160	0.274
UQ	3	34366201	34366500	0.800	1.431	0.395	0.254
UQ	3	34744801	34745100	1.221	0.729	0.633	0.735
UQ	3	34808701	34809000	0.769	0.983	0.506	0.974
UQ	3	35207701	35208000	0.505	0.185	0.399	0.137
UQ	3	35320501	35320800	0.799	0.399	0.395	0.603
UQ	3	35355901	35356200	2.999	1.296	0.921	1.313
UQ	3	35515801	35516100	2.023	0.465	0.881	0.983
UQ	3	35537101	35537400	2.355	0.891	1.001	1.217
UQ	3	35742301	35742600	0.507	0.303	0.111	0.112

UQ	3	36003301	36003600	0.637	0.397	0.399	0.497
UQ	3	37370401	37370700	0.505	0.350	0.296	0.279
UQ	3	37392301	37392600	0.268	0.491	0.502	0.639
UQ	3	37605301	37605600	0.318	0.702	0.938	0.700
UQ	3	37755601	37755900	1.387	0.429	0.495	0.522
UQ	3	37772101	37772400	1.300	1.637	0.769	0.877
UQ	3	38472901	38473200	0.744	0.302	0.214	0.324
UQ	3	38964001	38964300	1.573	0.701	0.468	0.903
UQ	3	39289501	39289800	0.957	1.399	1.489	1.298
UQ	3	39429301	39429600	2.231	2.903	1.608	2.966
UQ	3	39786901	39787200	0.294	0.466	0.453	0.497
UQ	3	40144501	40144800	0.744	0.307	0.301	0.279
UQ	3	40194301	40194600	0.373	0.841	0.671	1.024
UQ	3	40212901	40213200	0.455	1.191	1.028	1.044
UQ	3	40313401	40313700	0.453	0.210	0.560	0.248
UQ	3	40400701	40401000	0.106	0.399	0.502	0.446
UQ	3	40459201	40459500	0.897	2.216	2.033	1.932
UQ	3	40875901	40876200	0.665	1.026	1.008	0.918
UQ	3	41135701	41136000	0.615	0.023	0.103	0.218
UQ	3	41167501	41167800	0.716	0.399	0.210	0.304
UQ	3	41190601	41190900	0.771	0.185	0.288	0.106
UQ	3	41307001	41307300	0.824	0.403	0.424	0.324
UQ	3	42122101	42122400	0.026	0.185	0.477	0.279
UQ	3	42213901	42214200	0.555	0.139	0.107	0.101
UQ	3	42218401	42218700	1.194	0.384	0.317	0.142
UQ	3	42433501	42433800	1.089	0.491	0.555	0.497
UQ	3	43308001	43308300	0.242	0.796	0.580	0.527
UQ	3	45251401	45251700	0.690	0.300	0.234	0.112
UQ	3	45938401	45938700	0.531	0.818	0.156	0.963
UQ	3	46329001	46329300	4.917	5.396	6.541	6.860
UQ	3	46707601	46707900	2.629	3.647	3.788	3.756
UQ	3	47371201	47371500	0.055	0.446	0.346	0.329
UQ	3	47505301	47505600	1.412	0.637	0.629	0.731
UQ	3	47574301	47574600	0.690	0.324	0.321	0.315
UQ	3	47847301	47847600	0.345	0.652	0.617	0.832
UQ	3	49313701	49314000	0.826	0.350	0.613	0.527
UQ	3	51117001	51117300	0.266	0.678	0.852	0.918
UQ	3	51468001	51468300	0.186	0.328	0.346	0.578
UQ	3	51927001	51927300	0.534	0.257	0.210	0.167
UQ	3	52617901	52618200	1.274	0.869	0.736	0.776
UQ	3	54311401	54311700	0.955	1.191	0.716	0.725
UQ	3	55674001	55674300	0.668	0.350	0.272	0.329
UQ	3	56857501	56857800	0.665	0.401	0.267	0.223
UQ	3	57008401	57008700	1.060	0.286	0.196	0.137
UQ	3	57008701	57009000	0.823	0.140	0.049	0.056
UQ	3	57281101	57281400	0.957	0.747	0.403	0.603
UQ	3	57446101	57446400	1.273	1.894	1.839	1.957
UQ	3	57734701	57735000	0.615	0.305	0.263	0.218
UQ	3	57793201	57793500	1.545	0.916	0.613	0.893
UQ	3	57825601	57825900	0.160	0.491	0.399	0.385
UQ	3	58725601	58725900	0.160	0.444	0.321	0.274
UQ	3	59590801	59591100	0.852	0.470	0.502	0.583
UQ	3	59636401	59636700	0.797	0.465	0.584	0.578
UQ	3	60816301	60816600	0.987	0.605	0.580	0.385
UQ	3	60857101	60857400	3.004	5.044	4.955	5.345
UQ	3	60979801	60980100	0.505	0.281	0.317	0.193
UQ	3	61448101	61448400	0.534	0.375	0.185	0.137
UQ	3	61449901	61450200	1.115	0.727	0.629	0.603
UQ	3	61645201	61645500	0.269	1.099	0.745	0.659
UQ	3	61739101	61739400	0.503	0.139	0.152	0.086
UQ	3	61751101	61751400	0.319	0.536	0.584	0.583
UQ	3	62027701	62028000	1.542	1.028	2.049	1.896
UQ	3	62044201	62044500	2.531	1.500	4.246	4.174
UQ	3	62279701	62280000	1.571	0.747	0.979	1.222
UQ	3	62294101	62294400	0.795	0.539	0.317	0.497
UQ	3	62374801	62375100	0.132	0.517	0.560	0.527
UQ	3	63254101	63254400	0.534	0.234	0.160	0.213

UQ	3	63265801	63266100	2.502	1.892	1.522	1.816
UQ	3	63271201	63271500	0.879	2.343	1.494	1.425
UQ	3	63582001	63582300	0.505	0.303	0.107	0.279
UQ	3	63775501	63775800	3.141	0.764	1.133	0.817
UQ	3	63996601	63996900	0.292	0.704	0.724	0.877
UQ	3	64842901	64843200	0.666	0.328	0.395	0.329
UQ	3	65422801	65423100	0.716	0.444	0.473	1.303
UQ	3	66150601	66150900	0.503	0.912	0.983	1.029
UQ	3	66256801	66257100	0.584	0.350	0.267	0.411
UQ	3	67749901	67750200	0.586	0.257	0.132	0.497
UQ	3	67962301	67962600	1.036	0.491	0.535	0.461
UQ	3	68449201	68449500	0.105	0.444	0.346	0.466
UQ	3	68542501	68542800	0.421	1.236	1.102	1.267
UQ	3	69253501	69253800	4.301	1.783	1.757	2.871
UQ	3	69331201	69331500	0.053	0.258	0.185	0.466
UQ	3	69667201	69667500	1.728	1.071	0.881	1.323
UQ	3	70304101	70304400	1.489	1.075	0.881	0.877
UQ	3	70460401	70460700	1.013	0.564	0.564	0.162
UQ	3	70464901	70465200	2.363	1.519	1.908	3.251
UQ	3	71428801	71429100	0.321	0.611	2.151	1.364
UQ	3	71688901	71689200	0.610	0.303	1.037	0.248
UQ	3	74426701	74427000	0.213	0.702	0.506	0.533
UQ	3	74594701	74595000	0.531	0.116	0.218	0.187
UQ	3	75605701	75606000	0.905	0.564	0.580	0.573
UQ	3	75750301	75750600	0.558	0.234	0.189	0.137
UQ	3	76614601	76614900	4.063	3.053	2.472	2.798
UQ	3	76925401	76925700	1.920	0.373	0.444	1.186
UQ	3	76925701	76926000	1.655	0.258	0.312	1.050
UQ	3	77226901	77227200	1.626	0.929	1.012	0.786
UQ	3	77317201	77317500	1.590	0.663	0.716	0.619
UQ	3	77353201	77353500	0.640	0.139	0.263	0.000
UQ	3	77702101	77702400	3.888	2.133	3.250	2.702
UQ	3	78395101	78395400	0.745	0.375	0.189	0.086
UQ	3	79215601	79215900	0.450	0.139	0.156	0.223
UQ	3	79896601	79896900	3.124	1.155	0.691	0.614
UQ	3	80496001	80496300	2.174	0.987	0.821	0.902
UQ	3	80535001	80535300	4.307	3.765	4.710	3.823
UQ	3	81256501	81256800	0.477	0.678	0.267	0.360
UQ	3	81520501	81520800	0.689	0.352	0.395	0.355
UQ	3	81737401	81737700	0.584	0.161	0.189	0.051
UQ	3	81971701	81972000	0.745	0.257	0.379	0.268
UQ	3	82216501	82216800	0.716	0.068	0.025	0.025
UQ	3	82273801	82274100	0.081	0.401	0.321	0.385
UQ	3	82728301	82728600	0.769	0.236	0.346	0.411
UQ	3	83560801	83561100	0.503	0.212	0.156	0.187
UQ	3	83625901	83626200	0.797	0.465	0.296	0.218
UQ	3	83640001	83640300	1.297	0.322	0.481	0.329
UQ	3	83873101	83873400	0.505	0.187	0.127	0.031
UQ	3	85281301	85281600	4.015	6.663	5.714	7.733
UQ	3	86075701	86076000	0.800	0.465	0.448	0.329
UQ	3	87227101	87227400	0.507	0.303	0.181	0.167
UQ	3	87247201	87247500	0.186	0.584	0.453	0.304
UQ	3	87343201	87343500	0.776	0.212	0.181	0.193
UQ	3	87516001	87516300	1.836	1.195	0.843	1.095
UQ	3	87777601	87777900	0.053	0.753	1.037	0.755
UQ	3	88889701	88890000	0.507	0.187	0.078	0.223
UQ	3	88927501	88927800	0.477	0.024	0.165	0.132
UQ	3	89823301	89823600	1.595	0.891	0.966	1.019
UQ	3	89842501	89842800	1.276	0.890	0.794	0.567
UQ	3	90102301	90102600	0.294	0.444	0.448	0.142
UQ	3	90573901	90574200	0.560	0.185	0.185	0.076
UQ	3	91035301	91035600	0.560	0.373	0.317	0.248
UQ	3	91045801	91046100	0.160	0.586	0.317	0.304
UQ	3	91158301	91158600	0.426	0.676	0.239	0.248
UQ	3	91288201	91288500	0.878	0.421	0.531	0.725
UQ	3	91704901	91705200	0.398	0.515	0.292	0.720
UQ	3	91750501	91750800	0.158	0.444	0.395	0.411

UQ	3	92180701	92181000	1.645	2.781	2.677	2.211
UQ	3	93087301	93087600	0.481	0.185	0.000	0.279
UQ	3	93160501	93160800	0.929	0.702	0.399	0.776
UQ	3	93304801	93305100	0.452	1.888	1.614	3.854
UQ	3	93380101	93380400	1.458	1.053	0.987	0.918
UQ	3	93385201	93385500	1.302	0.657	0.901	0.812
UQ	3	93798601	93798900	1.889	1.217	1.264	1.141
UQ	3	94850101	94850400	1.834	2.707	2.403	2.459
UQ	3	95004601	95004900	0.481	0.232	0.234	0.193
UQ	3	95077201	95077500	0.797	0.491	0.267	0.913
UQ	3	95116501	95116800	0.455	0.165	0.107	0.137
UQ	3	95206501	95206800	1.036	0.369	0.502	0.456
UQ	3	95292301	95292600	0.239	0.494	0.716	0.578
UQ	3	95694601	95694900	1.676	1.058	1.086	0.882
UQ	3	96232201	96232500	0.639	0.397	0.317	0.299
UQ	3	96234601	96234900	0.613	0.232	0.230	0.137
UQ	3	96323701	96324000	0.026	0.283	0.419	0.385
UQ	3	97115101	97115400	5.532	3.770	3.743	3.438
UQ	3	97356901	97357200	0.531	0.375	0.321	0.335
UQ	3	98099101	98099400	0.507	0.352	0.132	0.142
UQ	3	98627701	98628000	0.108	0.680	0.555	0.796
UQ	3	98667601	98667900	0.586	0.702	0.428	0.472
UQ	3	98908201	98908500	0.639	0.395	0.346	0.279
UQ	3	98958601	98958900	1.276	0.753	0.267	0.593
UQ	3	99000001	99000300	0.824	0.489	0.292	0.558
UQ	3	99515701	99516000	0.850	0.255	0.390	0.198
UQ	3	99755701	99756000	0.744	0.375	0.292	0.380
UQ	3	99959401	99959700	0.450	0.234	0.214	0.248
UQ	3	99975301	99975600	0.560	0.302	0.292	0.304
UQ	3	100346401	100346700	1.563	0.865	0.992	0.994
UQ	3	100362301	100362600	0.879	0.511	0.560	0.527
UQ	3	101197801	101198100	0.452	0.725	0.160	0.309
UQ	3	101336701	101337000	0.450	0.187	0.111	0.031
UQ	3	101337001	101337300	1.463	0.566	0.510	0.137
UQ	3	101343601	101343900	0.081	0.706	0.395	0.411
UQ	3	101486701	101487000	1.063	0.699	0.613	0.801
UQ	3	101574901	101575200	0.558	0.236	0.156	0.355
UQ	3	101608501	101608800	4.852	2.180	1.353	3.706
UQ	3	101608801	101609100	2.149	0.775	0.678	1.451
UQ	3	101627101	101627400	2.605	0.873	0.629	1.363
UQ	3	101627401	101627700	1.202	0.676	0.526	0.578
UQ	3	101641501	101641800	6.320	4.705	5.150	4.751
UQ	3	101641801	101642100	0.642	0.189	0.218	0.162
UQ	3	101654101	101654400	3.149	1.152	1.074	1.439
UQ	3	101654401	101654700	4.233	2.324	1.953	2.241
UQ	3	101655001	101655300	4.528	1.729	1.645	1.996
UQ	3	101655301	101655600	4.193	1.172	1.262	1.103
UQ	3	101730301	101730600	0.900	0.440	0.239	0.218
UQ	3	101735401	101735700	0.665	0.330	0.370	0.391
UQ	3	101994001	101994300	0.294	0.749	0.555	0.695
UQ	3	102640501	102640800	2.876	2.012	2.115	1.922
UQ	3	102945601	102945900	1.065	0.590	0.571	0.056
UQ	3	103390501	103390800	1.729	1.916	1.300	1.552
UQ	3	103472401	103472700	2.152	2.813	1.415	1.613
UQ	3	103853101	103853400	1.970	0.907	1.050	0.822
UQ	3	103934101	103934400	3.910	1.772	2.310	2.575
UQ	3	104610601	104610900	2.605	1.963	1.723	2.347
UQ	3	104632801	104633100	2.634	1.732	1.752	1.871
UQ	3	104761801	104762100	2.609	0.774	0.639	0.223
UQ	3	104793001	104793300	0.507	0.189	0.239	0.254
UQ	3	104911201	104911500	1.010	0.721	0.584	0.578
UQ	3	105252901	105253200	5.957	4.630	4.739	5.633
UQ	3	105667801	105668100	0.560	0.260	0.210	0.243
UQ	3	105990001	105990300	1.065	0.678	0.724	0.690
UQ	3	107195701	107196000	0.481	0.163	0.127	0.167
UQ	3	107421901	107422200	1.118	0.628	0.711	0.558
UQ	3	107856001	107856300	0.081	0.448	0.399	0.466

UQ	3	107996101	107996400	1.300	0.438	0.415	0.426
UQ	3	108088501	108088800	4.544	5.877	7.262	6.171
UQ	3	108189901	108190200	0.720	0.281	0.531	0.608
UQ	3	108262801	108263100	1.249	3.181	3.197	3.148
UQ	3	108267001	108267300	0.561	0.023	0.132	0.031
UQ	3	108311401	108311700	0.290	1.101	0.716	0.583
UQ	3	108363601	108363900	0.502	0.140	0.049	0.051
UQ	3	108489901	108490200	2.397	1.480	1.210	1.465
UQ	3	109086301	109086600	0.345	0.538	0.210	0.218
UQ	3	109086601	109086900	0.507	0.701	0.127	0.193
UQ	3	109148101	109148400	0.319	0.444	0.082	0.218
UQ	3	109862401	109862700	1.599	1.918	1.275	2.292
UQ	3	109881901	109882200	0.718	0.678	0.506	0.304
UQ	3	110242501	110242800	0.799	0.185	0.243	0.218
UQ	3	110317201	110317500	0.531	0.092	0.140	0.081
UQ	3	110854501	110854800	0.771	0.330	0.428	0.360
UQ	3	111352801	111353100	0.426	0.515	0.267	0.360
UQ	3	111655501	111655800	2.015	1.354	0.983	1.496
UQ	3	112281601	112281900	0.477	0.629	0.321	0.279
UQ	3	114166201	114166500	0.532	0.867	0.852	0.776
UQ	3	114417301	114417600	0.637	0.234	0.243	0.309
UQ	3	114424501	114424800	1.724	0.792	0.876	1.166
UQ	3	114428401	114428700	1.594	1.403	1.197	0.994
UQ	3	114432601	114432900	0.692	0.493	0.531	0.832
UQ	3	115257301	115257600	0.584	0.069	0.152	0.218
UQ	3	115257601	115257900	0.642	0.140	0.123	0.137
UQ	3	115288201	115288500	2.806	4.401	1.670	4.198
UQ	3	116205301	116205600	0.507	0.326	0.263	0.223
UQ	3	116385601	116385900	1.307	1.869	0.160	0.259
UQ	3	116395801	116396100	0.957	0.515	0.399	0.238
UQ	3	116519101	116519400	2.521	1.609	1.222	1.440
UQ	3	116899801	116900100	0.639	0.307	0.107	0.031
UQ	3	117235201	117235500	1.065	0.701	0.160	0.198
UQ	3	117235501	117235800	1.061	0.508	0.029	0.112
UQ	3	118579501	118579800	2.238	1.847	1.654	1.577
UQ	3	118767001	118767300	0.428	1.285	0.843	0.842
UQ	3	118796101	118796400	0.905	0.468	0.448	0.431
UQ	3	118885801	118886100	0.182	1.238	2.455	1.287
UQ	3	119215801	119216100	0.853	0.564	0.481	0.527
UQ	3	119258701	119259000	0.239	0.094	0.025	0.547
UQ	3	119405701	119406000	0.479	0.257	0.638	0.329
UQ	3	119635801	119636100	2.257	1.141	1.152	1.262
UQ	3	120142201	120142500	2.079	1.358	1.259	1.465
UQ	3	122398801	122399100	0.347	0.562	0.638	0.527
UQ	3	122719801	122720100	0.716	0.421	0.424	0.396
UQ	3	123337501	123337800	0.819	0.116	0.087	0.117
UQ	3	124053601	124053900	0.505	0.118	0.098	0.187
UQ	3	124746901	124747200	1.565	0.521	0.622	0.690
UQ	3	124747201	124747500	1.142	0.708	0.477	0.690
UQ	3	125514301	125514600	0.558	0.234	0.296	0.106
UQ	3	126422701	126423000	1.625	0.908	1.115	1.206
UQ	3	126489301	126489600	1.417	0.118	0.160	0.106
UQ	3	126489601	126489900	0.690	0.142	0.152	0.086
UQ	3	127524001	127524300	0.584	0.232	0.881	0.187
UQ	3	128262601	128262900	6.172	4.772	5.047	4.979
UQ	3	128531401	128531700	1.918	1.199	0.856	0.999
UQ	3	129000301	129000600	0.532	0.326	0.346	0.335
UQ	3	129243601	129243900	0.211	0.466	0.531	0.385
UQ	3	129591601	129591900	0.661	0.350	0.263	0.274
UQ	3	129924001	129924300	1.031	0.024	0.054	0.152
UQ	3	129951901	129952200	0.639	0.232	0.424	0.329
UQ	3	130078801	130079100	1.382	1.143	0.774	0.416
UQ	3	130146301	130146600	0.955	0.609	0.745	0.411
UQ	3	130291501	130291800	0.878	0.513	0.370	0.588
UQ	3	130324801	130325100	0.374	0.562	0.185	0.193
UQ	3	130818601	130818900	0.560	0.350	0.399	0.274
UQ	3	131303101	131303400	0.879	1.382	0.584	0.137

UQ	3	131875201	131875500	1.807	0.953	0.934	1.131
UQ	3	132017401	132017700	3.321	1.560	2.521	2.499
UQ	3	132049201	132049500	1.170	1.849	1.634	2.160
UQ	3	132087901	132088200	0.481	0.213	0.214	0.167
UQ	3	132648301	132648600	1.991	0.202	1.255	0.785
UQ	3	133531501	133531800	0.527	1.238	1.012	1.116
UQ	3	133837201	133837500	0.957	0.371	0.638	0.664
UQ	3	133837501	133837800	0.507	0.092	0.000	0.228
UQ	3	134074801	134075100	0.797	0.631	0.716	0.497
UQ	3	134109301	134109600	0.665	0.446	0.210	0.254
UQ	3	134139601	134139900	0.773	0.371	0.370	0.360
UQ	3	134304001	134304300	0.532	0.184	0.156	0.248
UQ	3	134489101	134489400	0.795	0.330	0.448	1.100
UQ	3	134581801	134582100	0.294	0.444	0.156	0.137
UQ	3	135024601	135024900	0.715	1.401	1.193	1.389
UQ	3	135071701	135072000	0.426	0.562	0.214	0.223
UQ	3	136039501	136039800	0.636	0.961	0.399	0.335
UQ	3	136214101	136214400	1.037	1.358	1.411	0.675
UQ	3	136248901	136249200	0.929	0.466	0.341	0.533
UQ	3	136530001	136530300	1.892	1.476	1.480	0.938
UQ	3	136912801	136913100	1.224	0.631	0.789	0.857
UQ	3	137083501	137083800	0.769	0.515	0.292	0.441
UQ	3	137161201	137161500	1.278	1.635	0.613	0.963
UQ	3	138760501	138760800	1.271	0.352	0.357	0.000
UQ	3	138889201	138889500	0.505	0.187	0.296	0.162
UQ	3	139152301	139152600	0.903	0.045	0.029	0.086
UQ	3	139325101	139325400	2.020	1.684	1.329	1.389
UQ	3	139607401	139607700	0.373	0.212	0.716	0.167
UQ	3	140338801	140339100	0.160	0.609	0.078	0.279
UQ	3	142080601	142080900	0.160	0.466	0.346	0.056
UQ	3	142558501	142558800	1.011	0.631	0.555	0.304
UQ	3	142563901	142564200	0.587	0.255	0.127	0.187
UQ	3	143029201	143029500	0.613	0.281	0.288	0.051
UQ	3	143163901	143164200	0.639	0.399	0.366	0.391
UQ	3	143164201	143164500	0.690	0.375	0.390	0.391
UQ	3	144001501	144001800	0.055	0.350	0.531	0.193
UQ	3	144227701	144228000	0.692	0.324	0.341	0.243
UQ	3	144806401	144806700	0.666	0.448	0.107	0.360
UQ	3	144849601	144849900	1.091	0.536	0.876	0.669
UQ	3	146203501	146203800	0.532	0.257	0.074	0.193
UQ	3	147393301	147393600	0.398	0.303	0.477	0.248
UQ	3	147438601	147438900	0.611	0.513	0.477	0.745
UQ	3	148226401	148226700	1.223	0.749	0.662	0.669
UQ	3	148525801	148526100	0.982	0.140	0.399	0.370
UQ	3	149265901	149266200	0.106	0.466	0.399	0.360
UQ	3	150659401	150659700	0.823	0.678	0.239	0.391
UQ	3	151061701	151062000	0.689	0.375	0.366	0.360
UQ	3	151480801	151481100	2.255	0.940	1.119	1.055
UQ	3	151904401	151904700	2.204	1.287	1.181	1.451
UQ	3	151992001	151992300	0.081	1.247	0.711	0.902
UQ	3	151992301	151992600	0.081	1.642	0.867	1.135
UQ	3	154077001	154077300	0.586	0.187	0.288	0.228
UQ	3	154466401	154466700	0.026	0.187	0.214	0.527
UQ	3	154505701	154506000	0.665	0.210	0.078	0.243
UQ	3	154794601	154794900	0.773	0.373	0.189	0.051
UQ	3	154930801	154931100	1.439	0.524	0.575	0.187
UQ	3	154934101	154934400	2.502	1.916	1.344	1.897
UQ	3	155147401	155147700	2.447	1.661	1.721	1.734
UQ	3	155512801	155513100	0.426	0.770	0.716	0.756
UQ	3	155636101	155636400	0.053	0.489	0.370	0.355
UQ	3	156186901	156187200	3.296	1.907	1.284	1.758
UQ	3	156238501	156238800	0.240	0.725	0.560	0.725
UQ	3	156711901	156712200	0.132	0.517	0.481	0.360
UQ	3	157508101	157508400	0.505	0.234	0.296	0.142
UQ	3	157575301	157575600	0.481	0.118	0.160	0.056
UQ	3	158321101	158321400	2.499	2.075	1.694	2.043
UQ	3	158732401	158732700	1.089	0.538	0.856	0.274

UQ	3	159429901	159430200	0.505	0.000	0.098	0.000
UQ	3	160584901	160585200	1.039	0.375	0.497	0.558
UQ	3	161055301	161055600	0.290	0.747	0.963	0.639
UQ	3	161492101	161492400	0.745	0.448	0.403	0.198
UQ	3	161589301	161589600	0.132	0.468	0.292	0.304
UQ	3	161867401	161867700	0.958	0.629	0.374	0.396
UQ	3	162189301	162189600	2.289	1.188	1.387	1.759
UQ	3	162684001	162684300	0.373	0.212	0.609	0.112
UQ	3	162868801	162869100	0.852	1.189	1.135	0.527
UQ	3	163791301	163791600	0.132	0.444	0.370	0.416
UQ	3	164634001	164634300	1.013	0.583	0.568	0.522
UQ	3	165326101	165326400	0.932	1.332	1.543	1.181
UQ	3	165374401	165374700	3.028	2.229	4.189	1.820
UQ	3	165553201	165553500	1.173	1.403	0.798	1.546
UQ	3	165750601	165750900	0.160	0.468	0.370	0.304
UQ	3	165830401	165830700	0.558	0.399	0.317	0.441
UQ	3	166127401	166127700	0.426	0.539	0.267	0.248
UQ	3	166909501	166909800	0.292	0.890	0.160	0.497
UQ	3	168126901	168127200	0.611	0.395	0.185	0.391
UQ	3	169023601	169023900	0.584	0.185	0.218	0.218
UQ	3	170550301	170550600	3.460	2.716	2.764	2.890
UQ	3	172639501	172639800	0.850	0.210	0.156	0.288
UQ	3	173105401	173105700	0.823	0.401	0.506	0.329
UQ	3	173143501	173143800	2.097	1.208	1.059	1.131
UQ	3	173335501	173335800	1.968	0.302	1.117	1.100
UQ	3	174582601	174582900	2.102	1.566	1.418	1.298
UQ	3	174690601	174690900	1.091	0.536	0.515	0.441
UQ	3	175251901	175252200	2.284	2.854	2.780	3.463
UQ	3	175329601	175329900	0.610	0.303	0.272	0.254
UQ	3	176115001	176115300	0.263	0.891	0.633	0.751
UQ	3	176149201	176149500	0.690	0.326	0.230	0.112
UQ	3	176248201	176248500	1.355	0.794	0.526	0.614
UQ	3	176248501	176248800	1.439	0.751	0.571	0.664
UQ	3	176422201	176422500	2.880	1.715	1.102	0.862
UQ	3	176676901	176677200	0.373	0.584	0.531	0.304
UQ	3	177445801	177446100	0.557	0.350	0.029	0.223
UQ	3	178070701	178071000	0.957	0.421	0.428	0.492
UQ	3	178203601	178203900	0.610	0.352	0.214	0.198
UQ	3	178291801	178292100	1.163	0.191	0.399	0.401
UQ	3	178292101	178292400	1.382	0.352	0.457	0.446
UQ	3	178327201	178327500	0.531	0.865	0.136	0.259
UQ	3	178327501	178327800	0.347	0.493	0.054	0.086
UQ	3	178627801	178628100	2.772	2.197	1.839	2.205
UQ	3	178637401	178637700	3.297	2.461	2.468	1.856
UQ	3	179591401	179591700	4.680	4.205	2.829	3.773
UQ	3	179721301	179721600	2.241	1.000	1.259	1.485
UQ	3	179793601	179793900	0.639	0.376	0.374	0.385
UQ	3	180064501	180064800	0.481	0.727	0.979	0.999
UQ	3	180523501	180523800	2.636	1.899	1.583	1.475
UQ	3	180539101	180539400	2.100	1.495	1.415	2.753
UQ	3	180570301	180570600	3.268	2.381	2.390	2.321
UQ	3	180628501	180628800	6.629	5.263	5.301	5.744
UQ	3	180629101	180629400	1.568	2.171	1.201	1.222
UQ	3	181791301	181791600	0.105	0.534	0.584	0.391
UQ	3	181966201	181966500	1.254	1.826	1.966	1.841
UQ	3	182086201	182086500	0.132	0.395	0.399	0.695
UQ	3	183313801	183314100	0.053	0.279	0.448	0.193
UQ	3	184435801	184436100	0.742	0.375	0.189	0.167
UQ	3	184530301	184530600	0.668	0.253	0.210	0.218
UQ	3	185481001	185481300	1.145	0.794	0.682	0.751
UQ	3	186573601	186573900	1.010	0.772	1.217	0.720
UQ	3	187957501	187957800	0.266	0.538	0.054	0.522
UQ	3	188420101	188420400	0.558	0.114	0.292	0.223
UQ	3	188471401	188471700	0.955	0.723	0.691	0.274
UQ	3	188544001	188544300	0.905	0.401	0.337	0.497
UQ	3	189132001	189132300	3.770	0.607	1.657	0.106
UQ	3	189132301	189132600	1.858	0.315	0.678	0.162

UQ	3	189736201	189736500	1.010	0.751	0.633	0.608
UQ	3	189752701	189753000	0.106	0.487	0.453	0.391
UQ	3	189948001	189948300	0.532	0.234	0.346	0.142
UQ	3	190189201	190189500	0.558	0.279	0.210	0.329
UQ	3	191558701	191559000	0.477	0.092	0.234	0.218
UQ	3	191888701	191889000	0.692	0.189	0.453	0.385
UQ	3	193103701	193104000	0.507	0.257	0.160	0.299
UQ	3	193425001	193425300	0.532	1.287	1.016	0.826
UQ	3	193483201	193483500	1.042	0.468	0.539	0.497
UQ	3	193841701	193842000	0.718	0.307	0.370	0.243
UQ	3	193871401	193871700	0.106	0.444	0.263	0.329
UQ	3	195108601	195108900	1.355	0.841	0.896	0.826
UQ	3	195870001	195870300	0.690	0.465	0.930	0.441
UQ	3	196628101	196628400	1.087	0.611	0.575	0.588
UQ	3	197611501	197611800	1.147	0.380	0.526	0.512
UQ	3	198858901	198859200	0.800	0.515	0.267	0.304
UQ	3	200820301	200820600	0.292	0.745	0.930	0.628
UQ	3	201029701	201030000	0.584	0.324	0.210	0.218
UQ	3	201291901	201292200	0.503	0.140	0.189	0.081
UQ	3	201732001	201732300	0.318	0.562	0.560	0.162
UQ	3	202607101	202607400	0.716	0.279	0.176	0.162
UQ	3	202607401	202607700	1.092	0.491	0.275	0.254
UQ	3	202707601	202707900	0.160	0.352	0.448	0.411
UQ	3	202811701	202812000	0.668	0.212	0.160	0.193
UQ	3	203581801	203582100	0.584	0.187	0.214	0.112
UQ	3	203613301	203613600	0.586	0.446	0.374	0.304
UQ	3	203669701	203670000	0.503	0.234	0.321	0.193
UQ	3	204429601	204429900	0.507	0.184	0.087	0.056
UQ	3	205247701	205248000	1.410	2.032	1.757	1.085
UQ	3	205455901	205456200	0.426	0.609	0.054	0.167
UQ	3	205552201	205552500	0.666	0.421	0.374	0.411
UQ	3	207579001	207579300	0.507	0.165	0.234	0.187
UQ	3	207584101	207584400	0.928	0.444	0.609	0.304
UQ	3	207959401	207959700	2.870	1.377	1.921	1.880
UQ	3	207996301	207996600	0.745	1.330	1.349	1.075
UQ	3	208073701	208074000	0.319	0.468	0.214	0.416
UQ	3	208098601	208098900	0.611	0.330	0.341	0.918
UQ	3	208207801	208208100	1.011	0.468	0.633	0.720
UQ	3	208340401	208340700	0.184	0.727	0.555	0.547
UQ	3	208357801	208358100	0.505	0.281	0.259	0.167
UQ	3	208408201	208408500	0.984	0.534	0.510	0.608
UQ	3	208540201	208540500	1.010	0.513	0.506	0.527
UQ	3	208609201	208609500	2.287	2.570	1.670	1.795
UQ	3	208722601	208722900	0.769	0.515	0.263	0.213
UQ	3	209124601	209124900	1.655	0.605	0.938	0.857
UQ	3	209131201	209131500	0.979	0.449	0.317	0.254
UQ	3	209744701	209745000	4.518	3.673	2.542	3.052
UQ	3	209745001	209745300	3.591	2.161	1.944	2.201
UQ	3	209776801	209777100	1.249	0.773	0.760	0.720
UQ	3	209908801	209909100	0.826	0.163	0.292	0.299
UQ	3	210050701	210051000	0.929	0.629	0.560	0.340
UQ	3	210401101	210401400	7.975	6.151	5.819	6.668
UQ	3	210578401	210578700	1.228	2.219	1.708	1.602
UQ	3	210615901	210616200	3.409	1.897	2.147	2.662
UQ	3	210681601	210681900	3.804	3.267	2.818	2.459
UQ	3	210883201	210883500	0.132	0.684	0.617	0.553
UQ	3	210930301	210930600	0.931	0.702	0.506	0.684
UQ	3	211100101	211100400	3.320	2.264	2.695	2.591
UQ	3	211128001	211128300	4.438	3.066	3.756	4.011
UQ	3	211177501	211177800	1.226	0.706	0.736	0.720
UQ	3	211394101	211394400	0.560	0.185	0.288	0.193
UQ	3	212224801	212225100	0.773	1.307	1.070	1.410
UQ	3	212532601	212532900	1.887	1.313	1.353	0.943
UQ	3	212685601	212685900	0.740	0.279	0.296	0.304
UQ	3	212982901	212983200	0.744	0.168	0.136	0.365
UQ	3	213000901	213001200	0.716	0.393	0.185	0.254
UQ	3	213168001	213168300	0.640	0.350	0.317	0.411

UQ	3	213194701	213195000	3.155	2.131	1.946	2.018
UQ	3	213205801	213206100	0.507	0.257	0.181	0.304
UQ	3	213331801	213332100	0.319	0.468	0.399	0.385
UQ	3	213344701	213345000	0.374	0.656	0.156	0.142
UQ	3	213584401	213584700	1.729	1.167	1.255	1.136
UQ	3	213652801	213653100	0.694	0.000	0.049	0.061
UQ	3	213665401	213665700	1.436	0.185	0.111	0.304
UQ	3	213786901	213787200	1.862	1.564	1.444	1.019
UQ	3	213795901	213796200	3.139	2.920	2.682	3.671
UQ	3	213899401	213899700	2.154	1.732	1.699	1.673
UQ	3	213966301	213966600	0.881	0.420	0.234	0.497
UQ	3	214055401	214055700	0.400	0.654	0.588	0.162
UQ	3	214081201	214081500	0.771	0.397	0.502	0.603
UQ	3	214122901	214123200	1.116	0.654	0.881	0.832
UQ	3	214279801	214280100	0.560	1.049	1.144	1.131
UQ	3	214319701	214320000	1.652	1.148	0.831	0.988
UQ	3	214402501	214402800	1.626	1.143	0.629	1.004
UQ	3	214864201	214864500	1.757	1.191	1.411	1.019
UQ	3	215014201	215014500	0.586	0.234	0.399	0.279
UQ	3	215123701	215124000	0.744	0.423	0.399	0.492
UQ	3	215372701	215373000	1.860	1.219	0.814	1.440
UQ	3	215889301	215889600	1.199	0.680	0.740	0.715
UQ	3	215939401	215939700	1.094	2.437	1.616	1.881
UQ	3	216172201	216172500	6.167	6.657	5.582	5.228
UQ	3	216365401	216365700	3.704	1.715	1.686	1.925
UQ	3	216450001	216450300	0.531	0.165	0.103	0.218
UQ	3	216489901	216490200	4.846	3.119	3.574	3.199
UQ	3	216528601	216528900	0.984	0.699	0.613	0.588
UQ	3	216683701	216684000	0.453	0.772	0.876	0.918
UQ	3	217455601	217455900	2.738	4.014	3.982	3.853
UQ	3	217641001	217641300	3.165	3.572	2.871	2.439
UQ	3	217917301	217917600	0.610	0.305	0.156	0.167
UQ	3	218007001	218007300	0.347	0.564	0.638	0.142
UQ	3	218066101	218066400	1.091	0.472	0.745	0.720
UQ	3	218142001	218142300	1.087	0.635	0.613	0.553
UQ	3	218173801	218174100	1.142	0.510	0.288	0.482
UQ	3	218394001	218394300	2.817	1.906	1.629	1.516
UQ	3	218526601	218526900	3.836	2.677	1.371	1.647
UQ	3	218580301	218580600	5.161	4.682	4.702	3.686
UQ	3	218667001	218667300	0.455	0.163	0.000	0.223
UQ	3	218742001	218742300	0.186	0.588	0.539	0.583
UQ	3	218772901	218773200	3.113	2.141	1.937	3.797
UQ	3	218941201	218941500	1.597	0.933	0.934	1.105
UQ	3	218956201	218956500	3.244	2.034	3.698	3.509
UQ	3	218957401	218957700	2.154	1.916	1.596	2.565
UQ	3	218962801	218963100	1.226	0.539	0.729	0.751
UQ	3	218972101	218972400	2.236	2.736	2.838	3.078
UQ	3	219066601	219066900	2.842	1.712	1.708	1.952
UQ	3	219167701	219168000	0.610	0.118	0.214	0.248
UQ	3	219365101	219365400	0.161	0.538	0.506	0.335
UQ	3	219536401	219536700	0.186	0.418	0.774	0.664
UQ	3	219822601	219822900	2.665	1.963	1.362	1.967
UQ	3	219858901	219859200	0.715	0.300	0.292	0.299
UQ	3	219952501	219952800	4.790	2.935	2.592	2.161
UQ	3	219960001	219960300	2.897	1.944	1.699	1.744
UQ	3	219960301	219960600	0.955	0.725	0.424	0.411
UQ	3	219960601	219960900	4.665	2.732	1.801	1.480
UQ	3	219999601	219999900	3.062	2.034	2.192	2.220
UQ	3	220996501	220996800	8.564	7.235	7.148	6.865
UQ	3	221569201	221569500	2.289	0.886	1.518	1.815
UQ	3	221626201	221626500	2.971	2.043	4.053	2.115
UQ	3	221697001	221697300	1.491	0.846	0.517	0.669
UQ	3	221730301	221730600	3.172	1.877	1.427	1.266
UQ	3	221918401	221918700	1.357	0.980	2.093	1.070
UQ	3	222150901	222151200	4.059	2.341	1.982	2.373
UQ	3	222178201	222178500	1.011	0.865	0.609	1.166
UQ	3	222279001	222279300	3.854	2.281	2.838	2.637

UQ	3	222315001	222315300	0.774	0.371	0.317	0.137
UQ	3	222320701	222321000	0.768	0.238	0.370	0.142
UQ	3	222375601	222375900	0.292	0.586	0.136	0.553
UQ	3	222440101	222440400	1.466	0.935	0.774	0.983
UQ	3	222440401	222440700	2.289	1.751	1.275	1.647
UQ	3	222525601	222525900	0.907	0.397	0.448	0.639
UQ	3	222670501	222670800	0.345	1.056	0.852	1.136
UQ	3	222716401	222716700	4.281	2.392	1.944	3.170
UQ	3	222753901	222754200	1.831	0.525	0.526	0.710
UQ	3	222754801	222755100	1.142	0.564	0.531	0.740
UQ	3	222924001	222924300	4.441	4.860	5.067	4.812
UQ	3	223053601	223053900	0.558	0.279	0.111	0.335
UQ	3	223200901	223201200	3.647	2.587	2.488	1.319
UQ	3	223288501	223288800	1.855	0.590	0.896	0.461
UQ	3	223574101	223574400	9.671	8.121	8.362	7.118
UQ	3	223601401	223601700	2.233	1.682	1.538	1.962
UQ	3	224010601	224010900	2.480	1.448	1.835	1.653
UQ	4	536401	536700	0.931	0.536	0.617	0.411
UQ	4	1347001	1347300	0.347	0.652	0.058	0.852
UQ	4	3186301	3186600	0.610	0.114	0.160	0.279
UQ	4	3413701	3414000	0.637	0.277	0.399	0.335
UQ	4	3488701	3489000	0.106	0.442	0.539	1.055
UQ	4	3489001	3489300	0.103	0.564	0.531	0.974
UQ	4	3685201	3685500	0.505	0.210	0.341	0.304
UQ	4	4375201	4375500	0.160	0.656	0.239	0.304
UQ	4	4508401	4508700	6.100	3.105	2.825	2.666
UQ	4	5098801	5099100	1.091	2.060	1.897	2.156
UQ	4	5122801	5123100	0.026	1.009	0.876	0.573
UQ	4	5124601	5124900	0.424	1.075	1.246	0.695
UQ	4	5208601	5208900	1.670	0.755	0.716	0.847
UQ	4	5233501	5233800	0.718	0.442	0.473	1.415
UQ	4	5239201	5239500	4.910	3.330	2.135	3.078
UQ	4	5239501	5239800	2.267	1.129	0.526	0.628
UQ	4	5463301	5463600	0.505	0.187	0.292	0.248
UQ	4	5472601	5472900	2.095	0.727	1.123	1.176
UQ	4	6548701	6549000	1.279	0.888	0.852	0.614
UQ	4	7038001	7038300	0.477	0.772	0.263	0.187
UQ	4	7342201	7342500	0.931	1.453	0.691	0.664
UQ	4	7539901	7540200	2.799	2.064	1.313	3.651
UQ	4	7569001	7569300	0.000	0.612	0.403	0.684
UQ	4	7571701	7572000	0.800	0.185	0.325	0.421
UQ	4	7641601	7641900	0.610	0.350	0.181	0.274
UQ	4	7684501	7684800	1.173	1.777	1.703	1.577
UQ	4	8120101	8120400	0.505	0.654	0.239	0.416
UQ	4	8332801	8333100	1.511	0.562	0.809	0.785
UQ	4	8467801	8468100	6.639	3.358	3.170	4.255
UQ	4	8468101	8468400	3.582	1.393	0.620	1.750
UQ	4	8672401	8672700	0.823	0.493	0.234	0.299
UQ	4	8698201	8698500	0.848	0.279	0.243	0.101
UQ	4	8698501	8698800	2.920	1.042	0.613	0.476
UQ	4	9573601	9573900	0.586	0.073	0.288	0.061
UQ	4	9581101	9581400	0.453	0.985	0.156	0.740
UQ	4	9696901	9697200	0.771	1.051	1.148	1.237
UQ	4	9999301	9999600	2.205	3.012	3.351	2.992
UQ	4	10050601	10050900	0.692	0.257	0.448	0.324
UQ	4	10657501	10657800	0.213	0.418	0.428	0.025
UQ	4	11255401	11255700	1.408	0.614	0.437	0.745
UQ	4	12253801	12254100	1.446	0.510	0.276	0.411
UQ	4	12423901	12424200	1.245	0.631	0.560	0.740
UQ	4	13704001	13704300	0.452	0.258	0.292	0.603
UQ	4	15735901	15736200	5.962	4.581	3.425	3.873
UQ	4	15739501	15739800	0.186	0.468	0.346	0.385
UQ	4	16112701	16113000	0.902	0.562	0.477	0.588
UQ	4	16286101	16286400	1.144	0.935	1.066	0.553
UQ	4	16592101	16592400	0.000	0.420	0.243	0.223
UQ	4	20710201	20710500	1.544	0.839	0.963	0.771
UQ	4	20710501	20710800	2.284	1.176	1.237	1.359

UQ	4	21287401	21287700	0.132	0.442	0.419	0.411
UQ	4	22254001	22254300	0.240	0.631	0.078	0.051
UQ	4	22470301	22470600	1.886	1.352	1.378	1.064
UQ	4	22532401	22532700	0.665	0.421	0.292	0.380
UQ	4	23982301	23982600	0.692	0.307	0.218	0.365
UQ	4	24507001	24507300	2.341	3.043	4.003	3.803
UQ	4	24527401	24527700	0.319	0.515	0.613	0.547
UQ	4	24943201	24943500	1.010	0.773	0.477	0.360
UQ	4	25315801	25316100	1.412	0.964	0.925	1.926
UQ	4	25621201	25621500	0.450	0.096	0.185	0.187
UQ	4	26721001	26721300	1.913	1.427	1.201	1.613
UQ	4	26860801	26861100	0.773	0.420	0.555	0.497
UQ	4	27917401	27917700	0.982	0.747	0.638	0.497
UQ	4	28166101	28166400	0.450	0.185	0.111	0.031
UQ	4	28698601	28698900	0.637	0.446	0.214	0.223
UQ	4	29678401	29678700	0.584	0.283	0.111	0.162
UQ	4	30130501	30130800	2.742	1.171	1.148	2.144
UQ	4	30216301	30216600	0.027	0.446	0.292	0.329
UQ	4	30414001	30414300	0.373	0.843	0.716	0.776
UQ	4	30891301	30891600	1.091	0.656	0.531	0.421
UQ	4	31159501	31159800	1.434	0.888	0.745	1.009
UQ	4	31196401	31196700	1.250	0.517	0.464	0.274
UQ	4	31673101	31673400	0.694	0.348	0.317	0.284
UQ	4	31949401	31949700	0.613	0.232	0.341	0.162
UQ	4	31965301	31965600	0.665	0.444	0.366	0.218
UQ	4	32494501	32494800	0.582	0.161	0.025	0.031
UQ	4	33952801	33953100	0.429	0.023	0.078	0.051
UQ	4	34226401	34226700	0.503	0.281	0.136	0.137
UQ	4	34446901	34447200	0.477	0.047	0.165	0.157
UQ	4	36987901	36988200	0.134	0.348	0.317	0.497
UQ	4	37137601	37137900	0.665	0.421	0.350	0.304
UQ	4	37252201	37252500	0.665	0.352	0.448	0.497
UQ	4	40128901	40129200	0.560	0.796	0.774	0.274
UQ	4	40364101	40364400	0.852	0.517	0.350	0.603
UQ	4	42663301	42663600	0.663	0.236	0.243	0.157
UQ	4	43053301	43053600	0.374	0.562	0.078	0.167
UQ	4	43592401	43592700	0.560	0.350	0.160	0.355
UQ	4	43796101	43796400	0.398	0.629	0.584	0.553
UQ	4	43819201	43819500	0.615	0.350	0.239	0.304
UQ	4	44333401	44333700	1.254	0.865	0.823	0.938
UQ	4	44522701	44523000	0.292	0.116	0.695	0.031
UQ	4	45929701	45930000	0.742	0.399	0.263	0.324
UQ	4	46083001	46083300	4.112	3.107	3.023	2.961
UQ	4	46439401	46439700	0.876	1.450	1.790	1.298
UQ	4	48507301	48507600	0.452	0.260	0.239	0.634
UQ	4	48676501	48676800	0.795	0.189	0.346	0.472
UQ	4	48841201	48841500	1.676	1.025	0.867	1.237
UQ	4	48999301	48999600	1.916	1.424	1.246	1.577
UQ	4	49275001	49275300	0.718	0.257	0.254	0.086
UQ	4	49275301	49275600	1.381	0.466	0.522	0.370
UQ	4	49430101	49430400	0.799	0.258	0.354	0.466
UQ	4	49938901	49939200	0.848	0.185	0.127	0.411
UQ	4	50831701	50832000	0.187	0.701	0.646	0.472
UQ	4	50836201	50836500	1.731	1.051	1.168	1.267
UQ	4	53003401	53003700	0.186	0.538	0.555	0.492
UQ	4	53466301	53466600	0.106	0.515	0.317	0.411
UQ	4	55666201	55666500	1.987	3.090	2.954	3.301
UQ	4	55759501	55759800	4.018	2.617	2.962	3.483
UQ	4	56087401	56087700	0.558	0.257	0.267	0.324
UQ	4	57048001	57048300	2.657	1.076	0.386	0.288
UQ	4	58791601	58791900	0.398	0.468	0.132	0.472
UQ	4	58845001	58845300	0.106	0.657	0.374	0.614
UQ	4	59042401	59042700	0.615	0.096	0.127	0.274
UQ	4	60432901	60433200	0.826	0.468	0.214	0.426
UQ	4	60688501	60688800	2.234	1.824	2.669	1.744
UQ	4	61048801	61049100	0.690	0.352	0.267	0.441
UQ	4	61076701	61077000	0.424	0.562	0.243	0.248

UQ	4	61642801	61643100	0.160	0.466	0.239	0.360
UQ	4	62002801	62003100	0.531	0.798	0.160	0.193
UQ	4	62252701	62253000	0.319	0.985	0.638	0.588
UQ	4	62480701	62481000	0.187	0.328	0.292	0.497
UQ	4	62959801	62960100	0.534	0.212	0.165	0.112
UQ	4	64328401	64328700	2.239	1.384	1.177	1.050
UQ	4	64537801	64538100	0.824	0.258	0.386	0.329
UQ	4	64538101	64538400	0.720	0.421	0.218	0.421
UQ	4	64596001	64596300	0.907	0.241	0.078	0.309
UQ	4	64765501	64765800	1.113	0.140	0.165	0.086
UQ	4	65231101	65231400	0.428	0.980	0.814	0.882
UQ	4	65609101	65609400	0.878	0.423	0.272	0.421
UQ	4	66055801	66056100	0.534	0.161	0.189	0.132
UQ	4	66118201	66118500	0.476	0.212	0.136	0.167
UQ	4	66147001	66147300	1.192	0.749	0.555	0.771
UQ	4	66377701	66378000	0.768	0.420	0.346	0.380
UQ	4	66427801	66428100	0.531	0.279	0.185	0.187
UQ	4	66445201	66445500	2.818	3.669	1.409	1.982
UQ	4	66454801	66455100	0.186	0.444	0.078	0.304
UQ	4	66484501	66484800	0.534	0.142	0.181	0.187
UQ	4	66492001	66492300	0.639	0.213	0.296	0.309
UQ	4	66492301	66492600	0.560	0.140	0.189	0.248
UQ	4	67473601	67473900	1.353	1.706	0.852	0.927
UQ	4	68026201	68026500	1.333	0.536	0.584	0.628
UQ	4	68048701	68049000	3.118	2.156	1.567	1.825
UQ	4	68924701	68925000	5.096	3.583	3.393	3.467
UQ	4	69295201	69295500	2.575	1.330	1.616	1.576
UQ	4	69320401	69320700	1.621	0.997	0.662	0.619
UQ	4	69341101	69341400	0.106	0.350	0.535	0.391
UQ	4	69427801	69428100	0.665	0.210	0.506	0.852
UQ	4	69608101	69608400	1.010	1.167	1.173	0.857
UQ	4	70315501	70315800	1.299	0.629	0.272	0.533
UQ	4	70398601	70398900	0.453	0.350	0.239	0.664
UQ	4	70718701	70719000	0.852	1.191	1.329	1.197
UQ	4	70730401	70730700	0.319	0.187	0.453	0.497
UQ	4	70938301	70938600	0.586	0.167	0.185	0.284
UQ	4	71214601	71214900	0.531	0.142	0.214	0.187
UQ	4	71755201	71755500	0.558	0.303	0.296	0.304
UQ	4	71965501	71965800	0.371	0.701	0.987	0.776
UQ	4	73949101	73949400	0.557	1.240	1.501	1.161
UQ	4	73949401	73949700	0.421	1.343	1.608	1.445
UQ	4	74335501	74335800	0.931	0.489	0.695	0.385
UQ	4	74912401	74912700	0.661	1.639	1.081	1.511
UQ	4	74949601	74949900	1.118	0.652	0.493	0.517
UQ	4	76059001	76059300	0.531	1.049	0.745	0.806
UQ	4	76218901	76219200	1.621	1.058	1.173	1.080
UQ	4	76249801	76250100	0.718	0.352	0.054	0.106
UQ	4	76377001	76377300	1.195	0.511	0.382	0.614
UQ	4	77274301	77274600	0.663	0.397	0.181	0.309
UQ	4	77295301	77295600	0.213	0.562	0.160	0.304
UQ	4	77364001	77364300	3.964	2.154	2.811	3.291
UQ	4	77412301	77412600	0.477	0.236	0.185	0.187
UQ	4	77489701	77490000	0.986	1.356	0.575	0.634
UQ	4	77603401	77603700	0.670	0.142	0.194	0.193
UQ	4	77665501	77665800	0.481	0.257	0.107	0.304
UQ	4	77787001	77787300	0.558	0.094	0.214	0.243
UQ	4	78412801	78413100	2.768	0.732	0.506	0.543
UQ	4	78413101	78413400	1.013	0.375	0.263	0.376
UQ	4	78964201	78964500	0.477	0.210	0.288	0.248
UQ	4	79119001	79119300	1.223	0.916	0.901	0.715
UQ	4	79575001	79575300	0.532	0.303	0.292	0.274
UQ	4	80182801	80183100	0.716	0.373	0.379	0.411
UQ	4	80311801	80312100	0.615	0.305	0.317	0.223
UQ	4	81895801	81896100	0.481	0.189	0.136	0.112
UQ	4	82191301	82191600	0.292	1.899	2.965	1.429
UQ	4	82207501	82207800	0.319	0.607	0.160	0.218
UQ	4	83243701	83244000	0.615	0.191	0.218	0.081

UQ	4	83337901	83338200	3.122	1.424	1.946	2.286
UQ	4	84900601	84900900	1.254	0.629	0.457	0.913
UQ	4	85317601	85317900	0.505	0.328	0.321	0.274
UQ	4	87044101	87044400	0.532	0.258	0.214	0.360
UQ	4	87890101	87890400	0.477	0.234	0.296	0.086
UQ	4	87945301	87945600	0.639	0.375	0.321	0.472
UQ	4	88116901	88117200	0.295	0.747	0.823	0.558
UQ	4	88212001	88212300	0.558	0.466	0.267	0.248
UQ	4	88388701	88389000	0.613	0.137	0.165	0.243
UQ	4	89932201	89932500	0.716	0.375	0.288	0.436
UQ	4	90340801	90341100	1.671	0.567	0.798	0.741
UQ	4	90909001	90909300	0.505	0.281	0.103	0.274
UQ	4	91411201	91411500	0.721	0.096	0.127	0.173
UQ	4	91411501	91411800	2.350	0.453	0.546	0.598
UQ	4	92037301	92037600	0.106	0.420	0.239	0.248
UQ	4	92150401	92150700	0.239	0.820	0.424	0.446
UQ	4	92361001	92361300	0.692	0.466	0.234	0.355
UQ	4	92478001	92478300	2.902	2.182	1.790	2.211
UQ	4	92754001	92754300	1.200	0.493	0.564	0.684
UQ	4	93644701	93645000	1.058	0.584	0.239	0.527
UQ	4	94150201	94150500	1.887	0.875	0.930	0.669
UQ	4	94215901	94216200	0.477	0.045	0.214	0.132
UQ	4	94555801	94556100	0.987	0.656	0.642	0.411
UQ	4	94743301	94743600	2.930	0.929	1.558	1.936
UQ	4	95333101	95333400	1.223	0.777	0.740	0.472
UQ	4	95466301	95466600	0.744	0.215	0.103	0.228
UQ	4	95524801	95525100	0.660	1.734	1.522	2.454
UQ	4	95571901	95572200	1.091	0.678	1.650	1.298
UQ	4	95647801	95648100	0.611	0.444	0.395	0.254
UQ	4	95793901	95794200	0.507	0.328	0.292	0.360
UQ	4	95848501	95848800	0.931	0.307	0.321	0.659
UQ	4	95889001	95889300	1.144	0.375	0.905	0.669
UQ	4	95946001	95946300	1.197	0.146	0.160	0.274
UQ	4	96012601	96012900	0.960	0.472	0.453	0.446
UQ	4	96101701	96102000	0.558	0.373	0.239	0.218
UQ	4	96141001	96141300	0.558	0.281	0.448	0.167
UQ	4	96147001	96147300	0.610	0.328	0.296	0.335
UQ	4	96320101	96320400	0.826	1.446	1.683	1.323
UQ	4	96347401	96347700	1.065	0.487	0.243	0.740
UQ	4	96375601	96375900	1.568	1.056	1.061	0.365
UQ	4	96456001	96456300	0.821	1.098	1.119	1.496
UQ	4	96456901	96457200	1.036	2.154	1.741	2.297
UQ	4	97527301	97527600	0.852	0.449	0.317	0.380
UQ	4	97660201	97660500	0.505	0.373	0.370	0.360
UQ	4	98359801	98360100	4.826	1.433	1.394	1.029
UQ	4	98360101	98360400	3.476	0.822	1.168	0.846
UQ	4	98374501	98374800	1.808	2.249	1.387	1.303
UQ	4	99385801	99386100	0.373	0.701	0.078	0.137
UQ	4	99540601	99540900	0.560	0.255	0.185	0.335
UQ	4	100202701	100203000	0.611	0.328	0.214	0.329
UQ	4	100992001	100992300	1.302	0.628	0.729	0.751
UQ	4	101184001	101184300	0.876	0.163	0.239	0.745
UQ	4	101259901	101260200	0.292	0.538	0.346	0.193
UQ	4	101610301	101610600	0.584	0.444	0.243	0.441
UQ	4	101708401	101708700	1.120	0.324	0.111	0.142
UQ	4	101843101	101843400	1.115	0.678	0.658	0.497
UQ	4	101843401	101843700	1.353	0.824	0.682	0.639
UQ	4	102239101	102239400	1.544	1.075	2.040	1.141
UQ	4	102381301	102381600	2.531	1.367	1.480	1.572
UQ	4	102432001	102432300	1.145	0.305	0.905	0.684
UQ	4	102910201	102910500	0.824	0.583	0.321	0.517
UQ	4	102944101	102944400	3.086	2.244	1.469	1.987
UQ	4	102951901	102952200	0.636	0.914	1.061	0.943
UQ	4	103219501	103219800	2.623	4.023	3.687	4.416
UQ	4	103569901	103570200	0.531	0.350	0.214	0.355
UQ	4	104209801	104210100	2.791	2.051	2.238	2.130
UQ	4	104445001	104445300	0.610	2.191	2.757	3.048

UQ	4	104445301	104445600	0.450	0.963	1.226	1.455
UQ	4	104488501	104488800	0.452	0.116	0.218	0.639
UQ	4	104857801	104858100	4.359	3.459	2.936	3.292
UQ	4	104862001	104862300	0.611	0.285	0.243	0.243
UQ	4	105073501	105073800	0.690	0.350	0.210	0.340
UQ	4	105126901	105127200	0.902	0.238	0.428	0.431
UQ	4	105300001	105300300	0.477	0.751	0.740	0.745
UQ	4	105525901	105526200	0.532	0.184	0.185	0.218
UQ	4	105667501	105667800	0.505	0.210	0.181	0.360
UQ	4	106715401	106715700	0.718	0.560	0.395	0.882
UQ	4	106877401	106877700	0.848	0.146	0.263	0.198
UQ	4	107147401	107147700	0.799	0.350	0.584	0.416
UQ	4	107262601	107262900	2.045	1.659	1.436	1.323
UQ	4	107262901	107263200	3.217	2.412	1.908	2.226
UQ	4	109245301	109245600	0.481	0.045	0.189	0.051
UQ	4	109567501	109567800	0.344	0.910	0.132	0.938
UQ	4	109758601	109758900	2.733	2.055	1.781	2.043
UQ	4	110105701	110106000	0.531	0.678	0.346	0.441
UQ	4	110221201	110221500	0.716	0.350	0.432	0.416
UQ	4	110318701	110319000	3.270	2.875	2.299	2.398
UQ	4	110503801	110504100	0.477	1.008	1.057	0.248
UQ	4	110504101	110504400	1.273	2.292	1.971	0.725
UQ	4	110881201	110881500	1.620	0.614	0.901	0.907
UQ	4	110881501	110881800	1.087	0.423	0.560	0.436
UQ	4	110907901	110908200	2.526	3.066	2.171	3.737
UQ	4	111360301	111360600	4.089	3.253	3.344	2.895
UQ	4	111699901	111700200	3.213	2.343	1.810	2.185
UQ	4	111714001	111714300	2.921	1.802	1.313	1.491
UQ	4	111765301	111765600	3.759	2.336	1.233	2.049
UQ	4	111765601	111765900	2.615	1.665	0.881	1.481
UQ	4	111772801	111773100	1.515	1.098	1.164	1.222
UQ	4	111915001	111915300	1.382	0.663	0.374	0.634
UQ	4	112112101	112112400	3.851	3.152	3.348	4.670
UQ	4	112119301	112119600	2.287	1.517	1.674	1.876
UQ	4	112304101	112304400	0.637	0.348	0.346	0.365
UQ	4	112430101	112430400	0.347	0.538	0.103	0.051
UQ	4	112924201	112924500	0.611	0.236	0.185	0.213
UQ	4	112941901	112942200	0.663	0.215	0.103	0.167
UQ	4	112955701	112956000	1.470	3.085	3.357	2.863
UQ	4	113287501	113287800	0.560	0.114	0.185	0.329
UQ	4	113370601	113370900	0.424	0.678	0.132	0.213
UQ	4	113387101	113387400	3.830	4.164	2.769	3.285
UQ	4	113543701	113544000	4.728	3.577	3.380	3.600
UQ	4	113646901	113647200	0.845	1.686	1.505	1.485
UQ	4	113713201	113713500	3.242	2.504	2.423	2.531
UQ	4	113751001	113751300	2.426	1.798	1.411	1.156
UQ	4	113751301	113751600	2.188	1.264	1.259	1.096
UQ	4	113914501	113914800	0.878	0.350	0.588	0.715
UQ	4	113973301	113973600	1.707	1.099	0.778	0.938
UQ	4	114159301	114159600	0.452	0.586	0.082	0.223
UQ	4	114276301	114276600	0.319	0.491	0.214	0.583
UQ	4	114307801	114308100	0.799	0.373	0.337	0.370
UQ	4	114352201	114352500	1.468	0.798	0.909	0.639
UQ	4	114479101	114479400	0.052	0.513	0.185	0.248
UQ	4	114590701	114591000	3.242	1.641	2.323	2.200
UQ	4	114592801	114593100	0.505	0.375	0.292	0.304
UQ	4	114779101	114779400	1.570	0.611	0.629	0.806
UQ	4	114834901	114835200	5.719	3.267	4.026	4.552
UQ	4	114893401	114893700	2.121	1.571	0.901	0.907
UQ	4	114898801	114899100	0.637	1.352	0.901	0.913
UQ	4	114981601	114981900	1.195	0.331	0.303	0.497
UQ	4	115079701	115080000	1.249	0.794	0.720	0.902
UQ	4	115103401	115103700	2.176	0.815	0.332	0.269
UQ	4	115208401	115208700	4.915	2.791	3.092	3.225
UQ	4	115208701	115209000	3.439	1.223	1.407	1.628
UQ	4	115218901	115219200	0.507	0.092	0.156	0.132
UQ	4	115624201	115624500	0.745	0.466	0.370	0.268

UQ	4	115633801	115634100	0.665	1.543	1.284	1.343
UQ	4	115662001	115662300	1.650	1.030	1.074	1.060
UQ	4	115748401	115748700	1.942	0.981	1.181	1.602
UQ	4	115770601	115770900	0.634	0.165	0.140	0.142
UQ	4	115802701	115803000	2.791	1.339	1.393	1.460
UQ	4	115893301	115893600	0.720	0.562	0.584	0.416
UQ	4	115908301	115908600	0.582	0.023	0.185	0.173
UQ	4	115967101	115967400	2.283	0.701	0.992	1.237
UQ	4	115967401	115967700	2.367	1.171	1.411	1.181
UQ	4	115998001	115998300	1.755	1.403	1.259	0.938
UQ	4	116192401	116192700	2.341	0.757	0.573	5.897
UQ	4	116194801	116195100	4.550	2.287	2.008	7.389
UQ	4	116197501	116197800	1.195	1.405	1.888	1.663
UQ	4	116431201	116431500	1.649	0.277	0.272	0.304
UQ	4	116608201	116608500	2.078	1.476	1.460	1.359
UQ	4	116736301	116736600	3.489	2.268	2.448	2.900
UQ	4	116850001	116850300	3.184	3.905	2.555	4.533
UQ	4	117219901	117220200	4.068	3.250	2.818	3.113
UQ	4	117498301	117498600	0.800	0.189	0.210	0.370
UQ	4	117887701	117888000	1.568	1.826	1.781	1.906
UQ	4	118003501	118003800	8.363	7.065	7.034	7.033
UQ	4	118003801	118004100	3.912	2.544	3.097	2.977
UQ	4	118108501	118108800	4.228	4.606	3.496	3.245
UQ	4	118221301	118221600	1.963	1.191	1.226	0.907
UQ	4	118221601	118221900	3.657	2.066	2.280	1.557
UQ	4	118271401	118271700	2.363	1.663	1.514	1.770
UQ	4	118697701	118698000	2.049	1.152	1.353	1.424
UQ	4	118759201	118759500	2.624	1.538	1.645	1.145
UQ	4	119198401	119198700	0.720	0.444	0.560	0.553
UQ	5	301201	301500	0.557	0.114	0.082	0.243
UQ	5	1114801	1115100	1.915	1.452	1.041	1.212
UQ	5	1168501	1168800	0.771	0.517	0.506	0.243
UQ	5	1209601	1209900	0.560	0.212	0.127	0.254
UQ	5	1637701	1638000	0.584	0.350	0.370	0.472
UQ	5	1870201	1870500	0.982	1.285	0.564	0.598
UQ	5	2337001	2337300	0.720	0.421	0.555	0.304
UQ	5	2640901	2641200	2.396	2.926	2.851	3.833
UQ	5	2761201	2761500	4.147	2.688	3.065	3.615
UQ	5	3817501	3817800	0.186	0.328	0.555	0.385
UQ	5	3996001	3996300	2.128	1.216	0.575	0.897
UQ	5	4018801	4019100	0.878	0.253	0.210	0.263
UQ	5	4019101	4019400	0.718	0.068	0.165	0.157
UQ	5	4197601	4197900	2.423	0.785	1.473	1.359
UQ	5	4303201	4303500	2.396	1.476	1.346	1.577
UQ	5	4438501	4438800	2.705	3.626	2.057	3.910
UQ	5	4521901	4522200	1.171	0.702	0.642	0.704
UQ	5	4555201	4555500	1.492	1.798	1.173	1.192
UQ	5	4890901	4891200	0.557	0.328	0.263	0.162
UQ	5	4952701	4953000	1.996	0.863	1.518	1.512
UQ	5	4998601	4998900	1.226	1.590	1.534	1.987
UQ	5	5155201	5155500	1.432	0.536	0.769	0.943
UQ	5	5160601	5160900	0.292	0.493	0.136	0.497
UQ	5	5248501	5248800	1.065	0.024	0.145	0.213
UQ	5	5391601	5391900	0.294	0.446	0.107	0.167
UQ	5	5769901	5770200	0.345	0.609	0.453	0.664
UQ	5	5883901	5884200	4.869	3.613	3.210	3.858
UQ	5	6743701	6744000	0.106	0.234	0.239	0.466
UQ	5	6915901	6916200	0.398	0.212	0.613	0.198
UQ	5	6944101	6944400	0.639	0.257	0.152	0.137
UQ	5	6944401	6944700	0.745	0.330	0.176	0.157
UQ	5	7147501	7147800	4.254	1.858	3.059	3.058
UQ	5	7277101	7277400	0.266	0.654	0.769	0.740
UQ	5	7424101	7424400	3.057	2.641	2.477	2.180
UQ	5	7653301	7653600	2.315	1.594	1.855	1.937
UQ	5	7824301	7824600	0.532	0.283	0.156	0.162
UQ	5	8248801	8249100	1.966	4.433	3.775	4.296
UQ	5	8606101	8606400	1.116	0.444	0.292	0.446

UQ	5	9103801	9104100	0.531	0.302	0.243	0.112
UQ	5	9159001	9159300	0.852	0.281	0.453	0.396
UQ	5	9493801	9494100	1.595	2.454	2.044	2.327
UQ	5	9569401	9569700	0.186	1.006	0.633	0.553
UQ	5	9625801	9626100	0.955	0.378	0.354	0.299
UQ	5	9866401	9866700	4.672	1.428	1.953	1.836
UQ	5	9866701	9867000	1.629	0.483	0.497	0.492
UQ	5	10121401	10121700	0.769	0.326	0.350	0.248
UQ	5	10294501	10294800	0.690	0.069	0.234	0.223
UQ	5	10303201	10303500	0.424	0.281	0.588	0.223
UQ	5	10314301	10314600	0.106	0.532	0.477	0.497
UQ	5	10599301	10599600	0.587	0.159	0.054	0.218
UQ	5	10897801	10898100	0.186	0.303	0.428	0.527
UQ	5	11141701	11142000	1.065	0.631	0.535	0.547
UQ	5	11219701	11220000	0.481	0.163	0.160	0.142
UQ	5	11319901	11320200	2.257	1.273	1.197	1.460
UQ	5	11610001	11610300	0.718	0.206	0.165	0.162
UQ	5	11708401	11708700	0.637	0.285	0.259	0.031
UQ	5	11726401	11726700	0.319	0.444	0.185	0.167
UQ	5	12066601	12066900	0.823	0.182	0.185	0.396
UQ	5	12123901	12124200	1.118	0.423	0.609	0.669
UQ	5	12172201	12172500	0.905	0.397	0.473	0.497
UQ	5	12319201	12319500	0.503	0.185	0.107	0.137
UQ	5	12370801	12371100	1.250	0.818	0.662	0.684
UQ	5	12483901	12484200	0.613	0.208	0.054	0.213
UQ	5	12484201	12484500	0.477	0.139	0.000	0.132
UQ	5	12659701	12660000	4.046	3.527	2.867	3.088
UQ	5	13125601	13125900	0.742	0.330	0.317	0.268
UQ	5	13273801	13274100	0.452	1.004	0.774	0.801
UQ	5	13412401	13412700	0.184	0.442	0.481	0.578
UQ	5	13424401	13424700	0.928	1.264	0.609	0.664
UQ	5	14185501	14185800	0.239	0.350	0.374	0.527
UQ	5	14381701	14382000	1.165	0.607	0.584	0.441
UQ	5	14836201	14836500	0.665	0.446	0.424	0.436
UQ	5	15411601	15411900	1.276	0.912	1.012	0.938
UQ	5	15707401	15707700	1.486	0.860	0.522	0.710
UQ	5	15780601	15780900	0.903	0.277	0.448	0.553
UQ	5	15915901	15916200	0.505	0.328	0.321	0.304
UQ	5	15985501	15985800	3.639	3.042	2.604	4.031
UQ	5	16143301	16143600	2.502	1.641	1.761	1.714
UQ	5	16206301	16206600	3.802	2.044	1.988	2.611
UQ	5	16352401	16352700	1.116	0.656	0.716	0.690
UQ	5	16373401	16373700	0.318	0.773	0.588	0.578
UQ	5	16542901	16543200	1.408	0.983	0.925	0.943
UQ	5	16569301	16569600	0.186	1.098	0.818	0.619
UQ	5	16853101	16853400	1.303	0.682	0.646	0.405
UQ	5	17165701	17166000	0.345	0.466	0.054	0.218
UQ	5	17206201	17206500	0.694	0.255	0.210	0.254
UQ	5	17240101	17240400	0.771	0.330	0.473	0.248
UQ	5	17240401	17240700	0.850	1.281	1.329	1.323
UQ	5	17345701	17346000	4.628	2.249	1.839	1.871
UQ	5	17354401	17354700	1.140	0.303	0.457	0.268
UQ	5	17570701	17571000	0.215	0.699	0.555	0.441
UQ	5	17725501	17725800	2.794	1.729	1.822	1.623
UQ	5	18015301	18015600	0.799	0.446	0.555	0.583
UQ	5	18905701	18906000	1.566	2.107	2.599	2.119
UQ	5	20045701	20046000	1.726	0.773	1.280	1.384
UQ	5	20073001	20073300	2.368	1.877	1.480	1.465
UQ	5	20967301	20967600	0.531	0.112	0.127	0.142
UQ	5	21424801	21425100	0.695	0.000	0.000	0.112
UQ	5	21688801	21689100	0.289	0.863	0.724	0.837
UQ	5	21950101	21950400	0.481	0.118	0.136	0.162
UQ	5	23292001	23292300	0.742	0.283	0.317	0.360
UQ	5	23810701	23811000	0.508	0.234	0.107	0.223
UQ	5	24143401	24143700	0.584	0.185	0.165	0.329
UQ	5	24459001	24459300	0.345	0.656	0.477	0.223
UQ	5	24640501	24640800	0.132	1.279	2.156	2.394

UQ	5	24663601	24663900	0.690	0.285	0.078	0.025
UQ	5	24792001	24792300	0.106	0.350	0.160	0.553
UQ	5	26483401	26483700	0.558	0.116	0.205	0.248
UQ	5	26817601	26817900	0.558	0.375	0.214	0.335
UQ	5	26895901	26896200	0.450	0.068	0.078	0.056
UQ	5	29962501	29962800	2.497	1.777	1.835	2.023
UQ	5	30365401	30365700	1.487	0.678	1.070	1.136
UQ	5	30396001	30396300	0.740	0.212	0.267	0.000
UQ	5	31077001	31077300	0.531	0.399	0.292	0.193
UQ	5	31769401	31769700	1.037	0.285	0.341	0.142
UQ	5	33102001	33102300	0.161	0.489	0.827	0.573
UQ	5	33140101	33140400	0.907	0.423	0.408	0.315
UQ	5	33790801	33791100	0.613	0.255	0.082	0.203
UQ	5	34338301	34338600	1.387	2.561	0.388	2.904
UQ	5	35075401	35075700	0.640	2.416	2.832	2.028
UQ	5	35317501	35317800	0.503	0.890	1.119	0.720
UQ	5	35486401	35486700	0.745	0.305	0.234	0.304
UQ	5	35557201	35557500	0.507	0.305	0.185	0.193
UQ	5	35672401	35672700	0.531	0.279	0.267	0.243
UQ	5	36356401	36356700	0.373	0.676	0.724	0.882
UQ	5	36555601	36555900	1.460	0.916	0.704	0.583
UQ	5	37133101	37133400	0.055	0.440	0.588	0.517
UQ	5	38412001	38412300	1.195	0.749	0.729	0.634
UQ	5	38956201	38956500	0.907	0.118	0.383	0.147
UQ	5	39287101	39287400	0.211	0.491	0.403	0.416
UQ	5	40584301	40584600	0.158	0.448	0.477	0.446
UQ	5	40727401	40727700	1.355	1.191	0.798	0.695
UQ	5	40727701	40728000	1.437	0.678	0.510	0.644
UQ	5	41203801	41204100	1.673	1.098	0.999	1.070
UQ	5	41236501	41236800	0.984	0.843	0.667	1.359
UQ	5	42076501	42076800	1.279	1.849	0.642	0.720
UQ	5	42324601	42324900	1.037	0.749	0.642	0.472
UQ	5	42359401	42359700	0.237	0.444	0.613	0.441
UQ	5	43124401	43124700	0.534	0.375	0.160	0.081
UQ	5	44000701	44001000	0.398	0.539	0.185	0.274
UQ	5	44290201	44290500	0.558	0.114	0.111	0.167
UQ	5	45088201	45088500	0.477	0.212	0.239	0.254
UQ	5	45402601	45402900	0.689	0.118	0.152	0.173
UQ	5	45552901	45553200	3.994	2.545	3.048	3.438
UQ	5	45919501	45919800	0.558	0.375	0.317	0.299
UQ	5	46044001	46044300	0.797	0.348	0.502	0.421
UQ	5	46075201	46075500	0.477	0.303	0.317	0.335
UQ	5	46440301	46440600	0.797	1.053	1.295	1.105
UQ	5	47325001	47325300	0.824	0.538	0.481	0.547
UQ	5	48502201	48502500	0.268	0.654	0.424	0.608
UQ	5	48786001	48786300	1.195	0.980	0.823	0.938
UQ	5	48817501	48817800	1.707	1.056	1.099	1.050
UQ	5	48833701	48834000	0.510	1.075	1.099	0.999
UQ	5	48957001	48957300	0.507	0.255	0.127	0.193
UQ	5	49228801	49229100	0.507	0.094	0.205	0.142
UQ	5	49834501	49834800	0.081	0.326	0.341	0.492
UQ	5	49905901	49906200	1.784	1.075	1.057	2.257
UQ	5	49935901	49936200	0.666	0.330	0.366	0.274
UQ	5	50706901	50707200	0.584	0.185	0.107	0.193
UQ	5	50927701	50928000	0.160	0.493	0.432	0.441
UQ	5	51003901	51004200	1.171	0.539	0.481	0.837
UQ	5	52066801	52067100	0.507	0.891	0.769	0.832
UQ	5	52446001	52446300	0.371	0.631	0.477	0.193
UQ	5	52735801	52736100	0.477	0.140	0.136	0.076
UQ	5	53340901	53341200	0.507	0.305	0.711	0.167
UQ	5	53419501	53419800	0.266	0.562	0.428	0.695
UQ	5	53673001	53673300	1.678	0.820	0.626	0.405
UQ	5	54415801	54416100	0.503	0.165	0.165	0.173
UQ	5	54750601	54750900	0.476	0.189	0.136	0.167
UQ	5	55160401	55160700	0.773	0.399	0.477	1.095
UQ	5	55395301	55395600	0.400	0.584	0.049	0.142
UQ	5	55582201	55582500	0.850	0.629	0.477	0.608

UQ	5	56125201	56125500	0.158	0.628	0.675	0.608
UQ	5	56247901	56248200	0.452	0.562	0.321	0.583
UQ	5	56614801	56615100	0.503	0.185	0.160	0.193
UQ	5	56838001	56838300	0.426	0.236	0.185	0.553
UQ	5	56887801	56888100	0.957	0.583	0.716	0.533
UQ	5	57030901	57031200	1.252	0.547	0.481	0.608
UQ	5	57381001	57381300	1.037	0.729	0.584	0.715
UQ	5	57647101	57647400	0.160	0.375	0.477	0.441
UQ	5	58353901	58354200	0.505	0.094	0.152	0.076
UQ	5	58665001	58665300	0.081	0.466	0.210	0.527
UQ	5	58969201	58969500	0.720	0.230	0.218	0.365
UQ	5	58984501	58984800	0.479	0.796	0.239	0.218
UQ	5	59071801	59072100	2.554	1.562	2.107	1.714
UQ	5	59072101	59072400	3.855	2.313	2.715	2.469
UQ	5	59282401	59282700	0.668	0.212	0.205	0.187
UQ	5	59410501	59410800	3.794	1.333	1.122	0.841
UQ	5	59410801	59411100	1.400	0.193	0.410	0.268
UQ	5	59534101	59534400	1.595	1.120	1.037	1.770
UQ	5	59970001	59970300	4.223	3.160	3.248	2.926
UQ	5	61233001	61233300	0.187	0.867	0.424	0.421
UQ	5	61389001	61389300	0.720	0.352	0.370	0.360
UQ	5	61804801	61805100	0.610	0.260	0.127	0.086
UQ	5	62605201	62605500	0.637	0.307	0.214	0.254
UQ	5	63331801	63332100	2.502	1.216	1.229	1.124
UQ	5	63377701	63378000	0.450	0.069	0.210	0.106
UQ	5	63913501	63913800	0.929	0.401	0.276	0.441
UQ	5	64519501	64519800	3.059	3.884	4.082	4.335
UQ	5	66496201	66496500	2.124	1.541	2.394	2.403
UQ	5	66908101	66908400	0.376	1.008	0.979	1.040
UQ	5	68006401	68006700	0.505	1.332	0.749	0.918
UQ	5	68732401	68732700	2.847	2.182	1.579	2.236
UQ	5	69400501	69400800	0.400	1.262	0.103	0.218
UQ	5	69437701	69438000	0.507	0.281	0.049	0.167
UQ	5	70151101	70151400	0.531	0.208	0.210	0.198
UQ	5	70548301	70548600	0.747	0.331	0.267	0.112
UQ	5	73993801	73994100	0.106	0.676	0.531	0.385
UQ	5	73994101	73994400	0.081	0.609	0.453	0.299
UQ	5	74873101	74873400	0.773	0.277	0.205	0.081
UQ	5	74962501	74962800	0.929	0.517	0.370	0.365
UQ	5	75128101	75128400	1.278	0.862	0.580	0.690
UQ	5	76371901	76372200	0.132	0.487	0.531	0.416
UQ	5	76460401	76460700	0.637	0.794	0.453	0.806
UQ	5	76592701	76593000	0.345	1.009	0.638	0.603
UQ	5	76913101	76913400	0.694	0.397	0.317	0.466
UQ	5	77273401	77273700	0.345	0.116	0.580	0.578
UQ	5	77343001	77343300	1.382	1.663	1.041	1.998
UQ	5	77536501	77536800	0.294	0.656	0.160	0.167
UQ	5	78480301	78480600	0.823	0.418	0.379	0.284
UQ	5	78580201	78580500	1.755	1.167	1.337	1.384
UQ	5	80601601	80601900	5.803	3.083	2.513	3.900
UQ	5	81462001	81462300	0.503	0.189	0.214	0.137
UQ	5	82397701	82398000	0.640	0.328	0.346	0.416
UQ	5	83172901	83173200	0.450	0.069	0.239	0.142
UQ	5	85013401	85013700	0.265	0.586	0.687	0.472
UQ	5	85132201	85132500	0.187	0.373	0.481	0.466
UQ	5	85545001	85545300	1.441	2.034	1.937	1.856
UQ	5	85827301	85827600	1.410	0.609	0.794	0.842
UQ	5	86759101	86759400	0.666	0.399	0.448	0.391
UQ	5	87020101	87020400	1.410	0.515	0.555	0.614
UQ	5	87020401	87020700	0.611	0.137	0.205	0.228
UQ	5	87203701	87204000	0.477	1.214	0.691	0.725
UQ	5	87291901	87292200	0.106	0.468	0.457	0.365
UQ	5	87555901	87556200	0.960	0.307	0.279	0.421
UQ	5	88690801	88691100	0.242	0.727	0.667	0.740
UQ	5	88812001	88812300	0.853	0.305	0.428	0.405
UQ	5	88812301	88812600	1.092	0.521	0.551	0.461
UQ	5	88971601	88971900	0.187	0.468	0.399	0.304

UQ	5	89070901	89071200	3.054	1.929	1.168	0.704
UQ	5	89854801	89855100	0.718	0.330	0.379	0.268
UQ	5	89991901	89992200	0.239	0.421	0.477	0.421
UQ	5	91127101	91127400	0.213	0.701	0.374	0.472
UQ	5	91177801	91178100	1.673	0.522	1.003	1.131
UQ	5	91498801	91499100	2.441	0.742	0.749	0.786
UQ	5	91557601	91557900	1.628	0.747	0.729	0.999
UQ	5	91902301	91902600	0.369	0.794	0.691	1.050
UQ	5	91953601	91953900	1.066	0.674	0.749	0.329
UQ	5	92015101	92015400	0.132	0.584	0.370	0.279
UQ	5	92413201	92413500	0.340	0.863	0.847	0.862
UQ	5	92802601	92802900	1.491	1.004	0.700	1.141
UQ	5	93099001	93099300	1.192	1.942	1.703	2.135
UQ	5	93233401	93233700	2.815	0.921	1.830	0.725
UQ	5	93816001	93816300	2.128	1.455	1.222	1.363
UQ	5	95327101	95327400	5.006	3.553	3.351	3.443
UQ	5	95458501	95458800	0.747	0.281	0.239	0.466
UQ	5	95612701	95613000	0.477	0.161	0.107	0.187
UQ	5	96566401	96566700	0.639	0.935	0.959	0.994
UQ	5	97083301	97083600	0.345	0.798	0.613	0.614
UQ	5	97444801	97445100	0.558	0.281	0.247	0.274
UQ	5	97696501	97696800	0.479	0.747	0.852	0.776
UQ	5	97913401	97913700	0.640	0.961	0.905	0.416
UQ	5	98051101	98051400	0.771	0.348	0.526	0.279
UQ	5	98531701	98532000	0.105	0.536	0.658	0.416
UQ	5	98990101	98990400	0.558	0.281	0.395	0.081
UQ	5	99192601	99192900	1.618	1.099	1.135	1.192
UQ	5	99534601	99534900	0.373	0.961	0.662	0.745
UQ	5	99702901	99703200	0.560	0.232	0.078	0.081
UQ	5	101087101	101087400	1.215	0.213	0.281	0.248
UQ	5	102249601	102249900	0.105	1.002	0.695	0.710
UQ	5	102249901	102250200	1.539	2.931	2.932	3.093
UQ	5	102328201	102328500	0.797	0.583	0.341	0.355
UQ	5	102336901	102337200	0.557	0.326	0.103	0.274
UQ	5	102555601	102555900	0.957	0.257	0.539	0.431
UQ	5	102784501	102784800	0.477	0.258	0.267	0.218
UQ	5	103052701	103053000	0.481	0.116	0.239	0.304
UQ	5	103249501	103249800	0.694	0.168	0.214	0.187
UQ	5	103787401	103787700	1.091	1.380	0.584	0.857
UQ	5	103912201	103912500	6.274	5.046	3.885	4.517
UQ	5	103980601	103980900	0.850	0.491	0.642	0.664
UQ	5	104247001	104247300	0.745	0.423	0.189	0.421
UQ	5	104552101	104552400	0.531	0.118	0.123	0.081
UQ	5	104566501	104566800	2.078	3.416	2.608	3.006
UQ	5	104624701	104625000	0.744	0.515	0.613	0.527
UQ	5	104916901	104917200	0.586	0.096	0.321	0.284
UQ	5	104929201	104929500	2.897	1.240	1.605	0.932
UQ	5	105019501	105019800	2.710	1.553	0.560	1.759
UQ	5	105347401	105347700	2.025	1.178	1.164	1.100
UQ	5	105465001	105465300	0.587	0.212	0.103	0.117
UQ	5	105490801	105491100	0.613	0.303	0.374	0.279
UQ	5	105611401	105611700	0.345	1.242	0.580	0.862
UQ	5	106049401	106049700	0.584	1.635	1.246	2.110
UQ	5	106049701	106050000	0.532	1.399	1.135	1.496
UQ	5	106456201	106456500	0.584	0.159	1.003	1.430
UQ	5	106460701	106461000	1.011	0.611	0.535	0.578
UQ	5	107114101	107114400	2.576	1.597	1.469	1.917
UQ	5	107117401	107117700	1.278	2.350	2.209	2.231
UQ	6	101401	101700	1.194	0.820	0.847	0.771
UQ	6	225601	225900	0.558	0.796	0.374	0.720
UQ	6	259501	259800	1.915	1.401	2.316	1.739
UQ	6	1351801	1352100	1.434	1.047	0.827	0.745
UQ	6	2204101	2204400	0.534	0.213	0.029	0.223
UQ	6	3330001	3330300	1.355	0.865	1.037	1.546
UQ	6	3358201	3358500	0.505	0.167	0.127	0.187
UQ	6	3384301	3384600	0.847	0.399	0.453	0.335
UQ	6	3576301	3576600	0.557	0.210	0.189	0.335

UQ	6	3765301	3765600	1.144	1.028	1.703	1.410
UQ	6	3793201	3793500	0.345	0.421	0.506	0.223
UQ	6	4561801	4562100	1.520	0.817	0.597	0.938
UQ	6	4711201	4711500	4.843	3.031	3.298	3.924
UQ	6	4716601	4716900	1.884	0.286	0.301	0.689
UQ	6	5687101	5687400	0.611	0.283	0.428	0.329
UQ	6	5876701	5877000	1.487	1.124	1.115	1.060
UQ	6	6060001	6060300	0.639	0.466	0.374	0.254
UQ	6	6739201	6739500	0.213	0.466	0.321	0.360
UQ	6	6832801	6833100	0.532	0.305	0.267	0.248
UQ	6	7041001	7041300	0.187	0.513	0.054	0.391
UQ	6	7141801	7142100	0.350	1.751	2.385	1.819
UQ	6	7142101	7142400	0.515	1.912	2.385	1.819
UQ	6	7232401	7232700	0.479	0.656	0.346	0.385
UQ	6	7560301	7560600	0.611	0.167	0.317	0.360
UQ	6	8386201	8386500	0.639	0.191	0.111	0.126
UQ	6	8823301	8823600	0.821	0.324	0.424	0.248
UQ	6	9003601	9003900	1.594	0.369	2.697	2.885
UQ	6	9184501	9184800	0.663	0.468	0.424	0.472
UQ	6	11057701	11058000	0.771	0.493	0.317	0.441
UQ	6	11635201	11635500	0.106	0.607	0.029	0.193
UQ	6	11686801	11687100	0.531	0.069	0.136	0.360
UQ	6	11818801	11819100	0.690	0.303	0.312	0.299
UQ	6	11862301	11862600	0.477	0.092	0.210	0.279
UQ	6	12069001	12069300	0.824	0.539	0.535	0.446
UQ	6	12244801	12245100	3.505	2.446	1.786	1.881
UQ	6	12409201	12409500	3.962	3.113	3.054	2.175
UQ	6	12898801	12899100	0.797	0.401	0.165	0.385
UQ	6	13238101	13238400	0.503	0.163	0.156	0.198
UQ	6	13492201	13492500	0.611	0.281	0.453	0.416
UQ	6	13555201	13555500	0.505	0.584	0.769	0.608
UQ	6	14147401	14147700	0.932	0.303	0.622	0.274
UQ	6	14629201	14629500	3.615	1.712	1.524	1.053
UQ	6	15798301	15798600	0.294	0.444	0.156	0.441
UQ	6	16113301	16113600	0.613	0.281	0.132	0.162
UQ	6	16233901	16234200	1.813	0.094	0.747	0.364
UQ	6	16870501	16870800	0.505	0.279	0.165	0.167
UQ	6	17249401	17249700	1.278	0.890	0.765	0.832
UQ	6	17499601	17499900	0.052	0.305	0.448	0.335
UQ	6	17586001	17586300	1.570	0.938	1.144	0.918
UQ	6	17667601	17667900	0.213	0.446	0.054	0.329
UQ	6	18969601	18969900	0.560	0.187	0.160	0.360
UQ	6	20119501	20119800	0.637	1.146	0.477	0.497
UQ	6	20193901	20194200	4.712	6.044	6.139	6.313
UQ	6	20593801	20594100	1.647	1.032	1.201	0.988
UQ	6	20594101	20594400	1.570	1.056	1.173	1.004
UQ	6	21071701	21072000	0.160	0.747	0.428	0.806
UQ	6	21269101	21269400	0.505	0.212	0.194	0.198
UQ	6	22245601	22245900	0.771	1.167	1.242	1.075
UQ	6	22257301	22257600	0.505	0.350	0.078	0.274
UQ	6	22616401	22616700	0.426	0.768	0.774	0.913
UQ	6	22642501	22642800	0.373	0.468	0.506	0.193
UQ	6	23106601	23106900	0.055	0.395	0.374	0.335
UQ	6	23297701	23298000	1.492	0.725	0.477	0.655
UQ	6	23721001	23721300	0.345	0.656	0.054	0.132
UQ	6	23802901	23803200	0.318	0.466	0.054	0.081
UQ	6	24063301	24063600	0.132	0.468	0.214	0.385
UQ	6	24067801	24068100	1.037	0.584	0.831	0.527
UQ	6	24749701	24750000	0.292	0.234	0.477	0.167
UQ	6	25108801	25109100	0.690	0.258	0.296	0.497
UQ	6	25764901	25765200	0.105	0.281	0.506	0.304
UQ	6	25881001	25881300	0.881	0.328	0.374	0.365
UQ	6	26036401	26036700	1.326	0.378	0.568	0.623
UQ	6	26329801	26330100	2.102	1.317	1.460	1.009
UQ	6	26464201	26464500	0.666	0.305	0.000	0.203
UQ	6	26657401	26657700	0.532	0.049	0.350	0.137
UQ	6	26738701	26739000	0.821	0.491	0.317	0.391

UQ	6	26795101	26795400	0.268	0.069	0.560	0.112
UQ	6	27502801	27503100	2.336	1.360	1.505	1.395
UQ	6	28301401	28301700	0.960	0.468	0.535	0.573
UQ	6	28727101	28727400	0.373	0.560	0.103	0.248
UQ	6	29732101	29732400	1.329	0.888	0.789	0.923
UQ	6	29965201	29965500	0.503	0.326	0.132	0.137
UQ	6	30776701	30777000	1.170	0.867	0.983	0.715
UQ	6	30819601	30819900	0.477	0.094	0.103	0.223
UQ	6	31318201	31318500	0.027	0.491	0.136	0.223
UQ	6	31446901	31447200	1.379	0.581	0.711	0.771
UQ	6	31466701	31467000	3.223	1.742	1.333	1.917
UQ	6	32600701	32601000	0.749	0.069	0.152	0.187
UQ	6	33696601	33696900	4.443	1.946	3.081	2.854
UQ	6	34419001	34419300	0.106	0.330	0.321	0.553
UQ	6	35102401	35102700	0.850	0.399	0.444	0.497
UQ	6	35712301	35712600	0.795	0.465	0.477	1.100
UQ	6	35849401	35849700	1.300	0.678	0.626	0.695
UQ	6	36221701	36222000	1.118	0.682	0.506	0.765
UQ	6	36993901	36994200	0.689	0.328	0.370	0.441
UQ	6	37383601	37383900	0.428	0.257	0.025	0.583
UQ	6	38301601	38301900	0.503	0.187	0.049	0.117
UQ	6	38420401	38420700	0.295	0.699	0.736	0.852
UQ	6	39415201	39415500	0.239	0.631	0.473	0.659
UQ	6	39861601	39861900	0.507	0.023	0.140	0.193
UQ	6	40273801	40274100	0.613	0.305	0.346	0.187
UQ	6	41940601	41940900	0.507	0.302	0.292	0.167
UQ	6	42110101	42110400	1.462	0.137	0.580	0.584
UQ	6	42227701	42228000	0.611	0.187	0.239	0.162
UQ	6	42545101	42545400	0.108	0.566	0.638	0.517
UQ	6	43373101	43373400	0.744	0.255	0.350	0.193
UQ	6	43660501	43660800	0.476	0.165	0.132	0.218
UQ	6	44980801	44981100	1.092	0.423	0.555	0.502
UQ	6	45079201	45079500	0.503	0.163	0.082	0.274
UQ	6	45126301	45126600	0.134	0.727	0.560	0.533
UQ	6	45127201	45127500	0.558	0.912	0.716	0.913
UQ	6	45315001	45315300	0.771	0.401	0.239	0.405
UQ	6	45866401	45866700	0.613	0.279	0.054	0.213
UQ	6	46890001	46890300	1.360	0.571	0.575	0.527
UQ	6	47430001	47430300	0.587	0.255	0.292	0.299
UQ	6	48812101	48812400	0.079	0.444	0.288	0.274
UQ	6	49243501	49243800	0.560	0.213	0.103	0.112
UQ	6	49716601	49716900	1.862	1.073	0.856	1.024
UQ	6	50939701	50940000	0.481	0.137	0.127	0.137
UQ	6	51693301	51693600	1.197	0.936	0.983	0.988
UQ	6	52056901	52057200	1.597	1.127	1.173	1.217
UQ	6	52490701	52491000	1.910	0.431	0.785	0.862
UQ	6	52619701	52620000	0.508	0.139	0.136	0.137
UQ	6	52721101	52721400	0.479	1.245	1.057	0.988
UQ	6	53924101	53924400	0.639	0.397	0.074	0.365
UQ	6	54261901	54262200	0.186	0.772	0.811	0.634
UQ	6	55938601	55938900	0.453	0.210	0.695	0.061
UQ	6	55977601	55977900	0.826	0.631	1.222	0.299
UQ	6	56465401	56465700	2.746	1.768	1.460	1.957
UQ	6	56517001	56517300	1.674	0.888	0.999	0.994
UQ	6	56842501	56842800	1.231	2.100	2.361	1.993
UQ	6	57999001	57999300	0.426	0.584	0.560	0.329
UQ	6	58295401	58295700	0.715	0.167	0.214	0.228
UQ	6	59429701	59430000	0.931	0.682	1.304	0.639
UQ	6	59711401	59711700	0.529	0.096	0.189	0.112
UQ	6	59826601	59826900	1.547	0.841	0.716	0.466
UQ	6	59884501	59884800	0.505	0.912	0.901	0.796
UQ	6	59955001	59955300	1.752	0.862	1.362	0.887
UQ	6	59955601	59955900	0.986	0.468	0.691	0.533
UQ	6	60489301	60489600	0.158	0.489	0.350	0.720
UQ	6	60900301	60900600	0.744	1.056	1.066	1.080
UQ	6	61201801	61202100	1.521	0.935	0.531	0.644
UQ	6	61479601	61479900	0.828	1.244	0.165	1.253

UQ	6	61491601	61491900	1.063	0.493	0.481	0.684
UQ	6	61745101	61745400	0.558	0.281	0.424	0.142
UQ	6	62087401	62087700	0.773	1.148	0.502	0.466
UQ	6	62471101	62471400	0.665	0.983	1.061	1.070
UQ	6	64378201	64378500	1.039	0.279	0.477	0.248
UQ	6	64527301	64527600	0.558	0.238	0.181	0.223
UQ	6	64809001	64809300	0.187	0.562	0.671	0.466
UQ	6	65903101	65903400	0.584	0.187	0.239	0.213
UQ	6	66851401	66851700	0.481	0.912	0.691	0.715
UQ	6	67549201	67549500	0.374	0.161	0.609	0.137
UQ	6	67803601	67803900	0.934	0.468	0.468	0.502
UQ	6	67877401	67877700	0.665	0.142	0.267	0.466
UQ	6	68177101	68177400	0.745	0.163	0.111	0.299
UQ	6	68924701	68925000	0.477	0.328	0.136	0.274
UQ	6	69143401	69143700	0.319	0.444	0.214	0.218
UQ	6	69760201	69760500	1.173	0.792	0.687	0.690
UQ	6	70424701	70425000	1.010	0.560	0.337	0.452
UQ	6	72003901	72004200	0.771	0.376	0.054	0.157
UQ	6	72891601	72891900	0.477	0.071	0.292	0.243
UQ	6	73551001	73551300	0.773	0.933	0.399	0.608
UQ	6	73805701	73806000	3.933	2.045	2.365	2.454
UQ	6	76440901	76441200	0.477	0.656	0.613	0.304
UQ	6	76719901	76720200	1.891	1.453	1.518	1.323
UQ	6	78699601	78699900	0.639	0.303	0.317	0.213
UQ	6	79770301	79770600	0.477	0.210	0.346	0.223
UQ	6	84161401	84161700	0.345	0.513	0.613	0.248
UQ	6	86246701	86247000	0.881	0.262	0.346	0.248
UQ	6	86522101	86522400	0.531	0.210	0.239	0.299
UQ	6	87775201	87775500	0.134	0.539	0.473	0.583
UQ	6	88174501	88174800	1.037	0.798	0.769	0.299
UQ	6	90487201	90487500	0.586	0.184	0.136	0.025
UQ	6	91022101	91022400	0.505	0.656	0.292	0.304
UQ	6	91842301	91842600	0.558	0.257	0.272	0.228
UQ	6	92878801	92879100	2.693	0.865	1.472	1.297
UQ	6	93324901	93325200	2.039	0.895	0.987	0.853
UQ	6	95620501	95620800	0.531	0.258	0.140	0.193
UQ	6	95653501	95653800	0.637	0.232	0.350	0.441
UQ	6	95767201	95767500	0.584	0.184	0.165	0.106
UQ	6	96230701	96231000	0.477	0.232	0.107	0.279
UQ	6	96328801	96329100	0.665	0.217	0.185	0.106
UQ	6	96437401	96437700	1.516	0.800	0.568	0.821
UQ	6	96965401	96965700	0.824	0.354	0.399	0.340
UQ	6	97372801	97373100	0.953	2.289	2.782	2.727
UQ	6	97399801	97400100	0.323	0.931	0.872	0.745
UQ	6	97628401	97628700	0.532	0.283	0.210	0.223
UQ	6	97712701	97713000	0.134	0.513	0.292	0.472
UQ	6	97737901	97738200	0.161	0.560	0.638	0.527
UQ	6	97748401	97748700	0.347	0.929	0.774	0.907
UQ	6	97883701	97884000	0.850	1.053	0.613	0.385
UQ	6	98024701	98025000	0.266	0.862	0.724	0.786
UQ	6	98116201	98116500	3.062	0.972	1.612	1.520
UQ	6	98117401	98117700	1.113	0.144	0.156	0.472
UQ	6	98493001	98493300	0.795	0.285	0.330	0.304
UQ	6	98609701	98610000	2.870	2.311	1.757	2.504
UQ	6	98731501	98731800	0.560	0.189	0.103	0.213
UQ	6	98731801	98732100	0.534	0.260	0.078	0.137
UQ	6	98932801	98933100	0.321	0.704	0.609	0.826
UQ	6	99378001	99378300	0.211	0.421	0.502	0.411
UQ	6	99528901	99529200	0.292	1.026	0.662	1.080
UQ	6	100191601	100191900	1.908	0.935	0.704	0.421
UQ	6	100191901	100192200	1.352	0.633	0.461	0.441
UQ	6	101165401	101165700	0.426	0.212	0.214	0.614
UQ	6	101450101	101450400	0.345	0.118	0.078	0.695
UQ	6	102334801	102335100	0.799	0.395	0.497	0.411
UQ	6	102580801	102581100	0.160	0.330	0.691	0.304
UQ	6	102800401	102800700	1.489	3.072	3.266	3.277
UQ	6	102800701	102801000	0.452	1.268	0.938	1.019

UQ	6	102883801	102884100	0.742	0.255	0.189	0.228
UQ	6	102969001	102969300	0.560	0.092	0.234	0.162
UQ	6	103011601	103011900	0.613	0.305	0.185	0.259
UQ	6	103418401	103418700	1.652	1.148	0.945	1.136
UQ	6	103579801	103580100	1.621	1.212	1.164	1.166
UQ	6	103655401	103655700	2.578	1.656	1.268	1.840
UQ	6	103853101	103853400	0.744	1.002	0.350	0.441
UQ	6	104389501	104389800	3.725	2.109	2.278	2.277
UQ	6	104691601	104691900	0.321	0.766	0.765	0.771
UQ	6	104902201	104902500	1.049	2.386	2.122	2.561
UQ	6	104908201	104908500	1.041	0.584	0.580	0.452
UQ	6	105047401	105047700	0.319	0.517	0.054	0.031
UQ	6	105055801	105056100	0.266	0.395	0.107	0.025
UQ	6	105292501	105292800	0.134	0.515	0.424	0.304
UQ	6	105410701	105411000	0.423	0.165	0.082	0.056
UQ	6	105466201	105466500	0.529	0.096	0.000	0.142
UQ	6	105786601	105786900	0.507	0.213	0.136	0.167
UQ	6	108072301	108072600	0.239	0.629	0.506	0.634
UQ	6	108648901	108649200	0.373	0.493	0.555	0.248
UQ	6	109517701	109518000	4.654	4.025	3.159	3.924
UQ	6	109803601	109803900	0.610	0.212	0.185	0.243
UQ	6	110279101	110279400	1.810	1.377	0.860	1.430
UQ	6	111073201	111073500	0.720	0.305	0.296	0.472
UQ	6	111447001	111447300	0.477	0.257	0.210	0.243
UQ	6	111649201	111649500	1.086	0.566	0.560	0.502
UQ	6	111690901	111691200	3.396	4.907	6.048	5.481
UQ	6	111889201	111889500	4.018	2.778	2.927	3.108
UQ	6	112021501	112021800	0.477	0.140	0.111	0.117
UQ	6	112233601	112233900	5.161	7.419	7.636	7.454
UQ	6	112449901	112450200	0.134	0.607	0.593	0.639
UQ	6	112730101	112730400	1.700	2.946	2.127	2.408
UQ	6	112961401	112961700	2.342	1.193	1.066	1.212
UQ	6	113451301	113451600	2.505	0.862	1.460	1.760
UQ	6	113619601	113619900	1.650	2.122	2.292	2.490
UQ	6	113625301	113625600	1.707	0.841	1.152	0.806
UQ	6	113667601	113667900	1.284	2.508	2.341	2.911
UQ	6	113667901	113668200	0.720	1.245	1.324	1.293
UQ	6	113699101	113699400	1.063	0.191	0.584	0.832
UQ	6	113715001	113715300	0.694	0.210	0.403	0.187
UQ	6	113756701	113757000	3.776	2.587	2.176	2.278
UQ	6	113780701	113781000	1.010	0.607	0.486	0.639
UQ	6	113884801	113885100	0.558	0.210	0.156	0.213
UQ	6	114001201	114001500	2.370	1.051	1.790	1.151
UQ	6	114184801	114185100	1.858	1.073	1.494	1.359
UQ	6	114413701	114414000	1.442	0.429	0.613	0.553
UQ	6	114414001	114414300	1.808	0.947	0.765	0.907
UQ	6	114681001	114681300	6.377	4.270	5.241	9.725
UQ	6	115828201	115828500	1.041	1.474	1.761	0.517
UQ	6	115834501	115834800	0.981	0.376	0.506	0.268
UQ	6	116407501	116407800	1.784	1.474	1.061	0.923
UQ	6	116580601	116580900	2.207	1.332	2.020	2.515
UQ	6	117017101	117017400	0.690	0.470	0.399	0.223
UQ	7	745201	745500	4.170	3.088	2.855	2.494
UQ	7	1115701	1116000	0.958	0.609	0.502	1.237
UQ	7	1482901	1483200	0.795	0.262	0.058	0.061
UQ	7	1483201	1483500	0.881	0.229	0.000	0.086
UQ	7	1764601	1764900	1.412	0.421	0.410	0.446
UQ	7	2319001	2319300	0.637	0.466	0.453	0.411
UQ	7	2514001	2514300	0.476	0.023	0.185	0.056
UQ	7	3126601	3126900	0.958	0.489	0.312	0.304
UQ	7	3151201	3151500	1.274	0.397	0.296	0.320
UQ	7	3536701	3537000	0.158	0.375	0.531	0.446
UQ	7	4117201	4117500	3.188	1.742	2.093	1.977
UQ	7	4610401	4610700	2.076	1.221	1.168	1.613
UQ	7	4644601	4644900	0.505	0.772	0.424	0.639
UQ	7	4660801	4661100	1.916	0.936	1.037	1.157
UQ	7	4692601	4692900	0.718	0.331	0.399	0.274

UQ	7	5096401	5096700	1.520	0.798	0.814	0.958
UQ	7	5130901	5131200	2.423	1.822	1.893	1.982
UQ	7	5354701	5355000	0.881	0.189	0.107	0.315
UQ	7	5577901	5578200	0.694	1.732	1.016	1.222
UQ	7	5694001	5694300	1.063	0.674	0.539	0.608
UQ	7	5868601	5868900	0.984	1.148	0.374	0.720
UQ	7	6552001	6552300	4.360	3.405	3.150	3.509
UQ	7	6888901	6889200	0.850	0.191	0.165	0.207
UQ	7	7152601	7152900	0.477	0.303	0.082	0.274
UQ	7	7464001	7464300	0.532	0.839	0.350	0.720
UQ	7	7619401	7619700	0.268	1.757	1.258	0.938
UQ	7	7640701	7641000	0.776	0.279	0.267	0.360
UQ	7	7902001	7902300	1.544	1.845	1.148	1.181
UQ	7	7963201	7963500	0.531	0.238	0.185	0.248
UQ	7	8076901	8077200	0.665	0.423	0.321	0.446
UQ	7	8444101	8444400	0.423	0.914	0.954	0.806
UQ	7	8556901	8557200	1.066	0.440	0.506	0.715
UQ	7	8821801	8822100	0.958	0.352	0.497	0.436
UQ	7	8977201	8977500	1.466	1.777	0.604	2.236
UQ	7	9411301	9411600	0.160	0.421	0.633	0.553
UQ	7	9648301	9648600	6.407	8.127	7.667	7.022
UQ	7	9801301	9801600	0.665	0.399	0.239	0.309
UQ	7	10527001	10527300	2.851	1.909	1.304	1.531
UQ	7	10926601	10926900	1.144	0.772	0.876	0.654
UQ	7	10943701	10944000	0.345	0.727	0.716	0.614
UQ	7	11954701	11955000	0.108	0.584	0.370	0.304
UQ	7	12114001	12114300	1.520	2.367	2.287	2.449
UQ	7	12198901	12199200	0.666	0.418	0.263	0.360
UQ	7	12199201	12199500	0.505	0.350	0.210	0.274
UQ	7	12216901	12217200	0.400	0.539	0.239	0.553
UQ	7	12627901	12628200	1.142	0.886	0.399	0.639
UQ	7	12789601	12789900	2.194	0.936	1.066	0.821
UQ	7	12842701	12843000	0.532	0.444	0.399	0.218
UQ	7	13290001	13290300	0.450	0.281	0.185	0.193
UQ	7	13348201	13348500	0.505	0.142	0.111	0.254
UQ	7	13354201	13354500	2.820	2.482	2.492	2.322
UQ	7	13406401	13406700	0.079	0.420	0.185	0.274
UQ	7	13419901	13420200	1.886	0.983	1.045	0.873
UQ	7	13420201	13420500	1.728	1.077	1.152	0.842
UQ	7	13661401	13661700	0.586	0.397	0.424	0.411
UQ	7	14396701	14397000	0.265	0.446	0.424	0.497
UQ	7	14472301	14472600	0.213	0.727	0.419	0.446
UQ	7	14476801	14477100	0.876	1.877	1.930	1.917
UQ	7	14768401	14768700	0.532	0.161	0.263	0.218
UQ	7	14867401	14867700	0.850	0.607	0.667	0.522
UQ	7	15118201	15118500	1.728	1.315	1.280	0.812
UQ	7	15399601	15399900	0.373	0.631	0.218	0.137
UQ	7	15421201	15421500	1.328	0.000	0.049	0.056
UQ	7	15421501	15421800	4.552	0.719	0.709	0.466
UQ	7	15519301	15519600	0.560	0.163	0.370	0.142
UQ	7	15682501	15682800	3.864	2.236	2.107	1.643
UQ	7	15732901	15733200	0.587	0.955	0.925	0.994
UQ	7	16040101	16040400	2.823	1.940	1.308	1.131
UQ	7	16165501	16165800	0.239	0.513	0.531	0.725
UQ	7	16181701	16182000	2.133	1.611	1.465	1.552
UQ	7	16238101	16238400	0.929	0.513	0.526	0.639
UQ	7	16293301	16293600	3.165	2.478	2.341	2.444
UQ	7	16546201	16546500	0.187	0.350	0.477	0.416
UQ	7	16604701	16605000	2.707	3.557	3.397	3.483
UQ	7	16714801	16715100	1.125	0.240	0.176	0.279
UQ	7	16803901	16804200	0.448	0.071	0.054	0.081
UQ	7	16839001	16839300	1.376	0.369	0.234	0.487
UQ	7	17891701	17892000	0.106	0.444	0.399	0.340
UQ	7	18275701	18276000	0.929	0.536	0.321	0.533
UQ	7	18761701	18762000	0.611	0.210	0.054	0.254
UQ	7	18905701	18906000	3.860	1.995	1.799	2.060
UQ	7	19128901	19129200	2.286	1.525	1.752	1.516

UQ	7	19341301	19341600	0.398	0.140	0.160	0.614
UQ	7	19517701	19518000	1.250	0.773	0.847	0.608
UQ	7	19819801	19820100	1.174	0.657	0.473	0.421
UQ	7	20388901	20389200	0.610	0.352	0.370	0.391
UQ	7	20400301	20400600	1.037	1.403	0.765	0.740
UQ	7	20470501	20470800	0.634	0.142	0.103	0.173
UQ	7	20573101	20573400	0.640	0.114	0.214	0.355
UQ	7	20577001	20577300	1.010	0.935	0.691	1.136
UQ	7	20602201	20602500	2.452	3.506	3.939	3.499
UQ	7	20838301	20838600	0.450	0.161	0.029	0.081
UQ	7	20925301	20925600	0.292	0.654	0.399	0.441
UQ	7	21127201	21127500	5.004	4.390	4.093	4.061
UQ	7	21172501	21172800	0.878	1.264	1.708	1.242
UQ	7	21608101	21608400	0.081	0.210	0.588	0.248
UQ	7	21627901	21628200	0.507	0.258	0.136	0.218
UQ	7	21867301	21867600	2.552	1.496	2.000	1.953
UQ	7	21873301	21873600	1.223	0.564	0.642	0.538
UQ	7	21880501	21880800	1.674	0.935	1.197	1.222
UQ	7	21894301	21894600	5.527	2.626	3.618	3.340
UQ	7	23295001	23295300	0.690	0.375	0.239	0.365
UQ	7	23314501	23314800	0.558	0.307	0.267	0.193
UQ	7	23337601	23337900	1.041	0.423	0.325	0.441
UQ	7	23507101	23507400	1.324	0.474	0.334	0.482
UQ	7	23881801	23882100	0.932	0.564	0.461	0.248
UQ	7	23901001	23901300	0.613	0.350	0.185	0.223
UQ	7	23992801	23993100	1.728	1.259	1.329	1.354
UQ	7	24015601	24015900	0.557	0.328	0.346	0.355
UQ	7	25944001	25944300	0.136	0.373	0.428	0.608
UQ	7	26823001	26823300	0.636	0.281	0.424	0.167
UQ	7	27004801	27005100	0.689	0.285	0.267	0.299
UQ	7	27275401	27275700	1.489	0.568	2.550	0.025
UQ	7	27297301	27297600	0.929	0.560	0.662	0.614
UQ	7	27425101	27425400	0.771	0.631	0.535	0.355
UQ	7	27847201	27847500	1.273	0.957	0.662	0.968
UQ	7	28869301	28869600	0.611	0.352	0.403	0.416
UQ	7	29470501	29470800	1.786	2.617	2.374	2.596
UQ	7	29637301	29637600	0.318	0.773	0.613	0.614
UQ	7	29801701	29802000	0.318	0.772	0.658	0.583
UQ	7	30297601	30297900	0.744	0.328	0.127	0.254
UQ	7	30329401	30329700	0.213	0.420	0.346	0.416
UQ	7	30456301	30456600	0.265	0.468	0.481	0.695
UQ	7	30723601	30723900	0.507	0.281	0.267	0.360
UQ	7	31595701	31596000	0.319	0.910	1.012	0.751
UQ	7	32222701	32223000	0.639	0.307	0.346	0.309
UQ	7	33997201	33997500	1.994	1.021	1.209	1.283
UQ	7	34227901	34228200	1.778	1.212	0.838	0.796
UQ	7	34524301	34524600	0.213	0.491	0.473	0.416
UQ	7	34824001	34824300	0.534	0.234	0.218	0.832
UQ	7	35322601	35322900	1.463	0.732	0.379	0.907
UQ	7	35475001	35475300	3.193	1.569	1.794	2.220
UQ	7	35475301	35475600	0.692	0.258	0.181	0.137
UQ	7	36588901	36589200	0.611	0.234	0.239	0.025
UQ	7	36981901	36982200	0.586	0.159	0.074	0.223
UQ	7	38733601	38733900	0.558	0.232	0.107	0.061
UQ	7	40157101	40157400	0.900	0.311	0.272	0.193
UQ	7	40318501	40318800	0.421	0.865	0.876	0.974
UQ	7	40747201	40747500	0.713	0.163	0.107	0.147
UQ	7	41134801	41135100	1.544	1.332	1.772	1.024
UQ	7	41215201	41215500	0.505	0.305	0.370	0.279
UQ	7	41604601	41604900	1.515	0.702	0.840	0.842
UQ	7	41720401	41720700	0.371	0.631	0.765	0.771
UQ	7	42129601	42129900	0.797	0.493	0.457	0.502
UQ	7	42209101	42209400	0.613	0.843	1.353	0.421
UQ	7	43773901	43774200	1.755	1.360	0.807	1.359
UQ	7	44022001	44022300	0.639	0.257	0.374	0.223
UQ	7	44061001	44061300	1.413	0.772	0.595	0.710
UQ	7	44150101	44150400	0.610	0.094	0.181	0.182

UQ	7	44154601	44154900	0.163	0.772	0.526	0.502
UQ	7	44419801	44420100	2.126	1.188	1.193	1.298
UQ	7	44956801	44957100	0.903	0.586	0.366	0.608
UQ	7	45081301	45081600	0.027	0.584	0.210	0.248
UQ	7	45300301	45300600	1.484	0.680	0.466	0.274
UQ	7	45300601	45300900	0.745	0.303	0.432	0.304
UQ	7	45406801	45407100	1.707	0.802	1.028	0.720
UQ	7	45444001	45444300	0.555	0.094	0.074	0.162
UQ	7	45444301	45444600	0.534	0.212	0.210	0.218
UQ	7	45528001	45528300	0.503	0.257	0.156	0.081
UQ	7	45849601	45849900	0.187	0.446	0.510	0.416
UQ	7	46329601	46329900	0.660	1.856	1.736	1.841
UQ	7	46409701	46410000	2.813	1.970	1.864	1.166
UQ	7	46611901	46612200	0.879	1.525	1.358	1.348
UQ	7	46664401	46664700	2.342	1.734	1.670	1.703
UQ	7	47682301	47682600	0.584	0.215	0.132	0.025
UQ	7	47808001	47808300	0.982	0.539	0.551	0.329
UQ	7	47862901	47863200	0.240	0.094	0.502	0.025
UQ	7	48213601	48213900	1.357	0.817	0.457	1.029
UQ	7	48777601	48777900	0.665	0.326	0.267	0.228
UQ	7	49237201	49237500	1.413	0.470	0.765	0.837
UQ	7	49237501	49237800	0.958	0.399	0.468	0.583
UQ	7	49622101	49622400	1.087	0.423	0.432	0.776
UQ	7	49847701	49848000	0.481	0.049	0.185	0.218
UQ	7	49848001	49848300	0.532	0.120	0.243	0.187
UQ	7	49876501	49876800	0.558	0.165	0.395	0.279
UQ	7	49906201	49906500	1.331	0.401	0.724	0.877
UQ	7	50238601	50238900	0.476	0.096	0.185	0.106
UQ	7	50837701	50838000	0.637	0.212	0.214	0.238
UQ	7	51171601	51171900	0.560	1.120	0.765	0.913
UQ	7	53841301	53841600	0.797	0.352	0.457	0.994
UQ	7	54362701	54363000	2.365	1.478	1.850	1.557
UQ	7	54584401	54584700	2.105	1.118	1.547	1.658
UQ	7	54683701	54684000	4.498	2.508	1.914	1.723
UQ	7	54972601	54972900	1.676	1.032	0.490	0.735
UQ	7	55941301	55941600	0.989	0.491	0.370	0.441
UQ	7	56511001	56511300	0.611	0.466	0.243	0.304
UQ	7	58640701	58641000	2.186	1.105	1.293	1.192
UQ	7	58712101	58712400	0.957	0.187	0.560	0.472
UQ	7	58769401	58769700	3.644	2.896	3.090	2.941
UQ	7	59281201	59281500	0.665	0.493	0.502	0.223
UQ	7	59359801	59360100	1.644	1.098	1.081	0.857
UQ	7	59856001	59856300	0.850	0.378	0.132	0.228
UQ	7	59904901	59905200	0.694	0.257	0.321	0.304
UQ	7	60447001	60447300	0.931	0.607	0.267	0.086
UQ	7	60453301	60453600	0.823	0.538	0.580	0.522
UQ	7	60828901	60829200	0.053	0.423	0.296	0.279
UQ	7	61059301	61059600	1.249	0.796	0.453	0.558
UQ	7	61646701	61647000	2.133	1.446	1.135	1.318
UQ	7	61697101	61697400	0.507	0.094	0.136	0.223
UQ	7	62013601	62013900	0.266	1.051	0.564	0.466
UQ	7	63817801	63818100	1.116	0.822	0.555	0.335
UQ	7	64025401	64025700	0.958	0.257	0.461	0.385
UQ	7	64074601	64074900	0.132	0.491	0.341	0.385
UQ	7	64104001	64104300	2.573	1.708	1.344	1.536
UQ	7	64223101	64223400	2.792	2.246	1.719	2.165
UQ	7	64308301	64308600	2.037	0.215	0.334	0.177
UQ	7	64308601	64308900	1.199	0.168	0.223	0.051
UQ	7	64608001	64608300	0.529	0.094	0.189	0.112
UQ	7	64742101	64742400	5.659	4.456	3.103	3.386
UQ	7	65261401	65261700	1.442	0.626	0.374	0.365
UQ	7	65414701	65415000	0.477	0.328	0.082	0.218
UQ	7	65428801	65429100	0.902	0.421	0.419	0.391
UQ	7	65588101	65588400	0.821	0.144	0.325	0.243
UQ	7	65588401	65588700	0.718	0.069	0.198	0.051
UQ	7	65719801	65720100	0.373	0.234	0.535	0.715
UQ	7	66372901	66373200	2.480	1.480	1.280	1.648

UQ	7	66560101	66560400	1.436	0.820	0.823	1.044
UQ	7	67047301	67047600	0.505	0.165	0.189	0.137
UQ	7	67806901	67807200	0.507	0.096	0.136	0.126
UQ	7	67839901	67840200	0.132	0.466	0.515	0.801
UQ	7	67884601	67884900	1.674	0.212	0.189	0.375
UQ	7	68184001	68184300	0.292	0.678	0.588	0.588
UQ	7	68476801	68477100	0.479	0.747	0.745	1.105
UQ	7	69639001	69639300	0.879	1.285	1.251	1.212
UQ	7	70132201	70132500	0.584	0.208	0.160	0.254
UQ	7	70173901	70174200	0.694	0.212	0.107	0.472
UQ	7	70223101	70223400	0.610	0.238	0.210	0.218
UQ	7	70454701	70455000	0.424	0.656	0.234	0.248
UQ	7	70462201	70462500	0.532	0.184	0.181	0.248
UQ	7	73932001	73932300	0.477	0.185	0.029	0.112
UQ	7	74039701	74040000	0.558	0.232	0.189	0.391
UQ	7	74198701	74199000	0.402	0.815	0.852	0.801
UQ	7	74200201	74200500	0.826	0.468	0.415	0.411
UQ	7	74726701	74727000	0.426	0.609	0.107	0.254
UQ	7	74763001	74763300	0.824	0.326	0.176	0.081
UQ	7	75405901	75406200	0.292	1.079	0.667	0.806
UQ	7	76240801	76241100	0.853	1.144	0.481	0.553
UQ	7	77015401	77015700	0.850	0.234	0.317	0.335
UQ	7	77304001	77304300	2.047	1.283	1.349	1.257
UQ	7	77485501	77485800	1.412	0.536	1.090	1.024
UQ	7	77520601	77520900	0.823	0.448	0.395	0.466
UQ	7	78183901	78184200	2.950	4.077	0.568	2.028
UQ	7	78234601	78234900	0.610	0.399	0.267	0.051
UQ	7	78543301	78543600	0.666	0.354	0.185	0.223
UQ	7	78672001	78672300	0.345	0.539	0.774	0.634
UQ	7	79220701	79221000	1.092	0.657	0.691	0.710
UQ	7	79297201	79297500	2.337	1.590	1.554	1.542
UQ	7	79354201	79354500	1.513	1.824	0.502	1.273
UQ	7	79997401	79997700	0.534	0.232	0.181	0.198
UQ	7	80182501	80182800	1.965	1.272	1.094	1.080
UQ	7	80519401	80519700	3.965	3.324	2.550	3.052
UQ	7	80945701	80946000	0.531	0.232	0.156	0.193
UQ	7	81072001	81072300	0.503	0.328	0.321	0.025
UQ	7	81129301	81129600	1.565	0.961	0.876	0.603
UQ	7	81145801	81146100	0.984	0.420	0.510	0.533
UQ	7	81426301	81426600	1.226	1.991	0.444	0.821
UQ	7	81887401	81887700	1.410	0.691	0.366	0.335
UQ	7	81907801	81908100	0.912	0.165	0.132	0.112
UQ	7	82610401	82610700	2.768	2.219	2.135	1.734
UQ	7	82842601	82842900	0.929	0.356	0.312	0.502
UQ	7	83261101	83261400	3.588	3.111	3.056	2.835
UQ	7	83464501	83464800	1.300	0.420	0.769	0.679
UQ	7	83655301	83655600	2.841	2.126	1.786	1.704
UQ	7	83904301	83904600	0.481	0.161	0.000	0.076
UQ	7	84053401	84053700	1.860	1.633	1.226	1.379
UQ	7	84621601	84621900	0.639	0.257	0.374	0.223
UQ	7	84718201	84718500	0.374	0.607	0.609	0.776
UQ	7	84832801	84833100	0.824	0.511	0.189	0.391
UQ	7	84837301	84837600	6.258	4.280	9.403	4.089
UQ	7	84932101	84932400	0.423	0.165	0.054	0.081
UQ	7	85180501	85180800	0.398	0.515	0.185	0.304
UQ	7	85642201	85642500	1.437	1.774	1.757	2.545
UQ	7	85821601	85821900	0.292	0.444	0.136	0.193
UQ	7	86022001	86022300	0.640	0.165	0.214	0.193
UQ	7	86549101	86549400	0.560	0.140	0.346	0.193
UQ	7	86706601	86706900	0.108	0.420	0.267	0.497
UQ	7	86943301	86943600	0.692	0.281	0.419	0.167
UQ	7	87010801	87011100	3.665	4.304	4.438	2.646
UQ	7	87053401	87053700	1.620	0.961	0.925	1.064
UQ	7	87076801	87077100	0.666	0.399	0.296	0.299
UQ	7	87113401	87113700	0.507	0.208	0.156	0.254
UQ	7	87304501	87304800	0.718	0.210	0.477	0.416
UQ	7	87566701	87567000	0.534	0.092	0.165	0.137

UQ	7	88761601	88761900	1.828	0.461	1.086	0.639
UQ	7	90023401	90023700	1.944	0.566	1.016	0.324
UQ	7	90163801	90164100	1.378	2.105	0.510	0.765
UQ	7	90451201	90451500	0.613	0.163	0.292	0.329
UQ	7	90793501	90793800	0.802	0.258	0.230	0.309
UQ	7	91838101	91838400	0.666	0.281	0.350	0.284
UQ	7	92388901	92389200	0.481	0.187	0.082	0.167
UQ	7	93159901	93160200	0.670	0.096	0.054	0.126
UQ	7	93751801	93752100	0.584	0.421	0.346	0.335
UQ	7	94467601	94467900	0.532	0.839	0.346	0.274
UQ	7	95420101	95420400	0.213	0.843	0.502	0.355
UQ	7	95713201	95713500	0.319	0.560	0.189	0.527
UQ	7	95961901	95962200	0.155	0.867	0.798	0.634
UQ	7	96190801	96191100	3.747	2.238	2.066	2.373
UQ	7	96240001	96240300	1.142	0.751	0.695	0.628
UQ	7	96259201	96259500	1.625	0.361	0.395	0.086
UQ	7	96342001	96342300	0.642	0.279	1.057	0.193
UQ	7	96466801	96467100	0.826	0.255	0.239	0.122
UQ	7	96951601	96951900	0.718	0.515	0.555	0.497
UQ	7	96954301	96954600	0.237	0.584	0.029	0.441
UQ	7	97248001	97248300	0.584	0.281	0.267	0.385
UQ	7	97444501	97444800	2.078	1.219	1.188	0.680
UQ	7	97473301	97473600	3.539	2.154	2.118	1.905
UQ	7	97509601	97509900	1.384	0.678	0.823	0.731
UQ	7	97522501	97522800	1.278	1.802	1.460	1.775
UQ	7	97537501	97537800	3.029	1.861	0.794	1.811
UQ	7	97546201	97546500	0.079	0.468	0.370	0.274
UQ	7	97782301	97782600	4.098	3.487	3.077	2.748
UQ	7	97858801	97859100	0.053	0.538	0.555	0.497
UQ	7	97944001	97944300	6.340	3.976	4.148	4.716
UQ	7	97995301	97995600	0.799	0.496	1.592	1.491
UQ	7	98056501	98056800	0.586	0.373	0.292	0.411
UQ	7	98065501	98065800	0.821	1.476	0.444	0.187
UQ	7	98076001	98076300	1.221	0.474	0.571	0.446
UQ	7	98196001	98196300	1.381	0.936	0.671	0.821
UQ	7	98211301	98211600	0.184	0.931	0.667	0.573
UQ	7	98323801	98324100	0.979	0.397	0.560	0.355
UQ	7	98443201	98443500	2.155	1.658	0.972	1.035
UQ	7	98450401	98450700	7.872	6.752	6.222	7.089
UQ	7	98635501	98635800	1.436	0.910	1.901	0.751
UQ	7	98646001	98646300	0.239	0.538	0.054	0.137
UQ	7	98675701	98676000	3.539	1.555	2.241	2.484
UQ	7	98714101	98714400	9.239	3.782	6.105	7.246
UQ	7	98996101	98996400	0.692	1.026	0.346	0.466
UQ	7	99029101	99029400	1.011	0.656	0.526	0.669
UQ	7	99081001	99081300	1.815	2.412	3.007	2.687
UQ	7	99088201	99088500	0.398	0.303	0.156	0.497
UQ	7	99225001	99225300	1.328	0.770	0.428	0.846
UQ	7	99225301	99225600	6.006	3.605	3.335	3.931
UQ	7	99438001	99438300	0.479	1.002	0.999	0.907
UQ	7	99807301	99807600	2.841	4.072	4.158	4.260
UQ	7	99834301	99834600	4.949	3.484	3.396	3.479
UQ	8	11701	12000	0.184	0.564	0.580	0.913
UQ	8	334801	335100	0.507	0.213	0.243	0.081
UQ	8	384601	384900	0.582	0.281	0.189	0.173
UQ	8	585901	586200	0.663	0.303	0.366	0.248
UQ	8	1863001	1863300	1.091	0.494	0.346	0.279
UQ	8	2514601	2514900	0.637	0.843	0.103	0.472
UQ	8	2562001	2562300	0.958	0.139	0.145	0.416
UQ	8	2562301	2562600	1.066	0.255	0.198	0.527
UQ	8	3425101	3425400	0.584	0.354	0.029	0.304
UQ	8	4515301	4515600	2.447	1.328	1.576	1.587
UQ	8	4659901	4660200	3.646	2.624	2.020	2.738
UQ	8	5217601	5217900	0.213	0.446	0.584	0.801
UQ	8	5907601	5907900	0.106	0.468	0.243	0.329
UQ	8	6091201	6091500	1.657	0.891	0.653	0.745
UQ	8	6180301	6180600	0.532	0.330	0.156	0.223

UQ	8	6496801	6497100	2.480	0.865	1.126	1.099
UQ	8	6530401	6530700	0.053	0.420	0.189	0.274
UQ	8	6650401	6650700	0.771	0.493	0.370	0.228
UQ	8	6827101	6827400	0.265	0.633	0.502	0.472
UQ	8	7087501	7087800	0.108	0.421	0.321	0.558
UQ	8	7178701	7179000	0.160	0.702	0.448	0.446
UQ	8	7458601	7458900	0.532	0.210	0.218	0.092
UQ	8	8285101	8285400	0.240	0.069	0.609	0.112
UQ	8	8516101	8516400	0.505	0.257	0.424	0.690
UQ	8	9359101	9359400	1.807	1.382	0.876	1.293
UQ	8	9725401	9725700	0.532	0.375	0.346	0.198
UQ	8	10846201	10846500	0.531	0.212	0.317	0.137
UQ	8	11402101	11402400	0.450	0.118	0.107	0.132
UQ	8	11917801	11918100	0.610	0.397	0.399	0.335
UQ	8	12827701	12828000	0.239	0.468	0.370	0.497
UQ	8	13288801	13289100	0.373	0.232	0.082	0.522
UQ	8	13427101	13427400	0.455	0.843	0.954	0.933
UQ	8	13639501	13639800	1.654	2.317	2.374	2.753
UQ	8	14505001	14505300	0.531	0.185	0.205	0.218
UQ	8	14858701	14859000	1.063	0.354	0.667	0.486
UQ	8	14859001	14859300	0.981	0.303	0.551	0.603
UQ	8	15221701	15222000	0.055	0.446	0.399	0.329
UQ	8	15642001	15642300	0.873	0.045	0.350	0.137
UQ	8	15666001	15666300	0.848	0.283	0.506	0.416
UQ	8	15864001	15864300	0.239	0.863	0.448	0.411
UQ	8	16087501	16087800	0.826	0.444	0.502	0.294
UQ	8	17111701	17112000	1.195	0.586	0.740	0.812
UQ	8	18486601	18486900	1.036	0.701	0.588	0.335
UQ	8	18499801	18500100	0.557	0.118	0.370	0.056
UQ	8	18987001	18987300	0.507	0.185	0.185	0.162
UQ	8	19186501	19186800	1.274	0.845	0.740	0.756
UQ	8	19263901	19264200	0.637	0.232	0.214	0.294
UQ	8	19282501	19282800	0.453	0.893	1.012	0.801
UQ	8	20019901	20020200	0.536	0.049	0.054	0.193
UQ	8	21594901	21595200	0.505	0.236	0.317	0.167
UQ	8	21763801	21764100	1.752	4.255	4.709	3.261
UQ	8	24105001	24105300	0.292	0.444	0.103	0.142
UQ	8	24962701	24963000	3.247	2.191	2.443	2.662
UQ	8	25807501	25807800	0.450	0.096	0.103	0.086
UQ	8	25811101	25811400	1.254	0.493	0.379	0.543
UQ	8	25842001	25842300	1.010	1.169	0.691	0.862
UQ	8	25894501	25894800	0.853	1.101	0.531	0.502
UQ	8	25995601	25995900	0.881	1.452	0.564	0.553
UQ	8	26348401	26348700	1.357	0.957	0.539	0.893
UQ	8	26383501	26383800	0.692	0.466	0.555	0.329
UQ	8	27177601	27177900	0.160	0.489	0.444	0.527
UQ	8	27272101	27272400	0.000	0.257	0.424	0.466
UQ	8	27272401	27272700	0.026	0.279	0.531	0.477
UQ	8	27335401	27335700	0.800	0.375	0.288	0.274
UQ	8	27335701	27336000	0.982	0.468	0.341	0.411
UQ	8	27646201	27646500	0.531	0.258	0.259	0.112
UQ	8	27648001	27648300	0.292	0.116	0.082	0.466
UQ	8	27755701	27756000	2.065	0.354	0.453	0.841
UQ	8	28028401	28028700	2.184	1.566	1.518	2.510
UQ	8	28703701	28704000	0.826	0.654	0.555	0.441
UQ	8	28897801	28898100	0.747	0.262	0.078	0.117
UQ	8	30069001	30069300	0.132	0.444	0.317	0.056
UQ	8	30294001	30294300	0.720	0.258	0.330	0.335
UQ	8	30330301	30330600	0.476	0.114	0.029	0.137
UQ	8	31193101	31193400	0.610	0.258	0.317	0.299
UQ	8	31242301	31242600	0.773	0.491	0.535	0.492
UQ	8	31526101	31526400	0.373	0.725	0.292	0.223
UQ	8	31617601	31617900	0.558	0.303	0.247	0.137
UQ	8	31747801	31748100	0.532	0.167	0.111	0.162
UQ	8	31763101	31763400	0.160	0.515	0.807	0.522
UQ	8	31826401	31826700	0.531	0.303	0.185	0.279
UQ	8	31893001	31893300	2.765	1.828	0.836	1.420

UQ	8	31893301	31893600	0.879	0.493	0.444	0.553
UQ	8	31914901	31915200	0.532	0.908	0.140	0.999
UQ	8	31967701	31968000	6.536	4.495	4.659	4.694
UQ	8	33044401	33044700	1.091	0.796	0.802	0.771
UQ	8	33114601	33114900	0.265	1.071	0.905	0.679
UQ	8	35723701	35724000	0.000	0.541	0.682	0.360
UQ	8	35724001	35724300	0.052	0.517	0.613	0.482
UQ	8	36887401	36887700	0.423	0.096	0.082	0.112
UQ	8	37326901	37327200	0.716	0.279	0.283	0.243
UQ	8	37543501	37543800	0.134	0.489	0.584	0.547
UQ	8	37728901	37729200	0.266	0.562	0.103	0.527
UQ	8	38058601	38058900	0.265	0.538	0.453	0.553
UQ	8	38302501	38302800	0.211	0.444	0.448	0.385
UQ	8	39500101	39500400	0.587	0.210	0.239	0.112
UQ	8	39640801	39641100	1.595	1.124	0.892	0.923
UQ	8	39910501	39910800	0.797	0.517	0.457	0.274
UQ	8	40136701	40137000	0.610	0.375	0.292	0.360
UQ	8	41127601	41127900	1.034	0.635	0.613	0.628
UQ	8	41431501	41431800	0.557	0.114	0.210	0.117
UQ	8	46548601	46548900	0.744	1.217	1.378	1.379
UQ	8	46667101	46667400	0.455	1.794	2.163	1.866
UQ	8	46891501	46891800	0.531	0.213	0.132	0.162
UQ	8	47163001	47163300	0.745	0.401	0.317	0.360
UQ	8	47303101	47303400	0.426	0.704	0.214	0.248
UQ	8	47458201	47458500	0.771	0.305	0.283	0.335
UQ	8	47586601	47586900	0.132	0.654	0.473	0.380
UQ	8	47733901	47734200	0.797	0.562	0.321	0.639
UQ	8	47934301	47934600	0.557	1.306	1.099	0.994
UQ	8	48024601	48024900	0.665	0.307	0.263	0.218
UQ	8	48086101	48086400	4.627	4.418	4.175	3.808
UQ	8	49118401	49118700	2.263	1.637	1.431	1.755
UQ	8	49207801	49208100	2.473	1.706	1.904	1.064
UQ	8	49600801	49601100	0.792	0.094	0.000	0.137
UQ	8	49634101	49634400	0.876	1.236	1.436	1.633
UQ	8	50086801	50087100	0.694	0.116	0.189	0.142
UQ	8	50261701	50262000	0.131	0.727	0.617	0.583
UQ	8	51299101	51299400	0.505	0.794	0.881	0.695
UQ	8	51479401	51479700	0.134	0.283	0.370	0.608
UQ	8	51524401	51524700	0.745	0.491	0.185	0.421
UQ	8	51810001	51810300	2.896	4.040	4.343	3.951
UQ	8	51833401	51833700	0.507	0.112	0.160	0.167
UQ	8	52890901	52891200	0.558	0.397	0.214	0.355
UQ	8	53280901	53281200	0.636	1.143	0.263	0.385
UQ	8	54858901	54859200	0.156	0.560	0.453	0.466
UQ	8	56269201	56269500	0.799	0.442	0.535	0.608
UQ	8	57107101	57107400	0.636	1.216	0.856	1.465
UQ	8	57317401	57317700	2.337	1.708	1.016	1.856
UQ	8	58111501	58111800	0.905	0.047	0.103	0.031
UQ	8	58145101	58145400	1.031	2.103	1.736	1.922
UQ	8	58656601	58656900	0.319	0.444	0.428	0.416
UQ	8	60403801	60404100	0.505	0.328	0.160	0.355
UQ	8	60773401	60773700	0.186	0.465	0.424	0.446
UQ	8	61227001	61227300	0.771	0.167	0.247	0.268
UQ	8	61545601	61545900	2.131	2.735	1.313	1.633
UQ	8	61853101	61853400	3.060	2.032	1.768	2.063
UQ	8	62200201	62200500	0.213	0.328	0.107	0.522
UQ	8	64428901	64429200	0.477	0.232	0.239	0.187
UQ	8	64666501	64666800	2.504	1.871	2.004	1.764
UQ	8	66088801	66089100	0.477	0.096	0.132	0.167
UQ	8	66479701	66480000	0.534	0.184	0.103	0.218
UQ	8	66567901	66568200	0.720	0.302	0.341	0.324
UQ	8	67245301	67245600	0.902	0.539	0.584	0.558
UQ	8	67552501	67552800	0.692	0.286	0.292	0.218
UQ	8	67931101	67931400	1.307	0.770	0.707	0.745
UQ	8	68113201	68113500	0.081	0.611	0.481	0.385
UQ	8	68213701	68214000	0.958	0.564	0.379	0.396
UQ	8	68775601	68775900	0.105	0.609	0.263	0.391

UQ	8	68786101	68786400	0.690	1.453	1.016	1.192
UQ	8	69223501	69223800	0.428	1.259	0.831	0.846
UQ	8	69304501	69304800	0.503	0.326	0.185	0.112
UQ	8	69343501	69343800	0.823	0.560	0.481	0.527
UQ	8	69768901	69769200	0.106	0.350	0.370	0.553
UQ	8	69934201	69934500	1.863	1.307	0.992	1.181
UQ	8	70103101	70103400	0.505	0.397	0.292	0.162
UQ	8	70890001	70890300	0.477	0.187	0.107	0.299
UQ	8	71744401	71744700	0.584	0.283	0.370	0.385
UQ	8	72127201	72127500	0.826	0.448	0.419	0.466
UQ	8	72546001	72546300	2.152	1.244	1.087	1.064
UQ	8	72641401	72641700	1.436	0.957	0.629	0.213
UQ	8	73988101	73988400	0.374	0.633	1.003	0.634
UQ	8	73993501	73993800	0.560	0.116	0.132	0.223
UQ	8	74177401	74177700	0.213	0.772	0.317	0.081
UQ	8	74385901	74386200	2.980	2.133	2.007	2.256
UQ	8	74511901	74512200	0.557	0.234	0.214	0.335
UQ	8	74697901	74698200	0.982	1.195	0.502	0.745
UQ	8	74754901	74755200	0.881	0.165	0.181	0.497
UQ	8	74801401	74801700	0.345	0.656	0.210	0.137
UQ	8	75599101	75599400	0.081	0.517	0.453	0.497
UQ	8	75721801	75722100	0.476	1.450	1.037	1.105
UQ	8	75900301	75900600	1.174	0.635	0.687	0.533
UQ	8	77691901	77692200	0.374	0.794	0.535	0.137
UQ	8	78386401	78386700	0.618	2.788	2.831	2.779
UQ	8	78741301	78741600	0.636	0.185	0.239	0.309
UQ	8	78835501	78835800	0.105	0.472	0.526	0.466
UQ	8	79083001	79083300	1.223	0.626	0.531	0.745
UQ	8	79227301	79227600	0.720	0.425	0.054	0.441
UQ	8	79329001	79329300	0.424	0.562	0.740	0.968
UQ	8	79414501	79414800	0.477	0.305	0.078	0.137
UQ	8	79807501	79807800	0.000	0.631	0.473	0.513
UQ	8	80105401	80105700	4.228	2.575	2.649	2.836
UQ	8	80105701	80106000	2.150	1.259	0.959	1.247
UQ	8	80145001	80145300	0.666	0.352	0.337	0.248
UQ	8	80541001	80541300	0.824	0.560	0.506	0.603
UQ	8	80563501	80563800	0.447	1.609	1.697	1.521
UQ	8	80672101	80672400	1.944	3.017	2.863	3.073
UQ	8	80715901	80716200	0.932	0.633	0.448	0.547
UQ	8	80893501	80893800	3.191	2.483	3.870	3.798
UQ	8	80972401	80972700	1.491	1.804	1.037	0.751
UQ	8	81093601	81093900	5.582	4.499	4.282	4.639
UQ	8	81532801	81533100	0.345	0.234	0.535	0.527
UQ	8	81687901	81688200	1.837	1.242	1.320	2.286
UQ	8	81754501	81754800	1.939	2.568	2.699	2.626
UQ	8	81817801	81818100	0.560	0.303	0.234	0.279
UQ	8	81834601	81834900	3.773	1.755	2.349	2.039
UQ	8	81834901	81835200	0.715	0.326	0.341	0.329
UQ	8	81885301	81885600	1.916	1.082	1.012	1.140
UQ	8	82072501	82072800	0.903	0.519	0.341	0.522
UQ	8	82364401	82364700	0.924	0.163	0.205	0.178
UQ	8	82567801	82568100	1.520	0.701	0.132	0.350
UQ	8	82747201	82747500	3.452	2.472	2.526	1.977
UQ	8	82776901	82777200	1.331	2.030	2.189	2.256
UQ	8	82831501	82831800	0.477	0.305	0.642	0.832
UQ	8	82995301	82995600	0.637	0.352	0.428	0.193
UQ	8	83018401	83018700	1.729	2.437	2.485	3.138
UQ	8	83109601	83109900	1.228	2.102	2.533	2.343
UQ	8	83464501	83464800	0.316	0.609	0.691	0.603
UQ	8	83876701	83877000	0.318	0.633	0.560	0.497
UQ	8	84110401	84110700	1.250	1.098	0.633	0.578
UQ	8	84131401	84131700	0.663	0.515	0.424	0.304
UQ	8	84437101	84437400	0.161	0.656	0.613	0.821
UQ	8	84470101	84470400	1.961	0.983	1.181	0.801
UQ	8	84706201	84706500	3.141	2.596	1.037	1.967
UQ	8	84772801	84773100	1.060	0.234	0.272	0.254
UQ	8	84939001	84939300	2.157	1.448	1.387	1.263

UQ	8	84999301	84999600	0.187	0.397	0.267	0.497
UQ	8	85119601	85119900	4.127	3.051	3.036	2.901
UQ	8	85411801	85412100	0.744	0.238	0.366	0.299
UQ	8	85726801	85727100	1.649	1.244	1.201	1.029
UQ	8	85741501	85741800	1.487	0.680	0.510	0.628
UQ	8	85871101	85871400	0.426	0.773	0.711	0.801
UQ	8	85889401	85889700	0.745	0.356	0.321	0.365
UQ	8	85946701	85947000	3.641	2.124	2.280	1.668
UQ	8	86449801	86450100	0.347	0.513	0.107	0.472
UQ	8	86590801	86591100	1.492	0.521	0.633	0.801
UQ	8	86723701	86724000	2.224	1.075	0.386	1.314
UQ	8	86874001	86874300	1.994	1.474	1.913	2.266
UQ	8	87344701	87345000	0.692	0.420	0.312	0.218
UQ	8	87409201	87409500	7.230	6.001	5.368	6.079
UQ	8	87487501	87487800	1.829	1.219	0.716	0.558
UQ	8	87708601	87708900	1.063	0.699	0.633	0.436
UQ	8	87840901	87841200	0.584	0.212	0.049	0.385
UQ	8	87841501	87841800	2.928	1.568	1.616	1.804
UQ	8	87933301	87933600	0.665	0.375	0.189	0.472
UQ	8	88048801	88049100	2.134	2.879	3.114	4.599
UQ	8	88366801	88367100	6.581	4.111	4.873	5.157
UQ	8	88391701	88392000	4.160	1.675	1.708	1.511
UQ	8	88576501	88576800	3.989	2.721	2.983	3.219
UQ	8	88654201	88654500	4.120	3.551	3.237	3.154
UQ	8	88697701	88698000	2.899	2.476	2.164	2.124
UQ	8	88826701	88827000	1.115	1.714	1.839	1.917
UQ	8	88842901	88843200	0.507	0.303	0.263	0.162
UQ	8	89034001	89034300	4.623	3.716	3.982	4.056
UQ	8	89034601	89034900	0.531	0.701	0.292	0.243
UQ	8	89043301	89043600	1.702	0.865	1.222	1.546
UQ	8	89129101	89129400	1.250	1.146	0.769	0.943
UQ	8	89157001	89157300	0.292	1.006	1.806	0.472
UQ	8	89392801	89393100	2.847	2.405	1.962	1.973
UQ	8	89490601	89490900	1.568	2.147	0.814	2.191
UQ	8	89609101	89609400	0.505	1.330	0.745	0.893
UQ	8	89832901	89833200	0.960	0.631	0.613	0.517
UQ	8	89970001	89970300	2.476	1.774	1.888	1.586
UQ	8	90362701	90363000	0.797	1.493	1.123	1.278
UQ	8	90547801	90548100	0.876	1.261	0.716	0.553
UQ	9	271801	272100	6.511	3.450	2.392	4.454
UQ	9	607501	607800	1.007	0.448	0.444	0.421
UQ	9	860701	861000	2.736	2.381	1.893	1.993
UQ	9	989101	989400	1.163	0.449	0.321	0.213
UQ	9	1614901	1615200	0.450	0.232	0.239	0.193
UQ	9	2481001	2481300	1.915	1.240	1.291	1.197
UQ	9	2485501	2485800	0.508	0.071	0.082	0.025
UQ	9	2485801	2486100	0.640	0.140	0.107	0.081
UQ	9	2641201	2641500	0.108	0.796	0.419	0.477
UQ	9	3306901	3307200	0.505	0.163	0.214	0.106
UQ	9	3768301	3768600	0.692	0.328	0.531	0.385
UQ	9	3998701	3999000	1.518	0.408	0.390	0.157
UQ	9	4190101	4190400	0.615	0.139	0.136	0.076
UQ	9	5995501	5995800	1.244	0.399	0.185	0.254
UQ	9	5995801	5996100	1.774	0.918	0.361	0.315
UQ	9	6285301	6285600	0.826	0.279	0.390	0.304
UQ	9	6388501	6388800	0.742	0.283	0.321	0.268
UQ	9	7396201	7396500	0.747	0.421	0.473	0.472
UQ	9	7446901	7447200	0.347	0.094	0.716	0.081
UQ	9	7779601	7779900	0.611	0.281	0.078	0.126
UQ	9	7874401	7874700	0.694	0.189	0.259	0.248
UQ	9	8045401	8045700	0.902	0.442	0.399	0.213
UQ	9	9014101	9014400	1.326	0.723	0.428	0.791
UQ	9	11668201	11668500	0.637	0.305	0.214	0.279
UQ	9	12692101	12692400	0.689	0.279	0.399	0.243
UQ	9	12789001	12789300	0.611	0.234	0.502	0.943
UQ	9	13195201	13195500	0.910	1.425	2.200	2.246
UQ	9	14225701	14226000	1.066	1.287	0.609	0.857

UQ	9	14529601	14529900	0.505	0.165	0.132	0.178
UQ	9	14559301	14559600	1.011	0.000	0.049	0.051
UQ	9	14571601	14571900	0.266	0.045	0.798	0.573
UQ	9	14662501	14662800	1.723	1.023	0.789	0.751
UQ	9	14675701	14676000	0.665	0.328	0.341	0.365
UQ	9	14811301	14811600	2.528	1.189	0.740	1.044
UQ	9	15072901	15073200	0.507	0.303	0.346	0.279
UQ	9	15162601	15162900	1.039	0.794	0.560	0.745
UQ	9	15414301	15414600	0.984	0.584	0.823	1.024
UQ	9	15426001	15426300	0.319	0.538	1.411	0.061
UQ	9	15608101	15608400	4.341	3.031	3.295	2.768
UQ	9	15850801	15851100	0.853	1.358	1.436	1.222
UQ	9	16097701	16098000	2.954	1.751	2.076	2.059
UQ	9	16127701	16128000	12.816	6.432	10.041	6.257
UQ	9	16204801	16205100	0.689	1.611	1.480	1.770
UQ	9	16410001	16410300	2.394	1.609	1.378	1.607
UQ	9	16501201	16501500	0.134	0.421	0.370	0.573
UQ	9	16883101	16883400	1.807	0.510	1.123	1.293
UQ	9	17054401	17054700	1.600	0.863	0.774	1.156
UQ	9	17095201	17095500	3.405	2.257	2.448	2.524
UQ	9	17120701	17121000	1.776	1.058	1.119	0.968
UQ	9	17310001	17310300	0.429	0.024	0.078	0.000
UQ	9	17442901	17443200	0.586	0.397	0.399	0.360
UQ	9	18078901	18079200	0.532	0.234	0.210	0.198
UQ	9	18761701	18762000	0.508	0.023	0.074	0.051
UQ	9	19364701	19365000	1.173	0.725	0.646	0.740
UQ	9	19543801	19544100	0.769	0.494	0.214	0.329
UQ	9	19749601	19749900	0.797	1.008	1.703	1.136
UQ	9	19806901	19807200	1.041	0.609	0.646	0.360
UQ	9	20013001	20013300	0.932	1.077	0.691	0.497
UQ	9	20099401	20099700	2.176	1.026	1.395	1.197
UQ	9	20511001	20511300	0.637	0.326	0.165	0.355
UQ	9	20723101	20723400	0.639	0.232	0.288	0.466
UQ	9	20958001	20958300	0.749	0.397	0.321	0.329
UQ	9	21048001	21048300	0.476	0.140	0.136	0.112
UQ	9	21086101	21086400	0.716	0.423	0.136	0.173
UQ	9	21390301	21390600	2.023	1.144	1.320	1.111
UQ	9	21623701	21624000	2.624	1.594	1.157	1.678
UQ	9	21661801	21662100	1.837	1.244	0.876	1.136
UQ	9	21770701	21771000	2.607	3.109	3.946	3.433
UQ	9	21964501	21964800	4.257	3.883	3.143	3.366
UQ	9	22216201	22216500	0.824	0.560	0.473	0.999
UQ	9	22633201	22633500	0.850	0.609	0.584	0.436
UQ	9	22894201	22894500	0.611	0.538	0.374	0.497
UQ	9	22922701	22923000	1.274	0.820	0.749	0.684
UQ	9	23067901	23068200	0.557	0.305	0.292	0.329
UQ	9	23211901	23212200	0.239	0.468	0.531	0.391
UQ	9	23331601	23331900	0.984	0.442	0.506	0.832
UQ	9	23346001	23346300	0.531	0.330	0.214	0.329
UQ	9	23392801	23393100	0.531	0.725	0.687	0.329
UQ	9	23393101	23393400	0.481	0.069	0.078	0.101
UQ	9	23490301	23490600	2.797	3.392	1.843	1.871
UQ	9	23603701	23604000	0.532	0.232	0.049	0.081
UQ	9	23604001	23604300	0.505	0.230	0.000	0.031
UQ	9	23774401	23774700	0.534	0.236	0.078	0.081
UQ	9	24113401	24113700	1.570	0.959	1.115	1.024
UQ	9	24116701	24117000	0.532	0.092	0.136	0.056
UQ	9	25129501	25129800	0.239	0.513	0.642	0.608
UQ	9	25293001	25293300	0.558	0.279	0.210	0.117
UQ	9	25403101	25403400	0.536	0.114	0.136	0.167
UQ	9	25452001	25452300	4.867	3.604	2.849	2.960
UQ	9	25707901	25708200	0.905	0.489	0.424	0.628
UQ	9	25718101	25718400	0.479	1.049	1.050	1.044
UQ	9	26307301	26307600	0.665	0.375	0.350	0.391
UQ	9	26614501	26614800	0.720	0.397	0.341	0.441
UQ	9	26877901	26878200	0.026	0.395	0.210	0.193
UQ	9	27530401	27530700	1.039	0.747	0.428	0.756

UQ	9	27869701	27870000	0.745	0.656	0.453	0.472
UQ	9	28428001	28428300	0.742	0.212	0.136	0.396
UQ	9	28842001	28842300	0.878	0.562	1.230	0.603
UQ	9	29687101	29687400	1.278	1.026	0.687	1.044
UQ	9	29706601	29706900	0.213	0.468	0.132	0.304
UQ	9	29874901	29875200	0.213	0.583	0.584	0.335
UQ	9	31812901	31813200	0.692	0.068	0.225	0.086
UQ	9	31813201	31813500	0.505	0.161	0.210	0.051
UQ	9	31948201	31948500	1.649	0.919	0.341	0.513
UQ	9	32042401	32042700	0.265	0.116	0.448	0.056
UQ	9	32724601	32724900	10.279	8.485	7.667	9.081
UQ	9	32992801	32993100	2.079	1.448	1.378	1.044
UQ	9	33664801	33665100	1.113	0.656	0.593	0.533
UQ	9	33682501	33682800	2.575	1.877	1.465	1.876
UQ	9	33695401	33695700	1.405	0.654	0.765	0.644
UQ	9	33915001	33915300	0.584	0.212	0.078	0.086
UQ	9	34228501	34228800	0.400	1.191	0.745	0.720
UQ	9	35591701	35592000	1.575	0.933	0.876	0.816
UQ	9	36833401	36833700	1.542	1.332	0.789	1.050
UQ	9	37239601	37239900	0.986	0.303	0.370	0.324
UQ	9	37410601	37410900	0.665	0.421	0.288	0.411
UQ	9	37721101	37721400	0.771	0.421	0.473	0.466
UQ	9	38652601	38652900	4.120	3.031	2.858	3.311
UQ	9	38862901	38863200	0.907	0.510	0.346	0.497
UQ	9	39250801	39251100	0.529	0.212	0.267	0.223
UQ	9	39661201	39661500	1.594	1.334	0.798	0.907
UQ	9	39907501	39907800	0.637	1.633	1.016	1.035
UQ	9	40100701	40101000	0.237	0.682	0.584	0.695
UQ	9	40692601	40692900	1.204	2.130	2.258	2.271
UQ	9	40699501	40699800	0.132	0.491	0.486	0.502
UQ	9	40848901	40849200	0.852	0.213	0.506	0.396
UQ	9	40849201	40849500	2.525	1.176	1.710	1.541
UQ	9	40970701	40971000	3.960	4.718	3.253	5.152
UQ	9	41076901	41077200	0.663	0.118	0.267	0.092
UQ	9	41448301	41448600	0.106	0.562	0.481	0.385
UQ	9	41920201	41920500	2.339	1.826	1.498	1.491
UQ	9	42005101	42005400	0.666	0.257	0.321	0.182
UQ	9	42798301	42798600	0.636	0.277	0.263	0.243
UQ	9	42798601	42798900	0.665	0.277	0.317	0.340
UQ	9	43430401	43430700	0.373	0.538	0.662	0.502
UQ	9	43744801	43745100	0.558	0.213	0.239	0.213
UQ	9	43747801	43748100	1.358	0.910	0.814	0.715
UQ	9	44732101	44732400	1.116	0.496	0.711	0.502
UQ	9	45378601	45378900	0.503	1.236	1.086	0.983
UQ	9	45702001	45702300	1.199	0.309	0.691	0.628
UQ	9	46533001	46533300	0.616	0.120	0.025	0.132
UQ	9	46533301	46533600	0.610	0.096	0.058	0.051
UQ	9	46729501	46729800	0.557	0.092	0.189	0.056
UQ	9	46788301	46788600	0.616	1.452	1.366	1.582
UQ	9	47272201	47272500	0.237	0.468	0.667	0.416
UQ	9	47500201	47500500	0.132	0.539	0.448	0.492
UQ	9	47505901	47506200	0.558	0.118	0.189	0.056
UQ	9	47724001	47724300	0.240	0.888	0.617	0.608
UQ	9	48177001	48177300	0.187	0.397	0.555	0.421
UQ	9	48622201	48622500	0.745	0.448	0.370	0.436
UQ	9	48873601	48873900	0.265	0.513	0.560	0.137
UQ	9	49280701	49281000	0.637	0.210	0.370	0.309
UQ	9	49299601	49299900	0.503	0.165	0.049	0.137
UQ	9	49299901	49300200	1.466	0.755	0.526	0.370
UQ	9	49378801	49379100	0.158	0.541	0.638	0.659
UQ	9	49413901	49414200	3.426	2.008	2.020	2.561
UQ	9	49434601	49434900	0.108	0.538	0.350	0.411
UQ	9	49504201	49504500	0.266	0.140	0.428	0.558
UQ	9	49604401	49604700	0.557	0.305	0.107	0.243
UQ	9	49651501	49651800	0.052	0.420	0.872	0.309
UQ	9	50373001	50373300	1.305	0.981	0.856	1.019
UQ	9	50404201	50404500	0.665	0.794	0.267	0.522

UQ	9	50475601	50475900	0.610	0.305	0.317	0.329
UQ	9	52848301	52848600	0.665	0.444	0.292	0.391
UQ	9	53264101	53264400	0.106	0.517	0.580	0.441
UQ	9	54114901	54115200	0.531	0.328	0.243	0.117
UQ	9	54168001	54168300	0.503	0.654	0.132	0.360
UQ	9	54491101	54491400	0.695	0.257	0.239	0.223
UQ	9	54681301	54681600	1.486	0.936	0.987	0.771
UQ	9	55707001	55707300	0.984	0.682	0.481	0.608
UQ	9	55922701	55923000	0.962	0.470	0.457	0.380
UQ	9	56005501	56005800	0.907	0.539	0.526	0.497
UQ	9	56716801	56717100	0.398	0.491	0.506	0.527
UQ	9	56719501	56719800	1.092	0.307	0.098	0.193
UQ	9	56735401	56735700	1.086	0.257	0.374	0.553
UQ	9	56735701	56736000	0.955	0.210	0.531	0.486
UQ	9	57049201	57049500	0.505	0.631	0.263	0.218
UQ	9	57163201	57163500	0.982	1.240	1.699	1.942
UQ	9	57629701	57630000	0.529	0.912	1.451	0.893
UQ	9	57685201	57685500	0.476	0.023	0.058	0.061
UQ	9	57685501	57685800	0.582	0.023	0.000	0.056
UQ	9	57709801	57710100	0.608	1.867	2.271	1.586
UQ	9	57710101	57710400	0.505	1.706	1.478	1.440
UQ	9	57722401	57722700	1.513	0.678	0.662	0.384
UQ	9	58301701	58302000	0.321	0.163	0.477	0.056
UQ	9	58593901	58594200	0.160	0.281	0.502	0.329
UQ	9	58644001	58644300	0.477	0.281	0.243	0.198
UQ	9	58747201	58747500	0.189	0.442	0.662	0.441
UQ	9	59112301	59112600	0.479	0.609	0.292	1.136
UQ	9	59179501	59179800	0.237	0.581	0.638	0.806
UQ	9	59213701	59214000	0.744	0.307	0.341	0.472
UQ	9	59910601	59910900	1.250	0.725	0.765	0.821
UQ	9	60227101	60227400	0.369	0.699	0.716	0.690
UQ	9	60927301	60927600	1.300	0.676	0.531	0.558
UQ	9	61354501	61354800	0.266	0.538	0.346	0.304
UQ	9	62016001	62016300	0.665	0.397	0.506	0.472
UQ	9	62699401	62699700	0.639	0.513	0.424	0.218
UQ	9	63537001	63537300	0.690	0.348	0.346	0.309
UQ	9	64287901	64288200	0.774	0.442	0.424	0.365
UQ	9	64419301	64419600	0.321	1.053	0.774	0.710
UQ	9	66249601	66249900	1.066	0.635	0.716	0.690
UQ	9	66630001	66630300	0.503	0.212	0.136	0.248
UQ	9	67063501	67063800	0.586	1.455	1.197	1.075
UQ	9	67211701	67212000	0.481	0.208	0.025	0.081
UQ	9	68139301	68139600	0.695	1.262	1.391	1.369
UQ	9	69584101	69584400	0.690	1.004	1.139	1.359
UQ	9	69717001	69717300	0.903	0.513	0.341	0.690
UQ	9	69848101	69848400	0.531	0.305	0.292	0.385
UQ	9	69939601	69939900	0.505	0.303	0.399	0.304
UQ	9	70947301	70947600	0.345	0.607	0.424	0.223
UQ	9	72645601	72645900	0.423	0.023	0.103	0.086
UQ	9	73047301	73047600	1.013	0.444	0.444	0.228
UQ	9	73173601	73173900	0.637	0.373	0.160	0.497
UQ	9	73382701	73383000	0.582	0.191	0.189	0.086
UQ	9	74094901	74095200	0.292	0.515	0.185	0.472
UQ	9	74134201	74134500	0.160	0.305	0.317	0.466
UQ	9	74314501	74314800	0.505	0.236	0.218	0.223
UQ	9	74703001	74703300	1.171	0.800	0.424	0.644
UQ	9	74775001	74775300	2.255	1.354	1.144	0.725
UQ	9	74931301	74931600	0.079	0.446	0.185	0.193
UQ	9	75104401	75104700	1.592	1.075	0.974	0.938
UQ	9	75922801	75923100	0.610	0.142	0.267	0.187
UQ	9	76780501	76780800	0.398	0.607	0.642	0.614
UQ	9	77105101	77105400	4.062	3.407	2.370	3.296
UQ	9	78453001	78453300	0.797	1.075	1.066	1.415
UQ	9	78461101	78461400	1.647	2.802	2.316	1.298
UQ	9	78558301	78558600	0.505	0.090	0.107	0.031
UQ	9	78617701	78618000	0.373	0.468	0.263	0.248
UQ	9	78878701	78879000	0.558	0.187	0.234	0.056

UQ	9	78883501	78883800	0.477	0.094	0.132	0.031
UQ	9	79222501	79222800	0.690	0.912	0.346	0.340
UQ	9	79474801	79475100	1.731	0.914	3.030	3.386
UQ	9	79495801	79496100	1.836	2.549	1.329	1.714
UQ	9	80557501	80557800	2.530	1.581	1.456	0.954
UQ	9	80595001	80595300	0.869	2.613	2.414	1.566
UQ	9	80612701	80613000	1.116	0.541	0.448	0.405
UQ	9	80625301	80625600	4.252	3.856	3.480	3.712
UQ	9	80713801	80714100	0.637	0.375	0.214	0.284
UQ	9	80787601	80787900	0.532	0.189	0.267	0.193
UQ	9	80788201	80788500	1.039	0.515	0.419	0.370
UQ	9	81557101	81557400	0.640	0.281	0.374	0.329
UQ	9	81886201	81886500	0.771	1.193	0.535	0.533
UQ	9	82526701	82527000	0.637	0.352	0.210	0.446
UQ	9	82639801	82640100	1.066	0.605	0.481	0.502
UQ	9	82650601	82650900	0.105	0.444	0.481	0.533
UQ	9	82665901	82666200	0.397	0.725	1.543	0.776
UQ	9	83057101	83057400	0.477	0.725	0.288	0.634
UQ	9	83994301	83994600	2.394	1.566	1.741	1.587
UQ	9	84227101	84227400	0.531	0.234	0.107	0.391
UQ	9	84291601	84291900	0.747	0.165	0.399	0.248
UQ	9	84615601	84615900	0.534	0.210	0.292	0.279
UQ	9	86059801	86060100	0.186	0.468	0.317	0.360
UQ	9	86467501	86467800	1.089	0.448	0.395	0.426
UQ	9	86488201	86488500	2.471	1.523	2.098	1.764
UQ	9	87459601	87459900	0.502	0.069	0.082	0.167
UQ	9	87629701	87630000	0.503	0.144	0.160	0.193
UQ	9	87632701	87633000	0.637	0.326	0.399	0.274
UQ	9	87707701	87708000	0.744	0.560	0.239	0.299
UQ	9	87835501	87835800	1.274	0.820	0.983	0.751
UQ	9	88019401	88019700	4.014	2.774	2.967	2.931
UQ	9	88480801	88481100	0.266	0.515	0.374	0.472
UQ	9	89122201	89122500	3.412	2.148	2.209	2.093
UQ	9	89307601	89307900	2.787	1.622	1.407	1.167
UQ	9	89593201	89593500	3.227	2.128	2.138	2.013
UQ	9	89639101	89639400	1.171	0.371	0.695	0.669
UQ	9	89674201	89674500	0.477	0.049	0.132	0.218
UQ	9	89763901	89764200	0.745	0.144	0.189	0.218
UQ	9	90381001	90381300	0.316	1.279	1.806	1.414
UQ	9	91566001	91566300	0.292	0.515	0.481	0.527
UQ	9	91594801	91595100	0.718	0.094	0.379	0.254
UQ	9	91800901	91801200	0.503	0.047	0.214	0.173
UQ	9	91966201	91966500	0.637	0.468	0.346	0.360
UQ	9	92301601	92301900	0.479	0.631	0.210	0.335
UQ	9	92814901	92815200	3.702	2.270	2.313	2.470
UQ	9	93164101	93164400	0.745	0.420	0.292	0.385
UQ	9	93165601	93165900	0.534	1.680	2.174	2.342
UQ	9	93196801	93197100	1.357	0.916	0.760	0.907
UQ	9	93324301	93324600	0.663	0.185	0.230	0.142
UQ	9	93357301	93357600	0.558	0.444	0.424	0.608
UQ	9	93429601	93429900	3.593	1.603	1.268	1.714
UQ	9	93904801	93905100	0.879	0.352	0.243	0.304
UQ	9	93929101	93929400	1.039	0.305	0.247	0.446
UQ	9	93946501	93946800	0.558	0.376	0.827	0.385
UQ	9	93999301	93999600	0.239	0.446	0.481	0.502
UQ	9	94031701	94032000	1.729	1.071	0.881	2.099
UQ	9	94071301	94071600	1.491	2.800	2.602	3.833
UQ	9	94155901	94156200	4.494	7.547	7.762	7.327
UQ	9	94464301	94464600	1.331	1.852	0.954	0.213
UQ	X	79801	80100	0.268	0.770	0.481	0.558
UQ	X	334801	335100	3.376	4.229	2.296	2.698
UQ	X	339601	339900	3.617	2.828	2.871	3.114
UQ	X	384601	384900	6.275	4.311	3.970	4.272
UQ	X	384901	385200	5.880	3.015	3.259	3.365
UQ	X	433501	433800	0.105	0.749	0.267	0.416
UQ	X	442201	442500	2.949	2.197	1.601	2.484
UQ	X	480301	480600	1.195	0.935	1.522	1.516

UQ	X	567901	568200	3.084	2.510	2.229	2.510
UQ	X	779701	780000	4.601	3.152	3.117	3.529
UQ	X	920401	920700	0.161	0.539	0.560	0.335
UQ	X	947101	947400	0.802	1.148	0.156	0.329
UQ	X	1150501	1150800	0.447	1.777	2.114	1.861
UQ	X	1170601	1170900	0.292	0.094	0.025	0.497
UQ	X	1447801	1448100	4.989	7.175	4.336	5.892
UQ	X	1533001	1533300	0.847	2.993	4.168	2.977
UQ	X	1537501	1537800	1.960	4.151	3.834	5.582
UQ	X	1581301	1581600	2.461	4.971	4.557	3.858
UQ	X	1745401	1745700	0.716	0.281	0.370	0.396
UQ	X	1981801	1982100	0.744	0.489	0.239	0.299
UQ	X	2242801	2243100	4.383	0.522	2.416	2.659
UQ	X	2530801	2531100	1.278	0.935	0.881	1.643
UQ	X	2733301	2733600	0.745	0.466	0.531	0.268
UQ	X	2864101	2864400	0.532	0.255	0.288	0.167
UQ	X	3073201	3073500	1.412	0.792	0.983	0.948
UQ	X	3089101	3089400	0.826	0.466	0.395	0.324
UQ	X	3288601	3288900	0.552	1.483	1.534	2.297
UQ	X	3321901	3322200	2.126	1.478	1.431	1.227
UQ	X	3391501	3391800	0.560	0.421	0.267	0.664
UQ	X	3615601	3615900	2.846	1.798	1.819	1.328
UQ	X	3615901	3616200	5.151	3.536	2.523	1.803
UQ	X	3617101	3617400	0.371	0.796	0.584	0.664
UQ	X	3741901	3742200	3.143	2.268	1.850	2.317
UQ	X	4161601	4161900	0.105	0.491	0.321	0.279
UQ	X	4166101	4166400	1.278	1.450	0.987	1.055
UQ	X	4531501	4531800	0.663	1.847	1.304	1.333
UQ	X	4545901	4546200	0.160	0.493	0.317	0.335
UQ	X	4579201	4579500	2.794	2.008	2.229	1.384
UQ	X	4734001	4734300	2.233	1.637	0.987	1.414
UQ	X	5012101	5012400	1.037	0.607	0.317	0.416
UQ	X	5121001	5121300	5.186	4.349	3.781	4.716
UQ	X	5211301	5211600	1.520	1.939	2.122	2.398
UQ	X	5211901	5212200	0.000	1.800	1.775	2.354
UQ	X	5212201	5212500	0.000	4.404	5.180	5.496
UQ	X	5256601	5256900	0.345	0.562	0.214	0.522
UQ	X	5371801	5372100	2.683	1.023	1.518	1.709
UQ	X	5404201	5404500	0.507	0.375	0.292	0.279
UQ	X	5807701	5808000	2.744	1.762	1.436	0.922
UQ	X	5808001	5808300	2.579	1.622	1.235	0.902
UQ	X	5817001	5817300	1.771	0.494	0.210	0.594
UQ	X	5817301	5817600	4.238	2.319	2.855	3.017
UQ	X	6116401	6116700	0.240	0.470	0.477	0.522
UQ	X	6124801	6125100	2.713	1.828	1.476	1.461
UQ	X	6280801	6281100	3.034	0.811	1.929	0.685
UQ	X	6281101	6281400	2.008	0.118	0.239	0.198
UQ	X	6281401	6281700	5.475	2.330	3.116	2.135
UQ	X	6357601	6357900	0.265	0.820	0.836	0.862
UQ	X	6492601	6492900	4.015	2.770	3.166	4.862
UQ	X	6581701	6582000	6.575	4.746	4.129	3.062
UQ	X	6582001	6582300	4.607	3.267	3.302	2.378
UQ	X	6721501	6721800	1.912	0.914	0.945	0.716
UQ	X	6750601	6750900	0.936	0.395	0.366	0.421
UQ	X	6782701	6783000	3.746	5.385	4.810	4.918
UQ	X	7173301	7173600	0.771	1.053	1.032	1.024
UQ	X	7254901	7255200	1.965	1.427	1.547	1.278
UQ	X	7255201	7255500	3.806	3.014	2.777	2.084
UQ	X	7680901	7681200	0.186	0.303	0.370	0.497
UQ	X	8264701	8265000	0.637	0.468	0.239	0.243
UQ	X	8715001	8715300	0.213	0.536	0.584	0.507
UQ	X	9036601	9036900	0.902	0.322	0.395	0.416
UQ	X	10948501	10948800	0.505	0.189	0.127	0.162
UQ	X	11272801	11273100	0.586	0.444	0.691	0.329
UQ	X	11699401	11699700	3.725	2.780	2.430	2.206
UQ	X	12305701	12306000	0.586	0.423	0.160	0.329
UQ	X	12369601	12369900	0.132	0.446	0.481	0.466

UQ	X	12607501	12607800	2.154	2.360	3.134	2.687
UQ	X	13124401	13124700	0.773	0.330	0.350	0.472
UQ	X	13205101	13205400	0.215	0.820	0.760	0.695
UQ	X	13701601	13701900	0.215	0.562	0.399	0.441
UQ	X	14006701	14007000	0.532	0.258	0.156	0.051
UQ	X	14315401	14315700	0.213	0.584	0.136	0.304
UQ	X	14708101	14708400	0.027	0.187	0.350	0.466
UQ	X	14988601	14988900	0.266	0.584	0.399	0.497
UQ	X	15242401	15242700	3.665	2.216	2.891	2.367
UQ	X	15450601	15450900	0.371	0.815	0.802	1.359
UQ	X	16574701	16575000	0.850	1.077	0.325	0.223
UQ	X	16980601	16980900	1.037	0.794	0.560	0.887
UQ	X	20845501	20845800	0.557	0.189	0.160	0.304
UQ	X	20956501	20956800	1.116	0.794	0.671	0.725
UQ	X	20971501	20971800	0.640	0.184	0.288	0.187
UQ	X	21150601	21150900	0.584	0.232	0.288	0.167
UQ	X	21337501	21337800	0.558	0.397	0.239	0.497
UQ	X	22558801	22559100	0.903	1.290	0.531	1.222
UQ	X	22559101	22559400	1.008	1.407	0.604	1.359
UQ	X	22625401	22625700	3.888	2.955	2.748	2.540
UQ	X	22999801	23000100	0.187	0.444	0.346	0.411
UQ	X	23469301	23469600	0.561	0.279	0.210	0.248
UQ	X	24511501	24511800	4.175	2.577	2.343	2.143
UQ	X	25061401	25061700	0.508	0.142	0.185	0.137
UQ	X	25445401	25445700	2.770	1.633	1.197	1.536
UQ	X	25447201	25447500	0.558	0.257	0.140	0.137
UQ	X	27335401	27335700	0.558	0.701	0.798	0.771
UQ	X	28275601	28275900	0.908	0.515	0.510	0.497
UQ	X	28588501	28588800	0.558	0.444	0.267	0.086
UQ	X	28680001	28680300	0.508	0.047	0.107	0.112
UQ	X	29553001	29553300	0.715	0.959	0.399	0.304
UQ	X	29707501	29707800	1.166	0.208	0.486	0.547
UQ	X	31072801	31073100	0.240	0.071	0.745	0.603
UQ	X	31542001	31542300	0.982	0.444	0.477	0.608
UQ	X	33231301	33231600	3.428	2.068	1.933	2.312
UQ	X	34210801	34211100	0.957	0.584	0.852	1.024
UQ	X	34247701	34248000	0.823	0.096	0.337	0.259
UQ	X	34284301	34284600	0.613	0.328	0.370	0.913
UQ	X	34384801	34385100	0.586	0.185	0.218	0.254
UQ	X	34682401	34682700	0.987	1.682	1.716	1.734
UQ	X	35002501	35002800	0.531	0.303	0.156	0.335
UQ	X	36622501	36622800	1.302	0.886	0.789	0.497
UQ	X	36835801	36836100	0.586	0.047	0.181	0.279
UQ	X	37493701	37494000	0.718	0.331	0.292	0.329
UQ	X	38718001	38718300	0.873	0.446	0.214	0.329
UQ	X	38718301	38718600	0.771	0.330	0.132	0.193
UQ	X	38898301	38898600	0.558	0.302	0.210	0.198
UQ	X	39252901	39253200	0.455	1.270	1.206	1.111
UQ	X	39460801	39461100	0.692	0.255	0.263	0.218
UQ	X	39662101	39662400	0.374	0.212	0.531	0.056
UQ	X	40408501	40408800	0.319	0.635	0.609	0.664
UQ	X	40607101	40607400	0.584	0.187	0.399	0.329
UQ	X	42032701	42033000	0.374	0.753	1.090	0.771
UQ	X	42522301	42522600	0.532	0.165	0.210	0.268
UQ	X	43385401	43385700	1.199	0.912	0.609	0.902
UQ	X	44365801	44366100	1.195	0.981	0.745	0.771
UQ	X	45093601	45093900	1.011	0.701	0.827	0.603
UQ	X	45199501	45199800	0.558	0.258	0.292	0.324
UQ	X	46702201	46702500	0.560	0.397	0.350	0.193
UQ	X	47372101	47372400	0.184	0.629	0.695	0.634
UQ	X	47663701	47664000	0.318	0.094	0.214	0.608
UQ	X	48842701	48843000	0.398	1.221	0.934	0.988
UQ	X	50250601	50250900	0.744	0.421	0.477	0.553
UQ	X	52270801	52271100	0.800	0.562	0.481	0.385
UQ	X	52601401	52601700	0.611	0.395	0.827	0.857
UQ	X	52622101	52622400	2.234	1.639	1.917	1.962
UQ	X	52625401	52625700	1.140	2.332	1.971	1.724

UQ	X	52797301	52797600	0.186	0.586	0.555	0.533
UQ	X	53051401	53051700	0.290	0.727	0.584	0.669
UQ	X	53527801	53528100	0.639	0.303	0.234	0.193
UQ	X	54297001	54297300	1.144	1.358	0.798	1.460
UQ	X	57408001	57408300	0.505	0.303	0.156	0.243
UQ	X	58395001	58395300	0.477	0.096	0.267	0.162
UQ	X	58525201	58525500	1.061	0.635	0.522	0.639
UQ	X	58561201	58561500	0.824	0.702	0.185	0.578
UQ	X	59464201	59464500	0.826	0.208	0.332	0.299
UQ	X	59993701	59994000	0.373	0.631	0.506	0.578
UQ	X	60378001	60378300	1.439	0.629	0.836	0.943
UQ	X	60999601	60999900	0.400	0.538	0.535	0.745
UQ	X	61243201	61243500	0.105	0.328	0.428	0.684
UQ	X	61515301	61515600	1.060	0.468	0.354	0.603
UQ	X	65455501	65455800	0.160	0.375	0.617	0.466
UQ	X	65650201	65650500	0.132	0.350	0.506	0.553
UQ	X	66372301	66372600	0.584	0.257	0.292	0.187
UQ	X	66372601	66372900	0.824	0.539	0.292	0.441
UQ	X	66435001	66435300	1.360	2.343	2.171	2.043
UQ	X	66435301	66435600	1.837	3.274	2.151	2.130
UQ	X	66464401	66464700	0.481	0.118	0.082	0.025
UQ	X	66688201	66688500	0.665	0.285	0.243	0.309
UQ	X	68742601	68742900	0.426	0.234	0.560	0.193
UQ	X	68861401	68861700	0.053	0.212	0.477	0.243
UQ	X	69120601	69120900	0.503	0.140	0.267	0.137
UQ	X	69799201	69799500	0.744	0.562	0.395	0.329
UQ	X	69830401	69830700	0.507	0.187	0.239	0.329
UQ	X	70398001	70398300	0.347	0.493	0.078	0.025
UQ	X	71206801	71207100	0.297	0.918	1.144	0.902
UQ	X	71451301	71451600	0.716	0.330	0.288	0.360
UQ	X	73492201	73492500	0.106	0.371	0.671	0.497
UQ	X	74088901	74089200	0.957	0.652	1.139	0.745
UQ	X	76522501	76522800	0.560	0.326	0.395	0.882
UQ	X	76592101	76592400	1.276	1.588	0.818	1.714
UQ	X	76614901	76615200	1.812	0.764	1.094	0.593
UQ	X	76740601	76740900	0.771	0.654	0.399	0.497
UQ	X	77172301	77172600	0.718	0.373	0.374	0.421
UQ	X	77527501	77527800	1.037	0.515	0.506	0.943
UQ	X	77676301	77676600	1.487	0.860	0.651	0.690
UQ	X	77874601	77874900	0.823	1.191	1.168	0.477
UQ	X	77899201	77899500	0.184	0.980	0.453	0.553
UQ	X	78321301	78321600	0.505	0.167	0.058	0.193
UQ	X	78379201	78379500	0.134	0.562	0.267	0.416
UQ	X	78439201	78439500	1.647	1.989	2.102	2.124
UQ	X	79078201	79078500	0.503	0.071	0.029	0.132
UQ	X	79240801	79241100	0.558	0.142	0.292	0.132
UQ	X	79528501	79528800	0.532	0.096	0.132	0.106
UQ	X	79975501	79975800	0.637	0.307	0.239	0.279
UQ	X	80282401	80282700	1.589	0.837	0.789	0.806
UQ	X	80963101	80963400	0.534	0.120	0.029	0.106
UQ	X	81087001	81087300	0.613	0.116	0.243	0.223
UQ	X	81441001	81441300	0.824	0.330	0.337	0.441
UQ	X	82348201	82348500	0.773	0.399	0.321	0.329
UQ	X	83243701	83244000	0.347	0.727	0.794	0.634
UQ	X	84119701	84120000	0.186	0.281	0.267	0.553
UQ	X	84165001	84165300	1.547	0.820	0.834	0.639
UQ	X	85781701	85782000	2.976	2.038	2.017	3.671
UQ	X	86121901	86122200	0.637	0.303	0.317	0.157
UQ	X	86291701	86292000	2.189	1.146	1.217	0.578
UQ	X	86292001	86292300	1.224	0.637	0.716	0.466
UQ	X	87421501	87421800	1.537	0.375	0.432	0.284
UQ	X	87421801	87422100	1.545	0.350	0.346	0.254
UQ	X	88752901	88753200	0.294	0.493	0.453	0.553
UQ	X	89575801	89576100	1.566	2.015	2.613	2.373
UQ	X	92364301	92364600	0.718	0.399	0.341	0.391
UQ	X	93098701	93099000	0.505	0.232	0.272	0.248
UQ	X	93549601	93549900	2.255	1.614	1.694	1.638

UQ	X	93729901	93730200	0.799	0.468	0.432	0.279
UQ	X	93730201	93730500	0.694	0.397	0.481	0.279
UQ	X	93900301	93900600	0.774	0.395	0.395	0.198
UQ	X	94345201	94345500	0.400	0.749	0.774	1.080
UQ	X	94788901	94789200	0.505	0.000	0.025	0.025
UQ	X	95278501	95278800	0.505	0.654	0.317	0.162
UQ	X	95428501	95428800	0.079	0.560	0.263	0.411
UQ	X	95610901	95611200	0.586	0.257	0.259	0.274
UQ	X	95765101	95765400	0.690	0.234	0.481	0.416
UQ	X	95867101	95867400	0.745	0.187	0.366	0.309
UQ	X	96251701	96252000	1.115	0.725	0.789	0.751
UQ	X	99380401	99380700	0.665	0.399	0.346	0.324
UQ	X	99466201	99466500	0.718	0.324	0.288	0.274
UQ	X	100115101	100115400	1.491	1.009	0.560	0.263
UQ	X	100908001	100908300	0.105	0.491	0.453	0.558
UQ	X	101514901	101515200	0.718	0.279	0.214	0.416
UQ	X	102937801	102938100	0.079	0.517	0.317	0.248
UQ	X	103466101	103466400	0.665	0.305	0.082	0.340
UQ	X	103486501	103486800	0.771	0.468	0.535	0.360
UQ	X	105930301	105930600	0.639	0.352	0.234	0.365
UQ	X	106031401	106031700	3.292	2.525	2.497	2.109
UQ	X	106298401	106298700	0.347	0.468	0.214	0.472
UQ	X	107341201	107341500	1.037	0.468	0.555	0.355
UQ	X	107856301	107856600	0.186	0.375	0.448	0.385
UQ	X	108150901	108151200	0.290	0.770	0.970	0.826
UQ	X	109233901	109234200	0.586	0.303	0.321	0.309
UQ	X	110035201	110035500	0.850	0.418	0.127	0.162
UQ	X	110469001	110469300	2.473	1.609	1.398	1.465
UQ	X	110983801	110984100	0.692	0.373	0.292	0.365
UQ	X	111440701	111441000	1.381	1.824	1.859	1.709
UQ	X	113762101	113762400	0.053	0.442	0.346	0.223
UQ	X	114649501	114649800	0.957	0.841	0.823	0.826
UQ	X	115132801	115133100	0.507	0.189	0.156	0.117
UQ	X	115338901	115339200	4.010	2.414	2.341	3.291
UQ	X	115500001	115500300	0.689	0.277	1.008	1.019
UQ	X	116248801	116249100	1.115	0.539	0.551	0.289
UQ	X	116512201	116512500	0.771	0.253	0.424	0.193
UQ	X	116790601	116790900	2.545	3.735	3.636	3.651
UQ	X	116800501	116800800	0.637	0.258	0.259	0.309
UQ	X	117010801	117011100	1.515	1.869	1.806	2.423
UQ	X	117290101	117290400	0.160	0.560	0.239	0.279
UQ	X	118216201	118216500	0.582	0.092	0.103	0.142
UQ	X	118288201	118288500	0.077	0.375	0.695	0.304
UQ	X	118327801	118328100	0.477	0.236	0.239	0.106
UQ	X	119384101	119384400	0.532	0.116	0.296	0.299
UQ	X	119975101	119975400	1.065	0.888	0.852	0.745
UQ	X	120294601	120294900	0.850	0.466	0.403	0.385
UQ	X	121186801	121187100	0.053	0.326	0.477	0.461
UQ	X	121887301	121887600	0.666	0.305	0.325	0.248
UQ	X	122040001	122040300	0.000	0.258	0.419	0.365
UQ	X	122523901	122524200	0.477	0.375	0.185	0.223
UQ	X	122550901	122551200	0.769	0.448	0.448	0.279
UQ	X	122745001	122745300	0.187	0.468	0.317	0.416
UQ	X	123332101	123332400	0.211	0.538	0.370	0.608
UQ	X	125108101	125108400	0.747	0.421	0.239	0.441
UQ	X	125596501	125596800	0.771	0.350	0.419	0.497
UQ	X	125850601	125850900	0.428	0.747	0.798	0.639
UQ	X	126363301	126363600	0.211	0.560	0.473	0.547
UQ	X	126624601	126624900	0.345	0.539	0.506	0.497
UQ	X	128172601	128172900	0.450	0.258	0.185	0.025
UQ	X	128845801	128846100	0.531	0.165	0.214	0.193
UQ	X	129377701	129378000	0.534	0.305	0.136	0.248
UQ	X	130374001	130374300	0.560	0.348	0.370	0.112
UQ	X	131165401	131165700	0.744	0.491	0.477	0.304
UQ	X	132192301	132192600	0.794	0.350	0.259	0.324
UQ	X	132198601	132198900	2.128	1.751	1.353	1.861
UQ	X	133343401	133343700	0.081	0.444	0.185	0.167

UQ	X	133540801	133541100	0.797	0.513	0.403	0.254
UQ	X	134372401	134372700	0.426	0.629	0.214	0.690
UQ	X	134925601	134925900	0.720	0.515	0.428	0.248
Tan	1	24901	25200	5.093	3.257	4.639	4.898
Tan	1	62401	62700	0.774	1.313	0.580	0.512
Tan	1	290401	290700	1.541	2.820	1.665	1.674
Tan	1	426901	427200	0.450	0.749	0.555	0.329
Tan	1	661201	661500	0.640	1.004	0.613	0.690
Tan	1	986101	986400	2.712	2.124	2.949	2.504
Tan	1	1059901	1060200	0.694	0.418	1.090	0.745
Tan	1	1294501	1294800	0.450	1.056	0.317	0.391
Tan	1	1518901	1519200	1.436	3.903	1.239	2.579
Tan	1	1616401	1616700	3.871	2.682	4.410	3.924
Tan	1	1699201	1699500	0.292	0.729	0.341	0.299
Tan	1	1852501	1852800	0.266	0.562	0.156	0.223
Tan	1	1904401	1904700	1.884	3.017	3.529	2.368
Tan	1	1930801	1931100	1.355	2.242	1.052	1.962
Tan	1	2293801	2294100	0.268	0.560	0.107	0.218
Tan	1	2301301	2301600	3.083	2.058	3.121	2.774
Tan	1	2518801	2519100	0.345	0.727	0.259	0.254
Tan	1	2541001	2541300	0.907	1.289	0.473	0.639
Tan	1	2549701	2550000	3.196	4.046	2.949	3.038
Tan	1	2716801	2717100	0.081	0.425	0.054	0.081
Tan	1	2741401	2741700	0.134	0.371	0.000	0.112
Tan	1	2749801	2750100	4.275	3.810	4.434	5.167
Tan	1	2783401	2783700	0.744	1.313	0.481	0.776
Tan	1	2802601	2802900	1.276	2.086	1.155	0.765
Tan	1	2996401	2996700	0.186	0.538	0.230	0.167
Tan	1	3168301	3168600	0.532	0.281	0.346	0.223
Tan	1	3563701	3564000	0.158	0.586	0.029	0.238
Tan	1	3610801	3611100	2.844	3.298	2.271	2.286
Tan	1	3634501	3634800	0.186	0.607	0.185	0.193
Tan	1	3777301	3777600	0.398	0.725	0.321	0.324
Tan	1	3814201	3814500	2.289	0.768	1.694	2.019
Tan	1	3879001	3879300	0.581	1.221	0.453	0.461
Tan	1	3892801	3893100	0.426	0.607	0.374	0.472
Tan	1	3895201	3895500	0.242	0.588	0.160	0.137
Tan	1	3912901	3913200	0.237	0.560	0.263	0.198
Tan	1	4094401	4094700	0.639	0.354	0.662	0.776
Tan	1	4197301	4197600	0.266	0.890	0.337	0.441
Tan	1	4250401	4250700	1.092	2.113	1.130	0.578
Tan	1	4378801	4379100	0.666	0.281	0.453	0.497
Tan	1	4565401	4565700	0.132	0.466	0.214	0.578
Tan	1	4968601	4968900	0.584	0.375	0.633	0.583
Tan	1	5147101	5147400	0.187	0.558	0.214	0.223
Tan	1	5531701	5532000	0.345	0.584	0.239	0.340
Tan	1	5680801	5681100	0.239	0.470	0.239	0.248
Tan	1	5755501	5755800	1.939	2.270	1.601	1.825
Tan	1	5769001	5769300	0.663	1.210	0.325	0.558
Tan	1	5863201	5863500	1.625	1.165	1.835	1.982
Tan	1	6009001	6009300	0.928	2.276	0.872	1.100
Tan	1	6045301	6045600	0.055	0.468	0.078	0.173
Tan	1	6048301	6048600	0.371	1.264	0.252	0.329
Tan	1	6277501	6277800	0.321	0.584	0.267	0.223
Tan	1	6429601	6429900	0.665	1.006	0.473	0.533
Tan	1	6477601	6477900	0.745	1.457	0.662	0.913
Tan	1	6841501	6841800	1.194	0.521	1.144	1.491
Tan	1	6901201	6901500	0.161	0.446	0.087	0.137
Tan	1	7095301	7095600	0.055	0.420	0.127	0.051
Tan	1	7392901	7393200	0.213	0.749	0.267	0.299
Tan	1	7577401	7577700	3.084	2.109	2.863	3.407
Tan	1	7728901	7729200	1.729	2.010	1.115	2.099
Tan	1	8158201	8158500	0.186	0.418	0.156	0.137
Tan	1	8209201	8209500	2.473	1.006	1.741	1.582
Tan	1	8315101	8315400	0.505	0.770	0.263	0.396
Tan	1	8386201	8386500	0.319	0.515	0.234	0.137
Tan	1	8403601	8403900	1.008	1.424	0.999	0.943

Tan	1	8421901	8422200	0.186	0.515	0.317	0.106
Tan	1	8509501	8509800	0.398	0.633	0.292	0.355
Tan	1	8592301	8592600	0.132	0.539	0.292	0.254
Tan	1	8649601	8649900	2.025	2.761	1.801	1.505
Tan	1	8657101	8657400	0.136	0.513	0.185	0.223
Tan	1	8759101	8759400	0.718	0.841	1.008	0.695
Tan	1	8792401	8792700	0.156	0.607	0.111	0.051
Tan	1	8793001	8793300	0.876	1.635	0.847	0.897
Tan	1	8827501	8827800	0.398	0.792	0.152	0.309
Tan	1	8991601	8991900	1.041	2.206	0.979	1.146
Tan	1	9490801	9491100	0.161	0.657	0.234	0.157
Tan	1	9554101	9554400	2.576	1.186	2.225	2.378
Tan	1	9554401	9554700	3.881	2.364	3.583	4.476
Tan	1	9625801	9626100	0.134	0.656	0.350	0.421
Tan	1	9698701	9699000	0.239	0.942	0.194	0.203
Tan	1	9699001	9699300	0.240	1.109	0.189	0.396
Tan	1	9840601	9840900	0.883	1.470	0.395	0.380
Tan	1	9905401	9905700	2.654	3.757	2.452	1.926
Tan	1	9929701	9930000	1.252	2.345	1.344	1.287
Tan	1	9976801	9977100	2.916	0.618	3.525	4.376
Tan	1	10012501	10012800	1.008	0.657	0.218	0.218
Tan	1	10275301	10275600	0.240	0.560	0.214	0.132
Tan	1	10319701	10320000	0.448	2.066	1.440	1.044
Tan	1	10596301	10596600	1.278	1.002	1.567	1.460
Tan	1	10953001	10953300	1.752	2.461	1.436	1.764
Tan	1	11046601	11046900	2.712	1.873	2.829	2.890
Tan	1	11067601	11067900	2.154	2.729	1.772	1.643
Tan	1	11310601	11310900	0.608	1.169	0.214	0.594
Tan	1	11311201	11311500	1.436	2.454	0.859	0.867
Tan	1	11319601	11319900	0.563	1.030	0.288	0.324
Tan	1	11537701	11538000	1.219	1.847	0.885	1.172
Tan	1	11595601	11595900	7.504	3.078	6.710	6.724
Tan	1	12010801	12011100	0.265	0.706	0.156	0.051
Tan	1	12021601	12021900	0.211	0.745	0.210	0.243
Tan	1	12021901	12022200	0.321	0.940	0.107	0.497
Tan	1	12154801	12155100	0.527	1.611	0.573	0.466
Tan	1	12201901	12202200	0.213	0.493	0.189	0.279
Tan	1	12295501	12295800	1.144	1.262	1.407	1.496
Tan	1	12384301	12384600	1.118	1.923	1.025	1.105
Tan	1	12532801	12533100	0.213	0.633	0.272	0.254
Tan	1	12603601	12603900	0.266	0.468	0.317	0.304
Tan	1	12713101	12713400	1.729	2.130	1.427	1.678
Tan	1	13226401	13226700	1.642	2.853	1.505	1.298
Tan	1	13226701	13227000	0.828	1.568	0.888	0.852
Tan	1	13283701	13284000	1.492	0.401	1.799	1.836
Tan	1	13284001	13284300	3.725	1.328	4.974	3.711
Tan	1	13288501	13288800	0.211	0.560	0.230	0.167
Tan	1	13469701	13470000	0.213	0.517	0.321	0.112
Tan	1	13470301	13470600	0.823	0.468	1.086	0.913
Tan	1	13503601	13503900	1.171	0.704	1.115	1.212
Tan	1	13853701	13854000	0.611	0.328	0.399	0.553
Tan	1	13913401	13913700	0.106	0.493	0.218	0.193
Tan	1	14003701	14004000	0.962	1.496	0.892	0.669
Tan	1	14371801	14372100	0.266	0.721	0.317	0.167
Tan	1	14469601	14469900	0.210	1.470	0.267	0.552
Tan	1	14929501	14929800	0.794	1.306	0.778	0.740
Tan	1	14967901	14968200	0.907	0.283	0.531	0.695
Tan	1	15163201	15163500	0.134	0.539	0.078	0.086
Tan	1	15256201	15256500	1.618	2.203	1.048	0.988
Tan	1	15468001	15468300	0.610	2.399	0.742	0.786
Tan	1	15772201	15772500	0.319	0.704	0.370	0.385
Tan	1	15772801	15773100	3.665	1.841	4.524	3.382
Tan	1	15798001	15798300	0.160	0.423	0.078	0.248
Tan	1	15900301	15900600	0.106	0.423	0.214	0.218
Tan	1	15940801	15941100	0.694	1.781	0.581	0.933
Tan	1	15941101	15941400	0.853	2.109	0.845	1.201
Tan	1	15980101	15980400	1.274	3.386	1.395	1.484

Tan	1	16527601	16527900	2.683	3.613	1.016	2.109
Tan	1	16535701	16536000	0.055	0.444	0.078	0.193
Tan	1	17207701	17208000	0.345	0.538	0.317	0.299
Tan	1	17217001	17217300	0.642	1.384	0.823	0.644
Tan	1	17247301	17247600	0.160	0.515	0.247	0.112
Tan	1	17378101	17378400	0.314	1.339	0.600	0.553
Tan	1	17615701	17616000	0.373	0.468	0.346	0.553
Tan	1	17694001	17694300	0.563	1.377	0.461	0.385
Tan	1	17779501	17779800	0.532	0.702	0.189	0.167
Tan	1	17887501	17887800	1.303	0.796	1.255	1.815
Tan	1	17895601	17895900	1.942	2.409	1.407	1.694
Tan	1	17998501	17998800	3.273	3.997	3.126	3.230
Tan	1	18011401	18011700	0.134	0.768	0.312	0.441
Tan	1	18011701	18012000	0.584	1.878	0.662	1.070
Tan	1	18047401	18047700	0.477	0.818	0.662	0.472
Tan	1	18098701	18099000	1.810	1.311	1.797	1.825
Tan	1	18163801	18164100	2.078	1.706	1.144	2.084
Tan	1	18234601	18234900	0.395	1.468	1.090	1.055
Tan	1	18291001	18291300	0.694	1.026	0.419	0.472
Tan	1	18466501	18466800	0.400	0.846	0.243	0.350
Tan	1	18935101	18935400	0.240	0.704	0.288	0.187
Tan	1	18981901	18982200	1.991	2.405	1.538	1.982
Tan	1	19145101	19145400	0.213	0.773	0.424	0.355
Tan	1	19737601	19737900	1.199	1.995	0.909	1.176
Tan	1	19978801	19979100	0.131	0.470	0.165	0.167
Tan	1	20066101	20066400	0.527	0.961	0.535	0.553
Tan	1	20325301	20325600	0.584	0.935	0.078	0.588
Tan	1	20601001	20601300	1.518	2.373	0.979	1.399
Tan	1	20700001	20700300	0.240	0.777	0.321	0.309
Tan	1	20735401	20735700	0.505	0.843	0.370	0.452
Tan	1	21245401	21245700	0.160	0.517	0.239	0.248
Tan	1	21348301	21348600	0.156	0.609	0.214	0.137
Tan	1	21433801	21434100	0.639	0.914	0.662	0.502
Tan	1	21673801	21674100	0.589	1.285	0.593	0.684
Tan	1	22545301	22545600	0.239	0.794	0.272	0.263
Tan	1	22710601	22710900	0.134	0.444	0.140	0.132
Tan	1	23186401	23186700	1.355	0.912	0.535	0.563
Tan	1	23315401	23315700	0.981	0.493	0.823	1.050
Tan	1	24009301	24009600	0.106	0.395	0.111	0.056
Tan	1	24606601	24606900	1.226	2.100	1.394	1.440
Tan	1	25129801	25130100	0.160	0.470	0.132	0.051
Tan	1	25648801	25649100	0.052	0.418	0.000	0.051
Tan	1	25796701	25797000	0.319	0.444	0.370	0.527
Tan	1	26768101	26768400	0.131	0.727	0.116	0.137
Tan	1	27029701	27030000	0.294	0.674	0.234	0.268
Tan	1	27187201	27187500	0.186	1.311	0.502	0.776
Tan	1	27194401	27194700	0.266	0.466	0.107	0.274
Tan	1	27250501	27250800	1.197	1.802	1.103	0.907
Tan	1	27307201	27307500	0.077	0.558	0.189	0.137
Tan	1	27376201	27376500	0.266	0.583	0.127	0.218
Tan	1	27395401	27395700	0.182	1.306	0.201	0.365
Tan	1	27542401	27542700	4.496	1.712	3.110	3.788
Tan	1	27546601	27546900	0.108	0.468	0.140	0.081
Tan	1	27883501	27883800	1.649	2.510	1.757	1.627
Tan	1	27883801	27884100	1.750	2.665	2.147	1.734
Tan	1	28089301	28089600	0.878	1.661	1.032	1.080
Tan	1	28284301	28284600	0.160	0.513	0.185	0.132
Tan	1	28563301	28563600	0.210	0.611	0.218	0.056
Tan	1	28642501	28642800	0.507	0.865	0.510	0.436
Tan	1	28663501	28663800	0.826	0.515	0.938	0.882
Tan	1	28795201	28795500	0.215	0.538	0.247	0.248
Tan	1	28839601	28839900	0.371	0.654	0.234	0.365
Tan	1	28852201	28852500	0.458	1.262	0.230	0.137
Tan	1	28945501	28945800	0.081	0.423	0.078	0.137
Tan	1	29033101	29033400	0.081	0.442	0.082	0.056
Tan	1	29048101	29048400	0.242	0.706	0.214	0.167
Tan	1	29158801	29159100	0.397	0.187	0.399	0.634

Tan	1	29350501	29350800	0.136	0.562	0.321	0.279
Tan	1	29514901	29515200	0.586	0.376	0.214	0.193
Tan	1	30737701	30738000	0.426	0.916	0.497	0.385
Tan	1	31423801	31424100	0.184	0.493	0.210	0.167
Tan	1	31549801	31550100	0.400	0.891	0.239	0.340
Tan	1	32023201	32023500	0.852	1.075	0.720	0.573
Tan	1	32289901	32290200	0.108	0.444	0.160	0.142
Tan	1	33097801	33098100	0.428	0.747	0.535	0.497
Tan	1	33147901	33148200	0.850	1.053	0.239	0.492
Tan	1	33425401	33425700	0.186	1.169	0.408	0.391
Tan	1	33597001	33597300	0.210	0.513	0.156	0.106
Tan	1	33794101	33794400	0.081	0.468	0.127	0.086
Tan	1	33846601	33846900	0.479	0.865	0.185	0.999
Tan	1	34065301	34065600	0.187	0.723	0.194	0.162
Tan	1	34751701	34752000	0.507	0.912	0.374	0.360
Tan	1	34887001	34887300	0.266	0.536	0.259	0.279
Tan	1	35098501	35098800	0.244	0.841	0.453	0.365
Tan	1	35425801	35426100	0.397	0.931	0.049	0.340
Tan	1	35835301	35835600	1.353	0.843	1.395	1.379
Tan	1	35917501	35917800	0.240	0.772	0.308	0.335
Tan	1	36471901	36472200	0.079	0.446	0.136	0.173
Tan	1	36621901	36622200	0.184	0.588	0.239	0.254
Tan	1	37123501	37123800	0.163	0.652	0.243	0.304
Tan	1	37290601	37290900	1.702	0.935	1.712	1.506
Tan	1	37394701	37395000	0.160	0.489	0.107	0.254
Tan	1	37687501	37687800	0.210	0.678	0.292	0.340
Tan	1	37723501	37723800	0.268	0.888	0.415	0.335
Tan	1	37723801	37724100	0.213	0.792	0.288	0.340
Tan	1	37968001	37968300	0.132	0.423	0.054	0.218
Tan	1	38304901	38305200	0.052	0.586	0.054	0.061
Tan	1	38305201	38305500	0.292	1.103	0.087	0.228
Tan	1	38476201	38476500	1.326	1.869	1.436	1.384
Tan	1	38580001	38580300	2.313	2.806	1.884	2.367
Tan	1	38652601	38652900	0.134	0.633	0.054	0.162
Tan	1	39315901	39316200	0.266	0.489	0.263	0.248
Tan	1	39591901	39592200	0.718	0.305	0.477	0.826
Tan	1	39794101	39794400	0.960	2.500	1.301	0.740
Tan	1	39805201	39805500	1.386	2.476	1.786	1.562
Tan	1	40026001	40026300	3.344	4.774	2.624	3.596
Tan	1	40053901	40054200	0.234	0.796	0.267	0.284
Tan	1	40118401	40118700	0.850	1.004	0.584	0.639
Tan	1	40173301	40173600	0.319	0.818	0.127	0.248
Tan	1	40476301	40476600	0.000	0.371	0.054	0.031
Tan	1	40519801	40520100	0.160	0.603	0.152	0.198
Tan	1	40923901	40924200	0.158	0.444	0.132	0.142
Tan	1	41292301	41292600	0.586	0.446	0.239	0.329
Tan	1	42292201	42292500	0.532	0.863	0.214	0.385
Tan	1	42421501	42421800	0.079	0.586	0.317	0.329
Tan	1	42542701	42543000	0.160	0.657	0.317	0.315
Tan	1	42643801	42644100	0.403	1.440	0.366	0.319
Tan	1	42769501	42769800	1.091	0.798	0.481	0.547
Tan	1	42838801	42839100	0.240	0.564	0.054	0.157
Tan	1	42868801	42869100	1.676	0.862	1.629	1.430
Tan	1	43209001	43209300	0.610	0.185	0.638	0.628
Tan	1	43391101	43391400	0.347	0.607	0.189	0.268
Tan	1	43454401	43454700	0.187	0.466	0.267	0.248
Tan	1	43918501	43918800	0.373	0.865	0.395	0.289
Tan	1	44441401	44441700	0.668	0.206	0.724	0.659
Tan	1	44902801	44903100	0.345	0.539	0.341	0.218
Tan	1	44992501	44992800	0.000	0.425	0.103	0.025
Tan	1	45597901	45598200	0.266	0.513	0.243	0.335
Tan	1	45993601	45993900	0.239	0.444	0.132	0.279
Tan	1	46008001	46008300	0.161	0.513	0.288	0.274
Tan	1	46557901	46558200	0.532	0.421	0.156	0.081
Tan	1	46647001	46647300	0.345	1.008	0.466	0.370
Tan	1	46683301	46683600	1.089	0.515	1.159	1.471
Tan	1	47453401	47453700	0.213	0.515	0.259	0.137

Tan	1	47494801	47495100	0.266	0.538	0.317	0.385
Tan	1	47649301	47649600	0.160	0.420	0.054	0.162
Tan	1	47778301	47778600	0.398	0.839	0.354	0.248
Tan	1	47881201	47881500	0.584	0.120	0.453	0.877
Tan	1	48451801	48452100	0.453	0.798	0.555	0.142
Tan	1	49111501	49111800	0.502	1.077	0.745	0.335
Tan	1	49485001	49485300	0.106	0.420	0.321	0.248
Tan	1	50306101	50306400	0.290	0.798	0.189	0.274
Tan	1	51252301	51252600	0.589	1.120	0.321	0.400
Tan	1	53142901	53143200	0.797	1.525	0.379	0.700
Tan	1	53143201	53143500	0.319	0.609	0.218	0.187
Tan	1	55173301	55173600	0.081	0.420	0.185	0.081
Tan	1	55456801	55457100	0.186	0.420	0.185	0.112
Tan	1	56625601	56625900	0.716	1.169	0.745	0.675
Tan	1	56777101	56777400	0.132	0.491	0.210	0.304
Tan	1	57443701	57444000	0.715	1.332	0.593	0.893
Tan	1	57488701	57489000	0.426	0.749	0.296	0.162
Tan	1	57722401	57722700	0.319	0.628	0.234	0.198
Tan	1	57724501	57724800	0.026	0.539	0.259	0.193
Tan	1	57801001	57801300	0.292	0.493	0.317	0.279
Tan	1	59477101	59477400	0.266	0.702	0.535	0.502
Tan	1	59876701	59877000	0.321	0.890	0.410	0.385
Tan	1	60764701	60765000	0.053	0.494	0.103	0.162
Tan	1	61263001	61263300	0.532	0.796	0.609	0.547
Tan	1	61410301	61410600	0.373	0.538	0.082	0.355
Tan	1	61619101	61619400	0.345	0.489	0.638	0.304
Tan	1	61805101	61805400	0.557	0.818	0.633	1.050
Tan	1	61873501	61873800	0.373	0.796	0.205	0.436
Tan	1	62104501	62104800	0.158	0.588	0.152	0.000
Tan	1	62388601	62388900	0.186	0.468	0.317	0.360
Tan	1	62604301	62604600	0.611	0.069	0.424	0.477
Tan	1	62604601	62604900	0.560	0.069	0.428	0.477
Tan	1	62607601	62607900	0.237	1.360	0.366	0.568
Tan	1	62919301	62919600	0.371	0.818	0.432	0.477
Tan	1	62919601	62919900	0.558	0.890	0.609	0.715
Tan	1	63630601	63630900	0.666	0.865	0.535	0.411
Tan	1	64950001	64950300	0.079	0.628	0.218	0.228
Tan	1	65460301	65460600	0.052	0.444	0.156	0.137
Tan	1	65618401	65618700	0.160	0.446	0.132	0.274
Tan	1	66177601	66177900	0.137	1.259	0.392	0.177
Tan	1	66521101	66521400	1.912	3.529	2.677	2.743
Tan	1	66547801	66548100	0.349	0.820	0.247	0.167
Tan	1	66628501	66628800	0.081	0.468	0.288	0.056
Tan	1	66674401	66674700	0.131	0.442	0.111	0.137
Tan	1	66947401	66947700	0.639	0.165	0.448	0.461
Tan	1	67053001	67053300	0.134	0.371	0.029	0.056
Tan	1	67165201	67165500	3.383	1.564	3.322	3.357
Tan	1	67180201	67180500	1.358	1.751	1.251	1.141
Tan	1	67230601	67230900	0.105	0.420	0.132	0.187
Tan	1	67248001	67248300	0.318	1.077	0.629	0.329
Tan	1	67951201	67951500	0.210	0.633	0.185	0.248
Tan	1	68352901	68353200	0.160	0.491	0.160	0.268
Tan	1	68389501	68389800	0.402	1.122	0.366	0.567
Tan	1	68943001	68943300	0.931	1.691	1.061	0.786
Tan	1	69124801	69125100	0.345	0.652	0.379	0.248
Tan	1	69233101	69233400	0.376	0.745	0.156	0.193
Tan	1	69414301	69414600	0.211	0.470	0.078	0.086
Tan	1	69617401	69617700	0.477	1.053	0.711	0.826
Tan	1	70006801	70007100	0.692	0.818	0.531	0.634
Tan	1	70018801	70019100	1.116	0.281	1.217	1.273
Tan	1	70036201	70036500	0.479	0.704	0.453	0.279
Tan	1	70045501	70045800	0.661	0.328	0.959	0.963
Tan	1	70079701	70080000	0.318	0.680	0.428	0.385
Tan	1	70334701	70335000	0.797	0.468	0.370	0.634
Tan	1	70347901	70348200	1.166	0.437	1.344	1.172
Tan	1	70388701	70389000	0.079	0.395	0.160	0.081
Tan	1	70898401	70898700	0.053	0.515	0.054	0.268

Tan	1	71153101	71153400	0.187	0.536	0.132	0.193
Tan	1	71185501	71185800	0.134	0.494	0.165	0.086
Tan	1	71313601	71313900	0.213	0.586	0.218	0.279
Tan	1	71467501	71467800	0.869	1.912	0.230	1.161
Tan	1	72215701	72216000	6.612	1.770	4.129	4.790
Tan	1	72363601	72363900	0.694	0.161	0.658	0.669
Tan	1	72412501	72412800	1.382	2.605	1.215	1.394
Tan	1	72609001	72609300	0.079	0.629	0.160	0.193
Tan	1	72853801	72854100	0.186	0.465	0.214	0.218
Tan	1	73047001	73047300	0.000	0.448	0.058	0.162
Tan	1	73183801	73184100	0.931	1.476	1.152	0.254
Tan	1	73299001	73299300	0.266	0.659	0.321	0.274
Tan	1	73591501	73591800	0.853	0.375	0.881	0.614
Tan	1	73946101	73946400	0.266	0.442	0.185	0.248
Tan	1	74333701	74334000	0.292	0.723	0.346	0.173
Tan	1	74628901	74629200	1.808	3.841	2.178	1.426
Tan	1	75339601	75339900	1.115	1.802	1.021	0.947
Tan	1	75890101	75890400	0.371	0.822	0.185	0.452
Tan	1	75982501	75982800	1.250	2.064	0.892	0.927
Tan	1	76221601	76221900	0.321	0.631	0.292	0.187
Tan	1	76507201	76507500	0.557	0.258	0.802	0.502
Tan	1	76859401	76859700	0.720	0.305	0.617	0.578
Tan	1	76946401	76946700	0.342	1.326	0.353	0.329
Tan	1	77265301	77265600	0.799	1.916	0.836	1.101
Tan	1	77288101	77288400	0.106	0.493	0.156	0.132
Tan	1	77831701	77832000	2.579	1.397	1.835	1.739
Tan	1	78022801	78023100	0.106	0.470	0.025	0.137
Tan	1	79116901	79117200	0.479	0.725	0.453	0.421
Tan	1	80643601	80643900	1.492	0.607	0.901	0.893
Tan	1	80671501	80671800	0.745	0.865	0.292	0.603
Tan	1	80954101	80954400	0.136	0.772	0.395	0.142
Tan	1	80954401	80954700	0.027	0.605	0.272	0.056
Tan	1	81345001	81345300	0.266	0.116	0.580	0.385
Tan	1	81461401	81461700	0.690	0.843	0.428	0.695
Tan	1	81778801	81779100	0.318	1.004	0.551	0.502
Tan	1	82309801	82310100	0.268	0.704	0.301	0.304
Tan	1	83677801	83678100	0.903	0.448	0.827	0.837
Tan	1	83722501	83722800	0.187	0.699	0.214	0.198
Tan	1	83847301	83847600	0.161	0.609	0.263	0.416
Tan	1	83897701	83898000	0.161	0.493	0.189	0.162
Tan	1	84087301	84087600	0.605	1.356	0.457	0.751
Tan	1	85241401	85241700	2.315	3.534	1.728	2.260
Tan	1	85342201	85342500	0.323	0.837	0.403	0.304
Tan	1	85592101	85592400	0.844	1.770	0.876	0.599
Tan	1	85704601	85704900	0.187	0.794	0.390	0.380
Tan	1	85771201	85771500	0.294	0.564	0.288	0.198
Tan	1	85822501	85822800	0.266	0.656	0.399	0.279
Tan	1	86010001	86010300	0.373	0.092	0.502	0.396
Tan	1	86112001	86112300	0.211	0.773	0.424	0.213
Tan	1	86171101	86171400	0.450	1.481	0.593	0.481
Tan	1	86180701	86181000	0.374	0.772	0.247	0.421
Tan	1	86286301	86286600	3.991	5.327	3.194	4.107
Tan	1	86296201	86296500	1.171	1.880	0.778	0.938
Tan	1	86412001	86412300	1.571	1.189	1.719	0.821
Tan	1	86585401	86585700	0.424	0.236	0.506	0.025
Tan	1	86829901	86830200	1.240	2.390	0.945	1.136
Tan	1	86907901	86908200	0.186	0.420	0.189	0.167
Tan	1	87054601	87054900	0.428	0.935	0.354	0.446
Tan	1	87178201	87178500	0.268	0.772	0.403	0.355
Tan	1	87438901	87439200	0.079	0.541	0.103	0.162
Tan	1	87713701	87714000	0.613	1.197	0.662	0.705
Tan	1	88026001	88026300	0.240	0.493	0.239	0.223
Tan	1	88382401	88382700	2.581	4.257	2.553	3.108
Tan	1	88597501	88597800	0.397	0.865	0.346	0.659
Tan	1	88680901	88681200	0.216	0.659	0.214	0.162
Tan	1	89296501	89296800	0.242	0.609	0.239	0.238
Tan	1	89409601	89409900	0.081	0.519	0.111	0.056

Tan	1	89715901	89716200	0.582	1.223	0.560	0.396
Tan	1	89812201	89812500	0.244	0.871	0.259	0.248
Tan	1	89832901	89833200	0.132	0.586	0.292	0.147
Tan	1	89874301	89874600	0.160	0.515	0.185	0.198
Tan	1	89982601	89982900	5.741	8.417	4.367	4.977
Tan	1	90100501	90100800	0.081	0.562	0.239	0.329
Tan	1	90555601	90555900	0.318	0.444	0.054	0.274
Tan	1	91500001	91500300	0.184	0.674	0.194	0.223
Tan	1	91567201	91567500	0.532	1.051	0.711	0.664
Tan	1	91658401	91658700	0.402	1.071	0.642	0.279
Tan	1	91883101	91883400	0.611	0.493	0.156	0.304
Tan	1	91901101	91901400	0.292	0.701	0.111	0.396
Tan	1	92132401	92132700	0.476	1.056	0.535	0.628
Tan	1	92156401	92156700	2.020	2.386	1.431	1.638
Tan	1	92332501	92332800	0.187	0.674	0.223	0.213
Tan	1	92560801	92561100	0.266	0.420	0.267	0.223
Tan	1	93174001	93174300	2.283	2.731	1.723	2.185
Tan	1	93192001	93192300	0.189	0.539	0.181	0.025
Tan	1	93364501	93364800	0.237	0.489	0.189	0.167
Tan	1	93421501	93421800	0.213	0.470	0.189	0.056
Tan	1	93473401	93473700	0.321	0.768	0.506	0.248
Tan	1	93660601	93660900	1.862	1.450	1.790	1.770
Tan	1	93685201	93685500	1.674	0.856	1.710	2.054
Tan	1	94132501	94132800	0.265	0.725	0.510	0.497
Tan	1	95144401	95144700	0.079	0.324	0.078	0.553
Tan	1	96137701	96138000	0.131	0.465	0.082	0.051
Tan	1	96219601	96219900	0.321	0.800	0.000	0.365
Tan	1	96284101	96284400	0.450	0.140	0.419	0.522
Tan	1	96346201	96346500	0.106	0.586	0.116	0.137
Tan	1	96375301	96375600	0.240	0.517	0.189	0.254
Tan	1	96375601	96375900	0.294	0.678	0.218	0.248
Tan	1	96654601	96654900	0.215	0.442	0.078	0.218
Tan	1	96667501	96667800	0.344	0.678	0.243	0.218
Tan	1	96702901	96703200	0.294	0.560	0.239	0.441
Tan	1	96715501	96715800	0.560	0.910	0.390	0.446
Tan	1	96795901	96796200	0.131	0.890	0.127	0.086
Tan	1	96796201	96796500	0.079	0.792	0.111	0.187
Tan	1	96829201	96829500	0.345	0.466	0.823	0.360
Tan	1	97270801	97271100	0.371	0.560	0.185	0.771
Tan	1	97322401	97322700	0.187	0.350	0.502	0.193
Tan	1	97417501	97417800	0.106	0.586	0.160	0.248
Tan	1	97650301	97650600	0.720	0.489	1.280	0.771
Tan	1	97758301	97758600	0.294	1.244	0.330	0.568
Tan	1	97758601	97758900	0.186	0.940	0.330	0.391
Tan	1	97772101	97772400	0.265	0.515	0.082	0.187
Tan	1	98409901	98410200	0.587	0.936	0.312	0.421
Tan	1	98674201	98674500	0.586	1.165	0.881	0.472
Tan	1	98688301	98688600	0.319	0.680	0.317	0.365
Tan	1	98790001	98790300	0.184	1.474	0.357	0.214
Tan	1	98864701	98865000	2.579	3.396	2.941	2.429
Tan	1	98886601	98886900	0.532	0.916	0.321	0.299
Tan	1	98957101	98957400	0.799	1.051	0.399	0.628
Tan	1	98964301	98964600	0.239	0.815	0.379	0.441
Tan	1	99438001	99438300	0.266	0.583	0.395	0.329
Tan	1	99559801	99560100	0.082	0.845	0.267	0.061
Tan	1	99560101	99560400	0.240	0.657	0.374	0.223
Tan	1	99629101	99629400	0.505	0.303	0.185	0.193
Tan	1	99684001	99684300	0.290	0.747	0.288	0.315
Tan	1	99729901	99730200	0.690	1.519	0.396	0.639
Tan	1	99736201	99736500	0.318	0.631	0.210	0.324
Tan	1	99822001	99822300	0.186	0.633	0.210	0.193
Tan	1	99856501	99856800	0.160	0.611	0.263	0.324
Tan	1	99857701	99858000	0.665	1.334	0.798	0.796
Tan	1	100156201	100156500	0.636	1.006	0.580	0.588
Tan	1	100169701	100170000	0.742	0.513	0.136	0.857
Tan	1	100386601	100386900	0.398	0.538	0.317	0.254
Tan	1	100539301	100539600	2.307	3.480	2.514	2.150

Tan	1	100714201	100714500	0.398	0.682	0.296	0.112
Tan	1	100941901	100942200	0.349	0.633	0.263	0.248
Tan	1	101463901	101464200	1.621	0.607	1.551	1.420
Tan	1	101466901	101467200	0.158	0.446	0.160	0.106
Tan	1	101492701	101493000	0.186	0.513	0.181	0.187
Tan	1	101502601	101502900	0.477	0.069	0.403	0.329
Tan	1	101680501	101680800	2.768	4.108	2.122	3.219
Tan	1	101975401	101975700	0.055	0.725	0.272	0.193
Tan	1	102264601	102264900	0.342	0.749	0.321	0.254
Tan	1	102264901	102265200	0.237	0.586	0.239	0.086
Tan	1	102281701	102282000	0.584	0.912	0.502	0.583
Tan	1	102287701	102288000	2.075	3.010	1.415	1.613
Tan	1	102398401	102398700	0.294	0.493	0.082	0.279
Tan	1	102434401	102434700	0.106	0.611	0.152	0.086
Tan	1	102517501	102517800	0.239	1.453	0.504	0.208
Tan	1	102578401	102578700	0.878	0.515	0.609	0.634
Tan	1	102636301	102636600	0.587	1.199	0.671	0.639
Tan	1	103170601	103170900	8.161	3.834	5.769	6.394
Tan	1	103183501	103183800	0.479	1.032	0.745	0.715
Tan	1	103270801	103271100	0.615	0.957	0.243	0.634
Tan	1	103282201	103282500	0.484	1.906	0.165	0.643
Tan	1	103335601	103335900	0.290	0.747	0.346	0.466
Tan	1	103399801	103400100	1.834	3.088	1.677	0.628
Tan	1	103400101	103400400	1.631	3.279	1.171	1.145
Tan	1	103523401	103523700	0.426	0.935	0.539	0.547
Tan	1	103562401	103562700	0.345	0.919	0.152	0.198
Tan	1	103614601	103614900	0.902	0.493	0.827	0.821
Tan	1	103706101	103706400	0.661	1.481	0.802	0.497
Tan	1	103826701	103827000	0.428	0.914	0.461	0.502
Tan	1	104524801	104525100	0.400	0.234	0.395	0.634
Tan	1	104664301	104664600	0.853	2.019	0.954	1.003
Tan	1	104727601	104727900	1.092	0.470	0.749	1.217
Tan	1	104809801	104810100	0.239	0.468	0.165	0.167
Tan	1	105009901	105010200	0.319	0.914	0.403	0.628
Tan	1	105063301	105063600	0.292	0.628	0.346	0.243
Tan	1	105376201	105376500	0.689	0.326	0.711	0.776
Tan	1	105720301	105720600	1.142	1.403	0.934	0.466
Tan	1	105771001	105771300	0.292	0.444	0.054	0.218
Tan	1	106349701	106350000	0.240	0.583	0.136	0.243
Tan	1	106607101	106607400	0.158	0.444	0.292	0.167
Tan	1	106858201	106858500	0.507	0.938	0.346	0.350
Tan	1	106977001	106977300	1.384	1.004	1.654	1.557
Tan	1	107048101	107048400	1.252	1.779	1.016	0.882
Tan	1	107309701	107310000	0.160	0.420	0.234	0.167
Tan	1	107674801	107675100	0.081	0.491	0.111	0.142
Tan	1	107843101	107843400	0.295	0.699	0.140	0.162
Tan	1	108435001	108435300	0.136	0.515	0.189	0.173
Tan	1	108462901	108463200	1.649	2.178	1.596	1.537
Tan	1	108653101	108653400	0.481	0.818	0.510	0.436
Tan	1	109012501	109012800	0.189	0.513	0.210	0.076
Tan	1	109235401	109235700	0.239	0.824	0.152	0.309
Tan	1	109413601	109413900	3.902	3.366	2.472	3.889
Tan	1	109427101	109427400	0.637	1.075	0.711	0.715
Tan	1	109472701	109473000	0.531	0.350	0.239	0.218
Tan	1	110076301	110076600	0.053	0.493	0.214	0.279
Tan	1	110093101	110093400	1.545	0.846	1.324	1.389
Tan	1	110985301	110985600	0.237	0.468	0.292	0.081
Tan	1	111140701	111141000	0.452	1.178	0.486	0.446
Tan	1	111438301	111438600	0.158	0.538	0.082	0.157
Tan	1	111510301	111510600	1.142	1.751	1.197	1.354
Tan	1	111716101	111716400	0.187	0.893	0.325	0.421
Tan	1	111732901	111733200	0.000	0.491	0.049	0.076
Tan	1	111784501	111784800	0.239	0.745	0.214	0.208
Tan	1	111784801	111785100	0.215	0.513	0.210	0.248
Tan	1	111791101	111791400	0.374	0.676	0.263	0.268
Tan	1	111819601	111819900	5.051	2.336	5.257	4.847
Tan	1	111838501	111838800	0.292	0.468	0.263	0.274

Tan	1	111839101	111839400	1.384	2.285	1.453	1.496
Tan	1	111859201	111859500	2.847	3.574	1.514	2.301
Tan	1	111964201	111964500	0.319	0.397	0.531	0.553
Tan	1	112236301	112236600	0.957	1.540	0.707	0.553
Tan	1	112355701	112356000	0.213	0.493	0.054	0.106
Tan	1	112388101	112388400	0.557	1.122	0.687	0.664
Tan	1	112895701	112896000	0.902	1.334	0.983	0.659
Tan	1	113199901	113200200	0.216	1.455	0.749	0.076
Tan	1	113631301	113631600	0.106	0.444	0.107	0.218
Tan	1	113869501	113869800	1.170	1.665	0.847	1.176
Tan	1	114095101	114095400	0.105	0.493	0.103	0.193
Tan	1	114403501	114403800	0.294	0.749	0.194	0.299
Tan	1	114623701	114624000	2.075	3.002	1.422	1.820
Tan	1	114711301	114711600	0.747	1.075	0.477	0.690
Tan	1	114804001	114804300	1.515	1.892	1.436	1.526
Tan	1	115139701	115140000	0.215	0.773	0.156	0.254
Tan	1	115230601	115230900	0.452	0.118	0.560	0.669
Tan	1	115288501	115288800	0.079	0.609	0.185	0.101
Tan	1	115467601	115467900	1.408	1.590	1.329	1.212
Tan	1	115815601	115815900	0.984	0.538	0.852	1.420
Tan	1	115942201	115942500	0.292	0.584	0.160	0.355
Tan	1	115987801	115988100	0.655	1.866	0.515	0.462
Tan	1	116025601	116025900	0.402	0.727	0.029	0.350
Tan	1	116265901	116266200	0.186	0.588	0.239	0.335
Tan	1	116286601	116286900	0.373	1.152	0.296	0.304
Tan	1	116376001	116376300	0.371	0.607	0.214	0.360
Tan	1	117407401	117407700	0.292	0.513	0.210	0.187
Tan	1	117504901	117505200	0.477	1.028	0.337	0.507
Tan	1	117653701	117654000	0.318	0.822	0.448	0.304
Tan	1	117750601	117750900	0.457	0.841	0.054	0.173
Tan	1	117788401	117788700	0.773	1.670	0.399	0.481
Tan	1	117800701	117801000	0.165	0.839	0.127	0.243
Tan	1	117816301	117816600	0.082	0.489	0.049	0.112
Tan	1	117877801	117878100	0.055	0.790	0.000	0.187
Tan	1	118164001	118164300	0.263	1.079	0.223	0.137
Tan	1	118395901	118396200	0.266	0.541	0.292	0.193
Tan	1	118575901	118576200	0.210	0.586	0.243	0.223
Tan	1	118892701	118893000	2.124	1.335	1.670	1.658
Tan	1	119121601	119121900	0.345	0.865	0.580	0.583
Tan	1	119121901	119122200	0.240	0.678	0.234	0.248
Tan	1	119268901	119269200	0.294	0.702	0.395	0.385
Tan	1	119593201	119593500	0.292	0.584	0.346	0.299
Tan	1	119597401	119597700	1.168	1.914	1.308	1.044
Tan	1	119617801	119618100	3.994	2.176	3.170	3.655
Tan	1	119687401	119687700	0.637	0.957	0.593	0.583
Tan	1	119719501	119719800	0.136	0.515	0.267	0.274
Tan	1	119759101	119759400	1.381	2.040	1.168	1.516
Tan	1	119871001	119871300	0.934	1.573	0.901	0.948
Tan	1	119910901	119911200	1.437	1.706	1.251	2.028
Tan	1	120288001	120288300	0.106	0.491	0.189	0.193
Tan	1	120457501	120457800	6.719	4.829	6.043	6.870
Tan	1	120477001	120477300	0.577	1.878	0.638	0.832
Tan	1	120681601	120681900	0.373	0.611	0.181	0.081
Tan	1	120706801	120707100	2.016	1.056	1.489	1.577
Tan	1	120758101	120758400	0.611	0.935	0.350	0.507
Tan	1	120831001	120831300	3.049	4.458	2.751	2.642
Tan	1	120838501	120838800	0.053	0.418	0.054	0.137
Tan	1	120860401	120860700	2.925	2.105	3.263	3.163
Tan	1	120875401	120875700	0.398	0.725	0.189	0.162
Tan	1	120905101	120905400	2.149	1.201	2.620	3.595
Tan	1	120991801	120992100	1.223	1.569	0.843	1.141
Tan	1	121043401	121043700	0.292	0.704	0.312	0.223
Tan	1	121901701	121902000	0.371	0.916	0.457	0.315
Tan	1	122190301	122190600	0.345	0.891	0.531	0.446
Tan	1	122284801	122285100	0.955	0.258	0.881	0.664
Tan	1	122357701	122358000	0.134	0.796	0.054	0.147
Tan	1	122358001	122358300	0.108	0.612	0.078	0.122

Tan	1	122941501	122941800	0.244	0.863	0.058	0.391
Tan	1	123586801	123587100	0.665	1.116	0.374	0.715
Tan	1	123616201	123616500	0.294	0.818	0.243	0.360
Tan	1	123997201	123997500	0.344	0.023	0.374	0.527
Tan	1	123997501	123997800	1.066	0.163	1.268	1.424
Tan	1	124221001	124221300	0.215	0.468	0.160	0.304
Tan	1	124490401	124490700	0.134	0.423	0.082	0.167
Tan	1	124496401	124496700	0.965	1.927	0.881	0.846
Tan	1	124506901	124507200	0.266	0.536	0.243	0.274
Tan	1	125325001	125325300	0.211	0.744	0.263	0.309
Tan	1	125341201	125341500	3.060	1.682	2.361	2.469
Tan	1	125741401	125741700	0.110	1.152	0.382	0.421
Tan	1	125960701	125961000	0.294	0.564	0.156	0.137
Tan	1	125978101	125978400	0.610	0.303	0.555	0.608
Tan	1	126531001	126531300	0.295	0.747	0.123	0.360
Tan	1	127190401	127190700	0.749	1.525	0.881	0.796
Tan	1	127554001	127554300	0.211	0.513	0.370	0.248
Tan	1	127600801	127601100	0.132	0.775	0.448	0.522
Tan	1	128359501	128359800	0.611	0.421	0.292	0.360
Tan	1	128366101	128366400	2.073	2.624	1.908	1.627
Tan	1	128609701	128610000	0.797	1.174	0.609	0.771
Tan	1	128715001	128715300	0.268	0.609	0.350	0.254
Tan	1	128882701	128883000	0.805	2.274	1.251	1.348
Tan	1	128939101	128939400	0.266	0.466	0.189	0.304
Tan	1	128952001	128952300	0.453	0.749	0.312	0.441
Tan	1	129003001	129003300	1.010	2.334	1.340	1.238
Tan	1	129073501	129073800	0.213	0.818	0.432	0.380
Tan	1	129171601	129171900	0.079	0.420	0.103	0.086
Tan	1	129274201	129274500	0.265	0.701	0.346	0.198
Tan	1	129878701	129879000	0.239	0.470	0.243	0.248
Tan	1	130520701	130521000	1.197	0.513	0.638	0.695
Tan	1	131478301	131478600	0.532	0.305	0.695	0.634
Tan	1	131630101	131630400	0.824	0.399	0.823	0.243
Tan	1	131692801	131693100	0.852	1.311	0.745	0.405
Tan	1	131697301	131697600	0.077	0.425	0.025	0.081
Tan	1	132654301	132654600	0.477	0.234	0.049	0.051
Tan	1	133293001	133293300	0.186	0.446	0.214	0.223
Tan	1	134096401	134096700	0.134	0.442	0.127	0.132
Tan	1	134158801	134159100	0.053	0.401	0.185	0.112
Tan	1	136163701	136164000	0.532	0.981	0.667	0.385
Tan	1	136619101	136619400	0.105	0.513	0.111	0.329
Tan	1	137005801	137006100	0.426	0.699	0.111	0.187
Tan	1	137865301	137865600	0.640	2.077	0.700	1.389
Tan	1	137960101	137960400	0.134	0.448	0.058	0.142
Tan	1	139530601	139530900	1.477	2.667	1.476	0.816
Tan	1	139946701	139947000	0.161	0.794	0.321	0.411
Tan	1	140189701	140190000	0.132	0.328	0.189	0.522
Tan	1	141167401	141167700	0.636	0.936	0.506	0.187
Tan	1	141592201	141592500	0.690	0.423	0.239	0.639
Tan	1	141821401	141821700	0.852	0.562	0.424	0.472
Tan	1	141924301	141924600	0.508	0.257	0.774	0.578
Tan	1	142563301	142563600	0.376	0.940	0.457	0.441
Tan	1	142749001	142749300	0.398	0.845	0.346	0.452
Tan	1	143477101	143477400	1.170	1.335	0.930	0.608
Tan	1	143498101	143498400	0.373	0.491	0.346	0.335
Tan	1	144270301	144270600	0.160	0.493	0.107	0.117
Tan	1	144329701	144330000	0.718	1.408	0.852	0.933
Tan	1	144761701	144762000	0.878	1.455	1.016	0.938
Tan	1	144817501	144817800	0.239	0.420	0.082	0.274
Tan	1	145202101	145202400	0.345	0.584	0.481	0.360
Tan	1	145226401	145226700	1.539	2.341	1.233	1.404
Tan	1	145471201	145471500	0.213	0.513	0.218	0.213
Tan	1	145939801	145940100	0.903	1.244	0.798	0.862
Tan	1	146226001	146226300	0.715	0.283	1.061	0.776
Tan	1	146308201	146308500	0.982	0.461	1.094	1.197
Tan	1	146811901	146812200	0.558	1.309	0.580	0.582
Tan	1	146942101	146942400	0.582	0.981	0.506	0.522

Tan	1	147616501	147616800	1.279	2.426	1.629	1.450
Tan	1	147700801	147701100	0.292	0.515	0.292	0.309
Tan	1	147871201	147871500	0.637	1.551	0.082	0.573
Tan	1	147871501	147871800	0.771	1.639	0.058	0.771
Tan	1	148375501	148375800	0.105	0.584	0.201	0.112
Tan	1	149469601	149469900	0.292	0.725	0.453	0.547
Tan	1	150809401	150809700	0.263	0.747	0.156	0.142
Tan	1	151393501	151393800	0.242	0.629	0.218	0.162
Tan	1	151947001	151947300	1.091	1.476	1.081	0.690
Tan	1	152769301	152769600	0.186	0.730	0.239	0.147
Tan	1	153024001	153024300	0.929	0.543	1.304	0.882
Tan	1	153041701	153042000	0.106	0.448	0.103	0.025
Tan	1	153090001	153090300	0.294	0.444	0.189	0.193
Tan	1	153646801	153647100	0.163	0.515	0.156	0.167
Tan	1	153877501	153877800	1.037	1.541	0.938	0.948
Tan	1	154018201	154018500	3.026	2.403	1.719	1.770
Tan	1	154593301	154593600	0.347	0.539	0.321	0.304
Tan	1	154689001	154689300	0.053	0.425	0.054	0.025
Tan	1	154949701	154950000	0.108	1.349	0.078	0.213
Tan	1	154950001	154950300	0.136	1.405	0.074	0.345
Tan	1	155370301	155370600	0.799	0.397	0.695	0.715
Tan	1	155479801	155480100	0.292	0.725	0.283	0.385
Tan	1	155670001	155670300	0.423	0.980	0.185	0.299
Tan	1	155897101	155897400	0.266	0.729	0.223	0.193
Tan	1	155996701	155997000	0.373	0.871	0.346	0.385
Tan	1	156494101	156494400	0.215	0.558	0.160	0.243
Tan	1	156777001	156777300	0.369	1.146	0.276	0.173
Tan	1	156809701	156810000	0.213	0.466	0.218	0.223
Tan	1	157158301	157158600	0.402	0.798	0.312	0.218
Tan	1	158206501	158206800	0.292	0.096	0.288	0.664
Tan	1	158757301	158757600	0.265	0.562	0.259	0.167
Tan	1	159552901	159553200	0.240	0.468	0.243	0.248
Tan	1	161063101	161063400	0.161	0.442	0.025	0.056
Tan	1	161615701	161616000	0.077	0.423	0.160	0.081
Tan	1	161629801	161630100	0.795	0.165	0.678	0.745
Tan	1	161631901	161632200	0.314	0.749	0.214	0.304
Tan	1	161764801	161765100	0.213	0.657	0.214	0.259
Tan	1	162180601	162180900	0.266	0.562	0.288	0.299
Tan	1	162191401	162191700	0.239	0.466	0.218	0.025
Tan	1	162533701	162534000	0.373	0.863	0.432	0.421
Tan	1	162890701	162891000	0.189	0.609	0.181	0.112
Tan	1	163152901	163153200	0.665	1.026	0.646	0.639
Tan	1	163177501	163177800	0.563	1.573	0.695	0.507
Tan	1	163412101	163412400	0.532	0.865	0.408	0.223
Tan	1	163717201	163717500	0.160	0.586	0.346	0.162
Tan	1	163831501	163831800	0.184	0.701	0.210	0.000
Tan	1	163936201	163936500	0.106	0.472	0.103	0.031
Tan	1	163936501	163936800	0.106	0.701	0.147	0.000
Tan	1	164613901	164614200	0.345	0.635	0.292	0.304
Tan	1	165101401	165101700	0.637	0.399	0.745	0.669
Tan	1	166423201	166423500	0.186	0.489	0.210	0.106
Tan	1	166726501	166726800	0.186	1.485	0.571	0.187
Tan	1	166978201	166978500	0.561	0.258	0.909	0.614
Tan	1	167153401	167153700	0.053	0.418	0.160	0.167
Tan	1	167188501	167188800	0.455	1.006	0.477	0.608
Tan	1	169209301	169209600	0.374	0.680	0.165	0.365
Tan	1	169403101	169403400	4.034	1.641	2.287	2.596
Tan	1	169705801	169706100	0.265	0.631	0.049	0.243
Tan	1	170490001	170490300	1.408	1.637	1.362	1.876
Tan	1	170607901	170608200	0.106	0.393	0.107	0.056
Tan	1	170633401	170633700	0.215	0.674	0.243	0.198
Tan	1	170640301	170640600	0.158	0.612	0.127	0.106
Tan	1	170933101	170933400	1.065	1.478	0.992	0.588
Tan	1	171155101	171155400	0.081	0.423	0.078	0.162
Tan	1	171202501	171202800	0.268	0.931	0.448	0.315
Tan	1	171300001	171300300	0.156	0.869	0.230	0.259
Tan	1	171480601	171480900	0.239	0.517	0.132	0.137

Tan	1	171509101	171509400	0.558	0.964	0.531	0.329
Tan	1	171775501	171775800	0.450	0.936	0.461	0.452
Tan	1	172213201	172213500	2.289	4.375	1.982	1.925
Tan	1	172240801	172241100	1.544	2.032	1.415	1.572
Tan	1	172439101	172439400	0.371	0.701	0.321	0.228
Tan	1	173904001	173904300	0.318	0.631	0.283	0.248
Tan	1	174681001	174681300	0.292	0.539	0.350	0.193
Tan	1	174920101	174920400	0.418	1.281	0.132	0.350
Tan	1	174922201	174922500	0.395	1.008	0.176	0.081
Tan	1	175103101	175103400	0.321	0.513	0.185	0.167
Tan	1	175158601	175158900	1.249	2.126	1.387	1.253
Tan	1	175158901	175159200	1.116	1.940	1.177	0.968
Tan	1	175345501	175345800	0.158	0.468	0.214	0.173
Tan	1	175345801	175346100	0.000	0.751	0.107	0.142
Tan	1	175381201	175381500	0.450	0.749	0.399	0.492
Tan	1	176047501	176047800	0.000	0.371	0.029	0.000
Tan	1	176047801	176048100	0.055	0.631	0.058	0.081
Tan	1	176244601	176244900	0.584	0.210	0.078	0.329
Tan	1	176906101	176906400	0.398	1.306	0.419	0.512
Tan	1	176948701	176949000	0.395	1.266	0.506	0.771
Tan	1	176998801	176999100	0.294	0.536	0.267	0.243
Tan	1	177021001	177021300	0.106	0.682	0.210	0.162
Tan	1	177088501	177088800	0.160	0.448	0.185	0.137
Tan	1	177142501	177142800	1.544	1.264	0.876	1.653
Tan	1	177645001	177645300	0.448	1.026	0.555	0.492
Tan	1	178531801	178532100	1.224	1.732	1.217	1.242
Tan	1	179010601	179010900	0.161	0.560	0.210	0.137
Tan	1	179176501	179176800	0.453	0.257	0.477	0.527
Tan	1	180169501	180169800	0.213	0.635	0.194	0.132
Tan	1	180180001	180180300	0.694	1.006	0.798	0.522
Tan	1	180307201	180307500	0.186	0.442	0.185	0.173
Tan	1	181158301	181158600	0.239	0.584	0.477	0.137
Tan	1	181829401	181829700	0.428	0.676	0.317	0.385
Tan	1	181989301	181989600	0.239	0.420	0.267	0.274
Tan	1	182119201	182119500	1.723	0.772	2.620	1.785
Tan	1	182453101	182453400	0.957	0.678	0.346	0.335
Tan	1	182627701	182628000	0.423	1.058	0.531	0.441
Tan	1	182702401	182702700	0.486	1.244	0.510	0.365
Tan	1	182968201	182968500	2.391	3.042	1.295	2.357
Tan	1	183503701	183504000	0.160	0.466	0.185	0.279
Tan	1	183586501	183586800	0.769	0.609	0.292	0.391
Tan	1	184334701	184335000	1.513	2.778	1.509	2.059
Tan	1	184398001	184398300	0.186	0.820	0.457	0.243
Tan	1	184429801	184430100	0.424	0.798	0.453	0.309
Tan	1	185208301	185208600	0.239	0.841	0.386	0.329
Tan	1	185318401	185318700	0.132	0.444	0.210	0.243
Tan	1	185504701	185505000	1.776	0.983	1.799	2.048
Tan	1	185588701	185589000	0.239	0.730	0.132	0.335
Tan	1	185600101	185600400	0.428	0.839	0.370	0.340
Tan	1	185600401	185600700	0.455	1.238	0.468	0.416
Tan	1	185721001	185721300	2.450	4.106	2.620	2.566
Tan	1	186470401	186470700	0.908	1.800	0.738	1.080
Tan	1	186738901	186739200	0.266	0.605	0.267	0.284
Tan	1	186758701	186759000	0.450	0.981	0.424	0.720
Tan	1	186822301	186822600	1.011	0.631	0.531	0.441
Tan	1	187237801	187238100	0.398	0.704	0.288	0.187
Tan	1	187416301	187416600	0.398	0.586	0.374	0.081
Tan	1	187430101	187430400	0.161	0.517	0.156	0.117
Tan	1	187595101	187595400	0.160	0.536	0.292	0.254
Tan	1	187600801	187601100	0.132	0.395	0.160	0.132
Tan	1	187703401	187703700	2.738	3.664	2.354	2.307
Tan	1	187832101	187832400	0.186	0.373	0.535	0.162
Tan	1	187941001	187941300	0.398	0.817	0.276	0.213
Tan	1	188232001	188232300	0.292	0.517	0.156	0.142
Tan	1	188434201	188434500	0.268	1.384	0.568	0.381
Tan	1	188579701	188580000	0.103	0.444	0.132	0.167
Tan	1	189685501	189685800	0.103	0.446	0.107	0.137

Tan	1	189759601	189759900	0.053	0.841	0.317	0.183
Tan	1	189949501	189949800	0.189	0.633	0.267	0.329
Tan	1	190074601	190074900	0.932	1.262	0.860	0.502
Tan	1	190167601	190167900	0.931	1.860	0.575	0.751
Tan	1	190315801	190316100	0.349	0.678	0.185	0.446
Tan	1	190369801	190370100	0.426	0.772	0.049	0.355
Tan	1	190370101	190370400	0.979	2.133	0.823	1.217
Tan	1	190461601	190461900	0.026	0.448	0.132	0.162
Tan	1	190836901	190837200	0.294	0.701	0.325	0.299
Tan	1	191103901	191104200	0.768	1.306	0.624	0.329
Tan	1	191450701	191451000	1.644	0.835	1.616	1.566
Tan	1	191492401	191492700	0.350	0.815	0.321	0.355
Tan	1	191552401	191552700	0.105	0.513	0.234	0.056
Tan	1	191583001	191583300	0.558	0.794	0.481	0.522
Tan	1	191662801	191663100	0.187	0.586	0.185	0.304
Tan	1	191702401	191702700	0.611	0.796	0.346	0.603
Tan	1	191860801	191861100	1.353	0.695	1.501	1.901
Tan	1	191907901	191908200	0.690	0.446	0.720	0.974
Tan	1	192798601	192798900	1.063	1.262	0.905	0.852
Tan	1	192807301	192807600	0.347	0.908	0.390	0.360
Tan	1	193123501	193123800	0.184	0.583	0.185	0.268
Tan	1	194071501	194071800	0.189	0.656	0.165	0.218
Tan	1	194345101	194345400	0.986	0.348	0.613	0.634
Tan	1	195059101	195059400	1.089	0.676	0.453	0.887
Tan	1	196351201	196351500	0.161	0.446	0.074	0.106
Tan	1	196632601	196632900	0.373	0.727	0.350	0.391
Tan	1	196646101	196646400	0.369	0.869	0.234	0.137
Tan	1	196944901	196945200	0.239	0.470	0.078	0.223
Tan	1	197017201	197017500	0.295	0.607	0.239	0.167
Tan	1	197354701	197355000	0.261	0.729	0.263	0.137
Tan	1	197452501	197452800	0.345	0.697	0.156	0.335
Tan	1	197496601	197496900	2.126	2.620	2.189	1.901
Tan	1	197649001	197649300	0.295	0.701	0.341	0.268
Tan	1	197665801	197666100	0.318	0.515	0.136	0.142
Tan	1	197718001	197718300	0.589	1.247	0.346	0.416
Tan	1	197801701	197802000	0.426	0.702	0.341	0.304
Tan	1	197874901	197875200	0.398	0.678	0.390	0.279
Tan	1	197882101	197882400	0.266	0.654	0.312	0.284
Tan	1	197893201	197893500	1.066	0.515	1.634	1.004
Tan	1	198209701	198210000	0.610	0.281	0.716	0.700
Tan	1	198460801	198461100	0.823	0.185	0.633	0.695
Tan	1	198837901	198838200	0.027	0.635	0.267	0.324
Tan	1	199064401	199064700	4.538	3.594	4.887	4.852
Tan	1	199226401	199226700	0.106	0.489	0.127	0.117
Tan	1	199295101	199295400	0.239	0.466	0.243	0.274
Tan	1	199437301	199437600	0.079	0.515	0.243	0.076
Tan	1	199679101	199679400	0.481	1.082	0.374	0.385
Tan	1	199751101	199751400	0.690	1.028	0.827	0.659
Tan	1	200058301	200058600	1.594	2.315	1.449	1.323
Tan	1	200268001	200268300	0.347	0.491	0.370	0.112
Tan	1	200460601	200460900	0.131	0.745	0.252	0.187
Tan	1	200807101	200807400	0.266	0.888	0.350	0.112
Tan	1	200908801	200909100	0.239	0.444	0.370	0.248
Tan	1	200998201	200998500	0.984	1.783	0.794	1.328
Tan	1	201144301	201144600	0.769	0.421	0.814	0.999
Tan	1	201199501	201199800	0.239	0.491	0.292	0.117
Tan	1	201286501	201286800	0.134	0.562	0.160	0.223
Tan	1	201378301	201378600	0.639	1.195	0.691	0.791
Tan	1	201546301	201546600	0.321	0.701	0.453	0.112
Tan	1	201630301	201630600	5.430	4.555	5.685	5.800
Tan	1	201630601	201630900	6.882	4.344	5.971	6.450
Tan	1	201932401	201932700	0.424	0.910	0.432	0.436
Tan	1	202197901	202198200	0.240	0.657	0.267	0.203
Tan	1	202444201	202444500	0.294	0.609	0.399	0.385
Tan	1	202734001	202734300	0.211	0.650	0.218	0.106
Tan	1	203004601	203004900	0.213	0.583	0.341	0.137
Tan	1	203028901	203029200	0.160	0.446	0.181	0.167

Tan	1	203582101	203582400	0.505	0.933	0.580	0.553
Tan	1	203688901	203689200	0.477	0.815	0.341	0.522
Tan	1	204359401	204359700	0.132	0.446	0.107	0.173
Tan	1	204757801	204758100	0.266	0.491	0.263	0.081
Tan	1	204843001	204843300	0.292	0.491	0.613	0.304
Tan	1	205043101	205043400	0.239	0.468	0.107	0.299
Tan	1	205335601	205335900	0.369	0.871	0.214	0.254
Tan	1	205699501	205699800	0.829	1.963	1.300	1.075
Tan	1	205946401	205946700	0.266	0.635	0.185	0.137
Tan	1	206048401	206048700	1.192	1.890	1.144	1.003
Tan	1	206272801	206273100	0.345	0.751	0.107	0.112
Tan	1	206756401	206756700	0.211	0.047	0.588	0.248
Tan	1	207252301	207252600	0.211	0.491	0.205	0.162
Tan	1	207455701	207456000	0.397	0.749	0.424	0.365
Tan	1	207505501	207505800	1.094	1.687	0.987	0.791
Tan	1	207542701	207543000	0.505	0.328	0.239	0.167
Tan	1	207690901	207691200	0.158	0.583	0.218	0.243
Tan	1	207777301	207777600	0.397	0.822	0.296	0.274
Tan	1	207943801	207944100	0.132	0.446	0.054	0.162
Tan	1	209015701	209016000	0.160	0.442	0.058	0.112
Tan	1	209713501	209713800	0.797	0.607	0.506	0.720
Tan	1	211090501	211090800	0.211	0.444	0.267	0.106
Tan	1	211203601	211203900	0.158	0.661	0.107	0.198
Tan	1	211218901	211219200	0.132	0.536	0.181	0.254
Tan	1	211810501	211810800	0.266	0.558	0.160	0.213
Tan	1	211973101	211973400	0.292	0.468	0.214	0.243
Tan	1	212168701	212169000	0.555	1.193	0.555	0.481
Tan	1	212199901	212200200	0.452	1.030	0.283	0.598
Tan	1	212208601	212208900	0.106	0.420	0.136	0.248
Tan	1	212495401	212495700	0.269	0.790	0.292	0.056
Tan	1	212924101	212924400	0.373	0.631	0.325	0.299
Tan	1	213216901	213217200	0.292	0.702	0.230	0.274
Tan	1	213281101	213281400	0.347	0.704	0.210	0.279
Tan	1	213385201	213385500	0.823	1.878	0.963	0.893
Tan	1	213385501	213385800	0.371	1.071	0.535	0.461
Tan	1	213391801	213392100	0.371	0.745	0.424	0.405
Tan	1	213428701	213429000	0.079	0.446	0.078	0.254
Tan	1	213459901	213460200	0.715	1.199	0.292	0.218
Tan	1	213982501	213982800	0.266	0.420	0.181	0.608
Tan	1	214115101	214115400	0.644	1.382	0.526	0.852
Tan	1	214320901	214321200	1.278	2.435	0.836	1.202
Tan	1	214349101	214349400	0.661	1.219	0.526	0.405
Tan	1	214863601	214863900	0.268	0.539	0.267	0.243
Tan	1	215028301	215028600	0.953	0.326	1.226	0.801
Tan	1	215350801	215351100	0.215	0.538	0.160	0.335
Tan	1	215489401	215489700	0.611	0.165	0.399	0.826
Tan	1	215563801	215564100	0.584	0.865	0.691	0.583
Tan	1	215662201	215662500	0.531	0.397	0.267	0.304
Tan	1	215766001	215766300	0.240	0.684	0.185	0.304
Tan	1	215766301	215766600	0.158	0.494	0.132	0.218
Tan	1	215786101	215786400	0.503	0.770	0.292	0.527
Tan	1	216005701	216006000	2.207	2.806	1.986	2.007
Tan	1	216563101	216563400	0.132	0.442	0.058	0.142
Tan	1	217436401	217436700	0.053	0.255	0.054	0.492
Tan	1	217526701	217527000	0.027	0.611	0.160	0.198
Tan	1	217874401	217874700	2.171	3.062	1.188	2.347
Tan	1	217985401	217985700	0.876	0.607	0.798	0.441
Tan	1	218519701	218520000	0.450	0.863	0.510	0.527
Tan	1	219116401	219116700	0.561	1.098	0.695	1.415
Tan	1	219221101	219221400	0.347	0.187	0.370	0.466
Tan	1	219801901	219802200	0.292	0.935	0.457	0.436
Tan	1	220222201	220222500	0.294	0.701	0.292	0.243
Tan	1	221031001	221031300	0.108	0.446	0.156	0.081
Tan	1	221289901	221290200	0.215	0.749	0.283	0.411
Tan	1	221386801	221387100	0.373	1.053	0.564	0.796
Tan	1	224053801	224054100	0.136	0.562	0.103	0.157
Tan	1	224442001	224442300	0.426	0.538	0.239	0.391

Tan	1	224452201	224452500	0.294	0.862	0.136	0.335
Tan	1	225030301	225030600	0.319	0.562	0.453	0.441
Tan	1	225081001	225081300	0.211	0.444	0.189	0.187
Tan	1	225482101	225482400	2.839	1.753	2.628	2.272
Tan	1	225593701	225594000	1.200	2.320	0.938	1.375
Tan	1	225602401	225602700	1.008	1.849	1.041	1.471
Tan	1	225779701	225780000	0.423	0.938	0.408	0.492
Tan	1	227269801	227270100	0.292	0.802	0.370	0.304
Tan	1	227331601	227331900	0.081	0.515	0.049	0.193
Tan	1	227375401	227375700	0.428	1.002	0.522	1.354
Tan	1	227575501	227575800	1.171	0.845	1.193	1.192
Tan	1	227961001	227961300	0.292	0.513	0.218	0.279
Tan	1	228543601	228543900	0.237	0.682	0.132	0.203
Tan	1	229152901	229153200	0.692	0.185	0.481	0.502
Tan	1	229188601	229188900	0.452	0.841	0.239	0.360
Tan	1	229469701	229470000	0.534	1.377	0.646	0.938
Tan	1	229880701	229881000	0.131	0.420	0.103	0.167
Tan	1	229973701	229974000	0.289	0.796	0.247	0.324
Tan	1	230451301	230451600	1.965	1.332	1.732	1.881
Tan	1	231379201	231379500	0.235	1.105	0.526	0.360
Tan	1	231379501	231379800	0.184	0.749	0.366	0.274
Tan	1	231508201	231508500	0.239	0.491	0.127	0.051
Tan	1	231576601	231576900	0.290	0.562	0.317	0.254
Tan	1	232221901	232222200	1.037	1.238	0.963	1.496
Tan	1	232701001	232701300	0.053	0.420	0.029	0.167
Tan	1	232771501	232771800	0.850	1.120	0.667	0.669
Tan	1	232998301	232998600	0.398	0.983	0.448	0.644
Tan	1	233084701	233085000	0.131	0.466	0.132	0.117
Tan	1	233355001	233355300	0.027	0.652	0.152	0.086
Tan	1	235284301	235284600	0.055	0.444	0.160	0.081
Tan	1	235410001	235410300	0.424	0.798	0.205	0.472
Tan	1	235431901	235432200	0.766	1.588	0.687	0.604
Tan	1	235765201	235765500	0.319	0.421	0.185	0.497
Tan	1	236025301	236025600	0.294	0.562	0.189	0.193
Tan	1	236235901	236236200	0.132	0.564	0.214	0.051
Tan	1	236713201	236713500	0.187	0.539	0.267	0.268
Tan	1	237087001	237087300	0.557	0.818	0.288	0.391
Tan	1	237241501	237241800	0.611	1.536	0.798	1.044
Tan	1	237290401	237290700	0.668	1.821	1.086	1.318
Tan	1	237390301	237390600	0.132	0.493	0.107	0.274
Tan	1	238503001	238503300	0.345	0.749	0.428	0.218
Tan	1	238503301	238503600	1.213	3.049	1.601	1.227
Tan	1	238839001	238839300	0.479	0.747	0.638	0.466
Tan	1	238903201	238903500	0.800	1.448	1.748	0.806
Tan	1	239013601	239013900	0.426	0.560	0.160	0.329
Tan	1	239206501	239206800	0.266	0.586	0.214	0.254
Tan	1	239349901	239350200	0.160	0.423	0.136	0.218
Tan	1	240069301	240069600	0.136	0.468	0.103	0.142
Tan	1	241424101	241424400	0.321	0.822	0.127	0.380
Tan	1	241424401	241424700	0.294	0.747	0.054	0.167
Tan	1	242287501	242287800	0.374	0.629	0.296	0.218
Tan	1	242465101	242465400	5.204	2.581	5.811	5.891
Tan	1	242971801	242972100	0.134	0.395	0.165	0.137
Tan	1	243246601	243246900	0.106	0.588	0.214	0.274
Tan	1	243332101	243332400	0.160	0.446	0.189	0.132
Tan	1	243432601	243432900	0.424	0.583	0.370	0.887
Tan	1	243520501	243520800	0.318	1.444	0.359	0.583
Tan	1	243526201	243526500	0.347	1.118	0.457	0.436
Tan	1	243594301	243594600	1.710	2.600	0.439	0.765
Tan	1	243675601	243675900	0.108	0.513	0.243	0.279
Tan	1	243970201	243970500	1.302	1.680	1.135	1.247
Tan	1	244022401	244022700	1.092	0.352	0.954	0.988
Tan	1	244270801	244271100	0.213	0.303	0.506	0.497
Tan	1	244811701	244812000	0.187	0.725	0.366	0.238
Tan	1	244950301	244950600	0.292	0.824	0.321	0.329
Tan	1	245130001	245130300	0.158	0.586	0.370	0.243
Tan	1	245194501	245194800	0.266	0.588	0.160	0.056

Tan	1	245214001	245214300	0.184	0.534	0.136	0.167
Tan	1	245223901	245224200	0.266	0.588	0.243	0.106
Tan	1	245345401	245345700	0.984	0.652	1.139	1.050
Tan	1	245361901	245362200	3.489	1.776	2.829	2.621
Tan	1	245582101	245582400	0.160	0.536	0.160	0.279
Tan	1	245582701	245583000	0.187	0.729	0.185	0.335
Tan	1	245658601	245658900	0.186	0.539	0.288	0.198
Tan	1	246499501	246499800	1.884	1.120	1.752	2.034
Tan	1	246572701	246573000	0.240	0.727	0.160	0.173
Tan	1	246621601	246621900	0.210	0.536	0.185	0.218
Tan	1	246758101	246758400	0.055	0.539	0.176	0.162
Tan	1	246906001	246906300	0.161	0.511	0.000	0.142
Tan	1	247473001	247473300	0.158	0.538	0.374	0.279
Tan	1	247583101	247583400	0.186	0.652	0.296	0.284
Tan	1	247600801	247601100	0.106	0.633	0.098	0.157
Tan	1	247693501	247693800	1.087	0.466	0.996	1.004
Tan	1	248078101	248078400	0.131	0.871	0.276	0.304
Tan	1	248097901	248098200	2.000	3.706	1.687	1.987
Tan	1	248098201	248098500	1.654	2.910	1.139	1.562
Tan	1	248441701	248442000	0.105	0.420	0.058	0.167
Tan	1	248751301	248751600	0.186	0.491	0.296	0.162
Tan	1	248772301	248772600	0.316	1.099	0.539	0.567
Tan	1	248812801	248813100	0.290	0.635	0.185	0.112
Tan	1	248912401	248912700	2.950	3.480	2.838	3.006
Tan	1	248983801	248984100	0.457	1.171	0.486	0.481
Tan	1	250202101	250202400	0.424	2.291	0.539	0.507
Tan	1	250233001	250233300	0.531	0.257	0.506	0.446
Tan	1	250457401	250457700	0.266	0.489	0.107	0.167
Tan	1	250565101	250565400	0.903	0.611	0.905	0.826
Tan	1	251425801	251426100	0.242	0.773	0.341	0.472
Tan	1	252360901	252361200	0.398	1.498	0.214	0.461
Tan	1	252361201	252361500	0.161	1.152	0.049	0.238
Tan	1	252426301	252426600	1.086	2.637	1.741	1.942
Tan	1	252489601	252489900	0.347	0.843	0.189	0.452
Tan	1	252599701	252600000	0.453	1.099	0.395	0.344
Tan	1	252625801	252626100	0.318	0.704	0.176	0.274
Tan	1	252754801	252755100	0.266	0.165	0.477	0.279
Tan	1	253172701	253173000	0.376	1.105	0.531	0.329
Tan	1	253415101	253415400	0.294	0.586	0.189	0.218
Tan	1	253419301	253419600	0.156	0.916	0.165	0.112
Tan	1	253615801	253616100	0.345	0.187	0.292	0.527
Tan	1	254091301	254091600	1.202	0.631	1.197	1.222
Tan	1	254096101	254096400	0.771	0.890	0.424	0.466
Tan	1	254157001	254157300	0.081	0.420	0.136	0.218
Tan	1	254471101	254471400	0.426	0.187	0.506	0.365
Tan	1	255019501	255019800	0.131	0.635	0.181	0.223
Tan	1	255096001	255096300	0.079	0.468	0.156	0.142
Tan	1	255394201	255394500	0.507	0.702	0.453	0.329
Tan	1	255493201	255493500	0.450	0.049	0.535	0.385
Tan	1	255670801	255671100	0.639	0.985	0.617	0.497
Tan	1	255819901	255820200	1.036	1.614	0.892	0.745
Tan	1	256041901	256042200	0.769	0.232	0.963	0.522
Tan	1	256176601	256176900	0.979	1.523	0.633	0.751
Tan	1	256436701	256437000	5.887	4.111	4.891	5.253
Tan	1	256613701	256614000	0.689	1.328	0.658	0.649
Tan	1	257590501	257590800	0.857	1.452	0.555	0.765
Tan	1	257620201	257620500	2.739	4.605	2.131	2.638
Tan	1	257620501	257620800	0.398	0.955	0.292	0.628
Tan	1	257969701	257970000	0.131	0.749	0.145	0.051
Tan	1	257970001	257970300	0.026	0.639	0.058	0.000
Tan	1	257997301	257997600	1.726	0.583	1.514	1.597
Tan	1	258001801	258002100	0.103	0.466	0.103	0.061
Tan	1	258453301	258453600	0.160	0.446	0.185	0.223
Tan	1	258457801	258458100	0.026	0.848	0.058	0.112
Tan	1	259069201	259069500	1.626	2.641	1.222	1.740
Tan	1	259183801	259184100	0.265	0.562	0.341	0.162
Tan	1	259349701	259350000	0.237	0.936	0.350	0.193

Tan	1	259393801	259394100	0.345	0.772	0.486	0.309
Tan	1	259434601	259434900	0.081	0.449	0.078	0.031
Tan	1	259443301	259443600	0.318	0.187	0.346	0.634
Tan	1	259574701	259575000	2.415	0.820	2.620	2.019
Tan	1	259590001	259590300	1.544	1.755	1.275	2.013
Tan	1	259964701	259965000	1.328	2.487	1.752	1.369
Tan	1	260160601	260160900	0.532	0.702	0.424	0.416
Tan	1	260173501	260173800	0.079	0.590	0.156	0.198
Tan	1	260195101	260195400	0.507	0.845	0.399	0.421
Tan	1	260203801	260204100	0.132	0.515	0.214	0.187
Tan	1	260274901	260275200	0.131	0.678	0.189	0.178
Tan	1	260298001	260298300	0.290	0.539	0.156	0.167
Tan	1	260423101	260423400	5.054	3.607	5.354	5.303
Tan	1	260516701	260517000	0.290	0.654	0.111	0.309
Tan	1	260539201	260539500	0.453	0.654	0.243	0.360
Tan	1	260603401	260603700	1.887	0.940	1.567	1.552
Tan	1	260620801	260621100	0.537	1.356	0.417	0.426
Tan	1	260702401	260702700	5.106	3.733	5.845	4.787
Tan	1	260731801	260732100	1.545	2.270	1.485	1.562
Tan	1	260912101	260912400	0.424	0.891	0.267	0.132
Tan	1	261200101	261200400	0.213	0.586	0.234	0.198
Tan	1	261310201	261310500	1.091	1.285	0.695	0.751
Tan	1	261405001	261405300	4.364	2.390	4.011	3.012
Tan	1	261418801	261419100	0.903	1.731	1.016	0.989
Tan	1	261490201	261490500	0.821	1.424	0.588	0.882
Tan	1	261491101	261491400	0.611	1.307	0.412	0.608
Tan	1	261558001	261558300	0.400	0.680	0.370	0.497
Tan	1	261612601	261612900	1.170	0.678	0.510	1.080
Tan	1	261645301	261645600	0.584	0.208	0.399	0.385
Tan	1	261759001	261759300	0.160	0.468	0.214	0.218
Tan	1	261810001	261810300	1.086	1.935	0.966	1.064
Tan	1	261906301	261906600	0.582	0.957	0.160	0.446
Tan	1	261980101	261980400	1.041	1.401	0.749	0.771
Tan	1	261990601	261990900	0.081	0.628	0.160	0.259
Tan	1	262041301	262041600	0.216	0.751	0.098	0.243
Tan	1	262155901	262156200	3.104	1.003	3.208	4.073
Tan	1	262164901	262165200	0.402	1.116	0.303	0.329
Tan	1	262229401	262229700	2.975	2.315	3.538	3.884
Tan	1	262241401	262241700	1.384	2.341	1.112	1.202
Tan	1	262292701	262293000	0.347	0.493	0.185	0.248
Tan	1	262499701	262500000	1.305	2.442	1.291	1.506
Tan	1	262515001	262515300	0.505	0.161	0.481	0.517
Tan	1	262517701	262518000	1.676	2.414	0.959	1.618
Tan	1	262761601	262761900	0.611	0.303	0.613	0.466
Tan	1	262908001	262908300	0.373	0.491	0.267	0.279
Tan	1	262914901	262915200	0.186	0.491	0.156	0.223
Tan	1	263489401	263489700	0.290	0.702	0.185	0.142
Tan	1	263704801	263705100	0.926	1.620	0.745	0.963
Tan	1	263924701	263925000	0.637	0.139	0.638	0.841
Tan	1	263928001	263928300	0.186	0.420	0.049	0.218
Tan	1	264031201	264031500	0.639	0.560	0.638	0.857
Tan	1	264082501	264082800	0.055	0.446	0.156	0.248
Tan	1	264090601	264090900	1.273	1.970	1.135	1.131
Tan	1	264150601	264150900	0.213	0.779	0.181	0.137
Tan	1	264166201	264166500	4.070	2.106	4.492	3.859
Tan	1	264167101	264167400	0.903	1.950	0.863	0.873
Tan	1	264173401	264173700	0.929	2.013	0.914	0.837
Tan	1	264175801	264176100	2.005	4.336	2.403	2.397
Tan	1	264176101	264176400	1.877	3.042	1.670	2.008
Tan	1	264310501	264310800	0.265	0.073	0.691	0.416
Tan	1	264348001	264348300	2.102	1.253	2.209	2.312
Tan	1	264608101	264608400	0.344	0.607	0.181	0.162
Tan	1	264865801	264866100	0.155	0.699	0.087	0.000
Tan	1	265086301	265086600	1.274	1.963	1.308	1.277
Tan	1	265119301	265119600	1.837	4.549	1.823	2.870
Tan	1	266595601	266595900	8.365	3.054	8.350	8.244
Tan	1	266595901	266596200	3.504	1.515	3.583	3.781

Tan	1	266905801	266906100	0.739	1.496	0.950	0.751
Tan	1	266934601	266934900	0.186	0.584	0.239	0.335
Tan	1	267077701	267078000	0.321	0.777	0.210	0.254
Tan	1	267096301	267096600	0.721	0.348	0.876	0.765
Tan	1	267116101	267116400	0.453	1.152	0.517	0.355
Tan	1	267118501	267118800	3.539	2.615	3.698	3.215
Tan	1	267167701	267168000	0.134	0.395	0.107	0.112
Tan	1	267171601	267171900	0.187	0.517	0.136	0.218
Tan	1	267215401	267215700	0.266	0.682	0.210	0.385
Tan	1	267337501	267337800	0.211	0.491	0.160	0.274
Tan	1	267377101	267377400	0.052	0.328	0.560	0.167
Tan	1	267415501	267415800	0.292	0.654	0.181	0.162
Tan	1	267574501	267574800	0.344	0.609	0.214	0.279
Tan	1	267689401	267689700	0.053	0.656	0.288	0.182
Tan	1	267780901	267781200	0.213	0.584	0.189	0.238
Tan	1	267798301	267798600	0.744	0.983	0.720	0.751
Tan	1	267831301	267831600	0.744	1.143	0.740	0.781
Tan	1	267859801	267860100	0.187	0.539	0.103	0.162
Tan	1	268391701	268392000	3.722	2.414	3.792	3.502
Tan	1	268604401	268604700	0.105	0.567	0.078	0.106
Tan	1	268910101	268910400	0.560	1.150	0.560	0.594
Tan	1	268997101	268997400	0.986	0.283	1.255	1.014
Tan	1	269383801	269384100	0.373	0.210	0.000	0.466
Tan	1	269472301	269472600	1.779	1.075	2.628	1.780
Tan	1	269490901	269491200	0.953	2.688	0.727	1.213
Tan	1	269531101	269531400	2.773	4.819	2.307	2.931
Tan	1	269531401	269531700	0.398	0.871	0.457	0.329
Tan	1	269534701	269535000	0.419	1.105	0.160	0.117
Tan	1	269535001	269535300	0.419	2.244	0.308	0.198
Tan	1	269630401	269630700	3.083	4.229	2.358	2.980
Tan	1	270182401	270182700	0.424	0.860	0.156	0.436
Tan	1	270281701	270282000	1.462	2.219	0.927	1.415
Tan	1	270315601	270315900	10.481	9.123	10.384	11.848
Tan	1	270324301	270324600	1.605	3.931	1.379	1.267
Tan	1	270348001	270348300	0.373	0.444	0.531	0.664
Tan	1	270390001	270390300	0.268	0.704	0.317	0.193
Tan	1	270550501	270550800	0.318	0.538	0.156	0.385
Tan	1	270596701	270597000	0.342	0.884	0.292	0.360
Tan	1	270654601	270654900	0.795	1.287	0.667	0.913
Tan	1	270685501	270685800	0.131	0.564	0.210	0.173
Tan	1	270759901	270760200	0.213	0.517	0.082	0.223
Tan	1	270931801	270932100	0.240	0.420	0.185	0.198
Tan	1	271075201	271075500	0.132	0.465	0.136	0.056
Tan	1	271094101	271094400	0.240	0.581	0.127	0.137
Tan	1	271125901	271126200	0.637	0.888	0.555	0.502
Tan	1	271146301	271146600	3.007	2.281	3.357	2.992
Tan	1	271316101	271316400	0.426	0.702	0.160	0.294
Tan	1	271404301	271404600	0.774	1.547	0.901	1.105
Tan	1	271572901	271573200	0.482	0.981	0.296	0.340
Tan	1	271579801	271580100	3.131	4.019	2.682	2.957
Tan	1	271771501	271771800	0.184	0.511	0.214	0.081
Tan	1	271922401	271922700	0.453	0.846	0.448	0.497
Tan	1	272063701	272064000	0.611	0.983	0.374	0.391
Tan	1	272064001	272064300	0.398	0.747	0.234	0.274
Tan	1	272370301	272370600	0.453	0.140	0.604	0.477
Tan	1	272582101	272582400	0.318	0.775	0.263	0.299
Tan	1	272582401	272582700	0.263	0.729	0.292	0.198
Tan	1	272602801	272603100	0.503	1.034	0.453	0.553
Tan	1	272603701	272604000	2.705	4.452	1.871	2.165
Tan	1	272658001	272658300	0.395	1.073	0.361	0.268
Tan	1	272678701	272679000	0.052	0.448	0.103	0.081
Tan	1	272990701	272991000	2.604	3.557	2.673	2.915
Tan	1	273100501	273100800	0.292	0.674	0.263	0.223
Tan	1	273210601	273210900	0.026	0.517	0.189	0.117
Tan	1	273345001	273345300	3.399	4.119	2.887	3.458
Tan	1	273444301	273444600	0.560	1.030	0.526	0.486
Tan	1	273471001	273471300	0.534	0.820	0.399	0.466

Tan	1	273471301	273471600	0.848	1.774	0.831	0.750
Tan	1	273483601	273483900	0.637	1.543	0.506	1.100
Tan	1	273539401	273539700	0.452	0.680	0.428	0.274
Tan	1	273546301	273546600	0.134	0.584	0.156	0.173
Tan	1	273576001	273576300	0.424	0.775	0.346	0.416
Tan	1	273625501	273625800	0.957	1.360	0.885	0.751
Tan	1	273657001	273657300	6.364	4.999	5.994	4.072
Tan	1	273659701	273660000	0.132	0.607	0.205	0.279
Tan	1	273796501	273796800	1.776	2.721	1.587	1.653
Tan	1	273815701	273816000	0.134	0.491	0.259	0.137
Tan	1	273944101	273944400	0.182	0.609	0.218	0.193
Tan	1	274052101	274052400	0.105	0.466	0.049	0.248
Tan	1	274275901	274276200	1.037	2.225	1.371	0.700
Tan	1	274339501	274339800	1.955	4.274	2.089	2.910
Tan	1	274413001	274413300	0.477	0.656	0.403	0.167
Tan	1	274678801	274679100	2.901	1.208	2.989	3.336
Tan	1	274713301	274713600	4.093	2.450	4.082	3.890
Tan	1	274840201	274840500	0.898	2.004	1.291	1.278
Tan	1	275052301	275052600	0.584	2.335	0.466	0.710
Tan	1	275065201	275065500	0.821	0.393	0.794	1.273
Tan	1	275073901	275074200	0.428	0.747	0.354	0.248
Tan	1	275244901	275245200	0.529	0.796	0.374	0.522
Tan	1	275595001	275595300	2.131	1.633	2.321	2.216
Tan	10	134701	135000	0.823	1.380	0.580	0.432
Tan	10	327001	327300	0.055	0.395	0.136	0.056
Tan	10	705001	705300	0.558	0.963	0.296	0.365
Tan	10	956101	956400	0.213	0.420	0.263	0.304
Tan	10	1433701	1434000	0.026	0.420	0.058	0.056
Tan	10	1770901	1771200	0.502	1.399	0.419	0.299
Tan	10	1861801	1862100	0.534	0.749	0.477	0.472
Tan	10	1937701	1938000	0.055	0.517	0.214	0.117
Tan	10	2048101	2048400	0.448	1.030	0.477	0.553
Tan	10	2150101	2150400	0.611	0.281	0.794	0.167
Tan	10	2457601	2457900	0.402	0.727	0.370	0.385
Tan	10	2539801	2540100	0.421	0.916	0.239	0.365
Tan	10	2625001	2625300	5.258	2.317	4.133	4.189
Tan	10	2762401	2762700	1.984	3.234	1.266	1.003
Tan	10	2818201	2818500	2.468	0.657	3.232	2.307
Tan	10	2857201	2857500	0.958	1.334	0.847	0.963
Tan	10	3166801	3167100	0.160	1.200	0.481	0.426
Tan	10	3342901	3343200	0.428	1.259	0.506	0.725
Tan	10	3682801	3683100	0.371	0.729	0.399	0.279
Tan	10	4107901	4108200	0.240	0.468	0.160	0.076
Tan	10	5280001	5280300	1.168	0.114	0.720	0.699
Tan	10	5415301	5415600	0.374	0.865	0.510	0.218
Tan	10	8409901	8410200	0.266	0.723	0.189	0.299
Tan	10	8410201	8410500	0.186	0.607	0.160	0.268
Tan	10	9415501	9415800	0.132	0.371	0.078	0.025
Tan	10	9936601	9936900	0.639	1.105	0.588	0.502
Tan	10	10281901	10282200	0.321	0.680	0.292	0.391
Tan	10	10704301	10704600	0.210	1.427	0.382	0.445
Tan	10	10707901	10708200	0.077	0.491	0.025	0.086
Tan	10	10845901	10846200	0.213	0.584	0.078	0.162
Tan	10	10873201	10873500	0.240	0.654	0.263	0.309
Tan	10	10930801	10931100	0.584	1.146	0.588	0.507
Tan	10	10993801	10994100	0.586	1.266	0.531	0.542
Tan	10	11025601	11025900	0.274	1.403	0.419	0.086
Tan	10	11025901	11026200	0.271	1.191	0.254	0.076
Tan	10	11029801	11030100	0.079	1.124	0.160	0.593
Tan	10	11030101	11030400	0.027	1.193	0.127	0.476
Tan	10	11050201	11050500	1.807	2.920	2.018	1.845
Tan	10	11183701	11184000	0.484	2.334	1.109	0.425
Tan	10	11184001	11184300	0.321	0.796	0.613	0.360
Tan	10	11235301	11235600	0.134	0.603	0.263	0.162
Tan	10	11238901	11239200	0.237	0.936	0.522	0.492
Tan	10	11348701	11349000	0.186	0.538	0.156	0.203
Tan	10	11359201	11359500	0.215	0.539	0.058	0.198

Tan	10	11399401	11399700	0.053	0.629	0.107	0.076
Tan	10	11736601	11736900	1.300	2.130	0.930	0.933
Tan	10	11970901	11971200	1.063	1.502	0.749	0.913
Tan	10	11989501	11989800	2.973	4.746	3.999	3.793
Tan	10	12158101	12158400	0.105	0.420	0.111	0.076
Tan	10	12179101	12179400	0.187	0.444	0.210	0.223
Tan	10	12182401	12182700	0.213	0.493	0.136	0.218
Tan	10	12259201	12259500	0.987	1.824	0.827	1.085
Tan	10	12259501	12259800	0.929	1.753	0.909	1.064
Tan	10	12296101	12296400	3.356	5.250	2.038	2.326
Tan	10	12297001	12297300	0.160	0.562	0.078	0.218
Tan	10	12297601	12297900	0.426	0.633	0.239	0.243
Tan	10	12301801	12302100	0.134	0.491	0.160	0.223
Tan	10	12370801	12371100	0.106	0.446	0.103	0.061
Tan	10	12448201	12448500	0.369	0.818	0.390	0.162
Tan	10	12501901	12502200	1.089	3.386	1.317	1.120
Tan	10	12502201	12502500	0.424	0.989	0.395	0.492
Tan	10	13022101	13022400	0.345	0.818	0.473	0.583
Tan	10	13056001	13056300	1.029	3.737	1.199	1.096
Tan	10	13214101	13214400	1.441	1.892	1.373	0.938
Tan	10	13420201	13420500	0.106	0.656	0.160	0.106
Tan	10	13458301	13458600	0.613	1.051	0.457	0.720
Tan	10	13501501	13501800	0.395	1.197	0.515	0.436
Tan	10	13811101	13811400	0.213	0.444	0.189	0.112
Tan	10	13818301	13818600	0.558	0.961	0.432	0.416
Tan	10	13964101	13964400	8.793	6.696	8.192	9.851
Tan	10	14170201	14170500	0.610	1.081	0.662	0.446
Tan	10	14180401	14180700	3.455	3.015	2.702	2.155
Tan	10	14247601	14247900	1.520	2.343	1.337	0.953
Tan	10	14383801	14384100	0.186	0.420	0.214	0.132
Tan	10	14961601	14961900	0.634	1.191	0.551	0.441
Tan	10	14988001	14988300	0.027	0.444	0.136	0.000
Tan	10	15009001	15009300	0.319	0.983	0.593	0.421
Tan	10	15227701	15228000	0.718	1.051	0.448	0.329
Tan	10	15234301	15234600	0.452	0.609	0.448	0.391
Tan	10	15238501	15238800	0.186	1.807	0.205	0.086
Tan	10	15238801	15239100	0.103	1.120	0.074	0.000
Tan	10	15608701	15609000	0.426	1.103	0.535	0.568
Tan	10	15783301	15783600	0.957	1.873	0.609	0.628
Tan	10	15783601	15783900	1.082	2.560	1.090	1.511
Tan	10	15916801	15917100	0.319	0.702	0.350	0.421
Tan	10	16021801	16022100	0.319	0.513	0.103	0.198
Tan	10	16342201	16342500	2.765	2.971	3.217	2.819
Tan	10	16366801	16367100	1.087	0.348	1.159	0.751
Tan	10	16704901	16705200	0.295	0.745	0.160	0.248
Tan	10	16889701	16890000	0.186	0.444	0.185	0.193
Tan	10	16916101	16916400	2.817	3.394	2.965	2.383
Tan	10	16980601	16980900	1.800	3.812	1.235	1.725
Tan	10	17007301	17007600	0.639	1.210	0.468	0.385
Tan	10	17076901	17077200	0.610	1.311	0.366	0.761
Tan	10	17170501	17170800	0.186	0.536	0.029	0.254
Tan	10	18580501	18580800	0.263	0.656	0.189	0.335
Tan	10	18597301	18597600	0.266	0.633	0.234	0.309
Tan	10	18689701	18690000	0.769	1.188	0.691	0.765
Tan	10	19051801	19052100	0.240	0.560	0.132	0.198
Tan	10	19068601	19068900	0.318	0.747	0.403	0.360
Tan	10	19224601	19224900	0.931	0.678	0.925	0.918
Tan	10	19236601	19236900	0.319	1.049	0.633	0.335
Tan	10	19373401	19373700	0.610	0.069	0.481	0.812
Tan	10	19395001	19395300	3.431	4.070	3.041	3.418
Tan	10	19537501	19537800	0.292	0.517	0.160	0.218
Tan	10	19649101	19649400	0.265	0.560	0.074	0.162
Tan	10	19701901	19702200	0.215	1.004	0.386	1.582
Tan	10	19858201	19858500	0.294	0.560	0.395	0.106
Tan	10	19977901	19978200	0.742	1.326	0.769	0.852
Tan	10	20088601	20088900	0.187	0.493	0.136	0.081
Tan	10	20183101	20183400	0.240	0.538	0.243	0.329

Tan	10	20241901	20242200	0.690	0.328	0.560	0.223
Tan	10	20478601	20478900	0.690	1.262	0.544	0.756
Tan	10	20540101	20540400	0.290	0.936	0.194	0.086
Tan	10	20697601	20697900	1.036	1.536	0.930	1.080
Tan	10	21096001	21096300	1.600	0.629	1.634	1.546
Tan	10	21248401	21248700	1.058	0.187	1.023	1.992
Tan	10	21251101	21251400	0.055	0.466	0.267	0.137
Tan	10	21314401	21314700	0.079	0.584	0.132	0.061
Tan	10	21404101	21404400	1.357	2.246	1.387	1.926
Tan	10	21404401	21404700	1.460	4.212	1.907	3.265
Tan	10	21410401	21410700	0.665	0.959	0.325	0.776
Tan	10	21417001	21417300	1.319	3.877	1.843	2.242
Tan	10	21429601	21429900	0.453	1.197	0.354	0.462
Tan	10	21484801	21485100	1.731	1.290	1.826	2.185
Tan	10	21516601	21516900	0.560	1.290	0.671	0.497
Tan	10	21667501	21667800	1.412	2.478	1.226	1.460
Tan	10	21667801	21668100	0.239	1.972	0.366	0.404
Tan	10	21708001	21708300	0.984	1.216	0.633	1.105
Tan	10	21889201	21889500	0.424	1.193	0.263	0.786
Tan	10	21913801	21914100	0.184	0.420	0.082	0.112
Tan	10	22011901	22012200	0.163	0.753	0.098	0.193
Tan	10	22065001	22065300	0.215	0.697	0.087	0.157
Tan	10	22202401	22202700	2.446	1.850	2.461	2.545
Tan	10	22209901	22210200	2.287	3.038	2.205	2.201
Tan	10	22223401	22223700	3.068	1.534	3.230	2.961
Tan	10	22322401	22322700	1.065	1.656	1.061	0.913
Tan	10	22592701	22593000	0.344	2.137	0.473	0.635
Tan	10	22616401	22616700	1.142	2.573	1.739	1.633
Tan	10	22702501	22702800	0.160	0.375	0.613	0.187
Tan	10	23022301	23022600	2.334	1.377	1.975	2.373
Tan	10	23104801	23105100	0.239	0.466	0.082	0.223
Tan	10	23159701	23160000	0.716	4.087	1.800	1.343
Tan	10	23218801	23219100	6.600	4.097	6.806	7.708
Tan	10	23483401	23483700	0.345	0.678	0.194	0.162
Tan	10	23507401	23507700	0.211	0.798	0.054	0.081
Tan	10	23922601	23922900	0.663	1.150	0.428	0.628
Tan	10	24040501	24040800	0.186	0.727	0.317	0.238
Tan	10	24204901	24205200	0.053	0.371	0.074	0.051
Tan	10	24339901	24340200	0.265	0.375	0.560	0.497
Tan	10	24368101	24368400	0.106	0.583	0.185	0.162
Tan	10	24795901	24796200	0.213	0.541	0.210	0.106
Tan	10	24798301	24798600	0.132	0.444	0.292	0.137
Tan	10	25131901	25132200	0.081	0.493	0.058	0.142
Tan	10	25673101	25673400	0.158	0.491	0.288	0.112
Tan	10	25774501	25774800	0.640	0.305	0.823	0.654
Tan	10	25905301	25905600	0.344	0.657	0.341	0.360
Tan	10	25922101	25922400	3.591	2.613	3.306	3.311
Tan	10	25980001	25980300	1.989	3.076	1.690	1.795
Tan	10	25980301	25980600	0.555	2.544	0.531	0.903
Tan	10	26084101	26084400	0.292	0.493	0.136	0.137
Tan	10	26183701	26184000	0.345	0.794	0.419	0.461
Tan	10	26184001	26184300	0.532	1.236	0.760	0.740
Tan	10	26673601	26673900	0.665	1.030	0.613	0.679
Tan	10	26709901	26710200	1.360	0.656	1.866	1.582
Tan	10	26950801	26951100	0.240	0.562	0.210	0.228
Tan	10	26951101	26951400	0.160	0.539	0.176	0.173
Tan	10	27164701	27165000	0.482	0.912	0.234	0.218
Tan	10	27660901	27661200	0.290	0.719	0.210	0.193
Tan	10	27840601	27840900	0.186	0.493	0.189	0.228
Tan	10	27929401	27929700	0.268	0.725	0.234	0.329
Tan	10	28129801	28130100	0.371	0.725	0.419	0.254
Tan	10	28260001	28260300	3.421	5.042	3.558	4.142
Tan	10	28260301	28260600	0.694	0.912	0.317	0.608
Tan	10	28278601	28278900	0.187	0.517	0.132	0.137
Tan	10	28417501	28417800	0.186	0.607	0.132	0.391
Tan	10	28443601	28443900	1.625	0.910	1.489	2.297
Tan	10	28562401	28562700	0.137	0.702	0.185	0.178

Tan	10	28562701	28563000	0.110	0.890	0.160	0.086
Tan	10	28605301	28605600	1.031	1.824	0.515	1.136
Tan	10	28768501	28768800	0.398	0.772	0.448	0.472
Tan	10	28821001	28821300	1.329	2.147	1.262	1.451
Tan	10	28940701	28941000	0.160	0.607	0.145	0.051
Tan	10	29016601	29016900	6.811	8.760	6.649	6.899
Tan	10	29053201	29053500	0.186	0.607	0.218	0.254
Tan	10	29252401	29252700	0.481	0.961	0.374	0.446
Tan	10	29432701	29433000	0.369	0.869	0.481	0.304
Tan	10	29445601	29445900	0.187	0.420	0.189	0.056
Tan	10	29467801	29468100	0.131	0.444	0.165	0.000
Tan	10	29568601	29568900	0.268	0.564	0.292	0.218
Tan	10	29768401	29768700	0.582	0.957	0.584	0.578
Tan	10	29792401	29792700	0.186	0.328	0.210	0.527
Tan	10	29961301	29961600	0.711	2.167	0.573	0.533
Tan	10	29988601	29988900	0.318	0.583	0.399	0.335
Tan	10	30007801	30008100	1.808	0.980	1.558	1.410
Tan	10	30130501	30130800	0.287	0.914	0.210	0.263
Tan	10	30430201	30430500	0.106	0.633	0.074	0.243
Tan	10	30551101	30551400	0.656	1.547	0.662	0.426
Tan	10	30684901	30685200	0.902	1.759	0.457	0.892
Tan	10	30897001	30897300	0.106	0.916	0.288	0.533
Tan	10	30937201	30937500	1.947	3.043	1.904	1.634
Tan	10	31005001	31005300	0.720	1.219	0.798	0.628
Tan	10	31090501	31090800	1.061	1.783	0.979	0.766
Tan	10	31169701	31170000	0.318	1.221	0.350	0.365
Tan	10	31275301	31275600	0.132	0.493	0.214	0.137
Tan	10	31303201	31303500	0.345	0.586	0.321	0.187
Tan	10	31395001	31395300	2.157	1.311	2.176	2.002
Tan	10	31413001	31413300	0.479	1.075	0.716	0.497
Tan	10	31523101	31523400	1.170	0.935	0.695	0.664
Tan	10	31535401	31535700	0.824	0.212	0.564	0.502
Tan	10	31581901	31582200	0.106	0.466	0.239	0.385
Tan	10	31583701	31584000	0.106	0.489	0.058	0.167
Tan	10	31735501	31735800	0.187	0.446	0.127	0.162
Tan	10	31831501	31831800	0.187	0.395	0.185	0.106
Tan	10	31836301	31836600	0.215	0.513	0.239	0.304
Tan	10	31879501	31879800	0.505	0.232	0.346	0.583
Tan	10	32034301	32034600	0.695	1.242	0.497	0.695
Tan	10	32090101	32090400	0.823	1.525	0.515	0.923
Tan	10	32194501	32194800	0.163	0.536	0.111	0.137
Tan	10	32212801	32213100	0.186	0.446	0.218	0.025
Tan	10	32366701	32367000	0.637	1.633	0.825	0.771
Tan	10	32921401	32921700	0.292	1.261	0.346	0.538
Tan	10	32921701	32922000	0.081	0.841	0.136	0.228
Tan	10	32922301	32922600	0.426	0.092	0.366	0.553
Tan	10	33015601	33015900	0.373	0.584	0.296	0.279
Tan	10	33083701	33084000	0.371	0.747	0.432	0.335
Tan	10	33118501	33118800	0.265	0.612	0.078	0.162
Tan	10	33303601	33303900	1.817	0.783	1.745	2.855
Tan	10	33337201	33337500	2.684	4.901	3.047	1.430
Tan	10	33337501	33337800	2.373	5.823	3.174	0.086
Tan	10	33364801	33365100	1.060	2.581	0.979	1.156
Tan	10	33561901	33562200	2.865	1.654	2.907	3.077
Tan	10	33642901	33643200	2.071	2.875	2.278	2.540
Tan	10	33822301	33822600	0.639	0.863	0.695	0.416
Tan	10	33875401	33875700	0.240	0.727	0.432	0.117
Tan	10	33996601	33996900	0.611	0.914	0.716	0.608
Tan	10	34033501	34033800	0.294	0.704	0.210	0.218
Tan	10	34266901	34267200	0.853	1.408	0.724	0.684
Tan	10	34415701	34416000	0.371	0.607	0.234	0.218
Tan	10	34677301	34677600	0.845	1.596	0.330	0.396
Tan	10	34851601	34851900	1.036	2.478	1.073	1.228
Tan	10	35100301	35100600	0.426	0.966	0.403	0.309
Tan	10	35224501	35224800	0.716	0.888	0.745	0.527
Tan	10	35271901	35272200	0.213	0.515	0.263	0.304
Tan	10	35380501	35380800	0.481	1.169	0.519	0.690

Tan	10	35435701	35436000	0.292	0.491	0.374	0.223
Tan	10	35447101	35447400	1.355	0.729	1.012	1.055
Tan	10	35486101	35486400	0.455	1.240	0.720	0.628
Tan	10	35681401	35681700	0.215	0.863	0.370	0.375
Tan	10	35817601	35817900	0.797	1.468	0.740	0.735
Tan	10	36168601	36168900	3.331	2.090	3.333	3.316
Tan	10	36217801	36218100	0.639	1.098	0.658	0.740
Tan	10	36247201	36247500	0.561	1.098	0.633	0.690
Tan	10	36361801	36362100	0.452	0.583	0.691	0.416
Tan	10	36547201	36547500	2.416	1.253	2.033	2.353
Tan	10	36839701	36840000	0.347	0.564	0.267	0.106
Tan	10	36852001	36852300	0.797	1.877	1.021	1.166
Tan	10	36854701	36855000	0.292	0.541	0.160	0.218
Tan	10	36920401	36920700	0.242	0.701	0.205	0.248
Tan	10	36920701	36921000	0.295	1.311	0.506	0.593
Tan	10	36978601	36978900	0.216	0.963	0.350	0.248
Tan	10	37222501	37222800	0.294	0.468	0.263	0.142
Tan	10	37462801	37463100	0.342	1.011	0.218	0.299
Tan	10	37504501	37504800	0.665	0.818	1.090	0.492
Tan	10	39136501	39136800	0.955	1.571	0.497	0.872
Tan	10	39186601	39186900	0.903	1.667	0.502	0.948
Tan	10	39374101	39374400	1.434	0.935	0.473	0.456
Tan	10	39747901	39748200	0.294	0.822	0.321	0.279
Tan	10	40699201	40699500	0.081	0.472	0.054	0.137
Tan	10	40848301	40848600	0.106	0.420	0.132	0.081
Tan	10	41091901	41092200	0.106	0.371	0.082	0.061
Tan	10	41255101	41255400	0.347	0.657	0.267	0.142
Tan	10	41285701	41286000	0.186	0.489	0.267	0.142
Tan	10	41887801	41888100	0.398	0.629	0.374	0.360
Tan	10	42683401	42683700	0.505	0.584	0.399	0.360
Tan	10	42902701	42903000	0.586	0.466	0.321	0.751
Tan	10	43130101	43130400	0.136	0.466	0.111	0.106
Tan	10	43641601	43641900	0.610	1.695	0.408	0.543
Tan	10	44207401	44207700	0.265	0.539	0.263	0.228
Tan	10	45574501	45574800	1.842	0.704	1.529	2.007
Tan	10	46370101	46370400	0.082	0.281	0.082	0.527
Tan	10	46528201	46528500	1.037	1.216	0.667	0.695
Tan	10	46767301	46767600	0.557	0.841	0.317	0.380
Tan	10	46961701	46962000	0.479	0.820	0.292	0.391
Tan	10	47207401	47207700	2.341	1.888	2.332	1.476
Tan	10	47274001	47274300	0.160	0.515	0.350	0.167
Tan	10	47754301	47754600	0.345	0.768	0.424	0.350
Tan	10	47945401	47945700	0.847	1.543	0.662	0.644
Tan	10	47951101	47951400	0.531	0.654	0.239	0.527
Tan	10	47954101	47954400	0.106	0.493	0.160	0.086
Tan	10	48085501	48085800	0.215	0.558	0.210	0.274
Tan	10	49084201	49084500	0.160	0.493	0.098	0.112
Tan	10	49107601	49107900	0.932	1.242	0.506	0.862
Tan	10	49221601	49221900	0.132	0.491	0.239	0.147
Tan	10	49557601	49557900	1.334	2.617	1.498	1.419
Tan	10	49573201	49573500	0.027	0.472	0.160	0.106
Tan	10	49840201	49840500	1.036	0.935	0.823	0.664
Tan	10	49911601	49911900	0.268	0.564	0.243	0.274
Tan	10	50119501	50119800	0.610	1.217	0.399	0.826
Tan	10	50773801	50774100	2.047	2.457	1.213	1.389
Tan	10	51242701	51243000	0.208	0.817	0.181	0.243
Tan	10	51243001	51243300	1.529	3.141	0.807	0.979
Tan	10	52306501	52306800	0.531	0.863	0.531	0.279
Tan	10	52456201	52456500	0.368	1.654	0.424	0.416
Tan	10	52706101	52706400	0.129	0.633	0.116	0.137
Tan	10	53039701	53040000	0.106	0.493	0.132	0.203
Tan	10	53222401	53222700	0.108	1.278	0.025	0.142
Tan	10	53361601	53361900	0.134	0.491	0.321	0.223
Tan	10	53686501	53686800	0.131	0.513	0.127	0.147
Tan	10	53762701	53763000	0.026	0.352	0.535	0.167
Tan	10	53834701	53835000	0.876	0.584	0.218	0.862
Tan	10	54904501	54904800	0.400	0.749	0.428	0.416

Tan	10	55028101	55028400	0.136	0.491	0.058	0.117
Tan	10	55371001	55371300	0.077	0.420	0.107	0.137
Tan	10	55556401	55556700	0.213	0.494	0.107	0.142
Tan	10	55633501	55633800	0.082	0.515	0.107	0.137
Tan	10	55662001	55662300	1.329	0.961	0.749	1.410
Tan	10	56387401	56387700	5.057	2.931	4.608	5.322
Tan	10	56658001	56658300	0.027	0.846	0.243	0.233
Tan	10	57168601	57168900	0.108	0.446	0.210	0.031
Tan	10	57362401	57362700	0.184	0.609	0.214	0.198
Tan	10	57365701	57366000	0.052	0.491	0.210	0.193
Tan	10	57447901	57448200	0.081	0.371	0.132	0.025
Tan	10	57567001	57567300	0.582	1.328	0.720	1.694
Tan	10	57710701	57711000	0.452	0.116	0.560	0.436
Tan	10	57876601	57876900	0.294	0.609	0.350	0.279
Tan	10	58204201	58204500	0.479	0.187	0.531	0.421
Tan	10	59785501	59785800	0.132	0.401	0.132	0.142
Tan	10	61060501	61060800	0.134	0.749	0.428	0.268
Tan	10	61386301	61386600	0.240	0.652	0.424	0.416
Tan	10	61660201	61660500	0.134	0.420	0.132	0.248
Tan	10	61863901	61864200	0.187	0.538	0.296	0.187
Tan	10	62015701	62016000	0.773	1.191	0.675	0.522
Tan	10	62212501	62212800	0.108	0.631	0.292	0.248
Tan	10	62915101	62915400	0.186	0.395	0.078	0.081
Tan	10	62944501	62944800	0.265	0.635	0.136	0.284
Tan	10	63162301	63162600	0.269	0.654	0.214	0.218
Tan	10	63351301	63351600	0.374	0.839	0.239	0.385
Tan	10	63351601	63351900	0.268	0.860	0.239	0.375
Tan	10	63700201	63700500	0.052	0.446	0.165	0.106
Tan	10	63708901	63709200	0.452	1.278	0.350	0.517
Tan	10	63817201	63817500	0.344	0.657	0.296	0.162
Tan	10	63867601	63867900	0.294	0.745	0.308	0.254
Tan	10	64534501	64534800	0.453	0.702	0.506	0.309
Tan	10	65611501	65611800	0.555	1.098	0.506	0.522
Tan	10	66226201	66226500	1.065	1.500	0.386	1.055
Tan	10	66875401	66875700	0.186	0.539	0.185	0.167
Tan	10	68793301	68793600	0.398	0.773	0.189	0.405
Tan	10	68793601	68793900	0.508	1.081	0.346	0.431
Tan	10	68829001	68829300	0.295	0.513	0.185	0.801
Tan	10	69195901	69196200	0.715	1.586	1.023	0.573
Tan	10	69230701	69231000	0.131	0.487	0.029	0.051
Tan	10	69361801	69362100	0.239	0.588	0.181	0.086
Tan	10	69379201	69379500	0.290	0.583	0.103	0.223
Tan	10	69451201	69451500	0.347	0.845	0.341	0.274
Tan	10	69482701	69483000	0.771	1.219	0.662	0.669
Tan	10	69535201	69535500	0.879	1.360	0.963	0.725
Tan	10	69605101	69605400	0.424	0.815	0.432	0.106
Tan	10	69735601	69735900	0.561	1.551	0.560	0.518
Tan	10	70304701	70305000	0.929	2.135	0.296	0.676
Tan	10	70423801	70424100	0.473	1.526	0.551	0.710
Tan	10	70449901	70450200	0.106	0.397	0.107	0.583
Tan	10	70462501	70462800	0.077	0.468	0.132	0.218
Tan	10	70474801	70475100	0.295	0.933	0.448	0.527
Tan	10	71067301	71067600	0.265	0.704	0.288	0.335
Tan	10	71595001	71595300	0.240	0.446	0.189	0.193
Tan	10	71604901	71605200	0.213	0.584	0.082	0.329
Tan	10	71666401	71666700	0.052	0.588	0.029	0.101
Tan	10	71681101	71681400	0.313	1.099	0.049	0.608
Tan	10	71759701	71760000	0.077	0.539	0.000	0.187
Tan	10	71988901	71989200	0.240	0.423	0.049	0.000
Tan	10	72340501	72340800	0.000	0.493	0.107	0.000
Tan	10	72340801	72341100	0.160	1.906	0.310	0.735
Tan	10	72547201	72547500	0.610	0.281	0.560	0.502
Tan	10	72814201	72814500	0.349	1.150	0.415	0.248
Tan	10	72948301	72948600	0.292	0.678	0.321	0.268
Tan	10	73628401	73628700	3.697	5.613	4.019	5.075
Tan	10	73843201	73843500	1.803	2.528	1.572	1.242
Tan	10	74156101	74156400	0.081	0.187	0.531	0.304

Tan	10	74219401	74219700	0.823	0.185	0.403	0.446
Tan	10	74224201	74224500	0.240	0.539	0.103	0.274
Tan	10	74229901	74230200	1.539	3.864	2.599	1.911
Tan	10	74355601	74355900	0.670	1.927	0.930	0.908
Tan	10	74827201	74827500	0.745	1.425	0.724	0.659
Tan	10	75223501	75223800	0.213	0.704	0.317	0.218
Tan	10	75487801	75488100	0.745	1.238	0.938	0.968
Tan	10	75541801	75542100	0.292	0.515	0.239	0.198
Tan	10	75834901	75835200	0.081	0.466	0.267	0.198
Tan	10	75870901	75871200	1.092	1.594	1.152	0.887
Tan	10	75946201	75946500	0.690	1.779	0.950	0.649
Tan	10	76455901	76456200	0.692	0.513	0.399	0.776
Tan	10	76467001	76467300	0.295	0.657	0.321	0.360
Tan	10	76641601	76641900	0.216	0.749	0.321	0.213
Tan	10	76742401	76742700	0.931	0.449	1.008	0.902
Tan	10	76866901	76867200	0.345	0.725	0.399	0.198
Tan	10	76976701	76977000	0.398	0.729	0.370	0.112
Tan	10	77154901	77155200	0.106	0.442	0.239	0.248
Tan	10	77640301	77640600	0.292	0.772	0.379	0.396
Tan	10	77807101	77807400	0.265	0.609	0.317	0.365
Tan	10	77846101	77846400	0.874	1.380	0.724	0.527
Tan	10	77846401	77846700	2.466	4.465	2.340	1.891
Tan	10	77981701	77982000	0.292	0.656	0.210	0.234
Tan	10	78160801	78161100	0.477	1.006	0.568	0.507
Tan	10	78401101	78401400	0.561	1.103	0.568	0.472
Tan	10	78432901	78433200	0.902	1.491	0.974	0.573
Tan	10	79070101	79070400	0.769	0.468	0.346	0.608
Tan	10	79675801	79676100	0.455	0.959	0.617	0.634
Tan	10	80323801	80324100	0.239	0.729	0.234	0.187
Tan	10	80823901	80824200	0.666	0.981	0.584	0.421
Tan	10	80863201	80863500	0.289	0.773	0.321	0.335
Tan	10	80865301	80865600	1.652	2.152	1.181	1.597
Tan	10	80891101	80891400	0.450	1.101	0.653	0.547
Tan	10	81001501	81001800	0.376	0.656	0.983	1.217
Tan	10	81127501	81127800	0.452	0.163	0.506	0.279
Tan	10	81131101	81131400	0.321	0.656	0.366	0.198
Tan	10	81205201	81205500	0.321	0.912	0.560	0.396
Tan	10	81210901	81211200	0.268	0.725	0.234	0.396
Tan	10	81236101	81236400	2.159	1.450	1.962	2.830
Tan	10	81309301	81309600	1.620	2.409	1.077	1.861
Tan	10	81421501	81421800	0.105	0.721	0.107	0.112
Tan	10	81437701	81438000	1.229	2.343	1.287	1.282
Tan	10	81489601	81489900	4.252	2.544	4.039	3.280
Tan	10	81508201	81508500	0.553	1.566	0.731	0.923
Tan	10	81571201	81571500	0.613	0.260	0.613	0.558
Tan	10	81577201	81577500	3.652	1.388	4.302	4.051
Tan	10	81616501	81616800	1.731	0.886	1.884	1.500
Tan	10	81758401	81758700	0.079	0.446	0.029	0.061
Tan	10	81905101	81905400	0.294	0.564	0.132	0.279
Tan	10	81918001	81918300	0.507	0.981	0.448	0.452
Tan	10	81965701	81966000	0.400	1.034	0.296	0.228
Tan	10	81966001	81966300	0.695	1.478	0.814	0.877
Tan	10	82049401	82049700	0.453	0.935	0.374	0.588
Tan	10	82119601	82119900	0.479	0.845	0.564	0.497
Tan	10	82145401	82145700	0.345	0.882	0.346	0.319
Tan	10	82260601	82260900	0.450	0.773	0.181	0.335
Tan	10	82901101	82901400	0.347	0.890	0.506	0.248
Tan	10	83006101	83006400	0.239	0.629	0.312	0.299
Tan	10	83029201	83029500	0.134	0.583	0.312	0.360
Tan	10	83173801	83174100	0.184	0.515	0.267	0.274
Tan	10	83226601	83226900	0.453	1.309	0.584	0.771
Tan	10	83497201	83497500	1.379	2.135	1.045	1.273
Tan	10	83580601	83580900	0.186	0.541	0.098	0.167
Tan	10	83760901	83761200	2.329	1.330	2.349	2.292
Tan	10	83805001	83805300	2.231	3.592	2.867	1.876
Tan	10	83842501	83842800	1.570	2.313	1.023	1.521
Tan	10	83904901	83905200	0.529	0.912	0.312	0.385

Tan	10	84027901	84028200	0.318	0.869	0.395	0.299
Tan	10	84069901	84070200	0.294	0.702	0.181	0.299
Tan	10	84103501	84103800	5.770	4.170	5.553	6.435
Tan	10	84133801	84134100	0.823	1.006	0.560	0.578
Tan	10	84287401	84287700	0.079	0.538	0.029	0.329
Tan	10	84335401	84335700	0.373	0.845	0.497	0.329
Tan	10	84552901	84553200	0.668	1.526	0.794	0.568
Tan	10	84665401	84665700	0.536	1.101	0.539	0.517
Tan	10	84789901	84790200	1.065	0.723	1.016	1.212
Tan	10	84846001	84846300	0.239	0.612	0.292	0.223
Tan	10	84883801	84884100	2.600	1.817	3.172	2.606
Tan	10	85110901	85111200	1.758	1.165	1.699	1.845
Tan	10	85112701	85113000	0.771	1.358	0.568	0.624
Tan	10	85128301	85128600	1.755	1.496	1.246	0.826
Tan	10	85253401	85253700	1.749	0.648	1.427	1.861
Tan	10	85272901	85273200	1.168	0.483	1.094	1.131
Tan	10	85273201	85273500	0.187	0.326	0.502	0.167
Tan	10	85454401	85454700	1.887	2.547	1.926	1.617
Tan	10	85600501	85600800	3.034	0.826	1.908	2.095
Tan	10	85662301	85662600	12.704	8.304	11.654	13.706
Tan	10	85743901	85744200	1.252	2.890	0.901	1.536
Tan	10	85744501	85744800	0.773	1.551	0.749	0.786
Tan	10	85749601	85749900	0.941	3.073	0.941	1.049
Tan	10	85841401	85841700	0.560	1.661	0.539	0.628
Tan	10	85843501	85843800	6.445	4.085	7.220	7.660
Tan	10	85974301	85974600	0.345	0.657	0.346	0.248
Tan	10	86046601	86046900	2.767	1.624	2.513	2.565
Tan	10	86398201	86398500	0.160	0.444	0.243	0.106
Tan	11	17701	18000	33.829	41.811	27.944	32.802
Tan	11	121501	121800	1.674	2.482	1.093	1.116
Tan	11	199801	200100	2.396	2.875	1.514	1.734
Tan	11	1489501	1489800	0.345	0.584	0.214	0.365
Tan	11	1548901	1549200	0.452	0.914	0.667	0.416
Tan	11	1703701	1704000	0.215	0.701	0.156	0.223
Tan	11	1806301	1806600	0.000	0.579	0.000	0.000
Tan	11	1806601	1806900	0.000	0.579	0.000	0.000
Tan	11	2040901	2041200	0.398	0.678	0.399	0.360
Tan	11	3498001	3498300	0.053	0.393	0.029	0.061
Tan	11	3559201	3559500	0.026	0.418	0.029	0.132
Tan	11	4577401	4577700	0.052	0.539	0.111	0.000
Tan	11	4577701	4578000	0.105	0.446	0.082	0.061
Tan	11	4778401	4778700	0.213	0.916	0.526	0.421
Tan	11	5042101	5042400	0.079	0.448	0.189	0.056
Tan	11	5149501	5149800	0.527	1.380	0.769	0.963
Tan	11	5447701	5448000	0.266	0.605	0.234	0.304
Tan	11	5533801	5534100	0.319	0.564	0.346	0.304
Tan	11	5697901	5698200	0.481	0.890	0.526	0.411
Tan	11	5766601	5766900	0.237	0.721	0.263	0.268
Tan	11	5966701	5967000	0.395	0.860	0.292	0.385
Tan	11	6037801	6038100	0.479	0.120	0.448	0.461
Tan	11	6414901	6415200	0.423	1.077	0.588	0.421
Tan	11	6467101	6467400	0.373	0.802	0.132	0.137
Tan	11	6524701	6525000	0.081	0.511	0.103	0.304
Tan	11	6647401	6647700	0.294	0.916	0.174	0.254
Tan	11	6862801	6863100	0.398	1.176	0.584	0.527
Tan	11	6983101	6983400	0.160	0.491	0.243	0.187
Tan	11	7176601	7176900	0.132	0.371	0.078	0.086
Tan	11	7332001	7332300	1.302	1.821	1.155	1.141
Tan	11	7332301	7332600	0.239	0.628	0.312	0.248
Tan	11	7369801	7370100	0.318	0.609	0.292	0.228
Tan	11	7404601	7404900	1.996	3.452	1.563	1.811
Tan	11	7422301	7422600	0.794	1.800	0.635	0.791
Tan	11	7455001	7455300	1.063	1.712	0.889	1.009
Tan	11	7653601	7653900	4.499	6.563	3.626	4.071
Tan	11	7702501	7702800	0.639	3.152	1.988	1.573
Tan	11	7704901	7705200	3.880	5.711	3.914	3.956
Tan	11	7874401	7874700	0.611	1.032	0.588	0.416

Tan	11	7887901	7888200	3.136	4.643	3.212	3.514
Tan	11	7951201	7951500	0.745	1.835	0.896	0.923
Tan	11	8002501	8002800	6.617	3.411	6.402	7.847
Tan	11	8009401	8009700	0.826	1.171	0.802	0.882
Tan	11	8027101	8027400	1.066	1.828	0.794	0.295
Tan	11	8099401	8099700	3.086	1.321	2.864	2.495
Tan	11	8099701	8100000	2.875	1.676	2.934	2.945
Tan	11	8109301	8109600	0.186	0.905	0.399	0.248
Tan	11	8175901	8176200	0.182	0.912	0.132	0.198
Tan	11	8189701	8190000	0.971	2.624	0.659	0.872
Tan	11	8190001	8190300	0.579	1.382	0.544	0.492
Tan	11	8817901	8818200	0.690	1.708	0.781	0.913
Tan	11	8862601	8862900	0.503	0.210	0.638	0.547
Tan	11	8879701	8880000	0.106	0.493	0.243	0.112
Tan	11	9008701	9009000	1.487	3.570	1.201	1.674
Tan	11	9546601	9546900	0.134	0.513	0.346	0.274
Tan	11	9687601	9687900	0.768	1.502	0.653	0.578
Tan	11	9758101	9758400	0.477	1.099	0.395	0.533
Tan	11	9943201	9943500	0.397	0.963	0.428	0.238
Tan	11	10008901	10009200	0.400	0.725	0.189	0.583
Tan	11	10012801	10013100	0.186	0.562	0.292	0.248
Tan	11	10104301	10104600	0.690	1.075	0.468	0.421
Tan	11	10109401	10109700	0.292	0.539	0.267	0.142
Tan	11	10220401	10220700	0.134	0.536	0.321	0.279
Tan	11	10560001	10560300	1.008	1.639	0.481	1.222
Tan	11	10694401	10694700	0.294	0.560	0.292	0.092
Tan	11	11177101	11177400	0.477	0.118	0.453	0.355
Tan	11	11276701	11277000	0.689	1.332	0.444	0.974
Tan	11	11720401	11720700	0.184	0.511	0.111	0.142
Tan	11	11996701	11997000	0.266	0.815	0.588	0.142
Tan	11	12060601	12060900	0.398	0.871	0.317	0.405
Tan	11	12169201	12169500	0.134	0.395	0.078	0.137
Tan	11	12486301	12486600	0.290	0.893	0.288	0.167
Tan	11	12563101	12563400	1.037	1.217	0.774	0.999
Tan	11	12595201	12595500	1.594	0.912	1.456	1.262
Tan	11	12624601	12624900	0.477	0.820	0.337	0.279
Tan	11	12742801	12743100	0.718	0.470	0.185	0.329
Tan	11	13254601	13254900	0.295	0.609	0.292	0.031
Tan	11	13308001	13308300	0.158	0.704	0.337	0.193
Tan	11	13346701	13347000	0.613	0.985	0.526	0.628
Tan	11	13557001	13557300	0.190	0.985	0.252	0.299
Tan	11	13599001	13599300	1.037	1.620	0.798	0.948
Tan	11	13602601	13602900	0.265	0.420	0.239	0.112
Tan	11	14045701	14046000	0.211	0.612	0.160	0.218
Tan	11	14247901	14248200	0.400	0.704	0.239	0.187
Tan	11	14512201	14512500	3.275	4.085	2.633	2.763
Tan	11	14538001	14538300	0.507	1.047	0.370	0.309
Tan	11	14627101	14627400	0.161	0.442	0.160	0.056
Tan	11	14647501	14647800	0.744	0.212	0.374	0.553
Tan	11	14766901	14767200	0.213	0.466	0.214	0.329
Tan	11	14783101	14783400	0.345	0.539	0.132	0.304
Tan	11	15302701	15303000	1.848	3.302	1.835	1.409
Tan	11	15359101	15359400	0.160	1.555	0.671	0.628
Tan	11	15523801	15524100	0.345	0.515	0.239	0.355
Tan	11	15657001	15657300	4.157	2.620	4.780	4.098
Tan	11	15665101	15665400	1.412	0.978	1.625	0.578
Tan	11	15687301	15687600	0.931	0.446	0.823	0.669
Tan	11	15780901	15781200	0.131	0.584	0.160	0.304
Tan	11	16014901	16015200	0.452	0.863	0.526	0.360
Tan	11	16068301	16068600	0.453	1.169	0.571	0.603
Tan	11	16140601	16140900	0.321	0.867	0.486	0.355
Tan	11	16355701	16356000	0.477	0.936	0.588	0.405
Tan	11	16517401	16517700	0.929	1.637	0.987	0.578
Tan	11	16632301	16632600	0.426	0.725	0.457	0.284
Tan	11	16677001	16677300	0.636	0.212	0.720	0.603
Tan	11	16689301	16689600	0.374	0.092	0.535	0.547
Tan	11	16716601	16716900	0.240	0.558	0.181	0.173

Tan	11	16759801	16760100	0.936	1.686	0.916	0.867
Tan	11	16813801	16814100	0.663	1.875	0.609	0.973
Tan	11	16876801	16877100	6.086	5.464	6.750	6.932
Tan	11	16964701	16965000	0.771	0.511	0.872	0.801
Tan	11	16985701	16986000	0.661	1.289	0.588	0.705
Tan	11	16986901	16987200	0.534	1.009	0.502	0.547
Tan	11	17036401	17036700	2.095	1.075	2.102	1.577
Tan	11	17320801	17321100	0.132	0.584	0.346	0.167
Tan	11	17620801	17621100	0.373	0.515	0.160	0.167
Tan	11	18084001	18084300	0.240	1.515	0.818	0.974
Tan	11	18109201	18109500	0.268	0.629	0.152	0.223
Tan	11	18134101	18134400	0.429	0.929	1.884	0.452
Tan	11	18328201	18328500	0.397	0.790	0.312	0.360
Tan	11	18570301	18570600	0.476	0.845	0.453	0.248
Tan	11	18573601	18573900	0.453	1.079	0.165	0.391
Tan	11	18606601	18606900	0.637	1.146	0.695	0.893
Tan	11	18685801	18686100	0.079	0.517	0.136	0.162
Tan	11	18708001	18708300	2.291	3.918	2.932	2.911
Tan	11	18779701	18780000	0.268	0.798	0.165	0.315
Tan	11	18840001	18840300	1.091	1.309	1.596	0.659
Tan	11	18947101	18947400	0.103	0.678	0.147	0.076
Tan	11	19129501	19129800	1.599	1.332	0.827	1.105
Tan	11	19396201	19396500	1.760	2.772	1.587	1.577
Tan	11	19468801	19469100	0.557	1.079	0.613	0.567
Tan	11	19686901	19687200	0.081	0.466	0.160	0.304
Tan	11	20025901	20026200	1.326	1.959	0.620	0.461
Tan	11	20139001	20139300	0.266	0.702	0.214	0.243
Tan	11	20889901	20890200	0.373	0.560	0.160	0.385
Tan	11	20934301	20934600	0.611	0.914	0.448	0.664
Tan	11	21112501	21112800	0.560	1.028	0.531	0.720
Tan	11	21161701	21162000	0.374	0.538	0.136	0.167
Tan	11	21338701	21339000	0.268	1.026	0.403	0.446
Tan	11	21543901	21544200	0.155	0.586	0.160	0.142
Tan	11	21554701	21555000	1.058	1.923	1.135	0.796
Tan	11	21586201	21586500	0.319	0.584	0.312	0.309
Tan	11	21782401	21782700	0.184	0.468	0.185	0.137
Tan	11	21912301	21912600	1.516	1.944	1.538	1.566
Tan	11	21924901	21925200	1.113	1.729	0.959	0.893
Tan	11	21963301	21963600	0.878	1.382	0.954	1.105
Tan	11	21969601	21969900	0.534	0.047	0.292	0.304
Tan	11	21971701	21972000	0.239	0.560	0.160	0.173
Tan	11	22218301	22218600	0.907	0.633	0.374	0.329
Tan	11	22345501	22345800	0.292	0.798	0.370	0.248
Tan	11	22536001	22536300	0.932	1.538	0.888	0.715
Tan	11	22591801	22592100	0.268	0.538	0.160	0.355
Tan	11	22618801	22619100	0.531	0.725	0.239	0.248
Tan	11	22776601	22776900	0.211	0.442	0.160	0.162
Tan	11	22779901	22780200	0.529	0.916	0.350	0.162
Tan	11	22880701	22881000	0.321	0.824	0.312	0.187
Tan	11	22882801	22883100	0.824	1.596	0.560	0.821
Tan	11	22988401	22988700	0.215	0.468	0.239	0.106
Tan	11	23157601	23157900	0.319	0.611	0.210	0.274
Tan	11	23256901	23257200	0.452	1.264	0.515	0.776
Tan	11	23292601	23292900	1.626	2.579	1.712	1.451
Tan	11	23736901	23737200	0.452	0.747	0.189	0.162
Tan	11	23754901	23755200	0.132	0.468	0.082	0.254
Tan	11	23913001	23913300	0.187	0.539	0.247	0.248
Tan	11	24027301	24027600	1.707	1.146	1.801	1.577
Tan	11	24029101	24029400	1.060	0.605	1.090	1.247
Tan	11	24064801	24065100	1.568	1.193	1.460	0.907
Tan	11	24257401	24257700	0.213	0.562	0.210	0.213
Tan	11	24535801	24536100	0.373	0.562	0.181	0.137
Tan	11	24549901	24550200	0.294	0.773	0.107	0.385
Tan	11	24642601	24642900	2.021	1.261	2.258	2.382
Tan	11	24800701	24801000	2.049	1.356	2.082	2.165
Tan	11	24934801	24935100	0.718	1.307	0.921	0.781
Tan	11	25174501	25174800	3.917	1.939	3.635	4.294

Tan	11	25299301	25299600	0.529	1.122	0.642	0.628
Tan	11	25491901	25492200	0.189	0.790	0.132	0.335
Tan	11	25492201	25492500	0.108	0.929	0.078	0.411
Tan	11	25529701	25530000	0.186	0.491	0.185	0.173
Tan	11	25715101	25715400	0.239	0.517	0.049	0.173
Tan	11	25901401	25901700	0.265	0.470	0.107	0.112
Tan	11	25958701	25959000	0.079	0.468	0.181	0.081
Tan	11	26007901	26008200	1.389	2.853	1.265	1.425
Tan	11	26111701	26112000	3.823	2.444	3.781	3.727
Tan	11	26135401	26135700	0.213	0.566	0.185	0.218
Tan	11	26146801	26147100	0.215	0.706	0.107	0.329
Tan	11	26487301	26487600	1.037	1.545	0.983	1.739
Tan	11	26677501	26677800	0.342	0.800	0.341	0.223
Tan	11	26868601	26868900	0.319	0.584	0.395	0.416
Tan	11	26907301	26907600	0.211	0.493	0.214	0.142
Tan	11	27071101	27071400	0.186	0.511	0.263	0.254
Tan	11	27162301	27162600	0.792	0.097	0.831	0.751
Tan	11	27213301	27213600	0.847	1.873	0.791	0.786
Tan	11	27239701	27240000	0.132	0.494	0.181	0.193
Tan	11	27240301	27240600	0.134	0.444	0.181	0.086
Tan	11	27312001	27312300	1.407	2.592	1.451	1.257
Tan	11	27818101	27818400	0.740	1.335	0.651	0.664
Tan	11	27837601	27837900	0.640	1.446	0.613	0.452
Tan	11	27889801	27890100	3.584	1.347	3.130	3.403
Tan	11	27918901	27919200	0.211	0.420	0.210	0.198
Tan	11	27938401	27938700	0.347	0.562	0.074	0.162
Tan	11	27938701	27939000	0.773	1.620	0.562	0.527
Tan	11	28046701	28047000	0.481	0.772	0.531	0.522
Tan	11	28132801	28133100	0.716	1.075	0.593	0.634
Tan	11	28142101	28142400	0.215	0.702	0.374	0.213
Tan	11	28428901	28429200	0.318	0.845	0.370	0.254
Tan	11	28644601	28644900	0.507	0.985	0.526	0.639
Tan	11	28920901	28921200	0.319	0.863	0.564	0.132
Tan	11	28921501	28921800	0.187	0.420	0.243	0.137
Tan	11	29141401	29141700	3.668	2.802	3.620	3.382
Tan	11	29255101	29255400	0.718	1.290	0.613	0.603
Tan	11	29284201	29284500	0.055	1.071	0.392	0.193
Tan	11	29340601	29340900	0.215	0.654	0.374	0.416
Tan	11	29480101	29480400	0.508	1.910	0.103	0.324
Tan	11	29802901	29803200	0.319	0.749	0.361	0.365
Tan	11	30142501	30142800	0.213	0.511	0.234	0.218
Tan	11	30261001	30261300	0.161	1.015	0.049	0.268
Tan	11	30635701	30636000	0.265	0.581	0.107	0.167
Tan	11	30742201	30742500	2.607	1.034	2.377	2.722
Tan	11	30766501	30766800	0.663	1.056	0.604	0.634
Tan	11	30920701	30921000	0.318	0.863	0.390	0.553
Tan	11	30923701	30924000	1.244	2.266	1.117	1.222
Tan	11	30973201	30973500	0.318	0.865	0.502	0.507
Tan	11	31682701	31683000	0.321	0.609	0.267	0.360
Tan	11	31713001	31713300	0.826	1.573	0.444	0.745
Tan	11	31811401	31811700	1.407	0.983	1.514	1.435
Tan	11	31866901	31867200	0.079	0.425	0.029	0.056
Tan	11	32125801	32126100	0.794	1.715	0.593	0.655
Tan	11	32178601	32178900	0.240	0.511	0.234	0.142
Tan	11	32504701	32505000	1.039	1.732	0.740	0.958
Tan	11	32598601	32598900	1.629	2.409	1.694	1.100
Tan	11	32684701	32685000	3.880	4.960	3.590	3.777
Tan	11	32893501	32893800	1.118	1.216	0.983	1.354
Tan	11	32957701	32958000	0.186	0.489	0.054	0.243
Tan	11	32970001	32970300	0.106	0.470	0.132	0.223
Tan	11	32996101	32996400	0.295	0.584	0.185	0.279
Tan	11	33049501	33049800	0.613	1.150	0.564	0.684
Tan	11	33053701	33054000	0.374	0.775	0.399	0.391
Tan	11	33055201	33055500	0.187	0.607	0.210	0.421
Tan	11	33241801	33242100	0.452	0.862	0.194	0.365
Tan	11	33339901	33340200	1.300	1.942	1.209	1.100
Tan	11	33458101	33458400	0.932	1.545	0.881	1.009

Tan	11	33463801	33464100	5.016	2.753	5.472	5.445
Tan	11	33660601	33660900	1.460	0.536	1.115	1.004
Tan	11	33735001	33735300	0.558	0.955	0.609	0.628
Tan	11	33897901	33898200	1.276	1.852	1.324	1.206
Tan	11	34149301	34149600	0.745	0.961	0.506	0.690
Tan	11	34164301	34164600	0.771	1.171	0.745	0.812
Tan	11	34238701	34239000	0.215	0.706	0.160	0.137
Tan	11	34453801	34454100	0.055	0.423	0.127	0.025
Tan	11	34455001	34455300	0.373	0.895	0.395	0.329
Tan	11	34539301	34539600	1.516	1.965	1.576	1.151
Tan	11	34744801	34745100	8.267	4.374	7.476	7.884
Tan	11	34745101	34745400	4.172	1.478	2.906	3.580
Tan	11	34755301	34755600	0.027	0.393	0.074	0.000
Tan	11	34849501	34849800	0.931	1.195	0.604	0.502
Tan	11	34875301	34875600	2.205	0.579	2.229	2.166
Tan	11	34878301	34878600	2.189	0.455	2.250	2.129
Tan	11	34887001	34887300	2.583	5.126	3.993	3.239
Tan	11	34922101	34922400	0.237	0.468	0.054	0.162
Tan	11	35054101	35054400	0.292	0.583	0.321	0.416
Tan	11	35223901	35224200	0.213	0.654	0.049	0.081
Tan	11	35266201	35266500	0.266	0.633	0.337	0.248
Tan	11	35274901	35275200	0.503	0.914	0.473	0.268
Tan	11	35360101	35360400	1.762	0.549	1.908	1.561
Tan	11	35556301	35556600	3.947	1.770	3.785	3.352
Tan	11	35556601	35556900	2.818	1.435	2.370	2.261
Tan	11	35896801	35897100	0.053	0.466	0.123	0.137
Tan	11	36014101	36014400	0.960	1.614	0.807	1.070
Tan	11	36148201	36148500	0.240	0.446	0.136	0.137
Tan	11	36179701	36180000	0.240	0.489	0.210	0.193
Tan	11	36244501	36244800	0.613	0.303	0.185	0.441
Tan	11	36255001	36255300	2.286	1.914	2.419	2.434
Tan	11	36283501	36283800	0.161	0.494	0.160	0.223
Tan	11	36527701	36528000	1.139	1.523	0.716	1.136
Tan	11	36607201	36607500	0.666	0.466	0.823	0.882
Tan	11	36765601	36765900	1.158	2.178	0.987	1.131
Tan	11	36807301	36807600	1.621	2.393	1.424	1.410
Tan	11	36876301	36876600	0.105	0.423	0.078	0.137
Tan	11	36930301	36930600	1.087	1.547	0.954	1.100
Tan	11	37004701	37005000	0.106	0.418	0.132	0.162
Tan	11	37123201	37123500	0.265	1.174	0.568	0.289
Tan	11	37259401	37259700	0.989	0.092	1.099	1.698
Tan	11	37415101	37415400	6.249	5.774	6.840	4.664
Tan	11	37864801	37865100	0.426	0.584	0.156	0.299
Tan	11	37907401	37907700	1.015	1.701	1.008	1.050
Tan	11	38005201	38005500	0.187	0.538	0.165	0.157
Tan	11	38241301	38241600	0.692	0.584	0.399	0.466
Tan	11	38766601	38766900	1.036	0.438	1.028	1.212
Tan	11	38980801	38981100	1.250	1.807	1.329	0.938
Tan	11	39062401	39062700	0.344	0.678	0.267	0.421
Tan	11	39075601	39075900	0.555	1.242	0.403	0.751
Tan	11	39168301	39168600	0.160	0.303	0.448	0.142
Tan	11	39184201	39184500	0.744	1.077	0.646	0.441
Tan	11	39219601	39219900	0.616	1.240	0.568	0.497
Tan	11	39248701	39249000	1.699	0.373	3.105	1.293
Tan	11	39249001	39249300	1.781	0.243	3.163	1.430
Tan	11	39352201	39352500	2.822	4.890	2.893	2.986
Tan	11	39438301	39438600	0.295	0.963	0.247	0.142
Tan	11	39637801	39638100	1.942	1.214	1.558	2.535
Tan	11	39839701	39840000	0.373	0.798	0.205	0.350
Tan	11	39855901	39856200	0.160	0.423	0.132	0.187
Tan	11	39871201	39871500	0.081	0.543	0.082	0.051
Tan	11	39985501	39985800	0.216	0.586	0.239	0.254
Tan	11	40061101	40061400	0.768	1.588	0.794	1.151
Tan	11	40167301	40167600	1.566	2.060	1.529	1.334
Tan	11	40572601	40572900	0.642	1.193	0.354	0.502
Tan	11	40605601	40605900	0.292	0.721	0.259	0.187
Tan	11	40646701	40647000	4.344	2.688	3.993	4.512

Tan	11	40672801	40673100	0.211	0.536	0.243	0.274
Tan	11	40879801	40880100	0.132	0.423	0.239	0.223
Tan	11	41226901	41227200	0.211	0.562	0.267	0.187
Tan	11	41483101	41483400	4.838	3.478	5.700	5.735
Tan	11	41483401	41483700	2.025	1.427	3.090	2.424
Tan	11	41487301	41487600	1.921	3.401	1.622	1.693
Tan	11	41552401	41552700	0.957	0.541	0.769	0.223
Tan	11	41589601	41589900	1.326	2.038	1.369	1.070
Tan	11	41793301	41793600	3.112	2.435	1.933	2.956
Tan	11	41812201	41812500	0.027	0.612	0.111	0.081
Tan	11	41833501	41833800	0.213	0.556	0.214	0.223
Tan	11	41953801	41954100	0.160	0.564	0.181	0.137
Tan	11	42026701	42027000	0.321	0.538	0.185	0.299
Tan	11	42134401	42134700	0.529	1.193	0.506	0.517
Tan	11	42313501	42313800	1.434	1.144	1.757	1.724
Tan	11	42352201	42352500	0.453	0.773	0.341	0.416
Tan	11	42425701	42426000	0.347	1.058	0.453	0.268
Tan	11	42738901	42739200	0.497	2.201	0.455	0.391
Tan	11	42842401	42842700	0.082	0.517	0.132	0.112
Tan	11	43038901	43039200	2.476	0.998	1.732	2.475
Tan	11	43134301	43134600	0.479	0.609	0.156	0.248
Tan	11	43283701	43284000	0.397	0.772	0.555	0.497
Tan	11	43342201	43342500	1.886	2.197	1.436	1.709
Tan	11	43404001	43404300	0.689	1.644	0.838	0.776
Tan	11	43428001	43428300	2.476	1.388	2.024	2.023
Tan	11	43429501	43429800	0.637	0.910	0.720	1.247
Tan	11	43535401	43535700	0.186	0.446	0.132	0.112
Tan	11	43916401	43916700	0.931	0.157	0.740	0.731
Tan	11	43931401	43931700	0.213	0.420	0.210	0.193
Tan	11	43982701	43983000	2.020	1.753	1.139	0.806
Tan	11	43995001	43995300	0.239	0.489	0.185	0.304
Tan	11	44068501	44068800	0.319	0.678	0.453	0.274
Tan	11	44264701	44265000	0.132	0.637	0.214	0.137
Tan	11	44269801	44270100	2.480	0.748	2.905	2.819
Tan	11	44272501	44272800	0.131	0.635	0.098	0.187
Tan	11	44351401	44351700	1.758	2.079	1.489	1.471
Tan	11	44402401	44402700	0.368	1.056	0.428	0.299
Tan	11	44499001	44499300	0.398	0.796	0.341	0.466
Tan	11	44514001	44514300	0.670	1.195	0.642	0.583
Tan	11	44860201	44860500	2.605	3.821	1.993	2.733
Tan	11	45027901	45028200	0.639	1.335	0.357	0.396
Tan	11	45040801	45041100	0.160	0.678	0.317	0.421
Tan	11	45118201	45118500	0.239	0.539	0.132	0.218
Tan	11	45347101	45347400	1.226	1.614	1.130	2.124
Tan	11	45367501	45367800	1.116	0.701	1.008	0.918
Tan	11	45389101	45389400	0.295	0.583	0.263	0.304
Tan	11	45394201	45394500	0.637	0.935	0.486	0.502
Tan	11	45674101	45674400	0.874	1.332	0.506	0.791
Tan	11	45810601	45810900	0.242	0.656	0.341	0.137
Tan	11	46514701	46515000	1.647	0.586	1.723	1.374
Tan	11	47232601	47232900	3.543	2.201	3.794	3.528
Tan	11	47267101	47267400	0.848	1.498	1.052	0.533
Tan	11	47272501	47272800	0.560	0.232	0.667	0.938
Tan	11	47439601	47439900	1.223	2.466	1.237	1.156
Tan	11	47472901	47473200	0.321	0.656	0.370	0.329
Tan	11	47487301	47487600	0.584	1.382	0.646	0.567
Tan	11	47610301	47610600	1.408	2.223	1.572	1.739
Tan	11	47732401	47732700	0.294	0.912	0.646	0.309
Tan	11	47930401	47930700	0.189	0.609	0.218	0.198
Tan	11	48007201	48007500	0.745	0.536	0.399	0.690
Tan	11	48037501	48037800	0.134	0.442	0.165	0.112
Tan	11	48221701	48222000	0.292	0.560	0.288	0.355
Tan	11	48404101	48404400	0.960	1.452	0.716	0.999
Tan	11	48428401	48428700	0.373	0.586	0.239	0.076
Tan	11	48786301	48786600	0.239	0.468	0.078	0.360
Tan	11	48972301	48972600	0.932	1.667	0.551	0.558
Tan	11	49130101	49130400	0.508	0.818	0.243	0.522

Tan	11	49132801	49133100	2.689	1.564	2.407	2.951
Tan	11	49171501	49171800	0.211	0.421	0.239	0.639
Tan	11	49175401	49175700	1.061	1.852	1.099	1.328
Tan	11	49230601	49230900	2.126	1.311	1.835	2.007
Tan	11	49253101	49253400	3.054	1.706	3.084	3.155
Tan	11	49535401	49535700	1.595	1.472	1.061	1.019
Tan	11	49835101	49835400	0.187	0.423	0.132	0.162
Tan	11	50037301	50037600	1.568	1.143	0.901	1.845
Tan	11	50209801	50210100	0.400	0.985	0.317	0.492
Tan	11	50223301	50223600	3.088	2.386	3.756	3.878
Tan	11	50250001	50250300	0.962	1.877	1.110	1.206
Tan	11	50278201	50278500	0.844	2.109	0.480	0.886
Tan	11	50278501	50278800	1.113	1.901	1.115	1.070
Tan	11	50380801	50381100	2.169	0.882	3.045	3.697
Tan	11	50494501	50494800	0.237	0.562	0.210	0.299
Tan	11	50765701	50766000	1.144	0.697	1.197	1.176
Tan	11	50918701	50919000	0.158	0.515	0.218	0.279
Tan	11	50951701	50952000	0.374	1.051	0.366	0.380
Tan	11	51031801	51032100	0.319	1.107	0.267	0.329
Tan	11	51102001	51102300	0.715	3.323	1.222	1.104
Tan	11	51102301	51102600	0.400	1.079	0.363	0.416
Tan	11	51104101	51104400	0.958	0.260	0.687	1.116
Tan	11	51132301	51132600	0.106	0.401	0.107	0.167
Tan	11	51136501	51136800	0.897	2.030	0.765	1.273
Tan	11	51192601	51192900	0.474	1.075	0.633	0.583
Tan	11	51268201	51268500	1.005	1.635	0.852	1.044
Tan	11	51352201	51352500	0.345	0.515	0.189	0.137
Tan	11	51693901	51694200	6.598	4.396	7.498	7.058
Tan	11	51801901	51802200	3.194	2.001	3.117	3.093
Tan	11	51900301	51900600	1.865	2.714	1.592	2.104
Tan	11	51944401	51944700	2.176	1.476	2.492	2.160
Tan	11	52043401	52043700	2.234	0.972	1.872	1.851
Tan	11	52168201	52168500	1.171	0.744	0.428	1.100
Tan	11	52280401	52280700	0.292	0.725	0.267	0.492
Tan	11	52442701	52443000	1.015	1.748	1.128	0.832
Tan	11	52462801	52463100	0.773	1.592	0.814	0.842
Tan	11	52478101	52478400	1.281	2.236	1.527	1.273
Tan	11	52564201	52564500	2.817	3.529	2.485	2.104
Tan	11	52564501	52564800	1.036	1.405	0.930	1.019
Tan	11	52610401	52610700	0.316	0.893	0.132	0.081
Tan	11	52644001	52644300	0.637	1.030	0.662	0.725
Tan	11	52667701	52668000	0.584	1.214	0.074	0.574
Tan	11	52689301	52689600	1.731	2.334	1.942	1.790
Tan	11	52717801	52718100	1.308	2.062	1.079	1.278
Tan	11	52721701	52722000	3.113	3.720	2.829	3.154
Tan	11	52740301	52740600	1.170	1.710	1.003	0.943
Tan	11	52784401	52784700	2.952	3.602	1.703	2.960
Tan	11	52820401	52820700	0.319	0.907	0.223	0.263
Tan	11	52845601	52845900	0.186	0.472	0.132	0.112
Tan	11	52901101	52901400	0.158	0.824	0.292	0.309
Tan	11	52907101	52907400	0.587	0.116	0.428	0.477
Tan	11	52913401	52913700	4.156	3.130	4.731	5.137
Tan	11	53049001	53049300	0.665	1.144	0.707	0.695
Tan	11	53095501	53095800	0.294	0.773	0.272	0.309
Tan	11	53165701	53166000	1.010	1.946	0.845	1.141
Tan	11	53361001	53361300	0.716	1.311	0.602	0.684
Tan	11	53424601	53424900	0.347	0.699	0.292	0.213
Tan	11	53424901	53425200	0.799	1.431	0.642	0.527
Tan	11	53521201	53521500	0.239	0.444	0.346	0.274
Tan	11	53556001	53556300	1.463	1.918	1.021	1.354
Tan	11	53585101	53585400	1.381	2.365	1.560	1.658
Tan	11	53651401	53651700	0.131	0.466	0.054	0.218
Tan	11	53786401	53786700	0.266	0.489	0.132	0.187
Tan	11	53792401	53792700	0.239	0.534	0.267	0.274
Tan	11	53914201	53914500	0.452	0.234	0.658	0.025
Tan	11	53925901	53926200	0.721	1.240	0.646	0.731
Tan	11	54004201	54004500	0.053	0.395	0.214	0.081

Tan	11	54181801	54182100	0.156	0.772	0.147	0.223
Tan	11	54186601	54186900	0.742	1.658	0.825	0.781
Tan	11	54213301	54213600	2.823	2.038	2.579	2.657
Tan	11	54263101	54263400	0.292	0.466	0.078	0.081
Tan	11	54439801	54440100	3.641	1.618	3.240	3.773
Tan	11	54627301	54627600	0.453	0.798	0.321	0.416
Tan	11	54636601	54636900	1.091	0.251	0.778	0.806
Tan	11	54729001	54729300	0.613	1.590	1.066	0.918
Tan	11	54902701	54903000	0.429	2.288	0.499	0.655
Tan	11	54903001	54903300	0.381	3.418	0.702	0.863
Tan	11	54966301	54966600	0.186	0.420	0.132	0.117
Tan	11	55020301	55020600	0.821	0.094	0.789	0.634
Tan	11	55166401	55166700	0.292	0.165	0.263	0.553
Tan	11	55386901	55387200	0.186	0.446	0.263	0.137
Tan	11	55447501	55447800	0.476	1.169	0.510	0.567
Tan	11	55497901	55498200	0.942	3.257	0.849	1.114
Tan	11	55534501	55534800	0.744	0.513	0.745	0.391
Tan	11	55579501	55579800	1.436	2.742	1.387	1.181
Tan	11	55579801	55580100	0.555	1.075	0.477	0.248
Tan	11	55675501	55675800	0.081	0.539	0.152	0.248
Tan	11	55710001	55710300	0.558	1.596	0.551	0.761
Tan	11	55752601	55752900	0.265	1.105	0.334	0.396
Tan	11	55763401	55763700	6.275	3.389	5.567	6.277
Tan	11	55798801	55799100	0.213	0.423	0.103	0.081
Tan	11	55799401	55799700	0.240	0.916	0.074	0.193
Tan	11	55801501	55801800	2.094	3.602	1.661	1.796
Tan	11	55807201	55807500	0.313	1.974	0.276	0.543
Tan	11	55811701	55812000	0.685	2.485	0.448	0.421
Tan	11	55812001	55812300	1.052	2.382	0.801	0.731
Tan	11	55816501	55816800	0.299	1.658	0.165	0.772
Tan	11	55841101	55841400	1.550	3.057	1.358	1.953
Tan	11	55841401	55841700	0.429	1.408	0.272	0.365
Tan	11	55844401	55844700	0.395	1.519	0.901	0.756
Tan	11	55846201	55846500	0.347	1.452	0.263	0.345
Tan	11	55846501	55846800	0.189	1.030	0.254	0.446
Tan	11	55854601	55854900	3.118	4.514	2.203	2.957
Tan	11	55859401	55859700	0.052	0.448	0.078	0.061
Tan	11	55876201	55876500	1.916	2.952	1.489	2.176
Tan	11	55904701	55905000	0.529	1.056	0.399	0.411
Tan	11	55930801	55931100	0.237	0.539	0.214	0.193
Tan	11	55990201	55990500	0.608	2.180	1.141	0.953
Tan	11	56000101	56000400	0.186	0.661	0.082	0.218
Tan	11	56056201	56056500	1.941	1.126	1.641	1.703
Tan	11	56064601	56064900	1.966	1.307	2.024	2.014
Tan	11	56069101	56069400	2.605	0.773	1.661	1.790
Tan	11	56076901	56077200	5.761	2.108	6.431	6.240
Tan	11	56137801	56138100	2.985	1.160	2.508	2.322
Tan	11	56330701	56331000	1.758	2.416	1.427	1.273
Tan	11	56454301	56454600	0.477	0.961	0.716	0.502
Tan	11	56483701	56484000	0.290	0.564	0.156	0.223
Tan	11	56553901	56554200	0.534	1.075	0.658	0.522
Tan	11	56565901	56566200	4.583	2.680	4.820	4.696
Tan	11	56579101	56579400	1.753	2.057	1.522	1.739
Tan	11	56579701	56580000	3.925	2.527	4.024	4.178
Tan	11	56585101	56585400	3.725	2.100	2.640	2.844
Tan	11	56587501	56587800	1.837	3.431	1.110	1.165
Tan	11	56617501	56617800	0.268	0.493	0.263	0.248
Tan	11	56649301	56649600	6.696	6.215	5.549	8.027
Tan	11	56649601	56649900	2.421	1.866	3.114	2.763
Tan	11	56654701	56655000	0.292	0.468	0.185	0.254
Tan	11	56719801	56720100	0.398	0.745	0.366	0.167
Tan	11	56728801	56729100	1.697	2.521	3.201	1.667
Tan	11	56866501	56866800	0.081	1.113	0.140	0.203
Tan	11	57009601	57009900	1.729	3.576	1.599	2.226
Tan	11	57156301	57156600	0.452	1.313	0.671	0.416
Tan	11	57244201	57244500	0.158	0.635	0.230	0.223
Tan	11	57288601	57288900	1.576	3.422	1.335	1.044

Tan	11	57381301	57381600	0.239	0.798	0.317	0.254
Tan	11	57392701	57393000	0.266	0.489	0.185	0.117
Tan	11	57555301	57555600	1.310	2.214	1.206	0.983
Tan	11	57738901	57739200	0.692	0.397	0.638	0.558
Tan	11	57866701	57867000	0.265	1.013	0.210	0.411
Tan	11	57896401	57896700	0.587	1.032	0.506	0.745
Tan	11	58008901	58009200	0.160	0.257	0.082	0.578
Tan	11	58300501	58300800	1.202	2.221	1.378	1.358
Tan	11	58687501	58687800	1.704	1.221	1.950	2.353
Tan	11	58724401	58724700	0.318	0.584	0.346	0.472
Tan	11	58727401	58727700	3.426	2.598	3.743	3.442
Tan	11	58755901	58756200	0.161	0.895	0.337	0.086
Tan	11	58841401	58841700	0.481	0.723	0.428	0.441
Tan	11	58882501	58882800	0.642	2.013	0.635	0.912
Tan	11	58979101	58979400	0.345	0.846	0.341	0.391
Tan	11	59031601	59031900	0.108	0.744	0.205	0.126
Tan	11	59472601	59472900	0.507	1.744	0.934	0.983
Tan	11	59472901	59473200	1.420	5.233	2.744	3.098
Tan	11	59521501	59521800	0.534	1.009	0.444	0.365
Tan	11	59778001	59778300	1.334	2.882	0.468	0.973
Tan	11	59778301	59778600	0.932	1.950	0.259	0.319
Tan	11	59823001	59823300	0.587	0.888	0.448	0.578
Tan	11	59979601	59979900	0.398	0.491	0.638	0.416
Tan	11	60020701	60021000	0.215	0.583	0.152	0.081
Tan	11	60091201	60091500	2.104	1.167	1.650	1.881
Tan	11	60091501	60091800	1.753	1.009	1.329	1.739
Tan	11	60152401	60152700	1.702	1.776	1.460	2.018
Tan	11	60304201	60304500	3.304	4.611	2.831	2.661
Tan	11	60419101	60419400	0.637	0.794	0.662	0.608
Tan	11	60439801	60440100	1.387	2.077	1.041	1.450
Tan	11	60499501	60499800	0.795	1.553	0.510	0.745
Tan	11	60504301	60504600	5.824	4.035	5.562	5.972
Tan	11	60603901	60604200	1.432	2.173	1.514	1.334
Tan	11	60627601	60627900	2.738	1.826	3.032	2.470
Tan	11	60637501	60637800	2.287	1.188	0.317	0.583
Tan	11	60640801	60641100	2.762	4.452	2.501	3.620
Tan	11	60813301	60813600	0.374	0.796	0.111	0.421
Tan	11	60885901	60886200	0.979	1.916	0.916	1.004
Tan	11	60889201	60889500	4.756	3.810	4.681	4.279
Tan	11	60921901	60922200	0.928	2.085	0.959	0.999
Tan	11	60922201	60922500	1.923	4.000	1.926	1.998
Tan	11	60922501	60922800	0.876	1.457	0.700	0.360
Tan	11	60966901	60967200	2.050	2.719	1.908	1.201
Tan	11	60971101	60971400	0.347	0.682	0.374	0.218
Tan	11	61205101	61205400	9.334	6.391	9.035	9.562
Tan	11	61279801	61280100	0.027	0.487	0.078	0.137
Tan	11	61331101	61331400	4.515	6.943	3.936	3.190
Tan	11	61363801	61364100	0.639	1.217	0.522	0.461
Tan	11	61418101	61418400	0.848	1.495	0.564	0.872
Tan	11	61418401	61418700	0.721	1.807	0.996	0.968
Tan	11	61500001	61500300	5.734	3.291	7.118	5.623
Tan	11	61550101	61550400	1.733	2.485	1.489	1.516
Tan	11	61571101	61571400	2.715	4.102	2.599	2.586
Tan	11	61724701	61725000	0.532	1.006	0.551	0.461
Tan	11	61835701	61836000	1.081	2.528	1.509	1.471
Tan	11	61926301	61926600	0.479	0.772	0.136	0.284
Tan	12	412501	412800	0.184	0.611	0.317	0.193
Tan	12	500701	501000	0.242	0.678	0.165	0.238
Tan	12	523501	523800	0.134	0.420	0.156	0.248
Tan	12	577201	577500	0.663	0.305	0.477	0.553
Tan	12	754501	754800	2.322	3.154	1.164	1.526
Tan	12	754801	755100	2.738	3.248	1.712	2.394
Tan	12	826801	827100	0.134	0.420	0.263	0.081
Tan	12	916501	916800	0.189	0.682	0.165	0.304
Tan	12	1168501	1168800	1.199	1.403	0.901	1.136
Tan	12	1175701	1176000	0.560	1.687	0.609	0.680
Tan	12	1176001	1176300	0.898	2.789	0.687	0.989

Tan	12	1482301	1482600	0.479	0.142	0.629	0.583
Tan	12	1660801	1661100	0.424	0.775	0.477	0.304
Tan	12	1896601	1896900	0.718	0.890	0.453	0.669
Tan	12	2181601	2181900	0.265	0.470	0.054	0.274
Tan	12	2219401	2219700	0.081	0.515	0.156	0.132
Tan	12	2265601	2265900	0.529	1.191	0.588	0.882
Tan	12	2277301	2277600	0.187	0.466	0.263	0.193
Tan	12	2279101	2279400	1.460	2.060	0.959	1.374
Tan	12	2452801	2453100	0.374	0.727	0.243	0.391
Tan	12	2472601	2472900	4.046	3.180	3.999	3.808
Tan	12	2631301	2631600	0.611	0.491	0.263	0.360
Tan	12	2654701	2655000	0.450	1.126	0.720	0.715
Tan	12	2892901	2893200	0.161	0.442	0.000	0.081
Tan	12	2937301	2937600	2.023	1.631	2.439	2.048
Tan	12	2940901	2941200	1.116	0.727	1.275	0.907
Tan	12	3069001	3069300	1.065	1.637	1.246	1.070
Tan	12	3086701	3087000	0.105	0.468	0.189	0.076
Tan	12	3087601	3087900	1.941	1.098	1.509	1.739
Tan	12	3226501	3226800	0.213	0.468	0.296	0.274
Tan	12	3349501	3349800	0.950	1.914	1.070	0.902
Tan	12	3381001	3381300	0.507	0.794	0.506	0.380
Tan	12	3382801	3383100	0.292	0.957	0.535	0.507
Tan	12	3395701	3396000	1.168	0.352	0.987	0.801
Tan	12	3528001	3528300	0.532	0.140	0.370	0.360
Tan	12	3569101	3569400	1.912	1.540	1.971	2.074
Tan	12	3596101	3596400	0.505	1.171	0.531	0.867
Tan	12	3645901	3646200	0.213	1.369	0.156	0.228
Tan	12	3649501	3649800	0.774	1.618	0.959	0.760
Tan	12	3720301	3720600	0.426	0.702	0.424	0.497
Tan	12	3753001	3753300	0.213	0.423	0.160	0.106
Tan	12	3777001	3777300	0.690	1.150	0.609	0.598
Tan	12	4016401	4016700	2.466	1.394	2.432	2.545
Tan	12	4041901	4042200	0.265	0.938	0.074	0.320
Tan	12	4095901	4096200	0.263	0.777	0.185	0.228
Tan	12	4096201	4096500	0.186	0.657	0.205	0.228
Tan	12	4170301	4170600	4.788	3.546	5.147	5.005
Tan	12	4170601	4170900	4.422	2.699	4.340	4.270
Tan	12	4267201	4267500	0.158	0.820	0.403	0.218
Tan	12	4316401	4316700	0.103	0.442	0.029	0.000
Tan	12	4378201	4378500	0.720	1.053	0.535	0.284
Tan	12	4402801	4403100	1.939	1.306	1.797	2.130
Tan	12	5453101	5453400	0.187	0.023	0.477	0.309
Tan	12	5484001	5484300	0.211	0.562	0.374	0.391
Tan	12	5603401	5603700	0.663	1.172	0.502	0.542
Tan	12	5613601	5613900	1.106	3.474	1.930	2.059
Tan	12	5631901	5632200	0.319	0.560	0.374	0.385
Tan	12	5769001	5769300	2.265	0.927	2.602	2.257
Tan	12	5806801	5807100	3.917	1.894	3.823	3.570
Tan	12	5924101	5924400	0.136	0.895	0.103	0.025
Tan	12	6530101	6530400	0.055	0.903	0.054	0.162
Tan	12	6557401	6557700	0.079	0.448	0.127	0.025
Tan	12	6701101	6701400	0.292	0.116	0.424	0.634
Tan	12	6824101	6824400	1.281	1.757	1.275	1.136
Tan	12	7571401	7571700	0.105	0.871	0.049	0.142
Tan	12	7571701	7572000	0.077	0.991	0.098	0.086
Tan	12	7914001	7914300	0.636	0.185	0.477	0.558
Tan	12	7962901	7963200	0.211	0.513	0.205	0.031
Tan	12	9282901	9283200	0.531	1.467	0.653	0.324
Tan	12	9433501	9433800	0.344	1.266	0.025	0.233
Tan	12	9433801	9434100	0.344	1.244	0.225	0.400
Tan	12	9504301	9504600	2.892	1.991	1.159	3.264
Tan	12	9882901	9883200	1.226	0.465	1.333	1.247
Tan	12	10289101	10289400	1.036	1.708	1.197	1.262
Tan	12	10467001	10467300	0.397	1.390	0.432	0.507
Tan	12	10467301	10467600	0.342	1.245	0.419	0.472
Tan	12	10503301	10503600	0.452	0.794	0.428	0.492
Tan	12	10686301	10686600	2.394	1.242	2.276	2.205

Tan	12	11086201	11086500	0.108	0.515	0.243	0.086
Tan	12	11263201	11263500	0.161	0.491	0.054	0.031
Tan	12	11750101	11750400	0.213	0.539	0.185	0.213
Tan	12	11762701	11763000	0.318	0.657	0.189	0.223
Tan	12	12614401	12614700	1.092	1.942	1.311	1.085
Tan	12	12614701	12615000	0.187	0.770	0.230	0.223
Tan	12	13001101	13001400	0.560	0.328	0.564	0.056
Tan	12	13048501	13048800	0.611	0.213	0.555	0.527
Tan	12	14664901	14665200	0.213	0.515	0.160	0.223
Tan	12	14717701	14718000	0.160	0.466	0.214	0.279
Tan	12	15129901	15130200	0.182	0.558	0.160	0.162
Tan	12	15464701	15465000	0.350	0.869	0.205	0.309
Tan	12	15691501	15691800	0.211	0.730	0.223	0.051
Tan	12	16686901	16687200	0.432	1.124	0.448	0.446
Tan	12	16727401	16727700	0.450	0.818	0.230	0.299
Tan	12	16747501	16747800	0.132	0.466	0.214	0.329
Tan	12	16778701	16779000	1.678	1.146	0.573	1.825
Tan	12	16970701	16971000	0.273	0.916	0.107	0.228
Tan	12	16976401	16976700	0.426	1.195	0.437	0.391
Tan	12	16982101	16982400	0.160	0.466	0.103	0.193
Tan	12	17000101	17000400	0.240	0.678	0.346	0.173
Tan	12	17000401	17000700	0.374	0.702	0.390	0.385
Tan	12	17796601	17796900	0.079	0.420	0.049	0.198
Tan	12	17970001	17970300	0.878	0.403	1.246	0.740
Tan	12	18073501	18073800	0.161	0.566	0.156	0.061
Tan	12	18085501	18085800	0.876	1.517	0.959	0.512
Tan	12	18105001	18105300	0.269	0.721	0.267	0.304
Tan	12	18153901	18154200	1.061	1.262	0.852	1.435
Tan	12	18384601	18384900	0.186	0.420	0.160	0.132
Tan	12	18393301	18393600	0.376	1.755	0.573	0.690
Tan	12	18489301	18489600	0.634	1.174	0.481	0.497
Tan	12	18663301	18663600	0.160	0.446	0.132	0.081
Tan	12	18945901	18946200	0.850	0.047	0.762	0.725
Tan	12	19020901	19021200	0.371	0.609	0.292	0.380
Tan	12	19068901	19069200	0.907	2.041	0.856	0.886
Tan	12	19077901	19078200	0.484	1.895	0.558	0.745
Tan	12	19111801	19112100	0.081	0.513	0.292	0.218
Tan	12	19122601	19122900	2.955	4.287	3.337	2.226
Tan	12	19142101	19142400	0.850	0.583	0.453	0.416
Tan	12	19202701	19203000	0.052	0.517	0.181	0.051
Tan	12	19231201	19231500	0.131	0.566	0.132	0.137
Tan	12	19583401	19583700	0.982	0.146	1.246	0.765
Tan	12	19753801	19754100	0.186	0.564	0.107	0.304
Tan	12	19807201	19807500	0.158	0.440	0.054	0.000
Tan	12	19996501	19996800	0.077	0.751	0.205	0.137
Tan	12	20109601	20109900	2.931	0.996	2.426	2.845
Tan	12	20844601	20844900	0.055	0.442	0.136	0.162
Tan	12	21154801	21155100	0.186	0.513	0.218	0.173
Tan	12	21595501	21595800	0.189	0.489	0.165	0.112
Tan	12	22168201	22168500	0.400	0.633	0.346	0.167
Tan	12	22423501	22423800	0.479	0.839	0.477	0.527
Tan	12	22632001	22632300	0.106	0.566	0.082	0.106
Tan	12	22632301	22632600	0.186	0.798	0.111	0.340
Tan	12	23481901	23482200	1.063	1.547	0.974	0.801
Tan	12	23485801	23486100	0.266	0.654	0.288	0.213
Tan	12	23579101	23579400	0.156	0.656	0.267	0.243
Tan	12	23691001	23691300	0.242	0.584	0.165	0.076
Tan	12	23827801	23828100	0.239	0.420	0.267	0.248
Tan	12	24022501	24022800	0.453	0.725	0.399	0.411
Tan	12	24107101	24107400	1.384	0.373	0.760	0.796
Tan	12	24209101	24209400	3.812	4.965	4.022	2.870
Tan	12	24273901	24274200	0.237	0.697	0.317	0.309
Tan	12	24365401	24365700	0.290	0.912	0.145	0.405
Tan	12	24367201	24367500	10.036	6.507	8.191	9.157
Tan	12	24490201	24490500	0.134	0.444	0.078	0.106
Tan	12	24759301	24759600	1.571	2.147	1.529	1.552
Tan	12	24780901	24781200	0.716	2.145	2.550	2.606

Tan	12	24795001	24795300	0.211	1.324	0.098	0.112
Tan	12	25065301	25065600	0.160	0.609	0.243	0.294
Tan	12	25145101	25145400	0.613	1.573	0.617	0.725
Tan	12	25202701	25203000	5.745	3.025	5.871	7.652
Tan	12	25349701	25350000	0.079	0.423	0.111	0.117
Tan	12	25553401	25553700	0.794	0.140	0.724	1.085
Tan	12	25891801	25892100	0.769	1.332	0.901	0.781
Tan	12	25923601	25923900	0.189	0.444	0.687	0.664
Tan	12	26111101	26111400	0.189	0.588	0.185	0.137
Tan	12	26180401	26180700	0.479	0.963	0.210	0.391
Tan	12	26652901	26653200	0.265	0.792	0.160	0.294
Tan	12	26724601	26724900	0.161	0.489	0.025	0.198
Tan	12	27027301	27027600	1.786	0.654	1.057	1.253
Tan	12	27054601	27054900	0.186	0.584	0.136	0.198
Tan	12	27087601	27087900	0.878	0.539	1.226	1.278
Tan	12	27172501	27172800	1.060	2.105	1.099	1.076
Tan	12	27235201	27235500	1.060	1.759	0.834	0.938
Tan	12	27382501	27382800	0.134	0.423	0.160	0.106
Tan	12	27393001	27393300	0.665	0.914	0.403	0.472
Tan	12	27567301	27567600	2.157	3.712	1.893	2.454
Tan	12	27688801	27689100	0.187	0.442	0.210	0.106
Tan	12	27867001	27867300	0.720	1.290	0.691	0.812
Tan	12	28150501	28150800	0.184	0.792	0.292	0.350
Tan	12	28416001	28416300	0.718	0.375	0.535	0.522
Tan	12	29081401	29081700	0.321	0.609	0.165	0.279
Tan	12	29124001	29124300	0.316	0.890	0.296	0.431
Tan	12	29372101	29372400	0.718	0.165	0.346	0.279
Tan	12	29409901	29410200	0.077	0.420	0.185	0.142
Tan	12	29500501	29500800	0.374	0.957	0.439	0.385
Tan	12	29538301	29538600	0.027	0.440	0.160	0.142
Tan	12	30089101	30089400	0.213	0.773	0.247	0.218
Tan	12	30275701	30276000	2.787	3.898	2.728	1.987
Tan	12	30276001	30276300	1.913	3.274	2.180	1.111
Tan	12	30330901	30331200	0.452	1.030	0.568	0.431
Tan	12	30332701	30333000	1.758	3.077	1.645	2.151
Tan	12	30517801	30518100	0.292	0.468	0.210	0.193
Tan	12	30641101	30641400	2.287	1.242	2.193	2.849
Tan	12	30690601	30690900	1.518	2.551	1.696	3.503
Tan	12	30690901	30691200	1.171	2.208	1.313	3.063
Tan	12	30710101	30710400	0.955	2.787	0.616	1.404
Tan	12	31194901	31195200	0.079	0.423	0.054	0.092
Tan	12	31223401	31223700	0.295	0.820	0.424	0.421
Tan	12	31293901	31294200	0.931	1.854	1.102	1.070
Tan	12	31388101	31388400	0.263	0.721	0.181	0.213
Tan	12	31759501	31759800	0.453	0.631	0.370	0.335
Tan	12	31823701	31824000	0.053	0.611	0.160	0.162
Tan	12	32238901	32239200	0.773	0.281	0.646	0.669
Tan	12	32321101	32321400	0.161	0.489	0.103	0.076
Tan	12	32343601	32343900	0.026	0.886	0.281	0.279
Tan	12	32546101	32546400	0.132	0.493	0.156	0.076
Tan	12	32614501	32614800	0.215	0.560	0.205	0.223
Tan	12	32741701	32742000	0.105	0.420	0.160	0.112
Tan	12	33207901	33208200	0.319	0.538	0.370	0.416
Tan	12	33744901	33745200	0.187	0.491	0.214	0.157
Tan	12	34099201	34099500	0.371	0.234	0.477	0.416
Tan	12	34326001	34326300	0.210	0.656	0.230	0.167
Tan	12	34373101	34373400	1.278	0.845	0.580	1.212
Tan	12	34389301	34389600	0.105	0.699	0.205	0.167
Tan	12	34515001	34515300	0.294	0.775	0.234	0.268
Tan	12	34543801	34544100	0.053	0.401	0.082	0.086
Tan	12	34585801	34586100	0.134	0.466	0.218	0.193
Tan	12	34848001	34848300	2.020	2.783	1.835	1.912
Tan	12	34909501	34909800	0.081	0.305	0.506	0.193
Tan	12	35328001	35328300	0.053	0.753	0.296	0.223
Tan	12	35696101	35696400	0.344	0.772	0.395	0.254
Tan	12	36096301	36096600	0.158	0.444	0.156	0.106
Tan	12	36858601	36858900	0.319	0.163	0.526	0.360

Tan	12	37665601	37665900	0.294	0.539	0.234	0.061
Tan	12	38083501	38083800	2.578	3.060	1.665	1.871
Tan	12	38629501	38629800	0.161	0.326	0.506	0.056
Tan	12	39059701	39060000	0.345	0.140	0.341	0.553
Tan	12	39144001	39144300	0.186	0.515	0.239	0.268
Tan	12	39246001	39246300	0.637	1.620	0.983	0.898
Tan	12	39437401	39437700	0.106	0.395	0.103	0.086
Tan	12	39529801	39530100	0.161	0.607	0.239	0.051
Tan	12	39584101	39584400	0.398	0.824	0.350	0.355
Tan	12	39586501	39586800	0.398	1.008	0.535	0.558
Tan	12	39630901	39631200	1.355	1.002	1.391	0.608
Tan	12	39665401	39665700	0.319	0.749	0.288	0.340
Tan	12	39791701	39792000	0.823	1.405	0.724	0.867
Tan	12	40140601	40140900	1.300	1.798	1.300	0.913
Tan	12	40374301	40374600	0.292	0.493	0.185	0.329
Tan	12	40450501	40450800	0.663	1.573	0.896	0.938
Tan	12	40612801	40613100	0.534	0.914	0.296	0.614
Tan	12	40721401	40721700	0.132	0.444	0.267	0.248
Tan	12	40760401	40760700	0.158	0.515	0.263	0.243
Tan	12	40791301	40791600	0.773	1.077	0.510	0.669
Tan	12	41009701	41010000	1.432	2.551	1.942	2.236
Tan	12	41153701	41154000	0.962	1.759	0.531	0.953
Tan	12	41156701	41157000	0.340	0.908	0.424	0.416
Tan	12	41223901	41224200	0.215	0.605	0.234	0.248
Tan	12	41258701	41259000	1.034	1.585	0.925	0.877
Tan	12	41484601	41484900	0.105	0.729	0.247	0.279
Tan	12	41551501	41551800	0.584	0.845	0.374	0.466
Tan	12	41564101	41564400	0.106	0.515	0.205	0.248
Tan	12	41626501	41626800	0.502	0.916	0.477	0.385
Tan	12	41927701	41928000	2.589	1.081	2.994	3.037
Tan	12	42180301	42180600	0.265	0.564	0.156	0.187
Tan	12	42345901	42346200	0.663	0.983	0.588	0.690
Tan	12	42381301	42381600	3.376	2.899	3.763	3.255
Tan	12	42687901	42688200	0.292	0.867	0.243	0.274
Tan	12	42690601	42690900	0.156	0.702	0.136	0.238
Tan	12	42696301	42696600	0.797	1.480	0.749	0.816
Tan	12	42730201	42730500	0.905	1.519	0.838	1.075
Tan	12	42795901	42796200	1.297	1.942	1.329	1.095
Tan	12	42934501	42934800	0.931	0.303	0.481	0.614
Tan	12	43194901	43195200	0.398	0.702	0.205	0.223
Tan	12	43244101	43244400	1.066	2.311	1.125	0.968
Tan	12	43313101	43313400	0.665	0.796	0.506	0.279
Tan	12	43441201	43441500	0.053	0.465	0.214	0.086
Tan	12	43455301	43455600	0.234	0.981	0.000	0.076
Tan	12	43491301	43491600	0.294	0.607	0.477	0.441
Tan	12	43493701	43494000	0.750	1.905	0.700	0.948
Tan	12	43845001	43845300	0.106	0.401	0.054	0.142
Tan	12	43906801	43907100	0.132	0.418	0.111	0.081
Tan	12	43947301	43947600	0.240	0.564	0.263	0.167
Tan	12	44024101	44024400	0.532	1.268	0.749	0.695
Tan	12	44051401	44051700	1.092	0.543	1.505	1.161
Tan	12	44331901	44332200	1.166	2.051	1.190	1.125
Tan	12	44448901	44449200	1.036	1.238	0.901	0.963
Tan	12	44693401	44693700	0.213	0.773	0.205	0.309
Tan	12	44706901	44707200	0.266	0.871	0.234	0.167
Tan	12	44788501	44788800	0.347	1.706	0.967	0.481
Tan	12	44808001	44808300	0.079	0.420	0.078	0.076
Tan	12	44823901	44824200	0.400	0.888	0.263	0.452
Tan	12	44869201	44869500	0.239	0.491	0.160	0.218
Tan	12	44955301	44955600	5.080	5.422	3.961	4.214
Tan	12	44960401	44960700	3.960	3.250	2.851	3.746
Tan	12	44995801	44996100	0.747	1.289	0.317	0.513
Tan	12	45087301	45087600	0.613	1.309	0.655	0.573
Tan	12	45124201	45124500	0.344	1.008	0.312	0.325
Tan	12	45164101	45164400	0.848	1.641	0.667	0.902
Tan	12	45321601	45321900	0.428	0.047	0.477	0.431
Tan	12	45427201	45427500	0.426	0.727	0.428	0.553

Tan	12	45429001	45429300	0.263	1.032	0.239	0.320
Tan	12	45446401	45446700	0.958	1.736	0.818	1.050
Tan	12	45616801	45617100	0.484	1.433	0.350	0.405
Tan	12	45693301	45693600	0.318	1.954	0.321	1.024
Tan	12	45696301	45696600	0.666	1.408	0.687	0.553
Tan	12	45834901	45835200	0.429	1.129	0.263	0.254
Tan	12	45835201	45835500	0.448	1.743	0.497	0.588
Tan	12	46015801	46016100	0.132	0.603	0.054	0.187
Tan	12	46056301	46056600	0.186	0.493	0.165	0.198
Tan	12	46143901	46144200	3.252	2.066	3.201	3.741
Tan	12	46264201	46264500	0.397	0.140	0.399	0.533
Tan	12	46841401	46841700	1.060	1.961	0.996	1.059
Tan	12	46851001	46851300	0.136	0.586	0.239	0.274
Tan	12	47046601	47046900	0.450	1.073	0.428	0.315
Tan	12	47124001	47124300	0.824	0.983	1.324	1.217
Tan	12	47224801	47225100	0.958	0.303	0.798	0.796
Tan	12	47430901	47431200	3.405	1.551	2.673	2.698
Tan	12	47436901	47437200	0.718	1.214	0.580	0.695
Tan	12	47499901	47500200	0.373	0.538	0.321	0.254
Tan	12	47557801	47558100	3.212	1.751	3.476	3.189
Tan	12	47709001	47709300	2.794	2.122	3.830	2.819
Tan	12	47831401	47831700	0.665	0.957	0.667	0.725
Tan	12	47856301	47856600	2.099	1.755	1.222	1.348
Tan	12	47871001	47871300	0.294	0.118	0.691	0.248
Tan	12	48117001	48117300	0.316	0.680	0.296	0.137
Tan	12	48161401	48161700	2.152	1.468	1.995	1.831
Tan	12	48161701	48162000	1.707	0.643	1.670	1.998
Tan	12	48162301	48162600	0.853	2.905	1.080	1.241
Tan	12	48162601	48162900	0.373	1.498	0.346	0.426
Tan	12	48176401	48176700	0.824	0.725	0.370	0.578
Tan	12	48202501	48202800	0.160	0.914	0.136	0.401
Tan	12	48223501	48223800	1.305	0.538	0.769	0.056
Tan	12	48317701	48318000	0.610	1.551	0.863	0.690
Tan	12	48416101	48416400	0.132	0.775	0.156	0.081
Tan	12	48458401	48458700	1.671	0.691	1.801	2.185
Tan	12	48551101	48551400	1.087	1.948	0.860	0.978
Tan	12	48731101	48731400	0.694	1.407	0.831	0.842
Tan	12	48995701	48996000	0.557	1.493	0.774	0.700
Tan	12	49053901	49054200	0.318	0.910	0.308	0.421
Tan	12	49081201	49081500	0.213	0.536	0.210	0.031
Tan	12	49195201	49195500	0.318	0.139	0.477	0.329
Tan	12	49294801	49295100	2.610	1.817	2.731	2.641
Tan	12	49741201	49741500	0.744	1.101	0.646	0.664
Tan	12	49845601	49845900	3.260	1.961	3.134	3.239
Tan	12	49925101	49925400	0.211	0.468	0.107	0.173
Tan	12	50271001	50271300	2.078	3.120	1.472	1.901
Tan	12	50468101	50468400	0.292	0.468	0.346	0.335
Tan	12	50509501	50509800	0.106	0.423	0.127	0.106
Tan	12	50638501	50638800	0.371	0.749	0.337	0.329
Tan	12	50703301	50703600	0.266	0.539	0.165	0.218
Tan	12	51290101	51290400	1.882	0.626	1.431	1.840
Tan	12	51444601	51444900	0.210	0.611	0.239	0.173
Tan	12	51444901	51445200	0.208	0.772	0.230	0.031
Tan	12	51620701	51621000	0.265	0.744	0.185	0.248
Tan	12	51700801	51701100	0.584	1.126	0.613	0.857
Tan	12	51841501	51841800	1.915	1.122	1.567	2.165
Tan	12	51890701	51891000	0.134	0.446	0.267	0.162
Tan	12	51922801	51923100	0.479	1.002	0.481	0.492
Tan	12	51923101	51923400	0.344	1.073	0.395	0.512
Tan	12	51923401	51923700	0.079	0.423	0.082	0.051
Tan	12	51945301	51945600	0.106	0.494	0.127	0.112
Tan	12	52073101	52073400	0.292	0.656	0.350	0.218
Tan	12	52077001	52077300	0.345	0.869	0.230	0.304
Tan	12	52300501	52300800	0.771	0.584	0.802	0.335
Tan	12	52689601	52689900	0.026	0.371	0.111	0.112
Tan	12	53117101	53117400	1.303	1.824	1.411	1.658
Tan	12	53428801	53429100	0.161	0.517	0.111	0.218

Tan	12	53530501	53530800	0.211	0.768	0.259	0.248
Tan	12	53868301	53868600	0.106	0.420	0.189	0.279
Tan	12	54111601	54111900	0.319	0.562	0.210	0.268
Tan	12	54368101	54368400	0.744	1.866	0.869	0.710
Tan	12	54378601	54378900	0.560	1.009	0.395	0.664
Tan	12	54412801	54413100	0.613	0.935	0.510	0.527
Tan	12	54570601	54570900	7.679	6.291	8.589	9.025
Tan	12	54577801	54578100	0.426	0.631	0.346	0.441
Tan	12	54621001	54621300	0.536	1.300	0.531	0.436
Tan	12	54705301	54705600	0.905	1.937	1.440	1.288
Tan	12	54916201	54916500	1.008	0.633	0.959	0.380
Tan	12	55060801	55061100	0.215	0.721	0.205	0.284
Tan	12	55116301	55116600	0.132	0.586	0.210	0.259
Tan	12	55116601	55116900	0.132	0.562	0.160	0.198
Tan	12	55184101	55184400	1.625	0.818	0.481	1.739
Tan	12	55737001	55737300	0.397	1.008	0.531	0.639
Tan	12	56040601	56040900	1.568	2.242	1.494	1.607
Tan	12	56095501	56095800	1.223	1.899	1.135	1.435
Tan	12	56352601	56352900	1.013	2.133	1.159	1.323
Tan	12	56463901	56464200	0.240	0.513	0.136	0.248
Tan	12	56469601	56469900	0.239	0.657	0.210	0.238
Tan	12	56648101	56648400	0.905	0.513	0.687	0.279
Tan	12	57365701	57366000	0.026	0.257	0.502	0.137
Tan	12	57737701	57738000	0.532	0.047	0.399	0.461
Tan	12	57866401	57866700	2.921	2.176	2.809	2.855
Tan	12	57991201	57991500	3.268	3.551	2.706	2.794
Tan	12	58366801	58367100	1.170	2.191	1.460	1.165
Tan	12	58441801	58442100	2.500	3.059	2.040	2.632
Tan	12	58695901	58696200	1.604	2.611	1.329	1.096
Tan	12	58729801	58730100	0.532	0.888	0.337	0.446
Tan	12	59093101	59093400	1.034	1.470	0.575	0.887
Tan	12	59361601	59361900	0.531	0.234	0.428	0.472
Tan	12	59413801	59414100	0.503	0.747	0.292	0.173
Tan	12	59424901	59425200	1.276	0.513	0.847	1.039
Tan	12	59744401	59744700	0.266	0.841	0.247	0.152
Tan	12	59765701	59766000	0.586	0.747	0.448	0.416
Tan	12	60100201	60100500	0.582	1.030	0.642	0.745
Tan	12	60105001	60105300	2.205	2.875	1.563	1.593
Tan	12	60146101	60146400	0.156	1.002	0.058	0.081
Tan	12	60159901	60160200	0.184	0.442	0.103	0.137
Tan	12	60181801	60182100	1.382	1.753	1.451	1.105
Tan	12	60238501	60238800	2.123	2.854	1.663	2.150
Tan	12	60262201	60262500	0.055	0.418	0.054	0.106
Tan	12	60327001	60327300	1.145	1.821	1.016	1.111
Tan	12	60327301	60327600	2.157	3.604	1.554	2.155
Tan	12	60877201	60877500	0.479	0.749	0.374	0.385
Tan	12	61204801	61205100	0.455	2.178	0.495	0.426
Tan	12	61205101	61205400	0.428	1.099	0.292	0.223
Tan	12	61287901	61288200	0.531	0.940	0.267	0.391
Tan	12	61819201	61819500	0.268	0.586	0.239	0.279
Tan	12	62232001	62232300	3.158	4.630	3.591	3.878
Tan	12	62317501	62317800	0.158	0.491	0.029	0.117
Tan	12	62387101	62387400	2.471	3.817	1.897	2.663
Tan	12	62387401	62387700	2.926	4.181	2.102	2.820
Tan	12	62493001	62493300	0.210	0.918	0.140	0.142
Tan	12	62524201	62524500	0.532	0.258	0.695	0.659
Tan	12	62765701	62766000	0.637	1.032	0.403	0.573
Tan	12	63227101	63227400	0.106	0.395	0.132	0.061
Tan	12	63304501	63304800	0.690	1.124	0.954	0.882
Tan	12	63331501	63331800	0.105	0.564	1.057	0.213
Tan	12	63452101	63452400	0.156	0.886	0.272	0.167
Tan	12	63601201	63601500	0.132	0.702	0.210	0.187
Tan	12	63622201	63622500	0.082	0.588	0.049	0.056
Tan	12	63771601	63771900	0.400	0.890	0.522	0.477
Tan	12	63802201	63802500	1.620	2.723	1.382	1.485
Tan	12	63832201	63832500	0.929	1.356	0.609	0.862
Tan	12	63858301	63858600	0.132	0.442	0.185	0.132

Tan	12	63962701	63963000	0.239	0.465	0.132	0.193
Tan	12	64143001	64143300	0.160	0.611	0.247	0.193
Tan	12	64186501	64186800	0.237	0.657	1.086	1.146
Tan	12	64202401	64202700	0.508	1.082	0.510	0.436
Tan	12	64234201	64234500	0.452	0.562	0.370	0.279
Tan	12	65829001	65829300	0.477	1.120	0.580	0.639
Tan	12	66842401	66842700	1.197	1.914	1.090	1.207
Tan	12	67053601	67053900	0.637	1.195	0.464	0.690
Tan	12	67092601	67092900	1.350	6.883	2.513	2.030
Tan	12	67234801	67235100	2.050	1.543	1.921	2.545
Tan	12	67311901	67312200	0.742	1.360	0.909	0.857
Tan	12	67410601	67410900	0.803	1.714	0.769	0.720
Tan	12	67515601	67515900	1.515	2.053	0.959	1.532
Tan	12	67515901	67516200	1.358	2.079	0.945	1.582
Tan	12	67545901	67546200	0.450	1.079	0.288	0.426
Tan	12	67546201	67546500	0.163	1.245	0.312	0.330
Tan	12	67596601	67596900	0.132	0.538	0.263	0.081
Tan	12	67901701	67902000	1.029	2.690	0.707	1.259
Tan	12	68165101	68165400	0.027	0.446	0.165	0.198
Tan	12	68183701	68184000	0.560	0.798	0.160	0.309
Tan	12	68241901	68242200	1.010	1.755	0.638	0.995
Tan	12	68278501	68278800	1.700	2.695	1.618	1.044
Tan	12	68449801	68450100	1.518	2.409	1.625	1.383
Tan	12	68687101	68687400	0.345	0.676	0.370	0.396
Tan	12	68715301	68715600	0.905	0.421	0.745	0.893
Tan	12	68735701	68736000	1.166	2.090	0.823	1.121
Tan	12	68817301	68817600	0.720	0.539	0.321	0.360
Tan	12	68910601	68910900	0.077	0.493	0.074	0.106
Tan	12	69255601	69255900	4.786	2.992	5.067	4.762
Tan	12	69300001	69300300	4.275	5.010	3.344	3.732
Tan	12	69962401	69962700	0.665	1.290	0.711	0.578
Tan	12	69962701	69963000	0.929	1.725	0.749	0.679
Tan	12	70055701	70056000	0.637	1.079	0.593	0.350
Tan	12	70062301	70062600	0.857	1.496	0.604	0.502
Tan	12	70160401	70160700	0.081	0.745	0.156	0.324
Tan	12	70175401	70175700	0.398	0.584	0.321	0.391
Tan	12	70398901	70399200	0.319	0.773	0.374	0.405
Tan	12	70474801	70475100	0.079	0.541	0.160	0.132
Tan	12	70616701	70617000	0.503	0.140	0.395	0.391
Tan	12	70660501	70660800	0.824	1.193	0.774	0.887
Tan	12	71119801	71120100	1.513	0.236	1.037	0.877
Tan	12	71239801	71240100	0.266	1.143	0.234	1.850
Tan	12	71301601	71301900	0.665	0.890	0.687	0.502
Tan	12	71406001	71406300	0.319	0.678	0.448	0.385
Tan	12	71427301	71427600	0.689	0.983	0.366	0.502
Tan	12	71605501	71605800	0.558	1.200	0.564	0.573
Tan	12	71625001	71625300	2.155	0.300	0.896	1.532
Tan	12	71630101	71630400	0.929	1.124	0.555	0.497
Tan	12	71693101	71693400	4.646	3.791	4.641	2.646
Tan	12	71761501	71761800	0.690	1.290	0.774	0.675
Tan	12	71840701	71841000	1.013	1.665	0.740	1.111
Tan	12	71898001	71898300	0.397	0.882	0.350	0.279
Tan	12	71959201	71959500	0.718	1.573	0.497	0.999
Tan	12	72143101	72143400	0.290	0.560	0.107	0.360
Tan	12	72267001	72267300	1.561	2.804	1.103	1.126
Tan	12	72519001	72519300	0.507	1.313	0.305	0.375
Tan	12	72636001	72636300	2.502	3.040	2.307	1.719
Tan	12	73080601	73080900	0.131	0.583	0.078	0.112
Tan	12	73090801	73091100	1.783	2.768	1.351	1.435
Tan	12	73096201	73096500	3.965	3.478	2.604	3.042
Tan	12	73175101	73175400	0.349	1.289	0.765	0.913
Tan	12	73260301	73260600	0.347	0.611	0.288	0.223
Tan	12	73782301	73782600	0.397	0.071	0.584	0.355
Tan	12	73798801	73799100	0.479	1.169	0.156	0.720
Tan	12	73856701	73857000	0.374	1.764	0.519	0.513
Tan	12	73872901	73873200	0.507	0.843	0.419	0.309
Tan	12	74005801	74006100	0.108	0.420	0.160	0.162

Tan	12	74130601	74130900	0.558	0.912	0.633	0.391
Tan	12	74232901	74233200	0.345	0.863	0.234	0.264
Tan	12	74345701	74346000	0.347	0.961	0.230	0.268
Tan	12	74407801	74408100	2.020	2.691	1.817	1.552
Tan	12	74514301	74514600	0.402	1.082	0.437	0.385
Tan	12	74573101	74573400	0.131	0.511	0.185	0.248
Tan	12	74638201	74638500	0.313	1.036	0.234	0.248
Tan	12	74723101	74723400	0.213	0.511	0.111	0.193
Tan	12	74781901	74782200	3.326	5.825	3.139	3.513
Tan	12	74792401	74792700	0.636	1.051	0.374	0.644
Tan	12	74838001	74838300	0.216	0.609	0.189	0.254
Tan	12	74847901	74848200	0.586	0.935	0.189	0.405
Tan	12	75036301	75036600	1.252	1.847	1.427	0.963
Tan	12	75122401	75122700	1.384	1.686	1.329	1.328
Tan	12	75139501	75139800	0.636	1.845	0.606	0.680
Tan	12	75151501	75151800	0.081	0.330	0.477	0.167
Tan	12	75233401	75233700	0.373	0.656	0.288	0.340
Tan	12	75240901	75241200	0.292	0.539	0.317	0.355
Tan	12	75306301	75306600	1.008	1.569	1.222	1.029
Tan	12	75306601	75306900	0.160	0.515	0.399	0.223
Tan	12	75358201	75358500	6.088	4.518	7.223	6.565
Tan	12	75719401	75719700	2.045	3.306	1.436	1.779
Tan	12	75726601	75726900	3.715	0.316	3.247	3.350
Tan	12	75726901	75727200	0.926	0.049	1.048	1.151
Tan	12	75777301	75777600	0.660	1.257	0.379	0.659
Tan	12	75793201	75793500	0.531	0.328	0.111	0.112
Tan	12	75865501	75865800	0.668	1.075	0.604	0.558
Tan	12	75943201	75943500	0.503	0.938	0.317	0.396
Tan	12	75977701	75978000	1.645	3.174	1.522	1.304
Tan	12	76181701	76182000	0.161	0.612	0.160	0.025
Tan	12	76242001	76242300	0.318	1.110	0.272	0.309
Tan	12	76355101	76355400	3.914	2.328	4.407	4.091
Tan	12	76557301	76557600	0.186	0.442	0.263	0.056
Tan	12	76656901	76657200	0.958	1.984	1.251	1.172
Tan	12	76750501	76750800	4.770	3.705	2.069	2.727
Tan	12	76884301	76884600	1.194	2.004	0.517	0.674
Tan	12	76910401	76910700	7.792	5.329	7.184	7.852
Tan	12	77137501	77137800	1.857	1.124	2.040	2.003
Tan	12	77165401	77165700	0.266	0.611	0.263	0.360
Tan	12	77245501	77245800	5.473	4.184	4.989	5.070
Tan	12	77401201	77401500	0.482	1.032	0.477	0.329
Tan	12	77422801	77423100	1.453	2.590	1.197	1.400
Tan	12	77689501	77689800	0.026	0.446	0.160	0.223
Tan	12	77694601	77694900	0.292	0.706	0.321	0.248
Tan	12	77715301	77715600	1.465	0.848	0.321	1.587
Tan	12	77858401	77858700	6.114	4.649	5.578	5.932
Tan	12	77877301	77877600	0.424	0.938	0.642	0.522
Tan	12	77878501	77878800	0.932	1.238	0.925	0.882
Tan	12	77930701	77931000	8.277	5.617	7.361	7.069
Tan	12	77985901	77986200	2.851	4.583	2.825	2.840
Tan	12	78107701	78108000	0.663	1.867	0.473	0.755
Tan	12	78178801	78179100	0.766	1.217	0.424	0.360
Tan	12	78273001	78273300	2.079	3.143	2.062	1.845
Tan	12	78806401	78806700	0.828	0.251	0.905	0.745
Tan	12	78843001	78843300	2.397	1.560	2.151	2.135
Tan	12	78896101	78896400	3.399	2.431	3.411	3.478
Tan	13	466201	466500	0.744	0.914	0.662	0.497
Tan	13	1079701	1080000	0.186	0.442	0.189	0.223
Tan	13	1576801	1577100	4.412	5.589	3.243	4.052
Tan	13	1724401	1724700	0.344	0.729	0.272	0.254
Tan	13	1746601	1746900	0.053	0.491	0.239	0.117
Tan	13	1992301	1992600	0.347	0.631	1.032	0.441
Tan	13	2763301	2763600	0.266	0.538	0.292	0.167
Tan	13	3129901	3130200	0.266	0.588	0.272	0.223
Tan	13	3279301	3279600	0.266	0.725	0.292	0.547
Tan	13	3376501	3376800	0.289	0.725	0.234	0.329
Tan	13	3377101	3377400	2.260	2.547	1.810	2.130

Tan	13	3421801	3422100	0.026	0.517	0.078	0.101
Tan	13	3753301	3753600	0.160	0.491	0.194	0.031
Tan	13	3814501	3814800	0.160	0.581	0.165	0.173
Tan	13	3936601	3936900	0.347	0.234	0.477	0.385
Tan	13	4182601	4182900	0.314	0.727	0.321	0.274
Tan	13	4191301	4191600	0.321	0.680	0.218	0.132
Tan	13	4303801	4304100	0.584	0.466	0.263	0.081
Tan	13	4377601	4377900	0.319	0.210	0.317	0.522
Tan	13	4485301	4485600	0.347	0.702	0.239	0.132
Tan	13	4495201	4495500	0.931	1.710	0.584	0.877
Tan	13	4779601	4779900	0.263	0.682	0.292	0.254
Tan	13	4898101	4898400	3.400	4.074	3.311	2.814
Tan	13	5329201	5329500	0.718	1.588	1.090	0.801
Tan	13	5331901	5332200	0.108	0.611	0.140	0.193
Tan	13	5339101	5339400	0.136	0.682	1.099	1.050
Tan	13	5990701	5991000	0.690	1.289	0.267	0.446
Tan	13	6117601	6117900	0.395	0.961	0.448	0.553
Tan	13	6334801	6335100	7.209	5.005	6.828	7.976
Tan	13	6391501	6391800	0.848	1.214	0.580	0.740
Tan	13	6520501	6520800	0.589	1.547	0.497	0.649
Tan	13	6636001	6636300	0.189	0.818	0.205	0.416
Tan	13	6638701	6639000	0.160	0.470	0.214	0.137
Tan	13	6778201	6778500	1.089	2.163	0.760	0.562
Tan	13	6865801	6866100	0.285	1.330	0.289	0.284
Tan	13	6927301	6927600	1.071	2.530	0.941	0.973
Tan	13	6999901	7000200	0.213	0.395	0.185	0.218
Tan	13	7281601	7281900	0.799	0.328	0.662	0.720
Tan	13	7799101	7799400	0.213	0.442	0.136	0.218
Tan	13	7819801	7820100	0.108	0.631	0.321	0.309
Tan	13	8154001	8154300	1.010	0.584	1.217	0.411
Tan	13	8458501	8458800	0.876	1.148	0.535	0.771
Tan	13	8707501	8707800	0.186	0.539	0.107	0.112
Tan	13	8954701	8955000	0.053	0.347	0.029	0.031
Tan	13	8977801	8978100	1.616	2.572	1.632	1.856
Tan	13	9157801	9158100	0.265	0.466	0.132	0.167
Tan	13	9240001	9240300	0.586	0.701	0.424	0.329
Tan	13	9325201	9325500	0.266	0.466	0.288	0.248
Tan	13	9337501	9337800	0.187	0.000	0.234	0.715
Tan	13	9587101	9587400	0.721	1.363	0.526	0.497
Tan	13	9594301	9594600	0.982	0.539	0.881	1.044
Tan	13	9679201	9679500	0.720	1.678	0.682	1.192
Tan	13	9726601	9726900	0.744	1.697	0.383	0.324
Tan	13	9732901	9733200	1.518	3.386	1.920	1.095
Tan	13	9801301	9801600	0.215	0.539	0.296	0.223
Tan	13	9815401	9815700	3.172	1.521	3.292	3.600
Tan	13	10110301	10110600	0.134	0.659	0.107	0.198
Tan	13	10387201	10387500	0.239	0.676	0.189	0.025
Tan	13	10398301	10398600	0.184	0.701	0.379	0.329
Tan	13	10539601	10539900	0.160	0.680	0.432	0.360
Tan	13	10608001	10608300	0.744	1.144	0.575	0.553
Tan	13	10824001	10824300	0.108	0.513	0.025	0.031
Tan	13	11082301	11082600	0.458	1.984	0.325	0.726
Tan	13	11134501	11134800	0.186	0.751	0.185	0.112
Tan	13	11148001	11148300	1.886	1.476	2.307	1.805
Tan	13	11166901	11167200	0.186	0.517	0.185	0.274
Tan	13	11175601	11175900	4.304	2.025	3.725	3.453
Tan	13	11220901	11221200	0.216	0.723	0.296	0.360
Tan	13	11514901	11515200	2.738	1.444	2.499	3.204
Tan	13	11693701	11694000	0.450	0.796	0.366	0.391
Tan	13	11697301	11697600	0.211	0.773	0.214	0.167
Tan	13	11847301	11847600	0.373	0.818	0.350	0.517
Tan	13	11921701	11922000	0.928	1.433	0.419	0.553
Tan	13	11926501	11926800	1.842	0.719	1.835	2.220
Tan	13	11988301	11988600	0.426	0.633	0.292	0.466
Tan	13	12045301	12045600	0.374	0.629	0.341	0.335
Tan	13	12265201	12265500	0.637	1.244	0.662	0.913
Tan	13	12435301	12435600	0.158	0.674	0.312	0.137

Tan	13	12537001	12537300	1.180	3.220	1.129	1.288
Tan	13	12615601	12615900	0.929	1.568	0.762	0.628
Tan	13	12936601	12936900	0.187	0.444	0.210	0.279
Tan	13	13148701	13149000	0.215	0.536	0.127	0.051
Tan	13	13666801	13667100	0.211	0.489	0.074	0.056
Tan	13	13680601	13680900	4.107	5.911	3.168	3.698
Tan	13	13681201	13681500	1.745	3.064	2.143	2.185
Tan	13	14568901	14569200	0.132	0.448	0.074	0.112
Tan	13	14751601	14751900	0.160	0.423	0.082	0.162
Tan	13	14800801	14801100	0.211	0.446	0.127	0.137
Tan	13	14805901	14806200	0.160	0.489	0.160	0.173
Tan	13	14930701	14931000	0.477	0.938	0.477	0.431
Tan	13	15021001	15021300	0.347	0.985	0.214	0.228
Tan	13	15042301	15042600	0.211	0.491	0.210	0.173
Tan	13	15109801	15110100	0.800	1.427	0.925	0.806
Tan	13	15131701	15132000	0.615	1.324	0.720	0.472
Tan	13	15241501	15241800	1.457	2.613	1.741	1.592
Tan	13	15439201	15439500	0.373	0.657	0.029	0.248
Tan	13	15507301	15507600	0.240	0.468	0.210	0.137
Tan	13	15559501	15559800	1.407	2.791	0.979	1.446
Tan	13	15563401	15563700	0.294	0.583	0.078	0.304
Tan	13	15632401	15632700	0.266	0.867	0.288	0.517
Tan	13	15667501	15667800	1.647	2.504	1.505	1.714
Tan	13	15864001	15864300	0.586	1.358	0.867	0.943
Tan	13	15877801	15878100	0.503	0.841	0.312	0.446
Tan	13	15880801	15881100	0.294	0.538	0.156	0.187
Tan	13	15883801	15884100	0.447	1.672	0.720	0.720
Tan	13	15901201	15901500	0.105	0.766	0.198	0.218
Tan	13	16002601	16002900	0.239	0.466	0.292	0.198
Tan	13	16006201	16006500	0.186	0.564	0.132	0.193
Tan	13	16326301	16326600	0.819	1.309	1.893	0.756
Tan	13	16392601	16392900	0.640	0.139	0.428	0.466
Tan	13	16480801	16481100	5.824	4.997	5.999	6.693
Tan	13	16502101	16502400	1.194	0.766	1.324	1.683
Tan	13	16580401	16580700	0.081	0.446	0.029	0.056
Tan	13	16583101	16583400	0.132	0.420	0.234	0.056
Tan	13	16612501	16612800	0.265	0.704	0.165	0.299
Tan	13	16692901	16693200	0.237	0.513	0.156	0.254
Tan	13	16776901	16777200	0.108	0.442	0.054	0.142
Tan	13	16791001	16791300	0.263	0.562	0.239	0.193
Tan	13	16912501	16912800	0.936	1.665	0.662	0.979
Tan	13	16912801	16913100	0.561	1.304	0.292	0.381
Tan	13	16940401	16940700	1.887	3.017	1.886	2.110
Tan	13	16956001	16956300	0.690	1.575	0.642	0.872
Tan	13	16956301	16956600	0.160	0.723	0.165	0.193
Tan	13	17161801	17162100	0.131	0.446	0.160	0.198
Tan	13	17177101	17177400	0.110	0.560	0.132	0.117
Tan	13	17180101	17180400	0.581	1.219	0.662	0.695
Tan	13	17885401	17885700	2.954	2.131	2.624	2.774
Tan	13	18288601	18288900	0.210	0.890	0.147	0.329
Tan	13	18388801	18389100	0.476	1.122	0.185	0.329
Tan	13	18534901	18535200	0.903	0.515	1.206	0.852
Tan	13	18536701	18537000	0.477	1.028	0.403	0.583
Tan	13	18553801	18554100	0.186	0.635	0.160	0.025
Tan	13	18563401	18563700	0.160	0.635	0.107	0.162
Tan	13	18595201	18595500	0.189	0.929	0.189	0.202
Tan	13	18911401	18911700	0.369	0.656	0.082	0.142
Tan	13	19260001	19260300	0.266	0.702	0.419	0.274
Tan	13	19341601	19341900	0.055	0.371	0.107	0.056
Tan	13	19491601	19491900	0.055	0.395	0.054	0.051
Tan	13	20070301	20070600	0.960	1.332	0.818	0.988
Tan	13	20141701	20142000	1.116	1.781	1.255	1.410
Tan	13	20416501	20416800	0.426	0.843	0.444	0.238
Tan	13	20545801	20546100	0.136	0.729	0.201	0.223
Tan	13	21174001	21174300	0.132	0.515	0.239	0.142
Tan	13	21174301	21174600	0.373	0.562	0.296	0.167
Tan	13	21327601	21327900	0.053	0.919	0.247	0.106

Tan	13	21349801	21350100	0.529	0.794	0.321	0.411
Tan	13	21376501	21376800	1.228	2.724	0.956	1.374
Tan	13	21409501	21409800	0.321	0.770	0.276	0.248
Tan	13	21445201	21445500	1.355	0.933	1.407	1.298
Tan	13	21524701	21525000	0.105	0.517	0.123	0.112
Tan	13	21661801	21662100	0.398	1.122	0.464	0.492
Tan	13	21843601	21843900	0.081	0.395	0.132	0.142
Tan	13	21898501	21898800	0.453	0.822	0.379	0.446
Tan	13	22046401	22046700	2.738	2.946	2.312	2.657
Tan	13	22605301	22605600	0.321	1.032	0.370	0.619
Tan	13	22665301	22665600	0.211	0.465	0.189	0.137
Tan	13	22710901	22711200	0.131	0.517	0.160	0.137
Tan	13	22791301	22791600	1.249	1.781	0.856	1.328
Tan	13	22841701	22842000	0.634	1.435	0.379	0.350
Tan	13	23019901	23020200	0.213	0.564	0.181	0.304
Tan	13	23458501	23458800	0.347	0.867	0.230	0.268
Tan	13	23458801	23459100	0.215	0.701	0.205	0.173
Tan	13	23754001	23754300	0.557	0.959	0.473	0.466
Tan	13	23758501	23758800	0.852	0.654	0.424	0.421
Tan	13	23777101	23777400	0.502	1.429	0.473	0.659
Tan	13	24223501	24223800	0.587	0.961	0.588	0.553
Tan	13	24226801	24227100	0.424	0.865	0.296	0.578
Tan	13	24357001	24357300	0.186	0.397	0.181	0.583
Tan	13	24380401	24380700	0.621	1.944	0.959	0.254
Tan	13	24380701	24381000	0.508	1.686	0.979	0.452
Tan	13	24398401	24398700	1.539	2.362	1.830	1.987
Tan	13	24533101	24533400	1.360	1.939	1.291	1.227
Tan	13	24779701	24780000	0.426	0.871	0.341	0.360
Tan	13	24991501	24991800	6.349	4.540	6.425	7.056
Tan	13	25096501	25096800	0.479	0.886	0.361	0.335
Tan	13	25301401	25301700	0.160	0.753	0.259	0.243
Tan	13	25605001	25605300	1.171	2.083	1.239	1.491
Tan	13	25990801	25991100	0.505	0.865	0.473	0.441
Tan	13	26592601	26592900	0.215	0.588	0.103	0.025
Tan	13	26863501	26863800	0.397	0.938	0.346	0.376
Tan	13	27007501	27007800	0.797	1.569	1.188	0.731
Tan	13	27018301	27018600	3.325	1.912	2.827	3.498
Tan	13	27226201	27226500	3.029	1.384	2.718	2.520
Tan	13	27281701	27282000	0.266	0.470	0.156	0.137
Tan	13	27323401	27323700	0.134	0.420	0.078	0.051
Tan	13	28262401	28262700	1.036	1.729	0.778	1.029
Tan	13	28262701	28263000	1.005	1.611	0.983	0.756
Tan	13	28267201	28267500	0.000	0.590	0.098	0.117
Tan	13	28279801	28280100	0.160	0.517	0.054	0.112
Tan	13	28484701	28485000	1.281	0.723	1.313	1.379
Tan	13	28505701	28506000	3.519	2.259	3.237	3.524
Tan	13	28730101	28730400	0.210	0.562	0.189	0.137
Tan	13	29066701	29067000	0.265	0.584	0.205	0.187
Tan	13	29283901	29284200	0.053	0.401	0.107	0.137
Tan	13	29306101	29306400	0.745	0.356	0.831	1.465
Tan	13	29340901	29341200	0.134	0.446	0.210	0.218
Tan	13	29518501	29518800	0.982	1.335	0.568	0.745
Tan	13	29749801	29750100	0.584	1.504	0.633	0.933
Tan	13	29775301	29775600	0.452	0.952	0.399	0.472
Tan	13	30360901	30361200	0.398	0.867	0.390	0.426
Tan	13	30702901	30703200	0.187	0.468	0.074	0.198
Tan	13	30720001	30720300	0.344	1.047	0.272	0.502
Tan	13	31171501	31171800	0.184	0.493	0.160	0.167
Tan	13	32031301	32031600	0.692	1.736	0.740	0.457
Tan	13	32094301	32094600	0.482	0.912	0.473	0.522
Tan	13	32289001	32289300	0.481	0.963	0.292	0.396
Tan	13	32360401	32360700	0.240	0.564	0.267	0.228
Tan	13	32439601	32439900	0.026	0.466	0.054	0.193
Tan	13	32695501	32695800	3.320	1.577	3.687	3.377
Tan	13	32695801	32696100	2.852	1.105	3.383	3.271
Tan	13	32787301	32787600	0.608	1.118	0.593	0.558
Tan	13	32907001	32907300	0.745	0.936	0.506	0.634

Tan	13	33001801	33002100	4.889	2.126	5.640	4.437
Tan	13	33002101	33002400	3.799	1.948	3.727	3.514
Tan	13	33006301	33006600	2.226	0.770	1.900	1.906
Tan	13	33006601	33006900	2.221	0.699	1.830	1.871
Tan	13	33029701	33030000	3.050	4.141	3.168	2.702
Tan	13	33510901	33511200	0.692	0.470	0.210	0.279
Tan	13	33516001	33516300	0.850	1.169	0.847	0.675
Tan	13	33552901	33553200	0.134	0.753	0.317	0.248
Tan	13	33788401	33788700	0.244	1.337	0.535	0.527
Tan	13	33903901	33904200	0.053	0.212	0.477	0.365
Tan	13	33905101	33905400	1.016	2.126	1.473	1.435
Tan	13	33905401	33905700	1.705	3.879	2.071	2.054
Tan	13	33913201	33913500	0.393	0.863	0.370	0.248
Tan	13	34116301	34116600	1.140	1.895	0.885	1.055
Tan	13	34147201	34147500	2.713	1.431	2.439	2.464
Tan	13	34172401	34172700	5.312	4.297	5.488	5.330
Tan	13	34172701	34173000	1.812	2.287	1.612	1.800
Tan	13	34176601	34176900	0.426	0.846	0.419	0.299
Tan	13	34340401	34340700	0.342	1.032	0.259	0.411
Tan	13	34380301	34380600	1.784	2.341	1.358	1.496
Tan	13	34593001	34593300	2.667	3.501	2.443	2.758
Tan	13	34606801	34607100	0.319	0.446	0.239	0.248
Tan	13	34933201	34933500	0.610	1.233	0.473	0.391
Tan	13	35340301	35340600	0.507	1.146	0.531	0.649
Tan	13	35340601	35340900	0.558	0.935	0.370	0.553
Tan	13	35513701	35514000	0.979	2.523	0.945	1.770
Tan	13	35541601	35541900	0.079	0.867	0.123	0.167
Tan	13	35541901	35542200	0.235	1.313	0.160	0.207
Tan	13	35575801	35576100	0.000	0.420	0.111	0.051
Tan	13	35685901	35686200	0.105	0.612	0.132	0.101
Tan	13	36043201	36043500	0.213	0.511	0.074	0.142
Tan	13	36305401	36305700	2.533	0.806	2.027	2.378
Tan	13	36357001	36357300	0.561	1.150	0.622	0.639
Tan	13	36546301	36546600	0.240	0.468	0.136	0.243
Tan	13	36791101	36791400	0.106	0.534	0.185	0.223
Tan	13	36792901	36793200	0.271	2.098	0.769	0.710
Tan	13	36885901	36886200	0.186	0.493	0.107	0.213
Tan	13	36964501	36964800	0.481	0.185	0.424	0.441
Tan	13	36973201	36973500	2.094	2.716	1.061	1.840
Tan	13	37021201	37021500	0.558	0.845	0.214	0.391
Tan	13	37031101	37031400	1.571	2.573	1.628	1.729
Tan	13	37154101	37154400	0.292	0.607	0.234	0.167
Tan	13	37197001	37197300	0.052	0.420	0.210	0.137
Tan	13	37373401	37373700	0.132	1.006	0.403	0.583
Tan	13	37395901	37396200	1.197	1.588	1.144	0.857
Tan	13	38018101	38018400	0.215	0.657	0.185	0.259
Tan	13	38488501	38488800	0.665	0.981	0.593	0.614
Tan	13	38498101	38498400	1.015	0.654	0.346	0.193
Tan	13	38527501	38527800	0.160	0.704	0.403	0.193
Tan	13	38532001	38532300	0.242	0.772	0.312	0.234
Tan	13	38571601	38571900	0.294	0.586	0.296	0.243
Tan	13	38687101	38687400	0.237	0.916	0.288	0.436
Tan	13	38806801	38807100	1.896	2.879	2.102	1.866
Tan	13	38906101	38906400	0.239	0.491	0.205	0.218
Tan	13	39000001	39000300	0.347	0.538	0.263	0.355
Tan	13	39024901	39025200	1.042	1.830	1.003	0.918
Tan	13	39031501	39031800	2.554	2.221	1.843	1.937
Tan	13	39093601	39093900	2.174	4.197	1.915	2.217
Tan	13	39396301	39396600	0.026	0.421	0.156	0.801
Tan	13	39446101	39446400	0.319	0.800	0.453	0.355
Tan	13	39692101	39692400	0.292	0.446	0.132	0.081
Tan	13	39765301	39765600	0.137	0.725	0.239	0.112
Tan	13	39765601	39765900	0.211	0.607	0.082	0.051
Tan	13	39871201	39871500	0.077	0.468	0.029	0.193
Tan	13	39895801	39896100	1.036	1.238	0.448	0.614
Tan	13	39940801	39941100	0.160	0.444	0.156	0.173
Tan	13	40367701	40368000	0.986	0.629	1.148	1.354

Tan	13	40437001	40437300	0.161	0.745	0.189	0.238
Tan	13	40437901	40438200	0.163	0.867	0.214	0.365
Tan	13	40797301	40797600	0.587	0.888	0.403	0.558
Tan	13	40843501	40843800	0.318	0.701	0.140	0.248
Tan	13	40845901	40846200	1.968	1.362	1.893	1.947
Tan	13	40853701	40854000	0.611	1.051	0.769	0.329
Tan	13	40869601	40869900	0.079	0.466	0.132	0.193
Tan	13	40898101	40898400	0.452	1.030	0.317	0.664
Tan	13	41166001	41166300	0.026	0.395	0.107	0.086
Tan	13	41286001	41286300	0.237	0.611	0.243	0.228
Tan	13	41424601	41424900	2.047	2.783	1.674	1.880
Tan	13	41483701	41484000	0.294	0.491	0.054	0.335
Tan	13	41501101	41501400	0.213	0.444	0.181	0.218
Tan	13	41511901	41512200	1.089	1.332	0.802	0.806
Tan	13	41685901	41686200	3.720	4.489	3.890	2.945
Tan	13	41755501	41755800	0.769	1.302	0.691	0.502
Tan	13	41767201	41767500	0.503	1.056	0.205	0.400
Tan	13	41927401	41927700	0.450	1.658	0.477	1.278
Tan	13	41931601	41931900	0.158	0.491	0.160	0.248
Tan	13	42102001	42102300	0.266	0.515	0.058	0.360
Tan	13	42382801	42383100	1.008	1.543	0.876	1.227
Tan	13	42582301	42582600	1.353	0.978	1.329	0.533
Tan	13	42745201	42745500	0.507	0.656	0.502	0.360
Tan	13	42955801	42956100	0.555	1.219	0.724	0.502
Tan	13	43120501	43120800	0.505	1.094	0.448	0.594
Tan	13	43401601	43401900	0.665	0.938	0.535	0.553
Tan	13	43521001	43521300	0.134	0.401	0.049	0.137
Tan	13	43604101	43604400	0.186	0.420	0.074	0.142
Tan	13	43625701	43626000	0.292	0.629	0.346	0.421
Tan	13	43626001	43626300	0.371	0.515	0.267	0.279
Tan	13	43730401	43730700	0.429	0.890	0.312	0.355
Tan	13	43766101	43766400	1.303	2.015	1.222	1.516
Tan	13	43771801	43772100	0.584	0.773	0.341	0.527
Tan	13	44187601	44187900	1.542	2.107	1.605	2.585
Tan	13	44203801	44204100	1.489	2.367	1.612	1.232
Tan	13	44228701	44229000	0.319	0.893	0.103	0.254
Tan	13	44319901	44320200	1.568	3.974	1.993	1.283
Tan	13	44325901	44326200	0.745	1.714	1.217	1.080
Tan	13	44326201	44326500	0.563	1.765	0.577	0.411
Tan	13	44478001	44478300	3.909	2.573	2.896	3.737
Tan	13	44505601	44505900	0.318	0.491	0.317	0.360
Tan	13	44505901	44506200	0.529	0.980	0.535	0.634
Tan	13	44868901	44869200	0.292	0.538	0.214	0.751
Tan	13	45125101	45125400	0.371	0.792	0.234	0.284
Tan	13	45125401	45125700	1.013	2.435	0.825	0.927
Tan	13	45284701	45285000	1.324	0.442	1.371	1.293
Tan	13	45290701	45291000	0.268	0.656	0.301	0.051
Tan	13	45468601	45468900	0.158	0.395	0.136	0.137
Tan	13	45534301	45534600	1.570	0.699	1.003	1.141
Tan	13	45569101	45569400	0.266	0.444	0.346	0.162
Tan	13	46243501	46243800	0.876	1.635	1.066	0.781
Tan	13	46243801	46244100	0.848	1.446	0.925	0.761
Tan	13	46317901	46318200	0.237	0.755	0.267	0.218
Tan	13	46874101	46874400	0.105	0.444	0.160	0.248
Tan	13	47182501	47182800	0.105	0.583	0.078	0.081
Tan	13	47182801	47183100	0.160	0.420	0.054	0.081
Tan	13	47579101	47579400	1.412	0.959	1.494	1.820
Tan	13	47825101	47825400	0.295	0.751	0.194	0.198
Tan	13	48472201	48472500	0.718	1.167	0.691	0.492
Tan	13	48868801	48869100	0.239	0.423	0.054	0.142
Tan	13	48913801	48914100	0.294	0.629	0.272	0.228
Tan	13	50005801	50006100	0.663	0.865	0.588	0.279
Tan	13	50908801	50909100	0.507	0.839	0.374	0.380
Tan	13	51114001	51114300	0.134	0.466	0.214	0.279
Tan	13	51219001	51219300	0.319	0.118	0.341	0.578
Tan	13	51438901	51439200	0.211	0.373	0.638	0.553
Tan	13	51449701	51450000	0.374	0.704	0.243	0.162

Tan	13	51984901	51985200	0.639	1.075	0.497	0.533
Tan	13	52112401	52112700	0.319	0.538	0.078	0.198
Tan	13	52155001	52155300	0.824	0.935	0.720	0.522
Tan	13	52530001	52530300	0.319	0.818	0.481	0.279
Tan	13	52641901	52642200	0.958	2.171	1.226	1.014
Tan	13	53192101	53192400	0.292	0.682	0.399	0.360
Tan	13	53194201	53194500	0.239	0.468	0.267	0.329
Tan	13	53231101	53231400	0.586	1.573	0.765	0.700
Tan	13	53709001	53709300	0.344	0.862	0.288	0.452
Tan	13	53744401	53744700	0.424	0.938	0.234	0.533
Tan	13	53757001	53757300	0.371	1.032	0.662	0.309
Tan	13	53875201	53875500	0.160	0.517	0.181	0.198
Tan	13	53991001	53991300	0.883	2.023	0.580	0.756
Tan	13	54042001	54042300	1.200	2.483	1.587	1.618
Tan	13	54048901	54049200	1.086	0.350	1.008	0.796
Tan	13	54284401	54284700	0.745	0.347	0.765	0.821
Tan	13	54449401	54449700	0.718	0.888	0.580	0.578
Tan	13	54451201	54451500	0.053	0.448	0.078	0.081
Tan	13	54616501	54616800	0.452	0.212	0.658	0.446
Tan	13	54663301	54663600	2.152	2.894	2.198	1.845
Tan	13	54683401	54683700	0.477	1.004	0.798	0.776
Tan	13	54747601	54747900	0.106	0.446	0.267	0.274
Tan	13	54754801	54755100	0.616	1.234	0.564	0.452
Tan	13	54921601	54921900	1.647	2.223	0.765	1.476
Tan	13	54983401	54983700	0.452	0.654	0.428	0.497
Tan	13	54991801	54992100	0.187	0.444	0.214	0.254
Tan	13	55130401	55130700	0.639	0.096	0.317	0.335
Tan	13	55173601	55173900	0.106	0.423	0.078	0.137
Tan	13	55416001	55416300	0.345	1.225	0.531	0.492
Tan	13	55427701	55428000	0.186	0.609	0.292	0.076
Tan	13	55690501	55690800	0.373	0.468	0.210	0.304
Tan	13	55773601	55773900	1.779	0.781	1.942	1.836
Tan	13	55773901	55774200	1.907	0.974	2.053	1.967
Tan	13	56194801	56195100	1.170	0.910	1.362	1.217
Tan	13	56389801	56390100	0.531	0.281	0.160	0.553
Tan	13	56562601	56562900	4.805	7.076	4.639	4.862
Tan	13	56691301	56691600	0.531	0.910	0.716	0.527
Tan	13	56943901	56944200	1.087	2.122	1.015	1.075
Tan	13	57024001	57024300	0.134	0.395	0.049	0.112
Tan	13	57089401	57089700	1.783	2.410	1.480	1.647
Tan	13	57108901	57109200	0.373	0.633	0.292	0.248
Tan	13	57225001	57225300	0.105	0.423	0.103	0.117
Tan	13	57299101	57299400	0.319	0.680	0.419	0.441
Tan	13	57361201	57361500	0.957	1.504	0.497	0.745
Tan	13	57607501	57607800	0.957	1.122	1.489	0.943
Tan	13	57792901	57793200	0.716	0.255	0.745	0.705
Tan	13	57849601	57849900	0.134	0.401	0.107	0.081
Tan	13	57955201	57955500	0.666	1.841	0.604	0.603
Tan	13	58013401	58013700	0.342	0.910	0.181	0.142
Tan	13	58013701	58014000	0.529	1.240	0.382	0.324
Tan	13	58114801	58115100	1.037	0.421	0.321	0.882
Tan	13	58228801	58229100	0.053	1.214	2.633	1.886
Tan	13	58231201	58231500	0.400	0.212	0.506	0.391
Tan	13	58434601	58434900	0.268	0.794	0.330	0.137
Tan	13	58497001	58497300	0.452	1.053	0.593	0.598
Tan	13	58551901	58552200	0.720	0.375	0.531	0.497
Tan	13	58644901	58645200	0.507	0.908	0.531	0.391
Tan	13	59333101	59333400	0.053	0.446	0.243	0.142
Tan	13	59440801	59441100	4.764	2.702	5.020	3.813
Tan	13	59441101	59441400	4.544	1.826	5.663	3.655
Tan	13	59764201	59764500	0.081	0.515	0.132	0.335
Tan	13	59828401	59828700	0.371	0.513	0.132	0.274
Tan	13	59831701	59832000	0.213	0.680	0.296	0.147
Tan	13	59858701	59859000	0.424	0.656	0.292	0.254
Tan	13	59873401	59873700	0.263	0.860	0.243	0.182
Tan	13	59873701	59874000	0.216	1.294	0.325	0.334
Tan	13	60307801	60308100	0.186	0.468	0.292	0.243

Tan	13	60537001	60537300	1.595	2.828	1.558	1.972
Tan	13	60595201	60595500	2.233	3.665	1.972	2.525
Tan	13	60776101	60776400	0.237	0.562	0.296	0.193
Tan	13	60974701	60975000	0.747	1.030	0.662	0.608
Tan	13	60996601	60996900	1.623	2.294	1.864	1.369
Tan	13	61046401	61046700	0.161	0.586	0.214	0.385
Tan	13	61161601	61161900	0.582	1.714	0.645	0.755
Tan	13	61201201	61201500	0.161	0.468	0.160	0.086
Tan	13	61205401	61205700	0.318	0.560	0.292	0.162
Tan	13	61230301	61230600	0.156	0.446	0.132	0.000
Tan	13	61317001	61317300	0.769	1.480	0.453	0.562
Tan	13	61328401	61328700	3.715	4.885	3.818	3.696
Tan	13	61383301	61383600	0.668	0.348	0.987	0.882
Tan	13	61524001	61524300	0.294	0.657	0.160	0.324
Tan	13	61568401	61568700	0.186	0.515	0.267	0.086
Tan	13	61659001	61659300	0.240	0.588	0.103	0.248
Tan	13	61842001	61842300	0.160	0.654	0.337	0.304
Tan	13	62150401	62150700	0.531	0.189	0.399	0.391
Tan	13	62239201	62239500	0.611	1.379	0.881	0.893
Tan	13	62352601	62352900	0.477	0.820	0.580	0.279
Tan	13	62407501	62407800	0.613	0.908	0.555	0.385
Tan	13	62637901	62638200	1.039	1.384	0.609	1.044
Tan	13	63765301	63765600	0.161	0.841	0.341	0.329
Tan	13	63795301	63795600	0.453	0.167	0.473	0.553
Tan	13	63971101	63971400	0.716	1.311	1.037	0.852
Tan	13	64192801	64193100	0.160	0.517	0.239	0.187
Tan	13	64550701	64551000	0.186	0.423	0.160	0.137
Tan	13	64639501	64639800	0.295	0.611	0.243	0.279
Tan	13	64684201	64684500	3.847	4.787	3.578	3.595
Tan	13	64793401	64793700	0.507	0.373	0.239	0.578
Tan	13	64994401	64994700	0.106	0.466	0.074	0.142
Tan	13	65267101	65267400	0.184	0.719	0.205	0.051
Tan	13	65277601	65277900	0.292	1.272	0.357	0.340
Tan	13	65367001	65367300	0.557	1.030	0.510	0.533
Tan	13	65421001	65421300	0.398	0.633	0.370	0.335
Tan	13	65624101	65624400	0.721	1.287	0.798	0.679
Tan	13	65763601	65763900	1.170	1.888	1.057	1.217
Tan	13	65790901	65791200	0.344	1.148	0.259	0.391
Tan	13	65841001	65841300	0.240	0.792	0.448	0.112
Tan	13	65887801	65888100	0.532	0.397	0.239	0.112
Tan	13	65938801	65939100	1.992	1.403	0.987	1.982
Tan	13	66036001	66036300	0.668	1.762	0.967	0.948
Tan	13	66098701	66099000	0.826	0.605	0.292	0.355
Tan	13	66301201	66301500	0.477	0.068	0.374	0.365
Tan	13	66525901	66526200	0.105	0.539	0.127	0.198
Tan	13	66572701	66573000	0.560	0.963	0.243	0.583
Tan	13	67031101	67031400	0.271	1.103	0.383	0.411
Tan	13	67176301	67176600	3.568	4.533	3.234	3.544
Tan	13	67444501	67444800	1.250	0.600	1.534	2.018
Tan	13	67536601	67536900	0.027	0.446	0.181	0.198
Tan	13	67656001	67656300	2.157	2.528	2.098	1.764
Tan	13	67658101	67658400	0.848	1.807	0.624	0.776
Tan	13	68027101	68027400	0.292	0.560	0.346	0.309
Tan	13	68092801	68093100	1.276	1.592	1.217	1.131
Tan	13	68231701	68232000	0.052	0.232	0.049	0.527
Tan	13	68543101	68543400	0.426	0.652	0.477	0.832
Tan	13	68664301	68664600	0.321	0.772	0.308	0.243
Tan	13	68664601	68664900	0.481	0.845	0.210	0.218
Tan	13	68762701	68763000	0.268	0.607	0.132	0.329
Tan	13	68903701	68904000	0.161	0.395	0.107	0.167
Tan	13	69004201	69004500	1.276	0.510	1.209	1.141
Tan	13	69028801	69029100	1.032	1.732	1.041	1.029
Tan	13	69070501	69070800	1.199	0.612	1.522	0.228
Tan	13	69136801	69137100	0.555	1.470	0.571	0.518
Tan	13	69166201	69166500	1.170	1.498	0.749	1.100
Tan	13	69238501	69238800	0.318	0.609	0.370	0.887
Tan	13	69283201	69283500	0.079	0.444	0.058	0.142

Tan	13	69357301	69357600	0.239	0.628	0.181	0.223
Tan	13	69424801	69425100	1.547	0.255	1.264	0.852
Tan	13	69589201	69589500	1.539	1.148	1.543	0.659
Tan	13	69663001	69663300	0.213	0.564	0.189	0.309
Tan	13	69725401	69725700	2.873	4.049	3.068	1.754
Tan	13	69749401	69749700	0.136	0.446	0.132	0.137
Tan	13	69966901	69967200	0.452	0.654	0.239	0.324
Tan	13	70170301	70170600	1.516	0.888	1.387	1.566
Tan	13	70368901	70369200	0.716	0.185	0.716	0.472
Tan	13	70800901	70801200	1.061	0.425	0.950	1.120
Tan	13	70888801	70889100	0.187	0.773	0.218	0.051
Tan	13	71177401	71177700	0.265	0.511	0.214	0.198
Tan	13	71453701	71454000	0.292	0.612	0.214	0.279
Tan	13	71528401	71528700	0.263	0.813	0.272	0.187
Tan	13	71684401	71684700	0.715	1.665	0.794	0.882
Tan	13	71881501	71881800	0.429	1.150	0.395	0.547
Tan	13	71962501	71962800	0.240	0.539	0.000	0.187
Tan	13	72004501	72004800	6.600	5.181	6.329	6.673
Tan	13	72094501	72094800	0.928	1.663	0.945	0.979
Tan	13	72246301	72246600	0.160	0.536	0.127	0.228
Tan	13	72309001	72309300	0.292	0.444	0.317	0.274
Tan	13	72672901	72673200	0.426	1.172	0.662	0.664
Tan	13	72837301	72837600	0.931	1.075	0.827	1.273
Tan	13	72894601	72894900	1.734	2.716	1.409	1.653
Tan	13	72905401	72905700	0.424	0.772	0.317	0.279
Tan	13	73024201	73024500	0.345	0.725	0.395	0.203
Tan	13	73261801	73262100	1.907	2.849	1.266	1.288
Tan	13	73297801	73298100	0.160	0.491	0.181	0.025
Tan	13	73584001	73584300	0.156	0.751	0.145	0.198
Tan	13	73851901	73852200	1.292	2.864	1.284	1.191
Tan	13	73890001	73890300	0.105	0.513	0.078	0.122
Tan	13	73937101	73937400	0.081	0.420	0.107	0.223
Tan	13	74055001	74055300	0.239	0.538	0.185	0.335
Tan	13	74121601	74121900	4.525	3.710	4.501	4.578
Tan	13	74121901	74122200	4.335	3.860	4.093	4.259
Tan	13	74459101	74459400	0.186	0.423	0.132	0.031
Tan	13	74462701	74463000	0.883	1.867	1.197	0.974
Tan	13	74624701	74625000	1.381	0.957	1.576	1.420
Tan	13	74878501	74878800	0.718	1.150	0.457	0.751
Tan	13	74886301	74886600	0.637	0.399	0.662	0.634
Tan	13	74902501	74902800	5.696	3.943	5.851	6.009
Tan	13	74913901	74914200	0.558	1.433	0.852	0.842
Tan	13	74974801	74975100	0.268	0.586	0.263	0.268
Tan	13	74995501	74995800	1.041	0.519	0.983	0.963
Tan	13	75095701	75096000	0.184	0.515	0.189	0.167
Tan	13	75212701	75213000	0.532	0.185	0.374	0.385
Tan	13	75492601	75492900	2.015	4.328	6.991	2.429
Tan	13	75759301	75759600	0.637	0.959	0.716	0.751
Tan	13	75778501	75778800	3.615	4.853	3.899	2.586
Tan	13	75865201	75865500	1.195	1.474	0.831	0.522
Tan	13	75999301	75999600	0.584	0.185	0.428	0.391
Tan	13	76051201	76051500	2.660	1.748	2.789	2.820
Tan	13	76460701	76461000	5.824	3.816	1.197	1.934
Tan	13	76609201	76609500	0.239	0.672	0.234	0.198
Tan	13	76797301	76797600	4.736	6.362	4.752	4.334
Tan	13	76840201	76840500	0.876	1.285	0.876	0.948
Tan	13	76995001	76995300	2.763	1.500	2.176	2.884
Tan	13	77177701	77178000	1.008	1.856	0.724	0.892
Tan	13	77185501	77185800	1.966	2.223	2.555	1.764
Tan	13	77202301	77202600	1.916	1.009	1.884	1.445
Tan	13	77203501	77203800	0.665	0.444	0.181	0.254
Tan	13	77328901	77329200	0.692	0.959	0.662	0.740
Tan	13	77494501	77494800	0.240	0.511	0.132	0.254
Tan	13	77634601	77634900	1.274	1.075	0.798	0.659
Tan	13	77655301	77655600	1.254	1.543	0.847	1.080
Tan	13	77796901	77797200	1.065	0.676	1.003	0.994
Tan	13	77811901	77812200	0.958	2.393	1.308	1.414

Tan	13	77847601	77847900	0.558	1.313	0.510	0.791
Tan	13	77855101	77855400	1.812	1.030	1.703	1.693
Tan	13	78088501	78088800	1.802	3.543	1.857	2.216
Tan	13	78227101	78227400	0.477	0.933	0.379	0.533
Tan	13	78246601	78246900	2.720	4.283	2.918	3.058
Tan	13	78321901	78322200	1.671	0.761	1.739	2.221
Tan	13	78463801	78464100	0.371	0.633	0.403	0.304
Tan	13	78560701	78561000	1.039	1.403	1.012	0.781
Tan	13	78662401	78662700	1.010	1.568	0.905	1.688
Tan	13	78780301	78780600	1.405	0.395	1.233	1.444
Tan	13	78797401	78797700	5.580	3.962	5.395	5.629
Tan	13	78827701	78828000	0.398	0.895	0.403	0.411
Tan	13	78850501	78850800	0.797	1.077	0.901	0.913
Tan	13	78855601	78855900	2.263	2.942	2.176	1.678
Tan	13	78872401	78872700	0.852	0.279	0.424	0.745
Tan	13	78969901	78970200	6.165	4.259	6.470	6.820
Tan	13	79000801	79001100	0.507	0.818	0.531	0.391
Tan	13	79034701	79035000	5.033	3.420	4.113	4.913
Tan	13	79153201	79153500	1.544	0.873	0.379	1.359
Tan	13	79181701	79182000	3.151	5.001	3.504	3.635
Tan	13	79213801	79214100	3.883	3.021	3.578	4.127
Tan	13	79217701	79218000	1.166	2.079	1.048	1.247
Tan	13	79241401	79241700	1.516	1.918	1.066	1.496
Tan	13	79500301	79500600	0.852	1.659	1.152	1.131
Tan	13	79662901	79663200	1.808	1.079	1.930	1.770
Tan	13	79700101	79700400	0.611	2.556	0.354	1.466
Tan	13	79881001	79881300	0.345	0.586	0.272	0.329
Tan	13	80048401	80048700	0.079	0.470	0.103	0.248
Tan	13	80326501	80326800	0.403	0.890	0.370	0.259
Tan	13	80352601	80352900	0.105	0.491	0.239	0.304
Tan	13	80406301	80406600	5.740	7.202	5.950	5.714
Tan	13	80427901	80428200	2.171	3.796	1.615	2.014
Tan	13	80456401	80456700	0.745	0.395	0.609	0.664
Tan	13	80993401	80993700	0.373	0.964	0.444	0.446
Tan	13	80995801	80996100	0.215	0.611	0.210	0.173
Tan	13	81051901	81052200	0.934	2.105	1.206	1.233
Tan	13	81080701	81081000	0.531	1.217	0.671	0.603
Tan	13	81200101	81200400	1.333	1.639	0.934	1.080
Tan	13	81290401	81290700	0.187	0.751	0.152	0.391
Tan	13	81372001	81372300	0.529	1.581	0.390	0.405
Tan	13	81430501	81430800	0.502	1.034	0.497	0.497
Tan	13	81430801	81431100	0.474	1.124	0.531	0.497
Tan	13	81434401	81434700	1.041	2.461	0.049	1.029
Tan	13	81434701	81435000	0.907	2.017	0.221	0.866
Tan	13	81439501	81439800	9.069	7.205	6.730	6.693
Tan	13	81808501	81808800	0.584	1.141	0.415	0.472
Tan	13	81810901	81811200	0.507	0.890	0.667	0.558
Tan	13	81870901	81871200	0.240	0.888	0.247	0.441
Tan	13	82045201	82045500	1.778	0.830	2.666	1.967
Tan	13	82137601	82137900	0.771	1.053	0.774	0.213
Tan	13	82200001	82200300	4.652	1.562	2.742	3.140
Tan	13	82318501	82318800	6.111	3.295	6.622	6.434
Tan	13	82363801	82364100	1.039	1.663	0.736	1.217
Tan	13	82625701	82626000	0.477	0.770	0.419	0.522
Tan	13	82730701	82731000	1.462	2.075	1.353	1.343
Tan	13	82736701	82737000	0.984	1.678	0.613	0.913
Tan	13	82769401	82769700	1.994	1.586	2.036	2.048
Tan	13	83020201	83020500	0.611	1.053	0.798	0.882
Tan	14	290401	290700	0.000	1.025	0.270	0.000
Tan	14	564301	564600	4.125	2.467	3.961	4.122
Tan	14	564901	565200	1.803	2.911	1.950	1.759
Tan	14	597601	597900	1.515	2.032	2.782	0.882
Tan	14	615301	615600	4.506	2.736	4.389	4.507
Tan	14	743701	744000	1.805	2.388	1.879	1.647
Tan	14	777601	777900	0.581	1.452	0.403	0.355
Tan	14	789301	789600	0.557	2.910	0.754	0.654
Tan	14	798301	798600	2.015	0.891	1.654	2.104

Tan	14	838501	838800	1.405	2.313	1.431	1.039
Tan	14	891301	891600	0.131	1.575	0.205	0.142
Tan	14	891601	891900	0.132	0.536	0.156	0.061
Tan	14	903601	903900	0.774	0.210	0.872	0.968
Tan	14	917701	918000	0.766	2.305	1.152	0.537
Tan	14	920701	921000	0.235	0.562	0.214	0.193
Tan	14	926101	926400	1.034	0.215	1.019	0.907
Tan	14	935101	935400	0.213	0.633	0.205	0.335
Tan	14	1034401	1034700	0.694	1.588	0.921	1.014
Tan	14	1098301	1098600	0.452	1.176	0.432	0.533
Tan	14	1260301	1260600	0.105	0.611	0.181	0.360
Tan	14	1332901	1333200	2.531	3.607	2.385	2.662
Tan	14	1333201	1333500	2.313	2.944	2.091	2.155
Tan	14	1439401	1439700	0.586	0.210	0.132	0.137
Tan	14	1471801	1472100	1.466	2.991	1.434	1.723
Tan	14	1519801	1520100	0.321	0.751	0.292	0.228
Tan	14	2010601	2010900	1.937	2.890	1.460	1.622
Tan	14	2178301	2178600	0.294	0.584	0.243	0.213
Tan	14	2233501	2233800	1.013	1.661	0.983	1.141
Tan	14	2267701	2268000	0.211	0.541	0.181	0.223
Tan	14	2447401	2447700	0.239	0.493	0.243	0.304
Tan	14	2555101	2555400	0.215	0.699	0.189	0.238
Tan	14	2755201	2755500	0.158	0.564	0.296	0.274
Tan	14	3064501	3064800	0.960	0.208	0.840	0.994
Tan	14	3415501	3415800	0.931	1.523	1.008	0.933
Tan	14	3805501	3805800	2.129	3.914	2.592	2.809
Tan	14	4051201	4051500	0.960	1.804	0.930	0.826
Tan	14	4485601	4485900	0.294	0.446	0.239	0.223
Tan	14	4570201	4570500	0.186	0.538	0.292	0.335
Tan	14	4589701	4590000	0.769	0.253	0.753	0.832
Tan	14	4720501	4720800	0.773	1.541	0.959	0.700
Tan	14	4839901	4840200	0.187	0.519	0.160	0.137
Tan	14	4987501	4987800	0.584	1.008	0.588	0.522
Tan	14	5082301	5082600	3.107	3.898	4.911	4.736
Tan	14	5096701	5097000	0.134	0.826	0.165	0.178
Tan	14	5108401	5108700	0.608	1.126	0.330	0.527
Tan	14	5314201	5314500	2.394	2.892	2.378	1.577
Tan	14	5350501	5350800	1.757	2.437	1.694	1.445
Tan	14	5350801	5351100	1.758	2.573	2.118	1.744
Tan	14	5376901	5377200	3.330	4.392	2.633	3.849
Tan	14	5476801	5477100	0.984	1.358	1.066	1.192
Tan	14	5571001	5571300	2.502	3.568	2.588	1.662
Tan	14	5574601	5574900	1.342	3.413	1.430	0.923
Tan	14	5614201	5614500	1.326	2.113	1.181	1.384
Tan	14	5658001	5658300	0.373	0.820	0.341	0.405
Tan	14	5755501	5755800	2.154	2.691	1.884	2.297
Tan	14	5759101	5759400	2.044	3.296	1.694	2.388
Tan	14	5863801	5864100	0.584	0.208	0.588	0.522
Tan	14	5914201	5914500	0.960	1.714	0.987	0.847
Tan	14	5955601	5955900	0.397	0.845	0.560	0.329
Tan	14	6049501	6049800	1.731	3.222	1.318	1.865
Tan	14	6064201	6064500	3.237	1.465	3.350	3.579
Tan	14	6067201	6067500	1.274	1.472	0.695	0.913
Tan	14	6389701	6390000	0.210	0.656	0.189	0.304
Tan	14	6555001	6555300	0.132	0.657	0.346	0.193
Tan	14	6650701	6651000	1.037	1.594	0.847	0.796
Tan	14	6675601	6675900	0.692	1.120	0.539	0.857
Tan	14	6827401	6827700	7.129	2.864	6.222	6.386
Tan	14	6857701	6858000	0.453	1.058	0.296	0.436
Tan	14	7386601	7386900	8.216	7.659	7.058	9.339
Tan	14	7405201	7405500	0.371	0.845	0.403	0.522
Tan	14	7482901	7483200	1.150	1.912	1.230	1.161
Tan	14	7489801	7490100	2.614	3.723	2.465	2.733
Tan	14	7530001	7530300	4.235	7.091	5.079	4.405
Tan	14	7579801	7580100	2.257	3.012	1.957	1.572
Tan	14	7734901	7735200	0.666	1.375	0.671	0.796
Tan	14	7743601	7743900	6.333	7.174	5.961	6.302

Tan	14	7770601	7770900	1.199	1.543	0.778	1.212
Tan	14	7879501	7879800	1.702	3.192	1.687	1.572
Tan	14	8016901	8017200	0.666	0.444	0.218	0.167
Tan	14	8106601	8106900	0.105	0.425	0.107	0.000
Tan	14	8158201	8158500	1.257	2.504	0.963	1.293
Tan	14	8339701	8340000	0.665	0.120	0.642	0.542
Tan	14	8447701	8448000	0.371	0.766	0.399	0.198
Tan	14	8596501	8596800	1.192	2.055	1.494	1.460
Tan	14	8831701	8832000	0.158	0.468	0.185	0.167
Tan	14	9034501	9034800	0.265	0.468	0.267	0.031
Tan	14	9040501	9040800	0.237	0.605	0.165	0.248
Tan	14	9136201	9136500	0.186	0.444	0.160	0.218
Tan	14	9310201	9310500	0.611	1.240	0.502	0.350
Tan	14	9316501	9316800	0.726	2.600	0.749	0.690
Tan	14	9316801	9317100	0.240	0.865	0.374	0.456
Tan	14	9321301	9321600	0.265	0.871	0.210	0.101
Tan	14	9536701	9537000	0.110	0.727	0.132	0.223
Tan	14	9656401	9656700	1.994	1.493	1.933	1.181
Tan	14	9780001	9780300	0.265	0.466	0.210	0.162
Tan	14	9822301	9822600	1.462	0.678	1.275	1.253
Tan	14	9936901	9937200	0.186	0.494	0.103	0.173
Tan	14	10006801	10007100	0.400	0.987	0.361	0.360
Tan	14	10064401	10064700	0.745	0.378	0.979	1.166
Tan	14	10114501	10114800	0.720	1.766	0.551	0.710
Tan	14	10129501	10129800	1.286	2.465	0.987	1.120
Tan	14	10141501	10141800	0.665	0.210	0.687	0.614
Tan	14	10221301	10221600	0.640	1.929	0.707	0.679
Tan	14	10272901	10273200	0.314	0.912	0.205	0.213
Tan	14	10425601	10425900	0.773	0.515	0.267	0.335
Tan	14	10799701	10800000	2.552	2.311	2.526	2.048
Tan	14	10830601	10830900	1.113	1.663	0.852	1.172
Tan	14	10837201	10837500	0.082	0.489	0.136	0.193
Tan	14	10880101	10880400	0.373	0.187	0.555	0.436
Tan	14	10923601	10923900	0.450	1.006	0.267	0.588
Tan	14	11041801	11042100	0.452	0.114	0.477	0.796
Tan	14	11053501	11053800	1.197	2.624	1.538	1.841
Tan	14	11237101	11237400	0.266	0.963	0.156	0.268
Tan	14	11261101	11261400	0.618	1.755	0.749	0.877
Tan	14	11267101	11267400	0.211	0.539	0.049	0.173
Tan	14	11478901	11479200	5.406	3.282	5.257	4.872
Tan	14	11502601	11502900	0.156	0.493	0.054	0.142
Tan	14	11511901	11512200	4.456	1.257	4.253	3.360
Tan	14	11683801	11684100	2.021	1.307	1.728	1.729
Tan	14	11718001	11718300	3.222	1.928	3.487	3.393
Tan	14	11727301	11727600	0.886	1.663	0.560	0.801
Tan	14	11742001	11742300	0.450	1.030	0.403	0.472
Tan	14	11896501	11896800	1.039	1.736	0.695	1.217
Tan	14	11978401	11978700	0.161	0.631	0.165	0.274
Tan	14	11985301	11985600	2.805	0.571	4.372	2.440
Tan	14	11990101	11990400	5.695	3.544	5.608	5.096
Tan	14	12040501	12040800	1.329	0.775	1.436	1.369
Tan	14	12074401	12074700	2.124	1.191	2.153	2.236
Tan	14	12106801	12107100	5.003	2.618	3.904	4.179
Tan	14	12283201	12283500	0.558	0.305	0.691	0.446
Tan	14	12322201	12322500	0.079	0.423	0.185	0.187
Tan	14	12433801	12434100	0.350	0.773	0.341	0.329
Tan	14	12673801	12674100	3.988	1.727	5.203	3.503
Tan	14	12675601	12675900	0.479	1.116	0.292	0.669
Tan	14	12690301	12690600	0.958	0.511	0.827	0.776
Tan	14	12704701	12705000	0.186	0.470	0.160	0.254
Tan	14	12771001	12771300	2.208	2.836	1.872	1.957
Tan	14	13075501	13075800	0.663	1.006	0.390	0.583
Tan	14	13379701	13380000	0.826	1.238	0.395	0.436
Tan	14	13431601	13431900	4.775	3.194	4.632	4.340
Tan	14	13554001	13554300	0.160	0.560	0.288	0.360
Tan	14	13657801	13658100	0.986	0.279	0.526	0.669
Tan	14	13671301	13671600	0.371	0.654	0.366	0.416

Tan	14	13770001	13770300	2.791	1.626	3.228	2.876
Tan	14	13770901	13771200	3.847	2.294	3.946	3.352
Tan	14	13886401	13886700	4.544	6.293	3.976	4.219
Tan	14	13900501	13900800	0.395	1.032	0.325	0.400
Tan	14	13932301	13932600	0.374	0.584	0.239	0.279
Tan	14	13946101	13946400	0.265	0.725	0.234	0.370
Tan	14	14322601	14322900	1.379	0.583	1.237	1.343
Tan	14	14324101	14324400	0.479	0.654	0.181	0.274
Tan	14	14825701	14826000	0.106	0.515	0.082	0.274
Tan	14	15042601	15042900	0.345	0.702	0.288	0.157
Tan	14	15176701	15177000	0.903	1.639	1.094	0.700
Tan	14	15280501	15280800	0.027	0.493	0.156	0.198
Tan	14	15330601	15330900	0.053	0.442	0.029	0.081
Tan	14	15330901	15331200	0.381	1.144	0.000	0.147
Tan	14	15354001	15354300	0.428	0.987	0.403	0.416
Tan	14	15970201	15970500	0.347	1.317	0.671	0.542
Tan	14	16095901	16096200	0.160	0.444	0.239	0.142
Tan	14	16096201	16096500	0.290	0.747	0.214	0.416
Tan	14	16203901	16204200	1.360	2.663	1.039	1.400
Tan	14	16210201	16210500	4.709	6.368	3.682	5.197
Tan	14	16237801	16238100	0.211	0.493	0.054	0.000
Tan	14	16448101	16448400	0.187	0.584	0.185	0.309
Tan	14	16472101	16472400	0.134	0.886	0.252	0.207
Tan	14	16475401	16475700	0.393	1.813	0.490	0.426
Tan	14	16500001	16500300	0.269	0.657	0.029	0.162
Tan	14	16792201	16792500	0.187	0.515	0.317	0.385
Tan	14	16952701	16953000	0.505	1.328	0.789	0.857
Tan	14	17394901	17395200	0.398	0.515	0.374	0.223
Tan	14	17412601	17412900	0.134	0.466	0.054	0.142
Tan	14	17464201	17464500	0.265	0.725	0.263	0.304
Tan	14	17475601	17475900	6.059	3.592	5.234	4.983
Tan	14	17734801	17735100	0.428	0.747	0.477	0.492
Tan	14	17753101	17753400	0.503	1.127	0.468	0.486
Tan	14	17786401	17786700	0.426	0.560	0.263	0.279
Tan	14	17977501	17977800	0.584	0.142	0.321	0.360
Tan	14	18191701	18192000	1.358	0.485	1.077	1.181
Tan	14	18420601	18420900	0.265	0.560	0.263	0.877
Tan	14	18888901	18889200	0.742	1.783	0.379	0.902
Tan	14	19187101	19187400	0.371	0.702	0.214	0.370
Tan	14	19921501	19921800	0.429	1.272	0.497	0.385
Tan	14	20237701	20238000	0.134	0.418	0.103	0.162
Tan	14	20517301	20517600	4.812	4.205	4.973	5.562
Tan	14	20611801	20612100	1.834	1.427	1.081	1.004
Tan	14	20839801	20840100	0.984	1.878	1.181	0.745
Tan	14	20925001	20925300	0.158	0.418	0.156	0.025
Tan	14	20963401	20963700	0.106	1.011	0.029	0.223
Tan	14	20963701	20964000	0.269	1.298	0.227	0.243
Tan	14	21065101	21065400	0.419	1.315	0.337	0.639
Tan	14	21153901	21154200	0.081	0.444	0.210	0.243
Tan	14	21381301	21381600	1.539	0.721	1.144	1.181
Tan	14	21432601	21432900	0.319	0.727	0.132	0.294
Tan	14	21606301	21606600	0.400	0.515	0.267	0.304
Tan	14	21645901	21646200	0.508	1.687	0.562	0.587
Tan	14	21646201	21646500	1.207	3.332	1.418	1.282
Tan	14	21696001	21696300	0.295	0.863	0.301	0.329
Tan	14	21738301	21738600	0.823	1.283	0.852	0.857
Tan	14	21891601	21891900	0.452	1.028	0.539	0.461
Tan	14	21894901	21895200	0.347	0.538	0.292	0.391
Tan	14	21929101	21929400	0.052	0.723	0.243	0.355
Tan	14	21930601	21930900	0.082	0.513	0.074	0.076
Tan	14	21931201	21931500	0.155	0.657	0.214	0.198
Tan	14	21975001	21975300	0.318	0.629	0.267	0.360
Tan	14	21993001	21993300	0.852	1.425	0.642	0.654
Tan	14	22007101	22007400	0.373	0.656	0.399	0.248
Tan	14	22041601	22041900	3.160	2.437	3.290	3.042
Tan	14	22056601	22056900	0.879	1.740	0.638	0.654
Tan	14	22056901	22057200	0.776	2.058	0.774	0.902

Tan	14	22613101	22613400	0.531	0.935	0.584	0.634
Tan	14	23084701	23085000	0.136	0.981	0.185	0.051
Tan	14	23256901	23257200	0.636	0.985	0.502	0.553
Tan	14	23385901	23386200	0.187	0.539	0.263	0.254
Tan	14	23570701	23571000	0.187	0.493	0.082	0.157
Tan	14	23687701	23688000	0.132	0.680	0.272	0.411
Tan	14	24278101	24278400	0.027	0.513	0.185	0.112
Tan	14	24291901	24292200	0.053	0.515	0.140	0.117
Tan	14	24299101	24299400	0.347	0.656	0.234	0.112
Tan	14	24345001	24345300	0.797	0.418	0.814	0.832
Tan	14	24351301	24351600	1.783	2.197	1.168	1.638
Tan	14	24430501	24430800	0.053	0.401	0.132	0.167
Tan	14	24509101	24509400	0.479	0.142	0.473	0.421
Tan	14	24555301	24555600	0.398	0.676	0.506	0.472
Tan	14	24708901	24709200	0.376	1.219	0.107	0.340
Tan	14	24709201	24709500	0.082	0.468	0.058	0.086
Tan	14	24762901	24763200	0.239	0.423	0.078	0.137
Tan	14	24805801	24806100	0.158	0.725	0.111	0.279
Tan	14	24977401	24977700	0.716	1.004	0.526	0.639
Tan	14	25020301	25020600	0.347	0.817	0.279	0.243
Tan	14	25169101	25169400	2.075	3.326	2.365	1.917
Tan	14	25359301	25359600	0.266	0.704	0.267	0.243
Tan	14	25676101	25676400	0.132	0.448	0.160	0.086
Tan	14	25825801	25826100	0.132	0.517	0.107	0.126
Tan	14	25875001	25875300	1.457	2.440	1.407	1.622
Tan	14	27161401	27161700	0.292	0.790	0.346	0.274
Tan	14	27296401	27296700	0.318	1.006	0.395	0.385
Tan	14	27917401	27917700	0.160	0.446	0.111	0.076
Tan	14	27993901	27994200	0.503	1.077	0.573	0.502
Tan	14	28205701	28206000	0.160	0.987	0.218	0.309
Tan	14	28785001	28785300	0.026	0.491	0.025	0.000
Tan	14	28919101	28919400	0.428	0.820	0.560	0.274
Tan	14	29042401	29042700	0.239	0.470	0.156	0.218
Tan	14	29445301	29445600	0.215	0.466	0.214	0.162
Tan	14	29617801	29618100	0.687	1.054	0.292	0.391
Tan	14	29866201	29866500	0.344	0.772	0.403	0.147
Tan	14	30108601	30108900	0.371	0.817	0.140	0.416
Tan	14	30170701	30171000	0.106	0.626	0.082	0.223
Tan	14	30631801	30632100	0.718	0.515	0.399	0.279
Tan	14	32087401	32087700	0.823	1.148	0.843	0.436
Tan	14	32602801	32603100	0.694	1.073	0.667	0.690
Tan	14	32673901	32674200	2.049	3.208	2.078	2.145
Tan	14	32678101	32678400	0.079	0.418	0.107	0.081
Tan	14	32808901	32809200	2.901	1.847	3.114	2.879
Tan	14	32814301	32814600	0.507	0.893	0.288	0.360
Tan	14	33076201	33076500	2.207	4.085	2.486	2.514
Tan	14	33181501	33181800	0.690	0.981	0.823	0.497
Tan	14	33213601	33213900	1.437	2.182	1.164	1.064
Tan	14	33213901	33214200	1.029	2.058	0.974	0.643
Tan	14	33282001	33282300	0.213	0.541	0.152	0.137
Tan	14	33283201	33283500	1.570	0.612	1.288	1.343
Tan	14	33333901	33334200	0.587	0.258	0.029	0.051
Tan	14	33441301	33441600	0.639	0.096	0.263	0.411
Tan	14	33444901	33445200	6.538	5.447	6.117	6.211
Tan	14	33468601	33468900	0.402	1.124	0.383	0.299
Tan	14	33745201	33745500	0.295	0.938	0.254	0.350
Tan	14	33831301	33831600	0.213	0.607	0.346	0.162
Tan	14	33956101	33956400	1.063	0.817	0.983	1.080
Tan	14	33970201	33970500	1.360	0.440	1.186	1.460
Tan	14	34006501	34006800	0.187	0.489	0.049	0.112
Tan	14	34445401	34445700	0.297	0.942	0.247	0.193
Tan	14	34753201	34753500	0.455	0.987	0.497	0.441
Tan	14	34762501	34762800	2.450	3.036	1.670	1.840
Tan	14	34926901	34927200	1.041	1.800	1.201	0.943
Tan	14	35511901	35512200	0.184	0.541	0.156	0.223
Tan	14	35519401	35519700	0.266	0.513	0.234	0.218
Tan	14	35670001	35670300	1.628	2.365	1.665	1.709

Tan	14	35840401	35840700	0.160	0.564	0.374	0.167
Tan	14	35856601	35856900	0.319	0.912	0.448	0.533
Tan	14	36141301	36141600	0.237	0.605	0.160	0.309
Tan	14	36328501	36328800	1.432	2.173	1.650	1.343
Tan	14	36359401	36359700	0.344	0.933	0.564	0.533
Tan	14	36938101	36938400	0.398	0.818	0.453	0.416
Tan	14	37537201	37537500	2.926	2.549	1.897	1.744
Tan	14	37590301	37590600	0.429	0.798	0.428	0.223
Tan	14	37594801	37595100	3.246	2.542	3.752	2.992
Tan	14	38341501	38341800	0.558	1.011	0.448	0.436
Tan	14	38437801	38438100	1.620	2.319	1.888	1.435
Tan	14	38452501	38452800	0.398	0.584	0.239	0.304
Tan	14	38580601	38580900	0.105	0.491	0.127	0.137
Tan	14	39177601	39177900	0.426	0.843	0.506	0.507
Tan	14	39661501	39661800	1.783	0.863	1.770	1.465
Tan	14	39711001	39711300	0.105	0.448	0.082	0.162
Tan	14	39946201	39946500	0.560	0.163	0.760	0.776
Tan	14	39981001	39981300	1.511	2.752	1.950	1.618
Tan	14	39981301	39981600	1.441	2.495	1.699	1.359
Tan	14	40071301	40071600	0.269	0.818	0.189	0.411
Tan	14	40114201	40114500	0.132	0.515	0.194	0.223
Tan	14	40222801	40223100	0.823	1.289	0.736	0.846
Tan	14	40292101	40292400	0.163	0.607	0.210	0.254
Tan	14	40396801	40397100	0.055	0.605	0.147	0.137
Tan	14	40432801	40433100	0.931	1.729	1.061	0.720
Tan	14	40522201	40522500	0.534	0.987	0.588	0.527
Tan	14	40956301	40956600	0.639	0.845	0.321	0.223
Tan	14	41153101	41153400	0.215	0.517	0.160	0.086
Tan	14	41234401	41234700	0.481	1.214	0.230	0.664
Tan	14	41387101	41387400	0.797	0.303	0.695	0.533
Tan	14	41586001	41586300	0.213	0.470	0.132	0.193
Tan	14	41761501	41761800	2.252	1.360	2.747	3.113
Tan	14	41993101	41993400	1.413	0.676	1.712	1.602
Tan	14	42001501	42001800	0.373	0.210	0.395	0.522
Tan	14	42073501	42073800	1.089	1.569	0.700	0.385
Tan	14	42164401	42164700	0.345	0.800	0.243	0.329
Tan	14	42166801	42167100	1.750	2.242	1.099	1.105
Tan	14	42181801	42182100	0.800	1.618	0.682	0.664
Tan	14	42182101	42182400	0.749	1.566	0.740	0.710
Tan	14	42253201	42253500	0.424	1.008	0.575	0.446
Tan	14	42371401	42371700	0.160	0.423	0.107	0.193
Tan	14	42554401	42554700	1.170	2.315	1.394	2.774
Tan	14	42607501	42607800	0.477	0.725	0.535	0.360
Tan	14	42609601	42609900	0.577	2.154	0.662	0.695
Tan	14	42625201	42625500	0.345	0.963	0.296	0.324
Tan	14	42626101	42626400	0.321	0.725	0.321	0.882
Tan	14	42701101	42701400	1.595	1.172	2.015	1.719
Tan	14	42714301	42714600	0.665	1.234	0.419	0.700
Tan	14	42714601	42714900	0.294	0.629	0.234	0.360
Tan	14	42811801	42812100	0.079	0.564	0.156	0.360
Tan	14	42837601	42837900	0.426	0.723	0.366	0.132
Tan	14	42893101	42893400	0.347	0.491	0.321	0.274
Tan	14	42926101	42926400	0.769	0.493	0.691	0.690
Tan	14	42957601	42957900	1.044	1.802	0.831	0.760
Tan	14	43098601	43098900	0.266	0.564	0.234	0.117
Tan	14	43273801	43274100	0.134	0.418	0.156	0.167
Tan	14	43276201	43276500	0.186	0.766	0.230	0.213
Tan	14	43276501	43276800	0.819	2.268	0.617	0.883
Tan	14	43458001	43458300	1.837	2.124	1.431	1.739
Tan	14	43559701	43560000	0.610	1.405	0.267	0.710
Tan	14	43847101	43847400	0.161	0.470	0.136	0.167
Tan	14	44056501	44056800	0.398	0.843	0.185	0.472
Tan	14	44084701	44085000	0.106	1.154	0.078	0.279
Tan	14	44149501	44149800	0.582	0.935	0.247	0.243
Tan	14	44427301	44427600	1.621	0.536	1.966	1.906
Tan	14	44525701	44526000	0.403	1.049	0.332	0.324
Tan	14	44719501	44719800	0.850	0.676	0.424	0.335

Tan	14	44870401	44870700	0.108	0.470	0.181	0.056
Tan	14	45269401	45269700	2.416	2.783	1.736	2.216
Tan	14	45335401	45335700	0.479	0.865	0.350	0.659
Tan	14	45339001	45339300	0.237	0.493	0.243	0.248
Tan	14	45421501	45421800	0.479	0.159	0.502	0.608
Tan	14	45502501	45502800	0.450	0.931	0.152	0.274
Tan	14	45561301	45561600	4.814	3.057	4.242	5.735
Tan	14	45570901	45571200	0.982	1.429	0.905	0.983
Tan	14	45639001	45639300	0.637	0.418	0.160	0.081
Tan	14	45816601	45816900	2.710	3.928	2.207	2.363
Tan	14	45868201	45868500	0.452	0.258	0.029	0.436
Tan	14	46151401	46151700	0.079	0.541	0.127	0.157
Tan	14	46280101	46280400	0.160	0.468	0.263	0.106
Tan	14	46306501	46306800	0.400	0.657	0.107	0.162
Tan	14	46475101	46475400	0.344	0.678	0.205	0.243
Tan	14	46610701	46611000	1.702	1.129	1.674	1.714
Tan	14	46672501	46672800	0.400	0.794	0.239	0.243
Tan	14	46847401	46847700	0.268	0.980	0.617	0.436
Tan	14	46941901	46942200	0.532	0.281	0.374	0.664
Tan	14	47099101	47099400	0.266	0.609	0.152	0.117
Tan	14	47145001	47145300	0.615	1.354	0.390	0.567
Tan	14	47145301	47145600	0.957	1.871	0.558	0.537
Tan	14	47280901	47281200	1.623	2.180	0.959	1.668
Tan	14	47309101	47309400	0.505	0.326	0.185	0.527
Tan	14	47416501	47416800	0.318	0.513	0.267	0.335
Tan	14	47451301	47451600	3.965	2.877	4.702	4.593
Tan	14	47655001	47655300	0.613	0.253	0.633	0.725
Tan	14	47795701	47796000	0.453	0.328	0.638	0.218
Tan	14	48172201	48172500	0.184	0.661	0.189	0.086
Tan	14	48288001	48288300	0.452	0.845	0.325	0.340
Tan	14	48388501	48388800	0.266	0.558	0.107	0.268
Tan	14	48511201	48511500	1.046	2.832	1.507	1.546
Tan	14	48541801	48542100	0.318	0.609	0.288	0.324
Tan	14	48697501	48697800	0.318	0.187	0.477	0.360
Tan	14	49072201	49072500	2.025	0.461	2.318	2.491
Tan	14	49099501	49099800	0.453	1.169	0.700	0.538
Tan	14	49128301	49128600	1.589	0.506	1.382	1.678
Tan	14	49143301	49143600	0.423	0.865	0.526	0.254
Tan	14	49336501	49336800	1.702	2.036	1.563	1.430
Tan	14	49551901	49552200	3.009	1.922	2.802	2.545
Tan	14	49597201	49597500	1.403	1.892	0.872	1.389
Tan	14	49916701	49917000	0.269	1.008	0.359	0.254
Tan	14	50109901	50110200	0.799	0.609	0.341	0.411
Tan	14	50795701	50796000	0.579	1.496	0.584	0.933
Tan	14	50824201	50824500	0.287	0.863	0.103	0.031
Tan	14	50854801	50855100	1.142	0.680	0.390	0.365
Tan	14	50943001	50943300	0.426	1.008	0.428	0.619
Tan	14	50972401	50972700	0.292	0.538	0.317	0.360
Tan	14	51014401	51014700	4.577	5.186	4.109	4.680
Tan	14	51052501	51052800	0.771	0.393	1.148	1.070
Tan	14	51057001	51057300	0.268	0.489	0.239	0.051
Tan	14	51473401	51473700	0.821	1.736	1.052	0.852
Tan	14	51606001	51606300	0.319	0.558	0.321	0.218
Tan	14	51725401	51725700	0.850	0.605	1.197	0.882
Tan	14	51803401	51803700	1.752	1.987	1.514	1.496
Tan	14	51814201	51814500	1.387	0.586	1.032	1.080
Tan	14	51917101	51917400	0.955	2.393	1.028	1.003
Tan	14	51932401	51932700	0.186	0.773	0.230	0.309
Tan	14	51940201	51940500	2.657	3.694	1.857	2.524
Tan	14	52007101	52007400	5.231	3.813	4.762	4.786
Tan	14	52122601	52122900	0.292	0.444	0.321	0.329
Tan	14	52229701	52230000	1.063	2.444	0.905	1.359
Tan	14	52230001	52230300	1.061	1.899	0.667	1.084
Tan	14	52290001	52290300	0.637	1.096	0.506	1.415
Tan	14	52319401	52319700	0.292	0.470	0.239	0.745
Tan	14	52584901	52585200	0.398	0.749	0.296	0.578
Tan	14	52591501	52591800	0.616	1.433	0.461	0.522

Tan	14	52591801	52592100	0.106	1.008	0.058	0.112
Tan	14	52592101	52592400	0.937	2.199	0.577	0.344
Tan	14	52632601	52632900	2.370	2.809	1.893	2.205
Tan	14	52730101	52730400	0.161	0.680	0.239	0.380
Tan	14	52918801	52919100	0.903	0.204	0.724	0.882
Tan	14	53102401	53102700	0.400	0.749	0.189	0.112
Tan	14	53142001	53142300	0.345	0.839	0.609	0.547
Tan	14	53151901	53152200	2.229	1.916	2.497	2.844
Tan	14	53177401	53177700	0.211	0.682	0.165	0.279
Tan	14	53198101	53198400	0.373	0.773	0.506	0.411
Tan	14	53241601	53241900	0.423	1.032	0.555	0.669
Tan	14	53269501	53269800	0.424	0.749	0.210	0.360
Tan	14	53278201	53278500	0.929	1.287	0.909	1.050
Tan	14	53325001	53325300	0.187	0.652	0.292	0.142
Tan	14	53345101	53345400	0.393	1.313	0.374	0.563
Tan	14	53353201	53353500	0.103	0.515	0.152	0.137
Tan	14	53460001	53460300	0.531	0.069	0.432	0.391
Tan	14	53539501	53539800	1.733	3.180	1.911	1.774
Tan	14	53716801	53717100	0.295	0.682	0.127	0.274
Tan	14	54064501	54064800	0.958	0.607	0.317	0.315
Tan	14	54079201	54079500	0.268	0.564	0.267	0.132
Tan	14	54162001	54162300	0.321	0.846	0.374	0.416
Tan	14	54702901	54703200	3.636	2.100	2.994	3.098
Tan	14	55026001	55026300	1.195	1.659	1.188	0.806
Tan	14	55343401	55343700	5.240	4.696	5.455	6.165
Tan	14	55443001	55443300	0.479	0.867	0.424	0.436
Tan	14	55661701	55662000	0.160	0.420	0.025	0.025
Tan	14	55717201	55717500	0.637	0.371	0.979	0.852
Tan	14	55722001	55722300	0.424	0.768	0.419	0.385
Tan	14	56049001	56049300	1.144	2.038	1.509	1.227
Tan	14	56049901	56050200	4.548	3.070	4.673	4.544
Tan	14	56111401	56111700	0.211	0.446	0.160	0.198
Tan	14	56122501	56122800	0.345	0.513	0.243	0.360
Tan	14	56123101	56123400	0.587	0.912	0.448	0.527
Tan	14	56135701	56136000	0.771	0.023	0.588	0.751
Tan	14	56165101	56165400	1.011	1.656	1.077	0.907
Tan	14	56298901	56299200	0.584	0.444	0.847	0.887
Tan	14	56326201	56326500	4.980	1.961	5.940	6.062
Tan	14	56386801	56387100	0.344	0.678	0.140	0.167
Tan	14	56635501	56635800	0.558	1.103	0.646	0.608
Tan	14	56736601	56736900	0.450	0.794	0.477	0.461
Tan	14	57217501	57217800	0.290	0.873	0.156	0.329
Tan	14	57244201	57244500	0.000	0.854	0.000	0.000
Tan	14	57423001	57423300	0.344	0.676	0.263	0.248
Tan	14	57582301	57582600	0.081	0.442	0.058	0.223
Tan	14	57928801	57929100	1.250	2.060	1.230	1.171
Tan	14	57953401	57953700	0.426	0.792	0.419	0.324
Tan	14	58165201	58165500	0.552	1.431	0.604	0.370
Tan	14	58165501	58165800	0.081	0.446	0.078	0.051
Tan	14	58773601	58773900	0.665	1.259	0.745	0.771
Tan	14	58905601	58905900	0.455	0.890	0.531	0.533
Tan	14	59065501	59065800	0.186	0.444	0.584	0.304
Tan	14	59185501	59185800	0.529	1.053	0.493	0.522
Tan	14	59679901	59680200	0.345	0.607	0.481	0.329
Tan	14	60120001	60120300	0.186	0.770	0.272	0.173
Tan	14	60339901	60340200	2.612	3.886	2.606	2.637
Tan	14	60409201	60409500	0.931	1.472	0.901	0.664
Tan	14	60553501	60553800	1.333	3.062	1.407	1.648
Tan	14	60865501	60865800	1.120	0.678	1.115	1.562
Tan	14	60927901	60928200	2.287	3.368	1.886	2.054
Tan	14	60993601	60993900	0.503	1.408	0.716	0.731
Tan	14	61181101	61181400	7.818	4.627	8.336	8.116
Tan	14	61250701	61251000	4.819	3.345	5.244	4.918
Tan	14	61308601	61308900	5.136	4.064	4.521	3.681
Tan	14	61352101	61352400	0.158	0.517	0.205	0.137
Tan	14	61361701	61362000	1.887	2.336	1.522	1.521
Tan	14	61424401	61424700	0.505	1.261	0.580	0.462

Tan	14	61424701	61425000	1.590	2.701	1.514	1.323
Tan	14	61626601	61626900	0.211	0.538	0.214	0.117
Tan	14	61747201	61747500	0.294	0.987	0.254	0.350
Tan	14	62104501	62104800	0.398	0.773	0.288	0.248
Tan	14	62140201	62140500	2.476	1.892	2.200	3.159
Tan	14	62207101	62207400	0.424	1.054	0.510	0.614
Tan	14	62476801	62477100	0.531	1.032	0.350	0.751
Tan	14	62480701	62481000	1.382	0.401	0.860	1.206
Tan	14	62552401	62552700	5.185	6.769	4.893	5.501
Tan	14	62661001	62661300	0.134	0.491	0.239	0.086
Tan	14	62714401	62714700	0.347	0.096	0.239	0.527
Tan	14	62714701	62715000	2.678	0.749	2.919	3.543
Tan	14	62719201	62719500	1.481	0.674	1.398	2.256
Tan	15	80701	81000	1.008	1.951	0.901	0.766
Tan	15	150601	150900	1.307	2.229	1.387	1.552
Tan	15	488701	489000	0.507	0.678	0.346	0.472
Tan	15	837601	837900	0.215	0.612	0.082	0.243
Tan	15	1362301	1362600	0.027	0.189	0.560	0.385
Tan	15	1629901	1630200	0.110	0.538	0.214	0.142
Tan	15	2151301	2151600	0.158	0.562	0.205	0.137
Tan	15	2638801	2639100	0.294	0.702	0.054	0.309
Tan	15	3014701	3015000	0.134	0.446	0.078	0.198
Tan	15	3134701	3135000	0.292	0.656	0.263	0.396
Tan	15	3694501	3694800	0.957	1.815	1.115	0.832
Tan	15	3734101	3734400	0.400	0.938	0.127	0.167
Tan	15	3760801	3761100	0.158	0.442	0.160	0.051
Tan	15	3761101	3761400	0.450	1.627	0.321	0.324
Tan	15	4160101	4160400	1.174	0.071	1.823	1.430
Tan	15	4259401	4259700	0.215	0.891	0.354	0.218
Tan	15	4262101	4262400	0.079	0.440	0.025	0.142
Tan	15	4566601	4566900	1.923	4.302	1.957	2.348
Tan	15	4669201	4669500	0.557	1.577	0.528	0.436
Tan	15	4707901	4708200	0.292	0.631	0.288	0.092
Tan	15	4839301	4839600	0.215	0.706	0.321	0.279
Tan	15	4861501	4861800	0.242	1.126	0.145	0.076
Tan	15	5061901	5062200	1.144	0.682	1.144	1.156
Tan	15	5507401	5507700	0.132	0.420	0.321	0.248
Tan	15	5786101	5786400	0.213	0.607	0.288	0.446
Tan	15	6083701	6084000	0.105	0.446	0.078	0.112
Tan	15	7054801	7055100	1.013	1.972	1.041	1.222
Tan	15	7323901	7324200	0.026	0.538	0.000	0.061
Tan	15	7566001	7566300	0.079	0.779	0.263	0.051
Tan	15	7624201	7624500	0.692	1.120	0.765	0.299
Tan	15	7822501	7822800	0.026	0.515	0.000	0.173
Tan	15	8054101	8054400	0.053	0.919	0.239	0.193
Tan	15	8095201	8095500	0.850	0.189	0.510	0.588
Tan	15	8096101	8096400	0.106	0.706	0.107	0.132
Tan	15	8110801	8111100	0.347	1.025	0.279	0.304
Tan	15	8562901	8563200	0.237	1.358	0.831	0.664
Tan	15	8981101	8981400	0.292	0.373	0.477	0.664
Tan	15	9189001	9189300	0.187	0.564	0.317	0.329
Tan	15	10768501	10768800	0.982	0.255	0.852	1.050
Tan	15	10833901	10834200	0.053	0.560	0.140	0.162
Tan	15	11095801	11096100	0.426	0.676	0.000	0.086
Tan	15	11096101	11096400	0.108	0.463	0.049	0.056
Tan	15	12167401	12167700	0.079	0.588	0.185	0.248
Tan	15	12647401	12647700	0.027	0.446	0.078	0.167
Tan	15	13926001	13926300	0.984	1.545	0.847	1.120
Tan	15	13963801	13964100	0.290	0.753	0.312	0.243
Tan	15	14019301	14019600	0.213	0.588	0.292	0.167
Tan	15	14455801	14456100	0.905	1.944	0.999	1.283
Tan	15	14516701	14517000	0.347	0.676	0.453	0.527
Tan	15	14602801	14603100	1.065	1.875	1.304	1.404
Tan	15	14761501	14761800	0.132	0.493	0.160	0.279
Tan	15	14808901	14809200	0.778	1.620	0.729	0.416
Tan	15	15274801	15275100	0.026	0.607	0.098	0.137
Tan	15	15285301	15285600	4.147	1.127	5.566	5.195

Tan	15	15384601	15384900	0.132	0.465	0.132	0.061
Tan	15	15523501	15523800	0.526	1.073	0.312	0.497
Tan	15	15704701	15705000	0.187	0.519	0.107	0.162
Tan	15	16360801	16361100	0.290	0.562	0.292	0.329
Tan	15	16497301	16497600	0.160	0.611	0.111	0.117
Tan	15	16718701	16719000	0.398	0.586	0.189	0.329
Tan	15	17145301	17145600	1.963	2.613	0.682	1.734
Tan	15	17496601	17496900	0.136	0.583	0.214	0.213
Tan	15	17685001	17685300	0.265	0.511	0.802	0.218
Tan	15	17865301	17865600	0.265	0.656	0.276	0.218
Tan	15	18066601	18066900	0.132	0.513	0.259	0.056
Tan	15	18289201	18289500	0.371	0.772	0.399	0.396
Tan	15	18328201	18328500	0.265	0.609	0.399	0.167
Tan	15	18328501	18328800	0.266	0.442	0.292	0.304
Tan	15	19143601	19143900	0.079	0.470	0.239	0.198
Tan	15	19329001	19329300	0.106	0.187	0.263	0.497
Tan	15	19754401	19754700	0.371	0.701	0.185	0.385
Tan	15	19815601	19815900	0.187	0.659	0.165	0.218
Tan	15	20176801	20177100	1.089	1.403	0.983	0.695
Tan	15	20232301	20232600	0.213	0.466	0.218	0.248
Tan	15	20466901	20467200	0.265	0.702	0.370	0.335
Tan	15	20589301	20589600	0.423	0.963	0.428	0.553
Tan	15	20676001	20676300	0.879	1.425	0.930	1.080
Tan	15	20739001	20739300	0.586	0.817	0.428	0.391
Tan	15	21228901	21229200	0.184	0.676	0.370	0.173
Tan	15	21237301	21237600	0.160	0.468	0.078	0.299
Tan	15	21433501	21433800	5.582	4.860	5.495	6.257
Tan	15	21471001	21471300	1.381	2.012	1.226	1.627
Tan	15	21554701	21555000	0.531	0.964	0.457	0.522
Tan	15	21863401	21863700	0.423	0.773	0.292	0.360
Tan	15	22433701	22434000	0.639	0.114	0.350	0.466
Tan	15	22616701	22617000	0.797	0.560	0.740	0.913
Tan	15	22631401	22631700	1.244	0.678	1.246	1.262
Tan	15	22819201	22819500	0.266	0.420	0.214	0.218
Tan	15	23036401	23036700	0.903	1.328	0.959	0.882
Tan	15	23113801	23114100	0.266	0.444	0.234	0.051
Tan	15	23226001	23226300	0.319	0.772	0.234	0.472
Tan	15	23264701	23265000	1.065	1.450	1.567	1.247
Tan	15	23293801	23294100	1.625	0.963	1.378	1.501
Tan	15	23304001	23304300	0.690	0.348	0.671	0.994
Tan	15	23317801	23318100	0.187	0.538	0.346	0.248
Tan	15	23333701	23334000	0.268	0.820	0.390	0.112
Tan	15	23668801	23669100	0.371	0.682	0.346	0.309
Tan	15	23683501	23683800	1.015	2.251	1.378	1.420
Tan	15	23777101	23777400	0.184	0.657	0.210	0.279
Tan	15	23835601	23835900	0.479	0.562	0.107	0.248
Tan	15	24029701	24030000	0.160	1.450	0.473	0.405
Tan	15	24056401	24056700	1.442	2.435	1.287	1.307
Tan	15	24138301	24138600	0.534	1.455	0.802	0.812
Tan	15	24322801	24323100	0.000	0.541	0.078	0.056
Tan	15	24328201	24328500	2.308	4.995	3.070	2.018
Tan	15	24465301	24465600	0.345	0.657	0.374	0.228
Tan	15	24904801	24905100	0.316	0.886	0.379	0.157
Tan	15	25085101	25085400	0.239	0.560	0.185	0.132
Tan	15	25200901	25201200	0.189	0.656	0.247	0.274
Tan	15	25245901	25246200	2.950	3.396	2.575	2.870
Tan	15	25336501	25336800	2.260	1.599	2.336	2.495
Tan	15	25666501	25666800	0.265	0.629	0.259	0.218
Tan	15	25779601	25779900	0.345	0.867	0.272	0.299
Tan	15	25854001	25854300	1.991	2.892	1.743	1.784
Tan	15	25914001	25914300	0.187	0.611	0.025	0.061
Tan	15	26042401	26042700	0.213	0.446	0.111	0.198
Tan	15	26223301	26223600	0.558	0.421	0.132	0.193
Tan	15	26229301	26229600	0.163	0.513	0.136	0.081
Tan	15	26302501	26302800	0.079	0.395	0.054	0.106
Tan	15	26471701	26472000	0.905	1.148	0.740	0.106
Tan	15	26788201	26788500	0.905	0.440	0.843	0.918

Tan	15	26792101	26792400	0.292	0.163	0.263	0.497
Tan	15	26997301	26997600	3.433	2.615	3.447	3.705
Tan	15	26997601	26997900	3.370	2.351	3.480	3.635
Tan	15	27046201	27046500	0.160	0.517	0.317	0.274
Tan	15	27047401	27047700	0.265	0.444	0.136	0.279
Tan	15	27163201	27163500	0.294	0.468	0.243	0.248
Tan	15	27367201	27367500	1.095	1.732	1.106	1.029
Tan	15	27383101	27383400	0.160	0.448	0.132	0.056
Tan	15	27515101	27515400	0.561	1.122	0.424	0.446
Tan	15	27616501	27616800	0.131	0.818	0.292	0.187
Tan	15	27641401	27641700	1.120	2.723	3.603	1.334
Tan	15	27709801	27710100	3.731	1.349	2.693	2.840
Tan	15	27805201	27805500	0.213	0.560	0.136	0.132
Tan	15	27832501	27832800	0.371	0.822	0.325	0.329
Tan	15	27837301	27837600	3.643	1.891	3.328	3.808
Tan	15	27904201	27904500	1.005	2.349	1.295	1.354
Tan	15	28079101	28079400	0.187	0.468	0.165	0.137
Tan	15	28089301	28089600	2.602	4.448	2.949	3.190
Tan	15	28223401	28223700	0.505	0.094	0.234	0.527
Tan	15	28501201	28501500	0.161	0.444	0.078	0.137
Tan	15	28569901	28570200	0.318	0.024	0.580	0.304
Tan	15	28706401	28706700	0.477	1.122	0.597	0.446
Tan	15	28766401	28766700	0.213	0.753	0.288	0.304
Tan	15	28789801	28790100	2.344	3.234	2.218	2.302
Tan	15	28792801	28793100	0.531	0.980	0.337	0.486
Tan	15	29137801	29138100	0.052	0.395	0.000	0.056
Tan	15	29344801	29345100	0.347	0.676	0.366	0.167
Tan	15	29354101	29354400	0.239	0.420	0.263	0.137
Tan	15	29468101	29468400	0.052	0.425	0.025	0.112
Tan	15	29626501	29626800	0.239	0.605	0.292	0.173
Tan	15	29661901	29662200	0.240	0.588	0.292	0.198
Tan	15	29944201	29944500	0.692	0.281	0.613	0.654
Tan	15	29945401	29945700	0.160	0.517	0.189	0.173
Tan	15	29947501	29947800	0.079	0.442	0.107	0.162
Tan	15	30031201	30031500	0.292	0.583	0.827	0.416
Tan	15	30266401	30266700	1.676	0.948	1.815	1.876
Tan	15	30445201	30445500	0.027	0.442	0.185	0.112
Tan	15	30744601	30744900	0.187	0.444	0.214	0.223
Tan	15	30778801	30779100	0.237	0.584	0.185	0.193
Tan	15	30794401	30794700	0.265	0.584	0.312	0.142
Tan	15	31085401	31085700	0.797	1.335	0.914	0.751
Tan	15	31232101	31232400	0.240	0.491	0.111	0.304
Tan	15	31300801	31301100	0.374	0.987	0.461	0.452
Tan	15	31500601	31500900	0.403	1.098	0.424	0.355
Tan	15	31851601	31851900	3.596	2.341	3.876	4.032
Tan	15	32128801	32129100	0.878	1.171	0.827	0.684
Tan	15	32187601	32187900	0.108	0.562	0.136	0.056
Tan	15	32232901	32233200	0.632	1.261	0.346	0.527
Tan	15	32409601	32409900	1.007	1.738	1.008	1.039
Tan	15	32494501	32494800	0.106	0.470	0.214	0.279
Tan	15	32742301	32742600	0.215	0.942	0.140	0.167
Tan	15	32742601	32742900	0.773	2.909	0.684	1.157
Tan	15	32754001	32754300	0.106	0.536	0.132	0.086
Tan	15	32811601	32811900	0.079	0.588	0.107	0.162
Tan	15	32829601	32829900	0.213	1.240	0.317	0.254
Tan	15	32830201	32830500	0.500	1.382	0.517	0.324
Tan	15	32830501	32830800	0.557	1.266	0.473	0.355
Tan	15	33162301	33162600	0.558	0.350	0.580	0.639
Tan	15	33596701	33597000	0.720	1.127	0.502	0.502
Tan	15	33967801	33968100	0.265	0.584	0.189	0.335
Tan	15	34262701	34263000	1.036	1.757	1.275	0.461
Tan	15	34300501	34300800	0.529	1.082	0.481	0.522
Tan	15	34300801	34301100	0.106	0.612	0.243	0.187
Tan	15	34416901	34417200	0.161	0.562	0.156	0.187
Tan	15	34420201	34420500	0.503	0.890	0.370	0.218
Tan	15	34562101	34562400	0.158	0.493	0.103	0.187
Tan	15	34863001	34863300	3.622	4.471	2.998	3.747

Tan	15	35024101	35024400	0.369	0.749	0.312	0.254
Tan	15	35138701	35139000	0.265	0.045	0.403	0.608
Tan	15	35187601	35187900	0.824	0.393	0.078	0.167
Tan	15	35238601	35238900	0.266	0.491	0.111	0.274
Tan	15	35851201	35851500	0.266	0.539	0.267	0.254
Tan	15	35877601	35877900	0.161	0.607	0.247	0.243
Tan	15	35957401	35957700	0.634	1.216	0.424	0.756
Tan	15	36342001	36342300	0.265	0.773	0.317	0.527
Tan	15	36823501	36823800	0.134	0.676	0.390	0.081
Tan	15	36887701	36888000	0.424	0.187	0.588	0.329
Tan	15	36959101	36959400	1.091	2.360	1.509	1.810
Tan	15	37035601	37035900	0.292	0.629	0.283	0.299
Tan	15	37333801	37334100	0.505	0.257	0.403	0.056
Tan	15	37403401	37403700	0.850	0.959	0.745	1.131
Tan	15	37482901	37483200	0.347	0.914	0.399	0.608
Tan	15	38069701	38070000	0.344	0.910	0.259	0.497
Tan	15	38070001	38070300	0.423	0.747	0.189	0.304
Tan	15	38102101	38102400	0.532	0.702	0.481	0.553
Tan	15	38116201	38116500	0.239	0.772	0.185	0.466
Tan	15	38339401	38339700	0.905	1.425	1.090	1.039
Tan	15	38415901	38416200	0.694	0.515	0.346	0.776
Tan	15	38547901	38548200	0.268	0.657	0.185	0.360
Tan	15	38726101	38726400	0.452	0.749	0.312	0.553
Tan	15	38916901	38917200	0.371	0.678	0.288	0.335
Tan	15	39156901	39157200	0.321	1.015	0.181	0.223
Tan	15	39367801	39368100	0.532	1.056	0.593	0.502
Tan	15	39610501	39610800	0.345	0.680	0.366	0.254
Tan	15	39977701	39978000	0.108	0.588	0.127	0.076
Tan	15	40516501	40516800	0.292	0.517	0.214	0.248
Tan	15	40628401	40628700	0.106	0.448	0.160	0.132
Tan	15	40638301	40638600	0.186	0.517	0.292	0.142
Tan	15	40810201	40810500	0.773	1.334	0.731	0.436
Tan	15	40902001	40902300	2.184	1.757	1.275	1.105
Tan	15	41274001	41274300	0.455	1.551	0.564	0.268
Tan	15	41351101	41351400	0.240	0.837	0.457	0.380
Tan	15	41849701	41850000	0.131	0.586	0.288	0.254
Tan	15	41853301	41853600	2.531	0.676	1.793	1.374
Tan	15	42475501	42475800	0.982	0.491	0.749	0.725
Tan	15	42616501	42616800	0.160	0.446	0.769	0.137
Tan	15	42633001	42633300	0.292	0.800	0.395	0.162
Tan	15	42730801	42731100	0.132	0.558	0.111	0.142
Tan	15	42954001	42954300	2.213	1.521	2.218	2.292
Tan	15	43449901	43450200	0.158	0.614	0.160	0.106
Tan	15	43489501	43489800	0.187	0.350	0.502	0.142
Tan	15	43783501	43783800	0.081	0.493	0.263	0.193
Tan	15	43998901	43999200	1.247	2.015	1.402	0.370
Tan	15	44022301	44022600	0.186	0.491	0.140	0.106
Tan	15	44141701	44142000	0.374	0.609	0.292	0.243
Tan	15	44189701	44190000	0.186	0.560	0.107	0.086
Tan	15	44247901	44248200	0.237	0.678	0.296	0.315
Tan	15	44555701	44556000	0.479	1.004	0.691	0.315
Tan	15	44784901	44785200	0.161	0.729	0.341	0.309
Tan	15	44975101	44975400	0.134	0.562	0.169	0.198
Tan	15	45447301	45447600	0.294	0.534	0.025	0.279
Tan	15	45823201	45823500	2.610	1.150	2.675	2.596
Tan	15	45823501	45823800	8.069	3.292	9.533	8.584
Tan	15	45823801	45824100	2.641	1.452	3.134	2.535
Tan	15	45870301	45870600	1.008	1.714	0.794	1.192
Tan	15	46141201	46141500	0.292	0.564	0.317	0.329
Tan	15	46156801	46157100	0.160	0.584	0.218	0.198
Tan	15	46757101	46757400	0.718	0.397	0.555	0.745
Tan	15	46931701	46932000	0.213	0.753	0.132	0.329
Tan	15	48637501	48637800	0.132	0.541	0.263	0.167
Tan	15	48842401	48842700	1.436	2.648	0.873	1.247
Tan	15	49057201	49057500	0.479	0.841	0.457	0.472
Tan	15	49236901	49237200	0.132	0.375	0.954	0.106
Tan	15	49718701	49719000	1.118	0.536	1.110	1.096

Tan	15	49768201	49768500	1.781	2.961	1.654	1.668
Tan	15	50214001	50214300	0.932	1.427	0.662	0.725
Tan	15	50469001	50469300	0.345	0.721	0.205	0.360
Tan	15	50470801	50471100	2.186	3.319	1.745	2.156
Tan	15	50625901	50626200	0.240	0.420	0.160	0.198
Tan	15	50777101	50777400	1.573	2.594	1.592	1.861
Tan	15	50777401	50777700	1.812	2.549	1.625	1.820
Tan	15	50939101	50939400	3.689	1.358	3.348	2.845
Tan	15	50974801	50975100	0.345	0.466	0.214	0.142
Tan	15	50999101	50999400	1.436	0.721	1.570	2.641
Tan	15	51232501	51232800	0.184	0.678	0.165	0.132
Tan	15	51232801	51233100	0.290	0.628	0.214	0.056
Tan	15	51301801	51302100	0.189	0.789	0.116	0.193
Tan	15	51489901	51490200	0.426	0.747	0.243	0.304
Tan	15	51517501	51517800	0.215	0.468	0.234	0.167
Tan	15	51629701	51630000	0.158	0.541	0.029	0.056
Tan	15	52038301	52038600	0.053	0.472	0.087	0.112
Tan	15	52131301	52131600	0.663	1.313	0.745	0.852
Tan	15	52200301	52200600	0.426	0.727	0.312	0.299
Tan	15	52869301	52869600	0.799	1.195	0.473	0.781
Tan	15	52927501	52927800	1.142	1.519	0.564	0.821
Tan	15	52986901	52987200	2.368	1.849	1.358	2.226
Tan	15	53076901	53077200	0.397	0.607	0.243	0.137
Tan	15	53252701	53253000	0.106	0.468	0.140	0.167
Tan	15	53345401	53345700	2.152	3.092	2.185	2.135
Tan	15	53425201	53425500	1.808	2.903	1.501	1.926
Tan	15	53427001	53427300	0.134	0.470	0.165	0.086
Tan	15	53451601	53451900	0.132	0.538	0.272	0.142
Tan	15	53534101	53534400	1.013	0.347	0.914	1.287
Tan	15	53776801	53777100	2.892	1.515	2.225	2.252
Tan	15	54043801	54044100	0.181	0.978	0.218	0.233
Tan	15	54230101	54230400	0.345	0.163	0.477	0.274
Tan	15	54287101	54287400	0.160	0.444	0.156	0.223
Tan	15	55681201	55681500	0.666	0.215	1.308	1.050
Tan	15	56075101	56075400	0.400	0.725	0.481	0.553
Tan	15	56108401	56108700	0.319	0.659	0.263	0.223
Tan	15	56202001	56202300	0.292	0.470	0.239	0.223
Tan	15	56409001	56409300	0.187	0.420	0.263	0.193
Tan	15	56893201	56893500	0.184	0.517	0.160	0.218
Tan	15	57114301	57114600	0.821	1.667	0.859	0.771
Tan	15	57265501	57265800	0.369	0.631	0.054	0.218
Tan	15	57359401	57359700	0.158	0.468	0.136	0.061
Tan	15	57400501	57400800	0.531	0.328	0.481	0.472
Tan	15	57651901	57652200	0.186	0.517	0.321	0.304
Tan	15	58323601	58323900	0.344	0.631	0.205	0.329
Tan	15	58979701	58980000	0.213	0.468	0.317	0.248
Tan	15	59453401	59453700	0.079	0.493	0.185	0.213
Tan	15	59600101	59600400	0.448	0.890	0.379	0.396
Tan	15	60428101	60428400	1.623	2.528	1.732	1.805
Tan	15	60537301	60537600	0.134	0.745	0.218	0.142
Tan	15	60639301	60639600	0.242	0.773	0.111	0.152
Tan	15	60708301	60708600	0.026	0.371	0.078	0.056
Tan	15	61237501	61237800	2.291	1.658	2.296	2.252
Tan	15	61242901	61243200	0.079	0.770	0.103	0.152
Tan	15	61544401	61544700	0.266	0.729	0.341	0.329
Tan	15	61898401	61898700	1.729	2.386	1.427	1.009
Tan	15	62151901	62152200	0.160	1.283	0.156	0.526
Tan	15	62329801	62330100	1.386	2.607	1.297	1.176
Tan	15	62366101	62366400	0.294	0.678	0.395	0.309
Tan	15	62538001	62538300	0.132	0.539	0.296	0.193
Tan	15	62739301	62739600	0.893	4.502	2.491	2.946
Tan	15	62769001	62769300	0.134	0.395	0.160	0.198
Tan	15	62782801	62783100	0.158	0.446	0.189	0.248
Tan	15	63136201	63136500	0.053	0.395	0.185	0.223
Tan	15	63196801	63197100	0.316	0.657	0.210	0.274
Tan	15	63649201	63649500	0.833	2.285	0.678	0.700
Tan	15	63649501	63649800	0.621	2.008	0.337	0.426

Tan	15	63817801	63818100	0.323	1.386	0.296	0.486
Tan	15	64128901	64129200	0.507	0.865	0.510	0.477
Tan	15	64327501	64327800	0.373	0.753	0.346	0.268
Tan	15	64399801	64400100	1.518	0.513	1.895	1.852
Tan	15	64641301	64641600	0.716	1.167	0.604	0.380
Tan	15	64674901	64675200	0.400	0.680	0.399	0.472
Tan	15	65072701	65073000	0.903	1.075	0.798	0.472
Tan	15	65148001	65148300	0.268	0.629	0.325	0.218
Tan	15	65238601	65238900	0.294	0.562	0.267	0.284
Tan	15	65349901	65350200	0.845	1.334	0.794	0.771
Tan	15	65371201	65371500	0.134	0.420	0.127	0.132
Tan	15	65520601	65520900	0.718	0.888	0.403	0.416
Tan	15	65619301	65619600	0.347	0.656	0.214	0.284
Tan	15	65865301	65865600	0.344	0.893	0.403	0.294
Tan	15	66057301	66057600	1.752	3.690	1.074	2.177
Tan	15	66060601	66060900	0.239	0.730	0.185	0.203
Tan	15	67205101	67205400	0.690	0.818	0.453	0.466
Tan	15	67298401	67298700	0.132	0.446	0.025	0.061
Tan	15	67412101	67412400	0.292	0.444	0.103	0.193
Tan	15	68554501	68554800	0.268	0.493	0.078	0.162
Tan	15	69875101	69875400	2.057	3.662	2.170	1.896
Tan	15	70575001	70575300	0.160	0.494	0.156	0.112
Tan	15	70731601	70731900	0.000	0.485	0.000	0.000
Tan	15	70810201	70810500	0.213	0.350	0.555	0.137
Tan	15	71011501	71011800	0.187	0.491	0.263	0.198
Tan	15	71169001	71169300	0.558	0.654	0.453	0.472
Tan	15	71300401	71300700	0.213	0.442	0.189	0.218
Tan	15	71375401	71375700	0.637	1.004	0.609	0.720
Tan	15	71575501	71575800	0.132	0.584	0.428	0.385
Tan	15	71805301	71805600	1.439	0.234	1.262	0.817
Tan	15	71808001	71808300	0.507	0.187	0.395	0.441
Tan	15	71822101	71822400	0.158	0.513	0.239	0.223
Tan	15	71978701	71979000	0.742	1.289	0.667	0.628
Tan	15	72143101	72143400	0.473	1.551	0.234	0.182
Tan	15	72209701	72210000	0.477	1.408	0.602	0.624
Tan	15	72520501	72520800	1.623	0.905	1.529	1.825
Tan	15	72549001	72549300	0.750	0.000	1.123	0.846
Tan	15	72549601	72549900	2.573	0.000	2.224	2.165
Tan	15	72632401	72632700	0.160	0.446	0.103	0.248
Tan	15	72662701	72663000	0.531	1.335	0.531	0.497
Tan	15	72663001	72663300	0.536	1.026	0.448	0.583
Tan	15	72855901	72856200	0.339	1.448	0.749	0.710
Tan	15	72870901	72871200	1.142	3.072	1.288	1.917
Tan	15	72906601	72906900	0.110	0.487	0.107	0.086
Tan	15	72923401	72923700	0.507	0.350	0.239	0.776
Tan	15	73027501	73027800	0.106	0.678	0.243	0.329
Tan	15	73205701	73206000	0.240	0.491	0.243	0.690
Tan	15	73216801	73217100	0.239	0.560	0.321	0.187
Tan	15	73227901	73228200	1.011	1.453	1.090	0.948
Tan	15	73309501	73309800	0.716	0.983	0.531	0.669
Tan	15	73437001	73437300	3.735	6.817	3.387	4.817
Tan	15	73506001	73506300	5.454	3.152	4.781	6.294
Tan	15	73631701	73632000	0.690	1.261	0.707	0.806
Tan	15	73697101	73697400	1.752	2.629	1.592	1.739
Tan	15	74481001	74481300	0.186	0.515	0.111	0.081
Tan	15	74532901	74533200	0.611	0.843	0.633	0.502
Tan	15	74546101	74546400	0.269	0.609	0.132	0.218
Tan	15	74695801	74696100	0.213	0.446	0.111	0.198
Tan	15	74751601	74751900	0.347	0.775	0.283	0.167
Tan	15	74867701	74868000	0.187	0.538	0.156	0.187
Tan	15	75077401	75077700	0.081	0.448	0.025	0.056
Tan	15	75242101	75242400	1.113	1.498	0.794	0.771
Tan	15	75439501	75439800	0.216	0.796	0.267	0.268
Tan	15	75489001	75489300	1.625	1.985	1.099	1.125
Tan	15	75523801	75524100	0.771	1.459	0.609	0.644
Tan	15	75815101	75815400	0.850	1.377	0.827	0.654
Tan	15	75914101	75914400	0.184	1.028	0.468	0.304

Tan	15	75952501	75952800	0.108	0.448	0.185	0.137
Tan	15	75952801	75953100	0.828	1.337	0.700	0.771
Tan	15	75953401	75953700	0.503	0.069	0.535	0.472
Tan	15	76072501	76072800	0.342	0.820	0.234	0.365
Tan	15	76072801	76073100	0.640	1.126	0.243	0.431
Tan	15	76337101	76337400	0.268	0.513	0.218	0.137
Tan	15	76635001	76635300	0.242	0.676	0.087	0.117
Tan	15	77330701	77331000	1.087	1.571	1.021	2.606
Tan	15	77371201	77371500	1.649	0.517	0.774	1.125
Tan	15	77433601	77433900	0.213	0.444	0.210	0.218
Tan	15	77451301	77451600	0.292	0.513	0.288	0.193
Tan	15	77500201	77500500	0.106	0.446	0.140	0.081
Tan	15	77635801	77636100	2.578	3.268	1.723	2.581
Tan	15	77637901	77638200	0.663	1.337	0.740	0.812
Tan	15	77656201	77656500	0.505	0.656	0.428	0.527
Tan	15	77667301	77667600	0.079	0.517	0.189	0.198
Tan	15	77706901	77707200	0.853	0.538	0.905	1.075
Tan	15	77713201	77713500	1.013	1.877	1.052	0.730
Tan	15	77874301	77874600	0.507	0.891	0.535	0.533
Tan	15	77886001	77886300	3.426	4.641	3.491	3.584
Tan	15	78073501	78073800	1.705	2.105	1.222	1.627
Tan	15	78120301	78120600	0.584	1.377	0.617	0.963
Tan	15	78162001	78162300	0.505	0.678	0.506	0.578
Tan	15	78394201	78394500	6.566	1.553	4.589	4.500
Tan	15	78411301	78411600	2.100	1.455	2.377	2.616
Tan	15	78414901	78415200	1.515	1.826	1.275	1.607
Tan	15	78473401	78473700	0.211	0.682	0.292	0.324
Tan	15	78490201	78490500	0.584	1.081	0.346	0.603
Tan	15	78588901	78589200	3.644	1.731	3.516	5.976
Tan	15	78687001	78687300	0.108	0.631	0.049	0.223
Tan	15	78687301	78687600	0.289	0.916	0.263	0.446
Tan	15	78779701	78780000	0.134	0.654	0.218	0.380
Tan	15	78909901	78910200	1.056	0.232	1.090	0.933
Tan	15	79094101	79094400	0.529	1.124	0.560	0.344
Tan	15	79097401	79097700	0.450	0.751	0.399	0.355
Tan	15	79111801	79112100	0.658	2.811	0.555	0.634
Tan	15	79112101	79112400	0.990	3.073	0.588	0.862
Tan	15	79132501	79132800	8.339	4.230	9.136	8.298
Tan	15	79159501	79159800	0.239	0.581	0.160	0.304
Tan	15	79162201	79162500	0.720	1.386	0.745	0.593
Tan	15	79168501	79168800	0.479	0.680	0.477	0.527
Tan	15	79845301	79845600	0.294	0.468	0.239	0.254
Tan	15	79908301	79908600	8.619	3.966	6.774	7.413
Tan	15	80101501	80101800	0.747	1.309	0.546	0.588
Tan	15	80105701	80106000	1.008	2.483	1.525	0.923
Tan	15	80247301	80247600	1.525	2.856	2.011	1.410
Tan	15	80613001	80613300	0.452	0.654	0.424	0.411
Tan	15	80741401	80741700	0.268	0.562	0.160	0.360
Tan	15	80778001	80778300	1.729	2.291	1.888	1.856
Tan	15	80812501	80812800	1.011	1.098	1.436	0.801
Tan	15	80871601	80871900	0.268	0.421	0.584	0.583
Tan	16	97801	98100	0.210	0.562	0.214	0.223
Tan	16	155401	155700	1.058	2.259	1.366	0.751
Tan	16	253201	253500	0.000	0.470	0.000	0.167
Tan	16	280801	281100	0.797	1.510	0.802	0.857
Tan	16	313501	313800	0.319	1.545	0.499	0.416
Tan	16	313801	314100	0.108	0.489	0.185	0.106
Tan	16	528001	528300	1.171	1.495	1.358	0.745
Tan	16	557101	557400	0.558	1.264	0.510	0.826
Tan	16	571201	571500	0.610	0.987	0.584	0.527
Tan	16	740401	740700	0.027	0.494	0.156	0.173
Tan	16	756301	756600	4.630	1.295	5.420	5.226
Tan	16	963301	963600	0.497	1.425	0.419	0.472
Tan	16	967201	967500	0.503	0.257	0.662	0.695
Tan	16	969901	970200	0.215	0.818	0.239	0.223
Tan	16	970201	970500	0.187	0.657	0.107	0.294
Tan	16	998401	998700	1.516	1.920	1.135	1.273

Tan	16	1002601	1002900	2.097	1.206	1.979	3.012
Tan	16	1060501	1060800	4.088	1.914	3.083	4.513
Tan	16	1080001	1080300	6.657	4.469	6.394	6.906
Tan	16	1153501	1153800	0.268	0.633	0.292	0.279
Tan	16	1479601	1479900	4.628	3.969	4.851	4.538
Tan	16	1652401	1652700	0.557	2.654	1.116	0.521
Tan	16	1695301	1695600	0.266	0.588	0.082	0.162
Tan	16	1792801	1793100	0.455	1.318	0.301	0.436
Tan	16	1845901	1846200	0.345	0.751	0.403	0.365
Tan	16	1982101	1982400	0.586	0.891	0.457	0.558
Tan	16	2043301	2043600	0.160	0.493	0.189	0.147
Tan	16	2101201	2101500	0.426	1.030	0.350	0.268
Tan	16	2145901	2146200	0.453	1.216	0.588	0.512
Tan	16	2251501	2251800	0.557	1.051	0.575	0.461
Tan	16	2265301	2265600	0.345	0.234	0.107	0.466
Tan	16	2544901	2545200	5.556	3.961	5.925	5.167
Tan	16	2551501	2551800	0.505	1.302	0.210	0.355
Tan	16	2867701	2868000	0.803	1.495	0.905	0.857
Tan	16	2910601	2910900	0.239	0.494	0.078	0.193
Tan	16	3072301	3072600	0.321	0.751	0.189	0.218
Tan	16	3216901	3217200	1.892	0.936	1.879	1.784
Tan	16	3217801	3218100	0.318	1.216	0.288	0.203
Tan	16	3324001	3324300	0.400	1.028	0.604	0.492
Tan	16	3489001	3489300	0.689	1.172	0.502	0.669
Tan	16	3492001	3492300	0.186	0.446	0.185	0.279
Tan	16	3568201	3568500	0.266	0.562	0.234	0.335
Tan	16	3615601	3615900	0.637	1.897	1.239	0.365
Tan	16	3615901	3616200	0.055	0.584	0.259	0.223
Tan	16	3639901	3640200	1.408	1.963	1.317	0.760
Tan	16	3663301	3663600	0.319	0.045	0.477	0.279
Tan	16	3785101	3785400	0.563	1.051	0.481	0.608
Tan	16	3929101	3929400	0.052	0.444	0.049	0.061
Tan	16	3943501	3943800	3.823	3.088	4.093	4.274
Tan	16	3990601	3990900	0.924	3.091	1.177	4.557
Tan	16	4167301	4167600	0.371	0.916	0.486	0.309
Tan	16	4213801	4214100	0.503	1.850	0.531	0.685
Tan	16	4362301	4362600	1.250	0.378	1.106	1.861
Tan	16	4374301	4374600	0.826	2.410	0.573	0.891
Tan	16	4377301	4377600	0.557	0.985	0.448	0.634
Tan	16	4396201	4396500	0.505	1.663	0.548	0.801
Tan	16	4396501	4396800	0.105	0.936	0.058	0.329
Tan	16	4406401	4406700	0.160	0.631	0.234	0.243
Tan	16	4605001	4605300	1.173	0.418	0.901	1.192
Tan	16	4615201	4615500	0.292	0.839	0.432	0.466
Tan	16	4626601	4626900	1.681	3.332	2.004	2.083
Tan	16	4650901	4651200	1.031	2.135	0.856	1.258
Tan	16	4781701	4782000	0.587	1.341	0.510	0.538
Tan	16	4818601	4818900	0.158	0.828	0.054	0.086
Tan	16	4850101	4850400	0.189	0.637	0.136	0.198
Tan	16	4925101	4925400	0.584	1.030	2.015	0.634
Tan	16	5357101	5357400	0.584	1.099	0.383	0.375
Tan	16	5409001	5409300	1.010	0.725	0.580	0.360
Tan	16	5429401	5429700	0.345	0.421	0.584	0.223
Tan	16	5439301	5439600	0.527	1.174	0.374	0.527
Tan	16	5445001	5445300	0.534	0.163	0.497	0.578
Tan	16	5458501	5458800	1.269	2.203	0.876	1.232
Tan	16	5469601	5469900	3.622	1.569	3.375	3.345
Tan	16	5470801	5471100	0.289	0.919	0.185	0.117
Tan	16	5476201	5476500	3.393	1.414	3.148	3.047
Tan	16	5484001	5484300	0.587	0.000	0.843	0.983
Tan	16	5502001	5502300	0.294	0.766	0.234	0.238
Tan	16	5564101	5564400	3.405	4.250	3.369	3.615
Tan	16	5814301	5814600	0.613	1.219	0.147	0.563
Tan	16	5856301	5856600	1.916	4.358	1.870	1.568
Tan	16	6087001	6087300	0.160	0.513	0.239	0.416
Tan	16	6100801	6101100	2.566	3.832	2.222	2.464
Tan	16	6225901	6226200	1.010	0.794	0.638	0.664

Tan	16	6304501	6304800	0.476	1.362	0.370	0.614
Tan	16	6304801	6305100	0.368	0.940	0.049	0.137
Tan	16	6305101	6305400	0.208	0.633	0.103	0.137
Tan	16	6315001	6315300	0.240	0.611	0.259	0.228
Tan	16	6428701	6429000	0.452	0.936	0.370	0.431
Tan	16	6736801	6737100	0.479	0.798	0.502	0.522
Tan	16	7026001	7026300	2.547	3.922	2.575	2.458
Tan	16	7028401	7028700	1.145	1.873	0.996	1.125
Tan	16	7203301	7203600	0.345	0.468	0.210	0.360
Tan	16	7269901	7270200	0.371	1.373	0.263	0.573
Tan	16	7279501	7279800	0.373	0.775	0.424	0.416
Tan	16	7380001	7380300	0.797	0.376	0.609	0.583
Tan	16	7435801	7436100	0.160	0.612	0.136	0.187
Tan	16	7917001	7917300	1.010	2.088	1.130	1.334
Tan	16	7918801	7919100	0.527	1.261	0.740	0.801
Tan	16	8093401	8093700	0.453	1.004	0.720	0.558
Tan	16	8357701	8358000	0.290	0.725	0.453	0.274
Tan	16	8398501	8398800	1.092	0.914	0.745	0.360
Tan	16	8477701	8478000	0.132	0.494	0.127	0.137
Tan	16	8654401	8654700	1.228	2.150	0.684	0.710
Tan	16	8781601	8781900	0.160	0.446	0.210	0.167
Tan	16	8876101	8876400	0.373	0.629	0.350	0.329
Tan	16	9098701	9099000	0.611	0.867	0.477	0.411
Tan	16	9128401	9128700	0.476	1.122	0.477	0.441
Tan	16	9214201	9214500	0.610	0.212	0.481	0.466
Tan	16	9306601	9306900	0.373	0.564	0.346	0.304
Tan	16	9362701	9363000	0.108	0.725	0.296	0.284
Tan	16	9411001	9411300	2.884	1.292	3.029	2.819
Tan	16	9477901	9478200	0.457	1.379	0.818	0.304
Tan	16	9501601	9501900	0.294	0.420	0.185	0.527
Tan	16	9626701	9627000	0.507	0.891	0.453	0.198
Tan	16	9650701	9651000	0.447	1.054	0.346	0.502
Tan	16	9676201	9676500	0.186	0.468	0.267	0.187
Tan	16	9689101	9689400	0.720	0.888	0.317	0.411
Tan	16	9941101	9941400	0.663	1.407	0.658	0.567
Tan	16	10000201	10000500	0.636	1.122	0.448	0.472
Tan	16	10155301	10155600	0.265	0.584	0.218	0.112
Tan	16	10264201	10264500	1.046	2.944	1.589	1.319
Tan	16	10269601	10269900	0.026	0.283	0.682	0.608
Tan	16	10486801	10487100	0.505	1.053	0.419	0.594
Tan	16	10487101	10487400	0.318	0.890	0.370	0.320
Tan	16	10658401	10658700	0.240	0.511	0.156	0.198
Tan	16	10667101	10667400	0.349	0.775	0.341	0.335
Tan	16	10817101	10817400	3.641	2.504	4.017	3.544
Tan	16	11334901	11335200	0.505	0.890	0.522	0.492
Tan	16	11355601	11355900	0.081	0.423	0.049	0.051
Tan	16	11460301	11460600	0.773	1.379	0.638	0.543
Tan	16	12004201	12004500	0.237	0.493	0.185	0.162
Tan	16	12296701	12297000	0.639	1.809	0.510	1.050
Tan	16	12425401	12425700	2.126	3.312	4.046	2.520
Tan	16	12586201	12586500	0.829	1.429	0.662	0.512
Tan	16	12682801	12683100	0.211	0.845	0.370	0.268
Tan	16	12869401	12869700	0.531	0.184	0.823	0.446
Tan	16	12911101	12911400	0.240	0.539	0.136	0.198
Tan	16	13227001	13227300	0.371	0.609	0.321	0.254
Tan	16	13476901	13477200	0.398	0.682	0.370	0.416
Tan	16	13675801	13676100	0.423	1.060	0.054	0.396
Tan	16	13731901	13732200	0.344	0.583	0.292	0.304
Tan	16	13755301	13755600	0.294	0.751	0.156	0.335
Tan	16	13869901	13870200	0.670	1.540	0.731	0.405
Tan	16	13870201	13870500	0.721	2.034	0.928	0.497
Tan	16	13877101	13877400	0.826	2.113	1.159	1.136
Tan	16	14754001	14754300	0.053	0.423	0.103	0.112
Tan	16	14809801	14810100	0.400	0.652	0.243	0.081
Tan	16	14821801	14822100	0.453	0.609	0.239	0.304
Tan	16	15354301	15354600	0.347	0.116	0.588	0.380
Tan	16	16870201	16870500	0.239	0.491	0.243	0.254

Tan	16	16934701	16935000	0.316	0.820	0.078	0.360
Tan	16	16995301	16995600	1.466	0.980	0.544	0.527
Tan	16	17044801	17045100	0.158	0.702	0.107	0.193
Tan	16	17072701	17073000	0.269	0.657	0.078	0.142
Tan	16	17671801	17672100	0.873	1.472	0.629	0.907
Tan	16	17726101	17726400	1.458	1.890	1.197	1.435
Tan	16	17738101	17738400	0.586	0.376	0.638	0.583
Tan	16	17763901	17764200	3.405	2.457	3.204	3.179
Tan	16	17975401	17975700	1.436	2.386	1.496	1.278
Tan	16	18307501	18307800	0.455	0.140	0.827	0.573
Tan	16	18402001	18402300	0.160	0.442	0.058	0.106
Tan	16	18751201	18751500	2.633	4.344	4.809	4.011
Tan	16	19032001	19032300	1.513	1.214	0.959	0.745
Tan	16	19476301	19476600	0.666	1.195	0.600	0.547
Tan	16	19823701	19824000	0.240	0.895	0.239	0.304
Tan	16	19933801	19934100	0.613	1.152	0.506	0.466
Tan	16	20368201	20368500	0.764	0.071	0.823	0.634
Tan	16	20368801	20369100	0.261	2.503	0.575	0.432
Tan	16	20369101	20369400	0.234	1.924	0.399	0.457
Tan	16	20374201	20374500	4.315	1.095	5.031	4.838
Tan	16	20374501	20374800	4.010	1.236	4.759	4.159
Tan	16	20595001	20595300	1.087	1.736	0.662	0.648
Tan	16	20619901	20620200	0.132	0.491	0.185	0.142
Tan	16	21262501	21262800	1.357	0.818	1.333	1.206
Tan	16	21347401	21347700	0.186	0.420	0.292	0.522
Tan	16	21573601	21573900	0.266	0.536	0.321	0.193
Tan	16	21612001	21612300	0.187	0.633	0.399	0.355
Tan	16	22187701	22188000	0.081	0.444	0.136	0.187
Tan	16	22343401	22343700	0.161	0.491	0.156	0.101
Tan	16	22631101	22631400	0.158	0.631	0.205	0.360
Tan	16	22842601	22842900	0.105	0.612	0.132	0.117
Tan	16	23058601	23058900	0.081	0.846	0.218	0.228
Tan	16	23066101	23066400	0.184	0.749	0.395	0.411
Tan	16	24155701	24156000	0.371	0.513	0.185	0.137
Tan	16	24268801	24269100	2.528	3.137	2.000	1.947
Tan	16	24282001	24282300	0.771	1.481	0.872	0.684
Tan	16	24318001	24318300	0.931	1.403	0.778	0.735
Tan	16	24345601	24345900	0.505	0.114	0.502	0.411
Tan	16	24385201	24385500	0.852	1.770	1.181	1.156
Tan	16	24471001	24471300	0.215	0.609	0.292	0.299
Tan	16	24479701	24480000	0.187	0.517	0.111	0.117
Tan	16	24634501	24634800	0.424	1.332	0.539	0.324
Tan	16	25358701	25359000	1.060	1.590	0.774	1.019
Tan	16	25522501	25522800	0.132	0.684	0.194	0.198
Tan	16	26179801	26180100	0.266	0.493	0.214	0.198
Tan	16	26476501	26476800	0.345	1.245	0.419	0.533
Tan	16	26832301	26832600	0.665	0.281	0.765	0.639
Tan	16	27596101	27596400	0.242	1.217	0.337	0.512
Tan	16	29022301	29022600	0.244	0.631	0.082	0.198
Tan	16	29136901	29137200	4.038	4.992	2.238	3.209
Tan	16	29655601	29655900	0.266	0.611	0.424	0.248
Tan	16	30251701	30252000	0.106	0.352	0.555	0.690
Tan	16	30475801	30476100	0.211	0.513	0.140	0.051
Tan	16	30633001	30633300	0.292	0.515	0.136	0.051
Tan	16	31327501	31327800	0.557	0.747	0.399	0.391
Tan	16	31398001	31398300	0.345	0.491	0.189	0.223
Tan	16	31436101	31436400	0.106	0.706	0.263	0.304
Tan	16	31480501	31480800	0.266	0.800	0.160	0.238
Tan	16	31884301	31884600	4.365	3.096	4.826	4.675
Tan	16	32523901	32524200	0.158	0.491	0.152	0.162
Tan	16	32636401	32636700	0.479	0.704	0.395	0.441
Tan	16	32649001	32649300	0.953	1.335	0.531	0.659
Tan	16	32773801	32774100	3.936	4.345	3.204	5.116
Tan	16	32947201	32947500	0.158	0.423	0.214	0.193
Tan	16	35118901	35119200	0.077	1.172	0.165	0.695
Tan	16	35366401	35366700	0.292	0.491	0.127	0.248
Tan	16	35655301	35655600	0.239	0.420	0.210	0.193

Tan	16	36272101	36272400	0.239	0.822	0.156	0.254
Tan	16	36476401	36476700	0.319	0.444	0.189	0.162
Tan	16	36661801	36662100	0.718	0.376	0.818	0.669
Tan	16	36703501	36703800	0.294	0.631	0.448	0.106
Tan	16	36844501	36844800	0.589	1.410	0.736	0.634
Tan	16	37174801	37175100	0.531	0.891	0.448	0.416
Tan	16	37364701	37365000	0.771	1.453	0.872	0.963
Tan	16	37365001	37365300	0.131	0.584	0.140	0.112
Tan	16	37417801	37418100	0.398	1.000	0.296	0.248
Tan	16	37553701	37554000	0.186	0.607	0.223	0.274
Tan	16	37607701	37608000	0.158	0.701	0.292	0.477
Tan	16	38058901	38059200	0.081	0.583	0.189	0.218
Tan	16	38718901	38719200	0.081	0.470	0.210	0.142
Tan	16	38727001	38727300	0.187	0.489	0.127	0.081
Tan	16	38895301	38895600	0.186	0.959	0.305	0.441
Tan	16	38946301	38946600	0.318	0.513	0.321	0.360
Tan	16	38961901	38962200	0.479	1.315	0.769	0.832
Tan	16	39112201	39112500	0.400	0.749	0.123	0.441
Tan	16	39173701	39174000	0.053	0.702	0.267	0.472
Tan	16	39493501	39493800	0.371	0.702	0.346	0.132
Tan	16	39508201	39508500	0.582	0.908	0.481	0.497
Tan	16	39646501	39646800	0.186	0.538	0.132	0.218
Tan	16	39935101	39935400	0.345	0.796	0.399	0.207
Tan	16	40229101	40229400	0.965	2.388	0.802	0.892
Tan	16	40932301	40932600	0.690	1.403	0.872	1.105
Tan	16	40956601	40956900	0.292	1.174	0.263	0.578
Tan	16	41387701	41388000	0.584	0.279	0.531	0.588
Tan	16	41460301	41460600	0.527	1.116	0.560	0.497
Tan	16	41818501	41818800	1.139	1.687	0.827	0.740
Tan	16	41823601	41823900	1.719	0.985	2.015	3.022
Tan	16	42310501	42310800	0.160	0.539	0.169	0.198
Tan	16	42526201	42526500	0.187	0.494	0.000	0.162
Tan	16	42767101	42767400	1.268	2.751	0.843	1.282
Tan	16	42932701	42933000	0.586	0.120	0.346	0.304
Tan	16	43080601	43080900	0.373	0.680	0.419	0.248
Tan	16	43673701	43674000	0.345	0.629	0.205	0.254
Tan	16	44142301	44142600	0.156	0.562	0.214	0.218
Tan	16	45939901	45940200	0.134	0.745	0.214	0.233
Tan	16	46800001	46800300	2.262	1.760	0.798	1.212
Tan	16	47388001	47388300	0.136	0.420	0.107	0.106
Tan	16	47484301	47484600	0.294	0.609	0.176	0.056
Tan	16	48045601	48045900	0.108	0.749	0.330	0.355
Tan	16	48795301	48795600	0.081	1.047	0.185	0.153
Tan	16	48795601	48795900	0.132	1.075	0.025	0.117
Tan	16	49682401	49682700	0.240	0.470	0.185	0.112
Tan	16	49682701	49683000	0.295	0.562	0.054	0.086
Tan	16	50220301	50220600	0.136	0.420	0.111	0.086
Tan	16	51285001	51285300	0.373	0.586	0.740	0.441
Tan	16	51301501	51301800	0.400	0.747	0.317	0.324
Tan	16	52847401	52847700	0.265	0.725	0.477	0.147
Tan	16	52923601	52923900	0.186	0.566	0.292	0.000
Tan	16	53932801	53933100	0.266	0.517	0.214	0.248
Tan	16	54326101	54326400	0.237	0.539	0.078	0.081
Tan	16	55110901	55111200	0.240	0.584	0.230	0.279
Tan	16	55182601	55182900	0.589	0.936	0.560	0.416
Tan	16	55190101	55190400	0.266	0.513	0.243	0.187
Tan	16	55866901	55867200	0.371	0.702	0.239	0.527
Tan	16	55895101	55895400	1.170	0.583	1.148	0.862
Tan	16	55966501	55966800	0.240	0.964	0.486	0.106
Tan	16	56011801	56012100	0.265	0.678	0.325	0.411
Tan	16	56135701	56136000	0.584	0.189	0.724	0.441
Tan	16	56305201	56305500	0.586	0.421	0.292	0.527
Tan	16	56362501	56362800	0.505	0.656	0.395	0.360
Tan	16	56373901	56374200	0.771	0.981	0.695	0.771
Tan	16	56518801	56519100	0.721	1.551	0.276	0.603
Tan	16	56519101	56519400	0.347	0.702	0.082	0.385
Tan	16	56733001	56733300	0.134	0.466	0.107	0.132

Tan	16	57483001	57483300	0.400	0.929	0.419	0.391
Tan	16	57549901	57550200	0.558	0.258	0.395	0.664
Tan	16	57659101	57659400	0.665	1.009	0.642	0.502
Tan	16	57793501	57793800	0.000	0.446	0.181	0.167
Tan	16	58115101	58115400	0.345	0.656	0.132	0.380
Tan	16	58140901	58141200	0.237	0.865	0.243	0.223
Tan	16	58200301	58200600	1.700	1.543	1.329	1.247
Tan	16	58217101	58217400	0.373	0.513	0.189	0.385
Tan	16	58333201	58333500	0.400	1.261	0.711	0.598
Tan	16	58574101	58574400	1.115	1.515	1.144	0.659
Tan	16	58722601	58722900	2.356	5.770	2.232	2.177
Tan	16	58722901	58723200	1.494	2.860	1.563	1.283
Tan	16	58772701	58773000	0.534	1.049	0.580	0.527
Tan	16	59099701	59100000	0.187	0.444	0.165	0.193
Tan	16	59391001	59391300	0.239	0.635	0.210	0.117
Tan	16	59392501	59392800	0.795	0.189	0.584	0.634
Tan	16	59735701	59736000	0.106	0.371	0.136	0.056
Tan	16	59879401	59879700	0.213	0.466	0.132	0.132
Tan	16	59879701	59880000	0.105	0.536	0.107	0.157
Tan	16	59957701	59958000	0.505	0.444	0.267	0.167
Tan	16	60487501	60487800	0.618	1.783	0.876	1.009
Tan	16	60653101	60653400	0.160	0.420	0.560	0.274
Tan	16	60941701	60942000	3.859	2.229	3.531	3.513
Tan	16	60947401	60947700	0.321	0.586	0.374	0.360
Tan	16	61120501	61120800	1.173	0.654	1.295	1.100
Tan	16	61528201	61528500	0.186	0.446	0.025	0.132
Tan	16	61755601	61755900	0.561	1.444	0.539	0.081
Tan	16	61965301	61965600	1.115	1.991	1.188	1.262
Tan	16	61990201	61990500	0.186	0.837	0.272	0.182
Tan	16	62007001	62007300	0.156	0.699	0.234	0.284
Tan	16	62088301	62088600	0.160	0.611	0.049	0.329
Tan	16	62088601	62088900	0.323	0.989	0.239	0.405
Tan	16	62361301	62361600	0.952	1.659	0.943	1.014
Tan	16	62381701	62382000	1.328	1.586	0.930	1.131
Tan	16	62394001	62394300	1.594	2.311	1.300	1.724
Tan	16	62405401	62405700	0.132	0.448	0.136	0.117
Tan	16	62450401	62450700	0.503	0.888	0.468	0.522
Tan	16	62450701	62451000	1.329	1.847	0.885	0.902
Tan	16	62459101	62459400	0.505	1.217	0.473	0.587
Tan	16	62459701	62460000	0.156	0.609	0.189	0.304
Tan	16	62507101	62507400	0.531	1.200	0.564	0.634
Tan	16	62589601	62589900	1.229	2.156	1.293	0.907
Tan	16	62646901	62647200	1.010	1.405	0.950	0.826
Tan	16	62650501	62650800	0.505	0.680	0.321	0.335
Tan	16	62695201	62695500	0.426	0.961	0.160	0.279
Tan	16	62695501	62695800	0.479	1.199	0.214	0.405
Tan	16	62724301	62724600	0.428	1.339	0.301	0.512
Tan	16	62889901	62890200	1.144	2.641	1.281	1.201
Tan	16	63087901	63088200	0.958	0.468	0.346	0.279
Tan	16	63642601	63642900	0.187	0.536	0.243	0.228
Tan	16	63715801	63716100	0.268	0.800	0.346	0.329
Tan	16	63754801	63755100	1.674	2.130	1.308	1.258
Tan	16	63755101	63755400	1.407	1.757	0.934	0.968
Tan	16	63806101	63806400	2.901	2.319	3.023	3.610
Tan	16	63920101	63920400	0.666	1.523	0.814	0.517
Tan	16	64014901	64015200	1.010	0.725	0.531	0.882
Tan	16	64559101	64559400	1.886	1.103	1.567	1.937
Tan	16	64732801	64733100	0.318	0.588	0.210	0.193
Tan	16	64773601	64773900	2.521	4.546	2.891	2.947
Tan	16	65190901	65191200	0.081	0.863	0.330	0.112
Tan	16	65191201	65191500	0.137	0.938	0.272	0.056
Tan	16	65195401	65195700	0.108	0.747	0.506	0.502
Tan	16	65211601	65211900	0.266	0.444	0.082	0.132
Tan	16	65331001	65331300	0.771	1.004	0.588	1.293
Tan	16	65584201	65584500	2.871	3.251	2.789	2.484
Tan	16	65725201	65725500	0.584	0.723	0.399	0.248
Tan	16	65854501	65854800	0.400	0.891	0.502	0.664

Tan	16	66012601	66012900	0.323	2.079	0.103	0.315
Tan	16	66012901	66013200	0.108	1.009	0.000	0.162
Tan	16	66191101	66191400	0.319	0.652	0.267	0.228
Tan	16	66247501	66247800	0.824	1.006	0.720	0.806
Tan	16	66420601	66420900	0.689	1.264	0.658	0.553
Tan	16	66456001	66456300	0.158	0.538	0.243	0.304
Tan	16	66461401	66461700	0.452	0.841	0.481	0.492
Tan	16	66813301	66813600	2.442	3.088	2.459	2.540
Tan	16	67032901	67033200	8.159	5.621	6.572	7.833
Tan	16	67209001	67209300	0.000	0.650	0.165	0.218
Tan	16	67389901	67390200	5.607	4.016	5.936	6.246
Tan	16	67796401	67796700	0.163	0.749	0.132	0.173
Tan	16	67933801	67934100	1.674	0.955	1.485	1.831
Tan	16	67934101	67934400	3.562	3.811	3.484	4.365
Tan	16	68052601	68052900	0.321	0.652	0.350	0.355
Tan	16	68161201	68161500	0.211	0.848	0.189	0.263
Tan	16	68226901	68227200	0.611	1.120	0.720	0.634
Tan	16	68259901	68260200	0.636	1.281	0.588	0.618
Tan	16	68286301	68286600	1.728	2.292	0.996	1.820
Tan	16	68388901	68389200	0.240	0.515	0.292	0.274
Tan	16	68422201	68422500	0.290	1.030	0.374	0.335
Tan	16	68516401	68516700	1.541	1.143	1.757	1.445
Tan	16	68524501	68524800	0.369	0.702	0.374	0.391
Tan	16	68573401	68573700	0.800	1.382	0.444	0.902
Tan	16	68962501	68962800	0.244	1.332	0.196	0.594
Tan	16	68962801	68963100	0.161	0.721	0.234	0.106
Tan	16	68968201	68968500	1.087	2.109	1.311	0.923
Tan	16	69027901	69028200	0.749	1.455	0.646	0.771
Tan	16	69066001	69066300	0.529	1.212	0.531	0.335
Tan	16	69092401	69092700	0.321	0.912	0.386	0.466
Tan	16	69133201	69133500	4.802	2.684	4.684	5.726
Tan	16	69133801	69134100	3.747	2.109	2.686	4.107
Tan	16	69264601	69264900	7.154	5.484	7.116	7.032
Tan	16	69284101	69284400	0.611	0.466	0.234	0.304
Tan	16	69373501	69373800	3.567	3.223	2.579	4.041
Tan	16	69452101	69452400	2.605	1.755	2.412	2.510
Tan	16	69476701	69477000	2.911	1.418	3.986	3.143
Tan	16	69483301	69483600	0.319	0.466	0.243	0.248
Tan	16	69576301	69576600	0.505	1.075	0.798	0.821
Tan	16	69649501	69649800	1.487	1.240	1.436	1.019
Tan	16	69696001	69696300	0.958	0.657	1.037	1.415
Tan	16	69699901	69700200	5.269	3.031	6.024	7.388
Tan	16	69757801	69758100	4.496	3.548	6.922	5.120
Tan	16	69760501	69760800	0.631	1.262	0.210	0.466
Tan	16	69766801	69767100	1.197	1.736	1.139	1.050
Tan	16	69774901	69775200	1.144	0.772	0.588	0.547
Tan	16	69777601	69777900	0.077	0.584	0.103	0.081
Tan	16	69786001	69786300	2.826	5.242	1.632	1.627
Tan	16	69786301	69786600	2.478	5.536	1.603	1.213
Tan	16	69787801	69788100	1.333	4.032	0.127	0.786
Tan	16	69789001	69789300	3.320	5.666	1.761	1.982
Tan	16	69789301	69789600	2.277	4.168	1.249	1.247
Tan	16	69789901	69790200	6.085	8.475	4.429	2.949
Tan	16	69791101	69791400	0.744	3.056	0.591	0.457
Tan	16	69791401	69791700	1.308	3.431	0.967	0.837
Tan	16	69794101	69794400	6.687	10.131	4.391	3.719
Tan	16	69796201	69796500	8.141	12.792	3.419	4.691
Tan	16	69796501	69796800	2.530	4.654	1.262	1.709
Tan	16	69797401	69797700	0.532	1.453	0.136	0.644
Tan	16	69798301	69798600	2.717	4.066	1.103	0.968
Tan	16	69798601	69798900	1.807	2.536	0.724	1.014
Tan	16	69800701	69801000	0.531	1.363	0.361	0.432
Tan	16	69801001	69801300	1.299	2.751	0.431	0.669
Tan	16	69829501	69829800	0.347	0.491	0.292	0.193
Tan	16	69851401	69851700	0.665	0.234	0.749	1.085
Tan	16	69978601	69978900	0.581	1.195	0.464	0.360
Tan	16	70009201	70009500	0.187	1.006	0.399	0.288

Tan	16	70010401	70010700	1.378	0.566	1.612	1.491
Tan	16	70023301	70023600	1.140	0.515	1.237	1.273
Tan	16	70160701	70161000	1.774	2.673	1.558	1.759
Tan	16	70289701	70290000	0.455	1.096	0.502	0.512
Tan	16	70329001	70329300	2.920	5.836	3.633	5.019
Tan	16	70415401	70415700	3.988	3.124	5.245	3.939
Tan	16	70438201	70438500	3.070	0.751	3.117	4.716
Tan	16	70468501	70468800	0.481	0.023	0.321	0.309
Tan	16	70507801	70508100	1.360	1.918	1.164	1.172
Tan	16	70508401	70508700	6.727	3.986	5.794	6.636
Tan	16	70508701	70509000	3.907	1.924	4.457	4.283
Tan	16	70529101	70529400	1.941	2.427	2.000	1.998
Tan	16	70542301	70542600	0.452	0.258	0.477	0.391
Tan	16	70646701	70647000	0.879	1.264	0.881	0.938
Tan	16	70666201	70666500	4.362	3.227	3.834	3.995
Tan	16	70698301	70698600	6.411	4.881	5.565	4.097
Tan	16	70702801	70703100	0.874	1.656	0.983	0.988
Tan	16	70875001	70875300	1.382	2.090	0.950	0.994
Tan	16	70908901	70909200	0.532	0.210	0.132	0.081
Tan	16	71064001	71064300	2.142	0.858	2.751	2.753
Tan	16	71079601	71079900	1.250	1.448	1.222	0.862
Tan	16	71105701	71106000	1.736	2.598	1.480	1.674
Tan	16	71199601	71199900	0.689	1.309	0.497	0.502
Tan	16	71205001	71205300	1.752	2.570	2.093	1.896
Tan	16	71418601	71418900	0.479	0.538	0.239	0.304
Tan	16	71430601	71430900	3.835	1.246	2.506	2.920
Tan	16	71438101	71438400	0.687	2.719	1.030	0.899
Tan	16	71468401	71468700	3.870	1.274	2.896	2.677
Tan	16	71499301	71499600	2.526	3.396	2.316	2.353
Tan	16	71689501	71689800	1.329	0.390	1.288	1.546
Tan	16	71699701	71700000	0.931	0.657	1.251	0.943
Tan	17	934201	934500	1.671	2.148	1.358	1.607
Tan	17	1250101	1250400	0.161	0.541	0.107	0.187
Tan	17	1589101	1589400	0.799	0.234	0.424	0.436
Tan	17	2189401	2189700	0.215	0.069	0.399	0.583
Tan	17	2748901	2749200	0.266	0.489	0.082	0.299
Tan	17	3079801	3080100	0.105	0.564	0.181	0.106
Tan	17	3480901	3481200	0.398	0.513	0.292	0.218
Tan	17	3528301	3528600	0.079	0.423	0.000	0.051
Tan	17	3658501	3658800	0.371	0.609	0.292	0.360
Tan	17	3666601	3666900	0.319	0.466	0.263	0.112
Tan	17	3723601	3723900	0.237	0.964	0.205	0.193
Tan	17	3980401	3980700	0.903	0.444	0.720	0.725
Tan	17	4000201	4000500	2.053	4.834	1.278	2.379
Tan	17	4000501	4000800	4.102	6.646	3.068	3.855
Tan	17	4017601	4017900	0.240	0.493	0.210	0.279
Tan	17	4018201	4018500	0.268	1.245	0.214	0.162
Tan	17	4044301	4044600	0.476	0.187	0.506	0.578
Tan	17	4053601	4053900	1.649	0.702	0.847	0.801
Tan	17	4071001	4071300	1.011	1.903	1.144	1.237
Tan	17	4099201	4099500	0.158	0.513	0.049	0.147
Tan	17	4209301	4209600	0.744	1.075	0.432	0.832
Tan	17	4283401	4283700	0.689	0.397	0.930	0.664
Tan	17	4318501	4318800	0.903	1.732	0.609	1.064
Tan	17	4683301	4683600	0.215	0.541	0.136	0.025
Tan	17	4802401	4802700	0.481	0.929	0.132	0.335
Tan	17	4810801	4811100	0.426	0.586	0.136	0.411
Tan	17	4863901	4864200	6.328	5.029	5.823	6.444
Tan	17	4946101	4946400	0.481	1.006	0.419	0.639
Tan	17	4947901	4948200	0.768	1.405	0.564	0.781
Tan	17	5205601	5205900	0.829	1.925	0.539	0.644
Tan	17	5358301	5358600	0.498	1.313	0.432	0.405
Tan	17	5358601	5358900	0.447	1.167	0.272	0.101
Tan	17	5517901	5518200	0.342	0.938	0.461	0.167
Tan	17	5761201	5761500	0.345	0.513	0.156	0.248
Tan	17	5836501	5836800	0.132	0.401	0.049	0.031
Tan	17	5911501	5911800	1.463	2.219	1.308	1.349

Tan	17	5943301	5943600	0.852	1.262	1.518	1.566
Tan	17	5967001	5967300	4.625	6.715	5.745	5.908
Tan	17	6108001	6108300	0.558	0.747	0.609	0.527
Tan	17	6277801	6278100	0.692	0.118	0.325	0.578
Tan	17	6444901	6445200	1.170	0.631	0.395	1.267
Tan	17	6528601	6528900	3.039	4.111	2.990	2.789
Tan	17	6615001	6615300	0.132	0.470	0.152	0.167
Tan	17	6736501	6736800	0.821	2.485	0.945	1.293
Tan	17	6819301	6819600	0.611	0.395	0.156	0.608
Tan	17	6887701	6888000	0.692	1.289	0.802	0.963
Tan	17	6956101	6956400	1.065	2.227	0.756	1.152
Tan	17	7318801	7319100	0.266	0.468	0.185	0.329
Tan	17	7517101	7517400	0.873	1.762	0.749	0.736
Tan	17	7517401	7517700	1.925	4.049	1.487	2.312
Tan	17	7787401	7787700	0.823	1.053	0.588	0.720
Tan	17	8339701	8340000	0.210	0.583	0.210	0.081
Tan	17	8657101	8657400	0.187	0.494	0.185	0.187
Tan	17	8717101	8717400	0.694	1.630	0.785	0.893
Tan	17	8758801	8759100	0.187	0.541	0.025	0.248
Tan	17	9325801	9326100	0.106	0.491	0.123	0.112
Tan	17	9511501	9511800	0.636	1.916	0.736	0.466
Tan	17	9511801	9512100	1.705	2.944	1.090	1.566
Tan	17	9984601	9984900	0.053	0.257	0.000	0.578
Tan	17	10203301	10203600	0.505	0.796	0.317	0.507
Tan	17	10475701	10476000	0.503	1.493	1.003	0.558
Tan	17	10491901	10492200	0.218	1.124	0.346	0.157
Tan	17	10879801	10880100	0.369	0.796	0.350	0.243
Tan	17	11049301	11049600	0.132	0.466	0.218	0.162
Tan	17	11359501	11359800	0.161	0.493	0.218	0.162
Tan	17	11528401	11528700	0.239	0.534	0.239	0.243
Tan	17	11562901	11563200	0.000	0.444	0.152	0.106
Tan	17	11721901	11722200	1.439	1.821	0.691	0.695
Tan	17	11827201	11827500	0.663	0.933	0.502	0.634
Tan	17	12107101	12107400	0.294	0.444	0.214	0.223
Tan	17	12404101	12404400	0.936	1.687	1.032	1.009
Tan	17	12407401	12407700	0.534	0.959	0.633	0.243
Tan	17	12444601	12444900	0.932	1.290	0.852	0.553
Tan	17	12480001	12480300	2.712	2.036	2.584	2.850
Tan	17	12540301	12540600	0.821	0.187	0.658	0.700
Tan	17	12870301	12870600	0.984	0.562	0.424	0.852
Tan	17	12897001	12897300	0.189	0.635	0.239	0.137
Tan	17	13098901	13099200	0.452	0.562	0.691	0.329
Tan	17	13110601	13110900	0.263	0.607	0.160	0.142
Tan	17	13685701	13686000	0.477	0.163	0.453	0.279
Tan	17	13710301	13710600	0.318	0.609	0.239	0.304
Tan	17	14070601	14070900	0.106	0.517	0.127	0.248
Tan	17	14130301	14130600	0.027	0.395	0.111	0.086
Tan	17	14196301	14196600	0.105	0.702	0.111	0.162
Tan	17	15249301	15249600	0.294	0.794	0.140	0.315
Tan	17	15317701	15318000	0.289	1.075	0.386	0.315
Tan	17	15480001	15480300	0.160	0.442	0.243	0.086
Tan	17	15504601	15504900	0.081	0.448	0.078	0.137
Tan	17	15597001	15597300	1.515	1.167	0.662	0.852
Tan	17	15692701	15693000	0.186	0.446	0.078	0.051
Tan	17	16438501	16438800	0.265	1.081	0.642	0.634
Tan	17	16712401	16712700	0.105	0.605	0.317	0.142
Tan	17	16721701	16722000	0.636	1.667	1.139	0.690
Tan	17	16748401	16748700	0.052	0.371	0.082	0.000
Tan	17	17001901	17002200	0.582	1.077	0.802	0.497
Tan	17	17118001	17118300	1.110	2.002	0.814	0.994
Tan	17	17157901	17158200	0.799	0.348	0.617	0.695
Tan	17	17567701	17568000	2.412	1.009	2.192	1.876
Tan	17	17576101	17576400	0.395	0.936	0.354	0.340
Tan	17	17698801	17699100	0.558	0.676	0.239	0.472
Tan	17	17750701	17751000	0.584	0.980	0.312	0.547
Tan	17	17785201	17785500	1.168	2.156	1.280	1.035
Tan	17	18152701	18153000	0.400	0.890	0.535	0.639

Tan	17	18493501	18493800	0.640	1.008	0.555	0.502
Tan	17	18616201	18616500	0.131	0.611	0.087	0.173
Tan	17	19077001	19077300	0.103	0.562	0.185	0.187
Tan	17	19476001	19476300	0.318	0.727	0.214	0.259
Tan	17	20115301	20115600	0.134	0.395	0.160	0.112
Tan	17	20940901	20941200	0.079	0.448	0.160	0.193
Tan	17	21108601	21108900	0.106	0.448	0.210	0.056
Tan	17	21420901	21421200	0.319	0.444	0.321	0.218
Tan	17	22224001	22224300	0.053	0.378	0.078	0.031
Tan	17	22458301	22458600	0.134	0.515	0.107	0.228
Tan	17	22879501	22879800	0.213	0.494	0.132	0.187
Tan	17	24308101	24308400	0.266	0.607	0.370	0.472
Tan	17	24426901	24427200	0.505	0.723	0.481	0.335
Tan	17	24861901	24862200	0.263	0.538	0.214	0.198
Tan	17	25255801	25256100	0.077	0.491	0.107	0.142
Tan	17	25302001	25302300	0.505	0.745	0.350	0.360
Tan	17	25351801	25352100	0.400	0.607	0.218	0.056
Tan	17	25949701	25950000	0.586	0.421	0.156	0.274
Tan	17	26396101	26396400	0.082	0.491	0.127	0.198
Tan	17	26707201	26707500	0.290	0.517	0.054	0.193
Tan	17	27284101	27284400	0.052	0.395	0.025	0.167
Tan	17	28695001	28695300	0.876	0.538	0.160	0.821
Tan	17	28743901	28744200	0.531	0.727	0.132	0.365
Tan	17	28822201	28822500	0.081	0.442	0.132	0.248
Tan	17	28850701	28851000	0.290	0.513	0.292	0.279
Tan	17	29658901	29659200	0.295	0.562	0.025	0.112
Tan	17	29738101	29738400	0.347	0.657	0.263	0.355
Tan	17	29914201	29914500	0.077	0.519	0.078	0.000
Tan	17	30083701	30084000	0.160	0.466	0.292	0.335
Tan	17	30945001	30945300	0.773	1.671	0.573	0.634
Tan	17	32232601	32232900	0.186	0.446	0.185	0.248
Tan	17	32477701	32478000	0.398	0.607	0.502	0.360
Tan	17	32847001	32847300	0.106	0.511	0.058	0.081
Tan	17	32847301	32847600	0.136	0.740	0.029	0.162
Tan	17	32889301	32889600	0.134	0.395	0.103	0.051
Tan	17	32927101	32927400	1.515	2.452	1.414	1.517
Tan	17	33069901	33070200	0.531	0.678	0.506	0.527
Tan	17	33070801	33071100	0.052	0.444	0.078	0.193
Tan	17	33378301	33378600	0.187	0.423	0.103	0.106
Tan	17	33911101	33911400	0.981	1.967	0.809	1.044
Tan	17	33911401	33911700	1.176	1.948	0.892	1.136
Tan	17	34027501	34027800	0.218	0.938	0.312	0.238
Tan	17	34875001	34875300	0.347	0.678	0.350	0.147
Tan	17	35435401	35435700	0.531	0.839	0.535	0.436
Tan	17	35484901	35485200	0.237	0.609	0.346	0.117
Tan	17	36153301	36153600	0.132	0.657	0.136	0.243
Tan	17	36840001	36840300	0.718	1.261	0.526	0.537
Tan	17	36891301	36891600	0.522	1.659	0.382	0.375
Tan	17	37689001	37689300	0.474	0.985	0.444	0.497
Tan	17	38662201	38662500	0.026	0.395	0.107	0.106
Tan	17	38917501	38917800	0.132	0.442	0.111	0.137
Tan	17	39103201	39103500	0.507	0.931	0.341	0.583
Tan	17	39252901	39253200	0.905	0.562	1.045	0.963
Tan	17	39321601	39321900	0.373	1.000	0.374	0.391
Tan	17	39411001	39411300	0.184	0.493	0.210	0.051
Tan	17	39423001	39423300	0.132	0.423	0.103	0.137
Tan	17	39531001	39531300	0.373	0.515	0.823	0.416
Tan	17	39692401	39692700	0.053	0.420	0.152	0.112
Tan	17	40698601	40698900	0.132	0.538	0.214	0.254
Tan	17	41202601	41202900	0.747	1.521	0.745	0.943
Tan	17	41252101	41252400	0.505	0.258	0.687	0.527
Tan	17	41520901	41521200	0.879	1.571	0.798	1.131
Tan	17	41895001	41895300	0.347	0.560	0.214	0.254
Tan	17	41996401	41996700	0.610	1.075	0.695	0.654
Tan	17	42192001	42192300	0.211	0.517	0.132	0.274
Tan	17	42279301	42279600	0.213	0.466	0.214	0.117
Tan	17	42797101	42797400	0.239	0.468	0.234	0.248

Tan	17	43429501	43429800	0.053	0.420	0.103	0.142
Tan	17	43664101	43664400	0.240	0.513	0.346	0.254
Tan	17	43857301	43857600	0.689	1.506	0.765	0.751
Tan	17	43955701	43956000	0.160	0.558	0.127	0.173
Tan	17	44011801	44012100	1.192	2.139	0.802	0.761
Tan	17	44035801	44036100	0.689	0.279	0.736	0.857
Tan	17	44120701	44121000	1.242	2.223	3.576	3.047
Tan	17	44136301	44136600	2.342	1.961	1.505	2.484
Tan	17	44267101	44267400	3.409	1.659	2.494	2.961
Tan	17	44302501	44302800	1.199	0.674	1.440	1.425
Tan	17	44506801	44507100	0.903	0.161	0.588	0.913
Tan	17	44564401	44564700	0.937	1.852	0.999	0.806
Tan	17	45105001	45105300	0.213	0.493	0.214	0.309
Tan	17	45222001	45222300	1.460	0.185	0.809	0.954
Tan	17	45236401	45236700	0.718	0.092	0.593	0.533
Tan	17	45893101	45893400	0.055	0.371	0.025	0.081
Tan	17	46108201	46108500	0.053	0.515	0.292	0.228
Tan	17	46771201	46771500	0.268	0.562	0.259	0.106
Tan	17	46839001	46839300	0.557	1.334	0.633	0.324
Tan	17	46965301	46965600	0.531	0.820	0.506	0.522
Tan	17	47034601	47034900	1.889	0.985	1.480	1.359
Tan	17	47165101	47165400	2.423	3.343	2.608	2.115
Tan	17	47338501	47338800	0.290	0.796	0.243	0.547
Tan	17	47658301	47658600	0.586	1.195	0.651	0.431
Tan	17	47836801	47837100	0.503	0.933	0.288	0.614
Tan	17	48097201	48097500	0.452	0.680	0.292	0.492
Tan	17	48117001	48117300	1.408	0.727	1.144	0.887
Tan	17	48251701	48252000	0.211	0.605	0.239	0.248
Tan	17	49108801	49109100	0.584	0.841	0.473	0.243
Tan	17	49243501	49243800	0.134	0.701	0.341	0.218
Tan	17	49386301	49386600	48.941	57.241	40.551	48.033
Tan	17	49483801	49484100	0.294	0.633	0.424	0.360
Tan	17	49647601	49647900	0.290	0.680	0.239	0.268
Tan	17	49765201	49765500	0.290	0.701	0.078	0.056
Tan	17	49765501	49765800	0.235	0.654	0.127	0.031
Tan	17	49852501	49852800	0.027	0.498	0.025	0.025
Tan	17	49852801	49853100	0.082	0.588	0.025	0.051
Tan	17	50151901	50152200	0.294	0.772	0.473	0.162
Tan	17	50211001	50211300	0.215	0.538	0.272	0.274
Tan	17	50267101	50267400	0.318	0.933	0.247	0.304
Tan	17	50339701	50340000	0.402	1.262	0.403	0.751
Tan	17	50618401	50618700	0.079	0.519	0.189	0.167
Tan	17	50887801	50888100	0.106	0.465	0.214	0.162
Tan	17	50919301	50919600	0.426	1.122	0.651	0.578
Tan	17	51120601	51120900	1.066	1.822	1.016	1.039
Tan	17	51902401	51902700	0.211	0.558	0.127	0.142
Tan	17	51920401	51920700	1.436	2.133	1.251	1.308
Tan	17	52037101	52037400	0.000	0.489	0.132	0.157
Tan	17	52081801	52082100	0.347	0.770	0.526	0.248
Tan	17	52167301	52167600	0.557	1.216	0.794	0.837
Tan	17	52199701	52200000	0.266	0.560	0.136	0.243
Tan	17	52365601	52365900	0.292	0.539	0.292	0.284
Tan	17	52372201	52372500	1.595	2.399	1.761	1.638
Tan	17	52689301	52689600	0.852	1.513	0.876	0.968
Tan	17	52825201	52825500	1.750	2.549	1.300	1.424
Tan	17	52992901	52993200	0.397	0.629	0.374	0.385
Tan	17	53046301	53046600	1.881	2.708	1.790	1.856
Tan	17	53130601	53130900	0.294	0.607	0.132	0.472
Tan	17	53136301	53136600	0.637	0.189	0.526	0.700
Tan	17	53338501	53338800	0.794	1.290	0.695	0.639
Tan	17	53461801	53462100	0.239	0.423	0.107	0.081
Tan	17	53577601	53577900	0.479	0.725	0.379	0.274
Tan	17	53594701	53595000	0.637	1.712	0.930	1.070
Tan	17	53780101	53780400	0.213	0.678	0.424	0.502
Tan	17	54098101	54098400	0.131	0.517	0.165	0.142
Tan	17	54180901	54181200	0.642	0.116	0.740	1.100
Tan	17	54325501	54325800	0.158	0.395	0.160	0.167

Tan	17	54629401	54629700	1.570	2.173	1.534	1.856
Tan	17	54750301	54750600	0.319	0.611	0.325	0.218
Tan	17	54889501	54889800	0.323	0.914	0.584	0.441
Tan	17	54889801	54890100	0.663	2.019	1.271	0.978
Tan	17	55765801	55766100	0.452	0.678	0.243	0.466
Tan	17	56731201	56731500	0.211	0.444	0.292	0.274
Tan	17	56879701	56880000	0.878	1.189	0.740	0.857
Tan	17	56919601	56919900	2.552	0.986	3.205	2.540
Tan	17	56929201	56929500	0.608	1.053	0.671	0.466
Tan	17	57193201	57193500	0.907	0.371	0.749	1.156
Tan	17	57304501	57304800	2.367	1.450	1.875	1.947
Tan	17	57433201	57433500	0.292	0.611	0.399	0.279
Tan	17	57589801	57590100	0.794	2.075	0.519	0.533
Tan	17	57635701	57636000	0.318	0.493	0.214	0.248
Tan	17	58032901	58033200	0.395	0.725	0.267	0.274
Tan	17	58354501	58354800	0.557	1.034	0.457	0.391
Tan	17	58660201	58660500	0.477	1.073	0.551	0.806
Tan	17	58822501	58822800	0.265	0.680	0.272	0.132
Tan	17	58993501	58993800	3.004	2.662	1.855	2.130
Tan	17	59629801	59630100	0.453	0.631	0.453	0.299
Tan	17	59760901	59761200	0.373	0.725	0.580	0.329
Tan	17	59778301	59778600	0.507	0.916	0.531	0.527
Tan	17	59793301	59793600	0.213	0.444	0.205	0.056
Tan	17	59868001	59868300	0.371	0.916	0.468	0.187
Tan	17	59935801	59936100	0.240	1.171	0.560	0.679
Tan	17	60014701	60015000	0.081	0.444	0.127	0.198
Tan	17	60119101	60119400	0.055	0.671	0.156	0.167
Tan	17	60123301	60123600	4.335	5.390	4.135	3.336
Tan	17	60194401	60194700	0.319	0.747	0.230	0.198
Tan	17	60569101	60569400	0.266	0.611	0.185	0.355
Tan	17	60673801	60674100	2.207	2.976	2.086	2.140
Tan	17	60683101	60683400	0.186	0.421	0.531	0.304
Tan	17	60685801	60686100	0.239	0.609	0.292	0.248
Tan	17	60686101	60686400	1.673	2.609	1.699	1.512
Tan	17	60758401	60758700	0.350	0.929	0.218	0.193
Tan	17	60860701	60861000	0.398	0.961	0.720	0.436
Tan	17	60904201	60904500	2.057	3.703	1.466	1.906
Tan	17	60905701	60906000	0.131	0.839	0.205	0.360
Tan	17	60959401	60959700	0.450	0.701	0.457	0.329
Tan	17	60969901	60970200	0.615	1.279	0.749	0.578
Tan	17	61092601	61092900	1.303	0.416	1.180	1.125
Tan	17	61185301	61185600	0.424	0.723	0.346	0.578
Tan	17	61311601	61311900	0.745	1.356	0.691	0.720
Tan	17	61419601	61419900	0.453	0.869	0.267	0.578
Tan	17	61989901	61990200	0.237	0.560	0.346	0.274
Tan	17	62107201	62107500	3.445	4.489	2.905	3.276
Tan	17	62142901	62143200	1.116	0.817	0.584	1.050
Tan	17	62175301	62175600	4.596	6.213	3.061	4.453
Tan	17	62757301	62757600	3.752	4.422	3.331	3.559
Tan	17	62816101	62816400	0.745	1.217	0.437	0.826
Tan	17	63124201	63124500	0.531	1.099	0.736	0.583
Tan	17	63582901	63583200	0.853	0.584	0.321	0.877
Tan	17	63678301	63678600	1.139	2.600	1.683	1.166
Tan	17	63960001	63960300	0.292	0.837	0.214	0.243
Tan	17	63982501	63982800	4.961	7.520	3.832	4.782
Tan	17	63983401	63983700	0.110	0.513	0.165	0.051
Tan	17	63983701	63984000	0.216	0.515	0.082	0.193
Tan	17	63997801	63998100	0.424	0.916	0.234	0.466
Tan	17	64064401	64064700	1.757	1.141	2.250	1.957
Tan	17	64064701	64065000	4.785	3.362	7.110	5.740
Tan	17	64086601	64086900	0.495	2.371	0.428	0.735
Tan	17	64124701	64125000	0.161	0.680	0.189	0.248
Tan	17	64131601	64131900	1.302	1.054	0.560	0.573
Tan	17	64196401	64196700	0.397	0.794	0.390	0.304
Tan	17	64295701	64296000	0.447	1.236	0.551	0.132
Tan	17	64409401	64409700	0.400	1.049	0.308	0.324
Tan	17	64565401	64565700	0.266	0.730	0.292	0.254

Tan	17	64736701	64737000	1.939	1.210	1.893	1.865
Tan	17	64932901	64933200	1.145	0.657	0.239	1.186
Tan	17	64954801	64955100	1.539	0.841	1.255	1.186
Tan	17	64964401	64964700	0.265	0.468	0.210	0.162
Tan	17	65166001	65166300	0.611	0.865	0.428	0.492
Tan	17	65405101	65405400	0.211	0.820	0.103	0.051
Tan	17	65427301	65427600	0.186	0.448	0.082	0.167
Tan	17	65462101	65462400	0.874	1.219	0.453	0.751
Tan	17	65538901	65539200	0.131	0.446	0.103	0.051
Tan	17	65584501	65584800	0.824	0.607	0.477	0.776
Tan	17	65645401	65645700	0.799	0.403	0.798	0.826
Tan	17	65871601	65871900	2.021	2.776	1.708	1.714
Tan	17	65909401	65909700	0.160	0.423	0.127	0.167
Tan	17	65925001	65925300	5.061	3.443	4.907	5.395
Tan	17	65933401	65933700	3.268	2.148	3.036	3.128
Tan	17	65937901	65938200	0.316	0.905	0.288	0.228
Tan	17	66050401	66050700	0.982	0.586	1.139	0.979
Tan	17	66072301	66072600	1.170	0.494	1.081	1.075
Tan	17	66103801	66104100	0.558	0.375	0.263	0.223
Tan	17	66267601	66267900	0.187	0.423	0.156	0.137
Tan	17	66299401	66299700	0.350	1.030	0.453	0.482
Tan	17	66394501	66394800	0.053	0.603	0.098	0.076
Tan	17	66399001	66399300	4.812	2.457	4.495	3.716
Tan	17	66399301	66399600	4.362	3.205	4.646	4.153
Tan	17	66423901	66424200	2.579	0.257	2.570	2.143
Tan	17	66548101	66548400	1.115	2.021	0.916	0.943
Tan	17	66687601	66687900	0.589	2.545	1.447	0.553
Tan	17	66687901	66688200	0.184	0.702	0.321	0.173
Tan	17	66708601	66708900	0.773	1.581	0.555	0.796
Tan	17	66709201	66709500	3.612	1.699	3.898	3.626
Tan	17	66709501	66709800	3.331	1.487	4.025	3.621
Tan	17	66727801	66728100	0.426	1.273	0.419	0.456
Tan	17	66728101	66728400	0.505	1.670	0.638	0.538
Tan	17	66747301	66747600	2.758	1.047	2.151	1.602
Tan	17	66748501	66748800	3.455	2.218	3.504	4.569
Tan	17	66776101	66776400	0.344	2.377	0.354	0.299
Tan	17	66884401	66884700	0.268	0.963	0.497	0.547
Tan	17	66945001	66945300	2.020	1.191	1.358	1.410
Tan	17	66955801	66956100	0.268	0.773	0.506	0.274
Tan	17	66964801	66965100	1.116	1.335	0.720	0.913
Tan	17	67139701	67140000	0.240	0.446	0.025	0.223
Tan	17	67654801	67655100	0.477	0.326	0.477	0.193
Tan	17	67786501	67786800	0.824	1.309	0.473	0.700
Tan	17	67871701	67872000	0.079	0.468	0.234	0.634
Tan	17	67928101	67928400	0.132	0.468	0.136	0.132
Tan	17	68011501	68011800	1.494	2.573	1.420	1.562
Tan	17	68075701	68076000	0.586	0.701	0.428	0.527
Tan	17	68234101	68234400	0.898	4.021	1.400	0.690
Tan	17	68236201	68236500	0.799	0.935	0.662	0.608
Tan	17	68238301	68238600	0.053	0.541	0.243	0.142
Tan	17	68268601	68268900	0.557	1.262	0.346	0.446
Tan	17	68281501	68281800	0.187	0.493	0.156	0.223
Tan	17	68330701	68331000	6.275	8.483	4.975	6.681
Tan	17	68341501	68341800	2.423	3.867	2.111	2.339
Tan	17	68366401	68366700	1.565	0.414	1.472	1.339
Tan	17	68367001	68367300	1.542	3.064	2.798	1.552
Tan	17	68406001	68406300	3.407	0.720	3.367	4.370
Tan	17	68419201	68419500	0.613	2.152	0.876	0.745
Tan	17	68419501	68419800	0.771	2.704	0.399	0.857
Tan	17	68480401	68480700	0.455	1.759	0.756	0.335
Tan	17	68511001	68511300	1.323	3.452	1.570	1.228
Tan	17	68537101	68537400	1.244	4.882	1.441	1.293
Tan	17	68537401	68537700	0.136	1.180	0.156	0.056
Tan	17	68657101	68657400	0.795	1.661	0.662	0.963
Tan	17	68695201	68695500	0.160	0.538	0.049	0.092
Tan	17	68704201	68704500	4.207	2.772	4.790	4.827
Tan	17	68718601	68718900	0.187	0.444	0.136	0.254

Tan	17	68724601	68724900	0.824	1.498	0.419	0.654
Tan	17	68743801	68744100	0.189	0.446	0.156	0.056
Tan	17	68781301	68781600	0.639	1.352	0.152	0.472
Tan	17	68784601	68784900	1.757	2.888	1.081	1.282
Tan	17	68784901	68785200	2.052	3.624	1.708	2.134
Tan	17	68789701	68790000	7.903	4.851	8.141	8.013
Tan	17	68790901	68791200	6.507	2.371	6.526	6.684
Tan	17	68791501	68791800	1.650	2.721	1.066	1.364
Tan	17	68803801	68804100	1.882	0.253	1.324	1.247
Tan	17	68918401	68918700	0.319	0.444	0.321	0.248
Tan	17	69056701	69057000	0.481	0.772	0.317	0.360
Tan	17	69108001	69108300	0.242	0.586	0.239	0.228
Tan	17	69136501	69136800	0.591	2.379	0.999	0.942
Tan	17	69152101	69152400	0.507	0.257	0.448	0.446
Tan	17	69173101	69173400	1.113	2.311	1.636	1.019
Tan	17	69187501	69187800	2.772	2.124	3.212	2.961
Tan	17	69390001	69390300	2.792	1.392	2.497	2.301
Tan	17	69523801	69524100	4.185	5.742	3.591	4.284
Tan	17	69640201	69640500	1.889	1.266	1.567	2.124
Tan	17	69754201	69754500	3.586	4.109	3.718	3.154
Tan	17	69888901	69889200	1.731	2.686	1.937	2.033
Tan	17	69908401	69908700	1.887	0.886	1.259	1.364
Tan	17	69951001	69951300	1.434	2.225	1.547	1.561
Tan	17	70024801	70025100	1.541	1.847	0.662	1.181
Tan	17	70197001	70197300	0.106	0.257	0.502	0.051
Tan	17	70329601	70329900	9.340	5.531	10.307	9.872
Tan	17	70368001	70368300	2.881	5.886	3.495	3.858
Tan	17	70374601	70374900	6.629	3.198	7.420	6.116
Tan	17	70389301	70389600	0.610	1.004	0.584	0.268
Tan	17	70431601	70431900	2.260	1.377	3.536	2.484
Tan	17	70591801	70592100	3.756	4.859	3.375	3.747
Tan	17	70710301	70710600	1.460	2.062	1.703	1.273
Tan	17	70786801	70787100	0.134	0.468	0.103	0.092
Tan	17	70828801	70829100	0.640	0.163	0.560	0.573
Tan	17	71080801	71081100	0.398	0.092	0.584	0.335
Tan	17	71129401	71129700	1.323	2.330	1.045	0.756
Tan	17	71630401	71630700	1.784	2.837	1.797	1.851
Tan	17	71928901	71929200	0.692	1.126	0.539	0.745
Tan	17	72067801	72068100	1.776	2.568	1.962	1.552
Tan	17	72114301	72114600	0.855	0.229	0.818	1.019
Tan	18	6301	6600	1.386	1.849	1.168	1.369
Tan	18	80101	80400	0.371	0.845	0.560	0.492
Tan	18	101701	102000	1.784	2.971	1.086	1.582
Tan	18	120301	120600	1.087	1.568	0.881	1.273
Tan	18	1752901	1753200	0.316	0.560	0.029	0.025
Tan	18	1868701	1869000	0.507	0.725	0.239	0.355
Tan	18	1886401	1886700	1.649	2.669	1.750	1.343
Tan	18	1978201	1978500	3.191	2.242	3.179	3.448
Tan	18	1995001	1995300	0.345	0.609	0.272	0.193
Tan	18	3245701	3246000	0.428	1.569	0.451	0.214
Tan	18	3966601	3966900	1.170	0.373	0.609	0.771
Tan	18	4273501	4273800	0.105	0.468	0.234	0.167
Tan	18	4719301	4719600	2.628	4.278	2.760	2.630
Tan	18	5099401	5099700	0.345	1.148	0.428	0.477
Tan	18	5121901	5122200	5.472	4.628	3.277	3.736
Tan	18	5176801	5177100	0.373	0.747	0.457	0.254
Tan	18	5492701	5493000	0.903	0.609	0.852	0.720
Tan	18	5637001	5637300	0.189	0.489	0.136	0.106
Tan	18	5654701	5655000	4.465	1.493	4.068	4.337
Tan	18	5905201	5905500	0.053	0.652	0.223	0.092
Tan	18	6734701	6735000	1.439	0.865	1.592	1.724
Tan	18	6891301	6891600	0.876	0.560	0.905	0.999
Tan	18	7035601	7035900	0.345	0.586	0.341	0.385
Tan	18	7477201	7477500	0.768	1.380	0.760	0.654
Tan	18	7502701	7503000	0.292	1.285	0.626	0.370
Tan	18	8051101	8051400	0.321	0.468	0.107	0.137
Tan	18	8079901	8080200	0.477	0.305	0.156	0.000

Tan	18	8127901	8128200	0.129	1.099	0.239	0.426
Tan	18	8128201	8128500	0.077	0.584	0.181	0.279
Tan	18	8145001	8145300	0.265	0.609	0.296	0.228
Tan	18	8278501	8278800	2.362	1.113	2.559	2.606
Tan	18	8489101	8489400	0.371	0.721	0.346	0.086
Tan	18	8740801	8741100	0.186	0.489	0.210	0.132
Tan	18	9022501	9022800	0.106	0.423	0.160	0.218
Tan	18	9218701	9219000	0.426	0.820	0.415	0.335
Tan	18	9273601	9273900	0.424	0.629	0.292	0.218
Tan	18	9341101	9341400	0.611	0.489	0.160	0.223
Tan	18	9355501	9355800	0.474	1.150	0.312	0.254
Tan	18	9402301	9402600	0.423	1.098	0.435	0.396
Tan	18	9604801	9605100	1.645	2.707	1.806	1.865
Tan	18	9804001	9804300	0.344	0.631	0.165	0.248
Tan	18	9823501	9823800	0.266	0.607	0.292	0.243
Tan	18	10109101	10109400	2.712	1.731	1.913	2.043
Tan	18	10286401	10286700	0.081	0.371	0.029	0.076
Tan	18	10578901	10579200	0.505	0.350	0.210	0.522
Tan	18	10882501	10882800	0.878	1.339	0.847	0.857
Tan	18	10919701	10920000	0.344	0.609	0.374	0.329
Tan	18	11078701	11079000	0.452	0.609	0.263	0.218
Tan	18	11171101	11171400	1.063	2.669	1.839	1.775
Tan	18	11226901	11227200	0.158	0.562	0.214	0.329
Tan	18	11425801	11426100	2.368	4.201	2.689	2.555
Tan	18	11570401	11570700	0.184	0.562	0.156	0.081
Tan	18	11704501	11704800	0.402	1.008	0.695	0.309
Tan	18	11704801	11705100	0.907	1.659	1.050	0.781
Tan	18	12015901	12016200	0.477	0.116	0.296	0.279
Tan	18	12036601	12036900	1.010	2.665	1.289	1.182
Tan	18	12052501	12052800	1.328	2.358	1.708	1.505
Tan	18	12075001	12075300	0.689	0.957	2.361	1.323
Tan	18	12094501	12094800	0.134	0.494	0.160	0.167
Tan	18	12442201	12442500	1.753	1.380	1.946	3.088
Tan	18	12669901	12670200	0.345	0.607	0.399	0.254
Tan	18	12811201	12811500	1.199	0.633	0.374	0.279
Tan	18	12969601	12969900	1.649	0.929	1.937	1.415
Tan	18	13073401	13073700	1.863	2.343	1.255	1.557
Tan	18	13117801	13118100	0.347	0.068	0.502	0.436
Tan	18	13118101	13118400	0.265	0.023	0.584	0.360
Tan	18	13122301	13122600	0.476	0.747	0.395	0.472
Tan	18	13125601	13125900	0.265	0.564	0.267	0.274
Tan	18	13194601	13194900	0.661	1.802	0.682	0.624
Tan	18	13479301	13479600	0.213	0.376	0.584	0.720
Tan	18	13881901	13882200	0.268	0.751	0.111	0.385
Tan	18	14058001	14058300	2.287	3.070	1.554	1.917
Tan	18	14272501	14272800	0.292	1.223	0.136	0.319
Tan	18	14301601	14301900	0.742	1.289	0.729	0.745
Tan	18	14387401	14387700	0.747	2.193	1.242	1.091
Tan	18	15227101	15227400	0.958	1.822	0.455	0.542
Tan	18	15369301	15369600	0.240	0.747	0.346	0.106
Tan	18	15400201	15400500	0.560	1.049	0.604	0.299
Tan	18	15400501	15400800	1.417	2.500	1.572	1.095
Tan	18	15480001	15480300	0.347	0.609	0.317	0.329
Tan	18	15495901	15496200	0.479	1.227	0.267	0.461
Tan	18	15535801	15536100	0.405	1.974	1.173	0.862
Tan	18	15552601	15552900	0.213	0.631	0.098	0.198
Tan	18	15589501	15589800	2.849	1.326	2.599	2.196
Tan	18	15615301	15615600	6.117	3.536	4.839	4.654
Tan	18	15658201	15658500	5.720	7.026	5.845	5.187
Tan	18	15685801	15686100	2.260	3.779	2.015	2.531
Tan	18	15715801	15716100	3.835	5.340	3.010	3.570
Tan	18	15872401	15872700	0.026	0.371	0.054	0.112
Tan	18	15978901	15979200	0.189	0.702	0.160	0.218
Tan	18	15979201	15979500	0.539	1.886	0.620	0.466
Tan	18	16005901	16006200	0.453	0.184	0.555	0.664
Tan	18	16023901	16024200	1.226	2.017	1.300	1.485
Tan	18	16026901	16027200	0.213	0.513	0.185	0.193

Tan	18	16232701	16233000	0.697	2.118	0.539	0.736
Tan	18	16263901	16264200	0.132	0.493	0.263	0.137
Tan	18	16264801	16265100	1.116	1.352	0.983	0.669
Tan	18	16691401	16691700	0.828	1.727	0.943	1.070
Tan	18	16920901	16921200	0.190	0.678	0.140	0.112
Tan	18	16921201	16921500	0.136	0.423	0.107	0.031
Tan	18	16990801	16991100	0.294	0.914	0.408	0.340
Tan	18	17224801	17225100	0.265	0.772	0.555	0.335
Tan	18	17975101	17975400	1.465	2.083	1.415	1.404
Tan	18	18199201	18199500	0.639	0.792	0.584	0.329
Tan	18	18240301	18240600	0.215	0.562	0.243	0.309
Tan	18	18327301	18327600	2.102	3.686	1.587	1.983
Tan	18	18453301	18453600	0.853	1.268	1.645	0.659
Tan	18	18483901	18484200	0.747	1.923	1.324	1.161
Tan	18	18495001	18495300	1.674	0.888	1.398	1.161
Tan	18	18501301	18501600	0.211	0.584	0.341	0.274
Tan	18	18672301	18672600	2.286	1.961	2.390	2.241
Tan	18	18817801	18818100	1.602	2.433	1.551	1.617
Tan	18	18877801	18878100	0.052	1.098	0.174	0.025
Tan	18	18878101	18878400	0.079	1.749	0.289	0.376
Tan	18	18882601	18882900	0.055	0.418	0.000	0.137
Tan	18	18944401	18944700	2.436	5.177	2.363	3.195
Tan	18	18950401	18950700	1.961	0.834	1.913	2.383
Tan	18	18952501	18952800	0.213	0.701	0.234	0.234
Tan	18	18954901	18955200	0.161	0.706	0.239	0.223
Tan	18	19129501	19129800	0.189	0.953	0.288	0.142
Tan	18	19298701	19299000	0.106	0.536	0.210	0.223
Tan	18	19324201	19324500	0.266	0.513	0.399	0.167
Tan	18	19611601	19611900	0.160	0.489	0.127	0.193
Tan	18	19614301	19614600	2.478	3.581	1.300	2.291
Tan	18	19707001	19707300	0.532	0.352	0.584	0.603
Tan	18	19738501	19738800	3.223	2.525	3.090	3.285
Tan	18	19860001	19860300	0.026	0.539	0.263	0.198
Tan	18	19974301	19974600	4.630	2.656	3.964	3.984
Tan	18	20045101	20045400	0.344	0.820	0.403	0.497
Tan	18	20236501	20236800	0.265	0.822	0.428	0.502
Tan	18	20641201	20641500	1.829	2.764	1.650	1.587
Tan	18	20925901	20926200	0.453	0.772	0.613	0.553
Tan	18	20943001	20943300	0.318	0.444	0.132	0.086
Tan	18	20987701	20988000	0.844	1.951	0.733	0.684
Tan	18	21002101	21002400	0.106	0.491	0.234	0.329
Tan	18	21215101	21215400	0.108	0.446	0.214	0.137
Tan	18	21222301	21222600	4.833	5.655	3.841	4.558
Tan	18	21378901	21379200	0.932	1.167	1.596	1.516
Tan	18	21406801	21407100	0.744	0.307	0.584	0.695
Tan	18	21484501	21484800	0.132	0.466	0.049	0.051
Tan	18	21519301	21519600	0.881	1.307	0.827	0.502
Tan	18	21619201	21619500	0.398	0.165	0.403	0.527
Tan	18	21709201	21709500	0.644	1.330	0.453	0.700
Tan	18	21725701	21726000	0.423	1.599	0.740	0.812
Tan	18	21755701	21756000	3.297	2.667	4.196	4.311
Tan	18	22143901	22144200	0.606	1.405	0.658	0.518
Tan	18	22176601	22176900	1.916	1.592	1.308	1.024
Tan	18	22194001	22194300	3.773	3.459	4.492	5.004
Tan	18	22294501	22294800	0.505	0.938	0.531	0.461
Tan	18	22485901	22486200	0.266	0.633	0.239	0.299
Tan	18	22934401	22934700	0.689	1.150	0.667	0.583
Tan	18	23316601	23316900	0.503	0.305	0.774	0.583
Tan	18	23781601	23781900	0.294	0.584	0.267	0.254
Tan	18	24126301	24126600	0.213	0.444	0.103	0.193
Tan	18	24324001	24324300	1.120	1.847	1.175	0.796
Tan	18	24901501	24901800	0.290	0.657	0.321	0.268
Tan	18	25005901	25006200	0.663	0.935	0.769	0.416
Tan	18	25122001	25122300	0.477	0.375	0.214	0.193
Tan	18	25218901	25219200	0.186	0.444	0.132	0.051
Tan	18	25233301	25233600	0.477	0.912	0.638	0.340
Tan	18	25302301	25302600	1.515	2.131	1.066	1.319

Tan	18	25317001	25317300	0.905	1.337	0.843	0.659
Tan	18	25487701	25488000	0.477	0.845	0.424	0.578
Tan	18	25559701	25560000	1.807	2.403	1.320	1.627
Tan	18	26020201	26020500	0.397	0.702	0.370	0.436
Tan	18	26034901	26035200	0.294	0.843	0.361	0.360
Tan	18	26160601	26160900	0.503	1.317	0.477	0.649
Tan	18	26160901	26161200	0.397	1.315	0.481	0.624
Tan	18	26396101	26396400	0.850	1.075	0.798	0.943
Tan	18	26475901	26476200	0.477	0.609	0.317	0.472
Tan	18	26642401	26642700	1.589	0.489	1.415	1.105
Tan	18	26700301	26700600	2.531	3.366	2.280	2.449
Tan	18	26799901	26800200	0.237	1.180	0.194	0.284
Tan	18	27000301	27000600	0.136	0.586	0.214	0.198
Tan	18	27000601	27000900	0.215	0.657	0.370	0.218
Tan	18	27012301	27012600	0.134	0.470	0.103	0.187
Tan	18	27119401	27119700	0.319	0.515	0.272	0.137
Tan	18	27137401	27137700	2.607	0.741	2.173	2.252
Tan	18	27297901	27298200	0.211	0.468	0.156	0.193
Tan	18	27456301	27456600	3.723	4.536	3.591	3.761
Tan	18	27606901	27607200	0.266	0.446	0.156	0.274
Tan	18	27765901	27766200	0.452	0.586	0.321	0.248
Tan	18	28215601	28215900	0.532	0.350	0.185	0.441
Tan	18	28691701	28692000	0.132	0.609	0.325	0.299
Tan	18	29196001	29196300	0.186	0.395	0.107	0.142
Tan	18	29271001	29271300	0.132	0.420	0.210	0.162
Tan	18	29429101	29429400	0.318	0.588	0.267	0.193
Tan	18	29620201	29620500	1.329	1.472	0.876	1.024
Tan	18	29861101	29861400	0.768	1.597	0.653	0.877
Tan	18	30210901	30211200	0.344	0.918	0.263	0.309
Tan	18	30652201	30652500	0.347	0.513	0.292	0.218
Tan	18	30794101	30794400	0.186	0.305	0.560	0.578
Tan	18	30978901	30979200	0.558	0.680	0.346	0.472
Tan	18	31083901	31084200	0.932	1.408	0.687	0.553
Tan	18	31086601	31086900	0.507	0.818	0.555	0.304
Tan	18	31255201	31255500	0.398	0.652	0.350	0.355
Tan	18	31256401	31256700	0.134	0.727	0.317	0.193
Tan	18	31852201	31852500	1.011	0.841	0.954	0.720
Tan	18	31976101	31976400	0.342	1.053	0.584	0.725
Tan	18	32088601	32088900	1.326	0.395	1.173	0.913
Tan	18	32151301	32151600	0.347	0.491	0.267	0.360
Tan	18	32186101	32186400	0.108	0.517	0.239	0.243
Tan	18	32318101	32318400	0.266	0.420	0.267	0.198
Tan	18	32354401	32354700	0.266	0.511	0.136	0.304
Tan	18	32384101	32384400	1.594	2.600	1.427	1.724
Tan	18	32390101	32390400	0.532	0.161	0.724	0.644
Tan	18	32414101	32414400	0.373	0.656	0.156	0.218
Tan	18	32414401	32414700	0.213	0.952	0.218	0.173
Tan	18	32608801	32609100	0.692	1.287	0.731	0.563
Tan	18	32727001	32727300	0.052	0.446	0.074	0.132
Tan	18	32979001	32979300	0.211	0.654	0.424	0.380
Tan	18	33011101	33011400	0.211	0.444	0.185	0.223
Tan	18	33122701	33123000	0.373	0.916	0.428	0.411
Tan	18	33185101	33185400	0.265	0.612	0.189	0.112
Tan	18	33295201	33295500	0.132	0.586	0.152	0.117
Tan	18	33374101	33374400	0.424	0.959	0.379	0.385
Tan	18	33603301	33603600	0.134	0.611	0.132	0.137
Tan	18	33686701	33687000	0.319	0.775	0.383	1.247
Tan	18	33789001	33789300	0.265	0.704	0.267	0.315
Tan	18	34327501	34327800	0.740	1.644	0.000	0.934
Tan	18	34327801	34328100	0.718	0.959	0.132	0.614
Tan	18	34644601	34644900	0.902	2.060	1.674	1.202
Tan	18	35552701	35553000	0.213	0.493	0.181	0.223
Tan	18	37889401	37889700	0.586	0.139	0.321	0.385
Tan	18	38188201	38188500	0.292	0.468	0.267	0.223
Tan	18	38616001	38616300	0.265	0.654	0.239	0.441
Tan	18	38691601	38691900	0.290	0.491	0.082	0.304
Tan	18	39098401	39098700	0.211	0.513	0.210	0.081

Tan	18	39210601	39210900	1.092	1.429	1.797	0.832
Tan	18	40244101	40244400	1.671	3.324	1.950	2.241
Tan	18	40292701	40293000	0.426	0.629	0.346	0.279
Tan	18	40610701	40611000	1.589	2.959	1.436	1.363
Tan	18	41170801	41171100	0.665	0.185	1.645	1.152
Tan	18	41195701	41196000	0.321	0.749	0.132	0.193
Tan	18	41261101	41261400	0.292	0.583	0.292	0.355
Tan	18	41788801	41789100	2.049	0.828	1.991	2.109
Tan	18	42334201	42334500	0.211	0.539	0.160	0.167
Tan	18	42693601	42693900	0.187	0.541	0.156	0.193
Tan	18	42698701	42699000	0.666	1.219	0.593	0.639
Tan	18	42954601	42954900	2.581	2.013	2.970	2.803
Tan	18	43173601	43173900	0.319	0.515	0.263	0.223
Tan	18	43180501	43180800	0.186	0.678	0.263	0.157
Tan	18	43189801	43190100	0.292	0.843	0.506	0.472
Tan	18	43210201	43210500	0.292	0.586	0.374	0.218
Tan	18	43896301	43896600	0.266	0.489	0.185	0.299
Tan	18	44144701	44145000	0.187	0.395	0.078	0.162
Tan	18	44468401	44468700	0.186	0.468	0.136	0.167
Tan	18	44473501	44473800	0.560	0.863	0.477	0.639
Tan	18	44473801	44474100	0.561	1.008	0.453	0.664
Tan	18	44489701	44490000	1.694	0.813	2.192	2.261
Tan	18	44490001	44490300	1.174	0.347	1.735	1.978
Tan	18	44657101	44657400	0.344	0.953	0.346	0.279
Tan	18	45338101	45338400	0.186	0.590	0.189	0.193
Tan	18	45868501	45868800	1.120	1.901	0.967	1.206
Tan	18	45868801	45869100	2.413	4.223	2.640	2.733
Tan	18	47203201	47203500	0.161	0.493	0.103	0.117
Tan	18	47677201	47677500	0.292	0.420	0.078	0.193
Tan	18	48364801	48365100	0.532	1.075	0.604	0.573
Tan	18	48403501	48403800	0.319	0.773	0.267	0.396
Tan	18	49190401	49190700	1.105	0.092	2.263	1.105
Tan	18	49376401	49376700	0.318	0.583	0.370	0.254
Tan	18	50265601	50265900	0.108	0.442	0.214	0.112
Tan	18	51029401	51029700	0.319	0.539	0.662	0.304
Tan	18	51474301	51474600	0.321	0.678	0.395	0.324
Tan	18	52041001	52041300	0.161	0.489	0.103	0.117
Tan	18	52041301	52041600	0.161	0.466	0.054	0.193
Tan	18	52065901	52066200	0.081	0.371	0.082	0.106
Tan	18	52066201	52066500	0.213	0.966	0.374	0.254
Tan	18	53133301	53133600	0.532	0.936	0.461	0.507
Tan	18	53197801	53198100	0.774	1.897	0.749	0.878
Tan	18	53694301	53694600	0.106	0.448	0.107	0.198
Tan	18	54100501	54100800	2.446	3.107	2.102	2.657
Tan	18	54388201	54388500	0.479	1.197	0.724	0.421
Tan	18	54469201	54469500	0.239	0.538	0.239	0.324
Tan	18	54621001	54621300	0.347	0.978	0.403	0.365
Tan	18	54662101	54662400	0.186	0.420	0.127	0.193
Tan	18	54700801	54701100	0.477	0.772	0.667	0.441
Tan	18	55019401	55019700	0.371	1.408	0.506	0.482
Tan	18	55040101	55040400	0.215	1.118	0.314	0.324
Tan	18	55095901	55096200	4.678	7.533	4.496	4.720
Tan	18	55096201	55096500	3.183	4.433	2.173	2.718
Tan	18	55104301	55104600	0.269	0.725	0.259	0.279
Tan	18	55222201	55222500	1.487	1.006	0.457	0.486
Tan	18	55321501	55321800	0.215	0.491	0.239	0.187
Tan	18	55501201	55501500	0.398	0.654	0.424	0.745
Tan	18	55682701	55683000	0.477	1.124	0.522	0.644
Tan	18	55927801	55928100	0.745	0.045	0.539	0.619
Tan	18	55929601	55929900	0.242	0.701	0.272	0.274
Tan	18	56286001	56286300	0.211	0.633	0.058	0.268
Tan	18	56508901	56509200	0.663	1.193	0.765	0.938
Tan	18	56667901	56668200	1.358	2.231	1.028	1.420
Tan	18	56831101	56831400	0.745	1.530	0.720	0.573
Tan	18	56919301	56919600	1.681	2.459	1.287	1.552
Tan	18	57318001	57318300	0.158	0.983	0.366	0.228
Tan	18	57318301	57318600	0.319	1.712	0.604	0.507

Tan	18	57442801	57443100	0.052	0.446	0.160	0.092
Tan	18	57446101	57446400	5.893	4.570	5.874	6.718
Tan	18	57501601	57501900	0.369	1.582	0.325	0.411
Tan	18	57502501	57502800	0.553	1.380	0.441	0.279
Tan	18	57713701	57714000	0.053	0.493	0.058	0.137
Tan	18	58000801	58001100	0.160	0.564	0.214	0.385
Tan	18	58062601	58062900	0.931	0.418	0.638	0.639
Tan	18	58087501	58087800	0.187	0.612	0.136	0.061
Tan	18	58095901	58096200	0.479	0.725	0.473	0.421
Tan	18	58135201	58135500	0.079	0.560	0.156	0.355
Tan	18	58287301	58287600	0.187	0.444	0.292	0.279
Tan	18	58297201	58297500	1.595	1.053	1.295	1.404
Tan	18	58319701	58320000	0.507	0.845	0.506	0.324
Tan	18	58452901	58453200	0.079	0.588	0.189	0.167
Tan	18	58453201	58453500	0.105	0.680	0.243	0.187
Tan	18	58454101	58454400	0.345	0.846	0.247	0.360
Tan	18	58463101	58463400	3.247	2.266	3.282	2.920
Tan	18	58768801	58769100	0.639	0.843	0.560	0.745
Tan	18	58786801	58787100	0.503	0.957	0.493	0.385
Tan	18	58906201	58906500	0.292	0.745	0.214	0.380
Tan	18	58954201	58954500	1.013	1.562	0.860	0.771
Tan	18	59008801	59009100	0.237	0.940	0.386	0.385
Tan	18	59015101	59015400	0.878	1.354	0.881	0.907
Tan	18	59042401	59042700	3.489	6.226	3.966	3.362
Tan	18	59085001	59085300	0.187	0.701	0.239	0.329
Tan	18	59087701	59088000	0.239	0.562	0.321	0.335
Tan	18	59103001	59103300	0.215	0.541	0.029	0.025
Tan	18	59134801	59135100	0.239	0.702	0.239	0.279
Tan	18	59141401	59141700	0.265	0.421	0.185	0.578
Tan	18	59286601	59286900	0.400	0.633	0.296	0.329
Tan	18	59313301	59313600	0.213	0.446	0.210	0.086
Tan	18	59338501	59338800	0.319	0.444	0.136	0.248
Tan	18	59404801	59405100	1.140	2.808	1.375	1.284
Tan	18	59496301	59496600	0.986	0.539	0.160	0.132
Tan	18	59596201	59596500	0.160	0.446	0.263	0.193
Tan	18	59643301	59643600	0.934	1.751	0.970	1.120
Tan	18	59643601	59643900	0.745	1.266	0.740	0.486
Tan	18	59906701	59907000	0.158	0.539	0.218	0.193
Tan	18	59943901	59944200	0.132	0.446	0.140	0.106
Tan	18	60427201	60427500	1.673	2.223	1.489	1.906
Tan	18	60467101	60467400	0.079	0.446	0.317	0.193
Tan	18	60622501	60622800	0.529	1.197	0.745	0.472
Tan	18	60622801	60623100	0.266	0.446	0.210	0.198
Tan	18	60639901	60640200	0.134	0.538	0.234	0.203
Tan	18	60960001	60960300	0.079	0.472	0.054	0.132
Tan	18	60976501	60976800	0.266	0.609	0.247	0.299
Tan	18	61322401	61322700	0.161	0.491	0.194	0.162
Tan	18	61393201	61393500	0.345	0.818	0.555	0.664
Tan	18	61573201	61573500	0.136	0.493	0.082	0.117
Tan	18	61621201	61621500	0.345	0.751	0.453	0.416
Tan	18	61778101	61778400	0.852	1.918	0.753	1.197
Tan	18	61784701	61785000	0.795	1.287	0.765	0.380
Tan	18	61957501	61957800	0.553	1.408	0.481	0.193
Tan	18	62037301	62037600	0.131	0.586	0.127	0.228
Tan	18	62122201	62122500	0.479	1.193	0.473	0.385
Tan	18	62162701	62163000	0.558	1.150	0.633	0.634
Tan	18	62239201	62239500	0.213	0.654	0.234	0.441
Tan	18	62335201	62335500	0.771	0.936	0.424	0.776
Tan	18	62747401	62747700	1.297	1.895	1.222	1.227
Tan	18	62839501	62839800	1.140	1.663	1.090	1.217
Tan	18	62842801	62843100	0.268	0.515	0.160	0.142
Tan	18	62843101	62843400	0.210	0.729	0.292	0.284
Tan	18	63085201	63085500	0.297	1.144	0.210	0.173
Tan	18	63101101	63101400	4.043	6.477	3.417	3.929
Tan	18	63118501	63118800	1.961	2.942	1.750	2.043
Tan	18	63194401	63194700	2.174	1.315	2.073	2.769
Tan	18	63459301	63459600	0.160	0.442	0.239	0.056

Tan	18	63478801	63479100	1.487	1.751	1.387	1.298
Tan	18	63547201	63547500	0.582	1.054	0.515	0.502
Tan	18	63561001	63561300	0.960	0.238	0.876	0.893
Tan	18	63561301	63561600	2.131	0.444	1.962	1.754
Tan	18	63562801	63563100	0.318	1.131	0.288	0.254
Tan	18	63563101	63563400	0.186	0.704	0.354	0.167
Tan	18	63696301	63696600	0.773	0.307	0.720	0.740
Tan	18	63701401	63701700	4.844	2.214	5.299	6.174
Tan	18	63701701	63702000	1.545	0.710	2.008	2.115
Tan	18	63742801	63743100	0.718	0.421	0.749	0.893
Tan	18	63850201	63850500	0.215	0.517	0.214	0.167
Tan	18	63994201	63994500	1.597	2.122	1.230	1.633
Tan	18	64029001	64029300	0.452	0.942	0.477	0.446
Tan	18	64095901	64096200	1.011	1.731	1.005	1.019
Tan	18	64391101	64391400	3.999	2.439	5.573	5.025
Tan	18	64644901	64645200	2.688	3.133	2.189	2.277
Tan	18	64768501	64768800	1.039	1.405	0.617	0.614
Tan	18	64790401	64790700	2.691	0.968	2.615	2.713
Tan	18	64813801	64814100	2.691	3.768	2.686	2.810
Tan	18	64901401	64901700	0.371	0.815	0.395	0.411
Tan	18	64953901	64954200	0.855	1.264	0.749	0.857
Tan	18	65046001	65046300	0.616	1.051	0.609	0.497
Tan	18	65055301	65055600	0.374	0.657	0.321	0.355
Tan	18	65163601	65163900	0.531	0.187	0.428	0.416
Tan	18	65169001	65169300	2.420	1.689	2.570	2.504
Tan	18	65170801	65171100	2.762	1.963	2.698	2.551
Tan	18	65202901	65203200	0.321	0.631	0.234	0.228
Tan	18	65211601	65211900	0.824	0.206	0.852	0.603
Tan	18	65342101	65342400	2.667	4.955	3.159	1.845
Tan	18	65355901	65356200	0.745	2.470	1.239	0.599
Tan	18	65356201	65356500	0.371	2.051	0.510	0.254
Tan	18	65373601	65373900	0.447	1.240	0.477	0.751
Tan	18	65495701	65496000	5.550	7.606	5.245	5.908
Tan	18	65583001	65583300	4.472	6.553	3.698	4.645
Tan	18	65598601	65598900	5.104	8.541	4.588	5.506
Tan	18	65721301	65721600	0.957	0.440	1.173	1.288
Tan	18	65754601	65754900	0.694	0.935	0.691	1.136
Tan	18	65776801	65777100	0.531	0.933	0.218	0.299
Tan	18	66061801	66062100	0.266	0.659	0.263	0.243
Tan	18	66149701	66150000	0.428	0.815	0.481	0.385
Tan	18	66232201	66232500	3.747	2.212	3.344	3.818
Tan	18	66414901	66415200	0.423	0.863	0.419	0.385
Tan	18	66528301	66528600	0.924	1.476	0.239	0.639
Tan	18	66680401	66680700	0.215	0.586	0.234	0.213
Tan	18	66780301	66780600	0.505	0.187	0.267	0.497
Tan	18	66851401	66851700	0.689	0.137	0.740	0.522
Tan	18	66881401	66881700	0.079	0.584	0.152	0.142
Tan	18	66972601	66972900	3.415	1.311	4.113	3.504
Tan	18	66976801	66977100	0.823	1.077	0.531	0.664
Tan	18	67016401	67016700	0.611	1.624	0.580	0.852
Tan	18	67458901	67459200	5.215	3.590	5.085	4.882
Tan	18	67511401	67511700	0.637	0.094	0.370	0.279
Tan	18	67611901	67612200	0.958	0.354	1.094	0.872
Tan	18	67641301	67641600	0.342	1.772	0.395	0.873
Tan	18	67659001	67659300	0.239	0.466	0.218	0.142
Tan	18	67669201	67669500	1.170	2.296	0.782	1.485
Tan	18	67690501	67690800	0.239	0.536	0.288	0.299
Tan	18	67742401	67742700	3.983	5.767	2.329	3.899
Tan	18	67761901	67762200	3.193	2.086	4.068	2.850
Tan	18	67798501	67798800	2.081	0.807	1.971	2.018
Tan	18	67995001	67995300	1.250	1.828	0.613	1.207
Tan	18	68264101	68264400	0.586	1.669	0.934	0.887
Tan	18	68313301	68313600	0.397	1.227	0.448	0.396
Tan	18	68480101	68480400	1.889	4.302	1.978	2.556
Tan	18	68508901	68509200	4.276	3.267	4.175	4.680
Tan	18	68561101	68561400	0.979	1.667	1.008	0.862
Tan	19	40801	41100	2.250	4.937	2.419	4.001

Tan	19	92401	92700	0.292	0.491	0.243	0.279
Tan	19	123601	123900	0.239	0.633	0.103	0.355
Tan	19	198301	198600	0.453	0.656	0.243	0.446
Tan	19	302401	302700	0.555	1.446	0.468	0.796
Tan	19	384001	384300	0.321	0.891	0.263	0.553
Tan	19	418801	419100	0.237	0.723	0.395	0.243
Tan	19	823201	823500	0.215	0.611	0.140	0.243
Tan	19	834901	835200	1.037	1.453	0.798	0.690
Tan	19	1042201	1042500	0.237	1.006	0.082	0.274
Tan	19	1485001	1485300	0.265	0.470	0.160	0.137
Tan	19	1578001	1578300	0.720	1.412	0.453	0.634
Tan	19	1578301	1578600	0.215	0.656	0.239	0.248
Tan	19	1789801	1790100	0.350	0.910	0.432	0.416
Tan	19	2103601	2103900	0.053	0.489	0.160	0.157
Tan	19	2328901	2329200	0.452	0.704	0.453	0.355
Tan	19	2473501	2473800	0.687	1.379	0.593	0.598
Tan	19	2473801	2474100	2.476	3.409	1.950	2.338
Tan	19	2764501	2764800	0.426	0.279	0.453	0.466
Tan	19	2815201	2815500	0.213	0.494	0.156	0.223
Tan	19	3285901	3286200	0.742	1.452	0.711	0.812
Tan	19	3668101	3668400	0.450	0.888	0.346	0.522
Tan	19	3668401	3668700	0.690	1.285	0.687	1.014
Tan	19	3835801	3836100	0.476	0.865	0.341	0.553
Tan	19	3838801	3839100	0.239	0.609	0.234	0.254
Tan	19	3914101	3914400	0.213	0.515	0.263	0.248
Tan	19	4192501	4192800	0.561	1.165	0.560	0.522
Tan	19	4192801	4193100	0.534	1.139	0.506	0.497
Tan	19	4266901	4267200	0.134	0.423	0.210	0.137
Tan	19	4316401	4316700	0.106	0.420	0.000	0.228
Tan	19	4695301	4695600	0.799	1.337	0.896	0.826
Tan	19	5321401	5321700	0.587	1.101	0.749	0.745
Tan	19	5342101	5342400	0.240	0.792	0.419	0.248
Tan	19	5546401	5546700	0.213	0.468	0.078	0.355
Tan	19	5657101	5657400	0.532	0.980	0.575	0.274
Tan	19	5676001	5676300	0.665	0.985	0.448	0.299
Tan	19	5689801	5690100	0.106	0.583	0.292	0.279
Tan	19	5703901	5704200	0.158	0.659	0.243	0.031
Tan	19	5745601	5745900	0.799	1.216	0.539	0.740
Tan	19	5758801	5759100	0.637	0.933	0.370	0.603
Tan	19	5876401	5876700	0.211	0.586	0.395	0.193
Tan	19	6057601	6057900	0.052	1.214	0.753	0.193
Tan	19	6059401	6059700	0.505	1.351	0.716	0.588
Tan	19	6103201	6103500	0.266	0.562	0.292	0.187
Tan	19	6115501	6115800	0.640	0.000	0.395	0.533
Tan	19	6117601	6117900	5.551	3.154	3.953	4.630
Tan	19	6134101	6134400	1.891	0.817	1.518	1.622
Tan	19	6139501	6139800	4.874	3.313	4.713	5.662
Tan	19	6141901	6142200	4.173	5.757	3.794	3.558
Tan	19	6268801	6269100	6.688	4.842	6.079	6.282
Tan	19	6306001	6306300	0.319	0.466	0.292	0.279
Tan	19	6588601	6588900	0.240	0.491	0.267	0.137
Tan	19	6780001	6780300	1.489	1.987	1.480	1.206
Tan	19	6904501	6904800	0.373	0.562	0.399	0.472
Tan	19	7039801	7040100	5.317	6.241	5.187	6.851
Tan	19	7561501	7561800	1.086	1.970	0.778	0.805
Tan	19	7623301	7623600	0.158	0.612	0.210	0.051
Tan	19	7811401	7811700	3.957	4.834	2.920	3.984
Tan	19	7867201	7867500	0.403	1.457	0.542	0.295
Tan	19	7867501	7867800	0.373	1.268	0.214	0.356
Tan	19	8145901	8146200	1.626	2.909	1.855	1.632
Tan	19	8199601	8199900	0.294	0.491	0.181	0.137
Tan	19	8577001	8577300	0.213	0.444	0.107	0.173
Tan	19	8642101	8642400	3.500	1.073	2.602	2.600
Tan	19	8837101	8837400	0.160	0.493	0.136	0.193
Tan	19	8901901	8902200	3.504	1.077	3.431	2.871
Tan	19	8909401	8909700	0.419	1.363	0.357	0.401
Tan	19	9595501	9595800	0.192	0.912	0.210	0.112

Tan	19	9657601	9657900	1.065	2.319	1.453	1.277
Tan	19	9679201	9679500	0.557	0.867	0.399	0.436
Tan	19	10252501	10252800	0.500	1.549	0.361	0.421
Tan	19	10252801	10253100	0.448	1.388	0.361	0.288
Tan	19	10365001	10365300	0.263	0.865	0.176	0.421
Tan	19	10390801	10391100	1.765	2.959	1.333	1.663
Tan	19	10419901	10420200	0.744	1.410	0.481	0.776
Tan	19	10420201	10420500	0.215	0.921	0.259	0.193
Tan	19	10473001	10473300	0.531	0.350	0.103	0.061
Tan	19	10530001	10530300	0.187	0.541	0.103	0.061
Tan	19	10604401	10604700	0.527	1.613	0.390	0.527
Tan	19	11530501	11530800	0.768	1.691	0.760	0.756
Tan	19	11541301	11541600	0.345	0.867	0.473	0.248
Tan	19	11570701	11571000	0.131	0.470	0.111	0.137
Tan	19	11655901	11656200	0.874	1.573	0.774	0.862
Tan	19	11732701	11733000	0.582	1.034	0.481	0.492
Tan	19	11792701	11793000	0.452	0.723	0.502	0.391
Tan	19	11878201	11878500	1.120	2.068	0.976	1.055
Tan	19	11883001	11883300	0.989	1.704	0.954	1.055
Tan	19	11997001	11997300	3.083	1.720	3.039	2.779
Tan	19	12047101	12047400	6.033	4.864	8.037	7.184
Tan	19	12336001	12336300	0.077	0.446	0.132	0.193
Tan	19	12634801	12635100	1.808	0.863	1.585	1.946
Tan	19	13012501	13012800	0.586	0.281	0.453	0.664
Tan	19	13114801	13115100	0.720	1.410	0.662	0.669
Tan	19	13198801	13199100	0.266	0.605	0.210	0.193
Tan	19	13335001	13335300	2.284	1.051	1.634	1.998
Tan	19	13392901	13393200	1.113	1.856	0.789	0.776
Tan	19	13442701	13443000	0.452	0.631	0.321	0.441
Tan	19	13620601	13620900	1.597	2.613	1.316	1.912
Tan	19	13646401	13646700	0.878	0.678	0.584	0.776
Tan	19	13677901	13678200	0.345	0.680	0.321	0.279
Tan	19	13819801	13820100	1.091	2.074	1.349	1.262
Tan	19	13822501	13822800	0.481	0.935	0.399	0.370
Tan	19	13877101	13877400	0.747	1.262	0.395	0.513
Tan	19	13953001	13953300	0.242	0.768	0.132	0.340
Tan	19	14180401	14180700	0.184	0.493	0.185	0.076
Tan	19	14252101	14252400	0.740	1.753	1.320	1.029
Tan	19	14442601	14442900	0.290	0.633	0.259	0.304
Tan	19	14841001	14841300	0.344	0.678	0.346	0.132
Tan	19	15078601	15078900	0.290	0.564	0.214	0.137
Tan	19	15222601	15222900	0.052	1.000	0.000	0.122
Tan	19	15256501	15256800	2.260	0.770	1.465	1.439
Tan	19	15326401	15326700	4.038	2.959	3.859	4.735
Tan	19	15656701	15657000	1.037	0.560	0.852	0.715
Tan	19	16414801	16415100	0.132	0.468	0.239	0.106
Tan	19	16421701	16422000	0.105	0.395	0.107	0.162
Tan	19	16551301	16551600	2.204	2.873	1.045	1.860
Tan	19	16871701	16872000	0.611	1.225	0.477	0.380
Tan	19	16960201	16960500	1.376	3.023	1.164	1.236
Tan	19	16960801	16961100	1.575	4.624	1.606	1.783
Tan	19	17005501	17005800	1.384	2.560	1.507	1.197
Tan	19	17349301	17349600	0.186	0.468	0.152	0.142
Tan	19	17373901	17374200	0.211	0.517	0.263	0.193
Tan	19	17464801	17465100	1.252	1.847	1.407	1.339
Tan	19	17778601	17778900	0.428	0.727	0.296	0.304
Tan	19	17847901	17848200	0.266	0.702	0.366	0.360
Tan	19	18163501	18163800	0.319	0.815	0.276	0.299
Tan	19	18459901	18460200	0.242	0.633	0.185	0.259
Tan	19	18902401	18902700	0.237	0.682	0.160	0.101
Tan	19	19195201	19195500	0.855	2.004	0.586	0.725
Tan	19	19282501	19282800	0.321	1.158	0.214	0.223
Tan	19	19282801	19283100	0.426	1.529	0.305	0.507
Tan	19	19491601	19491900	0.132	0.491	0.107	0.304
Tan	19	19991401	19991700	0.081	0.446	0.098	0.167
Tan	19	20328901	20329200	0.398	0.841	0.415	0.309
Tan	19	21092401	21092700	0.505	1.032	0.403	0.614

Tan	19	21331501	21331800	0.026	0.466	0.181	0.167
Tan	19	21331801	21332100	0.297	0.865	0.165	0.350
Tan	19	21390901	21391200	0.455	1.266	0.538	0.436
Tan	19	21467401	21467700	0.210	0.678	0.160	0.223
Tan	19	21522001	21522300	0.240	0.446	0.136	0.193
Tan	19	21690001	21690300	3.133	4.246	3.019	3.361
Tan	19	21823801	21824100	0.481	1.030	0.408	0.396
Tan	19	22029001	22029300	0.476	1.006	0.317	0.776
Tan	19	22197601	22197900	0.450	0.891	0.609	0.583
Tan	19	22628101	22628400	2.679	1.869	2.423	2.626
Tan	19	22955701	22956000	0.319	0.444	0.321	0.142
Tan	19	23080501	23080800	0.319	0.890	0.379	0.400
Tan	19	23210101	23210400	0.161	0.843	0.078	0.137
Tan	19	23229601	23229900	0.503	1.270	0.395	0.634
Tan	19	23384701	23385000	0.108	0.893	0.457	0.329
Tan	19	24141901	24142200	0.242	0.538	0.239	0.193
Tan	19	24564901	24565200	0.294	0.727	0.210	0.385
Tan	19	25181101	25181400	0.053	0.493	0.082	0.051
Tan	19	26170801	26171100	1.223	1.729	0.838	1.156
Tan	19	26550301	26550600	0.132	0.401	0.082	0.051
Tan	19	26913601	26913900	0.160	0.511	0.029	0.162
Tan	19	27267601	27267900	0.108	0.401	0.054	0.025
Tan	19	27392701	27393000	0.560	0.981	0.370	1.166
Tan	19	28200001	28200300	0.508	0.818	0.481	0.411
Tan	19	28314001	28314300	1.357	1.541	1.016	0.360
Tan	19	28324501	28324800	0.186	0.466	0.107	0.157
Tan	19	28578901	28579200	0.586	0.442	0.317	0.801
Tan	19	28626301	28626600	0.769	1.287	0.704	0.720
Tan	19	28627501	28627800	0.239	0.489	0.239	0.248
Tan	19	28731301	28731600	0.053	0.423	0.132	0.162
Tan	19	29196901	29197200	0.161	0.704	0.399	0.335
Tan	19	29469901	29470200	0.187	0.588	0.189	0.086
Tan	19	29552701	29553000	0.213	0.539	0.165	0.248
Tan	19	29575501	29575800	0.215	0.863	0.172	0.233
Tan	19	29596201	29596500	0.106	0.631	0.132	0.284
Tan	19	29619301	29619600	1.170	1.380	1.061	0.994
Tan	19	29899801	29900100	0.931	1.639	0.551	0.897
Tan	19	30097201	30097500	0.158	0.446	0.132	0.223
Tan	19	30178201	30178500	0.186	0.448	0.078	0.081
Tan	19	30287701	30288000	0.211	0.564	0.103	0.329
Tan	19	30357601	30357900	1.570	0.985	1.605	1.465
Tan	19	30517201	30517500	4.936	3.473	4.688	5.071
Tan	19	30596701	30597000	1.881	3.128	1.119	1.961
Tan	19	30597001	30597300	0.932	1.470	0.613	0.893
Tan	19	30736801	30737100	0.026	0.468	0.132	0.117
Tan	19	31603201	31603500	0.342	0.929	0.317	0.223
Tan	19	31635601	31635900	0.132	0.757	0.140	0.056
Tan	19	31769701	31770000	0.136	0.749	0.198	0.243
Tan	19	31770001	31770300	0.371	1.169	0.421	0.299
Tan	19	31900201	31900500	0.692	0.912	0.745	0.279
Tan	19	32019601	32019900	0.903	0.444	0.695	0.988
Tan	19	32072701	32073000	0.423	1.051	0.584	0.614
Tan	19	32111701	32112000	0.319	0.584	0.292	0.335
Tan	19	32567101	32567400	0.294	0.657	0.189	0.355
Tan	19	33171301	33171600	0.426	0.609	0.165	0.335
Tan	19	33206701	33207000	0.266	0.466	0.054	0.162
Tan	19	33263101	33263400	0.213	0.493	0.132	0.051
Tan	19	33323401	33323700	0.215	0.732	0.078	0.051
Tan	19	33518101	33518400	0.158	0.800	0.054	0.081
Tan	19	33518401	33518700	0.131	0.470	0.082	0.076
Tan	19	34526401	34526700	0.237	0.517	0.082	0.248
Tan	19	34664401	34664700	0.134	0.560	0.074	0.112
Tan	19	35056501	35056800	1.516	1.708	1.119	1.359
Tan	19	35094001	35094300	0.718	1.306	0.658	0.598
Tan	19	35223001	35223300	0.426	1.225	0.325	0.396
Tan	19	35242201	35242500	0.210	0.678	0.185	0.304
Tan	19	35382001	35382300	0.981	1.285	0.633	0.553

Tan	19	35819101	35819400	0.290	0.867	0.354	0.259
Tan	19	35964601	35964900	0.158	0.659	0.103	0.243
Tan	19	36158401	36158700	1.303	1.940	1.213	1.384
Tan	19	36234301	36234600	0.561	1.172	0.774	0.608
Tan	19	36608701	36609000	0.924	1.569	0.687	0.907
Tan	19	36769501	36769800	0.319	0.863	0.584	0.380
Tan	19	36989101	36989400	0.026	0.609	0.111	0.213
Tan	19	37199101	37199400	1.328	2.109	1.395	1.044
Tan	19	37271101	37271400	0.274	1.403	0.029	0.243
Tan	19	37447801	37448100	0.186	0.584	0.366	0.304
Tan	19	37613701	37614000	0.661	1.096	0.477	0.695
Tan	19	37945201	37945500	0.266	0.751	0.267	0.497
Tan	19	38388601	38388900	0.079	0.283	0.477	0.137
Tan	19	38725801	38726100	0.457	1.032	0.437	0.223
Tan	19	38760901	38761200	0.294	0.635	0.243	0.162
Tan	19	38833201	38833500	0.426	0.584	0.317	0.248
Tan	19	39018601	39018900	0.608	1.289	0.560	0.304
Tan	19	39127501	39127800	0.558	0.397	0.453	0.360
Tan	19	39450301	39450600	0.371	0.749	0.424	0.274
Tan	19	39755401	39755700	0.318	1.030	0.346	0.420
Tan	19	39845101	39845400	0.268	0.047	0.963	0.335
Tan	19	39845401	39845700	0.266	0.047	0.720	0.279
Tan	19	40012501	40012800	0.240	0.444	0.107	0.243
Tan	19	40361401	40361700	0.477	0.633	0.160	0.274
Tan	19	40513801	40514100	0.979	1.571	1.012	0.943
Tan	19	40570801	40571100	0.239	0.493	0.111	0.112
Tan	19	40638901	40639200	1.707	2.645	1.884	1.957
Tan	19	40812001	40812300	0.560	0.023	0.288	0.248
Tan	19	40885801	40886100	2.392	1.920	2.303	1.577
Tan	19	41212201	41212500	0.318	0.633	0.234	0.324
Tan	19	41339701	41340000	0.105	0.470	0.078	0.147
Tan	19	41433301	41433600	1.173	1.946	1.284	1.410
Tan	19	41458501	41458800	1.760	1.118	2.682	3.189
Tan	19	41595901	41596200	0.187	0.775	0.259	0.274
Tan	19	41641801	41642100	0.666	1.351	0.292	0.457
Tan	19	41679001	41679300	0.558	0.210	0.428	0.533
Tan	19	41684701	41685000	1.120	1.472	1.173	0.887
Tan	19	41740201	41740500	0.771	1.028	0.502	0.801
Tan	19	41823301	41823600	0.505	1.148	0.486	0.462
Tan	19	42200701	42201000	0.771	1.197	0.633	0.664
Tan	19	42533701	42534000	0.958	1.405	0.959	0.877
Tan	19	42683701	42684000	0.186	0.654	0.296	0.304
Tan	19	42711301	42711600	0.108	0.444	0.214	0.142
Tan	19	42793201	42793500	0.423	0.891	0.457	0.497
Tan	19	42871801	42872100	4.544	2.609	4.232	5.127
Tan	19	42886501	42886800	3.208	2.356	3.344	3.645
Tan	19	43173301	43173600	0.186	0.721	0.312	0.274
Tan	19	43189201	43189500	1.195	1.826	1.206	1.226
Tan	19	43223701	43224000	1.355	1.641	0.979	1.328
Tan	19	43340101	43340400	0.186	0.611	0.156	0.360
Tan	19	43373401	43373700	0.881	1.403	0.720	0.583
Tan	19	43498201	43498500	1.381	2.609	0.524	1.526
Tan	19	43498501	43498800	1.511	3.094	0.873	1.805
Tan	19	43663501	43663800	0.239	0.609	0.218	0.193
Tan	19	44126101	44126400	0.184	0.680	0.424	0.391
Tan	19	44159401	44159700	0.134	0.517	0.103	0.223
Tan	19	44165401	44165700	0.878	0.350	1.201	0.735
Tan	19	44211901	44212200	0.426	0.583	0.189	0.360
Tan	19	44401201	44401500	0.237	0.609	0.165	0.248
Tan	19	44415601	44415900	0.402	0.845	0.424	0.466
Tan	19	44851501	44851800	4.018	2.898	5.301	4.142
Tan	19	44855701	44856000	0.744	0.350	0.526	0.522
Tan	19	45282301	45282600	0.960	2.027	1.230	1.227
Tan	19	45321301	45321600	1.836	0.657	0.223	1.091
Tan	19	45572701	45573000	1.089	0.272	1.222	1.125
Tan	19	45803401	45803700	0.266	0.862	0.156	0.147
Tan	19	45806101	45806400	0.294	0.848	0.160	0.335

Tan	19	45810601	45810900	0.240	0.446	0.156	0.112
Tan	19	45841801	45842100	1.805	0.215	1.110	0.614
Tan	19	46420801	46421100	0.026	0.420	0.000	0.056
Tan	19	46557301	46557600	0.213	0.704	0.185	0.391
Tan	19	46791001	46791300	1.862	1.279	2.011	1.871
Tan	19	46796701	46797000	0.294	0.468	0.687	0.254
Tan	19	46980901	46981200	0.134	0.423	0.127	0.106
Tan	19	47037001	47037300	0.136	0.558	0.160	0.162
Tan	19	47073901	47074200	4.202	4.885	3.961	3.752
Tan	19	47169301	47169600	0.349	0.985	0.588	0.472
Tan	19	47784601	47784900	0.106	0.938	0.172	0.132
Tan	19	47808301	47808600	0.319	0.446	0.185	0.167
Tan	19	47847001	47847300	0.824	1.500	0.441	0.842
Tan	19	47920501	47920800	0.666	0.397	0.638	0.745
Tan	19	47998501	47998800	1.166	2.152	1.217	1.263
Tan	19	48323701	48324000	2.693	1.440	3.007	2.880
Tan	19	48489001	48489300	1.197	0.555	1.119	1.384
Tan	19	48662401	48662700	0.160	0.684	0.263	0.254
Tan	19	48683101	48683400	1.195	1.614	1.251	1.278
Tan	19	48700801	48701100	0.745	1.377	0.727	0.583
Tan	19	48842701	48843000	0.508	1.221	0.555	0.466
Tan	19	48887701	48888000	0.740	1.380	0.934	0.751
Tan	19	48900301	48900600	0.395	2.120	0.366	0.477
Tan	19	48980701	48981000	0.210	0.562	0.210	0.162
Tan	19	49149301	49149600	2.470	3.598	2.595	3.083
Tan	19	49212901	49213200	0.160	0.491	0.132	0.187
Tan	19	49213201	49213500	0.265	0.517	0.239	0.248
Tan	19	49218301	49218600	0.610	1.077	0.497	0.573
Tan	19	49233001	49233300	0.160	0.446	0.346	0.304
Tan	19	49354201	49354500	0.481	0.818	0.481	0.446
Tan	19	49382401	49382700	0.426	0.586	0.185	0.385
Tan	19	49388101	49388400	2.021	1.448	1.008	0.918
Tan	19	49451101	49451400	0.455	1.171	0.341	0.502
Tan	19	49685401	49685700	0.239	0.607	0.132	0.248
Tan	19	49773901	49774200	0.081	0.564	0.160	0.132
Tan	19	49791601	49791900	0.187	0.515	0.267	0.821
Tan	19	49977301	49977600	0.349	0.704	0.288	0.223
Tan	19	50238601	50238900	0.134	0.446	0.136	0.025
Tan	19	50238901	50239200	0.079	1.077	0.132	0.025
Tan	19	50383801	50384100	2.100	1.313	1.567	1.658
Tan	19	50507701	50508000	3.270	2.053	3.023	2.646
Tan	19	50547601	50547900	0.428	0.798	0.321	0.421
Tan	19	51204301	51204600	1.457	2.057	1.090	1.424
Tan	19	51204601	51204900	2.684	4.328	3.219	5.634
Tan	19	51215101	51215400	0.982	1.358	0.823	1.111
Tan	19	51499801	51500100	0.932	1.639	0.909	1.212
Tan	19	52035601	52035900	0.106	0.515	0.317	0.441
Tan	19	52452301	52452600	0.479	0.989	0.419	0.497
Tan	19	52452601	52452900	0.215	0.513	0.160	0.142
Tan	19	52521001	52521300	0.448	1.073	0.642	0.664
Tan	19	52532701	52533000	0.079	0.446	0.292	0.254
Tan	19	52851001	52851300	1.549	2.933	1.492	1.505
Tan	19	52851301	52851600	0.237	0.541	0.160	0.218
Tan	19	52914601	52914900	0.323	0.867	0.252	0.223
Tan	19	53067301	53067600	0.319	0.468	0.370	0.137
Tan	19	53146501	53146800	1.091	0.772	0.510	0.472
Tan	19	53153101	53153400	0.532	1.054	0.593	0.684
Tan	19	53440801	53441100	8.043	5.705	7.691	7.419
Tan	19	53496601	53496900	0.000	0.444	0.107	0.162
Tan	19	53515801	53516100	0.984	2.111	0.697	0.700
Tan	19	53517301	53517600	0.053	0.586	0.054	0.213
Tan	19	53535301	53535600	0.269	0.775	0.263	0.259
Tan	19	53617501	53617800	2.100	1.706	1.494	0.974
Tan	19	53934901	53935200	0.805	1.925	0.711	1.055
Tan	19	54006001	54006300	0.611	0.867	0.317	0.309
Tan	19	54297601	54297900	0.290	1.523	0.707	0.720
Tan	19	54327001	54327300	3.509	2.208	3.513	3.540

Tan	19	54379801	54380100	0.211	0.513	0.239	0.329
Tan	19	54396001	54396300	0.344	0.701	0.272	0.162
Tan	19	54408901	54409200	5.238	3.611	4.418	5.126
Tan	19	54420601	54420900	0.878	0.515	0.745	0.329
Tan	19	54525301	54525600	1.061	2.381	1.219	1.222
Tan	19	54547501	54547800	2.204	1.588	2.588	2.504
Tan	19	54606601	54606900	0.186	0.914	0.325	0.203
Tan	19	54694501	54694800	1.460	0.811	1.333	1.825
Tan	19	54740701	54741000	3.964	3.364	4.648	2.961
Tan	19	54762601	54762900	0.424	0.723	0.535	0.112
Tan	19	54780901	54781200	0.505	0.794	0.399	0.547
Tan	19	54912901	54913200	0.240	0.444	0.132	0.218
Tan	19	54914401	54914700	0.474	2.062	0.972	0.761
Tan	19	54914701	54915000	0.393	1.547	0.379	0.792
Tan	19	54945601	54945900	0.505	0.959	0.419	0.248
Tan	19	54992401	54992700	0.349	1.025	0.428	0.578
Tan	19	55031101	55031400	1.215	2.191	0.941	0.533
Tan	19	55152901	55153200	1.303	0.961	1.226	1.770
Tan	19	55156801	55157100	1.650	2.102	1.074	1.536
Tan	19	55164901	55165200	2.786	3.748	2.613	2.966
Tan	19	55247101	55247400	0.637	1.075	0.778	0.659
Tan	19	55277401	55277700	0.026	1.427	0.716	0.415
Tan	19	55429501	55429800	3.381	2.289	1.855	3.149
Tan	19	55512901	55513200	5.775	3.622	5.872	5.977
Tan	19	55954501	55954800	0.184	0.466	0.214	0.167
Tan	19	56140201	56140500	0.534	0.916	0.098	0.497
Tan	19	56198401	56198700	0.531	1.313	0.588	0.624
Tan	19	56458201	56458500	1.460	2.545	1.298	1.202
Tan	19	56459401	56459700	0.718	2.109	1.048	1.121
Tan	19	56629201	56629500	0.287	1.365	0.510	0.380
Tan	19	56745901	56746200	5.614	3.414	5.052	5.506
Tan	19	56772901	56773200	0.160	0.611	0.082	0.142
Tan	19	56773201	56773500	0.507	1.270	0.288	0.173
Tan	19	57093601	57093900	2.075	2.815	1.509	1.541
Tan	19	57213301	57213600	0.558	1.070	1.686	0.553
Tan	19	57252901	57253200	1.910	2.635	1.353	1.501
Tan	19	57346201	57346500	0.774	1.811	0.789	0.664
Tan	19	57521401	57521700	1.568	0.813	2.082	1.906
Tan	19	57593101	57593400	0.369	1.197	0.181	0.720
Tan	19	57593401	57593700	0.426	1.008	0.301	0.628
Tan	19	57594601	57594900	1.326	2.534	1.663	1.643
Tan	19	57635401	57635700	0.479	0.094	0.346	0.558
Tan	19	57656101	57656400	1.437	1.852	1.440	1.004
Tan	19	57754201	57754500	1.547	2.718	1.050	1.886
Tan	19	57852301	57852600	1.913	2.764	1.719	2.231
Tan	19	57990901	57991200	2.081	0.622	2.064	1.739
Tan	19	58051201	58051500	1.513	0.773	1.527	1.343
Tan	19	58135201	58135500	2.420	2.010	2.225	2.850
Tan	19	58162801	58163100	2.104	1.491	3.233	2.286
Tan	19	58194601	58194900	0.958	1.777	2.303	0.978
Tan	19	58232701	58233000	0.982	0.182	0.782	0.695
Tan	19	58430701	58431000	5.772	4.362	6.291	5.217
Tan	19	58560901	58561200	4.549	3.431	4.778	4.650
Tan	19	59063401	59063700	0.728	1.950	0.709	0.517
Tan	19	59082901	59083200	7.836	5.818	7.696	7.651
Tan	19	59294401	59294700	1.042	2.946	4.200	4.431
Tan	19	59412301	59412600	1.602	2.763	1.728	1.476
Tan	19	59457901	59458200	0.881	2.266	0.653	0.654
Tan	19	59475901	59476200	0.550	1.356	0.444	0.477
Tan	19	59479201	59479500	3.025	0.635	2.798	2.964
Tan	19	59483101	59483400	0.160	0.448	0.132	0.167
Tan	19	59492401	59492700	2.853	5.203	2.458	2.509
Tan	19	59571601	59571900	2.305	1.337	2.307	3.077
Tan	19	59694001	59694300	4.340	6.366	5.511	5.126
Tan	19	59778001	59778300	1.382	1.077	0.629	1.379
Tan	19	59819701	59820000	2.031	3.435	2.242	1.886
Tan	19	60034801	60035100	1.971	0.600	1.694	1.425

Tan	19	60038701	60039000	6.244	3.572	5.938	5.568
Tan	2	54001	54300	0.344	0.800	0.136	0.248
Tan	2	68101	68400	0.532	0.841	0.638	0.715
Tan	2	146401	146700	2.584	1.326	2.049	1.871
Tan	2	165301	165600	0.500	1.026	0.481	0.452
Tan	2	190501	190800	0.960	0.420	0.778	0.857
Tan	2	236701	237000	0.455	0.959	0.633	0.391
Tan	2	244801	245100	4.226	1.644	4.160	3.767
Tan	2	300001	300300	0.453	0.680	0.350	0.198
Tan	2	564901	565200	9.711	11.161	8.946	12.970
Tan	2	939901	940200	0.240	0.468	0.185	0.112
Tan	2	969301	969600	6.382	4.922	5.907	6.398
Tan	2	1179001	1179300	3.244	2.098	2.798	3.681
Tan	2	1288801	1289100	3.113	4.143	2.878	3.088
Tan	2	1329901	1330200	1.487	2.396	1.037	1.201
Tan	2	1352401	1352700	0.507	1.079	0.609	0.644
Tan	2	1440001	1440300	0.720	1.450	0.691	0.679
Tan	2	1440301	1440600	0.448	1.026	0.667	0.639
Tan	2	1488901	1489200	6.383	8.252	6.041	6.951
Tan	2	1671901	1672200	4.951	3.032	3.763	4.771
Tan	2	1724401	1724700	2.995	1.491	2.756	2.616
Tan	2	1791901	1792200	0.210	0.631	0.123	0.218
Tan	2	1819801	1820100	0.000	0.738	0.025	0.000
Tan	2	2047201	2047500	0.479	1.079	0.629	0.360
Tan	2	2235901	2236200	0.211	0.517	0.189	0.243
Tan	2	2265901	2266200	0.079	0.517	0.218	0.167
Tan	2	2298901	2299200	2.050	2.811	1.864	1.957
Tan	2	2634601	2634900	2.176	1.424	2.122	2.261
Tan	2	2667601	2667900	1.144	0.869	0.473	0.441
Tan	2	2699701	2700000	0.237	0.846	0.267	0.243
Tan	2	2705701	2706000	0.529	0.957	0.535	0.486
Tan	2	2917501	2917800	5.827	4.105	5.822	5.988
Tan	2	2965501	2965800	0.213	0.466	0.136	0.248
Tan	2	2978701	2979000	0.819	1.749	0.847	0.801
Tan	2	3161401	3161700	6.256	5.207	6.570	6.470
Tan	2	3196201	3196500	3.786	1.167	2.896	3.014
Tan	2	3257401	3257700	3.519	1.405	3.825	3.747
Tan	2	3259201	3259500	2.712	1.289	2.349	2.007
Tan	2	3384001	3384300	0.879	0.562	0.267	0.416
Tan	2	3391501	3391800	0.240	0.723	0.049	0.167
Tan	2	3561601	3561900	0.878	2.193	0.769	0.898
Tan	2	3970801	3971100	2.070	3.709	1.641	0.755
Tan	2	4021201	4021500	0.292	0.538	0.317	0.223
Tan	2	4171801	4172100	0.240	0.465	0.239	0.137
Tan	2	4744501	4744800	0.534	0.118	0.399	0.360
Tan	2	4985401	4985700	0.052	0.442	0.185	0.193
Tan	2	5003401	5003700	0.586	0.796	0.214	0.243
Tan	2	5208601	5208900	0.134	0.708	0.267	0.218
Tan	2	5712901	5713200	0.161	0.515	0.181	0.274
Tan	2	5915101	5915400	0.239	0.893	0.350	0.411
Tan	2	5925001	5925300	0.558	0.867	0.642	0.639
Tan	2	6074101	6074400	0.292	0.515	0.296	0.167
Tan	2	6111301	6111600	0.263	0.538	0.185	0.162
Tan	2	6195601	6195900	0.666	1.242	0.702	0.466
Tan	2	6349201	6349500	0.055	0.674	0.243	0.284
Tan	2	6469201	6469500	1.862	1.214	2.036	1.886
Tan	2	6571201	6571500	1.357	2.244	1.465	1.146
Tan	2	6606901	6607200	0.211	0.421	0.560	0.112
Tan	2	7036501	7036800	4.554	2.808	3.435	3.726
Tan	2	7070701	7071000	0.186	0.564	0.296	0.106
Tan	2	7197901	7198200	1.812	2.716	1.061	2.083
Tan	2	7299301	7299600	1.278	0.721	1.259	1.699
Tan	2	7359901	7360200	0.847	1.729	1.041	0.963
Tan	2	7446301	7446600	0.558	0.049	0.243	0.279
Tan	2	7447201	7447500	0.826	2.393	1.023	1.455
Tan	2	7517101	7517400	0.158	0.534	0.210	0.198
Tan	2	8282701	8283000	0.321	0.628	0.132	0.193

Tan	2	8513701	8514000	3.215	0.835	3.319	2.403
Tan	2	8514001	8514300	2.499	0.880	3.179	2.231
Tan	2	8591401	8591700	0.740	1.939	0.522	0.314
Tan	2	8904301	8904600	0.077	0.605	0.156	0.101
Tan	2	8904601	8904900	0.239	0.609	0.350	0.137
Tan	2	9033901	9034200	0.190	0.635	0.156	0.112
Tan	2	9209701	9210000	0.105	0.423	0.111	0.112
Tan	2	9229501	9229800	2.768	1.064	2.254	2.367
Tan	2	9652201	9652500	0.503	1.107	0.477	0.477
Tan	2	9664501	9664800	0.744	1.051	0.506	0.598
Tan	2	9779401	9779700	0.397	0.607	0.321	0.142
Tan	2	10032001	10032300	0.187	0.560	0.288	0.274
Tan	2	10284001	10284300	0.186	0.513	0.058	0.299
Tan	2	10465201	10465500	0.265	0.631	0.399	0.299
Tan	2	10618801	10619100	1.034	2.100	1.440	1.206
Tan	2	10660501	10660800	0.665	0.796	0.560	0.466
Tan	2	11004601	11004900	0.292	0.560	0.107	0.360
Tan	2	11222701	11223000	1.065	1.495	1.074	0.776
Tan	2	11449201	11449500	0.161	0.446	0.165	0.031
Tan	2	12031201	12031500	0.448	1.708	0.901	0.628
Tan	2	12054601	12054900	1.723	2.997	1.340	1.724
Tan	2	12105601	12105900	0.000	0.654	0.078	0.056
Tan	2	12554701	12555000	0.319	0.539	0.321	0.309
Tan	2	12643201	12643500	0.055	0.470	0.082	0.162
Tan	2	12863401	12863700	0.398	0.560	0.239	0.223
Tan	2	13126801	13127100	0.397	0.633	0.160	0.193
Tan	2	13186201	13186500	0.211	0.538	0.189	0.162
Tan	2	13205701	13206000	0.186	0.094	0.214	0.497
Tan	2	13225801	13226100	0.105	0.401	0.078	0.056
Tan	2	13344601	13344900	0.239	0.725	0.189	0.385
Tan	2	13440001	13440300	0.160	0.420	0.243	0.162
Tan	2	13440301	13440600	0.400	0.584	0.341	0.360
Tan	2	13550701	13551000	0.106	0.395	0.132	0.056
Tan	2	13781701	13782000	0.186	0.536	0.103	0.248
Tan	2	14023201	14023500	0.160	0.444	0.156	0.092
Tan	2	14214001	14214300	0.803	1.429	0.646	0.801
Tan	2	14518801	14519100	0.187	0.423	0.156	0.137
Tan	2	14594101	14594400	0.345	0.560	0.140	0.137
Tan	2	15091501	15091800	0.531	0.680	0.107	0.497
Tan	2	15496801	15497100	0.349	0.796	0.210	0.299
Tan	2	15501601	15501900	0.479	0.725	0.473	0.304
Tan	2	15550801	15551100	0.532	0.936	0.502	1.217
Tan	2	15735001	15735300	1.231	2.622	1.551	1.627
Tan	2	15854401	15854700	0.211	0.661	0.132	0.218
Tan	2	15934201	15934500	0.161	0.448	0.132	0.112
Tan	2	16014001	16014300	0.106	0.466	0.234	0.279
Tan	2	16078801	16079100	1.726	2.175	1.636	1.683
Tan	2	16400701	16401000	0.053	0.378	0.025	0.031
Tan	2	16910701	16911000	0.586	1.172	0.415	0.553
Tan	2	17163901	17164200	3.587	0.972	3.983	4.557
Tan	2	17235901	17236200	0.874	1.616	0.831	0.725
Tan	2	17418901	17419200	1.462	1.777	1.168	1.298
Tan	2	17439001	17439300	1.046	2.130	1.329	1.415
Tan	2	17622901	17623200	0.919	2.381	0.847	1.242
Tan	2	17989201	17989500	0.161	0.493	0.049	0.162
Tan	2	18026701	18027000	0.287	1.129	0.156	0.238
Tan	2	18263401	18263700	0.294	1.026	0.424	0.315
Tan	2	18654901	18655200	0.158	0.418	0.103	0.081
Tan	2	19335001	19335300	0.398	0.583	0.370	0.304
Tan	2	19729801	19730100	2.897	0.652	2.784	2.651
Tan	2	20024101	20024400	2.794	1.399	2.127	2.074
Tan	2	20296801	20297100	0.211	0.584	0.132	0.086
Tan	2	20600701	20601000	0.452	0.326	0.638	0.751
Tan	2	20634301	20634600	0.266	0.513	0.132	0.198
Tan	2	20671801	20672100	0.158	0.423	0.078	0.081
Tan	2	21263701	21264000	0.345	0.723	0.419	0.274
Tan	2	21639601	21639900	0.295	1.543	0.531	0.173

Tan	2	21781501	21781800	0.587	0.165	0.453	0.477
Tan	2	22660201	22660500	0.132	0.470	0.263	0.218
Tan	2	22681201	22681500	0.184	0.802	0.156	0.238
Tan	2	22695301	22695600	0.479	2.113	0.346	0.173
Tan	2	22721401	22721700	0.426	1.294	0.272	0.385
Tan	2	22721701	22722000	0.398	0.989	0.210	0.375
Tan	2	22911601	22911900	0.373	0.068	0.609	0.634
Tan	2	23166301	23166600	0.342	1.098	0.223	0.385
Tan	2	23229001	23229300	4.520	1.318	4.608	4.139
Tan	2	23251801	23252100	0.640	0.024	0.370	0.731
Tan	2	23815501	23815800	0.081	0.444	0.165	0.162
Tan	2	24023701	24024000	0.665	0.843	0.555	0.391
Tan	2	24072901	24073200	1.302	2.176	0.709	1.430
Tan	2	24090901	24091200	0.081	0.633	0.218	0.385
Tan	2	24267001	24267300	0.611	0.747	0.584	0.385
Tan	2	24339301	24339600	0.294	1.408	0.214	0.476
Tan	2	24340201	24340500	0.503	1.011	0.497	0.304
Tan	2	24351601	24351900	0.211	0.792	0.111	0.345
Tan	2	24394501	24394800	0.294	0.727	0.296	0.259
Tan	2	24963001	24963300	0.637	0.350	0.531	0.639
Tan	2	25806601	25806900	0.105	0.376	0.687	0.137
Tan	2	26365801	26366100	0.294	0.770	0.419	0.396
Tan	2	26673001	26673300	0.800	0.328	0.905	0.558
Tan	2	26683201	26683500	0.318	0.749	0.481	0.304
Tan	2	26847901	26848200	0.345	0.633	0.823	0.223
Tan	2	26866801	26867100	1.463	2.034	1.008	1.307
Tan	2	27017401	27017700	0.294	0.588	0.239	0.167
Tan	2	27387601	27387900	0.239	0.420	0.156	0.248
Tan	2	27593401	27593700	0.186	0.607	0.374	0.385
Tan	2	27782701	27783000	0.265	0.446	0.136	0.137
Tan	2	27886201	27886500	0.187	0.560	0.292	0.213
Tan	2	27916801	27917100	0.319	0.444	0.239	0.360
Tan	2	27920701	27921000	1.173	2.158	0.852	1.455
Tan	2	27928801	27929100	2.023	3.251	2.613	1.805
Tan	2	27931801	27932100	0.132	0.187	0.239	0.608
Tan	2	27963601	27963900	0.795	1.714	0.695	1.217
Tan	2	28032901	28033200	0.663	0.983	0.230	0.274
Tan	2	28366801	28367100	0.903	0.395	0.765	0.720
Tan	2	28475101	28475400	0.734	2.118	0.738	0.710
Tan	2	28497601	28497900	2.871	1.367	2.566	2.636
Tan	2	28727101	28727400	0.323	0.893	0.288	0.122
Tan	2	29538001	29538300	0.531	0.914	0.370	0.588
Tan	2	29542201	29542500	0.637	1.099	0.584	0.461
Tan	2	29542501	29542800	0.665	0.935	0.564	0.477
Tan	2	29763301	29763600	0.210	1.062	0.325	0.137
Tan	2	30058801	30059100	0.211	0.446	0.136	0.162
Tan	2	30089401	30089700	0.560	1.056	0.444	0.553
Tan	2	30135001	30135300	0.318	0.846	0.346	0.243
Tan	2	30810301	30810600	1.168	1.614	1.144	1.095
Tan	2	30814801	30815100	0.426	1.360	0.876	0.659
Tan	2	31242601	31242900	0.081	0.614	0.132	0.132
Tan	2	31294501	31294800	0.532	0.165	0.510	0.436
Tan	2	31514401	31514700	1.008	1.807	0.983	1.212
Tan	2	31586701	31587000	0.213	0.468	0.346	0.355
Tan	2	31908901	31909200	0.424	0.727	0.288	0.391
Tan	2	31937701	31938000	0.132	0.869	0.160	0.228
Tan	2	31938001	31938300	0.055	1.028	0.247	0.223
Tan	2	32019001	32019300	0.931	1.425	0.863	0.669
Tan	2	32260501	32260800	0.479	0.775	0.477	0.248
Tan	2	32444701	32445000	0.186	0.418	0.613	0.695
Tan	2	32619001	32619300	0.053	0.395	0.078	0.086
Tan	2	32697001	32697300	0.156	0.515	0.107	0.218
Tan	2	33120001	33120300	0.108	0.981	0.098	0.117
Tan	2	33505501	33505800	0.852	1.762	0.765	0.731
Tan	2	34312501	34312800	0.134	0.581	0.058	0.112
Tan	2	34515001	34515300	0.534	1.030	0.740	0.522
Tan	2	34557001	34557300	0.052	0.493	0.218	0.248

Tan	2	34686301	34686600	0.532	0.725	0.312	0.360
Tan	2	35363701	35364000	0.292	0.515	0.243	0.167
Tan	2	36474601	36474900	0.189	0.541	0.160	0.112
Tan	2	36666001	36666300	1.219	1.658	1.090	1.212
Tan	2	36690001	36690300	0.558	0.912	0.745	0.360
Tan	2	36749701	36750000	3.787	2.454	4.253	3.849
Tan	2	36771301	36771600	6.294	5.319	6.951	7.240
Tan	2	37072201	37072500	1.561	3.015	1.469	2.007
Tan	2	37139401	37139700	0.373	0.674	0.267	0.309
Tan	2	37210801	37211100	0.213	0.586	0.239	0.198
Tan	2	37331701	37332000	0.265	0.611	0.214	0.299
Tan	2	37655101	37655400	0.557	0.912	0.539	0.502
Tan	2	37784701	37785000	0.213	0.701	0.531	0.274
Tan	2	37867801	37868100	0.319	0.652	0.239	0.268
Tan	2	38070901	38071200	0.987	1.332	0.847	0.690
Tan	2	38358601	38358900	1.223	0.539	1.086	1.131
Tan	2	38453401	38453700	0.208	1.120	0.214	0.370
Tan	2	38502901	38503200	0.187	0.910	0.321	0.254
Tan	2	38687101	38687400	0.931	0.560	1.349	1.222
Tan	2	38687401	38687700	1.200	0.493	1.373	1.207
Tan	2	38712001	38712300	0.848	1.191	0.745	0.700
Tan	2	39356101	39356400	0.161	0.676	0.325	0.299
Tan	2	39586801	39587100	0.784	2.864	0.820	1.287
Tan	2	39651601	39651900	0.108	0.489	0.049	0.187
Tan	2	39830401	39830700	1.541	2.341	1.670	1.785
Tan	2	39873301	39873600	0.211	0.468	0.239	0.248
Tan	2	40134601	40134900	0.239	0.983	0.078	0.325
Tan	2	40172101	40172400	0.079	0.536	0.185	0.213
Tan	2	40749901	40750200	0.397	0.725	0.317	0.441
Tan	2	41507701	41508000	0.265	0.558	0.160	0.279
Tan	2	41979001	41979300	0.079	0.491	0.321	0.081
Tan	2	42008101	42008400	0.560	1.008	0.638	0.578
Tan	2	42123901	42124200	0.448	1.077	0.568	0.477
Tan	2	42283801	42284100	0.908	1.240	0.802	0.391
Tan	2	42293101	42293400	0.129	0.628	0.132	0.117
Tan	2	42344401	42344700	0.026	0.468	0.078	0.056
Tan	2	42344701	42345000	0.026	0.468	0.210	0.086
Tan	2	42386401	42386700	1.089	1.847	0.370	1.313
Tan	2	42692401	42692700	0.426	0.912	0.346	0.603
Tan	2	42809101	42809400	0.239	0.539	0.239	0.228
Tan	2	43042201	43042500	0.134	0.586	0.214	0.173
Tan	2	43102801	43103100	0.160	0.420	0.074	0.193
Tan	2	43276201	43276500	0.532	0.727	0.477	0.441
Tan	2	43336801	43337100	1.244	2.324	1.164	1.359
Tan	2	43356301	43356600	1.249	0.701	1.008	1.044
Tan	2	43622101	43622400	0.482	1.347	0.359	0.416
Tan	2	43622401	43622700	0.452	0.862	0.296	0.527
Tan	2	44426401	44426700	0.605	1.358	0.283	0.309
Tan	2	44743501	44743800	0.639	0.910	0.481	0.720
Tan	2	44775601	44775900	1.173	0.822	1.415	1.825
Tan	2	45167101	45167400	0.079	0.513	0.078	0.081
Tan	2	45167401	45167700	0.106	0.607	0.111	0.218
Tan	2	45172201	45172500	0.407	1.118	0.107	0.112
Tan	2	45427801	45428100	1.092	2.113	1.008	0.933
Tan	2	45706201	45706500	0.452	0.772	0.638	0.497
Tan	2	45834901	45835200	1.331	0.257	0.633	0.705
Tan	2	46043101	46043400	1.647	2.105	1.752	1.668
Tan	2	46301101	46301400	0.984	1.283	0.432	0.527
Tan	2	46301401	46301700	0.237	1.122	0.181	0.223
Tan	2	46301701	46302000	0.215	0.745	0.103	0.132
Tan	2	46820101	46820400	0.319	0.421	0.555	0.553
Tan	2	47042701	47043000	0.318	0.515	0.346	0.304
Tan	2	47172601	47172900	0.053	0.468	0.292	0.279
Tan	2	47419201	47419500	0.079	0.420	0.214	0.056
Tan	2	47575801	47576100	0.108	0.489	0.087	0.132
Tan	2	48478201	48478500	0.132	0.513	0.087	0.193
Tan	2	48733501	48733800	0.610	1.193	0.671	0.821

Tan	2	48764101	48764400	0.371	0.723	0.457	0.411
Tan	2	48826201	48826500	0.560	0.931	0.428	0.553
Tan	2	48929701	48930000	0.828	1.923	1.264	0.502
Tan	2	48936001	48936300	0.269	0.893	0.111	0.329
Tan	2	48999001	48999300	0.106	0.538	0.147	0.081
Tan	2	49037101	49037400	0.266	0.515	0.263	0.213
Tan	2	49296301	49296600	0.531	0.798	0.481	0.360
Tan	2	49320901	49321200	0.453	0.607	0.185	0.360
Tan	2	49351201	49351500	0.156	0.566	0.082	0.112
Tan	2	49513201	49513500	1.091	0.375	1.324	1.055
Tan	2	49513501	49513800	0.745	0.114	0.584	0.644
Tan	2	49668601	49668900	0.428	0.862	0.428	0.522
Tan	2	49680301	49680600	0.345	1.054	0.506	0.628
Tan	2	49726801	49727100	0.349	0.586	0.107	0.193
Tan	2	49857901	49858200	0.373	0.584	0.210	0.228
Tan	2	49917001	49917300	0.319	0.185	0.531	0.441
Tan	2	49932001	49932300	0.294	0.489	0.210	0.193
Tan	2	50097301	50097600	0.769	0.208	0.691	0.623
Tan	2	50384101	50384400	0.507	0.770	0.453	0.385
Tan	2	50420701	50421000	0.134	0.517	0.239	0.198
Tan	2	50615101	50615400	0.581	1.335	0.724	0.812
Tan	2	50689201	50689500	0.929	1.261	0.588	0.812
Tan	2	50728201	50728500	0.131	0.586	0.218	0.218
Tan	2	51140101	51140400	0.345	0.631	0.424	0.254
Tan	2	51191401	51191700	1.432	2.821	1.790	1.694
Tan	2	51523501	51523800	3.228	1.952	3.598	3.255
Tan	2	51561001	51561300	0.426	0.817	0.276	0.416
Tan	2	51582301	51582600	0.831	1.571	0.609	0.502
Tan	2	51769501	51769800	0.053	0.513	0.107	0.112
Tan	2	51807901	51808200	0.718	0.631	0.374	0.274
Tan	2	52142401	52142700	0.265	0.845	0.432	0.243
Tan	2	52305901	52306200	0.294	0.513	0.312	0.081
Tan	2	52526701	52527000	1.348	3.960	1.086	1.083
Tan	2	52546801	52547100	0.294	0.586	0.321	0.335
Tan	2	52570501	52570800	0.823	0.227	0.905	0.877
Tan	2	53004301	53004600	0.611	1.384	0.950	0.938
Tan	2	53097001	53097300	0.398	0.680	0.346	0.223
Tan	2	53974801	53975100	0.531	1.245	0.642	0.558
Tan	2	54255301	54255600	0.161	0.611	0.000	0.117
Tan	2	54484501	54484800	0.452	0.678	0.214	0.324
Tan	2	55431601	55431900	0.426	0.749	1.028	0.994
Tan	2	56021701	56022000	0.160	0.401	0.107	0.167
Tan	2	56529601	56529900	0.294	0.656	0.263	0.360
Tan	2	56625301	56625600	1.462	1.661	1.304	1.521
Tan	2	56673601	56673900	0.955	0.193	0.671	0.690
Tan	2	57290101	57290400	0.079	0.607	0.160	0.943
Tan	2	57481501	57481800	0.240	0.538	0.205	0.243
Tan	2	57656701	57657000	0.079	0.538	0.230	0.142
Tan	2	58017601	58017900	0.292	0.564	0.317	0.243
Tan	2	58068001	58068300	0.290	0.725	0.103	0.086
Tan	2	58068601	58068900	6.057	7.192	4.641	5.192
Tan	2	58260301	58260600	0.108	0.468	0.214	0.304
Tan	2	58262701	58263000	0.240	0.470	0.243	0.081
Tan	2	58265401	58265700	0.106	1.307	0.243	0.228
Tan	2	58265701	58266000	0.052	1.169	0.214	0.234
Tan	2	58381201	58381500	0.799	1.028	0.609	0.385
Tan	2	58418401	58418700	0.134	0.446	0.107	0.223
Tan	2	58741201	58741500	0.266	0.564	0.132	0.198
Tan	2	59007301	59007600	0.369	0.867	0.453	0.492
Tan	2	59046301	59046600	0.426	0.796	0.502	0.411
Tan	2	59476801	59477100	0.744	0.539	0.263	0.142
Tan	2	59834701	59835000	3.539	5.229	4.229	3.315
Tan	2	60029101	60029400	0.319	0.096	0.531	0.421
Tan	2	61939801	61940100	0.290	0.560	0.267	0.117
Tan	2	62793301	62793600	0.266	0.515	0.185	0.173
Tan	2	63499501	63499800	0.106	0.444	0.111	0.112
Tan	2	63626401	63626700	0.823	1.424	0.481	1.156

Tan	2	63687001	63687300	1.350	2.362	1.206	1.500
Tan	2	63921601	63921900	0.852	0.515	0.691	0.634
Tan	2	65220601	65220900	0.237	0.513	0.136	0.112
Tan	2	65363701	65364000	0.397	0.094	0.531	0.360
Tan	2	65385001	65385300	0.824	1.658	0.510	1.014
Tan	2	65459701	65460000	0.637	1.098	0.560	0.659
Tan	2	65655301	65655600	2.844	0.646	2.423	2.201
Tan	2	65655601	65655900	2.871	0.643	2.550	1.993
Tan	2	65688301	65688600	0.213	0.468	0.214	0.329
Tan	2	65750701	65751000	0.639	0.820	0.403	0.416
Tan	2	65937601	65937900	0.240	0.468	0.185	0.162
Tan	2	66028501	66028800	0.479	0.375	0.506	0.279
Tan	2	66232801	66233100	0.663	1.006	0.555	0.771
Tan	2	66362401	66362700	0.715	1.193	0.675	0.583
Tan	2	66363301	66363600	0.240	0.611	0.103	0.092
Tan	2	66781201	66781500	0.163	0.635	0.156	0.198
Tan	2	67040701	67041000	2.542	0.307	2.069	1.399
Tan	2	67041001	67041300	1.563	0.163	1.480	0.938
Tan	2	67380301	67380600	2.973	3.839	2.456	2.976
Tan	2	67688701	67689000	0.987	0.541	1.280	1.050
Tan	2	67702501	67702800	0.764	1.401	0.493	2.033
Tan	2	68296201	68296500	0.294	0.723	0.346	0.238
Tan	2	68355001	68355300	0.131	0.420	0.078	0.117
Tan	2	68445901	68446200	0.560	0.727	0.185	0.360
Tan	2	68782801	68783100	0.479	0.773	0.399	0.527
Tan	2	68890201	68890500	0.000	0.420	0.189	0.167
Tan	2	69713701	69714000	0.373	0.491	0.321	0.086
Tan	2	69818101	69818400	0.240	0.656	0.176	0.243
Tan	2	69896401	69896700	0.187	0.466	0.185	0.360
Tan	2	69904201	69904500	0.294	0.682	0.283	0.223
Tan	2	70497601	70497900	0.690	1.098	0.502	1.561
Tan	2	70565701	70566000	0.134	0.914	0.303	0.268
Tan	2	70682101	70682400	0.397	0.747	0.317	0.497
Tan	2	70965601	70965900	0.345	0.163	0.560	0.274
Tan	2	71424901	71425200	0.079	0.491	0.116	0.132
Tan	2	71787901	71788200	0.397	0.702	0.370	0.304
Tan	2	71886901	71887200	0.316	0.701	0.321	0.294
Tan	2	71973301	71973600	0.131	0.607	0.136	0.228
Tan	2	72111901	72112200	0.161	0.420	0.239	0.167
Tan	2	72309301	72309600	0.156	0.515	0.156	0.106
Tan	2	72309601	72309900	0.477	1.150	0.580	0.583
Tan	2	72459301	72459600	0.082	0.564	0.136	0.167
Tan	2	72561901	72562200	0.108	0.472	0.103	0.112
Tan	2	72620101	72620400	0.294	0.796	0.317	0.380
Tan	2	73085101	73085400	0.266	0.444	0.160	0.218
Tan	2	73594801	73595100	0.161	0.442	0.189	0.081
Tan	2	74004601	74004900	0.132	0.659	0.210	0.335
Tan	2	74227201	74227500	0.608	1.245	0.403	0.553
Tan	2	74436301	74436600	0.186	0.491	0.116	0.167
Tan	2	74471101	74471400	0.685	1.592	0.341	0.806
Tan	2	75540601	75540900	0.106	0.493	0.243	0.248
Tan	2	75626401	75626700	0.079	0.448	0.165	0.112
Tan	2	75695401	75695700	0.240	0.444	0.218	0.112
Tan	2	76262401	76262700	0.685	1.427	0.493	0.725
Tan	2	76485601	76485900	0.294	0.839	0.272	0.299
Tan	2	76894501	76894800	0.106	0.914	0.165	0.137
Tan	2	77026201	77026500	0.053	0.701	0.058	0.126
Tan	2	77026501	77026800	0.131	0.648	0.087	0.112
Tan	2	78552001	78552300	0.692	0.163	0.506	0.583
Tan	2	79536001	79536300	0.160	0.448	0.189	0.031
Tan	2	80407201	80407500	0.400	0.678	0.428	0.274
Tan	2	80680801	80681100	0.160	0.541	0.243	0.223
Tan	2	81130801	81131100	0.134	0.539	0.111	0.081
Tan	2	81470401	81470700	1.915	1.407	1.937	1.896
Tan	2	81489301	81489600	0.476	0.865	0.346	0.365
Tan	2	81727501	81727800	0.292	0.980	0.584	0.695
Tan	2	82054201	82054500	0.292	0.583	0.346	0.324

Tan	2	82341301	82341600	0.958	0.772	1.012	1.131
Tan	2	82589101	82589400	0.452	0.234	0.667	0.715
Tan	2	82975201	82975500	0.663	0.376	0.798	0.715
Tan	2	82990801	82991100	0.215	0.489	0.107	0.056
Tan	2	83178601	83178900	0.106	0.511	0.078	0.031
Tan	2	83452201	83452500	0.347	0.629	0.210	0.329
Tan	2	83632801	83633100	0.239	0.468	0.107	0.228
Tan	2	84082801	84083100	0.294	0.444	0.267	0.274
Tan	2	84313801	84314100	0.189	0.517	0.160	0.162
Tan	2	84322801	84323100	0.319	0.633	0.259	0.248
Tan	2	85161301	85161600	0.319	0.631	0.481	0.279
Tan	2	85563901	85564200	0.158	0.465	0.107	0.137
Tan	2	85592101	85592400	0.132	0.539	0.239	0.086
Tan	2	85802101	85802400	0.586	0.140	0.341	0.497
Tan	2	86133301	86133600	0.660	1.306	1.957	0.431
Tan	2	86373301	86373600	0.000	0.704	0.132	0.309
Tan	2	86373601	86373900	0.134	0.704	0.152	0.228
Tan	2	86837401	86837700	2.925	2.592	2.365	2.180
Tan	2	87221701	87222000	0.297	1.169	0.288	0.608
Tan	2	87396301	87396600	0.265	0.069	0.428	0.578
Tan	2	87622201	87622500	0.505	0.933	0.321	0.411
Tan	2	87765901	87766200	0.055	0.401	0.103	0.056
Tan	2	87791101	87791400	0.211	0.676	0.263	0.340
Tan	2	87876001	87876300	0.981	0.543	1.008	1.136
Tan	2	87894901	87895200	0.081	0.493	0.132	0.092
Tan	2	87965101	87965400	0.371	0.818	0.691	0.522
Tan	2	88173901	88174200	0.215	0.539	0.103	0.142
Tan	2	88293901	88294200	0.190	1.006	0.609	0.634
Tan	2	88368601	88368900	0.160	0.444	0.029	0.360
Tan	2	88479901	88480200	0.670	1.472	0.691	0.846
Tan	2	88504501	88504800	0.134	0.773	0.259	0.380
Tan	2	88543201	88543500	0.106	0.444	0.234	0.198
Tan	2	88870801	88871100	0.452	0.629	0.317	0.304
Tan	2	89139601	89139900	1.003	2.555	0.687	1.440
Tan	2	89139901	89140200	0.744	3.209	0.865	1.111
Tan	2	89140201	89140500	0.266	1.853	0.370	0.365
Tan	2	89415601	89415900	0.211	0.513	0.152	0.218
Tan	2	89498401	89498700	0.376	0.817	0.292	0.472
Tan	2	89744701	89745000	0.082	0.680	0.189	0.086
Tan	2	90234901	90235200	0.106	0.401	0.103	0.106
Tan	2	90411901	90412200	1.305	0.674	1.765	1.424
Tan	2	91085101	91085400	0.589	1.217	0.584	0.715
Tan	2	91146601	91146900	0.160	0.936	0.123	0.137
Tan	2	91146901	91147200	0.131	1.103	0.103	0.193
Tan	2	91149001	91149300	0.797	1.714	0.588	0.618
Tan	2	91227301	91227600	0.799	1.517	0.847	0.553
Tan	2	91275601	91275900	0.186	0.654	0.234	0.268
Tan	2	91561501	91561800	0.132	0.511	0.267	0.304
Tan	2	91726201	91726500	0.479	1.004	0.415	0.522
Tan	2	91809901	91810200	0.397	0.678	0.239	0.284
Tan	2	92686201	92686500	0.505	0.234	0.374	0.360
Tan	2	92749201	92749500	0.742	1.682	0.954	0.836
Tan	2	93107701	93108000	0.134	0.493	0.160	0.076
Tan	2	93179401	93179700	0.189	0.538	0.132	0.112
Tan	2	94126201	94126500	0.160	0.539	0.098	0.157
Tan	2	94317301	94317600	0.321	0.680	0.205	0.304
Tan	2	94499101	94499400	0.850	1.122	0.239	0.431
Tan	2	94510501	94510800	0.560	1.693	0.347	0.106
Tan	2	94510801	94511100	0.586	1.833	0.405	0.162
Tan	2	94563601	94563900	0.955	1.240	1.012	0.639
Tan	2	94836301	94836600	0.156	0.588	0.082	0.025
Tan	2	95310901	95311200	0.161	0.890	0.205	0.126
Tan	2	95311201	95311500	0.189	0.680	0.189	0.101
Tan	2	95478001	95478300	0.665	0.375	0.584	0.198
Tan	2	95531101	95531400	0.131	0.654	0.267	0.355
Tan	2	95660701	95661000	0.081	0.423	0.127	0.051
Tan	2	96160501	96160800	0.240	0.491	0.370	0.223

Tan	2	98097901	98098200	2.283	1.588	2.176	2.180
Tan	2	98272501	98272800	0.132	0.609	0.272	0.218
Tan	2	99702901	99703200	0.879	0.326	0.852	1.253
Tan	2	99840601	99840900	0.477	0.116	0.502	0.669
Tan	2	99872101	99872400	0.210	1.390	0.116	0.289
Tan	2	100386901	100387200	0.398	0.680	0.374	0.142
Tan	2	100470601	100470900	0.132	0.631	0.160	0.274
Tan	2	100473001	100473300	0.797	1.195	0.802	0.695
Tan	2	100585501	100585800	4.711	3.175	4.884	4.680
Tan	2	100585801	100586100	5.883	3.563	6.852	6.708
Tan	2	100589401	100589700	5.781	2.425	5.280	5.438
Tan	2	100625401	100625700	0.398	0.796	0.477	0.203
Tan	2	100641601	100641900	0.242	0.586	0.214	0.137
Tan	2	100644001	100644300	0.505	0.281	0.564	0.497
Tan	2	100712101	100712400	0.428	0.933	0.210	0.198
Tan	2	100712401	100712700	0.424	0.790	0.214	0.274
Tan	2	100805701	100806000	0.187	2.019	0.570	0.056
Tan	2	100806001	100806300	0.108	0.701	0.174	0.031
Tan	2	101297101	101297400	3.865	4.652	3.130	3.179
Tan	2	101387101	101387400	0.319	0.914	0.408	0.117
Tan	2	101487001	101487300	0.156	0.841	0.210	0.405
Tan	2	101707801	101708100	0.586	1.073	0.325	0.416
Tan	2	101743501	101743800	0.479	0.745	0.210	0.360
Tan	2	101748001	101748300	0.161	0.444	0.107	0.248
Tan	2	101806501	101806800	0.268	0.727	0.156	0.365
Tan	2	102374401	102374700	0.847	1.495	0.930	0.918
Tan	2	102387601	102387900	1.034	2.163	0.967	1.116
Tan	2	102420301	102420600	0.512	1.483	0.473	0.279
Tan	2	102426601	102426900	2.204	3.628	1.803	2.470
Tan	2	102426901	102427200	1.918	3.519	1.531	2.510
Tan	2	102447301	102447600	0.424	0.891	0.366	0.436
Tan	2	102487501	102487800	0.586	1.056	0.662	0.684
Tan	2	102542101	102542400	0.215	0.657	0.169	0.162
Tan	2	102951301	102951600	1.221	1.897	1.291	1.440
Tan	2	103038001	103038300	0.400	0.560	0.370	0.335
Tan	2	103073101	103073400	0.424	1.023	0.288	0.360
Tan	2	103115101	103115400	1.495	3.579	1.437	0.947
Tan	2	103116001	103116300	1.129	2.853	1.277	0.776
Tan	2	103116301	103116600	0.529	1.341	0.432	0.375
Tan	2	103135801	103136100	0.345	0.745	0.223	0.218
Tan	2	103235101	103235400	0.240	1.107	0.239	0.228
Tan	2	103347301	103347600	0.268	1.037	0.321	0.441
Tan	2	103428901	103429200	0.215	0.468	0.210	0.162
Tan	2	103532101	103532400	0.211	0.444	0.181	0.167
Tan	2	103547701	103548000	0.295	1.077	0.633	0.279
Tan	2	103808401	103808700	1.408	4.006	1.385	2.278
Tan	2	103822201	103822500	3.831	2.658	4.309	4.194
Tan	2	103916701	103917000	2.100	1.495	1.868	1.912
Tan	2	103963501	103963800	5.636	6.597	4.853	4.493
Tan	2	104006101	104006400	0.371	0.586	0.189	0.391
Tan	2	104054701	104055000	0.323	0.727	0.132	0.274
Tan	2	104255101	104255400	0.429	1.079	0.473	0.603
Tan	2	104415001	104415300	0.161	0.609	0.132	0.213
Tan	2	104415301	104415600	0.824	2.240	0.564	0.901
Tan	2	104434801	104435100	2.841	1.163	2.737	2.774
Tan	2	104437201	104437500	0.237	0.985	0.332	0.350
Tan	2	104437501	104437800	0.240	0.511	0.185	0.051
Tan	2	105451501	105451800	0.210	0.749	0.453	0.329
Tan	2	105686401	105686700	0.424	0.704	0.160	0.193
Tan	2	105816901	105817200	0.582	1.053	0.691	0.669
Tan	2	106003201	106003500	0.344	0.818	0.223	0.218
Tan	2	106278301	106278600	1.753	2.573	1.667	1.425
Tan	2	106278601	106278900	1.379	3.139	1.293	0.893
Tan	2	106361701	106362000	0.373	0.609	0.272	0.142
Tan	2	106382101	106382400	0.505	1.509	0.254	0.527
Tan	2	106395001	106395300	4.328	1.436	4.124	5.018
Tan	2	106407601	106407900	0.026	0.657	0.025	0.000

Tan	2	106407901	106408200	0.026	0.772	0.025	0.000
Tan	2	106437901	106438200	0.319	0.628	0.341	0.279
Tan	2	106474801	106475100	0.026	0.607	0.156	0.329
Tan	2	106510801	106511100	0.452	0.770	0.453	0.664
Tan	2	106590901	106591200	1.705	2.551	1.358	1.293
Tan	2	106591201	106591500	1.037	1.474	0.555	0.781
Tan	2	106593901	106594200	0.132	0.393	0.132	0.112
Tan	2	106602001	106602300	0.500	1.212	0.341	0.411
Tan	2	106632601	106632900	0.292	0.749	0.107	0.268
Tan	2	106702201	106702500	0.081	0.725	0.098	0.112
Tan	2	106702501	106702800	0.081	0.532	0.049	0.056
Tan	2	106766401	106766700	0.186	0.511	0.185	0.167
Tan	2	106802701	106803000	0.211	0.350	0.481	0.553
Tan	2	106908901	106909200	0.215	0.773	0.370	0.370
Tan	2	106976101	106976400	0.108	0.395	0.078	0.106
Tan	2	107311801	107312100	0.773	1.779	0.745	1.343
Tan	2	107580601	107580900	0.769	1.836	0.905	0.766
Tan	2	107628901	107629200	0.239	0.656	0.078	0.137
Tan	2	107697301	107697600	0.240	0.491	0.185	0.228
Tan	2	108308401	108308700	0.268	0.657	0.074	0.101
Tan	2	108403201	108403500	0.819	0.283	1.398	0.877
Tan	2	108655801	108656100	0.876	1.772	0.729	0.604
Tan	2	109416001	109416300	0.053	0.513	0.152	0.198
Tan	2	109918201	109918500	0.453	0.112	1.016	0.583
Tan	2	109953601	109953900	0.106	1.431	0.239	0.613
Tan	2	109953901	109954200	0.266	0.611	0.181	0.193
Tan	2	110087401	110087700	0.639	1.216	0.593	0.679
Tan	2	110087701	110088000	0.426	0.607	0.321	0.446
Tan	2	110100001	110100300	0.347	0.049	0.370	0.547
Tan	2	110117401	110117700	0.797	1.523	0.959	0.857
Tan	2	110171401	110171700	0.508	1.832	0.617	0.669
Tan	2	110375101	110375400	0.295	0.706	0.317	0.304
Tan	2	110379001	110379300	0.745	1.420	0.691	0.816
Tan	2	110464801	110465100	1.822	3.373	1.224	1.542
Tan	2	110696701	110697000	0.660	1.425	0.695	0.776
Tan	2	110876101	110876400	2.236	1.515	2.292	2.013
Tan	2	110928601	110928900	2.320	1.053	1.736	1.683
Tan	2	110939101	110939400	3.199	2.128	2.991	3.032
Tan	2	111358801	111359100	0.345	0.822	0.453	0.309
Tan	2	111495301	111495600	0.213	0.539	0.165	0.106
Tan	2	111495601	111495900	0.134	0.466	0.136	0.218
Tan	2	111822001	111822300	1.963	2.547	1.904	1.901
Tan	2	111830701	111831000	1.860	1.354	1.949	2.429
Tan	2	112571401	112571700	0.240	0.517	0.214	0.162
Tan	2	112595101	112595400	0.108	0.420	0.029	0.101
Tan	2	112597201	112597500	0.242	0.792	0.428	0.309
Tan	2	112862101	112862400	0.187	0.489	0.214	0.106
Tan	2	112963801	112964100	0.265	1.199	0.203	0.243
Tan	2	112995301	112995600	0.344	0.701	0.341	0.092
Tan	2	113058601	113058900	0.213	0.489	0.136	0.112
Tan	2	113106301	113106600	0.106	0.513	0.210	0.254
Tan	2	113106901	113107200	0.213	0.423	0.185	0.193
Tan	2	113567401	113567700	0.797	0.656	1.032	0.416
Tan	2	113989801	113990100	0.106	0.423	0.239	0.248
Tan	2	114081301	114081600	0.213	0.584	0.214	0.335
Tan	2	114602401	114602700	0.211	0.466	0.210	0.198
Tan	2	115425901	115426200	0.447	1.174	0.588	0.492
Tan	2	115426201	115426500	0.500	1.453	0.531	0.618
Tan	2	115741201	115741500	0.213	0.730	0.346	0.248
Tan	2	115862701	115863000	0.319	0.727	0.502	0.391
Tan	2	116119801	116120100	0.639	1.216	0.831	0.533
Tan	2	116228701	116229000	1.597	2.527	1.759	1.455
Tan	2	116327101	116327400	0.426	1.004	0.412	0.547
Tan	2	116340601	116340900	1.702	2.929	1.222	1.419
Tan	2	116346001	116346300	0.215	0.470	0.103	0.112
Tan	2	116478901	116479200	0.477	0.701	0.502	0.446
Tan	2	116704501	116704800	0.136	0.800	0.296	0.112

Tan	2	116782201	116782500	0.321	0.094	0.395	0.466
Tan	2	116811601	116811900	0.431	1.009	0.379	0.411
Tan	2	117527701	117528000	0.239	0.470	0.054	0.112
Tan	2	117633901	117634200	0.689	1.433	0.667	0.462
Tan	2	117652501	117652800	2.360	2.995	1.411	2.059
Tan	2	117732001	117732300	1.937	2.971	1.415	1.977
Tan	2	117804301	117804600	0.318	0.560	0.239	0.223
Tan	2	118167601	118167900	0.108	0.723	0.239	0.385
Tan	2	118341601	118341900	0.106	0.444	0.136	0.147
Tan	2	118378201	118378500	0.749	1.500	0.486	0.659
Tan	2	118619401	118619700	0.347	0.723	0.205	0.274
Tan	2	118749301	118749600	0.426	0.326	0.185	0.578
Tan	2	118809901	118810200	0.689	1.143	0.642	0.745
Tan	2	118920601	118920900	0.398	1.193	0.934	0.832
Tan	2	119099701	119100000	0.345	0.629	0.321	0.142
Tan	2	119947801	119948100	0.266	0.468	0.132	0.198
Tan	2	120943801	120944100	0.053	0.395	0.049	0.061
Tan	2	121131601	121131900	0.134	0.420	0.185	0.142
Tan	2	121366801	121367100	0.215	0.420	0.156	0.081
Tan	2	121883701	121884000	0.081	0.423	0.103	0.112
Tan	2	121933501	121933800	0.160	0.536	0.292	0.167
Tan	2	121960801	121961100	4.430	1.928	3.431	4.883
Tan	2	122088601	122088900	0.213	0.936	0.098	0.512
Tan	2	122088901	122089200	0.132	0.558	0.239	0.274
Tan	2	122570401	122570700	3.299	2.285	3.233	3.468
Tan	2	123121201	123121500	0.161	0.493	0.263	0.137
Tan	2	123231001	123231300	0.294	0.794	0.296	0.289
Tan	2	123247801	123248100	1.545	2.285	1.353	1.586
Tan	2	123305101	123305400	0.426	1.077	0.408	0.461
Tan	2	124036801	124037100	0.106	0.420	0.132	0.137
Tan	2	124529401	124529700	0.163	0.515	0.210	0.137
Tan	2	124890301	124890600	0.269	0.656	0.107	0.329
Tan	2	125255401	125255700	1.113	0.373	0.740	0.690
Tan	2	125833501	125833800	0.265	0.730	0.292	0.268
Tan	2	125969401	125969700	0.053	0.543	0.127	0.106
Tan	2	127030201	127030500	0.374	0.818	0.580	0.608
Tan	2	127548601	127548900	0.187	0.729	0.210	0.385
Tan	2	127746601	127746900	0.292	0.493	0.263	0.218
Tan	2	127999801	128000100	0.106	0.519	0.107	0.193
Tan	2	128000101	128000400	0.106	0.586	0.107	0.193
Tan	2	128779801	128780100	0.987	2.038	1.210	0.593
Tan	2	129028201	129028500	1.087	2.246	0.425	0.441
Tan	2	129505801	129506100	0.160	0.515	0.156	0.198
Tan	2	129573301	129573600	5.097	4.188	5.281	5.071
Tan	2	129942601	129942900	0.079	0.446	0.210	0.086
Tan	2	129975901	129976200	1.760	0.931	1.518	1.435
Tan	2	130190401	130190700	0.507	0.258	0.794	0.477
Tan	2	130252801	130253100	0.507	0.749	0.506	0.492
Tan	2	130268401	130268700	0.160	0.493	0.132	0.218
Tan	2	130286101	130286400	1.037	0.817	1.460	1.141
Tan	2	130298101	130298400	0.321	0.560	0.082	0.076
Tan	2	130370401	130370700	0.903	1.217	1.012	0.684
Tan	2	130586101	130586400	0.532	0.678	0.881	0.441
Tan	2	130591201	130591500	0.131	0.470	0.156	0.162
Tan	2	130591801	130592100	0.158	0.444	0.078	0.254
Tan	2	130900201	130900500	0.323	0.682	0.272	1.242
Tan	2	131275501	131275800	0.474	1.144	0.638	0.193
Tan	2	131295601	131295900	1.144	0.485	1.168	1.739
Tan	2	131310001	131310300	0.240	0.536	0.078	0.061
Tan	2	131311501	131311800	0.160	0.371	0.107	0.000
Tan	2	131354701	131355000	0.161	0.723	0.098	0.182
Tan	2	131745901	131746200	0.213	0.586	0.160	0.117
Tan	2	132119701	132120000	0.211	1.317	0.818	0.659
Tan	2	132157501	132157800	0.318	0.538	0.165	0.167
Tan	2	132279001	132279300	0.637	0.189	0.609	0.628
Tan	2	132315601	132315900	0.349	1.362	0.210	0.507
Tan	2	132445801	132446100	2.155	2.575	2.312	1.709

Tan	2	132505501	132505800	0.379	1.845	0.468	0.522
Tan	2	132510001	132510300	0.373	0.989	0.140	0.441
Tan	2	132510301	132510600	0.373	0.888	0.107	0.461
Tan	2	132520801	132521100	0.265	0.654	0.267	0.162
Tan	2	132751801	132752100	0.718	0.560	0.292	0.446
Tan	2	133122001	133122300	0.215	0.682	0.341	0.299
Tan	2	133185901	133186200	0.610	1.101	0.658	0.801
Tan	2	133244701	133245000	0.158	0.515	0.288	0.137
Tan	2	133406101	133406400	0.663	0.890	0.160	0.309
Tan	2	133431901	133432200	0.295	0.611	0.025	0.137
Tan	2	133623301	133623600	0.400	0.983	0.408	0.563
Tan	2	133711801	133712100	1.116	1.691	1.119	0.776
Tan	2	134040001	134040300	0.082	0.657	0.160	0.268
Tan	2	134118601	134118900	0.448	0.936	0.580	0.522
Tan	2	134169301	134169600	1.726	2.839	1.478	1.816
Tan	2	134222701	134223000	0.505	1.171	0.357	0.628
Tan	2	134251201	134251500	0.237	0.695	0.107	0.106
Tan	2	134253001	134253300	0.715	1.214	0.350	0.731
Tan	2	135397201	135397500	0.347	0.656	0.234	0.385
Tan	2	136279201	136279500	2.844	1.661	3.087	3.073
Tan	2	136551901	136552200	1.573	2.853	1.736	1.222
Tan	2	136560901	136561200	1.010	1.802	1.186	0.801
Tan	2	136581301	136581600	0.453	0.822	0.481	0.360
Tan	2	136903501	136903800	0.294	0.729	0.296	0.335
Tan	2	137077501	137077800	0.426	0.790	0.453	0.441
Tan	2	137151001	137151300	0.239	0.609	0.288	0.167
Tan	2	137450401	137450700	0.319	0.611	0.185	0.304
Tan	2	137538001	137538300	0.292	0.444	0.107	0.056
Tan	2	137638501	137638800	0.318	1.032	0.477	0.466
Tan	2	138030001	138030300	0.347	0.513	0.107	0.218
Tan	2	138293101	138293400	0.534	0.116	0.341	0.329
Tan	2	138408901	138409200	0.000	0.395	0.103	0.142
Tan	2	138797401	138797700	1.170	1.779	1.070	0.634
Tan	2	139161601	139161900	0.639	0.863	0.720	0.720
Tan	2	139491001	139491300	0.134	0.466	0.156	0.081
Tan	2	139557901	139558200	0.239	0.538	0.317	0.187
Tan	2	139593001	139593300	7.069	4.568	7.882	7.022
Tan	2	139803901	139804200	0.423	1.008	0.497	0.492
Tan	2	139879801	139880100	0.189	0.493	0.132	0.187
Tan	2	139954201	139954500	0.986	1.309	0.613	0.821
Tan	2	139973401	139973700	0.106	0.654	0.127	0.147
Tan	2	140044801	140045100	0.529	1.032	0.580	0.558
Tan	2	140060101	140060400	0.081	0.895	0.354	0.248
Tan	2	140060401	140060700	0.052	0.562	0.292	0.279
Tan	2	140088301	140088600	0.740	1.307	0.617	0.832
Tan	2	140213101	140213400	0.160	0.444	0.214	0.299
Tan	2	140411101	140411400	4.438	1.476	4.835	3.297
Tan	2	140411401	140411700	1.652	0.322	1.431	1.136
Tan	2	140415001	140415300	1.307	4.240	1.284	0.917
Tan	2	140436001	140436300	2.605	1.614	2.245	2.423
Tan	2	140596201	140596500	0.187	0.511	0.185	0.304
Tan	2	140850001	140850300	0.081	0.487	0.185	0.112
Tan	2	141570001	141570300	0.213	0.444	0.370	0.304
Tan	2	142282201	142282500	0.215	0.517	0.243	0.142
Tan	2	142418701	142419000	0.239	0.562	0.292	0.274
Tan	2	142788001	142788300	0.215	0.470	0.054	0.142
Tan	2	142845901	142846200	0.292	0.584	0.283	0.248
Tan	2	143073901	143074200	0.400	0.957	0.374	0.238
Tan	2	143098801	143099100	0.160	0.423	0.107	0.137
Tan	2	143585101	143585400	0.369	1.056	0.361	0.461
Tan	2	143788501	143788800	0.292	0.491	0.267	0.254
Tan	2	145269601	145269900	0.398	0.633	0.267	0.355
Tan	2	145906801	145907100	0.818	1.429	0.506	0.547
Tan	2	146207101	146207400	0.261	0.656	0.210	0.223
Tan	2	146235301	146235600	0.213	0.564	0.296	0.329
Tan	2	146776501	146776800	0.507	0.912	0.502	0.695
Tan	2	146786701	146787000	0.292	0.511	0.716	0.248

Tan	2	147527401	147527700	0.668	1.285	0.560	0.720
Tan	2	148403101	148403400	1.254	1.821	1.230	1.287
Tan	2	148587001	148587300	0.000	0.371	0.054	0.167
Tan	2	149163301	149163600	0.453	1.053	0.691	0.603
Tan	2	149457901	149458200	0.132	0.420	0.292	0.248
Tan	2	150019201	150019500	0.106	0.395	0.127	0.056
Tan	2	150020101	150020400	0.213	0.607	0.428	0.329
Tan	2	150065701	150066000	0.423	0.938	0.292	0.466
Tan	2	150230101	150230400	0.534	0.165	0.428	0.497
Tan	2	150551401	150551700	0.374	0.862	0.189	0.370
Tan	2	151554301	151554600	0.768	1.637	0.749	0.847
Tan	2	151863901	151864200	0.213	0.723	0.189	0.254
Tan	2	152032501	152032800	0.053	0.423	0.087	0.112
Tan	2	152145901	152146200	3.933	3.624	4.496	5.258
Tan	2	152352601	152352900	0.105	0.466	0.152	0.106
Tan	2	152858101	152858400	0.160	0.744	0.136	0.234
Tan	2	152858401	152858700	0.160	0.747	0.160	0.335
Tan	2	153334501	153334800	0.265	0.584	0.453	0.360
Tan	2	154561501	154561800	0.294	0.820	0.223	0.304
Tan	2	155076001	155076300	0.239	0.465	0.025	0.086
Tan	2	155589001	155589300	0.639	0.959	0.740	0.547
Tan	2	155788801	155789100	0.507	1.165	0.571	0.553
Tan	2	156180601	156180900	1.003	3.003	1.206	0.841
Tan	2	156319801	156320100	7.990	6.634	8.023	9.259
Tan	2	156410701	156411000	2.150	3.177	1.761	2.373
Tan	2	156480901	156481200	1.118	0.865	0.535	0.664
Tan	2	156523201	156523500	0.402	1.025	0.468	0.553
Tan	2	156557701	156558000	0.210	0.796	0.424	0.243
Tan	2	156638701	156639000	0.663	1.197	0.515	0.628
Tan	2	157182601	157182900	0.505	0.633	0.317	0.472
Tan	2	157642501	157642800	0.318	0.983	0.301	0.380
Tan	2	157642801	157643100	0.347	1.122	0.374	0.360
Tan	2	157695001	157695300	0.263	0.820	0.267	0.502
Tan	2	157855801	157856100	0.160	0.401	0.132	0.086
Tan	2	158174401	158174700	0.052	0.449	0.078	0.025
Tan	2	158247601	158247900	0.184	0.586	0.292	0.162
Tan	2	158311801	158312100	0.265	0.654	0.132	0.228
Tan	2	158514301	158514600	0.239	0.678	0.234	0.193
Tan	2	159015601	159015900	0.453	0.629	0.210	0.218
Tan	2	159331201	159331500	0.134	0.442	0.132	0.025
Tan	2	159602401	159602700	0.105	0.702	0.276	0.279
Tan	2	159715801	159716100	0.237	0.633	0.341	0.162
Tan	2	159716101	159716400	0.952	1.948	1.173	1.064
Tan	2	159761401	159761700	0.692	0.959	0.613	0.715
Tan	2	159809101	159809400	0.720	0.350	0.662	0.751
Tan	2	160629901	160630200	0.213	0.723	0.395	0.193
Tan	2	160937401	160937700	0.132	0.444	0.210	0.162
Tan	2	160972501	160972800	0.560	0.985	0.593	0.603
Tan	2	161219101	161219400	0.052	0.446	0.160	0.000
Tan	2	161260801	161261100	0.720	1.143	0.716	0.482
Tan	2	161432101	161432400	0.134	0.494	0.082	0.223
Tan	2	161822701	161823000	0.161	0.470	0.189	0.193
Tan	2	162624001	162624300	0.268	0.539	0.214	0.254
Tan	2	162709801	162710100	0.661	0.279	0.560	0.857
Tan	2	162762901	162763200	0.108	0.558	0.243	0.132
Tan	2	164013301	164013600	0.213	0.633	0.296	0.248
Tan	2	164032501	164032800	0.026	0.494	0.189	0.051
Tan	2	164604001	164604300	0.350	0.796	0.346	0.132
Tan	2	165856501	165856800	1.037	1.637	0.729	1.136
Tan	2	166517701	166518000	0.187	0.841	0.415	0.223
Tan	2	168105001	168105300	0.321	1.004	0.317	0.385
Tan	2	168236401	168236700	0.186	0.560	0.136	0.213
Tan	2	169854901	169855200	0.108	0.515	0.058	0.117
Tan	2	170340301	170340600	0.134	0.674	0.214	0.259
Tan	2	170645401	170645700	0.581	1.313	0.560	0.796
Tan	2	170725801	170726100	0.481	0.071	0.876	0.502
Tan	2	172826701	172827000	0.081	0.650	0.210	0.025

Tan	2	173033101	173033400	0.134	0.515	0.218	0.132
Tan	2	173163901	173164200	0.160	0.631	0.234	0.472
Tan	2	173234401	173234700	0.268	0.588	0.239	0.274
Tan	2	173528101	173528400	0.805	2.178	0.954	0.674
Tan	2	173872501	173872800	0.318	0.884	0.267	0.355
Tan	2	173903701	173904000	0.052	0.442	0.107	0.106
Tan	2	173912401	173912700	0.369	0.721	0.341	0.279
Tan	2	173912701	173913000	0.294	0.611	0.370	0.355
Tan	2	174075901	174076200	0.136	0.491	0.210	0.117
Tan	2	174080101	174080400	0.211	0.586	0.214	0.259
Tan	2	174304201	174304500	0.266	0.397	0.267	0.578
Tan	2	174604501	174604800	0.265	0.699	0.267	0.391
Tan	2	174735601	174735900	0.158	0.491	0.054	0.137
Tan	2	175155301	175155600	1.116	2.780	0.967	1.100
Tan	2	175155601	175155900	0.829	2.221	0.695	0.938
Tan	2	175161001	175161300	0.558	1.332	0.595	0.578
Tan	2	175221301	175221600	0.055	0.586	0.272	0.162
Tan	2	175361101	175361400	0.507	0.725	0.346	0.553
Tan	2	175398001	175398300	0.055	1.143	0.111	0.173
Tan	2	175476001	175476300	0.453	0.886	0.288	0.365
Tan	2	176031001	176031300	0.186	0.423	0.239	0.137
Tan	2	176114101	176114400	0.160	0.423	0.185	0.051
Tan	2	176263501	176263800	0.799	0.468	0.370	0.720
Tan	2	176438701	176439000	0.160	0.491	0.272	0.137
Tan	2	178077301	178077600	1.113	2.463	0.967	0.923
Tan	2	180000001	180000300	0.507	0.701	0.930	0.411
Tan	2	181800601	181800900	0.268	0.517	0.078	0.167
Tan	2	182961301	182961600	0.215	0.539	0.185	0.193
Tan	2	183411901	183412200	0.294	0.818	0.296	0.405
Tan	2	183772801	183773100	0.611	1.099	0.510	0.628
Tan	2	184126201	184126500	0.450	0.725	0.132	0.299
Tan	2	184277701	184278000	0.452	0.729	0.156	0.248
Tan	2	184644901	184645200	0.505	0.938	0.419	0.527
Tan	2	184682101	184682400	0.403	1.032	0.473	0.274
Tan	2	185018101	185018400	0.584	0.865	0.526	0.603
Tan	2	185073601	185073900	3.796	2.384	2.949	2.997
Tan	2	185161801	185162100	0.423	0.796	0.448	0.274
Tan	2	185446201	185446500	0.958	1.380	0.593	0.492
Tan	2	185473201	185473500	0.615	0.959	0.526	0.664
Tan	2	185509801	185510100	1.089	0.865	0.560	0.659
Tan	2	185576101	185576400	0.637	0.772	0.481	0.527
Tan	2	185644501	185644800	0.160	0.562	0.156	0.279
Tan	2	185658001	185658300	0.131	0.562	0.185	0.198
Tan	2	185734501	185734800	0.373	0.560	0.374	0.304
Tan	2	185775301	185775600	0.826	1.755	1.015	0.913
Tan	2	185775601	185775900	1.199	2.465	1.068	1.019
Tan	2	185835301	185835600	0.455	1.171	0.901	0.796
Tan	2	186152401	186152700	0.158	0.564	0.181	0.198
Tan	2	187424101	187424400	0.373	0.815	0.341	0.324
Tan	2	187868101	187868400	0.186	0.562	0.370	0.355
Tan	2	188564701	188565000	0.563	1.326	0.205	0.375
Tan	2	190852801	190853100	0.187	0.539	0.107	0.274
Tan	2	190986301	190986600	0.211	0.071	0.506	0.335
Tan	2	191302201	191302500	0.052	0.704	0.049	0.081
Tan	2	191489701	191490000	0.208	0.701	0.267	0.132
Tan	2	191730601	191730900	0.079	0.494	0.029	0.061
Tan	2	191895901	191896200	0.879	1.382	0.836	0.745
Tan	2	191896201	191896500	0.984	1.669	1.012	0.639
Tan	2	191974801	191975100	0.266	0.491	0.214	0.142
Tan	2	192174901	192175200	0.319	1.618	0.370	0.360
Tan	2	192312301	192312600	0.452	1.058	0.267	0.187
Tan	2	192765601	192765900	3.499	1.234	3.304	3.259
Tan	2	192974101	192974400	0.026	0.468	0.234	0.223
Tan	2	193687201	193687500	0.661	1.188	0.617	0.472
Tan	2	194295901	194296200	0.373	0.163	0.502	0.416
Tan	2	194310301	194310600	0.689	1.189	0.716	0.315
Tan	2	195516001	195516300	0.505	0.375	0.267	0.274

Tan	2	195762601	195762900	0.347	0.938	0.428	0.259
Tan	2	196483801	196484100	1.116	0.257	1.329	0.968
Tan	2	196484101	196484400	1.139	0.281	1.173	1.070
Tan	2	196486801	196487100	0.000	0.539	0.029	0.203
Tan	2	196487101	196487400	0.000	0.515	0.029	0.203
Tan	2	196562401	196562700	3.055	1.266	2.378	2.276
Tan	2	196702201	196702500	0.557	0.862	0.234	0.329
Tan	2	196737001	196737300	0.294	0.513	0.239	0.299
Tan	2	197484901	197485200	0.344	0.749	0.234	0.385
Tan	2	197887201	197887500	0.558	1.266	0.341	0.679
Tan	2	199091701	199092000	0.740	1.517	0.687	0.872
Tan	2	199360501	199360800	2.025	1.519	2.078	1.962
Tan	2	199443901	199444200	0.611	0.305	1.070	0.644
Tan	2	200136601	200136900	0.266	0.493	0.346	0.112
Tan	2	200774101	200774400	0.563	1.116	0.502	0.477
Tan	2	200959501	200959800	1.274	1.053	1.806	0.628
Tan	2	201126601	201126900	0.319	0.701	0.448	0.309
Tan	2	201396001	201396300	0.266	0.635	0.239	0.056
Tan	2	201489001	201489300	0.292	0.629	0.218	0.416
Tan	2	201602101	201602400	0.186	0.493	0.288	0.167
Tan	2	201603001	201603300	0.876	1.311	0.950	0.821
Tan	2	202206301	202206600	0.344	0.813	0.263	0.198
Tan	2	202408801	202409100	0.395	1.051	0.205	0.432
Tan	2	202428901	202429200	0.636	0.865	0.584	0.335
Tan	2	202843801	202844100	0.239	0.493	0.189	0.279
Tan	2	203581201	203581500	0.134	0.536	0.317	0.279
Tan	2	203754601	203754900	0.400	0.751	0.341	0.355
Tan	2	204103501	204103800	0.187	0.446	0.185	0.086
Tan	2	204348901	204349200	0.532	0.773	0.453	0.502
Tan	2	204428101	204428400	0.210	0.775	0.312	0.360
Tan	2	204704701	204705000	1.621	1.144	1.402	1.831
Tan	2	204732901	204733200	0.289	0.798	0.317	0.274
Tan	2	205240201	205240500	0.055	0.418	0.160	0.167
Tan	2	205246801	205247100	0.027	0.468	0.107	0.117
Tan	2	206309101	206309400	0.161	0.515	0.218	0.137
Tan	2	206487301	206487600	0.294	0.605	0.312	0.081
Tan	2	206701201	206701500	0.239	0.446	0.189	0.279
Tan	2	206956201	206956500	1.174	0.260	0.716	1.055
Tan	2	208132801	208133100	0.345	0.680	0.337	0.335
Tan	2	208451401	208451700	0.452	1.384	0.379	0.720
Tan	2	208464001	208464300	0.505	0.723	0.321	0.466
Tan	2	208692301	208692600	0.053	0.491	0.210	0.268
Tan	2	208910701	208911000	0.802	0.302	0.789	1.156
Tan	2	209012101	209012400	1.528	3.302	0.852	1.146
Tan	2	209055301	209055600	0.158	0.491	0.132	0.173
Tan	2	209236801	209237100	1.534	0.663	1.572	3.636
Tan	2	209832901	209833200	0.718	0.631	1.061	0.527
Tan	2	209859301	209859600	0.186	0.510	0.156	0.137
Tan	2	210309301	210309600	0.479	0.609	0.160	0.411
Tan	2	210477001	210477300	0.240	0.444	0.132	0.112
Tan	2	210496201	210496500	0.426	0.657	0.453	0.385
Tan	2	210511801	210512100	0.105	0.494	0.132	0.142
Tan	2	211029301	211029600	0.165	1.096	0.390	0.117
Tan	2	211029601	211029900	0.161	0.470	0.165	0.025
Tan	2	211274401	211274700	1.226	2.124	0.905	1.151
Tan	2	211443001	211443300	0.079	0.420	0.181	0.051
Tan	2	211539601	211539900	0.740	0.116	0.609	0.679
Tan	2	212001901	212002200	0.189	0.470	0.185	0.056
Tan	2	212162701	212163000	0.479	0.092	0.609	0.628
Tan	2	213433201	213433500	0.158	0.423	0.111	0.112
Tan	2	213440701	213441000	0.532	0.120	0.292	0.360
Tan	2	213990601	213990900	0.398	0.772	0.292	0.263
Tan	2	215137201	215137500	0.158	0.395	0.078	0.112
Tan	2	216055801	216056100	0.290	0.865	0.461	0.243
Tan	2	216266401	216266700	1.249	0.910	0.370	0.538
Tan	2	216293701	216294000	1.018	2.034	1.032	1.298
Tan	2	217250401	217250700	0.160	0.558	0.292	0.106

Tan	2	217289101	217289400	0.211	0.586	0.230	0.193
Tan	2	217493401	217493700	0.663	1.099	0.646	0.502
Tan	2	217597801	217598100	2.636	1.731	2.748	2.459
Tan	2	217691101	217691400	2.025	1.545	0.963	1.004
Tan	2	217839901	217840200	0.321	0.680	0.234	0.254
Tan	2	217933501	217933800	0.371	0.609	0.160	0.309
Tan	2	218047501	218047800	0.237	0.489	0.160	0.218
Tan	2	218058601	218058900	0.347	1.129	0.473	0.329
Tan	2	218066701	218067000	0.265	1.004	0.473	0.355
Tan	2	218286001	218286300	5.111	4.106	5.783	5.791
Tan	2	218415901	218416200	0.371	0.652	0.214	0.309
Tan	2	219057001	219057300	0.900	1.498	1.012	0.751
Tan	2	219070501	219070800	0.213	0.659	0.132	0.101
Tan	2	219200401	219200700	1.965	2.976	2.205	2.059
Tan	2	219375301	219375600	0.955	1.714	0.945	1.009
Tan	2	219547801	219548100	0.345	0.961	0.502	0.690
Tan	2	219548101	219548400	0.850	1.875	1.115	1.308
Tan	2	219566401	219566700	0.452	0.656	0.424	0.360
Tan	2	219573601	219573900	0.213	0.470	0.181	0.081
Tan	2	219611401	219611700	2.863	4.384	2.735	2.707
Tan	2	219914701	219915000	0.213	0.513	0.107	0.223
Tan	2	219942601	219942900	0.213	0.562	0.341	0.329
Tan	2	219990901	219991200	0.239	0.466	0.609	0.335
Tan	2	220084201	220084500	0.211	0.652	0.205	0.203
Tan	2	220307701	220308000	2.609	1.352	2.737	2.678
Tan	2	220487101	220487400	1.195	1.927	0.831	0.740
Tan	2	220509001	220509300	1.942	1.354	1.761	1.775
Tan	2	220511701	220512000	0.240	0.564	0.267	0.254
Tan	2	220799101	220799400	0.160	0.071	0.691	0.167
Tan	2	221337301	221337600	0.081	0.210	0.477	0.441
Tan	2	221955001	221955300	0.531	0.940	0.399	0.472
Tan	2	222173701	222174000	0.106	0.444	0.082	0.162
Tan	2	222519001	222519300	3.704	4.803	3.150	3.006
Tan	2	223657201	223657500	0.824	1.573	0.847	0.791
Tan	2	223674901	223675200	0.850	0.444	0.531	0.832
Tan	2	223832401	223832700	1.881	3.199	4.378	1.612
Tan	2	224061901	224062200	0.263	0.794	0.432	0.248
Tan	2	224169601	224169900	0.400	0.560	0.818	1.410
Tan	2	224930701	224931000	0.136	1.363	0.435	0.126
Tan	2	224931001	224931300	0.103	0.794	0.283	0.051
Tan	2	226139401	226139700	1.621	1.777	1.431	1.136
Tan	2	226166101	226166400	0.026	0.493	0.234	0.112
Tan	2	226560601	226560900	0.319	0.515	0.695	0.695
Tan	2	226664101	226664400	0.108	0.538	0.136	0.203
Tan	2	226759801	226760100	0.213	0.446	0.243	0.223
Tan	2	227250601	227250900	0.294	0.652	0.267	0.380
Tan	2	227981401	227981700	2.442	1.433	2.118	2.180
Tan	2	228079801	228080100	0.158	0.702	0.189	0.117
Tan	2	228090601	228090900	0.878	1.328	0.604	0.715
Tan	2	228163201	228163500	0.584	0.702	0.424	0.167
Tan	2	228255901	228256200	3.459	4.302	3.458	3.463
Tan	2	228408301	228408600	0.905	0.611	0.901	1.136
Tan	2	228723001	228723300	0.189	0.446	0.078	0.056
Tan	2	229023001	229023300	0.132	0.423	0.132	0.167
Tan	2	229529401	229529700	0.079	0.279	0.399	0.527
Tan	2	229592401	229592700	0.187	0.515	0.103	0.092
Tan	2	230574601	230574900	0.239	0.539	0.189	0.112
Tan	2	230633401	230633700	0.661	2.105	0.432	1.003
Tan	2	230884801	230885100	0.457	0.981	0.403	0.517
Tan	2	231022201	231022500	0.290	0.796	0.588	0.335
Tan	2	231956701	231957000	0.505	0.772	0.247	0.411
Tan	2	231978001	231978300	0.186	0.489	0.263	0.279
Tan	2	231984301	231984600	0.982	1.472	0.658	1.161
Tan	2	232020601	232020900	0.452	0.656	0.366	0.304
Tan	2	232105201	232105500	0.869	3.111	1.119	0.609
Tan	2	232136101	232136400	0.319	0.773	0.350	0.547
Tan	2	232350601	232350900	0.292	0.605	0.082	0.147

Tan	2	232483201	232483500	0.211	0.566	0.185	0.167
Tan	2	232877101	232877400	0.773	1.028	0.564	0.659
Tan	2	233029501	233029800	2.796	2.148	1.230	1.512
Tan	2	233321401	233321700	1.621	1.054	1.625	1.739
Tan	2	233444701	233445000	0.132	0.444	0.029	0.193
Tan	2	233489401	233489700	4.785	3.319	4.445	4.603
Tan	2	233910301	233910600	1.273	1.824	1.280	0.486
Tan	2	234200401	234200700	0.637	0.914	0.531	0.340
Tan	2	234238201	234238500	0.211	0.536	0.239	0.167
Tan	2	234282001	234282300	0.424	0.729	0.132	0.391
Tan	2	234297001	234297300	0.237	0.701	0.160	0.365
Tan	2	234325501	234325800	0.215	0.609	0.263	0.329
Tan	2	234370201	234370500	0.266	0.491	0.189	0.309
Tan	2	234474901	234475200	0.853	1.191	0.711	0.669
Tan	2	234775801	234776100	0.636	0.981	0.346	0.329
Tan	2	234866101	234866400	0.294	0.891	0.288	0.578
Tan	2	234931501	234931800	2.326	0.817	1.810	3.524
Tan	2	235074301	235074600	0.555	0.985	0.428	0.527
Tan	2	235135801	235136100	0.428	0.792	0.395	0.492
Tan	2	235211401	235211700	0.295	0.749	0.317	0.304
Tan	2	235220401	235220700	0.131	0.466	0.127	0.162
Tan	2	235240501	235240800	0.215	0.491	0.103	0.254
Tan	2	235680601	235680900	2.471	4.765	3.234	3.935
Tan	2	235721701	235722000	0.292	0.517	0.263	0.198
Tan	2	235723201	235723500	2.020	1.261	1.431	1.075
Tan	2	235768501	235768800	0.160	0.234	0.424	0.497
Tan	2	236021701	236022000	0.666	0.867	0.424	0.583
Tan	2	236135401	236135700	0.161	0.468	0.160	0.173
Tan	2	236198401	236198700	0.106	0.472	0.082	0.167
Tan	2	236248201	236248500	0.692	0.444	0.584	0.553
Tan	2	236460901	236461200	0.266	0.583	0.140	0.081
Tan	2	236821501	236821800	0.160	0.820	0.502	0.558
Tan	2	236975401	236975700	0.477	0.094	0.778	0.669
Tan	2	236985001	236985300	0.053	0.493	0.156	0.228
Tan	2	237078001	237078300	0.213	0.611	0.103	0.391
Tan	2	237163201	237163500	0.557	0.820	0.419	0.223
Tan	2	237180301	237180600	0.345	0.891	0.486	0.304
Tan	2	237187501	237187800	0.508	0.959	0.395	0.380
Tan	2	237326701	237327000	0.052	0.423	0.136	0.000
Tan	2	237332701	237333000	0.161	0.560	0.127	0.117
Tan	2	237666901	237667200	0.477	1.077	0.575	0.547
Tan	2	237840301	237840600	0.266	0.678	0.049	0.243
Tan	2	237936001	237936300	1.462	2.422	1.255	1.116
Tan	2	238460701	238461000	2.341	3.388	1.636	1.978
Tan	2	238776601	238776900	1.996	1.223	2.040	2.327
Tan	2	238871401	238871700	0.106	0.539	0.210	0.187
Tan	2	238884901	238885200	0.182	0.633	0.205	0.162
Tan	2	238885201	238885500	0.321	0.936	0.399	0.319
Tan	2	238925101	238925400	0.900	1.667	0.687	0.907
Tan	2	239446801	239447100	1.092	0.326	0.802	0.776
Tan	2	239492101	239492400	1.521	3.365	1.480	1.785
Tan	2	239527801	239528100	0.237	0.605	0.218	0.173
Tan	2	239796901	239797200	5.854	3.430	6.873	5.901
Tan	2	239831701	239832000	0.294	0.444	0.263	0.279
Tan	2	239951101	239951400	1.381	1.731	1.057	1.247
Tan	2	240150901	240151200	3.296	2.367	3.779	3.656
Tan	2	240169201	240169500	3.052	4.293	3.034	2.910
Tan	2	240171601	240171900	0.345	0.678	0.321	0.268
Tan	2	240395101	240395400	1.254	0.420	0.945	1.247
Tan	2	240537901	240538200	0.134	0.423	0.132	0.051
Tan	2	240704701	240705000	0.503	0.702	0.185	0.391
Tan	2	240994501	240994800	0.136	0.753	0.288	0.248
Tan	2	241142401	241142700	2.636	3.205	2.414	2.591
Tan	2	241154101	241154400	0.903	1.148	0.453	0.725
Tan	2	241197901	241198200	0.321	1.225	0.246	0.213
Tan	2	241356901	241357200	0.903	1.053	0.560	1.298
Tan	2	241476901	241477200	1.276	1.776	1.008	1.136

Tan	2	241750501	241750800	0.423	0.656	0.107	0.385
Tan	2	241934701	241935000	0.323	0.817	0.292	0.446
Tan	2	241966201	241966500	0.561	0.912	0.370	0.533
Tan	2	241987201	241987500	0.265	0.515	0.234	0.112
Tan	2	242016601	242016900	0.213	0.539	0.185	0.213
Tan	2	242063701	242064000	0.958	0.680	1.193	0.527
Tan	2	242083501	242083800	0.132	0.401	0.107	0.142
Tan	2	242171701	242172000	0.479	0.680	0.292	0.167
Tan	2	242510701	242511000	0.639	0.978	0.395	0.547
Tan	2	242629801	242630100	0.134	0.817	0.172	0.223
Tan	2	242761801	242762100	0.532	0.817	0.506	0.527
Tan	2	242766001	242766300	1.118	0.539	0.905	0.963
Tan	2	242956801	242957100	1.652	2.523	1.543	1.942
Tan	2	243005701	243006000	1.199	1.852	1.144	0.902
Tan	2	243006001	243006300	0.452	0.747	0.428	0.360
Tan	2	243085801	243086100	0.265	0.706	1.086	1.111
Tan	2	243127501	243127800	0.720	0.210	0.642	0.659
Tan	2	243173401	243173700	0.795	0.399	0.818	0.882
Tan	2	243217201	243217500	1.249	1.918	1.110	1.161
Tan	2	243219901	243220200	1.862	3.529	3.184	2.804
Tan	2	243464401	243464700	1.721	0.594	2.205	2.226
Tan	2	243516301	243516600	3.661	2.098	0.247	4.172
Tan	2	243578701	243579000	0.905	1.098	0.905	0.669
Tan	2	243699001	243699300	0.265	0.583	0.243	0.274
Tan	2	243845401	243845700	3.802	2.276	3.718	4.087
Tan	2	244107301	244107600	0.558	0.938	0.551	0.583
Tan	2	244152601	244152900	0.610	1.030	0.560	0.512
Tan	2	244169701	244170000	0.342	1.127	0.468	0.547
Tan	2	244180501	244180800	0.529	1.304	0.765	0.446
Tan	2	244298701	244299000	0.721	1.401	0.774	0.457
Tan	2	244323301	244323600	1.065	1.933	1.008	0.821
Tan	2	244663501	244663800	0.745	0.863	0.453	0.690
Tan	2	244673701	244674000	0.398	0.773	0.432	0.254
Tan	2	244959301	244959600	1.807	1.500	1.173	2.150
Tan	2	244970101	244970400	0.265	0.749	0.288	0.274
Tan	2	244991101	244991400	0.158	0.395	0.185	0.167
Tan	2	244991401	244991700	0.213	0.468	0.321	0.385
Tan	2	245011801	245012100	0.905	0.281	0.609	0.477
Tan	2	245039401	245039700	0.184	0.583	0.239	0.279
Tan	2	245048401	245048700	0.397	0.938	0.506	0.603
Tan	2	245087101	245087400	0.718	1.472	0.987	0.761
Tan	2	245211001	245211300	0.450	0.745	0.243	0.117
Tan	2	245226601	245226900	1.010	0.354	1.262	0.882
Tan	2	245240401	245240700	1.308	2.858	0.936	1.035
Tan	2	245259301	245259600	0.474	1.032	0.301	0.329
Tan	2	245259601	245259900	0.661	2.474	0.749	0.578
Tan	2	245274601	245274900	2.633	1.631	2.685	3.418
Tan	2	245275501	245275800	1.115	0.283	0.999	1.420
Tan	2	245278501	245278800	0.608	1.051	0.629	0.603
Tan	2	245318401	245318700	0.105	0.768	0.189	0.294
Tan	2	245318701	245319000	0.132	0.841	0.218	0.319
Tan	2	245322001	245322300	1.092	2.514	1.052	1.202
Tan	2	245322301	245322600	1.863	3.911	1.456	1.702
Tan	2	245325301	245325600	0.163	1.311	0.111	0.167
Tan	2	245325601	245325900	0.053	0.489	0.087	0.025
Tan	2	245326501	245326800	0.424	1.832	0.428	0.765
Tan	2	245326801	245327100	0.108	1.178	0.259	0.416
Tan	2	245330401	245330700	0.321	0.633	0.317	0.274
Tan	2	245486701	245487000	0.314	0.725	0.218	0.304
Tan	2	245589301	245589600	1.013	1.283	0.740	0.857
Tan	2	245621101	245621400	0.347	0.584	0.346	0.335
Tan	2	245679901	245680200	0.079	0.468	0.098	0.117
Tan	2	245681101	245681400	0.395	0.914	0.308	0.355
Tan	2	245790301	245790600	0.294	0.468	0.189	0.137
Tan	2	245826301	245826600	2.818	1.920	3.429	3.159
Tan	2	245930701	245931000	0.797	1.150	0.617	0.664
Tan	2	245989801	245990100	0.081	0.470	0.185	0.173

Tan	2	246055801	246056100	2.599	3.901	2.265	2.272
Tan	2	246125401	246125700	1.887	2.173	1.625	1.962
Tan	2	246316201	246316500	7.716	7.125	6.438	8.052
Tan	2	246368701	246369000	0.347	0.964	0.408	0.416
Tan	2	246400801	246401100	0.187	0.560	0.399	0.223
Tan	2	246461401	246461700	0.187	0.446	0.103	0.162
Tan	2	246597001	246597300	0.984	0.541	1.164	1.136
Tan	2	246666001	246666300	5.759	3.392	6.782	7.079
Tan	2	246727201	246727500	1.487	0.981	2.164	1.876
Tan	2	246783601	246783900	0.661	1.238	0.604	0.675
Tan	2	246843001	246843300	3.455	4.158	2.731	3.032
Tan	2	246886801	246887100	1.996	3.519	2.127	2.004
Tan	2	246933601	246933900	3.538	2.201	2.735	2.839
Tan	2	246945301	246945600	0.636	0.375	0.818	0.943
Tan	2	247086301	247086600	2.595	1.732	2.608	0.898
Tan	2	247411201	247411500	0.161	0.493	0.214	0.142
Tan	2	247460701	247461000	0.242	0.560	0.239	0.132
Tan	2	247465501	247465800	1.723	2.553	1.612	1.723
Tan	2	247490401	247490700	0.986	1.401	0.959	1.156
Tan	2	247494901	247495200	6.229	7.398	5.974	4.897
Tan	2	247605001	247605300	0.345	0.584	0.234	0.218
Tan	2	247672201	247672500	6.285	4.061	6.947	7.088
Tan	2	247672501	247672800	11.954	6.159	12.298	12.798
Tan	2	247692901	247693200	0.292	0.491	0.239	0.228
Tan	2	247711201	247711500	3.154	1.682	3.085	3.123
Tan	2	247740901	247741200	0.881	0.324	0.916	1.019
Tan	2	247833001	247833300	3.795	0.984	3.395	3.296
Tan	2	247833301	247833600	3.017	1.163	3.663	4.037
Tan	2	247858801	247859100	3.426	2.457	3.395	3.524
Tan	2	247863001	247863300	0.584	0.279	0.453	0.522
Tan	2	247971601	247971900	9.179	6.484	7.638	7.767
Tan	2	247990501	247990800	0.823	1.446	0.999	0.806
Tan	2	248029801	248030100	0.321	1.143	0.497	0.522
Tan	2	248070901	248071200	1.889	0.629	1.456	1.369
Tan	2	248108401	248108700	0.797	1.307	0.678	0.441
Tan	2	248142901	248143200	3.110	2.547	2.225	2.317
Tan	2	248383801	248384100	1.626	2.332	1.679	1.744
Tan	2	248390701	248391000	1.542	1.970	0.963	1.161
Tan	2	248556301	248556600	0.187	0.515	0.210	0.218
Tan	2	248686501	248686800	0.929	0.657	0.379	0.304
Tan	2	248696401	248696700	0.558	1.268	0.551	0.720
Tan	2	248709001	248709300	2.152	3.236	2.156	2.540
Tan	2	248819101	248819400	2.866	2.246	2.851	3.062
Tan	2	248948401	248948700	0.210	0.633	0.321	0.162
Tan	20	549301	549600	0.745	1.189	0.568	0.781
Tan	20	1433401	1433700	0.156	0.635	0.082	0.081
Tan	20	1444801	1445100	1.494	0.914	1.480	1.815
Tan	20	1555201	1555500	0.371	0.629	0.127	0.162
Tan	20	1574401	1574700	0.445	1.259	0.354	0.315
Tan	20	1852201	1852500	0.137	0.704	0.239	0.228
Tan	20	2203801	2204100	0.239	0.517	0.165	0.248
Tan	20	2394601	2394900	0.213	0.584	0.136	0.051
Tan	20	2648701	2649000	0.477	0.846	0.424	0.385
Tan	20	2673301	2673600	0.663	1.714	0.769	0.573
Tan	20	2799301	2799600	0.240	0.539	0.160	0.309
Tan	20	2950201	2950500	0.052	0.564	0.234	0.198
Tan	20	2951401	2951700	0.426	0.798	0.481	0.167
Tan	20	3104101	3104400	0.318	0.468	0.267	0.304
Tan	20	3310501	3310800	0.557	1.030	0.370	0.669
Tan	20	3417001	3417300	0.371	0.515	0.321	0.304
Tan	20	3475201	3475500	0.345	0.562	0.292	0.391
Tan	20	3627601	3627900	0.349	1.510	0.564	0.765
Tan	20	3675901	3676200	0.532	0.281	0.185	0.360
Tan	20	3711301	3711600	0.026	0.890	0.029	0.000
Tan	20	3813301	3813600	2.686	4.158	3.143	3.007
Tan	20	3877201	3877500	0.186	0.588	0.025	0.203
Tan	20	4260901	4261200	0.823	1.242	0.769	0.502

Tan	20	4555801	4556100	0.136	0.468	0.127	0.132
Tan	20	4700701	4701000	1.328	1.757	1.251	0.735
Tan	20	4893301	4893600	0.637	1.122	0.736	0.715
Tan	20	4960801	4961100	0.376	0.985	0.580	0.583
Tan	20	5087101	5087400	0.213	0.493	0.103	0.187
Tan	20	5129401	5129700	0.186	0.513	0.214	0.309
Tan	20	5187901	5188200	0.053	0.371	0.000	0.076
Tan	20	5276401	5276700	0.507	0.704	0.453	0.218
Tan	20	5709301	5709600	0.237	0.751	0.185	0.142
Tan	20	5737201	5737500	0.131	0.442	0.111	0.081
Tan	20	5782501	5782800	5.498	3.710	5.703	6.363
Tan	20	6169501	6169800	0.027	0.444	0.078	0.101
Tan	20	6658501	6658800	0.557	0.839	0.321	0.466
Tan	20	6681301	6681600	1.731	0.751	1.694	1.166
Tan	20	6805801	6806100	0.745	1.317	0.477	0.578
Tan	20	6976501	6976800	0.265	0.513	0.160	0.248
Tan	20	7081501	7081800	0.292	0.562	0.181	0.167
Tan	20	7123201	7123500	4.470	2.077	3.531	4.604
Tan	20	7306801	7307100	0.428	0.142	0.477	0.639
Tan	20	7368001	7368300	4.141	5.325	3.912	4.538
Tan	20	7410001	7410300	5.147	4.162	5.323	5.552
Tan	20	7587301	7587600	6.819	8.814	6.079	6.444
Tan	20	7664101	7664400	0.321	0.753	0.263	0.335
Tan	20	7803601	7803900	0.134	0.491	0.239	0.243
Tan	20	7872601	7872900	0.266	0.678	0.876	1.136
Tan	20	7905301	7905600	0.773	1.290	0.687	0.619
Tan	20	7981801	7982100	1.812	2.770	1.761	1.602
Tan	20	8059201	8059500	1.731	1.047	1.547	1.516
Tan	20	8133901	8134200	1.223	0.680	1.411	1.253
Tan	20	8226001	8226300	1.355	0.940	1.431	1.840
Tan	20	8234401	8234700	0.186	0.444	0.267	0.162
Tan	20	8406901	8407200	0.984	1.493	0.756	0.887
Tan	20	8654401	8654700	1.487	2.158	1.509	1.511
Tan	20	8703601	8703900	0.268	0.538	1.275	0.385
Tan	20	8778601	8778900	0.237	0.397	0.239	0.664
Tan	20	8846401	8846700	0.507	0.257	0.667	0.391
Tan	20	9075301	9075600	0.294	0.959	0.234	0.304
Tan	20	9102601	9102900	1.303	2.710	1.287	1.997
Tan	20	9119101	9119400	0.184	0.466	0.103	0.223
Tan	20	9119701	9120000	0.156	0.536	0.136	0.092
Tan	20	9122101	9122400	0.424	1.118	0.448	0.518
Tan	20	9136201	9136500	0.507	0.796	0.218	0.081
Tan	20	9242701	9243000	0.106	0.468	0.107	0.132
Tan	20	9263701	9264000	0.426	0.609	0.296	0.248
Tan	20	9690601	9690900	0.608	1.670	0.609	0.730
Tan	20	9961801	9962100	0.215	0.633	0.267	0.147
Tan	20	10026601	10026900	1.094	2.605	1.237	1.673
Tan	20	10121101	10121400	0.239	0.444	0.054	0.137
Tan	20	10131001	10131300	0.586	1.290	0.490	0.432
Tan	20	10134001	10134300	0.106	0.446	0.160	0.051
Tan	20	10235401	10235700	0.426	0.584	0.321	0.446
Tan	20	10330201	10330500	0.160	0.466	0.181	0.056
Tan	20	10367101	10367400	0.266	0.444	0.214	0.056
Tan	20	10711501	10711800	0.503	0.281	0.555	0.583
Tan	20	10794301	10794600	0.242	0.822	0.111	0.335
Tan	20	10851301	10851600	1.115	1.687	1.193	0.862
Tan	20	11049301	11049600	0.455	1.058	0.346	0.218
Tan	20	11109001	11109300	0.661	1.339	0.617	0.710
Tan	20	11229001	11229300	0.345	0.770	0.308	0.335
Tan	20	11401501	11401800	0.081	0.446	0.082	0.081
Tan	20	11482201	11482500	1.197	1.682	1.099	1.014
Tan	20	11608201	11608500	1.918	1.399	1.904	1.901
Tan	20	11739001	11739300	0.773	1.510	0.457	0.720
Tan	20	11761801	11762100	0.532	0.678	0.185	0.279
Tan	20	11772001	11772300	0.215	0.607	0.214	0.268
Tan	20	12067501	12067800	0.266	0.538	0.214	0.385
Tan	20	12165901	12166200	0.213	0.751	0.218	0.385

Tan	20	12180301	12180600	0.371	0.867	0.395	0.446
Tan	20	12259201	12259500	0.158	0.684	0.267	0.274
Tan	20	12383101	12383400	0.323	0.888	0.234	0.578
Tan	20	12726901	12727200	0.239	0.539	0.267	0.274
Tan	20	12736201	12736500	0.797	1.264	0.852	0.948
Tan	20	12976201	12976500	3.993	2.285	2.934	2.961
Tan	20	13153501	13153800	0.318	0.863	0.189	0.295
Tan	20	13218601	13218900	3.905	3.034	3.620	4.133
Tan	20	13612801	13613100	0.266	0.682	0.181	0.304
Tan	20	13755901	13756200	0.132	0.470	0.210	0.117
Tan	20	13756801	13757100	0.295	0.796	0.325	0.259
Tan	20	13907101	13907400	1.089	1.706	1.099	1.151
Tan	20	13913701	13914000	0.105	0.517	0.136	0.142
Tan	20	14004601	14004900	1.165	2.922	1.380	1.205
Tan	20	14066401	14066700	0.426	0.586	0.477	0.304
Tan	20	14224501	14224800	0.371	0.747	0.181	0.477
Tan	20	14255401	14255700	0.747	1.219	0.292	0.416
Tan	20	14259001	14259300	1.145	0.730	1.596	1.780
Tan	20	14283901	14284200	2.820	1.487	2.245	2.211
Tan	20	14331001	14331300	0.292	0.682	0.292	0.142
Tan	20	14728201	14728500	0.158	0.676	0.283	0.193
Tan	20	14895001	14895300	0.134	0.534	0.103	0.193
Tan	20	14945101	14945400	0.216	0.704	0.181	0.106
Tan	20	14981101	14981400	0.268	0.682	0.160	0.025
Tan	20	15007501	15007800	0.371	0.023	0.379	0.492
Tan	20	15058801	15059100	0.532	0.981	0.633	0.492
Tan	20	15260401	15260700	0.081	0.491	0.025	0.162
Tan	20	15266701	15267000	1.170	0.208	1.119	0.878
Tan	20	15363601	15363900	1.218	2.150	0.522	1.055
Tan	20	15749401	15749700	0.529	1.476	0.867	0.700
Tan	20	16055401	16055700	0.823	1.259	0.272	0.816
Tan	20	16464901	16465200	2.897	2.032	2.599	2.581
Tan	20	16465501	16465800	3.930	2.909	5.693	4.482
Tan	20	16876201	16876500	5.136	6.357	5.045	4.675
Tan	20	16888801	16889100	0.213	0.562	0.341	0.218
Tan	20	16953301	16953600	0.187	0.541	0.267	0.274
Tan	20	17073001	17073300	1.699	2.965	1.757	2.139
Tan	20	17079601	17079900	1.781	2.499	1.489	1.627
Tan	20	17523301	17523600	0.132	0.468	0.181	0.193
Tan	20	17609401	17609700	1.011	1.242	0.584	1.541
Tan	20	17610001	17610300	1.252	1.824	0.675	1.247
Tan	20	17636401	17636700	0.424	0.749	0.292	0.299
Tan	20	17739001	17739300	0.213	0.303	0.560	0.167
Tan	20	17771701	17772000	0.131	0.442	0.185	0.167
Tan	20	17877901	17878200	1.171	0.817	1.115	1.131
Tan	20	17897701	17898000	0.265	1.026	0.292	0.370
Tan	20	17956501	17956800	0.208	0.839	0.243	0.223
Tan	20	17960401	17960700	0.239	0.725	0.374	0.365
Tan	20	18099001	18099300	0.505	0.330	0.185	0.112
Tan	20	18114001	18114300	0.665	0.515	0.267	0.664
Tan	20	18119401	18119700	2.126	3.424	2.069	1.936
Tan	20	18154501	18154800	0.290	0.676	0.243	0.391
Tan	20	18177301	18177600	0.027	0.510	0.103	0.142
Tan	20	18374101	18374400	0.186	0.517	0.054	0.173
Tan	20	18557101	18557400	0.532	1.058	0.457	0.533
Tan	20	18588301	18588600	0.187	0.418	0.160	0.137
Tan	20	18799201	18799500	2.100	2.292	2.127	2.738
Tan	20	18870601	18870900	0.186	0.468	0.054	0.243
Tan	20	19026301	19026600	0.321	0.562	0.160	0.360
Tan	20	19475101	19475400	0.426	0.701	0.481	0.391
Tan	20	19791301	19791600	0.213	0.423	0.107	0.000
Tan	20	19856101	19856400	0.319	0.444	0.214	0.279
Tan	20	19915501	19915800	0.349	0.980	0.082	0.152
Tan	20	19934101	19934400	0.239	1.009	0.296	0.193
Tan	20	20382301	20382600	1.200	1.498	0.428	0.634
Tan	20	20688301	20688600	0.319	0.749	0.555	0.279
Tan	20	21140101	21140400	2.109	3.394	2.089	1.780

Tan	20	21221101	21221400	0.294	0.723	0.370	0.441
Tan	20	21492901	21493200	0.108	0.536	0.074	0.051
Tan	20	21854701	21855000	0.560	0.324	0.107	0.112
Tan	20	22335901	22336200	0.453	0.869	0.374	0.370
Tan	20	22699201	22699500	0.321	0.657	0.267	0.254
Tan	20	23421601	23421900	0.242	0.586	0.214	0.329
Tan	20	23512501	23512800	0.292	0.491	0.243	0.193
Tan	20	23769601	23769900	1.145	1.611	0.876	1.262
Tan	20	24231301	24231600	0.239	0.725	0.350	0.304
Tan	20	24231601	24231900	1.041	2.107	1.244	0.765
Tan	20	24241801	24242100	0.053	0.465	0.127	0.162
Tan	20	24420301	24420600	0.240	0.794	0.165	0.315
Tan	20	24475501	24475800	0.160	0.656	0.366	0.117
Tan	20	24511501	24511800	2.789	3.201	1.752	1.764
Tan	20	24597001	24597300	0.611	1.362	0.671	0.675
Tan	20	24851701	24852000	2.638	1.940	2.584	2.520
Tan	20	24852001	24852300	9.217	6.292	9.474	9.656
Tan	20	24957601	24957900	1.842	3.092	1.621	2.180
Tan	20	25038301	25038600	0.507	1.165	0.288	0.756
Tan	20	25167601	25167900	0.160	0.444	0.132	0.142
Tan	20	25198501	25198800	1.197	2.066	2.855	1.440
Tan	20	25294801	25295100	0.158	0.803	1.579	0.167
Tan	20	26152801	26153100	0.161	0.493	0.054	0.106
Tan	20	26182201	26182500	0.081	0.444	0.156	0.248
Tan	20	26518501	26518800	0.215	0.745	0.239	0.436
Tan	20	26546701	26547000	2.291	1.798	1.275	1.034
Tan	20	27317101	27317400	0.240	0.444	0.711	0.223
Tan	20	27333001	27333300	0.240	0.541	0.185	0.162
Tan	20	27388501	27388800	0.963	1.571	0.720	0.938
Tan	20	27513001	27513300	1.274	2.004	1.378	1.222
Tan	20	27575701	27576000	0.694	1.504	0.769	0.821
Tan	20	27746401	27746700	0.237	0.538	0.267	0.228
Tan	20	27930001	27930300	0.708	1.899	0.299	0.370
Tan	20	27985201	27985500	0.290	0.562	0.078	0.228
Tan	20	28177801	28178100	0.266	0.888	0.388	0.254
Tan	20	28299601	28299900	0.079	0.609	0.301	0.299
Tan	20	28801201	28801500	1.753	1.045	0.535	0.365
Tan	20	29082001	29082300	0.077	0.659	0.176	0.193
Tan	20	29109301	29109600	0.345	0.699	0.370	0.350
Tan	20	29109601	29109900	0.739	1.397	0.526	0.654
Tan	20	29126401	29126700	0.537	1.026	0.477	0.441
Tan	20	29156101	29156400	0.398	0.794	0.346	0.198
Tan	20	29725201	29725500	0.158	0.538	0.103	0.137
Tan	20	30259801	30260100	0.739	2.130	0.526	0.777
Tan	20	30291601	30291900	0.324	1.056	0.205	0.411
Tan	20	30833101	30833400	0.186	0.539	0.181	0.132
Tan	20	30846601	30846900	0.857	1.976	0.609	0.963
Tan	20	31083601	31083900	1.460	2.105	1.451	1.451
Tan	20	31096501	31096800	0.670	1.382	0.624	0.659
Tan	20	31151101	31151400	1.781	1.171	1.358	1.552
Tan	20	31787701	31788000	1.197	0.891	0.486	0.304
Tan	20	31833901	31834200	0.555	0.165	0.502	0.553
Tan	20	32058901	32059200	1.921	0.955	1.772	2.252
Tan	20	32616001	32616300	0.158	0.517	0.239	0.248
Tan	20	33103201	33103500	0.347	0.538	0.317	0.198
Tan	20	33810601	33810900	0.160	0.493	0.103	0.299
Tan	20	33936301	33936600	0.611	1.500	1.008	0.801
Tan	20	34877101	34877400	0.823	1.517	0.506	1.141
Tan	20	35260801	35261100	0.132	0.468	0.160	0.248
Tan	20	35905501	35905800	0.398	0.491	0.399	0.274
Tan	20	36186301	36186600	0.318	0.938	0.361	0.446
Tan	20	36279301	36279600	0.161	0.614	0.054	0.162
Tan	20	36377701	36378000	0.426	0.605	0.054	0.304
Tan	20	36474301	36474600	0.690	0.330	0.765	0.720
Tan	20	36477901	36478200	0.187	0.699	0.481	0.411
Tan	20	36700201	36700500	0.184	0.541	0.132	0.106
Tan	20	36752401	36752700	0.482	1.219	0.432	0.268

Tan	20	36880801	36881100	0.319	0.725	0.103	0.370
Tan	20	37034101	37034400	4.410	2.959	5.043	4.793
Tan	20	37288501	37288800	0.213	0.446	0.132	0.106
Tan	20	37289101	37289400	0.266	0.539	0.160	0.137
Tan	20	37524001	37524300	0.160	0.470	0.107	0.279
Tan	20	38062201	38062500	0.695	1.956	0.419	0.805
Tan	20	38343301	38343600	0.611	0.820	0.181	0.527
Tan	20	38445901	38446200	0.398	0.631	0.321	0.243
Tan	20	38484901	38485200	0.265	0.491	0.263	0.608
Tan	20	38646901	38647200	0.715	1.869	0.954	1.055
Tan	20	38728501	38728800	2.410	4.420	3.110	3.220
Tan	20	38736901	38737200	0.349	1.476	0.424	0.781
Tan	20	38776501	38776800	0.239	0.845	0.453	0.157
Tan	20	38951401	38951700	0.294	0.611	0.243	0.248
Tan	20	39093601	39093900	0.476	0.796	0.395	0.385
Tan	20	39458401	39458700	0.239	0.708	0.025	0.198
Tan	20	39459001	39459300	0.426	0.959	0.210	0.385
Tan	20	39591001	39591300	2.763	4.547	2.990	2.939
Tan	20	40143601	40143900	0.263	0.702	0.292	0.299
Tan	20	40301101	40301400	0.132	0.609	0.296	0.335
Tan	20	40503901	40504200	2.396	2.780	1.806	2.571
Tan	20	40533901	40534200	0.373	0.633	0.156	0.198
Tan	20	40550401	40550700	0.881	2.060	0.901	0.857
Tan	20	40648801	40649100	0.186	0.446	0.210	0.187
Tan	20	40721401	40721700	1.060	1.878	0.856	0.877
Tan	20	40812901	40813200	0.081	0.371	0.103	0.081
Tan	20	40902901	40903200	0.052	0.519	0.136	0.061
Tan	20	40944601	40944900	1.108	2.041	1.197	0.355
Tan	20	40956301	40956600	0.215	0.491	0.160	0.173
Tan	20	40966501	40966800	0.323	0.818	0.296	0.182
Tan	20	41065201	41065500	0.479	0.959	0.461	0.507
Tan	20	41115301	41115600	0.081	0.466	0.234	0.137
Tan	20	41228101	41228400	1.271	2.819	1.316	1.384
Tan	20	41335501	41335800	0.560	0.936	0.473	0.563
Tan	20	41413801	41414100	0.132	0.539	0.160	0.304
Tan	20	41520001	41520300	3.004	1.238	2.028	2.099
Tan	20	41575501	41575800	0.634	1.897	0.506	0.669
Tan	20	41742001	41742300	0.507	0.725	0.210	0.335
Tan	20	41763601	41763900	0.852	1.710	0.938	0.350
Tan	20	41862301	41862600	0.718	1.403	0.408	0.533
Tan	20	41950501	41950800	0.295	0.678	0.317	0.416
Tan	20	42086701	42087000	0.532	0.938	0.341	0.370
Tan	20	42089401	42089700	0.265	0.729	0.214	0.274
Tan	20	42136501	42136800	0.423	0.817	0.510	0.497
Tan	20	42170701	42171000	0.557	0.914	0.584	0.492
Tan	20	42550201	42550500	0.187	0.468	0.218	0.137
Tan	20	42591601	42591900	0.374	0.678	0.185	0.355
Tan	20	42656401	42656700	0.398	1.450	0.604	0.705
Tan	20	42852001	42852300	0.453	0.749	0.317	0.452
Tan	20	42888301	42888600	0.428	0.702	0.403	0.360
Tan	20	43059301	43059600	0.240	0.442	0.185	0.167
Tan	20	43103701	43104000	0.211	0.489	0.267	0.000
Tan	20	43266601	43266900	0.266	0.659	0.267	0.167
Tan	20	43381501	43381800	0.740	1.450	0.749	0.684
Tan	20	43399501	43399800	1.536	3.450	2.138	1.764
Tan	20	43478101	43478400	0.428	1.056	0.457	0.517
Tan	20	43642801	43643100	2.099	1.171	1.988	2.367
Tan	20	43855501	43855800	0.426	0.699	0.160	0.391
Tan	20	44042401	44042700	1.010	2.300	1.337	1.410
Tan	20	44289601	44289900	0.160	0.584	0.160	0.243
Tan	20	44481601	44481900	0.239	1.249	0.424	0.391
Tan	20	44529001	44529300	1.149	3.012	1.518	1.998
Tan	20	44615701	44616000	0.105	0.704	0.292	0.385
Tan	20	44856001	44856300	0.213	0.493	0.078	0.248
Tan	20	44977501	44977800	1.197	2.549	0.999	1.647
Tan	20	45432901	45433200	0.160	0.566	0.210	0.142
Tan	20	45645601	45645900	0.292	0.995	2.546	1.633

Tan	20	45698701	45699000	0.134	0.470	0.107	0.218
Tan	20	45815401	45815700	0.265	0.489	0.054	0.167
Tan	20	45874801	45875100	2.928	1.641	2.620	2.667
Tan	20	45875101	45875400	3.089	1.611	2.499	2.459
Tan	20	45967501	45967800	1.171	2.116	1.181	1.035
Tan	20	45984301	45984600	3.591	5.677	2.899	2.316
Tan	20	46009501	46009800	0.161	0.534	0.087	0.025
Tan	20	46012201	46012500	0.502	1.002	0.312	0.329
Tan	20	46216801	46217100	2.202	3.341	2.592	4.077
Tan	20	46239301	46239600	2.854	1.051	3.426	2.986
Tan	20	46239601	46239900	3.065	0.425	3.742	2.863
Tan	20	46280401	46280700	1.329	2.081	1.308	1.019
Tan	20	46645501	46645800	0.105	0.444	0.107	0.157
Tan	20	46742701	46743000	0.108	0.744	0.205	0.106
Tan	20	46759201	46759500	0.269	0.775	0.259	0.299
Tan	20	46781401	46781700	0.639	1.028	0.983	0.608
Tan	20	46838701	46839000	0.105	0.446	0.214	0.279
Tan	20	47094301	47094600	0.639	1.290	0.711	0.852
Tan	20	47208601	47208900	0.266	0.974	0.198	0.198
Tan	20	47458801	47459100	0.477	0.845	0.448	0.324
Tan	20	47467801	47468100	2.037	3.480	2.428	2.155
Tan	20	47684701	47685000	0.745	1.536	0.843	0.771
Tan	20	47708701	47709000	1.515	3.062	2.280	1.308
Tan	20	47781901	47782200	0.505	0.257	0.535	0.907
Tan	20	47803801	47804100	0.955	1.667	0.740	0.736
Tan	20	47910901	47911200	0.211	0.423	0.160	0.112
Tan	20	47983501	47983800	5.077	1.122	4.746	5.378
Tan	20	48010501	48010800	1.783	1.028	1.543	1.455
Tan	20	48145801	48146100	0.613	1.172	0.597	0.522
Tan	20	48174001	48174300	3.068	5.020	2.919	3.443
Tan	20	48180301	48180600	1.034	1.878	0.453	0.792
Tan	20	48195601	48195900	1.650	0.918	1.694	1.480
Tan	20	48223501	48223800	2.652	4.579	3.462	2.946
Tan	20	48279901	48280200	3.807	1.371	2.898	3.407
Tan	20	48315601	48315900	0.294	0.444	0.210	0.081
Tan	20	48367201	48367500	0.290	0.798	0.395	0.360
Tan	20	48587401	48587700	1.783	1.019	2.410	1.967
Tan	20	48618601	48618900	0.503	0.794	0.453	0.968
Tan	20	48709201	48709500	0.052	0.420	0.189	0.137
Tan	20	48772201	48772500	0.531	1.077	0.769	0.720
Tan	20	48861601	48861900	2.394	3.605	2.131	2.500
Tan	20	49031701	49032000	0.161	0.729	0.234	0.284
Tan	20	49032301	49032600	0.823	0.656	0.905	1.328
Tan	20	49176901	49177200	0.636	1.075	0.506	0.781
Tan	20	49220701	49221000	0.929	1.261	0.802	1.521
Tan	20	49328701	49329000	3.109	1.266	3.197	3.078
Tan	20	49358701	49359000	1.300	0.483	1.291	1.521
Tan	20	49485601	49485900	0.316	0.841	0.374	0.421
Tan	20	49494901	49495200	1.276	2.800	1.451	0.796
Tan	20	49530301	49530600	1.468	3.635	1.883	1.029
Tan	20	49530601	49530900	2.367	3.884	2.548	1.343
Tan	20	49542601	49542900	0.955	1.785	0.350	1.080
Tan	20	49559401	49559700	0.187	0.938	0.346	0.319
Tan	20	49732501	49732800	5.580	4.388	7.640	5.187
Tan	20	49803601	49803900	1.700	1.446	1.061	0.913
Tan	20	49876201	49876500	3.641	3.205	4.151	3.767
Tan	20	50025901	50026200	1.516	2.126	2.595	1.638
Tan	20	50124301	50124600	2.284	2.974	2.082	1.566
Tan	20	50196001	50196300	0.587	2.193	0.493	0.461
Tan	20	50312101	50312400	0.347	0.605	0.288	0.329
Tan	20	50370301	50370600	0.928	1.622	0.852	0.963
Tan	20	50506801	50507100	0.186	0.515	0.239	0.112
Tan	20	50976901	50977200	1.278	0.539	1.268	1.425
Tan	20	51050401	51050700	0.928	1.523	0.687	0.897
Tan	20	51055801	51056100	0.481	0.161	0.457	0.608
Tan	20	51132901	51133200	0.134	0.395	0.054	0.031
Tan	21	29101	29400	4.589	2.991	4.304	4.542

Tan	21	138601	138900	0.027	0.446	0.054	0.279
Tan	21	332101	332400	0.845	1.656	0.938	0.664
Tan	21	847501	847800	1.465	1.777	1.300	0.882
Tan	21	916501	916800	0.079	0.423	0.160	0.223
Tan	21	1130401	1130700	0.852	1.382	0.716	0.923
Tan	21	1621501	1621800	0.215	0.536	0.239	0.167
Tan	21	1702201	1702500	2.920	1.822	2.777	2.632
Tan	21	1814101	1814400	1.305	3.131	1.545	1.222
Tan	21	1857301	1857600	0.266	0.541	0.214	0.162
Tan	21	2222101	2222400	0.053	0.449	0.054	0.025
Tan	21	2426701	2427000	0.453	0.208	0.531	0.547
Tan	21	4356601	4356900	0.106	0.583	0.054	0.106
Tan	21	4779601	4779900	0.639	0.399	0.214	0.248
Tan	21	5249701	5250000	0.213	0.420	0.082	0.274
Tan	21	5451301	5451600	0.132	0.303	0.189	0.608
Tan	21	5578201	5578500	0.216	0.674	0.288	0.193
Tan	21	5596201	5596500	2.600	3.962	2.221	2.394
Tan	21	5655301	5655600	0.421	0.933	0.370	0.238
Tan	21	5955301	5955600	0.373	0.562	0.234	0.274
Tan	21	6123901	6124200	0.079	2.519	1.145	0.993
Tan	21	6138301	6138600	0.292	0.562	0.346	0.198
Tan	21	6243301	6243600	0.694	1.167	0.214	0.648
Tan	21	6516001	6516300	0.910	2.452	1.529	0.437
Tan	21	6606601	6606900	0.373	0.654	0.370	0.268
Tan	21	6623401	6623700	1.812	1.216	1.736	1.993
Tan	21	6687301	6687600	0.052	0.446	0.074	0.051
Tan	21	6709201	6709500	0.344	0.770	0.132	0.076
Tan	21	6709501	6709800	0.368	1.465	0.230	0.076
Tan	21	6932401	6932700	0.131	0.446	0.103	0.106
Tan	21	7030501	7030800	3.270	1.037	2.979	3.215
Tan	21	7030801	7031100	3.036	1.154	3.037	3.114
Tan	21	7093801	7094100	0.131	0.626	0.000	0.025
Tan	21	7094101	7094400	0.319	0.747	0.098	0.187
Tan	21	7396201	7396500	0.479	1.053	0.724	0.603
Tan	21	7484701	7485000	0.265	1.176	0.613	0.639
Tan	21	7518901	7519200	3.567	5.321	2.923	3.523
Tan	21	7788001	7788300	0.132	0.725	0.210	0.198
Tan	21	7790401	7790700	0.106	1.146	0.082	0.147
Tan	21	7790701	7791000	0.156	1.009	0.074	0.142
Tan	21	8064301	8064600	0.316	0.846	0.243	0.385
Tan	21	8080801	8081100	0.773	1.755	0.967	1.247
Tan	21	8098501	8098800	2.208	3.094	2.278	2.373
Tan	21	8115901	8116200	0.481	0.865	0.502	0.497
Tan	21	8120101	8120400	2.047	1.659	2.492	2.115
Tan	21	8167501	8167800	0.587	1.833	0.390	1.039
Tan	21	8239201	8239500	0.266	0.586	0.259	0.198
Tan	21	8241001	8241300	0.450	0.702	0.457	0.385
Tan	21	8354701	8355000	0.423	1.030	0.444	0.405
Tan	21	8403001	8403300	0.428	0.912	0.350	0.664
Tan	21	8403301	8403600	0.211	0.749	0.103	0.583
Tan	21	8413801	8414100	3.612	2.643	3.619	4.208
Tan	21	8457301	8457600	0.000	0.401	0.103	0.106
Tan	21	8550601	8550900	5.369	3.252	5.613	5.396
Tan	21	8550901	8551200	3.212	1.693	3.312	3.317
Tan	21	8996101	8996400	0.749	1.257	0.555	0.436
Tan	21	9061501	9061800	2.770	3.418	2.115	2.119
Tan	21	9079801	9080100	0.268	0.983	0.321	0.513
Tan	21	9098701	9099000	0.345	1.176	0.185	0.142
Tan	21	9123901	9124200	0.182	0.938	0.230	0.274
Tan	21	9700801	9701100	0.292	0.187	0.346	0.497
Tan	21	9728101	9728400	0.213	0.586	0.243	0.193
Tan	21	9843901	9844200	0.184	0.725	0.424	0.198
Tan	21	9936601	9936900	0.215	0.753	0.263	0.193
Tan	21	10052401	10052700	0.982	2.137	0.671	0.988
Tan	21	10196401	10196700	0.481	1.622	0.863	0.725
Tan	21	10209601	10209900	0.239	2.029	0.865	0.954
Tan	21	10400701	10401000	0.871	1.614	0.963	0.614

Tan	21	10610101	10610400	0.347	0.118	0.560	0.355
Tan	21	10698001	10698300	0.319	0.725	0.272	0.405
Tan	21	10746301	10746600	0.158	0.491	0.058	0.137
Tan	21	11181901	11182200	0.239	0.612	0.189	0.228
Tan	21	11194501	11194800	0.266	0.586	0.214	0.441
Tan	21	11226301	11226600	0.695	1.360	0.736	0.648
Tan	21	11249401	11249700	0.779	2.364	0.888	0.518
Tan	21	11249701	11250000	0.561	2.382	0.747	0.426
Tan	21	11540101	11540400	0.560	0.908	0.535	0.117
Tan	21	11592601	11592900	0.876	1.592	0.847	1.111
Tan	21	11694301	11694600	5.516	2.967	5.717	6.500
Tan	21	11694601	11694900	2.528	1.285	2.934	2.708
Tan	21	11757001	11757300	0.026	0.539	0.049	0.137
Tan	21	11795701	11796000	0.374	1.053	0.466	0.294
Tan	21	11805601	11805900	1.115	2.879	2.066	1.115
Tan	21	11905201	11905500	1.302	2.736	1.544	1.480
Tan	21	11905501	11905800	1.513	2.768	1.551	1.642
Tan	21	12063601	12063900	0.161	0.444	0.054	0.254
Tan	21	12366601	12366900	0.053	0.470	0.103	0.056
Tan	21	12582901	12583200	0.187	0.556	0.000	0.142
Tan	21	12642901	12643200	0.563	2.032	0.624	0.678
Tan	21	12643801	12644100	0.055	0.493	0.185	0.254
Tan	21	12691501	12691800	2.579	0.831	2.927	2.863
Tan	21	12691801	12692100	4.341	1.015	5.135	4.917
Tan	21	12845101	12845400	0.211	1.729	0.214	1.171
Tan	21	13425601	13425900	0.189	0.468	0.078	0.187
Tan	21	13639201	13639500	0.052	0.395	0.107	0.167
Tan	21	13640401	13640700	0.374	0.772	0.350	0.324
Tan	21	13693201	13693500	0.292	0.114	0.798	0.385
Tan	21	13764601	13764900	0.290	0.560	0.292	0.299
Tan	21	13764901	13765200	0.292	0.674	0.325	0.243
Tan	21	13971301	13971600	0.610	2.081	1.518	1.232
Tan	21	14001601	14001900	3.854	0.116	1.658	4.154
Tan	21	14001901	14002200	3.615	0.139	1.741	4.454
Tan	21	14196901	14197200	0.610	0.279	0.580	0.751
Tan	21	14290801	14291100	0.160	0.515	0.239	0.335
Tan	21	14390401	14390700	0.852	1.800	0.609	0.745
Tan	21	14474701	14475000	0.932	1.525	0.785	0.846
Tan	21	14697601	14697900	0.077	0.515	0.082	0.137
Tan	21	14871301	14871600	0.211	0.708	0.082	0.167
Tan	21	14905201	14905500	0.424	0.910	0.267	0.542
Tan	21	15121201	15121500	0.184	0.612	0.054	0.132
Tan	21	15129901	15130200	0.265	1.103	0.337	0.669
Tan	21	15397801	15398100	0.395	0.980	0.403	0.238
Tan	21	15398101	15398400	0.235	0.914	0.074	0.162
Tan	21	15841501	15841800	0.371	0.515	0.979	0.720
Tan	21	16018501	16018800	2.444	2.944	2.403	2.495
Tan	21	16033201	16033500	0.160	1.172	0.185	0.315
Tan	21	16033501	16033800	0.079	1.214	0.074	0.618
Tan	21	16186201	16186500	1.249	0.446	1.407	0.725
Tan	21	16199701	16200000	0.239	0.560	0.350	0.279
Tan	21	16216201	16216500	0.581	1.442	0.740	0.497
Tan	21	16276201	16276500	0.477	1.405	0.526	0.421
Tan	21	16347901	16348200	1.521	2.291	1.086	0.994
Tan	21	16447501	16447800	1.665	3.907	1.073	1.293
Tan	21	16447801	16448100	1.533	3.358	0.941	1.070
Tan	21	16449601	16449900	2.521	0.941	2.468	3.058
Tan	21	16458301	16458600	0.828	1.422	0.745	0.659
Tan	21	16458601	16458900	0.750	1.832	0.626	0.588
Tan	21	16501201	16501500	0.187	0.562	0.160	0.137
Tan	21	16509601	16509900	0.481	0.116	0.399	0.416
Tan	21	16564501	16564800	0.902	1.500	0.769	1.060
Tan	21	16797601	16797900	0.508	0.234	0.531	0.608
Tan	21	16799101	16799400	0.211	0.674	0.165	0.187
Tan	21	16801801	16802100	0.797	0.118	1.061	0.861
Tan	21	16802401	16802700	1.086	0.238	1.264	0.913
Tan	21	16823401	16823700	0.158	0.794	0.263	0.162

Tan	21	16840501	16840800	0.244	1.011	0.254	0.238
Tan	21	16891501	16891800	0.587	1.098	0.551	0.335
Tan	21	16919101	16919400	0.240	0.586	0.185	0.223
Tan	21	17070001	17070300	0.531	1.234	0.279	0.563
Tan	21	17070901	17071200	0.242	0.813	0.205	0.025
Tan	21	17119201	17119500	1.749	4.025	1.398	1.597
Tan	21	17135101	17135400	0.265	0.493	0.292	0.223
Tan	21	17337001	17337300	0.053	0.466	0.107	0.081
Tan	21	17435701	17436000	1.568	0.983	2.134	2.277
Tan	21	17588101	17588400	2.584	3.371	2.617	2.591
Tan	21	17776501	17776800	0.374	0.796	0.234	0.370
Tan	21	17827801	17828100	0.132	0.515	0.239	0.248
Tan	21	17944501	17944800	0.134	1.478	0.210	0.340
Tan	21	17946901	17947200	0.052	0.442	0.165	0.137
Tan	21	18060001	18060300	0.239	0.729	0.078	0.137
Tan	21	18347401	18347700	0.265	0.603	0.185	0.198
Tan	21	18573901	18574200	0.984	0.069	0.613	0.299
Tan	21	18897301	18897600	0.026	0.491	0.107	0.056
Tan	21	18932701	18933000	0.106	0.258	0.609	0.360
Tan	21	18952801	18953100	0.742	0.352	0.695	0.781
Tan	21	18966001	18966300	0.294	0.680	0.276	0.167
Tan	21	19621501	19621800	0.136	0.442	0.185	0.112
Tan	21	20205001	20205300	0.187	0.564	0.103	0.228
Tan	21	20602201	20602500	0.132	0.884	0.292	0.315
Tan	21	20604901	20605200	0.268	0.605	0.267	0.137
Tan	21	20665501	20665800	0.345	0.869	0.243	0.132
Tan	21	20785201	20785500	0.239	0.491	0.234	0.304
Tan	21	21226501	21226800	0.187	0.470	0.054	0.167
Tan	21	21345001	21345300	0.055	0.538	0.317	0.173
Tan	21	21404701	21405000	0.771	0.609	0.243	0.304
Tan	21	21917101	21917400	0.560	0.303	0.185	0.664
Tan	21	22040101	22040400	0.161	0.560	0.107	0.218
Tan	21	22515601	22515900	6.549	1.786	11.404	5.988
Tan	21	22515901	22516200	2.520	0.745	3.859	2.381
Tan	21	22588201	22588500	0.263	1.026	0.049	0.268
Tan	21	22588501	22588800	0.210	1.075	0.074	0.187
Tan	21	22680301	22680600	0.982	1.195	0.506	0.416
Tan	21	22680601	22680900	1.204	1.845	0.778	0.608
Tan	21	23276101	23276400	0.952	1.450	0.934	0.796
Tan	21	23605801	23606100	0.452	0.893	0.535	0.472
Tan	21	23613001	23613300	0.106	0.496	0.103	0.112
Tan	21	23627401	23627700	0.294	0.918	0.361	0.309
Tan	21	23627701	23628000	0.268	0.822	0.239	0.238
Tan	21	23633701	23634000	0.936	2.069	0.818	0.974
Tan	21	23726701	23727000	0.505	0.584	0.267	0.360
Tan	21	23790901	23791200	0.189	0.468	0.165	0.167
Tan	21	23889301	23889600	0.720	1.358	0.604	0.700
Tan	21	23889601	23889900	0.908	1.871	0.892	1.217
Tan	21	23953501	23953800	0.477	1.174	0.555	0.436
Tan	21	23967301	23967600	1.086	1.991	0.776	0.812
Tan	21	24123301	24123600	0.373	0.584	0.214	0.137
Tan	21	24153601	24153900	0.878	1.126	0.473	0.720
Tan	21	24335101	24335400	0.352	1.193	0.544	0.558
Tan	21	24397201	24397500	0.160	0.466	0.185	0.173
Tan	21	24606001	24606300	0.661	1.216	0.629	0.765
Tan	21	24712801	24713100	0.292	0.468	0.267	0.279
Tan	21	24775201	24775500	0.718	1.030	0.350	0.598
Tan	21	24896401	24896700	0.160	0.513	0.116	0.162
Tan	21	24990001	24990300	0.508	1.480	0.934	0.832
Tan	21	25004401	25004700	0.955	1.613	1.304	0.157
Tan	21	25021201	25021500	0.187	0.753	0.214	0.274
Tan	21	25257301	25257600	0.561	1.028	0.497	0.507
Tan	21	25335301	25335600	7.958	6.284	7.859	8.523
Tan	21	25629601	25629900	0.502	1.199	0.580	0.664
Tan	21	25734901	25735200	0.589	1.216	0.716	0.583
Tan	21	26491201	26491500	0.160	0.423	0.181	0.086
Tan	21	27098101	27098400	0.321	1.077	0.539	0.268

Tan	21	27114301	27114600	0.932	1.759	0.843	0.674
Tan	21	27352801	27353100	2.420	1.448	1.674	1.688
Tan	21	27395401	27395700	0.400	0.727	0.481	0.497
Tan	21	27477301	27477600	0.503	0.843	0.448	0.436
Tan	21	27477601	27477900	0.715	1.054	0.555	0.664
Tan	21	27643801	27644100	1.996	1.210	1.801	1.840
Tan	21	27761401	27761700	2.142	3.954	1.904	1.958
Tan	21	27830401	27830700	1.408	0.352	1.242	1.135
Tan	21	27978901	27979200	0.136	1.776	0.823	0.025
Tan	21	27979501	27979800	1.276	0.938	1.217	1.440
Tan	21	27992401	27992700	1.011	1.240	0.749	0.725
Tan	21	28001401	28001700	0.235	1.435	0.275	0.173
Tan	21	28001701	28002000	0.737	2.959	0.687	0.720
Tan	21	28012201	28012500	0.455	0.841	0.428	0.578
Tan	21	28088401	28088700	0.160	0.656	0.210	0.259
Tan	21	28285201	28285500	0.266	0.420	0.317	0.167
Tan	21	28742401	28742700	0.716	1.822	0.702	0.761
Tan	21	28775401	28775700	0.428	0.841	0.160	0.396
Tan	21	28789501	28789800	0.134	0.702	0.181	0.360
Tan	21	28789801	28790100	0.797	2.163	1.099	0.684
Tan	21	28791001	28791300	8.417	5.910	9.026	9.664
Tan	21	29016601	29016900	0.186	0.654	0.502	0.502
Tan	21	29058901	29059200	0.421	1.956	0.225	0.579
Tan	21	29111101	29111400	0.720	1.146	0.419	0.614
Tan	21	29439301	29439600	1.305	0.167	1.197	1.480
Tan	21	29516101	29516400	0.747	0.393	1.037	1.019
Tan	21	29673301	29673600	0.266	0.539	0.239	0.740
Tan	21	29771101	29771400	0.079	0.562	0.210	0.198
Tan	21	30303901	30304200	0.055	0.558	0.123	0.061
Tan	21	30909001	30909300	0.345	0.725	0.312	0.218
Tan	21	30926401	30926700	0.319	0.491	0.346	0.223
Tan	21	31129801	31130100	0.160	0.493	0.189	0.193
Tan	21	31245901	31246200	1.971	3.000	1.768	1.729
Tan	21	31246201	31246500	1.415	2.100	1.152	1.151
Tan	21	31249201	31249500	0.349	1.493	0.218	0.400
Tan	21	31383901	31384200	2.159	3.059	1.527	2.408
Tan	21	31410901	31411200	1.063	1.568	0.778	0.690
Tan	21	31436401	31436700	0.000	0.489	0.205	0.031
Tan	21	31543201	31543500	0.292	0.536	0.350	0.167
Tan	21	32205001	32205300	0.398	0.775	0.354	0.248
Tan	21	32540701	32541000	0.213	0.328	0.210	0.522
Tan	21	32599501	32599800	0.344	0.631	0.185	0.355
Tan	21	32612701	32613000	0.934	0.204	0.914	1.060
Tan	21	32658001	32658300	0.682	2.201	0.921	0.695
Tan	21	32797501	32797800	0.261	0.839	0.272	0.142
Tan	21	32800501	32800800	0.158	0.607	0.165	0.086
Tan	21	32914201	32914500	0.266	0.633	0.263	0.157
Tan	21	33040801	33041100	0.319	0.560	0.346	0.243
Tan	21	33080401	33080700	1.491	2.761	3.208	2.008
Tan	21	33090901	33091200	1.358	1.890	0.987	0.877
Tan	21	33131401	33131700	0.160	0.586	0.058	0.274
Tan	21	33353401	33353700	0.508	0.914	0.259	0.299
Tan	21	33446101	33446400	0.187	0.466	0.140	0.162
Tan	21	33469201	33469500	0.239	0.468	0.263	0.304
Tan	21	33560401	33560700	0.681	2.124	0.470	0.892
Tan	21	33650701	33651000	0.213	0.494	0.136	0.218
Tan	21	33785701	33786000	4.678	5.424	3.221	3.322
Tan	21	33972001	33972300	1.010	0.206	0.823	0.634
Tan	21	34049401	34049700	0.110	0.465	0.103	0.112
Tan	21	34062901	34063200	0.534	1.189	0.546	0.370
Tan	21	34063801	34064100	0.373	0.676	0.424	0.441
Tan	21	34089601	34089900	1.728	0.863	1.206	1.374
Tan	21	34142401	34142700	0.187	0.562	0.317	0.324
Tan	21	34221001	34221300	0.692	1.028	0.638	0.517
Tan	21	34233301	34233600	1.197	1.051	0.769	0.771
Tan	21	34256401	34256700	0.398	0.185	0.370	0.583
Tan	21	34422601	34422900	0.345	0.609	0.428	0.360

Tan	21	34439701	34440000	2.341	0.708	1.955	2.676
Tan	21	34446901	34447200	0.321	0.654	0.259	0.335
Tan	21	34454401	34454700	5.901	3.175	5.836	5.818
Tan	21	34602601	34602900	2.607	1.687	1.152	2.475
Tan	21	34617301	34617600	0.692	1.422	0.876	0.974
Tan	21	34714501	34714800	1.065	1.663	0.646	0.821
Tan	21	34821001	34821300	2.715	1.641	2.769	2.449
Tan	21	34906801	34907100	1.479	4.650	1.209	1.460
Tan	21	34965001	34965300	0.665	0.891	0.638	0.664
Tan	21	35063101	35063400	0.132	0.472	0.136	0.061
Tan	21	35098801	35099100	0.239	1.054	0.078	0.644
Tan	21	35209201	35209500	0.235	0.654	0.263	0.299
Tan	21	35239501	35239800	0.424	0.680	0.160	0.304
Tan	21	35259301	35259600	0.376	1.188	0.412	0.583
Tan	21	35271901	35272200	2.565	4.832	1.765	3.645
Tan	21	35776501	35776800	2.557	1.824	2.428	2.378
Tan	21	35997001	35997300	0.319	0.702	0.321	0.254
Tan	21	36013501	36013800	0.318	0.165	0.531	0.466
Tan	21	36024601	36024900	0.321	0.515	0.185	0.223
Tan	21	36034501	36034800	0.561	1.845	0.740	0.683
Tan	21	36162301	36162600	0.371	0.491	0.374	0.193
Tan	21	36293701	36294000	0.079	0.378	0.078	0.000
Tan	21	36690601	36690900	0.507	1.120	0.604	0.481
Tan	21	36753601	36753900	0.242	0.848	0.082	0.132
Tan	21	36906901	36907200	0.081	0.564	0.185	0.254
Tan	21	36912301	36912600	0.237	0.680	0.218	0.162
Tan	21	37980901	37981200	0.373	0.493	0.132	0.360
Tan	21	38373901	38374200	0.398	0.910	0.078	0.497
Tan	21	38702401	38702700	0.694	0.981	0.555	0.578
Tan	21	39021601	39021900	2.418	2.075	2.818	1.430
Tan	21	39125101	39125400	0.134	0.493	0.082	0.106
Tan	21	39184501	39184800	0.290	0.564	0.189	0.112
Tan	21	39238801	39239100	0.079	0.586	0.239	0.268
Tan	21	39243601	39243900	0.823	1.403	0.909	0.983
Tan	21	39379501	39379800	0.371	0.837	0.424	0.223
Tan	21	39609901	39610200	0.905	0.491	0.691	0.801
Tan	21	39626401	39626700	2.609	1.287	1.839	1.577
Tan	21	39691801	39692100	0.532	1.174	0.399	0.700
Tan	21	39730801	39731100	0.879	1.053	0.687	0.801
Tan	21	39787501	39787800	3.299	2.410	3.221	3.179
Tan	21	39961801	39962100	0.239	0.446	0.185	0.198
Tan	21	40204201	40204500	0.290	0.652	0.160	0.360
Tan	21	40229101	40229400	0.373	0.918	0.395	0.472
Tan	21	40310101	40310400	3.194	2.705	2.040	1.688
Tan	21	40463101	40463400	0.452	0.772	0.395	0.461
Tan	21	40564501	40564800	0.136	0.493	0.210	0.142
Tan	21	40637101	40637400	0.266	0.633	0.301	0.193
Tan	21	41147701	41148000	0.240	0.749	0.477	0.477
Tan	21	41306401	41306700	0.131	0.444	0.025	0.223
Tan	21	41388901	41389200	0.347	1.009	0.234	0.234
Tan	21	41501401	41501700	1.039	1.687	0.745	1.060
Tan	21	41508001	41508300	0.560	0.792	0.555	0.391
Tan	21	41524801	41525100	2.636	3.808	1.921	2.175
Tan	21	41557501	41557800	2.179	2.693	2.164	2.043
Tan	21	41581801	41582100	2.315	1.485	2.588	2.297
Tan	21	41591701	41592000	0.823	1.171	0.662	0.563
Tan	21	41655301	41655600	1.357	1.073	0.872	0.832
Tan	21	41681701	41682000	0.665	0.936	0.560	0.669
Tan	21	41924101	41924400	1.700	1.937	1.115	1.627
Tan	21	42025801	42026100	0.953	1.637	1.135	0.776
Tan	21	42127801	42128100	1.215	0.442	1.222	1.496
Tan	21	42519601	42519900	2.510	4.418	2.156	2.525
Tan	21	42536401	42536700	0.345	0.633	0.078	0.081
Tan	21	42549601	42549900	3.601	5.027	2.138	3.720
Tan	21	42564001	42564300	0.158	0.466	0.185	0.279
Tan	21	42580201	42580500	1.973	0.997	3.429	3.280
Tan	21	42691201	42691500	0.345	0.444	0.132	0.274

Tan	21	42691501	42691800	0.077	0.517	0.160	0.167
Tan	21	42730801	42731100	0.344	0.513	0.132	0.304
Tan	21	42760501	42760800	1.352	2.343	0.602	0.772
Tan	21	42802801	42803100	0.240	0.420	0.160	0.112
Tan	21	42938101	42938400	0.720	1.032	0.506	0.411
Tan	21	42945901	42946200	3.025	1.496	2.448	2.520
Tan	21	43092301	43092600	0.453	0.891	0.448	0.446
Tan	21	43092601	43092900	0.450	0.792	0.403	0.360
Tan	21	43417201	43417500	0.963	1.833	0.745	0.583
Tan	21	43417501	43417800	0.508	1.032	0.486	0.259
Tan	21	43483501	43483800	2.579	1.686	0.802	1.177
Tan	21	43560901	43561200	0.637	1.873	1.324	1.009
Tan	21	43722001	43722300	1.041	0.347	0.934	1.060
Tan	21	43903501	43903800	5.169	3.248	4.204	4.264
Tan	21	43936501	43936800	0.106	0.751	0.136	0.385
Tan	21	43992901	43993200	0.400	0.798	0.267	0.553
Tan	21	44019901	44020200	0.345	0.702	0.428	0.248
Tan	21	44136001	44136300	2.033	0.672	2.127	1.892
Tan	21	44172601	44172900	0.318	0.652	0.267	0.365
Tan	21	44266201	44266500	0.292	0.444	0.210	0.248
Tan	21	44394301	44394600	0.423	1.101	0.551	0.380
Tan	21	44463901	44464200	0.584	0.772	0.210	0.477
Tan	21	44503201	44503500	0.347	0.817	0.403	0.268
Tan	21	44503501	44503800	0.239	0.719	0.160	0.304
Tan	21	44576701	44577000	1.061	2.393	0.769	1.060
Tan	21	44595301	44595600	1.386	0.907	1.522	1.435
Tan	21	44637901	44638200	0.878	1.234	0.720	0.882
Tan	21	44703601	44703900	0.400	0.770	0.477	0.421
Tan	21	44707501	44707800	0.052	0.423	0.074	0.112
Tan	21	44754001	44754300	0.450	0.959	0.526	0.603
Tan	21	44761501	44761800	5.417	3.487	5.223	4.922
Tan	21	44772001	44772300	2.528	1.049	2.889	1.992
Tan	21	44925901	44926200	0.161	0.513	0.205	0.218
Tan	21	45058801	45059100	0.213	0.468	0.132	0.335
Tan	21	45292501	45292800	0.848	2.086	1.282	1.100
Tan	21	45460801	45461100	0.507	1.193	0.729	0.751
Tan	21	45491401	45491700	1.710	3.025	1.458	1.445
Tan	21	45619201	45619500	1.836	1.101	1.534	1.541
Tan	21	45651001	45651300	0.400	0.955	0.695	0.385
Tan	21	45742201	45742500	1.223	1.706	1.102	1.161
Tan	21	45756601	45756900	5.288	2.720	5.148	5.079
Tan	21	45819001	45819300	2.105	3.334	2.283	1.932
Tan	21	45884101	45884400	0.268	0.680	0.234	0.167
Tan	21	46037401	46037700	0.426	0.584	0.317	0.411
Tan	21	46159501	46159800	2.123	2.710	1.904	2.033
Tan	21	46161901	46162200	0.613	0.959	0.667	0.553
Tan	21	46491301	46491600	2.964	1.549	2.706	3.067
Tan	21	46510201	46510500	5.954	4.158	5.835	5.775
Tan	21	46564801	46565100	0.400	1.011	0.457	0.391
Tan	21	46621201	46621500	11.583	6.496	9.145	9.517
Tan	21	46689901	46690200	0.581	1.906	0.816	0.751
Tan	21	46771201	46771500	1.710	2.673	1.715	1.582
Tan	21	46907701	46908000	0.239	0.770	0.539	0.304
Tan	21	46914901	46915200	1.518	2.058	0.945	1.511
Tan	21	46935601	46935900	4.788	3.444	2.780	5.760
Tan	21	46951801	46952100	1.887	0.880	1.830	1.471
Tan	21	47187601	47187900	5.686	3.349	5.727	5.582
Tan	21	47214001	47214300	0.398	0.796	0.205	0.466
Tan	21	47257201	47257500	0.505	0.375	0.531	0.578
Tan	21	47268601	47268900	2.128	2.761	2.336	2.033
Tan	21	47277601	47277900	3.160	4.576	3.072	3.048
Tan	21	47314801	47315100	0.774	1.744	0.789	0.882
Tan	21	47316301	47316600	6.122	4.153	5.604	5.811
Tan	21	47385301	47385600	0.266	0.817	0.510	0.385
Tan	21	47441701	47442000	0.529	1.727	0.535	1.111
Tan	21	47442001	47442300	0.369	1.427	0.720	0.715
Tan	21	47517001	47517300	3.186	2.088	3.039	3.534

Tan	21	47561101	47561400	0.477	0.281	0.160	0.167
Tan	21	47601001	47601300	3.246	1.757	2.374	2.921
Tan	21	47665801	47666100	1.092	1.611	0.807	1.181
Tan	21	47691601	47691900	0.132	0.607	0.210	0.274
Tan	21	47782801	47783100	0.215	0.835	0.243	0.324
Tan	21	47783101	47783400	1.299	2.263	1.583	1.485
Tan	21	47802901	47803200	0.000	1.236	0.058	0.000
Tan	21	48344401	48344700	0.481	1.101	0.613	0.832
Tan	21	48374101	48374400	0.958	2.422	0.967	1.110
Tan	21	48381001	48381300	0.213	0.515	0.107	0.162
Tan	21	48420301	48420600	0.321	0.699	0.098	0.274
Tan	21	48420601	48420900	1.305	1.639	0.185	1.262
Tan	21	48670501	48670800	0.984	1.869	0.765	1.029
Tan	21	48701701	48702000	2.078	0.794	1.534	1.399
Tan	21	48847501	48847800	0.106	0.446	0.160	0.056
Tan	21	48903901	48904200	10.402	5.414	8.531	8.916
Tan	21	49041001	49041300	0.134	0.468	0.160	0.147
Tan	21	49155601	49155900	9.781	7.091	11.416	9.203
Tan	21	49258801	49259100	7.116	3.191	7.554	8.403
Tan	21	49293001	49293300	11.246	5.765	9.815	11.497
Tan	21	49348501	49348800	0.718	1.452	1.099	2.048
Tan	21	49822801	49823100	1.439	1.075	1.781	1.688
Tan	21	49875001	49875300	0.373	0.676	0.263	0.304
Tan	22	1188301	1188600	1.299	4.474	2.348	2.311
Tan	22	1188601	1188900	1.620	3.165	1.271	1.925
Tan	22	1258201	1258500	0.213	0.350	0.555	0.198
Tan	22	1319101	1319400	0.576	1.526	0.025	0.187
Tan	22	1654801	1655100	0.424	0.699	0.160	0.335
Tan	22	1701901	1702200	1.460	0.843	1.197	1.303
Tan	22	2539201	2539500	0.266	0.846	0.049	0.396
Tan	22	3447301	3447600	0.027	0.446	0.049	0.279
Tan	22	3537601	3537900	0.531	0.212	0.374	0.472
Tan	22	4180801	4181100	0.186	0.564	0.107	0.061
Tan	22	4252501	4252800	0.907	1.940	2.419	0.771
Tan	22	4327801	4328100	0.505	0.328	0.531	0.725
Tan	22	4371301	4371600	0.268	0.749	0.098	0.324
Tan	22	4471801	4472100	0.053	0.448	0.000	0.218
Tan	22	5262301	5262600	0.239	0.515	0.156	0.167
Tan	22	5705101	5705400	0.292	0.609	0.317	0.411
Tan	22	5748601	5748900	0.136	0.633	0.181	0.147
Tan	22	6151201	6151500	0.371	0.727	0.296	0.299
Tan	22	6269101	6269400	0.215	0.652	0.136	0.193
Tan	22	6552601	6552900	0.156	0.723	0.185	0.203
Tan	22	6600901	6601200	1.116	0.912	1.037	1.303
Tan	22	6808801	6809100	2.705	7.879	3.205	3.231
Tan	22	6865201	6865500	0.237	1.710	0.074	1.115
Tan	22	7542301	7542600	0.371	0.586	0.321	0.254
Tan	22	8167501	8167800	0.400	0.966	0.234	0.380
Tan	22	8199901	8200200	0.295	0.701	0.210	0.315
Tan	22	8499001	8499300	0.369	1.262	0.584	0.812
Tan	22	8513401	8513700	0.431	1.405	0.428	0.502
Tan	22	8518501	8518800	0.215	0.538	0.259	0.137
Tan	22	8557501	8557800	0.421	1.259	0.691	0.517
Tan	22	8604601	8604900	0.347	0.682	0.263	0.355
Tan	22	8639101	8639400	0.161	0.468	0.140	0.193
Tan	22	8979901	8980200	0.369	0.910	0.638	0.608
Tan	22	9032701	9033000	2.789	4.390	3.112	2.645
Tan	22	9582601	9582900	0.344	0.657	0.370	0.304
Tan	22	9809401	9809700	0.000	0.468	0.132	0.122
Tan	22	9830401	9830700	0.640	0.069	0.263	0.248
Tan	22	9862801	9863100	0.373	0.586	0.132	0.081
Tan	22	9963001	9963300	0.452	0.560	0.321	0.167
Tan	22	10490701	10491000	0.290	0.699	0.263	0.391
Tan	22	10936801	10937100	0.557	0.961	0.428	0.497
Tan	22	10951201	10951500	0.132	0.652	0.263	0.162
Tan	22	11076601	11076900	0.240	1.495	0.856	1.080
Tan	22	11819701	11820000	0.957	1.613	1.139	0.867

Tan	22	12429901	12430200	0.398	3.103	0.734	0.228
Tan	22	12430201	12430500	0.208	2.038	0.406	0.234
Tan	22	12583501	12583800	0.321	1.084	0.448	0.223
Tan	22	12592801	12593100	0.265	0.777	0.078	0.335
Tan	22	12756901	12757200	0.263	0.835	0.243	0.223
Tan	22	13364401	13364700	0.189	0.539	0.107	0.167
Tan	22	13621501	13621800	0.082	0.567	0.078	0.086
Tan	22	13960501	13960800	0.263	0.845	0.223	0.279
Tan	22	13969201	13969500	0.239	0.470	0.189	0.218
Tan	22	14132401	14132700	6.441	3.952	5.955	7.655
Tan	22	14160901	14161200	1.678	2.111	0.695	1.055
Tan	22	14350201	14350500	0.294	0.515	0.111	0.274
Tan	22	14393701	14394000	0.239	0.446	0.214	0.086
Tan	22	14509801	14510100	0.345	0.656	0.234	0.329
Tan	22	14851801	14852100	0.292	0.916	0.448	0.193
Tan	22	15172801	15173100	0.400	1.058	0.481	0.385
Tan	22	15335401	15335700	1.174	1.878	1.032	1.024
Tan	22	15640501	15640800	0.237	0.444	0.160	0.112
Tan	22	15659701	15660000	0.211	0.491	0.107	0.142
Tan	22	15663901	15664200	0.428	0.794	0.613	0.553
Tan	22	15960901	15961200	0.134	0.494	0.136	0.162
Tan	22	16211101	16211400	0.134	0.281	0.160	0.522
Tan	22	16745101	16745400	0.265	0.588	0.156	0.304
Tan	22	17068501	17068800	0.795	1.349	0.823	0.603
Tan	22	17195101	17195400	1.515	2.393	1.197	1.400
Tan	22	17477101	17477400	0.186	0.444	0.078	0.274
Tan	22	17491501	17491800	0.745	1.053	0.428	0.796
Tan	22	17651101	17651400	2.560	3.201	2.200	2.266
Tan	22	17967601	17967900	1.650	2.337	1.333	1.577
Tan	22	18048301	18048600	0.665	1.476	0.863	0.806
Tan	22	18055801	18056100	0.319	0.704	0.132	0.360
Tan	22	18149401	18149700	1.037	0.773	1.275	1.004
Tan	22	18199501	18199800	0.773	0.538	1.066	0.806
Tan	22	18357001	18357300	0.690	0.584	0.317	0.329
Tan	22	18582001	18582300	0.639	0.163	0.390	0.385
Tan	22	18610501	18610800	0.534	0.794	0.531	0.446
Tan	22	19027201	19027500	0.077	0.539	0.189	0.142
Tan	22	19224901	19225200	0.000	0.494	0.049	0.056
Tan	22	19635901	19636200	1.221	1.968	0.682	1.227
Tan	22	19979701	19980000	0.240	0.723	0.350	0.304
Tan	22	19983901	19984200	1.539	3.073	1.050	1.303
Tan	22	20238601	20238900	0.160	0.513	0.288	0.329
Tan	22	20892001	20892300	0.345	0.609	0.317	0.913
Tan	22	20980801	20981100	0.213	0.493	0.054	0.173
Tan	22	21516301	21516600	0.318	0.491	0.136	0.274
Tan	22	21531901	21532200	0.982	1.448	0.992	0.882
Tan	22	21922501	21922800	1.334	0.865	1.835	2.079
Tan	22	21930901	21931200	1.089	0.794	1.222	0.938
Tan	22	22000201	22000500	0.347	0.723	0.370	0.396
Tan	22	22203901	22204200	1.223	1.309	1.037	1.161
Tan	22	22331401	22331700	0.053	0.449	0.078	0.056
Tan	22	22394101	22394400	3.139	2.594	2.102	1.825
Tan	22	22782901	22783200	0.026	0.423	0.160	0.193
Tan	22	22973701	22974000	0.187	0.517	0.181	0.173
Tan	22	23069701	23070000	0.242	0.564	0.234	0.167
Tan	22	23099101	23099400	0.932	1.609	0.687	1.019
Tan	22	23112601	23112900	0.342	1.833	0.330	0.751
Tan	22	23156701	23157000	1.274	2.229	1.322	0.624
Tan	22	23507101	23507400	0.373	0.538	0.239	0.391
Tan	22	23531101	23531400	2.844	4.364	2.679	2.673
Tan	22	23531401	23531700	3.065	4.860	3.181	2.972
Tan	22	23652901	23653200	0.268	0.538	0.185	0.274
Tan	22	23946901	23947200	0.823	1.313	0.695	0.628
Tan	22	28168201	28168500	0.081	0.470	0.160	0.112
Tan	22	28388401	28388700	0.398	0.633	0.350	0.304
Tan	22	29208901	29209200	0.347	0.772	0.399	0.218
Tan	22	29326801	29327100	0.663	0.957	0.379	0.553

Tan	22	29384401	29384700	0.294	0.701	0.535	0.472
Tan	22	29384701	29385000	1.142	1.888	1.266	1.050
Tan	22	29644801	29645100	0.266	0.751	0.243	0.329
Tan	22	29786701	29787000	0.266	0.071	0.531	0.198
Tan	22	29893201	29893500	1.403	2.594	1.826	1.735
Tan	22	29913001	29913300	1.092	1.401	0.749	1.141
Tan	22	29941801	29942100	2.817	1.547	2.394	2.494
Tan	22	29998501	29998800	0.211	0.493	0.267	0.223
Tan	22	30225601	30225900	0.319	0.609	0.395	0.385
Tan	22	30338101	30338400	0.345	0.654	0.350	0.193
Tan	22	30692401	30692700	0.586	0.796	0.321	0.690
Tan	22	31302301	31302600	0.158	0.517	0.214	0.248
Tan	22	32007901	32008200	0.694	1.169	0.827	0.608
Tan	22	32153101	32153400	0.611	0.513	0.292	0.360
Tan	22	32158201	32158500	0.186	0.493	0.214	0.193
Tan	22	32687701	32688000	1.707	2.909	1.404	1.019
Tan	22	32688001	32688300	0.886	1.854	0.711	0.152
Tan	22	32692801	32693100	0.610	0.818	0.584	0.355
Tan	22	32798401	32798700	0.957	1.307	0.580	0.826
Tan	22	32913601	32913900	2.178	2.778	1.525	1.912
Tan	22	33018601	33018900	0.900	1.571	0.531	0.983
Tan	22	33048901	33049200	0.314	0.869	0.181	0.173
Tan	22	33113401	33113700	0.403	1.124	0.194	0.106
Tan	22	33286801	33287100	0.371	0.745	0.156	0.193
Tan	22	33423001	33423300	0.479	1.171	0.383	0.238
Tan	22	33463201	33463500	0.108	0.423	0.025	0.061
Tan	22	33534901	33535200	0.452	0.772	0.457	0.527
Tan	22	33646201	33646500	0.240	0.468	0.239	0.299
Tan	22	33664201	33664500	0.079	0.893	0.029	0.086
Tan	22	33696901	33697200	0.824	1.148	0.633	0.725
Tan	22	33703201	33703500	0.103	0.541	0.107	0.132
Tan	22	33801301	33801600	0.240	0.773	0.181	0.355
Tan	22	33819901	33820200	0.158	0.588	0.214	0.162
Tan	22	34404001	34404300	0.263	0.702	0.341	0.243
Tan	22	34658701	34659000	0.213	0.491	0.243	0.106
Tan	22	34687501	34687800	0.161	0.466	0.214	0.274
Tan	22	34878001	34878300	0.452	0.068	0.613	0.329
Tan	22	35316601	35316900	0.347	0.583	0.156	0.304
Tan	22	35402401	35402700	0.269	1.056	0.437	0.497
Tan	22	35543401	35543700	0.424	0.560	0.185	0.441
Tan	22	35910901	35911200	0.105	0.538	0.312	0.081
Tan	22	36420001	36420300	0.584	0.165	0.604	0.533
Tan	22	36444901	36445200	0.161	0.635	0.194	0.056
Tan	22	36514801	36515100	1.031	1.828	1.023	1.085
Tan	22	36554101	36554400	0.529	0.916	0.395	0.391
Tan	22	36765001	36765300	0.105	1.004	0.486	0.167
Tan	22	36809101	36809400	0.213	0.444	0.214	0.137
Tan	22	36823801	36824100	0.132	0.768	0.317	0.193
Tan	22	36839701	36840000	0.665	2.628	1.797	0.781
Tan	22	36956101	36956400	0.344	0.727	0.428	0.081
Tan	22	36984601	36984900	0.347	0.257	0.531	0.664
Tan	22	37500901	37501200	8.211	6.160	8.484	8.025
Tan	22	37524301	37524600	0.266	0.704	0.448	0.162
Tan	22	37726501	37726800	0.240	0.611	0.243	0.117
Tan	22	37880101	37880400	0.928	0.584	1.012	0.918
Tan	22	38184001	38184300	0.131	0.420	0.132	0.193
Tan	22	38216101	38216400	1.436	0.794	1.320	1.647
Tan	22	38275501	38275800	2.689	1.562	2.156	2.099
Tan	22	38378401	38378700	0.984	0.251	0.881	1.055
Tan	22	38416801	38417100	0.481	0.863	0.444	0.527
Tan	22	38432701	38433000	0.424	1.073	0.239	0.497
Tan	22	38521501	38521800	1.673	0.852	1.563	2.353
Tan	22	38612101	38612400	1.659	2.834	1.683	1.825
Tan	22	38805001	38805300	0.055	0.588	0.078	0.254
Tan	22	39055801	39056100	0.160	0.470	0.160	0.086
Tan	22	39254101	39254400	0.344	0.704	0.370	0.304
Tan	22	39367201	39367500	0.079	0.513	0.058	0.101

Tan	22	39608101	39608400	1.382	0.961	1.547	1.298
Tan	22	39806701	39807000	0.053	0.496	0.000	0.137
Tan	22	39843001	39843300	0.665	0.818	0.584	0.472
Tan	22	39878401	39878700	4.014	5.192	4.162	4.522
Tan	22	40012201	40012500	0.979	1.521	0.938	0.522
Tan	22	40225501	40225800	0.292	0.470	0.078	0.274
Tan	22	40284301	40284600	0.239	0.489	0.049	0.142
Tan	22	40339201	40339500	0.745	0.539	0.399	0.198
Tan	22	40653001	40653300	0.265	0.541	0.189	0.137
Tan	22	40790701	40791000	0.373	0.798	0.152	0.396
Tan	22	40895701	40896000	1.436	1.849	1.320	1.197
Tan	22	40917901	40918200	1.165	1.686	0.312	1.186
Tan	22	40928101	40928400	0.774	0.185	0.860	0.913
Tan	22	40931701	40932000	1.489	1.845	1.213	1.212
Tan	22	40975801	40976100	0.349	1.006	0.740	0.771
Tan	22	41028901	41029200	8.087	7.415	8.302	8.437
Tan	22	41230801	41231100	0.106	0.494	0.082	0.162
Tan	22	41273401	41273700	1.355	2.017	1.308	1.172
Tan	22	41286901	41287200	0.634	1.285	0.736	0.573
Tan	22	41334901	41335200	0.311	1.195	0.354	0.198
Tan	22	41433601	41433900	0.158	0.560	0.214	0.248
Tan	22	41628901	41629200	2.639	1.530	2.633	3.752
Tan	22	41652001	41652300	0.744	1.405	0.678	0.674
Tan	22	41755501	41755800	0.215	0.539	0.267	0.142
Tan	22	41869801	41870100	0.537	1.150	0.609	0.411
Tan	22	41883001	41883300	0.402	1.103	0.292	0.619
Tan	22	41949901	41950200	0.398	0.981	0.317	0.335
Tan	22	41950201	41950500	0.371	0.929	0.374	0.147
Tan	22	42096601	42096900	0.610	1.004	0.473	0.533
Tan	22	42254401	42254700	0.319	0.562	0.370	0.274
Tan	22	42326401	42326700	1.300	0.745	1.344	1.328
Tan	22	42598201	42598500	1.195	0.841	1.230	1.273
Tan	22	43092301	43092600	3.573	1.660	3.603	3.819
Tan	22	43133701	43134000	0.718	1.143	0.901	0.527
Tan	22	43157101	43157400	0.211	0.772	0.103	0.315
Tan	22	43159201	43159500	4.464	5.473	4.096	4.336
Tan	22	43404901	43405200	2.920	3.765	2.109	2.784
Tan	22	43431001	43431300	0.610	0.961	0.531	0.274
Tan	22	43451701	43452000	0.532	0.938	0.515	0.527
Tan	22	44260201	44260500	0.053	0.440	0.127	0.056
Tan	22	44799601	44799900	0.294	0.633	0.243	0.259
Tan	22	44893201	44893500	0.265	0.633	0.156	0.335
Tan	22	45028801	45029100	0.532	0.770	0.477	0.360
Tan	22	45036301	45036600	0.453	0.723	0.267	0.187
Tan	22	45251101	45251400	0.450	0.775	0.214	0.193
Tan	22	45340201	45340500	0.290	0.609	0.234	0.031
Tan	22	45484501	45484800	4.404	2.058	3.825	3.863
Tan	22	45489601	45489900	2.789	1.318	2.568	2.896
Tan	22	45509701	45510000	1.779	3.090	1.801	1.791
Tan	22	45518401	45518700	2.610	1.547	1.986	2.272
Tan	22	45613501	45613800	0.690	1.006	0.399	0.882
Tan	22	45676501	45676800	0.450	1.245	0.604	0.259
Tan	22	46077901	46078200	0.079	0.466	0.185	0.213
Tan	22	46216801	46217100	0.053	0.446	0.111	0.167
Tan	22	46388401	46388700	3.268	2.274	3.353	3.413
Tan	22	46415401	46415700	0.397	0.676	0.321	0.380
Tan	22	46584301	46584600	0.665	1.502	0.602	0.786
Tan	22	46602601	46602900	0.213	0.607	0.111	0.268
Tan	22	46663501	46663800	0.161	0.584	0.267	0.416
Tan	22	46699501	46699800	0.242	0.609	0.239	0.243
Tan	22	46778701	46779000	2.576	1.124	1.935	2.110
Tan	22	46820401	46820700	0.345	0.444	0.210	0.248
Tan	22	46862701	46863000	0.531	1.032	0.638	0.740
Tan	22	46894501	46894800	0.237	0.586	0.321	0.335
Tan	22	46944601	46944900	0.265	0.515	0.317	0.335
Tan	22	46965601	46965900	0.292	0.839	0.432	0.259
Tan	22	46970701	46971000	0.027	0.702	0.205	0.441

Tan	22	47271901	47272200	1.449	3.030	1.443	1.369
Tan	22	47505301	47505600	0.345	0.633	0.321	0.137
Tan	22	47545501	47545800	0.661	1.624	0.403	0.527
Tan	22	47553301	47553600	0.826	1.193	0.671	0.527
Tan	22	47914801	47915100	0.239	0.493	0.292	0.137
Tan	22	47916301	47916600	0.557	0.933	0.399	0.355
Tan	22	47921701	47922000	0.742	1.099	0.794	0.826
Tan	22	48009001	48009300	2.289	3.087	2.216	2.028
Tan	22	48090001	48090300	2.315	1.222	4.900	3.174
Tan	22	48096301	48096600	6.446	3.038	6.940	8.275
Tan	22	48096901	48097200	1.195	1.991	0.497	1.404
Tan	22	48105001	48105300	1.781	2.354	0.510	0.786
Tan	22	48179701	48180000	2.470	1.137	2.209	2.545
Tan	22	48180001	48180300	1.436	0.409	1.395	1.587
Tan	22	48266101	48266400	3.036	1.954	2.855	3.503
Tan	22	48337501	48337800	0.187	0.444	0.165	0.056
Tan	22	48526801	48527100	0.960	1.379	0.575	0.533
Tan	22	48566101	48566400	0.265	0.824	0.169	0.279
Tan	22	48574801	48575100	0.878	1.783	1.119	1.044
Tan	22	48648001	48648300	0.715	1.656	0.856	0.801
Tan	22	48650401	48650700	3.799	0.536	3.041	2.810
Tan	22	48650701	48651000	3.660	1.060	3.039	2.429
Tan	22	48684001	48684300	2.205	1.195	1.781	2.191
Tan	22	48804601	48804900	1.056	2.234	0.597	0.401
Tan	22	48814801	48815100	0.423	0.867	0.288	0.284
Tan	22	48868201	48868500	0.371	0.770	0.194	1.399
Tan	22	48889201	48889500	5.875	3.520	5.794	6.033
Tan	22	48889501	48889800	8.813	5.601	10.931	11.916
Tan	22	48916801	48917100	3.849	2.079	4.026	3.690
Tan	22	48975301	48975600	2.021	0.851	1.835	2.033
Tan	22	49021501	49021800	0.742	1.463	0.716	0.578
Tan	22	49021801	49022100	0.776	1.525	0.680	0.583
Tan	22	49080901	49081200	2.741	3.690	2.430	2.895
Tan	22	49086301	49086600	1.941	1.261	1.032	1.663
Tan	22	49116001	49116300	0.239	0.489	0.267	0.142
Tan	22	49186801	49187100	1.676	2.808	1.826	1.804
Tan	22	49261801	49262100	1.382	0.886	2.011	1.283
Tan	22	49284301	49284600	1.862	2.504	1.957	1.339
Tan	22	49298701	49299000	0.850	1.543	0.675	0.724
Tan	22	49367701	49368000	1.570	3.025	1.456	1.369
Tan	22	49398001	49398300	2.602	1.862	2.666	3.057
Tan	22	49438201	49438500	5.627	4.540	6.447	6.840
Tan	22	49453801	49454100	1.250	1.877	0.671	0.578
Tan	22	49575301	49575600	1.654	0.090	0.778	1.420
Tan	22	49577101	49577400	1.786	1.210	2.122	2.018
Tan	22	49691701	49692000	2.901	3.394	2.004	2.135
Tan	22	49721101	49721400	1.494	2.652	1.516	1.491
Tan	22	49873801	49874100	6.249	4.669	6.077	5.982
Tan	22	49994401	49994700	0.481	0.936	0.473	0.472
Tan	22	50068501	50068800	0.426	0.631	0.448	0.411
Tan	22	50305501	50305800	6.842	7.745	6.619	6.369
Tan	22	50352001	50352300	1.941	0.629	1.608	1.998
Tan	22	50357101	50357400	0.771	0.230	0.740	0.963
Tan	22	50564701	50565000	5.911	4.325	5.199	6.018
Tan	22	50598901	50599200	0.660	1.120	0.720	0.132
Tan	22	50653801	50654100	1.121	2.997	1.699	1.100
Tan	22	50696101	50696400	1.783	0.463	1.414	2.257
Tan	22	50742901	50743200	3.299	2.176	2.818	3.463
Tan	22	50804401	50804700	5.700	3.330	4.624	7.424
Tan	23	96601	96900	0.477	0.116	0.214	0.248
Tan	23	342001	342300	0.134	0.395	0.214	0.223
Tan	23	402601	402900	0.373	0.562	0.448	0.304
Tan	23	564901	565200	0.160	0.444	0.317	0.223
Tan	23	669601	669900	0.529	1.588	0.292	0.534
Tan	23	691201	691500	0.823	1.911	0.587	0.928
Tan	23	704401	704700	0.345	1.261	0.103	0.416
Tan	23	704701	705000	0.752	2.684	0.843	0.959

Tan	23	741301	741600	1.836	2.789	1.859	1.978
Tan	23	748801	749100	2.674	3.532	4.401	2.601
Tan	23	1011901	1012200	1.915	0.689	1.858	2.054
Tan	23	1047301	1047600	2.212	3.735	2.831	2.864
Tan	23	1094101	1094400	2.928	2.012	1.752	2.855
Tan	23	1213801	1214100	1.197	0.556	1.193	1.375
Tan	23	1280401	1280700	3.162	2.236	4.118	4.264
Tan	23	1339801	1340100	0.428	0.749	0.234	0.173
Tan	23	1352101	1352400	3.628	5.954	4.744	3.858
Tan	23	1406401	1406700	0.374	0.656	0.341	0.329
Tan	23	1572901	1573200	2.447	1.474	2.225	2.297
Tan	23	1587301	1587600	0.344	0.860	0.354	0.274
Tan	23	1734901	1735200	2.346	1.403	2.053	1.906
Tan	23	1888201	1888500	0.161	0.588	0.156	0.248
Tan	23	1904101	1904400	3.272	1.473	3.078	4.943
Tan	23	1950301	1950600	1.274	2.086	0.943	1.247
Tan	23	1977601	1977900	1.063	1.354	0.881	1.024
Tan	23	1992001	1992300	0.106	0.279	0.136	0.466
Tan	23	2091601	2091900	0.558	0.373	0.263	0.248
Tan	23	2094301	2094600	0.400	0.725	0.263	0.466
Tan	23	2138101	2138400	0.902	2.349	0.557	0.710
Tan	23	2165701	2166000	0.773	1.245	0.341	0.578
Tan	23	2256601	2256900	1.113	2.381	1.206	1.430
Tan	23	2337601	2337900	0.907	2.043	1.094	1.115
Tan	23	2347501	2347800	1.841	2.967	1.937	2.120
Tan	23	2384101	2384400	7.637	5.512	7.330	8.118
Tan	23	2439901	2440200	0.237	0.749	0.292	0.340
Tan	23	2442601	2442900	0.428	3.206	4.349	0.173
Tan	23	2442901	2443200	0.000	3.439	6.410	0.000
Tan	23	2535301	2535600	0.268	0.820	0.136	0.315
Tan	23	2548801	2549100	0.610	1.105	0.613	0.502
Tan	23	2726701	2727000	1.834	2.296	1.465	1.430
Tan	23	2729101	2729400	0.400	0.845	0.366	0.360
Tan	23	2735101	2735400	2.205	2.474	1.757	1.993
Tan	23	2766901	2767200	1.011	1.547	1.188	0.968
Tan	23	3018901	3019200	4.014	2.605	3.627	3.458
Tan	23	3061501	3061800	0.476	1.009	0.160	0.142
Tan	23	3063601	3063900	0.106	0.448	0.160	0.112
Tan	23	3131701	3132000	0.376	0.845	0.239	0.466
Tan	23	3137401	3137700	0.640	0.841	0.346	0.603
Tan	23	3201001	3201300	1.920	4.012	1.421	1.440
Tan	23	3210601	3210900	2.037	3.194	2.118	1.638
Tan	23	3290701	3291000	4.671	1.933	3.982	4.604
Tan	23	3291001	3291300	5.419	2.679	5.581	6.231
Tan	23	3371701	3372000	0.479	0.961	0.453	0.482
Tan	23	3481801	3482100	1.242	2.409	1.389	1.501
Tan	23	3500101	3500400	0.987	0.298	0.905	0.918
Tan	23	3515401	3515700	0.426	0.957	0.341	0.350
Tan	23	3586201	3586500	0.881	1.571	0.653	0.927
Tan	23	3791401	3791700	0.907	2.062	0.782	1.313
Tan	23	3873901	3874200	1.994	3.335	2.140	1.643
Tan	23	3882001	3882300	0.665	0.234	0.539	0.608
Tan	23	3941101	3941400	6.155	9.325	7.441	6.572
Tan	23	4033201	4033500	1.276	0.605	1.226	1.480
Tan	23	4033801	4034100	0.342	1.255	0.325	0.218
Tan	23	4139101	4139400	0.581	1.122	0.531	0.567
Tan	23	4267801	4268100	0.645	2.600	0.305	0.344
Tan	23	4275901	4276200	0.108	0.442	0.103	0.137
Tan	23	4284601	4284900	0.213	0.515	0.317	0.254
Tan	23	4371301	4371600	9.003	3.996	7.568	8.867
Tan	23	4377601	4377900	1.749	0.335	1.944	1.698
Tan	23	4377901	4378200	1.889	0.524	1.953	2.241
Tan	23	4524301	4524600	0.537	1.120	0.399	0.558
Tan	23	4546201	4546500	0.352	1.122	0.234	0.335
Tan	23	4562401	4562700	0.318	0.049	0.671	0.365
Tan	23	4635601	4635900	0.690	0.538	0.399	0.254
Tan	23	4947001	4947300	1.173	0.583	1.130	1.308

Tan	23	5468101	5468400	0.721	1.502	0.551	0.907
Tan	23	5618701	5619000	0.532	0.843	0.453	0.553
Tan	23	5742901	5743200	0.240	0.444	0.107	0.112
Tan	23	5920201	5920500	0.265	0.678	0.502	0.218
Tan	23	6131101	6131400	0.381	1.270	0.156	0.259
Tan	23	6131401	6131700	0.824	1.362	0.760	0.669
Tan	23	6218701	6219000	0.452	0.817	0.325	0.360
Tan	23	6396001	6396300	0.637	0.959	0.506	0.284
Tan	23	6414001	6414300	0.505	0.727	0.263	0.391
Tan	23	6545401	6545700	0.400	0.845	0.292	0.198
Tan	23	6616801	6617100	0.903	1.217	0.934	0.857
Tan	23	6699301	6699600	2.562	1.512	3.134	2.819
Tan	23	6714601	6714900	0.479	0.749	0.267	0.405
Tan	23	6941701	6942000	1.065	1.496	0.667	0.852
Tan	23	6999601	6999900	0.106	0.423	0.156	0.167
Tan	23	7213501	7213800	0.373	0.515	0.185	0.385
Tan	23	7383601	7383900	0.742	1.478	0.687	0.639
Tan	23	7408801	7409100	1.566	2.156	1.723	1.136
Tan	23	7428301	7428600	0.481	0.961	0.461	0.452
Tan	23	7445101	7445400	1.039	1.452	0.584	1.105
Tan	23	7593001	7593300	0.184	0.635	0.156	0.000
Tan	23	7752001	7752300	1.111	1.852	0.700	0.948
Tan	23	7833301	7833600	0.055	0.682	0.317	0.335
Tan	23	7873501	7873800	0.503	1.242	0.571	0.537
Tan	23	7977901	7978200	0.294	0.955	0.374	0.664
Tan	23	8399401	8399700	0.987	1.710	1.106	0.877
Tan	23	8550901	8551200	0.345	0.657	0.152	0.274
Tan	23	8609101	8609400	0.187	0.513	0.189	0.335
Tan	23	8733601	8733900	0.213	0.584	0.395	0.162
Tan	23	8751301	8751600	0.186	0.395	0.103	0.162
Tan	23	8908501	8908800	0.532	0.281	0.481	0.583
Tan	23	9323101	9323400	0.211	0.682	0.132	0.101
Tan	23	9507901	9508200	0.053	0.515	0.049	0.092
Tan	23	10248301	10248600	0.187	0.699	0.288	0.248
Tan	23	10859101	10859400	0.077	0.631	0.312	0.086
Tan	23	11019001	11019300	3.784	5.471	2.802	2.977
Tan	23	11188501	11188800	0.134	0.515	0.234	0.329
Tan	23	11340901	11341200	0.616	1.236	0.573	0.466
Tan	23	12339301	12339600	0.319	0.629	0.288	0.335
Tan	23	12878101	12878400	0.507	1.148	0.428	0.396
Tan	23	13546501	13546800	0.132	0.352	0.526	0.167
Tan	23	14101201	14101500	0.105	0.494	0.165	0.162
Tan	23	14382301	14382600	0.318	0.890	0.642	0.573
Tan	23	14698501	14698800	0.374	0.725	0.379	0.329
Tan	23	15034501	15034800	0.400	0.772	0.312	0.350
Tan	23	15247201	15247500	0.160	0.706	0.165	0.147
Tan	23	15291301	15291600	0.958	2.231	0.533	0.340
Tan	23	15291601	15291900	0.718	1.613	0.460	0.335
Tan	23	15322801	15323100	0.666	1.658	0.593	0.786
Tan	23	15602401	15602700	0.477	0.723	0.477	0.522
Tan	23	15908101	15908400	0.584	0.210	0.852	0.832
Tan	23	16039501	16039800	0.160	0.328	0.181	0.527
Tan	23	16689001	16689300	0.319	1.261	0.439	0.603
Tan	23	17154001	17154300	0.371	0.139	0.346	0.634
Tan	23	18037801	18038100	0.505	0.232	0.424	0.391
Tan	23	19352401	19352700	0.344	0.657	0.346	0.162
Tan	23	19531801	19532100	1.011	1.708	1.126	0.796
Tan	23	19791001	19791300	0.586	0.397	0.609	0.776
Tan	23	19800001	19800300	0.763	1.560	0.716	0.391
Tan	23	19881601	19881900	0.345	0.560	0.267	0.167
Tan	23	19914301	19914600	0.687	1.197	0.428	0.472
Tan	23	19974001	19974300	0.398	0.586	0.448	0.193
Tan	23	20031301	20031600	1.991	0.699	1.603	1.374
Tan	23	20159701	20160000	1.468	2.133	1.382	1.379
Tan	23	20620201	20620500	0.349	0.820	0.424	0.294
Tan	23	20680201	20680500	0.668	1.495	0.600	0.526
Tan	23	20864401	20864700	0.105	0.444	0.127	0.081

Tan	23	20905801	20906100	0.266	0.420	0.082	0.198
Tan	23	20946901	20947200	1.250	1.847	0.999	1.314
Tan	23	20952301	20952600	0.689	1.699	0.624	0.451
Tan	23	20955601	20955900	0.268	1.030	0.370	0.380
Tan	23	20971501	20971800	0.160	0.938	0.493	0.218
Tan	23	21314701	21315000	1.640	3.688	1.758	1.373
Tan	23	21366301	21366600	0.373	0.515	0.189	0.385
Tan	23	21449401	21449700	0.613	0.165	0.399	0.329
Tan	23	21510301	21510600	0.349	1.337	0.334	0.513
Tan	23	21510601	21510900	0.610	2.219	0.626	0.599
Tan	23	21568801	21569100	0.507	0.841	0.720	0.497
Tan	23	21678601	21678900	0.106	0.423	0.049	0.112
Tan	23	21866101	21866400	0.373	0.513	0.078	0.162
Tan	23	21920701	21921000	0.858	2.352	1.032	0.339
Tan	23	21948301	21948600	8.724	6.243	7.985	9.209
Tan	23	21973801	21974100	0.292	1.568	0.843	0.791
Tan	23	21978001	21978300	1.836	2.476	1.288	1.586
Tan	23	21984601	21984900	0.345	0.444	0.214	0.112
Tan	23	21996001	21996300	0.663	0.069	0.317	0.497
Tan	23	22261501	22261800	0.876	0.302	0.633	0.553
Tan	23	22435501	22435800	3.031	3.581	2.670	2.966
Tan	23	22974601	22974900	0.428	0.863	0.526	0.472
Tan	23	23217601	23217900	0.156	0.466	0.103	0.056
Tan	23	23774401	23774700	0.108	0.470	0.160	0.218
Tan	23	23774701	23775000	0.081	0.423	0.107	0.193
Tan	23	23832601	23832900	2.392	1.541	2.782	2.601
Tan	23	24374701	24375000	0.240	0.517	0.103	0.193
Tan	23	24757501	24757800	0.771	1.212	0.662	0.826
Tan	23	24844501	24844800	1.831	2.909	1.616	1.597
Tan	23	25023901	25024200	0.106	0.489	0.074	0.167
Tan	23	25178401	25178700	1.223	1.568	1.226	1.141
Tan	23	25229101	25229400	0.081	0.442	0.103	0.198
Tan	23	25398601	25398900	0.319	0.824	0.267	0.268
Tan	23	25579801	25580100	0.290	0.562	0.267	0.243
Tan	23	25713001	25713300	0.213	0.699	0.317	0.441
Tan	23	26241901	26242200	0.240	0.611	0.312	0.162
Tan	23	27230101	27230400	0.266	0.607	0.288	0.187
Tan	23	27373501	27373800	0.429	0.024	0.403	0.416
Tan	23	28403701	28404000	0.290	0.749	0.189	0.086
Tan	23	28693201	28693500	2.478	1.351	2.307	3.170
Tan	23	29075701	29076000	1.800	3.004	1.161	1.415
Tan	23	29114101	29114400	0.184	0.564	0.152	0.051
Tan	23	29346301	29346600	0.158	0.541	0.160	0.248
Tan	23	29840101	29840400	0.215	0.584	0.267	0.355
Tan	23	30296401	30296700	0.266	0.588	0.132	0.243
Tan	23	30474301	30474600	0.321	0.985	0.506	0.380
Tan	23	30522601	30522900	0.213	0.846	0.428	0.446
Tan	23	30782101	30782400	0.134	0.401	0.132	0.081
Tan	23	31070101	31070400	0.903	1.684	0.620	0.486
Tan	23	31476301	31476600	0.398	0.633	0.239	0.380
Tan	23	31961101	31961400	0.531	0.890	0.424	0.304
Tan	23	32014201	32014500	0.237	0.777	0.218	0.228
Tan	23	32118601	32118900	2.528	3.310	2.073	2.367
Tan	23	32194501	32194800	1.723	0.292	1.565	1.602
Tan	23	32202301	32202600	0.560	1.487	0.535	0.614
Tan	23	32316001	32316300	3.064	4.193	2.863	3.129
Tan	23	32482201	32482500	0.398	0.539	0.267	0.776
Tan	23	32522101	32522400	0.879	1.940	0.782	1.076
Tan	23	32714101	32714400	0.156	0.846	0.198	0.248
Tan	23	32790301	32790600	0.374	0.607	0.267	0.391
Tan	23	33171901	33172200	1.889	0.768	1.971	1.491
Tan	23	33232201	33232500	0.268	0.633	0.259	0.167
Tan	23	33327601	33327900	0.397	0.910	0.428	0.573
Tan	23	33348001	33348300	0.402	0.843	0.218	0.411
Tan	23	33636301	33636600	1.011	2.305	0.856	1.237
Tan	23	33710101	33710400	0.453	0.933	0.263	0.411
Tan	23	33730801	33731100	0.268	0.654	0.395	0.162

Tan	23	33739201	33739500	0.292	0.515	0.243	0.162
Tan	23	33750301	33750600	0.186	0.633	0.230	0.193
Tan	23	33777601	33777900	0.239	0.607	0.074	0.223
Tan	23	34116601	34116900	0.158	0.442	0.185	0.081
Tan	23	34164301	34164600	0.294	0.536	0.267	0.254
Tan	23	34215901	34216200	0.244	0.725	0.234	0.254
Tan	23	34333801	34334100	0.318	0.629	0.156	0.324
Tan	23	34735801	34736100	0.242	0.584	0.181	0.198
Tan	23	34992301	34992600	3.055	3.855	2.920	2.550
Tan	23	35372701	35373000	1.516	1.804	1.300	1.410
Tan	23	35456101	35456400	0.079	0.395	0.029	0.051
Tan	23	35487901	35488200	0.318	0.491	0.695	0.112
Tan	23	35757601	35757900	0.452	0.629	0.263	0.360
Tan	23	35890201	35890500	0.186	0.609	0.317	0.173
Tan	23	35895001	35895300	0.505	0.747	0.243	0.309
Tan	23	35898901	35899200	3.165	4.044	2.445	2.652
Tan	23	36360001	36360300	0.323	0.796	0.263	0.223
Tan	23	36502201	36502500	0.502	1.217	0.522	0.664
Tan	23	36907201	36907500	1.274	0.773	1.037	1.055
Tan	23	37683301	37683600	1.621	1.098	1.625	1.532
Tan	23	37693201	37693500	0.852	1.172	0.502	0.913
Tan	23	37822801	37823100	0.507	1.785	0.642	1.105
Tan	23	37839901	37840200	0.266	0.633	0.239	0.268
Tan	23	38626801	38627100	0.400	0.165	0.720	0.304
Tan	23	38990101	38990400	0.211	0.446	0.078	0.187
Tan	23	39061801	39062100	3.139	4.641	2.319	2.637
Tan	23	39160801	39161100	0.077	1.182	0.181	0.117
Tan	23	39318601	39318900	0.715	1.571	1.003	0.614
Tan	23	39823501	39823800	0.344	0.609	0.399	0.304
Tan	23	39896401	39896700	0.079	0.425	0.103	0.086
Tan	23	39926401	39926700	1.142	0.496	0.987	0.913
Tan	23	39962101	39962400	0.189	0.517	0.107	0.223
Tan	23	40004701	40005000	0.134	0.466	0.185	0.137
Tan	23	40261201	40261500	0.318	0.654	1.094	0.365
Tan	23	40400401	40400700	0.186	0.517	0.054	0.198
Tan	23	40426801	40427100	0.452	0.283	0.477	0.497
Tan	23	40441801	40442100	1.410	0.511	0.827	1.050
Tan	23	40526101	40526400	2.025	3.012	1.826	2.115
Tan	23	40587301	40587600	0.687	1.457	0.707	0.654
Tan	23	40645201	40645500	0.108	0.494	0.189	0.137
Tan	23	40684201	40684500	1.516	1.034	1.587	1.541
Tan	23	40806001	40806300	0.779	1.614	0.769	0.380
Tan	23	41013601	41013900	0.345	0.680	0.156	0.162
Tan	23	41052001	41052300	0.426	0.142	0.424	0.527
Tan	23	41166601	41166900	0.957	1.939	1.103	0.827
Tan	23	41267401	41267700	0.213	0.423	0.156	0.218
Tan	23	41271001	41271300	0.158	0.448	0.156	0.167
Tan	23	41355901	41356200	0.295	0.777	0.288	0.137
Tan	23	41378401	41378700	0.053	0.420	0.103	0.167
Tan	23	41529901	41530200	1.111	1.946	0.785	1.136
Tan	23	41616901	41617200	0.184	0.564	0.214	0.223
Tan	23	41620801	41621100	2.234	0.998	1.888	1.445
Tan	23	41674501	41674800	1.508	0.538	2.385	1.531
Tan	23	41781901	41782200	0.237	0.584	0.296	0.335
Tan	23	42019501	42019800	0.611	1.453	0.624	0.391
Tan	23	42032701	42033000	0.611	0.914	0.551	0.603
Tan	23	42119701	42120000	1.086	0.588	1.168	1.181
Tan	23	42289801	42290100	0.799	1.422	0.867	0.614
Tan	23	42318001	42318300	0.689	1.869	0.560	1.389
Tan	23	42319501	42319800	2.557	0.749	2.008	1.718
Tan	23	42324301	42324600	0.349	0.890	0.263	0.578
Tan	23	42338101	42338400	0.161	0.863	0.185	0.142
Tan	23	42338401	42338700	0.294	1.650	0.424	0.421
Tan	23	42340501	42340800	0.344	1.740	0.263	0.279
Tan	23	42340801	42341100	0.215	1.877	0.107	0.147
Tan	23	42342601	42342900	0.752	2.152	0.468	0.679
Tan	23	42344401	42344700	1.060	0.425	1.177	1.029

Tan	23	42345901	42346200	0.026	0.611	0.074	0.132
Tan	23	42346201	42346500	0.081	0.444	0.132	0.031
Tan	23	42357601	42357900	3.020	1.193	3.333	3.302
Tan	23	42418801	42419100	0.158	0.534	0.049	0.092
Tan	23	42567901	42568200	4.936	2.471	4.410	5.456
Tan	23	42605401	42605700	0.368	1.961	0.788	0.086
Tan	23	42605701	42606000	2.663	4.789	3.371	2.586
Tan	23	42611701	42612000	0.131	0.468	0.058	0.056
Tan	23	42935701	42936000	2.573	1.459	2.998	2.876
Tan	23	43020001	43020300	0.294	0.955	0.551	0.477
Tan	23	43100701	43101000	0.265	0.768	0.185	0.477
Tan	23	43131001	43131300	0.929	1.777	1.144	1.476
Tan	23	43184101	43184400	0.828	1.356	0.700	0.938
Tan	23	43275301	43275600	2.396	2.920	3.801	4.913
Tan	23	43434001	43434300	0.240	0.466	0.132	0.243
Tan	23	43641001	43641300	0.213	0.444	0.029	0.279
Tan	23	43707001	43707300	0.106	0.515	0.107	0.335
Tan	23	43761601	43761900	0.265	0.536	0.136	0.228
Tan	23	43852801	43853100	0.349	0.773	0.263	0.254
Tan	23	44158801	44159100	0.290	0.631	0.346	0.340
Tan	23	44470501	44470800	1.797	0.324	1.674	1.359
Tan	23	44470801	44471100	2.095	0.468	2.468	1.714
Tan	23	44499601	44499900	0.211	0.466	0.214	0.142
Tan	23	44545501	44545800	0.027	0.515	0.098	0.112
Tan	23	44751301	44751600	0.184	0.704	0.054	0.284
Tan	23	44870101	44870400	0.187	0.448	0.107	0.142
Tan	23	45288301	45288600	0.213	0.583	0.082	0.360
Tan	23	45497701	45498000	0.344	1.197	0.357	0.502
Tan	23	45603301	45603600	0.158	0.494	0.132	0.142
Tan	23	45619801	45620100	0.292	0.983	0.544	0.441
Tan	23	45728401	45728700	0.319	0.564	0.239	0.243
Tan	23	45885001	45885300	0.690	0.843	0.317	0.527
Tan	23	45998701	45999000	0.723	1.614	0.515	0.780
Tan	23	46008001	46008300	0.529	0.987	0.374	0.558
Tan	23	46045201	46045500	0.453	0.723	0.321	0.477
Tan	23	46118101	46118400	0.215	0.719	0.156	0.223
Tan	23	46206601	46206900	2.020	3.251	1.835	2.104
Tan	23	46236301	46236600	0.368	1.006	0.350	0.416
Tan	23	46236601	46236900	0.369	1.009	0.276	0.268
Tan	23	46341301	46341600	0.345	0.891	0.481	0.213
Tan	23	46347901	46348200	0.539	1.886	0.526	0.665
Tan	23	46348201	46348500	0.160	0.980	0.370	0.350
Tan	23	46558501	46558800	1.516	1.798	1.300	1.141
Tan	23	46852201	46852500	0.187	0.863	0.140	0.198
Tan	23	46861801	46862100	0.371	0.657	0.374	0.218
Tan	23	46910701	46911000	0.211	0.633	0.214	0.081
Tan	23	47361901	47362200	0.742	0.210	0.831	0.776
Tan	23	47522101	47522400	0.665	1.217	0.609	0.907
Tan	23	47915401	47915700	1.245	0.650	1.605	1.273
Tan	23	47931301	47931600	0.400	0.768	0.218	0.472
Tan	23	47979901	47980200	0.290	0.515	0.239	0.223
Tan	23	48314401	48314700	0.477	0.000	0.593	0.355
Tan	23	48399001	48399300	0.240	0.541	0.214	0.198
Tan	23	48432601	48432900	2.581	3.454	2.345	2.165
Tan	23	48485401	48485700	0.187	0.446	0.189	0.132
Tan	23	48497401	48497700	1.007	1.684	0.403	0.517
Tan	23	48510001	48510300	0.931	1.835	0.667	1.004
Tan	23	48686701	48687000	0.318	0.727	0.457	0.441
Tan	23	48687001	48687300	0.237	0.704	0.341	0.309
Tan	23	48836101	48836400	0.453	0.142	0.453	0.547
Tan	23	48853801	48854100	0.505	1.051	0.789	0.329
Tan	23	49111201	49111500	0.163	0.729	0.140	0.167
Tan	23	49382401	49382700	1.250	0.682	1.498	1.369
Tan	23	49726501	49726800	0.799	0.983	0.798	0.355
Tan	23	49737301	49737600	0.550	1.424	0.526	0.644
Tan	23	49805101	49805400	0.108	0.583	0.107	0.025
Tan	23	49859101	49859400	0.852	1.289	0.794	0.893

Tan	23	49968001	49968300	0.747	1.075	0.531	0.411
Tan	23	50334901	50335200	0.931	0.747	0.852	1.075
Tan	23	50386201	50386500	3.462	1.869	3.506	3.128
Tan	23	50486101	50486400	3.215	4.192	2.403	3.539
Tan	23	50551201	50551500	0.292	0.609	0.189	0.299
Tan	23	50685301	50685600	0.800	1.172	0.691	0.659
Tan	23	50812501	50812800	0.344	0.723	0.453	0.391
Tan	23	50997301	50997600	0.216	1.264	0.382	0.547
Tan	23	51129901	51130200	0.984	2.289	3.188	2.591
Tan	23	51138901	51139200	1.116	0.654	0.794	1.298
Tan	23	51201001	51201300	0.453	0.987	0.560	0.452
Tan	23	51201301	51201600	0.323	0.916	0.321	0.472
Tan	23	51234001	51234300	1.156	3.714	1.692	2.459
Tan	23	51282001	51282300	2.902	2.165	1.197	1.171
Tan	23	51325501	51325800	0.077	0.493	0.132	0.157
Tan	23	51339601	51339900	0.586	1.410	0.446	0.558
Tan	23	51805801	51806100	0.319	0.841	0.185	0.299
Tan	23	52020601	52020900	0.848	1.652	0.720	1.111
Tan	23	52236601	52236900	0.213	0.493	0.638	0.051
Tan	23	52311301	52311600	0.374	0.888	0.317	0.268
Tan	23	52461901	52462200	0.052	0.328	0.477	0.142
Tan	23	52688701	52689000	0.426	0.163	0.239	0.745
Tan	23	52715101	52715400	0.215	0.676	0.288	0.304
Tan	23	52947601	52947900	2.181	1.639	1.230	2.089
Tan	23	53070301	53070600	0.481	1.847	1.139	1.172
Tan	23	53070601	53070900	0.240	0.959	0.448	0.639
Tan	23	53225401	53225700	0.052	0.637	0.029	0.223
Tan	23	53269201	53269500	0.294	0.489	0.267	0.167
Tan	23	53293201	53293500	0.210	0.631	0.296	0.279
Tan	23	53554501	53554800	0.584	1.150	0.794	0.806
Tan	23	53600401	53600700	0.211	0.446	0.132	0.056
Tan	23	54016801	54017100	0.481	0.867	0.428	0.583
Tan	23	54145801	54146100	0.266	0.609	0.374	0.446
Tan	23	54213001	54213300	0.027	0.378	0.029	0.056
Tan	23	54237001	54237300	0.053	1.172	0.000	0.061
Tan	23	54237301	54237600	0.027	0.701	0.000	0.000
Tan	23	54244501	54244800	0.184	0.541	0.185	0.106
Tan	23	54273301	54273600	1.463	0.959	0.823	1.080
Tan	23	54564001	54564300	0.773	1.373	0.687	0.857
Tan	23	54874801	54875100	1.249	0.631	0.930	0.806
Tan	23	55144501	55144800	2.315	2.010	1.349	1.172
Tan	23	55144801	55145100	2.683	1.895	1.255	1.314
Tan	23	55164901	55165200	1.463	1.259	0.769	1.602
Tan	23	55291501	55291800	0.532	0.936	0.341	0.547
Tan	23	55291801	55292100	0.452	0.656	0.419	0.304
Tan	23	55410301	55410600	0.479	0.538	0.185	0.664
Tan	23	55496701	55497000	1.279	0.487	1.086	1.141
Tan	23	55868401	55868700	2.371	2.755	1.830	1.987
Tan	23	55953601	55953900	0.265	0.517	0.267	0.198
Tan	23	55985401	55985700	0.476	1.032	0.609	0.603
Tan	23	56028301	56028600	0.639	0.352	0.580	0.634
Tan	23	56065501	56065800	1.728	2.712	1.609	1.182
Tan	23	56483401	56483700	0.769	1.006	0.584	0.806
Tan	23	56808901	56809200	0.634	1.686	0.901	1.070
Tan	23	56825401	56825700	0.611	1.599	0.885	0.918
Tan	23	56985301	56985600	3.242	2.596	2.093	3.366
Tan	23	57205201	57205500	0.374	0.629	0.341	0.360
Tan	23	57318301	57318600	0.161	0.515	0.263	0.329
Tan	23	57409801	57410100	0.106	0.513	0.205	0.223
Tan	23	57669901	57670200	0.527	1.216	0.136	0.223
Tan	23	57677401	57677700	1.308	2.154	1.427	1.374
Tan	23	57839701	57840000	0.742	1.006	0.473	0.695
Tan	23	57914101	57914400	2.578	2.057	2.205	1.714
Tan	23	57916201	57916500	3.562	3.956	2.419	5.126
Tan	23	57916801	57917100	3.904	1.760	2.477	2.571
Tan	23	57921901	57922200	1.889	0.796	1.692	1.323
Tan	23	58005901	58006200	1.039	1.710	0.805	0.720

Tan	23	58006201	58006500	1.357	1.704	0.789	1.050
Tan	23	58174201	58174500	1.739	2.797	1.415	1.688
Tan	23	58193101	58193400	2.021	2.474	1.476	1.709
Tan	23	58338001	58338300	1.595	0.983	1.275	1.516
Tan	23	58381201	58381500	0.503	1.101	0.526	0.542
Tan	23	58425901	58426200	0.639	0.328	0.588	0.497
Tan	23	58550701	58551000	0.557	1.169	0.885	0.608
Tan	23	58688701	58689000	0.560	1.734	0.549	0.380
Tan	23	58734601	58734900	0.555	1.287	0.506	0.411
Tan	23	58854301	58854600	0.347	0.678	0.346	0.391
Tan	23	58882501	58882800	3.441	1.341	3.139	2.997
Tan	23	59224801	59225100	0.319	0.800	0.243	0.198
Tan	23	59225101	59225400	0.400	0.794	0.136	0.198
Tan	23	59520301	59520600	0.294	0.652	0.189	0.193
Tan	23	59704801	59705100	0.670	1.098	1.460	0.365
Tan	23	59794801	59795100	0.505	0.865	0.350	0.441
Tan	23	59832901	59833200	0.319	0.612	0.292	0.193
Tan	23	59887201	59887500	0.479	0.139	0.341	0.583
Tan	23	59977501	59977800	0.268	1.202	0.239	0.268
Tan	23	59977801	59978100	0.319	0.678	0.346	0.391
Tan	23	60114301	60114600	0.800	0.279	0.682	1.060
Tan	23	60249601	60249900	3.088	3.722	3.036	2.799
Tan	23	60389701	60390000	0.371	0.862	0.346	0.218
Tan	23	60472501	60472800	0.266	0.611	0.132	0.416
Tan	23	60510601	60510900	0.611	0.444	0.321	0.223
Tan	23	60573301	60573600	0.266	0.723	0.239	0.142
Tan	23	60711301	60711600	0.905	2.579	0.838	1.030
Tan	23	60720601	60720900	1.729	2.062	1.465	2.490
Tan	23	60791101	60791400	0.776	1.335	0.481	0.771
Tan	23	60815401	60815700	0.639	0.794	0.555	0.497
Tan	23	60908401	60908700	0.158	0.583	0.214	0.304
Tan	23	60981001	60981300	0.531	0.796	0.214	0.329
Tan	23	61006501	61006800	0.000	0.491	0.098	0.142
Tan	23	61050601	61050900	5.578	4.209	5.825	5.465
Tan	23	61063201	61063500	6.679	4.761	5.627	6.292
Tan	23	61134301	61134600	3.280	5.366	2.878	3.382
Tan	23	61317001	61317300	2.360	0.896	2.287	2.241
Tan	23	61425901	61426200	0.210	0.725	0.477	0.137
Tan	23	61435801	61436100	1.299	0.631	1.217	1.577
Tan	23	61464601	61464900	1.941	4.010	1.904	2.966
Tan	23	61473601	61473900	0.745	2.516	1.173	1.607
Tan	23	61568701	61569000	1.145	1.878	0.889	1.151
Tan	23	61584001	61584300	0.376	0.890	0.160	0.405
Tan	23	61600501	61600800	3.435	1.071	2.231	3.433
Tan	23	61610701	61611000	0.398	0.116	0.419	0.380
Tan	23	61665301	61665600	2.420	3.042	2.234	2.007
Tan	23	61714201	61714500	0.319	0.491	0.263	0.335
Tan	23	61937101	61937400	2.041	2.733	1.950	1.967
Tan	23	61997101	61997400	0.345	0.165	0.580	0.355
Tan	23	62026501	62026800	0.265	0.444	0.239	0.142
Tan	23	62049901	62050200	5.814	6.829	4.791	5.962
Tan	23	62070301	62070600	1.434	0.772	1.456	1.445
Tan	23	62087401	62087700	0.507	0.940	0.428	0.309
Tan	23	62195401	62195700	3.633	1.489	4.688	4.854
Tan	23	62315701	62316000	0.426	0.725	0.156	0.360
Tan	24	31501	31800	0.828	0.159	3.542	0.922
Tan	24	64201	64500	1.353	1.006	2.073	1.724
Tan	24	169501	169800	0.106	0.466	0.132	0.101
Tan	24	351301	351600	3.143	1.083	2.871	2.703
Tan	24	357301	357600	1.166	1.972	1.057	1.034
Tan	24	618901	619200	1.487	0.747	1.168	1.303
Tan	24	846301	846600	1.302	1.028	1.333	0.806
Tan	24	911401	911700	0.476	0.116	0.486	0.502
Tan	24	1039501	1039800	2.086	0.227	3.665	3.337
Tan	24	1039801	1040100	2.672	0.705	4.396	3.873
Tan	24	1064401	1064700	1.011	1.474	0.593	1.125
Tan	24	1106401	1106700	0.694	1.077	0.477	0.578

Tan	24	1809001	1809300	0.823	1.261	0.531	0.639
Tan	24	1934401	1934700	0.373	1.077	0.432	0.533
Tan	24	2042701	2043000	0.800	1.813	0.909	0.720
Tan	24	2087701	2088000	0.745	0.348	0.716	0.628
Tan	24	2237701	2238000	0.694	0.957	0.403	0.385
Tan	24	2638501	2638800	1.497	3.736	1.162	1.513
Tan	24	2649901	2650200	0.589	1.245	0.341	0.431
Tan	24	2882101	2882400	1.334	3.450	2.091	1.966
Tan	24	2943901	2944200	0.692	0.348	0.658	0.801
Tan	24	3113101	3113400	0.905	1.195	0.769	0.614
Tan	24	3158101	3158400	0.081	0.468	0.181	0.198
Tan	24	3278401	3278700	0.527	0.957	0.453	0.416
Tan	24	3347101	3347400	0.027	0.401	0.025	0.137
Tan	24	3621901	3622200	5.983	4.027	6.065	6.042
Tan	24	3727501	3727800	0.397	1.176	0.555	0.573
Tan	24	4258201	4258500	0.452	0.680	0.506	0.416
Tan	24	4312801	4313100	0.823	2.036	0.860	1.308
Tan	24	4352701	4353000	0.371	0.794	0.506	0.477
Tan	24	4540801	4541100	0.160	0.519	0.107	0.167
Tan	24	4580401	4580700	1.724	2.620	1.694	2.002
Tan	24	4685401	4685700	0.160	0.465	0.185	0.167
Tan	24	4727101	4727400	0.795	1.306	0.638	0.385
Tan	24	4789501	4789800	0.242	0.916	0.243	0.553
Tan	24	4809001	4809300	0.668	1.118	0.497	0.502
Tan	24	4831201	4831500	0.184	0.820	0.292	0.263
Tan	24	4905901	4906200	0.557	0.818	0.453	0.309
Tan	24	4930501	4930800	0.881	2.034	1.061	0.898
Tan	24	4943101	4943400	0.213	0.539	0.317	0.223
Tan	24	5014501	5014800	0.213	0.494	0.132	0.117
Tan	24	5173201	5173500	0.850	0.586	0.103	0.279
Tan	24	5221501	5221800	2.100	2.899	1.893	2.398
Tan	24	5232601	5232900	1.515	1.332	1.835	2.236
Tan	24	5414401	5414700	2.351	0.635	2.876	2.221
Tan	24	5417401	5417700	1.189	2.397	0.979	1.156
Tan	24	5512201	5512500	1.355	2.060	0.992	1.334
Tan	24	5550901	5551200	0.957	0.536	0.818	1.192
Tan	24	5938201	5938500	2.952	3.531	2.756	2.580
Tan	24	5960701	5961000	1.279	1.944	0.885	1.314
Tan	24	5961001	5961300	1.115	1.822	0.881	1.359
Tan	24	6133201	6133500	0.106	0.656	0.288	0.268
Tan	24	6161401	6161700	0.268	0.515	0.136	0.198
Tan	24	6170701	6171000	0.452	0.772	0.551	0.329
Tan	24	6319201	6319500	0.658	1.592	0.711	0.710
Tan	24	6319501	6319800	0.656	1.635	0.560	0.887
Tan	24	6331201	6331500	0.053	0.448	0.103	0.167
Tan	24	6336301	6336600	0.936	2.023	0.930	0.907
Tan	24	6725701	6726000	1.140	2.180	1.436	1.095
Tan	24	6921901	6922200	0.215	0.466	0.025	0.218
Tan	24	7094401	7094700	0.106	0.517	0.185	0.147
Tan	24	7300201	7300500	0.106	0.560	0.111	0.061
Tan	24	7365901	7366200	0.587	1.266	0.983	0.695
Tan	24	7486201	7486500	0.319	0.609	0.194	0.243
Tan	24	7638601	7638900	0.689	1.077	0.720	0.695
Tan	24	7673101	7673400	0.982	1.618	0.424	1.004
Tan	24	7741801	7742100	1.447	3.227	2.015	1.567
Tan	24	7857301	7857600	2.505	4.051	2.446	1.842
Tan	24	7914001	7914300	0.292	1.037	0.428	0.416
Tan	24	7999201	7999500	0.345	1.536	0.510	0.609
Tan	24	8091001	8091300	0.213	0.560	0.288	0.299
Tan	24	8099101	8099400	0.215	0.633	0.350	0.254
Tan	24	8272201	8272500	0.105	0.442	0.185	0.056
Tan	24	8317201	8317500	0.186	0.562	0.321	0.411
Tan	24	8371501	8371800	0.290	0.513	0.210	0.223
Tan	24	8479801	8480100	0.240	0.466	0.234	0.193
Tan	24	8497501	8497800	0.371	0.607	0.239	0.380
Tan	24	8504101	8504400	0.294	0.725	0.267	0.243
Tan	24	8666101	8666400	0.211	0.541	0.214	0.086

Tan	24	8739301	8739600	1.250	2.577	1.580	0.836
Tan	24	8739601	8739900	0.266	0.701	0.321	0.106
Tan	24	8830501	8830800	0.795	0.180	1.148	1.004
Tan	24	8898901	8899200	0.106	0.423	0.243	0.000
Tan	24	9000301	9000600	0.158	0.420	0.082	0.132
Tan	24	9076801	9077100	0.053	0.515	0.078	0.051
Tan	24	9231301	9231600	0.373	0.704	0.210	0.324
Tan	24	9263101	9263400	0.474	1.399	0.807	0.472
Tan	24	9288901	9289200	0.108	0.633	0.263	0.248
Tan	24	9298801	9299100	2.178	1.448	0.970	2.160
Tan	24	9439201	9439500	1.329	1.873	1.344	0.923
Tan	24	9519901	9520200	0.319	0.468	0.107	0.329
Tan	24	9528301	9528600	0.507	0.234	0.716	0.614
Tan	24	9570901	9571200	0.426	0.893	0.317	0.360
Tan	24	9573901	9574200	0.374	0.725	0.288	0.441
Tan	24	9691801	9692100	1.623	2.219	0.823	0.832
Tan	24	9749101	9749400	1.276	0.324	0.765	0.720
Tan	24	9864001	9864300	1.110	1.937	0.468	0.426
Tan	24	9885301	9885600	1.300	2.328	1.139	1.115
Tan	24	9947101	9947400	1.041	1.684	1.081	1.267
Tan	24	9954601	9954900	0.957	0.395	0.765	0.608
Tan	24	9980701	9981000	0.373	1.791	0.325	1.115
Tan	24	10317301	10317600	0.053	0.539	0.176	0.132
Tan	24	10426801	10427100	0.424	0.747	0.580	0.304
Tan	24	10635001	10635300	0.265	0.843	0.531	0.472
Tan	24	10657201	10657500	0.132	0.635	0.234	0.248
Tan	24	10743301	10743600	1.003	1.776	0.847	1.009
Tan	24	10853401	10853700	0.426	0.328	0.136	0.497
Tan	24	10902601	10902900	3.833	6.544	3.734	3.868
Tan	24	11017801	11018100	0.160	0.444	0.107	0.167
Tan	24	11076601	11076900	0.345	0.564	0.185	0.167
Tan	24	11159101	11159400	0.582	1.008	0.312	0.405
Tan	24	11507701	11508000	0.132	0.773	0.127	0.198
Tan	24	11508001	11508300	0.213	0.863	0.312	0.304
Tan	24	11547601	11547900	0.105	0.985	0.160	0.081
Tan	24	11724301	11724600	0.292	1.004	0.263	0.329
Tan	24	12744901	12745200	0.239	0.468	0.185	0.279
Tan	24	13272901	13273200	0.136	0.584	0.103	0.218
Tan	24	13335901	13336200	3.330	5.424	1.852	2.915
Tan	24	13618501	13618800	0.584	0.862	0.584	0.583
Tan	24	13721701	13722000	0.957	2.276	0.918	1.516
Tan	24	13911901	13912200	0.984	1.468	0.731	0.715
Tan	24	14052001	14052300	0.403	1.053	0.555	0.497
Tan	24	14241001	14241300	0.426	0.725	0.292	0.340
Tan	24	14341201	14341500	0.184	0.729	0.325	0.248
Tan	24	14384401	14384700	0.321	0.701	0.156	0.304
Tan	24	14449201	14449500	1.659	2.742	1.206	3.631
Tan	24	14548201	14548500	0.079	0.401	0.160	0.167
Tan	24	14742601	14742900	0.189	0.491	0.185	0.167
Tan	24	14859001	14859300	0.555	0.936	0.473	0.304
Tan	24	15096901	15097200	1.010	1.307	0.881	0.893
Tan	24	15141301	15141600	0.318	0.654	0.341	0.106
Tan	24	15520801	15521100	0.955	2.317	1.190	1.485
Tan	24	15622201	15622500	0.428	0.822	0.403	0.472
Tan	24	15783001	15783300	0.156	0.513	0.185	0.218
Tan	24	16241101	16241400	0.132	0.558	0.214	0.147
Tan	24	16394401	16394700	0.244	0.629	0.160	0.223
Tan	24	16421101	16421400	0.666	0.397	0.584	0.218
Tan	24	16458901	16459200	0.268	0.957	0.448	0.497
Tan	24	16716001	16716300	0.187	0.753	0.267	0.385
Tan	24	16978201	16978500	0.110	0.517	0.111	0.086
Tan	24	17033401	17033700	0.266	0.515	0.267	0.304
Tan	24	17114101	17114400	0.160	0.446	0.210	0.162
Tan	24	17224201	17224500	0.690	1.223	0.638	0.452
Tan	24	17531401	17531700	0.237	0.560	0.140	0.218
Tan	24	17697301	17697600	0.426	1.030	0.160	0.598
Tan	24	17763001	17763300	0.695	1.687	0.428	0.724

Tan	24	17763301	17763600	0.776	1.448	0.424	0.593
Tan	24	17952001	17952300	2.823	3.607	1.719	2.956
Tan	24	18315901	18316200	0.186	0.466	0.239	0.193
Tan	24	18330001	18330300	0.106	0.558	0.054	0.076
Tan	24	18402001	18402300	0.371	0.609	0.263	0.385
Tan	24	18556201	18556500	0.131	0.635	0.074	0.218
Tan	24	18556501	18556800	0.184	0.914	0.074	0.238
Tan	24	19220401	19220700	0.690	1.592	0.909	1.100
Tan	24	19511701	19512000	0.239	0.538	0.403	0.193
Tan	24	19636501	19636800	0.215	0.446	0.160	0.193
Tan	24	19649101	19649400	0.318	1.223	0.272	0.441
Tan	24	19649401	19649700	0.265	0.957	0.267	0.411
Tan	24	19752901	19753200	0.240	0.843	0.098	0.254
Tan	24	19753201	19753500	0.108	0.605	0.074	0.137
Tan	24	20153701	20154000	0.240	0.534	0.243	0.193
Tan	24	20155201	20155500	0.239	0.560	0.312	0.329
Tan	24	20162101	20162400	1.273	1.845	0.930	1.253
Tan	24	20440801	20441100	0.132	0.446	0.234	0.056
Tan	24	20597701	20598000	0.240	0.562	0.218	0.198
Tan	24	20692501	20692800	0.689	0.446	1.139	0.751
Tan	24	21013201	21013500	0.318	0.800	0.181	0.365
Tan	24	21188701	21189000	2.470	2.826	1.991	1.942
Tan	24	21201001	21201300	1.190	2.043	0.831	1.217
Tan	24	21224101	21224400	3.670	2.367	5.254	4.539
Tan	24	21363901	21364200	3.973	2.583	6.004	4.036
Tan	24	21440101	21440400	0.431	0.959	0.296	0.228
Tan	24	21455401	21455700	0.158	0.446	0.103	0.137
Tan	24	21505201	21505500	0.486	1.354	0.564	0.675
Tan	24	21722101	21722400	0.477	0.886	0.526	0.573
Tan	24	22266001	22266300	0.611	1.103	0.252	0.583
Tan	24	22305301	22305600	0.586	0.399	0.292	0.527
Tan	24	22308901	22309200	0.134	0.375	0.107	0.740
Tan	24	22363501	22363800	0.740	2.302	0.716	0.771
Tan	24	22408501	22408800	0.376	0.775	0.239	0.223
Tan	24	22433701	22434000	0.292	0.652	0.424	0.223
Tan	24	22487701	22488000	0.553	1.148	0.617	0.446
Tan	24	22581301	22581600	0.344	1.205	0.350	0.344
Tan	24	22818001	22818300	0.347	0.513	0.876	0.639
Tan	24	23265301	23265600	0.160	0.538	0.214	0.213
Tan	24	23660401	23660700	0.663	0.395	0.745	0.669
Tan	24	23836201	23836500	0.690	1.238	0.711	1.050
Tan	24	24009001	24009300	0.240	0.538	0.234	0.309
Tan	24	24137701	24138000	0.213	0.609	0.263	0.304
Tan	24	24381901	24382200	0.347	0.539	0.210	0.137
Tan	24	24404401	24404700	0.316	0.609	0.292	0.304
Tan	24	24420301	24420600	1.226	1.899	0.827	1.395
Tan	24	24729001	24729300	0.613	0.935	0.408	0.446
Tan	24	24747901	24748200	0.505	0.798	0.107	0.497
Tan	24	24815401	24815700	0.565	1.150	1.719	0.588
Tan	24	24943801	24944100	0.398	0.584	0.477	0.441
Tan	24	24978301	24978600	0.797	0.277	0.687	0.776
Tan	24	25293001	25293300	0.242	1.026	0.346	0.309
Tan	24	25299001	25299300	0.689	1.354	0.597	0.761
Tan	24	25301401	25301700	0.192	1.008	0.263	0.446
Tan	24	25421401	25421700	0.345	0.727	0.390	0.304
Tan	24	25620001	25620300	0.239	0.536	0.239	0.203
Tan	24	25850401	25850700	0.294	0.586	0.239	0.213
Tan	24	25966501	25966800	0.242	0.607	0.321	0.274
Tan	24	26120701	26121000	0.158	0.491	0.267	0.243
Tan	24	26574601	26574900	0.718	0.352	0.580	0.553
Tan	24	26636401	26636700	0.477	0.631	0.189	0.472
Tan	24	26812201	26812500	0.720	1.006	0.473	0.553
Tan	24	26860801	26861100	2.016	2.530	1.382	1.602
Tan	24	26911501	26911800	0.156	0.706	0.049	0.254
Tan	24	27042901	27043200	6.175	1.993	6.527	4.745
Tan	24	27095701	27096000	1.381	2.135	1.193	1.369
Tan	24	27130501	27130800	1.566	1.987	1.534	1.273

Tan	24	27457501	27457800	0.665	1.311	0.745	1.075
Tan	24	27462601	27462900	0.318	0.444	0.214	0.112
Tan	24	27744901	27745200	0.694	1.397	2.234	0.538
Tan	24	27806401	27806700	0.663	1.053	0.675	0.639
Tan	24	28261201	28261500	0.481	1.047	0.428	0.376
Tan	24	28263301	28263600	0.345	0.723	0.403	0.228
Tan	24	28341001	28341300	0.426	1.004	0.267	0.340
Tan	24	28378501	28378800	0.744	0.536	0.346	0.355
Tan	24	28643101	28643400	0.428	1.148	0.506	0.725
Tan	24	29072701	29073000	0.876	0.393	1.032	0.963
Tan	24	29151901	29152200	0.268	0.493	0.185	0.142
Tan	24	29183701	29184000	0.186	0.491	0.189	0.243
Tan	24	29668201	29668500	3.670	3.182	3.690	3.422
Tan	24	29725501	29725800	1.810	1.210	1.835	1.841
Tan	24	29909401	29909700	0.077	0.659	0.029	0.112
Tan	24	29914201	29914500	0.266	0.446	0.185	0.223
Tan	24	30095701	30096000	0.077	0.442	0.156	0.137
Tan	24	30655801	30656100	0.398	0.656	0.218	0.274
Tan	24	31008301	31008600	0.053	0.515	0.210	0.223
Tan	24	31053901	31054200	0.587	0.912	0.580	0.441
Tan	24	31110901	31111200	0.108	0.541	0.152	0.112
Tan	24	31188001	31188300	0.213	0.517	0.185	0.254
Tan	24	31253101	31253400	0.398	0.893	0.395	0.193
Tan	24	31997101	31997400	0.532	1.480	0.577	0.309
Tan	24	32211001	32211300	2.686	1.509	3.413	3.073
Tan	24	32272201	32272500	1.087	1.867	0.885	1.196
Tan	24	32274901	32275200	0.398	1.006	0.481	0.654
Tan	24	32401801	32402100	0.294	0.609	0.103	0.274
Tan	24	32481901	32482200	1.355	1.030	0.794	1.242
Tan	24	32508901	32509200	0.398	0.702	0.453	0.492
Tan	24	32621401	32621700	0.292	0.674	0.321	0.299
Tan	24	32643001	32643300	0.189	0.538	0.140	0.162
Tan	24	32643301	32643600	0.106	0.753	0.054	0.106
Tan	24	32697601	32697900	0.400	0.843	0.292	0.248
Tan	24	32698801	32699100	1.036	1.564	1.008	1.354
Tan	24	32769601	32769900	0.187	0.515	0.239	0.223
Tan	24	32924401	32924700	3.139	2.714	2.114	2.353
Tan	24	32996401	32996700	2.531	1.746	2.521	2.727
Tan	24	33011701	33012000	0.373	0.796	0.078	0.446
Tan	24	33249601	33249900	4.391	2.950	3.504	3.940
Tan	24	33342901	33343200	2.020	1.468	1.937	2.074
Tan	24	33442801	33443100	0.452	0.775	0.481	0.416
Tan	24	33567001	33567300	0.690	0.399	0.181	0.751
Tan	24	33645601	33645900	3.475	4.486	2.205	2.651
Tan	24	33662701	33663000	0.505	0.656	0.292	0.279
Tan	24	33864001	33864300	0.428	1.764	0.798	0.862
Tan	24	33989701	33990000	0.265	0.515	0.263	0.274
Tan	24	34034101	34034400	0.792	1.334	0.497	0.745
Tan	24	34038301	34038600	0.774	1.334	0.691	0.826
Tan	24	34136701	34137000	0.720	1.122	0.740	0.441
Tan	24	34223101	34223400	1.018	2.443	0.470	0.710
Tan	24	34260301	34260600	0.395	0.890	0.506	0.502
Tan	24	34401901	34402200	0.294	0.991	0.181	0.238
Tan	24	34513201	34513500	0.266	0.420	0.185	0.193
Tan	24	34603201	34603500	0.240	0.538	0.127	0.198
Tan	24	34657501	34657800	0.803	2.442	0.743	0.715
Tan	24	34657801	34658100	2.612	4.387	2.410	2.741
Tan	24	34658101	34658400	4.118	7.541	3.810	4.733
Tan	24	34725901	34726200	1.873	3.514	1.790	2.245
Tan	24	34746001	34746300	0.132	0.466	0.132	0.117
Tan	24	34757101	34757400	0.453	1.150	0.609	0.522
Tan	24	35031901	35032200	1.534	3.090	1.859	1.810
Tan	24	35072401	35072700	1.197	1.659	1.139	0.943
Tan	24	35118601	35118900	0.505	1.341	0.548	0.299
Tan	24	35593801	35594100	0.000	1.300	0.000	0.000
Tan	24	35594101	35594400	0.000	4.081	0.000	0.000
Tan	24	35594401	35594700	0.000	3.678	0.000	0.000

Tan	24	35598601	35598900	0.000	1.056	0.000	0.000
Tan	24	35598901	35599200	0.000	1.099	0.000	0.000
Tan	24	35599501	35599800	0.000	0.798	0.000	0.000
Tan	24	35599801	35600100	0.027	0.449	0.000	0.000
Tan	24	35730301	35730600	1.333	2.255	1.139	1.247
Tan	24	35741401	35741700	1.683	2.691	1.777	1.541
Tan	24	35748301	35748600	0.926	1.796	1.217	0.999
Tan	24	35872801	35873100	4.762	3.729	4.590	5.536
Tan	24	35877601	35877900	0.266	0.657	0.230	0.137
Tan	24	35941801	35942100	0.608	1.193	0.526	0.695
Tan	24	35976901	35977200	2.367	1.418	1.859	2.571
Tan	24	36037801	36038100	0.266	0.730	0.049	0.309
Tan	24	36370201	36370500	2.284	3.040	2.292	2.271
Tan	24	36389101	36389400	0.265	0.513	0.160	0.218
Tan	24	36463801	36464100	0.318	0.770	0.321	0.553
Tan	24	36750301	36750600	0.532	0.794	0.243	0.411
Tan	24	36922201	36922500	0.265	0.489	0.189	0.218
Tan	24	36956101	36956400	0.136	0.657	0.243	0.167
Tan	24	37052401	37052700	0.373	0.607	0.374	0.243
Tan	24	37208401	37208700	3.544	2.321	3.554	3.980
Tan	24	37263901	37264200	0.505	1.047	0.321	0.533
Tan	24	37744501	37744800	0.632	1.453	0.653	0.684
Tan	24	37920601	37920900	0.344	0.766	1.509	0.248
Tan	24	37925701	37926000	5.636	5.374	4.282	4.969
Tan	24	37952101	37952400	0.105	0.536	0.132	0.182
Tan	24	38118601	38118900	0.982	1.431	0.934	1.004
Tan	24	38206501	38206800	0.931	1.448	0.584	1.070
Tan	24	38221501	38221800	0.239	0.562	0.267	0.299
Tan	24	38250301	38250600	2.368	1.710	2.760	2.282
Tan	24	38402701	38403000	0.318	0.908	0.424	0.558
Tan	24	38414701	38415000	0.397	1.152	0.370	0.350
Tan	24	38637601	38637900	0.903	1.708	0.905	0.638
Tan	24	38747701	38748000	0.958	1.408	0.506	0.974
Tan	24	38930701	38931000	1.520	2.246	0.995	1.476
Tan	24	38931601	38931900	4.197	3.098	4.307	4.405
Tan	24	38941201	38941500	3.039	3.884	3.003	3.174
Tan	24	38976301	38976600	0.134	0.538	0.181	0.147
Tan	24	39030001	39030300	0.505	0.305	0.531	0.639
Tan	24	39120601	39120900	0.397	1.408	0.513	0.391
Tan	24	39129601	39129900	8.777	5.258	7.902	9.020
Tan	24	39138001	39138300	1.877	3.781	1.692	2.084
Tan	24	39197701	39198000	0.289	0.775	0.214	0.299
Tan	24	39455401	39455700	1.094	1.914	0.856	1.035
Tan	24	39595201	39595500	9.340	7.982	10.072	9.511
Tan	24	39621301	39621600	2.629	4.383	2.617	2.803
Tan	24	39711601	39711900	8.748	9.814	5.988	13.719
Tan	24	39715201	39715500	0.455	0.845	0.325	0.299
Tan	24	39716401	39716700	3.668	1.175	2.426	3.184
Tan	24	39874801	39875100	2.078	2.832	1.645	2.434
Tan	24	39897601	39897900	0.374	0.656	0.239	0.329
Tan	24	39938701	39939000	0.319	0.444	0.321	0.106
Tan	24	40245301	40245600	1.513	0.626	2.033	1.471
Tan	24	40280401	40280700	2.791	1.529	3.908	3.488
Tan	24	40280701	40281000	4.428	2.306	4.758	4.533
Tan	24	40376401	40376700	2.742	1.624	3.380	2.702
Tan	24	40460101	40460400	4.341	2.113	4.070	3.995
Tan	24	40523401	40523700	5.117	3.027	5.230	5.005
Tan	24	40852501	40852800	0.477	0.914	0.481	0.533
Tan	24	41181301	41181600	2.155	3.029	2.060	2.013
Tan	24	41484901	41485200	0.347	0.068	0.457	0.497
Tan	24	41527201	41527500	1.010	1.360	0.794	0.826
Tan	24	41604301	41604600	8.798	7.784	6.714	8.918
Tan	24	41730601	41730900	0.639	0.914	0.564	0.466
Tan	24	41809201	41809500	1.623	0.925	1.654	2.023
Tan	24	41898601	41898900	0.318	0.768	0.239	0.268
Tan	24	42026401	42026700	4.331	3.600	4.077	3.113
Tan	25	331501	331800	0.689	1.307	0.707	0.542

Tan	25	663601	663900	0.687	1.377	0.412	0.684
Tan	25	730801	731100	0.637	0.375	0.127	0.117
Tan	25	821701	822000	0.158	0.491	0.136	0.223
Tan	25	850801	851100	0.136	0.981	0.461	0.385
Tan	25	1542001	1542300	0.215	0.470	0.185	0.137
Tan	25	1591501	1591800	0.292	0.798	0.239	0.441
Tan	25	1659001	1659300	0.292	0.493	0.267	0.218
Tan	25	1752301	1752600	0.534	1.071	0.604	0.608
Tan	25	2239801	2240100	0.187	0.420	0.160	0.106
Tan	25	2829601	2829900	1.992	2.409	1.551	1.886
Tan	25	3319201	3319500	0.292	0.118	0.506	0.304
Tan	25	3380101	3380400	1.010	1.193	0.711	0.801
Tan	25	3586201	3586500	0.294	0.845	0.259	0.248
Tan	25	3594001	3594300	1.711	5.032	2.535	1.941
Tan	25	3605701	3606000	8.243	6.091	7.854	7.936
Tan	25	3780901	3781200	0.773	0.328	0.682	0.695
Tan	25	3810901	3811200	0.186	0.584	0.247	0.254
Tan	25	4075501	4075800	0.668	1.266	0.638	0.725
Tan	25	4236001	4236300	0.771	0.584	0.399	0.385
Tan	25	4611301	4611600	1.192	2.304	1.132	0.826
Tan	25	4662601	4662900	1.168	0.397	0.802	0.801
Tan	25	4712401	4712700	0.768	0.094	0.959	0.882
Tan	25	4738801	4739100	1.041	2.212	1.016	1.222
Tan	25	4764301	4764600	0.513	1.290	0.205	0.279
Tan	25	4764601	4764900	0.374	1.369	0.185	0.218
Tan	25	4838701	4839000	0.319	0.539	0.049	0.132
Tan	25	4963801	4964100	0.103	0.729	0.243	0.309
Tan	25	5024401	5024700	0.857	2.733	1.482	0.623
Tan	25	5298601	5298900	1.781	1.043	2.093	2.490
Tan	25	5327101	5327400	0.929	0.350	0.740	1.131
Tan	25	5405701	5406000	0.344	0.916	0.247	0.436
Tan	25	5415301	5415600	0.189	0.584	0.176	0.137
Tan	25	5870101	5870400	0.558	1.306	0.506	0.806
Tan	25	6034501	6034800	0.929	1.287	0.575	0.355
Tan	25	6170701	6171000	1.803	2.718	1.563	1.790
Tan	25	6171001	6171300	1.568	2.433	1.186	1.425
Tan	25	6192601	6192900	0.187	0.513	0.160	0.132
Tan	25	6395101	6395400	0.610	0.914	0.642	0.664
Tan	25	6522601	6522900	0.397	0.751	0.419	0.416
Tan	25	6585901	6586200	0.161	0.802	0.160	0.228
Tan	25	6795901	6796200	0.136	0.770	0.160	0.335
Tan	25	6808501	6808800	0.745	0.142	0.399	0.639
Tan	25	6841501	6841800	0.424	0.747	0.395	0.446
Tan	25	6843901	6844200	1.303	0.236	0.584	0.882
Tan	25	6844201	6844500	2.284	0.573	1.823	2.658
Tan	25	6857401	6857700	0.160	0.539	0.074	0.193
Tan	25	6970801	6971100	0.294	0.562	0.025	0.157
Tan	25	6971101	6971400	0.689	2.069	0.283	0.101
Tan	25	6984301	6984600	2.105	1.242	2.740	2.333
Tan	25	7003501	7003800	0.507	0.888	0.502	0.173
Tan	25	7085401	7085700	0.294	0.562	0.296	0.198
Tan	25	7168501	7168800	0.771	1.774	0.691	1.019
Tan	25	7168801	7169100	0.476	1.120	0.495	0.492
Tan	25	7249801	7250100	0.186	0.657	0.243	0.086
Tan	25	7285201	7285500	0.266	0.656	0.156	0.187
Tan	25	7308901	7309200	0.318	0.611	0.082	0.335
Tan	25	7498501	7498800	0.586	1.903	0.591	0.599
Tan	25	7527301	7527600	0.450	0.865	0.555	0.385
Tan	25	7542901	7543200	0.636	1.008	0.584	0.573
Tan	25	7572601	7572900	0.213	0.560	0.078	0.218
Tan	25	7649701	7650000	0.557	0.749	0.370	0.472
Tan	25	7689901	7690200	0.186	0.444	0.156	0.167
Tan	25	7801801	7802100	0.215	0.820	0.317	0.603
Tan	25	8148001	8148300	0.398	0.914	0.321	0.715
Tan	25	8209201	8209500	5.956	4.609	5.250	6.008
Tan	25	8220901	8221200	0.634	2.457	0.444	0.614
Tan	25	8496001	8496300	0.105	0.489	0.140	0.112

Tan	25	8921101	8921400	3.134	2.547	2.007	2.961
Tan	25	9188701	9189000	2.949	3.722	4.394	2.306
Tan	25	9423301	9423600	0.211	0.538	0.288	0.299
Tan	25	9441901	9442200	0.398	1.731	1.255	1.075
Tan	25	9558301	9558600	1.405	2.105	1.391	1.359
Tan	25	9782101	9782400	0.642	1.498	0.254	0.603
Tan	25	9795001	9795300	0.186	0.566	0.214	0.167
Tan	25	9966001	9966300	0.558	0.723	0.267	0.355
Tan	25	10061401	10061700	4.419	3.212	4.287	4.953
Tan	25	10294501	10294800	0.160	0.470	0.181	0.218
Tan	25	10471501	10471800	0.292	0.511	0.132	0.193
Tan	25	12518101	12518400	1.223	0.772	0.506	0.639
Tan	25	12599701	12600000	2.070	1.684	0.827	2.261
Tan	25	12600001	12600300	1.996	1.641	0.667	2.256
Tan	25	12693601	12693900	0.079	0.491	0.098	0.167
Tan	25	13343401	13343700	0.508	1.223	0.564	0.486
Tan	25	13767901	13768200	1.857	2.626	1.547	1.638
Tan	25	13783201	13783500	0.239	0.444	0.111	0.142
Tan	25	13783501	13783800	0.187	0.442	0.111	0.142
Tan	25	13790701	13791000	0.240	0.770	0.181	0.284
Tan	25	13896001	13896300	0.160	0.442	0.239	0.056
Tan	25	13977901	13978200	1.252	1.592	0.588	1.136
Tan	25	14532601	14532900	1.755	2.916	1.828	1.586
Tan	25	14931001	14931300	0.369	0.790	0.317	0.218
Tan	25	15288301	15288600	0.610	1.195	0.847	0.690
Tan	25	15405301	15405600	1.515	2.150	1.099	1.703
Tan	25	15807901	15808200	0.853	1.613	0.967	0.847
Tan	25	15818701	15819000	0.213	0.491	0.317	0.198
Tan	25	15979501	15979800	0.876	1.358	1.801	1.633
Tan	25	16125601	16125900	0.240	0.611	0.399	0.086
Tan	25	16414201	16414500	0.345	0.515	0.317	0.335
Tan	25	16546501	16546800	0.345	0.727	0.473	0.416
Tan	25	17639101	17639400	0.507	0.187	0.453	0.355
Tan	25	19147801	19148100	1.144	1.523	0.921	0.664
Tan	25	19148401	19148700	0.268	0.538	0.288	0.223
Tan	25	19627201	19627500	0.529	0.817	0.312	0.137
Tan	25	19741501	19741800	0.266	0.538	0.267	0.268
Tan	25	19818601	19818900	0.211	1.011	0.254	0.329
Tan	25	20561401	20561700	0.240	0.446	0.074	0.000
Tan	25	21110101	21110400	0.319	0.489	0.239	0.162
Tan	25	22169701	22170000	0.479	0.305	0.588	0.578
Tan	25	22768201	22768500	0.718	0.513	0.823	0.441
Tan	25	24189901	24190200	2.071	1.633	2.118	2.490
Tan	25	24239401	24239700	1.886	2.384	2.044	2.155
Tan	25	24267601	24267900	0.184	0.730	0.181	0.187
Tan	25	24655801	24656100	0.158	0.517	0.160	0.223
Tan	25	24695401	24695700	0.242	0.515	0.189	0.223
Tan	25	25390501	25390800	0.555	1.262	0.374	0.492
Tan	25	25390801	25391100	0.929	1.236	0.432	0.938
Tan	25	25444501	25444800	2.842	1.452	3.221	2.600
Tan	25	25570501	25570800	2.746	3.321	1.522	2.241
Tan	25	25782001	25782300	0.345	1.174	0.510	0.547
Tan	25	25991401	25991700	0.560	0.843	0.584	0.309
Tan	25	26139601	26139900	0.105	0.586	0.214	0.182
Tan	25	26169301	26169600	0.853	1.755	1.148	1.015
Tan	25	26169601	26169900	1.149	2.077	1.514	1.172
Tan	25	26265601	26265900	0.263	0.753	0.321	0.187
Tan	25	26526001	26526300	0.136	0.609	0.399	0.167
Tan	25	26545801	26546100	1.410	0.910	1.251	1.253
Tan	25	26598301	26598600	1.195	0.865	0.588	1.131
Tan	25	26602201	26602500	5.136	2.864	4.204	4.371
Tan	25	26622601	26622900	0.344	0.725	0.132	0.416
Tan	25	26976901	26977200	2.334	3.180	2.682	1.709
Tan	25	27080401	27080700	0.240	0.659	0.239	0.167
Tan	25	27294901	27295200	0.269	0.751	0.296	0.360
Tan	25	27546601	27546900	0.213	0.629	0.424	0.938
Tan	25	27564001	27564300	0.215	0.470	0.210	0.056

Tan	25	27635401	27635700	0.240	0.513	0.399	0.279
Tan	25	27825901	27826200	3.129	5.034	3.077	3.305
Tan	25	27847201	27847500	6.619	6.945	7.782	6.551
Tan	25	28401001	28401300	0.345	0.865	0.424	0.456
Tan	25	29385001	29385300	0.211	0.493	0.239	0.198
Tan	25	29923501	29923800	0.213	0.539	0.205	0.279
Tan	25	29952301	29952600	0.186	0.798	0.453	0.502
Tan	25	30404101	30404400	0.186	0.611	0.243	0.329
Tan	25	30553801	30554100	1.726	2.551	1.774	1.273
Tan	25	30815101	30815400	0.134	0.491	0.189	0.076
Tan	25	30852601	30852900	0.053	0.562	0.156	0.193
Tan	25	30867001	30867300	2.331	4.529	2.247	1.978
Tan	25	30935401	30935700	0.160	0.420	0.243	0.248
Tan	25	30955801	30956100	0.237	0.584	0.317	0.187
Tan	25	31262401	31262700	2.310	1.214	1.929	0.542
Tan	25	31275301	31275600	0.105	0.895	0.152	0.112
Tan	25	31308001	31308300	0.240	0.442	0.210	0.056
Tan	25	31358701	31359000	1.673	1.307	1.946	2.110
Tan	25	31476001	31476300	0.187	0.605	0.176	0.167
Tan	25	31513501	31513800	2.442	1.011	3.326	2.098
Tan	25	31732201	31732500	0.318	0.583	0.243	0.329
Tan	25	31828801	31829100	0.692	0.118	0.341	0.279
Tan	25	32062801	32063100	0.215	0.678	0.239	0.380
Tan	25	32183701	32184000	0.287	0.865	0.107	0.187
Tan	25	32496301	32496600	2.442	1.167	1.884	2.180
Tan	25	32732701	32733000	0.581	1.122	0.453	0.614
Tan	25	32835601	32835900	0.210	0.772	0.428	0.380
Tan	25	32842801	32843100	0.932	1.616	1.070	1.014
Tan	25	32888101	32888400	0.292	0.635	0.346	0.994
Tan	25	32892301	32892600	1.034	1.828	0.769	1.348
Tan	25	32919601	32919900	0.134	0.588	0.205	0.142
Tan	25	33024601	33024900	1.036	0.702	1.086	0.963
Tan	25	33144001	33144300	0.187	0.631	0.136	0.274
Tan	25	33144301	33144600	0.373	1.238	0.296	0.233
Tan	25	33151201	33151500	1.065	0.770	1.188	1.323
Tan	25	33267901	33268200	0.160	0.444	0.214	0.279
Tan	25	33616501	33616800	0.269	0.631	0.296	0.279
Tan	25	33619501	33619800	1.913	0.494	1.596	1.455
Tan	25	33619801	33620100	0.953	1.654	0.493	0.862
Tan	25	33765001	33765300	1.789	3.248	0.992	2.515
Tan	25	33780601	33780900	4.183	1.799	4.282	4.385
Tan	25	33826501	33826800	0.160	0.444	0.107	0.031
Tan	25	33852901	33853200	0.611	1.053	0.613	0.533
Tan	25	33912901	33913200	0.290	1.197	0.415	0.335
Tan	25	33916801	33917100	0.292	0.466	0.584	0.335
Tan	25	33922801	33923100	0.371	0.562	0.346	0.416
Tan	25	33981601	33981900	1.592	1.214	1.908	1.546
Tan	25	33982201	33982500	4.257	3.362	4.187	4.259
Tan	25	34280701	34281000	0.108	0.727	0.317	0.167
Tan	25	34313101	34313400	0.106	0.747	0.074	0.025
Tan	25	34319401	34319700	0.215	1.427	0.490	0.527
Tan	25	34327201	34327500	5.904	3.435	5.137	4.679
Tan	25	34393501	34393800	1.436	0.721	1.402	1.486
Tan	25	34433101	34433400	1.515	1.635	1.144	0.968
Tan	25	34560001	34560300	0.344	0.867	0.259	0.228
Tan	25	34710901	34711200	0.215	0.541	0.136	0.223
Tan	25	34713001	34713300	1.065	1.609	1.099	0.988
Tan	25	34743601	34743900	0.235	0.826	0.239	0.248
Tan	25	34781101	34781400	4.443	2.793	3.583	3.625
Tan	25	34790401	34790700	0.531	0.987	0.346	0.522
Tan	25	34800601	34800900	0.294	0.702	0.473	0.223
Tan	25	34848001	34848300	0.134	0.493	0.098	0.117
Tan	25	35046301	35046600	0.239	0.862	0.243	0.304
Tan	25	35194201	35194500	0.160	0.491	0.181	0.187
Tan	25	35204101	35204400	0.081	0.401	0.103	0.086
Tan	25	35255101	35255400	0.292	0.656	0.132	0.193
Tan	25	35446801	35447100	0.137	0.583	0.156	0.051

Tan	25	35447101	35447400	0.213	0.837	0.259	0.132
Tan	25	35465701	35466000	0.903	0.373	1.003	0.614
Tan	25	35510101	35510400	0.398	0.702	0.419	0.385
Tan	25	35560201	35560500	0.532	0.914	0.535	1.192
Tan	25	35585101	35585400	0.718	0.959	0.876	0.578
Tan	25	35616601	35616900	0.132	0.539	0.111	0.051
Tan	25	35676301	35676600	0.239	0.938	0.176	0.106
Tan	25	35721901	35722200	0.292	0.538	0.189	0.355
Tan	25	36389701	36390000	0.211	0.566	0.239	0.142
Tan	25	36442801	36443100	0.424	1.219	0.481	0.725
Tan	25	36573901	36574200	0.818	2.094	0.571	0.446
Tan	25	37030201	37030500	0.426	1.351	1.973	0.335
Tan	25	37160701	37161000	0.690	1.238	0.716	0.943
Tan	25	37202401	37202700	0.160	0.420	0.243	0.086
Tan	25	37297801	37298100	0.665	0.139	0.267	0.385
Tan	25	37788601	37788900	0.187	0.489	0.160	0.228
Tan	25	37847401	37847700	0.268	0.657	0.234	0.274
Tan	25	38239501	38239800	0.826	0.536	0.794	0.832
Tan	25	38643901	38644200	1.912	2.873	2.073	2.251
Tan	25	38660401	38660700	0.981	1.309	0.901	0.907
Tan	25	38760001	38760300	0.531	1.082	0.399	0.578
Tan	25	38841901	38842200	0.081	0.539	0.181	0.173
Tan	25	38963401	38963700	0.639	0.258	0.588	0.837
Tan	25	39053401	39053700	0.160	0.515	0.111	0.279
Tan	25	39132901	39133200	1.379	1.845	1.201	1.253
Tan	25	39168601	39168900	0.450	0.912	0.350	0.234
Tan	25	39179701	39180000	0.294	0.609	0.317	0.218
Tan	25	39218701	39219000	3.610	1.495	3.192	2.727
Tan	25	39243001	39243300	0.079	0.420	0.049	0.086
Tan	25	39297901	39298200	1.515	0.815	1.494	1.480
Tan	25	39551701	39552000	0.931	1.401	0.794	0.994
Tan	25	40054801	40055100	0.160	1.523	0.446	0.506
Tan	25	40386901	40387200	0.639	1.356	0.354	0.457
Tan	25	40397101	40397400	1.116	1.684	0.999	1.064
Tan	25	40901701	40902000	1.670	0.598	2.325	1.719
Tan	25	40913101	40913400	2.174	3.352	1.978	1.348
Tan	25	40965601	40965900	0.665	0.069	0.346	0.391
Tan	25	41054401	41054700	0.161	0.536	0.288	0.223
Tan	25	41127601	41127900	0.106	0.983	0.132	0.274
Tan	25	41158201	41158500	6.230	4.814	5.997	6.192
Tan	25	41196601	41196900	0.242	0.682	0.189	0.218
Tan	25	41196901	41197200	0.290	0.843	0.457	0.527
Tan	25	41221201	41221500	0.979	1.918	0.720	0.979
Tan	25	41365501	41365800	0.589	1.337	0.502	0.492
Tan	25	41799601	41799900	0.266	1.109	0.132	0.213
Tan	25	41830201	41830500	0.239	0.893	0.350	0.284
Tan	25	42014401	42014700	2.205	1.216	2.530	2.277
Tan	25	42036601	42036900	2.021	1.594	0.745	1.039
Tan	25	42185701	42186000	0.799	1.126	0.486	0.771
Tan	25	42259501	42259800	0.266	0.466	0.185	0.223
Tan	25	42367801	42368100	0.106	0.444	0.185	0.132
Tan	25	42389401	42389700	1.168	1.873	0.894	1.050
Tan	25	42444901	42445200	0.237	1.120	0.477	0.826
Tan	25	42501001	42501300	0.749	1.330	0.753	0.710
Tan	25	42502801	42503100	0.795	0.354	0.847	1.055
Tan	25	42699001	42699300	0.824	0.487	0.823	0.771
Tan	25	42702901	42703200	0.345	0.491	0.160	0.279
Tan	25	42741601	42741900	2.471	2.006	1.440	2.474
Tan	25	42754501	42754800	4.707	3.192	4.425	4.360
Tan	25	42775501	42775800	0.132	0.466	0.165	0.142
Tan	25	42801901	42802200	0.158	0.657	0.341	0.193
Tan	25	42965101	42965400	1.300	0.820	0.321	0.431
Tan	25	43014901	43015200	2.662	3.841	2.218	2.864
Tan	25	43333801	43334100	0.297	0.796	0.214	0.340
Tan	25	43524301	43524600	0.263	0.980	0.214	0.213
Tan	25	43540501	43540800	2.272	4.358	2.306	2.733
Tan	25	43559401	43559700	0.453	0.725	0.350	0.360

Tan	25	43629301	43629600	0.294	0.796	0.116	0.243
Tan	25	43689001	43689300	3.323	1.871	3.522	3.220
Tan	25	43689301	43689600	3.828	1.980	4.034	3.559
Tan	25	43846801	43847100	0.369	0.845	0.448	0.436
Tan	25	44095801	44096100	0.694	1.457	0.847	0.542
Tan	25	44131201	44131500	4.283	3.411	3.805	4.193
Tan	25	44239501	44239800	1.913	1.356	1.888	1.866
Tan	25	44242801	44243100	0.773	1.210	0.671	0.715
Tan	25	44363101	44363400	0.561	1.911	0.825	0.862
Tan	25	44433301	44433600	0.477	1.748	0.811	0.547
Tan	25	44448901	44449200	0.932	2.017	1.050	0.826
Tan	25	44555401	44555700	1.936	1.053	1.690	1.633
Tan	25	44604301	44604600	2.129	2.736	1.835	1.927
Tan	25	44622301	44622600	0.876	0.296	0.905	1.044
Tan	25	44662501	44662800	0.850	1.099	0.798	0.720
Tan	25	44718301	44718600	0.507	0.140	0.296	0.385
Tan	25	44745001	44745300	3.746	5.932	5.250	5.202
Tan	25	44911201	44911500	4.706	2.557	4.184	3.671
Tan	25	44985001	44985300	0.186	0.511	0.214	0.173
Tan	25	44999401	44999700	0.292	0.466	0.136	0.274
Tan	25	45014101	45014400	1.674	1.452	0.934	0.796
Tan	25	45166201	45166500	3.854	3.227	2.468	2.617
Tan	25	45196801	45197100	0.082	0.680	0.214	0.101
Tan	25	45246901	45247200	0.105	0.629	0.127	0.228
Tan	25	45320401	45320700	0.874	1.594	0.745	0.857
Tan	26	14701	15000	0.265	0.560	0.296	0.279
Tan	26	212401	212700	1.010	0.375	0.720	0.700
Tan	26	239401	239700	1.165	2.152	0.736	1.511
Tan	26	241801	242100	0.187	0.468	0.189	0.198
Tan	26	729601	729900	0.319	0.444	0.103	0.112
Tan	26	738001	738300	0.295	0.818	0.127	0.218
Tan	26	775801	776100	0.426	0.749	0.296	0.254
Tan	26	805801	806100	0.824	1.073	0.852	0.725
Tan	26	818401	818700	1.224	1.699	0.395	0.796
Tan	26	917101	917400	0.668	1.748	0.972	0.730
Tan	26	998101	998400	2.231	1.506	2.867	2.333
Tan	26	1150201	1150500	0.984	1.144	0.633	0.720
Tan	26	1425901	1426200	0.211	1.034	0.691	0.391
Tan	26	1433701	1434000	0.295	0.940	0.107	0.263
Tan	26	1592401	1592700	0.294	0.652	0.272	0.329
Tan	26	1594501	1594800	0.981	1.729	0.905	1.177
Tan	26	1732801	1733100	0.239	0.650	0.116	0.223
Tan	26	1807201	1807500	0.450	0.772	0.399	0.309
Tan	26	1861501	1861800	0.881	0.257	0.987	0.522
Tan	26	2127301	2127600	2.016	1.096	0.444	1.750
Tan	26	2186401	2186700	0.768	1.922	1.081	1.222
Tan	26	2260201	2260500	0.398	0.515	0.321	0.329
Tan	26	2330101	2330400	0.529	1.101	0.399	0.431
Tan	26	2379601	2379900	0.398	0.607	0.136	0.000
Tan	26	2652601	2652900	1.278	2.090	0.809	1.206
Tan	26	2711101	2711400	0.452	0.796	0.370	0.426
Tan	26	2917201	2917500	1.250	2.749	0.671	1.140
Tan	26	2920801	2921100	0.345	0.725	0.419	0.304
Tan	26	3039301	3039600	1.037	2.006	0.873	0.761
Tan	26	3048901	3049200	0.240	0.444	0.267	0.248
Tan	26	3098101	3098400	0.398	0.846	0.239	0.446
Tan	26	3272401	3272700	0.482	1.491	0.843	0.502
Tan	26	3783601	3783900	0.661	1.287	0.493	0.553
Tan	26	3975601	3975900	0.558	1.124	0.691	0.796
Tan	26	4587001	4587300	0.561	0.890	0.584	0.547
Tan	26	4995601	4995900	0.105	0.444	0.078	0.142
Tan	26	4995901	4996200	0.186	0.730	0.317	0.193
Tan	26	5119501	5119800	0.373	0.584	0.292	0.198
Tan	26	5178601	5178900	0.373	0.702	0.325	0.132
Tan	26	5244001	5244300	0.608	1.403	0.490	0.461
Tan	26	5248201	5248500	0.211	0.468	0.165	0.162
Tan	26	5336101	5336400	3.057	0.819	2.535	2.408

Tan	26	5502301	5502600	0.108	0.513	0.214	0.117
Tan	26	5605201	5605500	0.426	0.258	0.531	0.106
Tan	26	5910001	5910300	0.134	0.442	0.054	0.137
Tan	26	5912101	5912400	0.213	0.446	0.218	0.223
Tan	26	5952601	5952900	2.047	3.279	2.118	2.769
Tan	26	5952901	5953200	3.188	4.437	2.553	3.129
Tan	26	6147001	6147300	0.186	0.751	0.341	0.472
Tan	26	6233101	6233400	0.586	0.867	0.555	0.547
Tan	26	6280201	6280500	0.960	1.313	0.740	0.324
Tan	26	6300601	6300900	0.534	1.807	0.581	0.648
Tan	26	6414001	6414300	0.213	0.584	0.259	0.243
Tan	26	7101601	7101900	0.369	1.448	0.341	0.335
Tan	26	7299601	7299900	0.379	2.019	0.140	0.283
Tan	26	7398601	7398900	0.081	0.444	0.082	0.254
Tan	26	7538701	7539000	0.718	0.210	0.424	0.391
Tan	26	7545901	7546200	0.213	0.466	0.103	0.137
Tan	26	8418901	8419200	0.132	0.493	0.160	0.101
Tan	26	8475601	8475900	0.345	0.682	0.453	0.223
Tan	26	8531401	8531700	0.000	0.425	0.058	0.000
Tan	26	8547001	8547300	0.644	1.658	0.889	0.648
Tan	26	9036001	9036300	0.186	0.444	0.346	0.720
Tan	26	9456601	9456900	0.187	0.517	0.165	0.248
Tan	26	9867301	9867600	0.592	2.296	0.292	0.248
Tan	26	9980701	9981000	0.187	0.863	0.025	0.243
Tan	26	9981001	9981300	0.134	0.423	0.025	0.167
Tan	26	10251301	10251600	0.237	0.749	0.078	0.355
Tan	26	10501801	10502100	0.613	0.910	0.613	0.639
Tan	26	10670701	10671000	0.986	1.330	0.856	0.472
Tan	26	10813501	10813800	0.292	0.513	0.210	0.167
Tan	26	10954801	10955100	0.881	0.399	1.480	0.968
Tan	26	10955701	10956000	0.268	0.654	0.181	0.294
Tan	26	11009701	11010000	0.106	0.446	0.210	0.142
Tan	26	11148001	11148300	0.186	0.515	0.296	0.081
Tan	26	11357101	11357400	0.665	1.242	0.909	0.573
Tan	26	11758201	11758500	2.157	3.010	2.575	3.478
Tan	26	11812201	11812500	0.027	0.517	0.136	0.187
Tan	26	12268201	12268500	0.850	0.586	0.979	1.080
Tan	26	12362701	12363000	0.986	0.727	0.403	0.938
Tan	26	12443401	12443700	0.211	0.629	0.123	0.157
Tan	26	12443701	12444000	0.186	0.676	0.201	0.193
Tan	26	12501001	12501300	4.844	4.109	5.174	5.699
Tan	26	12540001	12540300	0.106	0.586	0.292	0.335
Tan	26	12805801	12806100	0.690	0.983	0.687	1.237
Tan	26	12948001	12948300	0.960	2.015	1.514	1.663
Tan	26	13015801	13016100	0.215	0.612	0.132	0.112
Tan	26	13080601	13080900	0.240	0.659	0.243	0.193
Tan	26	13292401	13292700	1.144	1.935	1.431	1.227
Tan	26	13366501	13366800	2.363	1.491	3.027	2.723
Tan	26	13404301	13404600	0.079	0.592	0.054	0.112
Tan	26	13683301	13683600	0.108	0.420	0.214	0.137
Tan	26	13683601	13683900	0.160	0.446	0.160	0.137
Tan	26	13695301	13695600	0.265	0.706	0.374	0.304
Tan	26	13758001	13758300	0.160	0.560	0.156	0.142
Tan	26	13780201	13780500	2.604	3.133	3.549	3.590
Tan	26	13957801	13958100	1.757	2.079	1.387	1.348
Tan	26	14481901	14482200	0.903	0.772	0.555	0.999
Tan	26	14527501	14527800	0.507	0.886	0.395	0.426
Tan	26	14763301	14763600	0.055	0.534	0.103	0.051
Tan	26	14771701	14772000	0.340	1.569	0.272	0.573
Tan	26	14775301	14775600	0.453	1.377	0.317	0.264
Tan	26	14784601	14784900	2.131	0.238	1.594	1.095
Tan	26	15122101	15122400	0.237	0.846	0.263	0.198
Tan	26	15497101	15497400	3.074	1.311	2.813	2.600
Tan	26	15789901	15790200	0.294	0.678	0.292	0.254
Tan	26	16023601	16023900	0.687	0.140	0.691	0.679
Tan	26	16066201	16066500	0.160	0.517	0.082	0.223
Tan	26	16433101	16433400	0.134	0.466	0.082	0.157

Tan	26	16610701	16611000	0.986	1.427	0.613	0.756
Tan	26	17515201	17515500	0.507	0.773	0.185	0.365
Tan	26	17621401	17621700	0.637	0.961	0.477	0.598
Tan	26	17639701	17640000	0.611	0.985	0.535	0.659
Tan	26	17684101	17684400	0.160	0.423	0.132	0.198
Tan	26	17724001	17724300	0.450	0.727	0.132	0.147
Tan	26	17877301	17877600	0.398	0.258	0.374	0.527
Tan	26	17943001	17943300	0.239	1.195	0.867	0.832
Tan	26	18255301	18255600	0.268	0.817	0.152	0.355
Tan	26	18306301	18306600	0.027	0.423	0.054	0.112
Tan	26	18307201	18307500	0.189	0.721	0.185	0.153
Tan	26	18307501	18307800	0.053	0.560	0.210	0.056
Tan	26	18366301	18366600	0.587	0.891	0.448	0.527
Tan	26	18576601	18576900	0.503	0.862	0.506	0.477
Tan	26	18704701	18705000	0.263	0.631	0.259	0.167
Tan	26	18706201	18706500	0.160	0.423	0.107	0.173
Tan	26	18786901	18787200	1.166	2.618	0.658	1.718
Tan	26	18929101	18929400	0.400	0.656	0.428	0.173
Tan	26	19093501	19093800	0.265	0.517	0.189	0.025
Tan	26	19119001	19119300	0.266	0.612	0.239	0.193
Tan	26	20761501	20761800	0.319	0.680	0.288	0.315
Tan	26	20977501	20977800	0.397	0.918	0.210	0.112
Tan	26	20977801	20978100	0.318	0.846	0.234	0.112
Tan	26	21348601	21348900	0.771	1.053	0.283	0.715
Tan	26	21933001	21933300	0.958	1.309	0.974	0.614
Tan	26	22133401	22133700	0.957	2.045	0.753	1.080
Tan	26	22342801	22343100	1.358	0.822	1.320	1.242
Tan	26	22456501	22456800	0.132	0.539	0.140	0.157
Tan	26	22539001	22539300	0.371	0.633	0.424	0.385
Tan	26	22996201	22996500	0.055	0.517	0.098	0.142
Tan	26	23127301	23127600	0.240	0.584	0.136	0.299
Tan	26	23377201	23377500	0.213	0.628	0.181	0.173
Tan	26	24017701	24018000	0.106	0.519	0.210	0.081
Tan	26	24056101	24056400	0.881	1.330	0.448	0.756
Tan	26	24106501	24106800	2.718	1.268	2.274	2.332
Tan	26	24352801	24353100	0.160	0.423	0.054	0.162
Tan	26	24605101	24605400	0.979	1.453	0.905	0.776
Tan	26	24640201	24640500	0.211	0.626	0.160	0.218
Tan	26	24645001	24645300	1.302	2.206	1.139	1.282
Tan	26	24677701	24678000	0.321	0.820	0.453	0.522
Tan	26	24714601	24714900	0.294	0.674	0.243	0.147
Tan	26	24798301	24798600	2.181	3.015	1.580	1.912
Tan	26	24915001	24915300	2.526	1.366	2.867	2.850
Tan	26	25093501	25093800	2.279	4.171	2.691	2.454
Tan	26	25095601	25095900	3.633	1.708	4.446	3.808
Tan	26	25295701	25296000	0.134	0.442	0.210	0.137
Tan	26	25296001	25296300	0.186	0.493	0.082	0.274
Tan	26	25311001	25311300	0.081	0.446	0.156	0.167
Tan	26	25371601	25371900	2.310	3.424	2.031	2.459
Tan	26	25377901	25378200	0.186	0.611	0.107	0.193
Tan	26	25417501	25417800	0.768	1.667	0.843	0.765
Tan	26	25476901	25477200	0.136	0.489	0.156	0.193
Tan	26	26016301	26016600	0.108	0.442	0.029	0.223
Tan	26	26115601	26115900	0.453	0.772	0.987	0.385
Tan	26	26706901	26707200	0.158	0.583	0.140	0.223
Tan	26	27080701	27081000	0.215	0.560	0.194	0.248
Tan	26	27186301	27186600	0.639	1.195	0.477	0.634
Tan	26	27300001	27300300	1.086	1.684	0.974	1.091
Tan	26	27582901	27583200	0.239	0.539	0.214	0.198
Tan	26	27679501	27679800	0.158	1.332	0.426	0.203
Tan	26	28071001	28071300	1.994	2.525	1.971	3.001
Tan	26	28105501	28105800	0.239	0.824	0.288	0.117
Tan	26	28402501	28402800	0.158	1.562	0.127	0.355
Tan	26	28719901	28720200	1.276	2.392	0.671	0.756
Tan	26	29380201	29380500	0.429	1.148	0.551	0.669
Tan	26	29428801	29429100	0.187	0.768	0.317	0.279
Tan	26	30913801	30914100	1.279	1.892	0.609	1.293

Tan	26	31049401	31049700	0.426	0.560	0.156	0.248
Tan	26	31328401	31328700	0.452	0.629	0.185	0.279
Tan	26	31499401	31499700	0.269	1.255	0.481	0.506
Tan	26	31499701	31500000	0.268	0.790	0.453	0.360
Tan	26	31757401	31757700	0.211	0.657	0.218	0.304
Tan	26	31778401	31778700	0.477	0.255	0.667	0.502
Tan	26	31801501	31801800	0.160	0.401	0.078	0.142
Tan	26	31819201	31819500	2.557	4.218	1.025	2.484
Tan	26	32108401	32108700	0.211	0.586	0.189	0.391
Tan	26	32110801	32111100	0.294	0.657	0.263	0.162
Tan	26	32119501	32119800	0.294	0.704	0.127	0.309
Tan	26	32127001	32127300	0.397	0.680	0.243	0.355
Tan	26	32139601	32139900	0.294	0.869	0.185	0.401
Tan	26	32210401	32210700	5.025	3.107	1.304	3.970
Tan	26	32421901	32422200	0.665	1.118	0.526	0.598
Tan	26	32535901	32536200	0.318	0.629	0.078	0.248
Tan	26	32570401	32570700	1.434	1.706	0.613	1.151
Tan	26	32683201	32683500	0.716	1.448	0.856	0.573
Tan	26	32715901	32716200	0.158	0.539	0.181	0.142
Tan	26	33079201	33079500	0.347	0.725	0.350	0.309
Tan	26	33294601	33294900	0.319	0.629	0.453	0.472
Tan	26	33390301	33390600	0.055	0.446	0.054	0.147
Tan	26	33682801	33683100	0.210	0.611	0.185	0.147
Tan	26	33918001	33918300	0.213	0.539	0.267	0.284
Tan	26	33972001	33972300	0.132	0.444	0.189	0.056
Tan	26	34206001	34206300	0.319	0.442	0.025	0.193
Tan	26	34306501	34306800	0.349	0.701	0.346	0.223
Tan	26	34397101	34397400	0.132	0.446	0.074	0.025
Tan	26	34552801	34553100	4.123	2.939	3.941	4.001
Tan	26	34614001	34614300	0.211	0.562	0.185	0.157
Tan	26	34734601	34734900	0.749	1.810	0.205	0.436
Tan	26	34750201	34750500	0.158	0.678	0.243	0.340
Tan	26	34884901	34885200	0.637	1.049	0.740	0.497
Tan	26	34956901	34957200	1.724	1.053	1.957	1.582
Tan	26	35015701	35016000	0.186	0.536	0.210	0.132
Tan	26	35031001	35031300	0.666	1.152	0.481	0.360
Tan	26	35148001	35148300	0.184	0.517	0.156	0.187
Tan	26	35411101	35411400	2.094	3.116	1.957	2.302
Tan	26	35607901	35608200	0.797	1.006	0.584	0.852
Tan	26	35642101	35642400	1.781	2.264	2.834	2.596
Tan	26	35651701	35652000	4.233	6.199	2.989	4.058
Tan	26	35886601	35886900	0.186	0.609	0.341	0.416
Tan	26	35889301	35889600	0.850	1.120	0.827	0.771
Tan	26	35984401	35984700	2.002	3.766	2.513	2.012
Tan	26	36120601	36120900	0.265	0.654	0.078	0.274
Tan	26	36123301	36123600	3.865	5.821	3.834	4.984
Tan	26	36219601	36219900	0.639	1.343	0.428	0.578
Tan	26	36221401	36221700	2.157	1.281	1.835	2.576
Tan	26	36252601	36252900	0.903	1.901	0.838	0.877
Tan	26	36274801	36275100	8.601	10.288	8.319	8.158
Tan	26	36462601	36462900	0.053	0.656	0.243	0.254
Tan	26	36525601	36525900	1.202	2.673	0.742	1.500
Tan	26	36566701	36567000	0.160	0.446	0.127	0.193
Tan	26	36603901	36604200	0.213	0.609	0.321	0.340
Tan	26	36695101	36695400	0.661	1.573	0.638	0.649
Tan	26	36736501	36736800	0.215	0.820	0.234	0.309
Tan	26	36750301	36750600	0.344	2.860	0.577	0.770
Tan	26	36804601	36804900	0.558	1.103	0.366	0.355
Tan	26	36849901	36850200	2.078	2.716	1.246	2.014
Tan	26	36878101	36878400	0.187	0.468	0.111	0.162
Tan	26	36955501	36955800	0.053	0.420	0.103	0.167
Tan	26	36982501	36982800	0.290	1.026	0.374	0.421
Tan	26	37148401	37148700	0.611	1.006	0.502	0.578
Tan	26	37149001	37149300	1.576	0.863	2.011	1.694
Tan	26	37289101	37289400	2.076	1.523	0.967	2.439
Tan	26	37289401	37289700	1.862	1.354	1.041	1.714
Tan	26	37368601	37368900	2.223	0.702	2.303	1.916

Tan	26	37392301	37392600	5.335	6.780	5.199	5.466
Tan	26	37414201	37414500	0.613	0.303	0.136	0.142
Tan	26	37449301	37449600	0.958	0.212	0.769	0.644
Tan	26	37493401	37493700	0.161	1.264	0.156	0.193
Tan	26	37499401	37499700	0.160	0.423	0.185	0.248
Tan	26	37548901	37549200	0.345	0.745	0.419	0.106
Tan	26	37604401	37604700	0.957	1.523	0.925	0.405
Tan	26	38067001	38067300	0.263	0.963	0.317	0.431
Tan	26	38076901	38077200	0.424	0.751	0.341	0.380
Tan	26	38325001	38325300	0.319	0.682	0.374	0.360
Tan	26	38548801	38549100	0.666	1.266	0.905	0.913
Tan	26	38602801	38603100	0.611	1.077	0.564	0.679
Tan	26	38660101	38660400	0.373	0.560	0.239	0.329
Tan	26	38661301	38661600	0.797	1.079	0.428	0.832
Tan	26	38693401	38693700	0.210	0.656	0.181	0.137
Tan	26	38768401	38768700	6.679	4.941	6.399	6.531
Tan	26	38934601	38934900	7.340	3.778	6.335	5.110
Tan	26	38935201	38935500	4.894	1.639	3.834	2.947
Tan	26	38969701	38970000	8.808	6.271	9.479	7.423
Tan	26	39207001	39207300	1.063	1.496	1.148	1.085
Tan	26	39226201	39226500	0.853	1.472	0.720	0.558
Tan	26	39261301	39261600	0.134	0.543	0.025	0.031
Tan	26	39387601	39387900	0.503	1.070	0.580	0.461
Tan	26	39397501	39397800	0.718	0.159	0.448	0.664
Tan	26	39422701	39423000	3.541	2.736	3.357	3.550
Tan	26	39462301	39462600	0.347	1.053	0.662	0.385
Tan	26	39462601	39462900	1.999	5.376	3.734	1.816
Tan	26	39503701	39504000	0.108	0.401	0.049	0.056
Tan	26	39590401	39590700	0.452	0.843	0.419	0.360
Tan	26	39681901	39682200	0.426	0.656	0.428	0.355
Tan	26	39698701	39699000	0.745	0.352	0.531	0.664
Tan	26	39860101	39860400	0.371	1.699	0.357	0.416
Tan	26	39895501	39895800	0.531	0.187	0.526	0.527
Tan	26	40014301	40014600	1.197	0.678	0.189	0.852
Tan	26	40160701	40161000	0.371	0.583	0.370	0.329
Tan	26	40218001	40218300	2.415	1.892	2.633	1.420
Tan	26	40270201	40270500	5.157	3.941	2.093	6.399
Tan	26	40310101	40310400	1.194	2.261	1.650	0.968
Tan	26	40404901	40405200	0.503	1.200	0.584	0.634
Tan	26	40464601	40464900	0.474	1.004	0.457	0.578
Tan	26	40519501	40519800	1.623	1.141	1.485	2.231
Tan	26	40661701	40662000	0.584	1.566	0.909	1.206
Tan	26	40662001	40662300	0.687	1.467	0.856	0.837
Tan	26	40858501	40858800	0.718	1.096	0.745	0.315
Tan	26	40879501	40879800	1.408	0.699	1.271	1.394
Tan	26	40952701	40953000	1.623	2.410	1.300	1.860
Tan	26	40989301	40989600	0.187	0.047	1.115	0.223
Tan	26	41175901	41176200	0.531	1.008	0.317	0.355
Tan	26	41284201	41284500	3.938	4.557	5.002	3.863
Tan	26	41291101	41291400	0.426	0.890	0.638	0.502
Tan	26	41431501	41431800	0.452	0.751	0.346	0.309
Tan	26	41522701	41523000	0.160	0.611	0.399	0.223
Tan	26	41974501	41974800	0.211	0.629	0.399	0.472
Tan	26	42148201	42148500	0.584	0.980	0.716	0.547
Tan	26	42317101	42317400	0.265	0.987	0.272	0.558
Tan	26	42690601	42690900	0.826	1.452	1.037	1.095
Tan	26	42734701	42735000	0.053	0.584	0.165	0.360
Tan	26	42841801	42842100	0.240	0.466	0.321	0.695
Tan	26	42851701	42852000	0.981	1.285	0.979	0.684
Tan	26	42872101	42872400	0.294	0.912	0.136	0.274
Tan	26	42876601	42876900	0.373	0.680	0.453	0.218
Tan	26	42945001	42945300	0.613	0.118	0.395	0.355
Tan	26	42961801	42962100	0.239	0.493	0.239	0.299
Tan	26	43046701	43047000	4.326	2.370	4.055	4.528
Tan	26	43550101	43550400	0.373	0.071	0.457	0.497
Tan	26	43643401	43643700	0.189	1.262	0.386	0.329
Tan	26	43780201	43780500	0.479	0.212	0.667	0.421

Tan	26	43927201	43927500	0.452	0.865	0.662	0.553
Tan	26	43928701	43929000	0.237	0.584	0.152	0.132
Tan	26	43974301	43974600	1.999	1.240	1.748	1.957
Tan	3	255601	255900	0.105	0.586	0.156	0.187
Tan	3	261901	262200	0.347	0.843	0.218	0.385
Tan	3	348601	348900	0.768	1.257	1.888	0.725
Tan	3	462901	463200	6.328	8.479	5.832	6.511
Tan	3	489901	490200	0.294	0.964	0.419	0.309
Tan	3	524401	524700	4.999	3.098	4.885	5.228
Tan	3	648901	649200	0.905	0.397	1.576	0.832
Tan	3	983701	984000	10.347	7.275	8.971	9.516
Tan	3	1000801	1001100	0.400	1.034	0.564	0.218
Tan	3	1114201	1114500	8.803	4.448	9.670	8.959
Tan	3	1594501	1594800	1.858	2.549	1.201	2.038
Tan	3	1647301	1647600	2.176	2.877	1.663	1.892
Tan	3	1982701	1983000	1.650	2.435	1.193	1.516
Tan	3	2070001	2070300	0.984	0.723	0.881	0.968
Tan	3	2143801	2144100	1.836	2.266	1.971	1.622
Tan	3	2177101	2177400	0.608	0.303	0.745	0.968
Tan	3	2185201	2185500	3.239	2.375	3.393	3.251
Tan	3	2211301	2211600	0.611	0.326	0.584	0.573
Tan	3	2629801	2630100	7.889	4.108	12.587	7.542
Tan	3	2974801	2975100	1.697	2.605	1.415	1.673
Tan	3	2992501	2992800	3.855	2.690	3.150	3.940
Tan	3	3152401	3152700	1.353	1.849	1.324	1.120
Tan	3	3176701	3177000	2.157	1.403	0.609	0.582
Tan	3	3205801	3206100	0.505	0.348	0.189	0.112
Tan	3	3664201	3664500	0.294	0.702	0.346	0.324
Tan	3	3702001	3702300	0.423	1.176	0.555	0.553
Tan	3	3925501	3925800	1.539	2.154	1.148	1.795
Tan	3	3965401	3965700	0.718	1.054	0.740	0.832
Tan	3	4059901	4060200	0.957	1.495	0.580	0.983
Tan	3	4068901	4069200	3.428	1.965	3.353	2.768
Tan	3	4071901	4072200	0.769	2.420	0.789	0.952
Tan	3	4223101	4223400	3.717	2.454	3.868	3.756
Tan	3	4350001	4350300	1.783	2.382	1.472	1.714
Tan	3	4479901	4480200	1.242	2.051	1.206	0.695
Tan	3	4505701	4506000	0.374	0.869	0.189	0.187
Tan	3	4560601	4560900	0.613	1.073	0.555	0.492
Tan	3	4581001	4581300	0.797	0.538	0.847	0.877
Tan	3	4642801	4643100	0.319	0.794	0.132	0.583
Tan	3	4694401	4694700	0.558	0.444	0.613	0.553
Tan	3	4734001	4734300	0.132	0.468	0.074	0.162
Tan	3	4752301	4752600	1.063	1.785	0.789	1.111
Tan	3	4758901	4759200	2.638	4.096	1.491	3.006
Tan	3	4812901	4813200	0.294	1.075	0.116	0.639
Tan	3	4821001	4821300	0.136	0.517	0.165	0.061
Tan	3	4894501	4894800	0.160	0.680	0.259	0.329
Tan	3	4894801	4895100	1.036	2.613	1.005	1.226
Tan	3	4909201	4909500	6.279	4.634	5.464	6.693
Tan	3	5016301	5016600	1.812	1.425	1.859	1.825
Tan	3	5053201	5053500	2.739	3.890	2.336	1.852
Tan	3	5056201	5056500	2.081	3.583	2.107	2.232
Tan	3	5107201	5107500	4.027	2.566	4.138	3.757
Tan	3	5238601	5238900	0.448	0.794	0.428	0.466
Tan	3	5251501	5251800	3.110	4.235	2.878	3.559
Tan	3	5282701	5283000	2.494	3.454	2.234	2.414
Tan	3	5302201	5302500	0.531	0.961	0.555	0.426
Tan	3	5352301	5352600	0.398	0.513	0.107	0.248
Tan	3	5402101	5402400	2.767	0.890	2.715	2.531
Tan	3	5547301	5547600	0.476	0.884	0.350	0.416
Tan	3	5571001	5571300	1.362	0.577	1.378	1.328
Tan	3	5604601	5604900	0.318	0.491	0.267	0.304
Tan	3	5645701	5646000	0.611	0.114	0.588	0.649
Tan	3	5770201	5770500	0.773	1.748	0.818	0.772
Tan	3	5786101	5786400	1.491	2.154	0.898	0.826
Tan	3	5996701	5997000	0.558	0.963	0.510	0.360

Tan	3	6034201	6034500	0.081	0.723	0.296	0.405
Tan	3	6116401	6116700	1.994	2.637	1.498	1.810
Tan	3	6133801	6134100	1.783	0.579	1.291	1.480
Tan	3	6320101	6320400	0.186	0.487	0.181	0.112
Tan	3	6341101	6341400	1.542	0.605	1.436	1.557
Tan	3	6341401	6341700	5.254	2.778	5.125	4.681
Tan	3	6649501	6649800	4.506	2.673	6.238	8.273
Tan	3	6964201	6964500	0.428	0.820	0.473	0.335
Tan	3	7086301	7086600	0.079	0.489	0.185	0.254
Tan	3	7113301	7113600	7.901	6.990	6.392	7.864
Tan	3	7145401	7145700	1.118	1.335	0.852	0.968
Tan	3	7149901	7150200	2.523	3.178	2.649	2.470
Tan	3	7297501	7297800	0.211	0.515	0.136	0.304
Tan	3	7469101	7469400	0.295	0.656	0.321	0.335
Tan	3	7618501	7618800	0.400	0.584	0.292	0.304
Tan	3	7747201	7747500	0.398	0.747	0.283	0.411
Tan	3	7899901	7900200	0.397	0.727	0.370	0.441
Tan	3	7918201	7918500	0.557	1.171	0.613	0.654
Tan	3	8275201	8275500	0.531	1.008	0.613	0.684
Tan	3	8324101	8324400	0.082	0.466	0.103	0.142
Tan	3	8340301	8340600	0.690	1.223	0.539	0.360
Tan	3	8340601	8340900	0.531	1.244	0.638	0.477
Tan	3	8475601	8475900	0.213	0.491	0.078	0.329
Tan	3	8475901	8476200	0.215	0.652	0.185	0.441
Tan	3	8511601	8511900	0.237	0.678	0.239	0.309
Tan	3	8628301	8628600	1.595	2.266	1.230	1.892
Tan	3	8676001	8676300	0.960	1.543	1.094	1.070
Tan	3	8801401	8801700	0.666	0.328	0.453	0.497
Tan	3	9040201	9040500	0.532	0.845	0.502	0.299
Tan	3	9067501	9067800	0.476	1.056	0.321	0.254
Tan	3	9067801	9068100	0.447	1.390	0.428	0.416
Tan	3	9183301	9183600	0.268	0.468	0.185	0.223
Tan	3	9226801	9227100	0.321	0.770	0.205	0.365
Tan	3	9233401	9233700	1.299	2.399	1.509	1.307
Tan	3	9549001	9549300	1.060	2.163	0.827	1.267
Tan	3	9562801	9563100	2.870	1.429	2.027	2.804
Tan	3	9652801	9653100	0.661	1.403	0.655	0.289
Tan	3	9654001	9654300	0.584	0.841	0.370	0.106
Tan	3	9654301	9654600	0.369	0.701	0.321	0.132
Tan	3	9756901	9757200	0.800	0.584	0.239	0.309
Tan	3	9824401	9824700	0.398	0.607	0.243	0.335
Tan	3	9924601	9924900	0.742	1.429	0.682	0.812
Tan	3	10041001	10041300	2.923	2.204	1.650	1.764
Tan	3	10268401	10268700	0.106	0.401	0.025	0.112
Tan	3	10371901	10372200	2.112	4.104	1.641	2.300
Tan	3	10426501	10426800	0.371	0.702	0.341	0.340
Tan	3	10493101	10493400	1.781	0.560	1.482	1.481
Tan	3	10569301	10569600	0.869	1.965	0.785	1.050
Tan	3	10575301	10575600	1.199	1.878	1.226	1.131
Tan	3	10581601	10581900	1.061	0.094	0.909	0.736
Tan	3	10805101	10805400	0.903	1.382	0.415	0.867
Tan	3	10845001	10845300	0.132	0.373	0.535	0.031
Tan	3	10930801	10931100	0.240	0.444	0.185	0.304
Tan	3	10961701	10962000	2.534	1.304	2.526	3.300
Tan	3	11021101	11021400	2.576	1.839	3.199	2.925
Tan	3	11688001	11688300	0.745	1.054	0.691	0.745
Tan	3	11704501	11704800	0.081	0.210	0.107	0.583
Tan	3	11706601	11706900	0.053	0.281	0.111	0.497
Tan	3	12029401	12029700	0.266	0.611	0.288	0.193
Tan	3	12029701	12030000	0.240	0.657	0.292	0.329
Tan	3	12061801	12062100	0.476	1.077	0.691	0.659
Tan	3	12334501	12334800	0.371	0.633	0.317	0.274
Tan	3	12354901	12355200	0.186	0.442	0.185	0.142
Tan	3	12357601	12357900	0.532	0.187	0.341	0.441
Tan	3	12417301	12417600	0.586	0.281	0.481	0.497
Tan	3	13127701	13128000	0.319	1.431	0.432	0.178
Tan	3	13128001	13128300	0.186	1.126	0.239	0.203

Tan	3	13139101	13139400	0.105	0.757	0.082	0.056
Tan	3	13182901	13183200	1.884	0.378	1.933	1.409
Tan	3	13385101	13385400	3.767	2.755	3.883	3.767
Tan	3	13439701	13440000	0.108	0.442	0.054	0.187
Tan	3	13500601	13500900	0.292	0.491	0.292	0.304
Tan	3	13649401	13649700	0.319	0.751	0.210	0.355
Tan	3	13658701	13659000	0.318	0.745	0.201	0.299
Tan	3	13753801	13754100	3.081	2.201	3.665	3.630
Tan	3	13789501	13789800	2.073	2.922	2.296	2.333
Tan	3	13796701	13797000	0.106	0.590	0.189	0.187
Tan	3	14033101	14033400	0.500	1.127	0.481	0.441
Tan	3	14107501	14107800	0.134	0.489	0.152	0.112
Tan	3	14159101	14159400	0.745	1.071	0.794	0.553
Tan	3	14196001	14196300	0.398	0.676	0.424	0.497
Tan	3	14245801	14246100	0.850	1.455	0.881	0.725
Tan	3	14259601	14259900	0.108	0.676	0.189	0.178
Tan	3	14316901	14317200	0.136	0.957	0.288	0.198
Tan	3	14343301	14343600	0.160	0.637	0.205	0.198
Tan	3	14397301	14397600	0.240	0.493	0.346	0.025
Tan	3	14454301	14454600	0.799	1.172	0.774	0.243
Tan	3	14535301	14535600	0.611	1.026	0.428	0.385
Tan	3	14780401	14780700	0.981	0.277	1.300	1.374
Tan	3	15066001	15066300	0.161	0.536	0.078	0.243
Tan	3	15080701	15081000	0.476	1.481	0.740	0.648
Tan	3	15590401	15590700	0.558	0.281	0.160	0.142
Tan	3	15891301	15891600	5.185	4.590	3.817	4.016
Tan	3	16203001	16203300	0.829	1.687	0.727	0.690
Tan	3	16430701	16431000	0.292	0.564	0.239	0.173
Tan	3	16617001	16617300	0.960	1.826	0.671	0.558
Tan	3	16670701	16671000	0.795	1.399	0.823	0.533
Tan	3	16713001	16713300	4.101	3.411	4.813	2.586
Tan	3	16787701	16788000	0.186	0.684	0.185	0.182
Tan	3	16860001	16860300	0.132	0.395	0.054	0.117
Tan	3	16887601	16887900	0.505	0.348	0.132	0.056
Tan	3	17121901	17122200	0.161	0.661	0.082	0.086
Tan	3	17168401	17168700	0.235	1.425	0.259	0.178
Tan	3	17168701	17169000	0.136	0.936	0.156	0.142
Tan	3	17200201	17200500	0.268	0.605	0.181	0.304
Tan	3	17497801	17498100	0.266	0.538	0.341	0.274
Tan	3	17618701	17619000	0.316	0.775	0.296	0.340
Tan	3	17636401	17636700	0.108	0.420	0.107	0.223
Tan	3	17701501	17701800	0.690	0.258	0.613	0.654
Tan	3	17736901	17737200	0.479	1.032	0.580	0.603
Tan	3	17802001	17802300	0.108	0.511	0.189	0.198
Tan	3	17804401	17804700	0.237	0.706	0.218	0.218
Tan	3	17850301	17850600	0.160	0.684	0.205	0.198
Tan	3	18220801	18221100	0.134	0.538	0.263	0.254
Tan	3	18260701	18261000	0.292	0.446	0.214	0.193
Tan	3	18261001	18261300	0.213	0.468	0.239	0.223
Tan	3	18322501	18322800	5.985	7.728	6.581	6.835
Tan	3	18519001	18519300	0.265	0.446	0.156	0.081
Tan	3	18544201	18544500	2.526	0.991	2.028	1.988
Tan	3	18669601	18669900	0.163	0.775	0.312	0.299
Tan	3	18742801	18743100	0.242	0.538	0.136	0.254
Tan	3	18808801	18809100	0.211	0.493	0.662	0.274
Tan	3	18840901	18841200	0.294	0.657	0.189	0.360
Tan	3	18967801	18968100	0.294	0.468	0.160	0.254
Tan	3	19005901	19006200	1.192	1.663	1.217	0.776
Tan	3	19031701	19032000	0.266	0.631	0.185	0.203
Tan	3	19141201	19141500	3.533	1.940	3.337	3.291
Tan	3	19237801	19238100	0.345	0.536	0.243	0.304
Tan	3	19785601	19785900	0.160	0.607	0.346	0.309
Tan	3	19947901	19948200	0.136	0.350	0.107	0.603
Tan	3	19979701	19980000	1.329	2.173	1.674	1.435
Tan	3	20258101	20258400	0.239	0.444	0.181	0.142
Tan	3	20267401	20267700	0.105	0.446	0.127	0.167
Tan	3	20343601	20343900	0.423	1.028	0.613	0.644

Tan	3	20376901	20377200	0.160	0.517	0.181	0.137
Tan	3	20691601	20691900	0.081	0.371	0.136	0.081
Tan	3	21149401	21149700	0.239	0.682	0.321	0.167
Tan	3	21177901	21178200	1.828	2.781	1.587	1.654
Tan	3	21648901	21649200	0.718	1.030	0.711	0.628
Tan	3	22103701	22104000	0.557	0.116	0.526	0.533
Tan	3	22347601	22347900	0.239	0.513	0.210	0.360
Tan	3	23022301	23022600	0.294	0.513	0.210	0.335
Tan	3	23046301	23046600	0.211	0.511	0.218	0.223
Tan	3	23394301	23394600	1.358	0.633	0.205	0.132
Tan	3	23859001	23859300	0.211	0.676	0.263	0.421
Tan	3	23974501	23974800	0.321	0.891	0.390	0.405
Tan	3	23993401	23993700	0.345	0.792	0.325	0.218
Tan	3	24190801	24191100	0.800	1.098	0.633	0.553
Tan	3	24232201	24232500	0.186	0.420	0.029	0.248
Tan	3	24261601	24261900	0.637	0.747	0.453	0.634
Tan	3	24358201	24358500	1.036	1.884	1.157	1.105
Tan	3	24379201	24379500	0.158	0.539	0.194	0.198
Tan	3	24907501	24907800	0.186	0.607	0.194	0.132
Tan	3	25473001	25473300	0.239	0.470	0.267	0.223
Tan	3	25514401	25514700	0.744	1.075	0.604	0.466
Tan	3	25721101	25721400	0.344	0.586	0.346	0.218
Tan	3	25826401	25826700	0.239	0.420	0.136	0.056
Tan	3	26020201	26020500	0.347	0.657	0.214	0.254
Tan	3	26030401	26030700	0.398	0.633	0.185	0.411
Tan	3	26175901	26176200	1.808	1.613	1.966	1.166
Tan	3	26754001	26754300	0.374	0.725	1.139	0.365
Tan	3	27180301	27180600	0.428	1.103	0.526	0.644
Tan	3	27180601	27180900	0.215	0.773	0.259	0.340
Tan	3	27312901	27313200	0.186	0.444	0.210	0.218
Tan	3	27447601	27447900	0.560	0.701	0.374	0.416
Tan	3	27928201	27928500	0.957	1.968	0.921	0.968
Tan	3	27932101	27932400	0.347	0.562	0.745	0.304
Tan	3	28035601	28035900	0.110	0.950	0.214	0.213
Tan	3	28178401	28178700	0.776	1.405	0.555	0.943
Tan	3	28598101	28598400	0.373	0.704	0.263	0.309
Tan	3	28716901	28717200	0.210	0.770	0.243	0.284
Tan	3	28718101	28718400	0.381	1.193	0.301	0.289
Tan	3	28730701	28731000	0.165	0.987	0.325	0.319
Tan	3	29279701	29280000	0.132	0.723	0.292	0.315
Tan	3	29922901	29923200	0.187	0.654	0.317	0.411
Tan	3	31039501	31039800	0.053	0.493	0.127	0.279
Tan	3	31305901	31306200	1.089	0.772	0.502	0.284
Tan	3	32141401	32141700	0.848	2.197	0.604	0.846
Tan	3	32141701	32142000	1.011	2.315	0.856	1.060
Tan	3	32145901	32146200	0.752	2.045	0.789	0.897
Tan	3	32146201	32146500	0.529	1.343	0.477	0.441
Tan	3	32447101	32447400	0.134	0.567	0.136	0.112
Tan	3	32466301	32466600	2.925	1.985	1.460	2.551
Tan	3	32512501	32512800	1.145	0.843	1.201	1.658
Tan	3	32654401	32654700	0.265	0.586	0.214	0.254
Tan	3	32734801	32735100	0.690	0.354	0.765	0.740
Tan	3	32783101	32783400	0.158	0.493	0.132	0.132
Tan	3	33227101	33227400	0.505	1.034	0.403	0.360
Tan	3	33227401	33227700	0.373	0.657	0.341	0.223
Tan	3	33385501	33385800	0.632	1.377	0.544	0.664
Tan	3	33574501	33574800	0.239	0.534	0.156	0.137
Tan	3	33790801	33791100	0.263	0.981	0.424	0.355
Tan	3	33942601	33942900	0.160	0.401	0.132	0.142
Tan	3	34084201	34084500	0.215	0.699	0.267	0.441
Tan	3	34348501	34348800	0.452	0.657	0.160	0.274
Tan	3	34366201	34366500	0.800	1.431	0.395	0.254
Tan	3	34382101	34382400	0.240	0.468	0.292	0.279
Tan	3	34556701	34557000	1.971	2.663	1.378	1.958
Tan	3	35097001	35097300	0.666	1.341	0.745	0.695
Tan	3	35196301	35196600	0.161	0.395	0.185	0.167
Tan	3	35421001	35421300	0.479	0.609	0.531	0.385

Tan	3	35515801	35516100	2.023	0.465	0.881	0.983
Tan	3	35742301	35742600	0.507	0.303	0.111	0.112
Tan	3	35771701	35772000	0.079	0.446	0.181	0.193
Tan	3	36005401	36005700	0.108	0.980	0.111	0.238
Tan	3	36026401	36026700	0.402	0.863	0.395	0.299
Tan	3	36300301	36300600	0.160	1.296	0.441	0.355
Tan	3	36310201	36310500	0.161	0.446	0.132	0.173
Tan	3	36953101	36953400	0.053	0.444	0.234	0.162
Tan	3	37336801	37337100	0.560	0.884	0.560	0.553
Tan	3	37373101	37373400	0.239	0.517	0.132	0.223
Tan	3	37575001	37575300	0.052	0.704	0.234	0.340
Tan	3	37618801	37619100	0.081	0.446	0.029	0.076
Tan	3	37771801	37772100	0.187	0.444	0.189	0.025
Tan	3	37772101	37772400	1.300	1.637	0.769	0.877
Tan	3	38086201	38086500	0.213	0.468	0.152	0.193
Tan	3	38269201	38269500	0.132	0.893	0.346	0.431
Tan	3	38504401	38504700	0.699	1.334	0.584	0.391
Tan	3	38557801	38558100	1.912	2.589	1.761	2.048
Tan	3	38570701	38571000	0.158	0.629	0.185	0.137
Tan	3	38703301	38703600	2.020	2.452	1.933	2.094
Tan	3	38813101	38813400	0.505	0.654	0.506	0.553
Tan	3	38893201	38893500	0.450	0.845	0.403	0.157
Tan	3	38942401	38942700	0.773	0.421	0.662	0.751
Tan	3	39038101	39038400	1.541	2.461	1.398	1.638
Tan	3	39038401	39038700	0.237	0.654	0.296	0.340
Tan	3	39319201	39319500	0.079	0.472	0.111	0.106
Tan	3	39635701	39636000	0.290	0.772	0.399	0.355
Tan	3	39823801	39824100	0.186	0.444	0.687	0.086
Tan	3	39910501	39910800	1.565	2.438	1.235	1.212
Tan	3	39997501	39997800	0.742	1.929	0.781	0.837
Tan	3	40194301	40194600	0.373	0.841	0.671	1.024
Tan	3	41325001	41325300	0.052	0.545	0.054	0.051
Tan	3	41755801	41756100	0.426	1.122	0.588	0.558
Tan	3	41880001	41880300	0.371	0.768	0.210	0.466
Tan	3	42143701	42144000	0.266	0.493	0.156	0.274
Tan	3	42412501	42412800	0.453	0.633	0.321	0.274
Tan	3	42652801	42653100	0.687	1.197	0.453	0.690
Tan	3	42717301	42717600	0.558	0.818	0.374	0.466
Tan	3	42757201	42757500	0.316	1.036	0.252	0.142
Tan	3	42852901	42853200	1.544	1.124	1.456	1.831
Tan	3	42863701	42864000	0.529	2.010	0.646	0.934
Tan	3	42970201	42970500	0.507	0.865	0.539	0.477
Tan	3	43279801	43280100	0.266	0.633	0.399	0.228
Tan	3	43355101	43355400	0.265	0.539	0.239	0.223
Tan	3	43758901	43759200	0.268	0.656	0.346	0.446
Tan	3	43788901	43789200	0.321	0.725	0.424	0.324
Tan	3	43802701	43803000	0.186	0.511	0.082	0.117
Tan	3	43915501	43915800	0.319	0.607	0.749	0.416
Tan	3	44065501	44065800	0.558	0.933	0.720	0.664
Tan	3	45061201	45061500	0.079	0.468	0.082	0.228
Tan	3	45938401	45938700	0.531	0.818	0.156	0.963
Tan	3	46329001	46329300	4.917	5.396	6.541	6.860
Tan	3	46471801	46472100	0.134	0.489	0.127	0.218
Tan	3	46697101	46697400	0.479	0.818	0.379	0.304
Tan	3	46697401	46697700	0.345	0.702	0.189	0.238
Tan	3	46731601	46731900	0.529	0.936	0.239	0.477
Tan	3	47368201	47368500	0.450	0.820	0.374	0.350
Tan	3	48105601	48105900	0.745	1.032	0.740	0.441
Tan	3	48675001	48675300	0.373	0.914	0.408	0.396
Tan	3	49313701	49314000	0.826	0.350	0.613	0.527
Tan	3	49350001	49350300	0.186	0.395	0.239	0.527
Tan	3	50063101	50063400	0.081	0.418	0.136	0.162
Tan	3	50178001	50178300	2.044	1.053	1.431	1.998
Tan	3	50830201	50830500	0.345	0.560	0.111	0.274
Tan	3	51117001	51117300	0.266	0.678	0.852	0.918
Tan	3	51416701	51417000	0.187	0.442	0.263	0.659
Tan	3	52829101	52829400	0.616	1.923	1.044	0.902

Tan	3	53044501	53044800	0.797	1.026	0.691	0.472
Tan	3	54078001	54078300	1.513	1.077	1.806	1.836
Tan	3	54311401	54311700	0.955	1.191	0.716	0.725
Tan	3	54861901	54862200	0.611	0.232	0.609	0.796
Tan	3	55045501	55045800	0.794	0.094	0.938	0.771
Tan	3	55931701	55932000	0.184	0.560	0.263	0.187
Tan	3	56312701	56313000	0.398	0.865	0.453	0.695
Tan	3	56597101	56597400	0.477	1.410	0.288	0.654
Tan	3	56597401	56597700	0.452	1.551	0.361	0.466
Tan	3	56766901	56767200	0.373	0.701	0.087	0.466
Tan	3	56844001	56844300	0.186	0.446	0.078	0.274
Tan	3	56949601	56949900	0.266	0.611	0.214	0.228
Tan	3	57133201	57133500	0.452	0.725	0.419	0.391
Tan	3	57294001	57294300	0.558	0.961	0.424	0.547
Tan	3	57751801	57752100	0.132	0.635	0.292	0.274
Tan	3	57825601	57825900	0.160	0.491	0.399	0.385
Tan	3	57855601	57855900	0.500	1.706	1.266	0.441
Tan	3	57978601	57978900	0.053	0.444	0.243	0.086
Tan	3	58491901	58492200	0.371	0.936	0.481	0.547
Tan	3	58721701	58722000	0.052	0.513	0.185	0.279
Tan	3	58725601	58725900	0.160	0.444	0.321	0.274
Tan	3	58805401	58805700	0.432	1.538	0.646	0.284
Tan	3	58830001	58830300	2.796	4.081	2.423	3.026
Tan	3	59378101	59378400	0.265	0.794	0.263	0.345
Tan	3	59594401	59594700	0.349	1.045	0.272	0.355
Tan	3	59844901	59845200	1.116	0.796	1.090	0.913
Tan	3	60124801	60125100	0.589	1.098	0.588	0.466
Tan	3	60125101	60125400	0.373	0.952	0.399	0.416
Tan	3	60298201	60298500	0.294	0.845	0.399	0.421
Tan	3	60632701	60633000	0.477	0.165	0.448	0.502
Tan	3	60696601	60696900	0.373	0.727	0.272	0.360
Tan	3	60769201	60769500	0.823	1.635	0.765	0.892
Tan	3	60847501	60847800	0.292	0.493	0.181	0.193
Tan	3	60909301	60909600	0.079	0.395	0.185	0.081
Tan	3	61042501	61042800	0.584	0.933	0.687	0.603
Tan	3	61448101	61448400	0.534	0.375	0.185	0.137
Tan	3	61474801	61475100	0.905	1.566	2.167	0.923
Tan	3	61645201	61645500	0.269	1.099	0.745	0.659
Tan	3	61916401	61916700	0.455	0.820	0.399	0.527
Tan	3	61942501	61942800	3.878	4.652	3.812	3.975
Tan	3	62027701	62028000	1.542	1.028	2.049	1.896
Tan	3	62044201	62044500	2.531	1.500	4.246	4.174
Tan	3	62044801	62045100	1.812	3.405	0.923	0.568
Tan	3	62067001	62067300	0.708	2.468	0.681	0.178
Tan	3	62074801	62075100	0.453	0.068	0.622	0.852
Tan	3	62090701	62091000	1.194	2.322	1.073	0.969
Tan	3	62109901	62110200	0.508	0.116	0.453	0.765
Tan	3	62166901	62167200	0.268	0.749	0.374	0.375
Tan	3	62223301	62223600	2.584	1.236	2.241	1.917
Tan	3	62223601	62223900	5.366	1.973	5.740	5.319
Tan	3	62279701	62280000	1.571	0.747	0.979	1.222
Tan	3	62341501	62341800	0.371	0.513	0.185	0.360
Tan	3	62488801	62489100	0.187	0.536	0.181	0.254
Tan	3	62723101	62723400	0.344	0.772	0.399	0.335
Tan	3	62773501	62773800	0.026	0.515	0.160	0.228
Tan	3	63181501	63181800	0.371	0.562	0.267	0.274
Tan	3	63271201	63271500	0.879	2.343	1.494	1.425
Tan	3	63404101	63404400	2.286	1.777	2.303	2.286
Tan	3	63582601	63582900	0.561	1.191	0.600	0.228
Tan	3	63634801	63635100	0.666	0.375	0.856	0.614
Tan	3	63935101	63935400	0.345	0.118	0.321	0.553
Tan	3	63935701	63936000	0.398	0.562	0.292	0.198
Tan	3	66163501	66163800	0.215	0.723	0.243	0.198
Tan	3	66615001	66615300	0.027	0.517	0.058	0.076
Tan	3	67012501	67012800	0.266	0.466	0.239	0.193
Tan	3	67749901	67750200	0.586	0.257	0.132	0.497
Tan	3	67800301	67800600	0.423	0.818	0.395	0.274

Tan	3	67923301	67923600	0.503	1.152	0.667	0.547
Tan	3	68157001	68157300	0.160	0.472	0.132	0.137
Tan	3	68490301	68490600	0.853	1.313	0.740	0.837
Tan	3	68518201	68518500	0.239	0.118	0.477	0.223
Tan	3	68692201	68692500	0.560	0.255	0.481	0.497
Tan	3	68720701	68721000	0.666	1.240	0.769	0.801
Tan	3	68855701	68856000	0.426	1.053	0.535	0.583
Tan	3	68969701	68970000	3.055	1.774	2.760	3.255
Tan	3	69217201	69217500	0.134	0.444	0.132	0.101
Tan	3	69281401	69281700	0.266	0.493	0.214	0.187
Tan	3	69287101	69287400	0.900	1.431	0.736	0.720
Tan	3	69305101	69305400	0.848	1.547	0.700	0.958
Tan	3	69316201	69316500	0.158	0.515	0.111	0.162
Tan	3	69670201	69670500	0.347	0.583	0.185	0.254
Tan	3	69778801	69779100	0.428	0.867	1.337	0.527
Tan	3	70079701	70080000	0.160	0.494	0.239	0.193
Tan	3	70230601	70230900	0.268	0.699	0.370	0.223
Tan	3	70244701	70245000	0.240	0.742	0.296	0.162
Tan	3	70273201	70273500	0.744	1.008	0.642	0.446
Tan	3	70422601	70422900	0.160	0.395	0.107	0.198
Tan	3	70549801	70550100	0.158	0.744	0.198	0.218
Tan	3	70693201	70693500	2.397	0.682	2.192	2.082
Tan	3	70693501	70693800	1.303	0.213	1.275	0.978
Tan	3	71266501	71266800	0.398	0.584	0.296	0.360
Tan	3	71334001	71334300	0.213	0.468	0.214	0.254
Tan	3	71428801	71429100	0.321	0.611	2.151	1.364
Tan	3	71792101	71792400	0.134	0.513	0.214	0.157
Tan	3	72485701	72486000	0.129	0.562	0.136	0.112
Tan	3	73821601	73821900	0.294	0.635	0.317	0.279
Tan	3	73973701	73974000	0.158	0.395	0.132	0.137
Tan	3	74112301	74112600	0.532	0.935	0.564	0.294
Tan	3	74112601	74112900	0.689	1.188	0.539	0.380
Tan	3	74383201	74383500	0.292	0.674	0.243	0.304
Tan	3	74556901	74557200	0.374	0.890	0.366	0.477
Tan	3	74851501	74851800	2.509	0.399	2.967	1.642
Tan	3	74955301	74955600	0.345	0.607	0.403	0.218
Tan	3	74980801	74981100	0.108	0.515	0.210	0.223
Tan	3	75609901	75610200	0.077	0.423	0.054	0.086
Tan	3	75652801	75653100	0.213	0.468	0.288	0.218
Tan	3	75819301	75819600	0.749	2.206	0.675	0.528
Tan	3	75862501	75862800	0.481	1.599	0.622	0.315
Tan	3	75871201	75871500	1.941	1.025	2.722	2.302
Tan	3	75904501	75904800	0.398	1.433	0.152	0.376
Tan	3	76016701	76017000	0.187	0.444	0.074	0.198
Tan	3	76216801	76217100	0.373	0.607	0.074	0.385
Tan	3	76765501	76765800	0.240	0.538	0.181	0.335
Tan	3	77141701	77142000	0.210	0.729	0.160	0.309
Tan	3	77190901	77191200	0.611	0.983	0.497	0.522
Tan	3	77194201	77194500	0.723	1.281	0.736	0.695
Tan	3	77273101	77273400	0.928	0.347	1.280	0.898
Tan	3	77321101	77321400	0.132	0.564	0.218	0.187
Tan	3	77321401	77321700	0.105	0.401	0.078	0.051
Tan	3	77338201	77338500	0.290	0.515	0.317	0.248
Tan	3	77593801	77594100	0.773	0.538	0.774	0.745
Tan	3	77702101	77702400	3.888	2.133	3.250	2.702
Tan	3	77829901	77830200	0.692	0.985	0.564	0.553
Tan	3	77964901	77965200	0.161	0.938	0.428	0.457
Tan	3	78071101	78071400	1.571	2.171	1.465	1.273
Tan	3	78074401	78074700	1.358	1.708	1.300	1.318
Tan	3	78094801	78095100	0.266	0.468	0.181	0.051
Tan	3	78395101	78395400	0.745	0.375	0.189	0.086
Tan	3	78529501	78529800	0.215	0.586	0.214	0.309
Tan	3	79001701	79002000	0.345	0.161	0.448	0.608
Tan	3	79195501	79195800	0.423	1.034	0.468	0.502
Tan	3	79401601	79401900	1.007	1.631	1.144	1.166
Tan	3	79401901	79402200	1.036	1.424	0.798	0.887
Tan	3	79500001	79500300	0.347	0.978	0.214	0.370

Tan	3	79613101	79613400	0.265	0.796	0.432	0.355
Tan	3	80225701	80226000	1.171	0.468	0.896	1.197
Tan	3	80307601	80307900	0.132	0.421	0.535	0.167
Tan	3	80610301	80610600	1.094	1.734	1.008	0.958
Tan	3	80617501	80617800	0.507	1.101	0.526	0.781
Tan	3	81003301	81003600	0.134	0.515	0.127	0.056
Tan	3	81256501	81256800	0.477	0.678	0.267	0.360
Tan	3	81573901	81574200	0.505	1.289	0.662	0.771
Tan	3	81574201	81574500	0.503	0.818	0.506	0.517
Tan	3	81943801	81944100	0.450	0.773	0.346	0.527
Tan	3	82159201	82159500	0.268	0.729	0.254	0.193
Tan	3	82194001	82194300	0.187	0.493	0.189	0.228
Tan	3	82244401	82244700	0.560	1.098	0.687	0.781
Tan	3	82557301	82557600	0.560	0.978	0.502	0.243
Tan	3	82774201	82774500	0.161	0.420	0.132	0.137
Tan	3	83037601	83037900	0.469	2.090	0.651	0.557
Tan	3	83175301	83175600	0.398	0.142	0.374	0.527
Tan	3	83263801	83264100	0.398	0.955	0.395	0.385
Tan	3	83264101	83264400	0.319	0.817	0.453	0.329
Tan	3	83297101	83297400	0.982	0.583	0.959	0.893
Tan	3	84398101	84398400	0.532	0.279	0.564	0.522
Tan	3	84432601	84432900	0.345	0.515	0.243	0.137
Tan	3	84696001	84696300	0.957	1.642	1.037	0.877
Tan	3	84764101	84764400	0.186	0.466	0.234	0.218
Tan	3	85200901	85201200	0.108	0.487	0.054	0.076
Tan	3	85590601	85590900	0.082	0.536	0.107	0.162
Tan	3	85658101	85658400	0.265	0.657	0.160	0.213
Tan	3	85882201	85882500	2.588	4.311	1.935	2.302
Tan	3	85882501	85882800	1.973	3.453	1.687	1.866
Tan	3	86005801	86006100	0.161	0.515	0.296	0.248
Tan	3	86113201	86113500	0.189	0.772	0.196	0.106
Tan	3	86166001	86166300	0.426	0.985	0.408	0.233
Tan	3	86291401	86291700	0.720	1.953	0.597	0.522
Tan	3	86298001	86298300	0.316	1.261	0.642	0.466
Tan	3	86298301	86298600	0.479	1.618	0.992	0.786
Tan	3	86332201	86332500	0.426	0.631	0.312	0.416
Tan	3	86754001	86754300	0.473	1.579	0.568	0.583
Tan	3	86863801	86864100	0.321	0.865	0.292	0.405
Tan	3	87024301	87024600	0.398	0.629	0.082	0.304
Tan	3	87247201	87247500	0.186	0.584	0.453	0.304
Tan	3	87753601	87753900	0.156	0.635	0.029	0.086
Tan	3	87911701	87912000	0.189	0.730	0.165	0.182
Tan	3	87933301	87933600	1.673	0.637	1.371	1.303
Tan	3	88675201	88675500	0.640	1.311	0.457	0.472
Tan	3	88880701	88881000	1.168	0.701	1.016	0.999
Tan	3	89237701	89238000	0.424	0.701	0.453	0.441
Tan	3	90360301	90360600	0.082	0.560	0.132	0.101
Tan	3	90759601	90759900	0.105	0.423	0.185	0.142
Tan	3	90799501	90799800	0.106	0.444	0.736	0.187
Tan	3	90886801	90887100	0.134	0.869	0.272	0.258
Tan	3	90941701	90942000	0.294	0.609	0.205	0.117
Tan	3	90942001	90942300	2.854	3.898	2.858	1.415
Tan	3	91045801	91046100	0.160	0.586	0.317	0.304
Tan	3	91158301	91158600	0.426	0.676	0.239	0.248
Tan	3	91202701	91203000	0.160	0.491	0.074	0.106
Tan	3	91427401	91427700	0.962	1.946	1.070	1.055
Tan	3	91485301	91485600	0.136	0.494	0.132	0.132
Tan	3	91631401	91631700	0.082	0.448	0.054	0.000
Tan	3	91647901	91648200	0.160	0.541	0.160	0.248
Tan	3	91698301	91698600	0.294	1.163	0.288	0.506
Tan	3	91704901	91705200	0.398	0.515	0.292	0.720
Tan	3	92135401	92135700	0.266	0.187	0.321	0.527
Tan	3	92386801	92387100	0.239	0.654	0.247	0.101
Tan	3	92468101	92468400	0.634	1.317	0.403	0.441
Tan	3	92873101	92873400	0.215	0.745	0.370	0.329
Tan	3	92896501	92896800	0.663	1.424	0.740	0.452
Tan	3	92962501	92962800	0.186	0.729	0.321	0.279

Tan	3	92986501	92986800	3.457	2.285	3.603	3.868
Tan	3	93098401	93098700	0.536	1.053	0.510	0.664
Tan	3	93197101	93197400	1.699	2.365	1.538	1.475
Tan	3	93320401	93320700	0.749	1.498	0.649	0.457
Tan	3	93339301	93339600	1.358	2.154	0.674	1.009
Tan	3	93387301	93387600	1.623	0.914	1.703	1.293
Tan	3	93486901	93487200	1.996	1.028	2.073	2.206
Tan	3	93548701	93549000	4.088	4.892	3.518	3.899
Tan	3	93599101	93599400	0.268	0.491	0.160	0.248
Tan	3	93897001	93897300	0.239	1.011	0.127	0.086
Tan	3	94035001	94035300	0.131	0.536	0.239	0.193
Tan	3	94091701	94092000	0.132	0.702	0.354	0.228
Tan	3	94431001	94431300	0.240	0.539	0.239	0.198
Tan	3	94567801	94568100	0.453	0.820	0.292	0.299
Tan	3	94677001	94677300	0.318	0.607	0.346	0.173
Tan	3	94849801	94850100	0.721	1.680	0.856	1.055
Tan	3	94884601	94884900	0.529	0.236	0.506	0.720
Tan	3	94965001	94965300	0.694	1.172	0.370	0.801
Tan	3	94975801	94976100	0.829	1.805	0.538	0.730
Tan	3	94976101	94976400	0.027	0.657	0.049	0.167
Tan	3	94995001	94995300	1.199	1.687	1.003	1.095
Tan	3	95077201	95077500	0.797	0.491	0.267	0.913
Tan	3	95126401	95126700	0.745	0.465	0.930	0.781
Tan	3	95207101	95207400	0.455	0.841	0.296	0.578
Tan	3	95467501	95467800	0.106	0.581	0.239	0.086
Tan	3	95591101	95591400	0.240	0.536	0.185	0.187
Tan	3	95747401	95747700	1.966	2.317	1.806	1.714
Tan	3	95763001	95763300	1.555	3.714	2.419	2.697
Tan	3	95837101	95837400	0.319	0.562	0.156	0.391
Tan	3	95899801	95900100	0.266	0.605	0.272	0.223
Tan	3	96084901	96085200	0.323	1.155	0.288	0.213
Tan	3	96130801	96131100	0.266	0.468	0.185	0.132
Tan	3	96294901	96295200	0.294	0.588	0.054	0.304
Tan	3	96522301	96522600	0.105	0.541	0.132	0.101
Tan	3	96864301	96864600	0.960	2.017	1.237	0.898
Tan	3	96914401	96914700	0.132	0.442	0.189	0.112
Tan	3	97071001	97071300	0.105	0.470	0.132	0.106
Tan	3	97127401	97127700	2.100	2.575	1.580	1.876
Tan	3	97268101	97268400	0.797	2.268	1.083	1.339
Tan	3	97268401	97268700	0.294	0.633	0.370	0.391
Tan	3	97333201	97333500	0.760	2.308	0.490	0.857
Tan	3	97337401	97337700	0.639	1.008	0.419	0.517
Tan	3	97475401	97475700	0.639	1.283	0.711	0.791
Tan	3	97509001	97509300	1.042	0.609	1.275	0.162
Tan	3	97560601	97560900	1.115	1.968	0.979	1.064
Tan	3	97628101	97628400	2.121	1.358	2.151	2.531
Tan	3	97707001	97707300	0.213	0.729	0.317	0.309
Tan	3	98099101	98099400	0.507	0.352	0.132	0.142
Tan	3	98316301	98316600	0.160	0.487	0.156	0.106
Tan	3	98336701	98337000	0.797	0.515	1.012	0.776
Tan	3	98442001	98442300	0.318	0.562	0.346	0.162
Tan	3	98458201	98458500	1.037	2.199	1.291	1.273
Tan	3	98667601	98667900	0.586	0.702	0.428	0.472
Tan	3	98681701	98682000	0.318	0.704	0.296	0.309
Tan	3	98696701	98697000	0.347	0.773	0.448	0.360
Tan	3	98931901	98932200	0.448	1.436	0.444	0.533
Tan	3	98944801	98945100	0.187	0.631	0.379	0.304
Tan	3	99104701	99105000	1.673	1.032	1.522	1.982
Tan	3	99165301	99165600	0.665	0.916	0.667	0.335
Tan	3	99198301	99198600	0.290	0.657	0.263	0.268
Tan	3	99391201	99391500	0.424	0.796	0.312	0.193
Tan	3	99529501	99529800	0.213	0.491	0.370	0.223
Tan	3	99819001	99819300	2.733	3.766	2.488	2.940
Tan	3	99819301	99819600	2.236	3.319	2.354	2.494
Tan	3	99872401	99872700	0.160	0.729	0.205	0.162
Tan	3	99903901	99904200	1.066	2.562	0.869	1.410
Tan	3	99959701	99960000	0.106	0.448	0.165	0.167

Tan	3	100124701	100125000	0.292	0.470	0.082	0.304
Tan	3	100125601	100125900	1.902	3.859	0.954	1.587
Tan	3	100125901	100126200	0.803	1.802	0.419	0.517
Tan	3	100158301	100158600	0.366	1.188	0.296	0.228
Tan	3	100254001	100254300	0.507	0.959	0.638	0.654
Tan	3	100546201	100546500	0.160	0.519	0.132	0.132
Tan	3	100637101	100637400	0.160	0.395	0.078	0.112
Tan	3	100753801	100754100	1.465	0.955	1.572	1.566
Tan	3	100839601	100839900	0.158	0.468	0.074	0.218
Tan	3	101016001	101016300	0.529	1.081	0.564	0.466
Tan	3	101016301	101016600	0.552	1.360	0.560	0.466
Tan	3	101197801	101198100	0.452	0.725	0.160	0.309
Tan	3	101209801	101210100	0.131	0.491	0.136	0.106
Tan	3	101343601	101343900	0.081	0.706	0.395	0.411
Tan	3	101456701	101457000	0.376	1.172	0.232	0.248
Tan	3	101592301	101592600	0.586	0.234	0.058	0.690
Tan	3	101642401	101642700	0.132	0.515	0.140	0.147
Tan	3	101681701	101682000	0.823	1.197	0.847	0.720
Tan	3	101682901	101683200	0.052	0.893	0.403	0.335
Tan	3	101768101	101768400	0.560	0.326	0.662	0.659
Tan	3	101803801	101804100	5.935	6.840	4.722	5.800
Tan	3	101893501	101893800	1.333	2.060	1.395	1.181
Tan	3	101999401	101999700	0.186	0.656	0.379	0.391
Tan	3	102018601	102018900	0.106	0.472	0.000	0.076
Tan	3	102301501	102301800	0.393	1.079	0.346	0.238
Tan	3	102432901	102433200	0.027	0.442	0.210	0.198
Tan	3	102518101	102518400	0.079	0.536	0.074	0.112
Tan	3	102570001	102570300	0.908	1.309	0.823	0.436
Tan	3	103102501	103102800	0.106	0.491	0.123	0.081
Tan	3	103122601	103122900	0.552	1.714	0.789	0.562
Tan	3	103158601	103158900	1.170	2.133	1.180	0.837
Tan	3	103210801	103211100	0.636	0.957	0.506	0.659
Tan	3	103356001	103356300	0.235	0.706	0.218	0.223
Tan	3	103356601	103356900	1.065	1.311	0.954	1.055
Tan	3	103390501	103390800	1.729	1.916	1.300	1.552
Tan	3	103464901	103465200	0.161	0.420	0.185	0.142
Tan	3	103472401	103472700	2.152	2.813	1.415	1.613
Tan	3	103603801	103604100	1.566	0.618	1.518	1.546
Tan	3	103772101	103772400	1.249	1.030	0.633	1.298
Tan	3	103824301	103824600	0.319	0.489	0.613	0.248
Tan	3	104235301	104235600	0.637	0.987	0.296	0.659
Tan	3	104276401	104276700	1.862	0.862	1.674	1.623
Tan	3	104472001	104472300	1.273	0.382	1.699	1.319
Tan	3	104474401	104474700	0.323	0.773	0.247	0.106
Tan	3	104603101	104603400	1.542	2.133	1.366	1.166
Tan	3	104610601	104610900	2.605	1.963	1.723	2.347
Tan	3	104620501	104620800	1.650	0.955	1.723	1.790
Tan	3	104635501	104635800	0.163	0.607	0.239	0.187
Tan	3	104641201	104641500	0.347	0.918	0.263	0.472
Tan	3	104709301	104709600	1.278	1.776	1.288	0.938
Tan	3	104816401	104816700	1.863	2.815	1.081	1.780
Tan	3	104816701	104817000	2.075	3.330	1.929	1.836
Tan	3	104817901	104818200	0.213	0.470	0.156	0.167
Tan	3	104864401	104864700	0.318	0.777	0.292	0.243
Tan	3	104888701	104889000	0.426	0.914	0.272	0.385
Tan	3	104965501	104965800	1.645	0.511	1.222	1.307
Tan	3	105005101	105005400	0.452	1.202	0.395	0.461
Tan	3	105168901	105169200	0.079	0.607	0.247	0.243
Tan	3	105213901	105214200	0.186	0.633	0.214	0.284
Tan	3	105241801	105242100	0.081	0.371	0.029	0.086
Tan	3	105245401	105245700	0.957	2.231	0.662	0.796
Tan	3	105255301	105255600	0.507	2.150	0.723	0.822
Tan	3	105279901	105280200	0.132	0.487	0.103	0.193
Tan	3	105290101	105290400	0.210	0.654	0.169	0.218
Tan	3	105291901	105292200	6.411	2.321	6.901	7.551
Tan	3	105429901	105430200	0.240	0.468	0.181	0.112
Tan	3	105584401	105584700	0.452	0.725	0.453	0.578

Tan	3	105636601	105636900	0.749	1.821	0.682	0.516
Tan	3	105744601	105744900	0.237	0.727	0.272	0.279
Tan	3	105751501	105751800	1.334	2.628	1.402	1.455
Tan	3	105751801	105752100	1.862	3.278	2.086	2.399
Tan	3	105915301	105915600	0.211	0.539	0.218	0.274
Tan	3	105998101	105998400	0.290	0.725	0.350	0.324
Tan	3	106260901	106261200	0.373	0.815	0.194	0.441
Tan	3	106686301	106686600	1.328	1.684	0.938	1.181
Tan	3	106703401	106703700	0.639	1.397	0.827	0.867
Tan	3	107423701	107424000	1.087	0.401	1.398	1.334
Tan	3	107442901	107443200	2.018	2.482	1.966	1.714
Tan	3	107794201	107794500	0.239	0.541	0.136	0.243
Tan	3	107840101	107840400	0.582	1.214	0.390	0.405
Tan	3	107862901	107863200	0.745	1.382	0.727	0.826
Tan	3	108077401	108077700	1.515	2.622	1.629	1.820
Tan	3	108093301	108093600	0.160	0.491	0.185	0.147
Tan	3	108189901	108190200	0.720	0.281	0.531	0.608
Tan	3	108248401	108248700	0.240	0.513	0.239	0.162
Tan	3	108311401	108311700	0.290	1.101	0.716	0.583
Tan	3	108390001	108390300	0.161	0.564	0.292	0.274
Tan	3	108442501	108442800	0.105	0.631	0.156	0.254
Tan	3	108442801	108443100	0.237	0.729	0.127	0.137
Tan	3	108577201	108577500	0.292	0.607	0.074	0.365
Tan	3	108584101	108584400	0.136	0.721	0.136	0.259
Tan	3	108591001	108591300	0.079	0.586	0.107	0.106
Tan	3	108591301	108591600	0.053	0.556	0.156	0.112
Tan	3	108998401	108998700	0.184	0.541	0.054	0.081
Tan	3	109086301	109086600	0.345	0.538	0.210	0.218
Tan	3	109086601	109086900	0.507	0.701	0.127	0.193
Tan	3	109148101	109148400	0.319	0.444	0.082	0.218
Tan	3	109572901	109573200	0.531	0.330	0.642	0.913
Tan	3	109862401	109862700	1.599	1.918	1.275	2.292
Tan	3	109881901	109882200	0.718	0.678	0.506	0.304
Tan	3	110333101	110333400	0.079	1.199	0.417	0.132
Tan	3	110475001	110475300	0.558	0.839	0.481	0.441
Tan	3	110578201	110578500	0.902	0.350	0.711	0.715
Tan	3	110578501	110578800	0.797	0.538	0.802	0.963
Tan	3	110635801	110636100	0.134	0.468	0.194	0.112
Tan	3	110949001	110949300	0.237	0.466	0.132	0.142
Tan	3	111189601	111189900	0.268	0.560	0.218	0.279
Tan	3	111334201	111334500	0.457	1.053	0.502	0.279
Tan	3	111352801	111353100	0.426	0.515	0.267	0.360
Tan	3	111743101	111743400	0.000	1.174	0.185	0.117
Tan	3	111814801	111815100	0.586	1.105	0.370	0.486
Tan	3	111873601	111873900	0.292	0.725	0.419	0.396
Tan	3	112118701	112119000	0.347	0.845	0.210	0.213
Tan	3	112244401	112244700	0.373	0.609	0.267	0.304
Tan	3	112281601	112281900	0.477	0.629	0.321	0.279
Tan	3	112339501	112339800	0.160	0.588	0.132	0.162
Tan	3	112764301	112764600	0.268	0.964	0.399	0.345
Tan	3	113034301	113034600	1.663	0.024	1.204	2.560
Tan	3	114082201	114082500	0.210	0.721	0.185	0.284
Tan	3	114186601	114186900	0.265	1.223	0.448	0.284
Tan	3	114186901	114187200	0.769	1.146	0.580	0.654
Tan	3	114199801	114200100	0.373	0.912	0.493	0.299
Tan	3	114282901	114283200	0.239	0.611	0.296	0.268
Tan	3	114428401	114428700	1.594	1.403	1.197	0.994
Tan	3	114656101	114656400	0.424	1.317	0.279	0.401
Tan	3	114948901	114949200	0.579	1.459	0.568	0.583
Tan	3	115080601	115080900	0.131	0.470	0.107	0.056
Tan	3	115356301	115356600	1.115	1.495	0.909	1.166
Tan	3	115366801	115367100	0.611	0.234	0.551	0.502
Tan	3	115393801	115394100	0.957	1.748	0.807	0.877
Tan	3	115404601	115404900	1.491	2.130	1.736	1.577
Tan	3	115450501	115450800	0.428	0.978	0.370	0.401
Tan	3	115626301	115626600	0.447	1.103	0.160	0.486
Tan	3	115882501	115882800	0.266	0.564	0.156	0.218

Tan	3	116050801	116051100	0.690	1.317	0.794	0.715
Tan	3	116218501	116218800	0.665	1.150	0.700	0.472
Tan	3	116218801	116219100	0.931	1.628	0.814	0.614
Tan	3	116250901	116251200	0.505	0.914	0.584	0.472
Tan	3	116385601	116385900	1.307	1.869	0.160	0.259
Tan	3	116593801	116594100	0.347	0.631	0.448	0.391
Tan	3	116899801	116900100	0.639	0.307	0.107	0.031
Tan	3	117006301	117006600	0.265	0.491	0.292	0.279
Tan	3	117052501	117052800	0.186	0.423	0.136	0.025
Tan	3	117235201	117235500	1.065	0.701	0.160	0.198
Tan	3	117235501	117235800	1.061	0.508	0.029	0.112
Tan	3	117595801	117596100	1.303	1.804	1.061	1.399
Tan	3	118064701	118065000	0.186	0.420	0.029	0.198
Tan	3	118193101	118193400	1.460	0.612	1.139	1.410
Tan	3	118374301	118374600	0.347	0.802	0.243	0.304
Tan	3	118667401	118667700	0.268	0.656	0.140	0.279
Tan	3	118767001	118767300	0.428	1.285	0.843	0.842
Tan	3	119405701	119406000	0.479	0.257	0.638	0.329
Tan	3	119595901	119596200	0.213	0.706	0.132	0.051
Tan	3	121286701	121287000	0.666	0.159	0.613	0.796
Tan	3	121339801	121340100	0.186	0.517	0.156	0.223
Tan	3	121611901	121612200	0.321	0.680	0.152	0.299
Tan	3	121977301	121977600	0.077	0.420	0.103	0.162
Tan	3	122220601	122220900	1.886	1.054	1.930	1.714
Tan	3	122277901	122278200	0.240	1.028	0.337	0.376
Tan	3	122313301	122313600	0.505	1.127	0.341	0.477
Tan	3	122628901	122629200	0.210	1.049	0.205	0.106
Tan	3	122629201	122629500	0.265	1.148	0.152	0.248
Tan	3	122989501	122989800	0.266	0.800	0.194	0.081
Tan	3	122989801	122990100	0.000	0.493	0.078	0.025
Tan	3	123316501	123316800	0.000	0.446	0.049	0.112
Tan	3	123363901	123364200	0.932	0.541	1.057	1.694
Tan	3	123388801	123389100	0.292	0.837	0.279	0.076
Tan	3	124118101	124118400	1.487	0.676	1.456	1.136
Tan	3	124433101	124433400	0.215	0.611	0.156	0.218
Tan	3	124498201	124498500	0.611	0.891	0.395	0.299
Tan	3	124678201	124678500	0.213	0.536	0.210	0.243
Tan	3	125342401	125342700	0.184	0.584	0.243	0.193
Tan	3	125411701	125412000	0.266	0.704	0.160	0.329
Tan	3	125428501	125428800	0.479	0.140	0.584	0.507
Tan	3	125470801	125471100	0.685	2.161	1.008	0.710
Tan	3	125677201	125677500	0.106	0.395	0.160	0.142
Tan	3	125775901	125776200	0.137	0.607	0.078	0.112
Tan	3	125829001	125829300	0.452	0.701	0.453	0.583
Tan	3	125890501	125890800	0.132	0.564	0.103	0.106
Tan	3	125936401	125936700	0.928	1.852	0.807	1.151
Tan	3	126038101	126038400	0.803	2.111	0.695	1.491
Tan	3	126240901	126241200	0.184	0.867	0.210	0.203
Tan	3	126287701	126288000	0.371	1.429	0.296	0.425
Tan	3	126288001	126288300	0.398	1.195	0.317	0.263
Tan	3	126559201	126559500	0.289	0.772	0.243	0.167
Tan	3	126831601	126831900	0.213	0.837	0.103	0.086
Tan	3	126922201	126922500	0.687	0.161	1.016	1.100
Tan	3	127308301	127308600	0.081	0.395	0.000	0.061
Tan	3	127479001	127479300	0.027	0.446	0.136	0.061
Tan	3	127541401	127541700	0.261	1.391	0.350	0.350
Tan	3	127641601	127641900	0.318	0.985	0.617	0.553
Tan	3	127776301	127776600	0.240	0.656	0.366	0.385
Tan	3	127891501	127891800	0.481	1.337	0.597	0.497
Tan	3	128281501	128281800	0.186	0.699	0.218	0.137
Tan	3	128300401	128300700	0.557	0.328	0.506	0.081
Tan	3	128330701	128331000	0.962	1.760	0.687	0.704
Tan	3	128364301	128364600	1.194	2.410	1.463	1.172
Tan	3	128520301	128520600	2.500	1.028	2.423	0.462
Tan	3	128541001	128541300	0.373	0.560	0.317	0.355
Tan	3	128548501	128548800	0.110	0.650	0.025	0.061
Tan	3	128643601	128643900	0.213	0.442	0.292	0.193

Tan	3	128660401	128660700	0.953	2.528	1.251	1.140
Tan	3	128790001	128790300	1.205	2.412	1.021	1.277
Tan	3	128849701	128850000	0.163	0.676	0.288	0.274
Tan	3	128889301	128889600	0.479	0.772	0.374	0.533
Tan	3	129018601	129018900	1.410	2.326	1.094	1.338
Tan	3	129113101	129113400	0.053	0.470	0.082	0.304
Tan	3	129181801	129182100	1.889	2.849	1.665	1.729
Tan	3	129314101	129314400	0.266	0.024	0.292	0.492
Tan	3	129698701	129699000	0.266	0.584	0.317	0.248
Tan	3	129865801	129866100	0.268	0.466	0.025	0.279
Tan	3	130021801	130022100	0.932	1.906	0.909	0.968
Tan	3	130022401	130022700	0.800	1.446	0.885	0.963
Tan	3	130078801	130079100	1.382	1.143	0.774	0.416
Tan	3	130218301	130218600	0.352	1.126	0.296	0.482
Tan	3	130279801	130280100	0.400	0.751	0.337	0.335
Tan	3	130324801	130325100	0.374	0.562	0.185	0.193
Tan	3	130344301	130344600	2.851	4.957	3.188	2.966
Tan	3	130517401	130517700	1.802	0.980	2.858	1.622
Tan	3	130783201	130783500	1.862	2.615	2.189	1.764
Tan	3	130827601	130827900	1.094	1.805	1.115	0.852
Tan	3	130828201	130828500	0.398	0.916	0.560	0.533
Tan	3	131205301	131205600	0.213	0.446	0.189	0.137
Tan	3	131285101	131285400	0.613	0.165	0.580	0.695
Tan	3	131303101	131303400	0.879	1.382	0.584	0.137
Tan	3	131412001	131412300	0.187	0.423	0.103	0.167
Tan	3	131509801	131510100	0.319	1.002	0.457	0.669
Tan	3	131566201	131566500	0.957	1.616	1.066	0.760
Tan	3	131979601	131979900	1.755	2.716	1.801	1.719
Tan	3	132017401	132017700	3.321	1.560	2.521	2.499
Tan	3	132363301	132363600	0.000	0.234	0.000	0.497
Tan	3	132440701	132441000	0.132	0.423	0.025	0.086
Tan	3	132588001	132588300	0.344	0.845	0.354	0.228
Tan	3	132640501	132640800	1.011	1.311	0.959	0.968
Tan	3	132645601	132645900	0.266	0.560	0.267	0.248
Tan	3	132680101	132680400	0.690	1.195	0.856	0.781
Tan	3	132813901	132814200	0.184	0.936	0.156	0.538
Tan	3	133208101	133208400	0.716	0.912	0.720	0.441
Tan	3	133244101	133244400	0.847	1.620	0.954	0.720
Tan	3	133341301	133341600	0.481	0.772	0.399	0.558
Tan	3	133392601	133392900	0.531	0.884	0.531	0.365
Tan	3	133420501	133420800	4.144	1.839	3.425	4.507
Tan	3	133432501	133432800	0.481	1.009	0.181	0.218
Tan	3	133638001	133638300	1.647	1.077	1.592	1.780
Tan	3	133820401	133820700	0.689	1.326	0.671	0.765
Tan	3	133837201	133837500	0.957	0.371	0.638	0.664
Tan	3	133908601	133908900	0.239	0.539	0.263	0.254
Tan	3	133941001	133941300	1.140	0.556	1.235	1.298
Tan	3	134030401	134030700	0.240	0.959	0.165	0.187
Tan	3	134067001	134067300	0.724	2.249	0.814	0.801
Tan	3	134071801	134072100	0.161	0.635	0.103	0.157
Tan	3	134072101	134072400	0.268	1.543	0.390	0.502
Tan	3	134072401	134072700	0.481	1.360	0.399	0.675
Tan	3	134074801	134075100	0.797	0.631	0.716	0.497
Tan	3	134088001	134088300	0.186	0.395	0.189	0.081
Tan	3	134106601	134106900	1.279	0.487	1.074	1.060
Tan	3	134109301	134109600	0.665	0.446	0.210	0.254
Tan	3	134148601	134148900	1.142	0.272	1.090	1.552
Tan	3	134581801	134582100	0.294	0.444	0.156	0.137
Tan	3	134869201	134869500	0.745	0.444	0.749	0.695
Tan	3	135071701	135072000	0.426	0.562	0.214	0.223
Tan	3	135087601	135087900	0.158	0.702	0.395	0.193
Tan	3	135309301	135309600	0.292	0.586	0.374	0.355
Tan	3	135533701	135534000	0.158	0.442	0.189	0.142
Tan	3	136005001	136005300	0.211	0.466	0.267	0.248
Tan	3	136039501	136039800	0.636	0.961	0.399	0.335
Tan	3	136071001	136071300	0.426	0.704	0.341	0.441
Tan	3	136114801	136115100	0.105	0.513	0.107	0.167

Tan	3	136212901	136213200	0.531	0.114	0.374	0.416
Tan	3	136223701	136224000	1.281	0.701	1.251	1.232
Tan	3	136335301	136335600	0.318	0.517	0.267	0.112
Tan	3	136532701	136533000	8.053	5.926	8.095	7.610
Tan	3	137161201	137161500	1.278	1.635	0.613	0.963
Tan	3	137190301	137190600	0.240	0.515	0.194	0.218
Tan	3	137212801	137213100	2.021	1.498	1.971	2.018
Tan	3	137710801	137711100	0.132	0.444	0.288	0.167
Tan	3	137973601	137973900	1.647	2.802	1.654	1.480
Tan	3	137995801	137996100	0.452	0.964	0.321	0.355
Tan	3	138226501	138226800	0.266	0.676	0.107	0.289
Tan	3	138574501	138574800	0.108	0.465	0.074	0.117
Tan	3	138796501	138796800	1.115	0.796	1.226	1.100
Tan	3	138993301	138993600	0.134	0.395	0.189	0.193
Tan	3	139325101	139325400	2.020	1.684	1.329	1.389
Tan	3	139399501	139399800	0.344	0.096	0.477	0.360
Tan	3	140338801	140339100	0.160	0.609	0.078	0.279
Tan	3	140420401	140420700	1.065	1.523	0.843	0.846
Tan	3	140437801	140438100	0.265	0.729	0.263	0.284
Tan	3	141361501	141361800	0.371	0.562	0.288	0.142
Tan	3	141975001	141975300	2.278	1.026	2.040	3.119
Tan	3	141996901	141997200	0.106	0.468	0.210	0.254
Tan	3	142080301	142080600	0.374	0.676	0.366	0.355
Tan	3	142080601	142080900	0.160	0.466	0.346	0.056
Tan	3	142688701	142689000	0.294	0.657	0.214	0.137
Tan	3	142749601	142749900	2.257	2.944	1.765	2.327
Tan	3	143745301	143745600	0.265	0.538	0.292	0.360
Tan	3	144001501	144001800	0.055	0.350	0.531	0.193
Tan	3	144034501	144034800	0.594	2.248	1.168	0.902
Tan	3	144144601	144144900	0.347	0.609	0.374	0.274
Tan	3	144689401	144689700	0.079	0.493	0.107	0.106
Tan	3	144844801	144845100	0.213	0.513	0.136	0.157
Tan	3	144845101	144845400	2.136	3.403	2.098	1.333
Tan	3	144861601	144861900	0.134	0.423	0.107	0.142
Tan	3	145577401	145577700	0.134	0.395	0.132	0.162
Tan	3	146084101	146084400	0.265	1.073	0.123	0.517
Tan	3	146439301	146439600	0.161	0.612	0.103	0.137
Tan	3	147393301	147393600	0.398	0.303	0.477	0.248
Tan	3	148161601	148161900	0.452	0.187	0.526	0.477
Tan	3	148362601	148362900	0.395	1.259	0.544	0.441
Tan	3	148605901	148606200	0.237	1.545	0.078	0.923
Tan	3	148606201	148606500	0.344	1.680	0.176	1.141
Tan	3	148611301	148611600	1.654	1.099	1.752	1.703
Tan	3	148717501	148717800	0.160	0.401	0.107	0.000
Tan	3	149055901	149056200	0.718	1.290	0.575	0.826
Tan	3	149240401	149240700	0.584	0.961	0.395	0.299
Tan	3	149254801	149255100	0.239	0.564	0.156	0.329
Tan	3	149265901	149266200	0.106	0.466	0.399	0.360
Tan	3	149381401	149381700	0.718	0.328	0.526	0.608
Tan	3	149567701	149568000	0.240	0.631	0.239	0.142
Tan	3	149682001	149682300	0.344	0.609	0.292	0.173
Tan	3	149744401	149744700	0.479	0.654	0.263	0.477
Tan	3	150317101	150317400	0.374	0.656	0.239	0.218
Tan	3	150659401	150659700	0.823	0.678	0.239	0.391
Tan	3	151085401	151085700	0.452	0.772	0.321	0.309
Tan	3	151441501	151441800	0.240	0.465	0.214	0.223
Tan	3	151713601	151713900	0.558	0.916	0.526	0.553
Tan	3	151809901	151810200	0.584	0.936	0.111	0.380
Tan	3	152049901	152050200	0.479	0.232	0.535	0.466
Tan	3	152092501	152092800	0.428	0.773	0.317	0.309
Tan	3	152308801	152309100	0.878	1.051	0.798	1.404
Tan	3	152309101	152309400	1.274	1.590	1.280	1.932
Tan	3	152789701	152790000	0.584	0.185	0.419	0.527
Tan	3	152913301	152913600	0.213	0.607	0.160	0.228
Tan	3	153124201	153124500	0.105	0.375	0.185	0.608
Tan	3	153268501	153268800	0.347	0.747	0.330	0.304
Tan	3	153350701	153351000	0.187	0.704	0.152	0.299

Tan	3	153439501	153439800	0.398	0.165	0.346	0.527
Tan	3	153668401	153668700	0.371	0.515	0.317	0.223
Tan	3	153704701	153705000	0.108	0.659	0.210	0.137
Tan	3	154138801	154139100	0.423	0.770	0.341	0.335
Tan	3	154438201	154438500	0.321	0.845	0.210	0.350
Tan	3	154574401	154574700	0.295	0.940	0.312	0.416
Tan	3	154599001	154599300	0.081	0.401	0.054	0.142
Tan	3	154845601	154845900	0.611	0.330	0.642	0.720
Tan	3	154890601	154890900	0.186	0.682	0.288	0.259
Tan	3	155061601	155061900	0.189	0.729	0.214	0.182
Tan	3	155414101	155414400	0.266	0.676	0.243	0.147
Tan	3	155421601	155421900	0.532	0.773	0.560	0.385
Tan	3	155735401	155735700	0.186	0.631	0.267	0.167
Tan	3	156399901	156400200	0.215	0.798	0.189	0.193
Tan	3	157041001	157041300	0.879	1.873	0.629	0.643
Tan	3	158732401	158732700	1.089	0.538	0.856	0.274
Tan	3	160197001	160197300	0.555	0.212	0.720	0.695
Tan	3	160593001	160593300	0.156	0.491	0.160	0.142
Tan	3	161589301	161589600	0.132	0.468	0.292	0.304
Tan	3	161653801	161654100	0.795	1.596	0.753	0.933
Tan	3	161672101	161672400	0.398	1.096	0.671	0.608
Tan	3	161867401	161867700	0.958	0.629	0.374	0.396
Tan	3	162065701	162066000	0.186	0.468	0.194	0.162
Tan	3	162179701	162180000	1.862	3.158	1.618	1.593
Tan	3	162199801	162200100	0.319	0.071	0.560	0.385
Tan	3	162343801	162344100	1.092	1.877	1.115	1.374
Tan	3	162516601	162516900	0.053	0.371	0.082	0.000
Tan	3	162684001	162684300	0.373	0.212	0.609	0.112
Tan	3	162759301	162759600	0.608	1.002	0.526	0.502
Tan	3	162857401	162857700	0.324	1.105	0.399	0.263
Tan	3	163003801	163004100	0.347	0.515	0.078	0.254
Tan	3	163161001	163161300	0.318	0.682	0.296	0.360
Tan	3	163286101	163286400	0.213	0.423	0.239	0.218
Tan	3	163888501	163888800	0.268	0.609	0.156	0.056
Tan	3	165405901	165406200	4.068	3.551	3.241	4.209
Tan	3	165541801	165542100	0.639	1.238	0.588	0.826
Tan	3	165750601	165750900	0.160	0.468	0.370	0.304
Tan	3	165863701	165864000	0.349	0.871	0.239	0.243
Tan	3	165970501	165970800	0.215	0.421	0.691	0.106
Tan	3	166127401	166127700	0.426	0.539	0.267	0.248
Tan	3	166127701	166128000	0.294	0.609	0.263	0.279
Tan	3	166173601	166173900	0.426	0.908	0.325	0.553
Tan	3	166489801	166490100	0.450	0.843	0.267	0.309
Tan	3	166873201	166873500	0.479	0.869	0.506	0.553
Tan	3	166909501	166909800	0.292	0.890	0.160	0.497
Tan	3	167043001	167043300	0.000	0.210	0.000	0.497
Tan	3	167048701	167049000	0.266	0.586	0.107	0.335
Tan	3	167283601	167283900	0.184	0.466	0.107	0.173
Tan	3	167788501	167788800	1.252	1.686	0.852	1.004
Tan	3	167961301	167961600	0.477	0.071	0.424	0.193
Tan	3	168120001	168120300	0.797	0.961	0.662	0.304
Tan	3	168280201	168280500	2.155	1.307	1.786	2.251
Tan	3	169077001	169077300	0.292	0.491	0.296	0.112
Tan	3	169832101	169832400	0.237	0.607	0.103	0.203
Tan	3	170222701	170223000	0.902	0.208	0.638	0.573
Tan	3	170708701	170709000	0.692	1.266	0.925	0.558
Tan	3	170780101	170780400	0.776	1.560	0.354	0.547
Tan	3	170792401	170792700	0.158	0.661	0.160	0.112
Tan	3	171057001	171057300	0.266	0.513	0.156	0.268
Tan	3	171711001	171711300	0.373	0.862	0.223	0.147
Tan	3	171711301	171711600	0.294	0.863	0.243	0.193
Tan	3	172118101	172118400	2.284	1.011	2.111	2.419
Tan	3	172118401	172118700	4.513	3.027	4.401	4.944
Tan	3	172187401	172187700	0.268	0.629	0.247	0.198
Tan	3	172272001	172272300	0.397	0.891	0.403	0.405
Tan	3	172527301	172527600	1.063	1.448	0.827	0.979
Tan	3	172652701	172653000	0.106	0.588	0.234	0.173

Tan	3	172682101	172682400	0.213	0.517	0.210	0.198
Tan	3	172720501	172720800	0.215	0.628	0.259	0.198
Tan	3	172747501	172747800	0.161	0.444	0.239	0.167
Tan	3	172851601	172851900	0.055	0.395	0.054	0.167
Tan	3	173197801	173198100	0.160	0.448	0.156	0.162
Tan	3	173207401	173207700	1.223	1.970	0.901	1.476
Tan	3	173269501	173269800	1.812	2.972	1.565	1.302
Tan	3	173325001	173325300	0.160	0.539	0.136	0.193
Tan	3	173335501	173335800	1.968	0.302	1.117	1.100
Tan	3	173572201	173572500	0.213	0.682	0.210	0.309
Tan	3	173589901	173590200	0.211	0.631	0.259	0.218
Tan	3	173693701	173694000	0.105	0.470	0.082	0.142
Tan	3	173766301	173766600	0.213	0.721	0.252	0.198
Tan	3	173766601	173766900	0.581	1.408	0.798	0.740
Tan	3	174072601	174072900	0.534	0.255	0.740	0.720
Tan	3	174862201	174862500	0.481	0.772	0.457	0.421
Tan	3	175125601	175125900	0.158	0.702	0.234	0.147
Tan	3	175125901	175126200	0.027	0.420	0.210	0.112
Tan	3	175976701	175977000	0.134	0.629	0.317	0.294
Tan	3	175989301	175989600	1.405	2.377	1.378	0.867
Tan	3	175996501	175996800	0.395	0.891	0.477	0.329
Tan	3	176521201	176521500	0.106	0.491	0.082	0.193
Tan	3	176676901	176677200	0.373	0.584	0.531	0.304
Tan	3	176889301	176889600	0.799	0.187	0.526	0.614
Tan	3	176993101	176993400	0.266	0.092	0.448	0.411
Tan	3	177023101	177023400	0.265	0.802	0.074	0.304
Tan	3	177106201	177106500	0.398	0.845	0.419	0.472
Tan	3	177194701	177195000	0.163	0.991	0.132	0.324
Tan	3	177335101	177335400	0.481	0.818	0.267	0.527
Tan	3	177336601	177336900	0.826	1.197	0.502	0.659
Tan	3	177338101	177338400	0.418	1.356	0.210	0.324
Tan	3	177363601	177363900	0.108	0.442	0.132	0.223
Tan	3	177396601	177396900	0.560	0.839	0.481	0.558
Tan	3	177486001	177486300	0.824	1.779	0.729	0.629
Tan	3	177816601	177816900	0.211	0.468	0.214	0.304
Tan	3	177925201	177925500	0.052	0.510	0.107	0.142
Tan	3	178105801	178106100	0.240	0.560	0.194	0.112
Tan	3	178235701	178236000	0.161	1.043	0.054	0.086
Tan	3	178327201	178327500	0.531	0.865	0.136	0.259
Tan	3	178327501	178327800	0.347	0.493	0.054	0.086
Tan	3	178348501	178348800	0.266	0.466	0.321	0.279
Tan	3	178599901	178600200	0.186	0.493	0.127	0.132
Tan	3	178702801	178703100	0.694	1.148	0.444	0.573
Tan	3	178773301	178773600	0.345	1.525	0.876	0.644
Tan	3	179064301	179064600	3.615	4.300	3.335	3.787
Tan	3	179290501	179290800	0.292	0.584	0.152	0.248
Tan	3	179544901	179545200	0.878	1.120	0.419	0.826
Tan	3	179739901	179740200	1.252	3.201	1.264	1.369
Tan	3	179861401	179861700	2.452	0.747	1.531	2.333
Tan	3	180035101	180035400	0.265	0.631	0.165	0.112
Tan	3	180036301	180036600	0.187	0.558	0.210	0.243
Tan	3	180064501	180064800	0.481	0.727	0.979	0.999
Tan	3	180170101	180170400	0.611	0.841	0.185	0.669
Tan	3	180211801	180212100	0.266	0.800	0.049	0.213
Tan	3	180301201	180301500	2.391	1.285	2.776	2.181
Tan	3	180322201	180322500	0.474	1.026	0.428	0.533
Tan	3	180572401	180572700	1.118	0.605	1.201	0.948
Tan	3	180629101	180629400	1.568	2.171	1.201	1.222
Tan	3	180900601	180900900	0.161	0.446	0.103	0.132
Tan	3	181017001	181017300	0.265	0.444	0.210	0.051
Tan	3	181912501	181912800	0.158	0.730	0.107	0.187
Tan	3	182026501	182026800	0.452	0.843	0.560	0.695
Tan	3	182304001	182304300	0.265	0.633	0.263	0.294
Tan	3	182585701	182586000	0.557	0.187	0.531	0.472
Tan	3	183077401	183077700	0.902	1.354	0.667	0.867
Tan	3	183201901	183202200	0.639	0.916	0.374	0.639
Tan	3	183222901	183223200	0.905	1.663	0.769	1.019

Tan	3	183469501	183469800	0.103	0.938	1.304	0.223
Tan	3	183866401	183866700	0.265	0.491	0.317	0.335
Tan	3	184042201	184042500	0.292	0.659	0.263	0.162
Tan	3	185789101	185789400	0.507	0.891	0.366	0.268
Tan	3	185793901	185794200	0.079	0.446	0.189	0.112
Tan	3	186150301	186150600	0.374	0.914	0.399	0.436
Tan	3	186453901	186454200	0.350	0.770	0.267	0.355
Tan	3	186540301	186540600	0.508	1.053	0.205	0.517
Tan	3	186877201	186877500	1.195	1.944	0.838	1.096
Tan	3	187042201	187042500	0.081	0.371	0.107	0.106
Tan	3	187087501	187087800	0.027	0.371	0.107	0.137
Tan	3	187094401	187094700	1.120	1.755	1.255	1.237
Tan	3	187221001	187221300	0.160	0.541	0.049	0.193
Tan	3	187387501	187387800	0.161	0.487	0.160	0.193
Tan	3	187575301	187575600	0.265	0.446	0.210	0.193
Tan	3	187610701	187611000	1.276	1.770	0.568	1.024
Tan	3	188041201	188041500	0.210	1.212	0.691	0.416
Tan	3	188084701	188085000	0.452	0.860	0.374	0.466
Tan	3	188662201	188662500	0.263	0.680	0.234	0.218
Tan	3	188690101	188690400	0.240	0.581	0.160	0.142
Tan	3	189098401	189098700	0.186	0.489	0.156	0.254
Tan	3	189222001	189222300	0.373	0.560	0.350	0.274
Tan	3	189938701	189939000	0.137	0.751	0.189	0.254
Tan	3	189948001	189948300	0.532	0.234	0.346	0.142
Tan	3	190039201	190039500	0.131	1.231	0.156	0.198
Tan	3	190197601	190197900	0.161	0.538	0.078	0.193
Tan	3	190345201	190345500	0.557	1.405	0.370	0.527
Tan	3	190556701	190557000	0.184	0.468	0.189	0.056
Tan	3	190571101	190571400	0.158	0.395	0.160	0.056
Tan	3	191095501	191095800	0.106	0.511	0.082	0.051
Tan	3	191808001	191808300	0.345	0.609	0.321	0.223
Tan	3	191888701	191889000	0.692	0.189	0.453	0.385
Tan	3	192288001	192288300	0.108	0.371	0.107	0.000
Tan	3	192301501	192301800	1.300	2.315	1.572	1.206
Tan	3	192432601	192432900	0.213	0.442	0.107	0.167
Tan	3	192731701	192732000	0.052	0.395	0.082	0.162
Tan	3	193425001	193425300	0.532	1.287	1.016	0.826
Tan	3	193451101	193451400	0.265	0.444	0.156	0.248
Tan	3	193763101	193763400	0.239	0.729	0.296	0.309
Tan	3	193871401	193871700	0.106	0.444	0.263	0.329
Tan	3	194928601	194928900	0.103	0.562	0.127	0.132
Tan	3	195300901	195301200	0.210	0.659	0.136	0.218
Tan	3	195361201	195361500	0.077	0.515	0.049	0.162
Tan	3	195708301	195708600	0.265	0.515	0.263	0.228
Tan	3	195841201	195841500	0.652	2.699	0.386	0.726
Tan	3	195841501	195841800	0.187	0.702	0.103	0.193
Tan	3	196140901	196141200	0.240	0.069	0.535	0.299
Tan	3	197095201	197095500	0.161	0.513	0.267	0.081
Tan	3	197676301	197676600	0.292	0.539	0.346	0.274
Tan	3	198509101	198509400	0.239	0.491	0.136	0.309
Tan	3	198809401	198809700	0.373	0.704	0.296	0.416
Tan	3	198809701	198810000	0.215	0.446	0.185	0.162
Tan	3	198858901	198859200	0.800	0.515	0.267	0.304
Tan	3	198872101	198872400	0.026	0.281	0.502	0.142
Tan	3	199134301	199134600	0.318	0.023	0.560	0.396
Tan	3	199307101	199307400	0.215	0.564	0.234	0.132
Tan	3	199448101	199448400	0.344	0.584	0.189	0.051
Tan	3	199744201	199744500	0.745	1.120	0.794	0.669
Tan	3	200133301	200133600	0.477	0.794	0.477	0.405
Tan	3	200477101	200477400	0.294	0.444	0.189	0.776
Tan	3	200710201	200710500	0.402	1.101	0.390	0.588
Tan	3	200889901	200890200	0.290	0.800	0.341	0.162
Tan	3	200915701	200916000	0.637	0.420	0.667	0.634
Tan	3	200935201	200935500	0.213	0.612	0.165	0.137
Tan	3	201190201	201190500	0.242	0.635	0.243	0.274
Tan	3	201338701	201339000	0.242	0.564	0.267	0.218
Tan	3	201560101	201560400	2.846	3.914	3.188	3.062

Tan	3	201717001	201717300	0.452	0.751	0.263	0.391
Tan	3	202105501	202105800	0.215	0.775	0.132	0.268
Tan	3	202230601	202230900	0.261	1.152	0.205	0.466
Tan	3	202242901	202243200	0.905	1.663	0.602	0.994
Tan	3	202259101	202259400	0.450	1.328	0.189	0.557
Tan	3	202265401	202265700	0.321	1.126	0.555	0.507
Tan	3	202283701	202284000	1.492	3.898	0.802	2.962
Tan	3	202284001	202284300	0.474	1.906	0.519	0.579
Tan	3	202284301	202284600	0.371	0.657	0.292	0.228
Tan	3	202437901	202438200	0.716	1.373	0.415	0.664
Tan	3	202818001	202818300	0.721	1.075	0.366	0.608
Tan	3	202823101	202823400	0.345	0.768	0.214	0.234
Tan	3	202921801	202922100	0.453	0.936	0.395	0.690
Tan	3	203009701	203010000	0.265	0.747	0.243	0.441
Tan	3	203691901	203692200	0.584	0.747	0.428	0.497
Tan	3	203927701	203928000	0.053	0.493	0.082	0.162
Tan	3	204382801	204383100	0.160	0.586	0.156	0.416
Tan	3	204888901	204889200	0.692	1.126	0.588	0.690
Tan	3	205247701	205248000	1.410	2.032	1.757	1.085
Tan	3	205455901	205456200	0.426	0.609	0.054	0.167
Tan	3	205512901	205513200	0.186	0.489	0.243	0.243
Tan	3	205912201	205912500	0.106	0.466	0.136	0.187
Tan	3	206109901	206110200	0.215	0.470	0.160	0.000
Tan	3	206663101	206663400	0.903	0.465	1.061	0.751
Tan	3	207460201	207460500	2.076	1.026	2.004	1.932
Tan	3	207661801	207662100	0.239	0.583	0.239	0.309
Tan	3	207825301	207825600	0.240	0.657	0.267	0.081
Tan	3	208207801	208208100	1.011	0.468	0.633	0.720
Tan	3	208353901	208354200	0.371	0.491	0.613	0.279
Tan	3	208609201	208609500	2.287	2.570	1.670	1.795
Tan	3	208635301	208635600	0.557	0.234	0.424	0.441
Tan	3	208722601	208722900	0.769	0.515	0.263	0.213
Tan	3	208791301	208791600	0.213	0.562	0.181	0.086
Tan	3	208988101	208988400	0.319	0.558	0.317	0.304
Tan	3	209457301	209457600	0.400	0.682	0.399	0.193
Tan	3	209548501	209548800	1.733	3.135	1.891	2.261
Tan	3	209679601	209679900	0.534	0.112	0.535	0.547
Tan	3	209731501	209731800	0.398	0.775	0.448	0.355
Tan	3	209744701	209745000	4.518	3.673	2.542	3.052
Tan	3	209870701	209871000	0.134	0.395	0.185	0.223
Tan	3	210028501	210028800	0.316	0.701	0.165	0.304
Tan	3	210279901	210280200	6.493	1.040	7.778	6.865
Tan	3	210280201	210280500	3.830	0.687	4.424	3.588
Tan	3	210281101	210281400	0.929	1.424	0.481	0.873
Tan	3	210355501	210355800	2.554	1.923	2.740	2.520
Tan	3	210364201	210364500	0.053	0.418	0.054	0.137
Tan	3	210389701	210390000	2.461	3.957	2.722	2.585
Tan	3	210457801	210458100	0.266	1.129	0.210	0.426
Tan	3	210471601	210471900	1.531	0.472	1.946	1.501
Tan	3	210517501	210517800	1.111	1.828	1.032	1.035
Tan	3	210521401	210521700	0.754	2.598	0.779	0.877
Tan	3	210525301	210525600	0.318	0.656	0.350	0.299
Tan	3	210578401	210578700	1.228	2.219	1.708	1.602
Tan	3	210635101	210635400	0.531	1.002	0.374	0.619
Tan	3	210681601	210681900	3.804	3.267	2.818	2.459
Tan	3	210807301	210807600	0.929	1.264	0.852	0.639
Tan	3	210848701	210849000	0.345	0.491	0.317	0.634
Tan	3	210952501	210952800	5.023	3.791	5.158	3.200
Tan	3	210990901	210991200	0.027	0.586	0.116	0.000
Tan	3	211051801	211052100	0.292	0.468	0.243	0.248
Tan	3	211075501	211075800	1.386	1.751	0.814	1.186
Tan	3	211128001	211128300	4.438	3.066	3.756	4.011
Tan	3	211213801	211214100	0.186	0.281	0.477	0.137
Tan	3	211262101	211262400	0.053	0.444	0.189	0.137
Tan	3	211307701	211308000	2.262	3.899	2.222	1.846
Tan	3	211453501	211453800	0.161	0.560	0.230	0.223
Tan	3	211697401	211697700	3.062	2.598	2.078	3.224

Tan	3	211784101	211784400	0.211	0.491	0.078	0.187
Tan	3	212076301	212076600	0.823	1.401	0.860	0.776
Tan	3	212107801	212108100	0.398	0.749	0.379	0.385
Tan	3	212242201	212242500	1.704	2.103	1.485	1.613
Tan	3	212297701	212298000	1.570	0.834	1.625	1.541
Tan	3	212691001	212691300	1.755	1.242	1.621	1.714
Tan	3	212853601	212853900	0.611	1.004	0.613	0.751
Tan	3	213007501	213007800	0.692	1.292	0.642	0.690
Tan	3	213106801	213107100	0.426	0.798	0.374	0.355
Tan	3	213185401	213185700	0.745	1.480	0.680	0.796
Tan	3	213197401	213197700	6.071	4.864	3.804	6.521
Tan	3	213201901	213202200	0.639	1.468	0.896	0.679
Tan	3	213246901	213247200	1.910	3.028	1.641	1.201
Tan	3	213331801	213332100	0.319	0.468	0.399	0.385
Tan	3	213344701	213345000	0.374	0.656	0.156	0.142
Tan	3	213630301	213630600	0.797	1.006	0.424	0.751
Tan	3	213734701	213735000	2.842	4.042	3.208	2.321
Tan	3	213795901	213796200	3.139	2.920	2.682	3.671
Tan	3	213827701	213828000	0.586	1.099	0.432	0.456
Tan	3	214023001	214023300	0.186	0.444	0.136	0.173
Tan	3	214122901	214123200	1.116	0.654	0.881	0.832
Tan	3	214319401	214319700	0.268	0.491	0.189	0.304
Tan	3	214440901	214441200	0.453	0.794	0.457	0.466
Tan	3	214447801	214448100	0.905	1.386	0.720	0.857
Tan	3	214601101	214601400	0.610	1.545	0.374	0.812
Tan	3	214676401	214676700	0.481	0.161	0.453	0.492
Tan	3	214695301	214695600	0.503	0.279	0.638	0.608
Tan	3	214707301	214707600	4.389	2.162	4.385	3.610
Tan	3	214740301	214740600	0.749	1.453	0.959	0.684
Tan	3	214749301	214749600	0.292	0.515	0.082	0.187
Tan	3	214864201	214864500	1.757	1.191	1.411	1.019
Tan	3	215020801	215021100	1.678	0.929	1.558	1.516
Tan	3	215095201	215095500	0.534	0.069	0.502	0.740
Tan	3	215127901	215128200	0.694	1.051	0.769	0.725
Tan	3	215151001	215151300	1.676	3.383	1.480	2.409
Tan	3	215344201	215344500	0.531	1.096	0.573	0.547
Tan	3	215383201	215383500	0.134	0.395	0.132	0.137
Tan	3	215411701	215412000	0.266	0.607	0.370	0.324
Tan	3	215558401	215558700	0.213	0.796	0.160	0.365
Tan	3	215570101	215570400	0.239	0.444	0.185	0.081
Tan	3	215570401	215570700	0.213	0.558	0.103	0.112
Tan	3	215635501	215635800	0.163	0.680	0.346	0.329
Tan	3	215643001	215643300	0.400	0.633	0.346	0.223
Tan	3	215677201	215677500	0.818	1.906	0.860	0.695
Tan	3	215810101	215810400	0.240	0.843	0.272	0.350
Tan	3	215898301	215898600	1.144	2.012	1.110	1.399
Tan	3	215939401	215939700	1.094	2.437	1.616	1.881
Tan	3	216049201	216049500	0.292	0.584	0.218	0.193
Tan	3	216172201	216172500	6.167	6.657	5.582	5.228
Tan	3	216183001	216183300	0.586	0.916	0.613	0.578
Tan	3	216271501	216271800	0.610	1.152	0.506	0.431
Tan	3	216503701	216504000	0.479	0.938	0.424	0.533
Tan	3	216554101	216554400	0.482	1.026	0.510	0.588
Tan	3	216637801	216638100	0.215	0.491	0.263	0.274
Tan	3	216835501	216835800	0.374	0.800	0.379	0.279
Tan	3	217173001	217173300	3.029	2.362	2.784	3.331
Tan	3	217181101	217181400	0.613	1.478	0.542	0.456
Tan	3	217389001	217389300	5.234	2.390	4.757	4.396
Tan	3	217390501	217390800	0.132	0.564	0.103	0.243
Tan	3	217641001	217641300	3.165	3.572	2.871	2.439
Tan	3	217812001	217812300	0.371	0.674	0.243	0.254
Tan	3	217849801	217850100	0.479	0.654	0.428	0.223
Tan	3	217888201	217888500	0.452	0.631	0.263	0.502
Tan	3	217905601	217905900	0.132	0.517	0.218	0.137
Tan	3	217906501	217906800	0.455	0.794	0.321	0.365
Tan	3	218066101	218066400	1.091	0.472	0.745	0.720
Tan	3	218096401	218096700	3.505	1.905	3.640	3.108

Tan	3	218167801	218168100	0.187	0.584	0.424	0.187
Tan	3	218184301	218184600	2.499	2.963	1.921	2.110
Tan	3	218413801	218414100	0.268	0.678	0.263	0.304
Tan	3	218457001	218457300	0.587	0.163	0.370	0.614
Tan	3	218486101	218486400	2.470	4.092	2.260	2.743
Tan	3	218526601	218526900	3.836	2.677	1.371	1.647
Tan	3	218541601	218541900	0.318	1.126	0.622	0.279
Tan	3	218642101	218642400	0.132	0.489	0.239	0.248
Tan	3	218658001	218658300	2.444	4.482	2.967	2.470
Tan	3	218721601	218721900	0.294	0.657	0.107	0.335
Tan	3	218777101	218777400	0.215	0.562	0.243	0.061
Tan	3	218824801	218825100	0.184	0.560	0.078	0.182
Tan	3	218835601	218835900	0.347	0.538	0.214	0.304
Tan	3	218956201	218956500	3.244	2.034	3.698	3.509
Tan	3	218957401	218957700	2.154	1.916	1.596	2.565
Tan	3	218989501	218989800	2.050	1.160	1.942	2.115
Tan	3	219039901	219040200	0.692	1.146	0.760	0.745
Tan	3	219144001	219144300	0.955	1.455	0.691	0.740
Tan	3	219271801	219272100	1.752	0.693	1.601	1.735
Tan	3	219327601	219327900	0.453	1.025	0.239	0.563
Tan	3	219340201	219340500	0.532	0.817	1.037	0.502
Tan	3	219536401	219536700	0.186	0.418	0.774	0.664
Tan	3	219684601	219684900	0.874	1.446	0.983	0.801
Tan	3	219701701	219702000	1.379	2.362	1.280	1.151
Tan	3	219702601	219702900	0.586	1.146	0.560	0.432
Tan	3	219960301	219960600	0.955	0.725	0.424	0.411
Tan	3	220221301	220221600	1.224	2.255	1.226	1.471
Tan	3	220567801	220568100	1.544	2.012	1.395	0.826
Tan	3	220660801	220661100	3.799	3.126	2.530	3.944
Tan	3	221032201	221032500	0.027	0.513	0.111	0.218
Tan	3	221139601	221139900	1.140	2.379	1.313	1.643
Tan	3	221175901	221176200	6.756	4.029	6.059	5.441
Tan	3	221226301	221226600	7.025	3.920	5.867	7.190
Tan	3	221360701	221361000	1.542	2.624	3.027	1.404
Tan	3	221387101	221387400	1.542	1.802	1.139	1.435
Tan	3	221494201	221494500	1.336	2.701	1.217	1.495
Tan	3	221569201	221569500	2.289	0.886	1.518	1.815
Tan	3	221705401	221705700	0.347	0.584	0.374	0.745
Tan	3	222002401	222002700	0.878	1.309	0.983	0.786
Tan	3	222169501	222169800	0.929	1.360	0.662	0.943
Tan	3	222178201	222178500	1.011	0.865	0.609	1.166
Tan	3	222313501	222313800	0.431	1.053	0.325	0.533
Tan	3	222362701	222363000	0.105	0.446	0.214	0.274
Tan	3	222384901	222385200	1.752	3.409	2.180	1.921
Tan	3	222411001	222411300	0.397	0.772	0.210	0.360
Tan	3	222486901	222487200	4.880	3.121	5.386	5.343
Tan	3	222514801	222515100	1.513	0.555	1.514	1.552
Tan	3	223287001	223287300	3.272	1.605	3.127	2.677
Tan	3	223330501	223330800	3.836	2.426	4.213	3.331
Tan	3	223349701	223350000	4.983	2.945	5.294	5.729
Tan	3	223601401	223601700	2.233	1.682	1.538	1.962
Tan	3	223668901	223669200	0.932	0.206	0.506	0.690
Tan	3	223734601	223734900	0.134	0.513	0.292	0.223
Tan	3	223816801	223817100	1.513	2.819	1.420	1.562
Tan	3	223824901	223825200	2.317	1.184	2.122	2.083
Tan	3	224055901	224056200	1.863	0.755	1.855	2.307
Tan	3	224080801	224081100	0.187	0.517	0.292	0.279
Tan	3	224103301	224103600	1.357	1.588	1.329	2.069
Tan	3	224219701	224220000	1.812	0.749	1.777	1.724
Tan	4	445801	446100	0.131	0.515	0.132	0.051
Tan	4	963001	963300	0.237	0.560	0.210	0.137
Tan	4	2197201	2197500	1.110	1.798	1.139	1.024
Tan	4	2739301	2739600	0.211	0.493	0.111	0.187
Tan	4	3941701	3942000	0.299	1.748	0.802	0.796
Tan	4	4375201	4375500	0.160	0.656	0.239	0.304
Tan	4	4481101	4481400	0.907	1.399	0.896	0.943
Tan	4	4524301	4524600	0.347	1.051	0.214	0.173

Tan	4	4740901	4741200	0.239	0.586	0.292	0.441
Tan	4	5071501	5071800	0.082	0.442	0.181	0.745
Tan	4	5080201	5080500	3.162	1.738	3.422	2.734
Tan	4	5122501	5122800	0.263	1.622	1.066	0.527
Tan	4	5124601	5124900	0.424	1.075	1.246	0.695
Tan	4	5242801	5243100	1.135	2.272	1.130	0.655
Tan	4	5253601	5253900	0.429	2.206	0.976	0.883
Tan	4	5325601	5325900	0.319	0.584	0.074	0.385
Tan	4	5461201	5461500	0.694	1.244	0.695	0.776
Tan	4	5463301	5463600	0.505	0.187	0.292	0.248
Tan	4	5650501	5650800	0.237	0.560	0.263	0.187
Tan	4	6013801	6014100	0.586	0.959	0.691	0.405
Tan	4	6050101	6050400	0.265	0.586	0.267	0.187
Tan	4	6528301	6528600	0.265	0.935	0.341	0.578
Tan	4	6595501	6595800	0.589	1.098	0.600	0.279
Tan	4	6735301	6735600	0.371	0.609	0.341	0.218
Tan	4	6982201	6982500	0.898	2.541	0.746	0.923
Tan	4	7038001	7038300	0.477	0.772	0.263	0.187
Tan	4	7113301	7113600	0.345	0.652	0.350	0.329
Tan	4	7272301	7272600	0.589	1.334	0.876	0.867
Tan	4	7342201	7342500	0.931	1.453	0.691	0.664
Tan	4	7399201	7399500	0.215	0.539	0.243	0.173
Tan	4	7473001	7473300	0.689	1.099	0.395	0.644
Tan	4	7499701	7500000	0.266	0.845	0.259	0.183
Tan	4	7539901	7540200	2.799	2.064	1.313	3.651
Tan	4	7611901	7612200	0.371	0.513	0.263	0.137
Tan	4	7774501	7774800	0.000	0.371	0.054	0.061
Tan	4	7780501	7780800	2.552	4.042	2.225	2.803
Tan	4	7782601	7782900	0.344	0.654	0.194	0.112
Tan	4	7818601	7818900	0.290	0.914	0.486	0.385
Tan	4	8120101	8120400	0.505	0.654	0.239	0.416
Tan	4	8202001	8202300	0.319	0.841	0.486	0.446
Tan	4	8359501	8359800	0.531	0.794	0.448	0.533
Tan	4	8400301	8400600	0.690	0.515	0.740	0.690
Tan	4	8695501	8695800	0.187	0.515	0.082	0.274
Tan	4	8783101	8783400	3.272	4.070	2.621	3.362
Tan	4	8814901	8815200	0.986	0.541	1.037	0.893
Tan	4	9399001	9399300	0.160	0.258	0.506	0.137
Tan	4	9581101	9581400	0.453	0.985	0.156	0.740
Tan	4	9679801	9680100	0.774	1.124	0.312	0.558
Tan	4	9694501	9694800	0.611	0.935	0.642	0.634
Tan	4	9707401	9707700	1.142	1.950	1.164	1.125
Tan	4	9828301	9828600	0.721	1.195	0.642	0.826
Tan	4	9838201	9838500	1.097	1.980	0.519	0.304
Tan	4	10014601	10014900	0.000	0.371	0.025	0.061
Tan	4	10048501	10048800	0.265	0.981	0.239	0.623
Tan	4	10052701	10053000	2.975	4.096	2.241	3.175
Tan	4	10074601	10074900	0.347	0.656	0.210	0.335
Tan	4	10101901	10102200	0.105	0.725	0.234	0.335
Tan	4	10194301	10194600	0.132	0.420	0.165	0.112
Tan	4	11047501	11047800	0.637	0.983	0.633	0.654
Tan	4	11482801	11483100	0.187	0.375	0.210	0.527
Tan	4	12073801	12074100	0.503	1.380	0.473	0.574
Tan	4	12403501	12403800	0.400	0.629	0.395	0.254
Tan	4	12414301	12414600	0.536	1.764	0.517	0.857
Tan	4	12414601	12414900	0.398	0.963	0.239	0.492
Tan	4	12460201	12460500	0.557	1.214	0.731	0.274
Tan	4	12650701	12651000	0.847	1.762	0.818	0.441
Tan	4	13031401	13031700	0.507	0.213	0.535	0.522
Tan	4	13137301	13137600	0.026	0.423	0.082	0.223
Tan	4	13229101	13229400	0.503	0.957	0.453	0.684
Tan	4	13277701	13278000	0.134	0.648	0.185	0.193
Tan	4	13841401	13841700	0.187	0.444	0.132	0.198
Tan	4	14121901	14122200	1.547	0.517	1.003	1.125
Tan	4	14185501	14185800	0.426	0.893	0.428	0.299
Tan	4	14241001	14241300	0.161	0.538	0.181	0.142
Tan	4	14415601	14415900	0.342	0.862	0.428	0.421

Tan	4	14729401	14729700	0.424	1.053	0.428	0.644
Tan	4	15012901	15013200	0.265	0.629	0.214	0.132
Tan	4	15013201	15013500	0.295	0.629	0.127	0.193
Tan	4	15021901	15022200	0.423	0.933	0.437	0.268
Tan	4	15054301	15054600	0.240	0.538	0.292	0.173
Tan	4	15295801	15296100	1.544	0.843	1.460	1.526
Tan	4	15493801	15494100	2.255	1.526	2.764	2.778
Tan	4	15626401	15626700	0.824	1.195	0.374	0.812
Tan	4	15660301	15660600	0.161	0.466	0.107	0.157
Tan	4	15730201	15730500	0.797	1.188	0.687	0.690
Tan	4	15733801	15734100	0.290	0.654	0.292	0.416
Tan	4	15739501	15739800	0.186	0.468	0.346	0.385
Tan	4	15804001	15804300	1.219	0.513	1.587	1.389
Tan	4	15805801	15806100	0.265	0.538	0.296	0.329
Tan	4	15810601	15810900	1.173	2.279	1.094	1.070
Tan	4	16082401	16082700	3.311	1.283	3.539	5.719
Tan	4	16104901	16105200	0.106	0.423	0.214	0.112
Tan	4	16125301	16125600	0.215	0.515	0.218	0.198
Tan	4	16286101	16286400	1.144	0.935	1.066	0.553
Tan	4	16592101	16592400	0.000	0.420	0.243	0.223
Tan	4	18173701	18174000	0.131	0.560	0.243	0.198
Tan	4	18910201	18910500	0.532	0.352	0.477	0.553
Tan	4	18977401	18977700	0.134	0.446	0.082	0.101
Tan	4	19230001	19230300	0.345	0.584	0.346	0.324
Tan	4	19475101	19475400	0.160	0.515	0.189	0.167
Tan	4	19498501	19498800	0.452	0.940	0.531	0.329
Tan	4	20124601	20124900	0.132	0.775	0.132	0.497
Tan	4	21171301	21171600	0.879	1.513	0.905	0.806
Tan	4	22166401	22166700	0.027	0.420	0.054	0.187
Tan	4	22254001	22254300	0.240	0.631	0.078	0.051
Tan	4	22497301	22497600	1.412	2.688	0.976	0.720
Tan	4	22921501	22921800	0.447	1.219	0.517	0.411
Tan	4	23303401	23303700	0.160	0.448	0.160	0.162
Tan	4	24507001	24507300	2.341	3.043	4.003	3.803
Tan	4	24943201	24943500	1.010	0.773	0.477	0.360
Tan	4	24994501	24994800	0.186	0.697	0.156	0.248
Tan	4	25119301	25119600	1.944	2.999	1.647	1.865
Tan	4	25503301	25503600	0.215	0.586	0.234	0.187
Tan	4	25641601	25641900	0.161	0.562	0.317	0.112
Tan	4	25724101	25724400	0.266	0.657	0.283	0.081
Tan	4	26219701	26220000	0.584	0.890	0.448	0.573
Tan	4	26907301	26907600	0.187	0.611	0.321	0.167
Tan	4	27917401	27917700	0.982	0.747	0.638	0.497
Tan	4	28183201	28183500	0.821	1.682	0.742	0.654
Tan	4	28424701	28425000	0.586	0.910	0.560	0.563
Tan	4	28479901	28480200	3.270	2.311	3.168	3.006
Tan	4	28698601	28698900	0.637	0.446	0.214	0.223
Tan	4	30216301	30216600	0.027	0.446	0.292	0.329
Tan	4	30625501	30625800	2.016	1.174	1.884	2.028
Tan	4	30720301	30720600	0.347	0.562	0.267	0.391
Tan	4	30815701	30816000	0.081	0.517	0.082	0.137
Tan	4	30901201	30901500	0.984	1.500	0.745	0.669
Tan	4	31106101	31106400	0.211	0.564	0.234	0.218
Tan	4	31161601	31161900	0.479	0.890	0.506	0.472
Tan	4	31226101	31226400	0.555	1.096	0.493	0.421
Tan	4	31230001	31230300	0.187	0.466	0.263	0.198
Tan	4	31230601	31230900	0.897	2.158	1.164	0.862
Tan	4	31230901	31231200	1.008	1.852	1.016	0.939
Tan	4	31626901	31627200	0.663	1.199	0.671	0.497
Tan	4	32098801	32099100	0.318	0.770	0.272	0.243
Tan	4	32345401	32345700	0.160	0.395	0.132	0.248
Tan	4	32442601	32442900	0.160	0.442	0.214	0.112
Tan	4	33717901	33718200	0.893	2.263	0.408	0.527
Tan	4	33718201	33718500	0.663	1.468	0.379	0.426
Tan	4	33919801	33920100	0.266	0.466	0.346	0.304
Tan	4	35528701	35529000	0.558	0.232	0.609	0.446
Tan	4	35649901	35650200	0.239	0.515	0.185	0.329

Tan	4	36708901	36709200	0.239	0.491	0.267	0.162
Tan	4	37119601	37119900	0.189	0.654	0.029	0.132
Tan	4	37352101	37352400	0.760	2.279	1.057	0.715
Tan	4	37625401	37625700	0.398	0.723	0.136	0.279
Tan	4	37826401	37826700	0.160	0.607	0.292	0.142
Tan	4	37893001	37893300	1.590	2.674	1.531	1.161
Tan	4	38877001	38877300	0.239	0.470	0.025	0.086
Tan	4	39241501	39241800	0.053	0.539	0.107	0.355
Tan	4	39561601	39561900	0.505	0.350	0.502	0.056
Tan	4	39836401	39836700	0.187	1.068	0.370	0.178
Tan	4	40153501	40153800	0.955	2.367	1.003	1.246
Tan	4	40617001	40617300	0.131	0.420	0.049	0.142
Tan	4	41256301	41256600	1.223	0.822	1.489	1.354
Tan	4	41870401	41870700	0.345	0.513	0.317	0.304
Tan	4	41877901	41878200	0.211	0.657	0.399	0.193
Tan	4	42052201	42052500	0.187	0.519	0.160	0.081
Tan	4	42629401	42629700	0.426	0.118	0.609	0.477
Tan	4	42992701	42993000	0.136	0.395	0.107	0.025
Tan	4	43053301	43053600	0.374	0.562	0.078	0.167
Tan	4	43535101	43535400	0.186	0.491	0.165	0.132
Tan	4	43631401	43631700	0.555	1.262	0.390	0.751
Tan	4	43922101	43922400	0.213	0.418	0.132	0.167
Tan	4	43930801	43931100	0.290	0.699	0.218	0.324
Tan	4	44086201	44086500	0.289	0.723	0.321	0.360
Tan	4	44104801	44105100	0.371	0.562	0.370	0.304
Tan	4	44353501	44353800	0.027	0.378	0.000	0.025
Tan	4	44516701	44517000	0.187	0.609	0.267	0.223
Tan	4	44580601	44580900	0.400	0.584	0.477	0.416
Tan	4	44584801	44585100	0.161	0.609	0.078	0.122
Tan	4	44752501	44752800	0.158	0.652	0.296	0.355
Tan	4	45653401	45653700	0.853	1.433	0.930	0.720
Tan	4	45902401	45902700	0.345	0.633	0.296	0.243
Tan	4	46145401	46145700	1.034	1.828	1.057	0.958
Tan	4	46493401	46493700	0.294	0.770	0.399	0.360
Tan	4	46550401	46550700	0.426	1.266	0.370	0.228
Tan	4	46585501	46585800	1.750	3.073	1.877	1.861
Tan	4	46693801	46694100	0.265	0.466	0.239	0.223
Tan	4	46874401	46874700	1.116	1.540	0.843	1.075
Tan	4	46921801	46922100	0.158	0.444	0.049	0.081
Tan	4	47159401	47159700	0.715	1.099	0.740	0.603
Tan	4	47275201	47275500	0.213	0.607	0.263	0.365
Tan	4	47490601	47490900	0.187	0.511	0.210	0.248
Tan	4	47773501	47773800	0.213	0.511	0.111	0.218
Tan	4	47982901	47983200	0.292	0.491	0.317	0.223
Tan	4	48139801	48140100	0.553	1.103	0.312	0.497
Tan	4	48595801	48596100	0.239	0.493	0.243	0.187
Tan	4	48885901	48886200	0.369	1.009	0.350	0.243
Tan	4	48886201	48886500	1.037	2.036	0.987	0.497
Tan	4	49179601	49179900	0.187	0.517	0.074	0.218
Tan	4	49298401	49298700	0.081	0.425	0.078	0.076
Tan	4	50020801	50021100	0.000	0.541	0.049	0.092
Tan	4	50073601	50073900	0.294	0.605	0.239	0.248
Tan	4	50564701	50565000	0.186	0.564	0.111	0.203
Tan	4	50565001	50565300	0.081	0.468	0.000	0.228
Tan	4	51117601	51117900	0.481	1.242	0.584	0.918
Tan	4	51323401	51323700	0.613	0.938	0.642	0.466
Tan	4	51616201	51616500	0.824	1.169	0.798	1.506
Tan	4	51633601	51633900	1.353	1.099	1.514	0.725
Tan	4	51776701	51777000	0.316	1.051	0.296	0.436
Tan	4	51870301	51870600	0.184	0.538	0.272	0.167
Tan	4	52110001	52110300	0.558	1.006	0.395	0.517
Tan	4	52123501	52123800	0.294	0.792	0.399	0.355
Tan	4	53346601	53346900	0.187	0.676	0.239	0.472
Tan	4	53401501	53401800	0.292	0.562	0.214	0.173
Tan	4	53414101	53414400	0.158	0.753	0.374	0.416
Tan	4	54259201	54259500	0.345	0.680	0.346	0.391
Tan	4	54275701	54276000	0.158	0.796	0.252	0.238

Tan	4	55242601	55242900	1.247	0.350	1.016	0.826
Tan	4	55633501	55633800	0.239	0.654	0.395	0.274
Tan	4	55779301	55779600	0.215	0.629	0.205	0.268
Tan	4	56318101	56318400	0.160	0.609	0.136	0.187
Tan	4	56366101	56366400	0.694	1.096	0.374	0.614
Tan	4	57060001	57060300	0.319	0.539	0.234	0.051
Tan	4	58015201	58015500	0.666	0.961	0.695	0.411
Tan	4	58247401	58247700	0.213	0.446	0.239	0.173
Tan	4	58399801	58400100	0.242	0.588	0.239	0.106
Tan	4	59101201	59101500	0.158	0.494	0.185	0.162
Tan	4	59483101	59483400	1.135	2.401	1.005	1.197
Tan	4	60004801	60005100	0.558	0.376	0.189	0.608
Tan	4	60011101	60011400	1.303	2.339	1.316	0.918
Tan	4	60178201	60178500	1.965	2.264	1.723	1.658
Tan	4	60209101	60209400	0.531	1.171	0.600	0.481
Tan	4	60222901	60223200	0.211	0.446	0.160	0.218
Tan	4	60466201	60466500	0.828	1.429	0.856	0.963
Tan	4	60702001	60702300	0.239	0.446	0.243	0.086
Tan	4	60906601	60906900	0.106	0.539	0.346	0.193
Tan	4	60919501	60919800	0.771	1.783	0.698	0.533
Tan	4	60919801	60920100	0.481	1.335	0.374	0.375
Tan	4	60924301	60924600	1.018	2.309	1.159	0.674
Tan	4	60933001	60933300	0.161	0.980	0.308	0.295
Tan	4	60940801	60941100	0.611	0.092	0.687	0.385
Tan	4	60990001	60990300	0.134	0.586	0.111	0.198
Tan	4	61033501	61033800	0.160	0.732	0.214	0.081
Tan	4	61060201	61060500	0.081	0.468	0.049	0.051
Tan	4	61076701	61077000	0.424	0.562	0.243	0.248
Tan	4	61402201	61402500	0.481	1.509	0.448	0.512
Tan	4	61588501	61588800	0.584	0.279	0.588	0.553
Tan	4	61642801	61643100	0.160	0.466	0.239	0.360
Tan	4	61649401	61649700	0.266	0.588	0.234	0.274
Tan	4	62002801	62003100	0.531	0.798	0.160	0.193
Tan	4	62252701	62253000	0.319	0.985	0.638	0.588
Tan	4	62593201	62593500	0.316	0.723	0.189	0.193
Tan	4	63100501	63100800	0.613	0.421	0.613	0.223
Tan	4	63108301	63108600	0.374	0.165	0.555	0.385
Tan	4	63656701	63657000	0.373	0.652	0.395	0.193
Tan	4	63698401	63698700	0.984	0.425	1.206	1.197
Tan	4	63843301	63843600	0.345	0.468	0.263	0.360
Tan	4	64013401	64013700	1.087	0.493	0.885	0.938
Tan	4	64088701	64089000	1.355	0.631	1.382	1.247
Tan	4	64448701	64449000	0.802	1.452	0.716	0.329
Tan	4	64663201	64663500	0.265	0.511	0.132	0.025
Tan	4	64986901	64987200	0.424	1.058	0.439	0.340
Tan	4	65299501	65299800	0.186	0.395	0.136	0.025
Tan	4	65415601	65415900	0.318	0.656	0.210	0.340
Tan	4	65447101	65447400	0.323	1.380	0.510	0.751
Tan	4	65520901	65521200	1.219	2.152	1.168	1.404
Tan	4	65766601	65766900	0.452	0.768	0.239	0.380
Tan	4	65928301	65928600	0.373	0.515	0.288	0.664
Tan	4	66212101	66212400	1.013	1.760	1.094	1.120
Tan	4	66231301	66231600	0.211	0.702	0.210	0.294
Tan	4	66445201	66445500	2.818	3.669	1.409	1.982
Tan	4	66445501	66445800	0.902	1.167	0.613	0.796
Tan	4	66454801	66455100	0.186	0.444	0.078	0.304
Tan	4	66665701	66666000	0.239	0.466	0.132	0.223
Tan	4	66797401	66797700	0.105	0.470	0.132	0.147
Tan	4	66818701	66819000	0.318	0.491	0.132	0.254
Tan	4	67375801	67376100	3.196	4.398	3.026	3.179
Tan	4	67376101	67376400	0.802	2.584	0.838	0.958
Tan	4	67438801	67439100	0.371	0.094	0.526	0.446
Tan	4	67473601	67473900	1.353	1.706	0.852	0.927
Tan	4	67473901	67474200	1.353	1.972	0.901	0.913
Tan	4	67835401	67835700	1.491	2.182	0.967	1.607
Tan	4	67858801	67859100	0.160	0.491	0.214	0.329
Tan	4	67924801	67925100	0.455	0.094	0.453	0.360

Tan	4	68040601	68040900	0.373	0.845	0.526	0.421
Tan	4	68466601	68466900	0.242	0.820	0.296	0.248
Tan	4	68466901	68467200	0.156	1.084	0.288	0.254
Tan	4	68583601	68583900	0.132	0.420	0.160	0.132
Tan	4	68869501	68869800	0.187	0.581	0.165	0.243
Tan	4	69427801	69428100	0.665	0.210	0.506	0.852
Tan	4	69570001	69570300	0.292	0.562	0.288	0.304
Tan	4	69661801	69662100	0.186	0.328	0.502	0.167
Tan	4	69767401	69767700	0.295	0.729	0.325	0.248
Tan	4	69828601	69828900	0.371	0.584	0.156	0.329
Tan	4	69865501	69865800	0.558	1.141	0.575	0.355
Tan	4	69900601	69900900	0.613	0.279	0.695	0.558
Tan	4	70033801	70034100	0.213	0.446	0.127	0.198
Tan	4	70371301	70371600	0.158	0.538	0.136	0.203
Tan	4	70398601	70398900	0.453	0.350	0.239	0.664
Tan	4	70536601	70536900	0.266	0.751	0.194	0.243
Tan	4	70730401	70730700	0.319	0.187	0.453	0.497
Tan	4	70782901	70783200	0.297	1.099	0.439	0.482
Tan	4	70851001	70851300	0.586	1.543	0.597	0.724
Tan	4	70869901	70870200	0.345	0.513	0.292	0.299
Tan	4	70956601	70956900	0.237	0.942	0.181	0.187
Tan	4	70956901	70957200	0.215	0.848	0.181	0.167
Tan	4	71012701	71013000	0.349	0.633	0.292	0.248
Tan	4	71075401	71075700	0.799	1.384	0.983	0.933
Tan	4	71407801	71408100	3.333	2.001	3.950	3.336
Tan	4	71890801	71891100	0.608	1.309	0.662	0.664
Tan	4	72432001	72432300	1.226	0.933	1.193	0.466
Tan	4	72573901	72574200	0.319	0.607	0.259	0.137
Tan	4	72957301	72957600	0.239	0.584	0.111	0.299
Tan	4	73683601	73683900	0.532	1.476	0.435	0.426
Tan	4	73683901	73684200	0.455	1.148	0.424	0.335
Tan	4	74335801	74336100	0.613	0.397	0.531	0.274
Tan	4	74517301	74517600	1.463	1.008	1.197	1.384
Tan	4	74612101	74612400	0.189	0.586	0.185	0.218
Tan	4	74731201	74731500	0.292	0.656	0.312	0.385
Tan	4	74892001	74892300	0.213	0.470	0.156	0.187
Tan	4	74946901	74947200	0.292	0.489	0.078	0.309
Tan	4	74991301	74991600	0.213	0.566	0.181	0.086
Tan	4	75039001	75039300	0.879	1.993	1.184	0.882
Tan	4	75932101	75932400	0.477	0.279	0.025	0.446
Tan	4	76059001	76059300	0.531	1.049	0.745	0.806
Tan	4	76249801	76250100	0.718	0.352	0.054	0.106
Tan	4	76421701	76422000	0.136	0.468	0.165	0.162
Tan	4	76585801	76586100	0.186	0.468	0.239	0.162
Tan	4	76725601	76725900	1.037	1.407	0.823	0.837
Tan	4	76801201	76801500	5.078	3.626	4.460	4.497
Tan	4	76803301	76803600	0.239	0.704	0.341	0.350
Tan	4	76836301	76836600	0.557	1.073	0.486	0.436
Tan	4	76845901	76846200	0.213	0.541	0.210	0.274
Tan	4	76991101	76991400	2.415	1.300	2.702	2.196
Tan	4	77023801	77024100	4.841	3.096	5.214	5.841
Tan	4	77046901	77047200	0.106	0.448	0.185	0.056
Tan	4	77140201	77140500	1.597	0.813	1.489	1.328
Tan	4	77238601	77238900	0.319	0.515	0.165	0.279
Tan	4	77240101	77240400	0.371	0.652	0.909	0.391
Tan	4	77295301	77295600	0.213	0.562	0.160	0.304
Tan	4	77363701	77364000	2.097	0.611	1.400	1.795
Tan	4	77364001	77364300	3.964	2.154	2.811	3.291
Tan	4	77400601	77400900	0.081	0.519	0.136	0.112
Tan	4	77413501	77413800	0.134	0.517	0.185	0.167
Tan	4	77489701	77490000	0.986	1.356	0.575	0.634
Tan	4	77553301	77553600	0.108	0.583	0.127	0.101
Tan	4	77986801	77987100	1.516	2.036	1.505	1.425
Tan	4	78237001	78237300	0.318	0.959	0.292	0.406
Tan	4	78472801	78473100	1.060	0.230	0.827	0.628
Tan	4	78565801	78566100	0.055	0.637	0.074	0.051
Tan	4	78714901	78715200	0.079	0.401	0.103	0.056

Tan	4	78763501	78763800	0.211	0.442	0.189	0.000
Tan	4	78918001	78918300	1.199	0.564	1.665	1.105
Tan	4	79131301	79131600	0.266	0.631	0.301	0.360
Tan	4	79144501	79144800	0.105	0.539	0.214	0.304
Tan	4	79279201	79279500	0.132	0.330	0.588	0.112
Tan	4	79461001	79461300	1.281	2.143	1.494	1.303
Tan	4	79518901	79519200	0.479	2.734	0.983	0.404
Tan	4	81232501	81232800	0.374	0.657	0.317	0.167
Tan	4	81250201	81250500	1.278	2.221	0.811	1.044
Tan	4	82207501	82207800	0.319	0.607	0.160	0.218
Tan	4	82555201	82555500	0.560	1.008	0.350	0.497
Tan	4	82788301	82788600	0.213	0.680	0.263	0.056
Tan	4	82818901	82819200	0.718	1.099	0.642	0.731
Tan	4	83606101	83606400	0.082	0.515	0.210	0.218
Tan	4	84038701	84039000	0.266	0.466	0.160	0.309
Tan	4	85348201	85348500	0.082	0.446	0.000	0.167
Tan	4	85531501	85531800	0.344	0.678	0.234	0.441
Tan	4	86016901	86017200	1.116	2.244	1.080	0.837
Tan	4	86097001	86097300	0.290	0.749	0.403	0.385
Tan	4	86134501	86134800	0.476	1.096	0.662	0.690
Tan	4	86179501	86179800	0.290	0.867	0.312	0.203
Tan	4	86441701	86442000	0.747	0.168	0.646	0.801
Tan	4	86984401	86984700	0.639	0.792	0.584	0.329
Tan	4	87437401	87437700	0.505	0.584	0.477	0.304
Tan	4	87508501	87508800	0.347	0.723	0.288	0.350
Tan	4	87644101	87644400	0.211	0.513	0.103	0.132
Tan	4	87730801	87731100	0.239	0.583	0.370	0.193
Tan	4	88169401	88169700	0.294	0.605	0.205	0.167
Tan	4	88212001	88212300	0.558	0.466	0.267	0.248
Tan	4	88455301	88455600	0.213	0.375	0.214	0.527
Tan	4	88774201	88774500	0.239	0.680	0.448	0.391
Tan	4	88823101	88823400	0.824	1.358	0.925	0.452
Tan	4	88948501	88948800	0.534	0.867	1.177	0.466
Tan	4	89598301	89598600	1.058	0.380	1.465	1.262
Tan	4	89598601	89598900	1.982	0.678	2.706	2.241
Tan	4	89612101	89612400	0.106	0.470	0.214	0.248
Tan	4	89948701	89949000	0.081	0.303	0.477	0.117
Tan	4	89985901	89986200	0.321	0.727	0.189	0.299
Tan	4	90137401	90137700	0.106	0.635	0.123	0.173
Tan	4	90259201	90259500	1.360	0.253	1.023	1.145
Tan	4	90325201	90325500	0.132	0.465	0.185	0.112
Tan	4	90325501	90325800	0.215	0.560	0.218	0.193
Tan	4	90680101	90680400	0.026	0.845	0.058	0.051
Tan	4	90680401	90680700	0.027	0.584	0.049	0.162
Tan	4	90693301	90693600	1.781	3.081	1.612	2.332
Tan	4	90871801	90872100	0.265	0.676	0.214	0.263
Tan	4	91437001	91437300	0.186	0.584	0.272	0.335
Tan	4	91437301	91437600	1.008	2.040	1.012	1.065
Tan	4	91678801	91679100	0.531	0.886	0.312	0.578
Tan	4	91950001	91950300	0.186	0.584	0.087	0.112
Tan	4	92008201	92008500	0.265	0.536	0.346	0.274
Tan	4	92037301	92037600	0.106	0.420	0.239	0.248
Tan	4	92055601	92055900	1.150	0.142	1.209	1.496
Tan	4	92115601	92115900	0.402	1.234	0.667	0.553
Tan	4	92134201	92134500	0.689	1.240	0.497	0.517
Tan	4	92150101	92150400	0.423	1.240	0.629	0.466
Tan	4	92150401	92150700	0.239	0.820	0.424	0.446
Tan	4	92154901	92155200	0.450	0.116	0.506	0.360
Tan	4	92190001	92190300	0.400	1.126	0.417	0.441
Tan	4	92221201	92221500	3.318	6.312	2.204	3.767
Tan	4	92230501	92230800	0.215	0.420	0.078	0.218
Tan	4	92236801	92237100	0.187	0.684	0.267	0.137
Tan	4	92292301	92292600	1.037	2.025	0.753	1.328
Tan	4	92331001	92331300	1.111	2.890	1.507	1.434
Tan	4	92331301	92331600	1.089	2.993	1.427	0.994
Tan	4	92352601	92352900	3.160	4.474	2.720	3.016
Tan	4	92464801	92465100	2.550	3.665	2.082	2.458

Tan	4	92583301	92583600	0.079	0.440	0.107	0.056
Tan	4	92615101	92615400	0.637	0.890	0.691	1.055
Tan	4	92872801	92873100	0.426	0.633	0.267	0.837
Tan	4	92950501	92950800	0.774	1.335	0.646	0.913
Tan	4	93414301	93414600	0.450	0.727	0.321	0.309
Tan	4	93473101	93473400	1.434	1.967	0.934	1.369
Tan	4	93628801	93629100	0.558	0.820	0.555	0.502
Tan	4	93699001	93699300	0.237	0.950	0.321	0.304
Tan	4	93748501	93748800	0.423	0.727	0.346	0.218
Tan	4	93837001	93837300	0.371	0.721	0.346	0.385
Tan	4	93846601	93846900	0.587	0.961	0.613	0.603
Tan	4	93860701	93861000	0.479	0.676	0.531	0.360
Tan	4	93865201	93865500	0.690	1.189	0.588	0.690
Tan	4	94398601	94398900	2.362	3.279	2.419	1.723
Tan	4	94684501	94684800	2.183	0.230	2.967	2.427
Tan	4	94684801	94685100	3.023	0.830	4.070	2.863
Tan	4	94744801	94745100	0.132	1.491	0.267	0.182
Tan	4	94828501	94828800	0.979	1.967	1.465	1.156
Tan	4	94837801	94838100	0.187	0.420	0.103	0.142
Tan	4	94838101	94838400	0.134	0.395	0.132	0.086
Tan	4	95056801	95057100	0.479	0.891	0.424	0.365
Tan	4	95427001	95427300	1.173	0.745	1.168	1.192
Tan	4	95571901	95572200	1.091	0.678	1.650	1.298
Tan	4	95654401	95654700	0.189	0.586	0.136	0.112
Tan	4	95655001	95655300	1.816	3.855	1.505	1.532
Tan	4	95655301	95655600	0.350	1.592	0.247	0.178
Tan	4	95658001	95658300	0.156	2.030	0.189	0.117
Tan	4	95658301	95658600	0.105	1.751	0.169	0.112
Tan	4	95758501	95758800	0.529	0.772	0.506	0.248
Tan	4	95773801	95774100	0.574	1.796	0.275	0.340
Tan	4	95810101	95810400	0.508	1.264	0.765	0.877
Tan	4	95824501	95824800	0.240	0.468	0.189	0.254
Tan	4	95825401	95825700	0.211	0.631	0.230	0.193
Tan	4	95847001	95847300	0.318	1.626	0.470	0.482
Tan	4	95847301	95847600	0.129	0.796	0.174	0.106
Tan	4	95847601	95847900	1.063	3.309	0.914	0.724
Tan	4	95850601	95850900	0.156	0.959	0.189	0.238
Tan	4	95850901	95851200	0.158	0.491	0.140	0.132
Tan	4	95889001	95889300	1.144	0.375	0.905	0.669
Tan	4	95890501	95890800	0.216	0.725	0.325	0.243
Tan	4	95890801	95891100	0.079	1.287	0.082	0.025
Tan	4	95902801	95903100	0.210	0.749	0.403	0.391
Tan	4	95993401	95993700	2.337	0.498	1.914	1.918
Tan	4	96091501	96091800	0.878	0.465	0.967	0.857
Tan	4	96101701	96102000	0.558	0.373	0.239	0.218
Tan	4	96118501	96118800	0.503	1.056	0.395	0.517
Tan	4	96141001	96141300	0.558	0.281	0.448	0.167
Tan	4	96214801	96215100	0.828	1.405	0.740	0.796
Tan	4	96383701	96384000	0.266	0.491	0.288	0.167
Tan	4	96411901	96412200	1.597	0.989	1.645	1.516
Tan	4	96476401	96476700	1.247	2.150	1.066	1.511
Tan	4	96798301	96798600	0.186	0.515	0.029	0.167
Tan	4	96801901	96802200	0.986	0.470	1.016	0.852
Tan	4	96841801	96842100	0.373	0.957	0.366	0.284
Tan	4	96894901	96895200	0.557	0.189	0.399	0.776
Tan	4	96900001	96900300	0.424	0.629	0.214	0.329
Tan	4	97058401	97058700	0.213	0.605	0.107	0.167
Tan	4	97078501	97078800	0.263	1.416	0.439	0.405
Tan	4	97078801	97079100	0.105	0.773	0.370	0.274
Tan	4	97131901	97132200	0.161	0.420	0.054	0.137
Tan	4	97263301	97263600	1.944	2.341	1.621	1.845
Tan	4	97408801	97409100	0.106	0.494	0.074	0.137
Tan	4	97409701	97410000	0.240	0.444	0.189	0.162
Tan	4	97461601	97461900	0.081	0.637	0.082	0.086
Tan	4	97559401	97559700	0.720	1.056	0.292	0.771
Tan	4	97619101	97619400	0.716	1.968	0.774	0.790
Tan	4	97762201	97762500	1.684	3.272	1.067	0.791

Tan	4	98022601	98022900	0.318	0.723	0.259	0.355
Tan	4	98043001	98043300	0.344	0.890	0.667	0.365
Tan	4	98130901	98131200	0.265	0.701	0.169	0.025
Tan	4	98170201	98170500	0.424	0.792	0.234	0.137
Tan	4	98231701	98232000	0.934	2.013	0.775	0.740
Tan	4	98250901	98251200	0.237	0.586	0.239	0.279
Tan	4	98320801	98321100	0.474	1.717	0.531	0.862
Tan	4	98374201	98374500	1.620	2.641	1.358	1.471
Tan	4	98374501	98374800	1.808	2.249	1.387	1.303
Tan	4	98409901	98410200	0.160	0.611	0.160	0.162
Tan	4	98527801	98528100	2.075	3.281	1.696	1.926
Tan	4	98541601	98541900	0.266	0.751	0.239	0.198
Tan	4	98637001	98637300	0.132	0.420	0.210	0.061
Tan	4	99168601	99168900	0.452	0.562	0.346	0.385
Tan	4	99218401	99218700	0.213	0.755	0.288	0.081
Tan	4	99226801	99227100	0.263	0.699	0.350	0.335
Tan	4	99289501	99289800	0.424	0.773	0.510	0.527
Tan	4	99318001	99318300	0.292	0.539	0.239	0.162
Tan	4	99360601	99360900	0.290	0.747	0.350	0.167
Tan	4	99374701	99375000	0.053	0.513	0.189	0.117
Tan	4	99384301	99384600	0.453	1.528	0.276	0.380
Tan	4	99385801	99386100	0.373	0.701	0.078	0.137
Tan	4	99470101	99470400	0.953	1.950	1.094	0.736
Tan	4	99543601	99543900	0.161	0.489	0.239	0.279
Tan	4	99558301	99558600	0.781	2.611	0.889	1.425
Tan	4	99816301	99816600	0.689	1.054	0.506	0.664
Tan	4	100117501	100117800	0.615	0.985	0.403	0.578
Tan	4	100200301	100200600	0.692	1.377	0.646	0.659
Tan	4	100914001	100914300	0.826	1.543	0.872	0.603
Tan	4	100914601	100914900	0.610	1.098	0.437	0.385
Tan	4	101071201	101071500	0.156	0.678	0.132	0.112
Tan	4	101072701	101073000	0.718	1.725	0.517	0.557
Tan	4	101122201	101122500	0.189	0.562	0.234	0.274
Tan	4	101124001	101124300	0.108	0.603	0.185	0.254
Tan	4	101136601	101136900	0.482	0.983	0.526	0.573
Tan	4	101247601	101247900	0.239	0.560	0.288	0.173
Tan	4	101259901	101260200	0.292	0.538	0.346	0.193
Tan	4	101583901	101584200	0.237	0.824	0.000	0.025
Tan	4	101584201	101584500	0.289	1.034	0.025	0.025
Tan	4	101627401	101627700	0.423	1.079	0.388	0.304
Tan	4	101627701	101628000	0.237	0.468	0.160	0.198
Tan	4	102061201	102061500	0.134	0.489	0.078	0.081
Tan	4	102173101	102173400	1.008	1.566	0.852	1.085
Tan	4	102216001	102216300	0.211	0.446	0.160	0.117
Tan	4	102233101	102233400	1.297	2.394	0.856	1.267
Tan	4	102432001	102432300	1.145	0.305	0.905	0.684
Tan	4	102432301	102432600	1.249	0.330	1.525	1.226
Tan	4	102437701	102438000	0.292	0.983	0.588	0.147
Tan	4	102469501	102469800	1.910	4.437	1.920	2.100
Tan	4	102475501	102475800	1.041	2.184	0.522	1.100
Tan	4	102759301	102759600	0.902	1.453	0.872	1.029
Tan	4	102894601	102894900	0.292	0.562	0.234	0.355
Tan	4	102897901	102898200	1.817	3.034	1.799	1.967
Tan	4	102929401	102929700	0.161	0.562	0.082	0.086
Tan	4	102944101	102944400	3.086	2.244	1.469	1.987
Tan	4	103048801	103049100	0.450	0.890	0.468	0.385
Tan	4	103049401	103049700	4.643	2.169	4.650	5.255
Tan	4	103087801	103088100	6.996	5.462	7.005	6.515
Tan	4	103088101	103088400	8.069	5.747	8.407	7.843
Tan	4	103217101	103217400	0.242	0.678	0.156	0.355
Tan	4	103232401	103232700	0.081	0.701	0.243	0.153
Tan	4	103232701	103233000	0.606	1.764	0.691	0.594
Tan	4	103246801	103247100	1.842	3.312	1.023	1.688
Tan	4	103348201	103348500	0.106	0.560	0.111	0.051
Tan	4	103416601	103416900	0.108	0.491	0.156	0.228
Tan	4	103418101	103418400	0.081	0.465	0.132	0.248
Tan	4	103498201	103498500	0.826	1.448	0.700	0.791

Tan	4	103692901	103693200	0.634	1.099	0.671	0.603
Tan	4	103713301	103713600	0.400	1.129	0.481	0.426
Tan	4	103858501	103858800	0.426	0.749	0.448	0.299
Tan	4	104089801	104090100	0.265	0.837	0.301	0.243
Tan	4	104098801	104099100	0.160	0.466	0.165	0.223
Tan	4	104102101	104102400	0.803	1.452	0.682	0.715
Tan	4	104133901	104134200	0.211	0.446	0.239	0.112
Tan	4	104187301	104187600	0.799	1.987	0.390	0.795
Tan	4	104202001	104202300	0.294	0.682	0.136	0.335
Tan	4	104226301	104226600	1.039	2.051	0.941	0.760
Tan	4	104229001	104229300	0.737	2.047	0.522	0.817
Tan	4	104239201	104239500	0.106	1.680	0.500	0.076
Tan	4	104252101	104252400	0.611	1.313	0.332	0.350
Tan	4	104256001	104256300	0.103	0.966	0.025	0.025
Tan	4	104261401	104261700	0.211	0.558	0.078	0.274
Tan	4	104274901	104275200	2.746	1.362	2.628	2.611
Tan	4	104282101	104282400	1.589	3.984	0.678	1.122
Tan	4	104282401	104282700	0.928	2.611	0.473	0.568
Tan	4	104325901	104326200	0.479	0.843	0.564	0.274
Tan	4	104331601	104331900	1.219	2.534	1.064	0.816
Tan	4	104331901	104332200	0.373	1.071	0.403	0.132
Tan	4	104348401	104348700	0.371	0.775	0.325	0.279
Tan	4	104402701	104403000	0.106	0.564	0.107	0.228
Tan	4	104576701	104577000	0.428	1.641	0.428	1.136
Tan	4	104674501	104674800	1.168	2.444	1.037	1.480
Tan	4	104701801	104702100	0.215	0.794	0.136	0.416
Tan	4	104742901	104743200	1.039	3.768	0.881	1.523
Tan	4	104743201	104743500	1.176	4.021	1.190	1.463
Tan	4	104777101	104777400	2.584	3.184	2.049	2.606
Tan	4	104827501	104827800	2.365	1.131	2.348	1.998
Tan	4	104853601	104853900	0.242	0.629	0.185	0.329
Tan	4	105256801	105257100	1.884	1.079	1.806	1.739
Tan	4	105342301	105342600	1.549	3.939	1.929	2.013
Tan	4	105378901	105379200	0.156	0.491	0.058	0.112
Tan	4	105422401	105422700	0.292	0.701	0.337	0.324
Tan	4	105636601	105636900	0.958	0.534	0.959	0.877
Tan	4	105660301	105660600	0.423	0.747	0.424	0.411
Tan	4	105699001	105699300	0.450	0.888	0.296	0.553
Tan	4	105699301	105699600	0.666	1.214	0.617	0.796
Tan	4	105719101	105719400	0.823	1.242	0.774	0.862
Tan	4	105946201	105946500	0.181	1.118	0.259	0.000
Tan	4	106051801	106052100	0.161	0.607	0.296	0.254
Tan	4	106245301	106245600	0.479	0.837	0.477	0.380
Tan	4	106269601	106269900	0.215	0.985	0.305	0.344
Tan	4	106473901	106474200	0.077	0.395	0.136	0.112
Tan	4	106715401	106715700	0.718	0.560	0.395	0.882
Tan	4	106888801	106889100	0.266	0.140	0.477	0.279
Tan	4	107147401	107147700	0.799	0.350	0.584	0.416
Tan	4	107262601	107262900	2.045	1.659	1.436	1.323
Tan	4	107512201	107512500	0.213	0.470	0.218	0.167
Tan	4	107603701	107604000	0.479	0.955	0.390	0.263
Tan	4	108090301	108090600	0.932	0.702	1.066	0.994
Tan	4	108812701	108813000	0.371	0.839	0.428	0.441
Tan	4	108918001	108918300	0.079	0.656	0.058	0.248
Tan	4	109256101	109256400	0.134	0.303	0.103	0.497
Tan	4	109381801	109382100	0.345	0.938	0.551	0.162
Tan	4	109500601	109500900	0.426	0.674	0.370	0.274
Tan	4	109541401	109541700	1.462	0.633	1.478	1.760
Tan	4	109881901	109882200	0.424	1.779	0.713	0.832
Tan	4	110105701	110106000	0.531	0.678	0.346	0.441
Tan	4	110192701	110193000	1.303	1.731	0.831	1.222
Tan	4	110257501	110257800	0.644	1.262	0.461	0.360
Tan	4	110318701	110319000	3.270	2.875	2.299	2.398
Tan	4	110504101	110504400	1.273	2.292	1.971	0.725
Tan	4	110585701	110586000	0.874	1.474	0.736	0.857
Tan	4	110593801	110594100	0.479	1.442	0.509	0.573
Tan	4	110594101	110594400	0.479	1.278	0.296	0.380

Tan	4	110602201	110602500	0.673	1.448	0.617	0.720
Tan	4	110806801	110807100	0.132	0.472	0.078	0.117
Tan	4	110807101	110807400	0.557	1.976	0.468	0.710
Tan	4	110872201	110872500	0.079	0.609	0.078	0.106
Tan	4	110874301	110874600	1.839	0.753	2.345	1.824
Tan	4	110874601	110874900	1.386	0.189	1.592	1.540
Tan	4	110883301	110883600	3.794	1.337	3.377	3.970
Tan	4	110883601	110883900	2.815	1.200	2.492	2.931
Tan	4	110887201	110887500	1.324	0.236	1.551	1.313
Tan	4	110907901	110908200	2.526	3.066	2.171	3.737
Tan	4	110985001	110985300	0.318	0.583	0.103	0.218
Tan	4	111017701	111018000	0.265	0.584	0.263	0.274
Tan	4	111133501	111133800	0.484	1.150	0.535	0.436
Tan	4	111156301	111156600	0.347	0.562	0.185	0.385
Tan	4	111226201	111226500	0.323	0.936	0.584	0.299
Tan	4	111376201	111376500	0.611	1.191	0.354	0.608
Tan	4	111515401	111515700	0.239	0.493	0.132	0.223
Tan	4	111578101	111578400	0.163	0.654	0.160	0.248
Tan	4	111639001	111639300	0.266	0.446	0.267	0.223
Tan	4	111640801	111641100	1.387	1.850	0.847	1.430
Tan	4	111810901	111811200	0.982	1.642	0.366	0.893
Tan	4	111950401	111950700	2.758	2.150	2.789	2.855
Tan	4	112005601	112005900	1.329	0.485	1.522	1.161
Tan	4	112019701	112020000	1.011	1.746	2.214	0.832
Tan	4	112036501	112036800	0.213	0.539	0.210	0.137
Tan	4	112119301	112119600	2.287	1.517	1.674	1.876
Tan	4	112227901	112228200	0.505	1.236	0.542	0.466
Tan	4	112258801	112259100	0.213	0.442	0.267	0.081
Tan	4	112430101	112430400	0.347	0.538	0.103	0.051
Tan	4	112554001	112554300	0.397	0.745	0.292	0.329
Tan	4	112572301	112572600	0.186	0.446	0.160	0.162
Tan	4	112613101	112613400	0.292	0.489	0.292	0.248
Tan	4	112632601	112632900	0.235	0.629	0.136	0.162
Tan	4	112901701	112902000	1.355	2.753	1.016	1.216
Tan	4	112902001	112902300	1.836	4.574	1.779	2.229
Tan	4	113061901	113062200	0.237	0.796	0.408	0.385
Tan	4	113160601	113160900	0.345	0.466	0.317	0.360
Tan	4	113171701	113172000	1.194	2.158	0.943	1.070
Tan	4	113370601	113370900	0.424	0.678	0.132	0.213
Tan	4	113387101	113387400	3.830	4.164	2.769	3.285
Tan	4	113407201	113407500	0.187	0.796	0.243	0.365
Tan	4	113410501	113410800	2.923	4.199	2.751	3.108
Tan	4	113472001	113472300	0.871	1.922	0.930	1.044
Tan	4	113493301	113493600	0.131	0.745	0.252	0.051
Tan	4	113586001	113586300	0.610	1.221	0.584	0.751
Tan	4	113587801	113588100	0.373	0.584	0.267	0.355
Tan	4	113761801	113762100	2.691	5.025	2.887	2.449
Tan	4	113827201	113827500	0.292	1.062	0.210	0.167
Tan	4	113837701	113838000	1.297	2.197	1.538	1.278
Tan	4	113914501	113914800	0.878	0.350	0.588	0.715
Tan	4	113958301	113958600	5.173	2.701	3.888	5.071
Tan	4	113964901	113965200	0.156	0.684	0.132	0.173
Tan	4	114018601	114018900	0.981	1.285	0.847	0.669
Tan	4	114154501	114154800	0.052	0.511	0.152	0.162
Tan	4	114159301	114159600	0.452	0.586	0.082	0.223
Tan	4	114186301	114186600	0.637	0.983	0.560	0.659
Tan	4	114276301	114276600	0.319	0.491	0.214	0.583
Tan	4	114479101	114479400	0.052	0.513	0.185	0.248
Tan	4	114500701	114501000	0.833	2.175	1.161	0.877
Tan	4	114507601	114507900	0.823	1.287	0.687	0.918
Tan	4	114536101	114536400	0.240	1.082	0.267	0.284
Tan	4	114590701	114591000	3.242	1.641	2.323	2.200
Tan	4	114592801	114593100	0.505	0.375	0.292	0.304
Tan	4	114616201	114616500	2.552	4.754	3.148	3.376
Tan	4	114660901	114661200	2.336	1.126	2.325	2.748
Tan	4	114681001	114681300	0.344	1.006	0.580	0.538
Tan	4	114681901	114682200	0.981	1.616	0.896	0.502

Tan	4	114709801	114710100	2.921	1.603	3.065	2.820
Tan	4	114857401	114857700	0.266	1.056	0.259	0.365
Tan	4	114869401	114869700	0.106	0.541	0.214	0.248
Tan	4	114876901	114877200	0.055	0.775	0.432	0.137
Tan	4	114893401	114893700	2.121	1.571	0.901	0.907
Tan	4	114898801	114899100	0.637	1.352	0.901	0.913
Tan	4	114927301	114927600	0.294	0.869	0.424	0.583
Tan	4	114944701	114945000	0.586	0.796	0.473	0.360
Tan	4	114945001	114945300	0.615	0.935	0.506	0.380
Tan	4	115132801	115133100	0.369	0.820	0.325	0.315
Tan	4	115262701	115263000	1.249	2.382	1.113	0.998
Tan	4	115353001	115353300	0.823	1.755	0.823	0.887
Tan	4	115373701	115374000	1.299	0.536	1.451	1.627
Tan	4	115381801	115382100	0.534	1.431	0.568	0.735
Tan	4	115435801	115436100	0.268	0.678	0.103	0.248
Tan	4	115522201	115522500	0.639	1.189	0.259	0.745
Tan	4	115528201	115528500	2.047	1.161	2.131	2.272
Tan	4	115534501	115534800	0.450	0.794	0.325	0.477
Tan	4	115620601	115620900	1.626	0.940	2.287	1.683
Tan	4	115713601	115713900	0.398	0.745	0.424	0.187
Tan	4	115772101	115772400	0.586	2.036	0.979	0.866
Tan	4	115788601	115788900	0.055	1.891	0.285	0.137
Tan	4	115808401	115808700	1.808	0.609	1.861	1.892
Tan	4	115809301	115809600	2.679	3.574	2.410	1.617
Tan	4	116115601	116115900	0.687	1.281	0.662	0.370
Tan	4	116133901	116134200	0.268	0.607	0.325	0.106
Tan	4	116168701	116169000	2.356	4.776	6.442	3.337
Tan	4	116197501	116197800	1.195	1.405	1.888	1.663
Tan	4	116256901	116257200	0.423	1.105	0.497	0.355
Tan	4	116428801	116429100	0.344	1.101	0.564	0.619
Tan	4	116496301	116496600	2.713	3.501	2.751	2.555
Tan	4	116667901	116668200	1.202	1.946	1.028	1.024
Tan	4	116756701	116757000	1.681	2.128	1.547	1.100
Tan	4	116850001	116850300	3.184	3.905	2.555	4.533
Tan	4	116860501	116860800	3.301	2.154	2.756	3.022
Tan	4	117070501	117070800	0.400	0.607	0.317	0.355
Tan	4	117228001	117228300	2.179	2.572	1.822	2.211
Tan	4	117474001	117474300	0.928	0.420	0.872	0.867
Tan	4	117696901	117697200	5.070	3.939	5.957	5.298
Tan	4	117703801	117704100	7.658	6.379	7.595	8.073
Tan	4	117755401	117755700	3.459	2.879	2.575	3.215
Tan	4	117782101	117782400	1.113	0.416	0.901	0.974
Tan	4	118070701	118071000	0.215	0.491	0.082	0.086
Tan	4	118108501	118108800	4.228	4.606	3.496	3.245
Tan	4	118239001	118239300	3.404	5.092	2.542	3.610
Tan	4	118360201	118360500	0.663	0.140	0.609	0.391
Tan	4	118384201	118384500	1.620	0.390	1.444	1.801
Tan	4	119048101	119048400	0.561	0.890	0.078	0.507
Tan	4	119087401	119087700	0.294	0.538	0.087	0.304
Tan	4	119118301	119118600	0.108	0.446	0.082	0.142
Tan	4	119222101	119222400	1.595	2.995	0.947	1.689
Tan	4	119232901	119233200	0.294	1.103	0.551	0.598
Tan	5	137401	137700	0.398	0.845	0.312	0.284
Tan	5	277201	277500	0.398	0.800	0.263	0.248
Tan	5	576301	576600	0.294	1.410	0.502	0.380
Tan	5	602101	602400	2.942	4.266	2.954	2.845
Tan	5	620101	620400	0.184	0.562	0.234	0.173
Tan	5	623401	623700	0.131	0.517	0.078	0.147
Tan	5	1064101	1064400	0.720	1.176	0.263	0.695
Tan	5	1082701	1083000	1.328	2.079	1.164	1.759
Tan	5	1114801	1115100	1.915	1.452	1.041	1.212
Tan	5	1453201	1453500	1.968	0.985	0.600	2.261
Tan	5	1461001	1461300	0.110	0.631	0.234	0.025
Tan	5	1527001	1527300	0.424	0.165	0.506	0.441
Tan	5	1564801	1565100	0.584	0.281	0.724	0.857
Tan	5	1758301	1758600	2.021	1.289	2.040	2.297
Tan	5	1836601	1836900	0.397	1.081	0.210	0.527

Tan	5	1851001	1851300	0.184	0.661	0.049	0.081
Tan	5	1870201	1870500	0.982	1.285	0.564	0.598
Tan	5	2133001	2133300	0.376	0.867	0.321	0.345
Tan	5	2138401	2138700	1.436	0.463	1.295	0.958
Tan	5	2417401	2417700	2.528	1.725	2.916	2.996
Tan	5	2436001	2436300	1.144	1.540	0.967	1.050
Tan	5	2573101	2573400	0.505	0.961	0.535	0.608
Tan	5	2597701	2598000	0.160	0.395	0.103	0.056
Tan	5	2728501	2728800	0.263	0.846	0.140	0.187
Tan	5	2728801	2729100	0.213	0.775	0.227	0.167
Tan	5	2761201	2761500	4.147	2.688	3.065	3.615
Tan	5	2814001	2814300	0.874	1.379	0.633	0.513
Tan	5	2861701	2862000	1.494	0.378	1.705	1.825
Tan	5	3086701	3087000	0.347	1.176	0.453	0.492
Tan	5	3174601	3174900	0.584	1.171	0.453	0.821
Tan	5	3183901	3184200	0.505	0.843	0.448	0.355
Tan	5	3448801	3449100	6.193	1.947	6.123	7.558
Tan	5	3502201	3502500	6.066	3.007	7.350	7.004
Tan	5	3757201	3757500	0.368	1.210	0.502	0.274
Tan	5	3813901	3814200	0.134	0.446	0.078	0.218
Tan	5	3851101	3851400	1.331	2.263	1.551	1.374
Tan	5	3890401	3890700	0.294	0.491	0.185	0.086
Tan	5	4032901	4033200	1.200	2.441	0.889	1.238
Tan	5	4091701	4092000	0.931	0.584	0.794	0.918
Tan	5	4095601	4095900	0.879	2.682	0.493	0.760
Tan	5	4096801	4097100	1.565	2.942	1.028	1.578
Tan	5	4206001	4206300	0.350	1.300	0.223	0.517
Tan	5	4214701	4215000	3.141	4.319	2.040	3.002
Tan	5	4238401	4238700	3.352	2.830	3.710	3.564
Tan	5	4304701	4305000	0.347	0.980	0.497	0.573
Tan	5	4316701	4317000	2.075	2.667	1.576	2.069
Tan	5	4555201	4555500	1.492	1.798	1.173	1.192
Tan	5	4735201	4735500	2.181	1.614	2.127	0.816
Tan	5	4745101	4745400	1.063	2.294	1.255	0.917
Tan	5	4745401	4745700	2.341	4.529	2.399	1.820
Tan	5	4832701	4833000	1.884	2.948	1.126	1.790
Tan	5	4844401	4844700	0.186	0.680	0.103	0.126
Tan	5	4857001	4857300	0.240	0.680	0.267	0.329
Tan	5	4866901	4867200	0.557	0.818	0.506	0.547
Tan	5	4902601	4902900	0.158	0.513	0.210	0.248
Tan	5	4922101	4922400	0.344	0.657	0.210	0.198
Tan	5	4952701	4953000	1.996	0.863	1.518	1.512
Tan	5	4954501	4954800	0.852	0.440	0.769	0.882
Tan	5	5118301	5118600	0.452	0.867	0.506	0.634
Tan	5	5271001	5271300	10.581	9.751	10.790	10.820
Tan	5	5386801	5387100	0.692	1.026	1.271	0.578
Tan	5	5391601	5391900	0.294	0.446	0.107	0.167
Tan	5	5474101	5474400	0.321	0.584	0.239	0.268
Tan	5	5804101	5804400	0.158	0.373	0.214	0.522
Tan	5	5855101	5855400	0.134	0.637	0.181	0.218
Tan	5	5884201	5884500	3.773	1.693	2.559	3.042
Tan	5	5952901	5953200	2.875	3.604	2.604	2.596
Tan	5	5969401	5969700	7.206	5.520	7.006	6.973
Tan	5	5969701	5970000	3.512	2.004	3.176	3.346
Tan	5	6322201	6322500	5.245	2.166	4.687	5.953
Tan	5	6468301	6468600	0.000	0.494	0.078	0.162
Tan	5	6514501	6514800	1.434	2.017	1.373	1.471
Tan	5	6514801	6515100	1.946	2.873	1.957	2.225
Tan	5	6555301	6555600	1.170	1.639	1.431	1.298
Tan	5	6592201	6592500	0.055	0.566	0.078	0.193
Tan	5	6646201	6646500	0.213	0.558	0.205	0.162
Tan	5	6820801	6821100	0.723	1.571	0.930	1.019
Tan	5	7147501	7147800	4.254	1.858	3.059	3.058
Tan	5	7164001	7164300	0.082	0.708	0.189	0.137
Tan	5	7605001	7605300	0.186	0.635	0.152	0.198
Tan	5	7818001	7818300	0.129	0.584	0.185	0.198
Tan	5	9180301	9180600	2.631	3.961	2.526	2.469

Tan	5	9304201	9304500	0.292	0.699	0.354	0.223
Tan	5	9333001	9333300	0.821	1.526	0.575	0.492
Tan	5	9402601	9402900	0.186	0.657	0.156	0.187
Tan	5	9407701	9408000	0.426	0.657	0.424	0.223
Tan	5	9493801	9494100	1.595	2.454	2.044	2.327
Tan	5	9569401	9569700	0.186	1.006	0.633	0.553
Tan	5	9960301	9960600	0.318	0.583	0.317	0.106
Tan	5	9969301	9969600	0.852	0.094	0.769	0.649
Tan	5	10011301	10011600	0.268	0.680	0.283	0.274
Tan	5	10083601	10083900	0.292	1.152	0.214	0.461
Tan	5	10110601	10110900	1.879	0.566	1.940	2.266
Tan	5	10110901	10111200	1.518	0.556	1.833	1.627
Tan	5	10125001	10125300	1.353	0.348	1.295	0.817
Tan	5	10164901	10165200	0.721	0.983	0.453	0.583
Tan	5	10231801	10232100	0.106	0.510	0.054	0.106
Tan	5	10413301	10413600	1.752	2.502	1.830	2.033
Tan	5	10431001	10431300	5.106	4.351	5.118	6.094
Tan	5	10461601	10461900	0.397	0.916	0.468	0.411
Tan	5	10584301	10584600	2.154	1.012	2.209	2.150
Tan	5	10600801	10601100	3.430	2.780	3.241	3.275
Tan	5	10664101	10664400	1.626	1.030	1.645	1.750
Tan	5	10671301	10671600	0.269	0.794	0.346	0.340
Tan	5	10733401	10733700	0.318	0.751	0.341	0.380
Tan	5	10768501	10768800	0.376	0.938	0.379	0.502
Tan	5	10831801	10832100	0.984	1.659	1.081	1.212
Tan	5	10897801	10898100	0.186	0.303	0.428	0.527
Tan	5	10924501	10924800	2.812	3.858	1.628	2.171
Tan	5	11004601	11004900	0.615	1.171	0.453	0.512
Tan	5	11039401	11039700	1.037	1.519	1.148	0.781
Tan	5	11726401	11726700	0.319	0.444	0.185	0.167
Tan	5	11945701	11946000	2.712	1.892	2.800	2.910
Tan	5	12225601	12225900	0.744	0.163	0.593	0.664
Tan	5	12256501	12256800	0.237	0.539	0.132	0.051
Tan	5	12396301	12396600	2.150	3.092	1.764	1.922
Tan	5	12643801	12644100	0.160	0.420	0.214	0.223
Tan	5	12655201	12655500	0.186	0.493	0.103	0.299
Tan	5	12659701	12660000	4.046	3.527	2.867	3.088
Tan	5	12688501	12688800	0.294	0.631	0.156	0.360
Tan	5	12885001	12885300	0.081	0.491	0.082	0.051
Tan	5	13079401	13079700	0.610	0.983	0.588	0.461
Tan	5	13103701	13104000	0.398	0.957	0.424	0.614
Tan	5	13113901	13114200	0.294	0.792	0.296	0.279
Tan	5	13211701	13212000	0.532	1.257	0.593	0.725
Tan	5	13250101	13250400	2.207	3.182	2.178	1.982
Tan	5	13273801	13274100	0.452	1.004	0.774	0.801
Tan	5	13408201	13408500	0.718	1.386	0.575	0.731
Tan	5	13424401	13424700	0.928	1.264	0.609	0.664
Tan	5	13429801	13430100	0.826	1.143	0.609	0.801
Tan	5	13751401	13751700	0.398	0.871	0.165	0.299
Tan	5	13778401	13778700	0.319	0.773	0.205	0.162
Tan	5	13996801	13997100	0.319	0.723	0.259	0.167
Tan	5	14012401	14012700	6.146	3.866	6.518	5.715
Tan	5	14028301	14028600	0.503	1.099	0.617	0.456
Tan	5	14091901	14092200	1.515	2.703	1.572	2.023
Tan	5	14093701	14094000	1.942	2.589	1.810	1.861
Tan	5	14148601	14148900	0.398	0.867	0.448	0.547
Tan	5	14179801	14180100	0.586	1.032	0.593	0.553
Tan	5	14383801	14384100	0.423	0.980	0.667	0.329
Tan	5	14632501	14632800	2.367	1.515	2.200	2.114
Tan	5	14783101	14783400	0.319	0.489	0.239	0.360
Tan	5	14811901	14812200	0.211	0.470	0.156	0.112
Tan	5	15192901	15193200	0.079	0.279	0.827	0.056
Tan	5	15342901	15343200	0.269	1.109	0.185	0.304
Tan	5	15343201	15343500	0.649	2.735	0.435	0.751
Tan	5	15424801	15425100	0.158	0.468	0.214	0.218
Tan	5	15502201	15502500	0.053	0.657	0.132	0.051
Tan	5	15502501	15502800	0.081	0.684	0.136	0.106

Tan	5	15591901	15592200	0.424	1.195	0.419	0.421
Tan	5	15598801	15599100	0.244	1.004	0.267	0.421
Tan	5	15660301	15660600	1.594	1.871	0.934	1.389
Tan	5	15794401	15794700	0.584	0.185	0.502	0.720
Tan	5	15865801	15866100	1.991	0.713	1.601	2.079
Tan	5	15897901	15898200	0.266	0.420	0.132	0.218
Tan	5	15919501	15919800	0.934	1.783	0.934	0.892
Tan	5	15948901	15949200	2.114	3.368	1.605	2.022
Tan	5	15957001	15957300	0.610	0.350	0.716	0.659
Tan	5	15985501	15985800	3.639	3.042	2.604	4.031
Tan	5	16322401	16322700	0.292	0.680	0.218	0.411
Tan	5	16480201	16480500	0.265	0.562	0.296	0.198
Tan	5	16571701	16572000	0.292	0.541	0.292	0.193
Tan	5	16783201	16783500	2.370	1.066	2.463	1.800
Tan	5	17165701	17166000	0.345	0.466	0.054	0.218
Tan	5	17255101	17255400	0.105	0.538	0.111	0.137
Tan	5	17341801	17342100	0.349	0.910	0.457	0.502
Tan	5	17539201	17539500	2.044	1.729	2.448	2.043
Tan	5	17840101	17840400	0.240	0.680	0.374	0.304
Tan	5	17846701	17847000	0.345	0.749	0.292	0.461
Tan	5	17880901	17881200	0.397	0.745	0.424	0.162
Tan	5	17884501	17884800	0.055	0.420	0.107	0.056
Tan	5	17956201	17956500	0.584	1.386	0.437	0.639
Tan	5	18478801	18479100	1.891	1.279	2.064	2.484
Tan	5	18515101	18515400	1.942	1.427	1.826	2.007
Tan	5	18558601	18558900	0.106	0.493	0.160	0.218
Tan	5	18670201	18670500	0.215	0.564	0.205	0.106
Tan	5	18687001	18687300	2.073	3.077	2.020	1.905
Tan	5	18687301	18687600	1.729	2.313	1.929	1.912
Tan	5	18856501	18856800	0.242	0.536	0.078	0.162
Tan	5	18974101	18974400	0.529	1.028	0.403	0.466
Tan	5	18983701	18984000	3.430	2.809	3.545	3.458
Tan	5	19304101	19304400	0.161	0.515	0.160	0.248
Tan	5	19541401	19541700	0.292	0.699	0.419	0.355
Tan	5	19557001	19557300	1.010	2.107	1.030	0.989
Tan	5	19805701	19806000	0.344	0.841	0.317	0.401
Tan	5	20045701	20046000	1.726	0.773	1.280	1.384
Tan	5	20052001	20052300	1.857	2.523	1.378	1.724
Tan	5	20328301	20328600	0.428	0.775	0.395	0.335
Tan	5	21384901	21385200	0.799	1.480	0.802	0.968
Tan	5	21385201	21385500	0.879	1.382	0.774	0.826
Tan	5	21740701	21741000	0.424	0.656	0.132	0.299
Tan	5	21920701	21921000	0.373	0.586	0.370	0.385
Tan	5	22355701	22356000	0.132	0.513	0.098	0.167
Tan	5	22959001	22959300	0.318	0.961	0.058	0.385
Tan	5	23187001	23187300	0.294	0.420	0.267	0.167
Tan	5	23473501	23473800	0.186	0.541	0.243	0.106
Tan	5	23473801	23474100	0.134	0.777	0.234	0.106
Tan	5	23500201	23500500	0.213	0.420	0.132	0.254
Tan	5	23944501	23944800	0.134	0.564	0.296	0.142
Tan	5	24295201	24295500	0.345	0.094	0.555	0.279
Tan	5	24295501	24295800	0.426	0.094	0.662	0.324
Tan	5	24315601	24315900	0.878	0.330	0.809	0.918
Tan	5	24350101	24350400	0.611	1.096	0.749	0.725
Tan	5	24459001	24459300	0.345	0.656	0.477	0.223
Tan	5	24615901	24616200	0.398	0.680	0.292	0.284
Tan	5	24647401	24647700	0.156	0.588	0.160	0.218
Tan	5	24792001	24792300	0.106	0.350	0.160	0.553
Tan	5	24891601	24891900	0.108	0.680	0.346	0.274
Tan	5	25581301	25581600	1.300	0.684	1.316	1.440
Tan	5	25706401	25706700	0.345	0.751	0.366	0.248
Tan	5	26397601	26397900	0.158	0.583	0.181	0.122
Tan	5	26525401	26525700	8.376	6.108	7.947	8.705
Tan	5	26820001	26820300	0.453	0.114	0.486	0.446
Tan	5	26936401	26936700	0.186	0.835	0.165	0.259
Tan	5	27052801	27053100	1.597	1.049	1.514	0.513
Tan	5	27230401	27230700	0.134	0.470	0.165	0.117

Tan	5	27453001	27453300	1.726	2.386	1.587	0.903
Tan	5	27572701	27573000	0.082	0.470	0.127	0.025
Tan	5	27975601	27975900	0.208	0.629	0.132	0.106
Tan	5	28053601	28053900	0.345	0.704	0.477	0.162
Tan	5	28251301	28251600	0.797	2.051	0.555	0.948
Tan	5	28573801	28574100	0.081	0.425	0.078	0.025
Tan	5	28621201	28621500	0.295	0.538	0.239	0.056
Tan	5	28640101	28640400	2.822	2.004	2.867	2.966
Tan	5	29035201	29035500	0.558	1.116	0.457	0.542
Tan	5	29168701	29169000	0.799	1.287	0.827	0.898
Tan	5	29169001	29169300	0.636	1.077	0.638	0.512
Tan	5	29372101	29372400	0.958	1.309	0.950	1.050
Tan	5	29781901	29782200	0.319	0.725	0.395	0.248
Tan	5	29889901	29890200	0.161	0.538	0.136	0.031
Tan	5	30020701	30021000	0.213	0.446	0.136	0.142
Tan	5	30365401	30365700	1.487	0.678	1.070	1.136
Tan	5	30919201	30919500	0.292	0.584	0.346	0.355
Tan	5	32024101	32024400	0.690	0.185	0.642	0.558
Tan	5	33228901	33229200	0.134	0.541	0.160	0.076
Tan	5	33272401	33272700	0.239	0.468	0.317	0.279
Tan	5	33698401	33698700	0.082	0.466	0.127	0.025
Tan	5	33870001	33870300	0.158	0.517	0.234	0.167
Tan	5	33870301	33870600	0.347	0.611	0.263	0.360
Tan	5	34122601	34122900	0.106	0.513	0.210	0.106
Tan	5	34293301	34293600	0.347	0.586	0.078	0.304
Tan	5	34591201	34591500	0.134	0.350	0.687	0.193
Tan	5	35035801	35036100	0.134	1.493	0.189	0.147
Tan	5	35039401	35039700	0.426	0.633	0.317	0.355
Tan	5	35058901	35059200	0.186	1.167	0.296	0.588
Tan	5	35059201	35059500	0.105	1.448	0.363	0.832
Tan	5	35317501	35317800	0.503	0.890	1.119	0.720
Tan	5	35416501	35416800	0.881	1.901	1.016	0.725
Tan	5	36167101	36167400	0.661	1.363	0.341	0.573
Tan	5	36231601	36231900	0.426	0.916	0.292	0.547
Tan	5	36405001	36405300	0.805	1.976	0.629	0.745
Tan	5	36479101	36479400	0.265	0.140	0.292	0.497
Tan	5	36616201	36616500	0.692	1.586	0.510	0.786
Tan	5	36636001	36636300	1.649	2.289	0.760	1.667
Tan	5	37297201	37297500	0.211	0.468	0.156	0.243
Tan	5	37413301	37413600	0.027	0.657	0.189	0.137
Tan	5	37461601	37461900	0.186	0.706	0.165	0.147
Tan	5	37766101	37766400	0.292	0.444	0.210	0.198
Tan	5	37892101	37892400	0.026	0.465	0.025	0.031
Tan	5	37992601	37992900	0.239	0.680	0.296	0.411
Tan	5	38142601	38142900	1.121	0.418	0.901	1.212
Tan	5	39647101	39647400	0.316	0.656	0.370	0.360
Tan	5	40397101	40397400	0.134	0.517	0.239	0.167
Tan	5	40727401	40727700	1.355	1.191	0.798	0.695
Tan	5	40867801	40868100	0.345	0.822	0.390	0.360
Tan	5	40998001	40998300	0.240	0.564	0.107	0.193
Tan	5	41120101	41120400	0.186	0.562	0.247	0.137
Tan	5	41204101	41204400	0.799	1.360	0.823	0.674
Tan	5	41236501	41236800	0.984	0.843	0.667	1.359
Tan	5	41314501	41314800	0.634	1.594	0.700	0.999
Tan	5	41381401	41381700	0.318	0.869	0.267	0.441
Tan	5	41384401	41384700	0.077	0.607	0.370	0.056
Tan	5	41476201	41476500	0.716	1.480	0.731	0.634
Tan	5	41694601	41694900	0.321	0.775	0.428	0.142
Tan	5	42076501	42076800	1.279	1.849	0.642	0.720
Tan	5	42190201	42190500	0.265	0.674	0.165	0.335
Tan	5	42231601	42231900	0.398	0.744	0.346	0.162
Tan	5	42288301	42288600	0.134	0.401	0.160	0.112
Tan	5	42496201	42496500	0.184	0.860	0.477	0.304
Tan	5	42601501	42601800	0.450	1.096	0.477	0.533
Tan	5	42601801	42602100	0.503	1.122	0.506	0.426
Tan	5	42641701	42642000	0.026	0.420	0.078	0.167
Tan	5	42803401	42803700	0.455	1.098	0.370	0.735

Tan	5	42912901	42913200	0.213	0.444	0.111	0.162
Tan	5	42960901	42961200	0.510	1.148	0.399	0.436
Tan	5	43124401	43124700	0.534	0.375	0.160	0.081
Tan	5	43272001	43272300	0.773	1.146	0.662	0.679
Tan	5	43348501	43348800	1.460	1.993	1.415	1.267
Tan	5	43429201	43429500	0.316	1.030	0.321	0.502
Tan	5	43470901	43471200	0.292	0.588	0.234	0.274
Tan	5	43759201	43759500	0.161	1.120	0.510	0.126
Tan	5	43884001	43884300	0.132	0.446	0.127	0.198
Tan	5	43991401	43991700	0.774	1.148	0.535	0.603
Tan	5	44000701	44001000	0.398	0.539	0.185	0.274
Tan	5	44229901	44230200	0.079	0.466	0.165	0.187
Tan	5	44349001	44349300	0.131	0.511	0.214	0.081
Tan	5	44406601	44406900	0.534	0.914	0.403	0.335
Tan	5	44436301	44436600	0.374	0.729	0.424	0.248
Tan	5	44494201	44494500	0.211	0.562	0.181	0.167
Tan	5	44750701	44751000	0.210	0.747	0.317	0.284
Tan	5	44782801	44783100	0.452	1.217	0.515	0.771
Tan	5	45023701	45024000	1.836	2.635	1.034	1.734
Tan	5	45249301	45249600	0.531	0.303	0.687	0.502
Tan	5	45456901	45457200	0.874	1.262	0.667	0.745
Tan	5	45476701	45477000	0.184	0.513	0.074	0.193
Tan	5	45552901	45553200	3.994	2.545	3.048	3.438
Tan	5	45838201	45838500	2.499	1.354	1.991	2.362
Tan	5	46447501	46447800	1.357	1.590	1.193	1.267
Tan	5	46955101	46955400	0.368	0.987	0.234	0.380
Tan	5	46989901	46990200	0.134	0.423	0.074	0.167
Tan	5	47192701	47193000	0.639	0.957	0.531	0.664
Tan	5	47193901	47194200	0.187	0.541	0.049	0.142
Tan	5	47212501	47212800	0.479	0.283	0.827	0.547
Tan	5	47462101	47462400	0.855	1.712	0.823	1.267
Tan	5	47462401	47462700	0.802	1.944	0.814	1.314
Tan	5	47671801	47672100	0.213	0.468	0.185	0.025
Tan	5	47923801	47924100	0.344	0.631	0.160	0.335
Tan	5	48284701	48285000	0.213	0.373	0.642	0.167
Tan	5	48472801	48473100	0.160	0.444	0.107	0.254
Tan	5	48934201	48934500	0.239	0.725	0.214	0.466
Tan	5	49657801	49658100	0.079	0.470	0.132	0.112
Tan	5	49906201	49906500	7.372	4.332	6.960	8.468
Tan	5	50386501	50386800	0.187	0.444	0.239	0.198
Tan	5	50896501	50896800	0.132	0.442	0.132	0.223
Tan	5	51216601	51216900	0.263	0.981	0.272	0.558
Tan	5	51234901	51235200	0.266	0.657	0.152	0.360
Tan	5	51951301	51951600	0.477	0.727	0.374	0.411
Tan	5	51973801	51974100	0.239	0.444	0.214	0.254
Tan	5	52357801	52358100	0.263	0.584	0.189	0.218
Tan	5	52446001	52446300	0.371	0.631	0.477	0.193
Tan	5	53209501	53209800	0.560	1.054	0.444	0.619
Tan	5	53419501	53419800	0.266	0.562	0.428	0.695
Tan	5	53450701	53451000	0.398	0.843	0.564	0.248
Tan	5	53672401	53672700	1.100	2.057	0.781	1.055
Tan	5	53815501	53815800	0.132	0.423	0.160	0.193
Tan	5	53910001	53910300	0.318	0.635	0.346	0.335
Tan	5	54017101	54017400	0.026	0.564	0.082	0.254
Tan	5	54069301	54069600	0.160	0.612	0.107	0.248
Tan	5	54435301	54435600	0.321	0.678	0.205	0.223
Tan	5	54462901	54463200	0.773	1.703	1.012	0.821
Tan	5	54644401	54644700	0.081	0.401	0.058	0.056
Tan	5	54715501	54715800	1.350	0.635	1.543	1.334
Tan	5	54852901	54853200	0.265	0.470	0.025	0.254
Tan	5	55331401	55331700	1.992	2.615	3.328	1.906
Tan	5	55363801	55364100	0.239	0.493	0.288	0.193
Tan	5	55385401	55385700	0.479	0.862	0.473	0.355
Tan	5	55395301	55395600	0.400	0.584	0.049	0.142
Tan	5	55463101	55463400	0.424	0.751	0.346	0.527
Tan	5	55565101	55565400	0.263	0.822	0.107	0.335
Tan	5	55986601	55986900	0.794	1.493	0.753	0.801

Tan	5	56068801	56069100	0.000	0.541	0.189	0.173
Tan	5	56117701	56118000	0.479	0.633	0.905	0.472
Tan	5	56182501	56182800	0.376	0.891	0.214	0.294
Tan	5	56454001	56454300	0.424	1.126	0.575	0.695
Tan	5	57461101	57461400	0.689	1.169	0.789	0.238
Tan	5	57647101	57647400	0.160	0.375	0.477	0.441
Tan	5	58290301	58290600	0.345	0.676	0.457	0.274
Tan	5	58530601	58530900	0.347	0.676	0.296	0.421
Tan	5	58789801	58790100	0.081	0.489	0.185	0.137
Tan	5	58911601	58911900	1.415	2.714	1.612	1.374
Tan	5	58929901	58930200	0.132	0.517	0.132	0.198
Tan	5	58984501	58984800	0.479	0.796	0.239	0.218
Tan	5	59067301	59067600	0.477	0.185	0.374	0.416
Tan	5	59087701	59088000	0.347	0.751	0.210	0.223
Tan	5	59109001	59109300	0.902	1.596	0.700	0.837
Tan	5	59305801	59306100	0.344	0.656	0.296	0.309
Tan	5	59435101	59435400	1.862	1.569	1.090	1.825
Tan	5	59459401	59459700	0.615	1.504	0.678	0.649
Tan	5	59538901	59539200	0.136	0.652	0.189	0.218
Tan	5	59948101	59948400	1.115	1.706	0.794	1.314
Tan	5	59973301	59973600	0.319	0.652	0.263	0.294
Tan	5	59978701	59979000	0.294	0.468	0.263	0.193
Tan	5	60011701	60012000	0.314	0.936	0.399	0.492
Tan	5	60183601	60183900	1.087	2.420	1.093	1.318
Tan	5	60231901	60232200	4.012	2.517	5.219	4.792
Tan	5	60344401	60344700	0.237	0.515	0.267	0.254
Tan	5	60395401	60395700	0.208	1.099	0.111	0.304
Tan	5	61233001	61233300	0.187	0.867	0.424	0.421
Tan	5	61346401	61346700	0.239	0.564	0.181	0.193
Tan	5	61656001	61656300	0.161	0.674	0.156	0.268
Tan	5	62031901	62032200	0.531	0.770	0.403	0.446
Tan	5	62318701	62319000	0.237	0.680	0.127	0.162
Tan	5	62938501	62938800	0.158	0.470	0.156	0.223
Tan	5	62943301	62943600	0.132	0.440	0.054	0.061
Tan	5	63168301	63168600	0.821	1.259	0.428	0.639
Tan	5	63502501	63502800	0.292	0.515	0.341	0.193
Tan	5	63529801	63530100	0.397	0.772	0.453	0.472
Tan	5	63608701	63609000	0.186	0.612	0.156	0.198
Tan	5	63614401	63614700	0.268	0.702	0.346	0.436
Tan	5	63751801	63752100	0.156	0.468	0.049	0.142
Tan	5	64356601	64356900	0.536	1.270	0.448	0.391
Tan	5	64455001	64455300	0.665	0.097	1.028	0.730
Tan	5	64478101	64478400	1.713	2.918	1.375	0.897
Tan	5	64610701	64611000	0.292	0.869	0.428	0.329
Tan	5	64622401	64622700	0.532	0.745	0.481	0.527
Tan	5	65060401	65060700	0.187	0.517	0.054	0.061
Tan	5	65318401	65318700	0.266	0.629	0.288	0.142
Tan	5	65461201	65461500	0.213	0.373	0.181	0.553
Tan	5	65684401	65684700	0.371	0.884	0.185	0.178
Tan	5	65873401	65873700	0.242	0.609	0.210	0.304
Tan	5	65965501	65965800	0.402	0.773	0.341	0.391
Tan	5	66151801	66152100	0.587	0.818	0.288	0.411
Tan	5	66191401	66191700	0.132	0.442	0.078	0.193
Tan	5	66271201	66271500	0.474	1.691	0.720	0.532
Tan	5	66277201	66277500	0.400	1.004	0.444	0.538
Tan	5	66344701	66345000	1.223	2.414	0.838	1.378
Tan	5	66374401	66374700	1.279	2.113	1.106	1.125
Tan	5	66496201	66496500	2.124	1.541	2.394	2.403
Tan	5	66736801	66737100	1.116	1.403	0.954	1.019
Tan	5	67154701	67155000	0.347	0.628	0.346	0.254
Tan	5	67188301	67188600	0.265	0.491	0.156	0.106
Tan	5	67471801	67472100	0.186	0.611	0.165	0.254
Tan	5	68006401	68006700	0.505	1.332	0.749	0.918
Tan	5	68989501	68989800	1.654	2.938	1.732	1.755
Tan	5	69204601	69204900	0.613	0.841	0.580	0.578
Tan	5	69261301	69261600	0.266	0.541	0.239	0.137
Tan	5	69400201	69400500	0.962	3.041	0.631	0.553

Tan	5	69400501	69400800	0.400	1.262	0.103	0.218
Tan	5	69427801	69428100	0.319	1.199	0.403	0.288
Tan	5	70332301	70332600	0.666	0.234	0.584	0.715
Tan	5	70579501	70579800	1.994	1.334	1.864	1.926
Tan	5	71196301	71196600	0.184	0.468	0.185	0.056
Tan	5	71261101	71261400	0.265	0.564	0.239	0.142
Tan	5	71770801	71771100	0.319	0.818	0.292	0.436
Tan	5	72394201	72394500	1.039	0.631	1.197	0.948
Tan	5	72394501	72394800	1.228	0.605	1.810	1.314
Tan	5	72495901	72496200	0.428	0.980	0.230	0.355
Tan	5	72685801	72686100	0.134	0.444	0.087	0.112
Tan	5	72771601	72771900	0.292	0.749	0.346	0.497
Tan	5	72780601	72780900	0.156	0.538	0.103	0.025
Tan	5	73389901	73390200	0.215	0.682	0.087	0.086
Tan	5	73848301	73848600	0.081	0.465	0.156	0.086
Tan	5	73993801	73994100	0.106	0.676	0.531	0.385
Tan	5	73994101	73994400	0.081	0.609	0.453	0.299
Tan	5	74926201	74926500	0.079	0.423	0.132	0.117
Tan	5	75922201	75922500	0.237	0.491	0.234	0.218
Tan	5	75936901	75937200	0.158	0.609	0.395	0.137
Tan	5	76213501	76213800	0.134	0.444	0.029	0.076
Tan	5	76461601	76461900	0.186	0.487	0.136	0.086
Tan	5	76592701	76593000	0.345	1.009	0.638	0.603
Tan	5	77076901	77077200	0.266	0.562	0.312	0.274
Tan	5	77146801	77147100	0.213	0.539	0.210	0.162
Tan	5	77273401	77273700	0.345	0.116	0.580	0.578
Tan	5	77343001	77343300	1.382	1.663	1.041	1.998
Tan	5	77394901	77395200	0.161	0.757	0.049	0.132
Tan	5	77408101	77408400	0.158	0.515	0.156	0.086
Tan	5	77408401	77408700	0.156	0.637	0.111	0.112
Tan	5	77536501	77536800	0.294	0.656	0.160	0.167
Tan	5	77573401	77573700	0.874	1.431	0.930	0.715
Tan	5	77658001	77658300	0.373	0.678	0.346	0.259
Tan	5	77789101	77789400	0.424	0.751	0.370	0.248
Tan	5	77803501	77803800	0.294	0.538	0.288	0.167
Tan	5	78114001	78114300	0.160	0.637	0.136	0.101
Tan	5	78438601	78438900	0.850	1.195	0.633	0.710
Tan	5	78438901	78439200	2.092	3.403	1.933	2.043
Tan	5	78759601	78759900	1.597	2.322	1.362	1.176
Tan	5	79044301	79044600	0.374	0.817	0.243	0.248
Tan	5	80446501	80446800	0.452	1.030	0.366	0.486
Tan	5	81860701	81861000	0.055	0.470	0.160	0.056
Tan	5	82474801	82475100	0.318	0.468	0.160	0.329
Tan	5	82760701	82761000	0.613	0.959	0.658	0.603
Tan	5	83064001	83064300	0.850	2.317	1.605	0.781
Tan	5	83568301	83568600	0.850	1.793	0.987	0.959
Tan	5	84416701	84417000	0.266	0.444	0.288	0.583
Tan	5	85665601	85665900	0.268	0.865	0.317	0.167
Tan	5	85738501	85738800	0.374	0.633	0.296	0.304
Tan	5	85830601	85830900	0.158	0.420	0.185	0.056
Tan	5	86223901	86224200	0.244	0.676	0.082	0.137
Tan	5	86285401	86285700	0.369	0.839	0.403	0.416
Tan	5	86437801	86438100	5.449	1.644	5.525	5.915
Tan	5	86686201	86686500	0.052	0.491	0.160	0.218
Tan	5	86769901	86770200	0.265	0.491	0.234	0.025
Tan	5	86859301	86859600	0.423	1.013	0.453	0.360
Tan	5	86877001	86877300	0.795	1.525	0.872	0.796
Tan	5	87122101	87122400	2.199	0.908	2.138	2.119
Tan	5	87203701	87204000	0.477	1.214	0.691	0.725
Tan	5	87302401	87302700	1.039	0.491	1.008	0.846
Tan	5	87347401	87347700	0.184	0.659	0.267	0.274
Tan	5	87350101	87350400	0.027	0.468	0.181	0.086
Tan	5	87384601	87384900	0.452	0.283	0.424	0.664
Tan	5	87391201	87391500	0.158	0.661	0.185	0.061
Tan	5	87483901	87484200	1.886	2.570	1.480	1.698
Tan	5	87554401	87554700	1.315	3.302	0.722	1.329
Tan	5	87672601	87672900	0.186	0.845	0.613	0.299

Tan	5	87757201	87757500	0.131	0.493	0.160	0.076
Tan	5	88120201	88120500	1.444	2.868	0.847	1.545
Tan	5	88762201	88762500	0.052	0.401	0.132	0.000
Tan	5	89332201	89332500	0.292	0.420	0.156	0.193
Tan	5	89939401	89939700	0.186	0.444	0.210	0.142
Tan	5	90360301	90360600	0.103	1.045	0.049	0.000
Tan	5	90404701	90405000	0.210	0.584	0.205	0.187
Tan	5	90493201	90493500	0.374	0.843	0.403	0.608
Tan	5	90555601	90555900	0.392	3.113	0.866	0.992
Tan	5	90774601	90774900	0.321	0.560	0.189	0.137
Tan	5	90826201	90826500	0.240	0.753	0.160	0.173
Tan	5	90882301	90882600	0.103	0.564	0.132	0.173
Tan	5	91127101	91127400	0.213	0.701	0.374	0.472
Tan	5	91177501	91177800	1.728	0.260	1.425	1.775
Tan	5	91351801	91352100	0.213	0.820	0.218	0.288
Tan	5	91628101	91628400	0.531	0.981	0.609	0.578
Tan	5	91934401	91934700	0.239	0.493	0.234	0.223
Tan	5	92015101	92015400	0.132	0.584	0.370	0.279
Tan	5	92511601	92511900	0.158	0.444	0.132	0.193
Tan	5	92520601	92520900	0.237	0.802	0.210	0.304
Tan	5	92523901	92524200	0.134	0.442	0.029	0.187
Tan	5	92614501	92614800	0.081	0.558	0.321	0.106
Tan	5	92619301	92619600	0.371	1.526	0.671	0.685
Tan	5	92781301	92781600	0.400	0.680	0.239	0.213
Tan	5	93207301	93207600	0.052	0.444	0.156	0.162
Tan	5	93286501	93286800	0.081	0.727	0.239	1.197
Tan	5	93471001	93471300	0.610	1.126	0.522	0.598
Tan	5	93487801	93488100	0.266	0.841	0.156	0.319
Tan	5	93616801	93617100	0.053	0.440	0.107	0.000
Tan	5	93755701	93756000	0.292	0.562	0.292	0.086
Tan	5	93789901	93790200	0.263	0.678	0.259	0.279
Tan	5	93861901	93862200	0.026	0.566	0.239	0.137
Tan	5	94315501	94315800	0.263	0.656	0.210	0.173
Tan	5	94320601	94320900	0.158	0.493	0.078	0.187
Tan	5	94428601	94428900	0.453	0.165	0.531	0.472
Tan	5	94433101	94433400	0.398	1.099	0.468	0.472
Tan	5	94627501	94627800	0.213	0.420	0.160	0.167
Tan	5	94693201	94693500	0.186	0.305	0.506	0.137
Tan	5	94948201	94948500	0.158	0.470	0.049	0.137
Tan	5	95079601	95079900	0.211	0.656	0.136	0.173
Tan	5	95140201	95140500	0.584	0.330	0.609	0.608
Tan	5	95180701	95181000	0.371	0.611	0.321	0.223
Tan	5	95304301	95304600	1.437	2.010	1.157	1.471
Tan	5	95334601	95334900	1.382	2.176	1.556	1.440
Tan	5	95586901	95587200	4.251	3.223	2.562	4.599
Tan	5	95601301	95601600	1.163	0.466	1.402	1.186
Tan	5	96010501	96010800	1.758	3.180	1.431	2.404
Tan	5	96192901	96193200	0.507	0.749	0.535	0.497
Tan	5	96980401	96980700	0.026	0.609	0.194	0.218
Tan	5	97194301	97194600	0.481	0.234	0.477	0.492
Tan	5	97395601	97395900	0.077	0.395	0.000	0.112
Tan	5	97770001	97770300	0.187	0.609	0.107	0.304
Tan	5	98075101	98075400	0.213	0.468	0.205	0.025
Tan	5	98206801	98207100	0.215	1.025	0.535	0.411
Tan	5	98404201	98404500	0.532	0.212	0.453	0.583
Tan	5	98886301	98886600	0.345	0.583	0.296	0.335
Tan	5	98979901	98980200	1.904	0.023	3.141	2.881
Tan	5	98990101	98990400	0.558	0.281	0.395	0.081
Tan	5	99000001	99000300	0.424	0.890	0.510	0.391
Tan	5	99072601	99072900	2.502	0.839	1.891	1.897
Tan	5	99534601	99534900	0.373	0.961	0.662	0.745
Tan	5	99668701	99669000	0.768	1.832	0.408	0.853
Tan	5	99702901	99703200	0.560	0.232	0.078	0.081
Tan	5	99709201	99709500	0.106	0.468	0.078	0.106
Tan	5	99715501	99715800	0.134	0.491	0.243	0.162
Tan	5	99796501	99796800	0.476	1.099	0.551	0.547
Tan	5	100500901	100501200	0.081	0.583	0.374	0.198

Tan	5	100524601	100524900	0.211	0.466	0.156	0.137
Tan	5	100744801	100745100	0.106	0.916	0.247	0.248
Tan	5	100896301	100896600	0.266	0.635	0.317	0.223
Tan	5	101060401	101060700	0.189	0.562	0.058	0.218
Tan	5	101343601	101343900	0.239	1.101	0.488	0.497
Tan	5	102189901	102190200	0.186	0.751	0.301	0.329
Tan	5	102245701	102246000	0.134	0.820	0.432	0.274
Tan	5	102328201	102328500	0.797	0.583	0.341	0.355
Tan	5	103035001	103035300	0.106	0.536	0.218	0.279
Tan	5	103040401	103040700	0.584	1.054	0.604	0.619
Tan	5	103065001	103065300	0.453	0.865	0.403	0.573
Tan	5	103130101	103130400	0.902	1.236	0.531	0.806
Tan	5	103674301	103674600	1.437	2.373	1.558	1.465
Tan	5	103692901	103693200	0.398	0.654	0.185	0.365
Tan	5	103714201	103714500	1.111	1.925	1.094	0.933
Tan	5	103787401	103787700	1.091	1.380	0.584	0.857
Tan	5	103811701	103812000	1.781	2.341	1.563	1.562
Tan	5	103894201	103894500	1.204	1.956	1.295	2.707
Tan	5	103980601	103980900	0.850	0.491	0.642	0.664
Tan	5	104474101	104474400	0.347	0.468	0.160	0.360
Tan	5	104488801	104489100	0.319	0.815	0.292	0.284
Tan	5	104489101	104489400	0.953	1.476	0.831	0.877
Tan	5	104513401	104513700	0.184	0.860	0.054	0.319
Tan	5	104566501	104566800	2.078	3.416	2.608	3.006
Tan	5	104639101	104639400	0.323	0.729	0.239	0.218
Tan	5	104874301	104874600	4.881	6.604	4.427	4.086
Tan	5	104989501	104989800	1.037	0.747	0.560	1.044
Tan	5	105040501	105040800	0.215	0.468	0.107	0.279
Tan	5	105438901	105439200	1.328	2.085	1.073	1.313
Tan	5	105611101	105611400	0.771	1.311	0.613	0.791
Tan	5	105611401	105611700	0.345	1.242	0.580	0.862
Tan	5	105667201	105667500	0.773	1.126	0.560	0.669
Tan	5	106049401	106049700	0.584	1.635	1.246	2.110
Tan	5	106060801	106061100	1.254	2.191	1.086	1.602
Tan	5	106456201	106456500	0.584	0.159	1.003	1.430
Tan	5	106529401	106529700	0.268	0.539	0.263	0.167
Tan	5	106830301	106830600	3.696	2.665	1.966	3.442
Tan	5	107142901	107143200	0.398	0.846	0.457	0.355
Tan	5	107300701	107301000	0.505	0.817	0.419	0.396
Tan	5	107524501	107524800	0.584	0.936	0.510	0.497
Tan	5	107586001	107586300	0.186	0.654	0.325	0.056
Tan	6	2401	2700	0.452	0.910	0.395	0.639
Tan	6	259501	259800	1.915	1.401	2.316	1.739
Tan	6	326401	326700	0.397	0.680	0.428	0.913
Tan	6	334801	335100	0.187	0.605	0.239	0.228
Tan	6	424801	425100	0.132	0.395	0.189	0.167
Tan	6	1239001	1239300	0.395	0.983	0.539	0.441
Tan	6	1596001	1596300	0.560	0.096	0.374	0.416
Tan	6	1652401	1652700	0.211	0.491	0.156	0.248
Tan	6	3304801	3305100	0.134	1.233	0.317	0.420
Tan	6	3330001	3330300	1.355	0.865	1.037	1.546
Tan	6	3514801	3515100	0.265	0.888	0.292	0.299
Tan	6	3693301	3693600	0.186	0.420	0.156	0.112
Tan	6	3765301	3765600	1.144	1.028	1.703	1.410
Tan	6	3793201	3793500	0.345	0.421	0.506	0.223
Tan	6	3882301	3882600	0.237	0.654	0.366	0.193
Tan	6	4100101	4100400	0.292	0.749	0.337	0.223
Tan	6	4121701	4122000	0.106	0.515	0.136	0.360
Tan	6	4412401	4412700	3.241	2.124	2.967	3.163
Tan	6	4454701	4455000	0.240	0.466	0.210	0.106
Tan	6	4490701	4491000	0.400	0.818	0.473	0.517
Tan	6	4569301	4569600	1.649	1.047	1.538	1.714
Tan	6	4569601	4569900	1.865	0.953	1.868	1.647
Tan	6	4688401	4688700	0.398	0.747	0.419	0.279
Tan	6	4868401	4868700	0.374	1.148	0.477	0.639
Tan	6	5427901	5428200	0.292	0.444	0.239	0.223
Tan	6	5833501	5833800	0.637	0.914	0.526	0.472

Tan	6	6491101	6491400	0.160	0.468	0.288	0.167
Tan	6	6513601	6513900	3.857	5.776	3.308	4.833
Tan	6	6581401	6581700	1.223	1.901	1.324	1.253
Tan	6	6721201	6721500	0.316	0.584	0.214	0.137
Tan	6	6726901	6727200	0.321	0.657	0.272	0.309
Tan	6	6739201	6739500	0.213	0.466	0.321	0.360
Tan	6	7029601	7029900	0.481	0.912	0.296	0.391
Tan	6	7041001	7041300	0.187	0.513	0.054	0.391
Tan	6	7130101	7130400	0.213	0.491	0.234	0.198
Tan	6	7232401	7232700	0.479	0.656	0.346	0.385
Tan	6	7254301	7254600	0.400	0.918	0.346	0.294
Tan	6	7305601	7305900	0.132	0.420	0.160	0.279
Tan	6	7640701	7641000	0.482	1.004	0.481	0.234
Tan	6	7861501	7861800	0.160	0.515	0.189	0.268
Tan	6	7861801	7862100	0.081	0.446	0.267	0.193
Tan	6	8376301	8376600	1.426	3.409	2.082	2.251
Tan	6	8376601	8376900	1.647	4.184	2.336	2.615
Tan	6	8399401	8399700	0.134	0.566	0.082	0.086
Tan	6	8532001	8532300	0.108	0.442	0.132	0.173
Tan	6	9003601	9003900	1.594	0.369	2.697	2.885
Tan	6	9107701	9108000	0.527	1.530	0.593	0.517
Tan	6	9845101	9845400	0.426	0.749	0.432	0.411
Tan	6	10650301	10650600	0.055	0.442	0.185	0.106
Tan	6	10659001	10659300	0.160	0.517	0.160	0.081
Tan	6	10942801	10943100	0.615	1.586	0.889	1.024
Tan	6	11416201	11416500	0.529	2.785	1.005	0.457
Tan	6	11635201	11635500	0.106	0.607	0.029	0.193
Tan	6	11635501	11635800	0.160	0.822	0.029	0.263
Tan	6	11637601	11637900	0.184	0.723	0.136	0.182
Tan	6	11660101	11660400	0.077	0.635	0.054	0.056
Tan	6	11934601	11934900	0.235	0.820	0.165	0.274
Tan	6	12212401	12212700	0.319	0.493	0.082	0.254
Tan	6	12244801	12245100	3.505	2.446	1.786	1.881
Tan	6	12266701	12267000	0.292	0.684	0.263	0.248
Tan	6	12267301	12267600	4.067	3.010	4.463	4.467
Tan	6	12388501	12388800	0.637	0.916	0.428	0.329
Tan	6	12967801	12968100	0.426	0.631	0.395	0.304
Tan	6	13043401	13043700	0.263	1.538	0.410	0.243
Tan	6	13085701	13086000	0.163	0.796	0.263	0.117
Tan	6	13171501	13171800	0.345	0.820	0.457	0.198
Tan	6	13209301	13209600	0.161	0.515	0.140	0.061
Tan	6	13492201	13492500	0.611	0.281	0.453	0.416
Tan	6	13629601	13629900	0.452	0.817	0.337	0.329
Tan	6	13965901	13966200	0.295	0.723	0.185	0.294
Tan	6	14034601	14034900	1.091	1.424	1.168	1.247
Tan	6	14146501	14146800	0.134	0.465	0.239	0.223
Tan	6	14276701	14277000	0.213	0.352	0.103	0.527
Tan	6	14343901	14344200	0.215	0.421	0.613	0.223
Tan	6	14540701	14541000	0.160	0.723	0.379	0.355
Tan	6	14655001	14655300	0.105	0.418	0.185	0.137
Tan	6	14713801	14714100	0.240	0.423	0.054	0.167
Tan	6	15017401	15017700	0.345	0.071	0.497	0.335
Tan	6	15163801	15164100	0.235	1.051	0.337	0.441
Tan	6	15417901	15418200	0.160	0.423	0.082	0.142
Tan	6	16101901	16102200	0.900	1.787	0.782	0.846
Tan	6	16102201	16102500	0.797	1.740	0.778	0.963
Tan	6	16135201	16135500	0.244	2.825	0.622	2.347
Tan	6	16346701	16347000	0.105	0.465	0.107	0.081
Tan	6	16454701	16455000	0.319	0.489	0.160	0.279
Tan	6	16800901	16801200	0.529	0.049	0.506	0.542
Tan	6	16937401	16937700	0.161	0.468	0.087	0.167
Tan	6	17122501	17122800	0.373	1.105	0.526	0.512
Tan	6	17268301	17268600	0.053	0.446	0.078	0.051
Tan	6	17299501	17299800	0.292	0.609	0.239	0.304
Tan	6	17343001	17343300	1.063	1.424	0.740	0.893
Tan	6	17425201	17425500	1.542	3.274	1.444	1.475
Tan	6	17667601	17667900	0.213	0.446	0.054	0.329

Tan	6	17691601	17691900	0.481	1.266	0.410	0.517
Tan	6	17757001	17757300	0.239	0.493	0.181	0.274
Tan	6	17882401	17882700	0.106	0.395	0.103	0.137
Tan	6	18148201	18148500	0.187	0.517	0.160	0.173
Tan	6	18869701	18870000	0.158	0.493	0.132	0.167
Tan	6	19440901	19441200	0.398	0.631	0.428	0.106
Tan	6	19530901	19531200	0.292	1.771	0.234	0.553
Tan	6	19531201	19531500	0.716	2.410	0.448	0.861
Tan	6	19869301	19869600	0.079	0.466	0.185	0.031
Tan	6	19879201	19879500	0.507	0.654	0.481	0.304
Tan	6	20096101	20096400	2.521	3.780	2.332	2.059
Tan	6	20096401	20096700	1.254	2.130	1.144	1.161
Tan	6	20119501	20119800	0.637	1.146	0.477	0.497
Tan	6	20119801	20120100	0.510	1.407	0.506	0.710
Tan	6	20405101	20405400	0.718	0.448	0.823	0.806
Tan	6	20428801	20429100	0.240	0.444	0.210	0.248
Tan	6	20448901	20449200	0.881	0.257	0.613	0.628
Tan	6	20455201	20455500	0.428	0.045	0.457	0.974
Tan	6	21789601	21789900	0.292	0.564	0.317	0.137
Tan	6	22130401	22130700	0.158	0.468	0.181	0.132
Tan	6	22257301	22257600	0.505	0.350	0.078	0.274
Tan	6	23314501	23314800	1.061	2.579	1.402	1.603
Tan	6	23610001	23610300	0.400	1.214	0.493	0.563
Tan	6	23721001	23721300	0.345	0.656	0.054	0.132
Tan	6	23745001	23745300	0.290	0.863	0.292	0.360
Tan	6	23802901	23803200	0.318	0.466	0.054	0.081
Tan	6	24063301	24063600	0.132	0.468	0.214	0.385
Tan	6	24358501	24358800	0.507	0.796	0.638	0.411
Tan	6	24480601	24480900	0.215	0.513	0.000	0.167
Tan	6	24510601	24510900	0.371	0.633	0.374	0.441
Tan	6	24749701	24750000	0.292	0.234	0.477	0.167
Tan	6	24997201	24997500	0.423	1.056	0.303	0.279
Tan	6	25007101	25007400	1.462	2.221	1.293	1.252
Tan	6	25007401	25007700	0.584	1.105	0.568	0.391
Tan	6	25126201	25126500	0.132	0.397	0.160	0.608
Tan	6	25187101	25187400	0.371	1.131	0.399	0.309
Tan	6	25309801	25310100	0.749	1.736	0.486	1.035
Tan	6	25428001	25428300	3.081	1.429	3.370	3.438
Tan	6	25570201	25570500	0.106	0.279	0.082	0.497
Tan	6	25848301	25848600	0.692	1.405	0.662	0.694
Tan	6	25911901	25912200	0.323	0.963	0.288	0.248
Tan	6	25932601	25932900	0.215	0.511	0.214	0.193
Tan	6	25991101	25991400	0.160	0.448	0.136	0.137
Tan	6	26029801	26030100	0.182	0.678	0.234	0.167
Tan	6	26315101	26315400	0.211	0.584	0.214	0.284
Tan	6	26483701	26484000	0.187	0.560	0.165	0.268
Tan	6	26613901	26614200	0.265	0.818	0.267	0.132
Tan	6	27065101	27065400	1.034	1.613	0.852	0.396
Tan	6	27200701	27201000	0.187	0.491	0.259	0.081
Tan	6	27775501	27775800	0.106	0.605	0.350	0.112
Tan	6	27863101	27863400	0.268	0.538	0.292	0.329
Tan	6	28329601	28329900	0.108	0.588	0.160	0.106
Tan	6	28727101	28727400	0.373	0.560	0.103	0.248
Tan	6	29333101	29333400	0.876	1.894	0.700	1.227
Tan	6	29376601	29376900	0.398	1.090	0.263	0.157
Tan	6	29436901	29437200	0.294	0.697	0.160	0.268
Tan	6	29482501	29482800	0.240	0.562	0.165	0.218
Tan	6	29880901	29881200	0.244	0.654	0.132	0.193
Tan	6	29965201	29965500	0.503	0.326	0.132	0.137
Tan	6	30545401	30545700	0.187	0.444	0.189	0.162
Tan	6	31318201	31318500	0.027	0.491	0.136	0.223
Tan	6	31388101	31388400	0.186	0.468	0.317	0.223
Tan	6	31932601	31932900	0.055	0.466	0.029	0.056
Tan	6	32182501	32182800	0.347	0.116	0.477	0.360
Tan	6	32994901	32995200	0.132	0.517	0.082	0.076
Tan	6	33003901	33004200	0.644	1.259	0.526	0.502
Tan	6	34221901	34222200	0.450	0.116	0.428	0.416

Tan	6	34764301	34764600	0.160	0.446	0.234	0.142
Tan	6	34781101	34781400	0.184	1.315	0.189	0.587
Tan	6	34818601	34818900	0.349	0.961	0.439	0.411
Tan	6	34831501	34831800	0.108	0.534	0.239	0.193
Tan	6	34842601	34842900	0.161	0.513	0.239	0.081
Tan	6	34976701	34977000	0.720	1.212	0.642	0.400
Tan	6	35181901	35182200	0.424	0.303	0.662	0.416
Tan	6	35259901	35260200	0.213	0.468	0.103	0.243
Tan	6	35292601	35292900	0.397	0.680	0.214	0.380
Tan	6	35358301	35358600	1.462	2.360	1.572	1.770
Tan	6	35424901	35425200	0.424	0.888	0.448	0.452
Tan	6	35425201	35425500	0.349	0.983	0.428	0.340
Tan	6	35633101	35633400	0.350	1.989	0.210	0.268
Tan	6	35807101	35807400	0.507	0.328	0.214	0.497
Tan	6	35976601	35976900	0.371	0.729	0.399	0.441
Tan	6	36085501	36085800	0.750	1.686	0.600	1.050
Tan	6	36288901	36289200	0.237	0.466	0.210	0.248
Tan	6	36396301	36396600	0.292	1.045	0.325	0.431
Tan	6	37383601	37383900	0.428	0.257	0.025	0.583
Tan	6	37512301	37512600	2.643	4.085	2.526	1.962
Tan	6	37563901	37564200	0.108	0.822	0.350	0.279
Tan	6	37564201	37564500	0.190	1.335	0.707	0.538
Tan	6	38109601	38109900	0.560	1.124	0.675	0.452
Tan	6	38304001	38304300	0.319	0.798	0.296	0.502
Tan	6	39749101	39749400	1.626	2.128	1.074	1.572
Tan	6	39801301	39801600	1.174	2.049	1.313	1.111
Tan	6	39933601	39933900	0.266	0.867	0.185	0.202
Tan	6	39938101	39938400	0.077	0.470	0.185	0.051
Tan	6	40296901	40297200	0.295	0.723	0.267	0.182
Tan	6	41274301	41274600	0.187	0.588	0.160	0.254
Tan	6	41508301	41508600	0.266	0.468	0.082	0.162
Tan	6	41602801	41603100	0.323	0.933	0.357	0.223
Tan	6	41932501	41932800	0.211	0.420	0.239	0.051
Tan	6	42253801	42254100	0.106	0.792	0.267	0.492
Tan	6	42267601	42267900	0.161	0.468	0.239	0.198
Tan	6	43245301	43245600	0.477	1.009	0.453	0.380
Tan	6	43781401	43781700	1.118	0.466	0.938	1.064
Tan	6	44076901	44077200	1.729	2.130	1.625	1.703
Tan	6	44432701	44433000	0.132	0.466	0.185	0.167
Tan	6	44670001	44670300	0.424	0.654	0.185	0.284
Tan	6	44686501	44686800	0.055	0.401	0.103	0.086
Tan	6	44985601	44985900	0.294	0.631	0.239	0.466
Tan	6	45161401	45161700	0.981	1.564	0.959	0.923
Tan	6	45221401	45221700	0.266	0.541	0.078	0.081
Tan	6	45370801	45371100	0.268	0.980	0.346	0.345
Tan	6	45550501	45550800	0.263	0.865	0.526	0.411
Tan	6	45589201	45589500	0.292	0.942	0.123	0.248
Tan	6	45600301	45600600	0.663	0.326	0.584	0.639
Tan	6	45730801	45731100	0.211	0.494	0.239	0.193
Tan	6	45740401	45740700	0.294	0.609	0.346	0.497
Tan	6	45748201	45748500	0.584	2.457	0.392	0.563
Tan	6	45748501	45748800	0.266	0.775	0.000	0.173
Tan	6	45778201	45778500	0.424	0.796	0.267	0.365
Tan	6	45894901	45895200	0.189	0.607	0.189	0.268
Tan	6	46042801	46043100	0.368	1.262	0.473	0.477
Tan	6	46325101	46325400	0.639	0.418	0.798	0.857
Tan	6	46329901	46330200	0.507	1.101	0.551	0.583
Tan	6	46586101	46586400	1.515	2.147	1.402	1.613
Tan	6	46856401	46856700	0.027	0.395	0.078	0.000
Tan	6	47261701	47262000	0.319	0.699	0.346	0.350
Tan	6	47436601	47436900	0.000	0.425	0.107	0.081
Tan	6	47648401	47648700	0.187	0.631	0.321	0.243
Tan	6	48064201	48064500	0.663	0.423	0.189	0.690
Tan	6	48284101	48284400	0.052	0.541	0.127	0.218
Tan	6	48812101	48812400	0.079	0.444	0.288	0.274
Tan	6	49278901	49279200	0.108	0.560	0.214	0.051
Tan	6	49806001	49806300	0.266	0.633	0.185	0.081

Tan	6	50196001	50196300	0.242	0.745	0.370	0.340
Tan	6	50541601	50541900	0.079	0.466	0.111	0.132
Tan	6	50673001	50673300	1.996	1.152	1.699	1.694
Tan	6	50673301	50673600	3.581	1.502	3.533	2.511
Tan	6	50765701	50766000	0.213	1.558	0.727	0.178
Tan	6	51103801	51104100	2.244	3.435	2.250	2.313
Tan	6	52755901	52756200	2.918	1.659	2.403	2.388
Tan	6	53150701	53151000	0.110	0.558	0.107	0.056
Tan	6	54199501	54199800	0.345	0.466	0.317	0.304
Tan	6	54447001	54447300	0.240	0.725	0.288	0.157
Tan	6	54656101	54656400	0.134	0.513	0.317	0.086
Tan	6	55977601	55977900	0.826	0.631	1.222	0.299
Tan	6	56427001	56427300	0.266	0.562	0.296	0.248
Tan	6	56728201	56728500	0.268	0.987	0.281	0.238
Tan	6	56773201	56773500	0.242	0.747	0.194	0.112
Tan	6	56773501	56773800	0.350	0.935	0.218	0.147
Tan	6	56907001	56907300	0.131	0.444	0.103	0.167
Tan	6	57155401	57155700	0.134	0.566	0.054	0.198
Tan	6	57435001	57435300	0.052	0.425	0.049	0.031
Tan	6	57525901	57526200	0.245	0.938	0.218	0.268
Tan	6	57640501	57640800	0.082	0.442	0.156	0.112
Tan	6	57649801	57650100	1.303	1.995	1.242	1.009
Tan	6	57650101	57650400	0.744	1.008	0.448	0.751
Tan	6	57774001	57774300	0.589	1.506	0.691	0.710
Tan	6	58381501	58381800	1.036	1.686	1.081	1.298
Tan	6	58393801	58394100	0.345	0.584	0.181	0.441
Tan	6	58416001	58416300	0.211	0.867	0.305	0.122
Tan	6	58429501	58429800	0.235	1.429	0.510	0.573
Tan	6	58588801	58589100	0.079	0.487	0.054	0.173
Tan	6	58965301	58965600	0.560	0.701	0.160	0.466
Tan	6	59031001	59031300	0.318	0.985	0.363	0.268
Tan	6	59072701	59073000	0.637	0.794	0.638	0.583
Tan	6	59151901	59152200	0.292	0.446	0.214	0.167
Tan	6	59471101	59471400	0.319	0.727	0.399	0.416
Tan	6	59478001	59478300	0.611	0.935	0.584	0.492
Tan	6	59492101	59492400	0.477	0.165	0.695	0.411
Tan	6	59628301	59628600	0.268	0.699	0.321	0.335
Tan	6	59629801	59630100	0.349	0.794	0.481	0.492
Tan	6	59921101	59921400	0.321	0.607	0.399	0.304
Tan	6	60123001	60123300	0.376	1.096	0.629	0.472
Tan	6	60662101	60662400	1.592	1.051	1.694	1.937
Tan	6	60733201	60733500	0.350	0.891	0.399	0.466
Tan	6	60917401	60917700	0.053	0.420	0.103	0.162
Tan	6	61165501	61165800	0.294	0.466	0.210	0.218
Tan	6	61346401	61346700	0.239	0.611	0.181	0.193
Tan	6	61745101	61745400	0.558	0.281	0.424	0.142
Tan	6	61828501	61828800	0.187	0.751	0.181	0.340
Tan	6	62087401	62087700	0.773	1.148	0.502	0.466
Tan	6	62123401	62123700	0.290	0.678	0.292	0.284
Tan	6	63809101	63809400	0.400	0.727	0.370	0.335
Tan	6	64092001	64092300	0.079	0.466	0.160	0.198
Tan	6	65409001	65409300	0.240	0.513	0.132	0.213
Tan	6	66326101	66326400	0.314	1.155	0.111	0.279
Tan	6	66595801	66596100	0.134	0.444	0.132	0.025
Tan	6	66851401	66851700	0.481	0.912	0.691	0.715
Tan	6	67163101	67163400	0.689	1.433	0.486	0.279
Tan	6	67269901	67270200	0.429	1.152	0.473	0.309
Tan	6	67270201	67270500	0.371	1.197	0.468	0.335
Tan	6	67413001	67413300	0.452	0.704	0.321	0.274
Tan	6	67877701	67878000	0.663	0.212	0.609	0.751
Tan	6	67907401	67907700	0.160	0.588	0.165	0.173
Tan	6	68242201	68242500	0.077	0.631	0.296	0.309
Tan	6	68343601	68343900	0.186	0.328	0.111	0.497
Tan	6	68440501	68440800	0.186	0.680	0.165	0.126
Tan	6	68479501	68479800	0.182	0.916	0.136	0.375
Tan	6	68549401	68549700	0.591	1.212	0.082	0.345
Tan	6	68572501	68572800	0.532	0.768	0.296	0.527

Tan	6	68598601	68598900	0.537	1.701	0.424	0.258
Tan	6	68603101	68603400	1.358	2.414	0.992	0.994
Tan	6	68820901	68821200	0.266	0.468	0.189	0.248
Tan	6	69036901	69037200	0.373	0.586	0.317	0.416
Tan	6	69143401	69143700	0.319	0.444	0.214	0.218
Tan	6	69544801	69545100	0.134	0.444	0.205	0.167
Tan	6	69915301	69915600	0.319	0.841	0.374	0.426
Tan	6	70311301	70311600	0.160	0.562	0.317	0.213
Tan	6	71394601	71394900	0.186	0.444	0.082	0.142
Tan	6	71844001	71844300	0.189	0.583	0.160	0.112
Tan	6	71890501	71890800	0.721	1.122	0.774	0.573
Tan	6	72006301	72006600	0.000	0.448	0.185	0.198
Tan	6	72020101	72020400	0.237	0.678	0.054	0.315
Tan	6	72466801	72467100	0.477	0.704	0.267	0.335
Tan	6	72795601	72795900	0.666	0.352	0.633	0.776
Tan	6	72797401	72797700	0.479	0.234	0.531	0.583
Tan	6	72891601	72891900	0.477	0.071	0.292	0.243
Tan	6	73551001	73551300	0.773	0.933	0.399	0.608
Tan	6	75210901	75211200	0.639	1.073	0.560	0.456
Tan	6	75822301	75822600	0.373	0.676	0.370	0.274
Tan	6	77763301	77763600	0.290	0.536	0.292	0.198
Tan	6	78680701	78681000	0.611	0.936	0.321	0.492
Tan	6	79303801	79304100	0.398	0.583	0.263	0.385
Tan	6	82182001	82182300	0.400	0.189	0.453	0.466
Tan	6	83113501	83113800	0.421	0.843	0.370	0.441
Tan	6	83141101	83141400	0.428	0.938	0.395	0.294
Tan	6	83215201	83215500	0.184	0.629	0.234	0.248
Tan	6	84161401	84161700	0.345	0.513	0.613	0.248
Tan	6	85021201	85021500	0.132	0.448	0.185	0.106
Tan	6	86156401	86156700	0.186	0.515	0.234	0.132
Tan	6	86238001	86238300	1.171	2.135	1.235	0.882
Tan	6	86370001	86370300	0.424	1.755	0.312	0.416
Tan	6	86806201	86806500	0.103	0.493	0.025	0.117
Tan	6	88314601	88314900	0.105	0.442	0.210	0.223
Tan	6	89035501	89035800	0.739	1.551	0.461	0.573
Tan	6	89152801	89153100	0.186	0.420	0.078	0.061
Tan	6	89236801	89237100	0.586	0.515	0.638	0.385
Tan	6	89441701	89442000	0.134	0.511	0.103	0.081
Tan	6	89477701	89478000	0.211	0.607	0.292	0.056
Tan	6	89565301	89565600	0.319	0.674	0.218	0.112
Tan	6	89652601	89652900	0.962	1.614	0.568	0.837
Tan	6	90287401	90287700	0.503	1.054	0.490	0.517
Tan	6	90772501	90772800	1.542	2.489	1.300	1.622
Tan	6	90845401	90845700	0.265	0.468	0.136	0.198
Tan	6	90933301	90933600	0.610	1.004	0.477	0.380
Tan	6	91022101	91022400	0.505	0.656	0.292	0.304
Tan	6	91179601	91179900	0.000	0.401	0.029	0.081
Tan	6	91212001	91212300	0.560	0.257	0.448	0.522
Tan	6	91234201	91234500	2.552	3.248	2.535	2.099
Tan	6	92265601	92265900	0.239	0.659	0.288	0.223
Tan	6	93162001	93162300	0.345	0.701	0.399	0.350
Tan	6	93288001	93288300	0.266	0.466	0.189	0.248
Tan	6	93722401	93722700	0.321	0.538	0.210	0.198
Tan	6	94086301	94086600	0.582	1.053	0.486	0.563
Tan	6	94152001	94152300	0.716	0.232	0.934	0.547
Tan	6	94516801	94517100	0.134	0.539	0.078	0.309
Tan	6	94639201	94639500	1.005	2.137	0.930	0.912
Tan	6	94717801	94718100	0.213	0.517	0.214	0.086
Tan	6	95100901	95101200	0.586	0.820	0.555	0.547
Tan	6	95147401	95147700	0.053	0.704	0.103	0.031
Tan	6	95467501	95467800	0.611	1.478	0.600	0.628
Tan	6	96243001	96243300	0.134	0.491	0.103	0.157
Tan	6	96296401	96296700	0.376	0.933	0.296	0.466
Tan	6	96305401	96305700	0.242	0.583	0.210	0.274
Tan	6	96325201	96325500	0.132	0.470	0.185	0.106
Tan	6	96487801	96488100	0.131	0.515	0.156	0.279
Tan	6	96567001	96567300	0.106	0.466	0.136	0.274

Tan	6	96742501	96742800	0.186	0.470	0.160	0.031
Tan	6	96747001	96747300	0.187	0.494	0.160	0.218
Tan	6	96792901	96793200	1.300	2.339	1.683	1.592
Tan	6	97218301	97218600	0.211	0.493	0.136	0.086
Tan	6	97266901	97267200	0.981	1.545	0.778	1.044
Tan	6	97464901	97465200	0.297	1.062	0.296	0.268
Tan	6	97533601	97533900	0.319	1.009	0.551	0.486
Tan	6	97569301	97569600	0.079	0.444	0.103	0.167
Tan	6	97883701	97884000	0.850	1.053	0.613	0.385
Tan	6	97934701	97935000	0.106	0.470	0.185	0.198
Tan	6	97951201	97951500	0.237	0.721	0.132	0.187
Tan	6	98003101	98003400	0.823	1.146	0.736	0.553
Tan	6	98026501	98026800	0.453	0.792	0.453	0.213
Tan	6	98142901	98143200	0.905	0.371	0.831	0.812
Tan	6	98143201	98143500	1.165	0.354	1.255	1.294
Tan	6	98688001	98688300	0.847	0.376	1.251	0.771
Tan	6	98867701	98868000	0.215	0.633	0.205	0.167
Tan	6	98955601	98955900	0.108	0.654	0.267	0.355
Tan	6	99280501	99280800	0.134	0.491	0.210	0.309
Tan	6	99294901	99295200	0.105	0.493	0.165	0.025
Tan	6	100262401	100262700	0.266	0.420	0.107	0.223
Tan	6	100390501	100390800	0.266	0.584	0.453	0.309
Tan	6	100617301	100617600	0.266	0.564	0.181	0.243
Tan	6	100649101	100649400	0.290	0.961	0.403	0.588
Tan	6	100773601	100773900	0.160	0.517	0.156	0.218
Tan	6	101473501	101473800	0.186	0.513	0.185	0.112
Tan	6	101487901	101488200	0.477	0.747	0.448	0.477
Tan	6	101490301	101490600	0.799	1.032	0.638	0.634
Tan	6	101875201	101875500	0.186	0.517	0.165	0.112
Tan	6	101882401	101882700	1.936	0.929	0.330	1.582
Tan	6	102489901	102490200	0.108	0.538	0.132	0.142
Tan	6	102530101	102530400	3.588	1.856	3.821	3.413
Tan	6	102636601	102636900	0.211	0.633	0.189	0.335
Tan	6	102636901	102637200	0.265	0.680	0.267	0.466
Tan	6	102645301	102645600	0.400	1.098	0.457	0.395
Tan	6	102786301	102786600	4.683	5.492	3.723	4.339
Tan	6	102973201	102973500	0.423	0.961	0.259	0.431
Tan	6	103018501	103018800	1.223	1.500	1.012	0.801
Tan	6	103055101	103055400	0.292	0.586	0.774	0.254
Tan	6	103230301	103230600	0.319	0.919	0.205	0.304
Tan	6	103230601	103230900	0.373	0.895	0.156	0.279
Tan	6	103231501	103231800	0.079	0.420	0.210	0.142
Tan	6	103316401	103316700	0.268	0.727	0.259	0.228
Tan	6	103663201	103663500	0.428	0.936	0.370	0.329
Tan	6	103775101	103775400	0.503	0.961	0.267	0.421
Tan	6	103835101	103835400	0.268	0.702	0.145	0.137
Tan	6	103835401	103835700	0.244	1.613	0.330	0.218
Tan	6	103853101	103853400	0.744	1.002	0.350	0.441
Tan	6	104139601	104139900	0.718	1.541	0.859	0.679
Tan	6	104139901	104140200	0.663	1.120	0.638	0.690
Tan	6	104183101	104183400	0.265	0.444	0.210	0.051
Tan	6	104290801	104291100	0.953	0.206	0.785	0.938
Tan	6	104291101	104291400	4.247	1.390	4.427	4.270
Tan	6	104335201	104335500	0.026	0.395	0.214	0.162
Tan	6	104384401	104384700	0.235	0.723	0.107	0.309
Tan	6	104457001	104457300	0.826	1.594	0.921	0.745
Tan	6	104463901	104464200	0.160	0.423	0.136	0.218
Tan	6	104697001	104697300	0.184	0.607	0.136	0.167
Tan	6	104812201	104812500	0.482	1.143	0.082	0.344
Tan	6	104817901	104818200	0.716	1.586	0.740	0.853
Tan	6	105021001	105021300	0.161	0.423	0.025	0.106
Tan	6	105047401	105047700	0.319	0.517	0.054	0.031
Tan	6	105055801	105056100	0.266	0.395	0.107	0.025
Tan	6	105094801	105095100	0.186	0.657	0.239	0.234
Tan	6	105292501	105292800	0.134	0.515	0.424	0.304
Tan	6	105601501	105601800	0.773	1.631	0.720	0.705
Tan	6	106183501	106183800	0.163	0.493	0.103	0.167

Tan	6	106257601	106257900	0.106	0.538	0.058	0.167
Tan	6	106380901	106381200	0.290	0.560	0.210	0.309
Tan	6	106821901	106822200	0.186	0.489	0.098	0.137
Tan	6	106960801	106961100	0.424	0.629	0.107	0.304
Tan	6	107636701	107637000	0.666	1.811	0.667	0.654
Tan	6	107863801	107864100	0.187	0.420	0.107	0.223
Tan	6	108022201	108022500	0.239	0.562	0.687	0.248
Tan	6	108176101	108176400	0.290	0.609	0.267	0.254
Tan	6	108344701	108345000	0.694	1.212	0.633	0.614
Tan	6	108414001	108414300	0.215	0.468	0.127	0.137
Tan	6	108437101	108437400	0.290	0.813	0.325	0.335
Tan	6	108911701	108912000	0.132	0.423	0.136	0.117
Tan	6	108912601	108912900	0.268	0.515	0.107	0.167
Tan	6	110039401	110039700	0.931	1.807	1.023	0.907
Tan	6	110073301	110073600	0.106	0.680	0.107	0.187
Tan	6	110082901	110083200	0.423	1.079	0.588	0.659
Tan	6	110127301	110127600	0.026	0.466	0.267	0.076
Tan	6	110192401	110192700	0.136	0.538	0.082	0.137
Tan	6	110473801	110474100	0.428	1.008	0.526	0.461
Tan	6	110498401	110498700	0.434	1.313	0.165	0.243
Tan	6	110769001	110769300	0.479	0.963	0.259	0.578
Tan	6	110809501	110809800	0.349	0.702	0.243	0.167
Tan	6	110837401	110837700	0.132	0.517	0.049	0.025
Tan	6	110971801	110972100	2.952	1.324	2.403	2.474
Tan	6	110972101	110972400	2.523	1.234	2.207	2.211
Tan	6	111334801	111335100	0.428	0.745	0.321	0.324
Tan	6	111391201	111391500	0.269	0.753	0.263	0.299
Tan	6	111500401	111500700	0.081	0.489	0.185	0.167
Tan	6	111636001	111636300	0.397	0.933	0.276	0.436
Tan	6	111886201	111886500	0.240	0.770	0.296	0.335
Tan	6	111897901	111898200	0.053	0.536	0.132	0.228
Tan	6	111898201	111898500	0.079	0.442	0.132	0.081
Tan	6	111922201	111922500	0.239	0.719	0.136	0.193
Tan	6	112063501	112063800	0.479	1.129	0.448	0.441
Tan	6	112296601	112296900	0.292	1.030	0.370	0.355
Tan	6	112460101	112460400	0.160	0.493	0.160	0.304
Tan	6	112483501	112483800	0.213	0.584	0.145	0.193
Tan	6	112599601	112599900	0.610	0.796	0.531	0.583
Tan	6	112650601	112650900	0.665	1.126	0.695	0.426
Tan	6	112730101	112730400	1.700	2.946	2.127	2.408
Tan	6	112791001	112791300	2.454	3.680	2.274	2.566
Tan	6	112839601	112839900	0.131	0.562	0.214	0.228
Tan	6	113136901	113137200	0.318	0.583	0.160	0.218
Tan	6	113170501	113170800	0.452	0.818	0.584	0.446
Tan	6	113208301	113208600	0.744	1.099	0.765	0.608
Tan	6	113362501	113362800	1.560	2.433	1.415	1.399
Tan	6	113362801	113363100	1.515	2.523	1.674	1.638
Tan	6	113369401	113369700	1.328	0.375	0.983	0.989
Tan	6	113369701	113370000	0.452	1.154	0.272	0.370
Tan	6	113451301	113451600	2.505	0.862	1.460	1.760
Tan	6	113459401	113459700	0.319	0.725	0.453	0.243
Tan	6	113565601	113565900	1.224	1.616	1.003	0.806
Tan	6	113674201	113674500	3.079	2.100	2.941	3.088
Tan	6	113699101	113699400	1.063	0.191	0.584	0.832
Tan	6	113771701	113772000	2.439	1.491	2.563	2.601
Tan	6	113803801	113804100	3.160	3.875	2.595	2.891
Tan	6	113851201	113851500	0.694	0.322	0.716	0.801
Tan	6	114012601	114012900	1.307	0.586	1.061	1.308
Tan	6	114030601	114030900	0.266	0.444	0.210	0.086
Tan	6	114128401	114128700	0.960	1.614	0.921	1.267
Tan	6	114171001	114171300	0.132	0.444	0.210	0.254
Tan	6	114295801	114296100	0.958	1.238	0.720	0.968
Tan	6	114383101	114383400	2.670	1.191	2.485	2.921
Tan	6	114392401	114392700	2.528	0.472	1.858	3.027
Tan	6	114681001	114681300	6.377	4.270	5.241	9.725
Tan	6	114743101	114743400	0.932	0.676	0.987	1.080
Tan	6	114999901	115000200	3.223	1.833	2.985	2.916

Tan	6	115137001	115137300	0.745	0.981	0.613	0.690
Tan	6	115177201	115177500	6.551	4.411	5.858	5.978
Tan	6	115209601	115209900	4.412	2.840	4.641	4.117
Tan	6	115437901	115438200	6.117	3.971	5.453	5.031
Tan	6	115589101	115589400	0.265	0.609	0.234	0.248
Tan	6	115744501	115744800	0.773	1.665	0.575	0.745
Tan	6	115747501	115747800	0.776	1.450	0.580	0.776
Tan	6	115764601	115764900	2.078	0.423	1.728	2.200
Tan	6	115764901	115765200	2.162	0.496	1.921	2.469
Tan	6	115790701	115791000	0.505	1.195	0.448	0.725
Tan	6	115813501	115813800	0.402	0.654	0.321	0.360
Tan	6	115845901	115846200	0.158	0.468	0.263	0.162
Tan	6	115890901	115891200	3.363	5.220	3.623	3.580
Tan	6	115896301	115896600	0.211	0.515	0.025	0.268
Tan	6	115901401	115901700	0.081	0.465	0.239	0.248
Tan	6	116061901	116062200	1.197	2.015	1.387	1.156
Tan	6	116407501	116407800	1.784	1.474	1.061	0.923
Tan	6	116580601	116580900	2.207	1.332	2.020	2.515
Tan	6	116681401	116681700	10.671	8.026	9.607	11.870
Tan	6	116697001	116697300	8.451	6.541	8.514	9.512
Tan	6	116927401	116927700	0.611	0.210	0.667	0.695
Tan	6	116986201	116986500	0.347	0.729	0.160	0.259
Tan	7	37201	37500	0.565	2.210	0.658	0.878
Tan	7	316801	317100	3.883	3.201	4.184	3.990
Tan	7	468301	468600	1.011	2.302	0.908	0.963
Tan	7	596101	596400	1.224	2.337	1.661	1.400
Tan	7	683401	683700	0.505	1.888	0.493	0.396
Tan	7	692101	692400	0.290	1.000	0.415	0.182
Tan	7	971401	971700	0.215	1.442	0.239	0.203
Tan	7	971701	972000	0.764	3.684	0.722	0.725
Tan	7	972001	972300	0.184	0.657	0.185	0.187
Tan	7	1018201	1018500	2.365	2.974	1.937	1.648
Tan	7	1485901	1486200	0.637	0.916	0.346	0.664
Tan	7	1505401	1505700	0.295	0.680	0.350	0.309
Tan	7	1517701	1518000	0.769	1.800	0.651	0.792
Tan	7	1611301	1611600	0.158	0.468	0.054	0.051
Tan	7	2092501	2092800	0.187	0.538	0.243	0.268
Tan	7	2152201	2152500	0.589	1.172	0.638	0.482
Tan	7	3090001	3090300	0.160	0.588	0.321	0.031
Tan	7	3140401	3140700	0.586	0.912	0.325	0.421
Tan	7	3140701	3141000	0.398	0.680	0.346	0.304
Tan	7	3273601	3273900	0.294	0.562	0.160	0.228
Tan	7	4077601	4077900	0.292	0.491	0.321	0.193
Tan	7	4136701	4137000	0.294	0.654	0.263	0.248
Tan	7	4236901	4237200	0.292	0.751	0.419	0.279
Tan	7	4462801	4463100	0.586	1.004	0.691	0.806
Tan	7	4574101	4574400	0.826	1.422	0.814	0.669
Tan	7	4594201	4594500	0.350	0.773	0.214	0.193
Tan	7	4644601	4644900	0.505	0.772	0.424	0.639
Tan	7	4696501	4696800	0.294	0.796	0.330	0.187
Tan	7	4928701	4929000	0.158	0.629	0.214	0.142
Tan	7	5086201	5086500	0.555	0.094	0.374	0.720
Tan	7	5090101	5090400	7.742	5.234	7.181	8.777
Tan	7	5120701	5121000	0.184	0.822	0.272	0.142
Tan	7	5482201	5482500	0.160	0.631	0.296	0.315
Tan	7	5505301	5505600	3.167	4.806	3.549	2.992
Tan	7	5577901	5578200	0.694	1.732	1.016	1.222
Tan	7	5759701	5760000	0.187	0.444	0.160	0.228
Tan	7	5829901	5830200	0.106	0.395	0.111	0.056
Tan	7	5868601	5868900	0.984	1.148	0.374	0.720
Tan	7	6295801	6296100	0.319	0.562	0.317	0.304
Tan	7	6455701	6456000	0.186	0.517	0.185	0.101
Tan	7	6526201	6526500	2.773	4.010	2.813	2.449
Tan	7	6552301	6552600	5.185	4.428	3.620	5.380
Tan	7	6637501	6637800	0.531	0.373	0.132	0.522
Tan	7	6672901	6673200	0.453	0.798	0.346	0.522
Tan	7	6868201	6868500	0.398	0.843	0.432	0.533

Tan	7	7083901	7084200	0.189	0.493	0.103	0.162
Tan	7	7212601	7212900	0.186	0.538	0.189	0.243
Tan	7	7222801	7223100	0.824	0.513	0.395	0.720
Tan	7	7284301	7284600	0.345	0.702	0.218	0.385
Tan	7	7309501	7309800	0.553	1.448	0.337	0.329
Tan	7	7491901	7492200	0.240	0.466	0.160	0.061
Tan	7	7648501	7648800	0.261	0.820	0.283	0.309
Tan	7	7785301	7785600	0.211	0.513	0.346	0.106
Tan	7	7822501	7822800	0.560	1.422	0.539	0.472
Tan	7	7902001	7902300	1.544	1.845	1.148	1.181
Tan	7	7979101	7979400	0.237	0.515	0.160	0.193
Tan	7	8193301	8193600	0.079	0.511	0.127	0.167
Tan	7	8242201	8242500	0.398	0.725	0.424	0.274
Tan	7	8379301	8379600	0.158	0.631	0.210	0.243
Tan	7	8394601	8394900	0.213	0.635	0.234	0.203
Tan	7	8977201	8977500	1.466	1.777	0.604	2.236
Tan	7	9266401	9266700	0.319	0.539	0.370	0.335
Tan	7	9315901	9316200	0.294	0.701	0.214	0.421
Tan	7	9402301	9402600	0.344	0.961	0.457	0.461
Tan	7	9403801	9404100	0.536	1.498	0.312	0.806
Tan	7	9404101	9404400	0.611	1.826	0.477	0.862
Tan	7	9411301	9411600	0.160	0.421	0.633	0.553
Tan	7	9529501	9529800	0.345	0.704	0.321	0.259
Tan	7	9800401	9800700	0.263	0.586	0.263	0.248
Tan	7	9880801	9881100	0.658	2.113	0.756	0.887
Tan	7	9883501	9883800	0.160	0.493	0.239	0.254
Tan	7	9999301	9999600	0.184	0.603	0.165	0.137
Tan	7	10272901	10273200	0.266	0.723	0.301	0.304
Tan	7	10482001	10482300	0.110	0.493	0.054	0.142
Tan	7	10922101	10922400	0.479	1.169	0.296	0.578
Tan	7	11697601	11697900	0.266	0.538	0.156	0.335
Tan	7	11765101	11765400	1.013	0.423	0.901	0.781
Tan	7	11954701	11955000	0.108	0.584	0.370	0.304
Tan	7	12073801	12074100	1.039	1.590	0.785	1.070
Tan	7	12256501	12256800	0.666	1.912	0.760	0.584
Tan	7	12256801	12257100	1.784	3.306	2.053	1.739
Tan	7	12334801	12335100	9.612	3.863	8.911	9.406
Tan	7	12416701	12417000	0.455	0.955	0.370	0.608
Tan	7	12437701	12438000	0.131	0.796	0.346	0.254
Tan	7	12461101	12461400	0.158	0.442	0.132	0.106
Tan	7	12513001	12513300	0.158	0.560	0.176	0.223
Tan	7	12627901	12628200	1.142	0.886	0.399	0.639
Tan	7	12793801	12794100	1.092	2.575	1.168	1.205
Tan	7	13176001	13176300	0.160	0.446	0.239	0.081
Tan	7	13406401	13406700	0.079	0.420	0.185	0.274
Tan	7	13431001	13431300	0.290	0.631	0.107	0.228
Tan	7	13520701	13521000	0.318	0.798	0.448	0.553
Tan	7	13521001	13521300	0.345	0.676	0.366	0.254
Tan	7	13613401	13613700	0.397	1.009	0.473	0.309
Tan	7	13684201	13684500	8.351	7.065	8.973	9.334
Tan	7	13917901	13918200	0.081	0.395	0.136	0.086
Tan	7	14244601	14244900	0.371	0.770	0.308	0.187
Tan	7	14374801	14375100	0.424	0.747	0.247	0.254
Tan	7	14396101	14396400	1.705	0.656	1.567	2.184
Tan	7	14429401	14429700	0.900	1.431	0.415	0.938
Tan	7	14472301	14472600	0.213	0.727	0.419	0.446
Tan	7	14520901	14521200	0.134	0.423	0.107	0.081
Tan	7	14821501	14821800	0.294	0.916	0.210	0.258
Tan	7	14822101	14822400	1.144	1.948	1.329	1.166
Tan	7	14841001	14841300	0.290	0.513	0.210	0.193
Tan	7	14921701	14922000	0.319	0.583	0.263	0.268
Tan	7	15309901	15310200	2.160	1.360	2.044	2.367
Tan	7	15315001	15315300	1.942	1.034	1.608	1.602
Tan	7	15332401	15332700	0.186	0.635	0.049	0.167
Tan	7	15399601	15399900	0.373	0.631	0.218	0.137
Tan	7	15454501	15454800	0.081	0.257	0.477	0.106
Tan	7	15949501	15949800	0.186	0.586	0.214	0.223

Tan	7	16040101	16040400	2.823	1.940	1.308	1.131
Tan	7	16154401	16154700	0.534	0.963	0.341	0.340
Tan	7	16244701	16245000	0.878	1.290	0.403	0.740
Tan	7	16336201	16336500	0.423	0.798	0.424	0.436
Tan	7	16373401	16373700	0.134	0.560	0.189	0.137
Tan	7	16494901	16495200	1.333	2.536	1.674	1.734
Tan	7	16536301	16536600	0.532	0.283	0.453	0.441
Tan	7	16771801	16772100	0.186	0.536	0.136	0.213
Tan	7	16798501	16798800	0.187	0.725	0.477	0.243
Tan	7	16853701	16854000	0.347	0.631	0.239	0.162
Tan	7	16855201	16855500	0.160	0.539	0.185	0.355
Tan	7	16865401	16865700	0.290	0.515	0.292	0.162
Tan	7	16909801	16910100	3.139	1.839	2.802	3.381
Tan	7	16994701	16995000	0.453	0.751	0.403	0.385
Tan	7	17012701	17013000	0.242	0.659	0.239	0.173
Tan	7	17050201	17050500	0.189	0.420	0.160	0.167
Tan	7	17064901	17065200	0.773	1.478	0.745	0.953
Tan	7	17147401	17147700	0.237	0.541	0.160	0.193
Tan	7	17439601	17439900	0.955	1.352	0.979	0.715
Tan	7	17490901	17491200	0.692	1.077	0.515	0.628
Tan	7	17561701	17562000	0.000	0.371	0.584	0.137
Tan	7	18063301	18063600	0.848	0.444	0.802	0.801
Tan	7	18763201	18763500	0.108	0.444	0.181	0.193
Tan	7	19566601	19566900	0.187	1.176	0.444	0.263
Tan	7	19721101	19721400	0.027	0.425	0.000	0.000
Tan	7	19908301	19908600	0.106	0.517	0.296	0.137
Tan	7	20160901	20161200	0.242	0.980	0.218	0.319
Tan	7	20242501	20242800	0.611	1.283	0.531	0.598
Tan	7	20324701	20325000	1.702	2.130	1.217	1.688
Tan	7	20396101	20396400	3.294	4.046	3.384	3.215
Tan	7	20400301	20400600	1.037	1.403	0.765	0.740
Tan	7	20430001	20430300	1.810	1.070	2.406	1.592
Tan	7	20485201	20485500	0.773	1.354	0.765	0.938
Tan	7	20577001	20577300	1.010	0.935	0.691	1.136
Tan	7	20925301	20925600	0.292	0.654	0.399	0.441
Tan	7	20938801	20939100	0.319	0.511	0.321	0.913
Tan	7	20981701	20982000	1.642	2.337	1.444	0.918
Tan	7	20982001	20982300	1.381	2.057	1.023	0.837
Tan	7	21032101	21032400	0.215	0.493	0.127	0.137
Tan	7	21263401	21263700	0.158	0.611	0.321	0.173
Tan	7	21377401	21377700	1.008	1.401	0.633	1.024
Tan	7	21399901	21400200	0.213	0.444	0.136	0.106
Tan	7	21400201	21400500	0.265	0.491	0.288	0.081
Tan	7	21554401	21554700	0.424	0.963	0.321	0.446
Tan	7	21867301	21867600	2.552	1.496	2.000	1.953
Tan	7	21880501	21880800	1.674	0.935	1.197	1.222
Tan	7	21897301	21897600	0.079	0.470	0.263	0.112
Tan	7	22457401	22457700	0.797	0.328	0.749	0.634
Tan	7	22531801	22532100	0.668	1.861	0.551	0.796
Tan	7	22934101	22934400	1.007	1.734	1.177	0.913
Tan	7	23237401	23237700	0.371	0.751	0.506	0.411
Tan	7	23274001	23274300	0.158	0.727	0.181	0.198
Tan	7	23442601	23442900	0.268	0.583	0.189	0.142
Tan	7	23865601	23865900	0.105	0.468	0.111	0.106
Tan	7	23901001	23901300	0.613	0.350	0.185	0.223
Tan	7	24123301	24123600	2.842	1.783	2.535	2.611
Tan	7	24286501	24286800	0.374	0.912	0.584	0.405
Tan	7	24330901	24331200	0.136	0.564	0.243	0.162
Tan	7	25276501	25276800	1.997	0.978	1.995	1.607
Tan	7	25650601	25650900	0.213	0.609	0.156	0.056
Tan	7	25709701	25710000	0.131	0.584	0.107	0.228
Tan	7	25737901	25738200	0.266	0.468	0.662	0.218
Tan	7	26024101	26024400	0.266	0.729	0.370	0.142
Tan	7	26686801	26687100	1.274	1.967	1.201	1.121
Tan	7	26763901	26764200	0.106	0.446	0.087	0.092
Tan	7	27719101	27719400	1.195	1.637	1.295	1.323
Tan	7	27848101	27848400	0.160	0.494	0.156	0.112

Tan	7	28001101	28001400	0.611	1.171	0.667	0.457
Tan	7	28625701	28626000	0.371	0.727	0.321	0.360
Tan	7	29681701	29682000	0.345	0.701	0.346	0.157
Tan	7	30726901	30727200	0.453	0.699	0.428	0.355
Tan	7	31094101	31094400	0.319	0.517	0.346	0.137
Tan	7	31150501	31150800	0.239	0.536	0.370	0.248
Tan	7	31502401	31502700	0.240	0.680	0.312	0.299
Tan	7	32290501	32290800	0.477	0.678	0.317	0.472
Tan	7	32314201	32314500	1.221	1.659	1.266	2.094
Tan	7	32392201	32392500	0.373	0.633	0.267	0.268
Tan	7	32687701	32688000	0.611	0.395	0.267	0.664
Tan	7	32738101	32738400	0.134	0.701	0.214	0.268
Tan	7	32788501	32788800	0.795	1.240	0.604	0.918
Tan	7	33114301	33114600	0.931	1.732	1.094	0.923
Tan	7	33202801	33203100	0.239	0.466	0.296	0.223
Tan	7	33306901	33307200	0.932	1.358	0.831	0.542
Tan	7	33564301	33564600	0.536	1.382	0.769	1.050
Tan	7	33901501	33901800	0.507	1.169	0.651	0.573
Tan	7	34011601	34011900	1.463	1.751	1.028	1.303
Tan	7	34283101	34283400	1.224	0.556	1.032	0.887
Tan	7	34782601	34782900	0.318	0.538	0.218	0.167
Tan	7	34791601	34791900	0.448	1.073	0.564	0.547
Tan	7	34856101	34856400	0.211	0.468	0.132	0.248
Tan	7	35025001	35025300	0.266	0.539	0.267	0.329
Tan	7	35058301	35058600	1.195	0.607	1.099	1.253
Tan	7	35316901	35317200	0.106	0.543	0.127	0.142
Tan	7	35959501	35959800	0.081	0.420	0.000	0.167
Tan	7	36966301	36966600	0.263	0.584	0.263	0.173
Tan	7	37122901	37123200	0.134	0.538	0.218	0.167
Tan	7	39155701	39156000	0.558	0.326	0.695	0.583
Tan	7	39674101	39674400	2.099	2.410	1.703	2.074
Tan	7	39702901	39703200	0.186	0.848	0.140	0.086
Tan	7	40143901	40144200	0.000	0.466	0.210	0.106
Tan	7	40319101	40319400	0.505	1.259	0.600	0.350
Tan	7	40743901	40744200	0.211	0.517	0.263	0.254
Tan	7	41134801	41135100	1.544	1.332	1.772	1.024
Tan	7	41146201	41146500	3.107	2.079	0.952	3.012
Tan	7	41253001	41253300	0.215	0.513	0.160	0.223
Tan	7	41418601	41418900	0.452	0.654	0.453	0.441
Tan	7	42061201	42061500	0.055	0.468	0.239	0.198
Tan	7	42209101	42209400	0.613	0.843	1.353	0.421
Tan	7	43615501	43615800	0.529	1.577	0.419	0.690
Tan	7	43615801	43616100	0.263	0.800	0.127	0.213
Tan	7	43641901	43642200	0.263	0.583	0.189	0.193
Tan	7	43863601	43863900	0.611	0.959	0.720	0.380
Tan	7	44352001	44352300	0.186	0.534	0.189	0.218
Tan	7	44935801	44936100	0.134	0.519	0.029	0.112
Tan	7	45022801	45023100	1.139	1.659	0.687	1.029
Tan	7	45081301	45081600	0.027	0.584	0.210	0.248
Tan	7	45097201	45097500	0.132	0.446	0.103	0.132
Tan	7	45133501	45133800	0.874	2.180	0.986	1.050
Tan	7	45198301	45198600	0.369	0.676	0.292	0.223
Tan	7	45219301	45219600	0.611	1.347	0.341	0.193
Tan	7	45219601	45219900	0.663	1.442	0.535	0.436
Tan	7	45510601	45510900	2.363	3.036	1.654	2.342
Tan	7	45532501	45532800	0.374	0.562	0.239	0.223
Tan	7	45796501	45796800	0.423	0.792	0.477	0.329
Tan	7	46203001	46203300	0.239	0.684	0.263	0.274
Tan	7	46271401	46271700	0.373	0.702	0.181	0.218
Tan	7	46322101	46322400	1.007	1.944	1.209	0.857
Tan	7	46639801	46640100	0.426	0.210	0.526	0.360
Tan	7	46976401	46976700	0.369	1.032	0.082	0.472
Tan	7	47352001	47352300	0.052	0.753	0.049	0.167
Tan	7	47365201	47365500	1.492	0.354	1.930	1.662
Tan	7	47365501	47365800	1.326	0.354	1.715	1.221
Tan	7	47547901	47548200	0.106	0.725	0.230	0.081
Tan	7	47673301	47673600	0.452	0.069	0.486	0.553

Tan	7	47882701	47883000	0.265	0.964	0.526	0.274
Tan	7	48016201	48016500	0.158	0.446	0.136	0.771
Tan	7	48045601	48045900	0.215	0.494	0.214	0.112
Tan	7	48213601	48213900	1.357	0.817	0.457	1.029
Tan	7	48606601	48606900	0.187	0.519	0.214	0.137
Tan	7	48892501	48892800	0.340	1.148	0.370	0.461
Tan	7	48892801	48893100	0.129	0.706	0.239	0.243
Tan	7	49271401	49271700	0.187	0.446	0.189	0.112
Tan	7	49591801	49592100	0.374	0.794	0.432	0.421
Tan	7	49601401	49601700	0.663	0.890	0.642	0.527
Tan	7	49676101	49676400	0.239	3.325	1.743	0.167
Tan	7	49676401	49676700	0.369	4.072	2.503	0.563
Tan	7	49765201	49765500	0.844	1.972	0.535	0.599
Tan	7	49765501	49765800	0.524	2.021	0.403	0.391
Tan	7	49775401	49775700	2.415	0.303	1.807	2.403
Tan	7	49775701	49776000	2.652	0.354	2.146	2.524
Tan	7	49802101	49802400	0.081	0.468	0.132	0.198
Tan	7	49848601	49848900	0.505	1.195	0.296	0.542
Tan	7	49848901	49849200	0.428	2.043	0.408	0.532
Tan	7	49906201	49906500	1.331	0.401	0.724	0.877
Tan	7	49971001	49971300	0.186	0.395	0.132	0.142
Tan	7	49985701	49986000	1.086	2.069	0.996	0.771
Tan	7	49991101	49991400	0.505	0.792	0.267	0.502
Tan	7	50107501	50107800	0.568	1.564	0.254	0.446
Tan	7	50130601	50130900	0.053	0.519	0.214	0.218
Tan	7	50328301	50328600	0.268	0.609	0.263	0.198
Tan	7	50488201	50488500	0.213	0.446	0.189	0.218
Tan	7	50773501	50773800	0.184	0.493	0.210	0.223
Tan	7	50857201	50857500	0.081	0.989	0.230	0.248
Tan	7	50979301	50979600	0.373	0.562	0.399	0.391
Tan	7	51171601	51171900	0.560	1.120	0.765	0.913
Tan	7	51654901	51655200	0.211	0.635	0.214	0.335
Tan	7	51715801	51716100	0.713	0.257	0.691	0.776
Tan	7	52115701	52116000	1.011	1.684	1.130	0.832
Tan	7	52302601	52302900	0.132	0.494	0.054	0.092
Tan	7	52303201	52303500	0.184	0.517	0.078	0.132
Tan	7	52555201	52555500	1.168	0.442	0.885	1.197
Tan	7	52596001	52596300	0.824	0.511	0.132	0.740
Tan	7	52607101	52607400	0.557	0.114	0.453	0.533
Tan	7	52654201	52654500	0.665	0.326	0.774	0.751
Tan	7	52703101	52703400	0.187	0.560	0.152	0.142
Tan	7	52941601	52941900	0.826	1.328	0.403	0.771
Tan	7	53175301	53175600	0.077	0.446	0.058	0.117
Tan	7	53364001	53364300	0.160	0.470	0.160	0.086
Tan	7	53917201	53917500	2.039	0.704	1.901	2.089
Tan	7	54117301	54117600	0.131	0.612	0.136	0.117
Tan	7	54374101	54374400	1.036	0.536	0.856	0.832
Tan	7	54584401	54584700	2.105	1.118	1.547	1.658
Tan	7	54613501	54613800	0.213	0.493	0.234	0.137
Tan	7	54684301	54684600	0.397	0.770	0.325	0.335
Tan	7	54898201	54898500	0.369	0.820	0.395	0.350
Tan	7	55084501	55084800	0.132	0.583	0.239	0.243
Tan	7	55084801	55085100	0.292	0.609	0.247	0.329
Tan	7	55148701	55149000	4.072	5.808	4.545	4.283
Tan	7	55299301	55299600	0.215	0.584	0.156	0.335
Tan	7	55664101	55664400	0.052	0.545	0.054	0.051
Tan	7	55862401	55862700	0.665	1.240	0.502	0.624
Tan	7	55904101	55904400	0.374	1.219	0.410	0.513
Tan	7	55947901	55948200	0.450	0.820	0.502	0.522
Tan	7	56038501	56038800	5.507	2.791	6.218	6.412
Tan	7	56197801	56198100	0.081	0.401	0.107	0.137
Tan	7	56230501	56230800	3.212	1.641	3.438	3.504
Tan	7	56230801	56231100	3.055	1.616	3.583	3.667
Tan	7	56465101	56465400	0.900	1.491	0.838	0.806
Tan	7	56511001	56511300	0.611	0.466	0.243	0.304
Tan	7	56702701	56703000	0.582	1.103	0.473	0.461
Tan	7	57107401	57107700	0.240	1.872	0.082	0.193

Tan	7	57107701	57108000	0.079	1.303	0.054	0.117
Tan	7	57126901	57127200	0.160	1.037	0.346	0.421
Tan	7	57324901	57325200	0.082	0.564	0.123	0.112
Tan	7	58346701	58347000	0.108	0.423	0.082	0.117
Tan	7	58389601	58389900	0.239	0.468	0.127	0.248
Tan	7	58712101	58712400	0.957	0.187	0.560	0.472
Tan	7	58858801	58859100	0.718	0.258	0.760	0.837
Tan	7	59111401	59111700	0.902	2.345	0.674	0.993
Tan	7	59541001	59541300	1.541	0.729	1.572	1.247
Tan	7	59628901	59629200	0.584	1.262	0.736	0.690
Tan	7	59727001	59727300	0.239	0.564	0.292	0.223
Tan	7	60171601	60171900	1.194	1.523	1.173	0.781
Tan	7	60447001	60447300	0.931	0.607	0.267	0.086
Tan	7	60506101	60506400	0.186	0.747	0.473	0.329
Tan	7	60706201	60706500	0.398	0.680	0.312	0.391
Tan	7	60740701	60741000	0.211	0.611	0.263	0.112
Tan	7	60936901	60937200	0.265	0.609	0.399	0.360
Tan	7	61004101	61004400	0.400	0.701	0.049	0.228
Tan	7	61059301	61059600	1.249	0.796	0.453	0.558
Tan	7	61186801	61187100	0.292	0.515	0.374	0.335
Tan	7	61348501	61348800	0.213	0.442	0.243	0.086
Tan	7	61540201	61540500	1.734	2.806	2.085	1.770
Tan	7	61713001	61713300	0.452	0.112	0.481	0.558
Tan	7	61743301	61743600	0.771	0.298	0.823	0.690
Tan	7	61951801	61952100	0.289	0.837	0.165	0.355
Tan	7	61992901	61993200	1.328	0.772	1.197	1.116
Tan	7	62013601	62013900	0.266	1.051	0.564	0.466
Tan	7	62130301	62130600	0.131	0.584	0.098	0.086
Tan	7	62130601	62130900	0.079	0.628	0.078	0.112
Tan	7	62209201	62209500	0.186	0.751	0.225	0.198
Tan	7	62951401	62951700	2.160	2.905	1.810	2.043
Tan	7	63334201	63334500	0.211	0.588	0.082	0.157
Tan	7	63471301	63471600	0.744	1.124	0.753	0.771
Tan	7	63564001	63564300	0.347	0.607	0.243	0.218
Tan	7	63817801	63818100	1.116	0.822	0.555	0.335
Tan	7	63846001	63846300	0.505	0.725	0.473	0.355
Tan	7	64197901	64198200	0.319	0.629	0.317	0.497
Tan	7	64505401	64505700	0.344	0.586	0.210	0.304
Tan	7	64515001	64515300	1.595	1.120	1.592	1.577
Tan	7	64742101	64742400	5.659	4.456	3.103	3.386
Tan	7	64842001	64842300	0.398	0.745	0.341	0.391
Tan	7	65150101	65150400	0.131	0.727	0.082	0.081
Tan	7	65180701	65181000	0.292	0.607	0.243	0.228
Tan	7	65409601	65409900	1.704	3.521	1.991	1.663
Tan	7	65416201	65416500	0.450	0.980	0.502	0.690
Tan	7	65428501	65428800	2.846	1.405	2.413	2.738
Tan	7	65546701	65547000	0.373	0.629	0.321	0.254
Tan	7	65719801	65720100	0.373	0.234	0.535	0.715
Tan	7	66027301	66027600	0.426	0.657	0.448	0.943
Tan	7	66492601	66492900	0.158	0.702	0.223	0.279
Tan	7	66564301	66564600	0.371	0.890	0.506	0.517
Tan	7	67334101	67334400	0.108	0.420	0.132	0.056
Tan	7	67821301	67821600	0.952	1.639	0.889	0.720
Tan	7	68539201	68539500	0.716	0.277	0.613	0.639
Tan	7	68620801	68621100	0.503	0.908	0.156	0.284
Tan	7	68674501	68674800	0.105	0.470	0.189	0.142
Tan	7	68713501	68713800	0.186	0.470	0.267	0.193
Tan	7	69405301	69405600	2.741	3.924	2.642	3.386
Tan	7	69484201	69484500	0.715	1.099	0.588	0.578
Tan	7	69681901	69682200	0.239	0.423	0.025	0.167
Tan	7	70083901	70084200	0.400	1.030	0.432	0.527
Tan	7	70395301	70395600	0.158	0.584	0.156	0.092
Tan	7	70418401	70418700	1.247	1.984	1.144	1.101
Tan	7	70418701	70419000	1.037	1.684	0.662	0.954
Tan	7	70454701	70455000	0.424	0.656	0.234	0.248
Tan	7	70566901	70567200	0.789	2.071	0.823	0.832
Tan	7	70632601	70632900	0.158	0.637	0.078	0.223

Tan	7	70632901	70633200	0.184	0.891	0.210	0.309
Tan	7	70653001	70653300	0.215	0.794	0.140	0.350
Tan	7	71306401	71306700	0.213	0.491	0.267	0.051
Tan	7	71691601	71691900	0.429	1.403	0.366	0.355
Tan	7	71691901	71692200	0.266	0.794	0.301	0.162
Tan	7	71692801	71693100	0.240	0.466	0.214	0.304
Tan	7	72183601	72183900	0.187	0.420	0.214	0.137
Tan	7	72388201	72388500	0.481	0.964	0.140	0.056
Tan	7	72833101	72833400	0.268	0.848	0.267	0.294
Tan	7	72833401	72833700	0.586	1.146	0.267	0.436
Tan	7	72865201	72865500	0.132	0.586	0.189	0.329
Tan	7	72960301	72960600	0.106	0.470	0.181	0.056
Tan	7	73329301	73329600	0.294	0.588	0.025	0.274
Tan	7	73499701	73500000	0.690	0.938	0.448	0.639
Tan	7	73710001	73710300	0.052	0.463	0.054	0.000
Tan	7	73818901	73819200	0.665	0.206	0.711	0.639
Tan	7	74107201	74107500	1.570	2.180	1.612	1.546
Tan	7	74726701	74727000	0.426	0.609	0.107	0.254
Tan	7	74880901	74881200	0.269	0.654	0.259	0.279
Tan	7	75092401	75092700	0.800	1.616	1.012	0.943
Tan	7	75405901	75406200	0.292	1.079	0.667	0.806
Tan	7	76240801	76241100	0.853	1.144	0.481	0.553
Tan	7	76303201	76303500	0.586	1.172	0.609	0.623
Tan	7	76388401	76388700	0.426	0.867	0.428	0.268
Tan	7	76670701	76671000	0.211	0.562	0.210	0.112
Tan	7	76899001	76899300	0.131	0.517	0.239	0.223
Tan	7	77203801	77204100	0.079	0.466	0.239	0.142
Tan	7	77249101	77249400	0.371	0.792	0.288	0.218
Tan	7	77265901	77266200	0.263	0.749	0.259	0.238
Tan	7	77345701	77346000	0.720	1.427	0.584	0.669
Tan	7	77485501	77485800	1.412	0.536	1.090	1.024
Tan	7	77554201	77554500	0.477	0.208	0.560	0.745
Tan	7	77578801	77579100	1.674	2.752	1.322	2.069
Tan	7	77951401	77951700	0.605	1.216	0.506	0.547
Tan	7	78183901	78184200	2.950	4.077	0.568	2.028
Tan	7	78200701	78201000	1.631	3.369	1.395	1.912
Tan	7	78202801	78203100	0.584	0.352	0.584	0.806
Tan	7	78300901	78301200	0.186	0.539	0.160	0.106
Tan	7	78336001	78336300	0.294	0.635	0.239	0.274
Tan	7	78572401	78572700	1.252	0.517	1.721	2.221
Tan	7	78674701	78675000	0.240	1.088	0.185	0.254
Tan	7	78892201	78892500	0.294	0.656	0.366	0.198
Tan	7	79171801	79172100	0.292	0.633	0.292	0.309
Tan	7	79354201	79354500	1.513	1.824	0.502	1.273
Tan	7	79432501	79432800	0.161	0.470	0.107	0.198
Tan	7	80006401	80006700	1.278	0.702	0.457	1.197
Tan	7	80389501	80389800	0.345	0.940	0.296	0.452
Tan	7	80993701	80994000	0.903	1.360	2.069	1.029
Tan	7	81149101	81149400	0.263	0.873	0.292	0.304
Tan	7	81426301	81426600	1.226	1.991	0.444	0.821
Tan	7	81426601	81426900	1.042	2.227	0.346	0.628
Tan	7	81479401	81479700	0.398	0.796	0.399	0.401
Tan	7	81524101	81524400	0.160	0.444	0.288	0.248
Tan	7	81601501	81601800	0.828	1.313	0.486	0.776
Tan	7	81615901	81616200	1.118	1.399	0.794	0.988
Tan	7	81960601	81960900	0.345	0.586	0.214	0.299
Tan	7	82151401	82151700	0.158	0.536	0.136	0.335
Tan	7	82527301	82527600	2.213	3.225	2.100	1.926
Tan	7	82625401	82625700	0.694	1.167	0.653	0.715
Tan	7	82847101	82847400	0.426	0.629	0.292	0.309
Tan	7	82871101	82871400	2.763	2.452	2.234	2.794
Tan	7	83280001	83280300	0.735	1.611	0.716	0.731
Tan	7	83355301	83355600	0.505	1.071	0.539	0.563
Tan	7	83458501	83458800	0.639	1.363	0.515	0.690
Tan	7	83557801	83558100	0.160	0.611	0.288	0.193
Tan	7	83562301	83562600	2.660	4.626	3.163	2.945
Tan	7	83674201	83674500	0.077	0.446	0.082	0.101

Tan	7	83705101	83705400	0.852	1.122	0.613	0.740
Tan	7	83967001	83967300	3.821	5.330	3.663	3.792
Tan	7	84053401	84053700	1.860	1.633	1.226	1.379
Tan	7	84182701	84183000	9.485	7.820	10.564	8.979
Tan	7	84197701	84198000	0.903	1.238	0.604	0.999
Tan	7	84227101	84227400	0.108	0.564	0.078	0.025
Tan	7	84393601	84393900	2.396	3.163	1.982	2.205
Tan	7	84407401	84407700	0.853	1.431	0.950	0.882
Tan	7	84524101	84524400	0.318	0.468	0.346	0.223
Tan	7	84547801	84548100	0.213	0.539	0.111	0.218
Tan	7	84792901	84793200	0.560	0.916	0.374	0.416
Tan	7	84837601	84837900	2.052	0.905	4.149	1.906
Tan	7	84952501	84952800	0.082	0.538	0.127	0.092
Tan	7	85180501	85180800	0.398	0.515	0.185	0.304
Tan	7	85257001	85257300	0.213	0.534	0.082	0.223
Tan	7	85519801	85520100	0.397	0.772	0.481	0.553
Tan	7	85674301	85674600	0.345	0.564	0.156	0.106
Tan	7	85762201	85762500	0.239	0.539	0.136	0.198
Tan	7	85821301	85821600	0.239	0.493	0.239	0.309
Tan	7	85821601	85821900	0.292	0.444	0.136	0.193
Tan	7	86432701	86433000	1.092	1.963	1.222	1.197
Tan	7	86760301	86760600	0.908	1.618	0.983	0.841
Tan	7	86817301	86817600	0.213	0.837	0.296	0.360
Tan	7	87304501	87304800	0.718	0.210	0.477	0.416
Tan	7	87312001	87312300	0.079	0.611	0.127	0.142
Tan	7	87315301	87315600	0.290	0.846	0.276	0.299
Tan	7	87361801	87362100	0.666	2.504	0.729	0.827
Tan	7	87590101	87590400	0.344	0.751	0.214	0.279
Tan	7	87590401	87590700	0.347	0.846	0.234	0.335
Tan	7	87880201	87880500	1.149	1.899	1.226	0.938
Tan	7	88436101	88436400	0.211	0.629	0.428	0.167
Tan	7	88492501	88492800	3.920	1.426	3.810	3.807
Tan	7	88801801	88802100	0.582	0.890	0.510	0.335
Tan	7	88895701	88896000	0.134	0.491	0.127	0.173
Tan	7	88982401	88982700	0.720	1.148	0.609	0.715
Tan	7	89144101	89144400	0.000	0.560	0.160	0.076
Tan	7	89636701	89637000	0.239	0.494	0.214	0.162
Tan	7	89703001	89703300	0.392	1.006	0.292	0.309
Tan	7	89831101	89831400	0.077	0.605	0.218	0.193
Tan	7	90163801	90164100	1.378	2.105	0.510	0.765
Tan	7	90451201	90451500	0.613	0.163	0.292	0.329
Tan	7	91170601	91170900	0.026	0.466	0.054	0.187
Tan	7	91740301	91740600	0.266	0.701	0.267	0.416
Tan	7	91801501	91801800	0.637	1.077	0.497	0.720
Tan	7	92420401	92420700	0.106	0.635	0.156	0.112
Tan	7	92942101	92942400	0.373	0.680	0.346	0.340
Tan	7	92945401	92945700	0.213	0.423	0.107	0.137
Tan	7	93620401	93620700	0.373	0.701	0.272	0.299
Tan	7	94115101	94115400	0.294	0.817	0.444	0.502
Tan	7	94467601	94467900	0.532	0.839	0.346	0.274
Tan	7	94472701	94473000	0.186	0.470	0.156	0.223
Tan	7	94618801	94619100	0.081	0.371	0.078	0.137
Tan	7	95068501	95068800	0.313	1.054	0.288	0.314
Tan	7	95420101	95420400	0.213	0.843	0.502	0.355
Tan	7	95420401	95420700	0.105	1.081	0.317	0.345
Tan	7	95711401	95711700	0.213	0.727	0.223	0.228
Tan	7	95744701	95745000	0.156	0.511	0.111	0.025
Tan	7	95926201	95926500	0.426	0.910	0.390	0.492
Tan	7	96387601	96387900	0.106	1.056	0.263	0.558
Tan	7	96398401	96398700	0.052	0.465	0.160	0.081
Tan	7	96434101	96434400	1.521	0.258	1.197	1.384
Tan	7	96444901	96445200	0.636	1.330	0.702	0.715
Tan	7	96452401	96452700	0.190	0.605	0.103	0.056
Tan	7	96660601	96660900	0.636	2.049	0.691	0.745
Tan	7	96667801	96668100	0.479	1.861	0.802	0.801
Tan	7	96702301	96702600	1.010	1.852	1.173	1.065
Tan	7	96743401	96743700	1.781	3.903	2.095	1.353

Tan	7	96861001	96861300	0.452	0.305	0.448	0.167
Tan	7	96909001	96909300	0.689	1.352	0.243	0.547
Tan	7	97006201	97006500	0.321	0.837	0.428	0.441
Tan	7	97185001	97185300	1.491	1.143	0.667	1.647
Tan	7	97351801	97352100	0.532	1.354	0.363	0.618
Tan	7	97356901	97357200	0.215	0.611	0.107	0.268
Tan	7	97369501	97369800	0.026	0.635	0.156	0.126
Tan	7	97450801	97451100	0.268	0.706	0.082	0.162
Tan	7	97546201	97546500	0.079	0.468	0.370	0.274
Tan	7	97629901	97630200	2.073	3.031	1.906	2.144
Tan	7	97631101	97631400	1.705	1.214	1.826	2.221
Tan	7	97683301	97683600	10.543	6.511	10.961	12.279
Tan	7	97688401	97688700	2.504	3.576	2.227	2.556
Tan	7	97752901	97753200	5.937	8.121	4.988	5.632
Tan	7	97959001	97959300	1.444	2.418	1.168	1.242
Tan	7	97963501	97963800	0.349	0.837	0.165	0.441
Tan	7	97995301	97995600	0.799	0.496	1.592	1.491
Tan	7	98027401	98027700	1.197	0.421	0.983	0.927
Tan	7	98035801	98036100	2.578	6.366	1.288	1.618
Tan	7	98065501	98065800	0.821	1.476	0.444	0.187
Tan	7	98100301	98100600	0.774	1.285	0.934	0.938
Tan	7	98116801	98117100	0.452	0.631	0.424	0.385
Tan	7	98231101	98231400	1.436	2.128	1.661	1.541
Tan	7	98350201	98350500	7.898	6.379	6.741	7.681
Tan	7	98439001	98439300	4.625	3.250	3.868	4.741
Tan	7	98443201	98443500	2.155	1.658	0.972	1.035
Tan	7	98444101	98444400	5.138	2.896	4.425	4.650
Tan	7	98467201	98467500	4.681	3.152	3.665	4.487
Tan	7	98620501	98620800	0.213	0.446	0.029	0.254
Tan	7	98646001	98646300	0.239	0.538	0.054	0.137
Tan	7	98679601	98679900	1.149	2.173	2.653	1.024
Tan	7	98714101	98714400	9.239	3.782	6.105	7.246
Tan	7	98722801	98723100	0.750	2.263	0.560	0.759
Tan	7	98898001	98898300	1.142	1.738	0.740	1.105
Tan	7	98996101	98996400	0.692	1.026	0.346	0.466
Tan	7	99088201	99088500	0.398	0.303	0.156	0.497
Tan	7	99468601	99468900	0.189	1.332	0.426	0.223
Tan	7	99475501	99475800	0.527	1.309	0.642	0.740
Tan	7	99482101	99482400	3.515	1.132	2.555	2.880
Tan	7	99499201	99499500	1.221	1.757	0.687	1.181
Tan	7	99528301	99528600	0.160	0.446	0.185	0.051
Tan	7	99698701	99699000	0.589	0.210	0.716	0.664
Tan	7	99738901	99739200	5.190	6.033	5.290	4.478
Tan	7	99942301	99942600	5.729	2.212	5.162	5.136
Tan	8	175801	176100	0.373	1.442	0.390	0.837
Tan	8	200701	201000	0.239	0.538	0.374	0.218
Tan	8	216601	216900	1.032	1.807	0.959	0.603
Tan	8	216901	217200	0.852	1.405	0.729	0.553
Tan	8	222301	222600	0.266	0.421	0.609	0.223
Tan	8	381901	382200	2.623	0.442	2.674	3.853
Tan	8	386701	387000	0.186	2.122	0.593	0.674
Tan	8	1789201	1789500	0.400	0.607	0.214	0.309
Tan	8	1901701	1902000	0.369	1.422	0.292	0.365
Tan	8	2110801	2111100	0.263	0.560	0.243	0.167
Tan	8	2290801	2291100	0.160	0.420	0.078	0.132
Tan	8	2448001	2448300	1.224	1.991	1.177	1.004
Tan	8	2514601	2514900	0.637	0.843	0.103	0.472
Tan	8	2557501	2557800	1.675	5.040	1.673	2.089
Tan	8	2557801	2558100	0.634	1.731	0.428	0.907
Tan	8	2574301	2574600	0.374	2.128	0.742	0.811
Tan	8	2574601	2574900	0.268	1.296	0.359	0.350
Tan	8	2624101	2624400	1.726	2.624	1.739	1.592
Tan	8	2852401	2852700	1.678	2.513	1.280	1.303
Tan	8	3242101	3242400	0.345	0.976	0.305	0.324
Tan	8	3242401	3242700	0.321	0.935	0.379	0.182
Tan	8	3253801	3254100	0.156	1.710	0.256	0.254
Tan	8	3254101	3254400	0.373	2.789	0.622	0.486

Tan	8	3624901	3625200	0.799	0.607	0.769	0.365
Tan	8	3858001	3858300	0.292	0.775	0.283	0.304
Tan	8	4078801	4079100	0.426	0.680	0.189	0.228
Tan	8	4105501	4105800	0.106	0.511	0.239	0.304
Tan	8	4161301	4161600	0.395	0.963	1.657	0.274
Tan	8	4342501	4342800	0.131	0.468	0.210	0.776
Tan	8	5057701	5058000	1.833	0.719	1.982	2.134
Tan	8	5217601	5217900	0.213	0.446	0.584	0.801
Tan	8	5537401	5537700	0.505	1.077	0.695	0.553
Tan	8	5907601	5907900	0.106	0.468	0.243	0.329
Tan	8	5910601	5910900	0.240	0.629	0.288	0.187
Tan	8	5976901	5977200	0.132	0.448	0.136	0.081
Tan	8	6046501	6046800	0.450	0.768	0.453	0.279
Tan	8	6056701	6057000	2.844	2.570	2.842	3.503
Tan	8	6141901	6142200	1.060	1.521	1.037	1.044
Tan	8	6256801	6257100	0.184	0.538	0.127	0.193
Tan	8	6257101	6257400	0.318	0.790	0.288	0.391
Tan	8	6464401	6464700	0.529	0.957	0.370	0.329
Tan	8	6486301	6486600	0.532	0.234	0.321	0.553
Tan	8	6530401	6530700	0.053	0.420	0.189	0.274
Tan	8	6798601	6798900	2.162	3.279	1.288	1.537
Tan	8	6956101	6956400	0.505	0.890	0.054	0.583
Tan	8	7051501	7051800	0.160	0.684	0.181	0.187
Tan	8	7161301	7161600	0.134	0.493	0.243	0.223
Tan	8	7178701	7179000	0.160	0.702	0.448	0.446
Tan	8	7214401	7214700	0.457	0.957	0.448	0.385
Tan	8	7260901	7261200	0.690	1.401	0.704	0.426
Tan	8	7728001	7728300	0.313	1.011	0.234	0.173
Tan	8	7854301	7854600	0.027	0.395	0.107	0.142
Tan	8	7922401	7922700	0.295	0.047	0.687	0.299
Tan	8	7971901	7972200	0.105	0.468	0.218	0.198
Tan	8	8258701	8259000	0.689	1.053	0.457	0.583
Tan	8	8440801	8441100	0.239	0.444	0.181	0.198
Tan	8	8488501	8488800	5.265	3.536	5.608	5.445
Tan	8	8516101	8516400	0.505	0.257	0.424	0.690
Tan	8	8712001	8712300	0.421	0.845	0.132	0.218
Tan	8	9295201	9295500	0.668	1.126	0.506	0.634
Tan	8	9515401	9515700	0.263	0.957	0.390	0.218
Tan	8	9735001	9735300	0.318	0.562	0.185	0.299
Tan	8	9789901	9790200	0.131	0.423	0.107	0.051
Tan	8	10053301	10053600	0.240	0.654	0.259	0.167
Tan	8	10161001	10161300	0.108	0.493	0.082	0.187
Tan	8	10307101	10307400	0.718	1.056	0.424	0.527
Tan	8	10688401	10688700	0.213	0.541	0.185	0.254
Tan	8	10932301	10932600	0.237	0.633	0.156	0.122
Tan	8	11467801	11468100	0.079	0.584	0.025	0.207
Tan	8	11760001	11760300	0.797	1.124	0.667	0.781
Tan	8	11865001	11865300	0.852	0.137	0.584	0.913
Tan	8	11949901	11950200	2.991	1.512	3.344	3.681
Tan	8	12163201	12163500	1.063	0.725	1.601	1.262
Tan	8	12367801	12368100	1.357	0.910	0.457	1.374
Tan	8	12420601	12420900	0.187	0.607	0.267	0.416
Tan	8	12492301	12492600	0.106	0.444	0.132	0.025
Tan	8	12516601	12516900	1.034	2.259	1.217	1.445
Tan	8	12581401	12581700	0.237	0.633	0.296	0.243
Tan	8	12632701	12633000	0.321	0.586	0.160	0.117
Tan	8	12735001	12735300	0.744	1.719	0.629	0.679
Tan	8	13023601	13023900	1.536	3.064	1.518	1.537
Tan	8	13174201	13174500	0.474	1.034	0.267	0.274
Tan	8	13287601	13287900	3.139	2.055	3.219	3.123
Tan	8	13288801	13289100	0.373	0.232	0.082	0.522
Tan	8	13407901	13408200	1.303	0.770	1.703	1.562
Tan	8	13483201	13483500	0.374	0.792	0.263	0.162
Tan	8	14300701	14301000	0.318	0.798	0.185	0.396
Tan	8	14308201	14308500	0.211	0.605	0.025	0.106
Tan	8	14434801	14435100	0.397	0.959	0.437	0.527
Tan	8	14526001	14526300	0.081	0.493	0.082	0.031

Tan	8	14604301	14604600	0.213	0.564	0.267	0.198
Tan	8	15726901	15727200	1.958	3.450	1.679	2.362
Tan	8	15805501	15805800	0.318	0.701	0.243	0.329
Tan	8	15864001	15864300	0.239	0.863	0.448	0.411
Tan	8	16621801	16622100	0.850	2.268	0.849	0.863
Tan	8	16622101	16622400	0.371	1.618	0.575	0.142
Tan	8	16624201	16624500	0.266	0.444	0.243	0.137
Tan	8	17073001	17073300	0.213	0.730	0.049	0.274
Tan	8	17290501	17290800	0.082	0.466	0.136	0.086
Tan	8	17689801	17690100	0.347	0.704	0.374	0.248
Tan	8	17713201	17713500	0.081	0.425	0.025	0.061
Tan	8	18381601	18381900	1.487	1.914	1.382	1.471
Tan	8	19238101	19238400	1.357	1.911	0.872	1.451
Tan	8	19492801	19493100	0.131	0.560	0.049	0.112
Tan	8	20032201	20032500	0.558	0.187	0.515	0.893
Tan	8	20505901	20506200	0.158	0.423	0.078	0.112
Tan	8	20539501	20539800	0.692	1.032	0.671	0.360
Tan	8	20655901	20656200	0.768	0.253	0.716	0.907
Tan	8	20659801	20660100	0.345	0.609	0.346	0.385
Tan	8	21157801	21158100	0.402	0.845	0.477	0.497
Tan	8	21375901	21376200	0.792	1.382	0.847	0.634
Tan	8	21433801	21434100	0.640	1.191	0.600	0.441
Tan	8	21709201	21709500	0.824	1.165	0.827	0.796
Tan	8	21766501	21766800	0.268	0.491	0.156	0.112
Tan	8	22263901	22264200	0.369	0.749	0.160	0.228
Tan	8	22618201	22618500	0.053	0.470	0.218	0.167
Tan	8	22731601	22731900	0.689	1.665	0.441	0.765
Tan	8	23071501	23071800	0.265	0.466	0.160	0.218
Tan	8	23261401	23261700	0.400	0.751	0.395	0.355
Tan	8	24105001	24105300	0.292	0.444	0.103	0.142
Tan	8	24651901	24652200	0.026	0.466	0.029	0.157
Tan	8	25054201	25054500	1.518	1.098	0.802	1.622
Tan	8	25103101	25103400	0.374	0.701	0.214	0.259
Tan	8	25339801	25340100	0.210	0.893	0.301	0.259
Tan	8	25394101	25394400	0.265	0.515	0.156	0.162
Tan	8	25761601	25761900	0.106	0.556	0.107	0.137
Tan	8	25842001	25842300	1.010	1.169	0.691	0.862
Tan	8	25889401	25889700	0.663	1.193	0.486	0.695
Tan	8	25894501	25894800	0.853	1.101	0.531	0.502
Tan	8	25995601	25995900	0.881	1.452	0.564	0.553
Tan	8	26237401	26237700	0.134	0.371	0.132	0.086
Tan	8	26663401	26663700	1.539	2.130	1.123	1.359
Tan	8	27272101	27272400	0.000	0.257	0.424	0.466
Tan	8	27272401	27272700	0.026	0.279	0.531	0.477
Tan	8	27336601	27336900	0.106	0.470	0.181	0.112
Tan	8	27786901	27787200	0.134	0.401	0.078	0.112
Tan	8	28051801	28052100	0.611	1.127	0.308	0.578
Tan	8	28342501	28342800	2.921	1.912	2.370	3.123
Tan	8	28394401	28394700	0.136	0.519	0.160	0.137
Tan	8	28745701	28746000	0.529	0.987	0.448	0.385
Tan	8	28756801	28757100	0.265	0.657	0.156	0.248
Tan	8	28778701	28779000	0.240	0.607	0.243	0.340
Tan	8	29559001	29559300	0.778	1.798	0.622	0.933
Tan	8	29985301	29985600	0.292	0.468	0.321	0.218
Tan	8	30036301	30036600	0.292	0.538	0.272	0.243
Tan	8	30069001	30069300	0.132	0.444	0.317	0.056
Tan	8	30336301	30336600	1.149	2.021	1.066	0.731
Tan	8	30503401	30503700	0.582	2.867	0.813	0.624
Tan	8	30786601	30786900	0.239	0.470	0.234	0.167
Tan	8	30881401	30881700	0.239	0.468	0.082	0.228
Tan	8	31010401	31010700	0.134	0.583	0.107	0.324
Tan	8	31437301	31437600	0.292	0.753	0.288	0.223
Tan	8	31526101	31526400	0.373	0.725	0.292	0.223
Tan	8	31698601	31698900	2.362	1.633	2.847	2.363
Tan	8	31968301	31968600	0.823	1.547	0.756	0.887
Tan	8	32489401	32489700	0.318	0.845	0.354	0.385
Tan	8	33071101	33071400	0.269	1.188	0.490	0.400

Tan	8	33461701	33462000	0.666	1.431	0.896	0.492
Tan	8	34643101	34643400	0.132	0.466	0.111	0.061
Tan	8	35374501	35374800	0.053	0.489	0.160	0.274
Tan	8	36326701	36327000	0.106	0.680	0.243	0.365
Tan	8	36668101	36668400	0.424	0.702	0.341	0.497
Tan	8	36691801	36692100	0.239	1.124	0.156	0.279
Tan	8	36826201	36826500	1.594	2.176	1.893	1.744
Tan	8	36976801	36977100	0.371	0.942	0.346	0.360
Tan	8	37019401	37019700	0.531	1.266	0.678	0.695
Tan	8	37254301	37254600	0.268	0.564	0.243	0.279
Tan	8	37689901	37690200	1.620	2.249	1.567	1.593
Tan	8	37931701	37932000	0.186	1.148	0.446	0.284
Tan	8	38482201	38482500	0.132	0.493	0.111	0.248
Tan	8	38886001	38886300	0.239	0.420	0.292	0.248
Tan	8	38927701	38928000	0.266	0.491	0.263	0.167
Tan	8	39221401	39221700	0.587	1.099	0.653	0.522
Tan	8	39615301	39615600	0.055	0.423	0.107	0.117
Tan	8	39651901	39652200	0.374	0.914	0.321	0.452
Tan	8	40103101	40103400	0.616	1.262	0.515	0.466
Tan	8	40998001	40998300	0.319	0.163	0.370	0.497
Tan	8	42466501	42466800	0.507	0.187	0.370	0.446
Tan	8	42762301	42762600	0.160	0.680	0.370	0.223
Tan	8	44666101	44666400	0.421	0.794	0.424	0.360
Tan	8	46048801	46049100	0.186	0.448	0.078	0.137
Tan	8	46966201	46966500	0.373	0.963	0.247	0.254
Tan	8	47213401	47213700	0.318	0.564	0.263	0.223
Tan	8	47303101	47303400	0.426	0.704	0.214	0.248
Tan	8	47611201	47611500	0.692	1.326	0.461	0.659
Tan	8	47722501	47722800	0.345	0.562	0.292	0.254
Tan	8	47855701	47856000	0.182	0.564	0.136	0.167
Tan	8	48078301	48078600	0.239	0.773	0.419	0.380
Tan	8	48086101	48086400	4.627	4.418	4.175	3.808
Tan	8	48156601	48156900	0.292	0.588	0.263	0.223
Tan	8	48188701	48189000	0.371	0.607	0.424	0.304
Tan	8	48285901	48286200	0.424	0.983	0.502	0.173
Tan	8	48461701	48462000	0.636	0.912	0.267	0.497
Tan	8	49003801	49004100	0.026	0.470	0.107	0.198
Tan	8	49101901	49102200	0.903	1.476	0.789	0.816
Tan	8	49115701	49116000	0.476	0.820	0.267	0.416
Tan	8	49634101	49634400	0.876	1.236	1.436	1.633
Tan	8	49738801	49739100	1.089	1.571	0.419	1.019
Tan	8	49781401	49781700	1.379	2.296	1.242	1.647
Tan	8	50821201	50821500	0.132	0.444	0.214	0.243
Tan	8	52225801	52226100	0.639	0.375	0.662	0.497
Tan	8	52268101	52268400	0.398	0.725	0.379	0.385
Tan	8	52377001	52377300	0.266	0.706	0.296	0.248
Tan	8	53163301	53163600	0.106	0.493	0.078	0.086
Tan	8	53251801	53252100	0.398	0.701	0.366	0.329
Tan	8	53280901	53281200	0.636	1.143	0.263	0.385
Tan	8	53281201	53281500	0.239	0.511	0.054	0.162
Tan	8	53537401	53537700	0.079	0.701	0.239	0.289
Tan	8	54520201	54520500	1.353	2.058	1.534	1.060
Tan	8	55020601	55020900	0.560	0.798	0.317	0.441
Tan	8	56218201	56218500	0.027	0.446	0.029	0.198
Tan	8	57060901	57061200	0.955	0.517	1.226	0.923
Tan	8	57107101	57107400	0.636	1.216	0.856	1.465
Tan	8	57627901	57628200	5.935	5.183	6.799	6.318
Tan	8	57850801	57851100	1.491	0.841	1.300	1.541
Tan	8	57865801	57866100	0.211	0.515	0.214	0.385
Tan	8	59306701	59307000	0.663	1.103	0.613	0.436
Tan	8	59597701	59598000	0.108	0.468	0.025	0.076
Tan	8	59941201	59941500	0.108	0.442	0.107	0.162
Tan	8	60272101	60272400	0.502	0.843	0.481	0.497
Tan	8	60498001	60498300	0.239	0.659	0.234	0.162
Tan	8	60674101	60674400	0.450	0.701	0.292	0.527
Tan	8	60761701	60762000	0.160	0.539	0.234	0.142
Tan	8	60816301	60816600	0.371	0.092	0.510	0.669

Tan	8	61545601	61545900	2.131	2.735	1.313	1.633
Tan	8	61634701	61635000	0.903	0.236	0.838	0.841
Tan	8	61716601	61716900	0.371	0.768	0.127	0.112
Tan	8	62093701	62094000	0.429	1.487	0.497	0.517
Tan	8	62200201	62200500	0.213	0.328	0.107	0.522
Tan	8	63109801	63110100	0.213	0.511	0.078	0.000
Tan	8	63231301	63231600	0.321	0.794	0.160	0.380
Tan	8	63493801	63494100	1.140	1.517	1.086	1.055
Tan	8	63835501	63835800	0.268	0.725	0.292	0.355
Tan	8	63924601	63924900	0.421	1.172	0.633	0.654
Tan	8	64060801	64061100	0.211	0.468	0.107	0.173
Tan	8	64223701	64224000	0.819	1.382	0.852	0.756
Tan	8	66968101	66968400	0.186	0.768	0.194	0.112
Tan	8	67110601	67110900	0.211	0.491	0.160	0.274
Tan	8	67311001	67311300	0.876	0.680	0.292	0.887
Tan	8	67676401	67676700	0.769	1.261	0.856	0.405
Tan	8	68073901	68074200	0.597	1.970	0.247	0.218
Tan	8	68592901	68593200	0.108	0.468	0.156	0.173
Tan	8	68593201	68593500	0.187	0.493	0.214	0.254
Tan	8	68661601	68661900	0.213	0.448	0.054	0.193
Tan	8	68726701	68727000	0.053	0.395	0.132	0.162
Tan	8	68775601	68775900	0.105	0.609	0.263	0.391
Tan	8	68997001	68997300	0.160	0.790	0.272	0.411
Tan	8	69109201	69109500	0.371	1.590	0.234	0.772
Tan	8	69223501	69223800	0.428	1.259	0.831	0.846
Tan	8	69845401	69845700	0.349	0.800	0.160	0.142
Tan	8	70103101	70103400	0.505	0.397	0.292	0.162
Tan	8	70139101	70139400	0.077	0.371	0.078	0.081
Tan	8	70189201	70189500	0.642	0.232	0.691	0.639
Tan	8	70547101	70547400	0.923	2.444	1.015	0.816
Tan	8	70803301	70803600	0.131	1.167	0.239	0.243
Tan	8	70804201	70804500	1.274	1.639	1.052	0.553
Tan	8	71054701	71055000	0.666	1.099	0.662	1.354
Tan	8	71175901	71176200	0.237	0.493	0.185	0.223
Tan	8	71578201	71578500	1.115	1.760	1.066	1.050
Tan	8	71672701	71673000	0.398	0.725	0.424	0.324
Tan	8	72600001	72600300	0.189	0.566	0.132	0.086
Tan	8	72641401	72641700	1.436	0.957	0.629	0.213
Tan	8	72673801	72674100	0.450	0.890	0.481	0.370
Tan	8	73006801	73007100	0.294	0.745	0.424	0.112
Tan	8	73007101	73007400	0.553	1.167	0.395	0.370
Tan	8	73096201	73096500	0.953	1.703	0.925	0.821
Tan	8	74074801	74075100	0.589	1.240	0.461	0.659
Tan	8	74177401	74177700	0.213	0.772	0.317	0.081
Tan	8	74189701	74190000	0.235	0.586	0.136	0.167
Tan	8	74343901	74344200	0.266	0.628	0.205	0.162
Tan	8	74423401	74423700	0.106	0.468	0.132	0.061
Tan	8	74425801	74426100	1.437	2.057	1.152	1.034
Tan	8	74460601	74460900	1.458	2.147	1.173	2.910
Tan	8	74625301	74625600	0.106	0.423	0.049	0.051
Tan	8	74646301	74646600	0.081	0.401	0.160	0.167
Tan	8	74697901	74698200	0.982	1.195	0.502	0.745
Tan	8	74800201	74800500	0.584	0.867	0.296	0.416
Tan	8	74801401	74801700	0.345	0.656	0.210	0.137
Tan	8	75011101	75011400	0.560	1.217	0.526	0.639
Tan	8	75203101	75203400	0.371	0.654	0.152	0.193
Tan	8	75343501	75343800	1.699	2.890	1.235	0.740
Tan	8	75343801	75344100	0.797	1.480	0.617	0.628
Tan	8	75699901	75700200	0.666	0.955	0.609	0.639
Tan	8	75721801	75722100	0.476	1.450	1.037	1.105
Tan	8	75729901	75730200	0.266	0.586	0.214	0.268
Tan	8	75915601	75915900	0.448	0.820	0.428	0.329
Tan	8	76629001	76629300	0.344	0.727	0.346	0.360
Tan	8	76629301	76629600	0.426	1.176	0.453	0.603
Tan	8	77061301	77061600	0.163	0.654	0.210	0.335
Tan	8	77433901	77434200	0.240	0.586	0.181	0.274
Tan	8	77473201	77473500	0.187	0.446	0.189	0.223

Tan	8	77480101	77480400	0.160	0.772	0.165	0.248
Tan	8	77688001	77688300	1.758	2.909	1.801	1.638
Tan	8	77691901	77692200	0.374	0.794	0.535	0.137
Tan	8	77882101	77882400	0.240	0.444	0.234	0.218
Tan	8	77918401	77918700	4.680	3.158	4.483	4.902
Tan	8	78014101	78014400	0.186	0.562	0.312	0.218
Tan	8	78015601	78015900	0.584	1.384	0.613	0.846
Tan	8	78070201	78070500	0.374	0.562	0.370	0.248
Tan	8	78197401	78197700	0.695	1.637	0.778	1.242
Tan	8	78507001	78507300	0.268	0.684	0.107	0.167
Tan	8	78876301	78876600	0.160	0.491	0.160	0.106
Tan	8	78889801	78890100	0.532	0.375	0.691	0.553
Tan	8	79329001	79329300	0.424	0.562	0.740	0.968
Tan	8	79414501	79414800	0.477	0.305	0.078	0.137
Tan	8	79681801	79682100	0.081	0.682	0.210	0.238
Tan	8	79795201	79795500	0.184	1.129	0.225	0.365
Tan	8	79975801	79976100	6.052	4.697	6.934	8.377
Tan	8	80206501	80206800	0.345	0.772	0.234	0.441
Tan	8	80338201	80338500	0.426	0.959	0.272	0.193
Tan	8	80481301	80481600	1.139	2.369	0.999	1.445
Tan	8	80481601	80481900	0.187	0.775	0.082	0.112
Tan	8	80506501	80506800	0.715	1.519	0.760	0.502
Tan	8	80515501	80515800	0.292	0.562	0.185	0.254
Tan	8	80517301	80517600	1.544	1.216	1.514	2.434
Tan	8	80658301	80658600	0.344	0.702	0.350	0.391
Tan	8	80745901	80746200	2.312	1.369	2.305	2.358
Tan	8	80825101	80825400	0.531	0.326	0.160	0.502
Tan	8	80893501	80893800	3.191	2.483	3.870	3.798
Tan	8	80972401	80972700	1.491	1.804	1.037	0.751
Tan	8	80986801	80987100	0.160	0.541	0.152	0.025
Tan	8	80987401	80987700	0.294	0.607	0.259	0.162
Tan	8	81267901	81268200	0.610	1.129	0.535	0.466
Tan	8	81281401	81281700	2.916	4.510	3.146	2.895
Tan	8	81300601	81300900	0.132	0.539	0.029	0.101
Tan	8	81532801	81533100	0.345	0.234	0.535	0.527
Tan	8	81544201	81544500	0.690	0.955	0.588	0.690
Tan	8	81675901	81676200	0.397	0.772	0.361	0.335
Tan	8	81746701	81747000	0.558	1.148	0.296	0.553
Tan	8	81756901	81757200	0.552	1.877	0.707	0.984
Tan	8	81765001	81765300	4.152	2.442	3.431	4.086
Tan	8	82080901	82081200	2.389	3.892	2.263	1.945
Tan	8	82123501	82123800	0.428	1.849	0.718	0.275
Tan	8	82123801	82124100	0.558	2.156	0.781	0.437
Tan	8	82216801	82217100	1.334	2.566	0.999	1.491
Tan	8	82257601	82257900	0.529	1.009	0.395	0.248
Tan	8	82278901	82279200	0.555	0.940	0.156	0.553
Tan	8	82472701	82473000	2.820	2.362	2.706	3.108
Tan	8	82501201	82501500	0.453	0.701	0.481	0.360
Tan	8	82574401	82574700	0.424	0.654	0.370	0.299
Tan	8	82643701	82644000	0.161	0.446	0.132	0.132
Tan	8	82707601	82707900	0.479	0.843	0.535	0.452
Tan	8	82800601	82800900	0.026	0.768	0.103	0.162
Tan	8	82800901	82801200	0.240	1.405	0.370	0.340
Tan	8	82802101	82802400	0.426	1.101	0.802	0.421
Tan	8	82831501	82831800	0.477	0.305	0.642	0.832
Tan	8	82939801	82940100	5.257	3.825	5.493	5.542
Tan	8	83115601	83115900	1.810	2.682	2.196	1.491
Tan	8	83356501	83356800	0.560	1.141	0.535	0.588
Tan	8	83359801	83360100	0.747	1.150	0.259	0.745
Tan	8	83419501	83419800	1.939	2.789	2.082	1.881
Tan	8	83522101	83522400	0.213	0.845	0.510	0.223
Tan	8	83580901	83581200	0.584	1.240	0.638	0.512
Tan	8	83637301	83637600	0.979	2.053	1.048	0.704
Tan	8	83649901	83650200	2.710	1.399	3.221	2.830
Tan	8	83711701	83712000	0.932	0.421	1.061	0.907
Tan	8	83798101	83798400	0.345	0.790	0.453	0.497
Tan	8	83837701	83838000	0.774	2.068	0.517	0.649

Tan	8	83849701	83850000	0.053	1.223	0.147	0.167
Tan	8	84008101	84008400	0.318	0.654	0.428	0.218
Tan	8	84012601	84012900	1.353	0.577	1.115	1.161
Tan	8	84110401	84110700	1.250	1.098	0.633	0.578
Tan	8	84370201	84370500	0.479	0.258	0.457	0.527
Tan	8	84706201	84706500	3.141	2.596	1.037	1.967
Tan	8	84929401	84929700	0.534	1.116	0.555	0.502
Tan	8	84999301	84999600	0.187	0.397	0.267	0.497
Tan	8	85114801	85115100	3.270	2.438	3.175	3.093
Tan	8	85231201	85231500	5.788	3.510	6.137	5.770
Tan	8	85268101	85268400	7.387	5.520	8.560	7.043
Tan	8	85331101	85331400	0.265	0.446	0.078	0.142
Tan	8	85498801	85499100	1.341	2.628	1.213	1.460
Tan	8	85682101	85682400	2.576	3.602	2.294	2.368
Tan	8	85917901	85918200	0.158	0.654	0.214	0.162
Tan	8	85960201	85960500	0.848	1.616	0.905	0.873
Tan	8	86547001	86547300	0.165	0.747	0.267	0.167
Tan	8	86692501	86692800	0.182	0.631	0.234	0.223
Tan	8	86748901	86749200	0.211	0.466	0.029	0.162
Tan	8	86874001	86874300	1.994	1.474	1.913	2.266
Tan	8	86900101	86900400	0.186	0.515	0.210	0.173
Tan	8	87036001	87036300	0.663	1.210	0.502	0.457
Tan	8	87037801	87038100	0.160	0.470	0.210	0.000
Tan	8	87214801	87215100	0.289	0.749	0.267	0.274
Tan	8	87318301	87318600	1.195	1.723	1.008	1.125
Tan	8	87332701	87333000	3.462	1.135	3.477	3.672
Tan	8	87487501	87487800	1.829	1.219	0.716	0.558
Tan	8	87714601	87714900	0.213	0.444	0.165	0.137
Tan	8	87780901	87781200	2.239	0.921	2.218	2.343
Tan	8	87840901	87841200	0.584	0.212	0.049	0.385
Tan	8	87933301	87933600	0.665	0.375	0.189	0.472
Tan	8	88018801	88019100	0.318	0.723	0.403	0.187
Tan	8	88093201	88093500	2.787	0.789	2.417	1.795
Tan	8	88098301	88098600	8.355	2.709	9.894	8.876
Tan	8	88124701	88125000	3.172	1.650	2.867	3.155
Tan	8	88126501	88126800	4.546	3.163	4.301	2.139
Tan	8	88152901	88153200	2.960	1.255	2.932	2.997
Tan	8	88153201	88153500	8.355	4.020	8.089	9.038
Tan	8	88155301	88155600	2.421	4.506	2.825	3.174
Tan	8	88256401	88256700	4.389	2.817	3.939	4.269
Tan	8	88291201	88291500	1.113	2.154	1.280	1.456
Tan	8	88376401	88376700	0.642	1.517	0.624	0.725
Tan	8	88398601	88398900	2.415	1.916	1.538	2.520
Tan	8	88409701	88410000	0.371	0.491	0.267	0.360
Tan	8	88474801	88475100	9.789	5.806	10.060	11.171
Tan	8	88547101	88547400	0.769	1.219	0.638	0.690
Tan	8	88654201	88654500	4.120	3.551	3.237	3.154
Tan	8	88729801	88730100	1.008	0.494	1.086	1.111
Tan	8	88786201	88786500	8.661	3.357	8.476	8.023
Tan	8	88825801	88826100	6.931	3.735	5.361	6.019
Tan	8	89027701	89028000	6.461	5.445	7.141	6.490
Tan	8	89031301	89031600	1.389	2.764	1.569	1.258
Tan	8	89034601	89034900	0.531	0.701	0.292	0.243
Tan	8	89043301	89043600	1.702	0.865	1.222	1.546
Tan	8	89049001	89049300	1.862	1.139	2.160	2.140
Tan	8	89129101	89129400	1.250	1.146	0.769	0.943
Tan	8	89157001	89157300	0.292	1.006	1.806	0.472
Tan	8	89295901	89296200	10.629	6.848	11.331	12.069
Tan	8	89392801	89393100	2.847	2.405	1.962	1.973
Tan	8	89424001	89424300	2.292	1.023	1.917	1.917
Tan	8	89446501	89446800	1.702	1.217	1.558	1.552
Tan	8	89609101	89609400	0.505	1.330	0.745	0.893
Tan	8	89649301	89649600	1.381	1.824	1.440	1.410
Tan	8	89796901	89797200	0.505	0.978	0.531	0.547
Tan	8	89998201	89998500	0.106	0.515	0.152	0.162
Tan	8	90204901	90205200	0.690	0.277	0.560	0.608
Tan	8	90263401	90263700	5.736	3.802	4.995	5.208

Tan	8	90287101	90287400	0.582	0.985	0.539	0.446
Tan	8	90428101	90428400	0.716	1.075	0.424	0.740
Tan	8	90547801	90548100	0.876	1.261	0.716	0.553
Tan	8	90600001	90600300	0.934	1.614	0.595	0.979
Tan	8	90633301	90633600	0.932	2.227	0.909	1.764
Tan	9	25501	25800	2.241	3.967	2.002	1.933
Tan	9	360901	361200	0.608	0.961	0.288	0.472
Tan	9	384601	384900	0.319	0.539	0.185	0.385
Tan	9	474301	474600	0.316	0.770	0.321	0.405
Tan	9	624301	624600	0.319	1.710	0.339	0.329
Tan	9	790801	791100	0.347	0.800	0.127	0.274
Tan	9	846601	846900	0.345	0.586	0.317	0.279
Tan	9	860701	861000	2.736	2.381	1.893	1.993
Tan	9	1120501	1120800	2.905	4.727	2.429	3.062
Tan	9	1255501	1255800	0.132	0.395	0.185	0.086
Tan	9	2029201	2029500	0.371	0.584	0.428	0.279
Tan	9	2641201	2641500	0.108	0.796	0.419	0.477
Tan	9	3291601	3291900	0.319	1.182	0.132	0.228
Tan	9	3440101	3440400	0.266	0.538	0.054	0.218
Tan	9	3472201	3472500	0.771	1.289	0.662	0.877
Tan	9	3567601	3567900	0.027	0.628	0.107	0.112
Tan	9	3735601	3735900	0.213	0.846	0.341	0.396
Tan	9	4084501	4084800	0.108	0.444	0.054	0.142
Tan	9	4771801	4772100	0.505	1.054	0.588	0.751
Tan	9	5429101	5429400	0.266	0.659	0.267	0.248
Tan	9	5514901	5515200	0.639	1.317	0.716	0.644
Tan	9	5705701	5706000	0.266	0.446	0.156	0.223
Tan	9	6009301	6009600	0.481	0.068	0.424	0.355
Tan	9	6932401	6932700	0.294	0.558	0.103	0.223
Tan	9	7929001	7929300	0.481	1.483	0.548	0.431
Tan	9	8118001	8118300	0.186	0.751	0.239	0.304
Tan	9	8249401	8249700	0.239	0.564	0.370	0.304
Tan	9	9347401	9347700	0.239	0.466	0.214	0.248
Tan	9	9898201	9898500	0.215	0.704	0.267	0.248
Tan	9	10040701	10041000	0.132	0.395	0.136	0.193
Tan	9	10075201	10075500	0.237	0.815	0.259	0.086
Tan	9	10667701	10668000	1.224	0.744	0.419	1.440
Tan	9	10840801	10841100	0.134	0.517	0.239	0.248
Tan	9	10995301	10995600	0.079	0.489	0.263	0.137
Tan	9	11036101	11036400	0.345	0.680	0.346	0.502
Tan	9	11161201	11161500	0.134	0.418	0.107	0.193
Tan	9	12288901	12289200	0.532	0.772	0.535	0.533
Tan	9	12789001	12789300	0.611	0.234	0.502	0.943
Tan	9	12856201	12856500	0.345	0.539	0.292	0.132
Tan	9	12896101	12896400	1.386	2.345	1.086	0.963
Tan	9	12963601	12963900	0.824	1.661	1.300	0.933
Tan	9	13110901	13111200	1.116	0.539	1.201	1.085
Tan	9	13189501	13189800	0.134	0.517	0.098	0.137
Tan	9	13195201	13195500	0.910	1.425	2.200	2.246
Tan	9	13632601	13632900	2.281	0.510	1.012	1.937
Tan	9	13870201	13870500	5.338	3.105	4.994	5.516
Tan	9	13960801	13961100	0.053	1.006	0.203	0.056
Tan	9	14225701	14226000	1.066	1.287	0.609	0.857
Tan	9	14228101	14228400	0.027	0.395	0.029	0.142
Tan	9	14329801	14330100	1.352	0.459	2.241	1.577
Tan	9	14358601	14358900	0.557	1.978	0.818	0.923
Tan	9	14506501	14506800	0.239	0.588	0.210	0.274
Tan	9	14571601	14571900	0.266	0.045	0.798	0.573
Tan	9	14894401	14894700	2.284	2.828	2.242	2.373
Tan	9	14941501	14941800	0.187	0.536	0.156	0.243
Tan	9	15109801	15110100	0.318	0.539	0.346	0.279
Tan	9	15235201	15235500	3.646	2.135	3.810	3.782
Tan	9	15402301	15402600	0.663	1.103	0.497	0.527
Tan	9	15414301	15414600	0.984	0.584	0.823	1.024
Tan	9	15426001	15426300	0.319	0.538	1.411	0.061
Tan	9	15625201	15625500	0.452	0.891	0.374	0.365
Tan	9	15679801	15680100	1.470	2.553	1.420	1.450

Tan	9	15786601	15786900	0.934	1.888	0.934	1.065
Tan	9	15945301	15945600	3.579	5.137	3.504	3.346
Tan	9	15945601	15945900	3.297	3.888	2.637	3.073
Tan	9	16027801	16028100	0.186	0.699	0.419	0.304
Tan	9	16056301	16056600	0.106	0.466	0.156	0.299
Tan	9	16116901	16117200	6.140	0.800	7.604	7.790
Tan	9	16117201	16117500	3.731	0.984	3.244	4.238
Tan	9	16414801	16415100	2.155	3.101	1.222	1.800
Tan	9	16658401	16658700	0.082	0.628	0.029	0.106
Tan	9	16716901	16717200	0.215	0.586	0.292	0.385
Tan	9	16736701	16737000	0.423	0.770	0.189	0.142
Tan	9	16883101	16883400	1.807	0.510	1.123	1.293
Tan	9	17004001	17004300	1.491	1.000	1.489	1.389
Tan	9	17034301	17034600	0.740	1.238	0.588	0.806
Tan	9	17093401	17093700	0.797	1.453	0.959	0.350
Tan	9	17437801	17438100	0.216	0.800	0.259	0.243
Tan	9	17547901	17548200	1.120	0.236	0.925	0.963
Tan	9	17565001	17565300	0.244	0.882	0.296	0.112
Tan	9	18446701	18447000	0.106	0.465	0.054	0.162
Tan	9	18667501	18667800	0.265	0.935	0.169	0.112
Tan	9	18958201	18958500	0.211	0.612	0.210	0.056
Tan	9	19149001	19149300	0.292	0.491	0.160	0.355
Tan	9	19185901	19186200	0.186	0.446	0.267	0.193
Tan	9	19368301	19368600	2.260	3.006	1.522	1.967
Tan	9	19953301	19953600	1.410	1.054	1.440	1.298
Tan	9	20013001	20013300	0.932	1.077	0.691	0.497
Tan	9	20050501	20050800	1.807	2.246	0.740	1.586
Tan	9	20140201	20140500	5.451	3.585	5.281	5.462
Tan	9	20236201	20236500	3.052	3.720	2.753	4.518
Tan	9	20261701	20262000	0.369	0.907	0.205	0.173
Tan	9	20349601	20349900	0.369	0.721	0.214	0.279
Tan	9	20533501	20533800	0.581	1.433	0.524	0.416
Tan	9	20686501	20686800	0.184	0.420	0.103	0.167
Tan	9	20805301	20805600	2.018	2.525	1.476	1.906
Tan	9	20879401	20879700	0.615	1.351	0.419	0.761
Tan	9	21086101	21086400	0.716	0.423	0.136	0.173
Tan	9	21176701	21177000	0.477	0.096	0.366	0.360
Tan	9	21212101	21212400	0.637	0.305	0.160	0.634
Tan	9	21260401	21260700	0.505	0.680	0.292	0.446
Tan	9	21363301	21363600	0.505	0.727	0.481	0.218
Tan	9	21389101	21389400	0.189	1.030	0.234	0.690
Tan	9	21786901	21787200	0.268	0.605	0.181	0.162
Tan	9	21964501	21964800	4.257	3.883	3.143	3.366
Tan	9	22133401	22133700	0.213	0.468	0.214	0.213
Tan	9	22551901	22552200	0.373	0.656	0.448	0.254
Tan	9	22689901	22690200	1.541	3.152	1.550	1.384
Tan	9	22777801	22778100	0.156	0.513	0.160	0.117
Tan	9	23059801	23060100	0.239	0.539	0.239	0.218
Tan	9	23463001	23463300	0.742	1.169	0.526	0.740
Tan	9	23490301	23490600	2.797	3.392	1.843	1.871
Tan	9	23516701	23517000	1.971	2.832	1.589	1.800
Tan	9	23603701	23604000	0.532	0.232	0.049	0.081
Tan	9	23604001	23604300	0.505	0.230	0.000	0.031
Tan	9	23621101	23621400	0.239	0.584	0.267	0.365
Tan	9	23623201	23623500	0.345	0.210	0.374	0.745
Tan	9	23837701	23838000	1.166	1.802	0.992	1.227
Tan	9	24007201	24007500	0.318	0.631	0.288	0.360
Tan	9	24113101	24113400	0.558	0.257	0.526	0.502
Tan	9	24113401	24113700	1.570	0.959	1.115	1.024
Tan	9	24306001	24306300	0.132	0.466	0.189	0.106
Tan	9	24873901	24874200	0.400	0.747	0.453	0.608
Tan	9	25249201	25249500	1.061	1.519	0.642	1.105
Tan	9	25365901	25366200	2.897	1.521	2.572	2.561
Tan	9	25400401	25400700	1.091	1.569	0.892	0.765
Tan	9	25559101	25559400	1.250	1.942	0.711	1.334
Tan	9	25567501	25567800	0.531	1.247	0.687	0.578
Tan	9	25631101	25631400	0.373	0.208	0.555	0.497

Tan	9	26089501	26089800	0.265	0.444	0.210	0.051
Tan	9	26138401	26138700	2.509	4.089	2.140	2.498
Tan	9	26154901	26155200	0.316	0.652	0.346	0.279
Tan	9	26197801	26198100	0.692	0.302	0.691	0.588
Tan	9	26442601	26442900	0.132	0.494	0.074	0.106
Tan	9	26467201	26467500	0.532	0.860	0.424	0.360
Tan	9	26467501	26467800	2.928	4.175	3.290	2.383
Tan	9	26784901	26785200	0.160	0.609	0.058	0.198
Tan	9	26877901	26878200	0.026	0.395	0.210	0.193
Tan	9	27464101	27464400	0.661	1.165	0.658	0.715
Tan	9	27485101	27485400	1.570	2.103	1.152	1.394
Tan	9	27558001	27558300	0.505	0.631	0.370	0.527
Tan	9	27583801	27584100	1.994	2.626	1.411	1.881
Tan	9	27706501	27706800	0.373	0.657	0.156	0.335
Tan	9	27809401	27809700	3.931	3.368	4.213	4.569
Tan	9	27869701	27870000	0.745	0.656	0.453	0.472
Tan	9	28014901	28015200	0.213	0.373	0.506	0.223
Tan	9	28276201	28276500	1.250	2.242	1.382	1.460
Tan	9	28335901	28336200	0.374	0.891	0.481	0.608
Tan	9	28410301	28410600	0.450	1.028	0.243	0.568
Tan	9	28450801	28451100	0.314	0.845	0.239	0.274
Tan	9	28644001	28644300	0.560	1.120	0.901	0.558
Tan	9	29116501	29116800	0.106	0.395	0.078	0.132
Tan	9	29436601	29436900	0.373	0.584	0.424	0.466
Tan	9	29681101	29681400	1.190	1.850	0.867	1.212
Tan	9	29681401	29681700	2.707	3.757	1.884	2.343
Tan	9	29706601	29706900	0.213	0.468	0.132	0.304
Tan	9	29709601	29709900	0.242	0.609	0.156	0.335
Tan	9	30599401	30599700	0.929	0.727	0.959	0.502
Tan	9	30775201	30775500	0.371	1.530	0.330	0.329
Tan	9	30955501	30955800	0.905	1.238	0.987	0.547
Tan	9	31071001	31071300	0.158	0.515	0.136	0.335
Tan	9	31309801	31310100	0.292	0.777	0.370	0.304
Tan	9	31954201	31954500	0.213	0.539	0.156	0.187
Tan	9	31988101	31988400	0.876	1.849	0.729	1.359
Tan	9	32091901	32092200	0.349	0.676	0.346	0.268
Tan	9	32320201	32320500	0.720	1.287	0.667	0.513
Tan	9	32325001	32325300	0.265	0.657	0.123	0.167
Tan	9	32369401	32369700	0.266	0.515	0.214	0.309
Tan	9	32393101	32393400	2.150	3.369	2.102	2.799
Tan	9	32682001	32682300	2.284	0.646	2.842	2.354
Tan	9	32706301	32706600	7.617	5.616	10.877	8.644
Tan	9	32946301	32946600	0.426	0.916	0.288	0.517
Tan	9	33141901	33142200	0.210	0.657	0.288	0.223
Tan	9	33223801	33224100	1.249	0.603	1.358	1.512
Tan	9	33459601	33459900	0.187	0.444	0.189	0.274
Tan	9	33483901	33484200	0.052	0.517	0.078	0.056
Tan	9	33726901	33727200	0.429	0.893	0.296	0.081
Tan	9	33747001	33747300	0.318	0.800	0.370	0.243
Tan	9	33971101	33971400	0.453	1.023	0.350	0.391
Tan	9	34074601	34074900	0.958	1.571	0.959	1.039
Tan	9	34218001	34218300	0.477	0.865	0.531	0.583
Tan	9	34228501	34228800	0.400	1.191	0.745	0.720
Tan	9	34275001	34275300	0.132	0.395	0.239	0.112
Tan	9	34338001	34338300	0.589	1.146	0.535	0.568
Tan	9	34919701	34920000	0.479	0.302	0.531	0.527
Tan	9	34957501	34957800	1.118	1.493	0.765	1.181
Tan	9	35038801	35039100	0.265	0.699	0.049	0.147
Tan	9	35702701	35703000	0.266	0.468	0.185	0.279
Tan	9	35854801	35855100	0.613	0.888	0.613	0.335
Tan	9	35864101	35864400	0.349	0.931	0.408	0.106
Tan	9	35960701	35961000	0.239	0.775	0.111	0.416
Tan	9	36070501	36070800	0.319	0.586	0.189	0.304
Tan	9	36073501	36073800	0.715	0.167	1.139	0.644
Tan	9	36431701	36432000	0.797	1.315	0.399	0.614
Tan	9	36499501	36499800	0.369	0.749	0.181	0.360
Tan	9	36833401	36833700	1.542	1.332	0.789	1.050

Tan	9	37627201	37627500	0.134	0.629	0.107	0.472
Tan	9	37770001	37770300	1.303	1.918	1.012	1.424
Tan	9	37779601	37779900	0.453	0.747	0.370	0.436
Tan	9	37922101	37922400	0.292	0.888	0.366	0.411
Tan	9	37954201	37954500	0.240	0.656	0.234	0.259
Tan	9	38135701	38136000	0.508	0.980	0.374	0.421
Tan	9	38277301	38277600	0.081	0.538	0.098	0.223
Tan	9	38422801	38423100	0.397	0.936	0.341	0.573
Tan	9	38423101	38423400	0.345	1.058	0.366	0.391
Tan	9	38718001	38718300	0.052	0.472	0.078	0.137
Tan	9	38850901	38851200	0.132	0.420	0.103	0.061
Tan	9	38911201	38911500	0.215	0.744	0.160	0.198
Tan	9	38938801	38939100	1.788	1.216	1.801	2.119
Tan	9	39138301	39138600	0.079	0.564	0.288	0.198
Tan	9	39193501	39193800	0.318	0.701	0.296	0.234
Tan	9	39292801	39293100	0.769	1.264	0.774	0.740
Tan	9	39377401	39377700	5.161	5.636	5.281	4.497
Tan	9	39486001	39486300	0.294	0.744	0.132	0.335
Tan	9	39587401	39587700	0.295	0.586	0.078	0.167
Tan	9	39661201	39661500	1.594	1.334	0.798	0.907
Tan	9	39678901	39679200	0.477	0.281	0.481	0.441
Tan	9	39847801	39848100	0.053	0.583	0.210	0.126
Tan	9	39875101	39875400	0.821	3.025	1.556	1.513
Tan	9	39889201	39889500	0.160	0.494	0.189	0.051
Tan	9	39900901	39901200	1.970	3.051	1.638	2.104
Tan	9	39907501	39907800	0.637	1.633	1.016	1.035
Tan	9	40155001	40155300	0.027	0.494	0.111	0.162
Tan	9	40182001	40182300	0.106	0.633	0.152	0.126
Tan	9	40830601	40830900	0.055	0.912	0.341	0.268
Tan	9	41306701	41307000	0.132	0.448	0.107	0.142
Tan	9	41752801	41753100	0.134	0.513	0.074	0.279
Tan	9	41920201	41920500	2.339	1.826	1.498	1.491
Tan	9	42109501	42109800	0.187	0.519	0.136	0.167
Tan	9	42196201	42196500	0.766	1.496	1.008	0.938
Tan	9	42200101	42200400	0.294	0.633	0.374	0.218
Tan	9	42208501	42208800	0.239	0.654	0.296	0.421
Tan	9	42717301	42717600	0.398	0.704	0.395	0.187
Tan	9	42850201	42850500	0.239	0.515	0.317	0.187
Tan	9	43229401	43229700	0.452	0.773	0.395	0.436
Tan	9	43424101	43424400	1.116	1.714	0.983	0.846
Tan	9	43513501	43513800	0.136	0.470	0.132	0.162
Tan	9	43555501	43555800	0.852	0.281	0.667	0.846
Tan	9	43583401	43583700	0.186	0.515	0.210	0.335
Tan	9	43583701	43584000	0.531	1.594	0.435	0.766
Tan	9	43589701	43590000	0.552	2.396	0.823	0.939
Tan	9	43869901	43870200	1.545	2.442	1.391	1.435
Tan	9	44938501	44938800	0.132	0.633	0.136	0.101
Tan	9	46230001	46230300	0.850	1.077	0.617	0.720
Tan	9	46269901	46270200	1.697	1.004	2.049	1.410
Tan	9	46727101	46727400	0.477	0.798	0.341	0.056
Tan	9	46758301	46758600	0.294	0.635	0.127	0.299
Tan	9	46986901	46987200	0.211	0.657	0.185	0.238
Tan	9	47205001	47205300	0.265	0.564	0.267	0.218
Tan	9	47724001	47724300	0.240	0.888	0.617	0.608
Tan	9	48020701	48021000	0.186	0.420	0.267	0.162
Tan	9	48923401	48923700	0.294	0.489	0.321	0.248
Tan	9	48923701	48924000	0.477	0.747	0.263	0.213
Tan	9	49352401	49352700	0.323	0.798	0.296	0.304
Tan	9	49414801	49415100	0.957	0.395	0.999	0.801
Tan	9	49504201	49504500	0.266	0.140	0.428	0.558
Tan	9	49745101	49745400	0.289	1.328	0.194	0.664
Tan	9	49906801	49907100	0.479	0.862	0.432	0.522
Tan	9	49948801	49949100	1.858	2.615	1.333	1.020
Tan	9	50404201	50404500	0.665	0.794	0.267	0.522
Tan	9	50542201	50542500	0.081	0.423	0.156	0.081
Tan	9	51134701	51135000	0.476	0.963	0.292	0.365
Tan	9	51165901	51166200	0.161	0.538	0.185	0.360

Tan	9	51306001	51306300	0.481	1.098	0.390	0.451
Tan	9	52577101	52577400	0.134	0.468	0.243	0.162
Tan	9	54168001	54168300	0.503	0.654	0.132	0.360
Tan	9	54985501	54985800	0.640	1.006	0.633	0.644
Tan	9	55512001	55512300	0.105	0.813	0.254	0.025
Tan	9	55723801	55724100	0.532	0.725	0.448	0.522
Tan	9	55729801	55730100	0.186	0.517	0.127	0.248
Tan	9	55991101	55991400	0.240	0.701	0.263	0.578
Tan	9	56038501	56038800	0.774	1.442	0.497	0.594
Tan	9	56074801	56075100	0.321	0.725	0.296	0.309
Tan	9	56077501	56077800	0.349	0.798	0.103	0.315
Tan	9	56293501	56293800	0.318	0.912	0.267	0.720
Tan	9	56293801	56294100	0.398	1.127	0.239	0.690
Tan	9	56303701	56304000	0.824	0.444	0.959	1.206
Tan	9	56418301	56418600	0.265	0.588	0.239	0.243
Tan	9	56475301	56475600	0.505	0.236	0.448	0.466
Tan	9	56760601	56760900	9.145	7.914	9.425	9.031
Tan	9	57049201	57049500	0.505	0.631	0.263	0.218
Tan	9	57096601	57096900	1.541	2.544	1.501	1.876
Tan	9	57163201	57163500	0.982	1.240	1.699	1.942
Tan	9	58766401	58766700	0.240	0.517	0.317	0.193
Tan	9	58936801	58937100	0.369	0.678	0.234	0.254
Tan	9	58987201	58987500	0.347	0.678	0.292	0.182
Tan	9	59091001	59091300	0.345	0.607	0.403	0.360
Tan	9	59112301	59112600	0.479	0.609	0.292	1.136
Tan	9	59140201	59140500	0.476	0.985	0.535	0.634
Tan	9	59153401	59153700	1.412	2.029	1.407	1.100
Tan	9	59564701	59565000	0.266	0.420	0.160	0.218
Tan	9	59805301	59805600	0.294	0.491	0.185	0.360
Tan	9	59939701	59940000	0.670	1.289	0.609	0.740
Tan	9	60591601	60591900	0.161	0.848	0.210	0.126
Tan	9	60686701	60687000	0.027	0.371	0.025	0.106
Tan	9	60981901	60982200	0.424	0.841	0.428	0.522
Tan	9	61073401	61073700	0.744	0.981	0.769	0.517
Tan	9	61280101	61280400	0.239	0.444	0.156	0.117
Tan	9	61354501	61354800	0.266	0.538	0.346	0.304
Tan	9	61516501	61516800	3.807	6.604	2.449	3.654
Tan	9	61693501	61693800	0.318	0.725	0.395	0.492
Tan	9	61753201	61753500	0.266	0.541	0.156	0.248
Tan	9	61804801	61805100	0.106	0.491	0.267	0.112
Tan	9	62016001	62016300	0.665	0.397	0.506	0.472
Tan	9	62373901	62374200	0.369	0.680	0.189	0.304
Tan	9	62383201	62383500	0.318	0.980	0.359	0.329
Tan	9	64326301	64326600	0.184	0.654	0.127	0.329
Tan	9	64419301	64419600	0.321	1.053	0.774	0.710
Tan	9	66768601	66768900	1.939	0.998	2.169	1.946
Tan	9	67611901	67612200	0.640	1.101	0.481	0.558
Tan	9	67721401	67721700	0.452	0.656	0.428	0.421
Tan	9	67783201	67783500	1.353	2.300	0.885	1.186
Tan	9	68002201	68002500	0.053	0.420	0.156	0.187
Tan	9	68097601	68097900	0.161	0.588	0.194	0.086
Tan	9	68136601	68136900	0.318	0.633	0.267	0.248
Tan	9	68192701	68193000	0.213	0.558	0.160	0.360
Tan	9	68660101	68660400	0.797	1.073	0.827	0.832
Tan	9	68664001	68664300	0.819	1.407	0.580	0.862
Tan	9	69575101	69575400	0.081	0.543	0.107	0.025
Tan	9	69696901	69697200	0.156	0.468	0.111	0.025
Tan	9	69717001	69717300	0.903	0.513	0.341	0.690
Tan	9	70487701	70488000	0.103	0.541	0.107	0.193
Tan	9	70871101	70871400	2.123	1.028	2.167	1.546
Tan	9	70947301	70947600	0.345	0.607	0.424	0.223
Tan	9	71326501	71326800	0.479	0.258	0.477	0.360
Tan	9	71357701	71358000	0.106	0.466	0.239	0.142
Tan	9	71393701	71394000	0.515	2.354	0.970	0.391
Tan	9	71911501	71911800	1.016	2.485	1.045	1.217
Tan	9	72067801	72068100	0.160	0.446	0.156	0.254
Tan	9	72081001	72081300	0.347	0.539	0.346	0.385

Tan	9	73173601	73173900	0.637	0.373	0.160	0.497
Tan	9	73522501	73522800	0.213	0.468	0.321	0.193
Tan	9	73789801	73790100	0.476	1.360	0.695	0.669
Tan	9	74124001	74124300	1.008	1.676	0.749	0.533
Tan	9	74166001	74166300	1.805	2.573	0.927	1.521
Tan	9	74491801	74492100	0.082	0.465	0.029	0.051
Tan	9	74717101	74717400	0.053	0.468	0.156	0.101
Tan	9	74868301	74868600	0.186	0.511	0.132	0.223
Tan	9	74931301	74931600	0.079	0.446	0.185	0.193
Tan	9	74931601	74931900	0.269	0.680	0.272	0.243
Tan	9	75318301	75318600	1.590	0.376	1.786	1.268
Tan	9	75344701	75345000	0.398	0.867	0.082	0.213
Tan	9	75435001	75435300	0.239	0.755	0.156	0.051
Tan	9	75822601	75822900	0.665	0.983	0.366	0.527
Tan	9	75878701	75879000	0.318	0.539	0.317	0.193
Tan	9	75933001	75933300	0.345	0.674	0.453	0.223
Tan	9	75954301	75954600	0.186	0.446	0.136	0.193
Tan	9	76232101	76232400	0.160	0.420	0.160	0.025
Tan	9	76251301	76251600	0.324	0.959	0.243	0.421
Tan	9	76358701	76359000	0.790	1.450	0.613	0.578
Tan	9	76448101	76448400	1.118	1.967	0.843	1.145
Tan	9	76452001	76452300	0.239	0.560	0.127	0.309
Tan	9	76473901	76474200	0.187	0.702	0.395	0.309
Tan	9	77038801	77039100	0.134	0.466	0.160	0.112
Tan	9	78455401	78455700	0.398	0.702	0.210	0.315
Tan	9	78455701	78456000	0.397	0.869	0.448	0.324
Tan	9	78461101	78461400	1.647	2.802	2.316	1.298
Tan	9	78570001	78570300	0.716	1.122	0.584	0.603
Tan	9	78617701	78618000	0.373	0.468	0.263	0.248
Tan	9	78857701	78858000	0.611	1.099	0.564	0.710
Tan	9	78897601	78897900	0.290	0.562	0.132	0.254
Tan	9	78903601	78903900	0.481	1.051	0.390	0.542
Tan	9	78975901	78976200	0.802	1.380	0.584	0.477
Tan	9	78998401	78998700	0.237	0.895	0.312	0.284
Tan	9	79209001	79209300	0.266	0.468	0.317	0.223
Tan	9	79222501	79222800	0.690	0.912	0.346	0.340
Tan	9	79350301	79350600	0.186	0.517	0.243	0.025
Tan	9	79418101	79418400	0.453	0.303	0.506	0.193
Tan	9	79428301	79428600	0.077	0.468	0.189	0.106
Tan	9	79428601	79428900	0.105	0.536	0.160	0.309
Tan	9	79469401	79469700	1.065	0.330	1.449	1.648
Tan	9	79474801	79475100	1.731	0.914	3.030	3.386
Tan	9	79477201	79477500	0.881	0.399	0.802	0.862
Tan	9	79495801	79496100	1.836	2.549	1.329	1.714
Tan	9	79496101	79496400	0.639	0.914	0.399	0.558
Tan	9	79527301	79527600	0.531	1.056	0.288	0.538
Tan	9	79530001	79530300	0.874	1.480	0.646	0.690
Tan	9	79536601	79536900	0.186	0.515	0.078	0.086
Tan	9	79739401	79739700	0.213	0.493	0.103	0.081
Tan	9	79961101	79961400	0.637	0.886	0.604	1.217
Tan	9	80430001	80430300	0.160	0.493	0.189	0.248
Tan	9	80488801	80489100	1.144	1.356	0.633	1.105
Tan	9	80594401	80594700	0.560	1.150	0.560	0.567
Tan	9	80633401	80633700	0.423	0.794	0.444	0.142
Tan	9	80643901	80644200	0.319	0.657	0.428	0.360
Tan	9	80701501	80701800	0.215	0.588	0.292	0.248
Tan	9	80845201	80845500	0.558	0.912	0.457	0.583
Tan	9	81518701	81519000	0.503	0.888	0.588	0.502
Tan	9	81556801	81557100	1.458	0.863	1.353	1.323
Tan	9	81729001	81729300	0.239	0.491	0.267	0.218
Tan	9	81750601	81750900	0.931	1.165	0.905	0.578
Tan	9	81758701	81759000	2.471	2.759	2.472	3.407
Tan	9	81886201	81886500	0.771	1.193	0.535	0.533
Tan	9	82298401	82298700	0.000	0.420	0.132	0.132
Tan	9	82451701	82452000	4.872	3.626	4.688	5.116
Tan	9	82738201	82738500	0.216	0.820	0.399	0.340
Tan	9	82842901	82843200	0.210	0.609	0.107	0.274

Tan	9	83045701	83046000	0.189	0.657	0.267	0.198
Tan	9	83171401	83171700	0.376	0.775	0.267	0.385
Tan	9	83446501	83446800	0.397	0.846	0.312	0.304
Tan	9	83770201	83770500	0.131	1.115	0.288	0.152
Tan	9	83770501	83770800	0.106	0.948	0.239	0.000
Tan	9	84227101	84227400	0.531	0.234	0.107	0.391
Tan	9	84474601	84474900	0.266	0.470	0.132	0.112
Tan	9	84517201	84517500	0.373	0.560	0.403	0.000
Tan	9	84597301	84597600	0.106	0.420	0.078	0.106
Tan	9	84603301	84603600	0.187	0.588	0.263	0.137
Tan	9	85062901	85063200	0.611	0.255	0.642	0.603
Tan	9	85080301	85080600	5.403	4.503	5.727	5.730
Tan	9	85263901	85264200	1.197	1.959	1.201	1.328
Tan	9	85619701	85620000	0.161	0.517	0.049	0.193
Tan	9	85807801	85808100	0.883	1.762	0.847	1.024
Tan	9	86013001	86013300	0.295	0.611	0.136	0.223
Tan	9	86059801	86060100	0.186	0.468	0.317	0.360
Tan	9	86451001	86451300	0.905	1.431	0.802	0.791
Tan	9	86488201	86488500	2.471	1.523	2.098	1.764
Tan	9	87091201	87091500	1.044	0.376	1.008	1.729
Tan	9	87111601	87111900	0.639	0.841	0.638	0.380
Tan	9	87293401	87293700	0.347	0.682	0.272	0.167
Tan	9	87308401	87308700	0.402	1.244	0.736	0.309
Tan	9	87308701	87309000	0.828	1.517	1.012	0.502
Tan	9	87339001	87339300	0.457	1.403	0.466	0.796
Tan	9	87340501	87340800	1.508	2.761	1.962	1.947
Tan	9	87562501	87562800	2.925	4.216	3.344	2.966
Tan	9	87707701	87708000	0.744	0.560	0.239	0.299
Tan	9	88287601	88287900	0.428	0.820	0.424	0.452
Tan	9	88684501	88684800	0.160	0.564	0.341	0.223
Tan	9	88752601	88752900	2.475	4.371	1.806	2.496
Tan	9	88783501	88783800	0.932	2.103	1.190	1.278
Tan	9	88839001	88839300	0.768	1.618	0.720	0.741
Tan	9	88975801	88976100	0.292	0.468	0.136	0.274
Tan	9	89164801	89165100	3.052	4.304	2.932	3.225
Tan	9	89346901	89347200	0.186	0.446	0.214	0.218
Tan	9	89494501	89494800	2.265	1.453	2.343	2.408
Tan	9	89571601	89571900	0.642	0.212	0.720	0.938
Tan	9	89639101	89639400	1.171	0.371	0.695	0.669
Tan	9	89951701	89952000	0.213	0.466	0.165	0.223
Tan	9	90048901	90049200	0.760	2.622	0.205	0.759
Tan	9	90576301	90576600	0.240	0.513	0.181	0.279
Tan	9	90597601	90597900	3.088	1.675	4.428	2.951
Tan	9	90734101	90734400	0.424	0.747	0.205	0.391
Tan	9	90778501	90778800	0.591	1.191	0.370	0.405
Tan	9	90787501	90787800	0.344	0.657	0.321	0.223
Tan	9	90801001	90801300	0.213	0.541	0.132	0.254
Tan	9	90858901	90859200	0.529	1.178	0.296	0.335
Tan	9	90939601	90939900	0.476	0.794	0.374	0.274
Tan	9	91188001	91188300	0.156	0.611	0.156	0.031
Tan	9	91366801	91367100	0.455	0.867	0.399	0.497
Tan	9	91440301	91440600	1.442	2.414	1.449	1.597
Tan	9	91440601	91440900	1.252	2.583	1.378	1.562
Tan	9	91602901	91603200	0.186	0.633	0.288	0.162
Tan	9	91966201	91966500	0.637	0.468	0.346	0.360
Tan	9	92301601	92301900	0.479	0.631	0.210	0.335
Tan	9	92641501	92641800	0.161	0.468	0.111	0.056
Tan	9	92665501	92665800	0.266	0.586	0.160	0.335
Tan	9	92719201	92719500	0.400	0.236	0.613	0.497
Tan	9	92880301	92880600	0.503	0.912	0.535	0.411
Tan	9	92971501	92971800	2.436	3.978	2.925	2.388
Tan	9	93021901	93022200	1.034	0.538	0.852	1.019
Tan	9	93025201	93025500	2.365	3.587	2.080	2.424
Tan	9	93125701	93126000	3.481	4.370	3.312	3.239
Tan	9	93387301	93387600	0.397	1.225	0.305	0.497
Tan	9	93474301	93474600	0.871	2.102	1.440	1.232
Tan	9	93549901	93550200	1.278	1.922	0.959	1.085

Tan	9	93599701	93600000	0.369	0.980	0.497	0.583
Tan	9	93615601	93615900	0.210	0.863	0.308	0.243
Tan	9	93627901	93628200	1.516	2.598	1.501	1.667
Tan	9	93650101	93650400	0.426	1.360	0.276	0.303
Tan	9	93656701	93657000	0.715	1.339	0.600	0.466
Tan	9	93709501	93709800	0.371	0.798	0.058	0.132
Tan	9	93817801	93818100	0.268	0.633	0.210	0.112
Tan	9	93849601	93849900	1.382	1.901	1.173	1.161
Tan	9	93973201	93973500	0.321	0.721	0.395	0.416
Tan	9	94228501	94228800	0.161	0.790	0.267	0.203
Tan	9	94242901	94243200	0.027	0.652	0.000	0.000
Tan	9	94247101	94247400	0.239	0.558	0.259	0.162
Tan	9	94296301	94296600	5.417	3.239	5.183	6.370
Tan	9	94310401	94310700	2.018	3.680	1.914	1.754
Tan	9	94318801	94319100	1.462	0.384	1.574	2.272
Tan	9	94326901	94327200	0.108	0.654	0.230	0.228
Tan	9	94329901	94330200	0.266	0.794	0.263	0.365
Tan	9	94392301	94392600	0.450	0.888	0.535	0.452
Tan	9	94427401	94427700	0.531	1.098	0.613	0.740
Tan	9	94464301	94464600	1.331	1.852	0.954	0.213
Tan	9	94475701	94476000	1.408	0.790	1.431	1.197
Tan	9	94555201	94555500	0.186	0.725	0.254	0.132
Tan	9	94573201	94573500	0.215	0.609	0.296	0.173
Tan	9	94611601	94611900	0.502	1.446	0.905	0.538
Tan	9	94695301	94695600	0.158	0.697	0.234	0.198
Tan	X	79801	80100	0.268	0.770	0.481	0.558
Tan	X	127801	128100	4.048	6.552	4.508	3.366
Tan	X	227701	228000	0.428	1.944	0.727	0.254
Tan	X	243901	244200	7.304	4.799	6.224	6.591
Tan	X	274801	275100	0.989	1.833	0.881	0.948
Tan	X	287701	288000	1.008	1.748	0.852	0.877
Tan	X	334801	335100	3.376	4.229	2.296	2.698
Tan	X	345001	345300	0.848	0.116	1.016	0.999
Tan	X	384301	384600	0.749	1.401	0.802	0.639
Tan	X	387001	387300	1.065	1.897	1.505	1.100
Tan	X	433501	433800	0.105	0.749	0.267	0.416
Tan	X	442201	442500	2.949	2.197	1.601	2.484
Tan	X	444001	444300	0.799	1.101	0.535	0.614
Tan	X	477301	477600	3.292	5.057	3.030	3.422
Tan	X	480301	480600	1.195	0.935	1.522	1.516
Tan	X	519901	520200	0.613	0.116	0.497	0.472
Tan	X	537301	537600	2.239	1.375	3.369	2.511
Tan	X	538501	538800	8.262	0.560	8.189	12.016
Tan	X	538801	539100	5.693	0.890	5.613	7.297
Tan	X	543601	543900	2.310	4.203	1.914	1.674
Tan	X	581101	581400	0.211	0.493	0.000	0.213
Tan	X	700501	700800	1.139	1.781	1.119	1.192
Tan	X	740401	740700	1.673	2.931	1.629	1.790
Tan	X	759601	759900	0.534	0.792	0.346	0.198
Tan	X	771601	771900	1.521	0.912	1.382	1.410
Tan	X	926701	927000	0.161	0.682	0.325	0.248
Tan	X	947101	947400	0.802	1.148	0.156	0.329
Tan	X	951001	951300	0.799	1.399	0.789	0.527
Tan	X	1170601	1170900	0.292	0.094	0.025	0.497
Tan	X	1229401	1229700	0.692	1.101	0.461	0.573
Tan	X	1294501	1294800	2.421	0.989	2.916	2.754
Tan	X	1294801	1295100	2.733	0.303	2.858	2.697
Tan	X	1344301	1344600	0.213	0.494	0.185	0.248
Tan	X	1447801	1448100	4.989	7.175	4.336	5.892
Tan	X	1543801	1544100	0.213	0.603	0.185	0.279
Tan	X	1608601	1608900	2.886	4.184	2.234	2.653
Tan	X	1644301	1644600	0.319	1.292	0.473	0.588
Tan	X	1644601	1644900	1.650	2.556	0.889	0.978
Tan	X	1657801	1658100	0.881	0.302	1.193	0.948
Tan	X	1708201	1708500	0.639	1.429	0.678	0.771
Tan	X	1735201	1735500	0.586	2.494	0.636	0.709
Tan	X	1738501	1738800	3.565	1.142	3.288	3.205

Tan	X	1773001	1773300	0.773	1.006	1.275	0.588
Tan	X	1826701	1827000	0.587	0.191	1.251	0.654
Tan	X	1882201	1882500	0.292	0.094	0.502	0.248
Tan	X	1947601	1947900	1.324	0.890	1.485	2.130
Tan	X	1967401	1967700	0.189	0.515	0.185	0.243
Tan	X	1981801	1982100	0.744	0.489	0.239	0.299
Tan	X	2050801	2051100	0.457	1.079	0.428	0.274
Tan	X	2092501	2092800	0.345	0.656	0.267	0.441
Tan	X	2242801	2243100	4.383	0.522	2.416	2.659
Tan	X	2243701	2244000	0.189	0.936	0.506	0.162
Tan	X	2307001	2307300	1.166	2.268	1.226	1.612
Tan	X	2307301	2307600	0.316	1.287	0.551	0.573
Tan	X	2323801	2324100	0.823	1.704	0.938	0.801
Tan	X	2335801	2336100	0.697	1.470	0.468	0.497
Tan	X	2520601	2520900	0.106	0.635	0.218	0.137
Tan	X	2839801	2840100	0.347	0.800	0.111	0.360
Tan	X	3153301	3153600	2.760	5.501	3.824	3.493
Tan	X	3332401	3332700	0.694	1.597	0.513	0.248
Tan	X	3391501	3391800	0.560	0.421	0.267	0.664
Tan	X	3438901	3439200	2.083	3.738	2.328	1.981
Tan	X	3500701	3501000	0.134	1.056	0.546	0.173
Tan	X	3607801	3608100	0.344	0.843	0.506	0.309
Tan	X	3613201	3613500	1.961	3.585	1.331	1.784
Tan	X	3617101	3617400	0.371	0.796	0.584	0.664
Tan	X	3642001	3642300	0.215	0.420	0.107	0.223
Tan	X	3648901	3649200	0.537	1.817	0.635	0.765
Tan	X	3734101	3734400	0.668	1.496	0.909	0.319
Tan	X	3742501	3742800	1.145	1.569	0.745	1.237
Tan	X	3763801	3764100	0.105	0.511	0.185	0.025
Tan	X	3783901	3784200	2.686	3.341	2.615	1.785
Tan	X	3878101	3878400	0.321	0.539	0.346	0.248
Tan	X	3910501	3910800	1.140	1.704	1.226	1.222
Tan	X	3990301	3990600	0.690	1.313	0.704	0.497
Tan	X	4059001	4059300	1.970	2.381	1.358	1.784
Tan	X	4161601	4161900	0.105	0.491	0.321	0.279
Tan	X	4166101	4166400	1.278	1.450	0.987	1.055
Tan	X	4210501	4210800	1.303	2.832	2.011	1.667
Tan	X	4241401	4241700	0.319	0.538	0.321	0.304
Tan	X	4256401	4256700	0.532	1.124	0.539	0.558
Tan	X	4293901	4294200	1.202	1.637	0.987	0.578
Tan	X	4398001	4398300	0.903	1.892	1.164	0.457
Tan	X	4402801	4403100	4.083	5.773	3.157	3.352
Tan	X	4531501	4531800	0.663	1.847	1.304	1.333
Tan	X	4545901	4546200	0.160	0.493	0.317	0.335
Tan	X	4579201	4579500	2.794	2.008	2.229	1.384
Tan	X	4627201	4627500	0.369	0.929	0.239	0.385
Tan	X	4639201	4639500	0.613	0.796	0.531	0.522
Tan	X	4658101	4658400	0.158	0.448	0.058	0.056
Tan	X	4719901	4720200	0.294	0.890	0.259	0.167
Tan	X	5012101	5012400	1.037	0.607	0.317	0.416
Tan	X	5053801	5054100	0.213	0.539	0.239	0.284
Tan	X	5219401	5219700	0.587	1.553	0.510	0.355
Tan	X	5219701	5220000	0.321	0.908	0.395	0.213
Tan	X	5269801	5270100	1.297	3.164	0.802	1.279
Tan	X	5277601	5277900	0.398	0.723	0.283	0.329
Tan	X	5296201	5296500	0.132	0.489	0.000	0.228
Tan	X	5343601	5343900	0.718	1.266	0.745	0.513
Tan	X	5431801	5432100	0.666	0.212	0.395	0.695
Tan	X	5513101	5513400	1.992	1.476	1.937	1.105
Tan	X	5600701	5601000	0.742	1.244	0.526	0.533
Tan	X	5654701	5655000	0.799	1.363	0.555	0.492
Tan	X	5765701	5766000	0.876	1.240	0.979	0.806
Tan	X	6010201	6010500	0.294	0.586	0.292	0.279
Tan	X	6135901	6136200	0.853	1.614	0.930	0.771
Tan	X	6217501	6217800	0.215	0.515	0.234	0.187
Tan	X	6261301	6261600	2.959	4.798	2.766	3.235
Tan	X	6304801	6305100	0.426	1.530	0.446	0.218

Tan	X	6305101	6305400	0.594	1.472	0.263	0.401
Tan	X	6319801	6320100	0.689	1.219	0.740	0.594
Tan	X	6333901	6334200	0.156	0.659	0.210	0.081
Tan	X	6395401	6395700	0.907	2.725	1.563	0.634
Tan	X	6408301	6408600	0.613	1.476	0.617	0.547
Tan	X	6411901	6412200	0.108	0.513	0.288	0.167
Tan	X	6542701	6543000	1.687	3.881	1.030	0.811
Tan	X	6543001	6543300	0.730	2.667	0.234	0.405
Tan	X	6580201	6580500	0.452	0.303	0.613	0.441
Tan	X	6619501	6619800	0.924	1.498	0.695	0.862
Tan	X	6655801	6656100	0.769	1.897	0.850	0.893
Tan	X	6681301	6681600	2.126	3.323	2.122	1.835
Tan	X	6801901	6802200	3.562	4.619	2.905	3.372
Tan	X	6857401	6857700	1.566	2.622	1.415	1.303
Tan	X	8219101	8219400	0.134	0.465	0.107	0.056
Tan	X	8264701	8265000	0.637	0.468	0.239	0.243
Tan	X	8352301	8352600	0.453	0.798	0.214	0.279
Tan	X	8395801	8396100	2.049	1.525	2.069	1.982
Tan	X	8669701	8670000	13.095	9.970	13.192	12.626
Tan	X	8673301	8673600	0.505	0.745	0.346	0.527
Tan	X	9232801	9233100	0.240	0.446	0.160	0.218
Tan	X	9879901	9880200	0.316	0.609	0.263	0.274
Tan	X	10309801	10310100	0.053	0.515	0.239	0.117
Tan	X	10386001	10386300	0.423	0.096	0.424	0.477
Tan	X	10664101	10664400	0.873	1.605	0.740	0.968
Tan	X	11267101	11267400	1.783	1.495	1.839	2.135
Tan	X	11272801	11273100	0.586	0.444	0.691	0.329
Tan	X	11451901	11452200	0.187	0.727	0.234	0.385
Tan	X	11489401	11489700	0.158	0.448	0.082	0.137
Tan	X	11844601	11844900	0.266	0.350	0.506	0.248
Tan	X	12288301	12288600	0.240	0.583	0.136	0.051
Tan	X	12498601	12498900	0.079	0.560	0.205	0.157
Tan	X	12498901	12499200	0.136	1.026	0.444	0.517
Tan	X	12607501	12607800	2.154	2.360	3.134	2.687
Tan	X	12762601	12762900	0.079	0.442	0.132	0.000
Tan	X	13285201	13285500	0.160	0.468	0.156	0.187
Tan	X	13319401	13319700	2.066	3.141	2.171	2.058
Tan	X	14283001	14283300	0.558	0.350	0.181	0.497
Tan	X	14315401	14315700	0.213	0.584	0.136	0.304
Tan	X	14420701	14421000	1.515	1.940	0.987	1.318
Tan	X	14708101	14708400	0.027	0.187	0.350	0.466
Tan	X	14988601	14988900	0.266	0.584	0.399	0.497
Tan	X	15216901	15217200	0.263	0.751	0.288	0.329
Tan	X	15223201	15223500	0.211	0.656	0.214	0.162
Tan	X	15319801	15320100	0.450	1.480	0.218	0.579
Tan	X	15443701	15444000	0.720	1.028	0.613	0.720
Tan	X	15519901	15520200	0.344	0.817	0.325	0.502
Tan	X	15688201	15688500	0.345	0.773	0.317	0.355
Tan	X	16384801	16385100	2.157	0.963	1.868	1.384
Tan	X	16574701	16575000	0.850	1.077	0.325	0.223
Tan	X	16727401	16727700	0.321	0.845	0.374	0.472
Tan	X	16980601	16980900	1.037	0.794	0.560	0.887
Tan	X	17922301	17922600	1.333	2.813	1.730	1.354
Tan	X	18481201	18481500	0.108	0.420	0.107	0.086
Tan	X	18992401	18992700	0.160	0.423	0.189	0.162
Tan	X	19212001	19212300	0.160	0.395	0.029	0.056
Tan	X	21175201	21175500	0.134	0.442	0.185	0.223
Tan	X	21197401	21197700	0.474	0.912	0.453	0.198
Tan	X	21337501	21337800	0.558	0.397	0.239	0.497
Tan	X	21395101	21395400	0.481	1.826	0.593	1.024
Tan	X	21548401	21548700	0.106	0.465	0.054	0.187
Tan	X	21639301	21639600	0.294	0.631	0.321	0.112
Tan	X	22377901	22378200	0.160	0.489	0.189	0.187
Tan	X	22422601	22422900	0.397	0.798	0.283	0.162
Tan	X	22976401	22976700	0.105	0.635	0.194	0.132
Tan	X	23238901	23239200	0.586	0.185	0.481	0.329
Tan	X	23557801	23558100	0.314	0.751	0.107	0.112

Tan	X	24443701	24444000	0.242	0.818	0.399	0.502
Tan	X	25006801	25007100	0.210	0.893	0.288	0.294
Tan	X	25007101	25007400	0.158	0.706	0.239	0.274
Tan	X	25079701	25080000	0.106	0.401	0.156	0.112
Tan	X	25510801	25511100	0.082	0.423	0.103	0.086
Tan	X	25543201	25543500	0.373	0.721	0.424	0.411
Tan	X	25872601	25872900	0.507	0.818	0.325	0.441
Tan	X	25904401	25904700	1.171	1.495	1.119	0.791
Tan	X	26207101	26207400	0.347	0.562	0.156	0.198
Tan	X	26799901	26800200	0.213	0.468	0.136	0.218
Tan	X	26993701	26994000	0.319	0.773	0.082	0.380
Tan	X	27026101	27026400	0.131	0.539	0.239	0.274
Tan	X	27428701	27429000	0.400	0.704	0.325	0.329
Tan	X	27524401	27524700	0.266	0.515	0.214	0.279
Tan	X	27894001	27894300	0.342	0.916	0.296	0.472
Tan	X	28310101	28310400	0.184	0.513	0.185	0.223
Tan	X	28438801	28439100	0.053	0.680	0.296	0.238
Tan	X	28490701	28491000	0.156	0.468	0.107	0.142
Tan	X	28588501	28588800	0.558	0.444	0.267	0.086
Tan	X	28666201	28666500	0.477	1.234	0.575	0.669
Tan	X	28666501	28666800	0.690	1.356	0.560	0.806
Tan	X	28692901	28693200	0.161	0.706	0.259	0.304
Tan	X	28816801	28817100	0.131	0.511	0.160	0.162
Tan	X	28914901	28915200	0.398	1.468	0.374	0.435
Tan	X	29373301	29373600	0.634	1.313	0.687	0.806
Tan	X	29409301	29409600	0.106	0.701	0.054	0.309
Tan	X	29553001	29553300	0.715	0.959	0.399	0.304
Tan	X	29573701	29574000	0.398	0.846	0.379	0.411
Tan	X	29824801	29825100	0.398	0.515	0.346	0.416
Tan	X	30014401	30014700	0.158	0.423	0.078	0.137
Tan	X	30175201	30175500	0.211	0.721	0.272	0.248
Tan	X	30351001	30351300	2.450	2.944	2.521	2.236
Tan	X	30655801	30656100	0.876	1.146	0.745	0.943
Tan	X	31072801	31073100	0.240	0.071	0.745	0.603
Tan	X	31210501	31210800	0.026	0.579	0.029	0.076
Tan	X	31210801	31211100	0.105	0.420	0.029	0.081
Tan	X	34193401	34193700	4.135	2.736	3.990	3.985
Tan	X	34210801	34211100	0.957	0.584	0.852	1.024
Tan	X	34414501	34414800	0.052	0.466	0.078	0.254
Tan	X	34903201	34903500	2.396	4.418	2.561	2.545
Tan	X	35999701	36000000	0.606	1.433	0.526	0.704
Tan	X	36109801	36110100	0.163	0.841	0.243	0.274
Tan	X	36110701	36111000	0.132	0.401	0.132	0.112
Tan	X	36703501	36703800	0.558	0.865	1.407	0.405
Tan	X	36718501	36718800	0.552	1.783	0.573	0.629
Tan	X	36904201	36904500	1.970	0.970	1.781	1.851
Tan	X	36927001	36927300	0.215	0.513	0.263	0.162
Tan	X	37032601	37032900	0.158	0.747	0.259	0.167
Tan	X	37086001	37086300	0.321	0.772	0.181	0.213
Tan	X	37110001	37110300	0.211	0.719	0.346	0.329
Tan	X	37191001	37191300	0.187	0.442	0.292	0.112
Tan	X	37287001	37287300	1.166	0.605	1.275	1.024
Tan	X	37446601	37446900	0.903	1.452	0.974	0.933
Tan	X	37642501	37642800	0.265	0.845	0.399	0.142
Tan	X	37797601	37797900	0.134	0.468	0.214	0.162
Tan	X	38045701	38046000	0.213	0.654	0.337	0.279
Tan	X	38248801	38249100	0.424	0.609	0.321	0.218
Tan	X	38498401	38498700	0.134	0.395	0.078	0.112
Tan	X	38499301	38499600	0.239	0.652	0.185	0.355
Tan	X	38589001	38589300	0.769	0.470	0.905	1.105
Tan	X	38718301	38718600	0.771	0.330	0.132	0.193
Tan	X	38812801	38813100	0.429	1.266	0.515	0.425
Tan	X	39662101	39662400	0.374	0.212	0.531	0.056
Tan	X	39778201	39778500	0.239	0.515	0.210	0.335
Tan	X	39959401	39959700	0.160	0.515	0.176	0.112
Tan	X	40056601	40056900	0.108	0.371	0.029	0.025
Tan	X	40448701	40449000	0.531	1.008	0.555	0.578

Tan	X	40607101	40607400	0.584	0.187	0.399	0.329
Tan	X	41470201	41470500	0.503	0.863	0.486	0.553
Tan	X	41577601	41577900	0.132	0.446	0.321	0.112
Tan	X	44350501	44350800	0.534	1.200	0.531	0.193
Tan	X	44350801	44351100	0.797	1.450	0.658	0.284
Tan	X	44365801	44366100	1.195	0.981	0.745	0.771
Tan	X	44424901	44425200	0.319	0.493	0.296	0.664
Tan	X	44439301	44439600	1.781	2.673	1.962	1.800
Tan	X	44946901	44947200	0.134	0.468	0.160	0.279
Tan	X	45010801	45011100	0.321	0.773	0.473	0.472
Tan	X	45557701	45558000	0.134	0.511	0.189	0.031
Tan	X	46500301	46500600	0.237	0.586	0.156	0.228
Tan	X	46659301	46659600	0.081	0.442	0.156	0.051
Tan	X	47663701	47664000	0.318	0.094	0.214	0.608
Tan	X	47735401	47735700	1.008	1.925	1.139	0.847
Tan	X	47957101	47957400	1.276	0.768	1.188	1.572
Tan	X	49659901	49660200	0.531	1.453	0.390	0.517
Tan	X	49690201	49690500	0.292	0.536	0.156	0.335
Tan	X	49749301	49749600	0.294	0.633	0.317	0.411
Tan	X	49771501	49771800	0.347	0.586	0.189	0.162
Tan	X	50336101	50336400	0.268	0.702	0.239	0.385
Tan	X	50355901	50356200	0.211	0.420	0.239	0.198
Tan	X	51055501	51055800	0.295	1.094	0.354	0.350
Tan	X	51095401	51095700	0.926	2.006	0.667	0.938
Tan	X	51348301	51348600	0.105	0.444	0.107	0.092
Tan	X	51575701	51576000	0.907	0.354	0.802	0.715
Tan	X	51576001	51576300	1.140	0.399	0.881	1.176
Tan	X	51621901	51622200	1.091	1.776	0.983	0.963
Tan	X	51906901	51907200	0.319	0.584	0.210	0.268
Tan	X	52293001	52293300	0.400	0.770	0.408	0.279
Tan	X	52503301	52503600	0.213	0.442	0.107	0.132
Tan	X	52601401	52601700	0.611	0.395	0.827	0.857
Tan	X	52622101	52622400	2.234	1.639	1.917	1.962
Tan	X	52970101	52970400	1.176	2.272	0.927	1.055
Tan	X	53310601	53310900	0.424	0.957	0.633	0.517
Tan	X	53403601	53403900	0.136	0.446	0.136	0.193
Tan	X	53444101	53444400	0.158	0.515	0.160	0.132
Tan	X	54291901	54292200	0.240	0.466	0.218	0.137
Tan	X	54303601	54303900	0.239	0.562	0.317	0.329
Tan	X	54399301	54399600	0.481	0.090	0.477	0.547
Tan	X	54399601	54399900	0.557	0.068	0.432	0.416
Tan	X	54575701	54576000	0.560	0.820	0.564	0.416
Tan	X	55335301	55335600	1.116	0.513	1.099	1.172
Tan	X	55476301	55476600	0.158	0.472	0.029	0.056
Tan	X	55810201	55810500	0.105	0.446	0.156	0.162
Tan	X	57029101	57029400	0.373	0.702	0.379	0.441
Tan	X	57387001	57387300	0.292	0.538	0.132	0.284
Tan	X	57828001	57828300	0.106	0.581	0.292	0.187
Tan	X	57945301	57945600	0.240	0.609	0.296	0.279
Tan	X	57976801	57977100	0.053	0.510	0.082	0.101
Tan	X	57995701	57996000	0.213	1.077	0.221	0.112
Tan	X	58561201	58561500	0.824	0.702	0.185	0.578
Tan	X	58657501	58657800	1.199	1.725	1.012	1.298
Tan	X	58669501	58669800	0.345	0.631	0.428	0.385
Tan	X	58686001	58686300	0.347	0.564	0.210	0.248
Tan	X	58687801	58688100	0.211	0.446	0.025	0.248
Tan	X	58792501	58792800	0.213	0.538	0.350	0.274
Tan	X	58979401	58979700	0.186	0.420	0.210	0.086
Tan	X	59074201	59074500	1.121	1.759	0.493	0.771
Tan	X	59536501	59536800	0.187	0.420	0.189	0.117
Tan	X	59833801	59834100	0.213	0.684	0.098	0.142
Tan	X	59889301	59889600	0.855	1.431	0.444	0.690
Tan	X	60403801	60404100	0.345	0.912	0.374	0.314
Tan	X	60862501	60862800	0.557	0.957	0.502	0.644
Tan	X	61219501	61219800	0.189	0.541	0.078	0.193
Tan	X	61392601	61392900	0.213	0.657	0.259	0.304
Tan	X	61634101	61634400	0.371	0.678	0.127	0.268

Tan	X	62493901	62494200	0.213	0.395	0.160	0.248
Tan	X	62905801	62906100	0.266	0.702	0.218	0.421
Tan	X	63425401	63425700	0.187	0.468	0.025	0.061
Tan	X	63599101	63599400	0.878	1.264	0.925	0.832
Tan	X	63785701	63786000	0.268	0.657	0.218	0.218
Tan	X	64286101	64286400	0.186	0.468	0.132	0.056
Tan	X	64782001	64782300	0.211	0.442	0.214	0.218
Tan	X	65318101	65318400	0.773	0.959	1.378	0.664
Tan	X	65650201	65650500	0.132	0.350	0.506	0.553
Tan	X	65761801	65762100	0.240	0.493	0.296	0.304
Tan	X	66372601	66372900	0.824	0.539	0.292	0.441
Tan	X	66433501	66433800	0.294	0.611	0.296	0.279
Tan	X	66435301	66435600	1.837	3.274	2.151	2.130
Tan	X	66495301	66495600	0.160	0.470	0.132	0.081
Tan	X	66495601	66495900	0.106	0.444	0.132	0.106
Tan	X	66587701	66588000	0.668	1.075	0.473	0.588
Tan	X	66588001	66588300	0.479	0.863	0.399	0.345
Tan	X	66721801	66722100	1.731	0.519	1.893	2.049
Tan	X	66937501	66937800	0.507	0.232	0.555	0.497
Tan	X	67456501	67456800	3.296	2.879	3.315	3.519
Tan	X	67739401	67739700	0.265	0.517	0.292	0.304
Tan	X	69519001	69519300	0.105	0.444	0.107	0.025
Tan	X	69799201	69799500	0.744	0.562	0.395	0.329
Tan	X	70398001	70398300	0.347	0.493	0.078	0.025
Tan	X	70423201	70423500	0.134	0.515	0.189	0.117
Tan	X	71542801	71543100	0.053	0.395	0.049	0.193
Tan	X	71803501	71803800	0.398	0.678	0.531	0.365
Tan	X	71839501	71839800	0.266	0.564	0.317	0.254
Tan	X	73432501	73432800	0.321	0.538	0.317	0.248
Tan	X	74934301	74934600	0.131	0.612	0.029	0.076
Tan	X	74991601	74991900	0.398	0.513	0.292	0.335
Tan	X	75734701	75735000	1.034	1.564	1.177	0.943
Tan	X	75889801	75890100	0.158	0.493	0.185	0.279
Tan	X	76574701	76575000	3.109	4.493	3.899	3.336
Tan	X	76637401	76637700	0.373	0.069	0.539	0.502
Tan	X	76740601	76740900	0.771	0.654	0.399	0.497
Tan	X	76905901	76906200	1.326	2.410	0.580	1.688
Tan	X	76992301	76992600	1.252	0.489	1.465	1.075
Tan	X	77082301	77082600	0.586	1.240	0.531	0.938
Tan	X	77098201	77098500	0.319	0.702	0.444	0.106
Tan	X	77134801	77135100	0.799	1.171	0.642	0.801
Tan	X	77140201	77140500	1.432	0.320	1.164	1.197
Tan	X	77250301	77250600	1.331	1.946	1.300	1.465
Tan	X	77449201	77449500	2.686	3.015	1.815	2.500
Tan	X	77824201	77824500	0.426	0.843	0.419	0.365
Tan	X	77843401	77843700	0.692	0.935	0.399	0.654
Tan	X	77899201	77899500	0.184	0.980	0.453	0.553
Tan	X	78093601	78093900	0.931	0.204	0.885	0.806
Tan	X	78151201	78151500	0.055	0.562	0.098	0.106
Tan	X	78316201	78316500	0.266	0.564	0.317	0.162
Tan	X	78347101	78347400	0.586	0.980	0.774	0.547
Tan	X	78379201	78379500	0.134	0.562	0.267	0.416
Tan	X	78669001	78669300	0.237	0.820	0.457	0.324
Tan	X	78813901	78814200	0.215	0.468	0.292	0.137
Tan	X	79294801	79295100	0.507	0.210	0.399	0.659
Tan	X	79592101	79592400	0.318	0.749	0.370	0.279
Tan	X	79805101	79805400	0.502	1.165	0.646	0.669
Tan	X	79845001	79845300	0.079	0.560	0.132	0.182
Tan	X	79921501	79921800	0.503	0.328	0.136	0.527
Tan	X	80059201	80059500	0.265	0.935	0.588	0.370
Tan	X	80955601	80955900	0.423	0.727	0.428	0.274
Tan	X	81057901	81058200	0.210	0.513	0.136	0.086
Tan	X	81142501	81142800	0.242	1.203	0.107	0.203
Tan	X	81313801	81314100	1.174	0.607	1.456	1.166
Tan	X	81323401	81323700	0.184	0.468	0.185	0.025
Tan	X	82261201	82261500	0.161	0.491	0.292	0.279
Tan	X	82273801	82274100	0.398	0.562	0.243	0.416

Tan	X	82441201	82441500	0.160	0.564	0.107	0.391
Tan	X	83136001	83136300	0.292	0.635	0.214	0.243
Tan	X	84822001	84822300	0.239	0.656	0.419	0.355
Tan	X	85935001	85935300	0.405	1.006	0.259	0.198
Tan	X	86020501	86020800	0.106	0.470	0.111	0.162
Tan	X	87702001	87702300	0.186	0.581	0.321	0.167
Tan	X	87756901	87757200	0.211	0.468	0.132	0.243
Tan	X	88250401	88250700	0.371	0.633	0.292	0.355
Tan	X	88366801	88367100	1.628	0.863	1.761	2.195
Tan	X	88429501	88429800	0.186	0.493	0.239	0.329
Tan	X	88947001	88947300	0.160	0.538	0.160	0.274
Tan	X	89831401	89831700	0.292	0.421	0.506	0.248
Tan	X	90199501	90199800	0.163	0.650	0.000	0.106
Tan	X	90433201	90433500	0.345	0.562	0.317	0.309
Tan	X	90791701	90792000	0.479	0.560	0.346	0.497
Tan	X	90820201	90820500	0.187	0.420	0.103	0.162
Tan	X	90899101	90899400	0.155	0.586	0.107	0.162
Tan	X	90999001	90999300	0.453	0.863	0.317	0.492
Tan	X	91294801	91295100	0.132	0.493	0.185	0.218
Tan	X	91353901	91354200	0.213	0.418	0.054	0.137
Tan	X	91572901	91573200	0.428	0.654	0.243	0.441
Tan	X	91802401	91802700	0.105	0.515	0.160	0.228
Tan	X	92776501	92776800	0.187	0.444	0.218	0.112
Tan	X	93632401	93632700	0.397	0.631	0.346	0.360
Tan	X	93953701	93954000	2.662	2.242	2.925	3.108
Tan	X	94108501	94108800	0.106	0.489	0.189	0.279
Tan	X	94446001	94446300	0.768	1.306	0.453	0.502
Tan	X	95278501	95278800	0.505	0.654	0.317	0.162
Tan	X	95428501	95428800	0.079	0.560	0.263	0.411
Tan	X	95663401	95663700	0.608	0.234	0.658	0.751
Tan	X	95765101	95765400	0.690	0.234	0.481	0.416
Tan	X	95948401	95948700	0.263	0.560	0.156	0.198
Tan	X	96134401	96134700	0.319	0.584	0.132	0.391
Tan	X	96479101	96479400	0.158	0.510	0.107	0.086
Tan	X	96646801	96647100	0.605	1.496	0.493	0.731
Tan	X	96712201	96712500	0.290	0.539	0.132	0.167
Tan	X	96750001	96750300	0.374	0.837	0.419	0.254
Tan	X	96779101	96779400	0.345	0.092	0.477	0.274
Tan	X	97043701	97044000	0.211	0.824	0.176	0.117
Tan	X	97240201	97240500	0.105	0.519	0.156	0.162
Tan	X	97542001	97542300	0.213	0.656	0.218	0.203
Tan	X	97551001	97551300	0.265	0.560	0.234	0.299
Tan	X	98270401	98270700	3.938	4.632	3.545	3.686
Tan	X	98493901	98494200	0.027	0.442	0.165	0.081
Tan	X	98868601	98868900	0.345	0.773	0.263	0.167
Tan	X	99044101	99044400	0.239	0.611	0.272	0.187
Tan	X	99887401	99887700	0.215	1.034	0.439	0.477
Tan	X	100115101	100115400	1.491	1.009	0.560	0.263
Tan	X	100812301	100812600	0.160	0.448	0.214	0.193
Tan	X	100883401	100883700	0.239	0.586	0.160	0.360
Tan	X	101766301	101766600	4.067	3.060	4.055	4.477
Tan	X	102168301	102168600	0.213	0.511	0.218	0.193
Tan	X	102803401	102803700	0.536	0.910	0.506	0.411
Tan	X	102937801	102938100	0.079	0.517	0.317	0.248
Tan	X	102981601	102981900	0.136	0.727	0.082	0.000
Tan	X	103119901	103120200	0.108	0.493	0.210	0.223
Tan	X	103145401	103145700	0.105	0.470	0.185	0.112
Tan	X	103232101	103232400	1.434	0.959	1.601	1.410
Tan	X	103348501	103348800	0.266	0.800	0.267	0.421
Tan	X	103522201	103522500	0.132	0.401	0.132	0.142
Tan	X	103532401	103532700	0.376	0.820	0.399	0.411
Tan	X	106094101	106094400	0.586	0.167	0.424	0.527
Tan	X	106291501	106291800	0.240	0.470	0.185	0.137
Tan	X	106454101	106454400	0.715	1.386	0.526	0.547
Tan	X	107256001	107256300	0.215	0.609	0.288	0.274
Tan	X	107547601	107547900	0.373	0.468	0.136	0.360
Tan	X	107620801	107621100	0.027	0.532	0.054	0.086

Tan	X	107659501	107659800	1.094	0.281	0.881	1.004
Tan	X	108264301	108264600	0.345	0.820	0.374	0.365
Tan	X	108265501	108265800	0.134	0.727	0.218	0.502
Tan	X	108273901	108274200	0.239	0.466	0.296	0.112
Tan	X	108565501	108565800	0.160	0.588	0.267	0.243
Tan	X	109058701	109059000	0.373	0.940	0.194	0.477
Tan	X	109206901	109207200	0.537	1.169	0.526	0.320
Tan	X	109490701	109491000	0.400	0.817	0.239	0.472
Tan	X	110035201	110035500	0.850	0.418	0.127	0.162
Tan	X	111394201	111394500	2.474	4.971	2.624	0.999
Tan	X	111557101	111557400	0.186	0.493	0.239	0.254
Tan	X	111854701	111855000	0.587	0.049	1.235	0.623
Tan	X	113298901	113299200	0.186	0.581	0.267	0.274
Tan	X	113341501	113341800	0.345	0.678	0.243	0.350
Tan	X	114270601	114270900	0.505	0.841	0.486	0.304
Tan	X	114742501	114742800	0.795	1.051	0.510	0.720
Tan	X	115500001	115500300	0.689	0.277	1.008	1.019
Tan	X	115742401	115742700	0.716	1.453	0.711	0.618
Tan	X	115742701	115743000	0.508	1.103	0.428	0.502
Tan	X	117081301	117081600	0.347	1.480	0.490	0.324
Tan	X	117290101	117290400	0.160	0.560	0.239	0.279
Tan	X	117436501	117436800	0.239	0.654	0.214	0.411
Tan	X	117539101	117539400	0.294	0.635	0.185	0.187
Tan	X	117619201	117619500	0.929	1.480	0.765	0.573
Tan	X	117773101	117773400	1.623	1.167	0.905	1.613
Tan	X	117815701	117816000	0.160	1.465	0.901	0.558
Tan	X	117877201	117877500	0.211	0.702	0.301	0.167
Tan	X	118046401	118046700	0.505	0.865	0.424	0.466
Tan	X	119015401	119015700	0.105	0.635	0.189	0.213
Tan	X	119384101	119384400	0.532	0.116	0.296	0.299
Tan	X	119564101	119564400	0.026	0.511	0.296	0.056
Tan	X	120301201	120301500	0.053	0.444	0.107	0.162
Tan	X	120596401	120596700	0.290	0.656	0.370	0.304
Tan	X	121739101	121739400	0.321	0.069	0.526	0.335
Tan	X	121841401	121841700	0.103	0.560	0.243	0.167
Tan	X	122523901	122524200	0.477	0.375	0.185	0.223
Tan	X	122923501	122923800	0.797	0.564	1.197	0.857
Tan	X	123210601	123210900	0.189	0.704	0.341	0.248
Tan	X	123555601	123555900	0.211	0.628	0.205	0.223
Tan	X	124661701	124662000	0.795	1.455	0.760	0.675
Tan	X	124989601	124989900	0.105	0.448	0.136	0.051
Tan	X	125061901	125062200	0.239	0.489	0.127	0.223
Tan	X	125473501	125473800	0.321	0.770	0.354	0.279
Tan	X	126327901	126328200	0.398	0.727	0.165	0.248
Tan	X	126356701	126357000	0.163	0.515	0.078	0.162
Tan	X	126933901	126934200	0.874	1.380	0.892	0.801
Tan	X	127008601	127008900	0.106	0.423	0.136	0.193
Tan	X	127142401	127142700	0.371	0.140	0.560	0.279
Tan	X	127617001	127617300	0.374	0.820	0.243	0.142
Tan	X	127617301	127617600	0.266	0.491	0.234	0.137
Tan	X	127881601	127881900	0.292	0.560	0.103	0.391
Tan	X	128506201	128506500	0.718	1.290	0.584	0.624
Tan	X	128638201	128638500	0.479	0.910	0.568	0.522
Tan	X	128750701	128751000	0.610	0.257	0.078	0.522
Tan	X	129528901	129529200	0.852	1.410	0.905	0.882
Tan	X	130050601	130050900	1.279	0.654	1.271	1.125
Tan	X	130156801	130157100	0.187	0.442	0.156	0.167
Tan	X	130945501	130945800	0.266	0.583	0.243	0.360
Tan	X	131051101	131051400	0.318	0.657	0.267	0.193
Tan	X	131462401	131462700	0.208	0.818	0.160	0.284
Tan	X	131508301	131508600	0.210	0.865	0.087	0.137
Tan	X	133343401	133343700	0.081	0.444	0.185	0.167
StH	1	78901	79200	0.053	0.251	1.021	0.061
StH	1	101701	102000	0.260	0.725	2.332	0.295
StH	1	139201	139500	0.934	1.169	2.980	0.765
StH	1	151801	152100	0.850	0.545	1.975	0.735
StH	1	306001	306300	0.134	0.654	2.305	0.284

StH	1	337801	338100	2.278	3.267	6.406	3.001
StH	1	342301	342600	1.323	1.499	3.382	1.344
StH	1	342601	342900	0.134	0.229	0.740	0.248
StH	1	367201	367500	3.270	2.899	2.107	2.951
StH	1	453301	453600	0.340	0.836	4.364	0.812
StH	1	511501	511800	0.211	0.135	0.865	0.117
StH	1	520201	520500	0.158	0.705	2.597	0.320
StH	1	520501	520800	0.574	1.061	4.300	0.660
StH	1	552601	552900	0.952	0.227	2.330	0.395
StH	1	570601	570900	2.020	2.175	1.137	1.952
StH	1	608401	608700	0.160	0.204	0.896	0.167
StH	1	618601	618900	0.213	0.517	2.004	0.462
StH	1	645601	645900	0.027	0.023	0.658	0.025
StH	1	645901	646200	0.213	0.429	1.614	0.391
StH	1	720901	721200	0.161	0.068	0.996	0.207
StH	1	734701	735000	0.398	0.478	1.547	0.339
StH	1	757801	758100	0.160	0.161	0.802	0.000
StH	1	835801	836100	0.026	0.090	0.626	0.051
StH	1	853801	854100	0.398	0.499	2.940	0.178
StH	1	879301	879600	0.158	0.116	0.624	0.000
StH	1	890101	890400	0.081	0.225	1.569	0.106
StH	1	903301	903600	5.364	4.242	11.052	4.979
StH	1	907801	908100	0.053	0.090	0.597	0.092
StH	1	930001	930300	0.345	0.680	2.352	0.594
StH	1	936001	936300	0.591	0.800	2.390	0.553
StH	1	946201	946500	0.316	0.643	1.950	0.506
StH	1	995101	995400	0.482	0.736	1.875	0.624
StH	1	995401	995700	1.267	1.574	4.748	1.232
StH	1	1059901	1060200	0.694	0.418	1.090	0.745
StH	1	1060201	1060500	0.671	0.710	2.004	0.614
StH	1	1095901	1096200	0.605	0.834	2.409	0.608
StH	1	1156201	1156500	0.131	0.249	1.400	0.031
StH	1	1512301	1512600	3.254	3.731	5.510	2.793
StH	1	1565701	1566000	0.923	1.325	4.762	1.913
StH	1	1877401	1877700	1.145	1.053	1.790	0.922
StH	1	1904401	1904700	1.884	3.017	3.529	2.368
StH	1	1930801	1931100	1.355	2.242	1.052	1.962
StH	1	1938601	1938900	0.744	0.843	0.247	0.628
StH	1	1942201	1942500	2.746	2.713	7.748	2.433
StH	1	1949401	1949700	2.229	2.358	1.253	2.231
StH	1	1954801	1955100	0.905	0.978	0.205	1.111
StH	1	1957201	1957500	9.653	9.402	7.580	9.481
StH	1	1981201	1981500	1.750	1.450	0.885	1.532
StH	1	2039701	2040000	0.026	0.206	1.126	0.213
StH	1	2155801	2156100	0.992	0.558	2.198	0.836
StH	1	2177701	2178000	0.484	1.040	4.558	0.391
StH	1	2201401	2201700	0.235	0.613	2.423	0.608
StH	1	2326501	2326800	1.031	1.014	2.764	1.015
StH	1	2355301	2355600	2.416	2.010	1.209	2.014
StH	1	2535901	2536200	1.441	1.536	0.852	0.355
StH	1	2607901	2608200	2.401	1.785	3.957	0.542
StH	1	2657401	2657700	0.079	0.225	1.177	0.061
StH	1	2772301	2772600	2.604	3.150	3.888	2.900
StH	1	2783401	2783700	0.744	1.313	0.481	0.776
StH	1	2830501	2830800	0.987	1.199	0.029	1.455
StH	1	2830801	2831100	1.708	2.229	0.234	2.672
StH	1	2931001	2931300	0.587	0.577	1.565	0.563
StH	1	2933701	2934000	1.015	0.204	3.394	0.806
StH	1	2971801	2972100	0.455	0.590	2.147	0.289
StH	1	3052801	3053100	2.545	2.360	1.658	2.464
StH	1	3168301	3168600	0.532	0.281	0.346	0.223
StH	1	3221701	3222000	3.272	3.062	2.613	3.422
StH	1	3376201	3376500	0.481	0.538	0.058	0.299
StH	1	3501901	3502200	0.584	0.704	0.263	0.700
StH	1	3704401	3704700	3.297	2.699	1.258	3.620
StH	1	3945301	3945600	2.044	1.753	2.847	2.089
StH	1	4101601	4101900	2.550	3.486	1.761	3.580

StH	1	4337701	4338000	0.982	1.259	0.539	1.166
StH	1	4378501	4378800	0.610	0.536	1.115	0.472
StH	1	4380601	4380900	1.700	1.470	1.070	1.597
StH	1	4439401	4439700	0.773	1.077	1.465	1.197
StH	1	4505701	4506000	0.824	0.421	0.617	0.441
StH	1	4517401	4517700	0.527	0.787	1.608	0.669
StH	1	4566001	4566300	0.874	0.848	1.712	2.531
StH	1	4638901	4639200	1.481	1.289	0.403	1.318
StH	1	4717801	4718100	0.160	0.257	0.588	0.213
StH	1	4870201	4870500	2.124	2.015	1.567	1.029
StH	1	4952401	4952700	0.186	0.142	0.502	0.187
StH	1	5080201	5080500	4.886	3.742	6.088	4.742
StH	1	5238901	5239200	1.034	1.240	0.461	0.988
StH	1	5445001	5445300	2.367	1.365	0.678	1.424
StH	1	5445301	5445600	2.688	1.335	0.809	1.587
StH	1	5514301	5514600	0.826	0.697	0.341	0.776
StH	1	5543701	5544000	0.027	0.184	0.502	0.086
StH	1	5548201	5548500	0.531	0.466	0.156	0.385
StH	1	5582701	5583000	0.989	0.794	0.201	0.771
StH	1	5583001	5583300	0.987	0.536	0.181	0.583
StH	1	6344101	6344400	1.783	1.802	1.275	1.825
StH	1	6695101	6695400	3.050	3.324	2.196	3.275
StH	1	7295101	7295400	0.000	0.090	0.522	0.000
StH	1	7449001	7449300	0.716	0.867	0.288	0.695
StH	1	7630501	7630800	2.452	2.379	1.616	2.353
StH	1	7701601	7701900	0.932	0.940	0.317	0.948
StH	1	7728901	7729200	1.729	2.010	1.115	2.099
StH	1	7762201	7762500	0.505	0.420	0.049	0.517
StH	1	7946101	7946400	3.325	3.649	2.916	3.640
StH	1	8103301	8103600	0.450	0.397	0.716	0.274
StH	1	8119201	8119500	0.639	0.772	0.419	0.639
StH	1	8415901	8416200	0.902	0.725	0.448	0.715
StH	1	8421901	8422200	0.186	0.515	0.317	0.106
StH	1	8642701	8643000	2.575	2.420	3.718	2.023
StH	1	8643001	8643300	2.243	2.270	3.246	1.831
StH	1	8706601	8706900	0.235	0.300	0.905	0.142
StH	1	8759101	8759400	0.718	0.841	1.008	0.695
StH	1	9382201	9382500	0.052	0.140	0.477	0.198
StH	1	9449401	9449700	0.265	0.423	0.765	0.304
StH	1	9784201	9784500	0.053	0.180	1.103	0.147
StH	1	9791101	9791400	0.211	0.513	0.856	0.507
StH	1	9956701	9957000	1.171	1.148	0.909	1.521
StH	1	9976201	9976500	0.136	0.300	0.662	0.248
StH	1	10197001	10197300	2.500	2.264	1.543	2.423
StH	1	10201201	10201500	0.342	0.697	3.412	1.090
StH	1	10229101	10229400	0.082	0.092	0.419	0.112
StH	1	10252501	10252800	0.211	0.303	0.531	0.198
StH	1	10428601	10428900	1.752	1.240	0.852	1.242
StH	1	10964101	10964400	0.479	0.489	0.218	0.634
StH	1	11310601	11310900	0.608	1.169	0.214	0.594
StH	1	11336101	11336400	0.398	0.741	1.978	0.603
StH	1	11484301	11484600	0.292	0.326	0.535	0.299
StH	1	11513101	11513400	0.052	0.047	0.448	0.157
StH	1	11535301	11535600	1.831	1.901	1.025	2.069
StH	1	11737501	11737800	0.690	0.583	0.370	0.578
StH	1	11893201	11893500	0.558	0.513	0.399	0.527
StH	1	11898001	11898300	1.091	0.952	2.614	1.085
StH	1	11916601	11916900	0.398	0.466	0.267	0.527
StH	1	12199501	12199800	1.831	1.744	4.434	1.557
StH	1	12295501	12295800	1.144	1.262	1.407	1.496
StH	1	12507001	12507300	0.795	1.032	0.341	0.765
StH	1	12605701	12606000	0.637	0.444	0.267	0.497
StH	1	12763801	12764100	0.292	0.564	1.119	0.720
StH	1	12973201	12973500	1.116	1.214	0.749	0.999
StH	1	13470301	13470600	0.823	0.468	1.086	0.913
StH	1	13853701	13854000	0.611	0.328	0.399	0.553
StH	1	13977601	13977900	0.610	0.517	0.111	0.634

StH	1	13987201	13987500	4.748	3.733	1.016	4.196
StH	1	14094601	14094900	1.516	1.658	2.102	1.617
StH	1	14680201	14680500	0.768	0.867	0.403	0.801
StH	1	14701501	14701800	0.584	0.607	0.905	0.228
StH	1	14830501	14830800	0.369	0.185	0.716	0.329
StH	1	15107401	15107700	1.539	1.053	0.560	1.131
StH	1	15123601	15123900	1.061	0.846	1.716	2.327
StH	1	15300001	15300300	1.460	1.590	1.012	1.541
StH	1	15303001	15303300	0.424	0.539	0.185	0.385
StH	1	15422701	15423000	0.318	0.420	0.687	0.472
StH	1	15742501	15742800	0.692	0.723	0.370	0.882
StH	1	15765601	15765900	1.113	0.867	0.346	1.156
StH	1	15765901	15766200	0.531	0.562	0.267	0.887
StH	1	15772801	15773100	3.665	1.841	4.524	3.382
StH	1	15862801	15863100	1.436	1.495	0.711	1.288
StH	1	16025401	16025700	0.824	0.631	0.317	0.690
StH	1	16112701	16113000	0.747	0.699	1.353	0.761
StH	1	16489801	16490100	1.465	1.547	0.930	1.395
StH	1	16527601	16527900	2.683	3.613	1.016	2.109
StH	1	16541701	16542000	0.294	0.446	0.049	0.603
StH	1	17052901	17053200	1.580	1.540	2.624	1.267
StH	1	17326501	17326800	0.294	0.234	0.671	0.391
StH	1	17895601	17895900	1.942	2.409	1.407	1.694
StH	1	18047401	18047700	0.477	0.818	0.662	0.472
StH	1	18051901	18052200	0.132	0.652	2.713	0.228
StH	1	18163801	18164100	2.078	1.706	1.144	2.084
StH	1	18981901	18982200	1.991	2.405	1.538	1.982
StH	1	19062001	19062300	0.210	0.452	2.632	0.452
StH	1	19275001	19275300	0.908	0.817	0.210	0.608
StH	1	19314601	19314900	0.289	0.620	2.216	0.487
StH	1	19764901	19765200	1.329	1.394	0.473	1.201
StH	1	20210401	20210700	0.242	0.236	0.588	0.193
StH	1	20325301	20325600	0.584	0.935	0.078	0.588
StH	1	20334901	20335200	0.452	0.493	0.156	0.304
StH	1	20339401	20339700	2.852	2.551	1.502	3.021
StH	1	20728801	20729100	0.426	0.446	0.082	0.340
StH	1	21372601	21372900	1.008	0.841	0.156	0.812
StH	1	21665101	21665400	0.613	0.399	0.087	0.391
StH	1	21772201	21772500	0.129	0.182	0.827	0.031
StH	1	21772501	21772800	0.215	0.527	2.283	0.522
StH	1	21916501	21916800	4.389	4.306	4.793	3.402
StH	1	22224601	22224900	0.428	0.139	0.847	0.274
StH	1	22508101	22508400	1.199	1.144	1.489	1.019
StH	1	22646701	22647000	1.228	1.197	0.720	1.242
StH	1	22866601	22866900	0.398	0.281	0.687	0.365
StH	1	22882501	22882800	0.161	0.161	0.609	0.147
StH	1	22924501	22924800	0.428	0.657	0.000	0.365
StH	1	25543501	25543800	0.213	0.328	0.560	0.391
StH	1	25934701	25935000	0.584	0.538	0.263	0.553
StH	1	26411101	26411400	0.077	0.302	1.387	0.562
StH	1	26985901	26986200	2.233	2.500	1.382	2.053
StH	1	27187201	27187500	0.186	1.311	0.502	0.776
StH	1	27194401	27194700	0.266	0.466	0.107	0.274
StH	1	27343201	27343500	1.116	1.004	1.324	0.690
StH	1	27679801	27680100	0.344	0.326	0.613	0.360
StH	1	28089601	28089900	0.160	0.303	0.555	0.365
StH	1	28288801	28289100	0.610	0.489	0.292	0.776
StH	1	28385101	28385400	0.373	0.423	0.609	0.360
StH	1	28519501	28519800	0.108	0.163	0.613	0.025
StH	1	28658101	28658400	0.237	0.142	0.535	0.193
StH	1	28706401	28706700	2.736	1.702	4.180	2.215
StH	1	28787701	28788000	2.818	2.785	0.960	2.503
StH	1	28832701	28833000	0.397	0.444	0.802	0.466
StH	1	29005801	29006100	1.091	0.455	2.328	1.165
StH	1	29152501	29152800	0.558	0.442	1.086	0.452
StH	1	29705701	29706000	0.531	0.538	0.189	0.421
StH	1	30039901	30040200	0.640	0.863	0.296	0.745

StH	1	30096001	30096300	1.118	1.330	0.695	0.963
StH	1	30682201	30682500	0.584	0.652	0.189	0.416
StH	1	30948301	30948600	0.610	0.513	0.132	0.533
StH	1	30969001	30969300	0.290	0.187	0.613	0.213
StH	1	31178101	31178400	4.623	5.001	5.566	4.264
StH	1	31321801	31322100	0.187	0.165	0.510	0.228
StH	1	31380901	31381200	0.503	0.047	0.321	0.051
StH	1	31407601	31407900	1.276	1.450	0.934	1.724
StH	1	31549501	31549800	0.479	0.539	0.111	0.360
StH	1	31616701	31617000	0.426	0.493	0.160	0.857
StH	1	31617001	31617300	0.907	0.611	0.000	1.277
StH	1	31793101	31793400	0.161	0.142	0.535	0.051
StH	1	31986601	31986900	0.108	0.073	0.658	0.391
StH	1	33140701	33141000	0.637	0.564	0.876	0.248
StH	1	33147901	33148200	0.850	1.053	0.239	0.492
StH	1	33483901	33484200	0.694	0.680	0.181	1.313
StH	1	33543001	33543300	0.768	0.822	0.165	0.654
StH	1	33615301	33615600	0.319	0.371	0.054	0.279
StH	1	33846601	33846900	0.479	0.865	0.185	0.999
StH	1	33846901	33847200	0.560	0.539	0.296	0.745
StH	1	34037701	34038000	0.640	0.863	1.148	0.751
StH	1	34139701	34140000	0.239	0.444	0.082	0.472
StH	1	35814001	35814300	0.292	0.300	0.584	0.112
StH	1	36318601	36318900	1.463	1.452	0.736	1.430
StH	1	36453001	36453300	0.239	0.371	0.745	0.243
StH	1	36639301	36639600	0.106	0.212	0.847	0.279
StH	1	37676401	37676700	0.479	0.493	0.214	0.578
StH	1	38553001	38553300	2.576	3.175	2.102	2.956
StH	1	38580001	38580300	2.313	2.806	1.884	2.367
StH	1	38987401	38987700	0.344	0.371	0.054	0.335
StH	1	39591901	39592200	0.718	0.305	0.477	0.826
StH	1	40201501	40201800	0.105	0.210	0.930	0.431
StH	1	40729201	40729500	0.692	0.656	0.263	0.533
StH	1	41192401	41192700	0.213	0.260	0.604	0.223
StH	1	41302801	41303100	0.000	0.047	0.497	0.142
StH	1	42292201	42292500	0.532	0.863	0.214	0.385
StH	1	42667801	42668100	0.106	0.118	0.000	0.522
StH	1	43145101	43145400	0.398	0.517	0.111	0.360
StH	1	43432801	43433100	0.665	0.678	0.856	0.274
StH	1	45867001	45867300	1.091	1.004	0.642	0.913
StH	1	45993601	45993900	0.239	0.444	0.132	0.279
StH	1	47106601	47106900	0.292	0.258	0.025	0.578
StH	1	47649301	47649600	0.160	0.420	0.054	0.162
StH	1	48235501	48235800	1.644	1.356	0.876	1.445
StH	1	51393601	51393900	0.477	0.468	0.802	0.436
StH	1	52133401	52133700	0.134	0.140	0.515	0.112
StH	1	54091501	54091800	0.053	0.069	0.506	0.051
StH	1	54822001	54822300	1.944	1.806	4.204	1.518
StH	1	54901801	54902100	1.302	1.687	2.044	1.714
StH	1	55173301	55173600	0.081	0.420	0.185	0.081
StH	1	57198901	57199200	0.266	0.303	0.769	0.218
StH	1	57652501	57652800	0.606	0.519	1.415	0.391
StH	1	57652801	57653100	0.606	0.470	1.732	0.472
StH	1	57833101	57833400	0.106	0.116	0.419	0.031
StH	1	58113901	58114200	0.611	0.609	0.301	1.014
StH	1	58259701	58260000	0.426	0.607	0.185	0.553
StH	1	59651401	59651700	0.053	0.139	0.568	0.193
StH	1	60009301	60009600	1.005	1.731	3.390	1.186
StH	1	60191701	60192000	0.292	0.352	0.736	0.254
StH	1	60941401	60941700	0.158	0.139	0.539	0.193
StH	1	61410301	61410600	0.373	0.538	0.082	0.355
StH	1	61619101	61619400	0.345	0.489	0.638	0.304
StH	1	62195101	62195400	0.213	0.302	0.609	0.203
StH	1	62901601	62901900	4.464	5.619	6.588	5.436
StH	1	63180901	63181200	0.240	0.212	0.502	0.076
StH	1	64091101	64091400	0.373	0.305	0.506	0.193
StH	1	64791001	64791300	0.211	0.258	0.535	0.106

StH	1	65573401	65573700	0.774	0.822	0.107	0.547
StH	1	65945401	65945700	0.215	0.069	0.564	0.112
StH	1	66628501	66628800	0.081	0.468	0.288	0.056
StH	1	66969001	66969300	0.184	0.187	0.745	0.254
StH	1	67226101	67226400	1.091	1.165	0.662	1.085
StH	1	67248001	67248300	0.318	1.077	0.629	0.329
StH	1	67317601	67317900	0.132	0.187	0.473	0.218
StH	1	67975201	67975500	0.858	1.019	2.845	1.593
StH	1	68977201	68977500	2.365	2.227	1.665	2.490
StH	1	69476701	69477000	0.452	0.446	0.185	0.391
StH	1	70006801	70007100	0.692	0.818	0.531	0.634
StH	1	70334701	70335000	0.797	0.468	0.370	0.634
StH	1	70441201	70441500	1.197	1.169	0.798	1.369
StH	1	70921801	70922100	0.237	0.376	0.720	0.223
StH	1	72387901	72388200	0.826	0.987	0.350	0.918
StH	1	72531301	72531600	0.213	0.161	0.638	0.198
StH	1	72874501	72874800	1.034	0.773	0.317	0.715
StH	1	74577601	74577900	0.505	0.489	0.185	0.411
StH	1	75195601	75195900	0.931	0.976	1.485	0.958
StH	1	75350701	75351000	0.986	0.820	1.382	0.781
StH	1	75780001	75780300	0.398	0.468	1.050	0.365
StH	1	76507201	76507500	0.557	0.258	0.802	0.502
StH	1	76657201	76657500	0.529	0.656	0.296	0.031
StH	1	77746201	77746500	0.853	1.250	4.271	1.234
StH	1	78263101	78263400	0.477	0.303	0.132	0.355
StH	1	80026801	80027100	0.265	0.234	0.716	0.254
StH	1	80671501	80671800	0.745	0.865	0.292	0.603
StH	1	80954101	80954400	0.136	0.772	0.395	0.142
StH	1	81345001	81345300	0.266	0.116	0.580	0.385
StH	1	81461401	81461700	0.690	0.843	0.428	0.695
StH	1	81689701	81690000	0.479	0.657	0.165	0.441
StH	1	81939001	81939300	0.215	0.189	0.531	0.187
StH	1	82204501	82204800	0.344	0.330	0.930	0.319
StH	1	82988101	82988400	0.507	0.513	0.399	1.273
StH	1	83700301	83700600	0.366	0.654	2.592	0.432
StH	1	83889901	83890200	0.402	0.330	0.716	0.254
StH	1	84055201	84055500	0.557	0.444	0.239	0.441
StH	1	84688801	84689100	0.318	0.446	0.916	0.304
StH	1	84921001	84921300	0.027	0.189	0.646	0.112
StH	1	85241701	85242000	0.823	0.676	0.395	0.690
StH	1	85715101	85715400	8.208	8.960	6.471	8.483
StH	1	85822501	85822800	0.266	0.656	0.399	0.279
StH	1	86034001	86034300	0.345	0.423	0.107	0.304
StH	1	86079601	86079900	1.326	0.980	0.642	0.974
StH	1	86135101	86135400	0.929	1.262	3.091	0.872
StH	1	86262901	86263200	0.611	0.682	0.296	0.639
StH	1	86319601	86319900	1.408	1.165	0.774	1.019
StH	1	86831701	86832000	0.371	0.420	0.107	0.304
StH	1	86850301	86850600	0.558	0.564	0.341	0.664
StH	1	87077701	87078000	0.479	0.584	0.856	0.441
StH	1	87092101	87092400	0.187	0.139	0.506	0.309
StH	1	87772501	87772800	3.060	2.708	3.600	2.515
StH	1	88043101	88043400	0.453	0.912	0.711	0.913
StH	1	88380001	88380300	0.671	0.564	1.473	0.654
StH	1	88503601	88503900	1.570	1.101	0.687	1.293
StH	1	88747801	88748100	0.560	0.564	0.346	0.690
StH	1	88866001	88866300	0.610	0.536	0.243	0.472
StH	1	89253601	89253900	0.453	0.399	0.691	0.862
StH	1	89673601	89673900	2.579	2.603	1.349	2.514
StH	1	90100501	90100800	0.081	0.562	0.239	0.329
StH	1	90345901	90346200	2.478	2.255	1.378	2.337
StH	1	90555601	90555900	0.318	0.444	0.054	0.274
StH	1	90555901	90556200	0.450	0.470	0.082	0.391
StH	1	91883101	91883400	0.611	0.493	0.156	0.304
StH	1	91974001	91974300	0.026	0.045	0.419	0.086
StH	1	92156401	92156700	2.020	2.386	1.431	1.638
StH	1	92217901	92218200	0.347	0.444	0.214	0.360

StH	1	92731801	92732100	1.008	1.103	0.584	0.943
StH	1	92789701	92790000	0.455	0.725	0.214	0.608
StH	1	92872801	92873100	1.199	1.285	0.724	1.192
StH	1	92885401	92885700	3.029	3.308	2.060	3.595
StH	1	93174001	93174300	2.283	2.731	1.723	2.185
StH	1	94187101	94187400	1.008	1.281	0.424	1.145
StH	1	94370701	94371000	0.184	0.330	0.720	0.355
StH	1	94864201	94864500	0.931	1.068	1.699	0.542
StH	1	94956001	94956300	0.981	0.676	1.563	0.598
StH	1	95431201	95431500	0.423	0.352	0.000	0.248
StH	1	95439301	95439600	0.455	0.517	0.896	0.329
StH	1	95759401	95759700	0.079	0.045	0.502	0.187
StH	1	96094501	96094800	0.160	0.212	0.448	0.142
StH	1	96219601	96219900	0.321	0.800	0.000	0.365
StH	1	96351901	96352200	0.400	0.421	0.642	0.304
StH	1	96427801	96428100	3.055	2.993	1.900	2.666
StH	1	96681901	96682200	1.307	1.337	2.004	1.136
StH	1	96829201	96829500	0.345	0.466	0.823	0.360
StH	1	97270801	97271100	0.371	0.560	0.185	0.771
StH	1	97322401	97322700	0.187	0.350	0.502	0.193
StH	1	97329301	97329600	0.586	0.609	0.132	0.355
StH	1	97650301	97650600	0.720	0.489	1.280	0.771
StH	1	97791901	97792200	0.477	0.328	0.132	0.304
StH	1	97946401	97946700	0.160	0.189	0.497	0.106
StH	1	98212501	98212800	1.192	0.798	0.259	0.715
StH	1	98661001	98661300	4.767	4.774	3.520	4.847
StH	1	98674201	98674500	0.586	1.165	0.881	0.472
StH	1	98957101	98957400	0.799	1.051	0.399	0.628
StH	1	99012601	99012900	0.742	1.169	1.489	1.136
StH	1	99154801	99155100	3.313	2.854	1.830	3.108
StH	1	99248101	99248400	0.345	0.236	0.531	0.248
StH	1	99437401	99437700	7.045	6.769	5.397	6.656
StH	1	99819301	99819600	0.373	0.491	0.054	0.243
StH	1	100169701	100170000	0.742	0.513	0.136	0.857
StH	1	100422301	100422600	1.436	1.352	0.856	1.105
StH	1	101680501	101680800	2.768	4.108	2.122	3.219
StH	1	101737801	101738100	0.052	0.024	0.448	0.112
StH	1	101901001	101901300	0.371	0.515	0.823	0.294
StH	1	102398401	102398700	0.294	0.493	0.082	0.279
StH	1	102567301	102567600	0.321	0.832	2.822	0.547
StH	1	103204501	103204800	0.560	0.628	1.066	0.517
StH	1	103231801	103232100	1.494	1.568	0.753	1.465
StH	1	103270801	103271100	0.615	0.957	0.243	0.634
StH	1	103477201	103477500	2.071	2.414	1.222	1.891
StH	1	103729201	103729500	0.290	0.330	0.054	0.603
StH	1	103911601	103911900	0.158	0.251	1.320	0.725
StH	1	103911901	103912200	0.210	0.118	1.570	0.437
StH	1	103980301	103980600	1.247	1.195	0.490	1.156
StH	1	104676901	104677200	0.297	0.900	3.315	0.735
StH	1	104727601	104727900	1.092	0.470	0.749	1.217
StH	1	104729701	104730000	2.391	2.487	0.745	2.227
StH	1	104734201	104734500	0.690	0.798	0.156	0.436
StH	1	104830201	104830500	0.106	0.384	1.692	0.061
StH	1	104831101	104831400	0.611	0.659	0.210	0.583
StH	1	104832601	104832900	0.186	0.137	0.662	0.086
StH	1	104932801	104933100	0.750	0.594	2.419	0.248
StH	1	104945401	104945700	0.371	0.472	0.852	0.365
StH	1	104945701	104946000	0.349	0.527	2.524	0.345
StH	1	105095701	105096000	3.658	3.766	2.426	4.310
StH	1	105408601	105408900	0.082	0.071	0.419	0.081
StH	1	105465001	105465300	0.476	0.875	1.930	0.396
StH	1	105511801	105512100	0.134	0.305	0.477	0.304
StH	1	105720301	105720600	1.142	1.403	0.934	0.466
StH	1	105771001	105771300	0.292	0.444	0.054	0.218
StH	1	105794101	105794400	0.931	0.935	0.691	0.634
StH	1	106607101	106607400	0.158	0.444	0.292	0.167
StH	1	106975801	106976100	0.400	0.352	0.103	0.547

StH	1	107109601	107109900	1.091	1.077	0.633	0.852
StH	1	107803801	107804100	1.458	1.341	0.765	2.322
StH	1	108352801	108353100	1.439	1.273	3.891	1.171
StH	1	108388801	108389100	0.371	0.376	0.642	0.385
StH	1	108423901	108424200	0.189	0.818	1.337	0.456
StH	1	109154101	109154400	0.797	1.009	1.476	0.968
StH	1	109413601	109413900	3.902	3.366	2.472	3.889
StH	1	110060701	110061000	0.373	0.420	0.555	0.385
StH	1	110408401	110408700	0.132	0.210	0.720	0.461
StH	1	110408701	110409000	0.158	0.189	0.662	0.411
StH	1	110815501	110815800	1.642	1.330	2.847	3.712
StH	1	111483601	111483900	0.426	0.212	0.609	0.137
StH	1	111854701	111855000	0.965	0.399	1.641	0.639
StH	1	111859201	111859500	2.847	3.574	1.514	2.301
StH	1	112036201	112036500	0.132	0.096	0.444	0.031
StH	1	112715401	112715700	0.105	0.163	0.419	0.137
StH	1	112730401	112730700	0.000	0.120	0.473	0.051
StH	1	112846201	112846500	0.027	0.090	0.593	0.056
StH	1	112847101	112847400	0.027	0.362	1.415	0.112
StH	1	113199901	113200200	0.216	1.455	0.749	0.076
StH	1	113322301	113322600	0.319	0.139	0.613	0.051
StH	1	113621701	113622000	0.290	0.375	0.716	0.472
StH	1	113981101	113981400	0.611	0.609	0.424	0.329
StH	1	114039901	114040200	0.239	0.232	0.526	0.137
StH	1	114587701	114588000	0.400	0.395	0.972	0.396
StH	1	114636601	114636900	1.410	0.307	1.897	0.608
StH	1	114711301	114711600	0.747	1.075	0.477	0.690
StH	1	115719301	115719600	0.481	0.536	0.181	0.466
StH	1	115859401	115859700	0.771	0.727	0.477	0.801
StH	1	115942201	115942500	0.292	0.584	0.160	0.355
StH	1	115977001	115977300	0.692	0.727	0.506	0.826
StH	1	116001301	116001600	0.345	0.444	0.132	0.365
StH	1	116025601	116025900	0.402	0.727	0.029	0.350
StH	1	116376001	116376300	0.371	0.607	0.214	0.360
StH	1	116461801	116462100	0.156	0.352	1.090	0.538
StH	1	116835301	116835600	0.184	0.184	0.555	0.132
StH	1	116865601	116865900	2.489	2.283	0.752	2.353
StH	1	117592201	117592500	0.106	0.000	0.515	0.101
StH	1	117619201	117619500	0.105	0.071	0.486	0.081
StH	1	117688801	117689100	0.373	0.399	0.691	0.436
StH	1	117817501	117817800	0.371	0.187	0.535	0.081
StH	1	118894801	118895100	5.680	2.886	4.541	3.498
StH	1	118975801	118976100	1.195	1.191	1.547	1.303
StH	1	119089501	119089800	7.266	7.267	6.318	7.853
StH	1	119188501	119188800	0.510	0.936	1.373	0.882
StH	1	119204701	119205000	7.448	7.409	5.275	7.431
StH	1	119205001	119205300	7.642	7.779	5.110	7.500
StH	1	119851801	119852100	1.224	1.356	0.831	1.348
StH	1	119946901	119947200	2.179	2.159	2.686	3.210
StH	1	120330301	120330600	1.091	0.867	0.584	0.806
StH	1	120366901	120367200	2.471	3.092	1.839	2.864
StH	1	120502801	120503100	0.666	0.513	0.979	0.639
StH	1	120914401	120914700	0.314	0.302	0.914	0.294
StH	1	120961201	120961500	0.426	0.611	0.798	0.497
StH	1	121258201	121258500	3.559	3.924	2.591	4.062
StH	1	121263301	121263600	5.288	5.986	2.200	6.045
StH	1	121500001	121500300	0.266	0.373	0.667	0.380
StH	1	121699201	121699500	0.637	0.584	0.292	0.634
StH	1	121744201	121744500	0.958	1.075	0.497	0.751
StH	1	121784701	121785000	2.397	1.939	1.333	1.719
StH	1	121802701	121803000	1.999	2.178	0.731	1.450
StH	1	122359501	122359800	0.132	0.420	0.267	0.385
StH	1	122475301	122475600	0.081	0.167	0.477	0.051
StH	1	122587801	122588100	3.562	2.894	2.118	2.718
StH	1	122786701	122787000	0.345	0.330	0.160	0.527
StH	1	123213901	123214200	0.347	0.403	0.979	0.315
StH	1	124470901	124471200	0.694	0.749	0.321	0.756

StH	1	124746301	124746600	0.187	0.185	0.473	0.081
StH	1	124901701	124902000	0.319	0.399	0.103	0.522
StH	1	127253101	127253400	1.097	1.144	0.506	1.237
StH	1	127291201	127291500	0.266	0.258	0.905	0.416
StH	1	127581301	127581600	1.834	1.708	1.148	2.002
StH	1	128359501	128359800	0.611	0.421	0.292	0.360
StH	1	128562901	128563200	0.774	0.798	0.350	0.740
StH	1	128869201	128869500	1.010	0.657	0.341	0.639
StH	1	130046101	130046400	0.426	0.399	0.292	0.497
StH	1	130623301	130623600	0.240	0.255	0.477	0.112
StH	1	131323501	131323800	0.395	0.399	0.852	0.360
StH	1	132633001	132633300	0.239	0.257	0.638	0.355
StH	1	134556001	134556300	0.266	0.096	0.555	0.173
StH	1	134934301	134934600	2.202	2.347	1.275	2.139
StH	1	135449101	135449400	0.160	0.210	0.448	0.173
StH	1	136163701	136164000	0.532	0.981	0.667	0.385
StH	1	136722301	136722600	0.186	0.236	0.477	0.142
StH	1	136804201	136804500	0.132	0.234	0.486	0.056
StH	1	137695201	137695500	0.479	0.277	0.745	0.309
StH	1	137796301	137796600	0.637	0.628	0.214	0.497
StH	1	138424801	138425100	0.132	0.281	0.510	0.031
StH	1	139353601	139353900	0.799	0.867	0.506	1.085
StH	1	141592201	141592500	0.690	0.423	0.239	0.639
StH	1	142258501	142258800	0.373	0.444	0.029	0.558
StH	1	142332601	142332900	0.081	0.118	0.675	0.117
StH	1	142342201	142342500	0.161	0.068	0.473	0.193
StH	1	142554601	142554900	0.721	1.375	1.991	1.425
StH	1	143211301	143211600	0.082	0.090	0.535	0.051
StH	1	143477101	143477400	1.170	1.335	0.930	0.608
StH	1	143501401	143501700	0.426	0.303	0.609	0.117
StH	1	144817501	144817800	0.239	0.420	0.082	0.274
StH	1	144843001	144843300	0.106	0.092	0.473	0.162
StH	1	145202101	145202400	0.345	0.584	0.481	0.360
StH	1	145429201	145429500	1.918	1.465	0.382	1.896
StH	1	145753801	145754100	0.268	0.118	0.753	0.299
StH	1	145874701	145875000	0.213	0.213	0.580	0.218
StH	1	146044201	146044500	0.239	0.210	0.617	0.092
StH	1	146226001	146226300	0.715	0.283	1.061	0.776
StH	1	146535601	146535900	0.186	0.165	0.473	0.198
StH	1	147871201	147871500	0.637	1.551	0.082	0.573
StH	1	147871501	147871800	0.771	1.639	0.058	0.771
StH	1	148190401	148190700	0.455	0.823	2.900	0.452
StH	1	148518901	148519200	0.450	0.560	0.078	0.335
StH	1	150013501	150013800	0.081	0.090	0.444	0.086
StH	1	150097801	150098100	0.106	0.234	0.419	0.193
StH	1	150101101	150101400	0.215	0.352	0.633	0.254
StH	1	151562401	151562700	0.347	0.375	0.584	0.360
StH	1	153024001	153024300	0.929	0.543	1.304	0.882
StH	1	154568401	154568700	3.672	1.538	2.793	1.861
StH	1	155714101	155714400	1.331	1.382	0.881	1.131
StH	1	156834601	156834900	0.187	0.258	0.629	0.167
StH	1	156965101	156965400	0.424	0.491	0.160	0.751
StH	1	158319601	158319900	0.637	0.611	0.399	0.634
StH	1	158476201	158476500	0.292	0.328	0.560	0.117
StH	1	160040401	160040700	0.426	0.423	0.000	0.461
StH	1	160611001	160611300	0.663	0.583	0.987	0.472
StH	1	161838601	161838900	0.079	0.165	0.477	0.101
StH	1	162114601	162114900	2.133	2.483	1.509	2.327
StH	1	162313801	162314100	0.053	0.047	0.584	0.223
StH	1	162472501	162472800	0.716	0.373	0.103	0.446
StH	1	163710001	163710300	1.041	1.118	0.439	1.328
StH	1	163717201	163717500	0.160	0.586	0.346	0.162
StH	1	163752901	163753200	0.211	0.279	0.613	0.117
StH	1	164190001	164190300	1.165	1.150	0.468	1.273
StH	1	164491801	164492100	0.160	0.116	0.526	0.126
StH	1	164547901	164548200	0.239	0.418	0.025	0.248
StH	1	164874601	164874900	0.213	0.208	0.568	0.137

StH	1	164973601	164973900	0.158	0.375	0.555	0.329
StH	1	165135601	165135900	0.398	0.440	0.934	0.614
StH	1	165578101	165578400	0.345	0.234	0.633	0.472
StH	1	166101301	166101600	0.182	0.185	0.745	0.243
StH	1	166139401	166139700	1.358	1.581	0.736	1.881
StH	1	166279501	166279800	0.187	0.187	0.613	0.193
StH	1	166530601	166530900	0.081	0.163	0.448	0.223
StH	1	166647001	166647300	0.265	0.236	0.477	0.274
StH	1	166978201	166978500	0.561	0.258	0.909	0.614
StH	1	167684701	167685000	0.106	0.163	0.477	0.203
StH	1	167990101	167990400	0.160	0.236	0.580	0.213
StH	1	168707401	168707700	0.187	0.118	0.448	0.031
StH	1	169254301	169254600	0.131	0.023	0.448	0.112
StH	1	169705801	169706100	0.265	0.631	0.049	0.243
StH	1	170896201	170896500	5.408	5.684	3.997	5.542
StH	1	172341601	172341900	0.345	0.375	0.239	0.522
StH	1	172357801	172358100	0.295	0.146	1.028	0.086
StH	1	173088001	173088300	0.160	0.142	0.642	0.162
StH	1	173163601	173163900	0.345	0.513	1.032	0.608
StH	1	175856701	175857000	0.186	0.139	0.809	0.162
StH	1	175857001	175857300	0.187	0.094	0.584	0.137
StH	1	175898101	175898400	0.666	0.657	0.160	0.492
StH	1	176244601	176244900	0.584	0.210	0.078	0.329
StH	1	177142501	177142800	1.544	1.264	0.876	1.653
StH	1	177189001	177189300	1.889	1.189	1.616	1.334
StH	1	177202501	177202800	0.347	0.654	0.506	0.639
StH	1	177301201	177301500	1.089	1.455	0.609	1.085
StH	1	177660601	177660900	0.319	0.165	0.588	0.106
StH	1	180780901	180781200	0.800	0.910	1.168	0.776
StH	1	181158301	181158600	0.239	0.584	0.477	0.137
StH	1	181715101	181715400	0.106	0.142	0.555	0.279
StH	1	182119201	182119500	1.723	0.772	2.620	1.785
StH	1	182498101	182498400	0.611	0.652	0.292	0.725
StH	1	182924701	182925000	1.305	1.521	1.830	1.415
StH	1	182968201	182968500	2.391	3.042	1.295	2.357
StH	1	183276001	183276300	0.055	0.096	0.510	0.081
StH	1	184288201	184288500	0.531	0.609	0.205	0.588
StH	1	184761901	184762200	1.168	0.798	0.395	0.781
StH	1	184817701	184818000	1.412	1.609	0.769	2.099
StH	1	184968601	184968900	0.639	0.654	0.395	0.806
StH	1	185286601	185286900	1.408	1.448	1.850	1.323
StH	1	185524201	185524500	0.027	0.000	0.361	0.000
StH	1	185969701	185970000	0.397	0.747	0.185	0.776
StH	1	185971801	185972100	0.479	0.654	1.052	0.634
StH	1	186163201	186163500	0.187	0.120	0.473	0.167
StH	1	186439201	186439500	0.666	0.770	0.263	0.644
StH	1	186758401	186758700	0.477	0.539	0.160	0.492
StH	1	186822301	186822600	1.011	0.631	0.531	0.441
StH	1	186822601	186822900	0.503	0.491	0.160	0.452
StH	1	187171501	187171800	0.239	0.421	0.662	0.502
StH	1	187504501	187504800	0.027	0.585	4.063	0.031
StH	1	187832101	187832400	0.186	0.373	0.535	0.162
StH	1	188031301	188031600	0.507	0.470	0.239	0.466
StH	1	188697301	188697600	1.324	1.822	2.406	1.912
StH	1	188720101	188720400	0.242	0.258	0.707	0.193
StH	1	189034201	189034500	2.102	1.944	1.200	2.104
StH	1	189726301	189726600	1.432	1.448	2.205	1.444
StH	1	190369801	190370100	0.426	0.772	0.049	0.355
StH	1	190621501	190621800	0.508	0.517	0.058	0.695
StH	1	190690801	190691100	0.106	0.094	0.054	0.497
StH	1	190703101	190703400	0.802	0.768	1.291	0.268
StH	1	190906801	190907100	0.797	0.933	0.379	0.781
StH	1	191702401	191702700	0.611	0.796	0.346	0.603
StH	1	191972701	191973000	0.371	0.189	1.037	0.152
StH	1	192068701	192069000	0.158	0.545	1.173	0.259
StH	1	192069001	192069300	0.211	0.380	1.647	0.482
StH	1	192509401	192509700	1.726	2.156	0.836	1.916

StH	1	193063201	193063500	0.690	0.770	1.066	0.710
StH	1	195059101	195059400	1.089	0.676	0.453	0.887
StH	1	196162201	196162500	0.161	0.279	0.794	0.259
StH	1	197138101	197138400	0.134	0.206	0.555	0.218
StH	1	197357401	197357700	0.160	0.281	0.477	0.279
StH	1	197403601	197403900	2.075	2.208	1.295	2.043
StH	1	197893201	197893500	1.066	0.515	1.634	1.004
StH	1	197961001	197961300	0.450	0.628	1.037	0.522
StH	1	198230401	198230700	0.316	0.185	0.716	0.223
StH	1	198682501	198682800	0.160	0.112	1.329	0.137
StH	1	198682801	198683100	0.187	0.159	1.028	0.132
StH	1	198778501	198778800	0.747	0.770	1.115	0.796
StH	1	198838801	198839100	1.063	1.126	0.633	0.999
StH	1	198867901	198868200	3.491	2.131	6.114	3.400
StH	1	199382701	199383000	0.106	0.187	0.502	0.274
StH	1	199433401	199433700	0.132	0.069	0.419	0.142
StH	1	199450501	199450800	0.824	0.723	0.230	0.751
StH	1	200187301	200187600	0.611	1.030	0.210	0.791
StH	1	200298901	200299200	0.532	0.633	0.181	0.411
StH	1	200487601	200487900	0.532	0.493	0.288	0.472
StH	1	200604901	200605200	0.450	0.493	0.185	0.522
StH	1	200605201	200605500	0.476	0.493	0.049	0.421
StH	1	200709601	200709900	0.213	0.583	0.872	0.466
StH	1	200798101	200798400	1.039	1.240	0.580	1.131
StH	1	200903701	200904000	0.799	0.745	0.296	0.816
StH	1	200908801	200909100	0.239	0.444	0.370	0.248
StH	1	201022201	201022500	1.200	1.757	3.324	1.592
StH	1	201058201	201058500	1.833	2.596	2.078	2.601
StH	1	201715801	201716100	3.113	3.154	2.296	1.785
StH	1	202091101	202091400	0.876	0.796	0.325	0.614
StH	1	202152001	202152300	0.105	0.232	0.535	0.132
StH	1	202290601	202290900	1.542	1.569	1.066	1.430
StH	1	203520001	203520300	0.558	0.534	0.185	0.781
StH	1	203522101	203522400	0.848	1.099	0.359	1.192
StH	1	204190801	204191100	0.586	0.772	1.115	0.644
StH	1	204843001	204843300	0.292	0.491	0.613	0.304
StH	1	205043101	205043400	0.239	0.468	0.107	0.299
StH	1	206306401	206306700	0.215	0.281	0.526	0.279
StH	1	206559901	206560200	0.189	0.208	0.609	0.299
StH	1	206572201	206572500	0.876	0.959	0.617	1.024
StH	1	206703601	206703900	0.215	0.258	0.531	0.187
StH	1	206735701	206736000	0.239	0.258	0.716	0.279
StH	1	206756401	206756700	0.211	0.047	0.588	0.248
StH	1	207542701	207543000	0.505	0.328	0.239	0.167
StH	1	207997201	207997500	0.319	0.279	0.506	0.639
StH	1	208268701	208269000	0.400	0.303	0.506	0.304
StH	1	209196301	209196600	0.186	0.163	0.502	0.248
StH	1	209713501	209713800	0.797	0.607	0.506	0.720
StH	1	210298201	210298500	0.105	0.118	0.526	0.193
StH	1	210324001	210324300	0.505	0.584	1.003	0.441
StH	1	210705901	210706200	0.618	0.319	2.109	0.462
StH	1	211692601	211692900	0.345	0.399	0.687	0.421
StH	1	212194501	212194800	0.453	0.421	0.662	0.193
StH	1	212203501	212203800	0.378	0.274	1.340	0.106
StH	1	213436501	213436800	0.477	0.397	0.720	0.446
StH	1	213486001	213486300	0.718	0.918	0.107	0.558
StH	1	213505501	213505800	0.106	0.354	1.483	0.147
StH	1	213698401	213698700	0.771	0.326	0.531	0.411
StH	1	214188001	214188300	0.079	0.279	0.535	0.198
StH	1	215124301	215124600	1.513	1.708	1.193	1.572
StH	1	215489401	215489700	0.611	0.165	0.399	0.826
StH	1	215563801	215564100	0.584	0.865	0.691	0.583
StH	1	215786101	215786400	0.503	0.770	0.292	0.527
StH	1	216001501	216001800	0.639	0.305	0.107	0.223
StH	1	216101701	216102000	1.250	1.261	1.037	0.821
StH	1	217280101	217280400	0.081	0.137	1.132	0.234
StH	1	217315201	217315500	0.503	0.536	0.716	0.304

StH	1	217874401	217874700	2.171	3.062	1.188	2.347
StH	1	217886101	217886400	0.108	0.230	0.642	0.137
StH	1	217886401	217886700	0.136	0.302	0.724	0.076
StH	1	218007301	218007600	1.412	1.242	1.917	1.369
StH	1	218227801	218228100	0.106	0.210	0.564	0.193
StH	1	219030601	219030900	0.373	0.257	0.700	0.335
StH	1	219895201	219895500	0.294	0.326	0.609	0.162
StH	1	221096101	221096400	0.082	0.189	0.560	0.081
StH	1	221392201	221392500	0.692	0.118	0.403	0.198
StH	1	221580601	221580900	0.371	0.139	0.638	0.248
StH	1	221957701	221958000	0.240	0.279	0.551	0.162
StH	1	222227101	222227400	0.850	0.869	0.395	0.933
StH	1	222860401	222860700	0.452	0.584	0.214	0.659
StH	1	223003201	223003500	2.075	1.783	1.280	1.825
StH	1	223338301	223338600	0.850	0.843	0.486	0.882
StH	1	224050501	224050800	0.424	0.328	0.555	0.167
StH	1	224442001	224442300	0.426	0.538	0.239	0.391
StH	1	224851501	224851800	0.215	0.191	0.560	0.112
StH	1	225411901	225412200	0.744	0.998	1.913	1.354
StH	1	225473701	225474000	0.187	0.140	0.580	0.092
StH	1	225852001	225852300	0.344	0.266	1.090	0.421
StH	1	227371201	227371500	0.479	0.491	0.189	0.329
StH	1	228259801	228260100	0.027	0.142	0.497	0.092
StH	1	228964801	228965100	0.052	0.157	0.943	0.223
StH	1	228965101	228965400	0.103	0.275	1.259	0.223
StH	1	229188601	229188900	0.452	0.841	0.239	0.360
StH	1	229842601	229842900	0.000	0.161	0.531	0.061
StH	1	230192701	230193000	0.292	0.326	0.560	0.279
StH	1	230212501	230212800	0.108	0.165	0.473	0.031
StH	1	230276701	230277000	0.553	0.581	1.609	0.731
StH	1	231629701	231630000	0.186	0.071	0.419	0.086
StH	1	232477801	232478100	0.052	0.167	0.448	0.106
StH	1	234541201	234541500	0.450	0.444	0.189	0.527
StH	1	235765201	235765500	0.319	0.421	0.185	0.497
StH	1	237291301	237291600	0.374	0.376	0.881	0.608
StH	1	238373701	238374000	7.792	8.268	4.661	8.305
StH	1	238839001	238839300	0.479	0.747	0.638	0.466
StH	1	238903201	238903500	0.800	1.448	1.748	0.806
StH	1	239013601	239013900	0.426	0.560	0.160	0.329
StH	1	239862901	239863200	0.532	0.279	0.082	0.254
StH	1	240120601	240120900	0.187	0.210	0.502	0.106
StH	1	240125701	240126000	1.115	1.365	2.328	1.348
StH	1	241424401	241424700	0.294	0.747	0.054	0.167
StH	1	241939801	241940100	0.505	0.466	0.185	0.436
StH	1	242974501	242974800	2.739	3.158	2.468	3.047
StH	1	243177301	243177600	2.531	1.886	0.921	1.770
StH	1	243772801	243773100	0.426	0.680	0.189	0.533
StH	1	244201201	244201500	1.837	1.873	1.094	1.491
StH	1	244416601	244416900	0.479	0.538	0.205	0.558
StH	1	244987501	244987800	2.573	2.939	1.514	2.444
StH	1	245641201	245641500	1.063	0.869	0.419	0.988
StH	1	245898001	245898300	0.670	0.963	1.596	1.004
StH	1	246125401	246125700	0.663	0.772	0.477	0.999
StH	1	246156601	246156900	0.400	0.489	0.078	0.461
StH	1	246421501	246421800	1.589	1.614	0.856	1.516
StH	1	246783301	246783600	0.079	0.139	0.448	0.076
StH	1	247110001	247110300	0.265	0.399	0.831	0.309
StH	1	247184101	247184400	0.213	0.185	0.622	0.248
StH	1	247376701	247377000	0.132	0.279	0.555	0.248
StH	1	247695601	247695900	0.244	0.234	0.613	0.193
StH	1	247711801	247712100	0.290	0.187	0.506	0.081
StH	1	247943701	247944000	0.400	0.375	0.667	0.360
StH	1	248138401	248138700	0.557	0.652	5.016	0.929
StH	1	248921101	248921400	2.150	2.763	1.132	2.572
StH	1	250304101	250304400	0.610	0.513	0.239	0.466
StH	1	251072401	251072700	0.586	0.633	0.823	0.522
StH	1	251133001	251133300	0.800	0.936	0.390	0.918

StH	1	251347501	251347800	0.505	0.513	0.399	0.639
StH	1	251459701	251460000	2.263	2.365	1.641	2.211
StH	1	251460001	251460300	1.520	1.547	0.987	1.521
StH	1	252172501	252172800	1.597	2.106	5.700	1.832
StH	1	252754801	252755100	0.266	0.165	0.477	0.279
StH	1	253031401	253031700	0.560	0.560	1.079	0.497
StH	1	253041001	253041300	0.319	0.444	0.239	0.472
StH	1	253368601	253368900	0.242	0.024	0.609	0.228
StH	1	253371001	253371300	4.034	3.467	2.036	4.076
StH	1	253572901	253573200	1.382	0.770	0.379	0.796
StH	1	253576501	253576800	3.551	3.411	5.493	3.478
StH	1	253636501	253636800	1.863	0.828	3.137	1.470
StH	1	253657501	253657800	1.640	1.670	5.618	2.615
StH	1	253981501	253981800	0.053	0.071	0.506	0.228
StH	1	256920901	256921200	0.345	0.515	0.214	0.446
StH	1	259500601	259500900	0.235	0.189	0.774	0.279
StH	1	259590001	259590300	1.544	1.755	1.275	2.013
StH	1	260156701	260157000	4.817	4.677	3.642	5.440
StH	1	260222701	260223000	0.689	0.727	1.066	0.385
StH	1	260437201	260437500	0.905	1.283	0.604	1.055
StH	1	260610901	260611200	0.558	0.470	0.823	0.477
StH	1	260835301	260835600	0.424	0.418	0.160	0.411
StH	1	260946001	260946300	1.812	2.345	3.134	2.216
StH	1	260947201	260947500	0.426	0.260	0.691	0.173
StH	1	260984101	260984400	1.755	1.334	2.107	1.389
StH	1	261014701	261015000	2.326	1.763	3.924	1.673
StH	1	261373801	261374100	0.560	0.865	0.312	0.943
StH	1	261405001	261405300	4.364	2.390	4.011	3.012
StH	1	261612601	261612900	1.170	0.678	0.510	1.080
StH	1	261906301	261906600	0.582	0.957	0.160	0.446
StH	1	261906601	261906900	0.584	0.656	0.210	0.391
StH	1	261921301	261921600	1.752	2.182	0.745	1.836
StH	1	262160701	262161000	0.958	0.891	0.609	1.217
StH	1	262216201	262216500	1.455	1.767	6.034	1.152
StH	1	262233901	262234200	0.879	0.714	1.930	0.866
StH	1	262338901	262339200	1.434	1.283	0.876	1.156
StH	1	262431301	262431600	0.376	0.914	2.806	0.294
StH	1	262461001	262461300	1.994	2.360	1.137	2.306
StH	1	262517701	262518000	1.676	2.414	0.959	1.618
StH	1	262710001	262710300	0.636	1.076	3.568	1.247
StH	1	262795501	262795800	0.106	0.225	1.242	0.295
StH	1	262865401	262865700	0.745	0.629	1.008	0.740
StH	1	262929301	262929600	1.089	1.515	0.588	1.318
StH	1	262970701	262971000	0.268	0.283	0.555	0.198
StH	1	263071501	263071800	2.468	2.321	4.661	2.449
StH	1	263072101	263072400	0.371	0.888	3.181	0.518
StH	1	263326801	263327100	3.291	3.637	4.496	3.550
StH	1	263757001	263757300	3.207	3.343	2.164	3.321
StH	1	263880301	263880600	0.321	0.257	0.054	0.659
StH	1	264101401	264101700	3.605	4.023	2.334	3.849
StH	1	264125701	264126000	0.745	1.063	3.910	0.782
StH	1	264164701	264165000	1.984	2.427	0.638	2.541
StH	1	264294601	264294900	0.103	0.341	1.859	0.324
StH	1	264310501	264310800	0.265	0.073	0.691	0.416
StH	1	264310801	264311100	0.210	0.212	0.720	0.193
StH	1	264496201	264496500	2.607	2.916	3.600	2.743
StH	1	264906301	264906600	0.132	0.328	0.613	0.391
StH	1	264909601	264909900	0.081	0.421	0.749	0.452
StH	1	265472401	265472700	0.237	0.208	0.671	0.274
StH	1	265668601	265668900	0.637	0.536	0.210	0.553
StH	1	265963801	265964100	0.237	0.189	0.502	0.223
StH	1	266360701	266361000	3.026	2.783	2.225	2.855
StH	1	266415601	266415900	0.027	0.137	0.675	0.031
StH	1	266610301	266610600	0.876	0.631	0.107	0.492
StH	1	266777401	266777700	0.218	0.365	1.792	0.208
StH	1	267012601	267012900	0.694	1.169	0.341	1.212
StH	1	267306301	267306600	0.311	1.377	3.359	1.055

StH	1	267306601	267306900	0.363	1.819	4.553	1.116
StH	1	267330301	267330600	4.624	4.999	7.105	2.367
StH	1	267377101	267377400	0.052	0.328	0.560	0.167
StH	1	267498301	267498600	0.240	0.431	2.114	0.487
StH	1	267606301	267606600	1.250	1.335	0.695	1.339
StH	1	267641101	267641400	1.412	1.143	0.510	1.375
StH	1	267732301	267732600	0.295	0.375	1.239	0.628
StH	1	268509901	268510200	4.889	7.668	10.242	6.174
StH	1	268713601	268713900	0.663	0.303	0.107	0.329
StH	1	269020501	269020800	1.664	1.657	7.556	1.692
StH	1	269020801	269021100	1.301	0.161	4.388	1.296
StH	1	269021701	269022000	1.784	2.665	10.536	2.857
StH	1	269137201	269137500	4.704	4.950	3.596	4.355
StH	1	269307901	269308200	1.673	1.659	1.066	1.805
StH	1	269367301	269367600	0.824	0.772	0.432	0.832
StH	1	269383801	269384100	0.373	0.210	0.000	0.466
StH	1	269445601	269445900	0.481	0.399	0.132	0.436
StH	1	269448001	269448300	0.082	0.187	0.535	0.248
StH	1	269472301	269472600	1.779	1.075	2.628	1.780
StH	1	269513401	269513700	0.208	0.590	1.226	0.492
StH	1	269528401	269528700	1.441	1.103	2.341	1.233
StH	1	269970601	269970900	0.958	0.891	0.419	0.751
StH	1	270121201	270121500	0.720	0.863	0.321	0.639
StH	1	270280201	270280500	0.295	0.345	1.264	0.208
StH	1	270330601	270330900	0.314	0.260	1.660	0.380
StH	1	270348001	270348300	0.373	0.444	0.531	0.664
StH	1	270378301	270378600	0.821	0.863	1.444	0.513
StH	1	270550501	270550800	0.318	0.538	0.156	0.385
StH	1	271023901	271024200	0.587	0.633	1.012	0.603
StH	1	271144801	271145100	0.903	0.890	1.066	0.826
StH	1	271169101	271169400	1.118	1.191	0.736	1.105
StH	1	271210801	271211100	0.905	0.817	0.481	0.923
StH	1	271260301	271260600	1.218	1.120	0.399	1.019
StH	1	271269901	271270200	1.257	1.038	3.056	0.988
StH	1	271424101	271424400	2.713	2.849	2.312	3.108
StH	1	271742101	271742400	0.215	0.247	1.297	0.147
StH	1	271965001	271965300	0.981	1.501	5.585	0.969
StH	1	272069401	272069700	0.189	0.303	0.687	0.304
StH	1	272103001	272103300	1.910	2.135	1.119	1.825
StH	1	272118901	272119200	1.676	3.664	2.300	3.615
StH	1	272165101	272165400	1.697	1.873	3.460	1.512
StH	1	272184001	272184300	1.386	1.120	4.961	1.324
StH	1	272224201	272224500	2.573	2.853	1.683	3.164
StH	1	272269201	272269500	1.558	1.587	3.551	1.111
StH	1	272277001	272277300	1.760	1.944	0.872	1.384
StH	1	272703301	272703600	1.302	1.025	0.555	1.024
StH	1	272792101	272792400	0.534	0.747	1.271	0.791
StH	1	273115801	273116100	0.955	0.796	0.506	0.771
StH	1	273125101	273125400	1.925	2.950	4.093	2.505
StH	1	273345001	273345300	3.399	4.119	2.887	3.458
StH	1	273525301	273525600	1.916	2.334	2.934	2.155
StH	1	273623401	273623700	2.538	2.600	1.242	2.566
StH	1	273754801	273755100	0.479	0.515	0.210	0.573
StH	1	273836101	273836400	2.794	3.166	1.921	3.651
StH	1	274256101	274256400	3.407	2.853	2.093	2.960
StH	1	274256701	274257000	0.265	0.836	3.255	0.279
StH	1	274257001	274257300	0.000	0.090	0.729	0.056
StH	1	274636201	274636500	1.702	2.055	0.704	1.857
StH	1	275013601	275013900	0.929	0.950	2.992	0.826
StH	1	275013901	275014200	2.600	2.370	4.601	2.174
StH	1	275104201	275104500	0.532	0.494	0.189	0.725
StH	1	275226301	275226600	0.455	0.583	1.402	0.431
StH	1	275364301	275364600	0.323	0.742	3.451	0.417
StH	1	275415001	275415300	0.340	0.485	1.507	0.396
StH	1	275466901	275467200	0.261	0.319	1.757	0.385
StH	1	275467201	275467500	3.742	3.417	5.871	3.407
StH	1	275488201	275488500	0.610	0.588	0.185	0.644

StH	1	275544301	275544600	0.799	0.682	2.274	0.660
StH	10	171901	172200	0.160	0.362	1.346	0.167
StH	10	182101	182400	0.137	0.180	1.478	0.157
StH	10	188101	188400	0.000	0.023	0.831	0.000
StH	10	188401	188700	0.081	0.045	3.719	1.035
StH	10	795301	795600	0.903	0.933	0.481	0.690
StH	10	1749301	1749600	0.453	0.539	0.921	0.547
StH	10	1987201	1987500	0.055	0.094	0.674	0.086
StH	10	2150101	2150400	0.611	0.281	0.794	0.167
StH	10	2190301	2190600	0.423	0.701	0.074	0.527
StH	10	2457301	2457600	1.011	0.813	2.124	0.841
StH	10	2554501	2554800	0.266	0.232	0.584	0.112
StH	10	3219301	3219600	0.557	0.562	0.189	0.466
StH	10	3563101	3563400	0.108	0.071	0.477	0.254
StH	10	4235401	4235700	0.106	0.047	0.419	0.117
StH	10	5316901	5317200	0.134	0.071	0.638	0.274
StH	10	5384401	5384700	0.426	0.560	0.136	0.492
StH	10	5780101	5780400	0.053	0.094	0.419	0.025
StH	10	6230101	6230400	0.211	0.305	0.506	0.193
StH	10	7401601	7401900	0.532	0.560	0.954	0.563
StH	10	7794001	7794300	0.103	0.165	0.588	0.187
StH	10	8253901	8254200	0.292	0.281	0.564	0.056
StH	10	8415901	8416200	1.010	0.931	0.555	0.943
StH	10	8433301	8433600	0.213	0.144	0.604	0.193
StH	10	8577901	8578200	0.507	0.657	1.012	0.553
StH	10	8700601	8700900	0.106	0.165	0.473	0.173
StH	10	10263001	10263300	0.479	0.468	0.346	0.527
StH	10	10595101	10595400	0.055	0.023	0.468	0.000
StH	10	11027401	11027700	0.216	0.185	0.662	0.187
StH	10	11041201	11041500	0.587	0.421	0.189	0.497
StH	10	11082601	11082900	0.187	0.240	0.778	0.284
StH	10	11082901	11083200	0.184	0.283	1.066	0.173
StH	10	11101801	11102100	0.105	0.071	0.604	0.106
StH	10	11184001	11184300	0.321	0.796	0.613	0.360
StH	10	11205301	11205600	0.802	1.051	1.629	0.994
StH	10	11533201	11533500	2.047	2.547	1.556	2.738
StH	10	11868601	11868900	0.131	0.000	0.720	0.000
StH	10	12036901	12037200	6.059	5.257	3.458	5.820
StH	10	12048601	12048900	2.134	2.673	1.255	3.006
StH	10	12644701	12645000	2.257	2.349	1.144	2.460
StH	10	12701701	12702000	0.184	0.116	0.477	0.223
StH	10	13195201	13195500	0.265	0.279	0.695	0.304
StH	10	13248901	13249200	0.158	0.210	0.477	0.162
StH	10	13342801	13343100	0.532	0.616	1.337	0.628
StH	10	13659001	13659300	0.292	0.257	0.535	0.254
StH	10	13743901	13744200	0.668	0.629	0.267	0.634
StH	10	13776601	13776900	1.387	1.124	0.424	1.009
StH	10	14115001	14115300	0.345	0.609	0.165	0.608
StH	10	14154301	14154600	0.507	0.607	0.181	0.588
StH	10	14180401	14180700	3.455	3.015	2.702	2.155
StH	10	14246701	14247000	2.100	1.749	1.217	1.658
StH	10	14247001	14247300	2.179	2.010	1.304	1.952
StH	10	14336401	14336700	1.762	2.197	1.086	0.486
StH	10	14615101	14615400	0.156	0.212	0.638	0.187
StH	10	14790901	14791200	0.395	0.583	1.012	0.608
StH	10	14889601	14889900	0.318	0.373	0.695	0.416
StH	10	14988001	14988300	0.027	0.444	0.136	0.000
StH	10	15071101	15071400	0.532	0.607	0.243	0.690
StH	10	15227701	15228000	0.718	1.051	0.448	0.329
StH	10	15290101	15290400	0.507	0.466	0.263	0.497
StH	10	15535201	15535500	0.344	0.373	0.103	0.527
StH	10	15623101	15623400	0.852	0.375	0.609	0.385
StH	10	15747601	15747900	0.694	0.491	0.243	0.527
StH	10	15861301	15861600	1.058	1.054	0.428	1.070
StH	10	16025701	16026000	0.160	0.258	0.613	0.324
StH	10	16147501	16147800	0.508	0.656	3.661	0.350
StH	10	16194001	16194300	2.134	2.173	5.072	2.041

StH	10	16309501	16309800	1.250	1.560	3.606	1.558
StH	10	16342201	16342500	2.765	2.971	3.217	2.819
StH	10	17313601	17313900	0.374	0.513	0.078	0.421
StH	10	17429701	17430000	0.505	0.376	0.107	0.497
StH	10	18023101	18023400	0.759	0.654	3.058	1.298
StH	10	18023401	18023700	0.053	0.112	0.560	0.000
StH	10	18338101	18338400	0.292	0.727	0.103	0.578
StH	10	18478501	18478800	0.474	0.633	1.066	0.533
StH	10	18478801	18479100	0.369	0.513	0.934	0.533
StH	10	18582901	18583200	0.663	0.431	1.895	0.533
StH	10	18941701	18942000	0.105	0.328	0.502	0.218
StH	10	19236601	19236900	0.319	1.049	0.633	0.335
StH	10	19544101	19544400	1.379	1.120	1.995	1.384
StH	10	19681201	19681500	0.244	0.249	1.593	0.076
StH	10	19896301	19896600	0.239	0.568	1.683	0.304
StH	10	20212801	20213100	0.079	0.168	0.560	0.162
StH	10	20241901	20242200	0.690	0.328	0.560	0.223
StH	10	20323501	20323800	2.471	2.500	1.030	2.028
StH	10	20448301	20448600	0.103	0.258	0.662	0.218
StH	10	20519701	20520000	0.874	0.768	0.176	1.166
StH	10	20520001	20520300	2.705	2.929	1.380	3.518
StH	10	20697901	20698200	0.319	0.326	0.239	0.608
StH	10	20739601	20739900	0.132	0.185	0.531	0.254
StH	10	20945701	20946000	0.526	0.607	2.193	1.242
StH	10	20946001	20946300	0.235	0.315	2.873	0.704
StH	10	20946301	20946600	2.525	3.608	6.858	4.237
StH	10	21410401	21410700	0.665	0.959	0.325	0.776
StH	10	21477601	21477900	0.876	0.938	0.312	0.907
StH	10	21708001	21708300	0.984	1.216	0.633	1.105
StH	10	21929401	21929700	0.395	0.629	1.119	0.360
StH	10	21930301	21930600	0.000	0.090	0.892	0.000
StH	10	22242001	22242300	3.244	2.929	2.250	3.427
StH	10	22702501	22702800	0.160	0.375	0.613	0.187
StH	10	23104801	23105100	0.239	0.466	0.082	0.223
StH	10	23304601	23304900	0.292	0.330	0.753	0.391
StH	10	23751301	23751600	0.342	0.328	0.885	0.375
StH	10	24015001	24015300	0.132	0.140	0.473	0.132
StH	10	24090301	24090600	0.132	0.047	0.448	0.193
StH	10	24676201	24676500	2.052	1.895	1.431	1.876
StH	10	24798301	24798600	0.132	0.444	0.292	0.137
StH	10	25003201	25003500	2.470	2.731	1.616	2.358
StH	10	25504201	25504500	0.319	0.251	1.378	0.380
StH	10	25595401	25595700	1.913	1.639	1.387	1.653
StH	10	25719301	25719600	2.822	2.551	3.360	2.545
StH	10	25866601	25866900	6.287	7.051	4.519	7.417
StH	10	25878601	25878900	0.824	0.723	0.481	0.690
StH	10	27315901	27316200	0.292	0.167	0.560	0.112
StH	10	27374701	27375000	0.268	0.305	0.687	0.441
StH	10	28232101	28232400	2.292	2.201	1.157	2.140
StH	10	28260301	28260600	0.694	0.912	0.317	0.608
StH	10	28541101	28541400	0.319	0.420	0.662	0.324
StH	10	28605301	28605600	1.031	1.824	0.515	1.136
StH	10	29200801	29201100	0.586	0.539	0.292	0.659
StH	10	29268301	29268600	1.407	1.290	1.966	1.440
StH	10	29271301	29271600	1.439	1.427	1.826	1.242
StH	10	29354701	29355000	0.771	0.869	0.453	0.781
StH	10	29409301	29409600	0.131	0.144	0.580	0.187
StH	10	29789701	29790000	3.807	4.025	2.780	3.842
StH	10	29910901	29911200	1.705	1.630	0.765	1.450
StH	10	30655201	30655500	2.392	1.804	1.362	1.790
StH	10	30706501	30706800	1.140	0.845	0.408	1.192
StH	10	30854401	30854700	0.637	0.556	1.012	0.274
StH	10	30856201	30856500	2.604	2.388	1.494	1.845
StH	10	31119601	31119900	0.663	0.672	0.136	0.669
StH	10	31147201	31147500	1.946	1.762	2.834	2.074
StH	10	31259401	31259700	1.568	1.332	0.769	1.176
StH	10	31276801	31277100	1.511	1.403	2.214	1.172

StH	10	31413001	31413300	0.479	1.075	0.716	0.497
StH	10	31581901	31582200	0.106	0.466	0.239	0.385
StH	10	31601701	31602000	0.186	0.277	0.667	0.360
StH	10	31879501	31879800	0.505	0.232	0.346	0.583
StH	10	32508001	32508300	1.695	1.862	4.271	1.582
StH	10	32947501	32947800	0.948	1.075	2.998	0.604
StH	10	32974801	32975100	2.930	2.729	1.407	2.089
StH	10	33227101	33227400	0.213	0.184	0.526	0.137
StH	10	33285901	33286200	0.534	0.903	1.699	0.527
StH	10	33566401	33566700	2.124	2.008	1.460	2.216
StH	10	33917401	33917700	1.086	0.611	1.739	0.132
StH	10	33917701	33918000	1.026	0.938	2.028	0.279
StH	10	33953101	33953400	0.505	0.584	0.136	0.659
StH	10	34496401	34496700	1.307	1.611	3.745	1.344
StH	10	34496701	34497000	1.367	1.675	5.234	2.085
StH	10	34598101	34598400	2.755	2.961	1.609	3.001
StH	10	34946101	34946400	0.695	0.704	1.193	0.573
StH	10	35076601	35076900	0.850	0.727	0.403	0.796
StH	10	35201101	35201400	3.910	3.699	2.702	4.283
StH	10	35715301	35715600	1.913	1.641	0.738	1.755
StH	10	35754001	35754300	1.678	1.708	1.304	1.881
StH	10	36191401	36191700	0.424	0.376	0.054	0.573
StH	10	36230701	36231000	0.881	1.146	0.584	0.994
StH	10	36361801	36362100	0.452	0.583	0.691	0.416
StH	10	36698101	36698400	2.876	3.017	1.732	2.951
StH	10	36709801	36710100	1.144	1.259	0.346	1.070
StH	10	36953701	36954000	0.369	0.330	0.749	0.329
StH	10	37124101	37124400	0.319	0.373	0.687	0.497
StH	10	37504501	37504800	0.665	0.818	1.090	0.492
StH	10	37756501	37756800	0.532	0.257	0.370	0.162
StH	10	39312901	39313200	1.039	0.701	0.457	0.776
StH	10	40006201	40006500	0.479	0.680	0.321	0.583
StH	10	40954201	40954500	0.403	0.000	0.909	0.137
StH	10	41038501	41038800	0.718	0.583	0.341	0.583
StH	10	42018601	42018900	0.558	0.281	0.950	0.507
StH	10	42683401	42683700	0.505	0.584	0.399	0.360
StH	10	42902701	42903000	0.586	0.466	0.321	0.751
StH	10	43545601	43545900	0.108	0.236	0.526	0.193
StH	10	44176201	44176500	0.637	0.697	1.246	0.492
StH	10	44337601	44337900	0.108	0.140	0.374	0.603
StH	10	45762901	45763200	0.895	0.275	1.942	0.974
StH	10	46501201	46501500	0.079	0.185	0.526	0.106
StH	10	47176501	47176800	0.240	0.257	0.526	0.193
StH	10	47274001	47274300	0.160	0.515	0.350	0.167
StH	10	47951101	47951400	0.531	0.654	0.239	0.527
StH	10	48220501	48220800	0.189	0.116	0.560	0.203
StH	10	48545101	48545400	0.132	0.118	0.711	0.076
StH	10	49107601	49107900	0.932	1.242	0.506	0.862
StH	10	49446301	49446600	0.824	0.397	0.609	0.385
StH	10	49840201	49840500	1.036	0.935	0.823	0.664
StH	10	50119501	50119800	0.610	1.217	0.399	0.826
StH	10	50490901	50491200	0.215	0.213	0.555	0.117
StH	10	50580601	50580900	0.079	0.258	0.564	0.081
StH	10	51720301	51720600	0.266	0.116	0.473	0.223
StH	10	53361601	53361900	0.134	0.491	0.321	0.223
StH	10	53368501	53368800	0.823	0.865	0.510	1.075
StH	10	53762701	53763000	0.026	0.352	0.535	0.167
StH	10	53834701	53835000	0.876	0.584	0.218	0.862
StH	10	54160801	54161100	0.294	0.279	0.593	0.223
StH	10	54419701	54420000	0.505	0.517	0.881	0.472
StH	10	54628501	54628800	0.213	0.230	0.604	0.162
StH	10	55562401	55562700	0.529	0.676	0.210	0.603
StH	10	55568101	55568400	0.285	0.399	1.197	0.238
StH	10	55569301	55569600	0.053	0.092	0.486	0.086
StH	10	55569601	55569900	0.026	0.096	0.477	0.137
StH	10	55624201	55624500	0.934	0.798	1.748	0.497
StH	10	55662001	55662300	1.329	0.961	0.749	1.410

StH	10	56473501	56473800	0.266	0.393	0.662	0.279
StH	10	56883001	56883300	0.158	0.229	0.724	0.203
StH	10	56995201	56995500	0.903	0.693	1.601	0.502
StH	10	59252101	59252400	0.507	0.210	0.370	0.137
StH	10	59310901	59311200	0.081	0.258	0.477	0.117
StH	10	60049501	60049800	0.584	0.609	0.267	0.502
StH	10	60180301	60180600	0.161	0.094	0.486	0.112
StH	10	60425701	60426000	1.219	0.955	1.930	0.999
StH	10	60451501	60451800	0.955	1.008	0.374	1.034
StH	10	60980701	60981000	0.105	0.096	0.506	0.329
StH	10	61611301	61611600	0.053	0.118	0.473	0.051
StH	10	61843501	61843800	0.611	0.609	0.243	0.385
StH	10	62098201	62098500	0.479	0.468	0.292	0.695
StH	10	64566901	64567200	0.079	0.230	0.477	0.086
StH	10	65770801	65771100	1.031	1.002	0.243	0.837
StH	10	65999701	66000000	0.239	0.257	0.477	0.198
StH	10	66101401	66101700	0.105	0.137	0.564	0.218
StH	10	66226201	66226500	1.065	1.500	0.386	1.055
StH	10	67254601	67254900	1.489	2.263	0.749	2.023
StH	10	68683501	68683800	3.331	3.042	1.534	3.837
StH	10	69462901	69463200	0.240	0.517	0.983	0.710
StH	10	70746301	70746600	0.027	0.000	0.390	0.031
StH	10	70755301	70755600	0.027	0.000	0.415	0.031
StH	10	71604901	71605200	0.213	0.584	0.082	0.329
StH	10	71936101	71936400	0.531	0.375	0.082	0.309
StH	10	71970301	71970600	1.113	1.163	2.726	1.262
StH	10	72340501	72340800	0.000	0.493	0.107	0.000
StH	10	72456901	72457200	2.450	2.362	1.394	2.459
StH	10	72457201	72457500	2.446	2.528	1.574	2.743
StH	10	72547501	72547800	0.694	0.656	1.003	0.639
StH	10	73503901	73504200	0.240	0.356	1.032	0.122
StH	10	73560901	73561200	1.247	1.334	0.645	0.025
StH	10	73860901	73861200	0.826	0.843	0.390	0.812
StH	10	73861201	73861500	0.424	0.326	0.156	0.522
StH	10	74156101	74156400	0.081	0.187	0.531	0.304
StH	10	74667601	74667900	1.008	0.564	0.194	0.801
StH	10	75274801	75275100	0.481	0.470	0.098	0.497
StH	10	75435001	75435300	0.878	0.843	0.337	0.832
StH	10	75688801	75689100	0.319	0.421	0.160	0.492
StH	10	75832801	75833100	1.910	2.105	0.921	1.465
StH	10	76056901	76057200	7.011	7.790	5.375	7.271
StH	10	76089901	76090200	0.663	0.622	1.502	0.563
StH	10	76167901	76168200	0.314	0.993	2.205	0.344
StH	10	76239601	76239900	1.620	1.704	1.358	2.216
StH	10	76256101	76256400	1.086	1.167	0.419	0.857
StH	10	76287301	76287600	0.263	0.258	0.707	0.117
StH	10	76455901	76456200	0.692	0.513	0.399	0.776
StH	10	76527601	76527900	0.423	0.772	2.307	0.335
StH	10	76535101	76535400	0.426	0.446	0.243	0.416
StH	10	76806601	76806900	0.187	0.191	0.531	0.142
StH	10	76840801	76841100	0.797	0.794	0.283	0.553
StH	10	76915501	76915800	0.211	0.240	0.979	0.086
StH	10	77169301	77169600	0.799	0.652	0.147	0.740
StH	10	77223901	77224200	0.426	0.212	0.778	0.411
StH	10	77293801	77294100	0.455	0.699	1.313	0.624
StH	10	77447101	77447400	2.205	2.055	2.706	1.866
StH	10	77477101	77477400	0.932	1.084	2.192	0.836
StH	10	77489101	77489400	0.215	0.326	0.609	0.385
StH	10	77525701	77526000	1.032	1.167	0.457	0.943
StH	10	77962501	77962800	2.926	2.030	0.713	1.703
StH	10	78226501	78226800	5.339	5.139	3.362	5.404
StH	10	78234601	78234900	0.347	0.401	0.049	0.497
StH	10	78383701	78384000	0.694	0.729	1.387	0.603
StH	10	78388801	78389100	0.374	0.373	1.413	0.335
StH	10	78665701	78666000	1.120	1.626	2.472	1.491
StH	10	78692401	78692700	0.000	0.047	0.419	0.162
StH	10	78754801	78755100	0.052	0.234	0.448	0.086

StH	10	78905101	78905400	1.436	1.515	0.539	0.958
StH	10	79070101	79070400	0.769	0.468	0.346	0.608
StH	10	80577001	80577300	0.240	0.279	1.264	0.081
StH	10	80918701	80919000	0.742	0.637	0.107	0.547
StH	10	81100801	81101100	2.571	2.600	5.388	3.158
StH	10	81282601	81282900	0.374	0.425	1.128	0.472
StH	10	81282901	81283200	0.323	0.384	1.349	0.502
StH	10	81309301	81309600	1.620	2.409	1.077	1.861
StH	10	81390601	81390900	0.052	0.023	0.477	0.106
StH	10	81622801	81623100	0.294	0.562	0.399	0.583
StH	10	81859801	81860100	0.986	0.751	0.263	0.897
StH	10	82347901	82348200	3.227	3.856	2.329	4.396
StH	10	82406701	82407000	0.421	0.538	1.057	0.558
StH	10	82593901	82594200	0.637	0.421	0.156	0.416
StH	10	82622101	82622400	0.503	0.515	0.243	0.806
StH	10	82717201	82717500	1.010	0.749	1.329	0.776
StH	10	82901101	82901400	0.347	0.890	0.506	0.248
StH	10	83226601	83226900	0.453	1.309	0.584	0.771
StH	10	83506501	83506800	2.639	2.551	3.460	2.490
StH	10	83605801	83606100	1.931	1.983	6.707	1.608
StH	10	83706301	83706600	4.067	4.306	2.640	4.159
StH	10	83713501	83713800	1.774	1.894	0.950	1.709
StH	10	83727301	83727600	3.536	3.576	3.101	4.391
StH	10	83805001	83805300	2.231	3.592	2.867	1.876
StH	10	83838301	83838600	0.479	0.470	0.239	0.578
StH	10	83842501	83842800	1.570	2.313	1.023	1.521
StH	10	83865001	83865300	0.960	1.156	2.258	0.898
StH	10	83866801	83867100	0.792	0.862	1.757	0.644
StH	10	84042001	84042300	0.265	0.328	1.268	0.340
StH	10	84050401	84050700	2.452	1.569	0.979	1.663
StH	10	84068101	84068400	4.607	4.094	2.842	4.238
StH	10	84089401	84089700	0.290	0.305	0.613	0.324
StH	10	84225601	84225900	3.825	4.098	5.975	3.434
StH	10	84507001	84507300	0.240	0.722	2.927	0.228
StH	10	84588001	84588300	0.477	0.249	1.515	0.385
StH	10	84591901	84592200	9.150	8.777	7.504	10.101
StH	10	84593101	84593400	0.685	1.248	4.338	0.999
StH	10	84775801	84776100	0.531	0.437	1.090	0.355
StH	10	84842701	84843000	0.160	0.137	0.588	0.117
StH	10	84985801	84986100	0.000	0.157	3.155	0.122
StH	10	84991801	84992100	2.896	2.308	10.136	2.927
StH	10	85042201	85042500	0.821	0.865	0.428	0.943
StH	10	85125301	85125600	0.850	1.139	2.193	1.029
StH	10	85128301	85128600	1.755	1.496	1.246	0.826
StH	10	85138201	85138500	1.633	1.403	2.884	1.886
StH	10	85207801	85208100	1.276	1.171	0.769	1.253
StH	10	85239601	85239900	0.294	0.577	2.465	0.370
StH	10	85261501	85261800	1.558	1.812	4.495	1.436
StH	10	85273201	85273500	0.187	0.326	0.502	0.167
StH	10	85295101	85295400	6.961	6.404	4.937	6.257
StH	10	85382701	85383000	0.797	0.586	1.086	0.725
StH	10	85489801	85490100	0.161	0.907	4.345	0.268
StH	10	85552501	85552800	0.613	0.615	1.962	0.745
StH	10	85586101	85586400	1.221	1.744	2.715	1.841
StH	10	85606801	85607100	0.161	0.180	1.560	0.152
StH	10	85617901	85618200	0.213	0.902	3.558	0.299
StH	10	85726201	85726500	0.721	0.912	1.284	0.832
StH	10	85741201	85741500	0.344	1.220	4.326	0.594
StH	10	85809601	85809900	0.052	0.094	0.539	0.081
StH	10	85819501	85819800	0.474	1.621	5.883	1.517
StH	10	85821301	85821600	2.118	1.922	3.201	1.922
StH	10	85827901	85828200	1.029	0.948	2.955	0.513
StH	10	85873801	85874100	0.369	0.781	1.790	0.507
StH	10	85973701	85974000	2.636	2.851	2.028	2.774
StH	10	86055901	86056200	0.347	0.371	0.905	0.380
StH	10	86141101	86141400	0.479	0.322	0.979	0.126
StH	10	86217001	86217300	1.174	0.974	2.642	1.338

StH	10	86251501	86251800	8.697	8.750	7.223	8.689
StH	11	52801	53100	1.333	1.388	0.424	1.177
StH	11	642301	642600	0.079	0.139	0.419	0.117
StH	11	907501	907800	0.479	0.538	0.288	0.639
StH	11	1010701	1011000	1.142	1.234	1.801	1.379
StH	11	1113601	1113900	0.744	0.891	0.305	1.161
StH	11	1290601	1290900	0.160	0.142	0.419	0.137
StH	11	1548901	1549200	0.452	0.914	0.667	0.416
StH	11	1816201	1816500	0.026	0.068	0.506	0.193
StH	11	2187601	2187900	0.957	0.257	0.716	0.274
StH	11	3880201	3880500	0.527	0.562	1.251	0.669
StH	11	6088201	6088500	0.526	1.031	4.121	0.558
StH	11	6158701	6159000	0.292	0.352	0.477	0.608
StH	11	6214501	6214800	0.319	0.189	0.564	0.279
StH	11	6292201	6292500	0.611	0.305	0.453	0.274
StH	11	6672901	6673200	0.184	0.163	0.370	0.634
StH	11	6952201	6952500	1.010	0.583	0.374	0.684
StH	11	7107301	7107600	0.953	1.193	0.374	0.715
StH	11	7407601	7407900	0.853	0.964	1.735	0.943
StH	11	7527601	7527900	0.905	0.817	0.419	0.776
StH	11	7740901	7741200	0.745	1.006	3.127	1.501
StH	11	7758601	7758900	3.394	3.137	1.616	2.890
StH	11	7828501	7828800	2.207	2.105	1.057	2.151
StH	11	7836901	7837200	0.289	0.429	1.543	0.320
StH	11	7893301	7893600	0.934	0.938	0.432	1.029
StH	11	7956301	7956600	0.081	0.345	1.074	0.142
StH	11	8029201	8029500	0.636	0.841	0.370	0.720
StH	11	8080801	8081100	0.421	0.409	1.415	0.426
StH	11	8124301	8124600	4.015	4.266	3.114	3.935
StH	11	8446501	8446800	0.371	0.581	0.058	0.411
StH	11	8611201	8611500	0.292	0.260	0.531	0.248
StH	11	8805901	8806200	0.108	0.068	0.510	0.132
StH	11	8828701	8829000	0.158	0.270	1.538	0.122
StH	11	8838901	8839200	0.184	0.232	0.502	0.223
StH	11	8995201	8995500	0.802	0.912	1.398	0.913
StH	11	9112201	9112500	1.089	1.193	0.613	0.852
StH	11	9503101	9503400	2.654	2.412	1.752	2.439
StH	11	9626401	9626700	0.344	0.493	0.107	0.695
StH	11	9626701	9627000	0.426	0.560	0.136	0.477
StH	11	9847501	9847800	0.957	0.935	0.374	0.938
StH	11	9873601	9873900	0.213	0.257	0.346	0.522
StH	11	10008901	10009200	0.400	0.725	0.189	0.583
StH	11	10022401	10022700	2.633	2.532	1.699	2.743
StH	11	10388401	10388700	1.937	1.706	0.934	1.328
StH	11	10560001	10560300	1.008	1.639	0.481	1.222
StH	11	10589101	10589400	0.532	0.538	0.210	0.588
StH	11	10868701	10869000	2.578	2.498	0.604	2.306
StH	11	10869001	10869300	2.921	2.777	0.604	2.341
StH	11	11820001	11820300	0.450	0.468	0.160	0.583
StH	11	11996701	11997000	0.266	0.815	0.588	0.142
StH	11	12364501	12364800	1.300	1.424	0.930	1.440
StH	11	12382501	12382800	0.929	0.699	1.514	1.004
StH	11	12399601	12399900	0.345	0.167	2.408	0.543
StH	11	12399901	12400200	0.108	0.144	0.604	0.112
StH	11	12422401	12422700	0.574	0.433	1.830	0.411
StH	11	12563101	12563400	1.037	1.217	0.774	0.999
StH	11	12644701	12645000	0.881	1.023	2.135	0.953
StH	11	12725401	12725700	1.274	1.262	0.909	1.253
StH	11	12742801	12743100	0.718	0.470	0.185	0.329
StH	11	12998101	12998400	7.338	8.106	5.906	7.711
StH	11	13017301	13017600	1.413	1.099	0.740	1.080
StH	11	13077001	13077300	2.231	2.549	1.583	2.515
StH	11	13796101	13796400	1.144	1.188	0.760	0.187
StH	11	14051701	14052000	1.331	1.618	0.731	1.587
StH	11	14474401	14474700	1.947	2.100	0.752	1.546
StH	11	14647501	14647800	0.744	0.212	0.374	0.553
StH	11	14783101	14783400	0.345	0.539	0.132	0.304

StH	11	14879101	14879400	0.848	0.775	1.344	0.472
StH	11	14975101	14975400	0.797	1.036	2.893	0.522
StH	11	14986201	14986500	0.237	0.210	0.811	0.259
StH	11	15555901	15556200	0.213	0.238	0.560	0.173
StH	11	15593701	15594000	1.991	2.032	1.226	2.419
StH	11	15811801	15812100	0.265	0.468	0.082	0.391
StH	11	16527601	16527900	2.955	1.954	6.901	2.160
StH	11	16527901	16528200	1.796	1.287	4.897	1.906
StH	11	16615501	16615800	0.453	0.560	0.214	0.391
StH	11	16616101	16616400	0.639	0.818	0.292	0.979
StH	11	16844401	16844700	2.312	2.476	2.842	2.246
StH	11	17320801	17321100	0.132	0.584	0.346	0.167
StH	11	17334601	17334900	0.186	0.255	0.502	0.218
StH	11	17769601	17769900	6.086	6.535	3.452	6.012
StH	11	17959801	17960100	0.505	0.796	0.176	0.583
StH	11	17967301	17967600	0.481	0.438	0.963	0.365
StH	11	17997001	17997300	4.101	2.982	7.261	2.986
StH	11	18027601	18027900	3.060	3.053	2.138	2.976
StH	11	18134101	18134400	0.429	0.929	1.884	0.452
StH	11	18194401	18194700	0.292	0.281	0.885	0.558
StH	11	18557101	18557400	0.240	0.607	0.107	0.502
StH	11	18583801	18584100	0.318	0.352	0.959	0.335
StH	11	18840001	18840300	1.091	1.309	1.596	0.659
StH	11	19129501	19129800	1.599	1.332	0.827	1.105
StH	11	19129801	19130100	0.531	0.493	0.346	0.608
StH	11	19153801	19154100	0.289	0.418	0.945	0.223
StH	11	19500001	19500300	0.477	0.605	0.267	0.751
StH	11	19883101	19883400	0.481	0.538	0.156	0.477
StH	11	20009101	20009400	0.213	0.234	0.555	0.715
StH	11	20423101	20423400	0.586	0.466	0.288	0.608
StH	11	20889901	20890200	0.373	0.560	0.160	0.385
StH	11	20890201	20890500	0.452	0.583	0.267	0.466
StH	11	20934301	20934600	0.611	0.914	0.448	0.664
StH	11	21047401	21047700	0.026	0.163	0.448	0.147
StH	11	21052501	21052800	0.316	0.255	0.867	0.193
StH	11	21228301	21228600	0.266	0.257	0.132	0.497
StH	11	21386401	21386700	5.549	5.385	3.986	5.627
StH	11	21539401	21539700	0.344	0.328	0.156	0.553
StH	11	21929101	21929400	0.481	0.513	0.103	0.502
StH	11	22022701	22023000	0.265	0.185	0.580	0.304
StH	11	22056901	22057200	1.170	0.749	1.333	0.776
StH	11	22253401	22253700	1.036	1.236	0.410	1.237
StH	11	22310401	22310700	0.532	0.884	1.465	1.044
StH	11	22635301	22635600	0.508	0.564	0.078	0.507
StH	11	22702801	22703100	0.000	0.045	0.515	0.086
StH	11	22785001	22785300	1.781	2.100	1.019	2.378
StH	11	22942201	22942500	3.754	3.782	2.920	3.822
StH	11	22980301	22980600	3.594	3.684	2.248	3.595
StH	11	22997101	22997400	0.242	0.303	0.617	0.162
StH	11	23270101	23270400	0.534	0.560	0.346	0.968
StH	11	23721901	23722200	2.044	1.609	3.885	1.677
StH	11	24045001	24045300	0.584	0.629	0.267	0.639
StH	11	24115501	24115800	1.621	1.448	0.954	1.395
StH	11	24549901	24550200	0.294	0.773	0.107	0.385
StH	11	24728701	24729000	0.505	0.511	0.769	0.472
StH	11	24836701	24837000	0.424	0.607	0.872	0.497
StH	11	24918301	24918600	0.189	0.187	0.477	0.187
StH	11	25085101	25085400	3.136	2.873	2.299	3.296
StH	11	25085401	25085700	1.753	1.540	1.041	1.775
StH	11	25126201	25126500	0.613	0.536	0.103	0.421
StH	11	25173301	25173600	0.161	0.116	0.477	0.162
StH	11	25208401	25208700	0.295	0.303	0.682	0.106
StH	11	25565701	25566000	0.799	0.914	0.312	0.553
StH	11	25938601	25938900	0.158	0.442	0.720	0.279
StH	11	26190901	26191200	1.407	0.912	0.555	1.100
StH	11	26772301	26772600	5.985	6.271	4.684	6.195
StH	11	26870101	26870400	2.210	1.912	0.653	1.765

StH	11	27308401	27308700	0.453	0.676	1.045	0.335
StH	11	27321001	27321300	0.558	0.609	0.847	0.547
StH	11	27411601	27411900	0.215	0.279	0.921	0.426
StH	11	27513301	27513600	1.247	1.517	2.098	1.683
StH	11	27753001	27753300	1.678	1.384	2.156	1.176
StH	11	27850801	27851100	0.687	0.661	1.440	0.796
StH	11	28131301	28131600	1.302	1.262	0.876	0.999
StH	11	28132201	28132500	0.666	0.624	1.629	0.436
StH	11	28166701	28167000	4.147	3.974	3.049	4.543
StH	11	28236301	28236600	9.281	8.927	6.760	8.578
StH	11	28580101	28580400	0.450	0.556	0.987	0.360
StH	11	28626301	28626600	1.915	2.319	1.157	1.800
StH	11	28715701	28716000	0.136	0.116	0.564	0.132
StH	11	28893301	28893600	0.160	0.425	0.831	0.446
StH	11	28920901	28921200	0.319	0.863	0.564	0.132
StH	11	29184601	29184900	0.239	0.140	0.477	0.167
StH	11	29223901	29224200	4.065	4.403	2.831	3.656
StH	11	29360401	29360700	0.690	0.747	1.184	0.695
StH	11	29657401	29657700	0.265	0.208	0.531	0.173
StH	11	30094801	30095100	0.081	0.161	0.419	0.081
StH	11	31168801	31169100	0.373	0.326	0.477	0.081
StH	11	31663501	31663800	2.570	3.098	0.214	3.065
StH	11	31663801	31664100	2.810	3.374	0.548	3.728
StH	11	31730401	31730700	1.699	2.013	1.246	1.750
StH	11	31872601	31872900	2.258	2.393	3.497	2.160
StH	11	32104501	32104800	1.142	1.096	0.510	0.776
StH	11	32173801	32174100	0.211	0.142	1.609	0.493
StH	11	32203501	32203800	0.240	0.118	0.502	0.142
StH	11	32296201	32296500	0.477	0.468	0.214	0.416
StH	11	32893501	32893800	1.118	1.216	0.983	1.354
StH	11	33517801	33518100	0.611	0.701	0.111	0.527
StH	11	33605401	33605700	1.036	0.768	1.761	0.882
StH	11	33611701	33612000	0.297	0.135	0.974	0.122
StH	11	33692101	33692400	1.726	1.847	1.402	2.038
StH	11	33729001	33729300	0.450	0.328	0.687	0.385
StH	11	33852901	33853200	0.984	1.032	0.535	0.862
StH	11	33942601	33942900	8.085	8.986	12.888	9.459
StH	11	33969601	33969900	0.558	0.656	0.189	0.522
StH	11	34027801	34028100	1.150	0.926	3.473	0.719
StH	11	34059301	34059600	0.240	0.423	0.054	0.279
StH	11	34092001	34092300	2.801	2.804	1.700	2.799
StH	11	34149301	34149600	0.745	0.961	0.506	0.690
StH	11	34161901	34162200	1.097	0.677	3.619	0.817
StH	11	34421101	34421400	0.821	0.729	1.255	0.603
StH	11	34481701	34482000	0.052	0.320	1.660	0.274
StH	11	34554001	34554300	1.199	1.189	0.736	1.181
StH	11	34627201	34627500	3.005	2.197	1.469	2.286
StH	11	34662901	34663200	0.289	0.628	1.217	0.182
StH	11	34874701	34875000	0.560	0.612	0.107	0.517
StH	11	35376001	35376300	4.923	3.748	1.797	4.147
StH	11	35394301	35394600	0.907	0.935	0.424	0.715
StH	11	35653501	35653800	1.379	1.313	0.823	1.348
StH	11	35725201	35725500	0.986	0.893	0.399	0.801
StH	11	35742001	35742300	2.890	1.896	8.233	2.985
StH	11	35933101	35933400	1.036	1.154	0.350	1.050
StH	11	35943601	35943900	1.273	1.099	0.638	1.111
StH	11	36050101	36050400	3.163	2.397	1.431	2.196
StH	11	36197101	36197400	1.545	1.635	2.374	1.709
StH	11	36244501	36244800	0.613	0.303	0.185	0.441
StH	11	36257401	36257700	0.586	0.631	0.312	0.832
StH	11	36527701	36528000	1.139	1.523	0.716	1.136
StH	11	36537601	36537900	0.640	0.609	0.983	0.690
StH	11	36714601	36714900	0.213	0.232	0.551	0.173
StH	11	37415101	37415400	6.249	5.774	6.840	4.664
StH	11	37422301	37422600	2.079	1.684	2.570	1.993
StH	11	37687501	37687800	0.079	0.137	0.473	0.142
StH	11	37689301	37689600	0.239	0.234	0.662	0.324

StH	11	37734901	37735200	0.213	0.163	0.502	0.142
StH	11	37984801	37985100	0.186	0.255	0.506	0.248
StH	11	38125201	38125500	1.991	1.965	1.460	1.861
StH	11	38188201	38188500	0.290	0.279	0.588	0.106
StH	11	38199301	38199600	0.960	1.149	2.535	0.781
StH	11	38675101	38675400	0.900	0.691	2.017	0.948
StH	11	39075001	39075300	0.345	0.446	0.107	0.279
StH	11	39168301	39168600	0.160	0.303	0.448	0.142
StH	11	39231901	39232200	0.799	0.772	0.374	0.248
StH	11	39241501	39241800	0.453	0.255	2.085	0.406
StH	11	39248701	39249000	1.699	0.373	3.105	1.293
StH	11	39249001	39249300	1.781	0.243	3.163	1.430
StH	11	39637801	39638100	1.942	1.214	1.558	2.535
StH	11	40500301	40500600	0.213	0.420	0.720	0.547
StH	11	41012101	41012400	0.265	0.305	0.555	0.248
StH	11	41067301	41067600	0.268	0.049	0.769	0.137
StH	11	41454301	41454600	1.984	2.803	6.631	2.373
StH	11	41483401	41483700	2.025	1.427	3.090	2.424
StH	11	41596201	41596500	2.289	2.292	1.391	2.216
StH	11	41793301	41793600	3.112	2.435	1.933	2.956
StH	11	41901601	41901900	0.292	0.212	0.535	0.304
StH	11	41922901	41923200	0.981	1.006	1.699	1.186
StH	11	41995201	41995500	0.424	0.326	0.872	0.385
StH	11	42020701	42021000	0.503	0.843	0.292	0.715
StH	11	42116401	42116700	0.000	0.047	0.517	0.086
StH	11	42312001	42312300	2.866	2.315	1.830	2.367
StH	11	42410101	42410400	0.240	0.328	0.502	0.335
StH	11	42989401	42989700	0.586	0.320	1.369	0.345
StH	11	43038901	43039200	2.476	0.998	1.732	2.475
StH	11	43342201	43342500	1.886	2.197	1.436	1.709
StH	11	43414801	43415100	0.429	0.740	1.456	0.502
StH	11	43602001	43602300	6.275	6.166	5.170	5.674
StH	11	43982701	43983000	2.020	1.753	1.139	0.806
StH	11	44068501	44068800	0.319	0.678	0.453	0.274
StH	11	45238501	45238800	1.066	1.148	0.671	1.334
StH	11	45282901	45283200	0.824	0.820	0.535	0.994
StH	11	45371401	45371700	0.448	0.494	1.110	0.426
StH	11	45441301	45441600	1.303	1.068	1.942	1.105
StH	11	45598801	45599100	0.718	0.749	0.370	0.472
StH	11	45629701	45630000	1.063	1.195	0.695	1.602
StH	11	45654901	45655200	0.265	0.375	1.259	0.553
StH	11	45671401	45671700	0.129	0.140	0.477	0.137
StH	11	45718801	45719100	1.086	1.794	0.325	1.424
StH	11	46517401	46517700	0.321	0.618	1.813	0.695
StH	11	46654501	46654800	0.347	0.399	0.642	0.167
StH	11	46916401	46916700	1.092	0.701	0.160	0.852
StH	11	47187601	47187900	0.077	0.202	0.831	0.223
StH	11	47732401	47732700	0.294	0.912	0.646	0.309
StH	11	47750701	47751000	0.319	0.373	0.695	0.228
StH	11	48007201	48007500	0.745	0.536	0.399	0.690
StH	11	48404101	48404400	0.960	1.452	0.716	0.999
StH	11	48540001	48540300	0.642	0.755	1.777	0.716
StH	11	48630601	48630900	0.052	0.096	0.477	0.081
StH	11	48687601	48687900	0.587	0.472	1.094	0.522
StH	11	48786301	48786600	0.239	0.468	0.078	0.360
StH	11	48956401	48956700	2.581	2.577	1.596	2.388
StH	11	49023901	49024200	0.666	1.191	0.954	1.131
StH	11	49042501	49042800	0.211	0.258	0.510	0.720
StH	11	49130101	49130400	0.508	0.818	0.243	0.522
StH	11	49178101	49178400	1.116	1.079	0.658	1.024
StH	11	49529701	49530000	0.371	0.539	0.214	0.472
StH	11	49774501	49774800	2.949	2.407	3.418	2.378
StH	11	49837801	49838100	0.344	0.969	3.333	0.446
StH	11	49911901	49912200	1.252	1.214	0.588	1.445
StH	11	49956301	49956600	0.928	1.362	3.071	1.166
StH	11	50037301	50037600	1.568	1.143	0.901	1.845
StH	11	50157901	50158200	0.055	0.047	0.419	0.081

StH	11	50335201	50335500	0.371	0.446	0.132	0.335
StH	11	50375101	50375400	0.692	0.727	0.292	0.527
StH	11	50392801	50393100	0.958	0.935	0.687	0.391
StH	11	50567701	50568000	0.292	0.539	0.082	0.365
StH	11	50773201	50773500	0.716	1.173	2.855	1.020
StH	11	50773501	50773800	1.410	1.613	2.127	1.399
StH	11	50790601	50790900	0.900	0.953	1.572	0.832
StH	11	51142801	51143100	2.450	1.452	0.346	1.363
StH	11	51198601	51198900	0.052	0.722	2.572	0.487
StH	11	51198901	51199200	0.052	0.185	0.477	0.142
StH	11	51412201	51412500	0.587	0.584	0.317	0.690
StH	11	52050001	52050300	0.747	0.513	1.703	0.912
StH	11	52077301	52077600	0.749	0.843	0.296	0.715
StH	11	52168201	52168500	1.171	0.744	0.428	1.100
StH	11	52189201	52189500	1.521	1.512	2.535	3.731
StH	11	52262401	52262700	2.573	2.605	1.307	2.662
StH	11	52667701	52668000	0.584	1.214	0.074	0.574
StH	11	52675501	52675800	2.789	2.392	0.698	3.581
StH	11	52778401	52778700	0.450	0.854	3.187	0.594
StH	11	52784401	52784700	2.952	3.602	1.703	2.960
StH	11	52803601	52803900	1.518	0.238	0.716	0.391
StH	11	52886101	52886400	2.631	2.483	1.986	1.577
StH	11	53178601	53178900	0.477	0.468	0.716	0.472
StH	11	53375401	53375700	1.226	1.075	1.587	1.075
StH	11	53408101	53408400	0.186	0.234	0.448	0.187
StH	11	53556001	53556300	1.463	1.918	1.021	1.354
StH	11	53603101	53603400	3.430	3.081	2.481	3.067
StH	11	53664901	53665200	0.532	0.560	0.346	0.441
StH	11	53711401	53711700	3.005	2.305	9.338	3.669
StH	11	53711701	53712000	1.195	1.294	6.523	2.085
StH	11	53729701	53730000	2.650	3.278	5.337	3.225
StH	11	53792701	53793000	0.424	0.515	0.132	0.335
StH	11	53914201	53914500	0.452	0.234	0.658	0.025
StH	11	54004201	54004500	0.053	0.395	0.214	0.081
StH	11	54129001	54129300	0.319	0.444	0.967	0.527
StH	11	54182401	54182700	0.769	0.701	0.448	0.715
StH	11	54296101	54296400	2.997	2.997	3.997	2.225
StH	11	54422101	54422400	0.189	0.879	3.649	0.862
StH	11	54507601	54507900	0.929	0.770	0.374	0.974
StH	11	54627901	54628200	0.852	0.817	0.477	1.298
StH	11	54651001	54651300	0.823	1.460	4.821	1.105
StH	11	54667501	54667800	0.690	0.749	0.214	0.466
StH	11	54978901	54979200	0.027	0.294	1.681	0.153
StH	11	55114801	55115100	4.339	2.199	6.378	3.165
StH	11	55367101	55367400	0.292	0.637	2.924	0.061
StH	11	55474201	55474500	0.132	0.068	1.204	0.274
StH	11	55481401	55481700	4.814	4.680	3.393	3.995
StH	11	55593301	55593600	0.874	0.399	1.382	0.502
StH	11	56375701	56376000	0.081	0.431	2.724	0.284
StH	11	56414101	56414400	0.134	0.185	0.346	0.695
StH	11	56430601	56430900	3.054	3.227	2.069	1.420
StH	11	56454301	56454600	0.477	0.961	0.716	0.502
StH	11	56558401	56558700	1.252	1.285	0.798	1.161
StH	11	56649301	56649600	6.696	6.215	5.549	8.027
StH	11	56649601	56649900	2.421	1.866	3.114	2.763
StH	11	56649901	56650200	2.957	2.186	4.889	3.433
StH	11	56651101	56651400	0.774	0.963	1.846	0.583
StH	11	56728801	56729100	1.697	2.521	3.201	1.667
StH	11	56796601	56796900	0.452	0.135	1.259	0.624
StH	11	56821501	56821800	0.134	0.157	1.123	0.031
StH	11	56821801	56822100	0.505	1.492	4.958	0.761
StH	11	57004801	57005100	0.397	0.302	1.469	0.410
StH	11	57293101	57293400	3.275	3.469	1.933	4.005
StH	11	57383101	57383400	0.294	0.339	1.792	0.025
StH	11	57433801	57434100	0.932	0.909	2.786	0.948
StH	11	57556801	57557100	3.744	3.809	8.367	4.129
StH	11	58190401	58190700	0.773	0.727	0.395	0.907

StH	11	58224901	58225200	1.127	2.213	7.146	1.721
StH	11	58506901	58507200	0.398	0.281	0.658	0.365
StH	11	58615801	58616100	1.333	1.034	0.477	0.943
StH	11	58728601	58728900	0.105	0.189	0.448	0.223
StH	11	58843201	58843500	0.560	0.303	0.136	0.355
StH	11	58908601	58908900	0.027	0.094	0.390	0.031
StH	11	58920601	58920900	1.992	1.985	1.480	1.917
StH	11	58934401	58934700	1.312	1.392	2.806	1.400
StH	11	59161501	59161800	0.586	0.773	0.301	1.075
StH	11	59282401	59282700	0.160	0.305	0.506	0.248
StH	11	59303401	59303700	1.650	1.905	3.085	1.667
StH	11	59691901	59692200	1.216	1.167	2.602	1.374
StH	11	59736301	59736600	3.886	4.042	3.130	4.224
StH	11	59819701	59820000	0.457	0.702	1.226	0.477
StH	11	59979601	59979900	0.398	0.491	0.638	0.416
StH	11	60091201	60091500	2.104	1.167	1.650	1.881
StH	11	60091501	60091800	1.753	1.009	1.329	1.739
StH	11	60102001	60102300	0.907	1.152	4.237	1.025
StH	11	60102301	60102600	2.547	2.527	6.491	2.961
StH	11	60152401	60152700	1.702	1.776	1.460	2.018
StH	11	60235201	60235500	0.586	0.727	0.453	0.690
StH	11	60238501	60238800	1.384	1.411	5.810	0.974
StH	11	60239401	60239700	0.424	0.792	1.852	0.476
StH	11	60445801	60446100	4.842	4.359	10.045	4.771
StH	11	60492601	60492900	1.168	1.326	0.535	1.298
StH	11	60553801	60554100	1.647	1.450	2.229	1.926
StH	11	60558001	60558300	1.971	2.294	0.818	1.389
StH	11	60588301	60588600	4.093	1.650	0.682	2.089
StH	11	60626701	60627000	1.065	0.414	5.992	1.744
StH	11	60627601	60627900	2.738	1.826	3.032	2.470
StH	11	60637501	60637800	2.287	1.188	0.317	0.583
StH	11	60656101	60656400	0.426	0.446	0.214	0.466
StH	11	60660001	60660300	3.371	3.783	2.140	3.539
StH	11	60702301	60702600	3.113	2.982	4.060	2.911
StH	11	60768601	60768900	0.081	0.517	2.011	0.406
StH	11	60813301	60813600	0.374	0.796	0.111	0.421
StH	11	60822901	60823200	5.630	4.882	10.215	4.999
StH	11	60930901	60931200	0.027	0.204	0.938	0.182
StH	11	60955801	60956100	0.539	0.952	3.119	0.462
StH	11	60959101	60959400	2.573	2.958	4.354	2.637
StH	11	60966301	60966600	6.306	6.385	4.570	5.506
StH	11	61026601	61026900	2.896	2.555	3.794	2.925
StH	11	61092001	61092300	2.676	2.125	8.566	2.713
StH	11	61092301	61092600	1.384	1.236	0.682	1.633
StH	11	61130701	61131000	0.833	2.781	7.903	1.264
StH	11	61158301	61158600	1.331	1.765	3.097	2.059
StH	11	61218301	61218600	0.558	0.560	0.399	0.801
StH	11	61226101	61226400	1.046	1.332	2.171	0.801
StH	11	61283101	61283400	0.110	0.547	2.623	0.385
StH	11	61303201	61303500	1.131	1.388	3.268	1.344
StH	11	61488601	61488900	3.626	3.800	5.958	3.823
StH	11	61499401	61499700	1.269	1.103	2.341	1.307
StH	11	61500001	61500300	5.734	3.291	7.118	5.623
StH	11	61762201	61762500	2.435	2.371	5.166	2.367
StH	11	61762501	61762800	0.266	0.139	3.313	0.264
StH	11	61778701	61779000	0.192	0.723	2.713	0.452
StH	11	61795801	61796100	1.570	1.611	0.885	1.471
StH	11	61836601	61836900	0.497	0.748	2.634	0.527
StH	11	61841101	61841400	0.186	0.157	1.115	0.360
StH	11	61859101	61859400	1.752	1.916	3.629	1.790
StH	11	61867501	61867800	1.206	1.374	5.138	0.603
StH	11	61872901	61873200	0.587	1.308	5.797	0.436
StH	11	61873201	61873500	0.946	0.796	4.378	0.522
StH	11	61998901	61999200	0.082	0.112	1.019	0.152
StH	11	62007301	62007600	0.878	0.865	0.642	0.913
StH	12	321301	321600	1.513	1.757	1.021	1.546
StH	12	413401	413700	0.932	0.584	0.399	0.527

StH	12	665701	666000	2.925	2.575	2.053	2.378
StH	12	754801	755100	2.738	3.248	1.712	2.394
StH	12	826801	827100	0.134	0.420	0.263	0.081
StH	12	1168501	1168800	1.199	1.403	0.901	1.136
StH	12	1257601	1257900	2.399	1.946	4.664	1.825
StH	12	1404001	1404300	7.377	7.606	5.903	7.610
StH	12	1466401	1466700	0.745	0.584	0.312	0.690
StH	12	1641301	1641600	1.542	1.710	1.073	2.211
StH	12	1815301	1815600	0.584	0.672	1.193	0.365
StH	12	1896601	1896900	0.718	0.890	0.453	0.669
StH	12	1916701	1917000	0.184	0.260	0.729	0.218
StH	12	2067901	2068200	1.410	1.054	0.580	1.039
StH	12	2138401	2138700	0.324	0.903	3.622	0.634
StH	12	2181601	2181900	0.265	0.470	0.054	0.274
StH	12	2279101	2279400	1.460	2.060	0.959	1.374
StH	12	2332201	2332500	0.186	0.159	0.831	0.203
StH	12	2335501	2335800	1.432	0.970	3.535	1.496
StH	12	2456401	2456700	1.863	1.777	1.444	0.968
StH	12	2487001	2487300	0.745	0.676	0.272	0.745
StH	12	2631301	2631600	0.611	0.491	0.263	0.360
StH	12	2747101	2747400	0.611	0.583	0.930	0.452
StH	12	2937301	2937600	2.023	1.631	2.439	2.048
StH	12	2940901	2941200	1.116	0.727	1.275	0.907
StH	12	2961001	2961300	2.689	2.057	3.442	2.511
StH	12	3072301	3072600	5.443	6.171	4.251	5.928
StH	12	3087601	3087900	1.941	1.098	1.509	1.739
StH	12	3830401	3830700	2.102	2.274	1.587	2.327
StH	12	4740901	4741200	0.265	0.116	0.448	0.193
StH	12	5453101	5453400	0.187	0.023	0.477	0.309
StH	12	5602501	5602800	0.426	0.468	0.243	0.466
StH	12	6667801	6668100	0.242	0.047	0.774	0.233
StH	12	6701101	6701400	0.292	0.116	0.424	0.634
StH	12	9105601	9105900	0.373	0.305	0.609	0.385
StH	12	9239701	9240000	0.557	0.539	0.979	0.203
StH	12	9504301	9504600	2.892	1.991	1.159	3.264
StH	12	10110301	10110600	1.733	1.526	0.646	1.582
StH	12	10512901	10513200	0.292	0.421	0.667	0.268
StH	12	10752601	10752900	0.426	0.303	0.769	0.405
StH	12	11139001	11139300	0.079	0.140	0.497	0.132
StH	12	11319301	11319600	0.136	0.161	0.526	0.167
StH	12	12324901	12325200	2.389	1.427	1.900	1.440
StH	12	12936301	12936600	0.265	0.255	0.506	0.142
StH	12	14034301	14034600	0.560	0.981	0.370	0.852
StH	12	14123701	14124000	1.541	1.028	1.304	1.044
StH	12	14385301	14385600	0.373	0.399	0.156	0.578
StH	12	14497801	14498100	2.358	2.744	4.567	2.125
StH	12	15000001	15000300	0.342	0.489	0.925	0.436
StH	12	15515401	15515700	0.265	0.260	0.736	0.304
StH	12	16778701	16779000	1.678	1.146	0.573	1.825
StH	12	16820401	16820700	0.584	0.466	0.000	0.187
StH	12	17970001	17970300	0.878	0.403	1.246	0.740
StH	12	18153901	18154200	1.061	1.262	0.852	1.435
StH	12	18666301	18666600	0.797	1.098	0.468	1.192
StH	12	19197601	19197900	0.532	0.348	0.049	0.279
StH	12	19587001	19587300	0.187	0.140	1.806	0.187
StH	12	19587301	19587600	1.257	1.650	2.829	1.197
StH	12	19963201	19963500	0.847	1.122	1.879	0.974
StH	12	19992601	19992900	0.134	0.163	0.444	0.106
StH	12	20144701	20145000	0.106	0.097	0.622	0.117
StH	12	20390701	20391000	1.276	1.386	1.982	1.328
StH	12	20988901	20989200	0.160	0.328	0.535	0.355
StH	12	23675701	23676000	1.032	1.049	1.743	1.055
StH	12	24096301	24096600	0.265	0.348	0.638	0.380
StH	12	24672301	24672600	0.769	0.719	0.288	0.740
StH	12	24723601	24723900	0.587	0.446	1.041	0.634
StH	12	24945001	24945300	0.187	0.705	2.254	0.391
StH	12	25142401	25142700	1.381	1.122	0.872	1.166

StH	12	25325701	25326000	0.158	0.350	0.506	0.360
StH	12	25462501	25462800	0.053	0.112	0.622	0.031
StH	12	25755301	25755600	1.091	0.929	0.234	1.111
StH	12	26926501	26926800	0.853	0.725	0.370	0.776
StH	12	27077401	27077700	0.403	0.499	2.268	0.547
StH	12	28005901	28006200	0.637	0.611	0.992	0.558
StH	12	28183801	28184100	0.079	0.189	0.506	0.157
StH	12	28227901	28228200	1.326	1.450	0.531	1.182
StH	12	28398601	28398900	0.398	0.446	0.210	0.360
StH	12	28555201	28555500	0.053	0.092	0.341	0.588
StH	12	28962601	28962900	0.613	0.680	0.181	0.466
StH	12	29507101	29507400	1.489	1.126	0.575	0.933
StH	12	29587501	29587800	0.481	0.515	0.959	0.533
StH	12	29606401	29606700	0.929	0.824	0.259	1.029
StH	12	29889301	29889600	2.540	2.476	3.681	2.104
StH	12	30276001	30276300	1.913	3.274	2.180	1.111
StH	12	30471301	30471600	1.061	1.193	0.493	1.141
StH	12	30471601	30471900	0.611	0.654	0.296	0.776
StH	12	30650101	30650400	0.534	0.330	1.139	0.567
StH	12	30771601	30771900	0.424	0.444	0.321	0.193
StH	12	31252501	31252800	0.611	0.534	0.103	0.502
StH	12	31274701	31275000	0.582	0.699	0.267	0.553
StH	12	31275001	31275300	0.558	0.562	0.317	0.553
StH	12	31523701	31524000	0.453	0.423	0.716	0.198
StH	12	32527201	32527500	0.161	0.185	0.502	0.248
StH	12	32657701	32658000	0.213	0.236	0.510	0.167
StH	12	34351801	34352100	0.426	0.513	0.691	0.553
StH	12	34352101	34352400	0.636	0.562	1.300	0.918
StH	12	34373101	34373400	1.278	0.845	0.580	1.212
StH	12	34909501	34909800	0.081	0.305	0.506	0.193
StH	12	35336401	35336700	0.237	0.165	0.502	0.142
StH	12	36669001	36669300	0.639	0.897	2.370	0.441
StH	12	36858601	36858900	0.319	0.163	0.526	0.360
StH	12	38233801	38234100	1.199	1.307	0.905	1.410
StH	12	38629501	38629800	0.161	0.326	0.506	0.056
StH	12	39127501	39127800	0.398	0.558	0.160	0.411
StH	12	39303901	39304200	1.463	1.502	0.856	1.404
StH	12	39320101	39320400	0.745	0.320	1.476	0.497
StH	12	39329701	39330000	0.507	0.257	0.082	0.274
StH	12	39754501	39754800	0.536	0.515	1.159	0.578
StH	12	39976201	39976500	1.810	2.016	3.226	1.602
StH	12	40454101	40454400	0.558	0.584	0.745	0.497
StH	12	40605601	40605900	0.211	0.140	0.473	0.218
StH	12	40690201	40690500	2.123	2.137	1.201	2.033
StH	12	40797601	40797900	0.689	0.721	0.263	0.659
StH	12	41009701	41010000	1.432	2.551	1.942	2.236
StH	12	41073601	41073900	1.997	2.036	1.066	1.709
StH	12	41211301	41211600	2.978	2.233	3.937	2.799
StH	12	41797801	41798100	0.634	0.622	1.529	0.690
StH	12	42057601	42057900	1.413	1.429	0.963	1.435
StH	12	42353101	42353400	0.319	0.258	0.564	0.304
StH	12	42381301	42381600	3.376	2.899	3.763	3.255
StH	12	42436801	42437100	1.144	0.446	0.794	0.263
StH	12	42820501	42820800	1.145	0.867	0.633	0.913
StH	12	43176601	43176900	3.268	3.211	7.741	3.102
StH	12	43313101	43313400	0.665	0.796	0.506	0.279
StH	12	43486501	43486800	2.208	3.094	1.489	2.860
StH	12	43605001	43605300	0.161	0.189	0.555	0.117
StH	12	43648801	43649100	0.771	0.605	0.321	0.669
StH	12	43908001	43908300	0.318	0.208	0.736	0.268
StH	12	44046601	44046900	0.824	0.631	0.473	0.639
StH	12	44138401	44138700	1.095	1.169	1.893	1.131
StH	12	44145001	44145300	0.689	0.723	1.144	0.299
StH	12	44302801	44303100	0.929	1.099	0.617	0.259
StH	12	44432701	44433000	0.507	0.534	0.156	0.441
StH	12	44580601	44580900	2.178	1.961	1.144	1.750
StH	12	44594101	44594400	1.800	1.895	1.041	2.180

StH	12	44788501	44788800	0.347	1.706	0.967	0.481
StH	12	44801701	44802000	0.481	0.354	0.872	0.274
StH	12	44955301	44955600	5.080	5.422	3.961	4.214
StH	12	44960401	44960700	3.960	3.250	2.851	3.746
StH	12	45178801	45179100	0.852	0.800	0.272	0.745
StH	12	45742801	45743100	0.186	0.094	0.700	0.309
StH	12	46430101	46430400	0.560	0.586	0.292	0.628
StH	12	46691101	46691400	3.594	3.873	5.946	3.501
StH	12	46722301	46722600	0.740	0.701	1.427	0.826
StH	12	47213401	47213700	0.458	1.259	4.975	0.613
StH	12	47326501	47326800	0.529	0.584	1.028	0.441
StH	12	47344201	47344500	6.000	6.170	3.850	5.842
StH	12	47709001	47709300	2.794	2.122	3.830	2.819
StH	12	47747401	47747700	0.187	0.097	0.555	0.061
StH	12	47819701	47820000	1.616	1.661	0.992	1.546
StH	12	47871001	47871300	0.294	0.118	0.691	0.248
StH	12	47898001	47898300	1.381	1.474	1.119	1.546
StH	12	48009001	48009300	0.398	0.418	0.160	0.502
StH	12	48176401	48176700	0.824	0.725	0.370	0.578
StH	12	48179701	48180000	0.878	0.798	1.173	0.852
StH	12	48210301	48210600	0.052	0.274	1.209	0.086
StH	12	48223501	48223800	1.305	0.538	0.769	0.056
StH	12	48283201	48283500	0.106	0.652	1.177	0.542
StH	12	48299701	48300000	0.290	0.440	0.856	0.365
StH	12	48300001	48300300	0.500	0.958	3.283	0.927
StH	12	48300301	48300600	0.000	0.137	0.987	0.056
StH	12	48499201	48499500	2.897	2.579	1.609	2.656
StH	12	48888901	48889200	2.822	2.188	1.141	2.403
StH	12	49055101	49055400	0.371	0.470	0.029	0.380
StH	12	49152601	49152900	3.039	2.186	5.208	3.163
StH	12	49194901	49195200	0.265	0.135	0.896	0.223
StH	12	49195201	49195500	0.318	0.139	0.477	0.329
StH	12	49245001	49245300	0.105	0.114	0.448	0.117
StH	12	49297501	49297800	2.526	2.521	1.708	2.575
StH	12	49371901	49372200	0.319	0.255	0.785	0.167
StH	12	49397701	49398000	0.558	0.253	1.159	0.644
StH	12	49484101	49484400	0.505	0.401	0.823	0.466
StH	12	49878601	49878900	0.453	0.513	0.156	0.466
StH	12	50189701	50190000	2.473	2.695	3.217	2.702
StH	12	50293201	50293500	1.013	0.794	0.428	0.725
StH	12	50484001	50484300	0.345	0.702	0.453	0.776
StH	12	50612701	50613000	0.745	0.865	0.555	0.832
StH	12	50754001	50754300	0.907	0.772	0.374	0.846
StH	12	50813701	50814000	0.371	0.420	0.127	0.360
StH	12	51051601	51051900	4.393	4.141	2.954	5.351
StH	12	51504601	51504900	0.213	0.071	0.646	0.137
StH	12	51510001	51510300	0.105	0.092	0.317	0.547
StH	12	51841501	51841800	1.915	1.122	1.567	2.165
StH	12	53117101	53117400	1.303	1.824	1.411	1.658
StH	12	53228101	53228400	0.584	0.399	0.210	0.472
StH	12	53518501	53518800	0.186	0.163	0.551	0.142
StH	12	53620201	53620500	0.507	0.401	0.132	0.385
StH	12	54367201	54367500	0.161	0.236	0.633	0.173
StH	12	54577801	54578100	0.426	0.631	0.346	0.441
StH	12	55184101	55184400	1.625	0.818	0.481	1.739
StH	12	55270501	55270800	0.907	0.918	0.399	0.918
StH	12	55545901	55546200	0.132	0.212	0.473	0.081
StH	12	55985401	55985700	0.848	0.704	0.239	0.913
StH	12	56236201	56236500	1.355	1.474	0.992	1.410
StH	12	56637601	56637900	0.637	0.397	0.876	0.324
StH	12	56648101	56648400	0.905	0.513	0.687	0.279
StH	12	57365701	57366000	0.026	0.257	0.502	0.137
StH	12	58441801	58442100	2.500	3.059	2.040	2.632
StH	12	58702501	58702800	2.205	2.013	1.344	1.851
StH	12	60024601	60024900	0.266	0.350	0.074	0.558
StH	12	60125401	60125700	0.481	0.255	1.244	0.076
StH	12	60125701	60126000	0.923	1.015	2.581	0.973

StH	12	60685801	60686100	1.891	1.504	2.757	3.590
StH	12	60739201	60739500	0.160	0.140	0.486	0.076
StH	12	61162201	61162500	0.610	0.656	0.453	0.304
StH	12	61305001	61305300	3.268	2.845	1.197	1.916
StH	12	61407901	61408200	0.852	0.680	0.254	0.771
StH	12	61473901	61474200	5.665	5.353	4.813	5.455
StH	12	61550101	61550400	0.265	0.187	0.564	0.112
StH	12	61739701	61740000	1.997	1.933	3.098	1.937
StH	12	62257801	62258100	0.962	1.034	0.379	0.933
StH	12	62387401	62387700	2.926	4.181	2.102	2.820
StH	12	62947801	62948100	0.266	0.399	0.078	0.492
StH	12	63241201	63241500	0.373	0.305	0.555	0.086
StH	12	63331201	63331500	0.374	0.706	1.353	0.598
StH	12	63331501	63331800	0.105	0.564	1.057	0.213
StH	12	63521101	63521400	0.477	0.468	0.210	0.391
StH	12	63832201	63832500	0.929	1.356	0.609	0.862
StH	12	64012501	64012800	0.850	0.678	0.424	0.659
StH	12	64246801	64247100	0.158	0.376	0.584	0.274
StH	12	64337101	64337400	0.239	0.159	0.560	0.198
StH	12	65763301	65763600	0.371	0.397	0.996	0.203
StH	12	65763601	65763900	0.371	0.521	1.041	0.173
StH	12	65934901	65935200	0.163	0.255	0.765	0.193
StH	12	66851401	66851700	0.105	0.236	0.675	0.137
StH	12	67515601	67515900	1.515	2.053	0.959	1.532
StH	12	67515901	67516200	1.358	2.079	0.945	1.582
StH	12	67596601	67596900	0.132	0.538	0.263	0.081
StH	12	67708801	67709100	1.278	1.620	2.646	1.471
StH	12	68019901	68020200	0.079	0.096	0.555	0.101
StH	12	68330101	68330400	0.398	0.257	0.560	0.162
StH	12	69195001	69195300	0.213	0.045	0.691	0.106
StH	12	69299701	69300000	6.487	5.484	4.097	5.440
StH	12	69315001	69315300	3.267	3.274	2.504	4.351
StH	12	69982501	69982800	0.850	0.820	0.461	0.918
StH	12	70005601	70005900	0.397	0.331	0.827	0.365
StH	12	70692301	70692600	0.053	0.047	0.390	0.056
StH	12	70893301	70893600	0.639	0.446	0.160	0.477
StH	12	71075701	71076000	0.215	0.142	0.502	0.137
StH	12	71208301	71208600	0.452	0.397	0.720	0.421
StH	12	71625001	71625300	2.155	0.300	0.896	1.532
StH	12	71808901	71809200	0.347	0.303	0.127	0.527
StH	12	71844001	71844300	0.347	0.446	0.749	0.472
StH	12	72143101	72143400	0.290	0.560	0.107	0.360
StH	12	72382201	72382500	0.079	0.210	0.671	0.243
StH	12	72897301	72897600	0.211	0.212	0.477	0.167
StH	12	72967801	72968100	0.186	0.116	0.473	0.086
StH	12	73096201	73096500	3.965	3.478	2.604	3.042
StH	12	73244401	73244700	0.108	0.116	0.555	0.051
StH	12	73280701	73281000	0.663	0.678	0.267	0.441
StH	12	73299601	73299900	0.344	0.257	0.720	0.441
StH	12	73302301	73302600	3.608	2.879	1.469	2.940
StH	12	73782301	73782600	0.397	0.071	0.584	0.355
StH	12	73798801	73799100	0.479	1.169	0.156	0.720
StH	12	73935601	73935900	0.587	0.770	0.317	0.882
StH	12	74063101	74063400	2.823	3.038	2.390	2.023
StH	12	74326501	74326800	0.211	0.232	0.580	0.213
StH	12	74746801	74747100	3.823	4.019	2.615	4.283
StH	12	74834401	74834700	0.720	0.725	0.189	0.477
StH	12	74931301	74931600	1.016	0.843	3.511	1.038
StH	12	75090301	75090600	1.066	1.129	2.257	1.247
StH	12	75151501	75151800	0.081	0.330	0.477	0.167
StH	12	75178801	75179100	0.186	0.163	0.502	0.254
StH	12	75300301	75300600	1.147	1.120	0.185	0.639
StH	12	75300601	75300900	1.037	1.193	0.181	0.822
StH	12	75306601	75306900	0.160	0.515	0.399	0.223
StH	12	75584401	75584700	3.564	4.027	2.086	3.265
StH	12	75600901	75601200	0.132	0.255	1.070	0.314
StH	12	75676801	75677100	1.199	0.423	0.638	0.416

StH	12	75708901	75709200	0.529	0.418	0.107	0.715
StH	12	75710101	75710400	0.161	0.335	1.099	0.056
StH	12	75719701	75720000	4.027	3.901	2.662	4.082
StH	12	76249501	76249800	0.453	1.034	2.898	0.584
StH	12	76739701	76740000	0.824	1.143	1.679	1.029
StH	12	76892401	76892700	0.960	1.120	0.370	1.029
StH	12	77013601	77013900	0.318	0.328	0.502	0.335
StH	12	77234401	77234700	0.374	0.185	0.774	0.238
StH	12	77462401	77462700	0.531	0.747	0.399	0.715
StH	12	77520301	77520600	0.349	0.210	0.724	0.243
StH	12	77715301	77715600	1.465	0.848	0.321	1.587
StH	12	77720101	77720400	0.160	0.139	0.502	0.106
StH	12	77754301	77754600	0.240	0.440	1.589	0.223
StH	12	77801101	77801400	0.984	0.981	0.687	1.602
StH	12	77803201	77803500	0.428	0.493	1.627	0.315
StH	12	77842501	77842800	0.318	0.702	1.618	0.370
StH	12	77871901	77872200	0.079	0.112	0.510	0.167
StH	12	77882401	77882700	0.079	0.139	0.745	0.086
StH	12	77989801	77990100	0.742	0.800	2.313	0.304
StH	12	78044701	78045000	0.692	0.680	0.950	0.446
StH	12	78082501	78082800	0.318	0.326	0.078	0.436
StH	12	78133501	78133800	4.875	4.580	2.394	5.122
StH	12	78136501	78136800	0.053	0.045	0.651	0.173
StH	12	78175501	78175800	3.489	3.377	2.225	3.665
StH	12	78205501	78205800	1.068	1.538	3.357	1.156
StH	12	78266101	78266400	0.347	0.326	0.535	0.335
StH	12	78441901	78442200	0.052	0.204	0.749	0.147
StH	12	78442501	78442800	0.769	0.472	2.198	0.517
StH	12	78462001	78462300	1.168	1.617	5.500	1.222
StH	12	78502201	78502500	0.218	0.727	2.358	0.304
StH	12	78767101	78767400	0.397	0.270	1.456	0.533
StH	12	78805201	78805500	0.529	0.322	1.511	0.634
StH	12	78880801	78881100	0.345	0.731	2.086	0.284
StH	12	78900301	78900600	0.476	0.303	0.885	0.502
StH	12	78922801	78923100	1.367	1.721	3.404	1.573
StH	12	79007401	79007700	0.215	0.069	0.662	0.142
StH	12	79014301	79014600	0.103	0.225	1.061	0.193
StH	12	79032001	79032300	0.187	0.184	0.477	0.106
StH	13	138301	138600	2.796	2.901	2.062	2.727
StH	13	1047001	1047300	0.824	0.891	1.266	0.659
StH	13	1201501	1201800	0.582	0.912	1.304	0.837
StH	13	1206301	1206600	0.769	0.633	0.214	0.533
StH	13	1459201	1459500	0.216	0.257	0.778	0.335
StH	13	1710601	1710900	0.694	0.253	1.645	0.416
StH	13	1711501	1711800	0.345	0.303	0.617	0.086
StH	13	1868401	1868700	0.324	0.257	0.794	0.248
StH	13	1992001	1992300	0.316	0.466	0.774	0.391
StH	13	1992301	1992600	0.347	0.631	1.032	0.441
StH	13	2080501	2080800	1.728	1.802	0.798	1.905
StH	13	3207001	3207300	0.507	0.633	0.160	0.472
StH	13	3330601	3330900	0.507	0.493	0.317	1.075
StH	13	3377101	3377400	2.260	2.547	1.810	2.130
StH	13	3433501	3433800	1.674	1.751	2.550	1.095
StH	13	3437701	3438000	0.213	0.373	0.716	0.315
StH	13	4303801	4304100	0.584	0.466	0.263	0.081
StH	13	4378201	4378500	0.345	0.423	1.023	0.441
StH	13	4495201	4495500	0.931	1.710	0.584	0.877
StH	13	4522801	4523100	0.294	0.328	0.477	0.248
StH	13	4938601	4938900	0.720	0.685	1.765	0.958
StH	13	5074501	5074800	1.224	1.047	0.535	1.024
StH	13	5329201	5329500	0.718	1.588	1.090	0.801
StH	13	5582401	5582700	0.502	0.539	0.078	0.502
StH	13	6552001	6552300	0.505	0.397	0.132	0.416
StH	13	6574501	6574800	0.694	0.584	0.317	0.695
StH	13	6919201	6919500	0.931	0.933	0.383	0.954
StH	13	7033501	7033800	0.665	0.701	0.901	0.527
StH	13	7327501	7327800	1.516	0.940	2.028	1.070

StH	13	7391401	7391700	0.507	0.350	0.132	0.360
StH	13	7991701	7992000	0.131	0.118	0.448	0.056
StH	13	8074801	8075100	0.452	0.373	0.687	0.329
StH	13	8154001	8154300	1.010	0.584	1.217	0.411
StH	13	8458501	8458800	0.876	1.148	0.535	0.771
StH	13	9051301	9051600	0.136	0.139	0.473	0.056
StH	13	9121201	9121500	0.531	0.350	0.185	0.385
StH	13	9240001	9240300	0.586	0.701	0.424	0.329
StH	13	9416701	9417000	0.450	0.163	0.753	0.112
StH	13	9702601	9702900	0.587	0.629	0.194	0.751
StH	13	9847801	9848100	0.213	0.262	0.938	0.264
StH	13	9850201	9850500	3.583	3.373	2.439	4.791
StH	13	9865501	9865800	2.129	1.723	0.769	1.942
StH	13	9872701	9873000	3.627	3.881	2.160	3.936
StH	13	9948001	9948300	0.052	0.215	0.778	0.112
StH	13	10200301	10200600	0.850	0.796	0.453	0.690
StH	13	10265401	10265700	1.245	1.338	3.192	1.121
StH	13	10401301	10401600	5.456	5.402	4.385	4.761
StH	13	10442101	10442400	0.932	0.912	0.535	0.852
StH	13	10778401	10778700	0.345	0.605	0.185	0.634
StH	13	11034001	11034300	0.265	0.371	0.798	0.193
StH	13	11148001	11148300	1.886	1.476	2.307	1.805
StH	13	11189701	11190000	5.215	5.274	3.868	5.664
StH	13	11208001	11208300	1.436	1.614	1.197	1.552
StH	13	11228101	11228400	2.212	2.553	1.378	2.165
StH	13	11610301	11610600	0.907	0.938	0.288	1.125
StH	13	11988301	11988600	0.426	0.633	0.292	0.466
StH	13	12250501	12250800	0.584	0.562	0.140	0.416
StH	13	12285901	12286200	0.237	0.283	0.642	0.167
StH	13	13898401	13898700	3.033	2.405	1.897	2.545
StH	13	14923501	14923800	0.424	0.564	0.950	0.167
StH	13	15119701	15120000	3.007	3.341	2.221	2.859
StH	13	15235501	15235800	2.308	2.654	1.317	0.304
StH	13	15333001	15333300	1.407	0.305	0.642	0.294
StH	13	15439201	15439500	0.373	0.657	0.029	0.248
StH	13	15563401	15563700	0.294	0.583	0.078	0.304
StH	13	16150201	16150500	0.082	0.139	0.448	0.162
StH	13	16326301	16326600	0.819	1.309	1.893	0.756
StH	13	16488601	16488900	0.984	1.450	1.197	1.445
StH	13	16898101	16898400	2.263	2.051	1.427	2.216
StH	13	17001001	17001300	1.077	1.890	4.336	1.455
StH	13	17101201	17101500	0.423	0.446	0.136	0.472
StH	13	17461501	17461800	0.158	0.163	0.745	0.396
StH	13	17480401	17480700	2.983	4.025	3.504	4.264
StH	13	18141001	18141300	0.716	0.423	0.954	0.385
StH	13	18534901	18535200	0.903	0.515	1.206	0.852
StH	13	18713701	18714000	0.106	0.118	0.497	0.167
StH	13	18717601	18717900	1.244	1.073	2.851	0.583
StH	13	19271701	19272000	0.027	0.165	0.444	0.167
StH	13	20547601	20547900	0.452	0.566	0.979	0.492
StH	13	20893501	20893800	0.211	0.094	0.526	0.117
StH	13	21715801	21716100	0.318	0.326	0.662	0.243
StH	13	22046401	22046700	2.738	2.946	2.312	2.657
StH	13	22585801	22586100	0.106	0.116	0.506	0.061
StH	13	22743301	22743600	1.140	1.122	0.428	0.979
StH	13	22791301	22791600	1.249	1.781	0.856	1.328
StH	13	23172601	23172900	1.571	0.914	2.778	0.781
StH	13	23242501	23242800	0.161	0.185	0.588	0.193
StH	13	23561101	23561400	1.673	2.027	0.872	1.410
StH	13	24221701	24222000	0.692	0.936	2.428	0.792
StH	13	24380701	24381000	0.508	1.686	0.979	0.452
StH	13	25227601	25227900	1.520	1.054	0.526	0.979
StH	13	26001001	26001300	0.452	0.378	0.745	1.192
StH	13	26121901	26122200	0.239	0.236	0.526	0.031
StH	13	26698801	26699100	0.720	0.723	0.403	0.725
StH	13	27007501	27007800	0.797	1.569	1.188	0.731
StH	13	27018301	27018600	3.325	1.912	2.827	3.498

StH	13	27030301	27030600	1.166	1.137	0.267	0.907
StH	13	27032101	27032400	2.204	2.030	0.337	1.683
StH	13	27545701	27546000	0.398	0.376	0.745	0.522
StH	13	28175701	28176000	0.373	0.420	0.531	0.360
StH	13	28864501	28864800	0.477	0.444	0.185	0.416
StH	13	29278201	29278500	0.373	0.375	0.160	0.603
StH	13	29599501	29599800	0.215	0.139	0.555	0.132
StH	13	29617201	29617500	1.625	1.663	0.831	1.420
StH	13	30162001	30162300	0.426	0.071	0.860	0.203
StH	13	30385801	30386100	0.239	0.234	0.132	0.497
StH	13	30512101	30512400	0.158	0.140	0.477	0.198
StH	13	30532801	30533100	0.400	0.376	0.165	0.690
StH	13	30924001	30924300	0.532	0.236	0.000	0.137
StH	13	31124101	31124400	1.331	1.073	0.613	1.116
StH	13	31558801	31559100	0.240	0.234	0.428	0.751
StH	13	31573501	31573800	0.345	0.375	0.160	0.634
StH	13	31685101	31685400	0.426	0.442	0.613	0.329
StH	13	31959901	31960200	0.265	0.629	0.930	0.558
StH	13	31980601	31980900	0.132	0.212	0.497	0.248
StH	13	32104201	32104500	2.896	3.291	1.757	2.891
StH	13	32116801	32117100	0.450	0.584	0.058	0.329
StH	13	32118601	32118900	0.265	0.491	0.025	0.416
StH	13	32253601	32253900	0.587	0.538	0.239	0.416
StH	13	32303701	32304000	0.453	0.420	0.239	0.112
StH	13	32907001	32907300	0.745	0.936	0.506	0.634
StH	13	33447601	33447900	0.505	0.609	0.243	0.411
StH	13	33538501	33538800	5.708	4.757	7.154	8.801
StH	13	33567001	33567300	1.065	0.635	0.214	0.812
StH	13	33648301	33648600	0.536	0.513	0.185	0.522
StH	13	33715501	33715800	0.136	0.071	0.510	0.137
StH	13	33865501	33865800	0.428	0.213	0.798	0.527
StH	13	34133401	34133700	2.739	2.504	1.908	2.515
StH	13	34434901	34435200	0.584	0.656	0.346	0.112
StH	13	34452001	34452300	0.106	0.257	0.613	0.309
StH	13	34548001	34548300	0.477	0.399	0.111	0.466
StH	13	34615201	34615500	0.853	0.818	1.420	0.933
StH	13	34727701	34728000	0.397	0.489	0.872	0.396
StH	13	35340601	35340900	0.558	0.935	0.370	0.553
StH	13	35554801	35555100	0.879	0.699	0.259	0.583
StH	13	36163201	36163500	0.160	0.161	0.526	0.274
StH	13	36165601	36165900	0.106	0.116	0.390	0.112
StH	13	36689701	36690000	0.294	0.442	0.078	0.279
StH	13	36973201	36973500	2.094	2.716	1.061	1.840
StH	13	37060501	37060800	0.106	0.163	0.486	0.223
StH	13	37105201	37105500	5.909	6.529	7.934	6.839
StH	13	37269001	37269300	1.537	1.720	3.360	2.266
StH	13	37373401	37373700	0.132	1.006	0.403	0.583
StH	13	37380301	37380600	0.928	0.796	0.453	0.974
StH	13	37504501	37504800	1.462	2.221	1.879	2.130
StH	13	38221201	38221500	0.479	0.491	0.827	0.329
StH	13	38221501	38221800	0.584	0.539	0.769	0.274
StH	13	38351101	38351400	1.805	1.513	0.959	1.820
StH	13	38527501	38527800	0.160	0.704	0.403	0.193
StH	13	38644801	38645100	1.170	0.912	0.526	1.039
StH	13	38872801	38873100	0.186	0.283	0.638	0.421
StH	13	39236701	39237000	0.106	0.187	0.321	0.466
StH	13	39396301	39396600	0.026	0.421	0.156	0.801
StH	13	39498601	39498900	0.105	0.191	0.535	0.025
StH	13	39512401	39512700	0.718	0.328	0.560	0.335
StH	13	39961501	39961800	0.903	0.935	0.444	1.004
StH	13	40051801	40052100	0.665	0.886	1.572	1.172
StH	13	40362001	40362300	0.319	0.420	0.103	0.304
StH	13	40548601	40548900	0.268	0.395	0.667	0.223
StH	13	40853701	40854000	0.611	1.051	0.769	0.329
StH	13	40898101	40898400	0.452	1.030	0.317	0.664
StH	13	41068201	41068500	1.733	1.925	0.934	1.734
StH	13	41068501	41068800	1.863	2.015	0.941	1.825

StH	13	41131201	41131500	0.289	0.457	2.451	0.461
StH	13	41274301	41274600	0.639	0.749	0.254	0.715
StH	13	41415901	41416200	0.905	0.935	0.058	0.385
StH	13	41483701	41484000	0.294	0.491	0.054	0.335
StH	13	41555401	41555700	0.268	0.354	0.749	0.360
StH	13	41688601	41688900	0.398	0.515	0.189	0.416
StH	13	42102001	42102300	0.266	0.515	0.058	0.360
StH	13	42378001	42378300	4.386	4.368	4.097	5.415
StH	13	42779101	42779400	0.984	0.886	0.539	0.999
StH	13	43483501	43483800	0.213	0.605	0.930	0.644
StH	13	43771801	43772100	0.584	0.773	0.341	0.527
StH	13	43798801	43799100	0.026	0.045	0.573	0.031
StH	13	44000401	44000700	0.160	0.142	0.448	0.132
StH	13	44176201	44176500	0.610	0.612	0.185	0.578
StH	13	44478001	44478300	3.909	2.573	2.896	3.737
StH	13	45036001	45036300	3.209	3.470	1.356	3.174
StH	13	45199801	45200100	0.876	1.004	0.531	0.963
StH	13	45475801	45476100	0.984	0.961	0.555	1.136
StH	13	45642601	45642900	0.900	1.081	0.395	1.075
StH	13	45917701	45918000	0.400	0.629	0.214	0.522
StH	13	46022101	46022400	0.474	0.686	2.242	0.350
StH	13	46022401	46022700	0.129	0.405	1.745	0.132
StH	13	46395301	46395600	0.424	0.395	0.160	0.472
StH	13	47931901	47932200	0.694	0.611	0.959	0.553
StH	13	48712801	48713100	0.187	0.140	0.510	0.198
StH	13	50353501	50353800	0.213	0.307	0.662	0.324
StH	13	50421601	50421900	0.903	0.843	0.642	0.913
StH	13	51180301	51180600	1.254	0.981	0.481	0.882
StH	13	51320101	51320400	0.613	0.680	0.292	0.715
StH	13	51488101	51488400	1.065	1.079	1.670	0.887
StH	13	51950701	51951000	1.553	1.566	4.899	2.038
StH	13	51951001	51951300	0.261	0.480	2.623	0.643
StH	13	52112401	52112700	0.319	0.538	0.078	0.198
StH	13	52155001	52155300	0.824	0.935	0.720	0.522
StH	13	52194301	52194600	0.452	0.491	0.263	0.492
StH	13	52514401	52514700	1.657	1.778	3.946	1.816
StH	13	52530001	52530300	0.319	0.818	0.481	0.279
StH	13	52668301	52668600	1.783	1.878	1.217	2.347
StH	13	52792501	52792800	5.719	5.433	4.467	6.561
StH	13	53092801	53093100	0.240	0.328	0.720	0.279
StH	13	53146501	53146800	0.053	0.296	0.798	0.000
StH	13	53147101	53147400	0.210	0.364	1.482	0.228
StH	13	53374801	53375100	0.106	0.232	0.617	0.268
StH	13	53442601	53442900	1.224	1.193	0.930	1.222
StH	13	53708701	53709000	0.503	0.747	0.127	0.421
StH	13	53754301	53754600	0.316	0.369	1.066	0.497
StH	13	53757001	53757300	0.371	1.032	0.662	0.309
StH	13	53804401	53804700	0.186	0.258	0.584	0.218
StH	13	53820601	53820900	0.931	0.727	0.453	0.715
StH	13	54063001	54063300	0.608	0.659	1.168	0.436
StH	13	54131401	54131700	0.610	0.685	1.193	0.578
StH	13	54494701	54495000	2.160	1.984	1.161	2.160
StH	13	54511201	54511500	0.421	0.397	0.827	0.391
StH	13	54616501	54616800	0.452	0.212	0.658	0.446
StH	13	54699601	54699900	4.523	4.784	2.232	4.994
StH	13	54699901	54700200	3.507	2.914	1.801	3.884
StH	13	54745201	54745500	0.814	0.569	1.921	0.679
StH	13	54776701	54777000	0.265	0.562	0.769	0.533
StH	13	54921601	54921900	1.647	2.223	0.765	1.476
StH	13	55061701	55062000	0.319	0.305	0.658	0.416
StH	13	55082101	55082400	1.010	0.912	0.609	1.044
StH	13	55087801	55088100	0.848	0.773	0.481	1.024
StH	13	55132801	55133100	0.371	0.302	0.049	0.497
StH	13	55377001	55377300	1.060	1.453	1.917	1.217
StH	13	55430101	55430400	1.115	1.096	1.411	0.943
StH	13	55480501	55480800	0.637	0.674	0.346	0.659
StH	13	55526701	55527000	0.373	0.757	1.638	0.512

StH	13	55568101	55568400	1.570	1.474	1.884	1.683
StH	13	55690501	55690800	0.373	0.468	0.210	0.304
StH	13	55691701	55692000	0.613	0.539	0.267	0.695
StH	13	55773001	55773300	0.265	0.232	0.477	0.274
StH	13	55860601	55860900	0.240	0.285	0.584	0.193
StH	13	56077501	56077800	1.168	1.427	1.728	1.359
StH	13	56389501	56389800	0.477	0.281	0.054	0.198
StH	13	56389801	56390100	0.531	0.281	0.160	0.553
StH	13	56691301	56691600	0.531	0.910	0.716	0.527
StH	13	56777401	56777700	3.938	4.165	2.936	3.524
StH	13	56797201	56797500	0.823	0.629	1.340	0.446
StH	13	56827801	56828100	1.674	1.763	3.651	1.781
StH	13	57007801	57008100	1.671	2.047	3.201	1.770
StH	13	57147601	57147900	0.186	0.255	0.506	0.132
StH	13	57207601	57207900	0.263	0.523	1.514	0.502
StH	13	57207901	57208200	0.529	0.525	1.665	0.680
StH	13	57607501	57607800	0.957	1.122	1.489	0.943
StH	13	57742201	57742500	0.266	0.416	1.130	0.436
StH	13	58047601	58047900	2.937	3.653	5.822	3.199
StH	13	58114801	58115100	1.037	0.421	0.321	0.882
StH	13	58228501	58228800	0.134	1.000	2.069	1.257
StH	13	58228801	58229100	0.053	1.214	2.633	1.886
StH	13	58266001	58266300	0.852	0.562	1.037	0.527
StH	13	58929001	58929300	1.066	1.566	0.740	1.404
StH	13	58998301	58998600	0.158	0.279	0.477	0.304
StH	13	59201401	59201700	1.884	1.755	3.241	2.201
StH	13	59229601	59229900	0.079	0.208	0.539	0.193
StH	13	59301901	59302200	0.477	0.307	0.934	0.106
StH	13	59441101	59441400	4.544	1.826	5.663	3.655
StH	13	59496301	59496600	0.560	0.676	0.321	0.608
StH	13	59648101	59648400	0.374	0.560	0.136	0.497
StH	13	59777701	59778000	0.747	0.702	0.395	0.857
StH	13	59828401	59828700	0.371	0.513	0.132	0.274
StH	13	60021001	60021300	0.211	0.258	0.477	0.274
StH	13	60385801	60386100	0.000	0.139	0.473	0.025
StH	13	60779701	60780000	0.314	0.371	0.954	0.274
StH	13	60819601	60819900	0.402	0.549	2.339	0.365
StH	13	60819901	60820200	0.455	0.390	1.850	0.421
StH	13	60974101	60974400	0.158	0.362	1.222	0.213
StH	13	61980301	61980600	2.626	2.718	1.166	3.245
StH	13	62271901	62272200	3.460	3.317	2.490	3.524
StH	13	62286901	62287200	0.160	0.165	0.419	0.106
StH	13	62459101	62459400	2.308	2.626	1.333	1.982
StH	13	62527501	62527800	0.637	0.466	0.267	0.446
StH	13	62637901	62638200	1.039	1.384	0.609	1.044
StH	13	62705701	62706000	0.773	0.749	0.399	0.603
StH	13	63798301	63798600	0.052	0.071	0.546	0.112
StH	13	63798601	63798900	0.000	0.068	0.624	0.025
StH	13	63857401	63857700	0.208	0.373	0.831	0.304
StH	13	64282501	64282800	1.439	1.543	0.811	1.451
StH	13	64346101	64346400	6.041	5.639	3.803	5.396
StH	13	64349701	64350000	0.290	0.302	0.753	0.061
StH	13	64520401	64520700	2.533	1.777	1.081	1.673
StH	13	64753801	64754100	3.890	1.137	2.777	1.471
StH	13	64793401	64793700	0.507	0.373	0.239	0.578
StH	13	65080201	65080500	1.008	0.910	1.601	1.151
StH	13	65217901	65218200	0.749	0.491	0.103	0.558
StH	13	65259001	65259300	7.679	8.712	3.067	7.204
StH	13	65467801	65468100	2.441	2.510	1.473	2.753
StH	13	65841001	65841300	0.240	0.792	0.448	0.112
StH	13	65887801	65888100	0.532	0.397	0.239	0.112
StH	13	65938801	65939100	1.992	1.403	0.987	1.982
StH	13	66112801	66113100	0.292	0.140	0.716	0.345
StH	13	66158101	66158400	0.455	0.539	0.127	0.527
StH	13	66421501	66421800	0.105	0.253	1.427	0.182
StH	13	66572701	66573000	0.560	0.963	0.243	0.583
StH	13	66909001	66909300	0.771	0.560	0.292	0.533

StH	13	66991801	66992100	1.039	0.959	1.251	0.999
StH	13	67318801	67319100	0.266	0.515	0.370	0.583
StH	13	67442401	67442700	0.742	0.676	1.572	1.120
StH	13	67453801	67454100	0.560	0.654	0.214	0.502
StH	13	67460701	67461000	2.412	2.722	0.698	2.165
StH	13	67827001	67827300	0.989	1.401	0.477	1.100
StH	13	68007601	68007900	0.584	0.210	0.769	0.243
StH	13	68214601	68214900	0.481	0.444	0.798	0.502
StH	13	69166201	69166500	1.170	1.498	0.749	1.100
StH	13	69264901	69265200	2.844	3.014	1.986	2.997
StH	13	69433201	69433500	5.322	5.130	3.376	4.949
StH	13	70186501	70186800	0.558	0.678	0.288	0.578
StH	13	70478101	70478400	1.647	1.382	0.881	1.465
StH	13	70485301	70485600	0.531	0.603	1.230	0.360
StH	13	70497901	70498200	0.319	0.142	0.535	0.162
StH	13	70715401	70715700	0.319	0.300	0.609	0.000
StH	13	70716601	70716900	0.161	0.140	0.477	0.304
StH	13	71046601	71046900	0.160	0.373	0.535	0.304
StH	13	71364301	71364600	1.699	1.493	2.278	1.845
StH	13	71384701	71385000	0.924	0.751	1.645	0.538
StH	13	71484601	71484900	0.371	0.444	0.774	0.452
StH	13	71652901	71653200	0.532	0.607	0.399	0.826
StH	13	71743201	71743500	0.184	0.352	0.885	0.288
StH	13	71901001	71901300	0.266	0.330	0.609	0.441
StH	13	71962501	71962800	0.240	0.539	0.000	0.187
StH	13	72036601	72036900	0.450	0.303	0.132	0.304
StH	13	72107101	72107400	3.268	3.461	4.196	3.356
StH	13	72195901	72196200	0.265	0.472	1.790	0.340
StH	13	72429001	72429300	1.515	1.195	0.687	1.283
StH	13	72620101	72620400	1.753	1.772	1.387	0.821
StH	13	72790201	72790500	0.557	0.538	0.239	0.664
StH	13	72837301	72837600	0.931	1.075	0.827	1.273
StH	13	73069801	73070100	0.613	0.609	0.930	0.659
StH	13	73835701	73836000	0.239	0.395	0.025	0.355
StH	13	73840501	73840800	0.874	0.914	0.283	0.725
StH	13	74337901	74338200	6.359	6.008	5.044	6.059
StH	13	74352301	74352600	0.507	0.517	0.881	0.527
StH	13	74394001	74394300	0.132	0.285	0.613	0.329
StH	13	74821501	74821800	0.663	0.747	0.267	0.664
StH	13	74961001	74961300	0.428	0.493	0.950	0.664
StH	13	75290101	75290400	0.158	0.339	1.414	0.315
StH	13	75363001	75363300	1.659	1.673	3.036	1.314
StH	13	75384601	75384900	0.558	0.812	4.649	0.628
StH	13	75492601	75492900	2.015	4.328	6.991	2.429
StH	13	75493501	75493800	0.818	0.640	1.806	0.705
StH	13	75494101	75494400	1.278	1.317	2.741	1.009
StH	13	75495301	75495600	1.174	1.101	2.196	0.755
StH	13	75495601	75495900	1.171	1.109	2.122	0.781
StH	13	75497701	75498000	1.397	1.719	3.600	1.627
StH	13	75498301	75498600	0.294	0.701	1.235	0.441
StH	13	75503701	75504000	0.294	0.421	0.794	0.238
StH	13	75756901	75757200	2.765	2.753	1.612	2.338
StH	13	75852301	75852600	2.126	2.223	2.731	3.042
StH	13	75865201	75865500	1.195	1.474	0.831	0.522
StH	13	76091701	76092000	7.718	7.342	9.811	9.076
StH	13	76123501	76123800	0.163	0.302	0.749	0.213
StH	13	76166701	76167000	0.474	0.534	1.041	0.365
StH	13	76184701	76185000	0.081	0.208	0.502	0.162
StH	13	76208101	76208400	0.213	0.163	0.568	0.081
StH	13	76270501	76270800	0.373	0.420	0.160	0.502
StH	13	76629301	76629600	0.452	0.376	0.638	0.223
StH	13	76654201	76654500	1.116	0.841	0.613	0.806
StH	13	76953001	76953300	1.408	1.126	0.444	0.882
StH	13	76995001	76995300	2.763	1.500	2.176	2.884
StH	13	76998001	76998300	0.850	0.820	1.037	0.659
StH	13	77185501	77185800	1.966	2.223	2.555	1.764
StH	13	77202001	77202300	2.025	1.103	2.513	1.460

StH	13	77678101	77678400	2.786	2.834	1.765	2.611
StH	13	77678401	77678700	1.707	1.796	0.914	1.800
StH	13	77865001	77865300	2.446	2.845	3.569	2.707
StH	13	77920801	77921100	3.960	4.068	3.221	3.726
StH	13	78019801	78020100	0.105	0.230	0.638	0.117
StH	13	78163801	78164100	0.269	0.069	0.945	0.329
StH	13	78216001	78216300	1.752	1.824	0.916	1.871
StH	13	78312001	78312300	0.190	0.234	0.885	0.162
StH	13	78337501	78337800	1.458	0.835	2.669	1.729
StH	13	78429601	78429900	0.314	0.331	0.827	0.025
StH	13	78662401	78662700	1.010	1.568	0.905	1.688
StH	13	78715501	78715800	1.328	1.793	2.691	1.197
StH	13	78743401	78743700	0.479	0.395	0.107	0.436
StH	13	78812401	78812700	1.789	2.405	4.298	1.805
StH	13	78872401	78872700	0.852	0.279	0.424	0.745
StH	13	79005601	79005900	1.250	0.957	0.658	0.938
StH	13	79032601	79032900	0.661	0.566	1.226	0.375
StH	13	79032901	79033200	0.239	0.257	0.604	0.137
StH	13	79034701	79035000	5.033	3.420	4.113	4.913
StH	13	79096801	79097100	0.371	0.493	0.136	0.502
StH	13	79134301	79134600	0.721	0.635	0.243	0.715
StH	13	79153201	79153500	1.544	0.873	0.379	1.359
StH	13	79231801	79232100	0.368	0.069	1.019	0.355
StH	13	79241401	79241700	1.516	1.918	1.066	1.496
StH	13	79397401	79397700	0.321	0.326	0.938	0.329
StH	13	79440601	79440900	2.365	2.613	3.217	2.586
StH	13	79720801	79721100	0.132	0.069	0.444	0.167
StH	13	79810501	79810800	0.800	0.777	1.728	0.827
StH	13	79852201	79852500	0.584	0.373	0.214	0.416
StH	13	80092501	80092800	1.197	1.289	0.658	1.024
StH	13	80172601	80172900	0.690	0.328	0.555	0.360
StH	13	80395501	80395800	1.647	1.875	1.217	1.627
StH	13	80492701	80493000	0.397	0.328	0.749	0.335
StH	13	80622601	80622900	1.999	2.225	3.558	2.120
StH	13	80622901	80623200	5.209	4.810	6.830	5.011
StH	13	80798701	80799000	1.966	2.540	4.206	2.580
StH	13	80883001	80883300	2.129	1.403	1.032	1.699
StH	13	80940301	80940600	2.152	2.086	2.579	1.714
StH	13	81060901	81061200	3.799	3.594	2.407	3.235
StH	13	81072001	81072300	0.977	0.461	2.102	0.781
StH	13	81129001	81129300	0.955	0.843	1.304	1.024
StH	13	81177001	81177300	0.426	0.489	0.267	0.416
StH	13	81265801	81266100	2.447	2.544	3.870	2.541
StH	13	81400201	81400500	4.249	4.385	2.809	3.950
StH	13	81434401	81434700	1.041	2.461	0.049	1.029
StH	13	81441301	81441600	0.610	0.515	1.168	0.299
StH	13	81574201	81574500	0.828	0.738	1.910	0.857
StH	13	81654301	81654600	0.903	1.145	3.775	1.424
StH	13	82014601	82014900	1.996	2.223	1.518	2.089
StH	13	82297501	82297800	0.823	1.420	3.223	0.883
StH	13	82401301	82401600	0.160	0.255	0.506	0.193
StH	13	82524601	82524900	3.909	3.974	2.633	3.632
StH	13	82634101	82634400	2.533	2.315	1.558	2.292
StH	13	82736701	82737000	0.984	1.678	0.613	0.913
StH	13	82737901	82738200	1.623	1.684	3.776	1.750
StH	13	82852801	82853100	2.550	1.970	1.161	1.952
StH	13	82978801	82979100	2.212	1.822	2.780	1.993
StH	13	83020201	83020500	0.611	1.053	0.798	0.882
StH	14	539701	540000	1.333	1.216	0.843	1.597
StH	14	567001	567300	0.797	1.595	4.997	0.751
StH	14	567301	567600	1.163	1.608	5.059	1.268
StH	14	597601	597900	1.515	2.032	2.782	0.882
StH	14	630001	630300	2.284	1.776	3.137	1.683
StH	14	645301	645600	0.160	0.238	0.584	0.187
StH	14	663001	663300	0.319	0.257	0.638	0.228
StH	14	678901	679200	1.584	0.757	3.221	1.480
StH	14	882301	882600	0.239	0.540	2.345	0.203

StH	14	947401	947700	1.627	1.476	4.240	1.511
StH	14	1074001	1074300	1.460	1.330	0.925	1.633
StH	14	1118701	1119000	5.885	5.257	2.804	6.962
StH	14	1203901	1204200	0.266	0.204	1.073	0.106
StH	14	1328701	1329000	0.718	0.888	0.350	0.725
StH	14	1608901	1609200	0.742	1.077	0.395	0.862
StH	14	1768201	1768500	0.720	0.867	0.448	0.796
StH	14	1879201	1879500	1.229	1.102	3.994	1.309
StH	14	1964101	1964400	0.053	0.112	0.609	0.223
StH	14	2087401	2087700	1.279	1.335	1.835	1.283
StH	14	2190301	2190600	0.818	0.839	0.210	0.776
StH	14	2254201	2254500	1.702	1.807	0.921	1.552
StH	14	2276101	2276400	0.640	0.916	0.181	0.812
StH	14	2724301	2724600	0.319	0.399	0.531	0.218
StH	14	3097501	3097800	0.266	0.277	0.560	0.137
StH	14	3320101	3320400	0.215	0.444	0.025	0.360
StH	14	3372001	3372300	5.122	5.413	2.902	4.863
StH	14	4137901	4138200	0.136	0.375	0.802	0.218
StH	14	4247401	4247700	0.639	0.866	2.490	0.619
StH	14	4290901	4291200	0.878	0.963	0.555	0.918
StH	14	4429201	4429500	1.303	1.197	0.609	0.994
StH	14	4667401	4667700	2.394	2.409	1.443	2.185
StH	14	4856101	4856400	3.338	3.975	6.513	3.529
StH	14	4948201	4948500	0.502	0.421	0.107	0.416
StH	14	5075101	5075400	0.663	0.633	0.428	0.193
StH	14	5091601	5091900	5.454	5.130	3.542	4.538
StH	14	5131801	5132100	2.291	2.450	1.387	2.155
StH	14	5190901	5191200	2.129	2.266	1.324	1.638
StH	14	5310301	5310600	0.481	0.513	0.239	0.634
StH	14	5319301	5319600	0.558	0.538	0.395	0.193
StH	14	5350801	5351100	1.758	2.573	2.118	1.744
StH	14	5370901	5371200	6.280	5.945	4.846	6.100
StH	14	5376901	5377200	3.330	4.392	2.633	3.849
StH	14	5381701	5382000	0.473	1.009	1.741	0.781
StH	14	5570401	5570700	0.713	0.620	1.886	0.725
StH	14	5585101	5585400	0.424	0.541	0.049	0.365
StH	14	5670601	5670900	2.554	2.381	1.933	2.824
StH	14	5755501	5755800	2.154	2.691	1.884	2.297
StH	14	5775901	5776200	4.651	4.519	3.212	5.039
StH	14	5807101	5807400	2.609	2.617	1.453	2.925
StH	14	5809501	5809800	0.424	0.444	0.292	0.162
StH	14	5864401	5864700	0.694	0.678	0.424	0.715
StH	14	5941201	5941500	0.265	0.414	0.992	0.228
StH	14	6059701	6060000	0.395	0.378	0.847	0.360
StH	14	6067201	6067500	1.274	1.472	0.695	0.913
StH	14	6102901	6103200	1.491	1.380	0.843	1.278
StH	14	6209401	6209700	1.195	1.212	0.519	1.136
StH	14	6499201	6499500	0.668	0.717	1.407	0.396
StH	14	6503701	6504000	0.610	0.210	0.370	0.223
StH	14	6507601	6507900	1.592	1.534	0.769	1.805
StH	14	6592201	6592500	1.011	1.049	1.378	0.938
StH	14	6877801	6878100	0.027	0.165	0.448	0.056
StH	14	6928801	6929100	1.592	1.706	2.303	1.775
StH	14	7102501	7102800	0.443	0.523	2.713	0.370
StH	14	7102801	7103100	0.694	0.485	1.358	0.507
StH	14	7321501	7321800	0.186	0.184	0.555	0.167
StH	14	7379101	7379400	2.549	2.993	1.855	2.754
StH	14	7386601	7386900	8.216	7.659	7.058	9.339
StH	14	7613401	7613700	0.878	0.678	0.473	0.634
StH	14	7619401	7619700	1.165	1.335	0.497	1.339
StH	14	7770601	7770900	1.199	1.543	0.778	1.212
StH	14	7934701	7935000	1.487	1.753	0.838	1.562
StH	14	8080201	8080500	0.052	0.249	0.847	0.101
StH	14	8901301	8901600	0.773	0.770	1.094	0.806
StH	14	9732001	9732300	1.618	1.915	3.896	1.876
StH	14	9896401	9896700	0.297	0.414	1.342	0.167
StH	14	10023301	10023600	0.929	1.032	0.497	0.918

StH	14	10329301	10329600	0.503	4.388	2.335	4.447
StH	14	10361101	10361400	3.055	2.971	2.283	3.159
StH	14	10383001	10383300	7.096	6.086	4.517	5.516
StH	14	10472701	10473000	0.374	0.616	2.020	0.614
StH	14	10816501	10816800	2.363	2.292	1.287	1.937
StH	14	10858201	10858500	1.268	1.006	3.708	1.256
StH	14	10901101	10901400	0.289	0.069	0.662	0.248
StH	14	10924201	10924500	0.905	0.800	1.329	0.765
StH	14	10942501	10942800	0.290	0.283	0.531	0.248
StH	14	10948501	10948800	0.237	0.320	1.070	0.259
StH	14	11076901	11077200	0.455	0.837	1.353	0.634
StH	14	11106301	11106600	0.215	0.163	0.448	0.112
StH	14	11117701	11118000	0.239	0.227	1.083	0.117
StH	14	11235901	11236200	0.240	0.506	1.460	0.457
StH	14	11271601	11271900	0.984	0.959	0.745	1.192
StH	14	11985301	11985600	2.805	0.571	4.372	2.440
StH	14	12030901	12031200	0.953	1.124	0.535	1.111
StH	14	12055201	12055500	1.353	2.359	4.723	1.729
StH	14	12283201	12283500	0.558	0.305	0.691	0.446
StH	14	12412201	12412500	0.560	0.583	0.999	0.274
StH	14	12491701	12492000	0.424	0.229	1.045	0.173
StH	14	12622201	12622500	0.189	0.094	1.687	0.137
StH	14	12622501	12622800	0.186	0.140	0.745	0.329
StH	14	12669601	12669900	0.244	0.680	4.163	0.112
StH	14	12669901	12670200	0.749	1.310	6.957	0.866
StH	14	12676801	12677100	1.807	0.612	2.303	0.391
StH	14	12716701	12717000	0.663	0.697	1.139	0.583
StH	14	12806701	12807000	3.706	3.806	4.887	3.889
StH	14	12843601	12843900	1.300	1.401	1.003	1.856
StH	14	12930301	12930600	1.278	1.854	6.357	1.404
StH	14	12996301	12996600	2.818	2.875	2.160	2.900
StH	14	13023301	13023600	1.116	1.004	0.695	1.156
StH	14	13237501	13237800	0.158	0.234	0.506	0.056
StH	14	13296901	13297200	0.663	0.632	2.514	0.873
StH	14	13401001	13401300	0.962	1.217	0.502	0.948
StH	14	13481101	13481400	0.134	0.159	0.506	0.112
StH	14	13482601	13482900	0.319	1.199	2.969	0.553
StH	14	13511701	13512000	1.620	1.832	3.710	1.334
StH	14	13512001	13512300	2.792	3.220	4.592	2.546
StH	14	13870201	13870500	3.815	3.855	2.530	3.741
StH	14	13926301	13926600	1.223	1.152	0.506	0.913
StH	14	13958401	13958700	0.769	0.981	0.477	0.837
StH	14	14029801	14030100	0.665	0.714	1.469	0.553
StH	14	14035201	14035500	0.879	0.729	2.044	0.958
StH	14	14064901	14065200	0.106	0.184	0.535	0.274
StH	14	14463901	14464200	0.053	0.094	0.555	0.081
StH	14	14838901	14839200	0.905	0.910	0.740	0.441
StH	14	16106401	16106700	0.690	0.839	1.320	0.522
StH	14	16210201	16210500	4.709	6.368	3.682	5.197
StH	14	16220401	16220700	0.718	0.472	1.061	0.441
StH	14	16924501	16924800	0.239	0.442	0.745	0.416
StH	14	16987801	16988100	1.673	1.543	0.959	1.668
StH	14	17156401	17156700	0.400	0.562	0.132	0.578
StH	14	17156701	17157000	0.345	0.423	0.107	0.492
StH	14	17301301	17301600	1.224	1.500	0.531	1.009
StH	14	17936401	17936700	1.829	1.612	7.304	1.440
StH	14	18446101	18446400	0.239	0.182	1.148	0.284
StH	14	18470101	18470400	0.850	0.631	0.210	0.664
StH	14	18721501	18721800	1.492	1.358	1.864	1.430
StH	14	18844501	18844800	0.799	0.865	0.428	0.907
StH	14	19041901	19042200	2.021	1.944	2.414	2.130
StH	14	19549801	19550100	0.081	0.161	0.506	0.213
StH	14	19689901	19690200	0.558	0.513	0.321	0.583
StH	14	19919101	19919400	0.507	0.258	0.711	0.304
StH	14	19924201	19924500	0.316	0.234	0.740	0.411
StH	14	20100301	20100600	0.690	0.611	1.139	0.710
StH	14	20281201	20281500	1.439	1.172	0.729	1.770

StH	14	20343301	20343600	0.371	0.303	0.642	0.324
StH	14	20649001	20649300	0.873	0.558	2.135	0.740
StH	14	21649801	21650100	4.893	4.761	4.224	5.085
StH	14	21710701	21711000	3.776	3.810	2.608	3.077
StH	14	21759001	21759300	1.676	2.244	0.909	2.119
StH	14	21907801	21908100	1.142	0.938	0.515	1.222
StH	14	22103401	22103700	0.371	0.493	0.132	0.391
StH	14	22327201	22327500	0.400	0.236	0.609	0.162
StH	14	22748401	22748700	1.676	1.772	3.441	1.674
StH	14	22748701	22749000	0.744	0.697	1.672	0.497
StH	14	22749601	22749900	0.239	0.255	0.448	0.137
StH	14	23117101	23117400	0.452	0.468	0.370	0.193
StH	14	23212801	23213100	0.531	0.470	0.292	0.690
StH	14	23357101	23357400	0.373	0.420	0.267	0.497
StH	14	23527201	23527500	0.398	0.466	0.189	0.416
StH	14	23565901	23566200	0.398	0.444	0.181	0.416
StH	14	24213901	24214200	1.697	1.663	0.794	1.627
StH	14	24351301	24351600	1.783	2.197	1.168	1.638
StH	14	24588001	24588300	1.570	1.431	0.847	1.451
StH	14	25330801	25331100	0.263	0.682	2.238	0.299
StH	14	25497301	25497600	0.106	0.206	0.609	0.101
StH	14	26661301	26661600	0.931	1.032	0.691	1.247
StH	14	27618901	27619200	0.318	0.277	0.584	0.137
StH	14	28538401	28538700	1.276	0.863	0.667	0.832
StH	14	29137801	29138100	2.088	1.517	3.685	1.150
StH	14	29472001	29472300	0.160	0.236	0.448	0.218
StH	14	30108601	30108900	0.371	0.817	0.140	0.416
StH	14	30631801	30632100	0.718	0.515	0.399	0.279
StH	14	31964101	31964400	0.373	0.352	0.506	0.360
StH	14	32198701	32199000	0.373	0.420	0.156	0.304
StH	14	32217301	32217600	0.450	0.399	0.107	0.421
StH	14	32384701	32385000	0.426	0.185	0.555	0.279
StH	14	32811901	32812200	3.296	2.811	2.060	2.971
StH	14	33042601	33042900	1.118	0.800	1.679	1.197
StH	14	33093901	33094200	1.863	1.489	0.852	2.058
StH	14	33181501	33181800	0.690	0.981	0.823	0.497
StH	14	33624601	33624900	0.505	0.517	0.136	0.421
StH	14	33625501	33625800	3.378	3.068	1.933	2.946
StH	14	33706801	33707100	0.055	0.118	0.477	0.142
StH	14	33747001	33747300	0.186	0.219	1.242	0.614
StH	14	33831301	33831600	0.213	0.607	0.346	0.162
StH	14	33867601	33867900	4.417	4.534	6.543	4.543
StH	14	33937501	33937800	1.039	0.938	0.461	0.857
StH	14	33941701	33942000	1.302	0.983	0.613	0.988
StH	14	34138801	34139100	0.000	0.045	0.575	0.132
StH	14	34194901	34195200	0.984	0.773	0.502	0.771
StH	14	34404001	34404300	1.597	1.658	1.110	1.328
StH	14	34537501	34537800	4.711	4.299	3.081	4.000
StH	14	34553401	34553700	6.679	7.005	5.192	6.723
StH	14	34570801	34571100	0.215	0.118	0.473	0.086
StH	14	34595101	34595400	2.392	2.671	1.482	2.297
StH	14	35253901	35254200	0.505	0.633	0.267	0.522
StH	14	35345401	35345700	1.353	1.427	0.727	1.445
StH	14	35345701	35346000	1.328	1.549	0.818	1.465
StH	14	35697001	35697300	0.536	1.216	2.769	0.436
StH	14	35704801	35705100	0.371	0.444	0.711	0.416
StH	14	35763001	35763300	0.239	0.283	0.506	0.198
StH	14	35840401	35840700	0.160	0.564	0.374	0.167
StH	14	37088101	37088400	0.106	0.187	0.593	0.051
StH	14	37088401	37088700	0.132	0.187	0.497	0.000
StH	14	37385701	37386000	2.420	2.006	1.287	2.748
StH	14	37482601	37482900	3.381	2.506	1.632	2.211
StH	14	37541701	37542000	1.402	2.420	3.611	1.679
StH	14	37594801	37595100	3.246	2.542	3.752	2.992
StH	14	37988401	37988700	1.111	0.953	1.761	0.700
StH	14	38149501	38149800	1.166	1.476	0.745	1.303
StH	14	38435401	38435700	0.986	1.172	0.604	1.247

StH	14	38452501	38452800	0.398	0.584	0.239	0.304
StH	14	38724901	38725200	0.663	0.491	0.136	0.452
StH	14	38884801	38885100	0.481	0.896	2.728	1.166
StH	14	39144601	39144900	2.341	2.128	1.373	2.357
StH	14	39454501	39454800	1.331	1.238	1.003	0.502
StH	14	39816901	39817200	0.155	0.000	0.555	0.000
StH	14	40052701	40053000	2.184	1.939	2.642	1.181
StH	14	40261201	40261500	1.303	1.030	0.555	1.121
StH	14	40344001	40344300	3.672	3.461	2.829	3.681
StH	14	40432801	40433100	0.931	1.729	1.061	0.720
StH	14	40457101	40457400	0.878	0.818	0.584	0.801
StH	14	40457401	40457700	0.797	0.908	0.366	0.902
StH	14	40503901	40504200	0.321	0.420	0.716	0.198
StH	14	41078701	41079000	0.213	0.420	0.617	0.441
StH	14	41129101	41129400	3.851	3.883	2.920	4.152
StH	14	41234701	41235000	0.376	0.163	0.789	0.056
StH	14	42073501	42073800	1.089	1.569	0.700	0.385
StH	14	42378601	42378900	0.211	0.258	0.691	0.162
StH	14	42612901	42613200	0.027	0.163	0.535	0.867
StH	14	42701101	42701400	1.595	1.172	2.015	1.719
StH	14	42927301	42927600	1.829	1.307	0.847	1.546
StH	14	42979501	42979800	0.953	1.079	0.448	0.933
StH	14	42997501	42997800	0.902	0.697	1.527	0.731
StH	14	43010101	43010400	0.692	0.586	0.267	0.527
StH	14	43348201	43348500	0.292	0.326	0.132	0.466
StH	14	43433101	43433400	0.132	0.094	0.551	0.081
StH	14	43458001	43458300	1.837	2.124	1.431	1.739
StH	14	43559701	43560000	0.610	1.405	0.267	0.710
StH	14	43596601	43596900	0.240	0.279	0.502	0.137
StH	14	44025901	44026200	3.489	3.984	1.712	4.589
StH	14	44026201	44026500	3.064	2.774	1.096	4.250
StH	14	44056501	44056800	0.398	0.843	0.185	0.472
StH	14	44860801	44861100	1.195	1.075	0.617	0.893
StH	14	45142201	45142500	0.453	0.538	0.210	0.477
StH	14	45184801	45185100	0.715	0.611	1.304	0.436
StH	14	45269401	45269700	2.416	2.783	1.736	2.216
StH	14	45273601	45273900	0.876	0.841	1.177	0.776
StH	14	45458401	45458700	1.841	2.079	1.304	2.048
StH	14	45561301	45561600	4.814	3.057	4.242	5.735
StH	14	45682501	45682800	0.908	1.111	2.127	1.201
StH	14	45756901	45757200	0.695	0.866	3.274	0.690
StH	14	45868201	45868500	0.452	0.258	0.029	0.436
StH	14	45868801	45869100	0.160	0.213	0.638	0.142
StH	14	46501501	46501800	0.240	0.678	0.526	0.801
StH	14	46941901	46942200	0.532	0.281	0.374	0.664
StH	14	47258701	47259000	2.957	3.212	1.765	2.814
StH	14	47280901	47281200	1.623	2.180	0.959	1.668
StH	14	47309101	47309400	0.505	0.326	0.185	0.527
StH	14	47451601	47451900	3.854	3.714	2.299	3.525
StH	14	47498101	47498400	0.774	0.654	1.115	0.690
StH	14	47508601	47508900	1.412	0.633	0.959	0.695
StH	14	47636101	47636400	4.956	3.564	2.423	3.859
StH	14	47636401	47636700	5.602	4.302	2.477	3.986
StH	14	47775301	47775600	0.531	0.629	0.189	0.360
StH	14	47795701	47796000	0.453	0.328	0.638	0.218
StH	14	48014401	48014700	5.715	5.666	4.258	5.561
StH	14	48039001	48039300	3.964	4.143	2.762	3.686
StH	14	48071101	48071400	0.374	1.223	3.216	0.745
StH	14	48397501	48397800	4.596	4.696	3.509	4.416
StH	14	48474901	48475200	0.692	0.515	0.974	0.553
StH	14	48697501	48697800	0.318	0.187	0.477	0.360
StH	14	48749101	48749400	0.137	0.744	2.748	0.538
StH	14	48749401	48749700	0.349	0.483	1.570	0.405
StH	14	48755401	48755700	0.428	0.302	0.740	0.243
StH	14	48761401	48761700	0.105	0.068	0.629	0.025
StH	14	48768601	48768900	4.381	4.356	3.348	5.299
StH	14	48837301	48837600	1.008	0.628	0.292	0.664

StH	14	48879301	48879600	5.294	5.425	2.544	6.079
StH	14	48954601	48954900	0.981	0.942	0.136	1.886
StH	14	48954901	48955200	0.982	0.399	1.420	0.538
StH	14	48959101	48959400	2.796	4.038	1.137	3.630
StH	14	49013101	49013400	2.500	2.008	3.003	2.165
StH	14	49053301	49053600	2.050	1.849	1.456	2.043
StH	14	49121401	49121700	2.341	2.159	0.898	1.851
StH	14	49521601	49521900	3.617	3.343	2.566	3.569
StH	14	49521901	49522200	4.965	4.113	3.052	4.198
StH	14	49597201	49597500	1.403	1.892	0.872	1.389
StH	14	49954501	49954800	0.000	0.163	0.658	0.223
StH	14	49997101	49997400	0.132	0.274	0.843	0.167
StH	14	50052601	50052900	0.557	0.457	1.643	0.492
StH	14	50424001	50424300	1.360	1.540	0.613	1.287
StH	14	51014401	51014700	4.577	5.186	4.109	4.680
StH	14	51376201	51376500	0.292	0.212	0.448	0.223
StH	14	51539401	51539700	2.192	2.012	0.544	2.698
StH	14	51636001	51636300	1.250	1.182	2.000	0.918
StH	14	51725401	51725700	0.850	0.605	1.197	0.882
StH	14	51940801	51941100	5.854	6.157	8.005	4.457
StH	14	52364701	52365000	0.108	0.139	0.510	0.106
StH	14	52396801	52397100	0.371	0.303	0.613	0.446
StH	14	52438201	52438500	4.202	4.257	3.088	4.213
StH	14	52570801	52571100	1.039	0.890	0.408	0.902
StH	14	52715401	52715700	0.186	0.212	0.588	0.086
StH	14	53024701	53025000	0.398	0.423	0.160	0.385
StH	14	53188501	53188800	7.808	8.320	6.314	7.783
StH	14	53269501	53269800	0.424	0.749	0.210	0.360
StH	14	53481601	53481900	0.558	0.539	0.954	0.558
StH	14	53493001	53493300	1.307	1.261	4.641	1.117
StH	14	53661601	53661900	0.000	0.251	0.938	0.000
StH	14	53950201	53950500	0.265	0.404	1.282	0.477
StH	14	54424801	54425100	2.791	3.014	2.185	2.702
StH	14	54642901	54643200	3.004	3.177	1.739	3.109
StH	14	54653101	54653400	0.079	0.364	1.758	0.061
StH	14	54673201	54673500	0.134	0.140	0.448	0.101
StH	14	54678301	54678600	0.902	0.986	2.931	0.893
StH	14	54700801	54701100	6.383	5.718	3.790	6.955
StH	14	55046701	55047000	0.505	0.936	1.572	1.014
StH	14	55071301	55071600	2.552	2.105	1.558	2.216
StH	14	55314001	55314300	0.213	0.277	0.769	0.284
StH	14	55429801	55430100	0.211	0.230	0.588	0.304
StH	14	55464301	55464600	1.966	2.105	4.582	2.393
StH	14	55491601	55491900	0.213	0.165	0.613	0.329
StH	14	55506301	55506600	0.131	0.661	1.949	0.452
StH	14	55506601	55506900	0.237	0.324	0.876	0.274
StH	14	55524601	55524900	1.065	0.963	0.312	0.659
StH	14	55597201	55597500	8.495	8.969	6.396	8.319
StH	14	55615801	55616100	1.513	1.377	2.060	0.898
StH	14	55651801	55652100	1.010	0.921	1.683	0.553
StH	14	55686601	55686900	0.371	0.744	3.414	0.345
StH	14	55714201	55714500	0.186	0.212	0.486	0.137
StH	14	55750201	55750500	0.424	0.607	1.152	0.669
StH	14	55809301	55809600	0.161	0.094	0.419	0.167
StH	14	55843801	55844100	2.315	2.184	1.152	2.703
StH	14	55845301	55845600	0.316	1.109	4.227	0.522
StH	14	55864801	55865100	1.333	1.575	2.961	1.004
StH	14	56007901	56008200	0.428	0.856	1.893	0.933
StH	14	56135701	56136000	0.771	0.023	0.588	0.751
StH	14	56412301	56412600	0.690	1.806	4.955	1.304
StH	14	56412601	56412900	5.161	3.847	12.876	5.650
StH	14	56419501	56419800	0.824	1.199	1.781	0.918
StH	14	56507701	56508000	1.465	1.652	2.649	1.952
StH	14	57262501	57262800	0.799	0.768	0.350	0.634
StH	14	57364801	57365100	0.957	0.953	0.366	0.988
StH	14	57366601	57366900	0.082	0.142	0.448	0.167
StH	14	57425701	57426000	3.083	2.736	2.365	2.576

StH	14	57585001	57585300	0.055	0.116	0.461	0.056
StH	14	58550101	58550400	3.564	3.738	4.446	3.625
StH	14	58745401	58745700	2.018	1.616	1.041	1.566
StH	14	58806901	58807200	0.718	0.978	0.453	0.968
StH	14	58895701	58896000	0.240	0.283	0.506	0.193
StH	14	58995001	58995300	3.986	4.242	1.896	4.188
StH	14	58997101	58997400	1.403	1.519	0.674	1.627
StH	14	59000401	59000700	0.345	0.448	0.054	0.335
StH	14	59065501	59065800	0.186	0.444	0.584	0.304
StH	14	59132401	59132700	0.557	1.038	3.921	0.522
StH	14	59146801	59147100	0.081	0.137	0.651	0.031
StH	14	59235601	59235900	6.559	4.035	11.425	6.322
StH	14	59252101	59252400	2.150	1.850	3.491	2.225
StH	14	59319001	59319300	0.932	1.071	2.064	0.873
StH	14	59321401	59321700	0.724	1.216	1.884	0.679
StH	14	59388001	59388300	0.186	0.390	0.930	0.324
StH	14	59464801	59465100	0.610	1.233	3.798	0.685
StH	14	59465101	59465400	0.371	0.847	2.699	0.883
StH	14	59492101	59492400	0.586	0.933	0.346	0.887
StH	14	59493901	59494200	0.160	0.272	1.307	0.198
StH	14	59494201	59494500	0.026	0.090	0.843	0.056
StH	14	59499301	59499600	0.242	0.296	1.181	0.167
StH	14	59574301	59574600	1.094	1.268	2.466	1.176
StH	14	59576701	59577000	2.238	2.594	1.460	2.276
StH	14	59655001	59655300	2.952	3.083	2.163	2.865
StH	14	59679901	59680200	0.345	0.607	0.481	0.329
StH	14	59878801	59879100	2.210	2.334	0.888	1.876
StH	14	60055501	60055800	1.123	0.618	2.296	1.070
StH	14	60090001	60090300	1.018	1.122	2.169	0.947
StH	14	60183601	60183900	0.747	0.886	0.087	1.521
StH	14	60198901	60199200	1.462	1.440	2.414	1.582
StH	14	60224401	60224700	0.987	1.008	0.473	1.593
StH	14	60375301	60375600	1.187	0.749	2.187	0.963
StH	14	60566101	60566400	2.450	1.899	1.378	1.881
StH	14	60566401	60566700	4.170	3.946	3.170	4.883
StH	14	60603601	60603900	4.544	4.407	5.741	4.020
StH	14	60660001	60660300	1.041	1.008	0.667	1.024
StH	14	60717901	60718200	2.386	2.605	1.329	2.515
StH	14	60718201	60718500	2.041	1.961	0.760	2.034
StH	14	60741901	60742200	0.082	0.182	0.658	0.056
StH	14	60742201	60742500	0.055	0.092	0.461	0.000
StH	14	60751201	60751500	0.186	0.204	1.012	0.335
StH	14	60850201	60850500	0.132	0.112	0.613	0.056
StH	14	61059301	61059600	1.896	2.409	5.844	2.181
StH	14	61065001	61065300	0.265	0.748	3.414	0.329
StH	14	61091701	61092000	0.242	0.274	1.161	0.157
StH	14	61115401	61115700	0.292	0.352	0.662	0.441
StH	14	61116001	61116300	1.194	0.721	1.793	0.994
StH	14	61142101	61142400	0.720	0.536	1.431	0.624
StH	14	61176901	61177200	7.027	6.300	4.005	7.251
StH	14	61308601	61308900	5.136	4.064	4.521	3.681
StH	14	62029801	62030100	0.268	0.165	0.477	0.274
StH	14	62151601	62151900	0.364	0.650	2.342	0.299
StH	14	62328601	62328900	0.103	0.000	0.658	0.000
StH	14	62328901	62329200	0.363	0.023	1.765	0.056
StH	14	62330101	62330400	0.000	0.000	0.782	0.000
StH	14	62595301	62595600	5.873	6.625	4.733	6.018
StH	14	62661001	62661300	0.134	0.491	0.239	0.086
StH	15	196201	196500	0.266	0.187	0.502	0.193
StH	15	488701	489000	0.507	0.678	0.346	0.472
StH	15	594001	594300	0.239	0.328	0.580	0.193
StH	15	990601	990900	0.290	0.421	0.667	0.411
StH	15	1008301	1008600	3.636	3.693	1.761	4.289
StH	15	1023901	1024200	0.082	0.137	0.580	0.000
StH	15	1362301	1362600	0.027	0.189	0.560	0.385
StH	15	2638801	2639100	0.294	0.702	0.054	0.309
StH	15	4259101	4259400	1.833	1.290	0.823	1.546

StH	15	4923901	4924200	0.639	0.702	0.374	0.751
StH	15	5685001	5685300	2.842	2.815	1.560	2.605
StH	15	5776201	5776500	1.362	1.446	2.138	0.765
StH	15	5813101	5813400	0.561	0.513	0.136	0.441
StH	15	6661501	6661800	0.242	0.348	0.638	0.218
StH	15	6945901	6946200	2.504	2.362	1.465	1.851
StH	15	7053901	7054200	5.990	4.952	3.970	5.095
StH	15	7852501	7852800	0.186	0.144	0.506	0.167
StH	15	8981101	8981400	0.292	0.373	0.477	0.664
StH	15	9193501	9193800	3.381	1.424	2.414	1.673
StH	15	10641001	10641300	0.558	0.539	0.181	0.466
StH	15	11813701	11814000	6.854	7.977	5.275	7.549
StH	15	12811801	12812100	0.160	0.257	0.510	0.254
StH	15	13164601	13164900	0.160	0.185	0.502	0.112
StH	15	13267801	13268100	0.584	0.584	0.317	0.446
StH	15	13546201	13546500	0.400	0.373	0.132	0.745
StH	15	13748401	13748700	0.266	0.562	0.374	0.522
StH	15	13997401	13997700	0.450	0.421	0.239	0.553
StH	15	14079901	14080200	0.584	0.421	0.234	0.553
StH	15	14445601	14445900	0.081	0.068	0.604	0.112
StH	15	14569501	14569800	0.795	0.745	0.296	0.659
StH	15	15509701	15510000	2.954	3.109	4.096	3.159
StH	15	15675601	15675900	0.558	0.541	0.107	0.472
StH	15	15747301	15747600	0.132	0.255	0.560	0.304
StH	15	16976401	16976700	0.132	0.399	0.724	0.391
StH	15	17145301	17145600	1.963	2.613	0.682	1.734
StH	15	17685001	17685300	0.265	0.511	0.802	0.218
StH	15	17693101	17693400	0.187	0.165	0.486	0.198
StH	15	18328201	18328500	0.265	0.609	0.399	0.167
StH	15	18979201	18979500	0.053	0.139	0.662	0.213
StH	15	18979501	18979800	0.182	0.208	0.802	0.213
StH	15	19329001	19329300	0.106	0.187	0.263	0.497
StH	15	19632901	19633200	0.211	0.210	0.860	0.335
StH	15	19754401	19754700	0.371	0.701	0.185	0.385
StH	15	20099701	20100000	0.081	0.139	0.604	0.167
StH	15	20227501	20227800	1.224	0.399	0.740	0.396
StH	15	20487301	20487600	0.319	0.140	0.633	0.147
StH	15	20569501	20569800	0.477	0.538	1.028	0.452
StH	15	20889901	20890200	0.265	0.330	0.671	0.167
StH	15	21062401	21062700	1.228	1.474	0.707	1.491
StH	15	21496501	21496800	0.239	0.139	0.707	0.147
StH	15	21605701	21606000	2.550	2.697	1.772	3.017
StH	15	21695401	21695700	3.773	3.222	4.659	2.961
StH	15	21799201	21799500	0.268	0.258	0.687	0.360
StH	15	22206001	22206300	0.210	0.272	1.955	0.198
StH	15	22707301	22707600	1.145	0.730	1.572	0.603
StH	15	22903801	22904100	0.613	0.491	0.346	0.553
StH	15	23091001	23091300	0.371	0.609	0.769	0.553
StH	15	23150101	23150400	0.507	0.491	0.239	0.497
StH	15	23247301	23247600	0.026	0.952	4.039	0.031
StH	15	23264701	23265000	1.065	1.450	1.567	1.247
StH	15	23317801	23318100	0.187	0.538	0.346	0.248
StH	15	23569201	23569500	0.560	0.326	0.856	0.416
StH	15	23616001	23616300	0.345	0.399	0.156	0.497
StH	15	23835601	23835900	0.479	0.562	0.107	0.248
StH	15	23985901	23986200	0.611	0.345	1.173	0.527
StH	15	24050701	24051000	0.339	0.508	2.508	0.962
StH	15	24190501	24190800	1.008	0.723	0.243	0.608
StH	15	24615601	24615900	0.239	0.283	0.502	0.274
StH	15	24855601	24855900	0.768	0.648	1.456	0.644
StH	15	24951901	24952200	0.718	0.678	0.987	0.700
StH	15	25188601	25188900	0.876	0.957	1.201	0.695
StH	15	25467001	25467300	1.518	1.541	0.745	1.496
StH	15	25497301	25497600	0.369	0.442	2.095	0.699
StH	15	25497601	25497900	0.187	0.253	1.108	0.421
StH	15	25893901	25894200	0.105	0.210	0.448	0.086
StH	15	25967701	25968000	0.106	0.144	0.473	0.112

StH	15	26127601	26127900	0.266	0.187	0.395	0.578
StH	15	26223301	26223600	0.558	0.421	0.132	0.193
StH	15	26248201	26248500	0.187	0.210	0.477	0.162
StH	15	26742901	26743200	0.805	0.515	1.638	0.396
StH	15	26847601	26847900	0.211	0.137	0.794	0.223
StH	15	27628801	27629100	1.965	2.238	1.184	1.987
StH	15	27641401	27641700	1.120	2.723	3.603	1.334
StH	15	27755701	27756000	0.319	0.234	0.740	0.391
StH	15	28154101	28154400	0.374	0.446	0.074	0.385
StH	15	28184701	28185000	0.426	0.279	0.531	0.360
StH	15	28223401	28223700	0.505	0.094	0.234	0.527
StH	15	28266601	28266900	0.426	0.397	0.181	0.466
StH	15	28569901	28570200	0.318	0.024	0.580	0.304
StH	15	28988101	28988400	1.539	1.757	0.938	1.410
StH	15	29216701	29217000	0.531	0.257	0.160	0.248
StH	15	29235301	29235600	0.398	1.217	0.695	1.318
StH	15	29434501	29434800	1.299	1.620	0.515	1.186
StH	15	29473501	29473800	0.452	0.538	0.205	0.578
StH	15	29532301	29532600	0.687	0.886	1.842	0.466
StH	15	29556301	29556600	0.878	0.794	0.308	0.786
StH	15	29815801	29816100	0.292	0.352	0.107	0.466
StH	15	29920501	29920800	1.460	1.596	0.745	1.643
StH	15	30031201	30031500	0.292	0.583	0.827	0.416
StH	15	30308701	30309000	0.345	0.350	0.239	0.527
StH	15	30402301	30402600	0.452	0.444	0.239	0.446
StH	15	30828901	30829200	1.570	1.661	0.836	1.161
StH	15	30918901	30919200	0.324	0.376	1.103	0.416
StH	15	31037401	31037700	2.204	2.495	3.257	2.373
StH	15	31037701	31038000	1.649	1.793	2.428	1.734
StH	15	31062601	31062900	0.828	0.772	1.255	0.547
StH	15	31103101	31103400	0.240	0.493	0.054	0.527
StH	15	31301701	31302000	1.436	1.315	2.673	1.282
StH	15	31350901	31351200	0.397	0.470	0.720	0.416
StH	15	31372801	31373100	0.213	0.234	0.506	0.248
StH	15	31959301	31959600	1.781	1.622	0.836	1.541
StH	15	32318401	32318700	0.213	0.303	0.510	0.167
StH	15	32384401	32384700	0.452	0.393	0.798	0.492
StH	15	33161101	33161400	0.532	0.583	0.214	0.918
StH	15	33552301	33552600	0.318	0.258	0.506	0.218
StH	15	33564001	33564300	1.970	1.850	0.881	1.683
StH	15	34630501	34630800	1.091	1.049	1.411	0.907
StH	15	34676701	34677000	0.665	0.584	0.296	0.943
StH	15	34863001	34863300	3.622	4.471	2.998	3.747
StH	15	35238601	35238900	0.266	0.491	0.111	0.274
StH	15	36170701	36171000	0.213	0.189	0.555	0.385
StH	15	36589501	36589800	2.708	3.222	4.340	3.169
StH	15	36654301	36654600	1.281	1.124	2.348	1.121
StH	15	36823501	36823800	0.134	0.676	0.390	0.081
StH	15	36887701	36888000	0.424	0.187	0.588	0.329
StH	15	36903001	36903300	0.371	0.234	0.745	0.335
StH	15	37061401	37061700	0.398	0.238	0.831	0.187
StH	15	37403401	37403700	0.850	0.959	0.745	1.131
StH	15	38077201	38077500	0.053	0.135	0.531	0.167
StH	15	38415901	38416200	0.694	0.515	0.346	0.776
StH	15	38679901	38680200	0.639	0.704	0.370	0.776
StH	15	39445801	39446100	0.824	0.699	0.366	0.857
StH	15	39532801	39533100	0.319	0.493	0.107	0.497
StH	15	39793501	39793800	0.132	0.118	0.473	0.056
StH	15	40144501	40144800	1.947	2.051	1.003	2.022
StH	15	40345501	40345800	0.747	0.940	0.185	0.654
StH	15	40850401	40850700	1.037	0.794	0.477	0.933
StH	15	40950001	40950300	0.639	0.678	0.769	0.578
StH	15	42327601	42327900	1.199	1.189	1.748	1.258
StH	15	42490801	42491100	0.742	0.607	0.317	0.527
StH	15	42610201	42610500	3.481	3.654	0.817	2.800
StH	15	42610501	42610800	2.494	3.495	0.981	2.479
StH	15	42616501	42616800	0.160	0.446	0.769	0.137

StH	15	42671701	42672000	0.452	0.446	0.789	0.355
StH	15	42672001	42672300	0.507	0.489	0.872	0.492
StH	15	43226701	43227000	1.674	1.221	0.765	1.222
StH	15	43354801	43355100	2.025	1.772	1.230	2.048
StH	15	43489501	43489800	0.187	0.350	0.502	0.142
StH	15	44619001	44619300	1.041	0.860	1.514	0.893
StH	15	44720401	44720700	0.373	0.468	0.156	0.279
StH	15	44920501	44920800	0.213	0.260	0.477	0.137
StH	15	45447301	45447600	0.294	0.534	0.025	0.279
StH	15	45708301	45708600	0.398	0.401	0.160	0.497
StH	15	45816601	45816900	5.403	5.705	3.983	5.461
StH	15	46525201	46525500	0.187	0.185	0.486	0.198
StH	15	46757101	46757400	0.718	0.397	0.555	0.745
StH	15	47678701	47679000	0.824	1.236	1.465	1.166
StH	15	49223401	49223700	1.650	1.715	0.959	1.840
StH	15	49236901	49237200	0.132	0.375	0.954	0.106
StH	15	49237201	49237500	0.053	0.558	1.253	0.137
StH	15	49320301	49320600	1.623	1.933	1.110	1.800
StH	15	49882201	49882500	0.639	0.558	1.037	0.669
StH	15	49948201	49948500	0.266	0.281	0.560	0.385
StH	15	50145001	50145300	2.126	2.424	1.081	2.966
StH	15	50145301	50145600	3.849	4.488	2.934	4.426
StH	15	50402401	50402700	0.477	0.515	0.818	0.472
StH	15	50449801	50450100	5.475	6.055	4.383	5.825
StH	15	50874001	50874300	0.426	0.446	0.239	0.502
StH	15	51009001	51009300	0.690	0.822	0.296	0.639
StH	15	51252601	51252900	0.824	0.824	0.247	0.781
StH	15	51374101	51374400	6.220	6.499	5.372	6.617
StH	15	52504801	52505100	1.642	1.392	2.811	1.278
StH	15	52533001	52533300	0.560	0.448	1.344	0.391
StH	15	52725001	52725300	0.507	0.515	0.346	0.882
StH	15	52795801	52796100	1.353	1.417	3.696	1.192
StH	15	52853101	52853400	2.076	2.309	1.522	2.398
StH	15	52869301	52869600	0.799	1.195	0.473	0.781
StH	15	52925701	52926000	0.240	0.731	2.875	0.320
StH	15	52927501	52927800	1.142	1.519	0.564	0.821
StH	15	52986901	52987200	2.368	1.849	1.358	2.226
StH	15	53795401	53795700	1.010	1.169	0.724	1.044
StH	15	54230101	54230400	0.345	0.163	0.477	0.274
StH	15	54363001	54363300	0.213	0.328	0.662	0.309
StH	15	54664201	54664500	0.216	0.210	0.609	0.137
StH	15	54747301	54747600	3.380	3.667	2.158	4.228
StH	15	54755401	54755700	0.479	0.446	0.132	0.391
StH	15	54851101	54851400	0.055	0.303	0.531	0.299
StH	15	56230501	56230800	1.403	1.740	0.078	1.668
StH	15	56494801	56495100	0.242	0.187	0.671	0.218
StH	15	57357001	57357300	0.848	0.953	0.428	0.857
StH	15	57936301	57936600	0.294	0.212	0.082	0.608
StH	15	58120501	58120800	0.187	0.210	0.448	0.056
StH	15	58832101	58832400	0.239	0.258	0.535	0.309
StH	15	59933101	59933400	1.142	2.594	3.471	2.257
StH	15	59933401	59933700	0.344	1.414	2.209	1.151
StH	15	60112501	60112800	0.452	0.493	0.239	0.466
StH	15	60205501	60205800	0.929	0.820	0.399	0.578
StH	15	61130701	61131000	0.160	0.236	0.502	0.243
StH	15	61139101	61139400	1.199	1.006	0.588	1.222
StH	15	62012701	62013000	0.692	0.493	0.823	0.583
StH	15	62524801	62525100	0.373	0.746	2.543	0.441
StH	15	62933401	62933700	0.665	0.564	0.234	0.659
StH	15	62933701	62934000	0.586	0.536	0.214	0.634
StH	15	62973901	62974200	1.563	1.592	0.794	1.334
StH	15	63119401	63119700	0.266	0.350	0.535	0.385
StH	15	63569701	63570000	0.477	0.163	0.025	0.162
StH	15	64285501	64285800	0.560	0.558	1.402	0.380
StH	15	64655701	64656000	1.491	1.098	0.831	1.217
StH	15	64723201	64723500	1.487	1.985	1.016	1.861
StH	15	64790701	64791000	0.318	0.140	0.502	0.248

StH	15	64893301	64893600	1.829	1.889	0.185	1.790
StH	15	64896001	64896300	0.218	0.257	1.453	0.375
StH	15	64900201	64900500	0.574	0.581	2.261	0.983
StH	15	64949701	64950000	1.432	1.306	0.600	1.161
StH	15	65072701	65073000	0.903	1.075	0.798	0.472
StH	15	65154001	65154300	3.210	3.521	1.748	3.027
StH	15	65455201	65455500	0.108	0.189	0.809	0.259
StH	15	65617201	65617500	0.105	0.189	0.510	0.076
StH	15	65617501	65617800	0.134	0.144	0.477	0.000
StH	15	65927101	65927400	0.186	0.303	0.711	0.350
StH	15	66076501	66076800	0.529	0.238	1.201	0.345
StH	15	66751801	66752100	0.395	0.425	1.086	0.391
StH	15	67726501	67726800	1.879	0.139	0.836	0.117
StH	15	68510401	68510700	0.160	0.187	0.502	0.279
StH	15	68658601	68658900	0.290	0.328	0.531	0.137
StH	15	68951401	68951700	0.505	0.493	0.317	0.578
StH	15	68961001	68961300	0.507	0.212	0.374	0.137
StH	15	69019501	69019800	0.426	0.446	0.107	0.385
StH	15	69502501	69502800	1.674	2.427	2.205	2.515
StH	15	69773101	69773400	0.108	0.189	0.876	0.137
StH	15	69933301	69933600	0.211	0.230	0.555	0.167
StH	15	70508701	70509000	0.134	0.328	0.510	0.223
StH	15	70810201	70810500	0.213	0.350	0.555	0.137
StH	15	71208601	71208900	0.319	0.234	0.506	0.117
StH	15	71330701	71331000	0.505	0.399	0.243	0.112
StH	15	71764201	71764500	0.587	0.631	0.292	0.588
StH	15	71825101	71825400	3.989	4.345	2.863	3.591
StH	15	72024601	72024900	0.955	1.032	0.506	1.004
StH	15	72247501	72247800	0.053	0.096	0.477	0.056
StH	15	72317101	72317400	0.476	0.536	0.872	0.355
StH	15	72421801	72422100	1.360	1.073	0.370	0.897
StH	15	72426301	72426600	0.319	0.328	0.448	0.193
StH	15	72460501	72460800	2.368	1.411	3.627	2.171
StH	15	72568801	72569100	0.344	0.097	0.691	0.355
StH	15	72863401	72863700	1.487	1.407	0.624	1.287
StH	15	72923401	72923700	0.507	0.350	0.239	0.776
StH	15	73204201	73204500	0.424	0.611	0.136	0.416
StH	15	73311601	73311900	0.584	0.468	0.876	0.391
StH	15	73562101	73562400	0.455	0.376	0.000	0.365
StH	15	73596601	73596900	0.452	0.538	0.263	0.472
StH	15	73702801	73703100	0.132	0.375	0.477	0.360
StH	15	74043601	74043900	0.266	0.373	0.560	0.243
StH	15	74202601	74202900	0.106	0.330	0.477	0.248
StH	15	74499901	74500200	0.186	0.210	0.531	0.117
StH	15	74652001	74652300	2.633	2.671	1.913	2.754
StH	15	74903401	74903700	0.878	1.174	0.317	0.826
StH	15	75124801	75125100	0.187	0.236	0.564	0.137
StH	15	75228901	75229200	3.613	3.766	2.621	3.984
StH	15	75791101	75791400	3.059	3.358	1.879	2.673
StH	15	75791401	75791700	7.422	7.930	5.067	8.685
StH	15	75839701	75840000	0.926	1.274	3.761	1.319
StH	15	75869401	75869700	1.757	1.171	0.662	1.424
StH	15	75956401	75956700	2.787	3.225	2.356	3.235
StH	15	75970201	75970500	3.344	3.394	2.299	3.326
StH	15	76014301	76014600	0.160	0.633	0.453	0.664
StH	15	76710001	76710300	0.423	0.375	0.132	0.695
StH	15	76986001	76986300	0.534	1.008	0.769	1.055
StH	15	77231401	77231700	0.321	0.373	0.896	0.360
StH	15	77371201	77371500	1.649	0.517	0.774	1.125
StH	15	77384401	77384700	0.079	0.292	1.821	0.126
StH	15	77449201	77449500	0.477	0.210	0.720	0.218
StH	15	77508001	77508300	0.507	0.421	0.823	0.385
StH	15	77635801	77636100	2.578	3.268	1.723	2.581
StH	15	77636701	77637000	0.931	1.210	3.526	0.898
StH	15	77643901	77644200	6.231	6.717	3.495	6.094
StH	15	77696701	77697000	3.212	2.885	7.033	3.443
StH	15	77697001	77697300	4.273	3.355	7.573	3.399

StH	15	77719801	77720100	0.108	0.167	0.473	0.081
StH	15	77746501	77746800	0.155	0.225	1.271	0.254
StH	15	77805901	77806200	0.374	0.633	2.458	0.461
StH	15	77894701	77895000	0.929	1.101	0.428	0.872
StH	15	77950201	77950500	1.350	2.142	5.997	2.074
StH	15	78073501	78073800	1.705	2.105	1.222	1.627
StH	15	78414901	78415200	1.515	1.826	1.275	1.607
StH	15	78482101	78482400	3.109	3.083	2.588	3.067
StH	15	78614101	78614400	5.162	3.903	3.127	3.762
StH	15	78883801	78884100	0.106	0.234	0.419	0.193
StH	15	79130701	79131000	1.513	1.281	0.794	1.440
StH	15	79233301	79233600	0.529	0.764	1.402	0.654
StH	15	79283701	79284000	3.383	2.635	1.674	2.758
StH	15	79540201	79540500	0.242	0.423	0.827	0.396
StH	15	79568101	79568400	0.240	0.277	0.716	0.279
StH	15	79759201	79759500	0.423	1.224	4.199	0.614
StH	15	79782001	79782300	1.010	1.098	1.690	1.262
StH	15	79892101	79892400	2.258	1.939	1.427	2.231
StH	15	79988101	79988400	0.640	0.652	0.156	0.634
StH	15	79992301	79992600	4.859	5.179	6.224	4.168
StH	15	80045101	80045400	1.728	2.075	1.061	2.038
StH	15	80139301	80139600	2.739	2.708	2.027	0.974
StH	15	80381101	80381400	3.876	5.139	2.844	4.467
StH	15	80416501	80416800	0.268	1.201	3.756	0.579
StH	15	80429401	80429700	1.681	2.163	4.762	2.878
StH	15	80429701	80430000	0.215	0.527	2.756	0.633
StH	15	80442301	80442600	1.145	1.659	2.905	1.966
StH	15	80493001	80493300	0.268	0.564	1.946	0.370
StH	15	80499901	80500200	0.211	0.703	2.711	0.598
StH	15	80501701	80502000	3.586	3.615	6.389	4.081
StH	15	80595601	80595900	0.621	1.193	3.597	1.267
StH	15	80690101	80690400	0.319	1.042	3.341	0.493
StH	15	80812501	80812800	1.011	1.098	1.436	0.801
StH	15	80814901	80815200	0.450	0.487	1.458	0.452
StH	16	99601	99900	0.053	0.114	0.448	0.112
StH	16	182101	182400	1.008	1.096	0.535	1.024
StH	16	182401	182700	1.008	0.957	0.510	0.999
StH	16	489001	489300	2.020	2.287	1.168	2.438
StH	16	528001	528300	1.171	1.495	1.358	0.745
StH	16	579601	579900	0.134	0.137	0.531	0.147
StH	16	759901	760200	0.081	0.208	0.506	0.137
StH	16	894001	894300	2.045	1.991	1.280	2.382
StH	16	1054801	1055100	4.181	3.856	3.241	4.112
StH	16	1060501	1060800	4.088	1.914	3.083	4.513
StH	16	1316401	1316700	2.042	1.626	3.321	1.500
StH	16	1371001	1371300	0.158	0.283	0.584	0.385
StH	16	1697701	1698000	0.663	0.703	3.092	1.024
StH	16	1698001	1698300	0.290	0.392	1.123	0.446
StH	16	2054701	2055000	0.773	0.819	2.777	0.431
StH	16	2218501	2218800	0.747	0.914	0.243	0.786
StH	16	2265301	2265600	0.345	0.234	0.107	0.466
StH	16	2266201	2266500	0.663	0.656	0.156	0.634
StH	16	2576101	2576400	2.068	1.873	4.153	2.393
StH	16	2604301	2604600	0.532	0.418	0.916	1.354
StH	16	2898001	2898300	0.582	0.629	0.194	0.634
StH	16	3475201	3475500	1.195	1.176	1.933	1.085
StH	16	3615601	3615900	0.637	1.897	1.239	0.365
StH	16	3663301	3663600	0.319	0.045	0.477	0.279
StH	16	3755401	3755700	0.984	0.959	0.749	0.968
StH	16	4016701	4017000	1.171	1.098	0.847	1.055
StH	16	4049401	4049700	0.239	0.215	0.638	0.167
StH	16	4049701	4050000	0.132	0.118	0.836	0.142
StH	16	4213201	4213500	0.908	0.672	2.898	1.196
StH	16	4237201	4237500	0.531	0.352	0.132	0.329
StH	16	4491001	4491300	1.595	1.897	2.521	1.556
StH	16	4491301	4491600	1.882	2.204	3.540	1.763
StH	16	4605001	4605300	1.173	0.418	0.901	1.192

StH	16	4925101	4925400	0.584	1.030	2.015	0.634
StH	16	4933501	4933800	0.531	0.515	0.288	0.497
StH	16	5382001	5382300	1.089	1.101	1.402	1.075
StH	16	5409001	5409300	1.010	0.725	0.580	0.360
StH	16	5429401	5429700	0.345	0.421	0.584	0.223
StH	16	5865601	5865900	0.240	0.163	0.588	0.259
StH	16	5889301	5889600	0.079	0.586	0.325	0.583
StH	16	5993101	5993400	0.393	0.371	1.008	0.309
StH	16	6087001	6087300	0.160	0.513	0.239	0.416
StH	16	6294901	6295200	0.266	0.345	0.872	0.355
StH	16	6460201	6460500	0.239	0.583	0.428	0.578
StH	16	6794401	6794700	0.532	0.584	0.346	0.583
StH	16	6800701	6801000	0.447	0.611	1.139	0.614
StH	16	6980701	6981000	0.477	0.446	0.831	0.502
StH	16	7203301	7203600	0.345	0.468	0.210	0.360
StH	16	7235401	7235700	2.552	2.336	1.728	2.251
StH	16	7746601	7746900	0.903	0.935	0.588	0.877
StH	16	7930501	7930800	1.329	1.285	0.930	1.410
StH	16	8064901	8065200	0.694	0.421	0.136	0.547
StH	16	8153101	8153400	0.690	0.564	1.041	0.740
StH	16	8174401	8174700	0.373	0.187	0.502	0.142
StH	16	8216101	8216400	1.355	1.594	0.580	1.358
StH	16	8398501	8398800	1.092	0.914	0.745	0.360
StH	16	8556001	8556300	0.477	0.584	0.243	0.893
StH	16	8713801	8714100	0.213	0.257	0.497	0.137
StH	16	8983801	8984100	0.853	0.935	0.609	0.887
StH	16	9068101	9068400	0.398	0.465	0.909	0.355
StH	16	9189301	9189600	4.699	4.804	6.427	4.619
StH	16	9270601	9270900	0.187	0.071	0.633	0.243
StH	16	9413701	9414000	2.126	1.175	3.547	1.811
StH	16	9414001	9414300	0.505	0.369	2.313	0.684
StH	16	9477901	9478200	0.457	1.379	0.818	0.304
StH	16	9501601	9501900	0.294	0.420	0.185	0.527
StH	16	9758401	9758700	0.160	0.092	0.502	0.031
StH	16	9916501	9916800	0.531	0.888	0.272	1.044
StH	16	10610701	10611000	1.803	1.732	1.123	1.658
StH	16	10647901	10648200	1.255	1.569	0.588	1.435
StH	16	11365801	11366100	0.079	0.118	0.526	0.243
StH	16	11413801	11414100	5.212	5.233	3.119	5.572
StH	16	12203401	12203700	1.913	1.635	2.185	1.430
StH	16	12425401	12425700	2.126	3.312	4.046	2.520
StH	16	12435301	12435600	0.186	0.255	0.526	0.106
StH	16	12869401	12869700	0.531	0.184	0.823	0.446
StH	16	13302601	13302900	0.319	0.069	0.700	0.198
StH	16	14229301	14229600	0.160	0.163	0.419	0.031
StH	16	14524201	14524500	1.649	1.075	0.687	1.039
StH	16	15002701	15003000	0.455	0.444	0.745	0.279
StH	16	15354301	15354600	0.347	0.116	0.588	0.380
StH	16	15922501	15922800	0.213	0.118	0.419	0.056
StH	16	16029001	16029300	0.711	1.084	1.855	0.193
StH	16	16381201	16381500	0.428	0.373	0.716	0.284
StH	16	16753801	16754100	0.424	0.470	0.239	1.024
StH	16	16934701	16935000	0.316	0.820	0.078	0.360
StH	16	17155501	17155800	0.184	0.159	0.687	0.162
StH	16	17728801	17729100	0.292	0.275	0.687	0.248
StH	16	17729101	17729400	0.665	0.444	1.077	0.634
StH	16	17786401	17786700	0.479	0.423	0.107	0.411
StH	16	17871301	17871600	8.707	8.762	6.933	9.493
StH	16	18101701	18102000	0.319	0.122	0.905	0.360
StH	16	18318001	18318300	0.318	0.375	0.127	0.745
StH	16	18573901	18574200	1.278	1.626	4.138	2.566
StH	16	18751201	18751500	2.633	4.344	4.809	4.011
StH	16	19032001	19032300	1.513	1.214	0.959	0.745
StH	16	19745701	19746000	0.589	0.584	1.509	0.315
StH	16	20216401	20216700	0.452	0.489	0.691	0.472
StH	16	20283301	20283600	0.079	0.118	0.444	0.142
StH	16	20363401	20363700	0.371	0.491	0.687	0.360

StH	16	20382601	20382900	0.774	0.817	0.165	0.927
StH	16	20482201	20482500	0.452	0.395	0.078	0.274
StH	16	20710201	20710500	0.402	0.305	0.720	0.254
StH	16	21347401	21347700	0.186	0.420	0.292	0.522
StH	16	21408901	21409200	0.161	0.376	0.662	0.254
StH	16	21421201	21421500	0.211	0.167	0.531	0.025
StH	16	21691201	21691500	1.174	1.094	0.054	0.700
StH	16	21923401	21923700	0.132	0.208	0.510	0.248
StH	16	21993901	21994200	0.242	0.234	0.609	0.142
StH	16	21994201	21994500	0.402	0.225	1.431	0.411
StH	16	22714201	22714500	0.586	0.607	0.477	0.583
StH	16	22925401	22925700	0.240	0.187	0.506	0.112
StH	16	23769601	23769900	2.581	2.633	1.226	2.556
StH	16	24581101	24581400	0.292	0.322	0.925	0.370
StH	16	24854701	24855000	1.170	0.729	1.843	0.751
StH	16	24928501	24928800	0.373	0.444	0.132	0.725
StH	16	25911901	25912200	0.773	0.907	0.267	0.756
StH	16	26237401	26237700	3.301	3.510	5.147	3.234
StH	16	26395201	26395500	0.403	0.380	1.008	0.284
StH	16	26664001	26664300	0.453	0.468	0.185	0.421
StH	16	27677701	27678000	0.077	0.071	0.642	0.142
StH	16	27804901	27805200	0.053	0.069	0.798	0.000
StH	16	28280101	28280400	1.676	1.633	1.008	1.359
StH	16	28889701	28890000	0.558	0.674	0.263	0.614
StH	16	28961101	28961400	0.237	0.350	0.716	0.304
StH	16	29136901	29137200	4.038	4.992	2.238	3.209
StH	16	29336101	29336400	0.160	0.208	0.448	0.198
StH	16	29655601	29655900	0.266	0.611	0.424	0.248
StH	16	29715901	29716200	0.215	0.232	0.531	0.299
StH	16	30035701	30036000	0.134	0.187	0.448	0.187
StH	16	30113101	30113400	2.389	1.871	2.858	1.744
StH	16	30251701	30252000	0.106	0.352	0.555	0.690
StH	16	31448701	31449000	0.215	0.230	0.707	0.198
StH	16	31973701	31974000	0.184	0.212	0.671	0.112
StH	16	32773801	32774100	3.936	4.345	3.204	5.116
StH	16	33485101	33485400	0.106	0.116	0.983	0.117
StH	16	34931401	34931700	0.692	0.635	1.593	2.510
StH	16	35460901	35461200	0.290	0.212	0.560	0.187
StH	16	35477701	35478000	2.828	2.757	4.197	2.612
StH	16	35751301	35751600	0.557	0.536	0.856	0.502
StH	16	35822101	35822400	0.211	0.326	0.889	0.248
StH	16	36184801	36185100	0.081	0.583	0.992	0.461
StH	16	36445201	36445500	0.240	0.258	0.448	0.223
StH	16	36703501	36703800	0.294	0.631	0.448	0.106
StH	16	37086601	37086900	0.079	0.191	0.613	0.167
StH	16	37682701	37683000	0.265	0.307	0.662	0.355
StH	16	37838701	37839000	0.342	0.421	1.650	0.584
StH	16	37848001	37848300	0.852	0.828	0.185	0.857
StH	16	37863301	37863600	0.452	0.513	0.925	0.441
StH	16	38514301	38514600	0.266	0.305	0.132	0.578
StH	16	38942101	38942400	0.292	0.442	0.107	0.335
StH	16	39173701	39174000	0.053	0.702	0.267	0.472
StH	16	39283801	39284100	0.265	0.255	0.609	0.309
StH	16	39617101	39617400	1.092	1.103	1.859	1.115
StH	16	39705001	39705300	0.426	0.468	0.156	0.502
StH	16	39835201	39835500	0.507	0.423	0.107	0.497
StH	16	40053301	40053600	0.105	0.163	0.510	0.106
StH	16	40818901	40819200	2.207	1.545	1.173	1.541
StH	16	40932301	40932600	0.690	1.403	0.872	1.105
StH	16	41225401	41225700	0.453	0.420	0.136	0.304
StH	16	41320201	41320500	0.615	0.701	0.185	0.466
StH	16	41489701	41490000	0.902	1.568	2.036	1.293
StH	16	42167401	42167700	0.108	0.279	0.613	0.248
StH	16	42452701	42453000	0.690	0.719	1.168	0.745
StH	16	43149901	43150200	0.318	0.281	0.609	0.106
StH	16	43810201	43810500	0.266	0.348	0.613	0.441
StH	16	44591401	44591700	0.321	0.330	0.049	0.497

StH	16	44810401	44810700	0.268	0.326	0.555	0.218
StH	16	45719401	45719700	0.213	0.257	0.560	0.238
StH	16	45866401	45866700	0.558	0.487	1.164	0.542
StH	16	46573201	46573500	0.110	0.187	0.477	0.167
StH	16	46680601	46680900	1.434	0.611	1.037	0.649
StH	16	46800001	46800300	2.262	1.760	0.798	1.212
StH	16	47305501	47305800	0.745	0.652	0.374	0.913
StH	16	48660001	48660300	0.053	0.163	0.633	0.031
StH	16	49533901	49534200	0.373	0.305	1.119	0.132
StH	16	49758301	49758600	0.558	0.303	0.765	0.391
StH	16	50376601	50376900	0.453	0.399	0.778	0.223
StH	16	50751901	50752200	0.799	0.069	0.428	0.081
StH	16	50812501	50812800	0.797	0.024	0.477	0.000
StH	16	51285001	51285300	0.373	0.586	0.740	0.441
StH	16	51334501	51334800	0.397	0.466	0.185	0.391
StH	16	52137901	52138200	0.266	0.208	0.584	0.117
StH	16	52543201	52543500	0.052	0.045	0.613	0.279
StH	16	52861501	52861800	0.079	0.045	0.477	0.248
StH	16	53114401	53114700	0.452	0.446	0.346	0.248
StH	16	53175901	53176200	0.079	0.071	0.584	0.086
StH	16	54779701	54780000	0.187	0.208	0.448	0.193
StH	16	55731001	55731300	0.557	0.517	0.189	0.634
StH	16	55771201	55771500	0.294	0.257	0.629	0.279
StH	16	55866901	55867200	0.371	0.702	0.239	0.527
StH	16	56135401	56135700	0.239	0.350	0.934	0.340
StH	16	56212201	56212500	0.932	1.006	1.329	1.019
StH	16	56305201	56305500	0.586	0.421	0.292	0.527
StH	16	56335201	56335500	0.476	0.515	0.181	0.806
StH	16	56430601	56430900	0.507	0.375	0.959	0.461
StH	16	56459701	56460000	2.823	2.905	2.044	2.890
StH	16	56519101	56519400	0.347	0.702	0.082	0.385
StH	16	56580001	56580300	0.158	0.140	0.473	0.132
StH	16	57503401	57503700	0.319	0.330	0.555	0.248
StH	16	57549901	57550200	0.558	0.258	0.395	0.664
StH	16	58115101	58115400	0.345	0.656	0.132	0.380
StH	16	58217101	58217400	0.373	0.513	0.189	0.385
StH	16	58326301	58326600	1.831	1.807	1.123	1.795
StH	16	58390801	58391100	1.913	2.086	1.440	2.043
StH	16	58422301	58422600	0.479	0.446	0.267	0.446
StH	16	58503901	58504200	0.742	1.217	0.419	1.156
StH	16	58614001	58614300	3.899	3.712	2.445	3.828
StH	16	58661401	58661700	0.108	0.611	2.316	0.076
StH	16	58784101	58784400	3.578	3.403	1.592	3.702
StH	16	58914901	58915200	0.053	0.167	0.502	0.112
StH	16	59307001	59307300	1.065	0.983	1.275	0.857
StH	16	59492401	59492700	0.342	0.519	1.052	0.329
StH	16	59957701	59958000	0.505	0.444	0.267	0.167
StH	16	60473401	60473700	0.345	0.303	0.617	0.416
StH	16	60644101	60644400	0.081	0.168	0.765	0.259
StH	16	60653101	60653400	0.160	0.420	0.560	0.274
StH	16	61747201	61747500	0.345	0.418	0.769	0.329
StH	16	61898701	61899000	0.215	0.305	0.531	0.248
StH	16	61931701	61932000	0.321	0.446	0.852	0.218
StH	16	62191501	62191800	1.381	0.908	0.448	0.765
StH	16	62343001	62343300	0.163	1.152	2.060	1.079
StH	16	62429701	62430000	0.269	0.497	2.471	0.365
StH	16	62642401	62642700	2.581	2.736	0.502	2.236
StH	16	62642701	62643000	2.412	2.569	0.444	2.282
StH	16	62971801	62972100	0.266	0.283	0.584	0.228
StH	16	63930901	63931200	0.240	0.163	0.745	0.203
StH	16	64014901	64015200	1.010	0.725	0.531	0.882
StH	16	64559101	64559400	1.886	1.103	1.567	1.937
StH	16	64653301	64653600	1.862	1.661	2.896	1.274
StH	16	65274301	65274600	0.903	1.983	7.563	1.421
StH	16	65331001	65331300	0.771	1.004	0.588	1.293
StH	16	65342101	65342400	1.328	1.684	0.905	1.597
StH	16	65477701	65478000	1.752	1.923	0.881	1.678

StH	16	65649001	65649300	0.507	0.494	0.160	0.497
StH	16	65725201	65725500	0.584	0.723	0.399	0.248
StH	16	65922001	65922300	0.294	0.283	0.506	0.081
StH	16	66498601	66498900	0.448	0.504	1.324	0.421
StH	16	66547201	66547500	4.546	4.538	3.188	3.666
StH	16	66558001	66558300	1.999	1.004	1.543	1.060
StH	16	66678601	66678900	0.879	0.862	1.608	0.573
StH	16	66679801	66680100	1.295	1.705	4.345	1.791
StH	16	66702001	66702300	0.720	0.794	0.457	1.141
StH	16	66849901	66850200	0.266	0.279	0.560	0.167
StH	16	67032901	67033200	8.159	5.621	6.572	7.833
StH	16	67232401	67232700	0.132	0.120	0.506	0.142
StH	16	67240801	67241100	0.503	0.328	0.132	0.472
StH	16	67299001	67299300	2.049	1.923	1.280	2.048
StH	16	67322701	67323000	0.615	0.678	0.267	0.527
StH	16	67425001	67425300	1.734	1.937	1.090	1.871
StH	16	67594801	67595100	0.816	1.055	2.764	0.801
StH	16	67752901	67753200	0.053	0.045	0.390	0.056
StH	16	67856701	67857000	0.769	0.493	0.160	0.472
StH	16	68000101	68000400	0.878	1.032	0.609	1.075
StH	16	68035801	68036100	0.903	0.985	0.560	0.948
StH	16	68043601	68043900	0.532	0.350	0.000	0.360
StH	16	68286301	68286600	1.728	2.292	0.996	1.820
StH	16	68293501	68293800	3.376	3.538	1.641	3.336
StH	16	68358901	68359200	2.815	2.375	0.992	2.490
StH	16	68713501	68713800	0.482	1.162	3.524	0.927
StH	16	68977801	68978100	0.108	0.120	0.551	0.056
StH	16	68991901	68992200	2.897	2.452	4.301	2.702
StH	16	69016801	69017100	0.557	0.770	0.160	0.452
StH	16	69133801	69134100	3.747	2.109	2.686	4.107
StH	16	69182701	69183000	0.342	0.423	0.925	0.553
StH	16	69373501	69373800	3.567	3.223	2.579	4.041
StH	16	69435001	69435300	0.586	0.609	0.205	0.472
StH	16	69472201	69472500	0.474	0.236	1.028	0.421
StH	16	69495601	69495900	1.321	1.966	6.585	1.416
StH	16	69609601	69609900	0.453	0.489	0.214	0.416
StH	16	69635101	69635400	1.807	1.315	0.930	1.582
StH	16	69676201	69676500	0.690	0.470	0.189	0.441
StH	16	69729901	69730200	0.184	0.260	0.827	0.446
StH	16	69757801	69758100	4.496	3.548	6.922	5.120
StH	16	69797401	69797700	0.532	1.453	0.136	0.644
StH	16	69852001	69852300	1.592	1.985	6.634	2.536
StH	16	69852301	69852600	0.986	1.130	4.388	1.066
StH	16	69893401	69893700	0.342	0.515	1.037	0.279
StH	16	69993301	69993600	2.394	2.870	1.563	2.753
StH	16	70195801	70196100	0.452	0.880	2.257	0.446
StH	16	70415401	70415700	3.988	3.124	5.245	3.939
StH	16	70488301	70488600	2.155	2.362	1.070	1.734
StH	16	70608301	70608600	0.290	0.590	2.000	0.482
StH	16	70698301	70698600	6.411	4.881	5.565	4.097
StH	16	70741801	70742100	0.479	0.680	0.267	0.578
StH	16	70884301	70884600	0.400	0.746	3.787	0.385
StH	16	70889701	70890000	0.242	1.102	4.752	0.385
StH	16	70975801	70976100	0.311	0.517	2.140	0.446
StH	16	71080201	71080500	0.663	0.796	0.239	0.664
StH	16	71176801	71177100	0.318	0.294	1.696	0.573
StH	16	71192401	71192700	0.958	1.384	2.212	1.339
StH	16	71211001	71211300	0.452	1.307	2.178	0.923
StH	16	71231401	71231700	2.204	1.878	2.916	1.602
StH	16	71246101	71246400	0.576	0.901	3.318	0.472
StH	16	71323501	71323800	0.536	0.542	2.916	0.883
StH	16	71324101	71324400	0.768	1.151	5.990	1.225
StH	16	71324401	71324700	0.997	1.284	5.957	1.401
StH	16	71396101	71396400	0.663	0.908	1.197	0.720
StH	16	71418601	71418900	0.479	0.538	0.239	0.304
StH	16	71430301	71430600	0.186	0.180	1.223	0.187
StH	16	71434501	71434800	3.823	4.396	3.061	4.304

StH	16	71448601	71448900	0.349	1.014	4.039	0.381
StH	16	71452801	71453100	0.027	0.090	0.515	0.000
StH	16	71460301	71460600	0.184	0.495	3.485	0.299
StH	16	71501401	71501700	0.447	0.665	2.238	0.528
StH	16	71505601	71505900	1.615	1.316	4.765	1.080
StH	16	71522401	71522700	1.971	1.813	3.512	1.659
StH	16	71538301	71538600	8.921	10.400	5.120	9.185
StH	16	71550601	71550900	0.187	0.182	0.700	0.218
StH	16	71563801	71564100	1.405	1.495	0.856	1.532
StH	16	71613601	71613900	2.384	2.636	6.948	2.679
StH	16	71669101	71669400	0.857	0.975	3.016	0.725
StH	16	71674501	71674800	0.216	0.542	2.036	0.198
StH	16	71674801	71675100	0.055	0.386	1.527	0.137
StH	16	71681401	71681700	0.132	0.139	0.789	0.288
StH	16	71699101	71699400	0.500	1.199	4.796	0.822
StH	16	71699701	71700000	0.931	0.657	1.251	0.943
StH	17	132301	132600	0.211	0.212	0.551	0.274
StH	17	540001	540300	1.063	1.006	0.629	1.075
StH	17	1528201	1528500	0.134	0.189	0.473	0.198
StH	17	1970401	1970700	0.239	0.165	0.473	0.167
StH	17	1998601	1998900	0.079	0.073	0.736	0.208
StH	17	2189401	2189700	0.215	0.069	0.399	0.583
StH	17	2581201	2581500	0.319	0.420	0.132	0.385
StH	17	2748901	2749200	0.266	0.489	0.082	0.299
StH	17	3048301	3048600	0.850	0.517	1.280	0.567
StH	17	3727201	3727500	2.296	2.471	7.033	2.459
StH	17	3727501	3727800	0.984	1.574	4.297	1.537
StH	17	3866101	3866400	4.314	4.332	3.043	3.919
StH	17	3920401	3920700	1.702	1.802	0.992	1.485
StH	17	3938401	3938700	0.213	0.212	0.675	0.137
StH	17	3978601	3978900	0.742	0.629	0.205	0.614
StH	17	4209301	4209600	0.744	1.075	0.432	0.832
StH	17	4271101	4271400	0.740	0.820	1.893	0.644
StH	17	4283401	4283700	0.689	0.397	0.930	0.664
StH	17	4581301	4581600	1.826	1.712	2.686	1.334
StH	17	4653301	4653600	1.432	1.536	0.785	1.465
StH	17	4810801	4811100	0.426	0.586	0.136	0.411
StH	17	5343001	5343300	1.279	1.054	0.711	1.136
StH	17	5385601	5385900	0.424	0.378	0.740	0.248
StH	17	6277801	6278100	0.692	0.118	0.325	0.578
StH	17	6327001	6327300	0.584	0.348	0.107	0.391
StH	17	6328501	6328800	0.455	0.489	0.136	0.527
StH	17	6444901	6445200	1.170	0.631	0.395	1.267
StH	17	6819301	6819600	0.611	0.395	0.156	0.608
StH	17	7318801	7319100	0.266	0.468	0.185	0.329
StH	17	7386001	7386300	0.644	0.605	1.511	0.436
StH	17	7523701	7524000	0.421	0.230	1.625	0.862
StH	17	8524801	8525100	0.665	0.470	0.214	0.502
StH	17	8721901	8722200	1.410	1.075	0.522	0.933
StH	17	8760601	8760900	0.211	0.375	0.642	0.279
StH	17	9511801	9512100	1.705	2.944	1.090	1.566
StH	17	9748501	9748800	0.160	0.418	0.789	0.268
StH	17	9918001	9918300	0.347	0.444	0.087	0.391
StH	17	9940801	9941100	0.613	0.350	0.156	0.391
StH	17	10143001	10143300	0.426	0.539	0.239	0.446
StH	17	10164601	10164900	0.318	0.468	0.078	0.502
StH	17	10203001	10203300	2.337	2.130	1.431	2.398
StH	17	10296601	10296900	2.910	3.639	5.560	3.554
StH	17	10475701	10476000	0.503	1.493	1.003	0.558
StH	17	11334301	11334600	0.160	0.210	0.477	0.167
StH	17	11752201	11752500	0.132	0.191	0.584	0.162
StH	17	11830801	11831100	1.255	1.105	1.975	1.253
StH	17	12149701	12150000	0.239	0.375	0.613	0.329
StH	17	12347701	12348000	0.797	0.935	0.604	0.329
StH	17	12870301	12870600	0.984	0.562	0.424	0.852
StH	17	13098901	13099200	0.452	0.562	0.691	0.329
StH	17	14492401	14492700	0.134	0.330	0.506	0.223

StH	17	14544901	14545200	0.321	0.257	0.506	0.274
StH	17	15597001	15597300	1.515	1.167	0.662	0.852
StH	17	16232401	16232700	0.716	0.772	0.292	0.628
StH	17	16250101	16250400	0.352	0.330	1.168	0.254
StH	17	16267201	16267500	1.173	1.298	2.403	1.374
StH	17	16381201	16381500	0.186	0.258	0.506	0.223
StH	17	16611601	16611900	0.853	0.631	0.341	0.639
StH	17	16721701	16722000	0.636	1.667	1.139	0.690
StH	17	16755301	16755600	0.716	0.702	0.350	0.608
StH	17	17600101	17600400	1.702	1.890	2.258	1.861
StH	17	17698801	17699100	0.558	0.676	0.239	0.472
StH	17	17730301	17730600	2.065	2.499	0.954	2.388
StH	17	17734801	17735100	1.166	1.148	0.395	1.182
StH	17	20608501	20608800	2.712	2.808	2.278	2.571
StH	17	20709301	20709600	0.026	0.191	0.588	0.198
StH	17	21446701	21447000	0.186	0.068	0.477	0.223
StH	17	23842201	23842500	1.116	0.775	0.448	0.862
StH	17	24308101	24308400	0.266	0.607	0.370	0.472
StH	17	25098901	25099200	0.186	0.139	0.477	0.167
StH	17	25371001	25371300	0.055	0.159	0.551	0.162
StH	17	25749901	25750200	0.720	0.633	0.259	0.603
StH	17	25949701	25950000	0.586	0.421	0.156	0.274
StH	17	26225401	26225700	2.176	1.777	1.015	0.207
StH	17	28567501	28567800	0.828	0.588	0.181	1.348
StH	17	28646101	28646400	0.158	0.116	0.497	0.112
StH	17	28695001	28695300	0.876	0.538	0.160	0.821
StH	17	28715101	28715400	0.720	0.772	2.313	0.842
StH	17	28715401	28715700	0.644	0.566	2.042	0.675
StH	17	28743901	28744200	0.531	0.727	0.132	0.365
StH	17	28761601	28761900	3.439	2.620	1.424	3.215
StH	17	29253601	29253900	0.532	0.607	0.399	0.751
StH	17	29576401	29576700	0.106	0.094	0.486	0.061
StH	17	29786701	29787000	0.321	0.375	0.584	0.329
StH	17	29867401	29867700	0.294	0.232	0.506	0.081
StH	17	31167001	31167300	0.000	0.142	0.675	0.112
StH	17	32477701	32478000	0.398	0.607	0.502	0.360
StH	17	33919201	33919500	1.408	0.869	2.044	1.202
StH	17	34365601	34365900	0.290	0.330	0.642	0.193
StH	17	34434601	34434900	0.268	0.212	0.477	0.162
StH	17	34926901	34927200	2.221	2.926	1.090	2.545
StH	17	34927201	34927500	2.413	2.671	1.037	2.353
StH	17	36862201	36862500	0.771	1.174	0.263	0.852
StH	17	37346401	37346700	0.481	0.423	0.156	0.527
StH	17	37744501	37744800	0.106	0.347	0.695	0.228
StH	17	38649001	38649300	0.161	0.307	0.682	0.167
StH	17	39328201	39328500	1.381	1.403	1.748	0.213
StH	17	39498901	39499200	5.270	3.008	4.456	3.386
StH	17	39531001	39531300	0.373	0.515	0.823	0.416
StH	17	39717001	39717300	0.611	0.163	0.296	0.112
StH	17	40082701	40083000	1.091	1.056	0.695	1.156
StH	17	40084201	40084500	0.213	0.215	0.818	0.299
StH	17	40107901	40108200	0.878	1.028	0.473	0.887
StH	17	40352101	40352400	0.053	0.446	0.267	0.416
StH	17	40859401	40859700	0.345	0.236	0.633	0.416
StH	17	41252101	41252400	0.505	0.258	0.687	0.527
StH	17	41690101	41690400	0.398	0.328	0.814	0.441
StH	17	43273801	43274100	0.878	0.702	0.531	0.690
StH	17	43456201	43456500	0.929	0.822	0.477	0.999
StH	17	43521301	43521600	0.055	0.069	0.502	0.126
StH	17	44111101	44111400	2.318	2.195	1.728	2.231
StH	17	44123701	44124000	0.136	0.094	0.526	0.092
StH	17	44136301	44136600	2.342	1.961	1.505	2.484
StH	17	44230201	44230500	2.317	2.038	1.576	2.718
StH	17	44386501	44386800	0.187	0.096	0.448	0.117
StH	17	44506801	44507100	0.903	0.161	0.588	0.913
StH	17	44512501	44512800	2.802	3.984	6.917	3.036
StH	17	44512801	44513100	1.091	0.818	1.641	0.918

StH	17	44772001	44772300	0.611	0.607	0.288	0.527
StH	17	44880001	44880300	1.329	0.770	0.979	0.725
StH	17	44895001	44895300	0.689	0.489	1.144	0.268
StH	17	45314101	45314400	0.744	0.863	0.350	0.725
StH	17	46220401	46220700	0.718	0.702	1.016	0.715
StH	17	47145301	47145600	0.424	0.470	0.127	0.527
StH	17	47428201	47428500	0.481	0.401	0.078	0.492
StH	17	48035101	48035400	0.132	0.165	0.419	0.137
StH	17	48117001	48117300	1.408	0.727	1.144	0.887
StH	17	49002301	49002600	0.481	0.446	0.185	0.472
StH	17	49055101	49055400	1.986	2.251	0.292	1.683
StH	17	49279201	49279500	0.373	0.397	0.740	0.254
StH	17	49370101	49370400	1.329	0.843	0.234	0.943
StH	17	49386301	49386600	48.941	57.241	40.551	48.033
StH	17	49688101	49688400	1.039	1.641	1.329	1.633
StH	17	49721401	49721700	0.637	0.678	0.165	0.567
StH	17	50084701	50085000	2.371	1.970	3.270	1.982
StH	17	50939701	50940000	2.289	2.450	1.587	2.413
StH	17	50979601	50979900	0.532	0.633	0.374	0.826
StH	17	51168301	51168600	1.091	1.167	0.687	1.206
StH	17	51605701	51606000	0.771	0.444	0.243	0.522
StH	17	52081801	52082100	0.347	0.770	0.526	0.248
StH	17	52441201	52441500	0.560	0.747	0.288	0.547
StH	17	52632301	52632600	0.816	1.173	5.416	0.710
StH	17	52666501	52666800	9.217	9.567	6.342	9.340
StH	17	53130601	53130900	0.294	0.607	0.132	0.472
StH	17	53301301	53301600	3.246	3.456	2.163	3.605
StH	17	53422501	53422800	0.347	0.466	0.136	0.553
StH	17	53625001	53625300	0.186	0.142	0.506	0.243
StH	17	53872201	53872500	0.106	0.210	0.419	0.162
StH	17	53950201	53950500	0.186	0.212	0.629	0.056
StH	17	54206101	54206400	0.373	0.446	0.214	0.522
StH	17	54576001	54576300	0.026	0.140	0.526	0.117
StH	17	54698101	54698400	1.491	1.191	0.622	1.176
StH	17	54762301	54762600	0.745	0.796	0.379	0.664
StH	17	54918601	54918900	0.055	0.071	0.531	0.051
StH	17	54974401	54974700	0.266	0.281	0.448	0.198
StH	17	55115101	55115400	0.265	0.376	0.695	0.248
StH	17	55302001	55302300	0.505	0.926	3.470	0.725
StH	17	55304401	55304700	6.712	6.837	5.105	7.109
StH	17	55713601	55713900	0.108	0.144	0.575	0.167
StH	17	55765801	55766100	0.452	0.678	0.243	0.466
StH	17	56376001	56376300	0.160	0.210	0.419	0.137
StH	17	56392501	56392800	0.426	0.468	0.239	0.360
StH	17	56400601	56400900	0.053	0.023	0.419	0.198
StH	17	56482501	56482800	1.537	1.944	0.831	1.485
StH	17	56647201	56647500	0.505	0.513	0.292	0.446
StH	17	57246601	57246900	0.561	0.517	0.103	0.527
StH	17	57721201	57721500	3.965	4.169	2.542	4.478
StH	17	57772801	57773100	0.479	0.442	0.774	0.304
StH	17	58210801	58211100	0.637	0.586	0.185	0.329
StH	17	58313701	58314000	2.655	2.735	1.572	2.191
StH	17	58470601	58470900	0.452	0.609	0.239	0.492
StH	17	58993501	58993800	3.004	2.662	1.855	2.130
StH	17	59171101	59171400	3.321	2.957	1.480	3.012
StH	17	59171401	59171700	4.331	3.602	1.973	3.611
StH	17	59641801	59642100	1.735	2.414	4.347	2.454
StH	17	59686801	59687100	0.794	0.614	1.843	1.247
StH	17	59760901	59761200	0.373	0.725	0.580	0.329
StH	17	60355201	60355500	3.454	3.411	2.403	3.357
StH	17	60391201	60391500	1.862	2.218	1.518	2.216
StH	17	60455701	60456000	0.699	1.119	3.380	1.111
StH	17	60510301	60510600	0.639	0.652	0.925	0.355
StH	17	60683101	60683400	0.186	0.421	0.531	0.304
StH	17	60805501	60805800	0.534	0.489	1.052	0.426
StH	17	60860701	60861000	0.398	0.961	0.720	0.436
StH	17	61084801	61085100	0.400	0.399	0.267	0.664

StH	17	61664401	61664700	3.163	3.154	2.847	2.464
StH	17	61938601	61938900	0.692	0.654	0.346	0.740
StH	17	62137801	62138100	2.868	2.309	1.522	2.652
StH	17	62142901	62143200	1.116	0.817	0.584	1.050
StH	17	62147101	62147400	2.773	3.414	1.872	3.078
StH	17	62175301	62175600	4.596	6.213	3.061	4.453
StH	17	62197201	62197500	1.271	1.409	3.009	1.334
StH	17	62342701	62343000	0.136	0.976	3.775	0.497
StH	17	62473201	62473500	0.795	0.680	0.234	0.608
StH	17	62618101	62618400	1.907	1.625	6.053	1.826
StH	17	62862901	62863200	0.640	1.669	0.054	1.424
StH	17	62921401	62921700	0.371	0.446	0.054	0.472
StH	17	63033901	63034200	0.903	0.912	0.555	0.801
StH	17	63069601	63069900	0.752	1.061	4.267	1.202
StH	17	63069901	63070200	4.917	5.710	9.538	5.547
StH	17	63104401	63104700	0.800	1.006	3.781	1.241
StH	17	63110701	63111000	1.427	0.719	2.777	0.968
StH	17	63111001	63111300	2.032	0.845	3.921	1.344
StH	17	63280501	63280800	1.149	1.238	0.453	1.152
StH	17	63500101	63500400	0.108	0.114	1.529	0.101
StH	17	63500401	63500700	0.502	0.832	3.954	0.533
StH	17	63582601	63582900	1.647	1.569	1.012	2.257
StH	17	63582901	63583200	0.853	0.584	0.321	0.877
StH	17	63678301	63678600	1.139	2.600	1.683	1.166
StH	17	63760501	63760800	0.345	0.324	0.671	0.167
StH	17	63810001	63810300	1.434	1.637	0.987	1.511
StH	17	64064701	64065000	4.785	3.362	7.110	5.740
StH	17	64081201	64081500	0.158	0.204	1.087	0.218
StH	17	64193701	64194000	0.313	0.283	1.102	0.122
StH	17	64290001	64290300	1.511	1.375	0.736	1.471
StH	17	64355101	64355400	2.025	1.920	1.206	1.825
StH	17	64384501	64384800	0.987	1.169	0.239	1.561
StH	17	64387501	64387800	0.292	0.352	0.613	0.466
StH	17	64418101	64418400	0.239	0.328	0.604	0.132
StH	17	64855801	64856100	0.666	1.073	0.234	1.044
StH	17	64932901	64933200	1.145	0.657	0.239	1.186
StH	17	64990801	64991100	4.501	4.403	6.039	4.372
StH	17	64991101	64991400	4.388	3.920	5.047	4.325
StH	17	65013301	65013600	2.129	2.103	3.861	1.916
StH	17	65020501	65020800	0.344	0.393	0.774	0.137
StH	17	65140801	65141100	1.252	1.245	0.584	1.897
StH	17	65551801	65552100	1.673	1.257	0.638	1.131
StH	17	65584501	65584800	0.824	0.607	0.477	0.776
StH	17	65657101	65657400	1.066	1.079	0.254	0.994
StH	17	65689801	65690100	0.216	0.116	0.617	0.223
StH	17	65732701	65733000	0.665	0.794	0.181	0.628
StH	17	65766901	65767200	0.265	0.140	0.617	0.274
StH	17	65864401	65864700	0.505	0.817	3.397	1.045
StH	17	66388201	66388500	1.194	1.356	0.852	1.348
StH	17	66443401	66443700	2.741	3.109	3.957	2.935
StH	17	66480301	66480600	0.318	0.903	1.888	0.858
StH	17	66504601	66504900	3.143	2.729	2.098	3.712
StH	17	66668701	66669000	1.705	1.626	4.015	1.856
StH	17	66687601	66687900	0.589	2.545	1.447	0.553
StH	17	66747301	66747600	2.758	1.047	2.151	1.602
StH	17	66955801	66956100	0.268	0.773	0.506	0.274
StH	17	66964801	66965100	1.116	1.335	0.720	0.913
StH	17	67139701	67140000	0.240	0.446	0.025	0.223
StH	17	67782001	67782300	0.611	1.091	3.503	1.238
StH	17	67795201	67795500	3.297	3.132	4.911	2.779
StH	17	67833301	67833600	0.558	0.702	0.395	0.745
StH	17	67871701	67872000	0.079	0.468	0.234	0.634
StH	17	68104501	68104800	0.532	0.446	0.132	0.411
StH	17	68119201	68119500	0.106	0.392	0.876	0.213
StH	17	68166301	68166600	0.189	0.257	0.745	0.385
StH	17	68193901	68194200	1.016	0.929	2.271	0.594
StH	17	68330701	68331000	6.275	8.483	4.975	6.681

StH	17	68367001	68367300	1.542	3.064	2.798	1.552
StH	17	68419201	68419500	0.613	2.152	0.876	0.745
StH	17	68781301	68781600	0.639	1.352	0.152	0.472
StH	17	68817901	68818200	1.788	1.372	4.380	1.547
StH	17	69063601	69063900	0.778	0.890	1.670	0.913
StH	17	69095101	69095400	0.211	0.187	0.535	0.218
StH	17	69124801	69125100	0.079	0.116	0.502	0.132
StH	17	69149101	69149400	0.795	0.888	1.148	0.608
StH	17	69173101	69173400	1.113	2.311	1.636	1.019
StH	17	69194701	69195000	2.126	2.058	1.041	1.836
StH	17	69231001	69231300	0.240	0.184	0.785	0.268
StH	17	69243301	69243600	0.720	0.643	2.657	0.802
StH	17	69417001	69417300	0.134	0.229	0.959	0.081
StH	17	69570301	69570600	1.039	0.867	0.486	0.999
StH	17	69579301	69579600	3.222	3.351	2.506	3.965
StH	17	69640201	69640500	1.889	1.266	1.567	2.124
StH	17	69742501	69742800	0.479	1.175	2.484	1.202
StH	17	70024801	70025100	1.541	1.847	0.662	1.181
StH	17	70115401	70115700	1.386	1.498	0.749	1.253
StH	17	70197001	70197300	0.106	0.257	0.502	0.051
StH	17	70425301	70425600	1.219	1.234	0.642	1.202
StH	17	70431601	70431900	2.260	1.377	3.536	2.484
StH	17	70437601	70437900	1.250	1.189	0.720	1.201
StH	17	70437901	70438200	1.783	1.899	0.691	1.820
StH	17	70459801	70460100	0.027	0.023	0.419	0.061
StH	17	70474801	70475100	0.400	0.879	5.132	0.274
StH	17	70675201	70675500	0.052	1.014	4.471	0.401
StH	17	70727401	70727700	0.371	0.437	1.128	0.452
StH	17	70788901	70789200	0.373	0.229	1.754	0.218
StH	17	70870201	70870500	1.413	2.142	8.096	1.521
StH	17	70878301	70878600	0.079	0.253	0.613	0.142
StH	17	70891201	70891500	0.240	0.300	2.117	0.243
StH	17	70906501	70906800	0.826	1.306	4.839	0.704
StH	17	70910401	70910700	0.352	0.270	1.674	0.243
StH	17	70971901	70972200	0.670	0.579	1.456	0.760
StH	17	70974301	70974600	1.753	2.385	4.329	2.098
StH	17	71025901	71026200	0.321	0.609	2.113	0.558
StH	17	71031901	71032200	0.186	0.632	2.693	0.360
StH	17	71033101	71033400	0.077	0.337	1.845	0.086
StH	17	71040001	71040300	0.450	1.049	3.205	0.436
StH	17	71080801	71081100	0.398	0.092	0.584	0.335
StH	17	71087101	71087400	0.027	0.319	2.536	0.223
StH	17	71088001	71088300	0.216	0.607	2.951	0.361
StH	17	71103301	71103600	0.237	0.542	2.314	0.193
StH	17	71121901	71122200	0.608	1.072	2.847	0.614
StH	17	71190601	71190900	1.199	0.953	1.810	1.232
StH	17	71190901	71191200	0.747	0.386	1.658	0.603
StH	17	71247001	71247300	3.107	3.137	2.539	4.117
StH	17	71330401	71330700	1.376	1.395	2.341	1.430
StH	17	71359201	71359500	0.507	0.345	1.168	0.659
StH	17	71361601	71361900	0.294	0.611	2.313	0.507
StH	17	71386501	71386800	0.053	0.454	1.560	0.112
StH	17	71457601	71457900	0.132	0.157	0.695	0.173
StH	17	71467801	71468100	1.218	1.042	4.418	1.274
StH	17	71475301	71475600	0.371	1.018	3.347	0.715
StH	17	71475601	71475900	0.294	0.590	2.189	0.574
StH	17	71490901	71491200	0.000	0.092	0.626	0.056
StH	17	71536201	71536500	1.549	1.167	2.377	1.222
StH	17	71644801	71645100	0.106	0.341	1.259	0.198
StH	17	71653801	71654100	0.187	0.521	2.768	0.664
StH	17	71672101	71672400	0.265	0.654	2.325	0.208
StH	17	71672401	71672700	0.079	0.270	1.540	0.061
StH	17	71705101	71705400	0.636	0.815	2.608	0.695
StH	17	71707501	71707800	0.158	0.429	2.195	0.445
StH	17	71749201	71749500	0.431	0.699	3.971	0.497
StH	17	71754601	71754900	0.295	0.251	0.881	0.193
StH	17	71781301	71781600	0.158	1.038	4.229	0.295

StH	17	71871001	71871300	0.611	0.908	1.807	0.902
StH	17	71890801	71891100	1.329	1.686	4.781	1.430
StH	17	72005101	72005400	0.242	0.225	1.832	0.416
StH	17	72015301	72015600	0.263	0.613	2.144	0.187
StH	17	72036901	72037200	0.136	0.270	1.531	0.162
StH	17	72045301	72045600	0.189	0.159	0.995	0.193
StH	17	72126901	72127200	0.326	1.036	4.509	0.350
StH	17	72144901	72145200	0.666	1.218	4.171	1.167
StH	17	72153301	72153600	0.929	1.929	3.626	1.156
StH	17	72180601	72180900	0.292	0.112	0.740	0.076
StH	17	72180901	72181200	0.240	0.500	2.050	0.457
StH	17	72255301	72255600	0.452	0.163	0.560	0.142
StH	17	72271201	72271500	0.106	0.337	1.514	0.056
StH	18	12001	12300	2.123	2.392	1.209	2.358
StH	18	101701	102000	1.784	2.971	1.086	1.582
StH	18	116701	117000	1.758	1.670	5.842	1.400
StH	18	784501	784800	0.237	0.213	0.687	0.182
StH	18	1032601	1032900	0.077	0.140	0.502	0.142
StH	18	1440001	1440300	2.262	2.379	1.650	2.252
StH	18	1791301	1791600	0.211	0.140	0.473	0.112
StH	18	1919101	1919400	0.610	0.539	0.954	0.669
StH	18	1919401	1919700	2.497	1.905	6.734	2.410
StH	18	2313301	2313600	0.105	0.071	0.564	0.076
StH	18	3427501	3427800	0.795	0.704	1.674	0.837
StH	18	3844201	3844500	0.240	0.189	0.531	0.076
StH	18	3922201	3922500	0.345	0.328	0.769	0.365
StH	18	3966601	3966900	1.170	0.373	0.609	0.771
StH	18	4680901	4681200	1.541	1.448	0.805	1.709
StH	18	5511301	5511600	1.008	0.843	0.424	0.852
StH	18	5727601	5727900	0.692	0.723	0.259	0.801
StH	18	5853901	5854200	0.186	0.397	0.740	0.335
StH	18	6386101	6386400	0.402	0.468	0.132	0.502
StH	18	6972301	6972600	1.328	1.306	2.089	1.313
StH	18	6989101	6989400	0.823	0.841	0.667	0.857
StH	18	7003501	7003800	0.560	0.629	0.165	0.441
StH	18	7015201	7015500	0.668	0.375	1.003	0.329
StH	18	7159501	7159800	0.821	0.508	1.585	0.522
StH	18	7174201	7174500	1.329	1.309	1.489	1.111
StH	18	7207501	7207800	0.610	0.886	3.133	0.822
StH	18	7433701	7434000	1.386	1.968	0.827	2.003
StH	18	7593001	7593300	1.130	1.918	3.119	0.821
StH	18	7873201	7873500	2.866	2.707	1.797	2.935
StH	18	8006401	8006700	0.981	1.077	0.584	0.907
StH	18	8079901	8080200	0.477	0.305	0.156	0.000
StH	18	8365201	8365500	0.082	0.118	0.448	0.051
StH	18	8771701	8772000	0.211	0.234	0.502	0.223
StH	18	9281401	9281700	0.265	0.163	0.613	0.081
StH	18	9477301	9477600	2.763	2.478	1.603	2.485
StH	18	9593701	9594000	1.094	0.910	3.157	0.699
StH	18	9594001	9594300	0.774	0.768	1.759	0.452
StH	18	9788401	9788700	0.453	0.611	0.214	0.578
StH	18	10109101	10109400	2.712	1.731	1.913	2.043
StH	18	10140601	10140900	1.391	1.805	3.188	1.653
StH	18	10255501	10255800	1.144	1.356	1.654	1.242
StH	18	10578901	10579200	0.505	0.350	0.210	0.522
StH	18	11271601	11271900	0.344	0.140	0.584	0.243
StH	18	11366401	11366700	0.611	0.491	1.057	0.452
StH	18	11371501	11371800	0.345	0.397	0.667	0.279
StH	18	11704501	11704800	0.402	1.008	0.695	0.309
StH	18	11967901	11968200	4.728	3.875	6.150	4.182
StH	18	11977201	11977500	2.341	2.135	0.785	2.139
StH	18	12045301	12045600	0.053	0.094	0.658	0.887
StH	18	12075001	12075300	0.689	0.957	2.361	1.323
StH	18	12434101	12434400	1.063	1.144	0.802	1.491
StH	18	12553501	12553800	0.558	0.538	0.317	0.492
StH	18	12934801	12935100	0.215	0.232	0.448	0.223
StH	18	13118101	13118400	0.265	0.023	0.584	0.360

StH	18	13168801	13169100	0.242	0.139	0.760	0.056
StH	18	13311001	13311300	2.813	2.600	1.494	2.394
StH	18	13479301	13479600	0.213	0.376	0.584	0.720
StH	18	13519801	13520100	4.438	5.430	3.739	5.141
StH	18	13715101	13715400	1.308	0.903	2.737	0.832
StH	18	13899301	13899600	0.611	0.699	1.008	0.385
StH	18	14154901	14155200	3.358	2.753	0.312	4.785
StH	18	14299801	14300100	0.186	0.277	1.008	1.465
StH	18	15422701	15423000	2.128	1.901	1.019	2.201
StH	18	15426601	15426900	1.276	1.240	0.510	1.064
StH	18	15510001	15510300	2.868	2.663	1.589	2.597
StH	18	15716701	15717000	2.370	2.289	4.343	2.389
StH	18	15759601	15759900	0.239	0.300	0.700	0.137
StH	18	15759901	15760200	0.536	0.643	1.514	0.324
StH	18	15771901	15772200	4.601	4.679	3.668	5.553
StH	18	16263901	16264200	0.132	0.493	0.263	0.137
StH	18	17204701	17205000	0.453	0.470	0.156	0.553
StH	18	17224801	17225100	0.265	0.772	0.555	0.335
StH	18	17373901	17374200	0.266	0.279	0.448	0.167
StH	18	17388001	17388300	0.318	0.139	0.531	0.248
StH	18	18102901	18103200	2.857	3.811	1.404	3.995
StH	18	18103201	18103500	0.613	0.729	0.181	0.639
StH	18	18425401	18425700	0.692	0.607	0.428	0.745
StH	18	18453301	18453600	0.853	1.268	1.645	0.659
StH	18	18494701	18495000	0.611	0.491	0.341	0.472
StH	18	19246801	19247100	0.610	0.678	0.103	0.461
StH	18	19324201	19324500	0.266	0.513	0.399	0.167
StH	18	19334101	19334400	0.263	0.493	0.852	0.547
StH	18	19614301	19614600	2.478	3.581	1.300	2.291
StH	18	20051401	20051700	0.265	0.232	0.658	0.056
StH	18	20182201	20182500	1.005	1.253	2.214	0.745
StH	18	20185501	20185800	0.655	1.378	5.672	1.045
StH	18	20380801	20381100	0.132	0.300	0.716	0.274
StH	18	20543401	20543700	0.558	0.704	0.181	0.497
StH	18	20544901	20545200	0.960	1.332	0.580	1.262
StH	18	20589001	20589300	0.215	0.182	0.831	0.142
StH	18	20596201	20596500	0.745	0.772	1.395	0.943
StH	18	20683501	20683800	1.408	1.354	1.888	1.435
StH	18	20735701	20736000	1.081	2.136	5.182	1.206
StH	18	20848501	20848800	1.276	1.424	1.037	1.912
StH	18	20879401	20879700	0.400	0.399	0.107	0.603
StH	18	20935501	20935800	0.665	0.656	0.181	0.771
StH	18	21045601	21045900	0.558	0.558	0.234	0.659
StH	18	21091201	21091500	0.771	0.493	1.119	0.497
StH	18	21222301	21222600	4.833	5.655	3.841	4.558
StH	18	21251101	21251400	4.412	5.877	3.540	5.126
StH	18	22176601	22176900	1.916	1.592	1.308	1.024
StH	18	22194001	22194300	3.773	3.459	4.492	5.004
StH	18	22316701	22317000	0.613	0.639	1.605	0.654
StH	18	22382701	22383000	0.263	0.826	2.260	0.887
StH	18	22449901	22450200	0.666	0.414	1.735	0.766
StH	18	23316601	23316900	0.503	0.305	0.774	0.583
StH	18	23514601	23514900	0.557	0.609	0.263	0.502
StH	18	23565601	23565900	0.611	0.461	1.402	0.690
StH	18	23790001	23790300	0.108	0.210	0.502	0.198
StH	18	24682501	24682800	0.769	0.792	0.535	0.274
StH	18	24973801	24974100	0.240	0.208	0.584	0.279
StH	18	25399801	25400100	0.371	0.380	0.938	0.243
StH	18	25534801	25535100	0.426	0.489	0.691	0.385
StH	18	26265601	26265900	0.529	0.444	0.160	0.522
StH	18	26371201	26371500	0.560	0.521	0.054	0.715
StH	18	26475901	26476200	0.477	0.609	0.317	0.472
StH	18	27153601	27153900	0.240	0.140	0.564	0.329
StH	18	27206101	27206400	0.771	0.444	0.165	0.411
StH	18	27302401	27302700	0.531	0.442	0.185	0.547
StH	18	27596401	27596700	0.105	0.073	0.419	0.081
StH	18	28004701	28005000	0.237	0.118	0.551	0.223

StH	18	28215601	28215900	0.532	0.350	0.185	0.441
StH	18	28708201	28708500	1.797	1.731	0.390	1.678
StH	18	29200801	29201100	0.455	0.421	0.156	0.497
StH	18	29620201	29620500	1.329	1.472	0.876	1.024
StH	18	30025501	30025800	0.318	0.234	0.535	0.137
StH	18	30047101	30047400	1.594	1.849	1.086	1.638
StH	18	30078901	30079200	2.284	2.225	1.674	2.104
StH	18	30201601	30201900	0.848	0.936	1.193	0.801
StH	18	30523801	30524100	0.215	0.140	0.580	0.254
StH	18	30885601	30885900	0.398	0.232	0.613	0.274
StH	18	30978901	30979200	0.558	0.680	0.346	0.472
StH	18	31020601	31020900	0.156	0.139	0.539	0.081
StH	18	31223701	31224000	0.426	0.491	0.185	0.335
StH	18	31282501	31282800	1.862	1.996	0.963	1.729
StH	18	31584601	31584900	1.173	0.702	0.477	0.695
StH	18	31789201	31789500	1.970	1.319	4.331	1.597
StH	18	31828201	31828500	0.690	0.815	1.271	0.918
StH	18	31903801	31904100	0.187	0.253	0.604	0.248
StH	18	32000701	32001000	0.632	0.510	1.886	0.619
StH	18	32042401	32042700	0.613	0.491	0.292	0.466
StH	18	32252101	32252400	1.199	1.219	0.774	1.430
StH	18	32448001	32448300	0.160	0.360	1.280	0.112
StH	18	32448301	32448600	0.474	2.159	9.898	0.989
StH	18	32457601	32457900	0.160	0.236	0.551	0.193
StH	18	32579401	32579700	1.910	1.843	1.110	1.775
StH	18	33378301	33378600	0.347	0.864	2.969	0.355
StH	18	33458101	33458400	1.326	1.148	1.921	1.404
StH	18	33970801	33971100	1.566	1.337	0.502	1.262
StH	18	34157401	34157700	0.610	0.609	0.370	0.832
StH	18	34165201	34165500	0.558	0.328	0.025	0.421
StH	18	34271701	34272000	2.204	1.965	0.769	1.763
StH	18	34327501	34327800	0.740	1.644	0.000	0.934
StH	18	34327801	34328100	0.718	0.959	0.132	0.614
StH	18	34616101	34616400	0.211	0.185	0.539	0.198
StH	18	34644601	34644900	0.902	2.060	1.674	1.202
StH	18	35002501	35002800	0.106	0.281	0.580	0.268
StH	18	35485201	35485500	0.560	0.212	0.374	0.162
StH	18	35533801	35534100	0.663	0.818	0.350	0.695
StH	18	37755601	37755900	0.187	0.210	0.054	0.466
StH	18	38134801	38135100	1.808	1.242	0.749	1.091
StH	18	38171701	38172000	0.266	0.303	0.711	0.365
StH	18	38691601	38691900	0.290	0.491	0.082	0.304
StH	18	39210601	39210900	1.092	1.429	1.797	0.832
StH	18	39260101	39260400	0.187	0.092	0.609	0.472
StH	18	40076401	40076700	0.081	0.225	0.876	0.193
StH	18	40752301	40752600	0.424	0.513	0.103	0.274
StH	18	40954801	40955100	0.903	0.964	0.399	0.913
StH	18	41170801	41171100	0.665	0.185	1.645	1.152
StH	18	41830801	41831100	0.532	0.609	0.189	0.710
StH	18	41878501	41878800	0.186	0.092	0.522	0.106
StH	18	42111901	42112200	0.053	0.090	0.444	0.081
StH	18	42911701	42912000	0.239	0.232	0.506	0.659
StH	18	43193101	43193400	0.261	0.296	1.336	0.268
StH	18	43908001	43908300	0.398	0.513	0.136	0.466
StH	18	44307001	44307300	0.187	0.491	1.128	0.167
StH	18	44307301	44307600	0.240	0.628	1.128	0.309
StH	18	44497201	44497500	0.373	0.212	0.588	0.304
StH	18	44535901	44536200	0.586	0.328	0.156	0.355
StH	18	46016401	46016700	0.371	0.581	0.934	0.558
StH	18	46236001	46236300	0.797	0.837	1.476	0.821
StH	18	47091001	47091300	0.479	0.543	0.963	0.304
StH	18	47677201	47677500	0.292	0.420	0.078	0.193
StH	18	47684701	47685000	0.319	0.324	0.638	0.355
StH	18	48162901	48163200	5.159	4.814	3.364	4.548
StH	18	49190401	49190700	1.105	0.092	2.263	1.105
StH	18	50636701	50637000	0.400	0.423	0.638	0.279
StH	18	50999101	50999400	0.239	0.251	0.667	0.193

StH	18	51029401	51029700	0.319	0.539	0.662	0.304
StH	18	51931201	51931500	0.134	0.090	0.539	0.112
StH	18	51931501	51931800	0.215	0.157	1.135	0.223
StH	18	52385401	52385700	0.266	0.281	0.502	0.309
StH	18	53482201	53482500	0.636	0.656	1.028	0.385
StH	18	53733901	53734200	0.903	1.008	0.403	0.953
StH	18	53873101	53873400	0.852	0.815	0.379	0.826
StH	18	54100501	54100800	2.446	3.107	2.102	2.657
StH	18	54517201	54517500	0.158	0.283	0.609	0.416
StH	18	54604801	54605100	1.357	1.292	2.118	1.627
StH	18	54700801	54701100	0.477	0.772	0.667	0.441
StH	18	54802801	54803100	0.211	0.210	0.399	0.553
StH	18	55062901	55063200	0.558	0.562	0.210	0.385
StH	18	55648501	55648800	0.187	0.302	0.584	0.106
StH	18	56476801	56477100	2.021	1.849	2.151	1.770
StH	18	56485201	56485500	0.799	0.657	1.226	0.882
StH	18	56502001	56502300	0.106	0.165	0.403	0.639
StH	18	56571901	56572200	0.934	0.727	0.263	0.664
StH	18	56592601	56592900	2.866	3.094	1.759	3.276
StH	18	56950201	56950500	0.211	0.210	0.564	0.056
StH	18	57269101	57269400	0.373	0.470	0.132	0.365
StH	18	57462001	57462300	0.534	0.543	1.012	0.477
StH	18	57633901	57634200	0.744	1.545	1.081	1.521
StH	18	57645901	57646200	0.345	0.423	0.111	0.360
StH	18	57697501	57697800	0.773	0.744	0.419	1.242
StH	18	57906901	57907200	0.477	0.470	0.132	0.472
StH	18	58492501	58492800	1.328	1.386	0.852	1.410
StH	18	58616101	58616400	0.450	0.446	0.210	0.497
StH	18	58665601	58665900	2.736	2.618	1.815	2.307
StH	18	58700101	58700400	0.718	0.702	0.560	1.323
StH	18	58824301	58824600	0.211	0.212	0.667	0.162
StH	18	59149801	59150100	0.213	0.210	0.510	0.218
StH	18	59223301	59223600	0.371	0.583	0.905	0.527
StH	18	59338501	59338800	0.319	0.444	0.136	0.248
StH	18	59353201	59353500	0.292	0.331	0.029	0.553
StH	18	59397901	59398200	3.084	2.176	2.566	2.185
StH	18	59575501	59575800	0.613	0.558	0.214	0.553
StH	18	60195301	60195600	0.666	0.777	0.132	0.948
StH	18	60204901	60205200	0.453	0.539	0.745	0.198
StH	18	60225001	60225300	0.453	0.657	0.049	0.852
StH	18	60234901	60235200	6.282	7.507	3.228	8.171
StH	18	60273301	60273600	0.215	0.157	0.954	0.198
StH	18	60467101	60467400	0.079	0.446	0.317	0.193
StH	18	60540001	60540300	1.403	1.678	3.179	1.578
StH	18	60650401	60650700	0.613	0.515	0.909	0.614
StH	18	60783301	60783600	0.611	0.466	0.185	0.502
StH	18	60788701	60789000	0.345	0.515	0.107	0.340
StH	18	60913801	60914100	0.210	0.189	0.662	0.193
StH	18	60972901	60973200	0.266	0.397	0.633	0.304
StH	18	60999901	61000200	1.750	1.810	3.594	1.781
StH	18	61024801	61025100	4.104	5.988	5.036	6.627
StH	18	61393201	61393500	0.345	0.818	0.555	0.664
StH	18	61566301	61566600	0.398	0.772	0.584	0.695
StH	18	62216701	62217000	2.419	2.887	7.330	3.236
StH	18	62258701	62259000	0.105	0.159	0.580	0.051
StH	18	62335201	62335500	0.771	0.936	0.424	0.776
StH	18	62847301	62847600	0.263	0.483	1.090	0.112
StH	18	62848801	62849100	0.339	0.317	2.583	0.274
StH	18	62908201	62908500	1.171	1.030	0.847	0.639
StH	18	62958301	62958600	1.197	0.796	1.353	0.907
StH	18	63073801	63074100	1.149	0.980	2.519	1.116
StH	18	63181801	63182100	0.393	0.112	2.171	0.279
StH	18	63182101	63182400	0.369	0.367	2.780	0.243
StH	18	63213601	63213900	4.359	4.106	3.201	3.985
StH	18	63429601	63429900	0.560	0.489	0.210	0.446
StH	18	63711301	63711600	3.749	3.532	2.131	3.554
StH	18	63746701	63747000	0.611	0.897	2.007	0.396

StH	18	63752701	63753000	0.452	0.701	2.445	0.421
StH	18	63870001	63870300	0.218	0.392	1.070	0.274
StH	18	63870301	63870600	0.557	0.549	1.864	0.452
StH	18	63888601	63888900	1.487	1.491	0.361	2.354
StH	18	63906301	63906600	1.412	0.790	2.421	1.319
StH	18	63929401	63929700	0.745	0.446	1.061	0.628
StH	18	63948001	63948300	2.765	2.944	1.757	2.800
StH	18	63951901	63952200	2.015	2.096	6.278	2.028
StH	18	63952201	63952500	2.041	2.212	3.690	1.601
StH	18	63954601	63954900	0.537	0.598	1.576	0.695
StH	18	63994201	63994500	1.597	2.122	1.230	1.633
StH	18	64029901	64030200	0.745	1.364	4.378	0.508
StH	18	64089601	64089900	0.132	0.362	1.458	0.117
StH	18	64155901	64156200	0.186	0.272	1.259	0.405
StH	18	64156501	64156800	1.958	1.613	3.019	1.172
StH	18	64324801	64325100	1.412	1.124	2.000	1.308
StH	18	64422601	64422900	3.426	3.523	5.239	3.650
StH	18	64436701	64437000	1.200	1.590	2.390	1.395
StH	18	64930201	64930500	0.455	0.584	1.052	0.558
StH	18	65506801	65507100	0.450	0.343	1.654	0.173
StH	18	65507101	65507400	0.582	0.813	2.216	0.446
StH	18	65627701	65628000	0.800	0.773	1.090	0.806
StH	18	65964601	65964900	0.745	0.676	0.506	0.745
StH	18	66309601	66309900	7.624	7.861	9.429	8.092
StH	18	66319801	66320100	0.932	1.832	2.727	1.273
StH	18	66367801	66368100	0.745	0.789	1.803	0.740
StH	18	66442801	66443100	0.900	0.725	0.370	0.771
StH	18	66590701	66591000	0.803	1.621	6.622	1.158
StH	18	66595201	66595500	1.949	1.871	0.794	1.791
StH	18	66625501	66625800	0.294	0.350	0.580	0.218
StH	18	66625801	66626100	0.026	0.047	0.588	0.218
StH	18	66645901	66646200	0.319	0.234	0.502	0.218
StH	18	66740101	66740400	0.986	1.032	0.288	0.745
StH	18	66780301	66780600	0.505	0.187	0.267	0.497
StH	18	66858901	66859200	0.055	0.157	2.216	0.076
StH	18	66859201	66859500	0.794	0.427	2.656	0.578
StH	18	66862201	66862500	1.671	1.125	4.822	1.410
StH	18	66862501	66862800	0.155	0.270	1.573	0.147
StH	18	66866101	66866400	0.269	0.180	0.967	0.198
StH	18	66885601	66885900	5.543	5.172	1.173	6.565
StH	18	66896401	66896700	1.589	2.177	3.756	1.369
StH	18	66904201	66904500	0.794	2.143	5.557	1.430
StH	18	66910201	66910500	0.960	1.082	1.893	0.745
StH	18	66910501	66910800	2.332	2.399	3.799	1.573
StH	18	66911701	66912000	0.271	0.373	1.226	0.295
StH	18	66916801	66917100	0.211	0.167	0.531	0.223
StH	18	66936301	66936600	0.371	1.117	3.152	0.609
StH	18	66939901	66940200	0.053	0.399	1.137	0.365
StH	18	66942601	66942900	0.266	0.303	0.564	0.223
StH	18	67024801	67025100	1.463	1.181	5.923	0.888
StH	18	67025101	67025400	1.302	1.394	3.962	0.821
StH	18	67039801	67040100	0.611	0.657	0.156	0.578
StH	18	67217101	67217400	0.850	0.845	0.457	0.771
StH	18	67235101	67235400	1.516	1.452	0.872	1.273
StH	18	67245901	67246200	0.800	0.747	1.509	0.751
StH	18	67277701	67278000	0.053	0.045	0.506	0.076
StH	18	67312501	67312800	0.534	0.765	5.569	0.684
StH	18	67314601	67314900	0.053	0.112	0.658	0.000
StH	18	67400101	67400400	1.300	1.175	2.806	1.314
StH	18	67400401	67400700	0.134	0.362	1.230	0.248
StH	18	67482001	67482300	0.000	0.047	0.448	0.031
StH	18	67482301	67482600	0.081	0.047	0.526	0.122
StH	18	67510801	67511100	1.063	0.755	2.577	0.842
StH	18	67640101	67640400	3.751	2.808	6.843	4.797
StH	18	67716301	67716600	0.292	0.232	0.078	0.466
StH	18	67740601	67740900	3.728	2.325	7.777	3.836
StH	18	67742401	67742700	3.983	5.767	2.329	3.899

StH	18	67761901	67762200	3.193	2.086	4.068	2.850
StH	18	67848001	67848300	1.649	2.092	3.181	1.587
StH	18	67868401	67868700	2.210	2.579	4.803	2.803
StH	18	67877101	67877400	0.077	0.202	1.012	0.051
StH	18	67879801	67880100	0.132	0.478	2.507	0.442
StH	18	67888201	67888500	0.507	0.675	4.803	0.660
StH	18	67890601	67890900	0.000	0.000	0.555	0.112
StH	18	67893601	67893900	2.738	2.373	5.147	3.637
StH	18	67908601	67908900	0.211	0.476	2.555	0.178
StH	18	67927201	67927500	1.255	2.281	5.187	1.506
StH	18	67995001	67995300	1.250	1.828	0.613	1.207
StH	18	68030401	68030700	1.171	0.714	2.073	0.628
StH	18	68056201	68056500	0.558	0.607	2.526	0.684
StH	18	68058901	68059200	0.131	0.585	2.899	0.203
StH	18	68206501	68206800	0.137	0.202	1.812	0.243
StH	18	68210101	68210400	0.400	0.678	2.720	0.564
StH	18	68247901	68248200	0.319	0.855	4.079	0.329
StH	18	68252701	68253000	0.512	0.778	2.880	0.380
StH	18	68272501	68272800	0.369	0.281	0.765	0.391
StH	18	68284501	68284800	0.687	1.006	4.577	0.948
StH	18	68307601	68307900	4.602	4.024	6.092	4.664
StH	18	68353501	68353800	0.215	0.722	3.449	0.696
StH	18	68353801	68354100	1.213	0.884	3.234	1.212
StH	18	68355601	68355900	0.079	0.045	1.414	0.051
StH	18	68399401	68399700	0.498	0.618	2.701	0.690
StH	18	68418901	68419200	0.340	1.305	6.602	0.473
StH	18	68419201	68419500	0.582	0.967	4.899	0.406
StH	18	68440201	68440500	0.182	0.431	1.444	0.132
StH	19	198301	198600	0.453	0.656	0.243	0.446
StH	19	200101	200400	0.611	0.701	0.292	0.603
StH	19	728701	729000	0.319	0.348	0.580	0.218
StH	19	934501	934800	0.081	0.187	0.531	0.117
StH	19	1026601	1026900	0.265	0.142	0.662	0.324
StH	19	1150201	1150500	1.408	1.049	1.723	0.963
StH	19	1206301	1206600	0.134	0.191	0.667	0.238
StH	19	1237801	1238100	0.536	0.796	1.255	0.598
StH	19	1238101	1238400	0.242	0.373	0.687	0.243
StH	19	2364901	2365200	0.771	0.867	0.346	0.558
StH	19	2672701	2673000	0.452	0.678	1.164	0.477
StH	19	3561001	3561300	0.265	0.375	0.691	0.446
StH	19	3758101	3758400	0.507	0.607	0.165	0.669
StH	19	4422601	4422900	0.242	0.047	0.531	0.117
StH	19	5074201	5074500	1.144	0.772	0.486	0.720
StH	19	5109001	5109300	2.021	2.013	1.411	1.688
StH	19	5209801	5210100	0.160	0.161	0.473	0.193
StH	19	5338201	5338500	0.134	0.116	0.760	0.304
StH	19	5365501	5365800	0.265	0.189	0.502	0.223
StH	19	5424601	5424900	0.637	0.375	0.140	0.360
StH	19	5465401	5465700	0.453	0.354	0.852	0.213
StH	19	5540101	5540400	0.316	0.466	0.938	0.340
StH	19	5543101	5543400	0.292	0.116	0.580	0.092
StH	19	5546401	5546700	0.213	0.468	0.078	0.355
StH	19	5568601	5568900	0.428	0.397	0.843	0.405
StH	19	5758801	5759100	0.637	0.933	0.370	0.603
StH	19	5793301	5793600	0.184	0.167	0.653	0.193
StH	19	5876401	5876700	0.211	0.586	0.395	0.193
StH	19	5908801	5909100	3.294	3.600	2.265	3.256
StH	19	6057601	6057900	0.052	1.214	0.753	0.193
StH	19	6117601	6117900	5.551	3.154	3.953	4.630
StH	19	6286201	6286500	0.979	0.972	2.321	1.338
StH	19	6499201	6499500	0.213	0.230	0.724	0.299
StH	19	6499501	6499800	0.773	0.326	1.745	1.014
StH	19	6582301	6582600	0.690	0.886	1.275	0.375
StH	19	6709501	6709800	0.586	0.560	0.341	0.639
StH	19	6765001	6765300	0.295	0.425	0.827	0.335
StH	19	6776401	6776700	1.563	1.485	3.433	1.496
StH	19	6879601	6879900	0.398	0.165	0.560	0.137

StH	19	7327501	7327800	0.532	0.607	0.243	0.720
StH	19	7510801	7511100	0.584	0.536	0.243	0.558
StH	19	7587001	7587300	0.261	0.303	0.963	0.396
StH	19	7811401	7811700	3.957	4.834	2.920	3.984
StH	19	8129401	8129700	0.371	0.378	0.843	0.228
StH	19	8141401	8141700	0.531	0.539	0.267	0.497
StH	19	9315301	9315600	0.105	0.069	0.831	0.258
StH	19	9669301	9669600	3.097	2.999	1.251	2.748
StH	19	10086001	10086300	0.481	0.725	0.214	0.639
StH	19	10416301	10416600	2.851	3.180	2.283	3.245
StH	19	10577401	10577700	1.439	1.403	0.709	1.557
StH	19	10608001	10608300	0.531	0.678	0.239	0.756
StH	19	10904401	10904700	0.452	0.397	0.584	0.329
StH	19	11487001	11487300	1.647	1.901	0.925	1.724
StH	19	11499301	11499600	3.455	3.752	5.479	4.548
StH	19	11532601	11532900	0.558	0.772	0.205	0.583
StH	19	11813701	11814000	2.075	2.105	1.830	2.794
StH	19	11961901	11962200	0.507	0.607	1.012	0.740
StH	19	12047101	12047400	6.033	4.864	8.037	7.184
StH	19	12173401	12173700	0.240	0.234	0.535	0.076
StH	19	12490201	12490500	0.481	0.446	0.214	0.025
StH	19	12518701	12519000	3.881	3.319	2.542	3.599
StH	19	13012501	13012800	0.586	0.281	0.453	0.664
StH	19	13350901	13351200	0.424	0.279	0.716	0.396
StH	19	13442701	13443000	0.452	0.631	0.321	0.441
StH	19	13534801	13535100	0.210	0.189	0.740	0.198
StH	19	13539001	13539300	0.611	0.751	1.037	0.452
StH	19	13638601	13638900	0.160	0.114	1.226	0.254
StH	19	13643101	13643400	0.534	0.631	0.214	0.502
StH	19	13646401	13646700	0.878	0.678	0.584	0.776
StH	19	14044501	14044800	0.558	0.629	0.239	0.492
StH	19	14337301	14337600	1.326	3.755	2.796	3.874
StH	19	14337601	14337900	0.345	1.873	0.798	2.083
StH	19	14467801	14468100	1.274	0.317	5.567	2.229
StH	19	14468101	14468400	2.662	1.076	8.063	4.037
StH	19	14912701	14913000	1.865	1.637	0.769	1.794
StH	19	14918701	14919000	2.486	3.931	1.137	3.159
StH	19	14955601	14955900	0.828	0.832	1.826	0.608
StH	19	15012001	15012300	1.223	1.197	0.691	1.480
StH	19	15105601	15105900	0.239	1.244	0.682	1.131
StH	19	15116101	15116400	0.374	0.380	1.173	0.578
StH	19	15261601	15261900	0.189	0.300	0.814	1.617
StH	19	15357001	15357300	0.345	0.279	0.477	0.274
StH	19	15387001	15387300	0.797	0.701	0.477	0.669
StH	19	15631201	15631500	0.213	0.161	0.564	0.101
StH	19	15640801	15641100	2.263	2.074	1.415	2.626
StH	19	15641101	15641400	1.805	1.824	1.440	2.205
StH	19	15656701	15657000	1.037	0.560	0.852	0.715
StH	19	15778201	15778500	0.769	0.723	0.267	1.014
StH	19	15824701	15825000	2.041	1.255	3.262	1.826
StH	19	15865501	15865800	0.479	0.468	0.136	0.248
StH	19	16047001	16047300	0.290	0.140	0.477	0.025
StH	19	16551301	16551600	2.204	2.873	1.045	1.860
StH	19	16601101	16601400	1.163	1.167	0.560	1.440
StH	19	17122801	17123100	0.531	0.539	0.189	0.542
StH	19	17399401	17399700	0.477	0.352	0.107	0.385
StH	19	17561101	17561400	0.398	0.444	0.132	0.477
StH	19	17708701	17709000	4.044	4.559	2.905	4.417
StH	19	17721001	17721300	2.291	2.600	1.596	2.074
StH	19	17756401	17756700	1.568	1.148	0.720	1.014
StH	19	17790001	17790300	0.905	1.195	0.745	1.105
StH	19	18557701	18558000	0.294	0.165	0.856	0.664
StH	19	18897901	18898200	0.747	0.375	0.082	0.472
StH	19	20025301	20025600	0.477	0.448	0.185	0.553
StH	19	20577301	20577600	1.439	1.757	2.341	1.582
StH	19	20585701	20586000	0.616	0.283	1.119	0.452
StH	19	21270601	21270900	2.768	3.133	2.091	2.733

StH	19	21515401	21515700	3.514	3.802	2.332	3.489
StH	19	21638101	21638400	1.037	0.725	0.428	0.690
StH	19	22577401	22577700	0.371	0.421	0.210	0.522
StH	19	23943001	23943300	1.516	1.335	0.633	1.055
StH	19	25416001	25416300	0.560	0.446	0.111	0.329
StH	19	27392701	27393000	0.560	0.981	0.370	1.166
StH	19	27393001	27393300	0.637	0.935	0.341	0.852
StH	19	27710101	27710400	0.186	0.210	0.531	0.228
StH	19	28314001	28314300	1.357	1.541	1.016	0.360
StH	19	28402801	28403100	0.397	0.607	0.214	0.553
StH	19	28578901	28579200	0.586	0.442	0.317	0.801
StH	19	28633201	28633500	0.824	0.721	0.292	0.832
StH	19	28807801	28808100	0.342	0.399	1.337	0.821
StH	19	28808101	28808400	0.366	0.594	2.256	0.730
StH	19	29072701	29073000	0.663	0.890	1.217	0.517
StH	19	29236801	29237100	0.081	0.069	0.419	0.051
StH	19	29429701	29430000	0.266	0.234	0.477	0.198
StH	19	30597001	30597300	0.932	1.470	0.613	0.893
StH	19	31810201	31810500	0.558	0.470	0.243	0.639
StH	19	32019601	32019900	0.903	0.444	0.695	0.988
StH	19	32093701	32094000	2.976	2.811	1.946	2.880
StH	19	32530501	32530800	0.347	0.234	0.671	0.441
StH	19	32906101	32906400	0.536	0.834	2.349	0.863
StH	19	32928001	32928300	0.158	0.835	1.728	0.579
StH	19	33171301	33171600	0.426	0.609	0.165	0.335
StH	19	33206701	33207000	0.266	0.466	0.054	0.162
StH	19	33268501	33268800	0.395	0.792	0.054	0.771
StH	19	33693001	33693300	0.532	0.562	0.136	0.731
StH	19	33855001	33855300	0.158	0.236	0.506	0.187
StH	19	34152301	34152600	0.400	0.539	0.189	0.553
StH	19	34372501	34372800	0.266	0.232	0.506	0.254
StH	19	35056501	35056800	1.516	1.708	1.119	1.359
StH	19	35064601	35064900	0.878	0.562	0.745	0.614
StH	19	35490901	35491200	0.161	0.255	0.535	0.198
StH	19	35599201	35599500	0.721	0.845	0.087	0.578
StH	19	35603101	35603400	0.266	0.283	0.560	0.223
StH	19	35669701	35670000	0.878	1.120	1.456	1.247
StH	19	36028201	36028500	3.234	3.190	6.076	3.193
StH	19	36173701	36174000	1.492	1.659	2.114	1.572
StH	19	36769501	36769800	0.319	0.863	0.584	0.380
StH	19	37636201	37636500	0.852	0.959	0.617	0.832
StH	19	38256001	38256300	0.210	0.328	0.749	0.294
StH	19	38370601	38370900	0.160	0.094	0.448	0.223
StH	19	38388601	38388900	0.079	0.283	0.477	0.137
StH	19	38428201	38428500	0.826	0.680	0.321	0.796
StH	19	38833201	38833500	0.426	0.584	0.317	0.248
StH	19	39127501	39127800	0.558	0.397	0.453	0.360
StH	19	39260701	39261000	2.131	3.420	0.763	2.550
StH	19	39261001	39261300	1.631	2.646	0.809	2.104
StH	19	39442801	39443100	0.481	0.491	0.103	0.421
StH	19	39845101	39845400	0.268	0.047	0.963	0.335
StH	19	39845401	39845700	0.266	0.047	0.720	0.279
StH	19	40014301	40014600	0.079	0.000	0.448	0.081
StH	19	40538101	40538400	0.479	0.491	0.185	0.441
StH	19	41738401	41738700	1.010	0.751	0.301	0.918
StH	19	41740201	41740500	0.771	1.028	0.502	0.801
StH	19	41996701	41997000	0.481	0.348	0.794	0.112
StH	19	42274501	42274800	2.210	2.203	1.679	1.080
StH	19	43081201	43081500	0.931	1.186	0.205	1.096
StH	19	43223701	43224000	1.355	1.641	0.979	1.328
StH	19	43277401	43277700	0.235	0.227	0.925	0.173
StH	19	43408501	43408800	0.319	0.444	0.132	0.365
StH	19	43769401	43769700	0.850	0.770	0.473	0.751
StH	19	44165401	44165700	0.878	0.350	1.201	0.735
StH	19	44211901	44212200	0.426	0.583	0.189	0.360
StH	19	44851501	44851800	4.018	2.898	5.301	4.142
StH	19	45321301	45321600	1.836	0.657	0.223	1.091

StH	19	45398401	45398700	2.365	2.242	1.806	2.748
StH	19	45510301	45510600	0.349	0.348	0.638	0.279
StH	19	45841801	45842100	1.805	0.215	1.110	0.614
StH	19	46433401	46433700	0.426	0.420	0.078	0.360
StH	19	46785601	46785900	0.347	0.279	0.691	0.466
StH	19	46796701	46797000	0.294	0.468	0.687	0.254
StH	19	46920901	46921200	1.810	1.862	1.052	2.079
StH	19	47073901	47074200	4.202	4.885	3.961	3.752
StH	19	47114101	47114400	1.379	1.568	0.350	1.267
StH	19	47159701	47160000	0.477	0.691	1.609	0.340
StH	19	48127801	48128100	2.183	1.403	1.641	1.354
StH	19	48301801	48302100	0.771	0.702	1.057	0.801
StH	19	48343201	48343500	1.741	1.997	3.565	1.932
StH	19	48506701	48507000	1.089	1.549	3.725	1.030
StH	19	48756001	48756300	1.116	1.264	0.667	1.111
StH	19	48793501	48793800	0.402	0.257	0.876	0.639
StH	19	49382401	49382700	0.426	0.586	0.185	0.385
StH	19	49578901	49579200	0.902	0.863	1.222	0.751
StH	19	49685401	49685700	0.239	0.607	0.132	0.248
StH	19	49812301	49812600	0.318	0.538	0.103	0.416
StH	19	49830001	49830300	0.292	0.326	0.638	0.279
StH	19	50029501	50029800	0.981	1.313	2.008	0.999
StH	19	50166601	50166900	1.566	1.637	1.148	1.491
StH	19	50830801	50831100	0.879	1.122	2.858	0.487
StH	19	50838901	50839200	0.419	0.661	1.835	0.634
StH	19	51174301	51174600	1.333	1.664	4.939	1.009
StH	19	51255001	51255300	2.152	1.637	1.962	1.577
StH	19	51304501	51304800	3.297	3.201	2.789	3.271
StH	19	51312601	51312900	3.246	2.776	2.135	2.758
StH	19	51319501	51319800	0.376	0.139	0.769	0.340
StH	19	51335701	51336000	1.621	1.708	0.827	1.339
StH	19	51395401	51395700	0.237	1.042	3.282	0.513
StH	19	52035601	52035900	0.106	0.515	0.317	0.441
StH	19	52276201	52276500	2.818	2.974	2.497	3.509
StH	19	52365901	52366200	3.771	3.416	2.740	3.726
StH	19	52561801	52562100	2.497	2.199	1.295	2.281
StH	19	52639201	52639500	2.381	2.644	6.980	2.298
StH	19	52695601	52695900	0.423	0.137	0.769	0.274
StH	19	52714201	52714500	1.328	1.266	0.428	0.751
StH	19	52804201	52804500	0.082	0.023	1.077	0.086
StH	19	52804501	52804800	0.000	0.120	1.106	0.056
StH	19	52821001	52821300	2.686	3.160	1.946	3.351
StH	19	52902901	52903200	0.690	0.661	0.210	0.771
StH	19	53109301	53109600	1.413	1.663	2.069	1.552
StH	19	53167501	53167800	3.118	3.158	3.805	4.513
StH	19	53241601	53241900	0.479	0.538	0.317	0.446
StH	19	53270401	53270700	2.289	2.392	1.201	2.014
StH	19	53278201	53278500	0.928	1.564	2.341	1.328
StH	19	53617501	53617800	2.100	1.706	1.494	0.974
StH	19	53745301	53745600	3.138	3.064	4.387	3.239
StH	19	54268501	54268800	1.305	1.056	0.457	0.999
StH	19	54292501	54292800	0.000	0.112	0.702	0.086
StH	19	54367201	54367500	0.369	0.444	0.054	0.558
StH	19	54420601	54420900	0.878	0.515	0.745	0.329
StH	19	54445801	54446100	1.439	1.306	0.588	1.226
StH	19	54653401	54653700	1.516	1.796	0.930	1.612
StH	19	54683101	54683400	0.785	1.083	3.347	0.747
StH	19	54719401	54719700	0.587	0.493	0.074	0.472
StH	19	54724801	54725100	0.055	0.096	0.678	0.167
StH	19	54725101	54725400	0.053	0.163	0.638	0.234
StH	19	54740701	54741000	3.964	3.364	4.648	2.961
StH	19	54889501	54889800	0.373	0.144	1.023	0.345
StH	19	55090501	55090800	0.481	0.539	0.185	0.943
StH	19	55090801	55091100	0.716	0.798	0.395	1.019
StH	19	55156801	55157100	1.650	2.102	1.074	1.536
StH	19	55242001	55242300	3.507	2.251	1.313	2.124
StH	19	55262401	55262700	2.746	3.133	1.828	3.149

StH	19	55330801	55331100	0.768	0.818	0.453	0.938
StH	19	55336201	55336500	2.871	2.289	1.237	2.161
StH	19	55340701	55341000	1.170	1.221	0.526	0.974
StH	19	55429501	55429800	3.381	2.289	1.855	3.149
StH	19	55563301	55563600	0.269	0.182	0.778	0.223
StH	19	55572001	55572300	0.718	0.702	0.243	0.385
StH	19	55824601	55824900	1.597	1.478	0.943	1.521
StH	19	55883401	55883700	0.239	0.493	0.054	0.365
StH	19	55987201	55987500	1.194	1.332	1.815	1.323
StH	19	56140201	56140500	0.534	0.916	0.098	0.497
StH	19	56216401	56216700	1.020	0.852	2.617	0.648
StH	19	56301301	56301600	0.665	0.768	0.189	0.669
StH	19	56378701	56379000	0.373	0.350	0.613	0.472
StH	19	56417701	56418000	4.975	5.078	3.625	5.180
StH	19	56538301	56538600	0.907	0.936	0.613	0.913
StH	19	56640901	56641200	4.436	4.806	3.027	4.620
StH	19	57108901	57109200	4.233	3.540	1.801	3.482
StH	19	57213301	57213600	0.558	1.070	1.686	0.553
StH	19	57589801	57590100	1.111	1.482	2.599	1.065
StH	19	57596401	57596700	1.968	2.343	1.382	2.119
StH	19	57634801	57635100	4.223	4.325	3.185	4.660
StH	19	57635401	57635700	0.479	0.094	0.346	0.558
StH	19	57888001	57888300	4.567	3.862	2.854	4.270
StH	19	57993301	57993600	0.637	0.749	1.284	0.974
StH	19	58011301	58011600	3.375	3.193	4.994	3.525
StH	19	58114801	58115100	1.300	1.452	4.667	1.511
StH	19	58135201	58135500	2.420	2.010	2.225	2.850
StH	19	58162801	58163100	2.104	1.491	3.233	2.286
StH	19	58166401	58166700	0.742	0.322	1.580	0.659
StH	19	58181101	58181400	0.242	0.204	0.963	0.411
StH	19	58181401	58181700	1.032	1.539	5.549	2.192
StH	19	58182601	58182900	0.457	0.376	0.974	0.472
StH	19	58185601	58185900	0.510	0.851	2.845	0.801
StH	19	58194301	58194600	0.292	0.206	1.402	0.375
StH	19	58194601	58194900	0.958	1.777	2.303	0.978
StH	19	58473601	58473900	3.236	3.107	5.486	3.432
StH	19	58475101	58475400	1.652	1.616	0.707	2.018
StH	19	58592101	58592400	0.260	0.540	3.392	0.757
StH	19	58592401	58592700	0.268	0.135	1.032	0.289
StH	19	58683301	58683600	0.134	0.431	1.547	0.457
StH	19	58758301	58758600	1.144	1.105	2.805	1.029
StH	19	58802401	58802700	1.036	1.006	0.555	0.887
StH	19	58824601	58824900	0.711	0.528	2.300	0.669
StH	19	58829701	58830000	0.637	0.721	1.440	0.649
StH	19	58900501	58900800	0.397	0.534	1.398	0.432
StH	19	59097901	59098200	0.637	1.206	2.575	1.081
StH	19	59127001	59127300	2.341	2.379	1.777	2.951
StH	19	59152501	59152800	1.353	1.191	0.736	1.379
StH	19	59190901	59191200	0.053	0.519	1.946	0.523
StH	19	59204401	59204700	0.589	0.708	2.200	0.710
StH	19	59314501	59314800	2.260	2.736	1.601	2.652
StH	19	59322901	59323200	0.158	0.225	1.152	0.365
StH	19	59356201	59356500	0.110	0.661	2.770	0.227
StH	19	59501401	59501700	0.000	0.047	0.646	0.076
StH	19	59597701	59598000	8.724	8.520	7.147	9.239
StH	19	59616001	59616300	0.189	0.476	1.906	0.340
StH	19	59676301	59676600	0.027	0.592	2.211	0.147
StH	19	59711101	59711400	0.640	0.908	1.638	0.690
StH	19	59741101	59741400	1.113	1.283	3.932	0.979
StH	19	59742601	59742900	0.744	1.237	5.128	0.806
StH	19	59742901	59743200	0.594	1.733	4.845	0.918
StH	19	59778001	59778300	1.382	1.077	0.629	1.379
StH	19	59781001	59781300	1.339	1.994	6.116	1.832
StH	19	59796001	59796300	9.134	9.513	7.787	9.116
StH	19	59807401	59807700	0.684	0.888	3.363	0.401
StH	19	59840401	59840700	3.036	3.459	2.374	3.083
StH	19	60132601	60132900	0.611	1.126	4.734	0.751

StH	19	60170101	60170400	0.079	0.588	2.186	0.056
StH	19	60235801	60236100	1.728	1.783	0.905	1.997
StH	19	60243901	60244200	0.400	0.395	0.720	0.076
StH	19	60245701	60246000	0.952	1.763	3.054	1.075
StH	19	60246001	60246300	1.318	2.203	6.252	1.137
StH	19	60248701	60249000	0.053	0.069	0.622	0.000
StH	19	60286801	60287100	0.486	2.262	7.254	0.913
StH	19	60354001	60354300	3.828	3.785	3.418	2.545
StH	19	60381001	60381300	0.079	0.630	2.263	0.112
StH	2	9601	9900	0.690	0.536	0.876	0.472
StH	2	19501	19800	0.453	0.564	0.239	0.527
StH	2	195901	196200	0.773	0.322	1.614	0.675
StH	2	204301	204600	2.292	1.512	5.989	2.661
StH	2	291001	291300	3.378	3.435	2.267	4.359
StH	2	332701	333000	1.326	1.433	0.745	1.303
StH	2	356101	356400	0.720	0.785	2.541	0.624
StH	2	393301	393600	2.578	1.513	2.089	1.633
StH	2	425101	425400	0.586	0.454	2.124	0.309
StH	2	425401	425700	0.931	1.811	2.735	1.126
StH	2	477601	477900	0.432	0.204	1.340	0.411
StH	2	508501	508800	0.344	0.399	0.087	0.664
StH	2	564901	565200	9.711	11.161	8.946	12.970
StH	2	668101	668400	5.498	5.312	7.062	5.370
StH	2	714001	714300	0.532	0.862	1.387	0.690
StH	2	794101	794400	0.371	0.227	2.231	0.203
StH	2	846301	846600	0.507	0.584	0.872	0.608
StH	2	857101	857400	0.632	0.815	2.153	0.493
StH	2	913801	914100	0.724	0.781	2.582	1.065
StH	2	914101	914400	0.134	0.167	0.658	0.173
StH	2	974701	975000	0.675	0.851	2.857	0.826
StH	2	983401	983700	0.263	0.258	0.613	0.117
StH	2	1095901	1096200	0.582	0.369	1.337	0.299
StH	2	1138501	1138800	1.908	2.740	4.193	2.617
StH	2	1177501	1177800	1.896	1.940	2.916	1.997
StH	2	1179001	1179300	3.244	2.098	2.798	3.681
StH	2	1291501	1291800	0.186	0.118	0.564	0.162
StH	2	1298401	1298700	4.386	4.087	4.911	4.279
StH	2	1380001	1380300	1.923	1.765	4.848	1.592
StH	2	1407601	1407900	0.563	0.365	1.251	0.497
StH	2	1537801	1538100	0.452	0.395	1.239	0.547
StH	2	1602001	1602300	3.346	3.937	5.858	3.696
StH	2	1671901	1672200	4.951	3.032	3.763	4.771
StH	2	1692601	1692900	0.769	0.658	4.379	0.542
StH	2	1694701	1695000	0.897	0.795	3.442	0.760
StH	2	1695001	1695300	1.292	1.411	4.831	1.258
StH	2	1718701	1719000	0.905	0.905	3.702	0.649
StH	2	1777801	1778100	2.952	3.333	5.745	2.881
StH	2	1876801	1877100	0.986	1.006	0.366	1.201
StH	2	1914001	1914300	6.785	7.071	5.232	6.780
StH	2	1922101	1922400	0.027	0.068	0.419	0.031
StH	2	1928401	1928700	2.150	2.390	1.349	2.003
StH	2	2241001	2241300	0.902	0.863	0.502	1.105
StH	2	2314501	2314800	1.486	1.478	0.439	1.177
StH	2	2785801	2786100	0.242	0.232	0.560	0.198
StH	2	2786101	2786400	0.486	0.438	1.424	0.452
StH	2	2970301	2970600	1.899	2.346	6.621	2.103
StH	2	3324901	3325200	1.244	0.527	2.877	1.287
StH	2	3371101	3371400	0.374	0.609	0.132	0.522
StH	2	3383701	3384000	1.749	2.392	0.591	1.927
StH	2	3384001	3384300	0.879	0.562	0.267	0.416
StH	2	3455101	3455400	0.292	0.212	0.448	0.112
StH	2	3591001	3591300	0.557	0.558	0.210	0.700
StH	2	3692701	3693000	0.982	1.332	0.740	1.267
StH	2	3775501	3775800	0.637	0.586	0.169	0.573
StH	2	3859201	3859500	0.321	0.373	0.774	0.396
StH	2	4065901	4066200	0.879	1.079	0.424	0.745
StH	2	4200901	4201200	1.966	2.407	2.122	2.292

StH	2	4270801	4271100	4.510	4.061	6.126	3.981
StH	2	4428001	4428300	0.821	0.511	1.456	0.705
StH	2	4536301	4536600	0.424	0.584	0.847	0.522
StH	2	5709601	5709900	1.594	1.212	1.888	1.303
StH	2	6066301	6066600	0.156	0.330	0.914	0.350
StH	2	6408901	6409200	0.532	0.564	0.214	0.477
StH	2	6484801	6485100	0.637	0.654	0.181	0.644
StH	2	6606901	6607200	0.211	0.421	0.560	0.112
StH	2	6755101	6755400	1.245	1.472	0.716	1.343
StH	2	6880801	6881100	0.715	0.491	0.127	0.558
StH	2	7126501	7126800	7.505	8.456	5.968	7.691
StH	2	7197901	7198200	1.812	2.716	1.061	2.083
StH	2	7900801	7901100	0.158	0.140	0.419	0.081
StH	2	8514001	8514300	2.499	0.880	3.179	2.231
StH	2	8551201	8551500	0.534	0.444	0.905	0.588
StH	2	8645101	8645400	0.079	0.157	0.905	0.203
StH	2	9120601	9120900	1.279	1.824	1.538	1.800
StH	2	9215701	9216000	0.266	0.189	0.399	0.527
StH	2	9253501	9253800	0.558	0.586	0.909	0.466
StH	2	9260401	9260700	6.222	5.518	4.846	5.764
StH	2	9341701	9342000	0.900	1.000	1.761	0.781
StH	2	9447901	9448200	0.740	0.393	1.222	0.826
StH	2	9670801	9671100	2.688	2.407	1.768	3.321
StH	2	9747301	9747600	2.586	1.616	0.840	1.922
StH	2	9900001	9900300	1.542	1.523	0.901	1.485
StH	2	9913501	9913800	0.663	0.912	1.616	1.172
StH	2	10476601	10476900	0.503	0.631	0.078	0.558
StH	2	11004601	11004900	0.292	0.560	0.107	0.360
StH	2	11461801	11462100	0.477	0.633	0.185	0.664
StH	2	11956801	11957100	0.266	0.395	0.716	0.360
StH	2	12826801	12827100	0.239	0.210	1.382	0.614
StH	2	13270801	13271100	0.053	0.000	0.564	0.000
StH	2	13371001	13371300	0.773	0.656	0.954	0.411
StH	2	13756801	13757100	1.862	1.684	2.575	1.262
StH	2	14151301	14151600	0.532	0.770	0.341	0.695
StH	2	14781601	14781900	0.132	0.279	0.555	0.228
StH	2	15091501	15091800	0.531	0.680	0.107	0.497
StH	2	15609301	15609600	0.452	0.515	0.317	0.441
StH	2	15664801	15665100	3.801	4.044	5.364	4.735
StH	2	15751201	15751500	0.477	0.538	0.974	0.634
StH	2	15751501	15751800	0.529	0.704	1.159	0.628
StH	2	15785401	15785700	0.160	0.187	0.448	0.248
StH	2	16096801	16097100	0.876	1.075	0.560	1.166
StH	2	16133701	16134000	0.907	1.009	0.444	0.887
StH	2	16928701	16929000	0.852	0.747	1.037	0.547
StH	2	17181601	17181900	0.161	0.140	0.486	0.132
StH	2	17418901	17419200	1.462	1.777	1.168	1.298
StH	2	17794801	17795100	0.424	0.399	0.695	0.411
StH	2	17807401	17807700	0.689	0.517	0.132	0.492
StH	2	18153301	18153600	3.131	2.826	2.020	3.002
StH	2	18591301	18591600	0.213	0.187	0.448	0.137
StH	2	18907201	18907500	0.290	0.420	0.054	0.522
StH	2	20600701	20601000	0.452	0.326	0.638	0.751
StH	2	22662601	22662900	0.452	0.493	0.234	0.771
StH	2	22971601	22971900	0.716	0.749	0.341	0.796
StH	2	23104201	23104500	2.313	2.465	0.918	1.946
StH	2	23418601	23418900	0.452	0.465	0.638	0.994
StH	2	24033301	24033600	0.400	0.397	0.127	0.547
StH	2	24153001	24153300	0.345	0.375	0.617	0.193
StH	2	24393301	24393600	1.520	1.577	0.473	1.273
StH	2	24723301	24723600	0.560	0.326	0.103	0.416
StH	2	24775801	24776100	0.318	0.139	0.617	0.223
StH	2	24876901	24877200	0.319	0.185	0.526	0.329
StH	2	24963001	24963300	0.637	0.350	0.531	0.639
StH	2	25094101	25094400	0.663	0.867	0.301	0.806
StH	2	25806601	25806900	0.105	0.376	0.687	0.137
StH	2	26037901	26038200	0.479	0.420	0.214	0.421

StH	2	26128501	26128800	0.082	0.305	0.720	0.365
StH	2	26307001	26307300	1.199	1.216	1.543	1.131
StH	2	26683201	26683500	0.318	0.749	0.481	0.304
StH	2	26753401	26753700	0.884	1.073	1.694	0.948
StH	2	26847901	26848200	0.345	0.633	0.823	0.223
StH	2	27156301	27156600	0.532	0.420	0.181	0.690
StH	2	27203701	27204000	0.294	0.230	0.555	0.248
StH	2	27621601	27621900	1.410	1.307	0.925	1.430
StH	2	27855901	27856200	0.132	0.260	0.506	0.193
StH	2	27916801	27917100	0.319	0.444	0.239	0.360
StH	2	27928801	27929100	2.023	3.251	2.613	1.805
StH	2	27931801	27932100	0.132	0.187	0.239	0.608
StH	2	27961501	27961800	1.037	0.723	0.395	0.659
StH	2	28259101	28259400	2.267	2.433	1.460	2.464
StH	2	28344301	28344600	0.318	0.423	0.049	0.335
StH	2	28548601	28548900	0.344	0.302	0.720	0.365
StH	2	28759201	28759500	0.745	0.745	0.535	0.254
StH	2	29655001	29655300	1.116	1.148	0.473	1.070
StH	2	30332401	30332700	0.106	0.163	0.419	0.167
StH	2	31128301	31128600	0.902	1.004	0.383	1.399
StH	2	31304101	31304400	0.318	0.352	0.617	0.223
StH	2	31944301	31944600	0.823	0.820	0.243	0.517
StH	2	32391001	32391300	0.237	0.071	0.535	0.274
StH	2	32392501	32392800	0.453	0.538	0.346	0.553
StH	2	32562901	32563200	1.142	1.030	2.383	1.369
StH	2	32563201	32563500	0.424	0.723	0.852	0.664
StH	2	32854201	32854500	0.665	0.609	0.453	0.639
StH	2	33702001	33702300	0.369	0.369	0.885	0.385
StH	2	33702301	33702600	0.344	0.511	1.511	0.507
StH	2	34169401	34169700	0.244	0.230	0.794	0.254
StH	2	34350901	34351200	0.345	0.466	0.132	0.416
StH	2	34488301	34488600	0.694	0.747	1.057	0.522
StH	2	35665201	35665500	0.026	0.071	0.560	0.051
StH	2	36272401	36272700	0.531	0.517	0.925	0.461
StH	2	36690001	36690300	0.558	0.912	0.745	0.360
StH	2	37546501	37546800	0.265	0.118	0.588	0.385
StH	2	37784701	37785000	0.213	0.701	0.531	0.274
StH	2	37972801	37973100	0.636	0.517	0.156	0.547
StH	2	38902201	38902500	4.504	5.113	2.680	4.142
StH	2	39257101	39257400	0.345	0.560	0.662	0.497
StH	2	39295501	39295800	1.170	1.124	1.592	1.100
StH	2	39386701	39387000	0.213	0.163	0.473	0.167
StH	2	39567001	39567300	0.477	0.352	0.716	0.380
StH	2	39570001	39570300	0.532	0.421	0.136	0.365
StH	2	40064701	40065000	0.186	0.137	0.502	0.076
StH	2	40824601	40824900	2.918	1.011	1.978	0.882
StH	2	41113501	41113800	0.398	0.493	0.263	0.553
StH	2	41271901	41272200	1.274	1.474	0.633	1.363
StH	2	41817301	41817600	0.452	0.539	0.296	0.608
StH	2	41979001	41979300	0.079	0.491	0.321	0.081
StH	2	41987401	41987700	2.281	2.315	1.476	3.012
StH	2	42281701	42282000	0.684	1.225	2.443	1.064
StH	2	42318301	42318600	0.290	0.258	0.560	0.274
StH	2	42386401	42386700	1.089	1.847	0.370	1.313
StH	2	42455101	42455400	4.420	4.440	6.750	4.254
StH	2	42457501	42457800	2.181	1.948	1.061	1.719
StH	2	42543601	42543900	0.715	0.912	1.358	0.756
StH	2	43138501	43138800	0.847	0.577	1.485	0.745
StH	2	43592701	43593000	0.237	0.094	0.477	0.304
StH	2	43665601	43665900	0.132	0.140	0.419	0.162
StH	2	43802701	43803000	0.321	0.213	0.584	0.223
StH	2	44200501	44200800	1.284	1.195	2.114	1.222
StH	2	44421001	44421300	0.186	0.069	0.444	0.056
StH	2	44743501	44743800	0.639	0.910	0.481	0.720
StH	2	45275101	45275400	0.505	0.562	0.263	0.446
StH	2	45584701	45585000	0.265	0.187	0.477	0.218
StH	2	45706201	45706500	0.452	0.772	0.638	0.497

StH	2	46463701	46464000	0.106	0.213	0.510	0.218
StH	2	47094901	47095200	0.850	0.798	1.378	0.857
StH	2	47370001	47370300	3.954	4.424	3.114	4.664
StH	2	47419201	47419500	0.079	0.420	0.214	0.056
StH	2	47424301	47424600	2.252	2.320	0.916	3.098
StH	2	47678701	47679000	0.453	0.910	0.633	0.832
StH	2	47698501	47698800	1.120	1.450	0.617	1.709
StH	2	48464701	48465000	1.649	1.805	1.144	1.744
StH	2	48648601	48648900	0.289	0.305	0.695	0.106
StH	2	48733201	48733500	0.479	0.631	0.263	0.608
StH	2	48740101	48740400	0.211	0.397	0.506	0.360
StH	2	49068301	49068600	0.902	0.891	0.370	0.771
StH	2	49098301	49098600	1.039	1.099	0.609	1.105
StH	2	49152601	49152900	2.999	3.161	2.040	3.286
StH	2	49216801	49217100	0.986	0.985	0.560	0.968
StH	2	49320901	49321200	0.453	0.607	0.185	0.360
StH	2	49347301	49347600	0.505	0.562	0.370	0.527
StH	2	49513201	49513500	1.091	0.375	1.324	1.055
StH	2	49769101	49769400	0.532	0.470	0.950	0.324
StH	2	49917001	49917300	0.319	0.185	0.531	0.441
StH	2	51052801	51053100	0.428	0.399	0.716	0.284
StH	2	51515701	51516000	1.516	1.380	0.814	1.262
StH	2	51807901	51808200	0.718	0.631	0.374	0.274
StH	2	51993601	51993900	0.263	0.184	0.745	0.284
StH	2	52011301	52011600	0.079	0.187	0.473	0.162
StH	2	52029301	52029600	0.242	0.260	0.667	0.284
StH	2	52528801	52529100	1.939	2.298	1.291	1.906
StH	2	52544701	52545000	0.742	0.586	0.239	0.700
StH	2	52681201	52681500	1.378	1.729	4.206	1.253
StH	2	52832101	52832400	0.371	0.420	0.082	0.248
StH	2	53130301	53130600	0.611	0.468	0.720	0.527
StH	2	53250901	53251200	0.371	0.468	0.049	0.466
StH	2	53554201	53554500	0.773	0.727	0.403	0.826
StH	2	53671801	53672100	0.553	0.401	1.119	0.542
StH	2	53871001	53871300	0.161	0.137	0.506	0.051
StH	2	56280901	56281200	1.758	1.554	0.343	1.445
StH	2	56281201	56281500	1.912	1.835	0.495	1.623
StH	2	56625301	56625600	1.462	1.661	1.304	1.521
StH	2	58381201	58381500	0.799	1.028	0.609	0.385
StH	2	59398501	59398800	0.266	0.140	0.622	0.193
StH	2	59836201	59836500	0.294	0.161	0.502	0.193
StH	2	60025201	60025500	0.429	0.350	1.812	0.643
StH	2	62437801	62438100	0.769	0.867	1.226	0.882
StH	2	63051901	63052200	0.316	0.285	0.740	0.284
StH	2	63241201	63241500	0.586	0.397	0.267	0.411
StH	2	63626401	63626700	0.823	1.424	0.481	1.156
StH	2	65385001	65385300	0.824	1.658	0.510	1.014
StH	2	65527501	65527800	0.319	0.213	0.658	0.254
StH	2	65652301	65652600	0.795	0.749	0.337	0.887
StH	2	67840501	67840800	0.108	0.116	0.448	0.000
StH	2	68445901	68446200	0.560	0.727	0.185	0.360
StH	2	68481901	68482200	0.984	0.980	0.691	0.882
StH	2	68554201	68554500	2.174	2.053	0.954	1.546
StH	2	68893501	68893800	0.771	0.753	0.341	0.725
StH	2	69408601	69408900	0.108	0.212	0.448	0.142
StH	2	69559801	69560100	0.347	0.494	0.872	0.299
StH	2	70552801	70553100	0.893	2.303	7.922	1.060
StH	2	70965601	70965900	0.345	0.163	0.560	0.274
StH	2	71452501	71452800	0.716	0.843	0.321	0.639
StH	2	72108901	72109200	0.108	0.090	0.560	0.198
StH	2	72786301	72786600	0.769	0.862	0.403	1.247
StH	2	73281901	73282200	1.757	1.804	1.123	2.008
StH	2	76648201	76648500	0.586	0.684	0.210	0.776
StH	2	77828401	77828700	0.294	0.538	0.111	0.385
StH	2	77828701	77829000	0.582	0.680	0.165	0.603
StH	2	78452701	78453000	0.189	0.234	0.502	0.198
StH	2	78702901	78703200	0.611	0.491	0.181	0.507

StH	2	80961901	80962200	0.374	0.255	0.716	0.254
StH	2	81014401	81014700	0.826	0.702	0.502	0.801
StH	2	81093301	81093600	0.186	0.112	0.617	0.086
StH	2	81321901	81322200	0.266	0.234	0.025	0.547
StH	2	81615601	81615900	0.902	0.392	1.498	0.563
StH	2	82421701	82422000	0.529	0.560	0.194	0.963
StH	2	82819801	82820100	0.663	0.420	0.058	0.583
StH	2	83071501	83071800	0.158	0.236	0.551	0.081
StH	2	83179801	83180100	1.674	1.566	1.251	0.974
StH	2	83841001	83841300	0.215	0.069	0.555	0.304
StH	2	84024001	84024300	0.476	0.446	0.078	0.497
StH	2	84037201	84037500	0.160	0.163	0.419	0.162
StH	2	85161301	85161600	0.319	0.631	0.481	0.279
StH	2	85802101	85802400	0.586	0.140	0.341	0.497
StH	2	85894501	85894800	0.850	0.702	0.370	0.735
StH	2	85899001	85899300	1.568	1.757	0.925	1.572
StH	2	86133301	86133600	0.660	1.306	1.957	0.431
StH	2	86322901	86323200	0.290	0.468	0.934	0.497
StH	2	86355601	86355900	0.292	0.255	0.025	0.608
StH	2	86460301	86460600	7.503	6.091	10.313	6.039
StH	2	86837401	86837700	2.925	2.592	2.365	2.180
StH	2	87022501	87022800	5.339	5.514	2.974	4.751
StH	2	87396301	87396600	0.265	0.069	0.428	0.578
StH	2	87435301	87435600	0.319	0.468	0.189	0.385
StH	2	87579601	87579900	0.393	0.676	1.550	0.634
StH	2	87584101	87584400	0.186	0.167	0.584	0.193
StH	2	87965101	87965400	0.371	0.818	0.691	0.522
StH	2	88076101	88076400	0.105	0.094	0.575	0.112
StH	2	88368601	88368900	0.160	0.444	0.029	0.360
StH	2	88761001	88761300	0.239	0.234	0.448	0.112
StH	2	88878901	88879200	0.905	0.558	0.185	0.416
StH	2	89033401	89033700	0.640	0.611	0.111	0.472
StH	2	89091901	89092200	0.477	0.399	0.239	0.553
StH	2	89153101	89153400	0.824	0.772	0.588	1.050
StH	2	89586001	89586300	0.586	0.511	0.214	0.477
StH	2	90636001	90636300	0.215	0.161	0.707	0.228
StH	2	91336201	91336500	0.532	0.283	0.054	0.360
StH	2	91557001	91557300	0.292	0.094	0.740	0.208
StH	2	91802401	91802700	0.452	0.397	0.588	0.385
StH	2	92189101	92189400	0.455	0.774	2.927	0.223
StH	2	92211301	92211600	0.426	0.324	0.789	0.380
StH	2	92369401	92369700	0.505	0.491	0.218	0.477
StH	2	93608701	93609000	0.215	0.350	0.560	0.223
StH	2	93719101	93719400	0.268	0.116	0.531	0.061
StH	2	94411501	94411800	0.240	0.187	0.588	0.268
StH	2	95411101	95411400	0.477	0.609	0.963	0.522
StH	2	95439301	95439600	0.213	0.236	0.477	0.198
StH	2	95873101	95873400	0.371	0.350	0.794	0.319
StH	2	96160501	96160800	0.240	0.491	0.370	0.223
StH	2	97975501	97975800	0.393	0.350	1.045	0.309
StH	2	99925201	99925500	0.213	0.210	0.486	0.223
StH	2	101663101	101663400	0.448	0.626	1.217	0.558
StH	2	101697901	101698200	0.477	0.418	0.885	0.411
StH	2	101926501	101926800	0.426	0.515	0.243	0.502
StH	2	102002701	102003000	0.532	0.652	0.136	0.563
StH	2	102971401	102971700	8.964	8.847	11.449	7.692
StH	2	103047001	103047300	1.116	1.193	0.881	0.669
StH	2	103082401	103082700	0.507	0.444	0.234	0.522
StH	2	103230901	103231200	4.731	4.302	3.275	4.645
StH	2	103278301	103278600	0.215	0.236	0.477	0.137
StH	2	103441201	103441500	10.064	8.138	6.196	8.690
StH	2	103468801	103469100	0.824	0.427	2.193	0.867
StH	2	103547701	103548000	0.295	1.077	0.633	0.279
StH	2	103782601	103782900	0.319	0.560	0.983	0.533
StH	2	104006101	104006400	0.371	0.586	0.189	0.391
StH	2	104061901	104062200	0.852	0.863	1.324	0.887
StH	2	104177101	104177400	0.613	0.629	0.194	0.588

StH	2	104496901	104497200	0.531	0.513	0.798	0.553
StH	2	104637901	104638200	0.371	0.279	0.716	0.340
StH	2	105643501	105643800	0.134	0.257	0.555	0.187
StH	2	105958201	105958500	0.294	0.607	0.025	0.527
StH	2	106474801	106475100	0.026	0.607	0.156	0.329
StH	2	106550401	106550700	0.239	0.392	0.983	0.411
StH	2	106591201	106591500	1.037	1.474	0.555	0.781
StH	2	106835101	106835400	0.158	0.142	0.531	0.092
StH	2	107136301	107136600	0.507	0.867	0.317	0.720
StH	2	107219401	107219700	0.689	0.536	0.156	0.527
StH	2	107710801	107711100	0.211	0.234	0.604	0.187
StH	2	107927101	107927400	0.266	0.210	0.564	0.187
StH	2	108403201	108403500	0.819	0.283	1.398	0.877
StH	2	108721201	108721500	0.424	0.446	0.292	0.056
StH	2	109465201	109465500	1.061	1.120	0.682	0.329
StH	2	109863901	109864200	0.132	0.069	0.419	0.051
StH	2	109918201	109918500	0.453	0.112	1.016	0.583
StH	2	110265601	110265900	0.910	1.026	0.283	0.933
StH	2	110286601	110286900	6.753	5.493	3.789	6.175
StH	2	110341801	110342100	6.871	7.860	4.262	6.440
StH	2	110343901	110344200	4.312	4.965	3.311	5.289
StH	2	110365501	110365800	0.132	0.187	0.588	0.162
StH	2	110370301	110370600	2.204	1.428	9.650	3.095
StH	2	110562001	110562300	1.705	2.062	1.012	1.516
StH	2	110624701	110625000	3.684	2.987	1.353	2.976
StH	2	110634301	110634600	3.407	5.016	1.891	4.122
StH	2	110740801	110741100	0.376	0.947	3.917	0.350
StH	2	110768701	110769000	2.292	2.602	4.231	1.846
StH	2	110846701	110847000	2.381	2.886	4.588	2.003
StH	2	110880601	110880900	1.947	1.734	2.954	2.145
StH	2	110900401	110900700	1.673	1.996	3.181	1.932
StH	2	111072301	111072600	1.700	1.805	0.934	1.709
StH	2	111234301	111234600	0.266	0.384	1.494	0.365
StH	2	111343201	111343500	0.718	0.684	0.267	0.664
StH	2	111755101	111755400	1.063	1.148	0.642	1.120
StH	2	111874501	111874800	1.486	1.285	0.765	1.618
StH	2	113217301	113217600	1.010	0.935	0.481	1.055
StH	2	113268901	113269200	0.527	0.538	1.313	0.533
StH	2	113449201	113449500	0.318	0.489	0.107	0.441
StH	2	113567401	113567700	0.797	0.656	1.032	0.416
StH	2	113907601	113907900	0.027	0.335	1.201	0.356
StH	2	113907901	113908200	0.000	0.286	1.115	0.269
StH	2	113924701	113925000	5.012	5.467	2.731	4.930
StH	2	114155701	114156000	0.561	0.579	2.762	0.724
StH	2	114949501	114949800	0.610	0.609	0.317	0.751
StH	2	116103301	116103600	0.452	0.584	0.959	1.253
StH	2	116754601	116754900	0.345	0.421	0.638	0.421
StH	2	117114301	117114600	0.771	0.985	0.201	0.933
StH	2	117154501	117154800	2.442	2.306	3.090	3.732
StH	2	117172201	117172500	0.637	0.701	0.502	0.304
StH	2	117239401	117239700	0.265	0.165	0.502	0.329
StH	2	117528001	117528300	0.637	0.654	0.481	0.329
StH	2	117652501	117652800	2.360	2.995	1.411	2.059
StH	2	117732001	117732300	1.937	2.971	1.415	1.977
StH	2	117768901	117769200	1.516	1.212	0.588	1.217
StH	2	118212901	118213200	0.505	0.983	1.387	0.821
StH	2	118314601	118314900	0.400	0.463	1.284	0.355
StH	2	118721101	118721400	0.187	0.092	0.497	0.112
StH	2	118749301	118749600	0.426	0.326	0.185	0.578
StH	2	119302801	119303100	0.531	0.605	0.909	0.603
StH	2	119464501	119464800	0.557	0.560	0.292	0.669
StH	2	121021501	121021800	0.265	0.212	0.531	0.309
StH	2	121590901	121591200	0.634	0.721	1.222	0.558
StH	2	121933501	121933800	0.160	0.536	0.292	0.167
StH	2	122155201	122155500	0.158	0.208	0.477	0.198
StH	2	122778601	122778900	1.140	0.773	0.531	0.832
StH	2	123029401	123029700	0.582	0.680	0.267	0.720

StH	2	124983901	124984200	0.373	0.326	0.103	0.497
StH	2	125284201	125284500	0.371	0.541	0.798	0.497
StH	2	125616001	125616300	0.132	0.114	0.555	0.203
StH	2	127457401	127457700	0.160	0.210	0.564	0.081
StH	2	127738201	127738500	1.442	1.313	0.049	1.328
StH	2	127772701	127773000	0.134	0.142	0.502	0.254
StH	2	128001601	128001900	0.160	0.165	0.444	0.162
StH	2	128167201	128167500	0.160	0.116	0.539	0.228
StH	2	129166501	129166800	3.084	3.025	2.120	3.260
StH	2	129583801	129584100	0.319	0.210	0.695	0.365
StH	2	129840601	129840900	0.240	0.468	0.078	0.411
StH	2	130190401	130190700	0.507	0.258	0.794	0.477
StH	2	130228501	130228800	0.903	1.079	0.403	0.720
StH	2	130286101	130286400	1.037	0.817	1.460	1.141
StH	2	130348501	130348800	0.242	0.326	0.029	0.553
StH	2	130475701	130476000	2.284	2.424	0.925	2.313
StH	2	130542901	130543200	2.265	3.583	3.110	3.732
StH	2	130586101	130586400	0.532	0.678	0.881	0.441
StH	2	131142601	131142900	0.132	0.258	0.506	0.132
StH	2	131439001	131439300	1.171	1.053	0.905	1.024
StH	2	131909701	131910000	0.611	0.865	1.304	0.482
StH	2	131933401	131933700	0.532	0.373	0.136	0.416
StH	2	132422401	132422700	0.079	0.255	0.633	0.335
StH	2	132440701	132441000	0.426	0.423	0.609	0.385
StH	2	132510301	132510600	0.373	0.888	0.107	0.461
StH	2	132751801	132752100	0.718	0.560	0.292	0.446
StH	2	132901801	132902100	0.503	0.841	0.296	0.887
StH	2	134144401	134144700	0.428	0.421	0.896	0.315
StH	2	136316701	136317000	0.244	0.120	0.778	0.081
StH	2	136668001	136668300	0.878	0.682	0.272	0.695
StH	2	136872901	136873200	0.637	0.493	0.847	0.527
StH	2	137105101	137105400	0.000	0.116	0.502	0.218
StH	2	137423401	137423700	0.505	0.234	0.374	0.218
StH	2	137538001	137538300	0.292	0.444	0.107	0.056
StH	2	137891101	137891400	0.027	0.302	0.477	0.304
StH	2	138064201	138064500	2.030	2.231	3.406	1.799
StH	2	138354301	138354600	0.000	0.023	0.210	0.436
StH	2	138379801	138380100	1.760	1.467	2.633	1.400
StH	2	139096201	139096500	0.105	0.116	0.473	0.076
StH	2	139430401	139430700	0.479	0.187	0.667	0.254
StH	2	139694701	139695000	1.008	0.421	0.058	0.603
StH	2	140011501	140011800	0.371	0.352	0.107	0.497
StH	2	140179801	140180100	1.676	1.901	0.669	1.784
StH	2	141099601	141099900	0.318	0.262	0.967	0.228
StH	2	141570001	141570300	0.213	0.444	0.370	0.304
StH	2	141604201	141604500	1.140	1.171	0.448	1.607
StH	2	144202801	144203100	0.610	0.583	0.321	0.664
StH	2	145524601	145524900	0.079	0.073	0.526	0.051
StH	2	146073901	146074200	0.161	0.234	0.448	0.112
StH	2	146688601	146688900	0.799	0.822	0.428	0.862
StH	2	146786701	146787000	0.292	0.511	0.716	0.248
StH	2	148175701	148176000	0.428	0.260	0.818	0.466
StH	2	148285801	148286100	0.000	0.163	0.486	0.193
StH	2	148338601	148338900	0.213	0.352	0.617	0.162
StH	2	148945801	148946100	0.373	0.397	0.210	0.466
StH	2	148957801	148958100	0.879	0.706	0.247	0.806
StH	2	149846101	149846400	0.400	0.468	0.160	0.391
StH	2	150020101	150020400	0.213	0.607	0.428	0.329
StH	2	150867901	150868200	0.345	0.185	0.872	0.370
StH	2	151512001	151512300	1.329	0.972	2.935	0.512
StH	2	151512301	151512600	1.015	0.875	1.772	0.603
StH	2	152145901	152146200	3.933	3.624	4.496	5.258
StH	2	152329801	152330100	0.474	0.517	1.090	0.603
StH	2	153156901	153157200	0.771	0.935	0.560	0.796
StH	2	153334501	153334800	0.265	0.584	0.453	0.360
StH	2	153474601	153474900	0.027	0.068	0.992	0.031
StH	2	154869001	154869300	0.319	0.305	0.531	0.243

StH	2	154921801	154922100	0.561	0.631	1.173	0.918
StH	2	155174701	155175000	0.505	0.446	0.774	0.411
StH	2	155476201	155476500	0.240	0.234	0.593	0.304
StH	2	155691301	155691600	0.052	0.493	0.214	0.385
StH	2	156410701	156411000	2.150	3.177	1.761	2.373
StH	2	156480901	156481200	1.118	0.865	0.535	0.664
StH	2	157079701	157080000	0.824	0.682	0.292	0.740
StH	2	157106401	157106700	0.213	0.451	0.930	1.845
StH	2	157182601	157182900	0.505	0.633	0.317	0.472
StH	2	157223101	157223400	0.929	1.028	0.638	0.380
StH	2	157625701	157626000	0.287	0.023	1.495	0.309
StH	2	157626001	157626300	0.129	0.069	0.506	0.081
StH	2	158812201	158812500	0.345	0.373	0.049	0.517
StH	2	158884201	158884500	0.106	0.094	0.468	0.061
StH	2	159474301	159474600	0.426	0.277	0.691	0.274
StH	2	159642001	159642300	0.878	0.912	0.535	1.080
StH	2	160629901	160630200	0.213	0.723	0.395	0.193
StH	2	161447401	161447700	1.329	1.285	0.852	1.283
StH	2	162254401	162254700	0.424	0.371	0.843	0.391
StH	2	164494201	164494500	0.081	0.749	0.288	0.639
StH	2	165154501	165154800	1.221	1.644	0.638	1.460
StH	2	165417901	165418200	0.211	0.281	0.502	0.198
StH	2	165856501	165856800	1.037	1.637	0.729	1.136
StH	2	165868801	165869100	0.398	0.446	0.160	0.355
StH	2	167628601	167628900	1.174	1.174	0.448	1.369
StH	2	167953201	167953500	0.902	0.747	0.506	0.857
StH	2	168421501	168421800	0.137	0.399	1.012	0.239
StH	2	169216801	169217100	0.771	0.328	0.564	0.385
StH	2	169279501	169279800	0.319	0.116	0.555	0.137
StH	2	169536001	169536300	2.204	3.428	4.731	3.185
StH	2	169948501	169948800	0.558	0.421	0.296	0.416
StH	2	170725801	170726100	0.481	0.071	0.876	0.502
StH	2	172265401	172265700	0.373	0.212	0.531	0.299
StH	2	172767001	172767300	0.295	0.210	0.555	0.304
StH	2	173563801	173564100	0.213	0.191	0.667	0.324
StH	2	173715901	173716200	1.199	1.002	0.432	1.014
StH	2	174081001	174081300	0.960	0.957	0.395	0.608
StH	2	175291801	175292100	0.960	0.981	0.638	1.156
StH	2	175305301	175305600	2.746	2.345	1.576	2.596
StH	2	175361101	175361400	0.507	0.725	0.346	0.553
StH	2	175492801	175493100	4.065	3.694	2.309	3.428
StH	2	175654501	175654800	0.527	0.725	0.107	0.933
StH	2	175754401	175754700	0.771	0.395	0.243	0.497
StH	2	175758001	175758300	1.649	1.446	0.867	1.638
StH	2	176263501	176263800	0.799	0.468	0.370	0.720
StH	2	176452501	176452800	0.371	0.421	0.584	0.112
StH	2	176527201	176527500	1.408	0.985	0.604	1.019
StH	2	178874701	178875000	0.371	0.375	0.794	0.365
StH	2	179017501	179017800	0.295	0.236	0.724	0.248
StH	2	179960101	179960400	0.318	0.305	0.054	0.628
StH	2	179968201	179968500	0.694	0.631	0.925	0.385
StH	2	180000001	180000300	0.507	0.701	0.930	0.411
StH	2	180866401	180866700	1.092	0.985	0.617	1.278
StH	2	181565701	181566000	1.407	1.823	4.189	1.016
StH	2	181899001	181899300	0.319	0.326	0.588	0.274
StH	2	182160001	182160300	0.239	0.302	0.959	0.258
StH	2	183982501	183982800	1.247	1.146	0.642	1.156
StH	2	184569301	184569600	0.344	0.423	0.802	0.380
StH	2	184771201	184771500	1.278	1.053	0.662	1.075
StH	2	184871701	184872000	0.665	0.583	0.218	0.832
StH	2	185180101	185180400	0.928	0.773	0.325	0.776
StH	2	185600401	185600700	2.901	3.111	1.821	4.882
StH	2	185996101	185996400	0.369	0.260	0.691	0.335
StH	2	186423001	186423300	0.479	0.444	0.165	0.385
StH	2	186939901	186940200	1.066	1.450	0.490	1.364
StH	2	187054801	187055100	0.397	0.418	0.745	0.279
StH	2	187194601	187194900	0.639	0.491	0.292	0.411

StH	2	189268801	189269100	1.171	1.171	0.856	1.212
StH	2	189960001	189960300	0.771	0.981	0.350	1.070
StH	2	190142401	190142700	0.982	0.770	0.531	0.751
StH	2	190684201	190684500	0.398	0.562	0.827	0.578
StH	2	190740301	190740600	0.265	0.305	0.564	0.198
StH	2	190986301	190986600	0.211	0.071	0.506	0.335
StH	2	191117701	191118000	1.247	0.908	2.485	1.557
StH	2	191118001	191118300	1.271	1.289	3.123	1.877
StH	2	191406601	191406900	0.211	0.234	0.531	0.299
StH	2	192347101	192347400	0.160	0.139	0.720	0.259
StH	2	193306501	193306800	0.661	0.702	0.321	0.695
StH	2	194023501	194023800	0.505	0.494	0.239	0.664
StH	2	194067001	194067300	0.424	0.515	0.181	0.578
StH	2	196334701	196335000	0.452	0.517	0.156	0.527
StH	2	196483801	196484100	1.116	0.257	1.329	0.968
StH	2	196905301	196905600	2.823	2.639	1.906	2.911
StH	2	197093101	197093400	0.186	0.324	0.687	0.385
StH	2	199360801	199361100	1.381	0.955	1.670	1.212
StH	2	199443901	199444200	0.611	0.305	1.070	0.644
StH	2	199586101	199586400	1.247	1.448	0.374	1.602
StH	2	199586401	199586700	1.095	1.002	0.399	1.480
StH	2	199636801	199637100	0.319	0.303	0.535	0.279
StH	2	199637101	199637400	0.373	0.375	0.564	0.304
StH	2	200817001	200817300	0.187	0.210	0.502	0.112
StH	2	200873401	200873700	0.661	1.328	1.826	1.197
StH	2	200959501	200959800	1.274	1.053	1.806	0.628
StH	2	201647101	201647400	0.131	0.283	0.700	0.193
StH	2	202394401	202394700	4.249	3.969	3.188	4.654
StH	2	202584001	202584300	1.195	1.103	0.613	0.913
StH	2	203276101	203276400	0.958	1.056	0.633	1.242
StH	2	204251401	204251700	0.429	0.820	1.295	0.745
StH	2	204314101	204314400	1.482	1.732	0.575	1.865
StH	2	204587401	204587700	0.955	1.028	1.494	1.770
StH	2	204704701	204705000	1.621	1.144	1.402	1.831
StH	2	205175401	205175700	0.265	0.302	0.506	0.274
StH	2	205455301	205455600	0.134	0.189	0.535	0.157
StH	2	205488601	205488900	0.186	0.305	0.687	0.234
StH	2	206216401	206216700	0.132	0.140	0.638	0.324
StH	2	206694901	206695200	0.132	0.281	0.613	0.213
StH	2	206832301	206832600	0.642	0.487	1.177	0.690
StH	2	207345301	207345600	0.187	0.045	0.448	0.173
StH	2	208464001	208464300	0.505	0.723	0.321	0.466
StH	2	208821301	208821600	1.331	1.197	0.760	1.273
StH	2	208911301	208911600	1.089	1.054	0.609	1.105
StH	2	209038201	209038500	0.369	0.399	0.802	0.472
StH	2	209636101	209636400	0.079	0.116	0.419	0.031
StH	2	209832901	209833200	0.718	0.631	1.061	0.527
StH	2	210309301	210309600	0.479	0.609	0.160	0.411
StH	2	210971101	210971400	0.026	0.068	0.551	0.031
StH	2	210971401	210971700	0.211	0.414	1.115	0.324
StH	2	212081401	212081700	3.455	3.523	1.971	3.332
StH	2	212289001	212289300	0.558	0.446	0.239	0.416
StH	2	212337901	212338200	0.134	0.139	0.526	0.056
StH	2	213968701	213969000	0.661	0.631	0.317	0.608
StH	2	214665901	214666200	0.426	0.444	0.778	0.385
StH	2	214964701	214965000	0.398	0.421	0.560	0.162
StH	2	215728501	215728800	0.955	1.216	0.493	0.994
StH	2	215728801	215729100	0.476	0.494	0.132	0.472
StH	2	217210501	217210800	2.413	2.214	4.046	2.494
StH	2	217488601	217488900	0.398	0.395	0.082	0.304
StH	2	217791301	217791600	0.769	0.631	1.547	1.095
StH	2	217881601	217881900	1.091	0.678	0.930	0.664
StH	2	218118301	218118600	0.211	0.328	0.667	0.411
StH	2	218577001	218577300	0.560	0.796	0.959	0.720
StH	2	219547801	219548100	0.345	0.961	0.502	0.690
StH	2	219604801	219605100	2.312	2.111	1.431	2.540
StH	2	219621301	219621600	0.026	0.229	0.617	0.081

StH	2	219914701	219915000	0.213	0.513	0.107	0.223
StH	2	219944101	219944400	1.858	2.057	1.436	2.155
StH	2	219990901	219991200	0.239	0.466	0.609	0.335
StH	2	220190101	220190400	0.211	0.395	0.802	0.259
StH	2	220459501	220459800	0.239	0.225	1.415	0.543
StH	2	220717201	220717500	0.347	0.232	0.588	0.248
StH	2	220794301	220794600	1.060	0.886	1.761	1.348
StH	2	220799101	220799400	0.160	0.071	0.691	0.167
StH	2	220833901	220834200	0.773	0.761	2.845	0.902
StH	2	221787301	221787600	0.294	0.257	0.609	0.203
StH	2	222236101	222236400	0.213	0.163	0.448	0.051
StH	2	223674901	223675200	0.850	0.444	0.531	0.832
StH	2	223757101	223757400	0.824	0.955	0.477	0.968
StH	2	223828501	223828800	0.134	0.324	0.836	0.092
StH	2	223832401	223832700	1.881	3.199	4.378	1.612
StH	2	224169601	224169900	0.400	0.560	0.818	1.410
StH	2	225067201	225067500	3.270	3.149	2.004	3.062
StH	2	225147001	225147300	0.237	0.277	0.584	0.193
StH	2	225597901	225598200	0.371	0.395	0.107	0.360
StH	2	226139401	226139700	1.621	1.777	1.431	1.136
StH	2	226866601	226866900	0.079	0.444	0.263	0.391
StH	2	227130001	227130300	0.873	1.053	1.572	0.639
StH	2	227635201	227635500	0.584	0.772	0.987	0.832
StH	2	227811001	227811300	0.134	0.232	0.662	0.365
StH	2	228163201	228163500	0.584	0.702	0.424	0.167
StH	2	228252301	228252600	1.812	1.987	1.489	0.882
StH	2	228252601	228252900	1.144	1.289	0.905	0.720
StH	2	228513001	228513300	0.929	1.025	1.547	0.710
StH	2	228657301	228657600	0.905	1.049	0.424	0.634
StH	2	228662401	228662700	1.039	1.004	0.629	0.360
StH	2	229321501	229321800	3.717	3.772	1.866	3.443
StH	2	229529401	229529700	0.079	0.279	0.399	0.527
StH	2	229939501	229939800	0.661	0.914	1.378	0.690
StH	2	230287501	230287800	1.705	1.281	0.609	1.729
StH	2	230394601	230394900	0.531	0.513	0.107	0.679
StH	2	230439901	230440200	0.268	0.281	0.531	0.304
StH	2	231022201	231022500	0.290	0.796	0.588	0.335
StH	2	231271801	231272100	0.450	0.446	0.025	0.279
StH	2	231564301	231564600	0.079	0.187	0.419	0.142
StH	2	231847201	231847500	1.336	1.144	0.341	1.152
StH	2	231984301	231984600	0.982	1.472	0.658	1.161
StH	2	231990601	231990900	0.000	0.258	0.535	0.193
StH	2	232210801	232211100	0.215	0.236	0.477	0.193
StH	2	232407301	232407600	0.771	0.796	0.346	0.522
StH	2	232653901	232654200	0.690	0.888	0.428	0.740
StH	2	232665001	232665300	0.398	0.442	0.181	0.497
StH	2	233006401	233006700	1.329	1.261	0.872	1.267
StH	2	233112001	233112300	0.749	0.725	1.505	0.588
StH	2	233158201	233158500	0.134	0.229	1.050	0.142
StH	2	233424901	233425200	0.934	0.914	0.555	0.918
StH	2	233444701	233445000	0.132	0.444	0.029	0.193
StH	2	233614801	233615100	2.607	2.289	0.720	2.483
StH	2	233842201	233842500	0.558	0.466	0.181	0.385
StH	2	234261901	234262200	0.323	0.324	0.847	0.319
StH	2	234282001	234282300	0.424	0.729	0.132	0.391
StH	2	234577801	234578100	0.265	0.302	0.107	0.497
StH	2	234777601	234777900	0.237	0.326	0.749	0.340
StH	2	234826501	234826800	0.215	0.202	1.016	0.132
StH	2	234838201	234838500	0.106	0.202	0.847	0.076
StH	2	235000501	235000800	0.397	0.326	0.613	0.391
StH	2	235680601	235680900	2.471	4.765	3.234	3.935
StH	2	235723201	235723500	2.020	1.261	1.431	1.075
StH	2	235768501	235768800	0.160	0.234	0.424	0.497
StH	2	236059801	236060100	0.294	0.208	0.580	0.167
StH	2	236094301	236094600	0.134	0.142	0.889	0.106
StH	2	236172301	236172600	0.237	0.794	0.453	0.664
StH	2	236261701	236262000	0.663	0.601	1.444	0.659

StH	2	236450401	236450700	3.433	2.590	3.007	2.429
StH	2	236615101	236615400	0.823	0.749	0.453	0.279
StH	2	236710501	236710800	0.318	0.491	0.160	0.446
StH	2	236871001	236871300	0.586	0.468	0.152	0.416
StH	2	237080701	237081000	0.690	0.555	2.622	1.182
StH	2	237081001	237081300	0.082	0.253	1.911	0.567
StH	2	237084301	237084600	1.145	1.452	0.745	1.298
StH	2	237151501	237151800	0.424	0.206	1.235	0.213
StH	2	237174301	237174600	1.686	1.744	2.818	1.683
StH	2	237174601	237174900	0.352	0.478	1.494	0.304
StH	2	237209401	237209700	1.303	1.082	0.461	1.384
StH	2	237265501	237265800	2.920	3.154	2.151	2.758
StH	2	237840301	237840600	0.266	0.678	0.049	0.243
StH	2	238623901	238624200	1.010	1.054	0.662	1.197
StH	2	238828801	238829100	0.718	0.796	0.346	1.019
StH	2	239065801	239066100	3.273	2.937	2.082	4.061
StH	2	239595901	239596200	3.809	4.021	2.127	3.519
StH	2	239663701	239664000	1.142	1.283	1.567	0.887
StH	2	239673901	239674200	0.349	0.277	0.691	0.329
StH	2	239679901	239680200	1.436	1.334	0.787	1.465
StH	2	240546301	240546600	1.489	1.334	0.818	1.029
StH	2	240704701	240705000	0.503	0.702	0.185	0.391
StH	2	240788101	240788400	0.350	0.435	1.271	0.178
StH	2	240939901	240940200	0.027	0.161	0.448	0.000
StH	2	240949201	240949500	0.213	0.281	0.526	0.299
StH	2	241154101	241154400	0.903	1.148	0.453	0.725
StH	2	241295401	241295700	0.319	0.247	1.505	0.411
StH	2	241295701	241296000	0.853	0.807	2.158	0.796
StH	2	241310401	241310700	0.261	0.772	3.078	0.823
StH	2	241356901	241357200	0.903	1.053	0.560	1.298
StH	2	241476901	241477200	1.276	1.776	1.008	1.136
StH	2	241714201	241714500	1.386	1.262	1.723	1.019
StH	2	241750501	241750800	0.423	0.656	0.107	0.385
StH	2	242063701	242064000	0.958	0.680	1.193	0.527
StH	2	242152801	242153100	0.292	0.305	0.477	0.335
StH	2	242171701	242172000	0.479	0.680	0.292	0.167
StH	2	242286601	242286900	2.847	2.719	1.957	3.053
StH	2	242406601	242406900	0.106	0.140	0.502	0.025
StH	2	242786401	242786700	0.371	0.641	1.692	0.452
StH	2	242905801	242906100	1.087	0.961	0.374	0.695
StH	2	242914201	242914500	0.265	0.258	0.992	0.335
StH	2	243036001	243036300	0.907	0.931	3.346	0.431
StH	2	243120601	243120900	0.000	0.112	0.756	0.162
StH	2	243219901	243220200	1.862	3.529	3.184	2.804
StH	2	243488101	243488400	0.689	0.468	0.239	0.466
StH	2	243516301	243516600	3.661	2.098	0.247	4.172
StH	2	243792601	243792900	0.481	0.678	1.291	0.421
StH	2	243792901	243793200	1.200	1.032	2.385	1.323
StH	2	243933301	243933600	0.397	0.491	0.049	0.309
StH	2	244067101	244067400	1.061	0.695	1.794	0.857
StH	2	244107601	244107900	1.252	1.699	3.114	1.938
StH	2	244130101	244130400	5.358	5.285	3.010	5.375
StH	2	244663501	244663800	0.745	0.863	0.453	0.690
StH	2	244836601	244836900	0.213	0.542	1.781	0.086
StH	2	244870801	244871100	4.152	4.235	3.185	4.380
StH	2	244959301	244959600	1.807	1.500	1.173	2.150
StH	2	245467801	245468100	1.515	1.099	0.564	1.090
StH	2	245674801	245675100	5.978	5.896	4.082	6.871
StH	2	245743501	245743800	1.436	0.841	1.090	0.893
StH	2	245805901	245806200	1.379	1.476	0.959	1.552
StH	2	245860201	245860500	0.742	1.186	3.576	1.015
StH	2	245860501	245860800	0.079	0.229	1.039	0.167
StH	2	245974201	245974500	0.429	0.577	1.284	0.527
StH	2	246099901	246100200	0.471	0.643	2.143	0.452
StH	2	246125401	246125700	1.887	2.173	1.625	1.962
StH	2	246145201	246145500	5.758	5.750	4.162	5.963
StH	2	246170101	246170400	0.079	0.210	0.811	0.147

StH	2	246216601	246216900	1.753	1.731	0.776	1.978
StH	2	246244201	246244500	6.170	6.907	4.713	6.375
StH	2	246285601	246285900	8.637	9.003	7.762	8.421
StH	2	246309601	246309900	1.331	1.382	0.785	1.511
StH	2	246316201	246316500	7.716	7.125	6.438	8.052
StH	2	246400801	246401100	0.187	0.560	0.399	0.223
StH	2	246609301	246609600	0.828	0.942	2.508	1.025
StH	2	246637801	246638100	0.553	0.913	2.670	0.496
StH	2	246721201	246721500	3.515	3.283	2.283	3.326
StH	2	246749101	246749400	0.397	0.397	1.077	0.446
StH	2	246843001	246843300	3.455	4.158	2.731	3.032
StH	2	246908101	246908400	0.505	0.680	0.317	0.659
StH	2	247095901	247096200	0.982	0.652	0.346	0.614
StH	2	247105801	247106100	1.195	1.343	0.395	1.151
StH	2	247136701	247137000	0.984	0.912	0.308	0.690
StH	2	247250701	247251000	0.529	0.466	1.016	0.578
StH	2	247289701	247290000	4.764	5.259	3.473	5.495
StH	2	247683301	247683600	0.455	1.722	6.841	0.943
StH	2	247823401	247823700	0.963	0.824	2.766	1.161
StH	2	247824301	247824600	0.027	0.184	0.976	0.203
StH	2	247836301	247836600	1.011	1.287	3.813	1.095
StH	2	247925101	247925400	0.081	0.161	0.588	0.360
StH	2	247935901	247936200	2.095	1.939	0.930	2.301
StH	2	247945501	247945800	1.091	0.841	1.514	1.176
StH	2	248045101	248045400	0.637	0.525	1.551	0.360
StH	2	248097301	248097600	0.368	0.360	2.278	0.787
StH	2	248151001	248151300	0.290	0.322	1.074	0.167
StH	2	248151301	248151600	0.539	0.860	3.041	0.254
StH	2	248224801	248225100	0.560	0.390	1.168	0.517
StH	2	248246701	248247000	1.091	0.942	3.205	1.532
StH	2	248315401	248315700	1.036	1.165	2.193	0.760
StH	2	248455501	248455800	0.266	0.566	1.658	0.365
StH	2	248455801	248456100	0.131	0.137	1.930	0.137
StH	2	248507401	248507700	5.582	5.602	2.562	5.518
StH	2	248567401	248567700	0.136	0.272	1.074	0.086
StH	2	248567701	248568000	0.055	0.431	1.596	0.056
StH	2	248568001	248568300	1.192	2.323	5.927	2.124
StH	2	248568901	248569200	2.042	1.819	4.164	1.745
StH	2	248643901	248644200	0.266	0.455	1.275	0.340
StH	2	248677201	248677500	4.443	4.383	3.529	4.578
StH	2	248741701	248742000	0.565	0.446	1.509	0.679
StH	2	248748601	248748900	1.778	1.758	4.726	1.825
StH	2	248760301	248760600	2.755	3.430	9.049	2.874
StH	2	248775901	248776200	0.371	0.463	1.317	0.324
StH	2	248835301	248835600	0.129	0.487	1.210	0.228
StH	20	16501	16800	13.020	13.180	7.510	12.839
StH	20	96001	96300	0.532	0.232	0.132	0.248
StH	20	1086901	1087200	0.397	0.210	0.638	0.218
StH	20	2238301	2238600	0.026	0.283	0.584	0.198
StH	20	2776801	2777100	0.108	0.212	0.510	0.132
StH	20	2906401	2906700	0.400	0.465	0.160	0.416
StH	20	3112801	3113100	0.187	0.092	0.593	0.081
StH	20	3126001	3126300	1.197	1.021	2.036	0.999
StH	20	3675901	3676200	0.532	0.281	0.185	0.360
StH	20	3859801	3860100	0.318	0.440	1.293	0.426
StH	20	3909001	3909300	3.112	2.926	3.772	3.489
StH	20	4280101	4280400	0.158	0.159	0.642	0.086
StH	20	4393501	4393800	1.011	1.028	1.391	0.988
StH	20	4882501	4882800	1.115	1.356	0.539	1.111
StH	20	5581201	5581500	1.274	1.614	1.037	1.572
StH	20	6475501	6475800	7.500	5.945	3.947	5.146
StH	20	6475801	6476100	4.544	3.708	1.755	3.853
StH	20	6685501	6685800	0.132	0.232	0.555	0.309
StH	20	6740701	6741000	2.281	2.508	1.016	1.897
StH	20	6772201	6772500	1.542	0.912	0.551	0.974
StH	20	6824701	6825000	0.529	0.824	2.499	0.649
StH	20	6837601	6837900	1.244	1.240	0.593	1.227

StH	20	6905401	6905700	0.371	0.328	0.531	0.416
StH	20	7115401	7115700	1.144	1.191	0.667	1.531
StH	20	7123201	7123500	4.470	2.077	3.531	4.604
StH	20	7265401	7265700	0.218	0.112	0.852	0.173
StH	20	7746301	7746600	0.534	0.489	0.214	0.472
StH	20	7836301	7836600	0.373	1.201	5.536	0.507
StH	20	7842301	7842600	1.655	2.495	3.212	2.225
StH	20	7872601	7872900	0.266	0.678	0.876	1.136
StH	20	7881301	7881600	1.011	1.335	0.629	1.303
StH	20	8250601	8250900	0.319	0.470	0.789	0.309
StH	20	8440201	8440500	0.692	0.790	0.243	0.700
StH	20	8635801	8636100	3.871	3.265	2.107	4.097
StH	20	8703601	8703900	0.268	0.538	1.275	0.385
StH	20	8753401	8753700	0.666	0.539	0.210	0.522
StH	20	8846401	8846700	0.507	0.257	0.667	0.391
StH	20	9252601	9252900	4.294	4.604	3.056	4.685
StH	20	9354301	9354600	0.823	1.193	0.481	1.192
StH	20	9483001	9483300	0.503	0.562	0.847	0.335
StH	20	10121101	10121400	0.239	0.444	0.054	0.137
StH	20	10367101	10367400	0.266	0.444	0.214	0.056
StH	20	10631701	10632000	0.797	0.820	0.555	0.882
StH	20	10687501	10687800	0.558	0.489	0.740	0.279
StH	20	10717201	10717500	0.136	0.517	1.975	0.432
StH	20	10979401	10979700	0.424	0.702	1.057	0.588
StH	20	11039401	11039700	0.715	0.556	1.188	0.497
StH	20	11091301	11091600	1.034	0.701	0.247	0.826
StH	20	11417101	11417400	2.100	2.222	4.447	1.586
StH	20	11535301	11535600	0.321	0.234	0.580	0.360
StH	20	11538001	11538300	0.106	0.232	0.642	0.167
StH	20	11692201	11692500	1.645	1.586	0.823	1.562
StH	20	11761801	11762100	0.532	0.678	0.185	0.279
StH	20	12799201	12799500	0.213	0.210	0.473	0.137
StH	20	13056601	13056900	0.211	0.118	0.531	0.142
StH	20	13310701	13311000	0.452	0.305	0.856	0.350
StH	20	13605301	13605600	0.292	0.302	0.711	0.173
StH	20	14429701	14430000	1.808	2.594	1.986	2.515
StH	20	14513701	14514000	0.508	0.493	0.881	1.323
StH	20	14620201	14620500	0.242	0.305	0.720	0.213
StH	20	14672701	14673000	0.692	0.541	0.156	0.603
StH	20	15272101	15272400	4.117	4.943	2.708	4.816
StH	20	15286201	15286500	0.505	0.489	0.930	0.446
StH	20	15363601	15363900	1.218	2.150	0.522	1.055
StH	20	15493801	15494100	0.215	0.112	0.976	0.081
StH	20	15900601	15900900	4.968	4.928	4.110	4.934
StH	20	16055401	16055700	0.823	1.259	0.272	0.816
StH	20	16073401	16073700	0.907	1.004	3.586	0.969
StH	20	16166401	16166700	0.347	0.326	0.506	0.248
StH	20	16246201	16246500	0.160	0.165	0.419	0.081
StH	20	16458301	16458600	1.566	1.403	0.388	1.182
StH	20	16465501	16465800	3.930	2.909	5.693	4.482
StH	20	16693801	16694100	1.170	1.240	0.584	0.983
StH	20	16896601	16896900	0.929	1.006	0.390	0.846
StH	20	17077501	17077800	0.452	0.328	0.555	0.329
StH	20	17129701	17130000	0.242	0.208	0.729	0.112
StH	20	17141401	17141700	1.092	1.508	0.254	1.186
StH	20	17141701	17142000	0.477	0.654	0.127	0.466
StH	20	17379901	17380200	1.892	1.541	1.070	2.200
StH	20	17469001	17469300	0.692	0.444	0.317	0.446
StH	20	17583001	17583300	0.586	0.465	0.267	0.857
StH	20	17609401	17609700	1.011	1.242	0.584	1.541
StH	20	17610001	17610300	1.252	1.824	0.675	1.247
StH	20	17614201	17614500	0.587	0.766	1.632	0.841
StH	20	17739001	17739300	0.213	0.303	0.560	0.167
StH	20	17855101	17855400	0.082	0.204	0.687	0.112
StH	20	18114001	18114300	0.665	0.515	0.267	0.664
StH	20	18286801	18287100	3.227	2.735	1.443	2.621
StH	20	18858601	18858900	0.797	0.796	0.296	0.817

StH	20	18978901	18979200	1.328	1.547	0.802	1.465
StH	20	19026301	19026600	0.321	0.562	0.160	0.360
StH	20	19036801	19037100	0.477	0.375	0.189	0.472
StH	20	19329901	19330200	0.586	0.399	0.769	0.360
StH	20	19343101	19343400	2.287	3.004	0.907	2.318
StH	20	19357801	19358100	0.294	0.165	0.633	0.304
StH	20	20016301	20016600	0.558	0.538	0.181	0.385
StH	20	20095201	20095500	0.908	1.051	2.040	0.947
StH	20	20688301	20688600	0.319	0.749	0.555	0.279
StH	20	20885401	20885700	0.532	0.448	0.136	0.477
StH	20	21083701	21084000	0.105	0.094	0.593	0.243
StH	20	21277801	21278100	0.824	1.122	1.551	0.725
StH	20	21890701	21891000	0.373	0.352	0.185	0.527
StH	20	22089601	22089900	4.875	4.615	3.431	4.599
StH	20	22354201	22354500	0.665	0.631	0.827	0.527
StH	20	22508401	22508700	2.549	2.622	1.830	1.126
StH	20	22663501	22663800	0.611	0.071	0.214	0.137
StH	20	24503401	24503700	0.265	0.305	0.695	0.446
StH	20	24648901	24649200	0.611	0.656	0.453	0.999
StH	20	25007101	25007400	0.055	0.140	0.473	0.173
StH	20	25198501	25198800	1.197	2.066	2.855	1.440
StH	20	25225801	25226100	0.160	0.230	0.560	0.187
StH	20	25251001	25251300	0.186	0.260	0.638	0.142
StH	20	25294801	25295100	0.158	0.803	1.579	0.167
StH	20	25315801	25316100	1.226	2.006	0.408	1.684
StH	20	25793701	25794000	0.850	0.852	1.690	0.695
StH	20	26466301	26466600	0.026	0.112	1.038	0.025
StH	20	26596201	26596500	0.507	0.839	1.090	0.695
StH	20	26807401	26807700	0.823	0.725	0.267	0.533
StH	20	26856301	26856600	0.611	0.562	0.716	0.472
StH	20	27016801	27017100	0.215	0.157	0.667	0.162
StH	20	27317101	27317400	0.240	0.444	0.711	0.223
StH	20	27374101	27374400	4.091	4.677	2.907	4.897
StH	20	27996301	27996600	3.919	4.175	2.274	3.814
StH	20	29124601	29124900	0.000	0.187	0.580	0.182
StH	20	29548501	29548800	0.558	0.534	1.061	0.547
StH	20	30054601	30054900	2.617	3.130	1.498	2.774
StH	20	30240301	30240600	0.211	0.118	0.609	0.446
StH	20	30439501	30439800	0.081	0.096	0.444	0.086
StH	20	30449101	30449400	1.437	0.989	0.205	0.745
StH	20	30837601	30837900	2.099	1.547	1.115	1.410
StH	20	31059901	31060200	0.132	0.212	0.506	0.162
StH	20	31151101	31151400	1.781	1.171	1.358	1.552
StH	20	31733101	31733400	2.287	2.244	1.152	2.494
StH	20	31768201	31768500	0.984	0.843	0.560	0.857
StH	20	31839301	31839600	0.079	0.279	0.560	0.279
StH	20	31924801	31925100	3.568	3.495	2.245	3.199
StH	20	32101801	32102100	2.783	2.686	1.327	2.728
StH	20	32127901	32128200	2.178	2.626	1.612	0.902
StH	20	33742801	33743100	0.215	0.352	0.716	0.492
StH	20	34877101	34877400	0.823	1.517	0.506	1.141
StH	20	35078101	35078400	0.187	0.236	0.555	0.173
StH	20	35808901	35809200	0.108	0.187	0.419	0.167
StH	20	36377701	36378000	0.426	0.605	0.054	0.304
StH	20	36496501	36496800	0.240	0.444	0.082	0.391
StH	20	36721801	36722100	2.044	2.040	0.831	1.683
StH	20	37231801	37232100	0.371	0.326	0.132	0.466
StH	20	38040901	38041200	0.508	0.678	0.136	0.527
StH	20	38048101	38048400	0.132	0.213	0.502	0.137
StH	20	38229601	38229900	0.716	0.730	0.189	1.004
StH	20	38343301	38343600	0.611	0.820	0.181	0.527
StH	20	39446401	39446700	0.584	0.657	0.156	0.446
StH	20	39459001	39459300	0.426	0.959	0.210	0.385
StH	20	39997501	39997800	0.481	0.354	0.896	0.248
StH	20	40396501	40396800	2.734	2.851	1.748	2.256
StH	20	40410001	40410300	0.239	0.257	0.642	0.234
StH	20	40452301	40452600	0.450	0.701	1.807	0.877

StH	20	40503901	40504200	2.396	2.780	1.806	2.571
StH	20	40815601	40815900	5.533	5.291	3.786	5.228
StH	20	40873501	40873800	4.036	3.699	2.247	3.245
StH	20	41570401	41570700	0.160	0.538	0.477	0.553
StH	20	41591701	41592000	0.318	0.185	0.711	0.167
StH	20	41921101	41921400	1.303	1.407	0.876	1.404
StH	20	42226501	42226800	0.663	0.772	0.395	0.715
StH	20	42515101	42515400	2.315	2.382	2.078	2.850
StH	20	42637201	42637500	0.131	0.213	0.785	0.167
StH	20	42788101	42788400	0.424	0.448	0.716	0.385
StH	20	43028101	43028400	0.690	0.820	1.090	0.852
StH	20	43282801	43283100	3.296	3.621	2.358	3.336
StH	20	43628401	43628700	0.345	0.491	0.103	0.391
StH	20	43713601	43713900	3.801	3.605	2.805	3.950
StH	20	43855501	43855800	0.426	0.699	0.160	0.391
StH	20	44193601	44193900	2.258	2.221	3.674	1.481
StH	20	44193901	44194200	2.367	2.298	3.415	1.795
StH	20	44431801	44432100	4.194	4.139	3.246	4.081
StH	20	44432101	44432400	1.649	1.566	1.115	1.278
StH	20	44726701	44727000	1.113	0.508	1.772	0.675
StH	20	44729701	44730000	0.799	0.704	0.374	0.740
StH	20	44856001	44856300	0.213	0.493	0.078	0.248
StH	20	45073801	45074100	0.531	0.468	0.185	0.056
StH	20	45275101	45275400	0.744	0.390	1.768	0.583
StH	20	45645601	45645900	0.292	0.995	2.546	1.633
StH	20	46096201	46096500	0.613	0.701	1.032	0.446
StH	20	46283701	46284000	3.163	3.388	6.400	3.675
StH	20	46284601	46284900	0.345	0.401	0.025	0.329
StH	20	46354501	46354800	0.611	0.539	0.370	0.218
StH	20	46421401	46421700	1.224	1.152	0.481	1.019
StH	20	46781401	46781700	0.639	1.028	0.983	0.608
StH	20	46806601	46806900	0.184	0.163	0.707	0.223
StH	20	46820701	46821000	2.341	2.062	1.394	2.596
StH	20	47158501	47158800	2.562	2.365	3.781	2.536
StH	20	47314201	47314500	1.674	1.197	0.519	1.080
StH	20	47340301	47340600	3.005	2.963	2.020	2.844
StH	20	47708701	47709000	1.515	3.062	2.280	1.308
StH	20	47750401	47750700	1.382	1.127	0.740	1.440
StH	20	47750701	47751000	1.039	0.981	0.711	1.328
StH	20	47878801	47879100	2.539	1.969	4.324	2.231
StH	20	48181501	48181800	1.415	2.040	0.785	2.155
StH	20	48501901	48502200	4.149	4.412	2.370	3.701
StH	20	48606601	48606900	0.958	0.633	1.242	0.639
StH	20	48752101	48752400	1.970	3.042	5.044	2.783
StH	20	48783901	48784200	0.581	0.929	2.149	0.659
StH	20	48784201	48784500	1.528	1.728	5.982	1.603
StH	20	48795601	48795900	1.071	1.234	3.562	1.206
StH	20	49170301	49170600	3.481	4.445	4.989	4.133
StH	20	49185301	49185600	1.092	1.289	1.946	1.059
StH	20	49278901	49279200	0.637	0.764	2.127	0.623
StH	20	49293301	49293600	1.037	1.290	1.752	0.826
StH	20	49355701	49356000	0.610	0.650	1.308	0.441
StH	20	49531801	49532100	1.084	0.905	3.968	1.177
StH	20	49542601	49542900	0.955	1.785	0.350	1.080
StH	20	49645801	49646100	2.633	2.997	1.946	3.138
StH	20	49678801	49679100	1.484	1.725	0.876	1.876
StH	20	49732501	49732800	5.580	4.388	7.640	5.187
StH	20	49768501	49768800	0.666	0.725	0.301	0.639
StH	20	49876201	49876500	3.641	3.205	4.151	3.767
StH	20	50025901	50026200	1.516	2.126	2.595	1.638
StH	20	50043901	50044200	7.845	8.698	6.143	9.040
StH	20	50167801	50168100	0.027	0.157	1.061	0.432
StH	20	50301601	50301900	0.158	0.090	0.580	0.228
StH	20	50384701	50385000	0.374	1.784	6.749	0.996
StH	20	50397001	50397300	0.132	0.519	1.765	0.101
StH	20	50397301	50397600	0.532	1.190	3.444	0.822
StH	20	50542801	50543100	0.368	1.353	5.428	0.985

StH	20	50618401	50618700	0.718	1.205	4.588	0.947
StH	20	50634601	50634900	0.482	0.751	1.320	0.801
StH	20	50648101	50648400	0.479	0.409	1.910	0.386
StH	20	50648401	50648700	0.448	0.427	2.306	0.345
StH	20	50648701	50649000	0.502	0.545	1.881	0.320
StH	20	50756701	50757000	0.160	0.204	0.785	0.117
StH	20	50801401	50801700	0.747	1.130	3.119	1.233
StH	20	50820901	50821200	2.734	2.809	1.171	2.440
StH	20	50917501	50917800	3.038	2.899	2.221	2.961
StH	20	51006001	51006300	8.956	8.410	6.303	8.398
StH	21	25801	26100	1.997	2.058	2.468	2.738
StH	21	484501	484800	5.477	5.870	5.265	5.805
StH	21	501001	501300	0.665	0.772	0.395	0.578
StH	21	620701	621000	0.292	0.210	0.477	0.243
StH	21	1091101	1091400	2.179	1.873	1.251	1.674
StH	21	1120501	1120800	4.675	4.085	3.308	4.381
StH	21	1441501	1441800	1.066	1.006	0.448	0.832
StH	21	1460401	1460700	1.116	0.609	1.427	0.628
StH	21	1509001	1509300	0.611	0.560	0.346	0.578
StH	21	1776601	1776900	4.752	4.199	5.899	4.213
StH	21	2236201	2236500	0.398	0.611	0.185	0.527
StH	21	3898501	3898800	0.158	0.185	0.506	0.193
StH	21	4140901	4141200	0.747	0.732	1.975	0.938
StH	21	4556101	4556400	0.187	0.118	0.617	0.173
StH	21	4751701	4752000	0.134	0.120	0.930	0.223
StH	21	4752001	4752300	0.136	0.142	1.237	0.051
StH	21	4752301	4752600	0.053	0.068	0.638	0.031
StH	21	4752601	4752900	0.052	0.023	0.486	0.056
StH	21	5166901	5167200	0.026	0.023	0.506	0.228
StH	21	5249701	5250000	0.213	0.420	0.082	0.274
StH	21	5901001	5901300	0.452	0.586	0.087	0.558
StH	21	5946301	5946600	0.081	0.210	0.448	0.173
StH	21	6243301	6243600	0.694	1.167	0.214	0.648
StH	21	6480901	6481200	1.144	0.950	1.921	0.974
StH	21	6780901	6781200	0.986	1.099	0.439	1.019
StH	21	6888601	6888900	2.181	1.991	1.592	2.054
StH	21	7033501	7033800	0.239	0.212	0.633	0.254
StH	21	7546201	7546500	0.957	1.450	1.119	1.465
StH	21	7638301	7638600	0.718	0.685	1.304	0.507
StH	21	7904401	7904700	0.290	1.541	1.070	1.465
StH	21	8120101	8120400	2.047	1.659	2.492	2.115
StH	21	8337001	8337300	1.116	1.056	1.665	1.095
StH	21	8388901	8389200	0.290	0.442	0.914	0.461
StH	21	8503201	8503500	2.523	1.557	7.152	3.188
StH	21	8561101	8561400	0.476	0.583	1.090	0.486
StH	21	8768401	8768700	1.015	1.148	0.477	1.202
StH	21	8911501	8911800	5.278	5.461	6.831	8.903
StH	21	8939701	8940000	1.305	1.568	0.729	1.141
StH	21	9513001	9513300	0.108	0.206	0.818	0.208
StH	21	9700801	9701100	0.292	0.187	0.346	0.497
StH	21	9843901	9844200	0.184	0.725	0.424	0.198
StH	21	9957301	9957600	1.353	1.171	0.881	0.608
StH	21	10111501	10111800	0.905	0.725	0.424	0.776
StH	21	10610101	10610400	0.347	0.118	0.560	0.355
StH	21	10663201	10663500	0.639	0.234	0.395	0.223
StH	21	11607001	11607300	0.508	0.468	1.213	0.345
StH	21	11746501	11746800	0.965	0.556	1.830	0.751
StH	21	11805601	11805900	1.115	2.879	2.066	1.115
StH	21	11857501	11857800	0.426	0.352	0.165	0.583
StH	21	12025201	12025500	0.479	0.515	0.321	0.472
StH	21	12784201	12784500	0.584	0.513	0.716	0.279
StH	21	13114801	13115100	0.265	0.305	0.502	0.223
StH	21	13693201	13693500	0.292	0.114	0.798	0.385
StH	21	14001601	14001900	3.854	0.116	1.658	4.154
StH	21	14001901	14002200	3.615	0.139	1.741	4.454
StH	21	14290801	14291100	0.160	0.515	0.239	0.335
StH	21	14390401	14390700	0.852	1.800	0.609	0.745

StH	21	15432901	15433200	0.186	0.071	0.477	0.086
StH	21	15528301	15528600	0.215	0.189	0.575	0.081
StH	21	15684301	15684600	0.345	0.302	0.584	0.274
StH	21	15841501	15841800	0.371	0.515	0.979	0.720
StH	21	15924601	15924900	0.106	0.185	0.477	0.223
StH	21	16027501	16027800	0.402	0.420	0.132	0.446
StH	21	16172101	16172400	0.211	0.139	0.502	0.187
StH	21	16333501	16333800	2.587	1.937	6.446	2.884
StH	21	16351501	16351800	2.533	2.961	0.000	1.933
StH	21	16360201	16360500	1.226	1.028	0.497	0.948
StH	21	16394401	16394700	2.286	2.396	4.338	2.981
StH	21	16441801	16442100	1.570	1.843	0.651	1.755
StH	21	16674001	16674300	0.637	0.631	0.210	0.603
StH	21	16678201	16678500	0.587	0.538	0.214	0.553
StH	21	16824301	16824600	0.398	0.356	0.798	0.380
StH	21	17131801	17132100	2.571	2.648	3.442	4.365
StH	21	17183401	17183700	0.323	0.802	3.554	0.268
StH	21	17674801	17675100	1.803	2.364	0.979	1.973
StH	21	18045301	18045600	0.663	0.772	0.346	0.614
StH	21	18573901	18574200	0.984	0.069	0.613	0.299
StH	21	18693001	18693300	0.026	0.069	0.390	0.081
StH	21	18932701	18933000	0.106	0.258	0.609	0.360
StH	21	18959701	18960000	0.373	0.255	0.852	0.527
StH	21	18981601	18981900	0.187	0.212	0.609	0.279
StH	21	19878301	19878600	0.345	0.303	0.716	0.391
StH	21	21392401	21392700	0.503	0.466	0.239	0.720
StH	21	21917101	21917400	0.560	0.303	0.185	0.664
StH	21	22399801	22400100	0.637	0.863	0.176	0.527
StH	21	22515601	22515900	6.549	1.786	11.404	5.988
StH	21	22515901	22516200	2.520	0.745	3.859	2.381
StH	21	23041201	23041500	0.160	0.140	0.473	0.142
StH	21	23041501	23041800	0.239	0.139	0.477	0.142
StH	21	23097901	23098200	0.398	0.586	0.160	0.533
StH	21	23129701	23130000	0.211	0.350	0.662	0.279
StH	21	23252401	23252700	0.453	0.702	1.148	0.482
StH	21	23719201	23719500	1.058	1.148	0.526	0.056
StH	21	23726701	23727000	0.505	0.584	0.267	0.360
StH	21	24123301	24123600	0.373	0.584	0.214	0.137
StH	21	24153601	24153900	0.878	1.126	0.473	0.720
StH	21	24610201	24610500	0.316	0.328	0.925	0.482
StH	21	25004401	25004700	0.955	1.613	1.304	0.157
StH	21	25019701	25020000	0.027	0.096	0.544	0.025
StH	21	25134901	25135200	1.245	0.953	0.321	0.902
StH	21	25180501	25180800	1.036	0.910	0.506	0.857
StH	21	25680901	25681200	0.561	0.654	0.132	0.664
StH	21	25681201	25681500	0.424	0.536	0.107	0.441
StH	21	25789201	25789500	0.586	0.468	0.234	0.720
StH	21	25856101	25856400	0.663	0.764	1.304	0.801
StH	21	26057701	26058000	0.186	0.322	0.938	0.259
StH	21	26329801	26330100	0.160	0.120	0.477	0.106
StH	21	27469801	27470100	1.599	1.335	0.473	0.882
StH	21	27566401	27566700	0.507	0.274	1.300	0.506
StH	21	27978901	27979200	0.136	1.776	0.823	0.025
StH	21	28011601	28011900	0.720	0.654	0.399	0.603
StH	21	28297801	28298100	1.491	0.983	0.510	1.009
StH	21	28569001	28569300	0.077	0.491	0.267	0.441
StH	21	28926901	28927200	1.355	2.812	6.057	1.288
StH	21	28965601	28965900	1.891	3.744	2.570	3.458
StH	21	28973101	28973400	6.909	7.179	6.371	7.438
StH	21	28975501	28975800	0.505	1.073	0.802	1.075
StH	21	28980901	28981200	0.000	0.330	0.667	0.319
StH	21	28989301	28989600	0.479	0.560	0.127	0.421
StH	21	29127601	29127900	1.063	0.865	1.761	0.731
StH	21	29163601	29163900	0.239	0.258	1.148	0.486
StH	21	29183701	29184000	0.850	0.725	0.584	0.690
StH	21	29260801	29261100	0.928	1.221	0.453	0.913
StH	21	29429101	29429400	1.652	1.571	0.745	2.023

StH	21	29532601	29532900	3.954	3.386	2.336	3.418
StH	21	30965401	30965700	0.079	0.024	0.415	0.081
StH	21	31383901	31384200	2.159	3.059	1.527	2.408
StH	21	31482901	31483200	3.785	3.495	5.423	3.421
StH	21	31507801	31508100	1.916	2.158	1.329	0.837
StH	21	31534801	31535100	0.479	0.423	0.132	0.583
StH	21	31895401	31895700	0.742	0.725	0.218	0.684
StH	21	31915801	31916100	0.745	0.491	0.156	0.522
StH	21	32372101	32372400	1.889	2.062	2.517	2.925
StH	21	32730301	32730600	0.213	0.281	0.604	0.112
StH	21	32934001	32934300	0.423	0.375	0.029	0.385
StH	21	33032701	33033000	0.186	0.210	0.560	0.299
StH	21	33037201	33037500	2.899	2.870	7.565	1.779
StH	21	33049201	33049500	0.716	0.766	1.333	0.852
StH	21	33080401	33080700	1.491	2.761	3.208	2.008
StH	21	33082501	33082800	1.269	1.710	2.485	1.278
StH	21	33171001	33171300	1.118	0.987	0.502	1.156
StH	21	33599701	33600000	0.799	0.820	0.424	0.751
StH	21	34141201	34141500	6.522	5.469	7.918	6.679
StH	21	34529101	34529400	0.213	0.232	0.560	0.213
StH	21	34602601	34602900	2.607	1.687	1.152	2.475
StH	21	34634401	34634700	0.929	1.171	0.740	1.192
StH	21	34634701	34635000	5.215	4.529	2.049	4.289
StH	21	34701001	34701300	0.265	0.562	0.054	0.634
StH	21	34746901	34747200	0.398	0.493	0.185	0.446
StH	21	34854301	34854600	1.994	1.712	1.275	1.831
StH	21	35236201	35236500	1.145	1.212	1.654	1.125
StH	21	35239501	35239800	0.424	0.680	0.160	0.304
StH	21	35269201	35269500	2.059	3.001	5.196	2.468
StH	21	35456401	35456700	1.813	1.714	2.501	1.622
StH	21	35895301	35895600	1.087	0.983	0.642	1.511
StH	21	36300901	36301200	1.058	1.240	1.772	1.156
StH	21	36447901	36448200	0.550	1.400	5.715	0.554
StH	21	36810901	36811200	0.132	0.049	1.859	0.031
StH	21	37341301	37341600	0.240	0.096	2.795	0.737
StH	21	37341601	37341900	0.053	0.000	1.358	0.412
StH	21	37980901	37981200	0.373	0.493	0.132	0.360
StH	21	38373901	38374200	0.398	0.910	0.078	0.497
StH	21	39021601	39021900	2.418	2.075	2.818	1.430
StH	21	39344101	39344400	1.996	2.390	1.063	2.261
StH	21	39389701	39390000	0.529	0.588	1.313	0.614
StH	21	39408901	39409200	5.260	5.212	3.821	4.401
StH	21	39417601	39417900	0.344	0.275	1.322	0.456
StH	21	39426001	39426300	0.685	1.272	4.320	1.243
StH	21	39609901	39610200	0.905	0.491	0.691	0.801
StH	21	39618901	39619200	0.560	0.768	3.049	0.563
StH	21	39623401	39623700	0.448	0.528	1.418	0.436
StH	21	39626401	39626700	2.609	1.287	1.839	1.577
StH	21	40018501	40018800	0.982	0.936	0.486	0.974
StH	21	40357801	40358100	5.880	5.834	4.668	6.119
StH	21	40502401	40502700	0.823	0.914	0.428	0.634
StH	21	40759201	40759500	0.636	0.637	0.049	0.639
StH	21	40809001	40809300	0.450	0.538	0.720	0.335
StH	21	41079901	41080200	1.858	1.991	1.057	1.734
StH	21	41281801	41282100	0.052	0.234	0.448	0.218
StH	21	41312701	41313000	1.886	1.968	1.284	1.805
StH	21	41550001	41550300	1.437	1.287	0.896	1.815
StH	21	41626801	41627100	3.273	2.624	1.908	2.454
StH	21	41693401	41693700	0.211	0.159	1.204	0.218
StH	21	41746801	41747100	0.476	1.081	2.506	0.939
StH	21	41867101	41867400	3.007	2.830	1.764	2.576
StH	21	41877901	41878200	0.690	0.841	0.379	1.197
StH	21	41886301	41886600	1.803	1.493	0.921	1.825
StH	21	41924101	41924400	1.700	1.937	1.115	1.627
StH	21	41932501	41932800	2.533	3.111	1.400	2.516
StH	21	41933401	41933700	1.937	2.405	1.287	2.094
StH	21	41969401	41969700	0.505	0.444	1.280	0.573

StH	21	42167401	42167700	0.960	0.952	1.910	0.958
StH	21	42192601	42192900	4.115	4.177	5.679	4.199
StH	21	42375901	42376200	0.371	0.360	1.534	0.365
StH	21	42402901	42403200	2.708	2.637	2.024	2.758
StH	21	42403201	42403500	4.838	4.871	2.971	4.922
StH	21	42549601	42549900	3.601	5.027	2.138	3.720
StH	21	42582601	42582900	1.908	1.855	3.840	1.872
StH	21	42691201	42691500	0.345	0.444	0.132	0.274
StH	21	42730801	42731100	0.344	0.513	0.132	0.304
StH	21	42840301	42840600	0.957	1.154	2.276	1.192
StH	21	43086001	43086300	0.105	0.185	0.609	0.182
StH	21	43361701	43362000	0.423	0.466	0.872	0.441
StH	21	43379101	43379400	0.616	0.886	3.811	0.477
StH	21	43412401	43412700	0.507	0.534	0.132	0.832
StH	21	43483501	43483800	2.579	1.686	0.802	1.177
StH	21	43579201	43579500	1.065	1.614	0.613	1.394
StH	21	43659001	43659300	1.197	1.450	1.830	1.435
StH	21	43789501	43789800	0.903	0.867	0.662	0.943
StH	21	44463901	44464200	0.584	0.772	0.210	0.477
StH	21	44879401	44879700	10.984	12.536	8.122	12.031
StH	21	44889601	44889900	0.587	0.584	0.292	0.659
StH	21	45058801	45059100	0.213	0.468	0.132	0.335
StH	21	45179701	45180000	4.023	3.611	6.906	8.714
StH	21	45209401	45209700	0.105	0.045	0.560	0.112
StH	21	45387301	45387600	0.156	0.182	0.691	0.248
StH	21	45400501	45400800	0.931	1.408	0.535	1.105
StH	21	45470701	45471000	1.147	0.000	1.964	0.177
StH	21	45556801	45557100	4.015	3.564	1.537	5.212
StH	21	45567001	45567300	0.479	1.336	6.099	0.863
StH	21	45651001	45651300	0.400	0.955	0.695	0.385
StH	21	45686701	45687000	2.508	2.272	14.650	4.084
StH	21	45773701	45774000	0.450	0.560	0.794	0.309
StH	21	45841801	45842100	0.366	0.137	1.177	0.248
StH	21	45989401	45989700	1.084	0.790	0.210	2.236
StH	21	46186801	46187100	0.424	0.970	2.149	0.477
StH	21	46193401	46193700	1.997	1.826	1.048	1.683
StH	21	46311601	46311900	2.875	2.849	2.312	3.073
StH	21	46325701	46326000	0.187	0.157	0.789	0.132
StH	21	46405801	46406100	0.318	0.350	0.745	0.263
StH	21	46411201	46411500	0.242	0.360	1.658	0.411
StH	21	46557301	46557600	0.216	0.274	1.379	0.076
StH	21	46687501	46687800	1.113	1.407	3.739	1.552
StH	21	46738801	46739100	1.484	2.965	5.717	2.055
StH	21	46769401	46769700	1.468	1.793	2.793	1.476
StH	21	46830301	46830600	0.000	0.270	1.159	0.086
StH	21	46907701	46908000	0.239	0.770	0.539	0.304
StH	21	46914901	46915200	1.518	2.058	0.945	1.511
StH	21	46935601	46935900	4.788	3.444	2.780	5.760
StH	21	47019001	47019300	0.526	0.319	1.480	0.279
StH	21	47036701	47037000	0.479	1.272	4.170	0.492
StH	21	47273401	47273700	0.318	0.330	0.560	0.198
StH	21	47274301	47274600	3.050	2.736	1.794	3.027
StH	21	47301001	47301300	1.134	0.740	4.439	1.465
StH	21	47341201	47341500	6.433	5.936	9.967	7.197
StH	21	47601001	47601300	3.246	1.757	2.374	2.921
StH	21	47711401	47711700	0.319	0.230	0.896	0.436
StH	21	47994601	47994900	0.106	0.270	1.045	0.112
StH	21	48009601	48009900	1.242	1.293	2.813	1.288
StH	21	48020701	48021000	0.081	0.139	0.555	0.142
StH	21	48060301	48060600	0.261	0.324	0.934	0.365
StH	21	48190201	48190500	1.431	1.450	0.856	1.440
StH	21	48256201	48256500	2.189	2.475	5.852	2.273
StH	21	48261601	48261900	0.000	0.862	3.068	0.340
StH	21	48398101	48398400	1.513	1.696	5.501	1.258
StH	21	48408001	48408300	1.824	2.295	5.721	2.627
StH	21	48415501	48415800	0.319	0.409	2.042	0.766
StH	21	48420601	48420900	1.305	1.639	0.185	1.262

StH	21	48444001	48444300	3.268	3.189	1.900	3.929
StH	21	48454201	48454500	1.118	0.924	4.808	1.523
StH	21	48454501	48454800	0.423	0.701	2.552	0.497
StH	21	48601201	48601500	0.000	0.000	0.753	0.031
StH	21	48605401	48605700	0.447	0.600	2.035	0.583
StH	21	48641101	48641400	0.134	0.163	0.865	0.086
StH	21	48949501	48949800	0.477	1.272	3.982	0.882
StH	21	49062601	49062900	0.163	0.405	2.269	0.248
StH	21	49082701	49083000	2.315	3.104	6.688	3.229
StH	21	49114801	49115100	0.373	0.564	1.128	0.248
StH	21	49155601	49155900	9.781	7.091	11.416	9.203
StH	21	49160701	49161000	0.400	0.364	1.193	0.268
StH	21	49167601	49167900	0.081	0.182	0.671	0.101
StH	21	49222501	49222800	0.471	0.474	2.235	0.659
StH	21	49231501	49231800	0.294	0.658	2.091	0.167
StH	21	49247701	49248000	1.199	1.009	0.395	1.282
StH	21	49250101	49250400	0.266	0.588	2.984	0.624
StH	21	49254001	49254300	1.778	1.518	4.822	2.479
StH	21	49267201	49267500	1.303	1.665	0.827	1.262
StH	21	49284901	49285200	0.289	0.637	2.064	0.579
StH	21	49310101	49310400	0.106	0.045	0.506	0.106
StH	21	49340701	49341000	0.000	0.317	1.152	0.061
StH	21	49348501	49348800	0.718	1.452	1.099	2.048
StH	21	49434901	49435200	0.242	0.185	0.798	0.187
StH	21	49453201	49453500	0.027	0.068	0.600	0.101
StH	21	49639801	49640100	0.213	0.523	2.413	0.198
StH	21	49673701	49674000	0.402	1.248	4.616	0.533
StH	21	49674001	49674300	0.161	0.202	1.025	0.081
StH	21	49688101	49688400	0.294	0.159	0.691	0.203
StH	21	49900501	49900800	0.237	0.227	0.916	0.254
StH	21	49924801	49925100	1.442	1.311	2.936	1.389
StH	21	49993201	49993500	0.106	0.562	2.590	0.208
StH	21	50038501	50038800	0.187	0.362	2.092	0.335
StH	22	157501	157800	0.079	0.163	0.535	0.112
StH	22	593101	593400	1.833	1.427	2.481	1.647
StH	22	624901	625200	0.531	0.487	0.950	0.608
StH	22	664501	664800	0.189	0.236	0.609	0.198
StH	22	1018501	1018800	1.410	1.474	2.011	1.177
StH	22	1083301	1083600	0.213	0.191	0.876	0.243
StH	22	1258201	1258500	0.213	0.350	0.555	0.198
StH	22	1650001	1650300	0.611	0.302	1.086	0.218
StH	22	2075701	2076000	0.340	0.279	0.876	0.132
StH	22	2386201	2386500	0.426	0.350	0.831	0.421
StH	22	2602501	2602800	1.520	1.324	0.346	1.202
StH	22	2731801	2732100	0.429	0.470	1.012	0.365
StH	22	3231301	3231600	0.106	0.225	0.876	0.081
StH	22	3231601	3231900	0.160	0.343	1.313	0.122
StH	22	4252501	4252800	0.907	1.940	2.419	0.771
StH	22	4582201	4582500	0.529	0.510	0.054	0.558
StH	22	4690201	4690500	0.158	0.142	0.847	0.284
StH	22	4921501	4921800	0.636	0.862	1.242	0.826
StH	22	5125801	5126100	0.187	0.092	0.702	0.198
StH	22	5800201	5800500	0.720	0.867	0.506	1.050
StH	22	5863201	5863500	0.239	0.097	0.642	0.147
StH	22	6024001	6024300	0.424	0.418	0.769	0.248
StH	22	6099001	6099300	0.106	0.540	3.210	0.187
StH	22	6929701	6930000	0.077	0.305	0.531	0.299
StH	22	7237501	7237800	1.384	0.605	0.992	0.466
StH	22	7737601	7737900	0.428	0.375	0.876	0.396
StH	22	8052001	8052300	2.370	1.770	0.896	1.714
StH	22	8339401	8339700	0.558	0.607	0.218	0.608
StH	22	8533801	8534100	0.845	0.921	1.777	0.558
StH	22	8934601	8934900	1.471	1.749	0.593	2.043
StH	22	9963001	9963300	0.452	0.560	0.321	0.167
StH	22	10196701	10197000	0.931	0.120	0.526	0.203
StH	22	10211401	10211700	0.242	0.193	0.716	0.279
StH	22	11006401	11006700	2.284	2.478	1.683	2.474

StH	22	11076601	11076900	0.240	1.495	0.856	1.080
StH	22	11642101	11642400	1.091	1.099	0.593	1.247
StH	22	11642401	11642700	1.066	1.283	0.515	1.308
StH	22	11651701	11652000	0.452	0.446	0.165	0.436
StH	22	12360301	12360600	0.479	0.468	0.296	0.522
StH	22	12481801	12482100	2.391	2.871	4.158	3.260
StH	22	12687001	12687300	0.855	0.845	0.379	0.913
StH	22	12903901	12904200	0.181	0.762	1.846	0.173
StH	22	13305601	13305900	0.773	0.704	0.234	0.472
StH	22	13340701	13341000	0.584	0.583	0.823	0.385
StH	22	14158201	14158500	0.453	0.421	0.214	0.639
StH	22	14161501	14161800	0.400	0.281	0.736	0.391
StH	22	16092301	16092600	0.747	0.884	1.745	0.862
StH	22	16236301	16236600	1.144	1.427	1.826	1.445
StH	22	16366201	16366500	0.319	0.330	0.560	0.106
StH	22	16779301	16779600	3.223	3.207	0.638	2.205
StH	22	17020801	17021100	0.081	0.264	1.044	0.132
StH	22	17335201	17335500	0.182	0.371	0.881	0.355
StH	22	17477101	17477400	0.186	0.444	0.078	0.274
StH	22	17491501	17491800	0.745	1.053	0.428	0.796
StH	22	17587801	17588100	0.268	0.347	0.876	0.345
StH	22	17829001	17829300	1.884	1.873	0.809	1.557
StH	22	17843101	17843400	2.787	2.328	1.016	1.845
StH	22	17860801	17861100	1.223	1.285	1.572	0.846
StH	22	17884201	17884500	0.769	0.924	2.338	0.826
StH	22	17888101	17888400	1.249	1.216	0.437	1.364
StH	22	17967601	17967900	1.650	2.337	1.333	1.577
StH	22	18055801	18056100	0.319	0.704	0.132	0.360
StH	22	18149401	18149700	1.037	0.773	1.275	1.004
StH	22	18166801	18167100	0.426	0.232	0.687	0.324
StH	22	18187501	18187800	1.458	1.525	0.753	1.455
StH	22	18192601	18192900	2.179	2.135	0.789	1.799
StH	22	18199501	18199800	0.773	0.538	1.066	0.806
StH	22	18233701	18234000	0.160	0.045	0.419	0.112
StH	22	18324601	18324900	0.266	0.324	0.588	0.218
StH	22	18621901	18622200	3.328	3.171	4.378	3.321
StH	22	18736801	18737100	0.639	0.511	1.041	0.375
StH	22	18930601	18930900	0.158	0.208	0.564	0.243
StH	22	19635901	19636200	1.221	1.968	0.682	1.227
StH	22	19717201	19717500	1.813	1.918	1.300	1.800
StH	22	19778701	19779000	0.934	0.820	0.225	0.907
StH	22	19994701	19995000	0.266	0.069	0.531	0.173
StH	22	20413201	20413500	0.636	0.772	0.185	0.623
StH	22	20534701	20535000	1.407	1.217	0.560	1.019
StH	22	20562301	20562600	0.026	0.024	0.925	0.031
StH	22	20562601	20562900	0.000	0.045	0.691	0.031
StH	22	20595901	20596200	0.161	0.165	0.473	0.132
StH	22	20712601	20712900	2.397	2.471	3.382	2.485
StH	22	20786101	20786400	2.231	1.641	3.094	1.800
StH	22	20970601	20970900	0.319	0.283	0.535	0.304
StH	22	21217201	21217500	2.102	2.813	4.115	2.524
StH	22	21516301	21516600	0.318	0.491	0.136	0.274
StH	22	21734101	21734400	1.590	1.343	3.186	1.576
StH	22	21930901	21931200	1.089	0.794	1.222	0.938
StH	22	22148401	22148700	0.239	0.236	0.448	0.142
StH	22	22203901	22204200	1.223	1.309	1.037	1.161
StH	22	22365001	22365300	4.654	4.035	3.150	4.254
StH	22	22394101	22394400	3.139	2.594	2.102	1.825
StH	22	22668001	22668300	0.081	0.326	0.477	0.279
StH	22	23269501	23269800	0.529	0.609	0.127	0.588
StH	22	23355001	23355300	1.884	1.326	2.686	1.784
StH	22	23507101	23507400	0.373	0.538	0.239	0.391
StH	22	23923801	23924100	0.268	0.096	0.847	0.416
StH	22	24292501	24292800	0.349	0.236	0.925	0.350
StH	22	24466501	24466800	0.134	0.215	0.609	0.218
StH	22	24952501	24952800	0.505	0.420	0.267	0.081
StH	22	25380301	25380600	1.061	0.839	1.748	0.878

StH	22	25504501	25504800	0.187	0.187	0.531	0.117
StH	22	26491201	26491500	0.319	0.493	0.082	0.380
StH	22	26709601	26709900	0.428	0.275	0.999	0.025
StH	22	27247801	27248100	0.347	0.165	0.506	0.137
StH	22	29786701	29787000	0.266	0.071	0.531	0.198
StH	22	29913001	29913300	1.092	1.401	0.749	1.141
StH	22	29988601	29988900	1.707	1.687	0.749	1.612
StH	22	30108301	30108600	0.265	0.995	4.207	1.111
StH	22	30650101	30650400	0.292	0.326	0.506	0.056
StH	22	30692401	30692700	0.586	0.796	0.321	0.690
StH	22	31049101	31049400	0.561	0.609	0.156	0.603
StH	22	31492801	31493100	0.852	1.053	0.136	1.500
StH	22	31619701	31620000	1.223	0.727	1.037	0.776
StH	22	32153101	32153400	0.611	0.513	0.292	0.360
StH	22	32316901	32317200	0.240	0.281	0.473	0.112
StH	22	32386201	32386500	0.186	0.208	0.555	0.167
StH	22	32455801	32456100	4.790	4.503	3.596	4.752
StH	22	32654401	32654700	0.560	0.494	0.185	0.684
StH	22	32670001	32670300	1.010	0.890	0.395	0.943
StH	22	32798401	32798700	0.957	1.307	0.580	0.826
StH	22	33966301	33966600	0.184	0.843	0.448	0.715
StH	22	34218301	34218600	0.905	0.815	0.506	0.882
StH	22	34459201	34459500	0.266	0.281	0.662	0.198
StH	22	34530901	34531200	1.673	0.976	0.486	1.019
StH	22	34614301	34614600	0.156	0.144	0.580	0.056
StH	22	34712401	34712700	0.347	0.487	0.885	0.365
StH	22	34878001	34878300	0.452	0.068	0.613	0.329
StH	22	35217901	35218200	0.424	0.590	1.188	0.588
StH	22	35312701	35313000	0.242	0.140	0.477	0.081
StH	22	35344801	35345100	0.532	0.744	1.308	0.893
StH	22	35409601	35409900	1.091	1.352	0.564	0.918
StH	22	35543401	35543700	0.424	0.560	0.185	0.441
StH	22	35569501	35569800	1.065	0.607	0.954	0.608
StH	22	35910901	35911200	0.105	0.538	0.312	0.081
StH	22	36028201	36028500	0.984	0.706	0.341	0.776
StH	22	36510601	36510900	0.345	0.468	0.165	0.497
StH	22	36640201	36640500	1.329	1.384	0.564	1.491
StH	22	36765001	36765300	0.105	1.004	0.486	0.167
StH	22	36800101	36800400	0.053	0.140	0.448	0.147
StH	22	36839701	36840000	0.665	2.628	1.797	0.781
StH	22	36984601	36984900	0.347	0.257	0.531	0.664
StH	22	37178401	37178700	0.531	0.517	0.210	0.446
StH	22	38192701	38193000	4.310	4.443	3.293	4.376
StH	22	38432701	38433000	0.424	1.073	0.239	0.497
StH	22	38548501	38548800	1.541	1.659	1.271	1.982
StH	22	38551201	38551500	0.878	0.936	0.580	0.218
StH	22	38573401	38573700	3.697	3.253	4.389	2.997
StH	22	38890801	38891100	0.534	0.601	1.758	0.512
StH	22	39681601	39681900	0.210	0.234	0.593	0.000
StH	22	39843001	39843300	0.665	0.818	0.584	0.472
StH	22	39958501	39958800	0.507	0.560	0.243	0.547
StH	22	40225501	40225800	0.292	0.470	0.078	0.274
StH	22	40254601	40254900	0.136	0.163	0.695	0.187
StH	22	40254901	40255200	0.134	0.116	0.564	0.187
StH	22	40339201	40339500	0.745	0.539	0.399	0.198
StH	22	40353901	40354200	0.265	0.236	0.526	0.279
StH	22	40505101	40505400	2.494	2.584	1.032	2.267
StH	22	40713001	40713300	0.558	0.657	0.165	0.684
StH	22	40916701	40917000	0.077	0.116	0.560	0.223
StH	22	40917001	40917300	0.158	0.139	0.555	0.248
StH	22	40917901	40918200	1.165	1.686	0.312	1.186
StH	22	41373301	41373600	0.215	0.139	0.448	0.193
StH	22	41379901	41380200	3.615	2.965	2.435	2.936
StH	22	41704801	41705100	0.423	0.350	0.082	0.497
StH	22	42230701	42231000	0.268	0.303	0.477	0.167
StH	22	42390301	42390600	0.349	0.373	0.720	0.218
StH	22	42608701	42609000	1.334	1.751	0.885	2.079

StH	22	42993001	42993300	1.510	1.894	0.760	1.445
StH	22	43000501	43000800	0.452	0.723	2.394	0.655
StH	22	43105201	43105500	0.510	0.279	1.251	0.745
StH	22	43114501	43114800	0.615	0.710	1.786	0.669
StH	22	43133701	43134000	0.718	1.143	0.901	0.527
StH	22	43207501	43207800	1.408	1.379	1.119	1.379
StH	22	43275601	43275900	0.477	0.258	0.831	0.173
StH	22	43333501	43333800	5.717	3.974	4.795	4.061
StH	22	43416001	43416300	0.582	0.605	0.267	0.583
StH	22	43436101	43436400	2.247	2.506	4.610	2.139
StH	22	43507201	43507500	0.799	0.530	1.692	0.704
StH	22	43554301	43554600	0.881	0.983	1.300	0.720
StH	22	43757101	43757400	0.902	0.907	0.317	1.532
StH	22	43814701	43815000	0.132	0.092	0.531	0.162
StH	22	44106601	44106900	3.194	2.264	4.031	2.678
StH	22	44216101	44216400	0.905	0.818	1.906	0.826
StH	22	44220901	44221200	4.541	4.340	2.293	4.818
StH	22	44373601	44373900	1.908	1.738	2.884	1.334
StH	22	44373901	44374200	2.123	1.755	3.176	1.395
StH	22	44630701	44631000	1.992	1.976	4.037	2.160
StH	22	44881501	44881800	4.578	5.448	6.692	5.313
StH	22	44936701	44937000	0.665	0.936	0.230	0.715
StH	22	45034201	45034500	0.560	0.789	0.058	0.553
StH	22	45034501	45034800	4.920	4.510	0.667	2.734
StH	22	45084901	45085200	1.278	0.701	0.370	0.608
StH	22	45261601	45261900	0.344	0.257	0.662	0.137
StH	22	45320701	45321000	3.936	3.540	1.469	2.592
StH	22	45461101	45461400	3.318	3.506	2.007	3.027
StH	22	45592801	45593100	0.850	0.534	0.214	0.492
StH	22	45613501	45613800	0.690	1.006	0.399	0.882
StH	22	45869101	45869400	1.250	1.502	0.720	1.222
StH	22	45947401	45947700	0.371	0.350	0.103	0.436
StH	22	46030201	46030500	1.724	1.360	0.789	1.328
StH	22	46121401	46121700	0.081	0.090	0.613	0.173
StH	22	46208401	46208700	2.258	2.901	2.633	2.931
StH	22	46378501	46378800	1.226	1.171	0.707	1.278
StH	22	46495201	46495500	3.273	2.688	3.970	2.896
StH	22	46666801	46667100	2.396	2.055	1.674	2.130
StH	22	46717201	46717500	0.452	0.562	0.189	0.385
StH	22	46766401	46766700	4.972	4.293	6.333	4.691
StH	22	46825801	46826100	2.023	1.613	1.230	1.678
StH	22	46862401	46862700	2.370	2.922	1.797	2.875
StH	22	46912501	46912800	5.824	5.726	5.257	5.750
StH	22	46943701	46944000	0.108	0.068	0.510	0.132
StH	22	47014801	47015100	0.558	0.549	1.473	0.482
StH	22	47168101	47168400	3.065	2.974	2.234	1.516
StH	22	47619601	47619900	0.319	0.225	1.050	0.076
StH	22	47844301	47844600	2.952	2.967	2.448	3.570
StH	22	47867101	47867400	0.560	0.352	0.774	0.309
StH	22	47976001	47976300	0.216	0.806	1.679	0.284
StH	22	47991901	47992200	1.355	1.954	4.799	1.460
StH	22	48090001	48090300	2.315	1.222	4.900	3.174
StH	22	48096901	48097200	1.195	1.991	0.497	1.404
StH	22	48119701	48120000	0.473	0.817	2.615	0.608
StH	22	48125101	48125400	0.342	0.699	1.596	0.492
StH	22	48141601	48141900	0.644	0.577	2.053	0.613
StH	22	48154801	48155100	3.780	4.401	2.628	3.672
StH	22	48156601	48156900	1.144	1.049	0.593	1.334
StH	22	48210901	48211200	1.007	0.964	0.292	0.954
StH	22	48232201	48232500	0.673	0.972	2.345	0.558
StH	22	48264601	48264900	0.873	1.851	4.367	1.172
StH	22	48264901	48265200	1.685	1.903	4.309	1.695
StH	22	48474601	48474900	1.289	2.362	4.498	1.400
StH	22	48494701	48495000	0.584	0.493	0.234	0.497
StH	22	48519601	48519900	0.347	0.257	0.856	0.248
StH	22	48519901	48520200	1.297	1.512	2.711	1.131
StH	22	48520501	48520800	0.344	0.586	0.111	0.664

StH	22	48583501	48583800	1.594	1.746	2.550	1.642
StH	22	48602701	48603000	0.245	0.812	3.329	0.178
StH	22	48684001	48684300	2.205	1.195	1.781	2.191
StH	22	48833101	48833400	5.285	5.975	2.606	4.679
StH	22	48874201	48874500	0.982	0.959	1.222	0.994
StH	22	49016401	49016700	7.182	7.101	12.165	9.747
StH	22	49081201	49081500	5.798	5.885	3.533	5.151
StH	22	49086301	49086600	1.941	1.261	1.032	1.663
StH	22	49142101	49142400	0.721	0.796	0.321	0.852
StH	22	49177501	49177800	1.442	1.244	4.837	1.638
StH	22	49203001	49203300	0.160	0.208	0.477	0.218
StH	22	49212301	49212600	0.560	1.027	3.658	1.251
StH	22	49261801	49262100	1.382	0.886	2.011	1.283
StH	22	49264501	49264800	0.292	0.446	0.160	0.441
StH	22	49267801	49268100	0.402	0.575	1.324	0.329
StH	22	49301101	49301400	0.426	0.444	0.267	0.441
StH	22	49415401	49415700	3.771	4.355	2.949	4.412
StH	22	49575301	49575600	1.654	0.090	0.778	1.420
StH	22	49673701	49674000	0.737	2.282	6.591	0.862
StH	22	49741501	49741800	0.000	0.292	1.395	0.031
StH	22	49750801	49751100	0.318	0.440	1.115	0.344
StH	22	49795501	49795800	2.575	2.643	3.565	2.642
StH	22	49844401	49844700	0.215	0.184	0.691	0.157
StH	22	49938601	49938900	0.053	0.227	0.827	0.193
StH	22	49966801	49967100	4.578	3.873	3.094	4.203
StH	22	50036401	50036700	1.336	1.338	3.974	1.373
StH	22	50055901	50056200	0.026	0.947	3.560	0.259
StH	22	50059501	50059800	0.081	0.337	1.589	0.254
StH	22	50070301	50070600	0.529	0.744	1.144	0.740
StH	22	50173201	50173500	0.486	0.779	2.214	0.396
StH	22	50211301	50211600	0.966	1.132	2.336	0.898
StH	22	50312701	50313000	0.266	0.272	1.384	0.218
StH	22	50350501	50350800	0.419	0.755	2.020	0.513
StH	22	50406601	50406900	0.429	0.407	2.127	0.112
StH	22	50414701	50415000	0.000	0.116	0.580	0.025
StH	22	50431501	50431800	0.739	0.920	2.478	0.649
StH	22	50490601	50490900	0.984	1.077	0.453	0.806
StH	22	50499901	50500200	0.182	1.308	5.182	0.614
StH	22	50564701	50565000	5.911	4.325	5.199	6.018
StH	22	50566801	50567100	0.187	0.360	1.721	0.466
StH	22	50653801	50654100	1.121	2.997	1.699	1.100
StH	22	50654401	50654700	0.211	0.813	3.172	0.295
StH	22	50742901	50743200	3.299	2.176	2.818	3.463
StH	22	50772301	50772600	0.663	0.292	2.716	0.811
StH	22	50784001	50784300	0.242	0.210	0.477	0.193
StH	22	50788801	50789100	0.213	0.319	1.235	0.147
StH	22	50826601	50826900	0.079	0.206	1.041	0.056
StH	23	182401	182700	1.628	2.051	0.189	1.116
StH	23	186301	186600	0.053	0.096	0.675	0.000
StH	23	372301	372600	0.213	0.253	0.947	0.162
StH	23	552601	552900	4.582	4.632	3.233	4.330
StH	23	557701	558000	0.213	0.639	2.452	0.239
StH	23	558001	558300	0.079	0.427	2.040	0.214
StH	23	564901	565200	0.160	0.444	0.317	0.223
StH	23	647101	647400	0.611	1.304	3.917	1.014
StH	23	704401	704700	0.345	1.261	0.103	0.416
StH	23	748801	749100	2.674	3.532	4.401	2.601
StH	23	793801	794100	1.194	1.306	0.700	1.247
StH	23	798601	798900	2.526	1.808	3.640	1.642
StH	23	828001	828300	6.191	5.194	4.755	5.303
StH	23	918901	919200	0.160	0.232	0.477	0.279
StH	23	1094101	1094400	2.928	2.012	1.752	2.855
StH	23	1108201	1108500	2.020	2.209	4.953	2.293
StH	23	1151101	1151400	6.596	7.061	3.988	7.260
StH	23	1200301	1200600	2.846	2.570	1.850	2.358
[truncated: 759,838 more chars]
